# Integrated molecular analysis reveals complex interactions between genomic and epigenomic alterations in esophageal adenocarcinomas

**DOI:** 10.1038/srep40729

**Published:** 2017-01-19

**Authors:** DunFa Peng, Yan Guo, Heidi Chen, Shilin Zhao, Kay Washington, TianLing Hu, Yu Shyr, Wael El-Rifai

**Affiliations:** 1 Department of Surgery, Vanderbilt University Medical Center, Nashville, TN, USA; 2 Department of Biostatistics, Vanderbilt University, Nashville, Tennessee, USA; 3 Department of Cancer Biology, Vanderbilt University, Nashville, Tennessee, USA; 4 Department of Pathology, Vanderbilt University Medical Center, Nashville, TN, USA; 5 Department of Veterans Affairs, Tennessee Valley Healthcare System, Nashville, Tennessee, USA

## Abstract

The incidence of esophageal adenocarcinoma (EAC) is rapidly rising in the United States and Western countries. In this study, we carried out an integrative molecular analysis to identify interactions between genomic and epigenomic alterations in regulating gene expression networks in EAC. We detected significant alterations in DNA copy numbers (CN), gene expression levels, and DNA methylation profiles. The integrative analysis demonstrated that altered expression of 1,755 genes was associated with changes in CN or methylation. We found that expression alterations in 84 genes were associated with changes in both CN and methylation. These data suggest a strong interaction between genetic and epigenetic events to modulate gene expression in EAC. Of note, bioinformatics analysis detected a prominent K-RAS signature and predicted activation of several important transcription factor networks, including β-catenin, MYB, TWIST1, SOX7, GATA3 and GATA6. Notably, we detected hypomethylation and overexpression of several pro-inflammatory genes such as COX2, IL8 and IL23R, suggesting an important role of epigenetic regulation of these genes in the inflammatory cascade associated with EAC. In summary, this integrative analysis demonstrates a complex interaction between genetic and epigenetic mechanisms providing several novel insights for our understanding of molecular events in EAC.

The incidence of esophageal adenocarcinoma (EAC) has increased more than 6-fold over the past three decades in the United States and Western countries^1^,^2^,^3^. Chronic Gastroesophageal Reflux Disease (GERD) is a condition where esophageal epithelial cells are abnormally exposed to acidic bile salts and subsequently generates a high level of reactive oxygen species (ROS) and oxidative stress. GERD is the main risk factor for the development of a metaplastic glandular epithelium known as Barrett’s esophagus (BE), which can subsequently progress to high-grade dysplasia and EACs^2^,^3^. EAC is an aggressive malignancy characterized by unfavorable prognosis with 5-year survival at less than 15%, irrespective of treatment and tumour stage^4^,^5^.

Molecular studies have demonstrated complex patterns in EAC. Studies of DNA copy numbers using comparative genomic hybridization (CGH) have consistently shown complex genomic alterations that include gains and losses of multiple chromosomal regions with high level amplifications in 8q24, 17q21, and 20q13 and losses in 9p21, 17p, and 18q21^6^,^7^,^8^. Recent array-CGH and exome sequencing results have also indicated the presence of massive chromosomal and genomic instability^9^,^10^,^11^. The most frequent genetic changes that are implicated in EAC include silencing of p16 gene expression (by deletion or promoter hypermethylation), the loss of p53 expression (by mutation or deletion), and overexpression of cyclin D1^12^,^13^. Mutation analyses using whole-exome sequencing of EAC tumour-normal pairs confirmed that mutations of p53 are the most frequent alterations which occur in more than 50% of EAC, however, the frequency of mutation of any other individual gene falls below 5%^12^,^14^. In fact, these studies are all in agreement with the notion of high level of aneuploidy as a prominent feature of high-grade-dysplasia and EAC^15^,^16^.

Several genes have been reported to be downregulated or silenced in human cancers including EAC through epigenetic mechanisms that include promoter DNA hypermethylation^17^,^18^. Silencing of gene expression by promoter DNA methylation contributes to tumour development and progression. Examples include tumour suppressor genes CDKN2A (p16), APC, and CDH1; DNA damage repair genes such as MGMT; and antioxidant genes such as glutathione S transferase (GST) family and glutathione peroxidase family members^17^,^19^,^20^.

In this study, we have performed comprehensive integrated molecular analyses of gene expression, DNA copy number, and promoter DNA methylation using human EAC tissue samples. This integrated analyses approach identified a subset of genes where mRNA expression is associated with changes in copy number and/or methylation levels. We postulate that those genes that are regulated by more than one molecular mechanism are important drivers for the development of EAC. This could explain why cancer cells develop coordinated genetic and/or epigenetic mechanisms to regulate their expression.

## Materials and Methods

### Tissues Samples

Tissues were collected from 12 esophageal adenocarcinoma tumour samples and nine adjacent non-tumour histologically normal tissue samples (Supplementary Table S1). All tissue samples were examined for histological confirmation using haematoxylin and eosin staining followed by dissection of tumour tissue samples to enrich cancer cells content to ≥70%. All samples were subjected to molecular profiling that included comprehensive gene expression, copy number, and DNA methylation analyses. The use of de-identified specimens from the frozen tissue repository of the Department of Pathology was approved by the Vanderbilt University Institutional Review Board (IRB# 111096). All experiments were performed in accordance with the guidelines and regulations of Vanderbilt University Institutional Review Board and an informed consent was obtained from all human subjects. All experimental methods and protocols were approved by Vanderbilt University Biosafety Committee.

### Gene Expression Profiling

Total RNA from the tissue samples was prepared using Qiagen RNeasy Tissue kit (Qiagen, Germantown, MD). The total RNAs were evaluated at the Vanderbilt Microarray Core Lab. Affymetrix Human Gene 1.0 ST arrays (Affymetrix, Santa Clara, CA) were used for gene expression analysis.The RNA preparation, labeling and cDNA array hybridization were carried out following the manufacturer’s protocol. Raw data (CEL files) were processed using a robust multiarray averaging (RMA) approach to provide normalized expression data for each probe set on the arrays. Differential expression analysis (12 tumours vs. nine normal) was performed using limma package^21^. P-values were adjusted for multiple comparisons using the false discovery rate (FDR) method^22^. The thresholds for significance were set at p-value of 0.05 and fold change of two to determine the genes that were over- or under-expressed in tumours. Cluster analysis was carried out using R package Heatmap3^23^. Pathway and network analysis was performed using Ingenuity Pathway Analysis. Functional analysis was carried out using Gene Set Enrichment Analysis (GSEA)^24^. Gene expression data are available in Gene Expression Omnibus (GEO accession #GSE92396).

### DNA Methylation Analyses

DNA from the tissue samples were prepared using Qiagen DNeasy Tissue kit (Qiagen, Germantown, MD). Methylated DNA from each sample was enriched using Invitrogen MethylMiner™ Methylated DNA Enrichment Kit (ThermoFisher, Waltham, MA) following the manufacturer’s protocol. The captured DNA and input DNA were sent to Vanderbilt Microarray Core Lab for processing and hybridization. We used the NimbleGen 385 K array (Roche NimbleGen Inc., Madison, WI) which consists of 385,019 50-mer DNA probes with approximately 8 kb average spatial resolution. Normal samples were labeled with Cy5, and tumour samples were labeled with Cy3. The hybridizations were carried out following the NimbleGen’s protocol. The arrays were scanned at 5μm on an AXON 4000B scanner and analyzed using NimbleScan software v.2.6.0.0. Data were processed by Roche NimbleGen NimbleScan software (v1.9; NimbleGen). Three processing steps were involved: i) assigning scores to each probe, ii) finding peaks, and iii) annotating peaks. Signal intensity data were first extracted and processed to obtain a log2 ratio. A fixed-length window (750 bp) centered around the probe was selected to represent the distribution of the signal intensity of the probe, and the one-sided Kolmogorov-Smirnov (KS) test was applied to determine whether the probe was drawn from a significantly more positive distribution of intensity log-ratios than those in the rest of the array. The “finding peaks” step identified peaks as those consisting of at least two probes with scores above a minimum threshold of 2 (i.e. p-value < 0.05). The annotation step searched peaks in regions spanning 5 kb upstream and 1 kb downstream of the transcription start site (TSS). For a gene whose promoter region harbours multiple peaks, an average value of the scores was taken. In downstream analysis, each gene has one methylation level associated with its promoter region. The portion of probes aberrantly methylated in each TSS window (promoter region) was estimated. This was done by counting the probes in a called peak and dividing the count by the number of interrogated probes within a TSS window overlapping that peak. Calculations were done separately for hypermethylation and hypomethylation. For a gene possessing multiple transcription start sites, a peak might overlap multiple TSS windows associated with that single gene. In such a case, a TSS window around the most upstream TSS was used to represent the promoter region for the gene. The genomic positions were initially annotated by Human Genome Build version HG18 and were converted to use within a TSS window annotated by HG18_refGene. X and Y chromosomes were excluded from the analysis.

### Array Comparative Genomic Hybridization (aCGH)

DNA from the tissue samples was prepared using Qiagen DNeasy Tissue kit (Qiagen, Germantown, MD). aCGH was carried out using NimbleGen aCGH array (Roche NimbleGen Inc.). The array consists of 719,690 oligonucleotide DNA probes with approximately 4 kb average spatial resolution. Pooled DNA from three normal esophageal samples was used as the reference sample. Labeling and microarray processing were done according to the manufacturer’s protocol. All samples passed NimbleGen quality control assessment. Copy number (CN) was expressed as the log2 ratio of tumour:control DNA fluorescence intensity. CN data (log2 intensity ratios) were first processed by within-array normalization to remove spatial correlation and GC bias, followed by segmentation using circular binary segmentation (CBS) to translate noisy intensity measurements into regions of equal CN. The median absolute deviation (MAD) of the difference between the observed and segmented values was used to estimate sample-specific experimental variation. For each sample, a segment was declared gain or loss if the segment value was two times the sample MAD from the median segment of the autosomes. Probe value and CN status were determined based on the corresponding segment value and status. X and Y chromosome probes were excluded from analysis. A probe-wise frequency plot of CN alterations was based on gain/loss/normal status.

## Results and Discussion

### Gene Expression

Gene expression analysis identified 6,715 genes that are differentially expressed (FDR < 0.05) in tumour samples as compared to normal samples (3,488 genes were upregulated and 3,227 genes were downregulated in tumours) (Supplementary Table S2). Even though the majority of the samples were tumour-normal paired, unsupervised cluster analysis using all genes shows a clear separation between tumour and normal samples with one outlier (Supplementary Figure S1). These results confirm the presence of a strong difference in overall gene expression pattern between tumour and normal that is sufficient to distinguish tumours and adjacent normal tissues.

Gene ontology (GO) analysis using these differentially expressed genes identified several GO categories (Table 1). Many of the identified GO categories have been previously linked to esophageal cancer. For example, we identified two upregulated GO categories related to metallopeptidase. Aberrant expression of these proteins has been associated with esophageal adenocarcinomas^25^,^26^,^27^. Our finding suggests that the differential expression of metalloproteinases related genes is important for EAC. The other top upregulated GO categories identified are collagen^28^, proteinaceous extracellular matrix and extracellular matrix part^29^,^30^. Extracellular matrix interaction can be promoted by mucins – large o-glycoproteins, which are often overexpressed in cancer cells^31^,^32^. This may partially explain the identification of extracellular related GO categories. However, the stroma cells in tumour microenvironment may also contribute to this finding^27^. The representative downregulated GO categories in this analysis are the intermediate filament and intermediate filament cytoskeleton^33^. Downregulation of cytokeratins has been reported in esophageal cancers^34^.

Analysis of data using Ingenuity^®^ Pathway Analysis (IPA^®^, QIAGEN, Redwood City, www.qiagen.com/ingenuity) predicted activation of several upstream molecular pathways that included key transcription factors, growth factors, kinases, and enzymes (Table 2). These included activation of β-catenin (CTNNB1), MYB, SOX7, TWIST1, GATA3, and GATA6 transcription factors. These factors have been known to play essential roles in regulation of normal cell functions. Dysfunction of these transcription factors plays an important roles in tumourigenesis. For example, the wnt/β-catenin signaling pathway plays an important role in cell-cell adhesion. Activation of β-catenin due to mutations and/or overexpression of components of the β-catenin pathway have been associated with several tumours, including colorectal cancer^35^, lung cancer^36^, breast cancer^37^, as well as gastric^38^,^39^ and esophageal adenocarcinoma^40^,^41^. Increased expression of transcription factor MYB has been reported in many human cancers^42^, including esophageal adenocarcinoma^43^. MYB plays a key role as a regulator of stem and progenitor cells in the bone marrow, colonic crypts and a neurogenic region of the adult brain^42^,^44^. TWIST1 is a basic helix-loop-helix (bHLH) transcription factor and its dysfunction is associated with epithelial-mesenchymal transition (EMT), contributing to tumour metastasis^45^. GATA factors are zinc finger DNA binding proteins that control the development of diverse tissues by activating or repressing transcription^46^. It can be divided into two subfamilies: GATA1/2/3 and GATA4/5/6^47^. Dysfunction of these GATA members has been related to various human cancers. GATA3 plays a pivotal role in the normal mammary development and tumour differentiation of breast cancer^48^. GATA6 is overexpressed in Barrett’s esophagus showing a progressive increase in Barrett’s dysplasia and EAC^49^. Taken together, GATA transcription factors could play important roles in Barrett’s tumourigenesis; further studies are needed to establish their functional roles in EAC.

In addition, the IPA results also suggest activation of key growth factors such as TGFB, EGF, HGF, and ANGPT2 in EAC. These growth factors are important in repair of reflux-induced esophageal injury and play important roles in cellular homoeostasis, angiogenesis, and gastrointestinal carcinogenesis^50^,^51^,^52^,^53^.

Among kinases, pathways that were predicted to be activated by IPA in our EAC samples include, ERBB2, FGFR2, and MAPKs. Gene amplification and overexpression of ERBB2, FGFR2 and MAPKs have been reported in gastroesophageal cancers^54^,^55^,^56^. Of note, targeting signaling kinases has been a promising therapeutic approach in several cancer types^56^,^57^,^58^. In addition to these important signaling molecules, the results predicted activation of CD44 pathway, which plays an important role in the development and progression of cancer^59^,^60^. CD44 is known to define cells with stem cell properties^61^ and its activation has been associated with aggressive tumours and resistance to therapy^62^,^63^. Therefore, our findings are consistent with the biology of EACs, which are characterized by poor outcome and resistance to therapeutic approaches.

Gene Set Enriched Analysis (GSEA) was carried out based on gene expression data to determine the predominant oncogenic signature. This analysis predicted a signature that is overwhelmingly similar to KRAS activation observed in several cancer types including lung, prostate, and breast (Table 3). Although activating mutations in KRAS have been documented in several cancers^64^,^65^,^66^, these mutations are rare in EAC^14^,^67^. In this context, it is important to note that the observed KRAS signature in our EACs denotes the activation of signaling pathways downstream of KRAS and may contribute to the observed chemoresistance and poor clinical outcome in EACs as noted in other cancers with activated mutant KRAS^68^,^69^. The complete gene list for the predicted KRAS activation from GSEA analysis can be viewed in Table 3.

### DNA Copy Number Alterations and Correlation with Gene Expression

Overall, 661,383 (92%) probes showed copy number (CN) aberration (gain or loss) in our samples. Specifically, 454,299 (63%) probes showed CN gain and 458,201 (64%) showed CN loss. Supplementary Figure S3 shows the probe-wise frequency of CN changes across the entire genome (autosome) for all 12 tumours. A frequency value of 33% or more (i.e., at least 4 of 12 tumours) was set to identify commonly amplified or deleted probes. After filtering out probes in intergenic regions, 5,946 unique genes (1,115 amplified, 4,831 deleted) met this frequency cutoff. Details and frequency of genes that were amplified or deleted are given in Supplementary Tables S2 and S3. Our findings confirm the previously noted massive alterations in gene copy numbers in EAC^8^,^10^,^14^,^70^,^71^. A comparison of our results with previous publications is given in Supplementary Table S4.

**Figure 1:**
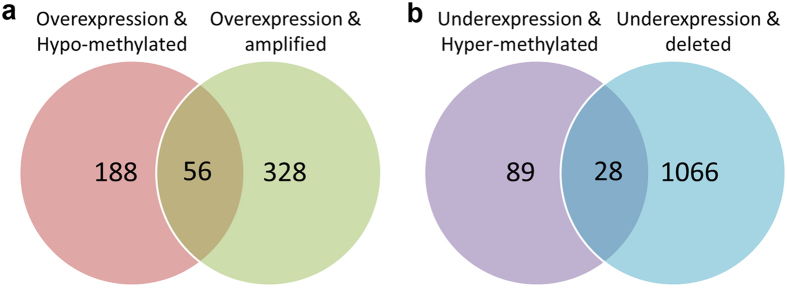
Number of genes with either genetic or epigenetic alterations. (**a**) Venn diagram shows the common overexpressed and hypomethylated genomic signatures vs overexpressed and amplified genes. (**b**) Venn diagram shows the common under-expressed and hypermethylated genomic signatures vs under-expressed and deleted genes.

Using integrated analysis of DNA copy number and gene expression, we identified 384 genes that were overexpressed with CN gain and 1,094 genes that were under-expressed with CN loss (Fig. 1 and Supplementary Tables S2 and S3). Mapping of the correlated genes (CN and expression) to the genome identified focused regions in which multiple CN alterations occurred (Supplementary Figure S4). This identified multiple focal CN losses and/or CN gains in several chromosomal regions such as 1p, 1q, 3p, 3q, 5p, 5q, 8q, 16, and 19 (Supplementary Figure S4). The frequently deleted genes in this study included well-known tumour suppressor genes: TP53 (41.7%, 5/12), SMAD4 (41.7%, 5/12), ARID1A (41.7%, 5/12), AXIN1 (50%, 6/12), CDKN2A (33.3%, 4/12), APC (25%, 3/12), and CDH1 (25%, 3/12) (Supplementary Table S2). These genes have been known to be frequently mutated in EAC^12^,^72^,^73^. Of note, many of these genes such as ARID1A, AXIN1, and APC showed significant downregulation in our samples (Supplementary Table S3). TP53, a well-known tumour suppressor gene, has been confirmed to be the most mutated gene in EAC, with much higher frequency than other genes^12^,^14^. Our analysis demonstrated deregulation of several targets of p53 in EAC (Supplementary Figure 2S). ARID1A gene, a member of the SWI/SNF chromatin-remodeling complex, has recently been identified to be a novel tumour suppressor gene in Barrett’s-related adenocarcinoma^74^. In agreement with our findings, deletion of CDKN2A is a common molecular mechanism for silencing expression of CDKN2A/p16 protein^75^. Of note, loss of functional p16 protein by deletions, mutations, or DNA methylation is an early event frequently detected during Barrett’s tumorigenesis and progression to EAC^14^,^76^. Consistent with previous studies, the loss of APC (3 of 12 tumours) coincided with either CDH1 loss (3 of 12) or AXIN1 loss (6 of 12). These genes are important players in the Wnt signaling pathway. Activation of the Wnt pathway through dysfunction of these genes has been observed in many cancers^77^, in particular gastrointestinal tumours^35^,^78^ including esophageal adenocarcinoma^41^. This is also consistent with our finding of activation of β-catenin as an upstream transcription factor (Table 3).

UGT2B17, GUCY1A2, LILRA3, LRP1B, and OR4F5 are the top 5 genes identified in our study with most frequent gene amplification (in 11, 9, 9, 8, 8 of 12 tumours, respectively, Supplementary Table S2). The functions of these genes in EAC are unknown, calling for further studies due to their high frequency of gene amplification. Genes with copy number gains in our study also included well-known genes like CDK6 (25%, 3/12), KRAS (25%, 3/12), PIK3CA (16.7%, 2/12), and EGFR (16.7%, 2/12) (Supplementary Tables S2 and S3). Similar findings have been reported in adenocarcinoma of gastroesophageal junction^71^,^79^,^80^,^81^. Of note, our analysis demonstrated a strong KRAS signature, as discussed above, whereas IPA analyses of gene expression data predicted activation of signaling pathways mediated by some of these amplified genes (Table 3).

### DNA Methylation and Correlation with Gene Expression

The median hypomethylated probe count per tumour was 11,324 (range of 5,935–22,746); and the median hypermethylated probe count per tumour was 6,011 (range: 5,629–26,895). Supplementary Figure S5 shows the probe-wise frequency of aberrant methylation status (hyper or hypo) across the entire genome (autosome) for 12 tumour samples. Overall, 765 genes showed promoter hypermethylation in one or more tumour samples, of which 331 were frequently hypermethylated in more than 60% of the tumour samples. On the other hand, 2,099 genes had hypomethylation in one or more tumour samples.

Integrated analysis of gene expression and DNA methylation identified 107 genes that showed DNA hypermethylation and mRNA downregulation (Fig. 1 and Supplementary Table S2). Among genes with frequent DNA hypermethylation, we found several gene clusters with significantly higher DNA methylation in EACs, as compared to normal tissues. These included several homeobox genes (HOX), forkhead box (FOX) families, G protein-coupled receptors (GPR), and zinc finger proteins (ZNF) (Supplementary Table S3). HOX genes belong to a large family that encodes for proteins functioning as critical master regulatory transcription factors during embryogenesis; where some of them reported overexpressed while others downregulated in cancer^82^,^83^. The roles of HOX genes methylation and downregulation in the biology of EAC or as biomarkers for cancer risk need to be determined. Several recent publications have reported DNA methylation of potassium channel related proteins (KCN) in human cancers^84^,^85^,^86^, including EAC^87^. Taken together, our findings call for functional analysis of the role of potassium channel related genes in the development and/or progression of EAC.

Our results showed that DSC3 (desmocollin 3) is among the top genes that were significantly downregulated and hypermethylated. This finding is consistent with an earlier report showing silencing of DSC3 by DNA methylation in advanced stages of esophageal adenocarcinoma^88^. We also detected downregulation and promoter hypermethylation of NDRG2. NDRG2 has some tumour suppressor functions as a potential metastasis suppressor gene in several cancers^89^,^90^. To date, the role of NDRG2 in Barrett’s tumourigenesis and EAC has not been explored.

We also identified 244 genes that showed DNA hypomethylation and mRNA overexpression (Fig. 1 and Supplementary Table S3). DNA hypomethylation has been reported as one of many mechanisms that regulate gene expression^91^,^92^. EAC develops in the background of chronic inflammation due to chronic gastroesophageal reflux disease where esophageal cells are abnormally exposed to acid and bile salts leading to the development of Barrett’s esophagus and its progression to EAC^93^,^94^. Interestingly, our data demonstrate hypomethylation of several inflammation-related genes in EAC. These included 16 interleukin family members such as IL8, IL23R, and as many as 19 interferon family members (Supplementary Table S3). We have previously reported that IL8 is induced by both neutral and acidic bile acids in esophageal epithelia^95^, consistent with the biology of EAC. Notably, PTGS2, also known as COX2, was also hypomethylated and overexpressed in our study. COX2 is one of the key players in inflammation-related cancers and is activated in esophageal cells following exposure to acid and bile salts and its overexpression has been reported in EAC^96^,^97^,^98^. Furthermore, consistent with the etiology of EAC, our analysis predicted activation of several pathways associated with cytokines that play a central role in the inflammatory processes (Table 2). Moreover, several genes that regulate invasion and metastasis were hypomethylated and overexpressed in our analysis of EAC (Supplementary Table S3). These genes included several members of the matrix metalloproteinases (MMPs) family; MMP1, MMP7, MMP12, MMP3, and MMP10. MMPs overexpression has been described in several cancers including EACs, and their activation is associated with matrix remodeling, invasion and angiogenesis^26^,^99^. We also detected DNA hypomethylation and overexpression of SPP1 and LYN. SPP1 (also known as Osteopontin), is a chemokine-like calcified ECM-associated protein, which plays a crucial role in determining the metastatic potential in gastrointestinal cancers^100^,^101^,^102^. LYN (v-yes-1 Yamaguchi sarcoma viral related oncogene homolog) gene is one of the 8 non-receptor tyrosine kinases (Src, Fyn, Yes, Lck, Lyn, Hck, Fgr and Blk) that interact with the intracellular domains of growth factor/cytokine receptors. These findings call for investigating the roles of SPP1 and LYN in Barrett’s carcinogenesis and EAC. Taken together, our results indicate the existence of a strong pro-inflammatory and pro-invasive environment in the development of EAC and suggest a previously unexplored interaction between promoter DNA hypomethylation and activation of these genes and networks during esophageal tumourigenesis.

### Integrated Analysis of Gene Expression, Copy Number and DNA Methylation

**Figure 2:**
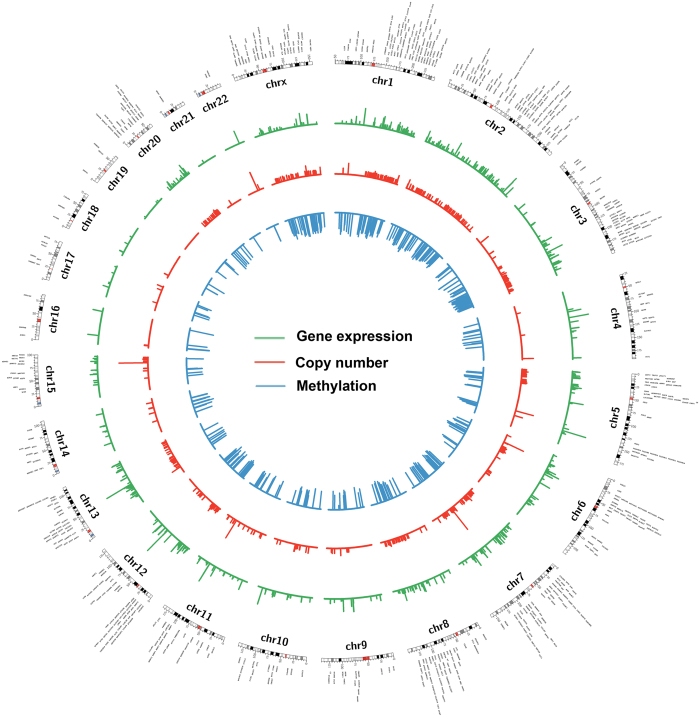
Genome wide integrative analysis of gene overexpression, DNA copy number gain and promoter DNA hypomethylation in esophageal adenocarcinoma. Circos plots that demonstrate the gene expression vs copy number that have the same directional changes. Gene expression and copy numbers vs methylation have opposite direction changes. Some high-level gene amplifications were shown in some chromosomes, such as 5, 6, 7, 11, 15, and 22. DNA hypomethylation is wide across the whole genome and denser in chromosome 1, 2, 3, 7, 8, 12 and X. Data shows the complexity of gene regulation through genetic and epigenetic mechanisms.

**Figure 3:**
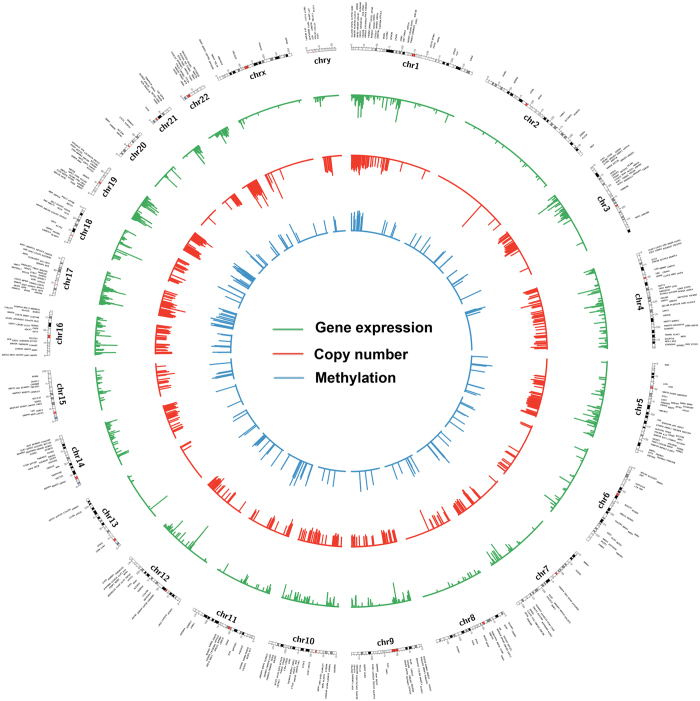
Genome wide integrative analysis of gene under-expression, DNA copy number loss and promoter DNA hypermethylation in esophageal adenocarcinoma. Circos plots that demonstrate gene expression versus copy number that have the same directional changes. Gene expression and copy number vs methylation have opposite directional changes. DNA copy number loss/deletions are more frequent than copy number gain/amplification as shown in Fig. 2. While some chromosomes such as 3, 4, 5, 10, 14, 16, 17, and 19 displayed more copy number losses, other chromosomes such as 2, 13, 20, and X showed fewer copy number losses. DNA hypermethylation was more frequent in chromosomes 1, 17 and 19. Overall, data shows the complexity of gene regulation through genetic and epigenetic mechanisms.

We have also carried out a fully integrated analysis that takes into account gene expression, methylation, and copy numbers (Figs 2 and 3, and Supplementary Table S2). We found 56 overexpressed genes that are associated with both CN gains and DNA hypomethylation whereas downregulation of 31 genes was associated with both CN losses and DNA hypermethylation (Fig. 1). Among these genes, CDH17 and GATA6 are examples with significant gene overexpression, promoter hypomethylation and copy number amplification in EACs. CDH17 is a member of the cadherin superfamily, encoding a calcium-dependent, membrane-associated glycoprotein that is specifically expressed in the gastrointestinal tract and pancreatic ducts. Recent studies indicated that CDH17 is involved in tumourigenesis and metastasis in gastrointestinal cancers^103^ and may serve as a prognostic biomarker^104^,^105^, although not studied previously in the context of EAC. GATA6 is one of the GATA transcription factor family members, which include GATA1–6^47^. We have discussed the importance of GATA6 in the above sections, and our results show a possible regulation of GATA6 by both genetic and epigenetic mechanisms in cancer cells (CN and methylation), and suggest an important role of GATA6 in esophageal tumourigenesis. On the other hand, ANXA8, ANXA8L1, and PPP2R2C were examples of downregulated genes with both DNA hypermethylation and copy number loss (Supplementary Table S2). Both ANXA8 and ANXA8L1 are members of the annexin family of evolutionarily conserved Ca2+ and phospholipid binding proteins that may function as an anticoagulant. Downregulation of ANXA8 has been shown to correlate with the morphologic changes of the epithelial-to-mesenchymal transition (EMT) and may contribute to cell scattering and metastasis in cholangiocarcinoma^106^. ANXA8 might also play an important role in calcium fluctuation-mediated HIF-1α transcriptional activation and cell viability in pancreatic cancers^107^. PPP2R2C is another interesting gene downregulated in EAC, with simultaneous copy number loss and hypermethylation (Supplementary Table S2). Dysfunction of PPP2R2C through microRNAs has been recently reported in some human cancers^108^,^109^. Our results indicate that dysfunction of these genes by both genetic and epigenetic interaction mechanisms may play an important role in Barrett’s tumourigenesis and EAC. Further studies are needed to elucidate the functions and regulatory mechanisms.

## Conclusion

Our study indicates that EACs have complex transcriptomic, genomic, and epigenomic alterations. Frequent copy number alterations highlight the chromosomal instability nature of these cancers. Although this study has some limitations due to a relatively small number of patients’ samples that were analyzed, our integrated analysis of gene expression, copy numbers, and methylation provided novel information regarding the complex molecular interactions in EACs. Our integrated approach identified several novel candidate genes and signaling networks that can be investigated for their biological functions and as possible diagnostic or prognostic biomarkers for EAC.

## Additional Information

**How to cite this article:** Peng, D. F. *et al*. Integrated molecular analysis reveals complex interactions between genomic and epigenomic alterations in esophageal adenocarcinomas. *Sci. Rep.* **7**, 40729; doi: 10.1038/srep40729 (2017).

**Publisher's note:** Springer Nature remains neutral with regard to jurisdictional claims in published maps and institutional affiliations.

## Supplementary Material

Supplementary Data

## Figures and Tables

**Table 1:** Gene Ontology Analysis.

GO category	Direction	adjusted p	Previous findings related to esophageal cancer
Metalloendopeptidase Activity	Up	<0.0001	Matrix metalloproteinases 21 and 26 in esophageal squamous cell cancer^25^ *
Metallopeptidase Activity	Up	<0.0001	Matrix metalloproteinase 9 and 13 in esophageal cancer^27^
Collagen	Up	<0.0001	Increased expression of integrins in human esophageal cancer cells^28^
Proteinaceous Extracellular Matrix	Up	0.001	Activin^29^, Periostin^30^ in esophageal cancers
Extracellular Matrix Part	Up	<0.0001	NA
Ectoderm Development	Down	<0.0001	NA
Epidermis Development	Down	<0.0001	NA
Tissue_Development	Down	0.002	NA
Intermediate Filament	Down	0.001	Cytokeratin 4 and 13 in esophageal cancers^34^
Intermediate Filament Cytoskeleton	Down	0.001	Microtubes in esophageal cancers^33^

^*^Indicates the reference number in the main text.

**Table 2:** Upstream Activated Molecular Pathways.

Upstream Regulator	P value of overlap	Target molecules in dataset
**Transcription Factor**		
CEBPB	8.55E-07	ALDH1A1, CFTR, COL10A1, COL1A2, COL5A2, CXCL8, DAB2, DHRS1, LYN, MGP, MMP1, MMP10, MMP3, NDRG4, NFATC2, PCK1, PPARG, PTGS2, SEMA3E, SGK1, SIM2, SPP1, TNFAIP6, VLDLR
CTNNB1	4.81E-05	ALDH1A1, ALDH3A2, BMP2, COL4A1, COL4A2, CXCL8, GHR, HSD17B2, ITGB7, MME, MMP1, MMP3, MMP7, NDRG2, NOTCH3, PTGS2, RAI14, SDC2, SEMA3C, SGK1, SIM2, SLC6A1, SPP1, TCF7L2, TNFRSF11B, TNIK, TSPAN8, VCAM1, WNT4
CEBPA	6.32E-05	ADH6, CCL20, COL10A1, COL1A2, CXCL8, EVPL, GATA6, GRHL3, NFATC2, OLR1, OVOL1, PCK1, PGD, PPARG, PPL, PTGS2, SEMA3E, SPP1, TIAM1, TNFAIP6, VLDLR
MYB	2.81E-04	ATP2B1, COL1A2, FUT8, MMP1, MMP3, NMU, PTGS2, SPP1
PPRC1	1.70E-03	CCL20, CXCL8, LAMB3, PTGS2, SPINK1
HMGB1	3.08E-03	CCL20, CXCL8, MMP1, MMP3, PTGS2, VCAM1
SOX7	7.91E-03	EVPL, GRHL3, OVOL1, PPL
TWIST1	8.21E-03	ALDH1A1, CXCL8, ETS1, FGFR3, MME, MMP1, PXDN, SPP1
PAX1	1.18E-02	EVPL, GRHL3, OVOL1, PPL
GATA6	2.13E-02	DAB2, EVPL, GRHL3, LTBP1, OVOL1, PPL, SEMA3C
GATA3	7.64E-02	ETS1, EVPL, FOXE1, GRHL3, OVOL1, PPARG, PPL
ETS1	2.12E-01	COL1A2, ETS1, MMP1, MMP3, MMP7, SPP1
**Growth Factor**		
TGFB1	1.40E-12	ABLIM3, ACVR1, ADAMTS12, AHNAK, ALDH3A2, ALOX12, ANXA8/ANXA8L1, AQP9, ASPN, BMP2, BUB1, CALB2, CCL20, CELSR2, CNN3, COL12A1, COL1A2, COL3A1, COL4A1, COL4A2, COL6A3, CXCL8, DAB2, DACH1, DLX5, DSP, ESPL1, ESRP2, ETS1, FAP, FNDC3B, FUT8, GGT6, GPR158, HLTF, INHBA, ITGA11, ITGAV, ITGB7, KCNJ3, KDELR3, LAMB3, LIFR, LTBP1, MGP, MMP1, MMP10, MMP12, MMP3, MMP7, NDRG4, NDST1, NFATC2, NOTCH3, OLR1, OVOL1, PLCB1, PMEPA1, PMM1, POSTN, PPARG, PTGS2, PTPRK, RAD51AP1, RARG, SGK1, SHMT1, SPP1, TNFAIP6, TNFRSF11B, TXNRD1, VAT1, VCAM1, WNT4, WNT5A
TGFB3	6.55E-07	ASPN, CDH6, COL1A2, COL3A1, ETS1, ITGAV, MGP, MICAL2, MMP1, MMP10, MMP3, TNFRSF11B
AGT	1.29E-06	ALOX12, ATP2B1, COL1A2, COL3A1, COL4A1, CXCL8, DAB2, ETS1, GATA6, ITGAV, ITGB7, MAPT, NOTCH3, OLR1, PDE3A, POSTN, PPARG, PTGS2, SGK1, SLC10A6, SPP1, STC1, TFPI, VCAM1, WNK4
HGF	3.75E-06	BMP2, BUB1, COL1A2, COL3A1, COL4A1, CXCL8, EMP2, ETS1, FNDC3A, GRB10, HK1, INHBA, LY75, MMP1, PRKAA1, PTGS2, PTPRR, SGK1, SLC9A3R1, SPP1, STC1, TMEM97, TNFAIP6, TRIP10, TRIP13, VCAM1
BTC	6.01E-05	CCL20, CXCL8, PTGS2, TNFAIP6
ANGPT2	8.37E-05	COL1A2, COL3A1, ETS1, GATA6, LYN, MMP1, MMP7, OLR1, POSTN, PTGS2, TLR3, TNIK, VCAM1
JAG1	1.79E-04	ITGAV, PTPRK, RNF128, SPP1, WNT4
VEGFA	3.91E-04	ACADM, CSTB, CXCL8, ETS1, ETV5, GRB10, HK1, MMP1, MMP12, PTGS2, STC1, TSPO, VCAM1
EGF	1.25E-03	ALOX12, AQP3, CCL20, COL1A2, COL3A1, CXCL8, DPP4, ETS1, FUT3, INHBA, MMP1, MMP10, MMP12, MMP3, PDE3A, PER1, PPARG, PTGS2, SPP1, VCAM1
**Kinase**		
ERBB2	1.14E-08	AHNAK, ANG, BNIP3, BUB1, C4BPB, CCL20, COL3A1, COL4A1, COL5A2, COL6A3, CXCL8, DAP, EPSTI1, ESPL1, ETV5, FAM134B, GHR, HEPH, LPCAT1, LUM, MME, MMP1, MMP10, MMP12, MMP3, MMP7, NDRG4, NDST1, NOTCH3, PMEPA1, PPARG, PTGS2, PTPRK, RAD51AP1, TFAP2C, WNT5A
FGFR2	1.57E-05	COL4A1, COL4A2, DAB2, GATA6, GPT2, GRB10, NOSTRIN, SPINK1, SPP1, TSPAN8, VCAM1
RET	5.93E-05	CCL20, COL1A2, CXCL8, HSPH1, MMP1, MMP10, MMP3, MMP7, PTPN13, STC1
CHUK	1.63E-04	BMP2, CXCL8, GM2A, LIFR, MGP, MMP3, OVOL1, PTGS2, SEMA3C, SGK1, SPP1, TNFRSF11B, VCAM1
PIK3R1	1.59E-03	COL1A2, CXCL8, PCK1, PPARG, PTGS2, VCAM1
MAP3K1	3.82E-03	COL3A1, COL4A1, CXCL8, MMP3, PTGS2
MAPK8	2.68E-02	CXCL8, ETS1, MMP1, MMP3, PTGS2, WNT4, WNT5A
MAPK1	1.13E-01	CPPED1, CXCL8, DAB2, ITGAV, LAMA4, MMP1, PTGS2, PTPRK, SPP1, TLR3
**Cytokine**		
OSM	7.79E-10	AQP9, C4BPA, CALB2, CASK, CCL20, CDA, CLIP1, COL3A1, CXCL8, DHCR24, EPHA1, EVPL, FMO5, LIFR, MGLL, MMP1, MMP10, MMP3, NOTCH3, PPARG, RARG, SCNN1B, SLC7A8, SULT2B1, TDO2, TECR, TLR3, TNFRSF11B, TYRO3, UBE2G1, UPK1A, USP46, VCAM1, WNT5A
IL1A	1.79E-07	ALDH1A1, ALDH3A2, CCL20, CXCL8, DPP4, INHBA, ITGAV, MMP1, MMP10, MMP12, MMP3, PPARG, PTGS2, SPP1, TICAM1, TNFRSF11B, VCAM1, WNT5A
TNF	1.51E-06	ACADM, ALOX15B, AQP3, AQP9, ATP2B1, BCKDHA, BMP2, CCL20, CFTR, COL1A2, COL3A1, CXCL8, DMBT1, DPP4, DSC3, EMP2, ETS1, FOXE1, FUT3, GHR, GM2A, INHBA, ITGAV, ITGB7, LAMA4, LAMB3, LIFR, LYN, MGP, MMP1, MMP10, MMP12, MMP3, MMP7, NFATC2, OLR1, PCK1, POSTN, PPARG, PTGS2, SCNN1B, SCNN1G, SCUBE2, SDC2, SEMA3C, SGK1, SLC7A8, SPP1, SYTL1, TFAP2C, TFPI, TICAM1, TLR3, TNFAIP6, TNFRSF11B, TXNRD1, VCAM1, WNT5A
IL1B	2.19E-05	ALDH7A1, BMP2, CCL20, CFTR, COL10A1, CXCL8, DAB2, DPP4, FGFR3, GHR, GM2A, INHBA, ITGAV, LAMB3, LIFR, MAPT, MMP1, MMP10, MMP12, MMP3, MMP7, OLR1, PLXDC2, POSTN, PPARG, PTGS2, SCNN1B, SCNN1G, SCUBE2, SPP1, SULT1E1, TLR3, TNFAIP6, TNFRSF11B, VCAM1
TNFSF11	3.33E-05	AQP9, CLOCK, CXCL8, DAB2, ECT2, ETS1, GRB10, ITGAV, MMP1, NFATC2, PAG1, PTGS2, SPP1, TNFRSF11B, TSPAN5, VCAM1
IL17F	1.79E-04	CCL20, CXCL8, MMP1, MMP3, PTGS2
IL6	1.13E-03	ACVR1, AHNAK, ANG, BMP2, BUB1, CCL20, COL3A1, CXCL8, DLX5, ITGAV, LIFR, MMP1, MMP10, MMP12, MMP3, MMP7, PPARG, PTGS2, REG1A, SGK1, SPP1, TLR3, TNFRSF11B, VCAM1, VLDLR, WNT5A
TNFSF12	1.92E-03	CXCL8, MMP1, MMP10, MMP12, MMP3, NOTCH3, VCAM1
EDN1	4.46E-03	COL1A2, COL4A1, CXCL8, ITGAV, MMP1, MMP3, MMP7, OLR1, PTGS2, VCAM1
IL18	5.00E-03	CCL20, CXCL8, MMP1, MMP3, PTGS2, SPP1, TNFRSF11B, VCAM1
MIF	1.14E-02	CXCL8, MMP1, MMP3, PTGS2, VCAM1
CSF2	1.37E-02	BUB1, CXCL8, F2RL2, HSPH1, INHBA, LY75, MME, MMP1, NDST1, NFATC2, PPARG, PTGS2, SGK1, SPP1, TICAM1, TRIP13
IL17A	3.53E-02	CCL20, CXCL8, MMP1, MMP3, PTGS2, TLR3, TNFRSF11B, VCAM1
**Enzyme**		
CD44	4.52E-05	COL3A1, COL5A2, ITGAV, KREMEN1, LTBP1, MMP12, MMP3, MMP7, PMEPA1, SPP1, TIAM1, VCAM1, WNT5A
PTGS2	9.84E-04	ANG, CXCL8, DLX5, ITGAV, MMP1, MMP7, NEB, PTGS2, STC1, TNFAIP6
FN1	1.71E-02	CXCL8, DHCR24, HK1, ITGAV, MGP, MMP1, MMP3, RRAGD, SPP1

**Table 3:** KRAS signature in EAC.

	Genes	References
Upregulated (85)	SPINK1, MMP1, TSPAN1, RBP4, CCL20, IL8, TOX3, PLAUR, INHBA, TMEM176B, HSD17B2, ANO1, STC1, FBP1, BIRC3, DENND5B, TNFAIP3, IGFBP3, TFPI, PTGS2, CXCL1, ID2, PTPRR, MMP9, ETS1, MMP15, GLRX, A2M, MMP10, TBX3, RNASE1, CXCL2, TNFSF15, ETV4, IL1B, BMP2, DDIT3, SCG5, ESM1, NR1H4, SEMA3B, MSMB, HAS2, GPR4, PIWIL1, ADAM8, G0S2, NRCAM, SPRY2, LPL, PRRX1, PLVAP, ADAMTS6, SEMA3A, CXCL5, PPP1R14D, DOCK4, GPR124, SPP1, ANPEP, USH1C, TGM2, MMP11, SYT1, UGT8, MMD, FABP3, MLXIPL, MGAT4A, ETV5, NRP1, TLR4, FMNL2, SCN2A, PELI2, LRCH1, ITGBL1, TRIB2, SOX9, CD80, CXCR4, DNAJA4, ZEB1, DUSP4, PLAT	http://software.broadinstitute.org/gsea/msigdb/cards/KRAS.600.LUNG.BREAST_UP.V1_UPhttp://software.broadinstitute.org/gsea/msigdb/cards/KRAS.LUNG.BREAST_UP.V1_UPhttp://software.broadinstitute.org/gsea/msigdb/cards/KRAS.600_UP.V1_UP
Downregulated (114)	CALML5, SH3GL3, CXCL14, AKAP6, MC5R, PLCH2, TRIM48, IGFBP2, VAV3, DEFB1, TF, GPNMB, MAFB, CALML3, S100A7, EPHB3, SLC6A9, KLK7, KRT16, SPTBN2, CRYAB, AKR1B10, UPK3B, KRT1, CYP2C18, SCGB1A1, SLC39A2, ACPP, BBOX1, LY6D, KLK12, KLK11, CRABP2, IVL, CYP4B1, LYPD3, FMO2, S100A12, ALDH3B2, SPRR3, KRT4, KRT13, TGM1, KRT15, ZNF750, RHCG, SERPINB3, SERPINB13, CLCA4, EPHX2, NOS1, RRAD, EPHB6, SPRR1B, PRODH, PLEKHH3, DLK2, BCL11B, HRASLS, FCGBP, ABCG4, IL19, DPT, EDN2, CYP2C19, MAML3, RASAL1, THBD, SLC30A4, CAMK1D, IMPA2, YOD1, PRRG4, CLDN8, FGFR3, BLNK, FETUB, KRT31, CD207, CLIC3, KLF8, HSPB8, DSG1, SPRR1A, DSG3, CLCA2, SIDT1, DTX2, FMO6P, HCN2, METTL7A, ALOX12B, GABRA4, BDKRB1, EPB41L4A, DENND2D, PLAC8, FAM189A2, SLC25A23, CACNA2D3, IL12A, PVRL1, EPHX3, SERPINB7, CD96, MUC4, ADAM23, PI3, ITGB7, RASGRP1, TFAP2B, CLDN17, S100A8, SCEL	http://software.broadinstitute.org/gsea/msigdb/cards/KRAS.LUNG_UP.V1_DNhttp://software.broadinstitute.org/gsea/msigdb/cards/KRAS.600_UP.V1_DNhttp://software.broadinstitute.org/gsea/msigdb/cards/KRAS.300_UP.V1_DNhttp://software.broadinstitute.org/gsea/msigdb/cards/KRAS.LUNG.BREAST_UP.V1_DNhttp://software.broadinstitute.org/gsea/msigdb/cards/KRAS.600.LUNG.BREAST_UP.V1_DNhttp://software.broadinstitute.org/gsea/msigdb/cards/KRAS.PROSTATE_UP.V1_DN
