# Supplementary material for: Integrated molecular analysis reveals complex interactions between genomic and epigenomic alterations in esophageal adenocarcinomas
Source: Sci Rep. 2017 Jan 19;7:40729. doi: 10.1038/srep40729 (PMC5244375; doi:10.1038/srep40729)
Supplement: Supplementary Data [file srep40729-s1.pdf]

Supplementary Figures and Tables

Integrated molecular analysis reveals complex interactions between genomic and epigenomic alterations in esophageal adenocarcinomas

DunFa Peng[1#]*, Yan Guo[#2,3], Heidi Chen[2,3], Shilin Zhao[2,3], Kay Washington[4], TianLing Hu[1], Yu Shyr[2], and Wael El-Rifai[1,3,5]*

[1]Department of Surgery and [4]Department of Pathology, Vanderbilt University Medical Center, Nashville, Tennessee, USA. [2]Department of Biostatistics. [3]Department of Cancer Biology, Vanderbilt University, Nashville, Tennessee, USA. [5]Department of Veterans Affairs, Tennessee Valley Healthcare System, Nashville, Tennessee, USA

[#] Equal contribution

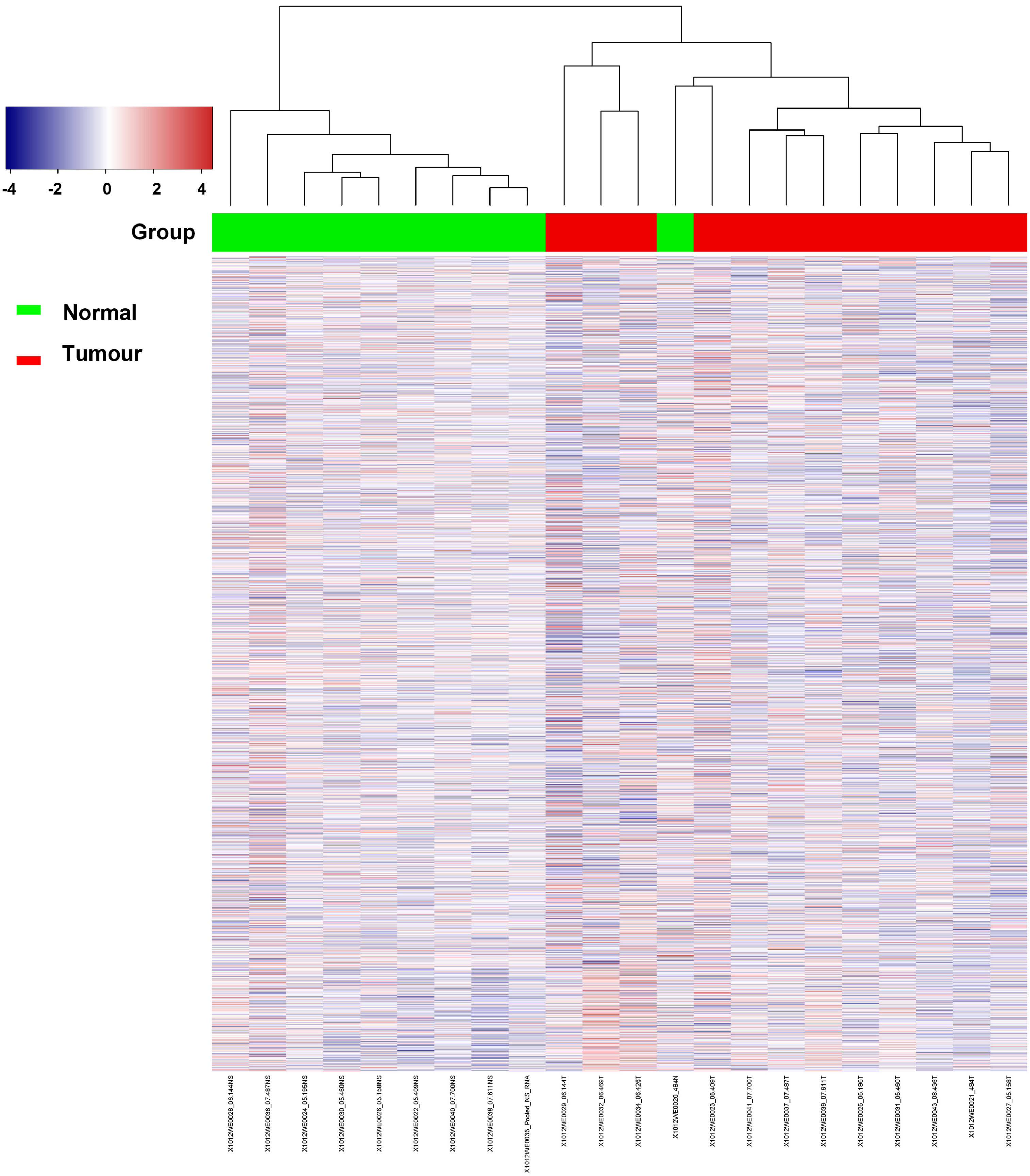

Supplementary Figure S1. Unsupervised cluster analysis of gene expression. Heatmap is based on gene expression of esophageal adenocarcinoma and normal samples. The results show significantly different gene expression profiles. Tumor and normal samples can be distinguished based on gene expression with only 1 normal sample clustered with tumor samples.

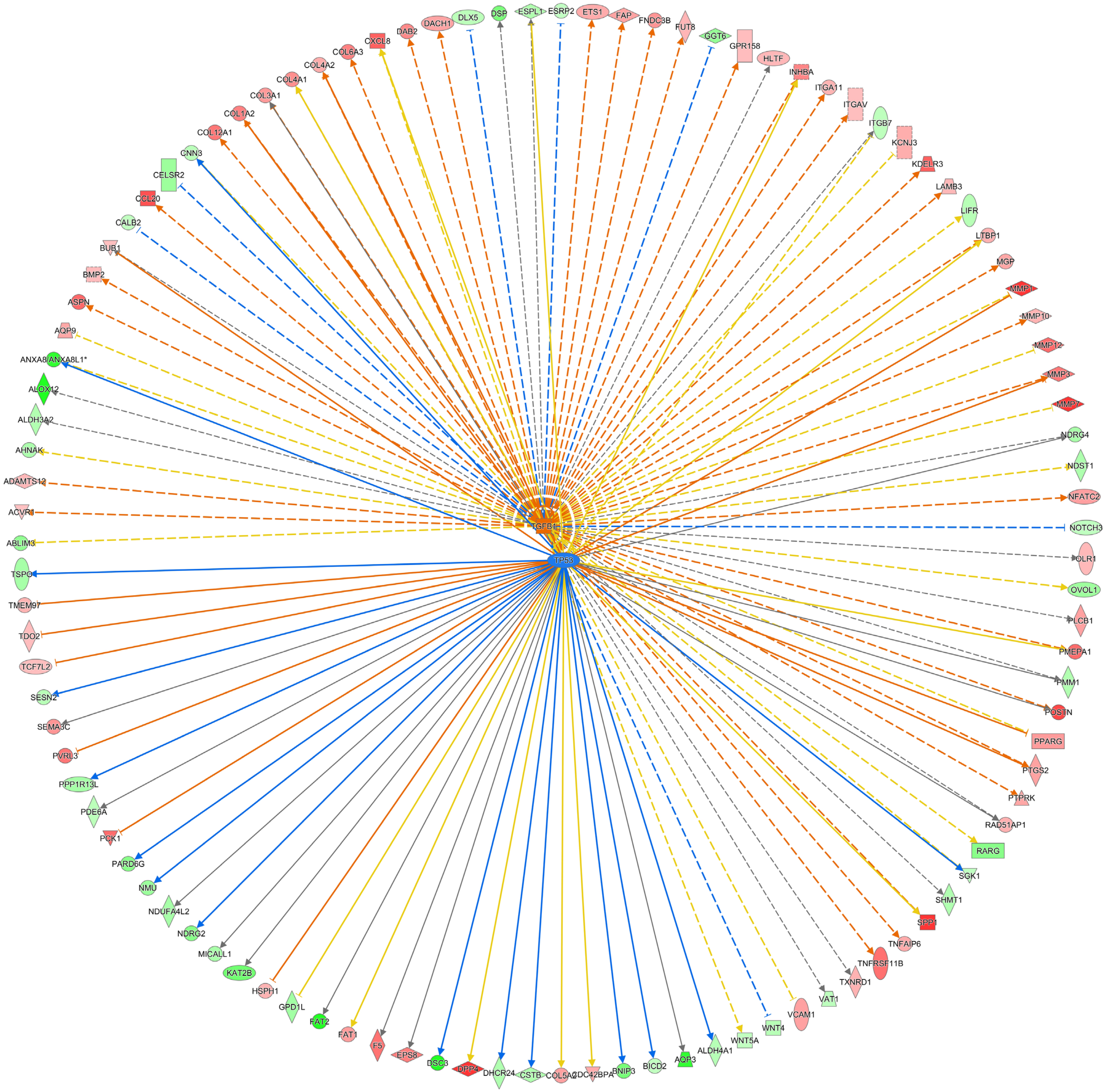

Supplementary Figure S2. Examples of pathway analyses. The results demonstrate upstream activation of TGFB1 and inhibition of TP53 pathways and their target genes.

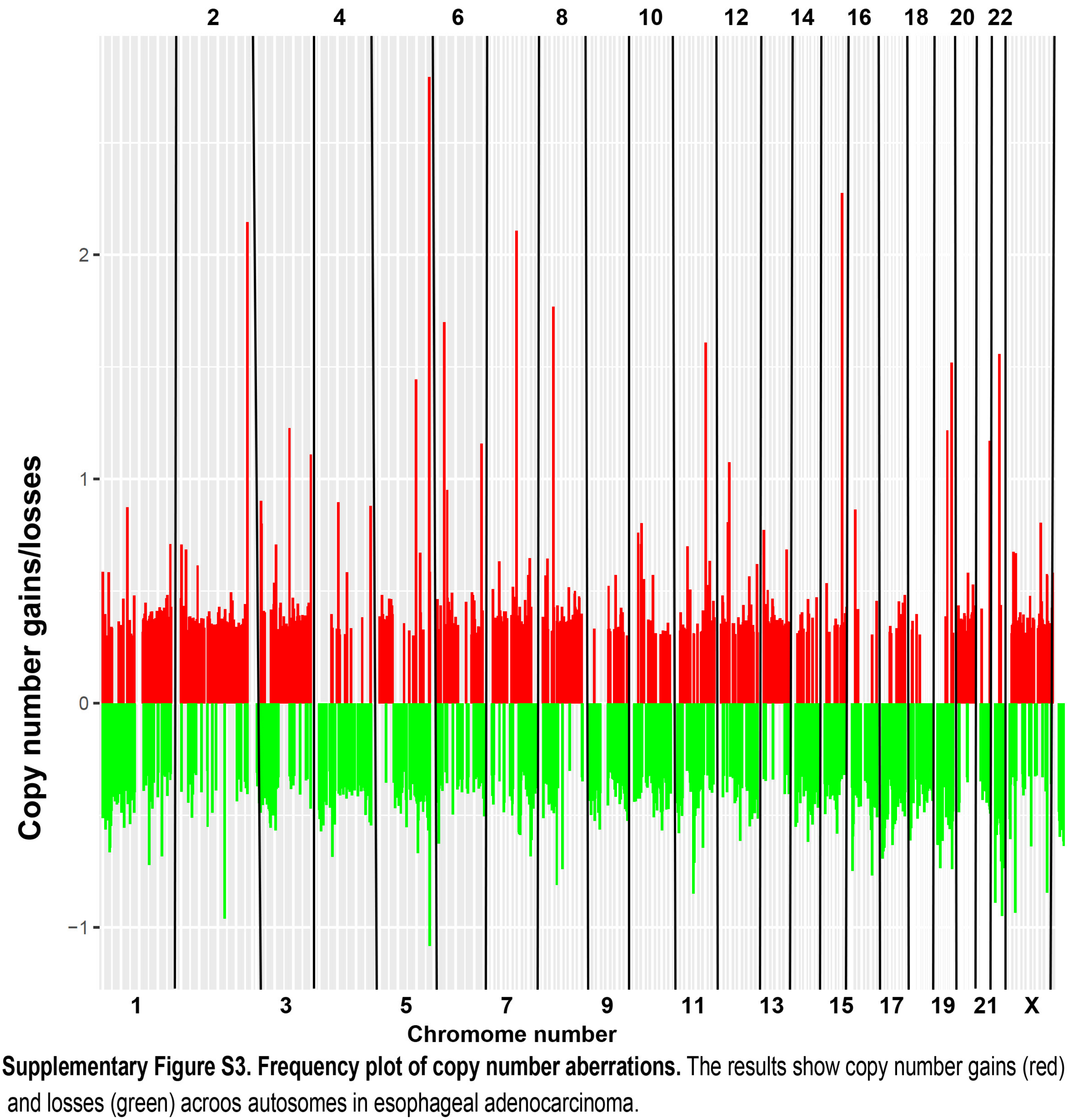

Supplementary Figure S3. Frequency plot of copy number aberrations. The results show copy number gains (red) and losses (green) acroos autosomes in esophageal adenocarcinoma.

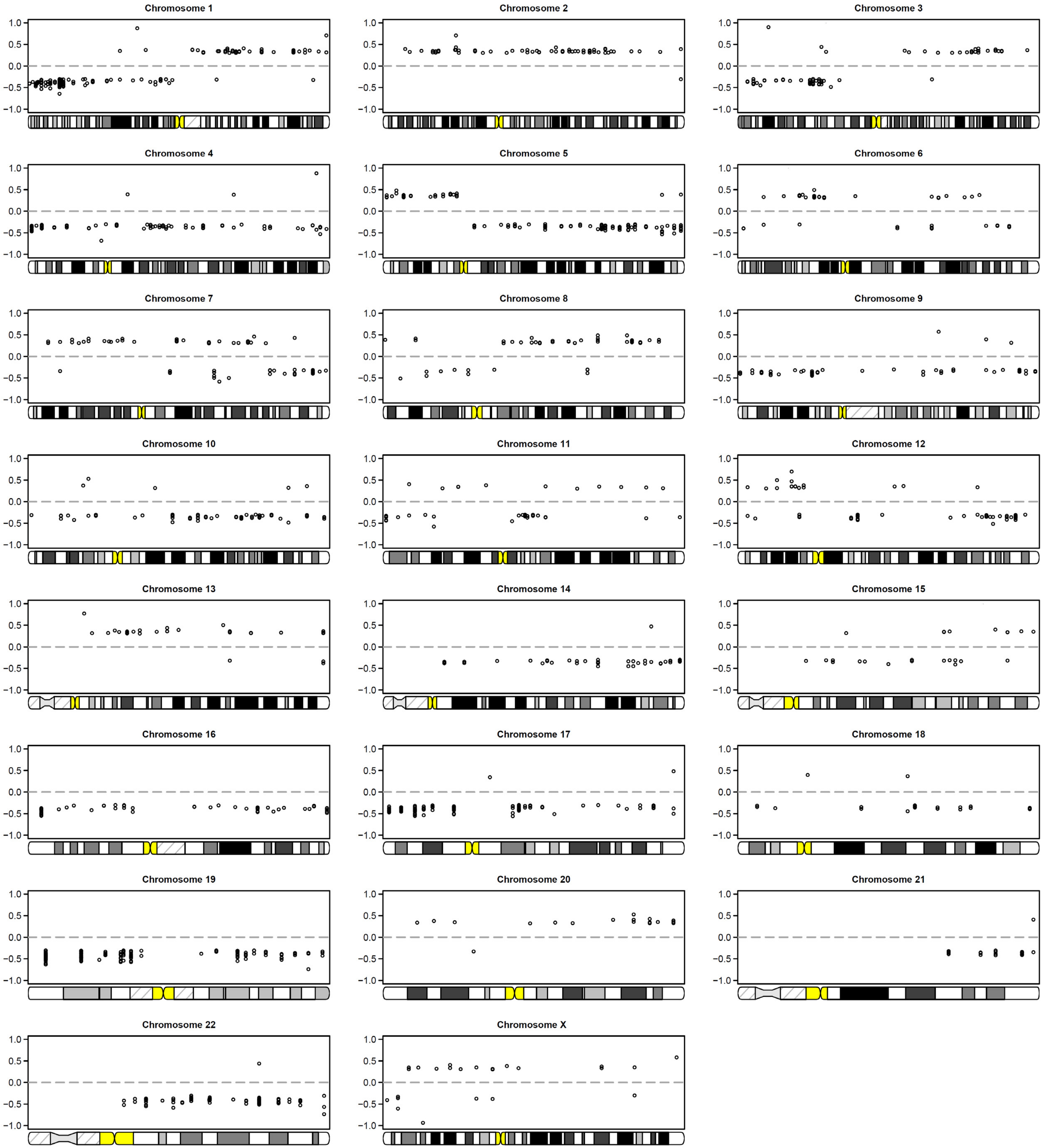

Supplemntary Figure S4. Identification of genomic hot spots in EAC. This figure identifies hot spots in chromosomes where copy number correlate well with gene expression (Y chromosome is excluded). The Y-axis is the copy number fold change between tumor and normal samples.

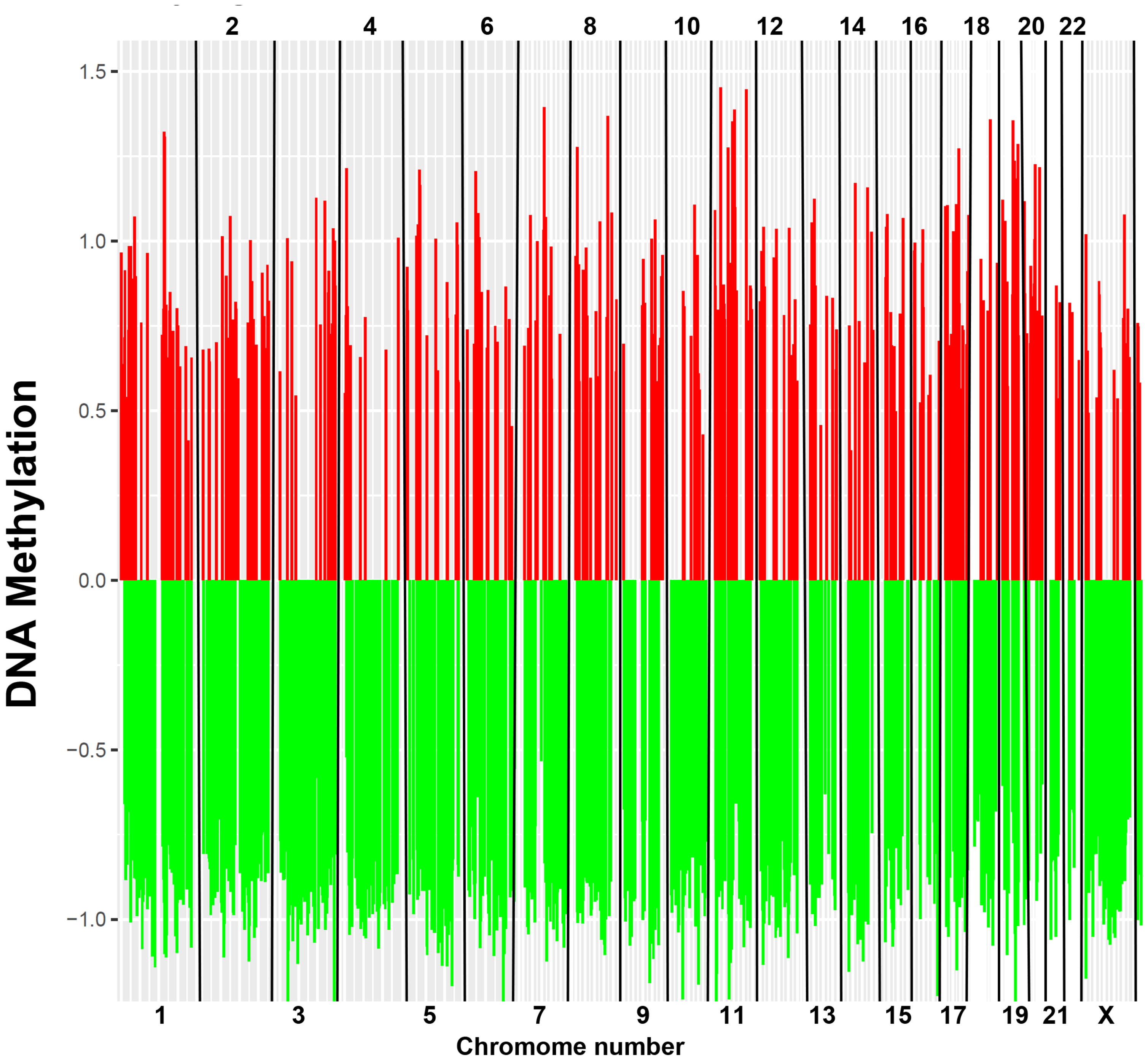

Supplementary Figure S5. Frequency plot of abnormal methylation events. The results show hyper- and hypo-methylation events in esophageal tumors. Hypermathylation is shown in red color whereas hypomethylation is shown in green color.

Supplementary Tables

Integrated molecular analysis reveals complex interactions between genomic and epigenomic alterations in esophageal adenocarcinomas

DunFa Peng[1#]*, Yan Guo[#2,3], Heidi Chen[2,3], Shilin Zhao[2,3], Kay Washington[4], TianLing Hu[1], Yu Shyr[2], and Wael El-Rifai[1,3,5]*

[1]Department of Surgery and [4]Department of Pathology, Vanderbilt University Medical Center, Nashville, Tennessee, USA. [2]Department of Biostatistics. [3]Department of Cancer Biology, Vanderbilt University, Nashville, Tennessee, USA. [5]Department of Veterans Affairs, Tennessee Valley Healthcare System, Nashville, Tennessee, USA

[#] Equal contribution

Table S1. Sample Description

Sample ID	Site	Histology	Age	Sex	Differentiati	Grade	TNM Stage
**484N**	esophagus	normal	54	M			
**484T**	esophagus	tumor	54	M	well	G1	pT1N0Mx
**05-409NS**	esophagus	normal	58	M			
**05-409T**	esophagus	tumor	58	M	poor	G4	T3N1cMx
**05-195NS**	esophagus	normal	59	M			
**05-195T**	esophagus	tumor	59	M	poor	G3	T3N1cMx
**05-158NS**	esophagus	normal	68	M			
**05-158T**	esophagus	tumor	68	M	well	G1	T3N0Mx
**06-144 NS**	esophagus	normal	72	M			
**06-144 T**	GEJ	tumor	72	M	poor	G3	T3N1Mx
**05-460 NS**	esophagus	normal	62	M			
**05-460 T**	GEJ	tumor	62	M	poor	G3	T3N1bMx
**06-469 T**	esophagus	tumor	NA	NA	moderate	G2	T3N1bMx
**06-426 T**	esophagus	tumor	NA	NA	moderate	G2	T2N0Mx
**07-487 NS**	esophagus	normal	NA	NA			
**07-487 T**	esophagus	tumor	NA	NA	moderate	G2	T1b N1a MX
**07-611 NS**	esophagus	normal	NA	NA			
**07-611 T**	esophagus	tumor	NA	NA	poor	G3	T3 N0 MX
**07-700 NS**	esophagus	normal	NA	NA			
**07-700 T**	esophagus	tumor	NA	NA	poor	G3	T1b N0 MX
**08-436 T**	esophagus	tumor	NA	NA	poor	G3	T3 N1 MX

Supplementary Table S2. Gene expression, DNA copy number, and DNA methylation signatures in EAC

symbol	GeneName	Cytoband	affy_log2_foldchg	affy_Praw	affy_PFDR	aCGH_log2_foldchg	aCGH_Praw	aCGH_PFDR	methy_log2_foldchg	methy_peak_score	methy_counts	overlap
MUC13	mucin 13, cell surface associated	3q21.2	5.856	0	0							1
A2BP1	Data not found					0.332	0.0332	0.1155	-1.127	3.80667	6	2
TSPAN8	tetraspanin 8	12q14.1-q21.1	5.48	0	1.00E-04				-0.697	2.99429	7	2
A2ML1	alpha-2-macroglobulin-like 1	12p13.31	-5.097	1.00E-05	3.00E-04							1
A4GALT	alpha 1,4-galactosyltransferase	22q13.2	-0.622	0.01232	0.0673	-0.449	3.00E-05	0.0044				2
AAA1	aortic aneurysm, familial abdominal 1	19q13				0.317	0.00136	0.0203	-0.933	3.11	5	2
AACS	acetoacetyl-CoA synthetase	12q24.31	-0.512	0.00601	0.0414	-0.309	7.00E-05	0.0059				2
AADACL1	Data not found								-0.798	3.266	5	1
AADACL2	arylacetamide deacetylase-like 2	3q25.1				0.384	0.00046	0.0124				1
AADACL4	arylacetamide deacetylase-like 4	1p36.21				-0.3475	0.005535	0.03855				1
AADAT	In multiple Geneids								-0.659	2.37167	6	1
OLFM4	olfactomedin 4	13q14.3	5.458	1.00E-05	4.00E-04	0.358	0.00075	0.0154				2
SPINK1	serine peptidase inhibitor, Kazal type 1	5q32	4.621	0	0				-0.769	2.86	8	2
MUC3B	In multiple Geneids		4.59	0	0							1
CTSE	cathepsin E	1q31	4.563	1.00E-05	4.00E-04							1
AANAT	aralkylamine N-acetyltransferase	17q25	-0.32	0.00461	0.0346							1
EPCAM	epithelial cell adhesion molecule	2p21	4.53	0	0							1
AARSD1	alanyl-tRNA synthetase domain containing 1	17q21.31				-0.397333333	0.002603333	0.0247				1
AASDH	aminoadipate-semialdehyde dehydrogenase	4q12							-1.028	3.18667	6	1
AASS	aminoadipate-semialdehyde synthase	7q31.3	-0.512	0.01621	0.0806							1
SLC44A4	solute carrier family 44, member 4	6p21.3	4.511	0	0							1
AATK	apoptosis-associated tyrosine kinase	17q25.3							0.712	2.81833	6	1
ABAT	4-aminobutyrate aminotransferase	16p13.2				-0.326	0.0133	0.0654				1
ABCA1	ATP-binding cassette, sub-family A (ABC1), member 1	9q31.1				-0.356	0.00018	0.0083				1
ABCA10	ATP-binding cassette, sub-family A (ABC1), member 10	17q24							-1.036	3.64875	8	1
ABCA11P	ATP-binding cassette, sub-family A (ABC1), member 11, pseudogene	4p16.3				-0.384	0.00013	0.0073				1
ABCA12	ATP-binding cassette, sub-family A (ABC1), member 12	2q34				0.33	0.001235	0.01755	-0.726	2.99	7	2
ABCA13	ATP-binding cassette, sub-family A (ABC1), member 13	7p12.3				0.404144928	0.013238406	0.062972464				1
ABCA17P	In multiple Geneids					-0.3582	0.018978	0.07941				1
ABCA3	ATP-binding cassette, sub-family A (ABC1), member 3	16p13.3				-0.452875	0.01425375	0.062825	0.636	2.6	5	2
ABCA5	ATP-binding cassette, sub-family A (ABC1), member 5	17q24.3	-0.712	0.00288	0.0248							1
ABCA6	ATP-binding cassette, sub-family A (ABC1), member 6	17q24.3				0.313	0.0059	0.0428				1
ABCA7	ATP-binding cassette, sub-family A (ABC1), member 7	19p13.3				-0.4535	0.0041075	0.031075				1
ABCA8	ATP-binding cassette, sub-family A (ABC1), member 8	17q24							-0.823	3.47857	7	1
ABCB1	ATP-binding cassette, sub-family B (MDR/TAP), member 1	7q21.12				0.304	0.0055	0.0412	-0.462	2.448	5	2
ABCB11	ATP-binding cassette, sub-family B (MDR/TAP), member 11	2q24				0.35975	1.50E-05	0.00265				1
ABCB4	ATP-binding cassette, sub-family B (MDR/TAP), member 4	7q21.1				0.339	0.00534	0.0406				1
ABCB5	In multiple Geneids								-0.92	2.96833	6	1
ABCB8	ATP-binding cassette, sub-family B (MDR/TAP), member 8	7q36	-0.42	0.00221	0.0205	-0.322	0.00397	0.0347				2
ABCB9	ATP-binding cassette, sub-family B (MDR/TAP), member 9	12q24				-0.337	0.004626667	0.033366667				1
ABCC1	ATP-binding cassette, sub-family C (CFTR/MRP), member 1	16p13.1				-0.383	0.00133	0.0201				1
ABCC11	ATP-binding cassette, sub-family C (CFTR/MRP), member 11	16q12.1							-0.875	2.99944	9	1
ABCC13	ATP-binding cassette, sub-family C (CFTR/MRP), member 13, pseudogene	21q11.2				-0.331	0.00687	0.0466				1
AGR2	anterior gradient 2 homolog (Xenopus laevis)	7p21.3	4.501	1.00E-05	3.00E-04							1
MUC17	mucin 17, cell surface associated	7q22.1	4.474	1.00E-05	3.00E-04							1
PROM1	prominin 1	4p15.32	4.144	0	0							1
ABCC5	ATP-binding cassette, sub-family C (CFTR/MRP), member 5	3q27	-0.726	0.01728	0.0841							1
ABCC6	ATP-binding cassette, sub-family C (CFTR/MRP), member 6	16p13.1				-0.33	0.0301	0.1088				1
ABCC6P1	ATP-binding cassette, sub-family C, member 6 pseudogene 1	16p12.3				-0.368	0.00216	0.0255				1
MMP1	matrix metallopeptidase 1 (interstitial collagenase)	11q22.3	3.956	0	0				-0.702	3.402	5	2
ABCC8	ATP-binding cassette, sub-family C (CFTR/MRP), member 8	11p15.1				-0.403	0.00316	0.0308				1
CDH17	cadherin 17, LI cadherin (liver-intestine)	8q22.1	3.882	0.00013	0.0027	0.372333333	0.000756667	0.0145	-0.848	3.16444	9	3
SPP1	secreted phosphoprotein 1	4q22.1	3.811	0	1.00E-04				-0.728	2.65167	6	2
ABCD4	ATP-binding cassette, sub-family D (ALD), member 4	14q24.3				-0.48175	0.0004475	0.010975				1
ABCE1	ATP-binding cassette, sub-family E (OABP), member 1	4q31							-0.625	2.76778	9	1
LGALS4	lectin, galactoside-binding, soluble, 4	19q13.2	3.81	1.00E-05	3.00E-04							1
ABCF2	In multiple Geneids								-0.984	2.59	5	1
TM4SF20	transmembrane 4 L six family member 20	2q36.3	3.729	1.00E-04	0.0023				-0.763	2.662	5	2
ABCG2	ATP-binding cassette, sub-family G (WHITE), member 2	4q22				-0.359333333	0.006968333	0.03825				1
ABCG4	ATP-binding cassette, sub-family G (WHITE), member 4	11q23.3	-0.676	0.00031	0.005							1
GOLM1	golgi membrane protein 1	9q21.33	3.712	0	0							1
ABHD12	abhydrolase domain containing 12	20p11.21	-0.362	0.00939	0.056							1
ABHD14B	abhydrolase domain containing 14B	3p21.2				-0.401	0.00024	0.0094				1
VIL1	villin 1	2q35	3.532	0	0							1
ABHD17A	Data not found		-0.425333333	0.00252	0.0205							1
ABHD17B	Data not found		-0.431	0.01296	0.0697							1

DPP4	dipeptidyl-peptidase 4	2q24.3	3.484	0	0	0.342833333	0.000223333	0.007916667	-0.928	3.07143	7	3
ABHD4	abhydrolase domain containing 4	14q11.2	-0.458	0.04232	0.1509	-0.3595	0.00087	0.0162				2
ABHD5	abhydrolase domain containing 5	3p21	-1.536	0	1.00E-04							1
ABHD6	abhydrolase domain containing 6	3p14.3				-0.38925	0.00176	0.01855				1
ABHD8	abhydrolase domain containing 8	19p13.11	-0.185	0.03849	0.1421	-0.414	0.00219	0.0256				2
ABI1	abl-interactor 1	10p11.2	-0.701	0.00136	0.0145	-0.305	0.01783	0.0797				2
MMP7	matrix metallopeptidase 7 (matrilysin, uterine)	11q21-q22	3.445	0.00061	0.0083				-0.791	2.862	5	2
ABI3BP	ABI family, member 3 (NESH) binding protein	3q12				0.333	0.017213333	0.075533333				1
ABL1	c-abl oncogene 1, non-receptor tyrosine kinase	9q34.1				-0.401	0.006925	0.03975				1
TSPAN1	tetraspanin 1	1p34.1	3.38	1.00E-05	3.00E-04							1
ABLIM1	actin binding LIM protein 1	10q25							-0.583	2.538	5	1
ABLIM2	actin binding LIM protein family, member 2	4p16.1	-0.701	0.00742	0.0478	-0.400647059	0.001403529	0.017211765				2
ABLIM3	actin binding LIM protein family, member 3	5q32	-1.801	0.00014	0.0029	-0.3175	0.002525	0.0272				2
ABR	active BCR-related	17p13.3	-0.599	0.01955	0.0913	-0.4299375	0.012906875	0.06003125				2
HNF1A-AS1	HNF1A antisense RNA 1 (non-protein coding)	12q24.31	3.378	1.00E-05	3.00E-04							1
ABTB2	ankyrin repeat and BTB (POZ) domain containing 2	11p13				-0.302	0.00018	0.0084				1
TMC5	transmembrane channel-like 5	16p12.3	3.362	0	1.00E-04							1
MUC12	mucin 12, cell surface associated	7q22	3.344666667	0.003053333	0.018566667							1
ACACA	acetyl-CoA carboxylase alpha	17q21				-0.631	0.00556	0.03885				1
ACACB	acetyl-CoA carboxylase beta	12q24.11				0.44	0.00127	0.0197				1
ACAD10	In multiple Geneids					-0.384	0.000655	0.01215				1
ACAD11	acyl-CoA dehydrogenase family, member 11	3q22.1				0.47	0	0.0014				1
ACAD8	acyl-CoA dehydrogenase family, member 8	11q25	-0.427	0.00741	0.0477							1
ACADM	acyl-CoA dehydrogenase, C-4 to C-12 straight chain	1p31	-1.733	0	0	-0.315	0.00155	0.0216				2
ACADVL	acyl-CoA dehydrogenase, very long chain	17p13.1	-0.538	0.00057	0.008							1
CLDN18	In multiple Geneids		3.31	0.00146	0.0153				-0.646	2.95833	6	2
ACAP1	ArfGAP with coiled-coil, ankyrin repeat and PH domains 1	17p13.1				-0.452	0.00051	0.0129				1
ACAP2	ArfGAP with coiled-coil, ankyrin repeat and PH domains 2	3q29	-0.626	0.00362	0.0292							1
ACAP3	ArfGAP with coiled-coil, ankyrin repeat and PH domains 3		-0.541	0.00581	0.0405							1
LGR5	leucine-rich repeat containing G protein-coupled receptor 5	12q22-q23	3.31	2.00E-05	6.00E-04							1
CLDN3	claudin 3	7q11.23	3.297	0	0							1
ACCN1	Data not found					0.3725	0.00462	0.03745				1
ACCN3	Data not found					-0.31	0.00025	0.0096				1
ACCN5	Data not found								-0.793	2.94571	7	1
ACCSL	1-aminocyclopropane-1-carboxylate synthase homolog (Arabidopsis)(non-fun	11p11.2	-0.27	0.00954	0.0566							1
TMC7	transmembrane channel-like 7	16p12.3	3.281	0	0							1
DMBT1	deleted in malignant brain tumors 1	10q26.13	3.257	0.00039	0.006	0.359	0.000796667	0.015733333				2
ACE2	angiotensin I converting enzyme (peptidyl-dipeptidase A) 2	Xp22				0.305	0.00561	0.0417				1
ACER1	alkaline ceramidase 1	19p13.3	-3.534	0	0	-0.4	0.00054	0.0133				2
ACER2	alkaline ceramidase 2	9p22.1				-0.454	0.001376	0.01484				1
ACER3	alkaline ceramidase 3	11q13.5	-0.517	0.03576	0.1353							1
ACF	Asymmetric crying facies (Cayler cardiofacial syndrome)	22q11							-0.874	3.20444	9	1
KIAA1324	KIAA1324	1p13.3	3.219	0.00037	0.0057							1
ACIN1	apoptotic chromatin condensation inducer 1	14q11.2				-0.339	0.00329	0.02785				1
REG4	regenerating islet-derived family, member 4	1p13.1-p12	3.191	0.00111	0.0126				-1.141	4.778	5	2
ACMSD	aminocarboxymuconate semialdehyde decarboxylase	2q21.3							-0.863	3.16125	8	1
ACO1	aconitase 1, soluble	9p21.1				-0.344875	0.004065	0.0312125				1
ACO2	aconitase 2, mitochondrial	22q13.2				-0.443916667	0.001888333	0.017766667				1
ACOT11	acyl-CoA thioesterase 11	1p32.3				-0.4205	0.004733333	0.033316667				1
ACOT12	acyl-CoA thioesterase 12	5q14.1				-0.3248	0.000106	0.00572				1
ACOT4	acyl-CoA thioesterase 4	14q24.3	-0.343	0.03965	0.1447							1
ACOT7	acyl-CoA thioesterase 7	1p36				-0.415666667	0.002943333	0.02				1
SERPINA1	serpin peptidase inhibitor, clade A (alpha-1 antiproteinase, antitrypsin), meml	14q32.1	3.158	0	1.00E-04							1
ACOX1	In multiple Geneids		-0.813	0.00416	0.0322	-0.3675	0.00458	0.0342				2
ACOX2	acyl-CoA oxidase 2, branched chain	3p14.3				-0.415	0.001422	0.01602				1
ACOX3	acyl-CoA oxidase 3, pristanoyl	4p15.3	-1.511	0	0	-0.383	0.00226875	0.020875				2
ACOXL	acyl-CoA oxidase-like	2q13				0.3545	0.00096	0.014025				1
PLA2G10	phospholipase A2, group X	16p13.1-p12	3.123	1.00E-05	4.00E-04							1
CYP2B6	cytochrome P450, family 2, subfamily B, polypeptide 6	19q13.2	3.084	0.00029	0.0048							1
ACP5	acid phosphatase 5, tartrate resistant	19p13.3-p13.2				-0.513	0.00234	0.0265				1
GDA	guanine deaminase	9q21.13	3.044	0	1.00E-04							1
RHPN2	rhophilin, Rho GTPase binding protein 2	19q13.11	3.033	0	0							1
ACPP	acid phosphatase, prostate	3q22.1	-2.544	8.00E-05	0.0018							1
ACPT	acid phosphatase, testicular	19q13.4	-0.302	0.00312	0.0263	-0.415	0.00011	0.0068				2
TMEM176A	transmembrane protein 176A	7q36.1	3.027	0	1.00E-04							1
ACRC	acidic repeat containing	Xq13.1				-0.326	0.00166	0.0224				1
ACSBG2	acyl-CoA synthetase bubblegum family member 2	19p13.3				-0.377444444	0.003377778	0.029277778				1
ACSF2	acyl-CoA synthetase family member 2	17q21.33				-0.335	0.01198	0.0633				1
ACSF3	acyl-CoA synthetase family member 3	16q24.3				0.456	9.00E-05	0.0064				1

ACSL1	acyl-CoA synthetase long-chain family member 1	4q35	-0.769	0.00231	0.0212	-0.43	0.003291818	0.025772727				2
ACSL3	acyl-CoA synthetase long-chain family member 3	2q34-q35	-0.397	0.02568	0.1092							1
EPS8L3	EPS8-like 3	1p13.3	3.007	0	0							1
TRIM31	tripartite motif containing 31	6p21.3	3.002	1.00E-05	0.00045							1
ACSL6	acyl-CoA synthetase long-chain family member 6	5q31				-0.39	0.002005	0.0186				1
ACSM2	Data not found								-0.876	3.27167	6	1
ACSM3	acyl-CoA synthetase medium-chain family member 3	16p13.11							-0.779	2.982	5	1
POSTN	periostin, osteoblast specific factor	13q13.3	2.984	2.00E-05	6.00E-04	0.315	0.00314	0.0307	-0.868	2.75833	6	3
ACTBL1	Data not found								0.891	2.81833	6	1
CST1	cystatin SN	20p11.21	2.972	0.00117	0.0131							1
ACTL6B	actin-like 6B	7q22	-0.201	0.03349	0.1295	-0.33	0.02074	0.0871				2
ACTL7B	actin-like 7B	9q31							0.658	2.604	5	1
ACTL8	actin-like 8	1p36.2-p35				-0.353285714	0.001482857	0.0172				1
LOC93432	maltase-glucoamylase (alpha-glucosidase) pseudogene	7q34	2.954	1.00E-05	5.00E-04							1
ACTN3	actinin, alpha 3	11q13.1				-0.3915	0.00128	0.0159				1
ACTN4	actinin, alpha 4	19q13				-0.318333333	0.008193333	0.049033333				1
ACTR10	actin-related protein 10 homolog (S. cerevisiae)	14q23.1	-0.42	0.02703	0.1127							1
ACTR1A	ARP1 actin-related protein 1 homolog A, centractin alpha (yeast)	10q24.32	-0.487	0.00156	0.016	-0.357	0.00191	0.023466667				2
ACTR3	ARP3 actin-related protein 3 homolog (yeast)	2q14.1				-0.487	0.00011	0.0069				1
ACTR3BP2	ARP3 actin-related protein 3 homolog B (yeast) pseudogene 2	2p11.1	-0.333	0.02852	0.11605							1
RBP4	retinol binding protein 4, plasma	10q23-q24	2.921	0.00029	0.0048							1
ACTR8	ARP8 actin-related protein 8 homolog (yeast)								-0.962	2.852	5	1
ACTRT1	actin-related protein T1	Xq25							-0.895	2.695	6	1
ACTRT2	actin-related protein T2	1p36.32	-0.27	0.01218	0.0669							1
HGD	homogentisate 1,2-dioxygenase	3q13.33	2.866	2.00E-05	6.00E-04							1
PLA2G7	phospholipase A2, group VII (platelet-activating factor acetylhydrolase, plasm	6p21.2-p12	2.843	0	0	0.3195	0.00337	0.03135				2
ACVR2A	activin A receptor, type IIA	2q22.3				0.331666667	6.33E-05	0.004966667				1
CCL20	chemokine (C-C motif) ligand 20	2q33-q37	2.813	0	2.00E-04				-0.574	2.692	5	2
PIGR	polymeric immunoglobulin receptor	1q31-q41	2.808	0.00534	0.0382							1
VCAN	versican	5q14.3	2.792	1.00E-05	4.00E-04							1
SULT1C2	sulfotransferase family, cytosolic, 1C, member 2	2q12.3	2.777	0.00023	0.004							1
ACYP1	acylphosphatase 1, erythrocyte (common) type	14q24.3				-0.301	0.01254	0.065				1
ACYP2	acylphosphatase 2, muscle type	2p16.2				0.314	0.001	0.0177				1
ADAD2	adenosine deaminase domain containing 2	16q24.1	-1.549	1.00E-05	5.00E-04	-0.317	0.00483	0.0385				2
ANPEP	alanyl (membrane) aminopeptidase	15q25-q26	2.765	3.00E-05	8.00E-04							1
ADAM11	ADAM metallopeptidase domain 11	17q21.3				-0.4075	0.01234	0.05725				1
KRT8	keratin 8	12q13	2.762	0	0							1
ADAM15	ADAM metallopeptidase domain 15	1q21.3	-0.413	0.01335	0.0708							1
ADAM18	ADAM metallopeptidase domain 18	8p11.22				0.311	0.003375	0.02565				1
ADAM19	ADAM metallopeptidase domain 19	5q33.3				-0.360111111	0.00141	0.013533333				1
ADAM20	ADAM metallopeptidase domain 20	14q24.1							-0.954	3.56333	6	1
ADAM21	ADAM metallopeptidase domain 21	14q24.1							-0.792	2.772	5	1
ADAM21P	Data not found					-0.344	3.00E-04	0.0104				1
HNF4G	hepatocyte nuclear factor 4, gamma	8q21.11	2.752	0	0	0.32	0.00359	0.0329	-0.823	3.0575	8	3
ADAM23	ADAM metallopeptidase domain 23	2q33	-0.699	0.04588	0.1587							1
ADAM28	ADAM metallopeptidase domain 28	8p21.2							-1.011	3.08857	7	1
ADAM29	ADAM metallopeptidase domain 29	4q34							-0.752	2.81333	9	1
CREB3L1	cAMP responsive element binding protein 3-like 1	11p11.2	2.744	4.00E-05	0.0012							1
ADAM7	ADAM metallopeptidase domain 7	8p21.2							-0.797	3.34	9	1
PLEKHS1	pleckstrin homology domain containing, family S member 1	10q25.3	2.728	0.00027	0.0045							1
SLC39A14	solute carrier family 39 (zinc transporter), member 14	8p21.3	2.718	0	0							1
REG1A	regenerating islet-derived 1 alpha	2p12	2.703	0.01514	0.0771				-0.978	3.07143	7	2
ADAMTS1	ADAM metallopeptidase with thrombospondin type 1 motif, 1	21q21.2				-0.3695	0.00195	0.02405				1
ADAMTS10	ADAM metallopeptidase with thrombospondin type 1 motif, 10	19p13.2				-0.479333333	0.002283333	0.023488889				1
GUCY2C	guanylate cyclase 2C (heat stable enterotoxin receptor)	12p12	2.699	2.00E-04	0.0037							1
ADAMTS13	ADAM metallopeptidase with thrombospondin type 1 motif, 13	9q34				-0.303	0.00372	0.0336				1
F2RL2	coagulation factor II (thrombin) receptor-like 2	5q13	2.692	0	0				-0.725	2.54167	6	2
ADAMTS16	ADAM metallopeptidase with thrombospondin type 1 motif, 16	5p15				0.374333333	0.001616667	0.019366667				1
ADAMTS17	ADAM metallopeptidase with thrombospondin type 1 motif, 17	15q24				0.320333333	0.01021	0.045233333				1
ADAMTS18	ADAM metallopeptidase with thrombospondin type 1 motif, 18	16q23				-0.346	0.00182	0.0234				1
GDF15	growth differentiation factor 15	19p13.11	2.657	0	0							1
ADAMTS20	ADAM metallopeptidase with thrombospondin type 1 motif, 20	12q12				0.357	0.00511	0.0397				1
FKBP10	FK506 binding protein 10, 65 kDa	17q21.2	2.642	0	0							1
SI	sucrase-isomaltase (alpha-glucosidase)	3q25.2-q26.2	2.64	0.00292	0.0251	0.3425	0.0019	0.019866667	-0.982	3.64714	7	3
GPR128	G protein-coupled receptor 128	3q12.2	2.62	1.00E-05	3.00E-04							1
UBD	ubiquitin D	6p21.3	2.613	2.50E-05	0.00075							1
AGR3	anterior gradient 3 homolog (Xenopus laevis)	7p21.1	2.603	0.00032	0.0052							1
ADAMTS8	ADAM metallopeptidase with thrombospondin type 1 motif, 8	11q25	-0.29	0.02153	0.0974							1
MISP	Data not found		2.579	0	1.00E-04							1

ADAMTSL1	ADAMTS-like 1	9p21.3							-0.816	3.086	5	1
IL8	interleukin 8	4q13-q21	2.564	1.00E-05	4.00E-04				-0.869	2.59167	6	2
ADAMTSL3	ADAMTS-like 3	15q25.2				0.381	0.001506667	0.0165				1
ADAMTSL4	ADAMTS-like 4	1q21.3	-1.387	6.00E-05	0.0016							1
ADAMTSL5	ADAMTS-like 5	19p13.3	-0.765	0.00206	0.0195	-0.305	0.0144	0.0704				2
SLC6A20	solute carrier family 6 (proline IMINO transporter), member 20	3p21.3	2.564	4.00E-05	0.0012							1
KDELR3	KDEL (Lys-Asp-Glu-Leu) endoplasmic reticulum protein retention receptor 3	22q13.1	2.553	0	1.00E-04	0.438	2.00E-05	0.0036				2
ADARB1	adenosine deaminase, RNA-specific, B1	21q22.3				-0.360833333	0.002583333	0.022516667	0.819	2.555	6	2
ADARB2	adenosine deaminase, RNA-specific, B2	10p15.3	-0.536	0.00014	0.0029	-0.3125	0.02054	0.08145				2
PIP5K1B	phosphatidylinositol-4-phosphate 5-kinase, type I, beta	9q13	2.552	3.00E-05	9.00E-04				-0.889	2.53	6	2
ADAT3	adenosine deaminase, tRNA-specific 3	19p13.3				-0.463	0.001915	0.02105				1
ADCK1	aarF domain containing kinase 1	14q24.3				-0.366333333	0.002095	0.023091667				1
ADCK2	aarF domain containing kinase 2	7q34				-0.3422	0.012204	0.05988				1
FAR2	fatty acyl CoA reductase 2	12p11.22	2.551	0	0	0.328	0.000435	0.0121				2
ADCK4	aarF domain containing kinase 4	19q13.2				-0.383	0.000645	0.0142				1
ADCY2	In multiple Geneids					0.355378378	0.00351027	0.028810811				1
ADCY5	adenylate cyclase 5	3q21.1				-0.345	0.0037	0.0335				1
ERN2	endoplasmic reticulum to nucleus signaling 2	16p12.2	2.55	1.00E-05	3.00E-04							1
ADCY7	adenylate cyclase 7	16q12.1	-0.505	0.00364	0.0293	-0.338	0.00264	0.0282				2
ADCY8	adenylate cyclase 8 (brain)	8q24				0.416413043	0.018691957	0.077063043				1
ADCY9	adenylate cyclase 9	16p13.3				-0.430516129	0.012275484	0.0599				1
ADCYAP1R1	adenylate cyclase activating polypeptide 1 (pituitary) receptor type I	7p14	-0.262	0.01963	0.0915							1
ADD1	adducin 1 (alpha)	4p16.3				-0.3835	0.0015275	0.018525				1
ADH1A	alcohol dehydrogenase 1A (class I), alpha polypeptide	4q23							-0.861	2.48286	7	1
ADH1B	alcohol dehydrogenase 1B (class I), beta polypeptide	4q23							-0.984	3.33833	6	1
ADH1C	alcohol dehydrogenase 1C (class I), gamma polypeptide	4q23							-0.784	2.65	6	1
ADH4	alcohol dehydrogenase 4 (class II), pi polypeptide	4q22							-0.936	3.4925	8	1
ADH5	alcohol dehydrogenase 5 (class III), chi polypeptide	4q23				-0.339	0.00331	0.0316				1
HNF1B	HNF1 homeobox B	17q12	2.545	0	0							1
ADH7	alcohol dehydrogenase 7 (class IV), mu or sigma polypeptide	4q23-q24							-0.866	3.28	8	1
ADHFE1	alcohol dehydrogenase, iron containing, 1	8q13.1	-1.54	0.00027	0.0045							1
ADIPOQ	adiponectin, C1Q and collagen domain containing	3q27							-0.662	2.74	5	1
ADIPOR1	adiponectin receptor 1	1q32.1	-0.836	1.00E-05	4.00E-04							1
ADIPOR2	adiponectin receptor 2	12p13.31	-0.546	0.00377	0.03							1
ADIRF	Data not found		-1.39	8.00E-05	0.0018							1
ADK	adenosine kinase	10q22	-0.992	0.00017	0.0033	-0.332	1.00E-04	0.0065				2
CFTR	cystic fibrosis transmembrane conductance regulator (ATP-binding cassette su	7q31.2	2.521	1.00E-05	3.00E-04	0.317	2.00E-05	0.0036				2
ADORA2A	adenosine A2a receptor	22q11.23				-0.484	0	0.0018	0.618	2.56833	6	2
ADORA2B	adenosine A2b receptor	17p12				-0.35925	0.00244	0.023325				1
IL17RB	interleukin 17 receptor B	3p21.1	2.505	0	0							1
ADPRH	ADP-ribosylarginine hydrolase	3q13.31-q13.33							-0.962	2.62667	6	1
ADPRHL1	ADP-ribosylhydrolase like 1	13q34				-0.3105	0.035105	0.1195				1
ADPRHL2	ADP-ribosylhydrolase like 2	1p34.3				-0.328	0.00989	0.0569				1
ADPRM	Data not found		-0.338	0.01936	0.0908							1
ADRA1A	adrenoceptor alpha 1A	8p21.2	-0.187	0.02925	0.1185				0.526	2.61	5	2
ADRA1B	adrenoceptor alpha 1B	5q33.3				-0.3305	0.0033175	0.025925				1
ADRB2	adrenoceptor beta 2, surface	5q31-q32	-0.941	0	0							1
ADRBK1	adrenergic, beta, receptor kinase 1	11q13.1				-0.317	0.0104	0.0585				1
ADRBK2	adrenergic, beta, receptor kinase 2	22q12.1				-0.341666667	0.001383333	0.015966667				1
ADSL	adenylosuccinate lyase	22q13.2				-0.322	0.00605	0.0434				1
ADSSL1	adenylosuccinate synthase like 1	14q32.33							0.737	3.05	7	1
HSD17B11	hydroxysteroid (17-beta) dehydrogenase 11	4q22.1	2.499	0	0							1
USH1C	In multiple Geneids		2.492	0	0							1
AES	amino-terminal enhancer of split	19p13.3	-0.322	0.04732	0.1616	-0.6315	0.000925	0.01685				2
AFAP1	actin filament associated protein 1	4p16	-0.26	0.00521	0.0375	-0.378870968	0.002082258	0.020664516				2
SEMA4G	In multiple Geneids		2.486	0	0							1
AFAP1L2	actin filament associated protein 1-like 2	10q25.3				-0.354	0.00044	0.0104				1
AFF1	AF4/FMR2 family, member 1	4q21				-0.356111111	0.002047778	0.021488889				1
AFF2	AF4/FMR2 family, member 2	Xq28				0.355128571	0.004516	0.032544286				1
AFF3	AF4/FMR2 family, member 3	2q11.2-q12				0.315	0.00193	0.021833333				1
AFF4	AF4/FMR2 family, member 4	5q31	-0.521	0.00065	0.0086	-0.3764	0.002912	0.02622				2
AFG3L1	Data not found					-0.326	0.00359	0.033				1
AFM	afamin	4q13.3				0.306	0.00171	0.0227				1
AFMID	arylformamidase	17q25.3				-0.3475	0.007255	0.04685				1
AFP	alpha-fetoprotein	4q13.3							-0.779	3.15	8	1
AFTPH	aftiphilin	2p14				0.318	2.00E-05	0.0036				1
AGA	aspartylglucosaminidase	4q34.3	-1.295	0.00014	0.0028	-0.325	0.00054	0.0134				2
HNF4A	hepatocyte nuclear factor 4, alpha	20q13.12	2.475	0	0							1
AGAP11	ankyrin repeat and GTPase domain Arf GTPase activating protein 11	10q23.2	-0.615	0.00195	0.0188							1

AGAP3	ArfGAP with GTPase domain, ankyrin repeat and PH domain 3	7q36.1				-0.33	0.0031875	0.029925				1
AGAP5	ArfGAP with GTPase domain, ankyrin repeat and PH domain 5	10q22.2	-0.468	0.00498	0.0364							1
AGBL1	In multiple Geneids					0.349961538	0.005091538	0.033367308				1
AGBL2	ATP/GTP binding protein-like 2	11p11.2				-0.314	0.01424	0.07				1
AGT	angiotensinogen (serpin peptidase inhibitor, clade A, member 8)	1q42.2	2.454	7.00E-05	0.0016							1
AGFG2	ArfGAP with FG repeats 2	7q22.1	-0.87	0.00174	0.0174							1
AGGF1	angiogenic factor with G patch and FHA domains 1	5q13.3				-0.401	5.00E-05	0.0051				1
AGL	amylo-alpha-1, 6-glucosidase, 4-alpha-glucanotransferase	1p21							-0.777	2.91667	9	1
PKDCC	protein kinase domain containing, cytoplasmic homolog (mouse)	2p21	2.443	0	0							1
SLC7A7	solute carrier family 7 (amino acid transporter light chain, y+L system), memb	14q11.2	2.437	0	2.00E-04							1
SLC41A2	solute carrier family 41, member 2	12q23.3	2.434	4.00E-05	0.0012	0.333	7.00E-05	0.0059	-0.873	2.925	8	3
ASPN	asporin	9q22	2.427	8.00E-05	0.0019				-0.84	2.585	6	2
CMBL	carboxymethylenebutenolidase homolog (Pseudomonas)	5p15.2	2.42	0.00012	0.0025							1
AGPAT3	1-acylglycerol-3-phosphate O-acyltransferase 3	21q22.3	-0.387	0.01679	0.0825	-0.373153846	0.000891538	0.014053846				2
MMP12	matrix metallopeptidase 12 (macrophage elastase)	11q22.3	2.413	0.00097	0.0114				-0.837	2.93167	6	2
AGPAT9	1-acylglycerol-3-phosphate O-acyltransferase 9	4q21.23	-1.189	0.00244	0.022	-0.359	0.0033425	0.0282				2
BPIFB1	BPI fold containing family B, member 1	20q11.21	2.411	0.00251	0.0225							1
TOX3	TOX high mobility group box family member 3	16q12.1	2.398	1.00E-05	5.00E-04							1
LUM	lumican	12q21.3-q22	2.391	3.00E-05	9.00E-04				-0.664	2.53667	6	2
SLC6A14	solute carrier family 6 (amino acid transporter), member 14	Xq23	2.389	0.00057	0.0078	0.331	0.000533333	0.010933333				2
C12orf75	chromosome 12 open reading frame 75	12q23.3	2.383	0	1.00E-04							1
AGTPBP1	ATP/GTP binding protein 1	9q21.33				-0.305	0.00608	0.0436				1
AGTR1	angiotensin II receptor, type 1	3q24				0.349	0.008846667	0.0404				1
AGTR2	angiotensin II receptor, type 2	Xq22-q23				0.33	0.00119	0.0191	-0.851	2.895	6	2
AGTRAP	angiotensin II receptor-associated protein	1p36.22				-0.431	2.00E-04	0.00865				1
AGXT2	alanine--glyoxylate aminotransferase 2	5p13				0.331	0.01655	0.0749				1
AGXT2L1	alanine-glyoxylate aminotransferase 2-like 1	4q25				-0.374	0.001736667	0.018566667				1
AGXT2L2	alanine-glyoxylate aminotransferase 2-like 2	5q35.3				-0.443	0.001174	0.01482				1
PMEPA1	prostate transmembrane protein, androgen induced 1	20q13.31-q13.33	2.382	2.00E-05	7.00E-04	0.424636364	0.012002727	0.059490909				2
AHCY	adenosylhomocysteinase	20q11.22				-0.335	0.00011	0.0069				1
AHCYL2	adenosylhomocysteinase-like 2	7q32.1				-0.3195	0.0067525	0.044875				1
AHDC1	AT hook, DNA binding motif, containing 1	1p36.13	-1.168	0	0	-0.387	0.008843	0.05037				2
HHLA2	HERV-H LTR-associating 2	3q13.13	2.377	0.00014	0.0028	0.355	0.00282	0.0291	-0.902	3.5	5	3
AHNAK	In multiple Geneids		-1.365	6.00E-05	0.0015	-0.354857143	0.002792857	0.025285714				2
AHNAK2	AHNAK nucleoprotein 2	14q32.33	-1.99	0.00107	0.0122	-0.3	0.00958	0.0559				2
PCK1	phosphoenolpyruvate carboxykinase 1 (soluble)	20q13.31	2.377	0.00019	0.0035	0.345	0.01955	0.0841				2
AHRR	In multiple Geneids					0.386	0.00256	0.0277				1
LYZ	lysozyme	12q15	2.372	0.00071	0.0092							1
TGM2	transglutaminase 2 (C polypeptide, protein-glutamine-gamma-glutamyltransfe	20q12	2.362	5.00E-05	0.0013							1
AIF1	allograft inflammatory factor 1	6p21.3							0.833	3.262	5	1
AIF1L	allograft inflammatory factor 1-like	9q34.13-q34.3	-2.623	0	2.00E-04							1
AIFM2	apoptosis-inducing factor, mitochondrion-associated, 2	10q22.1				-0.305	0.00528	0.0403				1
AIFM3	apoptosis-inducing factor, mitochondrion-associated, 3	22q11.21				-0.449	0.00301	0.02475				1
AIM1	absent in melanoma 1	6q21							-1.032	2.795	6	1
AIM1L	In multiple Geneids		-1.741	0	2.00E-04	-0.349	0.00143	0.0209				2
CP	ceruloplasmin (ferroxidase)	3q23-q25	2.361	0.00245	0.0221							1
HEPH	In multiple Geneids		2.361	2.00E-05	6.00E-04	0.347166667	0.000675	0.013366667	-0.783	3.005	5	3
AJAP1	adherens junctions associated protein 1	1p36.32	-0.333	0.02407	0.1044	-0.369666667	0.001001667	0.013433333				2
AJUBA	ajuba LIM protein	14q11.2	-0.536	0.04313	0.1527							1
AK3	adenylate kinase 3	9p24.1	-0.771	1.00E-05	5.00E-04	-0.3215	0.002425	0.02635				2
AK3L1	Data not found					-0.3354	0.003906	0.02952				1
AK7	adenylate kinase 7	14q32.2				-0.35	7.00E-05	0.0059				1
AK8	adenylate kinase 8	9q34.13	-0.254	0.04638	0.1597							1
AK9	Data not found		-0.305	0.00934	0.0558							1
CDHR5	cadherin-related family member 5	11p15.5	2.36	0	1.00E-04							1
AKAP10	A kinase (PRKA) anchor protein 10	17p11.1				-0.3395	0.01054	0.056325				1
RARRES1	retinoic acid receptor responder (tazarotene induced) 1	3q25.32	2.342	2.00E-05	7.00E-04							1
AKAP12	A kinase (PRKA) anchor protein 12	6q24-q25				-0.352333333	0.006751667	0.040233333				1
C2	complement component 2	6p21.3	2.321	0.000435	0.0054							1
AKAP2	A kinase (PRKA) anchor protein 2	9q31.3							1.063	2.55	5	1
AKAP3	A kinase (PRKA) anchor protein 3	12p13.3							-0.722	2.607	5	1
SEL1L3	sel-1 suppressor of lin-12-like 3 (C. elegans)	4p15.2	2.312	0	2.00E-04							1
AKAP6	A kinase (PRKA) anchor protein 6	14q12				0.3215	0.002285	0.0245				1
AKAP7	A kinase (PRKA) anchor protein 7	6q23							-1.033	3.13	5	1
AKAP8	A kinase (PRKA) anchor protein 8	19p13.1				-0.418125	0.00321625	0.0271				1
TNFRSF11B	tumor necrosis factor receptor superfamily, member 11b	8q24	2.296	2.00E-05	6.00E-04	0.42175	0.008275	0.043475				2
AKIP1	A kinase (PRKA) interacting protein 1	11p15.3	-0.629	0.00155	0.016							1
AKIRIN1	akirin 1	1p34.3				-0.313	0.01381	0.0687				1
AKIRIN2	akirin 2	6q15				-0.322333333	0.004236667	0.031366667				1

AKNA	AT-hook transcription factor	9q32				-0.357	0.00029	0.0102				1
AKNAD1	AKNA domain containing 1	1p13.3	-0.363	0.00198	0.0189							1
AKR1A1	aldo-keto reductase family 1, member A1 (aldehyde reductase)	1p33-p32				-0.389	0.01718	0.078				1
AKR1C1	aldo-keto reductase family 1, member C1 (dihydrodiol dehydrogenase 1; 20-al	10p15-p14	-1.105	0.04158	0.1493							1
AKR1C2	aldo-keto reductase family 1, member C2 (dihydrodiol dehydrogenase 2; bile a	10p15-p14							-0.596	2.262	5	1
CCDC68	coiled-coil domain containing 68	18q21	2.288	0	0							1
AKR1C4	aldo-keto reductase family 1, member C4 (chlordecone reductase; 3-alpha hy	10p15.1							-0.929	3.04143	7	1
AKR1C6P	Data not found		-0.357	0.00899	0.0543							1
AKR1D1	aldo-keto reductase family 1, member D1 (delta 4-3-ketosteroid-5-beta-reduc	7q32-q33				-0.339	0.0043	0.0362				1
METTL7B	methyltransferase like 7B	12q13.2	2.286	0	0							1
F5	coagulation factor V (proaccelerin, labile factor)	1q23	2.27	3.00E-05	0.001	0.343333333	0.00084	0.01385				2
AKT1S1	AKT1 substrate 1 (proline-rich)	19q13.33	-0.296	0.01249	0.068	-0.475	0.00021	0.0089				2
AKT2	v-akt murine thymoma viral oncogene homolog 2	19q13.1-q13.2	-0.502	0.00269	0.0236	-0.388	0.00091375	0.01655				2
AKT3	v-akt murine thymoma viral oncogene homolog 3 (protein kinase B, gamma)	1q44				0.385	0.002285	0.0209				1
ALAD	aminolevulinate dehydratase	9q33.1	-0.615	0.00046	0.0068	-0.361	0.00555	0.0414				2
ALAS1	aminolevulinate, delta-, synthase 1	3p21.1				-0.34	0.00148	0.0211				1
ALB	albumin	4q13.3							-0.814	3.09143	7	1
ALCAM	activated leukocyte cell adhesion molecule	3q13.1				0.31	0.00665	0.0439				1
ALDH16A1	aldehyde dehydrogenase 16 family, member A1	19q13.33				-0.416	7.00E-04	0.015				1
CLRN3	clarin 3	10q26.2	2.268	1.00E-05	3.00E-04							1
CYP2B7P1	cytochrome P450, family 2, subfamily B, polypeptide 7 pseudogene 1	19q13.2	2.265	0.00064	0.0086							1
ALDH1A2	aldehyde dehydrogenase 1 family, member A2	15q21.3							-0.778	3.158	5	1
ALDH1A3	aldehyde dehydrogenase 1 family, member A3	15q26.3				0.358	0.00078	0.0158				1
GSDMB	gasdermin B	17q12	2.26	0	0							1
ALDH2	aldehyde dehydrogenase 2 family (mitochondrial)	12q24.2				-0.355666667	0.001321667	0.01785				1
ALDH3A1	aldehyde dehydrogenase 3 family, member A1	17p11.2	-2.876	0	0							1
ALDH3A2	aldehyde dehydrogenase 3 family, member A2	17p11.2	-1.264	3.00E-05	8.00E-04	-0.402	0.004875	0.03275				2
ALDH3B2	aldehyde dehydrogenase 3 family, member B2	11q13	-3.603	0	0							1
ALDH4A1	aldehyde dehydrogenase 4 family, member A1	1p36	-1.197	0.00092	0.0111	-0.318	0.00409	0.0352				2
ALDH6A1	aldehyde dehydrogenase 6 family, member A1	14q24.3				-0.389333333	0.007186667	0.045833333				1
ALDH7A1	aldehyde dehydrogenase 7 family, member A1	5q31	-1.199	0.00014	0.0029	-0.401285714	0.001141429	0.015242857				2
ALDH9A1	aldehyde dehydrogenase 9 family, member A1	1q23.1	-1.505	0	0							1
ALDOAP2	aldolase A, fructose-bisphosphate pseudogene 2	10q26.13	-0.372	0.03431	0.1315							1
BGN	biglycan	Xq28	2.254	0	1.00E-04							1
CENPV	centromere protein V	17p11.2	2.24	0	1.00E-04							1
PLOD3	procollagen-lysine, 2-oxoglutarate 5-dioxygenase 3	7q22	2.24	0	0							1
ALG12	asparagine-linked glycosylation 12, alpha-1,6-mannosyltransferase homolog (S	22q13.33				-0.380666667	0.002286667	0.020333333				1
SLC39A5	In multiple Geneids		2.238	0.00014	0.0029							1
PLAUR	plasminogen activator, urokinase receptor	19q13	2.234	0	1.00E-04							1
NT5E	5'-nucleotidase, ecto (CD73)	6q14-q21	2.233	1.00E-05	4.00E-04							1
COL1A1	collagen, type I, alpha 1	17q21.33	2.231	0	2.00E-04							1
ALG9	asparagine-linked glycosylation 9, alpha-1,2-mannosyltransferase homolog (S.	11q23							-0.662	2.62667	6	1
ALK	anaplastic lymphoma receptor tyrosine kinase	2p23				0.3851	0.002613	0.01881				1
ALKBH1	alkB, alkylation repair homolog 1 (E. coli)	14q24.3				-0.34275	0.0081225	0.04395				1
ALKBH2	alkB, alkylation repair homolog 2 (E. coli)	12q24.11				-0.358	0.00033	0.0108				1
ALKBH3	alkB, alkylation repair homolog 3 (E. coli)	11p11.2	-0.338	0.0335	0.1295							1
ALKBH4	alkB, alkylation repair homolog 4 (E. coli)	7q22.1				-0.5255	0.00044	0.01215				1
ALKBH5	alkB, alkylation repair homolog 5 (E. coli)	17p11.2				-0.432	0.00092	0.017				1
ALKBH6	alkB, alkylation repair homolog 6 (E. coli)	19q13.12				-0.314	0.00591	0.0429				1
ALKBH7	alkB, alkylation repair homolog 7 (E. coli)	19p13.3							0.6225	2.73903	8.5	1
ALLC	allantoicase	2q35							0.68	2.675	6	1
LAMC2	laminin, gamma 2	1q25-q31	2.22	2.00E-05	6.00E-04							1
ALOX12	arachidonate 12-lipoxygenase	17p13.1	-3.889	0	0	-0.527	0.001205	0.01495				2
ALOX12B	arachidonate 12-lipoxygenase, 12R type	17p13.1				-0.365	0.00023	0.0092				1
ALOX15	arachidonate 15-lipoxygenase	17p13.3				-0.362	7.00E-04	0.015	0.94	2.789	5	2
ALOX15B	arachidonate 15-lipoxygenase, type B	17p13.1	-2.368	0	0	-0.451	0.00107	0.0155				2
ALOXE3	In multiple Geneids					-0.410666667	0.005826667	0.036733333				1
MEP1A	meprin A, alpha (PABA peptide hydrolase)	6p12-p11	2.22	0.00047	0.0068	0.307	0.0026	0.0279	-0.78	3.03444	9	3
ALPK1	alpha-kinase 1	4q25				-0.334	0.00052	0.01155				1
ALPK2	alpha-kinase 2	18q21.31							-0.861	3.34	5	1
ALPL	alkaline phosphatase, liver/bone/kidney	1p36.12				0.584	5.00E-04	0.0128				1
TMEM2	transmembrane protein 2	9q21.13	2.209	0	0							1
TM4SF4	transmembrane 4 L six family member 4	3q25	2.208	0.00422	0.0325							1
COL12A1	collagen, type XII, alpha 1	6q12-q13	2.205	6.00E-05	0.0016	0.349	0.00025	0.0096				2
ALS2CL	In multiple Geneids		-0.964	0.00069	0.0091	-0.342	9.00E-05	0.0064				2
ALS2CR7	Data not found								-0.685	2.704	5	1
AMACR	alpha-methylacyl-CoA racemase	5p13				0.385333333	0.00791	0.048				1
AMBRA1	autophagy/beclin-1 regulator 1	11p11.2				-0.315666667	0.004913333	0.032066667				1
AMD1	adenosylmethionine decarboxylase 1	6q21	-0.571	0.00703	0.046	-0.403	5.00E-05	0.0053				2

AMDHD1	amidohydrolase domain containing 1	12q23.1				-0.33	0.02413	0.0953				1
AMDHD2	amidohydrolase domain containing 2	16p13.3				-0.4075	0.01064	0.05875				1
AMELY	amelogenin, Y-linked	Yp11.2							-1.001	3.53375	8	1
AMFR	autocrine motility factor receptor, E3 ubiquitin protein ligase	16q21	-0.782	0.00713	0.0464							1
AMHR2	anti-Mullerian hormone receptor, type II	12q13				-0.417	0.0012	0.01805				1
AMICA1	In multiple Geneids		-0.662	0.02993	0.1204							1
PVRL3	poliovirus receptor-related 3	3q13	2.201	2.00E-05	7.00E-04	0.3295	0.010965	0.0529				2
AMMECR1	Alport syndrome, mental retardation, midface hypoplasia and elliptocytosis ch	Xq22.3				0.342666667	0.00355	0.024633333				1
ARSE	arylsulfatase E (chondrodysplasia punctata 1)	Xp22.3	2.199	5.00E-05	0.0013							1
MMP3	matrix metallopeptidase 3 (stromelysin 1, progelatinase)	11q22.3	2.194	2.00E-05	7.00E-04				-0.748	2.73667	6	2
AMOTL1	angiomotin like 1	11q14.3				0.3375	0.00811	0.0432				1
AMOTL2	In multiple Geneids		-0.675	0.00102	0.0118							1
AMPD1	adenosine monophosphate deaminase 1	1p13				-0.308	0.00737	0.04445				1
IGF2BP2	insulin-like growth factor 2 mRNA binding protein 2	3q27.2	2.188	0	1.00E-04							1
AMPD3	adenosine monophosphate deaminase 3	11p15							0.797	2.8455	5	1
AMPH	amphiphysin	7p14-p13				0.339666667	0.000486667	0.010866667				1
AMT	aminomethyltransferase	3p21.2-p21.1				-0.533666667	0.00057	0.011933333				1
AMY1A	amylase, alpha 1A (salivary)	1p21	-0.463	0.02653	0.1115	-0.4325	0.005588333	0.035833333				2
AMY1B	amylase, alpha 1B (salivary)	1p21				-0.4325	0.005588333	0.035833333				1
AMY1C	amylase, alpha 1C (salivary)	1p21				-0.4325	0.005588333	0.035833333				1
ACSL5	acyl-CoA synthetase long-chain family member 5	10q25.1-q25.2	2.187	0	2.00E-04							1
HMGCS2	3-hydroxy-3-methylglutaryl-CoA synthase 2 (mitochondrial)	1p13-p12	2.172	0.01128	0.0635				-0.523	2.51	5	2
ANAPC10	anaphase promoting complex subunit 10	4q31							-0.625	2.76778	9	1
ANAPC11	anaphase promoting complex subunit 11	17q25.3				-0.429	0.000975	0.0168	0.92825	3.6365725	5.5	2
ANAPC4	anaphase promoting complex subunit 4	4p15.2	-0.283	0.04038	0.1465	-0.349	0.005988	0.03816				2
ANAPC5	anaphase promoting complex subunit 5	12q24.31				-0.367	9.00E-05	0.0064				1
ANAPC7	anaphase promoting complex subunit 7	12q24.11	-0.525	0.00856	0.0525	-0.33	0.00059	0.0138				2
MMP11	matrix metallopeptidase 11 (stromelysin 3)	22q11.23	2.167	5.00E-04	0.0072							1
IGF2	insulin-like growth factor 2 (somatomedin A)	11p15.5	2.165	0.00116	0.0129							1
ANGPT1	angiopoietin 1	8q23.1				0.353142857	0.000239524	0.007185714	-1	2.69	5	2
SEZ6L2	seizure related 6 homolog (mouse)-like 2	16p11.2	2.164	0	2.00E-04							1
ANGPTL1	angiopoietin-like 1	1q25.2							-0.862	3.15	6	1
NR5A2	nuclear receptor subfamily 5, group A, member 2	1q32.1	2.144	1.00E-05	3.00E-04							1
ANGPTL3	angiopoietin-like 3	1p31.1-p22.3							-0.936	3.21857	7	1
ANGPTL4	In multiple Geneids		-0.955	0.00189	0.0183	-0.386	0.0085	0.0524				2
ANGPTL5	In multiple Geneids								-1.081	3.13429	7	1
ANGPTL6	angiopoietin-like 6	19p13.2				-0.533666667	0.000873333	0.014866667				1
ANGPTL7	angiopoietin-like 7	1p36				-0.402	0.00018	0.0083				1
ANK1	ankyrin 1, erythrocytic	8p11.1				-0.318833333	0.01845	0.075083333				1
ANK2	ankyrin 2, neuronal	4q25-q27							-0.816	2.90333	6	1
ANK3	ankyrin 3, node of Ranvier (ankyrin G)	10q21	-1.059	0	0							1
ANKDD1A	ankyrin repeat and death domain containing 1A	15q22.31				-0.415	0.00056	0.01295				1
ANKFN1	ankyrin-repeat and fibronectin type III domain containing 1	17q22				-0.462	0.00118	0.0191				1
ANKFY1	ankyrin repeat and FYVE domain containing 1	17p13.3				-0.3642	0.005393	0.03733				1
TESC	tescalcin	12q24.22	2.139	5.00E-05	0.0013							1
ANKHD1	ankyrin repeat and KH domain containing 1	5q31.3				-0.37175	0.000855	0.014625				1
ANKHD1-EIF4EB	ANKHD1-EIF4EBP3 readthrough	5q31.3	-0.29	0.00326	0.0272	-0.37175	0.000855	0.014625				2
ANKIB1	ankyrin repeat and IBR domain containing 1	7q21.2				0.326	0.00477	0.03795				1
ANKK1	ankyrin repeat and kinase domain containing 1	11q23.2				0.328	0.00029	0.0102				1
ANKLE2	ankyrin repeat and LEM domain containing 2	12q24.33				-0.372666667	0.001873333	0.0197				1
VNN1	vanin 1	6q23-q24	2.13	0.00049	0.007							1
PEG10	In multiple Geneids		2.122	0.00575	0.0402							1
ANKRD11	ankyrin repeat domain 11	16q24.3	-0.241	0.00944	0.0562	-0.380742857	0.004216	0.031317143				2
ANKRD13A	ankyrin repeat domain 13A	12q24.11	-0.582	3.00E-04	0.0049	-0.312	0.00273	0.0287				2
SERPINE1	serpin peptidase inhibitor, clade E (nexin, plasminogen activator inhibitor type	7q21.3-q22	2.122	0	2.00E-04							1
ANKRD13C	ankyrin repeat domain 13C	1p32.3-p31.3				-0.363	0.001225	0.01925				1
ANKRD13D	ankyrin repeat domain 13 family, member D	11q13.2	-0.262	0.01954	0.0913							1
ANKRD16	ankyrin repeat domain 16	10p15.1				-0.438	0.01391	0.069				1
ANKRD2	ankyrin repeat domain 2 (stretch responsive muscle)	10q23				-0.316	0.00023	0.0093				1
ANKRD20A1	ankyrin repeat domain 20 family, member A1	9q13	-1.541	4.00E-05	0.0011							1
ANKRD20A11P	In multiple Geneids		-3.256	0	0							1
ANKRD20A2	ankyrin repeat domain 20 family, member A2	9p12	-2.028	1.00E-05	4.00E-04							1
ANKRD20A3	ankyrin repeat domain 20 family, member A3	9p12	-2.133	0	1.00E-04							1
ANKRD20A5P	ankyrin repeat domain 20 family, member A5, pseudogene	18p11.21	-2.101	0	2.00E-04							1
ANKRD20A8P	ankyrin repeat domain 20 family, member A8, pseudogene	2q11.1	-1.711166667	0.006566667	0.030666667							1
ANKRD22	ankyrin repeat domain 22	10q23.31							-0.657	2.83571	7	1
ANKRD23	ankyrin repeat domain 23	2q11.2							0.714	3.16	5	1
ANKRD24	ankyrin repeat domain 24	19p13.3				-0.505375	0.00352125	0.027525				1
ANKRD27	ankyrin repeat domain 27 (VPS9 domain)	19q13.11				-0.338	0.003035	0.02735				1

ANKRD28	ankyrin repeat domain 28	3p25.1				-0.488	0.00395	0.0346				1
ANKRD30BL	ankyrin repeat domain 30B-like	2q21.2	-0.246	0.0358	0.1354							1
ANKRD31	ankyrin repeat domain 31	5q13.3	-1.299	0.00016	0.0031							1
ANKRD32	ankyrin repeat domain 32	5q15							-0.676	2.54	5	1
ANKRD34B	ankyrin repeat domain 34B	5q14.1				-0.3255	0.00061	0.01395				1
ANKRD34C	ankyrin repeat domain 34C	15q25.1				0.338	0.00145	0.021				1
ANKRD35	ankyrin repeat domain 35	1q21.1	-1.329	0	0							1
PXDN	peroxidasin homolog (Drosophila)	2p25	2.121	0	0	0.708	1.00E-05	0.0032				2
HKDC1	In multiple Geneids		2.119	0	0							1
ANKRD37	ankyrin repeat domain 37	4q35.1	-1.135	4.00E-05	0.0011							1
TIMP1	TIMP metallopeptidase inhibitor 1	Xp11.3-p11.23	2.116	0	2.00E-04							1
ANKRD40	ankyrin repeat domain 40	17q21.33							-0.76	2.544	5	1
ANKRD42	ankyrin repeat domain 42	11q14.1				-0.384	4.00E-04	0.0118				1
ANKRD46	ankyrin repeat domain 46	8q22.2	-0.422	0.02511	0.1074							1
SLC40A1	solute carrier family 40 (iron-regulated transporter), member 1	2q32	2.113	1.00E-05	3.00E-04							1
ANKRD5	ankyrin repeat domain 5	20p12.2				0.3265	0.01527	0.07125				1
ANKRD50	ankyrin repeat domain 50	4q28.1							-0.922	3.38875	8	1
ANKS4B	ankyrin repeat and sterile alpha motif domain containing 4B	16p12.2	2.11	0	0				-0.998	2.73714	7	2
ANKRD54	ankyrin repeat domain 54	22q13.1	-0.242	0.02624	0.1107	-0.376	0.00088	0.013666667				2
ANKRD55	ankyrin repeat domain 55	5q11.2	-0.351	0.00378	0.0301	-0.3753	0.000765	0.0129				2
ANKRD6	ankyrin repeat domain 6	6q14.2-q16.1				-0.389777778	0.001357778	0.016888889				1
ANKRD9	ankyrin repeat domain 9	14q32.31							0.721	2.82875	6	1
DAPK1	death-associated protein kinase 1	9q21.33	2.097	0	1.00E-04							1
ANKS1B	ankyrin repeat and sterile alpha motif domain containing 1B	12q23.1				0.391	0.00825	0.0515	-1.082	3.84333	6	2
ANKS3	ankyrin repeat and sterile alpha motif domain containing 3	16p13.3				-0.398333333	0.018416667	0.076233333				1
CHKA	choline kinase alpha	11q13.2	2.094	0	0							1
MYO1A	myosin IA	12q13-q14	2.094	7.00E-05	0.0018				-1.004	3.46667	6	2
EPS8	epidermal growth factor receptor pathway substrate 8	12p12.3	2.091	0	0	0.313333333	0.001533333	0.020233333				2
ANO10	anoctamin 10	3p22.1	-0.474	0.02348	0.1028	-0.330833333	0.00219	0.02235				2
ANO2	anoctamin 2	12p13.3				0.450222222	0.002362222	0.0211				1
ANO4	anoctamin 4	12q23.1				0.314	0.00428	0.0361				1
THY1	Thy-1 cell surface antigen	11q23.3	2.091	0	1.00E-04							1
ANO8	anoctamin 8	19p13.11	-0.688	2.00E-05	7.00E-04	-0.549333333	0.000263333	0.009766667				2
PTCH2	patched 2	1p34.1	2.084	0.00163	0.0165							1
TMPRSS3	transmembrane protease, serine 3	21q22.3	2.077	0	1.00E-04							1
SLC12A7	solute carrier family 12 (potassium/chloride transporters), member 7	5p15	2.076	0	1.00E-04							1
SLC12A2	solute carrier family 12 (sodium/potassium/chloride transporters), member 2	5q23.3	2.072	0.00018	0.0034							1
F2R	coagulation factor II (thrombin) receptor	5q13	2.071	0	0							1
BAMBI	BMP and activin membrane-bound inhibitor homolog (Xenopus laevis)	10p12.3-p11.2	2.068	5.00E-05	0.0014							1
SERPINA3	serpin peptidase inhibitor, clade A (alpha-1 antiproteinase, antitrypsin), meml	14q32.1	2.068	0.00071	0.0092							1
ANXA1	annexin A1	9q21.13				0.332	0.00093	0.017	-0.875	2.99571	7	2
ANXA10	annexin A10	4q33							-0.951	2.99556	9	1
ANXA11	annexin A11	10q23	-0.752	0.00145	0.0152	-0.38425	0.000375	0.0085				2
SERPINH1	serpin peptidase inhibitor, clade H (heat shock protein 47), member 1, (collag	11q13.5	2.068	0	0							1
ANXA2P3	annexin A2 pseudogene 3	10q21.3	-0.386	0.0471	0.1611							1
MSLN	mesothelin	16p13.3	2.057	0	2.00E-04							1
GPC3	glypican 3	Xq26.1	2.053	0.01747	0.0847	0.3509375	0.00839875	0.04594375				2
CTSL	Data not found		2.052	0	1.00E-04							1
ANXA7	annexin A7	10q22.2				-0.3975	0.001055	0.0149				1
ANXA8	annexin A8	10q11.22	-0.982	8.00E-05	0.0018	-0.37	0.01372	0.0685	0.742	2.77	5	3
ANXA8L1	In multiple Geneids		-3.413	1.00E-05	3.00E-04	-0.37	0.01372	0.0685	0.853	2.878	5	3
ANXA8L2	In multiple Geneids		-2.236	1.00E-05	3.00E-04							1
AOAH	acyloxyacyl hydrolase (neutrophil)	7p14-p12				0.345	0.00145	0.0207				1
TM4SF5	transmembrane 4 L six family member 5	17p13.3	2.051	0	1.00E-04							1
AOC2	amine oxidase, copper containing 2 (retina-specific)	17q21				-0.428	0.00262	0.028				1
AOC3	amine oxidase, copper containing 3 (vascular adhesion protein 1)	17q21				-0.317	0.00383	0.0341				1
AOF1	Data not found					-0.312	0.00528	0.0404				1
AP1B1	adaptor-related protein complex 1, beta 1 subunit	22q12.2	-0.831	0	1.00E-04	-0.469526316	0.000435263	0.009173684				2
AP1G1	adaptor-related protein complex 1, gamma 1 subunit	16q23	-0.608	0.00029	0.0048	-0.40225	0.0020425	0.0221				2
AP1M1	adaptor-related protein complex 1, mu 1 subunit	19p13.12							-1.029	2.642	5	1
AP1M2	adaptor-related protein complex 1, mu 2 subunit	19p13.2				-0.6	0.014855	0.0556				1
CFB	complement factor B	6p21.3	2.047333333	0.007386667	0.033666667							1
GPRC5A	G protein-coupled receptor, family C, group 5, member A	12p13-p12.3	2.044	0.00046	0.0067							1
AP2A1	adaptor-related protein complex 2, alpha 1 subunit	19q13.33				-0.3445	0.006345	0.0427				1
AP2A2	adaptor-related protein complex 2, alpha 2 subunit	11p15.5				-0.409538462	0.008166923	0.0429				1
MTMR11	myotubularin related protein 11	1q12-q21	2.042	0	1.00E-04							1
AP2M1	adaptor-related protein complex 2, mu 1 subunit	3q28				-0.338	0.00245	0.0272				1
AP2S1	adaptor-related protein complex 2, sigma 1 subunit	19q13.2-q13.3	-0.273	0.03205	0.1259							1
AP3B1	adaptor-related protein complex 3, beta 1 subunit	5q14.1	-0.449	0.00014	0.0029	-0.302	0.00287	0.0293				2

AP3B2	adaptor-related protein complex 3, beta 2 subunit	15q				-0.338	0.01222	0.0641				1
AP3D1	adaptor-related protein complex 3, delta 1 subunit	19p13.3				-0.4666	0.00091	0.0145				1
COL1A2	collagen, type I, alpha 2	7q22.1	2.041	2.00E-05	7.00E-04	0.349333333	0.006936667	0.040133333				2
COL6A3	collagen, type VI, alpha 3	2q37	2.041	3.00E-05	9.00E-04	0.327	0.01065	0.0593				2
AP4B1	adaptor-related protein complex 4, beta 1 subunit	1p13.2				-0.302	0.02631	0.1003				1
AP4E1	adaptor-related protein complex 4, epsilon 1 subunit	15q21.2	-0.363	0.04173	0.1496							1
AP5B1	adaptor-related protein complex 5, beta 1 subunit	11q13.1	-0.324	0.01077	0.0615							1
AP5M1	adaptor-related protein complex 5, mu 1 subunit	14q22.3	-0.536	0.00261	0.0231							1
CCL18	chemokine (C-C motif) ligand 18 (pulmonary and activation-regulated)	17q11.2	2.037	0.00051	0.0073							1
A1CF	APOBEC1 complementation factor	10q11.23	2.033	0.00034	0.0054							1
APBA1	amyloid beta (A4) precursor protein-binding, family A, member 1	9q13-q21.1	-0.418	3.00E-05	9.00E-04							1
APBA2	amyloid beta (A4) precursor protein-binding, family A, member 2	15q11-q12				-0.3165	0.00674	0.04525				1
APBA3	amyloid beta (A4) precursor protein-binding, family A, member 3	19p13.3				-0.593	0.000145	0.0076				1
APBB1	amyloid beta (A4) precursor protein-binding, family B, member 1 (Fe65)	11p15				-0.316	0.00407	0.0351				1
AGMAT	agmatine ureohydrolase (agmatinase)	1p36.21	2.029	2.00E-05	8.00E-04							1
CDH11	cadherin 11, type 2, OB-cadherin (osteoblast)	16q21	2.026	1.00E-04	0.0023							1
APBB3	amyloid beta (A4) precursor protein-binding, family B, member 3	5q31				-0.344	0.00673	0.0461				1
APC	adenomatous polyposis coli	5q21-q22	-0.66	0.00013	0.0027	-0.3354	4.80E-05	0.0045				2
APCDD1L	adenomatosis polyposis coli down-regulated 1-like	20q13.32				0.347	0.005403333	0.040466667				1
APCS	amyloid P component, serum	1q21-q23							-0.831	3.028	5	1
APEH	N-acylaminoacyl-peptide hydrolase	3p21.31				-0.44025	0.0008775	0.013125				1
APEX2	APEX nuclease (apurinic/apyrimidinic endonuclease) 2	Xp11.21							-0.664	2.34167	6	1
MYO7B	myosin VIIB	2q21.1	2.024	0.00019	0.0036							1
APIN	Data not found								-0.841	3.37625	8	1
APITD1	apoptosis-inducing, TAF9-like domain 1	1p36.22				-0.401333333	0.000556667	0.0135				1
APLF	aprataxin and PNKP like factor	2p13.3				0.321	0.00469	0.031166667				1
C3	complement component 3	19p13.3-p13.2	2.022	0.0039	0.0307							1
AOC1	Data not found		2.02	0	0							1
APLP1	amyloid beta (A4) precursor-like protein 1	19q13.1				-0.384333333	0.00262	0.0275				1
PLA2G16	phospholipase A2, group XVI	11q12.3	2.013	0	0							1
SCCPDH	saccharopine dehydrogenase (putative)	1q44	2.011	1.00E-05	4.00E-04							1
SYTL2	In multiple Geneids		2.002	8.00E-05	0.0019				-0.938	3.490835	7	2
APOB	apolipoprotein B (including Ag(x) antigen)	2p24-p23				0.3065	0.003945	0.03155				1
APOB48R	Data not found								0.529	2.54781	8	1
LOXL2	lysyl oxidase-like 2	8p21.3	2.001	0	0							1
APOBEC2	apolipoprotein B mRNA editing enzyme, catalytic polypeptide-like 2	6p21				0.40175	0.0010625	0.01685				1
APOBEC3A	In multiple Geneids					-0.53275	0.001085	0.016125				1
APOBEC3B	In multiple Geneids					-0.63	0.00207	0.025				1
APOBEC3C	In multiple Geneids		-0.984	0.00042	0.0063							1
APOBEC3D	apolipoprotein B mRNA editing enzyme, catalytic polypeptide-like 3D	22q13.1				-0.653	1.00E-05	0.0029				1
APOBEC3F	In multiple Geneids					-0.5825	2.00E-05	0.0033				1
APOBEC3G	In multiple Geneids					-0.706	8.00E-05	0.0061				1
APOBEC3H	apolipoprotein B mRNA editing enzyme, catalytic polypeptide-like 3H	22q13.1				-0.499	0.00234	0.0265				1
APOBEC4	apolipoprotein B mRNA editing enzyme, catalytic polypeptide-like 4 (putative)	1q25.3				0.306	0.00124	0.0195				1
ARHGAP42	Rho GTPase activating protein 42	11q22.1	1.998	0	0							1
APOC2	apolipoprotein C-II	19q13.2							0.699	2.452	5	1
THBS1	thrombospondin 1	15q15	1.993	0.00023	0.004							1
APOH	apolipoprotein H (beta-2-glycoprotein I)	17q23-qter							-0.875	3.158	5	1
IFI6	interferon, alpha-inducible protein 6	1p35	1.991	0.00028	0.0047							1
APOL3	apolipoprotein L, 3	22q13.1				-0.4112	0.000584	0.01				1
APOL4	apolipoprotein L, 4	22q11.2-q13.2				-0.351	0.00138	0.017933333				1
APOL5	apolipoprotein L, 5	22q12.3				-0.35975	0.000605	0.01325				1
APOL6	apolipoprotein L, 6	22q12.3				-0.4175	3.00E-05	0.00385				1
HID1	Data not found		1.969	0.00013	0.0028							1
APOO	apolipoprotein O	Xp22.11				-0.345	0.0031	0.0305				1
APOOL	apolipoprotein O-like	Xq21.1				0.312	0.00014	0.0074				1
MMP2	matrix metallopeptidase 2 (gelatinase A, 72kDa gelatinase, 72kDa type IV colla	16q13-q21	1.967	0.00034	0.0054							1
APPL1	In multiple Geneids		-0.418	0.00369	0.0296	-0.3745	0.000465	0.0116				2
IQGAP2	IQ motif containing GTPase activating protein 2	5q13.3	1.966	0.00474	0.0352							1
APRT	adenine phosphoribosyltransferase	16q24	-0.303	0.00696	0.0457							1
APTX	In multiple Geneids					-0.3815	0.002685	0.0265				1
AQP10	aquaporin 10	1q21.3				-0.312	0.00081	0.016				1
AQP3	aquaporin 3 (Gill blood group)	9p13	-3.381	0	2.00E-04	-0.427	8.00E-05	0.006				2
AQP4	aquaporin 4	18q11.2-q12.1							-0.872333333	2.647276667	5.333333333	1
AQP6	aquaporin 6, kidney specific	12q13	-0.337	0.00376	0.03	-0.301	3.00E-05	0.0042	0.912	4.135	6	3
AQP7	aquaporin 7	9p13				-0.488	0.00028	0.0101				1
AQP7P1	aquaporin 7 pseudogene 1	9q13	-0.711	0.01882	0.0892							1
CDHR2	cadherin-related family member 2	5q35.2	1.964	0.00027	0.0045							1
AQR	aquarius homolog (mouse)	15q14				-0.336	0.006355	0.0446				1

AR	androgen receptor	Xq12				0.3508125	0.001480625	0.01833125	-0.791	3.1075	8	2
ARAP1	ArfGAP with RhoGAP domain, ankyrin repeat and PH domain 1	11q13.4				-0.32	0.00042	0.012				1
ARAP2	ArfGAP with RhoGAP domain, ankyrin repeat and PH domain 2	4p14	-0.983	0.00424	0.0326							1
INHBA	inhibin, beta A	7p15-p13	1.955	0	2.00E-04				-1.009	2.996	5	2
SFRP4	secreted frizzled-related protein 4	7p14.1	1.953	0.0135	0.0713							1
KIAA1244	KIAA1244	6q23.3	1.948	3.00E-05	0.001							1
ARF3	ADP-ribosylation factor 3	12q13				-0.348666667	0.000696667	0.014133333				1
CALML4	calmodulin-like 4	15q23	1.945	0	0							1
ARF5	ADP-ribosylation factor 5	7q31.3	-0.584	7.00E-05	0.0017							1
ARF6	ADP-ribosylation factor 6	14q21.3	-1.153	9.00E-05	0.0021							1
TFF1	trefoil factor 1	21q22.3	1.945	0.03955	0.1445							1
SLC2A3	solute carrier family 2 (facilitated glucose transporter), member 3	12p13.3	1.938	1.00E-05	3.00E-04							1
ARFGEF1	ADP-ribosylation factor guanine nucleotide-exchange factor 1 (brefeldin A-inh	8q13				0.31	0.02638	0.1005				1
ARFIP1	ADP-ribosylation factor interacting protein 1	4q31.3				-0.349	0.00331	0.0316				1
ARFIP2	ADP-ribosylation factor interacting protein 2	11p15							-0.669	2.34833	6	1
ARG1	arginase, liver	6q23							-0.829	2.486	5	1
ARG2	arginase, type II	14q24.1	-0.978	0.00053	0.0075							1
ARGLU1	arginine and glutamate rich 1	13q33.3				0.364	0.01156	0.0621				1
ARHGAP10	Rho GTPase activating protein 10	4q31.23	-1.1375	4.50E-05	0.0011	-0.3472	0.01354	0.06276				2
SPRY4	sprouty homolog 4 (Drosophila)	5q31.3	1.938	0	0							1
GPA33	glycoprotein A33 (transmembrane)	1q24.1	1.937	0.00185	0.018	0.302	0.0013	0.0199				2
ARHGAP15	Rho GTPase activating protein 15	2q22.2-q22.3				0.349027778	0.002234722	0.018047222	-0.672	2.71429	7	2
UNC5CL	unc-5 homolog C (C. elegans)-like	6p21.1	1.936	4.00E-05	0.0011	0.326	0.01511	0.0724				2
ARHGAP19	Rho GTPase activating protein 19	10q24.1				-0.3488	0.000142	0.00732				1
ARHGAP20	Rho GTPase activating protein 20	11q23.1				0.338	0.0017	0.0227				1
ARHGEF38	Rho guanine nucleotide exchange factor (GEF) 38	4q24	1.925	0	2.00E-04							1
ARHGAP22	Rho GTPase activating protein 22	10q11.22				-0.376	0.00405	0.0351				1
ARHGAP24	Rho GTPase activating protein 24	4q22.1							-0.9945	2.801	5	1
C6orf222	chromosome 6 open reading frame 222	6p21.31	1.925	2.00E-05	7.00E-04							1
ARHGAP27	Rho GTPase activating protein 27	17q21.31	-0.9115	4.50E-05	0.00115	-0.357	0.00356	0.0328				2
ARHGAP28	Rho GTPase activating protein 28	18p11.31							-0.706	2.58714	7	1
GREM1	gremlin 1	15q13.3	1.907	0.01575	0.0791							1
ARHGAP30	Rho GTPase activating protein 30	1q23.3	-0.589	0.00048	0.007							1
ARHGAP31	Rho GTPase activating protein 31	3q13.33	-0.42	0.04196	0.15							1
ARHGAP32	Rho GTPase activating protein 32	11q24.3	-0.836	0.0024	0.0217							1
ARHGAP40	Rho GTPase activating protein 40	20q11.23	-1.906	0	1.00E-04							1
DAB2	disabled homolog 2, mitogen-responsive phosphoprotein (Drosophila)	5p13	1.903	0.00014	0.0028	0.382866667	0.012872667	0.05926				2
GPR160	G protein-coupled receptor 160	3q26.2-q27	1.903	9.00E-04	0.0109							1
ARHGAP5	Rho GTPase activating protein 5	14q12	-0.484	0.00883	0.0536							1
ARHGAP5-AS1	ARHGAP5 antisense RNA 1 (non-protein coding)	14q12	-0.849	0.00646	0.0434							1
ARHGAP6	Rho GTPase activating protein 6	Xp22.3				0.353823529	0.005921765	0.037335294				1
ARHGAP8	Rho GTPase activating protein 8	22q13.31				-0.44373913	0.002822174	0.018713043				1
ARHGAP9	In multiple Geneids					-0.418	0.00081	0.016				1
ARHGDIB	Rho GDP dissociation inhibitor (GDI) beta	12p12.3							-0.744	2.814	5	1
ARHGDIG	Rho GDP dissociation inhibitor (GDI) gamma	16p13.3							0.625	2.426	5	1
ARHGEF1	Rho guanine nucleotide exchange factor (GEF) 1	19q13.13				-0.4435	0.000395	0.0113	1.236	2.998	5	2
ARHGEF10L	Rho guanine nucleotide exchange factor (GEF) 10-like	1p36.13				-0.38405	0.0025485	0.02115	0.54	2.435	6	2
ARHGEF12	Rho guanine nucleotide exchange factor (GEF) 12	11q23.3				0.32	0.00058	0.0138				1
ARHGEF15	Rho guanine nucleotide exchange factor (GEF) 15	17p13.1				-0.381	0.00147	0.02025				1
HOXB6	homeobox B6	17q21.3	1.903	0	1.00E-04							1
ARHGEF18	Rho/Rac guanine nucleotide exchange factor (GEF) 18	19p13.3				-0.520444444	0.002244444	0.023188889				1
ARHGEF19	Rho guanine nucleotide exchange factor (GEF) 19	1p36.13	-0.54	6.00E-05	0.0016							1
ARHGEF26	Rho guanine nucleotide exchange factor (GEF) 26	3q25.2	-1.72	1.00E-05	3.00E-04							1
ARHGEF3	Rho guanine nucleotide exchange factor (GEF) 3	3p21-p13	-0.845	1.00E-05	3.00E-04	-0.375521739	0.001636087	0.018373913				2
ARHGEF37	Rho guanine nucleotide exchange factor (GEF) 37	5q32	-1.112	0	0							1
MICAL2	microtubule associated monoxygenase, calponin and LIM domain containing 2	11p15.3	1.893	0	1.00E-04	0.404	0	0.0021				2
SULF1	sulfatase 1	8q13.1	1.883	0.00319	0.0268	0.325	0.00888	0.0537				2
ARHGEF4	Rho guanine nucleotide exchange factor (GEF) 4	2q22	-1.442	0	2.00E-04							1
ANO1	anoctamin 1, calcium activated chloride channel	11q13.3	1.881	6.00E-05	0.0015							1
ARHGEF5	In multiple Geneids					-0.610222222	0.005138889	0.033511111				1
ARHGEF5L	Data not found					-0.647	0.00058	0.0138				1
ARHGEF6	Rac/Cdc42 guanine nucleotide exchange factor (GEF) 6	Xq26.3							-0.845	2.9775	8	1
GALNT4	UDP-N-acetyl-alpha-D-galactosamine:polypeptide N-acetylgalactosaminyltran	12q21.33	1.88	0.00011	0.0024				-0.558	2.408	5	2
ARHGEF9	Cdc42 guanine nucleotide exchange factor (GEF) 9	Xq11.1				0.3485625	0.00355125	0.0273				1
ARID1A	AT rich interactive domain 1A (SWI-like)	1p35.3	-0.279	0.04803	0.1631	-0.365785714	0.01431	0.064207143				2
ARID1B	AT rich interactive domain 1B (SWI1-like)	6q25.1							-0.721	2.694	5	1
ARID2	AT rich interactive domain 2 (ARID, RFX-like)	12q12				0.356	1.00E-05	0.0024				1
ARID3A	AT rich interactive domain 3A (BRIGHT-like)	19p13.3				-0.396	0.020382	0.07752				1
SYT1	synaptotagmin I	12cen-q21	1.876	5.00E-05	0.0013	0.361	0.00815	0.0511				2

MET	met proto-oncogene (hepatocyte growth factor receptor)	7q31	1.875	0	0							1
P2RX4	purinergic receptor P2X, ligand-gated ion channel, 4	12q24.32	1.872	2.00E-05	7.00E-04							1
ARID5B	AT rich interactive domain 5B (MRF1-like)	10q21.2				0.369	0.01286	0.066	-0.766	2.9275	8	2
ARIH2	ariadne homolog 2 (Drosophila)	3p21				-0.3720625	0.001805	0.0198875				1
ARL10	ADP-ribosylation factor-like 10	5q35.2	-0.333	0.00113	0.0127	-0.5155	0.000115	0.00685				2
ARL13A	ADP-ribosylation factor-like 13A	Xq22.1							-1.041	3.75	7	1
GALM	galactose mutarotase (aldose 1-epimerase)	2p22.1	1.87	1.00E-05	3.00E-04							1
MUC5B	In multiple Geneids		1.87	0.00784	0.0495							1
ARL15	ADP-ribosylation factor-like 15	5p15.2				-0.342818182	0.004812727	0.030418182				1
ARL16	ADP-ribosylation factor-like 16	17q25.3				-0.316	0.00858	0.0527				1
ARL17	Data not found					-0.383	0.00367	0.0333				1
ARL2BP	ADP-ribosylation factor-like 2 binding protein	16q13	-0.609	0.01666	0.082							1
ARL3	ADP-ribosylation factor-like 3	10q23.3				-0.3758	0.003872	0.03				1
ARL4A	ADP-ribosylation factor-like 4A	7p21.3	-0.257	0.03072	0.1226							1
ARL4D	ADP-ribosylation factor-like 4D	17q12-q21	-2.216	0	1.00E-04	-0.369	0.00415	0.0355				2
ARL5A	ADP-ribosylation factor-like 5A	2q23.3				0.306	0.00034	0.0109				1
ARL5C	ADP-ribosylation factor-like 5C	17q12				-0.337	0.00883	0.0535				1
NID2	nidogen 2 (osteonidogen)	14q22.1	1.868	0	1.00E-04							1
ARL8B	ADP-ribosylation factor-like 8B	3p26.1	-0.798	0.00013	0.0026	-0.3675	0.00211	0.0232				2
ARL9	In multiple Geneids					-0.54	1.00E-05	0.0032				1
HSD17B2	hydroxysteroid (17-beta) dehydrogenase 2	16q24.1-q24.2	1.865	0.00016	0.0031				-1.225	2.96	5	2
ARMC12	armadillo repeat containing 12	6p21.31	-0.317	0.00578	0.0404							1
ARMC2	armadillo repeat containing 2	6q21				-0.309	0.00013	0.0073				1
ARMC5	armadillo repeat containing 5	16p11.2	-0.198	0.02063	0.0947							1
ARMC6	armadillo repeat containing 6	19p13.11				-0.377333333	0.003793333	0.0267				1
MME	membrane metallo-endopeptidase	3q25.2	1.856	0.00388	0.0306	0.314	0.00393	0.0345				2
PTPRJ	protein tyrosine phosphatase, receptor type, J	11p11.2	1.856	2.00E-05	6.00E-04							1
C20orf112	chromosome 20 open reading frame 112	20q11.21	1.854	0	0							1
ARMCX6	armadillo repeat containing, X-linked 6	Xq21.33-q22.3							-0.967	2.58417	6	1
ARNT2	aryl-hydrocarbon receptor nuclear translocator 2	15q24				0.335	0.00384	0.0341				1
ARNTL	aryl hydrocarbon receptor nuclear translocator-like	11p15				0.306	0.00115	0.0188				1
ARNTL2	aryl hydrocarbon receptor nuclear translocator-like 2	12p12.2-p11.2				0.331	0.006925	0.0421				1
PGM2L1	phosphoglucomutase 2-like 1	11q13.4	1.853	0	0							1
ARPC2	actin related protein 2/3 complex, subunit 2, 34kDa	2q36.1	-0.566	0.00129	0.014							1
ARPC3	actin related protein 2/3 complex, subunit 3, 21kDa	12q24.11				-0.448	6.00E-05	0.0054				1
ARPC4	actin related protein 2/3 complex, subunit 4, 20kDa	3p25.3				-0.445	0.00112	0.0185				1
ARPC5L	actin related protein 2/3 complex, subunit 5-like	9q33.3				-0.384	0.00188	0.0238				1
ARPP-21	Data not found								-0.8572	2.802288	6.6	1
ARR3	arrestin 3, retinal (X-arrestin)	Xcen-q21				0.308	0.00101	0.0177	-0.969	2.952	5	2
IRF8	interferon regulatory factor 8	16q24.1	1.851	4.00E-05	0.0011							1
TNS3	tensin 3	7p12.3	1.849	0	1.00E-04	0.363555556	0.019058889	0.077533333				2
ARRDC2	arrestin domain containing 2	19p13.11				-0.56	0.00111	0.0185				1
ARRDC5	In multiple Geneids					-0.496	0.00029	0.0102				1
ARSA	arylsulfatase A	22q13.33	-0.393	0.00623	0.0424							1
PRKCA	protein kinase C, alpha	17q22-q23.2	1.848	0	1.00E-04							1
ARSD	arylsulfatase D	Xp22.3							0.64	2.93286	7	1
ODC1	In multiple Geneids		1.847	9.00E-05	0.0021							1
ARSF	arylsulfatase F	Xp22.3	-1.464	0	0							1
ARSG	arylsulfatase G	17q24.2	-0.828	3.00E-05	0.001	-0.389	0.00463	0.0376				2
ARSH	arylsulfatase family, member H	Xp22.33	-0.271	0.01108	0.0626							1
ART1	ADP-ribosyltransferase 1	11p15				-0.374	0.00353	0.0327				1
ART3	ADP-ribosyltransferase 3	4q21.1				-0.4025	0.008695	0.0473				1
ART5	ADP-ribosyltransferase 5	11p15.4				-0.314	0.00656	0.0454				1
ARVCF	armadillo repeat gene deleted in velocardiofacial syndrome	22q11.21				-0.3935	0.000335	0.00925				1
AS3MT	arsenic (+3 oxidation state) methyltransferase	10q24.32				-0.444	0.002803333	0.021766667				1
ASAM	Data not found					-0.306	0.001395	0.02				1
C4BPA	complement component 4 binding protein, alpha	1q32	1.842	0.00153	0.0158	0.388	0.0122	0.064				2
LIPG	lipase, endothelial	18q21.1	1.83	1.00E-05	5.00E-04							1
ASAP3	ArfGAP with SH3 domain, ankyrin repeat and PH domain 3	1p36.12	-0.685	3.00E-05	9.00E-04	-0.360666667	0.012226667	0.0547				2
TTYH3	tweety homolog 3 (Drosophila)	7p22	1.83	0	0							1
ASB11	ankyrin repeat and SOCS box containing 11	Xp22.31							-0.824	3.37667	6	1
ASB14	ankyrin repeat and SOCS box containing 14	3p21.1							-0.828	2.62	6	1
ASB15	ankyrin repeat and SOCS box containing 15	7q31.31							-0.684	3.08143	7	1
ASB16	ankyrin repeat and SOCS box containing 16	17q21.31	-0.219	0.04947	0.1661							1
ASB17	ankyrin repeat and SOCS box containing 17	1p31.1							-0.917	3.336	5	1
ASB3	ankyrin repeat and SOCS box containing 3	2p16-p14				0.326	0.00164	0.0223	-0.866	2.75333	6	2
ASB4	ankyrin repeat and SOCS box containing 4	7q21-q22				0.318333333	0.003076667	0.027166667				1
ASB5	ankyrin repeat and SOCS box containing 5	4q34.2							-0.686	2.942	5	1
ASB6	ankyrin repeat and SOCS box containing 6					-0.471	0.00079	0.0158				1

ASB7	ankyrin repeat and SOCS box containing 7					0.3635	0.005285	0.03285				1
ASB8	ankyrin repeat and SOCS box containing 8	12q13.11							-0.933	2.752	5	1
ASCC1	activating signal cointegrator 1 complex subunit 1	10pter-q25.3				-0.351666667	0.002516667	0.026633333				1
ASCC2	activating signal cointegrator 1 complex subunit 2	22q12.1	-0.934	1.00E-05	5.00E-04	-0.431111111	0.005451111	0.0352				2
ASCL1	achaete-scute complex homolog 1 (Drosophila)	12q23.2							0.898	2.946	5	1
ASCL3	achaete-scute complex homolog 3 (Drosophila)	11p15.3							-0.904	2.84	5	1
ASF1B	ASF1 anti-silencing function 1 homolog B (S. cerevisiae)	19p13.12				-0.40575	0.01106	0.05175				1
ASFMR1	Data not found					0.309	0.00038	0.0115				1
ASGR1	asialoglycoprotein receptor 1	17p13.2				-0.376	0.00096	0.0173				1
ASGR2	asialoglycoprotein receptor 2	17p				-0.368	0.00306	0.02945				1
ASH2L	ash2 (absent, small, or homeotic)-like (Drosophila)	8p11.2	-0.45	0.00042	0.0064	-0.305	0.00464	0.0377				2
SLC6A19	solute carrier family 6 (neutral amino acid transporter), member 19	5p15.33	1.829	0.00136	0.0145							1
ASMT	acetylserotonin O-methyltransferase	Xp22.3 or Yp11.3							-1.174	2.84571	7	1
ASMTL	acetylserotonin O-methyltransferase-like	Xp22.3; Yp11.3	-0.234	0.03278	0.1277	-0.605777778	0.000968889	0.012388889				2
ASNA1	arsA arsenite transporter, ATP-binding, homolog 1 (bacterial)	19q13.3				-0.363666667	0.00776	0.047666667				1
ASNSD1	asparagine synthetase domain containing 1	2p24.3-q21.3							-0.814	2.695	6	1
ASPA	aspartoacylase	17p13.3				-0.398833333	0.004361667	0.031566667				1
ASPH	aspartate beta-hydroxylase	8q12.1				0.337909091	0.000599091	0.011309091	-0.874	3.17333	6	2
PRR15L	proline rich 15-like	17q21.32	1.826	6.00E-05	0.0016							1
ASPHD2	aspartate beta-hydroxylase domain containing 2	22q12.1				-0.3858	0.00034	0.0099				1
ASPM	asp (abnormal spindle) homolog, microcephaly associated (Drosophila)	1q31				0.3305	0.00069	0.01285				1
TMEM176B	transmembrane protein 176B	7q36.1	1.814	0	2.00E-04							1
ASPSCR1	alveolar soft part sarcoma chromosome region, candidate 1	17q25.3				-0.306	0.00082	0.0161				1
ITM2C	In multiple Geneids		1.812	7.00E-05	0.0018							1
SPARC	secreted protein, acidic, cysteine-rich (osteonectin)	5q31.3-q32	1.804	0	0							1
EPHB2	EPH receptor B2	1p36.1-p35	1.8	0	1.00E-04							1
ASTN1	astrotactin 1	1q25.2				0.346166667	0.002473333	0.024383333				1
ASTN2	astrotactin 2	9q33.1				0.33	0.00531	0.0405				1
TNIK	TRAF2 and NCK interacting kinase	3q26.31	1.8	0	0	0.349860465	0.005945116	0.037353488				2
ASXL2	additional sex combs like 2 (Drosophila)	2p24.1							-0.709	2.83571	7	1
HTR1D	5-hydroxytryptamine (serotonin) receptor 1D, G protein-coupled	1p36.3-p34.3	1.798	0.00039	0.006							1
ATAD2B	ATPase family, AAA domain containing 2B	2p24.1-p23.3				0.301	0.00057	0.0136				1
ATAD3A	ATPase family, AAA domain containing 3A	1p36.33				-0.447333333	0.002076667	0.0225				1
ATAD3B	ATPase family, AAA domain containing 3B	1p36.33				-0.443	0.0084775	0.03675				1
COL4A1	collagen, type IV, alpha 1	13q34	1.792	0	0	0.316	0.04297	0.1354				2
TMEM133	transmembrane protein 133	11q22.1	1.791	0	0							1
ATCAY	In multiple Geneids					-0.449916667	0.003235	0.025391667				1
ATE1	arginyltransferase 1	10q26.13				-0.3465	0.01899	0.07295				1
PTPRH	protein tyrosine phosphatase, receptor type, H	19q13.4	1.785	0.00034	0.0054							1
ATF4	activating transcription factor 4 (tax-responsive enhancer element B67)	22q13.1				-0.525	0.00011	0.0068				1
ATF6	activating transcription factor 6	1q22-q23				0.352	0.000486667	0.011333333	-0.759	2.434	5	2
ATF7	activating transcription factor 7	12q13				-0.343	0.00057	0.0137				1
TSPAN15	tetraspanin 15	10q22.1	1.785	0	1.00E-04							1
ATG10	autophagy related 10	5q14.1	-0.562	0.00021	0.0038	-0.35475	7.50E-05	0.005425				2
ENPP1	ectonucleotide pyrophosphatase/phosphodiesterase 1	6q22-q23	1.784	0.00346	0.0283							1
C4A	complement component 4A (Rodgers blood group)	6p21.3	1.781	9.00E-05	0.0021							1
ATG16L2	autophagy related 16-like 2 (S. cerevisiae)	11q13.4	-0.397	0.00224	0.0207							1
ARPC1B	actin related protein 2/3 complex, subunit 1B, 41kDa	7q22.1	1.778	0	0							1
ATG2B	autophagy related 2B	14q32.2				-0.32	0.00102	0.0178				1
ATG4A	autophagy related 4A, cysteine peptidase	Xq22.1-q22.3				0.320333333	0.009246667	0.049266667				1
ATG4B	autophagy related 4B, cysteine peptidase	2q37.3				0.3375	0.004575	0.0368				1
ATG4D	autophagy related 4D, cysteine peptidase	19p13.2	-0.671	6.00E-05	0.0015							1
ATG7	autophagy related 7	3p25.3				-0.340083333	0.003360833	0.028508333				1
ATG9B	autophagy related 9B	7q36.1	-2.288	0	0	-0.401	0.00098	0.0174				2
RANBP17	RAN binding protein 17	5q34	1.772	1.00E-05	4.00E-04							1
HOXB5	homeobox B5	17q21.3	1.757	0	1.00E-04							1
ATL1	atlastin GTPase 1	14q22.1	-0.441	0.03548	0.1346							1
ATL3	atlastin GTPase 3	11q13.1	-0.511	0.01308	0.0699							1
MUC2	mucin 2, oligomeric mucus/gel-forming	11p15.5	1.754	0.01322	0.0703							1
ATMIN	ATM interactor	16q23.2	-0.661	0.000205	0.00355							1
ATOH8	atonal homolog 8 (Drosophila)	2p11.2	-0.256	0.01523	0.0774							1
ATP10A	ATPase, class V, type 10A	15q11.2				-0.355	0.01758	0.079				1
ATP10D	ATPase, class V, type 10D	4p12	-1.732	0	3.00E-04							1
LONRF3	LON peptidase N-terminal domain and ring finger 3	Xq24	1.751	0	1.00E-04							1
ATP11B	ATPase, class VI, type 11B	3q27	-1.011	0	1.00E-04							1
HOXC10	homeobox C10	12q13.3	1.75	0	2.00E-04							1
ATP12A	ATPase, H+/K+ transporting, nongastric, alpha polypeptide	13q12.12	-3.246	0	0							1
ATP13A1	ATPase type 13A1	19p13.11				-0.513	0.000185	0.0073				1
ATP13A2	ATPase type 13A2	1p36				-0.38	0.0011	0.0184				1

IFI30	interferon, gamma-inducible protein 30	19p13.1	1.749	1.00E-05	5.00E-04							1
ATP13A4	ATPase type 13A4	3q29				0.3384	0.002246	0.02304	-0.759	2.43857	7	2
ATP13A5	ATPase type 13A5	3q29				0.342625	0.004215	0.032175	-0.766	2.91333	6	2
ATP1A3	ATPase, Na+/K+ transporting, alpha 3 polypeptide	19q13.31				-0.378	0.00011	0.0069				1
ATP1B2	ATPase, Na+/K+ transporting, beta 2 polypeptide	17p13.1				-0.4595	0.000445	0.01165	0.877	2.59	5	2
ATP1B4	ATPase, Na+/K+ transporting, beta 4 polypeptide	Xq24	-0.253	0.03358	0.1297							1
ATP2A1	ATPase, Ca++ transporting, cardiac muscle, fast twitch 1	16p12.1				-0.406	0.00022	0.0091				1
ATP2A2	ATPase, Ca++ transporting, cardiac muscle, slow twitch 2	12q24.11				-0.314	0.00144	0.0207				1
TRAM2	translocation associated membrane protein 2	6p21.1-p12	1.746	0	0	0.492	4.00E-05	0.0049				2
DDC	dopa decarboxylase (aromatic L-amino acid decarboxylase)	7p12.2	1.741	0.00015	0.0029	0.4092	0.00286	0.02354				2
ATP2B2	ATPase, Ca++ transporting, plasma membrane 2	3p25.3				-0.391583333	0.0035975	0.020741667				1
CXorf61	chromosome X open reading frame 61	Xq23	1.739	0.0026	0.0231							1
GCNT1	glucosaminyl (N-acetyl) transferase 1, core 2	9q13	1.738	0.00193	0.0187							1
ATP4A	ATPase, H+/K+ exchanging, alpha polypeptide	19q13.1				-0.317	0.00312	0.0306				1
ATP5A1	ATP synthase, H+ transporting, mitochondrial F1 complex, alpha subunit 1, ca	18q21	-0.458	0.03239	0.1267	-0.347	0.009205	0.054				2
ATP5D	ATP synthase, H+ transporting, mitochondrial F1 complex, delta subunit	19p13.3							-0.606	2.5	6	1
STC1	stanniocalcin 1	8p21-p11.2	1.737	0.00019	0.0036				-0.698	2.79286	7	2
ATP5F1	ATP synthase, H+ transporting, mitochondrial Fo complex, subunit B1	1p13.2	-0.466	0.0028	0.0243							1
ATP5G2	ATP synthase, H+ transporting, mitochondrial Fo complex, subunit C2 (subunit	12q13.13							-0.764	2.678	5	1
ATP5H	In multiple Geneids		-0.457	2.00E-05	6.00E-04							1
ATP5I	ATP synthase, H+ transporting, mitochondrial Fo complex, subunit E	4p16.3	-0.525	0.00381	0.0302							1
ATP5J	ATP synthase, H+ transporting, mitochondrial Fo complex, subunit F6	21q21.1	-0.376	0.0199	0.0923							1
ATP5O	ATP synthase, H+ transporting, mitochondrial F1 complex, O subunit	21q22.11	-0.416	0.03914	0.1435	-0.36175	0.0092625	0.046925				2
ATP5S	ATP synthase, H+ transporting, mitochondrial Fo complex, subunit s (factor B)	14q21.3	-0.333	0.03879	0.1428							1
ATP6AP1L	ATPase, H+ transporting, lysosomal accessory protein 1-like	5q14.2	-0.521	0.00333	0.0276	-0.377	0.000275	0.0087				2
ATP6V0A1	ATPase, H+ transporting, lysosomal V0 subunit a1	17q21				-0.333	0.004695	0.03525				1
ATP6V0A2	ATPase, H+ transporting, lysosomal V0 subunit a2	12q24.31				-0.346666667	0.000233333	0.009133333				1
ATP6V0A4	ATPase, H+ transporting, lysosomal V0 subunit a4	7q34	-1.86	0	1.00E-04	-0.395857143	0.00872	0.044707143				2
CYSTM1	cysteine-rich transmembrane module containing 1	5q31.3	1.734	0.00106	0.0122							1
ATP6V0C	ATPase, H+ transporting, lysosomal 16kDa, V0 subunit c	16p13.3	-0.459	0.00919	0.0551	-0.509	0.00656	0.0454				2
ATP6V0D1	ATPase, H+ transporting, lysosomal 38kDa, V0 subunit d1	16q22.1	-0.607	0.00161	0.0164	-0.366166667	0.007383333	0.04285				2
ATP6V0E1	ATPase, H+ transporting, lysosomal 9kDa, V0 subunit e1	5q35.1				-0.4432	0.005124	0.02946				1
ATP6V1A	ATPase, H+ transporting, lysosomal 70kDa, V1 subunit A	3q13.31	-0.545	0.00762	0.0486							1
ANTXR2	anthrax toxin receptor 2	4q21.21	1.731	8.00E-05	0.0019							1
ATP6V1D	ATPase, H+ transporting, lysosomal 34kDa, V1 subunit D	14q23-q24.2							-0.876	2.89	6	1
ATP6V1E1	ATPase, H+ transporting, lysosomal 31kDa, V1 subunit E1	22q11.1	-0.523	0.00075	0.0096	-0.521	2.75E-05	0.003625				2
ATP6V1F	ATPase, H+ transporting, lysosomal 14kDa, V1 subunit F	7q32	-0.67	0.00045	0.0066	-0.406	2.00E-04	0.0088				2
ATP6V1G3	ATPase, H+ transporting, lysosomal 13kDa, V1 subunit G3	1q31.3				0.355	0.001363333	0.0191	-0.835	3.23714	7	2
ATP6V1H	ATPase, H+ transporting, lysosomal 50/57kDa, V1 subunit H	8q11.2				0.435	0.00081	0.016				1
ATP7A	ATPase, Cu++ transporting, alpha polypeptide	Xq21.1	-0.513	0.01821	0.0872							1
SAA2	serum amyloid A2	11p15.1-p14	1.719	0.00155	0.016							1
ATP8A1	ATPase, aminophospholipid transporter (APLT), class I, type 8A, member 1	4p13				-0.3225	0.004215	0.03545				1
ATP8A2	ATPase, aminophospholipid transporter, class I, type 8A, member 2	13q12				0.4385	0.003565	0.0213				1
ATP8B1	ATPase, aminophospholipid transporter, class I, type 8B, member 1	18q21.31				-0.390727273	0.006909091	0.041027273				1
ATP8B2	ATPase, aminophospholipid transporter, class I, type 8B, member 2	1q21.3							0.9295	2.760715	7	1
ATP8B3	In multiple Geneids					-0.3948	0.00285	0.02796				1
ATP8B4	In multiple Geneids								-1.029	3.10571	7	1
SEMA3C	sema domain, immunoglobulin domain (Ig), short basic domain, secreted, (ser	7q21-q31	1.718	0.00155	0.016	0.37175	0.0214575	0.087925				2
ATP9B	In multiple Geneids					-0.388818182	0.007598182	0.039809091	0.919	3.72714	7	2
ATPAF2	ATP synthase mitochondrial F1 complex assembly factor 2	17p11.2				-0.3425	0.00341	0.03195				1
ATPBD3	Data not found								0.768	2.596	5	1
ANXA4	annexin A4	2p13	1.717	0	1.00E-04							1
FNDC3B	fibronectin type III domain containing 3B	3q26.31	1.712	0	1.00E-04	0.339333333	0.001008333	0.014766667				2
ATRIP	ATR interacting protein	3p21.31				-0.364	0.001215	0.0191				1
ATRNL1	attractin-like 1	10q26							0.43	2.284	5	1
TGFBI	transforming growth factor, beta-induced, 68kDa	5q31	1.711	2.00E-05	8.00E-04							1
ATXN1	ataxin 1	6p23				-0.3165	0.01137	0.06135				1
ATXN10	ataxin 10	22q13.31				-0.338333333	0.001101667	0.016066667				1
ATXN1L	ataxin 1-like	16q22.2				-0.391222222	0.003905556	0.031122222				1
FUCA2	fucosidase, alpha-L- 2, plasma	6q24	1.709	1.00E-05	3.00E-04							1
ATXN2L	ataxin 2-like	16p11				-0.339	0.0066	0.0456				1
ATXN7	ataxin 7	3p21.1-p12				-0.332	0.00616	0.0439				1
ATXN7L1	In multiple Geneids					-0.343833333	0.005948333	0.03735				1
ANXA13	annexin A13	8q24.13	1.702	0.00015	0.003	0.315	0.017996	0.0754	-0.869	3.06429	7	3
TSPAN3	tetraspanin 3	15q24.3	1.702	0	0							1
TRPA1	transient receptor potential cation channel, subfamily A, member 1	8q13	1.701	0.0013	0.0141	0.337	0.002253333	0.0259				2
AURKC	aurora kinase C	19q13.43				-0.383	0.01273	0.0656				1
AUTS2	autism susceptibility candidate 2	7q11.22				0.36325	0.00278125	0.02355				1
AVEN	apoptosis, caspase activation inhibitor	15q13.1				-0.3435	0.00131	0.01965				1

MIR192	microRNA 192	11q13.1	1.7	3.00E-05	0.001							1
AVP	arginine vasopressin	20p13				-0.485	7.00E-05	0.0058				1
AVPR1A	arginine vasopressin receptor 1A	12q14-q15							-0.94	2.81333	6	1
AWAT2	acyl-CoA wax alcohol acyltransferase 2	Xq13.1	-0.255	0.00545	0.0387							1
AXIN1	axin 1	16p13.3				-0.465909091	0.014041818	0.061454545				1
AXIN2	axin 2	17q23-q24				-0.363	0.00121	0.0193				1
AXL	AXL receptor tyrosine kinase	19q13.1				-0.351	6.00E-05	0.0054				1
FBP1	fructose-1,6-bisphosphatase 1	9q22.3	1.698	0.00041	0.0062							1
AZI1	5-azacytidine induced 1	17q25.3				-0.357	0.00125	0.0195				1
AZI2	5-azacytidine induced 2	3p24.1							-1.324	2.874	5	1
CHDH	choline dehydrogenase	3p21.1	1.697	0	1.00E-04							1
B3GALT2	UDP-Gal:betaGlcNAc beta 1,3-galactosyltransferase, polypeptide 2	1q31							-0.753	2.69143	7	1
B3GALT4	UDP-Gal:betaGlcNAc beta 1,3-galactosyltransferase, polypeptide 4	6p21.3	-0.327	0.00427	0.0327							1
B3GALT5	UDP-Gal:betaGlcNAc beta 1,3-galactosyltransferase, polypeptide 5	21q22.3				-0.327	0.00178	0.0232				1
B3GALTL	beta 1,3-galactosyltransferase-like	13q12.3				0.402333333	0.014546667	0.060666667				1
B3GAT2	In multiple Geneids		-0.286	0.01304	0.0698				0.685	2.75	5	2
ACY3	aspartoacylase (aminocyclase) 3	11q13.2	1.696	0	2.00E-04							1
B3GNT3	UDP-GlcNAc:betaGal beta-1,3-N-acetylglucosaminyltransferase 3	19p13.1				-0.379	0.00499	0.0392				1
B3GNT4	UDP-GlcNAc:betaGal beta-1,3-N-acetylglucosaminyltransferase 4	12q24	-0.227	0.04774	0.1625							1
B3GNT6	UDP-GlcNAc:betaGal beta-1,3-N-acetylglucosaminyltransferase 6 (core 3 synt	11q13.4	-0.58	0.00088	0.0107							1
VPS13C	vacuolar protein sorting 13 homolog C (S. cerevisiae)	15q22.2	1.684	0	2.00E-04							1
B3GNT8	UDP-GlcNAc:betaGal beta-1,3-N-acetylglucosaminyltransferase 8	19q13.2	-1.065	0	1.00E-04							1
ENC1	ectodermal-neural cortex 1 (with BTB-like domain)	5q13	1.681	1.00E-05	3.00E-04							1
B3GNTL1	In multiple Geneids								0.891	3.4775	8	1
B4GALNT2	beta-1,4-N-acetyl-galactosaminyl transferase 2	17q21.32				-0.379	0.0134	0.0675	0.816	2.938	5	2
B4GALT1	UDP-Gal:betaGlcNAc beta 1,4- galactosyltransferase, polypeptide 1	9p13				-0.399230769	0.004362308	0.023761538				1
MSR1	macrophage scavenger receptor 1	8p22	1.681	1.00E-05	4.00E-04							1
MAN1A1	mannosidase, alpha, class 1A, member 1	6q22	1.673	3.00E-05	0.001							1
B4GALT6	UDP-Gal:betaGlcNAc beta 1,4- galactosyltransferase, polypeptide 6	18q11				-0.325	0.00868	0.053				1
B4GALT7	xylosylprotein beta 1,4-galactosyltransferase, polypeptide 7 (galactosyltransfe	5q35.2-q35.3				-0.461	8.00E-05	0.00615				1
B9D1	B9 protein domain 1	17p11.2				-0.346	0.00768	0.049				1
B9D2	B9 protein domain 2	19q13.2				-0.466	0.00516	0.0399				1
RARRES3	retinoic acid receptor responder (tazarotene induced) 3	11q23	1.673	0.00191	0.0185							1
BACE1	beta-site APP-cleaving enzyme 1	11q23.2-q23.3				-0.379	6.00E-04	0.014				1
BACE2	beta-site APP-cleaving enzyme 2	21q22.3				-0.3435	0.000295	0.00935				1
BACH2	BTB and CNC homology 1, basic leucine zipper transcription factor 2	6q15				-0.34	0.00163625	0.01915				1
BAD	BCL2-associated agonist of cell death	11q13.1	-0.191	0.01953	0.0913							1
BAG1	BCL2-associated athanogene	9p12				-0.366	0.00029	0.0101				1
IGFBP7	insulin-like growth factor binding protein 7	4q12	1.671	0	1.00E-04							1
BAG3	BCL2-associated athanogene 3	10q25.2-q26.2	-0.51	0.01062	0.061	-0.395	0.000565	0.01325				2
BAG5	BCL2-associated athanogene 5	14q32.33	-0.313	0.03977	0.145	-0.327	0.00075	0.0154				2
BAHD1	bromo adjacent homology domain containing 1	15q15.1				-0.336	0.00386	0.0322	0.56	2.60333	6	2
BAI2	brain-specific angiogenesis inhibitor 2	1p35				-0.391666667	6.67E-05	0.005				1
BAI3	brain-specific angiogenesis inhibitor 3	6q12				0.414	0.002323333	0.022366667				1
BAIAP2	BAI1-associated protein 2	17q25	-1.545	0	0							1
ST6GAL1	ST6 beta-galactosamide alpha-2,6-sialyltranferase 1	3q27-q28	1.669	0.00054	0.0075							1
ATP7B	ATPase, Cu++ transporting, beta polypeptide	13q14.3	1.667	0.00032	0.0052	0.362	0.01882	0.0822				2
FMO5	flavin containing monooxygenase 5	1q21.1	1.661	0.00109	0.0124	0.3065	0.01306	0.06635				2
LOC100128816	ACAH3104	12p13.32	1.66	0.00027	0.0045							1
BANK1	B-cell scaffold protein with ankyrin repeats 1	4q24							-0.955	2.764	5	1
BANP	BTG3 associated nuclear protein	16q24				-0.3615	0.007795	0.0477				1
BAP1	BRCA1 associated protein-1 (ubiquitin carboxy-terminal hydrolase)	3p21.31-p21.2	-0.29	0.0437	0.1538							1
BARD1	BRCA1 associated RING domain 1	2q34-q35				0.329	0.00013	0.0073				1
BARHL1	BarH-like homeobox 1	9q34							0.896	2.845	6	1
BARX2	BARX homeobox 2	11q25	-2.122	0	2.00E-04							1
BAT2	Data not found								0.838	2.96083	6	1
BAT4	Data not found								0.828	3.3	6	1
ADH6	alcohol dehydrogenase 6 (class V)	4q23	1.659	0.00291	0.025				-0.982	2.984	5	2
NPNT	nephronectin	4q24	1.657	0.00092	0.011							1
SGCE	sarcoglycan, epsilon	7q21.3	1.656	5.00E-04	0.0071	0.329666667	0.0024	0.026633333				2
BAZ1A	bromodomain adjacent to zinc finger domain, 1A	14q13.2				-0.397	0.02547	0.0984				1
DSG2	desmoglein 2	18q12.1	1.652	3.00E-05	0.001							1
BAZ2A	bromodomain adjacent to zinc finger domain, 2A	12q13.3				-0.407	0.00654	0.0453				1
BBC3	BCL2 binding component 3	19q13.3-q13.4				-0.354	0.001	0.0176	1.184	3.075	6	2
BBOX1	butyrobetaine (gamma), 2-oxoglutarate dioxygenase (gamma-butyrobetaine h	11p14.2							-0.83	2.92167	6	1
BBS1	Bardet-Biedl syndrome 1	11q13				-0.369	0.001153333	0.0182				1
ADAP1	ArfGAP with dual PH domains 1	7p22.3	1.65	0	0							1
BBS2	Bardet-Biedl syndrome 2	16q21	-0.744	0.00545	0.0388							1
BBS9	Bardet-Biedl syndrome 9	7p14				0.336666667	0.003777778	0.024077778				1

BBX	bobby sox homolog (Drosophila)	3q13.1				0.3105	0.016045	0.06325				1
BCAP29	B-cell receptor-associated protein 29	7q22-q31	-0.541	0.00069	0.0091							1
FARP1	FERM, RhoGEF (ARHGEF) and pleckstrin domain protein 1 (chondrocyte-derive	13q32.2	1.644	1.00E-05	3.00E-04							1
BCAR3	breast cancer anti-estrogen resistance 3	1p22.1	-0.306	0.04833	0.1638							1
BCAS1	breast carcinoma amplified sequence 1	20q13.2				0.482766667	0.016403	0.072766667				1
BCAS2	breast carcinoma amplified sequence 2	1p13.2				-0.351333333	0.001473333	0.0152				1
BCAS3	In multiple Geneids					-0.3305	0.00565	0.03915				1
PRTFDC1	phosphoribosyl transferase domain containing 1	10p12.1	1.641	1.00E-05	3.00E-04							1
BCAT2	branched chain amino-acid transaminase 2, mitochondrial	19q13				-0.341	0.0111025	0.059675				1
BCDIN3	Data not found								0.593	2.38625	8	1
BCDO2	Data not found								-0.807	3.03714	7	1
BCHE	butyrylcholinesterase	3q26.1-q26.2				0.353333333	0.00226	0.023733333	-0.845	3.16444	9	2
BCKDHA	branched chain keto acid dehydrogenase E1, alpha polypeptide	19q13.1-q13.2	-1.6	0	1.00E-04	-0.341	0.00055	0.0135	1.215	3.24667	6	3
BCKDHB	branched chain keto acid dehydrogenase E1, beta polypeptide	6q14.1							-1.047	2.98333	6	1
BCKDK	branched chain ketoacid dehydrogenase kinase	16p11.2				-0.304	0.01624	0.0755				1
BCL11A	B-cell CLL/lymphoma 11A (zinc finger protein)	2p16.1				0.614	5.00E-06	0.00265				1
BCL11B	B-cell CLL/lymphoma 11B (zinc finger protein)	14q32.2	-0.66	9.00E-05	0.002	-0.39	0.0007875	0.014675				2
BCL2	B-cell CLL/lymphoma 2	18q21.3	-0.399	0.01764	0.0853	-0.354428571	0.004364286	0.031028571				2
MFAP2	microfibrillar-associated protein 2	1p36.1-p35	1.64	3.00E-05	9.00E-04							1
SLC34A2	solute carrier family 34 (sodium phosphate), member 2	4p15.2	1.631	0.00123	0.0135							1
BCL2L10	BCL2-like 10 (apoptosis facilitator)	15q21	-0.494	0.02752	0.1138	-0.4	0.01814	0.0805				2
COL3A1	collagen, type III, alpha 1	2q31	1.629	6.00E-05	0.0015	0.373333333	0.000216667	0.008066667	-0.977	2.97833	6	3
SLC20A1	solute carrier family 20 (phosphate transporter), member 1	2q13	1.627	0	1.00E-04							1
BCL2L13	BCL2-like 13 (apoptosis facilitator)	22q11.1	-0.459	0.00061	0.0083	-0.421230769	0.003653077	0.023323077				2
ICAM1	intercellular adhesion molecule 1	19p13.3-p13.2	1.626	2.00E-05	7.00E-04							1
PLEKHB1	pleckstrin homology domain containing, family B (evectins) member 1	11q13.5-q14.1	1.626	0.00038	0.0059							1
BCL2L2	BCL2-like 2	14q11.2-q12	-0.43	8.00E-04	0.01							1
BCL3	B-cell CLL/lymphoma 3	19q13.1-q13.2				-0.459	0.00091	0.0169				1
BCL6	B-cell CLL/lymphoma 6	3q27				0.3165	0.00374	0.0335	-0.817	2.646	5	2
BCL7A	B-cell CLL/lymphoma 7A	12q24.13				-0.343	0.000785	0.01575				1
BCL7B	B-cell CLL/lymphoma 7B	7q11.23				-0.3965	0.012145	0.0639				1
BCL8	Data not found					-0.469	0.015756667	0.063733333				1
PODXL	podocalyxin-like	7q32-q33	1.623	0	0							1
TINAGL1	tubulointerstitial nephritis antigen-like 1	1p35.2	1.623	0	1.00E-04							1
BCMP11	Data not found								-0.762	2.81167	6	1
BCO2	beta-carotene oxygenase 2	11q22.3-q23.1	-0.499	0.01448	0.0747							1
BCOR	BCL6 corepressor	Xp11.4							0.539	2.49857	7	1
BCORL1	BCL6 corepressor-like 1	Xq25-q26.1				-0.392	0.00433	0.0363				1
BCORL2	Data not found					-0.561454545	0.021096364	0.083627273				1
BCR	breakpoint cluster region	22q11.23	-0.455666667	0.0015	0.0146	-0.370666667	0.000983333	0.0133				2
BCS1L	BCS1-like (S. cerevisiae)	2q33				-0.308	0.00384	0.0341				1
BDH1	3-hydroxybutyrate dehydrogenase, type 1	3q29	-0.876	0.00024	0.0041							1
BDH2	In multiple Geneids		-0.451	0.04971	0.1665							1
BDKRB1	bradykinin receptor B1	14q32.1-q32.2	-1.89	1.00E-05	3.00E-04							1
BDKRB2	bradykinin receptor B2	14q32.1-q32.2	-0.921	1.00E-05	3.00E-04	-0.334	0.00011	0.0069				2
BDNF	brain-derived neurotrophic factor	11p13							0.831	2.73857	7	1
BDNFOS	Data not found					-0.355	0.00374	0.0336				1
BDP1	B double prime 1, subunit of RNA polymerase III transcription initiation factor	5q13				-0.339666667	0.00034	0.010266667				1
BEAN	Data not found					-0.343	0.008235714	0.046614286				1
BEND2	BEN domain containing 2	Xp22.13				-0.368	0.01217	0.0639				1
BEND3	BEN domain containing 3	6q21				-0.365	0.003996667	0.028966667				1
BEND4	BEN domain containing 4	4p13				-0.3606	0.002178	0.02148				1
PPARG	peroxisome proliferator-activated receptor gamma	3p25	1.622	0.00014	0.0029				-0.883	3.71143	7	2
UGT8	UDP glycosyltransferase 8	4q26	1.619	0.00132	0.0143				-0.977	2.48	6	2
BEST2	bestrophin 2	19p13.2				-0.4055	0.016265	0.0645				1
RHOBTB3	Rho-related BTB domain containing 3	5q15	1.618	8.00E-05	0.0018							1
TFPI	tissue factor pathway inhibitor (lipoprotein-associated coagulation inhibitor)	2q32	1.618	6.00E-05	0.0015	0.338666667	0.000346667	0.010666667	-0.725	2.77857	7	3
BET3L	In multiple Geneids								-0.89	2.932	5	1
BEX4	brain expressed, X-linked 4	Xq22.1-q22.3	-1.048	0.00075	0.0096							1
POTEE	POTE ankyrin domain family, member E	2q21.1	1.617666667	0.00847	0.051433333							1
BFSP2	beaded filament structural protein 2, phakinin	3q22.1				-0.366	0.00231	0.0264				1
CYP4F30P	cytochrome P450, family 4, subfamily F, polypeptide 30, pseudogene	2q21.1	1.617	0.02232	0.0959							1
BHLHA9	basic helix-loop-helix family, member a9	17p13.3	-0.293	0.04699	0.1609							1
BHLHB8	Data not found								0.472	2.636	5	1
BHLHE40	basic helix-loop-helix family, member e40	3p26	-0.785	0.01805	0.0867							1
GLB1L	galactosidase, beta 1-like	2q35	1.616	0	0							1
BHMT2	betaine--homocysteine S-methyltransferase 2	5q13				-0.338666667	0.004483333	0.0303				1
KCNK5	potassium channel, subfamily K, member 5	6p21	1.616	1.00E-05	5.00E-04							1
PRR15	proline rich 15	7p14.3	1.612	4.00E-05	0.0011							1

BICD2	bicaudal D homolog 2 (Drosophila)	9q22.31	-1.024	1.00E-05	3.00E-04	-0.3835	0.01443	0.0582				2
ANKRD10	ankyrin repeat domain 10	13q34	1.608	0	1.00E-04	0.33	0.02377	0.0945				2
CD97	CD97 molecule	19p13	1.607	3.00E-05	8.00E-04							1
FAM198B	family with sequence similarity 198, member B	4q32.1	1.605	3.00E-05	8.00E-04							1
BIN2	bridging integrator 2	12q13				-0.3455	0.00095	0.0155				1
BIN3	In multiple Geneids					-0.3415	0.00111	0.0174				1
DDX60L	DEAD (Asp-Glu-Ala-Asp) box polypeptide 60-like	4q32.3	1.601	9.00E-05	0.002							1
DPY19L1	dpy-19-like 1 (C. elegans)	7p14.3-p14.2	1.601	0	1.00E-04	0.353666667	0.001773333	0.021166667				2
BIRC5	baculoviral IAP repeat containing 5	17q25				-0.348	0.00214	0.0254				1
BIRC6	baculoviral IAP repeat containing 6	2p22.3				0.387	0.00117	0.0189				1
BIVM	In multiple Geneids								-0.882	2.732	5	1
BLCAP	bladder cancer associated protein	20q11.23	-0.307	0.01357	0.0716							1
IGLJ3	immunoglobulin lambda joining 3	22q11.2	1.595	0.03468	0.1325							1
BLNK	B-cell linker	10q23.2-q23.33							-0.626	2.555	6	1
BLOC1S1	biogenesis of lysosomal organelles complex-1, subunit 1	12q13-q14	-0.579	0.00462	0.0346							1
BLOC1S2	biogenesis of lysosomal organelles complex-1, subunit 2	10q24.31				-0.334	8.00E-05	0.0061				1
BLOC1S3	biogenesis of lysosomal organelles complex-1, subunit 3	19q13.32				-0.479	0.00021	0.0089				1
ARL14	ADP-ribosylation factor-like 14	3q25.33	1.594	0.01688	0.0828							1
BLVRA	biliverdin reductase A	7p14-cen	-0.752	0.00312	0.0263							1
BLVRB	biliverdin reductase B (flavin reductase (NADPH))	19q13.1-q13.2	-0.94	0.00013	0.0027	-0.4215	0.00157	0.0218	1.101	2.99167	6	3
BLZF1	basic leucine zipper nuclear factor 1	1q24				0.365333333	0.000493333	0.0124				1
PIWIL4	In multiple Geneids		1.586	0.00025	0.0043							1
SLC39A7	solute carrier family 39 (zinc transporter), member 7	6p21.3	1.584	2.00E-05	6.00E-04							1
BMP1	bone morphogenetic protein 1	8p21.3				-0.421666667	0.00194	0.022333333				1
BMP10	bone morphogenetic protein 10	2p13.3	-0.199	0.04726	0.1615							1
MMD	monocyte to macrophage differentiation-associated	17q	1.583	2.00E-05	6.00E-04							1
BMP2K	BMP2 inducible kinase	4q21.21	-0.485	0.04814	0.1634							1
BMP4	bone morphogenetic protein 4	14q22-q23				-0.39	0.00171	0.0227				1
BMP5	bone morphogenetic protein 5	6p12.1				0.334333333	0.00858	0.048266667	-0.93	3.28125	8	2
BMP6	bone morphogenetic protein 6	6p24-p23				-0.33	0.00328	0.0314				1
BMP7	bone morphogenetic protein 7	20q13				0.3565	0.004585	0.03735				1
ATF7IP2	activating transcription factor 7 interacting protein 2	16p13.13	1.582	6.00E-05	0.0016							1
FSTL1	follistatin-like 1	3q13.33	1.582	6.00E-05	0.0015							1
BMPER	BMP binding endothelial regulator	7p14.3				0.386416667	0.001816667	0.020325				1
BMPR1A	bone morphogenetic protein receptor, type IA	10q22.3				-0.336	1.00E-05	0.0031				1
BMPR1B	bone morphogenetic protein receptor, type IB	4q22-q24				-0.336142857	0.00305	0.027571429				1
BMS1	BMS1 homolog, ribosome assembly protein (yeast)	10q11.21	-0.892	0.00196	0.0188							1
BMX	BMX non-receptor tyrosine kinase	Xp22.2				0.332	0.008435	0.05065				1
BNC1	basonuclin 1	15q25.2	-2.654	0	2.00E-04							1
BNC2	basonuclin 2	9p22.2				-0.381318841	0.003642319	0.027195652				1
BNIP1	BCL2/adenovirus E1B 19kDa interacting protein 1	5q33-q34				-0.334	0.003545	0.03055				1
BNIP3	BCL2/adenovirus E1B 19kDa interacting protein 3	10q26.3	-2.25	3.00E-05	8.00E-04	-0.397	0.00419	0.0357				2
BNIPL	In multiple Geneids		-3.868	0	0							1
BOC	Boc homolog (mouse)	3q13.2	-1.511	0.00325	0.0272							1
BOD1	biorientation of chromosomes in cell division 1	5q35.2				-0.302	0.00562	0.0417				1
BOD1L	Data not found					-0.342	0.0039	0.0344				1
BOK	BCL2-related ovarian killer	2q37.3				2.146	0	6.00E-04				1
NEK6	In multiple Geneids		1.582	0	1.00E-04							1
BOLA2B	bolA homolog 2B (E. coli)	16p11.2							0.9855	3.289245	8	1
BIRC3	baculoviral IAP repeat containing 3	11q22	1.58	0.00034	0.0054							1
BOP1	block of proliferation 1	8q24.3				-0.347	0.02255	0.0916				1
FAT1	FAT tumor suppressor homolog 1 (Drosophila)	4q35	1.58	0	2.00E-04	0.879	0.00032	0.0107				2
BPESC1	blepharophimosis, epicanthus inversus and ptosis, candidate 1 (non-protein c	3q23	-0.236	0.01424	0.0739							1
BPGM	2,3-bisphosphoglycerate mutase	7q31-q34	-1.179	5.00E-05	0.0014							1
BPHL	In multiple Geneids					-0.36	0.00085	0.0164				1
BPIFA3	BPI fold containing family A, member 3	20q11.21	-0.309	0.0086	0.0527							1
C4BPB	complement component 4 binding protein, beta	1q32	1.579	5.00E-05	0.0014				-0.649	2.78	5	2
BPIL2						-0.382888889	0.000315556	0.008377778				1
TNFRSF10D	tumor necrosis factor receptor superfamily, member 10d, decoy with truncate	8p21	1.576	0	0							1
BPY2	basic charge, Y-linked, 2	Yq11				-0.455	0.025697692	0.095526923				1
BPY2B	basic charge, Y-linked, 2B	Yq11.223				-0.455	0.025697692	0.095526923				1
BPY2C	basic charge, Y-linked, 2C	Yq11.223				-0.455	0.025697692	0.095526923				1
BRAF	v-raf murine sarcoma viral oncogene homolog B1	7q34				-0.309	0.04027	0.1301				1
BRAP	BRCA1 associated protein	12q24				-0.3475	0.0026375	0.02545				1
HIST2H4A	histone cluster 2, H4a	1q21.2	1.572	4.00E-05	0.0011							1
SLC17A9	solute carrier family 17, member 9	20q13.33	1.571	0	2.00E-04							1
BRCC2	Data not found								-0.824	3.14857	7	1
BRCC3	BRCA1/BRCA2-containing complex, subunit 3	Xq28				0.371	2.00E-04	0.0088				1
BRD1	bromodomain containing 1	22q13.33				-0.425875	0.00045875	0.0105375	0.58	2.76167	6	2

BRD2	bromodomain containing 2	6p21.3				0.324	0.00753	0.049				1
TNFAIP3	tumor necrosis factor, alpha-induced protein 3	6q23	1.571	2.00E-05	6.00E-04							1
BRD4	bromodomain containing 4	19p13.1				-0.372769231	0.004032308	0.031569231				1
BRD8	bromodomain containing 8	5q31							-0.767	2.53417	6	1
PON2	paraoxonase 2	7q21.3	1.567	0	2.00E-04							1
BRDG1	Data not found								-1.012	3.22571	7	1
BRDT	bromodomain, testis-specific	1p22.1				-0.45	0.00235	0.0266				1
BRE	brain and reproductive organ-expressed (TNFRSF1A modulator)	2p23.2				0.324	0.006545	0.04475				1
BRF1	BRF1 homolog, subunit of RNA polymerase III transcription initiation factor IIII	14q				-0.3204	0.010404	0.04688				1
CHN1	chimerin (chimaerin) 1	2q31.1	1.564	0	1.00E-04							1
DENND5B	DENN/MADD domain containing 5B	12p11.21	1.564	0.00013	0.0027							1
BHLHE41	basic helix-loop-helix family, member e41	12p12.1	1.558	0.00455	0.0343							1
BRINP1	Data not found		-0.388	0.03421	0.1312							1
EPSTI1	epithelial stromal interaction 1 (breast)	13q13.3	1.558	0.00121	0.0134	0.31	0.00804	0.0508				2
BRK1	BRICK1, SCAR/WAVE actin-nucleating complex subunit	3p25.3	-0.4975	0.01694	0.0771							1
ZG16B	zymogen granule protein 16 homolog B (rat)	16p13.3	1.558	5.00E-05	0.0013							1
BRP44	brain protein 44	1q24				0.332	0.00078	0.0157				1
BRPF1	bromodomain and PHD finger containing, 1	3p26-p25				-0.404	0.0011925	0.014675				1
SPATA13	spermatogenesis associated 13	13q12.12	1.555	1.00E-05	5.00E-04	0.316666667	0.006663333	0.0445				2
BRSK1	BR serine/threonine kinase 1	19q13.4				-0.417666667	0.01285	0.058266667	0.854	2.748	5	2
BRSK2	BR serine/threonine kinase 2	11p15.5				-0.362	0.00247	0.0272	0.868	2.925	6	2
BRUNOL4	Data not found					-0.364266667	0.014856	0.067686667				1
BRUNOL5	Data not found					-0.436727273	0.014858182	0.062936364				1
BRWD1	bromodomain and WD repeat domain containing 1	21q22.2				-0.417571429	0.001345714	0.016414286				1
BSCL2	Berardinelli-Seip congenital lipodystrophy 2 (seipin)	11q13				-0.3815	0.00109	0.01695	1.352	3.036	5	2
BSDC1	BSD domain containing 1	1p35.1				-0.40175	0.00975	0.046975				1
BSN	bassoon (presynaptic cytomatrix protein)	3p21.31				-0.377785714	0.00364	0.026785714				1
BSPRY	B-box and SPRY domain containing	9q32	-0.644	0.0013	0.0141	-0.317	0.00505	0.0394				2
MLPH	melanophilin	2q37.3	1.55	0.00073	0.0094							1
NFATC2	nuclear factor of activated T-cells, cytoplasmic, calcineurin-dependent 2	20q13.2	1.55	1.00E-05	3.00E-04	0.404333333	0.000503333	0.0112				2
BSX	brain-specific homeobox	11q24.1	-0.214	0.01484	0.076							1
BTBD10	BTB (POZ) domain containing 10	11p15.2	-0.467	0.01457	0.075							1
BTBD11	BTB (POZ) domain containing 11	12q23.3	-2.647	0	2.00E-04							1
BTBD12	Data not found					-0.524714286	0.002972857	0.027571429				1
NEK3	NIMA (never in mitosis gene a)-related kinase 3	13q14.13	1.548	0	1.00E-04							1
BTBD2	BTB (POZ) domain containing 2	19p13.3	-0.273	0.03809	0.1411	-0.5165	0.0006375	0.014025				2
WFDC2	WAP four-disulfide core domain 2	20q12-q13.2	1.547	0.00021	0.0038							1
BTBD7	BTB (POZ) domain containing 7	14q32.12	-0.328	0.01089	0.0619	-0.322	0.001596667	0.021633333				2
BTBD9	BTB (POZ) domain containing 9	6p21				0.3379	0.010808	0.05755				1
BTC	betacellulin	4q13-q21	-0.335	0.022	0.0988							1
BTD	In multiple Geneids					-0.374	5.00E-04	0.0129				1
BTF3	basic transcription factor 3	5q13.2				-0.348	0.01481	0.0716				1
BTF3L4	basic transcription factor 3-like 4	1p32.3	-0.434	0.02349	0.1028	-0.3705	0.000335	0.00945				2
TNFRSF10B	tumor necrosis factor receptor superfamily, member 10b	8p22-p21	1.545	0	0							1
BTG4	B-cell translocation gene 4	11q23				0.306	0.00103	0.0179				1
BTLA	B and T lymphocyte associated	3q13.2							-0.67	3.00667	6	1
ALDOB	aldolase B, fructose-bisphosphate	9q21.3-q22.2	1.544	0.04301	0.1525							1
DTX4	deltex homolog 4 (Drosophila)	11q12.1	1.544	0	0							1
TMEM92	transmembrane protein 92	17q21.33	1.543	4.00E-05	0.0012							1
LAMA4	laminin, alpha 4	6q21	1.54	6.00E-05	0.0016				-0.928	3.368	5	2
TMEM63A	transmembrane protein 63A	1q42.12	1.536	0	0							1
IGFBP3	insulin-like growth factor binding protein 3	7p13-p12	1.535	8.00E-05	0.0019							1
BTNL3	butyrophilin-like 3	5q35.3				-1.082333333	0.02037	0.085833333				1
ALDH1A1	aldehyde dehydrogenase 1 family, member A1	9q21.13	1.534	0.0107	0.0613				-0.975	3.456	5	2
BTNL9	butyrophilin-like 9	5q35.3				0.333	0.00141	0.0207				1
BTRC	beta-transducin repeat containing E3 ubiquitin protein ligase	10q24.32	-0.607	0.00058	0.008	-0.317333333	0.000526667	0.011933333				2
CDR2	cerebellar degeneration-related protein 2, 62kDa	16p12.3	1.528	0	0							1
C10orf10	chromosome 10 open reading frame 10	10q11.21	1.527	0.00099	0.0116							1
BXDC1	Data not found					-0.401	0	0.0023				1
BZRAP1	benzodiazapine receptor (peripheral) associated protein 1	17q22-q23	-0.246	0.00354	0.0287				0.82	2.90571	7	2
BZW2	basic leucine zipper and W2 domains 2	7p21.1				0.311	0.0016	0.022				1
RNU4-2	RNA, U4 small nuclear 2	12q24.31	1.527	0.01964	0.0915							1
C10orf107	chromosome 10 open reading frame 107	10q21.2							-0.743	2.53571	7	1
C10orf108	chromosome 10 open reading frame 108	10p15.3				-0.337	0.00263	0.0281				1
C10orf11	chromosome 10 open reading frame 11	10q22.3				-0.385	0.01406	0.0694				1
C10orf111	chromosome 10 open reading frame 111	10p13	-0.221	0.03972	0.1449							1
C10orf114	In multiple Geneids								-0.955	2.68167	6	1
C10orf118	chromosome 10 open reading frame 118	10q25.3							-0.754	2.62333	6	1
C10orf119	Data not found					-0.303	0.00011	0.0069				1

C10orf12	chromosome 10 open reading frame 12	10q24.1	-0.321	0.02823	0.1159							1
C10orf120	chromosome 10 open reading frame 120	10q26.13	-0.3	0.01303	0.0698							1
C10orf129	In multiple Geneids								-0.883	3.04	6	1
C10orf13	Data not found								-0.859	3.08714	7	1
C10orf141	Data not found					-0.34	0.00014	0.0075				1
ALDH1B1	aldehyde dehydrogenase 1 family, member B1	9p11.1	1.525	0.00012	0.0025							1
VCAM1	vascular cell adhesion molecule 1	1p32-p31	1.524	0.00013	0.0026				-0.932	3.10333	6	2
C10orf26	Data not found					-0.4245	0.002955	0.0246				1
C10orf46	chromosome 10 open reading frame 46	10q26.11				-0.322	0.00114	0.0187				1
C10orf54	chromosome 10 open reading frame 54	10q22.1	-0.469	0.00779	0.0493							1
C10orf55	chromosome 10 open reading frame 55	10q22.2				-0.320333333	0.00179	0.019866667				1
C10orf57	chromosome 10 open reading frame 57	10q22.3				-0.369	0.0016	0.022				1
C10orf58	Data not found					-0.369	4.00E-05	0.0045				1
C10orf62	chromosome 10 open reading frame 62	10q24.2				-0.414	0	0.0014				1
C10orf63	Data not found								-0.801	2.788	5	1
C10orf68	chromosome 10 open reading frame 68	10p11.22				0.487	7.00E-05	0.0059	-0.987	2.52714	7	2
C10orf7	Data not found								-0.733	2.62286	7	1
C10orf76	chromosome 10 open reading frame 76	10q24.32	-0.526	0.00017	0.0033	-0.345608696	0.001110435	0.015708696				2
C10orf79	Data not found					-0.302	0.00072	0.0152				1
C10orf85	chromosome 10 open reading frame 85	10q26.12							-0.98	2.58833	6	1
PCDHB8	protocadherin beta 8	5q31	1.521	0.00164	0.0166							1
C10orf90	chromosome 10 open reading frame 90	10q26.2							-0.77	2.5375	8	1
C10orf92	Data not found					-0.3195	0.00016	0.00795				1
C10orf93	chromosome 10 open reading frame 93	10q26.3				-0.353	0.00011	0.0068				1
C10orf96	Data not found					0.352	0.00049	0.0128				1
C10orf99	chromosome 10 open reading frame 99	10q23.1	-3.169	1.00E-05	5.00E-04							1
C11orf16	chromosome 11 open reading frame 16	11p15.3	-0.328	0.00148	0.0154							1
C11orf2	chromosome 11 open reading frame 2	11q13				-0.392	0.00052	0.0131				1
KCNE3	potassium voltage-gated channel, Isk-related family, member 3	11q13.4	1.52	4.00E-05	0.0012							1
C11orf34	chromosome 11 open reading frame 34	11q23.1	-0.269	0.02501	0.1071							1
C11orf35	chromosome 11 open reading frame 35	11p15.5	-0.29	0.0059	0.0409							1
C11orf38	Data not found								-0.964	3.064	5	1
C11orf40	chromosome 11 open reading frame 40	11p15.5							-0.807	3.01143	7	1
C11orf41	chromosome 11 open reading frame 41	11p13				-0.324	0.00052	0.0131				1
C11orf42	chromosome 11 open reading frame 42	11p15.4							-0.813	2.95857	7	1
C11orf43	chromosome 11 open reading frame 43	11p15.5							0.641	2.590446667	6	1
C11orf45	chromosome 11 open reading frame 45	11q24.3	-0.468	0.0028	0.0243							1
HAVCR1	hepatitis A virus cellular receptor 1	5q33.2	1.519	0.02464	0.106							1
CD276	CD276 molecule	15q23-q24	1.517	0	0							1
C11orf51	Data not found								-0.756	2.784	5	1
ONECUT2	one cut homeobox 2	18q21.31	1.517	0.00018	0.0033							1
C11orf54	chromosome 11 open reading frame 54	11q21							-0.956	3.064	5	1
C11orf65	chromosome 11 open reading frame 65	11q22.3				-0.321	0.00553	0.0414				1
C11orf66	Data not found					-0.357	0.00222	0.0203				1
C11orf69	chromosome 11 open reading frame 69	11p13							-0.872	2.90833	6	1
C11orf72	In multiple Geneids		-0.285	0.04383	0.1542							1
C11orf74	chromosome 11 open reading frame 74	11p12							-0.886	3.17429	7	1
C11orf75	chromosome 11 open reading frame 75	11q21				0.367	0	0.0016				1
C11orf76	Data not found								0.758	2.75667	6	1
C11orf80	chromosome 11 open reading frame 80	11q13.2	-0.548	0.029	0.1179							1
SLC38A11	solute carrier family 38, member 11	2q24.3	1.515	0.00071	0.0092	0.311	0.00234	0.0265				2
C11orf9	chromosome 11 open reading frame 9	11q12-q13.1				-0.604	0	0.0019				1
SCARNA17	small Cajal body-specific RNA 17	18q21.1	1.514	2.00E-05	6.00E-04							1
C12orf10	chromosome 12 open reading frame 10	12q13				-0.3025	0.00063	0.0142				1
ABCC2	ATP-binding cassette, sub-family C (CFTR/MRP), member 2	10q24	1.513	0.01432	0.0742							1
C12orf24	Data not found					-0.393	0.0028	0.029				1
C12orf27	Data not found					-0.325	0.00105	0.0181				1
C12orf29	chromosome 12 open reading frame 29	12q21.32							-0.867	3.17667	6	1
C12orf30	Data not found					-0.346	0.001335	0.0181				1
C12orf34	Data not found					-0.3475	5.00E-05	0.00515				1
C12orf39	chromosome 12 open reading frame 39	12p12.1				0.35	8.00E-04	0.01375				1
C12orf4	In multiple Geneids					0.3235	4.50E-05	0.0049	-0.857	3.17	6	2
C12orf41	Data not found					-0.359	0.00122	0.0193				1
C12orf42	chromosome 12 open reading frame 42	12q23.2							1.039	3.40667	6	1
C12orf43	chromosome 12 open reading frame 43	12q				-0.321	0.00054	0.0133				1
C12orf47	Data not found					-0.434	0.00033	0.0108				1
PLCB1	phospholipase C, beta 1 (phosphoinositide-specific)	20p12	1.513	8.00E-05	0.0018	0.34825	0.01503	0.06582				2
AZGP1	alpha-2-glycoprotein 1, zinc-binding	7q22.1	1.512	0.04949	0.1661							1
C12orf50	chromosome 12 open reading frame 50	12q21.32							-0.964	3.39111	9	1

C12orf51	chromosome 12 open reading frame 51	12q24.13				-0.355222222	0.0013	0.015511111				1
C12orf54	chromosome 12 open reading frame 54	12q13.11	-0.312	0.00086	0.0106							1
C12orf56	chromosome 12 open reading frame 56	12q14.2	-0.964	0.00516	0.0373	-0.306	0.00148	0.0212				2
C12orf60	chromosome 12 open reading frame 60	12p12.3							-0.795	2.625	6	1
C12orf65	chromosome 12 open reading frame 65	12q24.31				-0.367	4.00E-05	0.0047				1
C12orf70	chromosome 12 open reading frame 70	12p11.23				0.303	0.00653	0.0453				1
C12orf72	Data not found					-0.325	0.04591	0.1411				1
COL5A2	collagen, type V, alpha 2	2q14-q32	1.509	0	2.00E-04	0.36975	0.00046	0.01095	-0.777	2.48	5	3
C13orf15	chromosome 13 open reading frame 15					0.354	0.01352	0.0679				1
C13orf18	Data not found								0.804	2.52125	8	1
C13orf25	Data not found								0.603	2.6	5	1
C13orf26	Data not found					0.339	0.00779	0.0499				1
C13orf31	Data not found					0.318	0.00497	0.0391				1
C13orf34	Data not found					0.347	0.00068	0.0148				1
C13orf35	chromosome 13 open reading frame 35	13q34	-0.163	0.04624	0.1595	-0.339	0.02098	0.0877				2
C14orf102	chromosome 14 open reading frame 102	14q32.11				-0.3562	0.001174	0.01728				1
C14orf103	Data not found								-1.02	3.45125	8	1
C14orf105	chromosome 14 open reading frame 105	14q22.3							-0.719	3.04286	7	1
C14orf106	Data not found					-0.322	0.0065	0.0452				1
C14orf109	chromosome 14 open reading frame 109	14q32.12				-0.454	0.00025	0.0096				1
C14orf111	Data not found								-0.966	2.916	5	1
C14orf115	Data not found					-0.3	0.00016	0.008				1
C14orf119	In multiple Geneids					-0.324	0.00263	0.0281				1
C14orf126	In multiple Geneids								-0.619	2.52857	7	1
C14orf132	chromosome 14 open reading frame 132	14q32.2	-0.464	0.00233	0.0213							1
C14orf133	chromosome 14 open reading frame 133	14q24.3-q31				-0.323	0.004606667	0.035566667				1
C14orf135	chromosome 14 open reading frame 135	14q23.1							-0.901	3.52714	7	1
C14orf138	Data not found								-0.841	2.81333	6	1
C14orf143	Data not found					-0.321	3.00E-04	0.0104				1
C14orf145	Data not found					-0.304	0.01509	0.0723				1
C14orf155	Data not found								-1.066	3.82714	7	1
C14orf156	Data not found					-0.318	0.00518	0.04				1
C14orf159	chromosome 14 open reading frame 159	14q32.11	-0.432	0.04721	0.1614	-0.376888889	0.006141111	0.036388889				2
C14orf162	chromosome 14 open reading frame 162	14q24.1				-0.385	0.00179	0.0233				1
C14orf166	chromosome 14 open reading frame 166	14q22.1							-0.714	2.836	5	1
C14orf166B	chromosome 14 open reading frame 166B	14q24.3				-0.304	0.00086	0.0165				1
C14orf174	Data not found					-0.571	0.00019	0.0085				1
C14orf180	chromosome 14 open reading frame 180	14q32.33	-0.281	0.00195	0.0188	-0.339	0.00284	0.0292				2
C14orf43	chromosome 14 open reading frame 43	14q24.3				-0.372	0.004336667	0.0298				1
C14orf45	In multiple Geneids					-0.3605	0.00762	0.0456				1
C14orf48	Data not found					-0.352	0.0009625	0.01495				1
C14orf49	Data not found					-0.335	3.00E-05	0.0043				1
C14orf50	Data not found					-0.337	0.02132	0.0885				1
C14orf79	chromosome 14 open reading frame 79	14q32.33				-0.3905	0.000135	0.0074				1
C14orf8	Data not found								0.75	2.715	6	1
C14orf83	chromosome 14 open reading frame 83	14q23.3				-0.336333333	0.000393333	0.0109				1
C15orf15	Data not found								-0.896	2.818	5	1
C15orf21	Data not found								-0.782	2.76857	7	1
C15orf27	chromosome 15 open reading frame 27	15q24.2				-0.399	0.00531	0.0405				1
C15orf41	chromosome 15 open reading frame 41	15q14	-1.058	0.00059	0.0081							1
C15orf52	chromosome 15 open reading frame 52	15q15.1	-0.422	0.00219	0.0204							1
C15orf53	chromosome 15 open reading frame 53	15q14	-0.231	0.03758	0.1399							1
CTSK	cathepsin K	1q21	1.508	0.00169	0.0169							1
C15orf59	chromosome 15 open reading frame 59	15q24.1	-0.828	2.00E-05	5.00E-04							1
C15orf62	chromosome 15 open reading frame 62	15q15.1	-1.566	0	0							1
C16orf11	In multiple Geneids		-0.338	0.00555	0.0392							1
C16orf14	chromosome 16 open reading frame 14	16p13.3				-0.483	0.00114	0.0187				1
C16orf35	chromosome 16 open reading frame 35	16p13.3				-0.363666667	0.009746667	0.0564				1
C16orf38	chromosome 16 open reading frame 38	16p13.3				-0.582	0.00135	0.0203				1
C16orf45	chromosome 16 open reading frame 45	16p13.11				-0.32225	0.0015175	0.0199				1
C16orf46	chromosome 16 open reading frame 46	16q23.2				-0.34575	0.0017025	0.02185				1
C16orf5	chromosome 16 open reading frame 5	16p13.3				-0.459	0.012456667	0.057033333				1
PTGS2	prostaglandin-endoperoxide synthase 2 (prostaglandin G/H synthase and cycl	1q25.2-q25.3	1.506	0.00033	0.0053				-0.884	2.706	5	2
C16orf53	chromosome 16 open reading frame 53	16p11.2				-0.316	5.00E-05	0.0051				1
C16orf55	chromosome 16 open reading frame 55	16q24.3				-0.352	0.015025	0.0698				1
C16orf57	chromosome 16 open reading frame 57	16q21				-0.348	0.00016	0.0079				1
FCGR2A	Fc fragment of IgG, low affinity IIa, receptor (CD32)	1q23	1.502	0.00014	0.0029							1
C16orf60	Data not found								-0.819	2.634	5	1
C16orf61	Data not found								-0.819	2.634	5	1

C16orf62	chromosome 16 open reading frame 62	16p12.3	-0.722	0.01292	0.0695	-0.42	0.00014	0.0074				2
C16orf63	Data not found					-0.373	0.00327	0.0314				1
C16orf70	chromosome 16 open reading frame 70	16q22.1	-0.372	0.01929	0.0906	-0.372666667	0.004096667	0.0349				2
C16orf71	chromosome 16 open reading frame 71	16p13.3				-0.453333333	0.007623333	0.048466667				1
C16orf72	chromosome 16 open reading frame 72	16p13.2	-0.38	0.00378	0.0301							1
C16orf73	chromosome 16 open reading frame 73	16p13.3				-0.307	0.01584	0.0744				1
C16orf74	chromosome 16 open reading frame 74	16q24.1	-0.361	0.03543	0.1344	-0.3345	0.00321	0.03035				2
C16orf78	chromosome 16 open reading frame 78	16q12.1	-0.256	0.00469	0.0349							1
C16orf80	chromosome 16 open reading frame 80	16q21				-0.311	9.00E-05	0.0064				1
C16orf81	Data not found					-0.46	0.00408	0.0352				1
C16orf87	chromosome 16 open reading frame 87	16q11.2				-0.329	0.00136	0.0203				1
C16orf88	chromosome 16 open reading frame 88	16p12.3				-0.325	0.001765	0.02295				1
C16orf89	chromosome 16 open reading frame 89	16p13.3				-0.494	0.00202	0.02465				1
C16orf94	Data not found					-0.307	2.00E-05	0.0038				1
C17orf103	chromosome 17 open reading frame 103	17p11.2				-0.361	0.003485	0.0323				1
C17orf27	chromosome 17 open reading frame 27								0.796	2.704	5	1
C17orf28	In multiple Geneids					-0.337	0.00535	0.03975				1
C17orf39	chromosome 17 open reading frame 39	17p11.2				-0.367	0.00303	0.03005				1
C17orf45	Data not found					-0.392125	0.00247375	0.0245625				1
C17orf47	chromosome 17 open reading frame 47	17q22	-0.259	0.01315	0.0701							1
C17orf48	chromosome 17 open reading frame 48	17p13.1							-0.379	2.556	5	1
C17orf49	chromosome 17 open reading frame 49	17p13.1				-0.39	8.00E-05	0.0062				1
C17orf53	chromosome 17 open reading frame 53	17q21.31				-0.337	0.00819	0.0513				1
C17orf57	chromosome 17 open reading frame 57	17q21.32				0.322	0.00028	0.01				1
C17orf59	chromosome 17 open reading frame 59	17p13.1	-0.368	0.00343	0.0281	-0.409	0.00148	0.0212				2
C17orf60	Data not found					-0.352	0.02646	0.1007				1
C17orf61	chromosome 17 open reading frame 61	17p13.1				-0.518	0.00012	0.0072				1
CADPS2	Ca++-dependent secretion activator 2	7q31.3	1.501	0	2.00E-04	0.3205	0.00151	0.01935				2
C17orf64	chromosome 17 open reading frame 64	17q23.2							0.75	2.942	5	1
C17orf66	chromosome 17 open reading frame 66	17q12				-0.322	0.00038	0.0115				1
C17orf68	Data not found								-0.895	2.616	5	1
C17orf71	Data not found					-0.4025	0.00242	0.02285				1
C17orf72	chromosome 17 open reading frame 72	17q23.3				-0.314	0.02961	0.1077				1
C17orf74	chromosome 17 open reading frame 74	17p13.1							0.542	2.634	5	1
C17orf76	Data not found					-0.400111111	0.002203333	0.022366667				1
C17orf77	chromosome 17 open reading frame 77	17q25.1							-0.645	3.082	5	1
C17orf79	chromosome 17 open reading frame 79	17q11.2				-0.357	0.006445	0.0418				1
C17orf81	chromosome 17 open reading frame 81	17p13.1				-0.356	0.00119	0.0168				1
C17orf85	chromosome 17 open reading frame 85	17p13.2				-0.354	0.003485	0.031825				1
C17orf87	Data not found					-0.361142857	0.007898571	0.0476				1
C17orf91	Data not found					-0.399	0.02749	0.103				1
C17orf97	chromosome 17 open reading frame 97	17p13.3	-0.446	0.00815	0.0508							1
C17orf99	chromosome 17 open reading frame 99	17q25.3	-0.21	0.04588	0.1587							1
C18orf12	chromosome 18 open reading frame 12	18q21.1	-0.181	0.03713	0.1388							1
C18orf16	Data not found								-0.919	2.62583	6	1
C18orf20	Data not found								-0.783	3.832	5	1
C18orf22	Data not found					-0.387	0.01061	0.0592				1
C18orf25	chromosome 18 open reading frame 25	18q21.1	-1.869	0	0	-0.345	0.02664	0.098228571				2
C18orf26	chromosome 18 open reading frame 26	18q21.2							-0.949	3.01	5	1
FAM105A	family with sequence similarity 105, member A	5p15.2	1.501	5.00E-05	0.0014	0.375	0.00232	0.02605				2
ADAMTS9	ADAM metallopeptidase with thrombospondin type 1 motif, 9	3p14.1	1.496	9.00E-05	0.0021							1
C19orf18	chromosome 19 open reading frame 18	19q13.43				-0.316	0.00258	0.0279				1
C19orf22	Data not found					-0.468	0.00154	0.0216				1
C19orf25	chromosome 19 open reading frame 25	19p13.3				-0.319	0.03591	0.1212				1
C19orf26	chromosome 19 open reading frame 26	19p13.3							-0.606	2.5	6	1
C19orf28	Data not found					-0.388	0.0071	0.0421				1
C19orf29	chromosome 19 open reading frame 29	19p13.3				-0.435666667	0.00218	0.024833333				1
C19orf30	Data not found					-0.479	0.00261	0.028				1
C19orf38	chromosome 19 open reading frame 38	19p13.2				-0.48975	0.0026175	0.02115				1
C19orf39	Data not found					-0.342	0.00511	0.0397				1
C19orf40	chromosome 19 open reading frame 40	19q13.11				-0.301	0.00831	0.0517				1
C19orf41	Data not found					-0.333	0.0054	0.0408				1
C19orf42	chromosome 19 open reading frame 42	19p13.11				-0.44925	0.0041325	0.029125				1
C19orf43	chromosome 19 open reading frame 43	19p13.2				-0.5	0.00056	0.0135				1
C19orf44	chromosome 19 open reading frame 44	19p13.11				-0.4515	0.00599	0.04125				1
C19orf45	chromosome 19 open reading frame 45	19p13.2				-0.4045	0.001945	0.02365				1
C19orf47	chromosome 19 open reading frame 47	19q13.2				-0.343	0.00487	0.03615				1
C19orf48	chromosome 19 open reading frame 48	19q13.33				-0.353	0.00122	0.0193				1
C19orf50	Data not found					-0.469	0.000633333	0.013433333				1

SYCP2	synaptonemal complex protein 2	20q13.33	1.494	0.01036	0.06	0.355333333	0.004975556	0.036544444				2
C19orf55	chromosome 19 open reading frame 55	19q13.12				-0.302	7.00E-04	0.015				1
C19orf57	chromosome 19 open reading frame 57	19p13.12	-0.239	0.01137	0.0638	-0.3964	0.013848	0.06222				2
C19orf59	chromosome 19 open reading frame 59	19p13.2				-0.508	0.00057	0.0137				1
C19orf6	chromosome 19 open reading frame 6	19p13.3				-0.57	0.00055	0.0134				1
C19orf60	chromosome 19 open reading frame 60	19p13.11				-0.52	0.00012	0.007				1
C19orf62	Data not found					-0.449	0.00107	0.0182				1
C19orf63	Data not found					-0.3775	0.005575	0.0339				1
C19orf71	chromosome 19 open reading frame 71	19p13.3				-0.421	0.01327	0.0672				1
NOSTRIN	nitric oxide synthase trafficker	2q31.1	1.493	0.00198	0.0189	0.32	7.00E-05	0.0057	-1.019	3.56	7	3
C1D	C1D nuclear receptor corepressor	2p13-p12				0.356	9.00E-05	0.0062				1
C1GALT1	core 1 synthase, glycoprotein-N-acetylgalactosamine 3-beta-galactosyltransfe	7p21.3				0.325666667	0.000283333	0.009333333				1
VAV2	vav 2 guanine nucleotide exchange factor	9q34.1	1.493	0	0							1
C1orf105	chromosome 1 open reading frame 105	1q24.3				0.337333333	0.001293333	0.01935	-0.808	2.945	8	2
FAM134B	family with sequence similarity 134, member B	5p15.1	1.492	0.00143	0.0151	0.360125	0.00450875	0.034925				2
C1orf107	Data not found					0.32375	0.002355	0.01905				1
LOC440905	uncharacterized LOC440905	2q21.1	1.492	0.00461	0.0346							1
C1orf110	chromosome 1 open reading frame 110	1q23.3				0.334	0.00062	0.0142				1
C1orf112	chromosome 1 open reading frame 112	1q24.2				0.317	0.00027	0.01				1
C1orf114	chromosome 1 open reading frame 114	1q24				0.3436	0.005892	0.03726	-0.894	3.22429	7	2
PIK3AP1	phosphoinositide-3-kinase adaptor protein 1	10q24.1	1.491	1.00E-05	3.00E-04							1
C1orf116	chromosome 1 open reading frame 116	1q32.1				0.301	0.00127	0.0197				1
C1orf117	Data not found								1.178	2.52	5	1
C1orf124	chromosome 1 open reading frame 124	1q42.12-q43							-0.691	2.42286	7	1
C1orf126	chromosome 1 open reading frame 126	1p36.21				-0.366	0.00514	0.0392				1
C1orf127	chromosome 1 open reading frame 127	1p36.22				-0.479	0.00031	0.00965				1
C1orf129	chromosome 1 open reading frame 129	1q24.3				0.378333333	0.001481667	0.018933333	-0.318	2.45	7	2
C1orf130	chromosome 1 open reading frame 130	1p36.11				-0.394555556	0.007245556	0.032688889				1
FAP	fibroblast activation protein, alpha	2q23	1.486	0.00126	0.0138	0.347375	0.001854375	0.01708125	-0.898	3.63	8	3
C1orf135	chromosome 1 open reading frame 135	1p36.11				-0.3345	0.012855	0.0658				1
C1orf140	uncharacterized LOC400804	1q41							-0.951	2.80833	6	1
C1orf144	chromosome 1 open reading frame 144	1p36.13				-0.4124	0.001864	0.0213				1
C1orf145	chromosome 1 open reading frame 145	1q42.13							0.689	2.59563	8	1
C1orf146	chromosome 1 open reading frame 146	1p22.1	-0.205	0.04645	0.1598							1
C1orf151	Data not found					-0.355666667	0.00244	0.024366667				1
C1orf159	chromosome 1 open reading frame 159	1p36.33				-0.423	0.00029	0.009	0.752	2.649	5	2
C1orf163	Data not found								-0.788	2.49333	6	1
C1orf170	chromosome 1 open reading frame 170	1p36.33	-0.335	0.00146	0.0153							1
C1orf172	chromosome 1 open reading frame 172	1p36.11	-0.351	0.01431	0.0742	-0.438666667	0.007543333	0.0389				2
C1orf175	Data not found					-0.3872	0.00443	0.03114				1
C1orf177	chromosome 1 open reading frame 177	1p32.3	-2.265	0	0							1
C1orf178	Data not found								-0.702	3.26	5	1
C1orf180	chromosome 1 open reading frame 180	1p22.3							-0.704	2.744	5	1
C1orf187	chromosome 1 open reading frame 187	1p36.22				-0.39	0.00251	0.0275				1
C1orf192	chromosome 1 open reading frame 192	1q23.3							-0.904	2.874	5	1
FCER1G	Fc fragment of IgE, high affinity I, receptor for; gamma polypeptide	1q23	1.486	0.00083	0.0103							1
C1orf2	Data not found								0.702	2.492	5	1
C1orf200	chromosome 1 open reading frame 200	1p36.22	-0.2	0.03307	0.1284	-0.532	2.00E-05	0.004				2
C1orf201	chromosome 1 open reading frame 201	1p36.11				-0.333333333	0.003616667	0.032966667				1
C1orf21	chromosome 1 open reading frame 21	1q25				0.308	0.00034	0.011				1
C1orf213	chromosome 1 open reading frame 213	1p36.12				-0.356	0.00028	0.01				1
C1orf228	chromosome 1 open reading frame 228	1p34.1				-0.3985	0.003715	0.029775				1
BAAT	bile acid CoA: amino acid N-acyltransferase (glycine N-choloyltransferase)	9q22.3	1.483	0.01621	0.0806							1
C1orf35	chromosome 1 open reading frame 35	1q42.13	-0.209	0.04624	0.1595							1
C1orf38	chromosome 1 open reading frame 38	1p35.3				-0.469333333	0.0054	0.038				1
C1orf42	Data not found								-0.798	2.729	5	1
CST2	cystatin SA	20p11.21	1.483	0.00144	0.0152							1
C1orf53	chromosome 1 open reading frame 53	1q31.3				0.3255	0.000975	0.01555				1
SUSD1	sushi domain containing 1	9q31.3-q33.1	1.482	0	1.00E-04							1
C1orf57	Data not found					0.32	0.00045	0.0123				1
C1orf62	Data not found					-0.335	0.00293	0.0297				1
C1orf64	chromosome 1 open reading frame 64	1p36.13	-0.212	0.03157	0.1248							1
C1orf70	Data not found					-0.408	0.00024	0.0095				1
C1orf74	chromosome 1 open reading frame 74	1q32.2	-0.42	0.04768	0.1624							1
C1orf83	Data not found					-0.358	0.00265	0.0282				1
C1orf84	chromosome 1 open reading frame 84	1p34.2							-0.809	2.578	5	1
GOLIM4	golgi integral membrane protein 4	3q26.2	1.481	0	2.00E-04	0.350636364	0.001834545	0.020690909				2
C1orf88	chromosome 1 open reading frame 88	1p13.2				-0.337	0.00184	0.0236				1
C1orf9	In multiple Geneids					0.351823529	0.002152353	0.021258824	-0.92	3.48667	6	2

C1orf91	Data not found					-0.349	0.00291	0.0295				1
C1orf94	chromosome 1 open reading frame 94	1p34.3							0.91	3.02937	8	1
C1orf99	Data not found								-0.951	2.82667	6	1
NID1	nidogen 1	1q43	1.48	0.00027	0.0045							1
FABP3	fatty acid binding protein 3, muscle and heart (mammary-derived growth inhil	1p33-p32	1.477	0.0226	0.1003							1
C1QBP	complement component 1, q subcomponent binding protein	17p13.3				-0.311	0.00308	0.0304				1
HIBADH	3-hydroxyisobutyrate dehydrogenase	7p15.2	1.476	1.00E-05	3.00E-04	0.309	0.00137	0.0195				2
C1QL4	complement component 1, q subcomponent-like 4	12q13.12	-0.263	0.0289	0.1176	-0.371	0.00401	0.0349	0.951	2.81714	7	3
PDGFRB	platelet-derived growth factor receptor, beta polypeptide	5q33.1	1.475	6.00E-05	0.0015							1
C1QTNF2	C1q and tumor necrosis factor related protein 2	5q33.3				-0.306	0.00045	0.0124				1
C1QTNF3	C1q and tumor necrosis factor related protein 3	5p13				0.375666667	0.01348	0.066933333				1
CMTM7	CKLF-like MARVEL transmembrane domain containing 7	3p22.3	1.474	0.00011	0.0024							1
ANG	angiogenin, ribonuclease, RNase A family, 5	14q11.1-q11.2	1.473	0.00069	0.009				-0.792	2.892	5	2
C1QTNF8	C1q and tumor necrosis factor related protein 8	16p13.3	-0.256	0.0113	0.0636							1
C1QTNF9B-AS1	C1QTNF9B antisense RNA 1 (non-protein coding)	13q12	-0.594	0.02081	0.0952							1
GPR35	G protein-coupled receptor 35	2q37.3	1.473	0	0							1
FRZB	frizzled-related protein	2qter	1.471	4.00E-05	0.0012							1
RAB15	In multiple Geneids		1.471	0.00025	0.0043							1
THBS2	thrombospondin 2	6q27	1.471	0.00756	0.0483							1
CDC25B	cell division cycle 25 homolog B (S. pombe)	20p13	1.467	9.00E-05	0.0021							1
C20orf141	chromosome 20 open reading frame 141	20p13	-0.305	0.0058	0.0404							1
C20orf151	chromosome 20 open reading frame 151	20q13.33							1.112	3.75556	9	1
C20orf165	Data not found								0.773	2.39	5	1
C20orf173	chromosome 20 open reading frame 173	20q11.22	-0.285	0.00671	0.0446							1
C20orf177	Data not found					0.3885	0.00356	0.0324				1
C20orf194	chromosome 20 open reading frame 194	20p13	-0.651	0.01501	0.0766							1
C20orf197	chromosome 20 open reading frame 197	20q13.33				0.401	0.00423	0.0332				1
C20orf24	chromosome 20 open reading frame 24	20q11.23	-0.479	0.04846	0.164							1
C20orf26	chromosome 20 open reading frame 26	20p11.23				0.37	0.006518571	0.040685714				1
C20orf3	chromosome 20 open reading frame 3	20p11.2				0.306	0.00299	0.0299	-0.922	2.98333	6	2
C20orf39	Data not found					0.302	0.003005	0.02985				1
C20orf43	chromosome 20 open reading frame 43	20q13.31				0.3125	0.006575	0.0453				1
C20orf55	Data not found								0.683	3.084	5	1
C20orf56	Data not found					0.373	0.005665	0.0419				1
C20orf74	chromosome 20 open reading frame 74					0.373692308	0.005953077	0.0402				1
C20orf94	In multiple Geneids					0.371888889	0.009402222	0.047677778				1
GIPC2	GIPC PDZ domain containing family, member 2	1p31.1	1.467	0	0							1
C21orf109	Data not found					0.462	1.00E-05	0.0029				1
C21orf122	chromosome 21 open reading frame 122	21q22.3							0.819	2.555	6	1
C21orf128	chromosome 21 open reading frame 128	21q22.3	-0.318	0.0023	0.0212							1
C21orf129	Data not found					-0.329	0.00022	0.009				1
C21orf15	chromosome 21 open reading frame 15	21q11	-4.806	0	0							1
C21orf2	chromosome 21 open reading frame 2	21q22.3	-0.244	0.02493	0.1069	-0.4045	0.0006175	0.01195				2
C21orf29	Data not found					-0.3958125	0.001215	0.0143				1
C21orf33	In multiple Geneids		-0.444	0.00531	0.0381							1
C21orf34	chromosome 21 open reading frame 34	21q11							-0.8945	3.18604	8.5	1
C21orf45	Data not found					-0.329	0.00024	0.0094				1
C21orf54	In multiple Geneids					-0.329	0.00014	0.0075				1
C21orf56	In multiple Geneids					-0.339	0.0126	0.0652				1
C21orf57	Data not found					-0.324	0.00498	0.0391				1
C21orf58	chromosome 21 open reading frame 58	21q22.3				-0.404	0.00146	0.0211				1
C21orf59	chromosome 21 open reading frame 59	21q22.1				-0.326	0.00017	0.0081				1
C21orf62	chromosome 21 open reading frame 62	21q22.11							-0.621	2.30667	6	1
C21orf63	Data not found					-0.3700625	0.0022025	0.01954375				1
C21orf67	chromosome 21 open reading frame 67	21q22.3	-0.2	0.03941	0.1441							1
C21orf7	chromosome 21 open reading frame 7	21q22.3							-0.7525	2.81457	6	1
C21orf70	Data not found					-0.358	0.0026	0.02195				1
C21orf81	chromosome 21 open reading frame 81	21q11.2							0.742	2.666	5	1
C21orf84	Data not found					-0.337	0.01029	0.0471				1
C21orf88	chromosome 21 open reading frame 88	21q22.2				-0.3465	0.002585	0.0277				1
CHN2	chimerin (chimaerin) 2	7p15.3	1.466	1.00E-05	5.00E-04	0.327	0.00079	0.0159				2
C21orf91	chromosome 21 open reading frame 91	21q21.1	-0.76	3.00E-05	9.00E-04							1
C22orf13	In multiple Geneids					-0.422666667	0.00022	0.0085				1
C22orf15	chromosome 22 open reading frame 15	22q11.23	-0.224	0.0111	0.0627	-0.587	7.00E-05	0.0059				2
C22orf23	chromosome 22 open reading frame 23	22q13.1	-0.224	0.01623	0.0806	-0.409	0.00125	0.019				2
C22orf24	chromosome 22 open reading frame 24	22q12.1-q12.3				-0.399	0.003765714	0.0307				1
C22orf25	chromosome 22 open reading frame 25	22q11.21				-0.5216	0.000334	0.00868				1
C22orf26	chromosome 22 open reading frame 26	22q13.31				-0.3485	0.00049	0.0118				1
C22orf27	Data not found					-0.391	0.00276	0.0288				1

C22orf28	chromosome 22 open reading frame 28	22q12				-0.358	0.000705	0.012825				1
C22orf30	Data not found					-0.4375	0.001106429	0.013892857				1
C22orf31	chromosome 22 open reading frame 31	22q12.1	-0.239	0.01618	0.0805	-0.306	0.00046	0.0124				2
C22orf33	Data not found					-0.419428571	0.000392857	0.009757143				1
C22orf34	chromosome 22 open reading frame 34	22q13.33				-0.3846	0.000936	0.01102				1
C22orf36	Data not found					-0.44	5.00E-05	0.0052				1
C22orf40	chromosome 22 open reading frame 40	22q13.31				-0.4505	0.00022	0.00895				1
C22orf41	Data not found					-0.446	0	0.0012				1
C22orf42	chromosome 22 open reading frame 42	22q12.3	-0.843	0	2.00E-04	-0.395	0.000155	0.0064				2
C22orf43	chromosome 22 open reading frame 43	22q11.2				-0.397	0.000655	0.01445				1
C22orf9	chromosome 22 open reading frame 9					-0.4467	0.006522	0.02921				1
C2CD2	C2 calcium-dependent domain containing 2	21q22.3	-0.394	0.01398	0.0731	-0.397818182	0.00177	0.019481818				2
LAMB1	laminin, beta 1	7q22	1.466	0	1.00E-04							1
C2CD4C	C2 calcium-dependent domain containing 4C	19p13.3	-0.255	0.02174	0.0981							1
FABP1	fatty acid binding protein 1, liver	2p11	1.463	0.02264	0.1003							1
DACH1	dachshund homolog 1 (Drosophila)	13q22	1.462	0.00357	0.0289	0.334117647	0.004716471	0.034705882				2
C2orf16	chromosome 2 open reading frame 16	2p23.3							-0.762	2.94143	7	1
C2orf21	Data not found					0.3325	1.00E-05	0.003				1
C2orf3	Data not found					0.4115	0.00019	0.00725				1
C2orf30	Data not found					-0.462	5.00E-05	0.0053				1
C2orf32	Data not found								0.779	2.928	5	1
C2orf34	Data not found					0.3375	0.0009725	0.013475				1
IHH	Indian hedgehog	2q33-q35	1.462	0.00018	0.0035							1
C2orf53	chromosome 2 open reading frame 53	2p23.3	-0.301	0.00541	0.0386							1
C2orf54	chromosome 2 open reading frame 54	2q37.3	-2.384	0	1.00E-04							1
C2orf57	chromosome 2 open reading frame 57	2q37.1	-0.315	0.00803	0.0503							1
C2orf62	chromosome 2 open reading frame 62	2q35	-0.28	0.00321	0.0269							1
C2orf71	chromosome 2 open reading frame 71	2p23.2	-0.215	0.01652	0.0816							1
C2orf78	chromosome 2 open reading frame 78	2p13.1				-0.396	3.00E-05	0.0042				1
C2orf82	chromosome 2 open reading frame 82	2q37.1	-0.208	0.02607	0.1103							1
C2orf83	chromosome 2 open reading frame 83	2q36.3	-0.212	0.02658	0.1117							1
C2orf86	Data not found					0.402666667	0.000293333	0.008533333				1
SH3KBP1	In multiple Geneids		1.462	1.00E-04	0.0023							1
MINPP1	multiple inositol-polyphosphate phosphatase 1	10q23	1.46	7.00E-05	0.0017							1
CERS2	ceramide synthase 2	1q21.3	1.459	1.00E-05	5.00E-04							1
C3orf10	Data not found					-0.355	0.00167	0.0225				1
C3orf14	chromosome 3 open reading frame 14	3p14.2	-1.268	0.00042	0.0063							1
MYRF	Data not found		1.458	0.00085	0.0104							1
C3orf20	chromosome 3 open reading frame 20	3p25.1				-0.339	0.00084	0.0163				1
C3orf22	chromosome 3 open reading frame 22	3q21.3	-0.258	0.02931	0.1187							1
C3orf23	chromosome 3 open reading frame 23	3p21.31				1.976	0	4.00E-04				1
C3orf24	chromosome 3 open reading frame 24	3p25.3				-0.343	0.00454	0.030666667				1
C3orf31	Data not found								-0.679	2.54333	6	1
C3orf32	chromosome 3 open reading frame 32	3p26.1							-0.969	3.884	5	1
GLS	glutaminase	2q32-q34	1.454	5.00E-04	0.0072							1
C3orf35	chromosome 3 open reading frame 35	3p22.2				-0.306	0.0018	0.0233				1
C3orf39	Data not found								-0.787	2.9	7	1
C3orf51	Data not found					-0.312	0.03712	0.1237				1
PDE3A	phosphodiesterase 3A, cGMP-inhibited	12p12	1.454	5.00E-04	0.0071	0.3499375	0.00256	0.0191625				2
GRB10	growth factor receptor-bound protein 10	7p12.2	1.453	0	0	0.3683	0.0124245	0.05861				2
C3orf57	Data not found					0.3725	0.000975	0.0164				1
C3orf58	chromosome 3 open reading frame 58	3q24				0.315	0.00064	0.0143				1
C3orf59	Data not found								-0.808	2.846	5	1
C3orf60	Data not found								-0.599	2.332	5	1
C3orf62	chromosome 3 open reading frame 62	3p21.31	-0.508	0.01381	0.0725							1
C3orf63	Data not found					-0.359	0.00191	0.02225				1
C3orf64	Data not found								-0.667	2.496	5	1
C3orf67	chromosome 3 open reading frame 67	3p14.2	-1.652	0.00015	0.0029							1
C3orf74	chromosome 3 open reading frame 74	3p21.2				-0.465	2.00E-05	0.0036				1
C3orf75	chromosome 3 open reading frame 75	3p21.31				-0.3976	0.002838	0.02146				1
C3orf80	chromosome 3 open reading frame 80	3q25.33	-0.84	8.00E-05	0.0018							1
C3P1	complement component 3 precursor pseudogene	19p13.2				-0.4595	0.004982857	0.034042857				1
NR4A1	nuclear receptor subfamily 4, group A, member 1	12q13	1.453	0.00138	0.0146							1
C4B	In multiple Geneids								0.901	3.616	5	1
PDE3B	phosphodiesterase 3B, cGMP-inhibited	11p15.1	1.45	0	1.00E-04							1
GPC6	glypican 6	13q32	1.442	0.00172	0.0171	0.3462	0.009114364	0.050389091				2
C4orf10	Data not found					-0.40525	0.003955	0.025225				1
C4orf11	chromosome 4 open reading frame 11	4q21.3				-0.342666667	0.00094	0.015633333				1
C4orf12	Data not found					-0.333	0.00287	0.0293				1

C4orf17	chromosome 4 open reading frame 17	4q23							-0.721	2.81714	7	1
C4orf18	Data not found								-0.828	3.10875	8	1
OLFML2B	olfactomedin-like 2B	1q23.3	1.44	2.00E-05	7.00E-04							1
C4orf21	chromosome 4 open reading frame 21	4q25							-0.806	3.09857	7	1
C4orf22	chromosome 4 open reading frame 22	4q21.21	-0.228	0.01357	0.0716							1
C4orf23	Data not found					-0.4135	0.000105	0.0067				1
C4orf27	chromosome 4 open reading frame 27	4q33				-0.434	0.00029	0.0101				1
C4orf29	chromosome 4 open reading frame 29	4q28.2				-0.412	0.00073	0.01325				1
C4orf3	chromosome 4 open reading frame 3	4q26	-1.564	1.00E-05	5.00E-04							1
C4orf33	chromosome 4 open reading frame 33	4q28.2	-0.49	0.0475	0.162							1
C4orf34	chromosome 4 open reading frame 34	4p14				-0.375	0.012468	0.06274				1
C4orf36	chromosome 4 open reading frame 36	4q21.3	-0.547	0.00056	0.0078							1
C4orf37	chromosome 4 open reading frame 37	4q22.3-q23				-0.3366	0.00282	0.02574				1
C4orf38	Data not found					-0.359	0.00468	0.0378				1
C4orf39	Data not found					-0.319	0.00663	0.0457				1
C4orf40	chromosome 4 open reading frame 40	4q13.3	-0.218	0.0075	0.0481							1
C4orf41	Data not found					-0.419909091	0.006615455	0.038127273				1
C4orf44	Data not found					-0.403666667	0.0049	0.034233333				1
C4orf45	chromosome 4 open reading frame 45	4q32.1				-0.3635	0.010865	0.051975				1
C4orf48	chromosome 4 open reading frame 48	4p16.3				-0.516	0.00072	0.0152				1
C4orf49	Data not found					-0.367	0.01174	0.0626				1
C4orf6	chromosome 4 open reading frame 6	4p16.2							-0.819	3.08857	7	1
C4orf8	Data not found					-0.3975	0.00400625	0.030725				1
C5	complement component 5	9q33-q34				0.405	0.00043	0.012	-0.935	3.40714	7	2
AQP9	aquaporin 9	15q	1.437	0.00016	0.0031				-0.789	2.715	6	2
LPCAT1	lysophosphatidylcholine acyltransferase 1	5p15.33	1.436	0	0	0.37	0.01685	0.0771				2
C5orf20	chromosome 5 open reading frame 20	5q31.1	-0.374	0.00158	0.0162							1
C5orf21	Data not found								-0.873	3.40875	8	1
LRRC66	leucine rich repeat containing 66	4q12	1.436	0.00423	0.0326							1
C5orf23	chromosome 5 open reading frame 23	5p13.3							-0.811	2.85571	7	1
C5orf24	chromosome 5 open reading frame 24	5q31.1				-0.409	0.00039	0.0116				1
C5orf25	chromosome 5 open reading frame 25	5q35.2				-0.36125	0.00231	0.0227875				1
C5orf27	chromosome 5 open reading frame 27	5q15				-0.422	0.00322	0.0311				1
MLXIPL	MLX interacting protein-like	7q11.23	1.436	0.0022	0.0204							1
HRASLS2	HRAS-like suppressor 2	11q12.3	1.434	0.00141	0.0149							1
C5orf32	Data not found					-0.351666667	0.000266667	0.008433333				1
C5orf33	Data not found					0.424625	0.004805	0.0370625				1
FAM102B	family with sequence similarity 102, member B	1p13.3	1.433	0	1.00E-04							1
C5orf35	Data not found					-0.332	0.00016	0.0079				1
C5orf38	chromosome 5 open reading frame 38	5p15.33				0.354	0.00638	0.0447				1
C5orf4	chromosome 5 open reading frame 4	5q31-q32							-0.877	3.112	5	1
C5orf41	Data not found					-0.355666667	0.002573333	0.023355556				1
C5orf42	chromosome 5 open reading frame 42	5p13.2				0.336	0.02489	0.0971				1
C5orf45	chromosome 5 open reading frame 45	5q35.3				-0.4405	0.0014425	0.0174				1
C5orf47	chromosome 5 open reading frame 47	5q35.2	-0.221	0.01619	0.0805							1
C5orf48	chromosome 5 open reading frame 48	5q23.2				-0.425	8.00E-04	0.0145				1
C5orf49	chromosome 5 open reading frame 49	5p15.31				0.356	0.00216	0.0255				1
C5orf51	chromosome 5 open reading frame 51	5p13.1				0.45375	0.009705	0.05135				1
C5orf52	chromosome 5 open reading frame 52	5q33.3				-0.315	0.00715	0.0476				1
C5orf56	chromosome 5 open reading frame 56	5q31.1				-0.327	0.00329	0.0315				1
C6	complement component 6	5p13				0.463916667	0.009289583	0.050095833	-0.93	3.38714	7	2
C6orf10	chromosome 6 open reading frame 10	6p21.3				0.339	0.0302	0.109				1
C6orf103	Data not found					0.365	0.01111	0.05655				1
C6orf108	chromosome 6 open reading frame 108	6p21.1							0.986	2.58	5	1
C6orf111	Data not found								-0.856	3.17429	7	1
C6orf118	In multiple Geneids		-0.2	0.00918	0.0551							1
C6orf120	chromosome 6 open reading frame 120	6q27							-0.556	2.428	5	1
C6orf122	chromosome 6 open reading frame 122					-0.404	0.00029	0.0103				1
GRAMD1B	GRAM domain containing 1B	11q24.1	1.433	0.00463	0.0347							1
C6orf124	chromosome 6 open reading frame 124	6q27				-0.3615	0.011905	0.0616				1
C6orf132	In multiple Geneids		-1.5145	5.00E-06	3.00E-04							1
C6orf138	chromosome 6 open reading frame 138	6p12.3				0.359	0.0048075	0.03408125				1
C6orf139	Data not found								-0.851	3.002	5	1
C6orf142	Data not found					0.364	0.013836	0.06274	-1.03	3.37333	6	2
C6orf146	Data not found					-0.301	0.00631	0.0445				1
C6orf147	chromosome 6 open reading frame 147	6q13				-0.36525	0.00193	0.02255				1
C6orf150	Data not found					-0.371	0.00303	0.0302				1
C6orf165	chromosome 6 open reading frame 165	6q15							-0.848	2.74714	7	1
C6orf168	Data not found					-0.32	0.00076	0.0155				1

C6orf170	chromosome 6 open reading frame 170	6q22.31				-0.383	0.00169	0.0226				1
C6orf182	chromosome 6 open reading frame 182								-0.782	2.77833	6	1
C6orf188	Data not found								-0.89	2.932	5	1
C6orf190	Data not found								-1.109	3.88	5	1
C6orf201	In multiple Geneids					-0.35	0.02374	0.07555				1
C6orf203	chromosome 6 open reading frame 203	6q21	-0.304	0.04165	0.1494	-0.334666667	0.006686667	0.0411				2
C6orf206	Data not found								0.777	3.12143	7	1
C6orf208	Data not found					-0.404	0.00029	0.0103				1
SLC43A1	solute carrier family 43, member 1	11q12.1	1.43	0.00228	0.021							1
INSR	insulin receptor	19p13.3-p13.2	1.429	0	0							1
C6orf25	chromosome 6 open reading frame 25	6p21.33	-1.0735	0	0.00015							1
NAPEPLD	N-acyl phosphatidylethanolamine phospholipase D	7q22.1	1.429	0.00011	0.0024							1
C6orf49	Data not found								0.568	2.506	5	1
C6orf58	chromosome 6 open reading frame 58	6q22.33				0.409	0.00371	0.0335				1
C6orf64	Data not found					0.333	0.01961	0.0842				1
FLVCR1	feline leukemia virus subgroup C cellular receptor 1	1q32.3	1.427	0.00017	0.0033							1
C6orf97	Data not found					-0.627	2.00E-05	0.0039				1
C7	complement component 7	5p13				0.394142857	0.018578571	0.0755				1
C7orf10	chromosome 7 open reading frame 10	7p14.1				0.347363636	0.003855455	0.030890909				1
LOC388022	uncharacterized LOC388022	14q32.33	1.427	0.00025	0.0043							1
C7orf16	Data not found					0.354	4.00E-05	0.0049				1
C7orf25	chromosome 7 open reading frame 25	7p14.1							-0.831	2.446	5	1
C7orf26	chromosome 7 open reading frame 26	7p22.1				-0.308	0.04014	0.12985				1
C7orf33	chromosome 7 open reading frame 33	7q36.1	-0.204	0.0392	0.1436	-0.356	0.00366	0.03315				2
C7orf42	Data not found					-0.302	0.01423	0.0699				1
C7orf50	chromosome 7 open reading frame 50	7p22.3				-0.322	0.00123	0.0194				1
C7orf55	chromosome 7 open reading frame 55	7q34				-0.438	0.00182	0.0234				1
C7orf57	chromosome 7 open reading frame 57	7p12.3				0.314	0.02957	0.1076				1
C7orf58	chromosome 7 open reading frame 58	7q31.31				0.3552	0.006142	0.0277				1
C7orf60	chromosome 7 open reading frame 60	7q31.1	-0.447	0.03131	0.1242							1
C7orf62	chromosome 7 open reading frame 62	7q21.13	-0.255	0.04763	0.1623							1
C7orf64	Data not found					0.32	0.01943	0.0838				1
C7orf9	Data not found								-0.851	2.65	5	1
C8A	complement component 8, alpha polypeptide	1p32							-0.989	2.99833	6	1
C8orf12	chromosome 8 open reading frame 12	8p23.1				0.429	0.0454	0.1402				1
SLC16A4	solute carrier family 16, member 4 (monocarboxylic acid transporter 5)	1p13.3	1.425	0.00026	0.0044							1
C8orf34	chromosome 8 open reading frame 34	8q13				0.346777778	0.00294963	0.025874074	-0.947	3.29889	9	2
RNF128	ring finger protein 128, E3 ubiquitin protein ligase	Xq22.3	1.417	0.00018	0.0034				-0.803666667	2.56	5	2
C8orf41	Data not found								-0.698	2.61571	7	1
PCOLCE	procollagen C-endopeptidase enhancer	7q22	1.415	0.00053	0.0074							1
C8orf47	chromosome 8 open reading frame 47	8q22.2							1.035	2.764	5	1
C8orf48	chromosome 8 open reading frame 48	8p22	-0.541	0.00036	0.0056							1
C8orf56	chromosome 8 open reading frame 56	8q22.3	-0.212	0.03639	0.137							1
C8orf58	chromosome 8 open reading frame 58	8p21	-0.337	0.00044	0.0065							1
C8orf61	chromosome 8 open reading frame 61	8p23.3							1.204	3.76143	7	1
C8orf74	In multiple Geneids								0.931	3.084	5	1
C8orf80	Data not found					-0.306	0.00196	0.0243				1
C8orf83	Data not found					0.389444444	0.000197778	0.005777778				1
C8orf86	chromosome 8 open reading frame 86	8p11.22				-0.318	0.01473	0.0714				1
C9	complement component 9	5p14-p12				0.395833333	0.003365	0.029625				1
C9orf100	chromosome 9 open reading frame 100	9p13.3				-0.436	0.00049	0.009625				1
C9orf102	chromosome 9 open reading frame 102	9q22.32							-1.041	3.33	5	1
C9orf11	chromosome 9 open reading frame 11	9p21				-0.318	0.00527	0.0403				1
C9orf111	Data not found								0.959	3.134	5	1
C9orf117	chromosome 9 open reading frame 117	9q34.11				-0.3	0.03372	0.1166				1
C9orf121	Data not found								0.813	3.394	5	1
C9orf123	chromosome 9 open reading frame 123	9p24.1				-0.349	0.00805	0.0508				1
C9orf125	Data not found								-0.762	2.42714	7	1
C9orf128	Data not found					-0.34175	0.00074	0.010475				1
C9orf131	chromosome 9 open reading frame 131	9p13.3				-0.372	1.00E-05	0.0033				1
C9orf135	chromosome 9 open reading frame 135	9q21.12				0.5235	0.000765	0.0134				1
C9orf140	Data not found					-0.408	0.00073	0.0153				1
C9orf150	Data not found								-0.959	2.91571	7	1
C9orf156	chromosome 9 open reading frame 156	9q22.33							-0.875	2.458	5	1
C9orf16	chromosome 9 open reading frame 16	9q34.1				-0.346	0.00673	0.0461				1
C9orf169	chromosome 9 open reading frame 169	9q34.3	-1.4205	5.00E-05	0.00105	-0.357	7.00E-04	0.015				2
C9orf171	chromosome 9 open reading frame 171	9q34.13	-0.308	0.01983	0.0922	-0.331333333	0.01683	0.0722				2
C9orf18	chromosome 9 open reading frame 18								-0.804	2.95667	6	1
C9orf24	chromosome 9 open reading frame 24	9p13.3				-0.397	8.00E-05	0.006				1

C9orf25	Data not found					-0.3595	0.006315	0.0417				1
C9orf26	Data not found								-0.896	2.77	7	1
C9orf27	Data not found								-1.046	3.078	5	1
KRT18	keratin 18	12q13	1.4145	1.00E-05	0.00035							1
C9orf4	Data not found					-0.31	0.00359	0.0329				1
C9orf46	Data not found					-0.311	0.00522	0.0401				1
C9orf48	Data not found								-0.764	2.714	5	1
C9orf5	Data not found					0.307	0.00478	0.0383				1
C9orf57	chromosome 9 open reading frame 57	9q21.13	-0.218	0.0408	0.1474							1
MGP	matrix Gla protein	12p12.3	1.414	0.02096	0.0957				-1.011	3.57	5	2
C9orf66	chromosome 9 open reading frame 66	9p24.3	-0.27	0.00642	0.0432							1
C9orf68	Data not found								-0.756	2.91	8	1
C9orf7	Data not found								-0.728	2.55667	6	1
C9orf72	chromosome 9 open reading frame 72	9p21.2							-0.646	2.49	6	1
FGFR4	fibroblast growth factor receptor 4	5q35.1-qter	1.413	1.00E-05	3.00E-04							1
C9orf82	Data not found					-0.358285714	0.001181429	0.013942857				1
C9orf84	chromosome 9 open reading frame 84	9q31.3							0.56	2.88667	6	1
PTPRK	protein tyrosine phosphatase, receptor type, K	6q22.2-q22.3	1.413	1.00E-05	4.00E-04	0.353615385	0.024023333	0.092153846				2
C9orf9	chromosome 9 open reading frame 9	9q34	-0.501	0.00095	0.0113							1
C9orf93	Data not found					-0.353428571	0.002523333	0.023409524				1
C9orf95	Data not found					-0.316	0.01473	0.0714				1
C9orf97	Data not found								-0.22	2.643	5	1
C9orf98	Data not found					-0.301	0.00037	0.0113				1
CA10	In multiple Geneids					0.334	0.000856	0.01374				1
CA11	carbonic anhydrase XI	19q13.3	-0.295	0.03295	0.1281	-0.403333333	0.00267	0.024133333				2
CA12	carbonic anhydrase XII	15q22	-2.207	4.00E-05	0.0012							1
CA5A	carbonic anhydrase VA, mitochondrial	16q24.3				-0.3445	0.015155	0.06785	0.705	2.79	5	2
CA6	carbonic anhydrase VI	1p36.2				-0.4226	0.002118	0.02276				1
CA7	carbonic anhydrase VII	16q22.1				-0.390666667	0.002043333	0.0229				1
ECT2	epithelial cell transforming sequence 2 oncogene	3q26.1-q26.2	1.411	0.00018	0.0034	0.353	0.00191	0.024				2
CA9	carbonic anhydrase IX	9p12				-0.458	1.00E-05	0.00265				1
CAB39L	calcium binding protein 39-like	13q14.2							-0.9365	3.202	6	1
CABIN1	calcineurin binding protein 1	22q11.23				-0.36085	0.0015565	0.016155				1
CABLES1	Cdk5 and Abl enzyme substrate 1	18q11.2				-0.34	0.03177	0.1125				1
CABP1	In multiple Geneids					-0.3565	0.002345	0.0253				1
CABP5	calcium binding protein 5	19q13.33				-0.318666667	0.01158	0.0612				1
CABP7	calcium binding protein 7	22q12.2				-0.3525	0.002795	0.02675				1
PDZK1	PDZ domain containing 1	1q21	1.411	0.016	0.08							1
SNORA75	small nucleolar RNA, H/ACA box 75	2q37.1	1.406	0.00167	0.0168							1
CACHD1	cache domain containing 1	1p31.3				-0.404	0.00048	0.0126				1
CACNA1A	calcium channel, voltage-dependent, P/Q type, alpha 1A subunit	19p13				-0.406916667	0.011082833	0.055081667				1
LBH	limb bud and heart development homolog (mouse)	2p23.1	1.404	0	2.00E-04							1
CTSZ	cathepsin Z	20q13.32	1.403	0	1.00E-04							1
CACNA1E	calcium channel, voltage-dependent, R type, alpha 1E subunit	1q25-q31				0.308	0.00201	0.0232				1
CACNA1G	calcium channel, voltage-dependent, T type, alpha 1G subunit	17q22	-0.368	0.00151	0.0156	-0.312	0.00551	0.0413				2
CACNA1H	calcium channel, voltage-dependent, T type, alpha 1H subunit	16p13.3				-0.481333333	0.0054	0.039666667				1
CACNA1I	calcium channel, voltage-dependent, T type, alpha 1I subunit	22q13.1				-0.418304348	0.000645217	0.010795652				1
CACNA2D1	calcium channel, voltage-dependent, alpha 2/delta subunit 1	7q21-q22				0.376555556	0.023442963	0.09				1
CACNA2D2	calcium channel, voltage-dependent, alpha 2/delta subunit 2	3p21.3				-0.390933333	0.000861333	0.013566667				1
CACNA2D3	calcium channel, voltage-dependent, alpha 2/delta subunit 3	3p21.1	-1.334	0	0	-0.3435	0.006092	0.0373				2
CACNA2D4	calcium channel, voltage-dependent, alpha 2/delta subunit 4	12p13.33				0.34	0.00097	0.0173				1
CXCL1	chemokine (C-X-C motif) ligand 1 (melanoma growth stimulating activity, alph	4q21	1.403	7.00E-05	0.0017							1
CACNB2	calcium channel, voltage-dependent, beta 2 subunit	10p12							-0.664	2.966	5	1
CACNB4	calcium channel, voltage-dependent, beta 4 subunit	2q22-q23	-0.864	2.00E-05	7.00E-04							1
CACNG1	calcium channel, voltage-dependent, gamma subunit 1	17q24	-0.227	0.03336	0.1292							1
CACNG2	calcium channel, voltage-dependent, gamma subunit 2	22q13.1				-0.400741935	0.000824194	0.012422581				1
CACNG3	calcium channel, voltage-dependent, gamma subunit 3	16p12.1				-0.333	0.00748	0.0488				1
CACNG4	calcium channel, voltage-dependent, gamma subunit 4	17q24				-0.3	0.01109	0.0606				1
CACNG6	calcium channel, voltage-dependent, gamma subunit 6	19q13.4				-0.354	0.01182	0.0629				1
CACNG7	calcium channel, voltage-dependent, gamma subunit 7	19q13.4							0.574	2.512	5	1
CACNG8	calcium channel, voltage-dependent, gamma subunit 8	19q13.4				-0.551	0.00023	0.0092	0.973	3.31	6	2
CACUL1	Data not found		-0.371	0.03327	0.1289							1
DAP	death-associated protein	5p15.2	1.403	0	2.00E-04	0.331181818	0.007913636	0.044145455				2
CAD	carbamoyl-phosphate synthetase 2, aspartate transcarbamylase, and dihydro	2p22-p21				-0.387	0.00331	0.0316				1
CADM1	cell adhesion molecule 1	11q23.2				0.32275	0.0034625	0.0289625				1
CADM2	cell adhesion molecule 2	3p12.1				0.426	0.00059	0.0139				1
CADM4	cell adhesion molecule 4	19q13.31	-0.474	0.00038	0.0058	-0.378666667	0.010346667	0.043133333				2
ID2	inhibitor of DNA binding 2, dominant negative helix-loop-helix protein	2p25	1.403	5.00E-05	0.0013							1
HIATL1	hippocampus abundant transcript-like 1	9q22.32	1.401	0.00492	0.0361							1

CAGE1	cancer antigen 1	6p24.3				-0.355333333	0.00283	0.027466667				1
CALB1	calbindin 1, 28kDa	8q21.3				0.309	0.00028	0.0101				1
CALB2	calbindin 2	16q22.2	-1.028	0.02929	0.1186	-0.36175	0.000395	0.01095				2
CALCOCO1	calcium binding and coiled-coil domain 1	12q13.13				-0.338	0.00031	0.0105				1
CALCR	calcitonin receptor	7q21.3				0.339052632	0.006512632	0.039236842				1
CALCRL	calcitonin receptor-like	2q32.1				0.457333333	6.67E-05	0.005166667	-0.78	2.73	5	2
CALD1	caldesmon 1	7q33							-0.796	2.372	5	1
CALHM2	calcium homeostasis modulator 2	10q24.33				-0.423	7.00E-05	0.0059				1
CALM1	calmodulin 1 (phosphorylase kinase, delta)	14q24-q31	-0.593	3.00E-05	9.00E-04							1
IRS1	insulin receptor substrate 1	2q36	1.401	0	1.00E-04							1
IYD	iodotyrosine deiodinase	6q25.1	1.4	1.00E-05	3.00E-04							1
CALML3	calmodulin-like 3	10pter-p13	-0.992	5.00E-05	0.0013							1
FBN1	fibrillin 1	15q21.1	1.397	0.00464	0.0347							1
CALML5	calmodulin-like 5	10p15.1	-0.665	0.00111	0.0125							1
CALML6	calmodulin-like 6	1p36.33	-0.207	0.0324	0.1267							1
CALN1	calneuron 1	7q11				-0.3406	0.014965	0.06919				1
HLA-F	major histocompatibility complex, class I, F	6p21.3	1.397	0.000345	0.0054							1
CALR3	calreticulin 3	19p13.11	-0.323	0.00193	0.0186	-0.345	0.00571	0.034633333				2
MYADM	In multiple Geneids		1.397	0.00019	0.0035							1
DMXL2	In multiple Geneids		1.395	0	2.00E-04							1
CAMK1D	calcium/calmodulin-dependent protein kinase ID	10p13	-1.166	9.00E-05	0.0021	-0.397081081	0.010659189	0.054089189				2
CAMK1G	In multiple Geneids					0.3416	0.004786	0.0273				1
CAMK2A	calcium/calmodulin-dependent protein kinase II alpha	5q32				-0.3744	0.000204	0.00726				1
CAMK2B	calcium/calmodulin-dependent protein kinase II beta	7p14.3-p14.1	-0.371	0.00244	0.022							1
CAMK2D	calcium/calmodulin-dependent protein kinase II delta	4q26				-0.301	0.02645	0.1006				1
CAMK2G	calcium/calmodulin-dependent protein kinase II gamma	10q22				-0.348666667	0.002673333	0.025533333				1
CAMK2N2	calcium/calmodulin-dependent protein kinase II inhibitor 2	3q27.1							0.732	2.5	6	1
CAMKK1	In multiple Geneids					-0.492666667	0.001026667	0.013788889				1
CAMKK2	calcium/calmodulin-dependent protein kinase kinase 2, beta	12q24.2				-0.3682	0.000832	0.01332				1
CAMKV	CaM kinase-like vesicle-associated	3p21.31				-0.446	9.00E-05	0.0063				1
CAMLG	calcium modulating ligand	5q23				-0.3685	0.015915	0.06735				1
CAMP	cathelicidin antimicrobial peptide	3p21.3				-0.381	0.00142	0.0208				1
CAMSAP1	calmodulin regulated spectrin-associated protein 1	9q34.3				0.301	0.00714	0.0476				1
CAMSAP1L1	Data not found					0.431	0.00185	0.0236				1
CAMSAP3	calmodulin regulated spectrin-associated protein family, member 3	19p13.2	-0.771	0.0014	0.0148							1
CAMTA1	calmodulin binding transcription activator 1	1p36.31-p36.23				-0.352666667	0.000750238	0.012714286				1
CAMTA2	calmodulin binding transcription activator 2	17p13.2	-0.278	0.04061	0.147	-0.4565	0.00052	0.0109				2
CAND2	cullin-associated and neddylation-dissociated 2 (putative)	3p25.2				-0.366	0.000846667	0.014966667				1
CANT1	calcium activated nucleotidase 1	17q25.3				-0.408	0.00294	0.0276				1
HNF1A	HNF1 homeobox A	12q24.2	1.388	0	2.00E-04							1
CAP1	CAP, adenylate cyclase-associated protein 1 (yeast)	1p34.2				-0.318	0.00629	0.0444				1
CAP2	CAP, adenylate cyclase-associated protein, 2 (yeast)	6p22.3				-0.352333333	0.007533333	0.046033333				1
CAPN1	calpain 1, (mu/I) large subunit	11q13	-0.927	0.00073	0.0094	-0.342	0.00692	0.0465				2
APOC1	apolipoprotein C-I	19q13.2	1.387	8.00E-05	0.0019							1
CAPN13	calpain 13	2p22-p21							-0.806	3.05	6	1
CAPN2	calpain 2, (m/II) large subunit	1q41-q42	-0.911	0.00212	0.0199							1
CAPN3	calpain 3, (p94)	15q15.1							-0.969	3.30667	6	1
CAPN6	calpain 6	Xq23				0.322	0.0062	0.044	-0.647	2.58857	7	2
CAPN8	In multiple Geneids					-0.322	0.02094	0.0876				1
CAPNS1	calpain, small subunit 1	19q13.12	-0.752	0.00152	0.0158	-0.323	0.00058	0.0138				2
CAPNS2	calpain, small subunit 2	16q12.2							-0.897	2.64571	7	1
CAPS2	calcyphosine 2	12q14.1				0.308	0.02503	0.0974	-0.66	2.72429	7	2
CAPSL	calcyphosine-like	5p13.2				0.372333333	0.00743	0.0465	-0.795	2.89429	7	2
CAPZA1	capping protein (actin filament) muscle Z-line, alpha 1	1p13.2	-0.545	7.00E-04	0.0091	-0.311	0.00341	0.0291				2
CAPZA3	capping protein (actin filament) muscle Z-line, alpha 3	12p12.3							-0.768	3.175	6	1
CAPZB	capping protein (actin filament) muscle Z-line, beta	1p36.1				-0.360230769	0.001612308	0.019923077				1
LHFPL2	lipoma HMGIC fusion partner-like 2	5q14.1	1.385	0	1.00E-04							1
MGAT4A	mannosyl (alpha-1,3-)-glycoprotein beta-1,4-N-acetylglucosaminyltransferase,	2q12	1.385	1.00E-04	0.0022							1
CARD14	caspase recruitment domain family, member 14	17q25	-0.505	0.00304	0.0259				0.705	2.45	5	2
CARD16	caspase recruitment domain family, member 16					0.398	0.00105	0.018				1
CARD17	caspase recruitment domain family, member 17	11q22.3	-0.768	0.00195	0.0188							1
CARD6	caspase recruitment domain family, member 6	5p13.1							-0.808	2.98857	7	1
CARF	Data not found		-0.577	0.0106	0.0609							1
CARHSP1	calcium regulated heat stable protein 1, 24kDa	16p13.2	-0.741	0.00035	0.0055							1
CARKD	carbohydrate kinase domain containing	13q34	-0.554	5.00E-05	0.0013	-0.381	0.01238	0.0646				2
CARM1	coactivator-associated arginine methyltransferase 1	19p13.2				-0.410307692	0.005276923	0.035130769				1
CARS	cysteinyl-tRNA synthetase	11p15.5				-0.408	0.004631667	0.032266667	0.649	2.83617	6	2
CASC1	cancer susceptibility candidate 1	12p12.1				0.613	0.03079	0.1082				1
TFF3	trefoil factor 3 (intestinal)	21q22.3	1.385	0.00145	0.0152							1

CASC3	cancer susceptibility candidate 3	17q11-q21.3				-0.332	0.00195	0.0242				1
PTPRR	protein tyrosine phosphatase, receptor type, R	12q15	1.38	0.00082	0.0103				-0.874	2.958	5	2
SATB2	SATB homeobox 2	2q33	1.369	0	1.00E-04				-0.672	2.668	5	2
SLC19A3	solute carrier family 19, member 3	2q37	1.369	0.00131	0.0142							1
CASKIN1	CASK interacting protein 1	16p13.3				-0.499666667	0.006146667	0.0398				1
CASP1	caspase 1, apoptosis-related cysteine peptidase	11q23							-0.796	3.082	5	1
CASP14	In multiple Geneids		-0.356	0.0299	0.1204							1
CASP3	caspase 3, apoptosis-related cysteine peptidase	4q34				-0.476	4.00E-04	0.0117				1
CASP4	caspase 4, apoptosis-related cysteine peptidase	11q22.2-q22.3							-0.873	2.94667	6	1
CASP5	caspase 5, apoptosis-related cysteine peptidase	11q22.2-q22.3							-0.929	3.24833	6	1
TRAF5	TNF receptor-associated factor 5	1q32	1.368	0.00033	0.0053							1
IGF2BP1	insulin-like growth factor 2 mRNA binding protein 1	17q21.32	1.367	0.02482	0.1065							1
CD151	CD151 molecule (Raph blood group)	11p15.5	1.365	0	0							1
CASP8AP2	caspase 8 associated protein 2	6q15				-0.363	0.009376667	0.052233333				1
CASP9	caspase 9, apoptosis-related cysteine peptidase	1p36.21				-0.42	0.00145	0.02095				1
CASQ2	calsequestrin 2 (cardiac muscle)	1p13.3-p11				-0.334	0.000255	0.00945				1
CASR	calcium-sensing receptor	3q13	-0.349	0.00211	0.0198							1
CAST	calpastatin	5q15							-1.039	2.62	5	1
CASZ1	castor zinc finger 1	1p36.22	-0.816666667	0.002636667	0.023233333	-0.3761	0.000889	0.01473				2
CATSPER1	cation channel, sperm associated 1	11q12.1	-0.213	0.04877	0.1646	-0.454	0.00092	0.017				2
MMP9	matrix metallopeptidase 9 (gelatinase B, 92kDa gelatinase, 92kDa type IV colla	20q11.2-q13.1	1.365	2.00E-04	0.0037							1
CATSPER2P1	In multiple Geneids					-0.359	0.00851	0.0473				1
CATSPER3	cation channel, sperm associated 3	5q31.1				-0.3455	0.0015075	0.019175				1
CATSPER4	cation channel, sperm associated 4	1p35.3	-0.188	0.04562	0.1583	-0.313	0.00052	0.013				2
SGK2	serum/glucocorticoid regulated kinase 2	20q13.2	1.363	0.00257	0.0229							1
CATSPERG	catsper channel auxiliary subunit gamma	19q13.1				-0.407333333	0.002733333	0.0272				1
CBARA1	Data not found					-0.358333333	0.005013333	0.034244444				1
CBFA2T3	core-binding factor, runt domain, alpha subunit 2; translocated to, 3	16q24	-0.277	0.00721	0.0468	-0.392	0.004356667	0.0292				2
VEGFA	vascular endothelial growth factor A	6p12	1.361	0.00017	0.0032							1
SPTBN1	spectrin, beta, non-erythrocytic 1	2p21	1.36	0	0	0.335	0.0012925	0.018125				2
CBLC	Cbl proto-oncogene, E3 ubiquitin protein ligase C	19q13.2				-0.357	0.00936	0.0552				1
CBLN2	In multiple Geneids					-0.327	8.00E-05	0.0061				1
CBLN4	cerebellin 4 precursor	20q13				0.408	0.001325	0.0187				1
CBR1	carbonyl reductase 1	21q22.13	-0.998	0	0	-0.354	0.00773	0.04655				2
CBR3	carbonyl reductase 3	21q22.2	-0.666	0	2.00E-04	-0.307	0.00365	0.0333				2
CBR4	carbonyl reductase 4	4q32.3				-0.497	0.00052	0.0131				1
CBS	cystathionine-beta-synthase	21q22.3				-0.407	0.00333	0.0317				1
CBWD1	In multiple Geneids								-0.675	2.66	5	1
RCN1	reticulocalbin 1, EF-hand calcium binding domain	11p13	1.359	0	1.00E-04							1
CBX1	chromobox homolog 1	17q21.32				-0.522	0.00045	0.0124				1
CDC42BPA	In multiple Geneids		1.358	4.00E-05	0.0012	0.359	0.000605	0.014				2
CBX5	chromobox homolog 5	12q13.13	-0.641	0.00448	0.0339	-0.4005	0.00029	0.0083				2
CBX7	chromobox homolog 7	22q13.1				-0.49725	0.00017	0.007075				1
CBY1	chibby homolog 1 (Drosophila)	22q12				-0.412333333	0.001716667	0.0197				1
CC2D1A	coiled-coil and C2 domain containing 1A	19p13.12				-0.4285	0.0097775	0.048275				1
CC2D1B	coiled-coil and C2 domain containing 1B	1p32.3				-0.408	0.002485	0.02655				1
CC2D2A	coiled-coil and C2 domain containing 2A	4p15.32				-0.314	0.007025	0.0466				1
PDIA4	protein disulfide isomerase family A, member 4	7q35	1.358	1.00E-05	3.00E-04							1
CCBE1	collagen and calcium binding EGF domains 1	18q21.32	-0.442	0.0055	0.039	-0.353193548	0.003650645	0.029116129				2
CCBL2	cysteine conjugate-beta lyase 2	1p22.2	-0.784	0	0							1
CCBP2	chemokine binding protein 2	3p21.3				-0.308	0.00384	0.0341				1
CCDC100	Data not found								-0.741	3.15667	6	1
CCDC101	coiled-coil domain containing 101	16p11.2				-0.3445	0.001085	0.01505				1
CCDC102A	coiled-coil domain containing 102A	16q21				-0.386	0.00014	0.0075				1
SLCO1B3	solute carrier organic anion transporter family, member 1B3	12p12	1.358	0.0208	0.0952				-0.754	2.83444	9	2
CGN	cingulin	1q21	1.357	0	1.00E-04							1
CCDC105	coiled-coil domain containing 105	19p13.12				-0.3515	0.00185	0.023				1
CCDC107	coiled-coil domain containing 107	9p13.3				-0.415	3.00E-05	0.0041				1
CCDC109A	Data not found					-0.3425	0.007115	0.04665				1
CCDC109B	coiled-coil domain containing 109B	4q25				-0.3338	0.00222	0.02246				1
CCDC11	coiled-coil domain containing 11	18q21.1	-0.233	0.04931	0.1657							1
CCDC110	coiled-coil domain containing 110	4q35.1				-0.402666667	0.01171	0.056966667				1
CCDC111	coiled-coil domain containing 111	4q35.1				-0.4318	0.003345	0.02796				1
FADS2	In multiple Geneids		1.357	0.0079	0.0498							1
PDGFA	platelet-derived growth factor alpha polypeptide	7p22	1.355	8.00E-05	0.0018							1
CCDC114	coiled-coil domain containing 114	19q13.33				-0.361	0.014215	0.06565				1
CCDC115	coiled-coil domain containing 115	2q21.1	-0.406	0.00183	0.0179							1
CCDC116	coiled-coil domain containing 116	22q11.21	-0.197	0.04262	0.1516							1
CCDC117	coiled-coil domain containing 117	22q12.1				-0.309	0.00402	0.0349				1

CCDC12	coiled-coil domain containing 12	3p21.31	-0.389	0.00219	0.0204	-0.3793	0.000976	0.01414				2
CCDC120	coiled-coil domain containing 120	Xp11.23	-1.016	2.00E-05	7.00E-04							1
CCDC122	coiled-coil domain containing 122	13q14.11				0.355	0.00237	0.0267				1
CCDC123	Data not found					-0.35825	0.0017775	0.01975				1
CCDC124	coiled-coil domain containing 124	19p13.11	-0.304	0.02586	0.1097	-0.494	0.00082	0.0161				2
CCDC125	coiled-coil domain containing 125	5q13.2				-0.329	0.008673333	0.051133333				1
CCDC129	coiled-coil domain containing 129	7p14.3				0.373666667	0.00133	0.015233333				1
CCDC13	coiled-coil domain containing 13	3p22.1				-0.3546	0.001926	0.0189				1
CCDC130	coiled-coil domain containing 130	19p13.2				-0.4045	0.005815	0.03915				1
CCDC132	coiled-coil domain containing 132	7q21.3				0.326666667	0.007330667	0.043046667				1
CCDC134	coiled-coil domain containing 134	22q13.2				-0.446875	0.00100125	0.0118125				1
CCDC135	coiled-coil domain containing 135	16q21				-0.356	0.003045	0.02235				1
CCDC136	coiled-coil domain containing 136	7q33				-0.363	0.00276	0.0288				1
CCDC137	coiled-coil domain containing 137	17q25.3	-0.263	0.01296	0.0697							1
SLC39A10	solute carrier family 39 (zinc transporter), member 10	2q32.3	1.355	0	1.00E-04							1
CCDC140	coiled-coil domain containing 140	2q36.1	-0.19	0.04724	0.1614							1
CCDC141	coiled-coil domain containing 141	2q31.2				0.385	0.00021	0.0088				1
RNF157	ring finger protein 157	17q25.1	1.353	3.00E-04	0.0049							1
CCDC144C	coiled-coil domain containing 144C	17p11.2				-0.355	0.0059	0.0428				1
CCDC146	coiled-coil domain containing 146	7q11.23				0.5195	0.016185	0.06145				1
CCDC148	coiled-coil domain containing 148	2q24.1				0.342	1.00E-05	0.0027				1
CCDC149	coiled-coil domain containing 149	4p15.2				-0.3935625	0.002070625	0.02105625				1
CCDC15	coiled-coil domain containing 15	11q24.2							-0.853	2.76667	6	1
CCDC151	coiled-coil domain containing 151	19p13.2				-0.401666667	0.009153333	0.0515				1
CCDC152	coiled-coil domain containing 152	5p12				0.37025	0.0020525	0.024425				1
CCDC154	coiled-coil domain containing 154	16p13.3	-0.311	0.00072	0.0093							1
CCDC155	coiled-coil domain containing 155	19q13.33	-0.2	0.0403	0.1463	-0.522	0.000305	0.0089				2
CCDC157	coiled-coil domain containing 157	22q12.2	-0.349	0.00015	0.003	-0.374	0.001376667	0.0171				2
CCDC159	coiled-coil domain containing 159	19p13.2	-0.351	0.04281	0.152							1
CCDC17	coiled-coil domain containing 17	1p34.1				-0.436666667	0.00966	0.0467	0.709	2.89	5	2
CCDC171	coiled-coil domain containing 171	9p22.3	-0.446	0.00454	0.0342							1
CCDC177	Data not found		-0.342	0.00095	0.0113							1
TNFAIP6	tumor necrosis factor, alpha-induced protein 6	2q23.3	1.353	7.00E-05	0.0016				-0.958	3.52333	6	2
CCDC21	Data not found					-0.41075	0.0074625	0.0419				1
CCDC23	coiled-coil domain containing 23	1p34.2							-0.783	2.663	5	1
CCDC26	coiled-coil domain containing 26	8q24.21	-0.247	0.01038	0.0601							1
CCDC27	coiled-coil domain containing 27	1p36.32				-0.4075	7.50E-05	0.00535				1
CCDC28B	coiled-coil domain containing 28B	1p36.11-p34.2				-0.407	0.00018	0.0083				1
CCDC3	coiled-coil domain containing 3	10p13				-0.351142857	0.014974286	0.065328571				1
CCDC33	coiled-coil domain containing 33	15q24.1				-0.3155	0.007115	0.0468				1
DLGAP1-AS1	Data not found		1.352	0.00011	0.0023							1
CCDC36	coiled-coil domain containing 36	3p21.31				-0.375	0.001468333	0.018066667				1
CCDC38	coiled-coil domain containing 38	12q23.1	-0.199	0.0292	0.1184							1
CCDC4	Data not found								0.656	2.436	5	1
MEST	mesoderm specific transcript homolog (mouse)	7q32	1.35	6.00E-05	0.0016							1
CCDC42	coiled-coil domain containing 42	17p13.1	-0.227	0.03356	0.1297	-0.4175	0.001515	0.0187				2
CCDC42B	coiled-coil domain containing 42B	12q24.13	-0.301	0.00753	0.0482							1
CCDC45	Data not found								-0.644	3.06125	8	1
CCDC49	Data not found					-0.325	0.00742	0.0486				1
P4HA1	prolyl 4-hydroxylase, alpha polypeptide I	10q21.3-q23.1	1.349	4.00E-05	0.0012							1
CCDC51	coiled-coil domain containing 51	3p21.31				-0.3335	0.005935	0.04295				1
CCDC55	Data not found					-0.39	0.00037	0.0092				1
CCDC56	coiled-coil domain containing 56	17q21				-0.352	0.00091	0.0169	0.923	3.034	5	2
CCDC57	coiled-coil domain containing 57	17q25.3				-0.3562	0.000296	0.01014				1
CCDC6	coiled-coil domain containing 6	10q21				0.314	0.00078	0.0158				1
CCDC60	coiled-coil domain containing 60	12q24.23	-0.398	0.00214	0.02							1
CCDC61	coiled-coil domain containing 61	19q13.32	-0.234	0.03153	0.1247							1
CCDC62	coiled-coil domain containing 62	12q24.31	-0.238	0.03555	0.1348	-0.3555	0.000425	0.00995				2
CCDC64	coiled-coil domain containing 64	12q24.23	-0.726	3.00E-04	0.005	-0.33	0.00059	0.01375				2
CCDC64B	coiled-coil domain containing 64B	16p13.3	-1.111	0	2.00E-04	-0.398	0.0183	0.0768				2
CCDC65	coiled-coil domain containing 65	12q13.12	-0.337	0.00133	0.0144							1
ETS1	v-ets erythroblastosis virus E26 oncogene homolog 1 (avian)	11q23.3	1.348	6.00E-05	0.0016	0.328	0.00224	0.026				2
CCDC69	coiled-coil domain containing 69	5q33.1	-0.686	0.00899	0.0543	-0.3572	0.001588	0.01934				2
CCDC70	coiled-coil domain containing 70	13q14.3							-0.936	2.956	5	1
CCDC71	coiled-coil domain containing 71	3p21.31	-0.482	0.00289	0.0249	-0.396666667	0.00196	0.023566667				2
CCDC74A	coiled-coil domain containing 74A	2q21.1	-0.407	0.0363	0.1368							1
CCDC76	Data not found								-0.502	2.544	5	1
CCDC79	coiled-coil domain containing 79	16q22.1				-0.404	0.00609	0.0436				1
CCDC8	coiled-coil domain containing 8	19q13.32	-0.217	0.03218	0.1262							1

SLC46A3	solute carrier family 46, member 3	13q12.3	1.347	0.00135	0.0145							1
CCDC85A	coiled-coil domain containing 85A	2p16.1				0.328833333	0.001480833	0.0191				1
CCDC85C	coiled-coil domain containing 85C	14q32.31	-0.7115	0.000455	0.0058	-0.3232	0.002032	0.01918				2
AGMO	alkylglycerol monooxygenase	7p21.2	1.344	0.01029	0.0597							1
CCDC88A	coiled-coil domain containing 88A	2p16.1				-0.32	0.00091	0.0169				1
CCDC88C	coiled-coil domain containing 88C	14q32.11				-0.396333333	0.002126667	0.021546667				1
CCDC9	coiled-coil domain containing 9	19q13.32				-0.377	0.0045	0.03645				1
NEU1	sialidase 1 (lysosomal sialidase)	6p21.3	1.344	0.00356	0.0289							1
NFE2L3	nuclear factor (erythroid-derived 2)-like 3	7p15.2	1.344	2.00E-05	6.00E-04							1
CCDC94	coiled-coil domain containing 94	19p13.3	-0.331	0.01994	0.0925	-0.446	0.00508	0.03535				2
CCDC96	coiled-coil domain containing 96	4p16.1	-0.24	0.02115	0.0963							1
CCDC99	coiled-coil domain containing 99	5q35.1							-1.169	3.07167	6	1
CCER1	coiled-coil glutamate-rich protein 1	12q21.33	-0.279	0.0034	0.028							1
CCKAR	cholecystokinin A receptor	4p15.1-p15.2				-0.4535	0.000385	0.01145				1
CCL1	chemokine (C-C motif) ligand 1	17q12							-0.796	2.816	5	1
CCL13	chemokine (C-C motif) ligand 13	17q11.2							-0.869	3.20429	7	1
CCL17	chemokine (C-C motif) ligand 17	16q13	-0.317	0.00596	0.0411				0.605	2.648	5	2
GPR39	G protein-coupled receptor 39	2q21-q22	1.343	1.00E-04	0.0021	0.3525	0.00013	0.0068				2
LAMC1	laminin, gamma 1 (formerly LAMB2)	1q31	1.343	0	1.00E-04							1
CCL21	chemokine (C-C motif) ligand 21	9p13	-1.558	0.0088	0.0535							1
CCL22	chemokine (C-C motif) ligand 22	16q13	-0.733	0.02363	0.1032							1
CCL24	chemokine (C-C motif) ligand 24	7q11.23							0.864	3.76333	6	1
CCL27	chemokine (C-C motif) ligand 27	9p13	-0.207	0.01058	0.0609							1
C12orf23	chromosome 12 open reading frame 23	12q23.3	1.336	0	2.00E-04							1
FCGR3A	Fc fragment of IgG, low affinity IIIa, receptor (CD16a)	1q23	1.3355	0.00361	0.02445							1
SULT1E1	sulfotransferase family 1E, estrogen-preferring, member 1	4q13.1	1.335	0.0122	0.067	0.391	0.00062	0.0142	-0.925	2.90429	7	3
COL4A2	collagen, type IV, alpha 2	13q34	1.334	0	0	0.322	0.0224	0.0912				2
STAMBPL1	STAM binding protein-like 1	10q23.31	1.334	0	1.00E-04							1
CCL7	chemokine (C-C motif) ligand 7	17q11.2-q12							-0.947	2.854	5	1
CCM2	In multiple Geneids								0.765	2.89	5	1
CCNB1	cyclin B1	5q12				-0.37	0.008075	0.0481				1
CCNB1IP1	cyclin B1 interacting protein 1, E3 ubiquitin protein ligase	14q11.2							-0.884	2.977	5	1
CALU	calumenin	7q32.1	1.333	0	0							1
CCNB3	cyclin B3	Xp11				0.394	2.00E-05	0.0036	-0.901	2.92667	6	2
CCNC	cyclin C	6q21	-0.673	0.00841	0.0519							1
CCND1	In multiple Geneids		-0.375	0.01604	0.0801							1
CCNDBP1	In multiple Geneids		-0.43	0.00775	0.0491							1
CCNF	cyclin F	16p13.3				-0.3836	0.019554	0.07928	0.612	2.67167	6	2
CCNG1	cyclin G1	5q32-q34	-0.55	0.00909	0.0547							1
CCNG2	cyclin G2	4q21.1	-1.502	5.00E-05	0.0013							1
CCNH	cyclin H	5q13.3-q14							-0.89	3.08	5	1
CCNI	cyclin I	4q21.1	-0.672	0.00026	0.0045	-0.316	0.00639	0.0448				2
CLMN	calmin (calponin-like, transmembrane)	14q32.13	1.333	6.00E-05	0.0015	0.472	0.0015	0.0213				2
CCL28	chemokine (C-C motif) ligand 28	5p12	1.331	0.00248	0.0222							1
CCNJL	cyclin J-like	5q33.3				-0.3871	0.003689	0.02881				1
LY75	lymphocyte antigen 75	2q24	1.33	3.00E-04	0.0049	0.344	3.00E-05	0.0044				2
CCNL2	cyclin L2	1p36.33				-0.513	0.00016	0.008				1
CCNT1	cyclin T1	12q13.11							-0.679	2.602	5	1
MB21D1	Mab-21 domain containing 1	6q13	1.33	0.00105	0.0121							1
CCNYL1	cyclin Y-like 1	2q33.3	-1.002	5.00E-05	0.0013							1
CTGF	connective tissue growth factor	6q23.1	1.329	0.00467	0.0348							1
EFNA1	ephrin-A1	1q21-q22	1.327	2.00E-05	6.00E-04							1
LOC151009	uncharacterized LOC151009	2q13	1.325666667	0.00237	0.0178							1
CCR10	In multiple Geneids					-0.382	0.00292	0.0296				1
CCR3	chemokine (C-C motif) receptor 3	3p21.3							-1.064	2.874	5	1
CCR4	chemokine (C-C motif) receptor 4	3p24				-0.36	0.00021	0.0089				1
CCR5	chemokine (C-C motif) receptor 5 (gene/pseudogene)	3p21.31	-0.363	0.01202	0.0663							1
CCR7	chemokine (C-C motif) receptor 7	17q12-q21.2				-0.318	0.00584	0.0426				1
ITGA5	integrin, alpha 5 (fibronectin receptor, alpha polypeptide)	12q11-q13	1.324	0.00049	0.007							1
CCRL1	chemokine (C-C motif) receptor-like 1	3q22							-0.982	3.59375	8	1
AURKA	In multiple Geneids		1.323	0.00079	0.0099							1
CCRN4L	CCR4 carbon catabolite repression 4-like (S. cerevisiae)	4q31.1				-0.3345	0.012375	0.063325				1
CCSER2	Data not found		-0.838	3.00E-05	8.00E-04							1
MMP15	matrix metallopeptidase 15 (membrane-inserted)	16q13	1.323	0.00013	0.0028							1
C8orf4	chromosome 8 open reading frame 4	8p11.2	1.32	0.00079	0.0099				-0.892	3.33571	7	2
LOC100134868	uncharacterized LOC100134868	20p11.1	1.3175	0.00017	0.0033							1
MAP3K13	mitogen-activated protein kinase kinase kinase 13	3q27	1.317	1.00E-05	3.00E-04							1
CCT6B	chaperonin containing TCP1, subunit 6B (zeta 2)	17q12	-0.277	0.0119	0.0658							1
ASS1	argininosuccinate synthase 1	9q34.1	1.315	0.00044	0.0066							1

CCT8	chaperonin containing TCP1, subunit 8 (theta)	21q22.11							-0.79	3.03714	7	1
CD101	CD101 molecule	1p13	-0.527	0.0026	0.0231							1
CD109	CD109 molecule	6q13	-1.382	0.01247	0.0679							1
ATHL1	ATH1, acid trehalase-like 1 (yeast)	11p15.5	1.315	3.00E-04	0.0049							1
FZD5	frizzled family receptor 5	2q33.3	1.315	2.00E-05	6.00E-04							1
CD163	CD163 molecule	12p13.3							-0.807	2.87667	9	1
CD163L1	CD163 molecule-like 1	12p13.3				0.345	4.00E-04	0.0117	-0.82	3.2975	8	2
RAI14	retinoic acid induced 14	5p13.3-p13.2	1.315	0	0	0.3375	0.0358475	0.120625				2
CD164L2	CD164 sialomucin-like 2	1p36.11	-0.989	1.00E-05	3.00E-04	-0.394	0.0059	0.0428				2
CD177	CD177 molecule	19q13.2				-0.387	6.00E-05	0.0054				1
CD19	CD19 molecule	16p11.2				-0.303	0.00203	0.0247				1
CD1A	CD1a molecule	1q22-q23							-0.996	3.19714	7	1
CD1B	CD1b molecule	1q22-q23							-0.792	3.15333	6	1
CD1C	CD1c molecule	1q22-q23							-0.865	2.78167	6	1
CD1E	CD1e molecule	1q22-q23							-0.762	2.734	5	1
CD2	CD2 molecule	1p13.1							-0.718	2.65167	6	1
PAG1	phosphoprotein associated with glycosphingolipid microdomains 1	8q21.13	1.314	0.00014	0.0028	0.3322	0.00188	0.01474				2
CD200R1	CD200 receptor 1	3q13.2	-1.169	0	1.00E-04							1
CD200R2	Data not found								-0.938	3.35625	8	1
CD207	CD207 molecule, langerin	2p13	-2.318	0	1.00E-04							1
CD209	CD209 molecule	19p13				-0.5055	0.001385	0.01755				1
CD22	CD22 molecule	19q13.1				-0.302	0.00311	0.0306				1
CD226	CD226 molecule	18q22.3				-0.341	0.00552	0.0413				1
CD24	In multiple Geneids		-1.094	0.00094	0.0112							1
ARHGAP18	Rho GTPase activating protein 18	6q22.33	1.31	0.00081	0.0101	0.323	0.02582	0.0992	-1.304	3.16	5	3
CD274	CD274 molecule	9p24							-0.802	2.54333	6	1
GOLT1A	golgi transport 1A	1q32.1	1.31	0.00015	0.0029				-0.654	2.546	5	2
CTSH	cathepsin H	15q25.1	1.308	1.00E-05	5.00E-04							1
A2M	alpha-2-macroglobulin	12p13.31	1.306	0.00347	0.0283							1
CD300C	CD300c molecule	17q25.1				-0.311	0.0117	0.0625				1
DOCK5	dedicator of cytokinesis 5	8p21.2	1.305	0	1.00E-04							1
CD33	CD33 molecule	19q13.3				-0.302	0.00279	0.0289				1
GCNT3	glucosaminyl (N-acetyl) transferase 3, mucin type	15q21.3	1.305	0.00287	0.0248							1
CD36	CD36 molecule (thrombospondin receptor)	7q11.2				0.473	0.016795	0.0663	-0.891	3.215835	6	2
CD3D	CD3d molecule, delta (CD3-TCR complex)	11q23							0.559	2.738	5	1
CD3EAP	CD3e molecule, epsilon associated protein	19q13.3							-0.662	2.33	5	1
CD3G	CD3g molecule, gamma (CD3-TCR complex)	11q23							0.559	2.738	5	1
CD40LG	CD40 ligand	Xq26							-0.783	2.818	5	1
CD44	CD44 molecule (Indian blood group)	11p13				0.303	0.02045	0.0864				1
CD46	CD46 molecule, complement regulatory protein	1q32				0.338	0.003535	0.02855				1
CD47	CD47 molecule	3q13.1-q13.2				0.305	0.00845	0.0522				1
CD5	CD5 molecule	11q13				-0.358	0.009605	0.045				1
CD52	CD52 molecule	1p36				-0.525	0.00067	0.0147	0.737	3.048	5	2
LRP6	low density lipoprotein receptor-related protein 6	12p13.2	1.305	1.00E-05	3.00E-04							1
CD59	CD59 molecule, complement regulatory protein	11p13	-0.93	0.00053	0.0074							1
CD5L	CD5 molecule-like	1q21-q23							-0.823	2.94429	7	1
CD6	CD6 molecule	11q13				-0.35425	0.0025725	0.02505				1
PPP1R1B	In multiple Geneids		1.304	0.00279	0.0243							1
CD68	CD68 molecule	17p13				-0.645	2.00E-05	0.0036				1
CD7	CD7 molecule	17q25.2-q25.3							1.077	4.23062	8	1
CD70	CD70 molecule	19p13				-0.35	0.02424	0.08045				1
CD72	CD72 molecule	9p13.3				-0.382333333	0.00061	0.012566667				1
OCIAD2	OCIA domain containing 2	4p11	1.302	2.00E-05	6.00E-04							1
CD81	CD81 molecule	11p15.5				-0.383	0.00152	0.0215				1
CD82	In multiple Geneids		-0.514	0.02271	0.1005							1
TMEM56	transmembrane protein 56	1p21.3	1.301	0.00291	0.025							1
CD84	CD84 molecule	1q24							-0.983	3.16167	6	1
CD86	CD86 molecule	3q21							-1.068	3.294	5	1
CD8A	CD8a molecule	2p12	-0.529	0.04431	0.1554				0.897	2.70857	7	2
CD8B	CD8b molecule	2p12							0.819	2.78833	6	1
KCNJ3	potassium inwardly-rectifying channel, subfamily J, member 3	2q24.1	1.3	4.00E-05	0.0012	0.3418	0.000536	0.00992				2
KLHL23	kelch-like 23 (Drosophila)	2q31.1	1.3	0	0							1
CD99	CD99 molecule	Xp22.32 and Yp11.3				-0.38692	0.0027248	0.024232				1
CD99L2	CD99 molecule-like 2	Xq28				-0.361	0.00746	0.0487				1
CDA	cytidine deaminase	1p36.2-p35	-1.764	0.00359	0.029	-0.371333333	0.00129	0.018333333				2
CDADC1	cytidine and dCMP deaminase domain containing 1	13q14.2							-0.661	2.565	6	1
CDC123	cell division cycle 123 homolog (S. cerevisiae)	10p13				-0.3715	0.01415	0.065675				1
GALNT6	UDP-N-acetyl-alpha-D-galactosamine:polypeptide N-acetylgalactosaminyltran	12q13	1.298	9.00E-04	0.0109							1
CDC16	cell division cycle 16 homolog (S. cerevisiae)	13q34				0.312	0.01003	0.0573				1

CDC2	Data not found								-0.844	2.985415	6	1
CDC23	In multiple Geneids					-0.3835	0.001653333	0.020283333				1
CDC25A	cell division cycle 25 homolog A (S. pombe)	3p21				-0.386666667	6.67E-05	0.0047				1
RHOU	ras homolog family member U	1q42.11-q42.3	1.297	0.00099	0.0116							1
ANGPT2	angiopoietin 2	8p23.1	1.295	1.00E-05	5.00E-04							1
PROX1	prospero homeobox 1	1q41	1.293	0.00602	0.0414	0.320666667	0.000443333	0.012033333				2
CDC27	cell division cycle 27 homolog (S. cerevisiae)	17q21.32							-0.81	2.604	5	1
CDC2L1	Data not found					-0.4496	0.003219	0.02422				1
CDC2L2	cell division cycle 2-like 2 (PITSLRE proteins)	1p36.33				-0.5405	0.000235	0.00925				1
CDC2L6	Data not found					-0.363	0.002416667	0.021366667				1
CDC34	cell division cycle 34 homolog (S. cerevisiae)	19p13.3				-0.568	0.00164	0.0223				1
CDC37	cell division cycle 37 homolog (S. cerevisiae)	19p13.2				-0.478333333	0.004513333	0.031033333				1
CDC37L1	cell division cycle 37 homolog (S. cerevisiae)-like 1	9p24.1							-0.761	2.843435	8	1
ATIC	5-aminoimidazole-4-carboxamide ribonucleotide formyltransferase/IMP cyclo	2q35	1.29	0	0							1
CDC42BPB	CDC42 binding protein kinase beta (DMPK-like)	14q32.3				-0.333333333	0.001333333	0.017533333				1
CDC42BPG	CDC42 binding protein kinase gamma (DMPK-like)	11q13.1	-0.715	0	2.00E-04	-0.371	0.00117	0.0189				2
CDC42EP1	CDC42 effector protein (Rho GTPase binding) 1	22q13.1	-0.543	0.01681	0.0826	-0.486	0	0.0011				2
RNU4-1	RNA, U4 small nuclear 1	12q24.31	1.29	0.0078	0.0493							1
CDC42EP3	CDC42 effector protein (Rho GTPase binding) 3	2p21				0.3135	0.000665	0.01455				1
FLJ39632	uncharacterized LOC642477	14q11.2	1.289	0.00019	0.0035							1
CDC42SE1	CDC42 small effector 1	1q21.3	-0.509	0.01055	0.0608							1
LOC654433	uncharacterized LOC654433	2q13	1.289	0.00268	0.0236							1
CDC45L	Data not found					-0.386833333	0.000561667	0.0111				1
GATA4	GATA binding protein 4	8p23.1-p22	1.288	0.01985	0.0923							1
ADAMTS4	ADAM metallopeptidase with thrombospondin type 1 motif, 4	1q21-q23	1.286	0.00013	0.0026							1
CDC7	cell division cycle 7 homolog (S. cerevisiae)	1p22							-0.802	2.822	5	1
RAB20	RAB20, member RAS oncogene family	13q34	1.285	9.00E-05	0.0021							1
NEDD9	neural precursor cell expressed, developmentally down-regulated 9	6p25-p24	1.284	0.00034	0.0054							1
IL32	interleukin 32	16p13.3	1.283	2.00E-05	6.00E-04							1
PADI2	peptidyl arginine deiminase, type II	1p36.13	1.283	0.00779	0.0493							1
CDCP1	CUB domain containing protein 1	3p21.31	-0.781	0.03474	0.1326	-0.322833333	0.001578333	0.018416667				2
CDGAP	Data not found					0.448	1.00E-05	0.0032				1
CDH1	cadherin 1, type 1, E-cadherin (epithelial)	16q22.1				-0.371888889	0.007203889	0.042483333				1
CDH10	cadherin 10, type 2 (T2-cadherin)	5p14.2				0.373	0.002404444	0.020483333				1
RCN3	reticulocalbin 3, EF-hand calcium binding domain	19q13.33	1.283	0	0							1
CDH12	cadherin 12, type 2 (N-cadherin 2)	5p14.3				0.371922222	0.002927222	0.026105556	-0.851	3.50778	9	2
CDH13	cadherin 13, H-cadherin (heart)	16q23.3				-0.3625	0.007431667	0.0451				1
CDH16	cadherin 16, KSP-cadherin	16q22.1				-0.355	0.0038225	0.028925				1
POTEF	POTE ankyrin domain family, member F	2q21.1	1.281	0.00448	0.0319							1
CDH18	cadherin 18, type 2	5p14.3				0.363972973	0.003663514	0.027381081	-0.822	3.13429	7	2
CDH19	cadherin 19, type 2	18q22.1							-0.883	2.61111	9	1
CDH20	cadherin 20, type 2	18q21.33							-0.821	3.07286	7	1
CDH22	cadherin 22, type 2	20q13.1	-0.594	2.00E-05	8.00E-04							1
CDH23	cadherin-related 23	10q22.1				-0.325818182	0.002152727	0.0219				1
CDH24	In multiple Geneids					-0.424	0.00196	0.0243				1
CDH26	cadherin 26	20q13.33				0.360857143	0.008911429	0.051				1
CDH29	Data not found					-0.403	0.00464	0.0377				1
CDH3	cadherin 3, type 1, P-cadherin (placental)	16q22.1				-0.409111111	0.005754444	0.037888889				1
CDH4	cadherin 4, type 1, R-cadherin (retinal)	20q13.3				0.339076923	0.005089231	0.036823077				1
CTSS	cathepsin S	1q21	1.278	0.0029	0.0249							1
AAED1	AhpC/TSA antioxidant enzyme domain containing 1	9q22.32	1.277	0	1.00E-04							1
CDH9	cadherin 9, type 2 (T1-cadherin)	5p14				0.3876	0.003876	0.02585				1
CDHR1	cadherin-related family member 1	10q23.1	-1.729	0.00022	0.0039							1
BAG2	BCL2-associated athanogene 2	6p12.1-p11.2	1.275	0.00082	0.0102							1
CLIC5	chloride intracellular channel 5	6p12.3	1.275	0.00505	0.0368	0.306333333	0.00306	0.0303				2
CDIP1	Data not found		-0.454	0.00071	0.0092							1
CDK10	cyclin-dependent kinase 10	16q24				-0.372	0.00704	0.0472				1
GLRX	glutaredoxin (thioltransferase)	5q14	1.275	0.00274	0.024							1
REPS2	RALBP1 associated Eps domain containing 2	Xp22.2	1.275	0.00191	0.0185	0.308	0.00139	0.0206				2
HEXB	hexosaminidase B (beta polypeptide)	5q13	1.274	0	0							1
LGALS1	lectin, galactoside-binding, soluble, 1	22q13.1	1.274	0.00076	0.0096							1
CDK19	cyclin-dependent kinase 19	6q21	-0.493	0.00217	0.0202							1
CCNI2	cyclin I family, member 2	5q31.1	1.271	0	1.00E-04							1
CDK2AP1	cyclin-dependent kinase 2 associated protein 1	12q24.31				-0.4	0.00025	0.0095	0.828	3.20167	6	2
CDK3	cyclin-dependent kinase 3	17q22-qter				-0.371	0.00022	0.0091				1
CYR61	cysteine-rich, angiogenic inducer, 61	1p22.3	1.271	0.00723	0.047							1
CDK5R1	cyclin-dependent kinase 5, regulatory subunit 1 (p35)	17q11.2	-0.91	1.00E-05	3.00E-04							1
RAD51AP1	RAD51 associated protein 1	12p13.2-p13.1	1.27	8.00E-05	0.0019				-0.857	3.17	6	2
GATA6	GATA binding protein 6	18q11.1-q11.2	1.268	0.00084	0.0104	0.396	0.02574	0.099	-0.631	2.82286	7	3

GFPT1	glutamine--fructose-6-phosphate transaminase 1	2p13	1.268	0.00011	0.0024							1
CDK6	cyclin-dependent kinase 6	7q21-q22				0.334428571	0.009542857	0.052328571				1
CDK7	cyclin-dependent kinase 7	5q12.1							-0.875	3.216	5	1
CDKL1	cyclin-dependent kinase-like 1 (CDC2-related kinase)	14q21.3				-0.379666667	0.004768333	0.034866667				1
CDKL2	cyclin-dependent kinase-like 2 (CDC2-related kinase)	4q21.1				-0.324	0.012825	0.06395				1
CDKL3	cyclin-dependent kinase-like 3	5q31							-0.721	2.67714	7	1
CDKL4	cyclin-dependent kinase-like 4	2p22.1							-0.958	3.11333	6	1
CDKL5	cyclin-dependent kinase-like 5	Xp22	-1.215	0.00013	0.0026							1
RASGRP3	RAS guanyl releasing protein 3 (calcium and DAG-regulated)	2p25.1-p24.1	1.268	0.00012	0.0025							1
CDKN2AIP	CDKN2A interacting protein	4q35.1	-0.826	0	2.00E-04	-0.53	0.00081	0.016				2
CDKN2AIPNL	CDKN2A interacting protein N-terminal like	5q31.1				-0.367605263	0.004963684	0.030960526				1
CDKN2B	cyclin-dependent kinase inhibitor 2B (p15, inhibits CDK4)	9p21							-0.887	2.60833	6	1
CDKN2BAS	Data not found					-0.36475	0.00354875	0.0266375				1
CDKN2C	cyclin-dependent kinase inhibitor 2C (p18, inhibits CDK4)	1p32	-0.181	0.04101	0.148							1
CDKN2D	cyclin-dependent kinase inhibitor 2D (p19, inhibits CDK4)	19p13	-0.209	0.01832	0.0876							1
TBX3	T-box 3	12q24.1	1.268	1.00E-05	5.00E-04							1
SESTD1	In multiple Geneids		1.266	5.00E-05	0.0013	0.312	0	0.0011				2
ABHD2	abhydrolase domain containing 2	15q26.1	1.264	0.00047	0.0069	0.337	0.00631	0.0445				2
CDRT1	In multiple Geneids		-1.294	0.004586667	0.033366667	-0.3622	0.006514	0.03626				2
CDRT15	CMT1A duplicated region transcript 15	17p12	-0.246	0.01087	0.0618							1
CDRT4	In multiple Geneids					-0.322	0.00128	0.01965				1
CDS1	CDP-diacylglycerol synthase (phosphatidate cytidylyltransferase) 1	4q21.23	-0.893	0.00318	0.0268							1
LIF	leukemia inhibitory factor	22q12.2	1.264	0.00013	0.0027							1
TNFRSF12A	tumor necrosis factor receptor superfamily, member 12A	16p13.3	1.264	6.00E-05	0.0015							1
CDY1	chromodomain protein, Y-linked, 1	Yq11.23	-0.202	0.04622	0.1595	-0.635	0.024225	0.08945				2
CDY1B	chromodomain protein, Y-linked, 1B	Yq11.223				-0.635	0.024225	0.08945				1
CDYL	chromodomain protein, Y-like	6p25.1				-0.3491	0.002859	0.02631				1
CEACAM19	carcinoembryonic antigen-related cell adhesion molecule 19	19q13.31				-0.37	0.00103	0.0178				1
CEACAM21	carcinoembryonic antigen-related cell adhesion molecule 21	19q13.2	-0.302	0.0176	0.0852							1
CEACAM3	carcinoembryonic antigen-related cell adhesion molecule 3	19q13.2	-0.242	0.02563	0.109	-0.413	0.00103	0.0179				2
CEACAM4	carcinoembryonic antigen-related cell adhesion molecule 4	19q13.2	-0.447	0.00118	0.0131							1
CEACAM7	carcinoembryonic antigen-related cell adhesion molecule 7	19q13.2	-2.157	0.01281	0.0691							1
C19orf77	chromosome 19 open reading frame 77	19p13.3	1.262	8.00E-04	0.0101							1
CEBPE	CCAAT/enhancer binding protein (C/EBP), epsilon	14q11.2	-0.464	1.00E-04	0.0021							1
ZFAND2A	zinc finger, AN1-type domain 2A	7p22.3	1.261	5.00E-05	0.0012							1
CECR1	cat eye syndrome chromosome region, candidate 1	22q11.2				-0.4402	0.000188	0.00798				1
CECR2	cat eye syndrome chromosome region, candidate 2	22q11.2				-0.4236875	0.001335	0.0163625				1
CECR4	cat eye syndrome chromosome region, candidate 4 (non-protein coding)					-0.4325	0.000195	0.0082				1
CECR5	cat eye syndrome chromosome region, candidate 5					-0.42875	0.0003475	0.0088				1
CECR6	cat eye syndrome chromosome region, candidate 6		-0.265	0.01279	0.069	-0.462	4.00E-05	0.0047				2
CECR7	In multiple Geneids					-0.383	0.00197	0.01835				1
CELA1	chymotrypsin-like elastase family, member 1	12q13				-0.358	1.00E-05	0.0033				1
CELA2B	chymotrypsin-like elastase family, member 2B	1p36.21				-0.3535	0.0011	0.01655				1
CELA3A	chymotrypsin-like elastase family, member 3A	1p36.12	-0.334	0.01242	0.0677	-0.36	0.00904	0.0541				2
CELA3B	chymotrypsin-like elastase family, member 3B	1p36.12	-0.383	0.01074	0.0614							1
CELSR1	cadherin, EGF LAG seven-pass G-type receptor 1 (flamingo homolog, Drosophi	22q13.3	-0.994	0	1.00E-04	-0.4518	0.001216333	0.01351				2
CELSR2	cadherin, EGF LAG seven-pass G-type receptor 2 (flamingo homolog, Drosophi	1p21	-1.63	0	0	-0.371	0.01399	0.05675				2
SDC2	syndecan 2	8q22-q23	1.26	0.00156	0.016	0.311	0.02278	0.0921				2
CEMP1	cementum protein 1	16p13.3				-0.381	0.01387	0.0689				1
LTBP1	latent transforming growth factor beta binding protein 1	2p22-p21	1.259	0.00765	0.0487	0.339	0.0017	0.0226				2
MOGAT3	monoacylglycerol O-acyltransferase 3	7q22.1	1.259	0.00047	0.0068							1
CENPH	centromere protein H	5p15.2				-0.357	0.002756667	0.025966667				1
QPRT	quinolinate phosphoribosyltransferase	16p11.2	1.259	0.00764	0.0487							1
SALL4	sal-like 4 (Drosophila)	20q13.2	1.259	0.00514	0.0372							1
CENPN	centromere protein N	16q23.2				-0.3875	0.002915	0.0279				1
TMEM150B	transmembrane protein 150B	19q13.42	1.257	0.00136	0.0146							1
SNORA42	small nucleolar RNA, H/ACA box 42	1q22	1.256	0.0073	0.0473							1
PLS1	plastin 1	3q23	1.255	0.00025	0.0043							1
CENTD1	Data not found								-0.89	3.31429	7	1
FN1	fibronectin 1	2q34	1.2535	0.017455	0.06835							1
SOAT1	sterol O-acyltransferase 1	1q25	1.253	0.00054	0.0076							1
GCC2	GRIP and coiled-coil domain containing 2	2q12.3	1.252	0	2.00E-04							1
CEP170L	centrosomal protein 170kDa-like	4q26				1.419	0	9.00E-04				1
TMEM163	transmembrane protein 163	2q21.3	1.25	0.00332	0.0275							1
HLA-A	major histocompatibility complex, class I, A	6p21.3	1.248	0.00041	0.0058							1
CEP250	centrosomal protein 250kDa	20q11.22				0.581	0.00045	0.0124				1
PMP22	peripheral myelin protein 22	17p12	1.245	0.00539	0.0385							1
ATP11A	ATPase, class VI, type 11A	13q34	1.242	0.00036	0.0056							1
TNFSF15	tumor necrosis factor (ligand) superfamily, member 15	9q32	1.242	3.00E-05	9.00E-04							1

GUCY1A3	guanylate cyclase 1, soluble, alpha 3	4q31.1-q31.2	1.241	0.00442	0.0335							1
PARP14	poly (ADP-ribose) polymerase family, member 14	3q21.1	1.241	1.00E-04	0.0022				-0.733	2.804	5	2
FADS1	fatty acid desaturase 1	11q12.2-q13.1	1.24	0.02202	0.0988							1
CXCL2	chemokine (C-X-C motif) ligand 2	4q21	1.239	1.00E-05	4.00E-04							1
CERK	ceramide kinase	22q13.31				-0.382533333	0.001230667	0.012653333				1
CERKL	In multiple Geneids					0.4932	0.00012	0.00506				1
MIR622	microRNA 622	13q31.3	1.234	1.00E-05	5.00E-04							1
CERS3	ceramide synthase 3	15q26.3	-4.399	0	2.00E-04							1
CERS4	ceramide synthase 4	19p13.2	-1.769	0	0							1
CES2	In multiple Geneids		-0.848	0.02561	0.109	-0.4245	0.00036	0.0111				2
CES3	carboxylesterase 3	16q22.1				-0.3515	0.01103	0.06				1
CES8	Data not found					-0.339	0.00818	0.0492				1
CETP	cholesteryl ester transfer protein, plasma	16q21				-0.3275	0.00113	0.0179				1
TXNRD1	thioredoxin reductase 1	12q23-q24.1	1.234	0.00015	0.003				-0.77025	2.6701425	5.5	2
CFDP1	craniofacial development protein 1	16q22.2-q22.3				-0.4295	0.00543	0.0336				1
CFH	complement factor H	1q32				0.361666667	0.001521667	0.015283333				1
CFHR3	complement factor H-related 3	1q32				0.328	0.00089	0.0167	-0.922	3.17625	8	2
CFHR4	complement factor H-related 4	1q32							-0.876666667	2.97589	7.333333333	1
CFHR5	complement factor H-related 5	1q31.3				0.321	0.00616	0.0439				1
TCN2	transcobalamin II	22q12.2	1.233	2.00E-05	7.00E-04							1
ITLN1	intelectin 1 (galactofuranose binding)	1q21.3	1.231	0.01684	0.0827							1
CFL2	cofilin 2 (muscle)	14q12				-0.365	0.00863	0.0528				1
CFP	complement factor properdin	Xp11.4	-0.398	0.00903	0.0545							1
MMP10	matrix metallopeptidase 10 (stromelysin 2)	11q22.3	1.231	0.00031	0.005				-0.748	2.516	5	2
CGB	chorionic gonadotropin, beta polypeptide	19q13.32	-0.312	0.03117	0.1238				0.807636364	3.614180909	6.818181818	2
CGB1	chorionic gonadotropin, beta polypeptide 1	19q13.32	-0.378	0.00399	0.0312							1
CGB5	chorionic gonadotropin, beta polypeptide 5	19q13.32							0.778363636	3.471609091	6.636363636	1
CGB7	chorionic gonadotropin, beta polypeptide 7	19q13.32							0.607	2.526	5	1
CGB8	chorionic gonadotropin, beta polypeptide 8	19q13.32							0.85275	3.8617825	7	1
RNASE1	ribonuclease, RNase A family, 1 (pancreatic)	14q11.2	1.229	0.00997	0.0583							1
CGNL1	cingulin-like 1	15q21.3				0.347	0.00289	0.0294				1
CH25H	cholesterol 25-hydroxylase	10q23	-1.05	0.00956	0.0566							1
CHAC1	ChaC, cation transport regulator homolog 1 (E. coli)	15q15.1	-1.513	1.00E-05	3.00E-04							1
CHAC2	ChaC, cation transport regulator homolog 2 (E. coli)	2p16							-0.866	2.75333	6	1
CHAD	chondroadherin	17q21.33	-0.548	0.01627	0.0808							1
CHADL	chondroadherin-like	22q13.2	-0.219	0.0492	0.1654	-0.452	1.00E-04	0.006533333				2
CHAF1A	chromatin assembly factor 1, subunit A (p150)	19p13.3				-0.4383	0.003316	0.02876				1
CHAF1B	chromatin assembly factor 1, subunit B (p60)	21q22.13				-0.377333333	0.00106	0.016666667				1
CHAT	choline O-acetyltransferase	10q11.2				-0.326	0.00117	0.019	0.807	3.097055	7.5	2
CHCHD10	coiled-coil-helix-coiled-coil-helix domain containing 10	22q11.23				-0.301	0.00341	0.0321				1
DGKD	diacylglycerol kinase, delta 130kDa	2q37.1	1.228	0.00599	0.0413							1
LACTB2	lactamase, beta 2	8q13.3	1.228	0.00015	0.0029							1
PI15	peptidase inhibitor 15	8q21.11	1.228	0.00374	0.0299	0.3045	0.01	0.05525	-0.812	3.376	5	3
ETV5	ets variant 5	3q28	1.227	0.00096	0.0114				-0.436	2.384	5	2
HSPH1	heat shock 105kDa/110kDa protein 1	13q12.3	1.225	0.00077	0.0098	0.321	0.00738	0.0452				2
CHD3	chromodomain helicase DNA binding protein 3	17p13.1				-0.3655	0.0017	0.021625				1
DLL4	delta-like 4 (Drosophila)	15q14	1.224	0	0							1
CHD5	In multiple Geneids					-0.378166667	0.001955	0.021583333				1
CHD6	chromodomain helicase DNA binding protein 6	20q12				0.352	0.001355455	0.016054545				1
C5AR1	complement component 5a receptor 1	19q13.3-q13.4	1.223	2.00E-05	6.00E-04							1
CHD8	chromodomain helicase DNA binding protein 8	14q11.2				-0.352	0.001503333	0.0192				1
CHD9	chromodomain helicase DNA binding protein 9	16q12.2	-0.672	0.00028	0.0046	-0.3545	0.001525	0.016475				2
TGFB2	transforming growth factor, beta 2	1q41	1.223	0.0094	0.056							1
RNFT2	ring finger protein, transmembrane 2	12q24.22	1.221	4.00E-05	0.0011							1
CHEK2	checkpoint kinase 2	22q12.1				-0.377714286	0.003094286	0.0228				1
CHERP	calcium homeostasis endoplasmic reticulum protein	19p13.1				-0.486666667	0.001246667	0.0169				1
CHES1	Data not found								0.654	2.75333	6	1
CHFR	In multiple Geneids		-0.435	0.02339	0.1025	-0.375	0.001303333	0.019				2
CHIA	chitinase, acidic	1p13.2							-0.935	2.98833	6	1
CHIC1	cysteine-rich hydrophobic domain 1	Xq13.2				0.4775	0.001055	0.01365				1
CHIC2	cysteine-rich hydrophobic domain 2	4q11							-0.967	2.718	5	1
CHID1	chitinase domain containing 1	11p15.5				-0.35975	0.0050375	0.03395				1
TDRKH	tudor and KH domain containing	1q21	1.22	0.00046	0.0068							1
CHKB-CPT1B	CHKB-CPT1B readthrough (non-protein coding)	22q13.33				-0.488	2.00E-05	0.0038				1
CHL1	cell adhesion molecule with homology to L1CAM (close homolog of L1)	3p26.1	-1.362	0.00028	0.0047				0.616	2.36667	6	2
CHM	choroideremia (Rab escort protein 1)	Xq21.2				0.366	0.016746667	0.062666667	-1.074	3.292	5	2
SCARB1	scavenger receptor class B, member 1	12q24.31	1.219	0.00017	0.0033							1
CHMP1A	charged multivesicular body protein 1A	16q24.3	-0.329	0.0187	0.0888	-0.4155	0.001705	0.0218				2
CHMP1B	charged multivesicular body protein 1B	18p11.21							-0.711	2.66625	8	1

CHMP2A	charged multivesicular body protein 2A	19q	-0.485	0.01297	0.0697							1
CHMP4A	charged multivesicular body protein 4A	14q12				-0.383	0.00074	0.0154				1
CHMP4C	charged multivesicular body protein 4C	8q21.13				0.349	0.00017	0.0082				1
CHMP5	charged multivesicular body protein 5	9p13.3				-0.334	0.00014	0.0075				1
CHMP6	charged multivesicular body protein 6	17q25.3	-0.326	0.00438	0.0333							1
E2F5	E2F transcription factor 5, p130-binding	8q21.2	1.218	0.00034	0.0053							1
HOXC9	homeobox C9	12q13.3	1.218	0	1.00E-04							1
CHODL	chondrolectin	21q11.2	-0.364	0.00157	0.0161							1
SNAI1	snail homolog 1 (Drosophila)	20q13.2	1.216	0	0							1
CHP	Data not found					-0.384	0.011515	0.055375				1
CHP1	calcineurin-like EF hand protein 1	15q13.3	-0.465	0.0199	0.0923							1
CIB2	calcium and integrin binding family member 2	15q24	1.215	0.00036	0.0057							1
GPRC5B	G protein-coupled receptor, family C, group 5, member B	16p12	1.214	0.01827	0.0874							1
CHRD	chordin	3q27				-0.337	0.0145	0.0707				1
CHRDL1	chordin-like 1	Xq23				0.385666667	0.002586667	0.024866667				1
CHRFAM7A	CHRNA7 (cholinergic receptor, nicotinic, alpha 7, exons 5-10) and FAM7A (fam	15q13.1				-0.415	0.001125	0.0172				1
CHRM1	cholinergic receptor, muscarinic 1	11q13				-0.3	0.03169	0.1123	0.489	2.504	5	2
CHRM2	cholinergic receptor, muscarinic 2	7q31-q35				-0.3625	0.000545	0.01075				1
ITGA11	integrin, alpha 11	15q23	1.212	0.01008	0.0588	0.35	0.00067	0.0147				2
CHRM5	cholinergic receptor, muscarinic 5	15q26				-0.333	0.0034	0.032				1
RABL2A	RAB, member of RAS oncogene family-like 2A	2q13	1.212	0.00948	0.0563							1
CHRNA10	cholinergic receptor, nicotinic, alpha 10 (neuronal)	11p15.5				-0.329	0.02154	0.0891				1
CHRNA2	cholinergic receptor, nicotinic, alpha 2 (neuronal)	8p21				-0.314	0.00398	0.0348				1
CHRNA3	cholinergic receptor, nicotinic, alpha 3 (neuronal)	15q24	-0.217	0.04141	0.1489							1
EPB41L4B	erythrocyte membrane protein band 4.1 like 4B	9q31-q32	1.211	0.00115	0.0129							1
CHRNA9	cholinergic receptor, nicotinic, alpha 9 (neuronal)	4p14				-0.393	0.00066	0.013466667				1
CHRNB1	cholinergic receptor, nicotinic, beta 1 (muscle)	17p13.1				-0.466666667	0.003156667	0.023866667				1
CHRNB3	cholinergic receptor, nicotinic, beta 3 (neuronal)	8p11.2				-0.324	0.034135	0.11725				1
CHRNB4	cholinergic receptor, nicotinic, beta 4 (neuronal)	15q24	-0.883	0.00012	0.0025	-0.311	0.00085	0.0163				2
CHRNE	cholinergic receptor, nicotinic, epsilon (muscle)	17p13.2				-0.447	0.00322	0.0311				1
SLC35D2	solute carrier family 35, member D2	9q22.32	1.211	1.00E-05	5.00E-04							1
TM6SF2	transmembrane 6 superfamily member 2	19p13.3-p12	1.211	0.00472	0.0351							1
CHST12	carbohydrate (chondroitin 4) sulfotransferase 12	7p22				-0.333	0.00226	0.0261				1
CHST15	carbohydrate (N-acetylgalactosamine 4-sulfate 6-O) sulfotransferase 15	10q26				-0.351	0.00331	0.0316				1
CHST3	carbohydrate (chondroitin 6) sulfotransferase 3	10q22.1				-0.331333333	0.003233333	0.025633333				1
CHST4	carbohydrate (N-acetylglucosamine 6-O) sulfotransferase 4	16q22.2							-0.953	2.926	5	1
TLN2	talin 2	15q15-q21	1.21	8.00E-05	0.0019							1
CHST6	carbohydrate (N-acetylglucosamine 6-O) sulfotransferase 6	16q22	-0.403	0.00981	0.0576	-0.384333333	0.004158333	0.032066667				2
CHST8	carbohydrate (N-acetylgalactosamine 4-0) sulfotransferase 8	19q13.1				-0.3398	0.00323	0.029				1
TPM1	tropomyosin 1 (alpha)	15q22.1	1.21	0.00152	0.0157							1
CHTF8	CTF8, chromosome transmission fidelity factor 8 homolog (S. cerevisiae)	16q22.1				-0.363	0.019345	0.07595				1
CDH6	cadherin 6, type 2, K-cadherin (fetal kidney)	5p13.3	1.207	0.00025	0.0043	0.372470588	0.004743529	0.034523529				2
CHUK	conserved helix-loop-helix ubiquitous kinase	10q24-q25				-0.329	0.00026	0.0098				1
PPP1R9A	protein phosphatase 1, regulatory subunit 9A	7q21.3	1.207	0.00559	0.0394	0.302	0.00763	0.0493				2
TRIM15	tripartite motif containing 15	6p21.3	1.207	0	1.00E-04							1
APOL1	apolipoprotein L, 1	22q13.1	1.206	0.00085	0.0105							1
CIB3	calcium and integrin binding family member 3	19p13.12	-0.26	0.02472	0.1062	-0.337	0.00545	0.041				2
CIC	In multiple Geneids					-0.37	0.00296	0.0298	0.579	2.61	5	2
CIDEA	cell death-inducing DFFA-like effector a	18p11.21	-0.512	0.00098	0.0115							1
CIDEB	cell death-inducing DFFA-like effector b	14q12	-0.995	0.00561	0.0395	-0.345	0.00042	0.0119				2
CIDECP	cell death-inducing DFFA-like effector c pseudogene	3p25.3	-0.322	0.01079	0.0615	-0.422	0.00121	0.0193				2
CILP	cartilage intermediate layer protein, nucleotide pyrophosphohydrolase	15q22				-0.328	0.00456	0.0373				1
CILP2	cartilage intermediate layer protein 2	19p13.11				-0.491	0.000255	0.0085				1
CINP	cyclin-dependent kinase 2 interacting protein	14q32.31				-0.446	0.00175	0.023				1
CIR	Data not found								-0.672	2.52	6	1
FNDC3A	fibronectin type III domain containing 3A	13q14.2	1.206	1.00E-05	3.00E-04	0.349	0.00104	0.0168				2
CIRBP	cold inducible RNA binding protein	19p13.3	-0.565	0.01469	0.0754				0.665	2.495	6	2
CIRBP-AS1	CIRBP antisense RNA 1 (non-protein coding)	19p13.3	-0.277	0.02643	0.1112							1
CIRH1A	In multiple Geneids					-0.415833333	0.002311667	0.023833333				1
CISH	cytokine inducible SH2-containing protein	3p21.3	-0.333	0.0063	0.0427							1
POTEM	POTE ankyrin domain family, member M	14q11.2	1.206	0.010265	0.0592							1
CITED2	Cbp/p300-interacting transactivator, with Glu/Asp-rich carboxy-terminal dom	6q23.3	-0.919	0.00167	0.0168							1
CITED4	Cbp/p300-interacting transactivator, with Glu/Asp-rich carboxy-terminal dom	1p34.2	-0.416	0.02716	0.1131							1
CIZ1	CDKN1A interacting zinc finger protein 1	9q34.1				-0.3775	0.014685	0.06425				1
CKAP4	cytoskeleton-associated protein 4	12q23.3	-0.483	0.00652	0.0436							1
TMEM97	transmembrane protein 97	17q11.2	1.206	0.00169	0.0169				-0.799	2.714	5	2
CKLF	chemokine-like factor	16q21				-0.335	0.00289	0.0294				1
CKM	creatine kinase, muscle	19q13.32	-0.265	0.03661	0.1376	-0.3365	0.003135	0.03035				2
CKMT2	creatine kinase, mitochondrial 2 (sarcomeric)	5q13.3				-0.347	0.000205	0.0076				1

ACOT9	acyl-CoA thioesterase 9	Xp22.11	1.205	0	1.00E-04							1
CATSPERB	catsper channel auxiliary subunit beta	14q32.12	1.205	0.01556	0.0784							1
CLASP2	cytoplasmic linker associated protein 2	3p22.3							-0.987	3.01667	6	1
CLCA2	chloride channel accessory 2	1p22.3							-0.724	2.79667	6	1
CLCA3P	chloride channel accessory 3, pseudogene	1p31-p22	-0.858	0	1.00E-04							1
CLCA4	chloride channel accessory 4	1p31-p22	-5.528	0	0							1
CLCC1	chloride channel CLIC-like 1	1p13.3				-0.3075	0.0055	0.041				1
CLCN1	chloride channel, voltage-sensitive 1	7q35				-0.302	0.01546	0.0734				1
DRAM1	DNA-damage regulated autophagy modulator 1	12q23.2	1.204	0.00107	0.0123							1
CLCN3	chloride channel, voltage-sensitive 3	4q33	-0.86	5.00E-05	0.0014							1
SLC29A3	solute carrier family 29 (nucleoside transporters), member 3	10q22.1	1.203	2.00E-05	6.00E-04							1
SNORD14E	small nucleolar RNA, C/D box 14E	11q23.3-q25	1.203	0.01444	0.0746							1
TAPBP	TAP binding protein (tapasin)	6p21.3	1.2015	8.50E-05	0.00195							1
SLC5A12	solute carrier family 5 (sodium/glucose cotransporter), member 12	11p14.2	1.201	0.00309	0.0262	0.309	0.00671	0.046	-0.969	3.056	5	3
CLDN1	claudin 1	3q28-q29				0.327333333	0.00538	0.034066667				1
CLDN10	claudin 10	13q31-q34	-1.122	0.02439	0.1053							1
CLDN11	claudin 11	3q26.2-q26.3							0.848	2.796	5	1
C1S	complement component 1, s subcomponent	12p13	1.2	0.01749	0.0848							1
CLDN14	claudin 14	21q22.3				-0.399608696	0.003046957	0.024152174				1
FUT8	fucosyltransferase 8 (alpha (1,6) fucosyltransferase)	14q24.3	1.198	0.00343	0.0281				-0.892	3.46	6	2
CLDN16	claudin 16	3q28				0.361	0.006465	0.0423				1
CLDN17	claudin 17	21q22.11							-0.857	3.37875	8	1
BCL2L15	BCL2-like 15	1p13.2	1.197	0.00233	0.0213							1
ETV4	ets variant 4	17q21	1.197	5.00E-05	0.0013							1
CLDN22	claudin 22	4q35.1	-0.289	0.00554	0.0392							1
IL1B	interleukin 1, beta	2q14	1.197	0.03143	0.1244							1
C11orf24	chromosome 11 open reading frame 24	11q13	1.196	1.00E-05	3.00E-04							1
NRP1	neuropilin 1	10p12	1.194	1.00E-05	4.00E-04							1
CLDN8	claudin 8	21q22.11							-0.77	2.86	6	1
CLDND1	claudin domain containing 1	3q12.1	-0.953	0.00028	0.0046							1
CLEC10A	C-type lectin domain family 10, member A	17p13.1	-0.493	0.01553	0.0783	-0.387	0.00139	0.0205				2
CLEC11A	C-type lectin domain family 11, member A	19q13.3							0.728	2.925	6	1
CLEC12A	C-type lectin domain family 12, member A	12p13.2							-0.973	3.59714	7	1
CLEC12B	C-type lectin domain family 12, member B	12p13.2	-0.209	0.02686	0.1122							1
CLEC16A	C-type lectin domain family 16, member A	16p13.13				-0.332	0.005	0.0392				1
CLEC17A	C-type lectin domain family 17, member A	19p13.12				-0.3945	0.00354625	0.029175				1
CLEC18A	C-type lectin domain family 18, member A	16q22.1				-0.4625	0.001135	0.01865				1
CLEC18B	C-type lectin domain family 18, member B	16q22.3	-0.271	0.00442	0.0335	-0.453	2.00E-04	0.0087				2
CLEC1B	C-type lectin domain family 1, member B	12p13.2							-0.695	2.9675	8	1
CLEC2A	C-type lectin domain family 2, member A	12p13.31				0.359	0.00011	0.0069				1
CLEC2B	C-type lectin domain family 2, member B	12p13-p12				0.301	0.00123	0.0194	-0.85	3.23125	8	2
CLEC2D	C-type lectin domain family 2, member D	12p13							-0.839	3.288	5	1
CLEC2L	C-type lectin domain family 2, member L	7q34				-0.356666667	0.005623333	0.0353				1
CLEC3A	C-type lectin domain family 3, member A	16q23	-0.301	0.00281	0.0244							1
CLEC3B	C-type lectin domain family 3, member B	3p22-p21.3	-0.208	0.04868	0.1644							1
CLEC4C	C-type lectin domain family 4, member C	12p13.2-p12.3	-0.252	0.03111	0.1237							1
CLEC4D	C-type lectin domain family 4, member D	12p13.31	-0.185	0.04242	0.1512							1
CLEC4F	C-type lectin domain family 4, member F	2p13.3	-0.286	0.01035	0.06							1
CLEC4G	C-type lectin domain family 4, member G	19p13.2				-0.5275	0.000625	0.0138				1
CLEC4M	C-type lectin domain family 4, member M	19p13							-0.913	3.306	5	1
CLEC5A	C-type lectin domain family 5, member A	7q33							-1.012	2.63333	6	1
CLEC6A	C-type lectin domain family 6, member A	12p13							-0.916	3.56556	9	1
CLEC7A	C-type lectin domain family 7, member A	12p13.2							-0.825	3.0475	8	1
CLEC9A	C-type lectin domain family 9, member A	12p13.2	-0.309	0.00107	0.0123							1
CLGN	calmegin	4q28.3-q31.1							-0.762	2.526	5	1
PDIA5	protein disulfide isomerase family A, member 5	3q21.1	1.194	0.00052	0.0074							1
CLIC3	chloride intracellular channel 3	9q34.3	-2.413	0	0							1
ANXA6	annexin A6	5q33.1	1.193	0.00427	0.0327							1
CRISPLD2	cysteine-rich secretory protein LCCL domain containing 2	16q24.1	1.193	0.0026	0.0231							1
CLIC6	chloride intracellular channel 6	21q22.12				-0.3105	0.0027175	0.0252	0.536	2.64667	6	2
CLINT1	clathrin interactor 1	5q33.3	-0.657	0.00365	0.0294	-0.3645	0.003905	0.02645				2
CLIP1	CAP-GLY domain containing linker protein 1	12q24.3	-1.176	0	2.00E-04	-0.361833333	0.005711667	0.03655				2
PDE8A	phosphodiesterase 8A	15q25.3	1.191	0	2.00E-04							1
CLIP3	CAP-GLY domain containing linker protein 3	19q13.12				-0.369333333	0.000956667	0.017033333				1
CLIP4	CAP-GLY domain containing linker protein family, member 4	2p23.2				0.329	0.000425	0.0109				1
IL2RG	interleukin 2 receptor, gamma	Xq13.1	1.189	0.0155	0.0782							1
CLK4	CDC-like kinase 4	5q35				-0.3205	0.001255	0.0183				1
CLLU1	chronic lymphocytic leukemia up-regulated 1	12q22							-0.741	2.96	8	1
CLLU1OS	chronic lymphocytic leukemia up-regulated 1 opposite strand	12q22	-0.195	0.02355	0.1029							1

PARM1	prostate androgen-regulated mucin-like protein 1	4q13.3-q21.3	1.189	0.0126	0.0684							1
SLC26A6	solute carrier family 26, member 6	3p21.3	1.189	0.00015	0.003							1
HPS3	In multiple Geneids		1.185	2.00E-05	6.00E-04				-0.929	2.712	5	2
CLN8	ceroid-lipofuscinosis, neuronal 8 (epilepsy, progressive with mental retardatio	8p23	-0.727	0.01329	0.0706							1
CLNK	cytokine-dependent hematopoietic cell linker	4p16.1	-0.194	0.04907	0.1652	-0.339777778	0.004786667	0.030022222				2
CLOCK	clock homolog (mouse)	4q12	-1.082	0.00028	0.0047	-0.3205	0.01316	0.06215				2
PLCE1	phospholipase C, epsilon 1	10q23	1.185	0.00337	0.0278				-0.858	2.68333	6	2
CLPP	ClpP caseinolytic peptidase, ATP-dependent, proteolytic subunit homolog (E. c	19p13.3	-0.235	0.02371	0.1033	-0.488	0.00446	0.0369				2
CLPTM1	cleft lip and palate associated transmembrane protein 1	19q13.3				-0.382333333	0.00382	0.030033333				1
VTCN1	V-set domain containing T cell activation inhibitor 1	1p13.1	1.185	0.00297	0.0254							1
CLRN2	clarin 2	4p15.32				-0.3345	0.002785	0.02785				1
DDAH1	dimethylarginine dimethylaminohydrolase 1	1p22	1.184	0.00227	0.021							1
PCDH18	protocadherin 18	4q31	1.184	0.00358	0.0289							1
CLSTN1	calsyntenin 1	1p36.22				0.39775	0.00272	0.025925				1
CLSTN2	calsyntenin 2	3q23				0.366117647	0.002331765	0.022847059				1
EPB41L2	erythrocyte membrane protein band 4.1-like 2	6q23	1.182	6.00E-05	0.0015							1
CLTA	clathrin, light chain A	9p13				-0.4154	0.000522	0.01092				1
CLTB	clathrin, light chain B	5q35	-1.046	0.0016	0.0163	-0.46025	0.0003875	0.010425				2
DDX60	DEAD (Asp-Glu-Ala-Asp) box polypeptide 60	4q32.3	1.179	0.01086	0.0618							1
TLR4	toll-like receptor 4	9q33.1	1.179	0.01293	0.0695							1
CLTCL1	clathrin, heavy chain-like 1	22q11.21	-0.345	0.01228	0.0672	-0.404823529	0.001195882	0.013294118				2
CLU	clusterin	8p21-p12				-0.448	0.00071	0.0151				1
CLUAP1	clusterin associated protein 1	16p13.3				-0.438	0.0117925	0.061775	0.706	2.846	5	2
CLUH	Data not found		-0.426	0.00551	0.039							1
SLC4A4	solute carrier family 4, sodium bicarbonate cotransporter, member 4	4q21	1.178	0.02983	0.1202							1
CLUL1	clusterin-like 1 (retinal)	18p11.32							-0.785	3.03	5	1
CLVS1	clavesin 1	8q12.3	-0.641	0.00548	0.0389							1
CLYBL	citrate lyase beta like	13q32	-0.406	0.02829	0.1161	-0.32	0.010413333	0.058466667				2
CMA1	chymase 1, mast cell	14q11.2							-0.756	2.732	5	1
CMAS	In multiple Geneids		-0.572	0.01377	0.0724							1
SP140L	SP140 nuclear body protein-like	2q37.1	1.178	0.00027	0.0045							1
CMIP	c-Maf inducing protein	16q23				-0.378307692	0.00155	0.020907692				1
CMPK2	cytidine monophosphate (UMP-CMP) kinase 2, mitochondrial	2p25.2				0.329	0.00159	0.0219				1
TSPAN13	tetraspanin 13	7p21.1	1.178	0.00172	0.0171							1
SLC16A10	solute carrier family 16, member 10 (aromatic amino acid transporter)	6q21-q22	1.176	0.02004	0.0928							1
CMTM2	CKLF-like MARVEL transmembrane domain containing 2	16q21				-0.304	0.0344	0.118				1
CMTM3	CKLF-like MARVEL transmembrane domain containing 3	16q21				-0.501	2.50E-05	0.00385				1
CMTM4	CKLF-like MARVEL transmembrane domain containing 4	16q21-q22.1				-0.3732	0.003767	0.02927				1
CMTM5	CKLF-like MARVEL transmembrane domain containing 5	14q11.2	-0.261	0.02258	0.1002							1
CMTM6	CKLF-like MARVEL transmembrane domain containing 6	3p22.3				-0.385	0.00012	0.0071				1
PRO2012	uncharacterized protein PRO2012	1q42.13	1.175	0.00135	0.0145							1
ZNF761	zinc finger protein 761	19q13.42	1.174	0.00102	0.0118							1
GYG2	glycogenin 2	Xp22.3	1.17	0.01325	0.0704							1
CMTR2	Data not found		-0.433	0.00783	0.0495							1
CMYA3	cardiomyopathy associated 3								-0.862	3.05857	7	1
CMYA5	In multiple Geneids					-0.349714286	0.002041429	0.021228571				1
CNBD1	cyclic nucleotide binding domain containing 1	8q21.3				0.33	0.000163333	0.007266667				1
CNDP1	carnosine dipeptidase 1 (metallopeptidase M20 family)	18q22.3				-0.364333333	0.00515	0.031933333				1
CNDP2	CNDP dipeptidase 2 (metallopeptidase M20 family)	18q22.3				-0.301	0.00254	0.0276				1
CNEP1R1	CTD nuclear envelope phosphatase 1 regulatory subunit 1	16q12.1	-0.407	0.00614	0.042							1
CNFN	cornifelin	19q13.2	-4.155	9.00E-05	0.0021							1
CNGA1	cyclic nucleotide gated channel alpha 1	4p12	-0.732	0.01995	0.08225							1
CNGA4	cyclic nucleotide gated channel alpha 4	11p15.4	-0.234	0.04426	0.1553							1
CNGB1	cyclic nucleotide gated channel beta 1	16q13	-0.208	0.02721	0.1132	-0.309	0.00068	0.0147				2
CNGB3	cyclic nucleotide gated channel beta 3	8q21.3				0.346333333	0.000266667	0.008533333	-0.974	2.78	6	2
CNKSR1	connector enhancer of kinase suppressor of Ras 1	1p36.11	-0.377	0.03045	0.122	-0.419	0.00346	0.0323				2
CNKSR2	connector enhancer of kinase suppressor of Ras 2	Xp22.12				0.355777778	0.009333333	0.051622222				1
CNKSR3	CNKSR family member 3	6q25.2	-0.94	0.00033	0.0053	-0.369666667	0.00084	0.0133				2
CNN1	calponin 1, basic, smooth muscle	19p13.2-p13.1				-0.469	0.00105	0.01765				1
CNN2	calponin 2	19p13.3				-0.375	0.00544	0.041				1
CNN3	calponin 3, acidic	1p22-p21	-1.129	9.00E-05	0.0021	-0.312	0.00083	0.0162				2
AEBP1	AE binding protein 1	7p13	1.169	0.01347	0.0712							1
M1AP	Data not found		1.164	0.00014	0.0028							1
ADAMTS12	ADAM metallopeptidase with thrombospondin type 1 motif, 12	5q35	1.163	0.00017	0.0032	0.378857143	0.007015476	0.043914286				2
CNOT1	In multiple Geneids		-0.697	0.00024	0.0042	-0.3475	0.002955	0.0223				2
CNOT10	CCR4-NOT transcription complex, subunit 10	3p22.3				-0.377333333	0.002253333	0.023016667				1
TMEM106A	transmembrane protein 106A	17q21.31	1.163	5.00E-05	0.0014							1
CNOT3	CCR4-NOT transcription complex, subunit 3	19q13.4				-0.339	0.003976667	0.032466667				1
CNOT4	CCR4-NOT transcription complex, subunit 4	7q22-qter	-0.303	0.01996	0.0926	-0.452	1.00E-05	0.003				2

CNOT6	In multiple Geneids					-0.3286	0.011958	0.0592				1
CNOT6L	CCR4-NOT transcription complex, subunit 6-like	4q13.3	-0.391	0.03023	0.1213							1
CNP	2',3'-cyclic nucleotide 3' phosphodiesterase	17q21	-0.582	0.00121	0.0134	-0.393	0.00768	0.040733333				2
CNPY1	canopy 1 homolog (zebrafish)	7q36.3	-0.335	0.00182	0.0179							1
HLTF	helicase-like transcription factor	3q25.1-q26.1	1.161	0.00047	0.0068	0.318	0.00141	0.0207				2
CXCL16	chemokine (C-X-C motif) ligand 16	17p13	1.16	0.00098	0.0115							1
CNPY4	canopy 4 homolog (zebrafish)	7q22.1				-0.376	0.02585	0.0993				1
CNR2	cannabinoid receptor 2 (macrophage)	1p36.11				-0.344	0.00733	0.0479				1
ACAA2	acetyl-CoA acyltransferase 2	18q21.1	1.159	4.00E-05	0.0011							1
CNST	consortin, connexin sorting protein	1q44	-0.371	0.02804	0.1153							1
CNTD1	cyclin N-terminal domain containing 1	17q21.31							0.923	3.034	5	1
CNTD2	cyclin N-terminal domain containing 2	19q13.2				-0.423	0.00334	0.0317				1
CNTFR	ciliary neurotrophic factor receptor	9p13				-0.411	0.001118333	0.011433333				1
CNTLN	centlein, centrosomal protein	9p22.2				-0.3605	0.006783333	0.03615				1
CNTN1	contactin 1	12q11-q12	-1.032	0.00914	0.0549							1
CNTN3	contactin 3 (plasmacytoma associated)	3p12.3							-1.014	3.16889	9	1
CNTN4	In multiple Geneids								-0.862	3.59	6	1
CNTN5	contactin 5	11q21-q22.2				0.382454545	0.002650909	0.024709091				1
CNTN6	contactin 6	3p26-p25							-0.768	3.39	8	1
ETNK1	ethanolamine kinase 1	12p12.1	1.158	1.00E-05	4.00E-04	0.352333333	0.00063	0.014233333				2
CNTNAP2	contactin associated protein-like 2	7q35				-0.385	0.007515714	0.0403				1
CNTNAP3	contactin associated protein-like 3	9p13.1	-1.030333333	0.006416667	0.0409	-0.314	0.03462	0.1185				2
CNTNAP4	contactin associated protein-like 4	16q23.1	-0.26	0.00539	0.0385							1
CNTNAP5	contactin associated protein-like 5	2q14.3				0.3507	0.00224	0.02045				1
CNTROB	centrobin, centrosomal BRCA2 interacting protein	17p13.1				-0.4015	0.001185	0.019				1
COA3	Data not found		-0.516	0.00238	0.0216							1
SFXN3	In multiple Geneids		1.158	0	1.00E-04							1
COASY	CoA synthase	17q12-q21							-0.627	2.436	5	1
COBL	cordon-bleu homolog (mouse)	7p12.1				0.362083333	0.0196675	0.080541667				1
COBLL1	COBL-like 1	2q24.3				0.36475	1.25E-05	0.0032				1
BIN1	bridging integrator 1	2q14	1.153	5.00E-05	0.0013							1
POFUT1	protein O-fucosyltransferase 1	20q11	1.153	4.00E-05	0.0011							1
SEMA3E	sema domain, immunoglobulin domain (Ig), short basic domain, secreted, (ser	7q21.11	1.151	0.00583	0.0406	0.372	0.003249444	0.027383333	-1.124	3.11833	6	3
COG4	component of oligomeric golgi complex 4	16q22.1				-0.349666667	0.006801667	0.043016667				1
COG5	component of oligomeric golgi complex 5	7q31				0.364	8.00E-05	0.006	-0.893	2.566	5	2
COG6	component of oligomeric golgi complex 6	13q14.11				0.304	0.00388	0.0343	-0.871	3.206	5	2
COG7	component of oligomeric golgi complex 7	16p12.2				-0.314	0.02956	0.1076				1
COG8	component of oligomeric golgi complex 8	16q22.1				-0.505	0.00042	0.012				1
TPX2	TPX2, microtubule-associated, homolog (Xenopus laevis)	20q11.2	1.151	0.01057	0.0609							1
COL11A1	collagen, type XI, alpha 1	1p21				0.31	0.00497	0.0391				1
COL11A2	collagen, type XI, alpha 2	6p21.3	-0.24	0.01331	0.0706							1
KCTD14	potassium channel tetramerisation domain containing 14	11q14.1	1.15	0.01811	0.0868							1
COL13A1	collagen, type XIII, alpha 1	10q22	-0.204	0.03841	0.1419							1
COL14A1	collagen, type XIV, alpha 1	8q23				0.349692308	0.009146923	0.051561538				1
MFHAS1	malignant fibrous histiocytoma amplified sequence 1	8p23.1	1.15	0.00345	0.0282							1
COL16A1	collagen, type XVI, alpha 1	1p35-p34				-0.374	0.00012	0.007				1
COL17A1	collagen, type XVII, alpha 1	10q24.3	-1.381	0.00015	0.003	-0.301	0.00411	0.0353				2
COL18A1	collagen, type XVIII, alpha 1	21q22.3				-0.364166667	0.000523333	0.010816667				1
PLOD1	procollagen-lysine, 2-oxoglutarate 5-dioxygenase 1	1p36.22	1.149	0	2.00E-04							1
GALC	galactosylceramidase	14q31	1.147	0.01399	0.0731							1
COL22A1	collagen, type XXII, alpha 1	8q24.3				0.326	0.00664	0.0457				1
COL23A1	collagen, type XXIII, alpha 1	5q35.3	-0.285	0.01842	0.0879	-0.397104167	0.000653125	0.011504167	0.989	2.526	5	3
COL25A1	collagen, type XXV, alpha 1	4q25				-0.384	0.00167	0.02065				1
LYN	v-yes-1 Yamaguchi sarcoma viral related oncogene homolog	8q13	1.147	0.00296	0.0253				-0.718	2.506	5	2
COL29A1	Data not found					0.344	0.002833333	0.0285				1
LURAP1L	leucine rich adaptor protein 1-like	9p23	1.146	0.00049	0.007							1
ITPR3	inositol 1,4,5-trisphosphate receptor, type 3	6p21	1.145	7.00E-05	0.0017							1
PRKAA1	protein kinase, AMP-activated, alpha 1 catalytic subunit	5p12	1.145	9.00E-05	0.002	0.37	0.04093	0.1314				2
COL4A3BP	collagen, type IV, alpha 3 (Goodpasture antigen) binding protein	5q13.3	-0.525	0.00098	0.0115	-0.322	0.00023	0.0092				2
COL4A5	collagen, type IV, alpha 5	Xq22				0.352333333	0.00208	0.021033333				1
COL4A6	collagen, type IV, alpha 6	Xq22				0.3522	0.001484	0.01878				1
PSMB9	proteasome (prosome, macropain) subunit, beta type, 9 (large multifunctiona	6p21.3	1.145	0.00041	0.0063							1
GEM	GTP binding protein overexpressed in skeletal muscle	8q13-q21	1.143	0.00974	0.0574							1
COL5A3	collagen, type V, alpha 3	19p13.2				-0.510333333	0.002411111	0.023511111				1
FOXP4	forkhead box P4	6p21.1	1.142	0.00047	0.0068	0.346	0.02626	0.1002				2
MFI2	antigen p97 (melanoma associated) identified by monoclonal antibodies 133.2	3q28-q29	1.142	0.00099	0.0116							1
MSX2	msh homeobox 2	5q35.2	1.142	0.00048	0.0069							1
COL6A5	collagen, type VI, alpha 5	3q22.1	-0.545	0.00205	0.0194							1
COL6A6	collagen, type VI, alpha 6	3q22.1				0.3155	0.00058	0.0128				1

COL7A1	collagen, type VII, alpha 1	3p21.1				-0.443	0.00137	0.0146				1
COL8A1	collagen, type VIII, alpha 1	3q12.3				0.387	0.04071	0.131				1
COL9A2	collagen, type IX, alpha 2	1p33-p32				-0.329	0.00021	0.0089				1
COLEC10	collectin sub-family member 10 (C-type lectin)	8q23-q24.1				0.378142857	0.004997143	0.0352	-0.99	3.432	5	2
COLEC12	collectin sub-family member 12	18pter-p11.3				-0.612	3.00E-05	0.0043				1
SNORA1	small nucleolar RNA, H/ACA box 1	11q21	1.142	0.02724	0.1132							1
COLQ	collagen-like tail subunit (single strand of homotrimer) of asymmetric acetylch	3p25							-0.881	2.96125	8	1
COMMD1	copper metabolism (Murr1) domain containing 1	2p15	-0.556	9.00E-05	0.002							1
ACPL2	acid phosphatase-like 2	3q23	1.139	1.00E-05	5.00E-04							1
HSP90B1	heat shock protein 90kDa beta (Grp94), member 1	12q24.2-q24.3	1.139	0	2.00E-04							1
COMMD8	COMM domain containing 8	4p12							-0.968	2.968	5	1
COMMD9	COMM domain containing 9	11p13	-0.27	0.02782	0.1147							1
COMT	catechol-O-methyltransferase	22q11.21	-0.583	0.00151	0.0157	-0.39075	9.00E-05	0.004575				2
COMTD1	catechol-O-methyltransferase domain containing 1	10q22.2	-0.316	0.01158	0.0646							1
CNPY3	canopy 3 homolog (zebrafish)	6pter-p12.1	1.138	9.00E-05	0.0021							1
COPB	Data not found								-0.944	2.416	5	1
LAMB3	laminin, beta 3	1q32	1.137	0.00032	0.0051	0.344	0.00021	0.009				2
GLB1L2	galactosidase, beta 1-like 2	11q25	1.135	0.00328	0.0273							1
COPE	coatomer protein complex, subunit epsilon	19p13.11				-0.4775	0.00057	0.01225				1
SCARNA4	small Cajal body-specific RNA 4	1q22	1.135	0.00809	0.0505							1
COPG2	coatomer protein complex, subunit gamma 2	7q32							-0.91	2.702	5	1
COPS3	COP9 constitutive photomorphogenic homolog subunit 3 (Arabidopsis)	17p11.2	-0.507	0.00097	0.0115	-0.424666667	0.00083	0.015133333				2
COPS4	COP9 constitutive photomorphogenic homolog subunit 4 (Arabidopsis)	4q21.22	-0.527	0.00028	0.0046	-0.330333333	0.01109	0.054366667				2
COPS7A	COP9 constitutive photomorphogenic homolog subunit 7A (Arabidopsis)	12p13.31	-0.415	0.00803	0.0503	-0.391	6.00E-05	0.0056				2
CARD11	caspase recruitment domain family, member 11	7p22	1.134	0.00055	0.0077							1
PTP4A3	protein tyrosine phosphatase type IVA, member 3	8q24.3	1.133	0.00055	0.0077							1
COPZ1	In multiple Geneids					-0.401	0.00098	0.0174				1
COPZ2	coatomer protein complex, subunit zeta 2	17q21.32				-0.325	0.00867	0.053				1
COQ2	coenzyme Q2 homolog, prenyltransferase (yeast)	4q21.23				-0.368	0.003325	0.0315				1
COQ4	coenzyme Q4 homolog (S. cerevisiae)	9q34.11	-0.522	0.00062	0.0084	-0.308	0.01727	0.0782				2
COQ5	coenzyme Q5 homolog, methyltransferase (S. cerevisiae)	12q24.31							-0.869	2.576	5	1
COQ6	coenzyme Q6 homolog, monooxygenase (S. cerevisiae)	14q24.3	-0.2865	0.003085	0.02595	-0.3765	0.00291	0.0241				2
COQ9	coenzyme Q9 homolog (S. cerevisiae)	16q21	-0.446	0.00145	0.0153							1
CORO2A	coronin, actin binding protein, 2A	9q22.3				-0.32975	0.017185	0.07665	0.94	2.88667	6	2
CORO2B	coronin, actin binding protein, 2B	15q23				-0.31	0.00318	0.0309				1
CORO6	coronin 6	17q11.2							0.882	3.16	5.75	1
CORO7	coronin 7	16p13.3				-0.454	0.011027143	0.053514286				1
SLC43A2	solute carrier family 43, member 2	17p13.3	1.133	3.00E-05	8.00E-04							1
COX10	COX10 homolog, cytochrome c oxidase assembly protein, heme A: farnesyltra	17p12	-0.403	0.00502	0.0366	-0.334	0.00196	0.0243				2
CA8	carbonic anhydrase VIII	8q11-q12	1.132	0.00542	0.0386							1
PLCH1	phospholipase C, eta 1	3q25.31	1.132	5.00E-05	0.0014	0.391	0.00013	0.0072				2
SPR	sepiapterin reductase (7,8-dihydrobiopterin:NADP+ oxidoreductase)	2p14-p12	1.132	6.00E-05	0.0015							1
GLIS3	GLIS family zinc finger 3	9p24.2	1.131	1.00E-04	0.0022				-0.937	2.82571	7	2
GNS	glucosamine (N-acetyl)-6-sulfatase	12q14	1.131	1.00E-05	3.00E-04							1
COX4I1	cytochrome c oxidase subunit IV isoform 1	16q24.1				-0.365	0.00293	0.0297				1
COX4NB	Data not found					-0.378	0.00542	0.0409				1
COX6A1	cytochrome c oxidase subunit VIa polypeptide 1	12q24.2				-0.327	0.00285	0.0292				1
COX6B1	cytochrome c oxidase subunit VIb polypeptide 1 (ubiquitous)	19q13.1				-0.3765	0.0011	0.0184				1
COX7A2	cytochrome c oxidase subunit VIIa polypeptide 2 (liver)	6q12							-0.926	3.05333	6	1
COX7B	cytochrome c oxidase subunit VIIb	Xq21.1							-0.732	2.45714	7	1
COX7C	cytochrome c oxidase subunit VIIc	5q14							-1.031	2.954	5	1
COX8A	cytochrome c oxidase subunit VIIIA (ubiquitous)	11q12-q13	-0.4	0.00595	0.0411							1
KCNH8	potassium voltage-gated channel, subfamily H (eag-related), member 8	3p24.3	1.131	9.00E-05	0.0021							1
CPA4	carboxypeptidase A4	7q32	-2.866	0	0	-0.325	0.00018	0.0084				2
CPA5	carboxypeptidase A5	7q32	-0.276	0.00574	0.0402	-0.319	0.002	0.0245				2
CPA6	carboxypeptidase A6	8q13.2				0.3245	0.00515	0.0395				1
CPAMD8	C3 and PZP-like, alpha-2-macroglobulin domain containing 8	19p13.11				-0.42108	0.0045688	0.03072				1
CPB1	carboxypeptidase B1 (tissue)	3q24				0.334333333	0.003076667	0.0278				1
CPB2	carboxypeptidase B2 (plasma)	13q14.11				0.312	0.00239	0.0268				1
RASSF6	Ras association (RalGDS/AF-6) domain family member 6	4q13.3	1.131	0.00505	0.0368				-0.549	2.5125	8	2
CPE	carboxypeptidase E	4q32.3				-0.3705	0.001105	0.0185				1
CPEB2	cytoplasmic polyadenylation element binding protein 2	4p15.33	-1.415	0	0							1
CPEB3	cytoplasmic polyadenylation element binding protein 3	10q23.32	-1.208	0	0	-0.349666667	0.001395833	0.016408333				2
CPEB4	cytoplasmic polyadenylation element binding protein 4	5q21	-1.073	2.00E-05	6.00E-04							1
CPLX1	complexin 1	4p16.3				-0.391285714	0.001838571	0.019714286	0.551	2.56429	7	2
CPLX2	complexin 2	5q35.2				-0.3546	0.000636	0.01356				1
CPLX3	complexin 3	15q24.1	-0.351	0.00757	0.0484							1
CPLX4	complexin 4	18q21.32							-0.933	3.38857	7	1
CPN1	carboxypeptidase N, polypeptide 1	10q24.2				-0.334	0.000778333	0.014				1

CPNE1	copine I	20q11.22							0.797	3.53714	7	1
CPNE2	In multiple Geneids					-0.408	0.000125	0.00715				1
CPNE3	copine III	8q21.3				0.334	5.00E-05	0.0052				1
CPNE4	In multiple Geneids					0.3302	0.003902	0.0311				1
CPNE6	copine VI (neuronal)	14q11.2				-0.308	0.00036	0.0112				1
CPNE9	copine family member IX	3p25.3				-0.333	0.00099	0.0176				1
CPO	carboxypeptidase O	2q33.3							-0.738	2.77	5	1
CPPED1	calcineurin-like phosphoesterase domain containing 1	16p13.12	-1.445	0.00169	0.0169	-0.313	0.0041	0.0353				2
CPS1	carbamoyl-phosphate synthase 1, mitochondrial	2q35				0.345375	4.00E-04	0.0099125	-0.751	2.69286	7	2
PLIN2	perilipin 2	9p22.1	1.128	0.00101	0.0118							1
CPSF2	cleavage and polyadenylation specific factor 2, 100kDa	14q31.1				-0.308	0.01054	0.0589				1
PRAME	In multiple Geneids		1.128	0.02771	0.1144							1
CPSF3L	cleavage and polyadenylation specific factor 3-like	1p36.33				-0.468	6.00E-05	0.0054	0.867	3.368	5	2
OLR1	oxidized low density lipoprotein (lectin-like) receptor 1	12p13.2-p12.3	1.127	0.00226	0.0209				-0.688	3.056	5	2
FSTL3	follistatin-like 3 (secreted glycoprotein)	19p13	1.126	0.00011	0.0024							1
CPSF7	cleavage and polyadenylation specific factor 7, 59kDa	11q12.2				-0.339	0.01504	0.0722				1
CPT1B	carnitine palmitoyltransferase 1B (muscle)	22q13.33				-0.488	2.00E-05	0.0038				1
GUCY1B3	guanylate cyclase 1, soluble, beta 3	4q31.3-q33	1.125	0.00163	0.0165							1
CPT2	carnitine palmitoyltransferase 2	1p32	-0.306	0.03189	0.1255							1
CPVL	carboxypeptidase, vitellogenic-like	7p15.1				0.329	0.00971	0.0563	-0.753	2.70857	7	2
IL6	interleukin 6 (interferon, beta 2)	7p21	1.125	0.01012	0.059							1
CPXM2	carboxypeptidase X (M14 family), member 2	10q26.13				-0.309	0.00197	0.0244				1
CPZ	carboxypeptidase Z	4p16.1				-0.455	0.000935	0.01465				1
CR1	complement component (3b/4b) receptor 1 (Knops blood group)	1q32				0.4262	0.000476	0.0107				1
CR1L	complement component (3b/4b) receptor 1-like	1q32.1				0.341	0.002235	0.0244				1
CR2	complement component (3d/Epstein Barr virus) receptor 2	1q32				0.322	0.00078	0.0158	-0.743	2.61	5	2
CRABP2	cellular retinoic acid binding protein 2	1q21.3	-2.858	8.00E-05	0.0019							1
CRADD	CASP2 and RIPK1 domain containing adaptor with death domain	12q21.33-q23.1							-0.831	2.742	5	1
CRAMP1L	Crm, cramped-like (Drosophila)	16p13.3				-0.425846154	0.009266154	0.049623077				1
CRB1	crumbs homolog 1 (Drosophila)	1q31-q32.1				0.361041667	0.00164125	0.019641667	-1.099	3.046	5	2
CRB3	crumbs homolog 3 (Drosophila)	19p13.3				-0.616	0.00051	0.013				1
CYP27A1	cytochrome P450, family 27, subfamily A, polypeptide 1	2q33-qter	1.124	0.00514	0.0372							1
CRCT1	cysteine-rich C-terminal 1	1q21	-5.051	3.00E-05	9.00E-04							1
SOCS3	suppressor of cytokine signaling 3	17q25.3	1.123	0.00137	0.0146							1
TBC1D30	TBC1 domain family, member 30	12q14.3	1.123	0.00016	0.0031							1
CREB3L2	cAMP responsive element binding protein 3-like 2	7q34				-0.3185	0.01444	0.0625				1
CREB3L3	cAMP responsive element binding protein 3-like 3	19p13.3				-0.421	0.002684	0.02652				1
UGT2A3	UDP glucuronosyltransferase 2 family, polypeptide A3	4q13.2	1.123	0.01452	0.0748				-0.871	3.39333	9	2
CREB5	cAMP responsive element binding protein 5	7p15.1				0.324615385	0.002436923	0.022284615	-0.6575	3.077085	7	2
CREBBP	CREB binding protein	16p13.3				-0.426096774	0.012439032	0.058754839				1
PRELID2	PRELI domain containing 2	5q32	1.122	1.00E-04	0.0021							1
PKP2	plakophilin 2	12p11	1.121	0.00038	0.0058							1
CRELD2	cysteine-rich with EGF-like domains 2	22q13.33				-0.343	2.00E-05	0.0036				1
CREM	cAMP responsive element modulator	10p11.21							-0.855	2.988833333	6	1
CRHBP	corticotropin releasing hormone binding protein	5q11.2-q13.3				-0.3175	0.001185	0.019				1
CRHR1	corticotropin releasing hormone receptor 1	17q12-q22	-0.207	0.02837	0.1163							1
CRIM1	In multiple Geneids					0.377545455	0.00354	0.025945455				1
CDC6	cell division cycle 6 homolog (S. cerevisiae)	17q21.3	1.12	0.00205	0.0194							1
CRIP2	cysteine-rich protein 2	14q32.3	-0.968	0.00013	0.0027							1
PDIA3	protein disulfide isomerase family A, member 3	15q15	1.119	1.00E-05	5.00E-04							1
CRISP1	cysteine-rich secretory protein 1	6p21.3				0.411	0.0033	0.0316	-0.992	2.82143	7	2
CRISP2	cysteine-rich secretory protein 2	6p12.3	-0.883	0.00023	0.0041							1
CRISP3	cysteine-rich secretory protein 3	6p12.3				0.372	0.003645	0.033	-0.859	3.43	5	2
CRISPLD1	cysteine-rich secretory protein LCCL domain containing 1	8q21.11				0.3295	0.002335	0.0256				1
FAM101A	family with sequence similarity 101, member A	12q24.31	1.118	0.01877	0.089							1
CRK	v-crk sarcoma virus CT10 oncogene homolog (avian)	17p13.3				-0.437	0.0239475	0.093875				1
CRKL	v-crk sarcoma virus CT10 oncogene homolog (avian)-like	22q11.21				-0.38975	0.002125	0.0188				1
CRKRS	Data not found					-0.419	0.01092	0.0601				1
CRLF1	cytokine receptor-like factor 1	19p12				-0.4125	0.00029	0.0097				1
SPAG5	sperm associated antigen 5	17q11.2	1.118	0.00384	0.0305							1
ASAP2	ArfGAP with SH3 domain, ankyrin repeat and PH domain 2	2p25	1.117	3.00E-05	8.00E-04	0.433	0.0023	0.0263				2
CRMP1	collapsin response mediator protein 1	4p16.1				-0.401666667	0.00082	0.0129	1.015	2.87	5	2
CRNN	cornulin	1q21	-5.341	0	1.00E-04							1
CROCC	ciliary rootlet coiled-coil, rootletin	1p36.13	-0.264	0.01633	0.0809	-0.52	0.00017	0.0082				2
CROT	carnitine O-octanoyltransferase	7q21.1	-1.757	2.00E-05	6.00E-04							1
CRP	C-reactive protein, pentraxin-related	1q21-q23							-0.681	2.68	5	1
CRSP3	Data not found								-0.901	2.73833	6	1
CRSP8	Data not found								-0.912	2.526	5	1
CRTAC1	cartilage acidic protein 1	10q22	-1.482	0.00011	0.0023	-0.377	0.00134	0.0202				2

CRTAP	cartilage associated protein	3p22.3				-0.38425	0.0004275	0.01035				1
LPGAT1	lysophosphatidylglycerol acyltransferase 1	1q32	1.116	3.00E-05	9.00E-04							1
B2M	beta-2-microglobulin	15q21-q22.2	1.115	0.00037	0.0057							1
ISLR	immunoglobulin superfamily containing leucine-rich repeat	15q23-q24	1.115	0.04212	0.1504							1
CRX	cone-rod homeobox	19q13.3	-0.211	0.03638	0.137							1
SMPDL3B	sphingomyelin phosphodiesterase, acid-like 3B	1p35.3	1.115	0.00033	0.0052							1
CRYAB	crystallin, alpha B	11q22.3-q23.1	-1.591	0.006	0.0414							1
CRYBA4	crystallin, beta A4	22q12.1				-0.333	0.00302	0.0301				1
CRYBB1	crystallin, beta B1	22q12.1	-0.214	0.03524	0.134	-0.37875	0.0006675	0.0112				2
CRYBB2	crystallin, beta B2	22q11.23				-0.38	7.00E-05	0.0058				1
CRYBB3	crystallin, beta B3	22q11.23				-0.413	0.00352	0.0326	0.79	3.06	5	2
CRYBG3	beta-gamma crystallin domain containing 3	3q11.2				0.302	0.01085	0.0599				1
CRYGC	crystallin, gamma C	2q33-q35	-0.269	0.01992	0.0924							1
CRYGS	crystallin, gamma S	3q25-qter				0.326	0.00036	0.0112				1
CRYM	crystallin, mu	16p	-0.296	0.02625	0.1107							1
CRYM-AS1	CRYM antisense RNA 1 (non-protein coding)	16p12.2	-0.329	0.01317	0.0701							1
ENO2	enolase 2 (gamma, neuronal)	12p13	1.113	0.00037	0.0057							1
CRYZL1	crystallin, zeta (quinone reductase)-like 1	21q21.3				-0.4075	0.00285	0.0288				1
CS	citrate synthase	12q13.2	-0.394	0.00144	0.0152							1
CSAD	cysteine sulfinic acid decarboxylase	12q13.11-q14.3				-0.435	5.00E-05	0.0051				1
CSDA	cold shock domain protein A	12p13.1				0.321	0.00403	0.035				1
CSDC2	cold shock domain containing C2, RNA binding	22q13.2				-0.480333333	0.000233333	0.008633333				1
CSDE1	cold shock domain containing E1, RNA-binding	1p22				-0.42	0.00151	0.0214				1
CSF1	colony stimulating factor 1 (macrophage)	1p21-p13				-0.404	0.00099	0.0176				1
CSF1R	colony stimulating factor 1 receptor	5q32				-0.3425	0.0013725	0.01875				1
CSF2RA	colony stimulating factor 2 receptor, alpha, low-affinity (granulocyte-macroph	Xp22.32 and Yp11.3				-0.534	0.003323077	0.022838462	1.0196	3.875	6.8	2
CSF2RB	colony stimulating factor 2 receptor, beta, low-affinity (granulocyte-macropha	22q13.1				-0.354333333	0.00532	0.028766667				1
SNORD14C	small nucleolar RNA, C/D box 14C	11q23.3-q25	1.11	0.00197	0.0189							1
CSGALNACT1	chondroitin sulfate N-acetylgalactosaminyltransferase 1	8p21.3				0.329	0.00317	0.0309				1
IQGAP3	IQ motif containing GTPase activating protein 3	1q21.3	1.109	0.00074	0.0095							1
CSH1	chorionic somatomammotropin hormone 1 (placental lactogen)	17q24.2	-0.183	0.0415	0.1491				0.738	2.89333	6	2
CSH2	chorionic somatomammotropin hormone 2	17q24.2							0.66225	3.02476	6.75	1
CSHL1	chorionic somatomammotropin hormone-like 1	17q24.2							0.622	2.92833	6	1
CSK	c-src tyrosine kinase	15q24.1	-0.805	0.00014	0.0028	-0.312	0.02233	0.091				2
CSMD1	CUB and Sushi multiple domains 1	8p23.2				0.3515	0.023220625	0.08945				1
CSMD2	In multiple Geneids					0.333	0.00234	0.0266				1
CSMD3	In multiple Geneids					0.394883436	0.005789632	0.035355215				1
CSN1S1	casein alpha s1	4q21.1							-0.933	3.41778	9	1
CSN3	casein kappa	4q21.1	-0.249	0.01593	0.0797							1
CSNK1A1	casein kinase 1, alpha 1	5q32	-0.774	0.00029	0.0048	-0.4254	0.003018	0.02092				2
CSNK1E	casein kinase 1, epsilon	22q13.1				-0.484142857	0.000547143	0.009185714				1
CSNK1G1	casein kinase 1, gamma 1	15q22.1-q22.31	-0.443	0.00259	0.023	-0.303	0.00276	0.0288				2
CSNK1G2	casein kinase 1, gamma 2	19p13.3				-0.439	0.00212	0.020466667				1
CSNK1G3	casein kinase 1, gamma 3	5q23							-1.137	3.146	5	1
SLC3A1	solute carrier family 3 (cystine, dibasic and neutral amino acid transporters, ac	2p16.3	1.109	0.00048	0.0069							1
CSPG5	chondroitin sulfate proteoglycan 5 (neuroglycan C)	3p21.3				-0.447	2.00E-04	0.0088				1
CSRNP1	cysteine-serine-rich nuclear protein 1	3p22				-0.415	3.00E-05	0.004				1
CSRNP2	cysteine-serine-rich nuclear protein 2	12q13.11-q13.12				-0.39	0.000465	0.0122				1
CSRNP3	cysteine-serine-rich nuclear protein 3	2q24.3				0.324	0.000565	0.0106				1
CSRP2	cysteine and glycine-rich protein 2	12q21.1	-0.642	0.00141	0.0149							1
ST3GAL2	ST3 beta-galactoside alpha-2,3-sialyltransferase 2	16q22.1	1.108	0	2.00E-04							1
CST11	cystatin 11	20p11.21	-0.339	0.00803	0.0503							1
CST13P	Data not found		-0.228	0.03366	0.1298							1
FOXM1	forkhead box M1	12p13	1.107	0.0016	0.0163							1
SLC2A14	In multiple Geneids		1.105	0.00071	0.0092							1
AP2B1	adaptor-related protein complex 2, beta 1 subunit	17q11.2-q12	1.103	1.00E-05	3.00E-04							1
CST6	cystatin E/M	11q13	-0.426	0.04562	0.1583							1
CST8	cystatin 8 (cystatin-related epididymal specific)	20p11.21				0.401	0.00018	0.0083				1
CSTA	cystatin A (stefin A)	3q21	-4.843	1.00E-05	5.00E-04							1
CSTB	cystatin B (stefin B)	21q22.3	-1.305	5.00E-05	0.0013	-0.352	0.00432	0.0363				2
CSTF2	cleavage stimulation factor, 3' pre-RNA, subunit 2, 64kDa	Xq22.1	-0.534	0.048	0.163							1
BMP2	bone morphogenetic protein 2	20p12	1.102	0.00022	0.0039	0.343	0.007136667	0.042066667				2
CSTL1	cystatin-like 1	20p11.21							0.926	2.44	5	1
CT45A1	cancer/testis antigen family 45, member A1	Xq26.3				-0.788666667	2.00E-05	0.0034				1
CT45A2	cancer/testis antigen family 45, member A2	Xq26.3				-0.844666667	3.33E-06	0.001666667				1
CT45A3	cancer/testis antigen family 45, member A3	Xq26.3				-0.638666667	0.00119	0.0129				1
CT45A4	cancer/testis antigen family 45, member A4	Xq26.3				-0.773133333	0.000326	0.005993333				1
CT45A5	cancer/testis antigen family 45, member A5	Xq26.3				-0.704	1.00E-05	0.0027				1
CT45A6	cancer/testis antigen family 45, member A6	Xq26.3				-0.692	6.67E-06	0.002333333				1

CT47A6	cancer/testis antigen family 47, member A6	Xq24	-0.974	0.00012	0.0026							1
CTAGE1	cutaneous T-cell lymphoma-associated antigen 1	18p11.2							0.947	3.496	5	1
CTAGE5	CTAGE family, member 5	14q13.3							-0.7374	2.749004	8	1
CTAGE7P	CTAGE family, member 7, pseudogene	10q26.3	-0.286	0.00148	0.0154							1
CTBP1	C-terminal binding protein 1	4p16				-0.329	0.00401	0.0349				1
FOLR1	folate receptor 1 (adult)	11q13.3-q14.1	1.102	0.04585	0.1587							1
CTCF	CCCTC-binding factor (zinc finger protein)	16q21-q22.3				-0.329545455	0.006386364	0.042				1
CTCFL	CCCTC-binding factor (zinc finger protein)-like	20q13.31				0.3672	0.021556	0.08628				1
CTDP1	CTD (carboxy-terminal domain, RNA polymerase II, polypeptide A) phosphatas	18q23				-0.391	0.007306	0.04202				1
CTDSP1	CTD (carboxy-terminal domain, RNA polymerase II, polypeptide A) small phos	2q35				-0.309	0.0081	0.051				1
CTDSP2	CTD (carboxy-terminal domain, RNA polymerase II, polypeptide A) small phos	12q14.1				-0.401	0.001065	0.0178				1
CTDSPL2	CTD (carboxy-terminal domain, RNA polymerase II, polypeptide A) small phos	15q15.3-q21.1				-0.319	0.02564	0.0988				1
ASPHD1	aspartate beta-hydroxylase domain containing 1	16p11.2	1.1	0.00418	0.0323							1
LYPD6B	LY6/PLAUR domain containing 6B	2q23.2	1.1	0.01892	0.0895	0.336625	0.00028375	0.0084875				2
CTIF	CBP80/20-dependent translation initiation factor	18q21.1	-0.645	0.00309	0.0262							1
CTLA4	cytotoxic T-lymphocyte-associated protein 4	2q33							-0.83	2.87857	7	1
CTNNA1	catenin (cadherin-associated protein), alpha 1, 102kDa	5q31.2	-0.485	0.00146	0.0153	-0.35625	0.0046925	0.034558333				2
CTNNA2	catenin (cadherin-associated protein), alpha 2	2p12-p11.1				0.376615385	0.001152564	0.015166667				1
CTNNA3	In multiple Geneids					0.572	0	0.0021	-0.85	2.96667	6	2
CD248	CD248 molecule, endosialin	11q13	1.099	0.00015	0.0029							1
CTNNBIP1	catenin, beta interacting protein 1	1p36.22	-1.399	0	0	-0.395923077	0.008096923	0.045207692				2
FCGRT	Fc fragment of IgG, receptor, transporter, alpha	19q13.3	1.099	0	0							1
CTNND1	catenin (cadherin-associated protein), delta 1	11q11	-0.567	0.00055	0.0076							1
CTNND2	catenin (cadherin-associated protein), delta 2 (neural plakophilin-related arm-	5p15.2				0.396754098	0.003592377	0.027837705				1
CTNS	cystinosin, lysosomal cystine transporter	17p13				-0.412333333	0.005546667	0.031233333				1
FAM26E	family with sequence similarity 26, member E	6q22.1	1.098	0.00799	0.0501							1
CTR9	Ctr9, Paf1/RNA polymerase II complex component, homolog (S. cerevisiae)	11p15.3				-0.307	0.00502	0.0393				1
CTRB2	In multiple Geneids		-0.317	0.0108	0.0616							1
CTRC	chymotrypsin C (caldecrin)	1p36.21	-0.24	0.04632	0.1596							1
CTRL	chymotrypsin-like	16q22.1				-0.49	0.00051	0.013				1
NR4A3	nuclear receptor subfamily 4, group A, member 3	9q22	1.098	0.00609	0.0417							1
CTSD	cathepsin D	11p15.5							0.573	2.834	5	1
SEC16B	SEC16 homolog B (S. cerevisiae)	1q25.2	1.098	0.00072	0.0093							1
B3GNT7	UDP-GlcNAc:betaGal beta-1,3-N-acetylglucosaminyltransferase 7	2q37.1	1.097	0.00149	0.0155							1
DPYSL2	dihydropyrimidinase-like 2	8p22-p21	1.096	0.00082	0.0102							1
SHANK2	In multiple Geneids		1.096	0.00016	0.0032							1
UGGT2	UDP-glucose glycoprotein glucosyltransferase 2	13q32.1	1.096	0	2.00E-04	0.328666667	0.014936667	0.070633333				2
CBLB	Cbl proto-oncogene, E3 ubiquitin protein ligase B	3q13.11	1.095	0	1.00E-04							1
CTSV	Data not found		-0.714	0.04218	0.1506							1
CTSW	cathepsin W	11q13.1	-0.481	0.00896	0.0542							1
SLC5A9	solute carrier family 5 (sodium/glucose cotransporter), member 9	1p33	1.092	0.01528	0.0776							1
CTTNBP2	cortactin binding protein 2	7q31	-1.9	1.00E-04	0.0021							1
CTTNBP2NL	CTTNBP2 N-terminal like	1p13.2	-0.754	0.0031	0.0262	-0.3095	0.003255	0.03125				2
CTXN3	In multiple Geneids		-0.238	0.01497	0.0765							1
CUBN	cubilin (intrinsic factor-cobalamin receptor)	10p12.31							-0.794	2.87	6	1
CUEDC2	CUE domain containing 2	10q24.32				-0.3245	0.000315	0.01				1
CUGBP1	Data not found								-0.776	2.71	9	1
CUL1	cullin 1	7q36.1	-0.297	0.02854	0.1167	-0.377333333	0.01122	0.0534				2
CUL2	cullin 2	10p11.21				0.553	0.00609	0.0436				1
CUL3	cullin 3	2q36.2	-0.569	0.00145	0.0152							1
CUL4B	cullin 4B	Xq23	-0.366	0.04991	0.1669							1
CUL7	cullin 7	6p21.1							0.986	3.071	5	1
BLM	Bloom syndrome, RecQ helicase-like	15q26.1	1.091	0.00136	0.0146							1
CUTL1	Data not found								-0.841	2.52333	6	1
CUX1	cut-like homeobox 1	7q22.1				-0.401210526	0.005803684	0.037802632				1
CUX2	cut-like homeobox 2	12q24.12	-0.339	0.00341	0.0281	-0.353583333	0.002110833	0.019525				2
CUZD1	CUB and zona pellucida-like domains 1	10q26.13							-0.937	3.086	5	1
CWC22	CWC22 spliceosome-associated protein homolog (S. cerevisiae)	2q31.3				0.326	0.00012	0.007				1
CWF19L1	CWF19-like 1, cell cycle control (S. pombe)	10q24.31				-0.319333333	0.000823333	0.014166667				1
IGF2BP3	insulin-like growth factor 2 mRNA binding protein 3	7p11	1.091	0.01764	0.0853							1
CWH43	cell wall biogenesis 43 C-terminal homolog (S. cerevisiae)	4p11	-3.677	0	0	-0.302	0.00485	0.0386				2
CX36	Data not found								0.542	2.376	5	1
CX3CL1	chemokine (C-X3-C motif) ligand 1	16q13				-0.426	2.00E-04	0.0087				1
CXADR	coxsackie virus and adenovirus receptor	21q21.1				-0.4305	0.00155	0.0171				1
CXADRP2	coxsackie virus and adenovirus receptor pseudogene 2	15q11.2				-0.3595	0.02009	0.07625				1
NEB	nebulin	2q22	1.091	0.00177	0.0175	0.316	4.00E-04	0.0117	-0.822	3.07	5	3
CXCL10	chemokine (C-X-C motif) ligand 10	4q21							-0.911	3.60125	8	1
CXCL13	chemokine (C-X-C motif) ligand 13	4q21							-0.778	2.87	8	1
GNA12	guanine nucleotide binding protein (G protein) alpha 12	7p22.2	1.088	0	0							1

CXCL17	chemokine (C-X-C motif) ligand 17	19q13.2	-2.708	0	0							1
RPL39L	ribosomal protein L39-like	3q27	1.088	0.00055	0.0077							1
SSFA2	sperm specific antigen 2	2q31.3	1.088	9.00E-05	0.002	0.309	0.00015	0.0077				2
CXCR2	chemokine (C-X-C motif) receptor 2	2q35	-2.427	0	1.00E-04							1
GJB7	gap junction protein, beta 7, 25kDa	6q15	1.087	0.00044	0.0066							1
CXorf1	chromosome X open reading frame 1	Xq27.3							-0.952	3.69375	8	1
CXorf27	chromosome X open reading frame 27	Xp21.1	-0.244	0.03436	0.1316							1
CXorf30	In multiple Geneids					0.353333333	0.005916667	0.032566667				1
CXorf36	chromosome X open reading frame 36	Xp11.3				0.344	0.00122	0.0194				1
GK	glycerol kinase	Xp21.3	1.087	0.00809	0.0505							1
CXorf40B	chromosome X open reading frame 40B	Xq28							0.8	2.76	5	1
CXorf48	chromosome X open reading frame 48	Xq26.3				0.307	0.03551	0.1204				1
IFI35	interferon-induced protein 35	17q21	1.087	0.00093	0.0112							1
CXorf58	chromosome X open reading frame 58	Xp22.11	-0.317	0.00566	0.0397							1
CXorf59	chromosome X open reading frame 59	Xp21.1				0.376	0.00045	0.0123				1
TREM1	triggering receptor expressed on myeloid cells 1	6p21.1	1.086	5.00E-04	0.0072							1
CXorf64	chromosome X open reading frame 64	Xq25	-0.249	0.04006	0.1457							1
CXorf9	chromosome X open reading frame 9	Xq26							0.86	3.52857	7	1
CXXC1	CXXC finger protein 1	18q12				-0.308	0.00399	0.0348				1
CXXC11	CXXC finger protein 11	2q37.3	-0.248	0.01014	0.059							1
CXXC5	CXXC finger protein 5	5q31.2				-0.403666667	0.000223333	0.0079				1
CYB561D1	cytochrome b-561 domain containing 1	1p13.3	-0.41	0.00084	0.0104							1
DCDC2	doublecortin domain containing 2	6p22.1	1.085	0.02139	0.0971	1.6985	0.00028	0.0076				2
CYB5D2	cytochrome b5 domain containing 2	17p13.2	-0.329	0.01806	0.0867	-0.385666667	0.004556667	0.031766667				2
CYB5R1	cytochrome b5 reductase 1	1q32.1	-0.726	0.00043	0.0065							1
CYB5R2	cytochrome b5 reductase 2	11p15.4	-0.766	0.01945	0.0911							1
CYB5R3	cytochrome b5 reductase 3	22q13.2-q13.31				-0.47925	0.001185	0.015425				1
CYB5R4	cytochrome b5 reductase 4	6pter-q22.33							-0.759	3.05	6	1
CYB5RL	cytochrome b5 reductase-like	1p32.3				-0.3705	0.004295	0.03325				1
ITGB3	integrin, beta 3 (platelet glycoprotein IIIa, antigen CD61)	17q21.32	1.084	1.00E-05	3.00E-04							1
SLC39A1	solute carrier family 39 (zinc transporter), member 1	1q21	1.083	3.00E-05	9.00E-04							1
CYFIP1	cytoplasmic FMR1 interacting protein 1	15q11				-0.3775	0.047115	0.1434	0.893	3.63571	7	2
CYFIP2	cytoplasmic FMR1 interacting protein 2	5q33.3				-0.373833333	0.000993333	0.01115				1
CHRNA5	cholinergic receptor, nicotinic, alpha 5 (neuronal)	15q24	1.08	0.00058	0.008							1
CYLC2	cylicin, basic protein of sperm head cytoskeleton 2	9q31.1							-1.02	3.85	9	1
CYLD	cylindromatosis (turban tumor syndrome)	16q12.1	-0.454	0.02802	0.1153							1
CYorf14	Data not found					-0.394333333	0.034036667	0.116333333				1
CYorf15A	Data not found					-0.4452	0.017354	0.07612				1
CYorf15B	chromosome Y open reading frame 15B	Yq11.222							-0.869	3.23125	8	1
CYorf16	Data not found					-0.748	0.00131	0.0196				1
CYP11A1	cytochrome P450, family 11, subfamily A, polypeptide 1	15q23-q24	-1.31	1.00E-05	3.00E-04							1
CYP17A1	cytochrome P450, family 17, subfamily A, polypeptide 1	10q24.3				-0.481	2.00E-05	0.0039				1
CYP1A1	cytochrome P450, family 1, subfamily A, polypeptide 1	15q24.1	-0.452	0.02572	0.1093							1
CYP1B1-AS1	CYP1B1 antisense RNA 1 (non-protein coding)	2p22.2	-0.269	0.02594	0.1099							1
CYP21A2	cytochrome P450, family 21, subfamily A, polypeptide 2	6p21.3							0.95	3.51	5	1
CYP24A1	cytochrome P450, family 24, subfamily A, polypeptide 1	20q13				0.449	0.025188571	0.094085714				1
CYP26A1	cytochrome P450, family 26, subfamily A, polypeptide 1	10q23-q24				-0.337	1.00E-05	0.0028				1
CYP26B1	cytochrome P450, family 26, subfamily B, polypeptide 1	2p13.2							1.014	3.76667	6	1
SYT11	synaptotagmin XI	1q21.2	1.079	0.00226	0.0209							1
CYP27B1	cytochrome P450, family 27, subfamily B, polypeptide 1	12q13.1-q13.3				-0.367	0.0015	0.0213				1
CYP2A13	cytochrome P450, family 2, subfamily A, polypeptide 13	19q13.2				-0.363	0.04615	0.1416				1
CYP2A7	cytochrome P450, family 2, subfamily A, polypeptide 7	19q13.2	-0.247	0.04552	0.1581							1
FILIP1L	filamin A interacting protein 1-like	3q12.1	1.078	0.00589	0.0408							1
ICA1	islet cell autoantigen 1, 69kDa	7p22	1.076	2.00E-05	6.00E-04	0.3465	0.001735	0.019375				2
CYP2C18	cytochrome P450, family 2, subfamily C, polypeptide 18	10q24	-2.144	0.00049	0.007							1
CYP2C19	In multiple Geneids								-0.831	3.14429	7	1
CYP2C8	cytochrome P450, family 2, subfamily C, polypeptide 8	10q23.33							-0.857	3.09556	9	1
CYP2C9	cytochrome P450, family 2, subfamily C, polypeptide 9	10q24							-0.851	3.08167	6	1
CYP2D7P1	cytochrome P450, family 2, subfamily D, polypeptide 7 pseudogene 1	22q13				-0.666	1.00E-05	0.0034				1
CYP2E1	cytochrome P450, family 2, subfamily E, polypeptide 1	10q24.3-qter	-1.725	2.00E-05	6.00E-04							1
CYP2F1	cytochrome P450, family 2, subfamily F, polypeptide 1	19q13.2	-0.5	0.00305	0.0259	-0.313	0.00085	0.0163				2
CYP2J2	cytochrome P450, family 2, subfamily J, polypeptide 2	1p31.3-p31.2							-0.632	2.668	5	1
CYP2R1	In multiple Geneids		-0.613	0.00041	0.0062							1
CYP2S1	cytochrome P450, family 2, subfamily S, polypeptide 1	19q13.1				-0.434	0.000715	0.0141				1
CYP39A1	cytochrome P450, family 39, subfamily A, polypeptide 1	6p21.1-p11.2				0.301	0.01263	0.0653				1
BMP8A	bone morphogenetic protein 8a	1p34.3	1.075	3.00E-05	0.001							1
FMNL2	formin-like 2	2q23.3	1.073	8.00E-05	0.0018	0.334	0.00076	0.0156				2
CYP46A1	cytochrome P450, family 46, subfamily A, polypeptide 1	14q32.1				-0.367	0.0048225	0.031375				1
CYP4A11	cytochrome P450, family 4, subfamily A, polypeptide 11	1p33							-0.877	2.98667	6	1

CYP4A22	cytochrome P450, family 4, subfamily A, polypeptide 22	1p33	-0.349	0.01012	0.059							1
CYP4B1	cytochrome P450, family 4, subfamily B, polypeptide 1	1p34-p12							-0.802	2.715	6	1
CYP4F11	cytochrome P450, family 4, subfamily F, polypeptide 11	19p13.1				-0.393	0.00103	0.0179				1
CYP4F2	cytochrome P450, family 4, subfamily F, polypeptide 2	19p13.12				-0.375	0.00121	0.0193				1
CYP4F22	cytochrome P450, family 4, subfamily F, polypeptide 22	19p13.12	-3.066	0	1.00E-04	-0.3718	0.002228	0.02556				2
CYP4F3	cytochrome P450, family 4, subfamily F, polypeptide 3	19p13.2				-0.364	0.00083	0.0162				1
FOXA2	forkhead box A2	20p11	1.073	0.00106	0.0122							1
CYP4V2	cytochrome P450, family 4, subfamily V, polypeptide 2	4q35.2				-0.348666667	0.016273333	0.074966667				1
CYP4X1	cytochrome P450, family 4, subfamily X, polypeptide 1	1p33	-2.576	0	0							1
CYP4Z1	cytochrome P450, family 4, subfamily Z, polypeptide 1	1p33	-0.716	0.00016	0.0032							1
CYP7A1	cytochrome P450, family 7, subfamily A, polypeptide 1	8q11-q12							-0.892	3.06	8	1
CYP7B1	cytochrome P450, family 7, subfamily B, polypeptide 1	8q21.3				0.367214286	0.003877143	0.02745				1
ABCC3	ATP-binding cassette, sub-family C (CFTR/MRP), member 3	17q22	1.072	0.00235	0.0215							1
CYSLTR1	cysteinyl leukotriene receptor 1	Xq13.2-q21.1							-0.793	2.49	5	1
ARMC9	armadillo repeat containing 9	2q37.1	1.072	8.00E-05	0.0019							1
CYTH1	cytohesin 1	17q25	-0.545	0.00211	0.0199							1
CYTH2	cytohesin 2	19q13.33				-0.4435	0.001005	0.0169				1
CYTH3	cytohesin 3	7p22.1				-0.318	0.00485	0.0386				1
CYTH4	cytohesin 4	22q12.3-q13.1				-0.416666667	0.000749167	0.01035				1
CYTSA	Data not found					-0.350857143	0.001352143	0.012721429				1
CYTSB	Data not found					-0.3302	0.00888	0.0507				1
CYYR1	In multiple Geneids								-0.825	2.42429	7	1
D21S2056E	Data not found								0.817	3.51286	7	1
D4S234E	DNA segment on chromosome 4 (unique) 234 expressed sequence	4p16.3				-0.326	0.004646667	0.036333333				1
DAAM1	dishevelled associated activator of morphogenesis 1	14q23.1	-1.259	0	2.00E-04							1
DAAM2	dishevelled associated activator of morphogenesis 2	6p21.2				0.339	0.010035	0.05685				1
DAB1	disabled homolog 1 (Drosophila)	1p32-p31				0.364	0.01821	0.0807				1
ELOVL7	ELOVL fatty acid elongase 7	5q12.1	1.072	0.00041	0.0062							1
DAB2IP	DAB2 interacting protein	9q33.1-q33.3				-0.324	0.00508	0.0395				1
PTPRG	protein tyrosine phosphatase, receptor type, G	3p21-p14	1.072	0	1.00E-04	0.4415	0.00164	0.02195				2
DACH2	dachshund homolog 2 (Drosophila)	Xq21.3				0.3728125	0.001243125	0.01598125				1
MSI2	musashi homolog 2 (Drosophila)	17q22	1.071	1.00E-05	4.00E-04							1
DACT3	dapper, antagonist of beta-catenin, homolog 3 (Xenopus laevis)	19q13.32				-0.323	0.00223	0.0259				1
DAG1	dystroglycan 1 (dystrophin-associated glycoprotein 1)	3p21				-0.4285	0.00314375	0.0191625				1
DAGLA	diacylglycerol lipase, alpha	11q12.2				-0.407	0.000695	0.01495				1
DAGLB	diacylglycerol lipase, beta	7p22.1				-0.339	0.04216	0.1339				1
DAK	dihydroxyacetone kinase 2 homolog (S. cerevisiae)	11q12.2				-0.363	0.00124	0.0195				1
DALRD3	DALR anticodon binding domain containing 3	3p21.31							-0.599	2.332	5	1
DAND5	DAN domain family, member 5	19p13.2	-0.323	0.01446	0.0747							1
DAO	D-amino-acid oxidase	12q24				-0.353	0.00014	0.0055				1
DAOA	D-amino acid oxidase activator	13q33.2							-0.93	3.18286	7	1
HSD17B14	hydroxysteroid (17-beta) dehydrogenase 14	19q13.33	1.068	0.00307	0.0261							1
SULT1A2	sulfotransferase family, cytosolic, 1A, phenol-preferring, member 2	16p12.1	1.068	0.00102	0.0119							1
UCP2	uncoupling protein 2 (mitochondrial, proton carrier)	11q13	1.068	0.0055	0.039							1
DAPK2	death-associated protein kinase 2	15q22.31				-0.307	0.00111	0.0185				1
ABCC4	ATP-binding cassette, sub-family C (CFTR/MRP), member 4	13q32	1.066	0.00039	0.006							1
DAPL1	In multiple Geneids		-4.071	0	0							1
DAPP1	dual adaptor of phosphotyrosine and 3-phosphoinositides	4q25-q27				0.583	0	0.0017				1
DARC	Duffy blood group, chemokine receptor	1q21-q22	-0.678	0.04476	0.1563							1
PRKRIR	protein-kinase, interferon-inducible double stranded RNA dependent inhibito	11q13.5	1.065	1.00E-05	3.00E-04							1
DAZ1	deleted in azoospermia 1	Yq11.223				-0.523952381	0.016389048	0.071219048				1
DAZ2	deleted in azoospermia 2	Yq11.223				-0.513607143	0.019540357	0.080417857				1
DAZ3	deleted in azoospermia 3	Yq11.223				-0.507897436	0.019224615	0.079938462				1
DAZ4	deleted in azoospermia 4	Yq11.223				-0.518040816	0.018189796	0.07647551				1
DAZAP1	DAZ associated protein 1	19p13.3				-0.3275	0.035725	0.12035				1
DAZAP2	In multiple Geneids					-0.37	0.00082	0.0161				1
DBC1	deleted in bladder cancer 1	9q32-q33				0.32	0.001	0.0177				1
IFITM3	interferon induced transmembrane protein 3	11p15.5	1.064	2.00E-05	6.00E-04							1
DBI	diazepam binding inhibitor (GABA receptor modulator, acyl-CoA binding prote	2q12-q21	-0.696	0.00239	0.0217							1
DBN1	drebrin 1	5q35.3				-0.4056	0.000872	0.01586				1
DBNDD1	dysbindin (dystrobrevin binding protein 1) domain containing 1	16q24.3	-0.433	0.00199	0.019							1
SLC11A1	solute carrier family 11 (proton-coupled divalent metal ion transporters), men	2q35	1.064	0.00131	0.0142							1
DBNL	In multiple Geneids		-1.02	1.00E-05	3.00E-04							1
DBP	D site of albumin promoter (albumin D-box) binding protein	19q13.3	-0.332	0.02157	0.0975							1
DBT	dihydrolipoamide branched chain transacylase E2	1p31							-0.903	3.07857	7	1
DCAF10	DDB1 and CUL4 associated factor 10	9p13.2	-0.423	0.00511	0.037	-0.363	0.003376667	0.022733333				2
DCAF11	DDB1 and CUL4 associated factor 11	14q11.2	-0.485	0.00188	0.0182							1
DCAF12	DDB1 and CUL4 associated factor 12	9p13.3	-0.854	0.00015	0.003	-0.4466	0.002363	0.02389				2
IFITM1	interferon induced transmembrane protein 1	11p15.5	1.062	0.01283	0.0691							1

DCAF15	DDB1 and CUL4 associated factor 15	19p13.12				-0.602	0.00092	0.017				1
DCAF16	DDB1 and CUL4 associated factor 16	4p15.31				-0.44	0.00119	0.0191				1
DCAF4	DDB1 and CUL4 associated factor 4	14q24.3				-0.362142857	0.008018571	0.038985714				1
DCAF4L1	DDB1 and CUL4 associated factor 4-like 1	4p13				-0.303	1.00E-04	0.0065				1
DCAF5	DDB1 and CUL4 associated factor 5	14q23-q24.1	-0.675	3.00E-05	0.001	-0.3125	0.007875	0.04315				2
DCAKD	dephospho-CoA kinase domain containing	17q21.31	-0.602	0.00956	0.0566	-0.3415	0.00198	0.02435				2
DCAL1	Data not found								-0.694	2.626	5	1
TDO2	tryptophan 2,3-dioxygenase	4q31-q32	1.062	0.00675	0.0448	0.383	0.01889	0.0824	-1.031	3.70833	6	3
DCC	deleted in colorectal carcinoma	18q21.3				-0.336222222	0.00278	0.026733333				1
TRIP13	thyroid hormone receptor interactor 13	5p15.33	1.061	0.00075	0.0096	0.319	0.04801	0.1451				2
DCDC2B	doublecortin domain containing 2B	1p35.1				-0.442	0.00301	0.03				1
GPR158	G protein-coupled receptor 158	10p12.1	1.059	4.00E-05	0.0012	0.529	0.00052	0.0131				2
DCI	Data not found					-0.516	0.00209	0.0251				1
DCLK1	doublecortin-like kinase 1	13q13				0.3642	0.011566	0.0557				1
DCLK2	doublecortin-like kinase 2	4q31.3				-0.366	0.00453	0.03625				1
CASK	calcium/calmodulin-dependent serine protein kinase (MAGUK family)	Xp11.4	1.058	0	1.00E-04	0.309	0.00276	0.0288				2
DCLRE1B	DNA cross-link repair 1B	1p13.2				-0.487	0.00012	0.0072				1
DCN	decorin	12q21.33							-0.8665	3.165285	9.25	1
DCP1A	DCP1 decapping enzyme homolog A (S. cerevisiae)	3p21.1				-0.348428571	0.001235714	0.0188				1
DCP2	DCP2 decapping enzyme homolog (S. cerevisiae)	5q22.2				-0.383	0.00051	0.0116				1
DCPS	decapping enzyme, scavenger	11q24.2							0.849	3.084	5	1
DCST1	DC-STAMP domain containing 1	1q22							0.724	2.64812	8	1
DCST2	DC-STAMP domain containing 2	1q22	-0.263	0.03442	0.1317				0.724	2.64812	8	2
DCTD	dCMP deaminase	4q35.1				-0.401333333	0.007918333	0.046916667				1
DCTN2	In multiple Geneids		-0.882	7.00E-05	0.0016							1
DCTN3	dynactin 3 (p22)	9p13							-0.406	2.392	5	1
DCTN4	dynactin 4 (p62)	5q31-q32							-0.746	2.47833	6	1
MCAM	melanoma cell adhesion molecule	11q23.3	1.058	0.00237	0.0216							1
PCDHB14	protocadherin beta 14	5q31	1.058	0.00085	0.0105				-0.915	3.02667	6	2
DCX	doublecortin	Xq22.3-q23				0.325	0.001645	0.0208	-0.708	2.89375	8	2
DCXR	dicarbonyl/L-xylulose reductase	17q25.3	-0.885	2.00E-04	0.0037							1
DDA1	DET1 and DDB1 associated 1	19p13.11				-0.5	0.00053	0.0104				1
ECE1	endothelin converting enzyme 1	1p36.1	1.057	0	1.00E-04							1
PLAU	plasminogen activator, urokinase	10q24	1.057	0.00106	0.0122							1
DDB2	damage-specific DNA binding protein 2, 48kDa	11p12-p11	-0.732	0.00013	0.0028							1
F2RL1	coagulation factor II (thrombin) receptor-like 1	5q13	1.053	0.00034	0.0054							1
GXYLT2	glucoside xylosyltransferase 2	3p13	1.053	0.04467	0.1561							1
ITGAV	integrin, alpha V	2q31-q32	1.053	0.00019	0.0035	0.328	0.00027	0.0099				2
DDI2	DNA-damage inducible 1 homolog 2 (S. cerevisiae)	1p36.21				-0.358666667	0.00369	0.031333333				1
PNPT1	polyribonucleotide nucleotidyltransferase 1	2p15	1.052	6.00E-05	0.0015							1
DDIT4	DNA-damage-inducible transcript 4	10q22.1				-0.509	0.00036	0.0112				1
DDN	dendrin	12q13.12				-0.446	0.00015	0.0077				1
DDO	D-aspartate oxidase	6q21				-0.363	0.00064	0.0144				1
RGPD1	RANBP2-like and GRIP domain containing 1	2p11.2	1.052	0.01326	0.0704							1
DDR1	discoidin domain receptor tyrosine kinase 1	6p21.3							0.792	3.181	5	1
DDR2	discoidin domain receptor tyrosine kinase 2	1q23.3				0.334333333	0.00047	0.010266667				1
ROCK2	Rho-associated, coiled-coil containing protein kinase 2	2p24	1.052	0	1.00E-04							1
DDX1	DEAD (Asp-Glu-Ala-Asp) box helicase 1	2p24				0.3205	0.00347	0.0321				1
THBS4	thrombospondin 4	5q13	1.052	0.03972	0.1449							1
ATP2A3	ATPase, Ca++ transporting, ubiquitous	17p13.3	1.05	0.01007	0.0588							1
DDX17	DEAD (Asp-Glu-Ala-Asp) box helicase 17	22q13.1				-0.400222222	0.001223333	0.015755556				1
STRIP2	Data not found		1.05	0.00037	0.0058							1
DDX19A	DEAD (Asp-Glu-Ala-Asp) box polypeptide 19A	16q22.1				-0.40875	0.0019275	0.022475				1
VIMP	VCP-interacting membrane protein	15q26.3	1.05	3.00E-05	0.001							1
DDX21	DEAD (Asp-Glu-Ala-Asp) box helicase 21	10q21				-0.354	0.01155	0.062				1
DDX23	DEAD (Asp-Glu-Ala-Asp) box polypeptide 23	12q13.12				-0.375	0.00031	0.0105				1
DDX25	DEAD (Asp-Glu-Ala-Asp) box helicase 25	11q24	-0.265	0.00699	0.0458							1
DDX26B	DEAD/H (Asp-Glu-Ala-Asp/His) box polypeptide 26B	Xq26.3				0.374285714	0.002221429	0.024557143	-0.966	2.575	6	2
DNAJC10	DnaJ (Hsp40) homolog, subfamily C, member 10	2q32.1	1.049	0.00012	0.0025							1
DDX28	DEAD (Asp-Glu-Ala-Asp) box polypeptide 28	16q22.1				-0.502	4.00E-04	0.0117				1
DDX39	Data not found					-0.480333333	0.000423333	0.0116				1
SNX10	sorting nexin 10	7p15.2	1.049	0.00021	0.0038							1
DDX4	DEAD (Asp-Glu-Ala-Asp) box polypeptide 4	5p15.2-p13.1				-0.467	0.00019	0.0084				1
DDX41	DEAD (Asp-Glu-Ala-Asp) box polypeptide 41	5q35.3				-0.422	0.000775	0.01345				1
DDX46	DEAD (Asp-Glu-Ala-Asp) box polypeptide 46	5q31.1				-0.402	0.00334875	0.0241625				1
BST2	bone marrow stromal cell antigen 2	19p13.1	1.048	0.02735	0.1135							1
DDX49	DEAD (Asp-Glu-Ala-Asp) box polypeptide 49	19p12				-0.4375	0.002465	0.0269				1
DDX5	DEAD (Asp-Glu-Ala-Asp) box helicase 5	17q21							-0.644	3.06125	8	1
DDIT3	DNA-damage-inducible transcript 3	12q13.1-q13.2	1.048	0.00016	0.0031							1

DDX51	DEAD (Asp-Glu-Ala-Asp) box polypeptide 51	12q24.33				-0.362	0.00022	0.0091				1
DDX52	DEAD (Asp-Glu-Ala-Asp) box polypeptide 52	17q21.1							-0.515	2.402	5	1
DDX54	DEAD (Asp-Glu-Ala-Asp) box polypeptide 54	12q24.13	-0.291	0.0033	0.0274							1
TCF7L2	transcription factor 7-like 2 (T-cell specific, HMG-box)	10q25.3	1.047	1.00E-05	5.00E-04	0.321	0.0015	0.0213				2
FTL	ferritin, light polypeptide	19q13.33	1.045	3.00E-05	0.001							1
DDX58	DEAD (Asp-Glu-Ala-Asp) box polypeptide 58	9p12				-0.383571429	0.00138	0.015514286				1
DDX59	DEAD (Asp-Glu-Ala-Asp) box polypeptide 59	1q32.1	-0.237	0.02903	0.1179							1
ATP2B1	ATPase, Ca++ transporting, plasma membrane 1	12q21.3	1.043	0.00019	0.0036				-0.932	3.85556	9	2
BICC1	bicaudal C homolog 1 (Drosophila)	10q21.1	1.043	0.01927	0.0906	0.316	0.015005	0.06785				2
DEAF1	deformed epidermal autoregulatory factor 1 (Drosophila)	11p15.5	-0.536	0.00095	0.0113	-0.4288	0.003902	0.02852				2
DEC1	deleted in esophageal cancer 1	9q32	-0.367	0.00136	0.0146							1
DECR1	2,4-dienoyl CoA reductase 1, mitochondrial	8q21.3				0.316	6.00E-05	0.0056	-0.817	2.77143	7	2
EML4	echinoderm microtubule associated protein like 4	2p21	1.042	0	2.00E-04							1
DEF6	differentially expressed in FDCP 6 homolog (mouse)	6p21.33-p21.1	-0.41	0.00286	0.0247							1
DEF8	differentially expressed in FDCP 8 homolog (mouse)	16q24.3	-0.3	0.00716	0.0466							1
DEFB1	defensin, beta 1	8p23.1	-0.813	0.01483	0.076							1
DEFB104A	defensin, beta 104A	8p23.1							-1.0095	3.596285	6	1
DEFB105A	defensin, beta 105A	8p23.1							-0.964	3.73778	9	1
DEFB106A	defensin, beta 106A	8p23.1							-0.9035	3.52389	8	1
DEFB110	defensin, beta 110 locus	6p12.3				0.317	0.01689	0.07575	-0.887	3.19714	7	2
DEFB111	defensin, beta 111	6p21							-0.887	3.19714	7	1
DEFB112	defensin, beta 112	6p12.3				0.397	0.00323	0.0312	-1.006	3.52	6	2
DEFB113	defensin, beta 113	6p12.3							-0.888	3.138	5	1
DEFB114	defensin, beta 114	6p12.3							-0.991	3.54025	6.5	1
DEFB118	defensin, beta 118	20q11.21	-0.292	0.00263	0.0232							1
DEFB119	defensin, beta 119	20q11.21	-0.446	0.00022	0.0039							1
DEFB121	defensin, beta 121	20q11.1	-0.3	0.00642	0.0432							1
DEFB125	defensin, beta 125	20p13				0.308	0.00887	0.0536	-0.944	2.806	5	2
DEFB126	defensin, beta 126	20p13				0.367	0.00072	0.0152	-1.004	3.32286	7	2
DEFB127	defensin, beta 127	20p13				0.365	0.00134	0.0201	-1.016	3.50167	6	2
DEFB132	defensin, beta 132	20p13	-0.254	0.0114	0.0639							1
DEFB134	defensin, beta 134	8p23.1							-0.967	3.32286	7	1
DEGS2	delta(4)-desaturase, sphingolipid 2	14q32.2	-0.53	0.00341	0.028	-0.38	0.00176	0.0231				2
SLC45A4	solute carrier family 45, member 4	8q24.3	1.041	5.00E-05	0.0013							1
DEM1	defects in morphology 1 homolog (S. cerevisiae)	1p34.2				-0.333	0.00602	0.0433				1
EDN1	endothelin 1	6p24.1	1.04	0.00961	0.0568							1
DENND1B	DENN/MADD domain containing 1B	1q31.3				0.354666667	0.001582667	0.018486667				1
DENND1C	DENN/MADD domain containing 1C	19p13.3				-0.4634	0.000698	0.01414				1
DENND2A	DENN/MADD domain containing 2A	7q34				-0.3714	0.013138	0.06057				1
DENND2C	DENN/MADD domain containing 2C	1p13.2							-0.683	2.515	6	1
DENND2D	DENN/MADD domain containing 2D	1p13.3	-0.86	1.00E-05	5.00E-04	-0.306	0.00409	0.0352				2
SNORD13P2	small nucleolar RNA, C/D box 13 pseudogene 2	7p22.1	1.039	0.0018	0.0178							1
COL10A1	collagen, type X, alpha 1	6q21-q22	1.038	0.01501	0.0766				-0.83	3.19833	6	2
DNAH14	In multiple Geneids		1.038	0.02477	0.1064	0.36	0.00014	0.0074				2
DENND4C	DENN/MADD domain containing 4C	9p22.1				-0.3815	0.003675833	0.024575				1
DENND5A	DENN/MADD domain containing 5A	11p15.4				-0.37275	0.0075625	0.041775				1
RAB19	RAB19, member RAS oncogene family	7q34	1.037	1.00E-05	5.00E-04							1
DENR	density-regulated protein	12q24.31				-0.3145	0.00261	0.0277				1
IL2RA	interleukin 2 receptor, alpha	10p15-p14	1.036	0.00086	0.0105							1
DEPDC5	DEP domain containing 5	22q12.3	-0.311	0.0237	0.1033	-0.396904762	0.00221619	0.021066667				2
DEPDC6	Data not found								0.692	2.806	5	1
DERA	deoxyribose-phosphate aldolase (putative)	12p12.3				0.320666667	0.001003333	0.0156	-0.868	2.79286	7	2
DERL1	derlin 1	8q24.13				0.348	0.02213	0.0905				1
DESI1	desumoylating isopeptidase 1	22q13.2	-0.855	1.00E-05	3.00E-04							1
PELI2	pellino E3 ubiquitin protein ligase family member 2	14q21	1.036	0.00433	0.0331							1
DEXI	Dexi homolog (mouse)	16p13.13				-0.328	0.01399	0.0692				1
DFFA	DNA fragmentation factor, 45kDa, alpha polypeptide	1p36.3-p36.2				-0.344	0.007565	0.0435				1
HCP5	In multiple Geneids		1.034	0.00181	0.0178							1
PLK1	polo-like kinase 1	16p12.2	1.034	0.01448	0.0747							1
DGCR14	DiGeorge syndrome critical region gene 14	22q11.21	-0.175	0.03173	0.1251	-0.55125	0.00011	0.005475				2
DGCR2	DiGeorge syndrome critical region gene 2	22q11.21	-0.448	0.00072	0.0093	-0.379368421	0.000738421	0.011194737				2
DGCR5	DiGeorge syndrome critical region gene 5 (non-protein coding)	22q11				-0.566333333	0	0.0019				1
DGCR6	DiGeorge syndrome critical region gene 6	22q11.21	-0.398	0.00431	0.033							1
DGCR6L	DiGeorge syndrome critical region gene 6-like	22q11	-0.462	0.00011	0.0024	-0.39	0.00015	0.0077				2
DGCR8	DiGeorge syndrome critical region gene 8	22q11.2				-0.3992	0.000136	0.0071				1
DGKA	diacylglycerol kinase, alpha 80kDa	12q13.3	-1.652	2.00E-05	6.00E-04							1
DGKB	diacylglycerol kinase, beta 90kDa	7p21.2				0.355378378	0.00241027	0.021467568	-1.021	3.20667	6	2
ZNF670	zinc finger protein 670	1q44	1.032	0.00014	0.0028							1
PIM2	pim-2 oncogene	Xp11.23	1.031	0.00699	0.0458							1

DGKG	diacylglycerol kinase, gamma 90kDa	3q27.2-q27.3				0.432	1.00E-05	0.0028				1
DGKH	In multiple Geneids					0.355666667	0.005168333	0.033183333				1
DGKI	diacylglycerol kinase, iota	7q32.3-q33				0.647	2.00E-05	0.0036				1
DGKK	diacylglycerol kinase, kappa	Xp11.22				0.3535	0.006828	0.04109				1
DGKQ	diacylglycerol kinase, theta 110kDa	4p16.3				-0.365	0.00343	0.0322				1
DGKZ	diacylglycerol kinase, zeta	11p11.2				-0.341	0.006965	0.04625	1.275	3.37833	6	2
NUDT16P1	nudix (nucleoside diphosphate linked moiety X)-type motif 16 pseudogene 1	3q22.1	1.03	3.00E-05	0.001							1
DHCR24	24-dehydrocholesterol reductase	1p32.3	-1.298	6.00E-05	0.0015	-0.341	0.001005	0.01705				2
DHDDS	dehydrodolichyl diphosphate synthase	1p36.11	-0.384	0.02433	0.1051	-0.41	0.009056	0.04628				2
DHDH	dihydrodiol dehydrogenase (dimeric)	19q13.3				-0.3405	0.00197	0.02435				1
DHDPSL	Data not found					-0.393	2.50E-05	0.00325				1
DHFR	dihydrofolate reductase	5q11.2-q13.2				-0.314	0.00144	0.0191				1
DHFRL1	dihydrofolate reductase-like 1	3q11.1							-0.815	2.292	5	1
DHH	desert hedgehog	12q13.1				-0.331	0.00012	0.0072				1
DHODH	dihydroorotate dehydrogenase (quinone)	16q22				-0.341	0.00945	0.05254				1
DHPS	deoxyhypusine synthase	19p13.2				-0.532333333	0.002043333	0.020633333				1
DHRS1	dehydrogenase/reductase (SDR family) member 1	14q12	-1.997	0	1.00E-04	-0.347	0.00127	0.0197				2
DHRS3	dehydrogenase/reductase (SDR family) member 3	1p36.1	-0.437	0.0208	0.0952	-0.3412	0.00789	0.04472				2
DHRS4L2	dehydrogenase/reductase (SDR family) member 4 like 2	14q11.2	-0.447	0.00111	0.0126							1
DHRS7	dehydrogenase/reductase (SDR family) member 7	14q23.1							-1.06	2.718	5	1
DHRS7B	dehydrogenase/reductase (SDR family) member 7B	17p12				-0.421	0.00281	0.029				1
DHRS7C	dehydrogenase/reductase (SDR family) member 7C	17p13.1	-0.263	0.00883	0.0536	-0.3195	0.00272	0.0268				2
DHRS9	dehydrogenase/reductase (SDR family) member 9	2q31.1							-0.865	3.19563	8	1
DHRSX	In multiple Geneids					-0.463251534	0.004044724	0.028144172				1
DHTKD1	dehydrogenase E1 and transketolase domain containing 1	10p14				-0.388666667	0.010976667	0.059566667				1
DHX29	DEAH (Asp-Glu-Ala-His) box polypeptide 29	5q11.2	-0.544	0.00011	0.0023	-0.3435	0.002835	0.0273				2
DHX30	DEAH (Asp-Glu-Ala-His) box polypeptide 30	3p21.31				-0.382636364	0.001756364	0.020409091				1
DHX32	DEAH (Asp-Glu-Ala-His) box polypeptide 32	10q26.2	-0.51	4.00E-04	0.0061							1
ZNF124	zinc finger protein 124	1q44	1.03	0.00646	0.0434							1
ZNF224	zinc finger protein 224	19q13.2	1.03	0	1.00E-04							1
SIPA1L3	signal-induced proliferation-associated 1 like 3	19q13.13	1.029	1.00E-05	3.00E-04							1
DHX37	DEAH (Asp-Glu-Ala-His) box polypeptide 37	12q24.31				-0.360333333	0.000186667	0.008366667				1
DHX58	DEXH (Asp-Glu-X-His) box polypeptide 58	17q21.2				-0.324	0.00198	0.0244				1
DHX8	DEAH (Asp-Glu-Ala-His) box polypeptide 8	17q21.31				-0.3805	0.001125	0.015225				1
ZNF525	zinc finger protein 525	19q13.42	1.029	0.00246	0.0222							1
PCDHB13	protocadherin beta 13	5q31	1.028	0.0045	0.034				-0.904	2.755	6	2
DIAPH1	diaphanous homolog 1 (Drosophila)	5q31	-0.421	0.00393	0.0309	-0.342	0.000158	0.00782				2
DIAPH2	diaphanous homolog 2 (Drosophila)	Xq21.33							-0.993	2.61	5	1
DIAPH3	diaphanous homolog 3 (Drosophila)	13q21.2				0.3355	0.0083575	0.044175	-0.896	2.978	5	2
DICER1	dicer 1, ribonuclease type III	14q32.13	-0.655	0.00037	0.0057							1
FNDC1	fibronectin type III domain containing 1	6q25	1.026	0.01202	0.0663							1
SH3BP4	SH3-domain binding protein 4	2q37.1-q37.2	1.026	0.00018	0.0034							1
DIMT1	DIM1 dimethyladenosine transferase 1 homolog (S. cerevisiae)	5q12.1	-0.439	0.02123	0.0966							1
DIMT1L	Data not found					-0.3135	0.00053	0.0131				1
DIO2	deiodinase, iodothyronine, type II	14q24.2-q24.3							-0.835	2.998	5	1
DIO3OS	DIO3 opposite strand/antisense RNA (non-protein coding)	14q32.31				-0.386	0.00014	0.0075				1
DIP	interstitial pneumonitis, desquamative, familial								0.764	2.94667	6	1
DIP2A	DIP2 disco-interacting protein 2 homolog A (Drosophila)	21q22.3	-0.331	0.00886	0.0537	-0.348428571	0.003825714	0.030985714				2
DIP2B	DIP2 disco-interacting protein 2 homolog B (Drosophila)	12q13.12				0.307	0.00546	0.0411				1
TGOLN2	trans-golgi network protein 2	2p11.2	1.026	0	0				-0.85	2.875	8	2
DIRAS1	DIRAS family, GTP-binding RAS-like 1	19p13.3							0.531	2.231	5	1
DIRAS2	DIRAS family, GTP-binding RAS-like 2	9q22.2				0.329	0.02963	0.1078				1
DIRC1	In multiple Geneids					0.322666667	0.000546667	0.0121	-0.752	2.8575	8	2
DIRC2	disrupted in renal carcinoma 2	3q21.1	-0.484	0.01807	0.0867							1
DIRC3	In multiple Geneids					0.3126	0.00185	0.01756				1
DISC1	disrupted in schizophrenia 1	1q42.1				0.317	0.00247	0.0272				1
DISP1	dispatched homolog 1 (Drosophila)	1q41				0.326	0.00372	0.0336				1
DISP2	dispatched homolog 2 (Drosophila)	15q15.1				-0.364	6.00E-04	0.014				1
TGIF1	TGFB-induced factor homeobox 1	18p11.3	1.025	0.00141	0.01425							1
BUB1	budding uninhibited by benzimidazoles 1 homolog (yeast)	2q14	1.024	0.00985	0.0578	0.316	0.00226	0.0261				2
DKFZP434P211	Data not found								0.695	2.932	5	1
DKFZP564O082	Data not found								-0.831	2.75688	8	1
DKFZP686A101	Data not found								-0.811	3.11333	6	1
DKFZp686A162	Data not found					-0.349	0.00455	0.0373				1
DKFZp686E243	Data not found					-0.3805	0.007265	0.03925				1
DKFZp761D191	Data not found								0.752	2.54917	6	1
DKFZp779B154	Data not found								-0.832	3.144	5	1
DKFZp779O175	Data not found								1.356	3.01	5	1
DKK3	dickkopf 3 homolog (Xenopus laevis)	11p15.2	-0.222	0.03982	0.1451							1

DKK4	dickkopf homolog 4 (Xenopus laevis)	8p11.2-p11.1	-0.656	0.02816	0.1157	-0.304	0.01134	0.0614				2
EXOC6	exocyst complex component 6	10q23.33	1.024	2.00E-05	6.00E-04				-0.936	3.36667	6	2
DLEC1	deleted in lung and esophageal cancer 1	3p21.3				-0.336	0.001275	0.018				1
SCARNA6	small Cajal body-specific RNA 6	2q37.1	1.024	0.03165	0.1249							1
DLEU7	deleted in lymphocytic leukemia, 7	13q14.3				0.312333333	0.003413333	0.0311				1
DLG1	discs, large homolog 1 (Drosophila)	3q29				1.108666667	3.33E-06	0.0023				1
DLG2	discs, large homolog 2 (Drosophila)	11q14.1				0.316857143	0.002981429	0.021685714				1
DLG4	discs, large homolog 4 (Drosophila)	17p13.1				-0.3685	0.003625	0.0317				1
DLG5	discs, large homolog 5 (Drosophila)	10q23				-0.34675	0.00136875	0.014675				1
DLGAP1	discs, large (Drosophila) homolog-associated protein 1	18p11.31	-0.196	0.03311	0.1284	-0.34675	0.0047725	0.0362375				2
SLC35B2	solute carrier family 35, member B2	6p12.1-p11.2	1.024	2.00E-05	8.00E-04							1
DLGAP2	discs, large (Drosophila) homolog-associated protein 2	8p23	-0.346	0.00607	0.0417	-0.512	0.00377	0.0338				2
DLGAP3	discs, large (Drosophila) homolog-associated protein 3	1p35.3-p34.1				-0.35	0.00366	0.0333				1
FAM171B	family with sequence similarity 171, member B	2q32.1	1.023	0.00083	0.0103							1
DLK1	delta-like 1 homolog (Drosophila)	14q32				-0.465	0.00627	0.0443	1.027	3.35375	8	2
DLK2	delta-like 2 homolog (Drosophila)	6p21.1	-0.594	0.00329	0.0274							1
DLL1	delta-like 1 (Drosophila)	6q27	-0.674	0.00118	0.0132							1
PDIA3P	protein disulfide isomerase family A, member 3 pseudogene	1q21.1	1.023	1.00E-05	3.00E-04							1
DLX1	In multiple Geneids		-0.219	0.04382	0.1541							1
DLX4	distal-less homeobox 4	17q21.33				-0.338	0.04276	0.135				1
DLX5	distal-less homeobox 5	7q22	-1.105	0.00011	0.0024				0.809	3.978	5	2
CACNA1D	calcium channel, voltage-dependent, L type, alpha 1D subunit	3p14.3	1.022	0.00247	0.0222							1
DMBX1	diencephalon/mesencephalon homeobox 1	1p33	-0.209	0.01286	0.0693							1
DMC1	DMC1 dosage suppressor of mck1 homolog, meiosis-specific homologous recc	22q13.1	-0.222	0.04272	0.1518	-0.395375	0.00078875	0.01315				2
DMD	dystrophin	Xp21.2				0.383125	0.008632813	0.045378125	-0.768888889	2.885713333	7	2
DMGDH	dimethylglycine dehydrogenase	5q14.1	-0.265	0.01116	0.063	-0.34375	0.00104	0.01775				2
DMKN	dermokine	19q13.12	-1.896	1.00E-04	0.0022	-0.3365	0.0061	0.04295				2
DMP1	dentin matrix acidic phosphoprotein 1	4q21							-0.727	2.89714	7	1
DMPK	In multiple Geneids		-0.346	0.03379	0.1301	-0.394	0.00461	0.0375				2
DMRT1	doublesex and mab-3 related transcription factor 1	9p24.3	-0.222	0.0166	0.0818	-0.40432	0.005534	0.031144				2
DMRT2	doublesex and mab-3 related transcription factor 2	9p24.3	-0.232	0.01039	0.0601							1
DMRT3	doublesex and mab-3 related transcription factor 3	9p24.3				-0.435166667	0.001605	0.015816667				1
DMRTA1	DMRT-like family A1	9p21.3	-0.6	0.00016	0.0031							1
DMRTA2	DMRT-like family A2	1p32.3				-0.399	0.00243	0.0271	0.896	3.435	5	2
DMRTC2	DMRT-like family C2	19q13.2							0.829	2.98	6	1
DMTF1	cyclin D binding myb-like transcription factor 1	7q21				0.334	0.00069	0.0149				1
DMWD	dystrophia myotonica, WD repeat containing	19q13.3				-0.3625	0.00039	0.01155				1
DMXL1	Dmx-like 1	5q22	-0.852	0	0	-0.3516	0.000824	0.01388				2
ENAH	enabled homolog (Drosophila)	1q42.12	1.022	0.0016	0.0163							1
PPP4R1L	protein phosphatase 4, regulatory subunit 1-like	20q13.32	1.022	0	0	0.354	0.01413	0.066175				2
DNAAF3	dynein, axonemal, assembly factor 3	19q13.4	-0.213	0.0274	0.1136							1
DNAH1	In multiple Geneids					-0.394285714	0.001485714	0.016771429				1
DNAH10	In multiple Geneids					-0.3236	0.001338	0.01382				1
DNAH11	dynein, axonemal, heavy chain 11	7p21				0.3395	0.00383	0.03355				1
DNAH12	In multiple Geneids					-0.3505	0.009182	0.03542				1
CLSTN3	calsyntenin 3	12p13.31	1.021	0.00222	0.0206							1
DNAH17	dynein, axonemal, heavy chain 17	17q25.3							0.91	3.244	5	1
DNAH2	In multiple Geneids					-0.42244	0.0030396	0.022872				1
DNAH5	dynein, axonemal, heavy chain 5	5p15.2				0.36146875	0.003818125	0.0317				1
SERPINA4	serpin peptidase inhibitor, clade A (alpha-1 antiproteinase, antitrypsin), meml	14q31-q32.1	1.02	0.03467	0.1324							1
DNAH7	dynein, axonemal, heavy chain 7	2q32.3				0.304	0.00352	0.0326				1
DNAH8	dynein, axonemal, heavy chain 8	6p21.2				0.339818182	0.014740909	0.068				1
DNAH9	dynein, axonemal, heavy chain 9	17p12	-0.219	0.02704	0.1127	-0.3545	0.0033075	0.025081818				2
DNAI1	dynein, axonemal, intermediate chain 1	9p13.3				-0.374	0.000298333	0.007816667				1
DNAI2	dynein, axonemal, intermediate chain 2	17q25				-0.48	0.00162	0.0221				1
MYBL2	v-myb myeloblastosis viral oncogene homolog (avian)-like 2	20q13.1	1.019	0.00376	0.03							1
DNAJA2	DnaJ (Hsp40) homolog, subfamily A, member 2	16q12.1				-0.311	0.00107	0.0182				1
DNAJA3	DnaJ (Hsp40) homolog, subfamily A, member 3	16p13.3				-0.493666667	0.0038	0.032133333				1
PSORS1C3	In multiple Geneids		1.019	0.01816	0.087							1
DNAJB1	DnaJ (Hsp40) homolog, subfamily B, member 1	19p13.2	-0.589	0.03049	0.1221							1
GAL3ST1	galactose-3-O-sulfotransferase 1	22q12.2	1.018	4.00E-05	0.0012							1
DNAJB12	DnaJ (Hsp40) homolog, subfamily B, member 12	10q22.1				-0.3255	0.000405	0.01145				1
DNAJB14	DnaJ (Hsp40) homolog, subfamily B, member 14	4q23							-0.934	2.7	7	1
DNAJB3	DnaJ (Hsp40) homolog, subfamily B, member 3	2q37	-0.174	0.02289	0.101							1
DNAJB6	DnaJ (Hsp40) homolog, subfamily B, member 6	7q36.3	-0.539	0.0048	0.0355	-0.328333333	0.013476667	0.063966667				2
DNAJB8	DnaJ (Hsp40) homolog, subfamily B, member 8	3q21.3	-0.226	0.00792	0.0498				1.128	3.572	5	2
DNAJC1	DnaJ (Hsp40) homolog, subfamily C, member 1	10p12.31				-0.322	0.007	0.0471				1
HEYL	hairy/enhancer-of-split related with YRPW motif-like	1p34.3	1.018	0	0							1
DNAJC11	DnaJ (Hsp40) homolog, subfamily C, member 11	1p36.31				-0.4112	0.002895	0.0237				1

EFCAB4B	EF-hand calcium binding domain 4B	12p13.32	1.016	0.00083	0.0093	0.334	0.000663333	0.0125				2
DNAJC13	DnaJ (Hsp40) homolog, subfamily C, member 13	3q22.1				0.3225	0.00392	0.03295				1
NPC1L1	NPC1 (Niemann-Pick disease, type C1, gene)-like 1	7p13	1.016	0.02143	0.0972							1
DNAJC15	DnaJ (Hsp40) homolog, subfamily C, member 15	13q14.1				0.304	0.00654	0.0453				1
DNAJC16	DnaJ (Hsp40) homolog, subfamily C, member 16	1p36.1				-0.391333333	0.00646	0.0401				1
DNAJC17	DnaJ (Hsp40) homolog, subfamily C, member 17	15q15.1				-0.3465	0.000975	0.01735				1
DNAJC18	DnaJ (Hsp40) homolog, subfamily C, member 18	5q31.2				-0.3784	0.010366	0.04				1
RHOC	ras homolog family member C	1p13.1	1.016	0	0							1
DNAJC21	DnaJ (Hsp40) homolog, subfamily C, member 21	5p13.2				0.465	0.00232	0.0264				1
SRGN	serglycin	10q22.1	1.016	0.00244	0.022							1
C21orf90	chromosome 21 open reading frame 90	21q22.3	1.013	0.00116	0.013							1
DNAJC28	DnaJ (Hsp40) homolog, subfamily C, member 28	21q22.11	-0.806	2.00E-05	8.00E-04	-0.32	0.00324	0.0312				2
LEPROTL1	leptin receptor overlapping transcript-like 1	8p21	1.013	3.00E-05	8.00E-04							1
DNAJC30	DnaJ (Hsp40) homolog, subfamily C, member 30	7q11.23				-0.388	0.01511	0.0724				1
DNAJC4	DnaJ (Hsp40) homolog, subfamily C, member 4	11q13	-0.298	0.00976	0.05725							1
DNAJC5	DnaJ (Hsp40) homolog, subfamily C, member 5	20q13.33	-0.534	0.00084	0.0103							1
DNAJC5B	DnaJ (Hsp40) homolog, subfamily C, member 5 beta	8q13.1				0.319666667	0.005853333	0.039966667				1
DNAJC8	DnaJ (Hsp40) homolog, subfamily C, member 8	1p35.3				-0.4155	0.004	0.0318				1
DNAL4	dynein, axonemal, light chain 4	22q13.1	-0.393	0.00178	0.0176	-0.419166667	0.004601667	0.023266667				2
DNASE1L3	deoxyribonuclease I-like 3	3p14.3	-2.079	1.00E-05	3.00E-04	-0.345	0.00239	0.0268				2
CLPTM1L	CLPTM1-like	5p15.33	1.012	8.00E-05	0.0019							1
DNHD1	dynein heavy chain domain 1	11p15.4				-0.458	0.00066	0.0145				1
C4orf19	chromosome 4 open reading frame 19	4p14	1.011	0.00141	0.0149							1
DNM2	dynamin 2	19p13.2				-0.4491	0.006266	0.036993333				1
DNM3	dynamin 3	1q24.3				0.377557143	0.002155	0.021138571				1
ENPEP	glutamyl aminopeptidase (aminopeptidase A)	4q25	1.011	0	1.00E-04							1
DNMT1	DNA (cytosine-5-)-methyltransferase 1	19p13.2				-0.450769231	0.003568846	0.027934615				1
BTNL8	butyrophilin-like 8	5q35.3	1.0105	0.021345	0.09075	0.38625	0.0048125	0.037625	-0.874	3.586	5	3
DNMT3L	DNA (cytosine-5-)-methyltransferase 3-like	21q22.3				-0.3695	0.009405	0.05305	0.667	3.045	8	2
SLC50A1	solute carrier family 50 (sugar transporter), member 1	1q22	1.01	0.00015	0.0029							1
SNORA40	small nucleolar RNA, H/ACA box 40	11q21	1.0095	0.006855	0.0392							1
APMAP	Data not found		1.009	4.00E-05	0.0012							1
DOC1	Data not found								-0.883	2.662	5	1
DOC2B	double C2-like domains, beta	17p13.3				-0.473	0.001138333	0.015766667				1
DOCK1	dedicator of cytokinesis 1	10q26.13-q26.3				-0.331666667	0.002348889	0.021855556				1
DOCK10	dedicator of cytokinesis 10	2q36.2				0.316	0.00062	0.0141	-0.814	3.23333	9	2
DOCK2	dedicator of cytokinesis 2	5q35.1				-0.346166667	0.004541667	0.033483333				1
DOCK3	dedicator of cytokinesis 3	3p21.2	-0.843	5.00E-05	0.0013	-0.34155	0.000953	0.01519				2
MTHFD1L	methylenetetrahydrofolate dehydrogenase (NADP+ dependent) 1-like	6q25.1	1.008	3.00E-05	0.001							1
UBE2T	ubiquitin-conjugating enzyme E2T (putative)	1q32.1	1.008	0.01253	0.0681							1
DOCK6	dedicator of cytokinesis 6	19p13.2				-0.484285714	0.006351429	0.037235714				1
CDC14B	CDC14 cell division cycle 14 homolog B (S. cerevisiae)	9q22.3	1.007	0.00023	0.004							1
DOCK8	dedicator of cytokinesis 8	9p24.3	-0.663	0.01026	0.0596	-0.3686	0.0043265	0.031425				2
DOCK9	dedicator of cytokinesis 9	13q32.3	-1.325	0.00059	0.0081							1
DOHH	deoxyhypusine hydroxylase/monooxygenase	19p13.3				-0.497	0.002585	0.02555				1
HYOU1	hypoxia up-regulated 1	11q23.1-q23.3	1.007	8.00E-05	0.0018							1
DOK5	docking protein 5	20q13.2				0.453142857	0.019497429	0.080837143				1
DOK6	docking protein 6	18q22.2				-0.367	0.01078	0.0597				1
DOK7	docking protein 7	4p16.3				-0.391	0.00727	0.03935				1
LXN	latexin	3q25.32	1.007	0.00053	0.0075							1
DOPEY1	dopey family member 1	6q15	-0.273	0.01117	0.063							1
DOPEY2	dopey family member 2	21q22.2	-1.086	0.00016	0.0032	-0.4185	0.003489118	0.021538235				2
DOT1L	DOT1-like, histone H3 methyltransferase (S. cerevisiae)	19p13.3				-0.472454545	0.001378182	0.018336364				1
C2orf15	chromosome 2 open reading frame 15	2q11.2	1.005	0.00051	0.0073							1
DPCR1	diffuse panbronchiolitis critical region 1	6p21.33							1.094	5.094	5	1
DPEP1	dipeptidase 1 (renal)	16q24.3				-0.446	0.00104	0.0162				1
DPEP2	dipeptidase 2	16q22.1				-0.353	0.00341	0.0321				1
DPF1	D4, zinc and double PHD fingers family 1	19q13.2				-0.4125	0.004505	0.033				1
ACVR1	activin A receptor, type I	2q23-q24	1.004	0	2.00E-04	0.329	0.00017	0.0081	-0.892	3.17429	7	3
DPF3	D4, zinc and double PHD fingers, family 3	14q24.2				-0.346428571	0.001415714	0.0187				1
DPH1	DPH1 homolog (S. cerevisiae)	17p13.3				-0.395	0.00104	0.01795				1
DPP10	dipeptidyl-peptidase 10 (non-functional)	2q14.1				0.350634921	0.001275079	0.015584127				1
DPP3	dipeptidyl-peptidase 3	11q12-q13.1				-0.336	0.00518	0.04				1
HTRA1	HtrA serine peptidase 1	10q26.3	1.004	0.00064	0.0086							1
DPP6	dipeptidyl-peptidase 6	7q36.2	-0.199	0.03502	0.1334	-0.351555556	0.003408889	0.028455556				2
DPP9	dipeptidyl-peptidase 9	19p13.3				-0.4875	0.004128	0.02817				1
DPPA3	developmental pluripotency associated 3	12p13.31				-0.32	0.00316	0.0308				1
DPRX	divergent-paired related homeobox	19q13.42				-0.377	0.00661	0.0456				1
DPT	dermatopontin	1q12-q23				0.337333333	0.000423333	0.0103				1

MSH5	mutS homolog 5 (E. coli)	6p21.3	1.0035	0.003215	0.0249							1
TFRC	transferrin receptor (p90, CD71)	3q29	0.998	0.00261	0.0231							1
ZDHHC2	zinc finger, DHHC-type containing 2	8p22	0.997	0.02622	0.1106	0.374	0.00018	0.0084				2
DPYD	dihydropyrimidine dehydrogenase	1p22				0.367	0.00033	0.0108				1
DPYS	dihydropyrimidinase	8q22				0.318666667	4.00E-05	0.004566667				1
LOX	lysyl oxidase	5q23.2	0.996	0.03913	0.1434							1
POLD3	polymerase (DNA-directed), delta 3, accessory subunit	11q14	0.996	0.00027	0.0045							1
DRC1	Data not found		-0.191	0.01693	0.083							1
DRD1IP	Data not found								1.019	3.324	5	1
DRD3	dopamine receptor D3	3q13.3							-0.91	3.11167	6	1
DRD5	dopamine receptor D5	4p16.1	-0.328	0.039055	0.1423				0.808	3.4	6	2
DRG1	developmentally regulated GTP binding protein 1	22q12.2				-0.413285714	0.002958571	0.019785714				1
DRG2	developmentally regulated GTP binding protein 2	17p11.2				-0.3185	0.009325	0.0496				1
ITGAX	integrin, alpha X (complement component 3 receptor 4 subunit)	16p11.2	0.993	0.00741	0.0478							1
DSC1	desmocollin 1	18q12.1	-1.105	1.00E-05	5.00E-04							1
DSC2	desmocollin 2	18q12.1	-2.015	6.00E-05	0.0016							1
DSC3	desmocollin 3	18q12.1	-4.586	1.00E-05	5.00E-04				0.825	2.702	5	2
DSCAM	Down syndrome cell adhesion molecule	21q22.2				-0.347347826	0.001911304	0.019834783				1
DSCAML1	Down syndrome cell adhesion molecule like 1	11q23				0.635	0.00038	0.0114				1
MDK	midkine (neurite growth-promoting factor 2)	11p11.2	0.991	4.00E-05	0.0012							1
DSCR1	Data not found								-0.861	3.06857	7	1
DSCR3	Down syndrome critical region gene 3	21q22.2	-0.393	0.02235	0.0996	-0.386142857	0.001461429	0.017085714				2
DSCR4	Down syndrome critical region gene 4	21q22.2				-0.332	0.001005	0.0168				1
DSCR6	Down syndrome critical region gene 6	21q22.2				-0.4025	0.00249	0.02055				1
DSCR8	Down syndrome critical region gene 8	21q22.2				-0.316	0.01513	0.0724				1
DSCR9	Down syndrome critical region gene 9 (non-protein coding)	21q22.13				-0.405	0.00052	0.01025				1
DSG1	desmoglein 1	18q12.1	-3.314	0.00118	0.0131							1
BFAR	bifunctional apoptosis regulator	16p13.12	0.99	6.00E-05	0.0016							1
DSG3	desmoglein 3	18q12.1	-4.236	2.00E-04	0.0036							1
DSG4	desmoglein 4	18q12.1							-0.8	2.75167	6	1
IFT88	intraflagellar transport 88 homolog (Chlamydomonas)	13q12.1	0.99	0	3.00E-04							1
DSP	desmoplakin	6p24	-2.162	3.00E-05	0.001	-0.314	0.011376667	0.051466667				2
DSPG3	Data not found								-0.801	2.61286	7	1
DSPP	dentin sialophosphoprotein	4q21.3							-0.841	2.94286	7	1
DST	dystonin	6p12.1				0.356	0.00089	0.0167				1
STARD10	StAR-related lipid transfer (START) domain containing 10	11q13	0.99	0.00097	0.0114							1
DSTYK	dual serine/threonine and tyrosine protein kinase	1q32.1				0.316	0.00218	0.0256				1
DTNA	dystrobrevin, alpha	18q12							-0.978333333	3.66924	6.333333333	1
TSPAN18	tetraspanin 18	11p11.2	0.99	0.00526	0.0378							1
DTWD2	DTW domain containing 2	5q23.1	-0.476	0.01625	0.0807	-0.308	0.009466667	0.047033333				2
DTX2	deltex homolog 2 (Drosophila)	7q11.23	-0.517	0.0148	0.0759	-0.383	0.0122	0.064				2
DTX2P1-UPK3BI	DTX2P1-UPK3BP1-PMS2P11 readthrough (non-protein coding)	7q11.23	-0.526	0.0433	0.1531							1
SLC27A2	solute carrier family 27 (fatty acid transporter), member 2	15q21.2	0.987	0.02197	0.0987							1
ACY1	aminoacylase 1	3p21.1	0.986	0.00487	0.0359							1
DTYMK	deoxythymidylate kinase (thymidylate kinase)	2q37.3				0.3175	0.0039	0.03225				1
DULLARD	Data not found					-0.455	5.50E-05	0.00435				1
DUOX1	dual oxidase 1	15q15.3	-2.965	0	0							1
DUOX2	dual oxidase 2	15q15.3				-0.361	1.00E-05	0.003				1
DUOXA1	dual oxidase maturation factor 1	15q21.1	-2.36	0	0							1
DUOXA2	dual oxidase maturation factor 2	15q15.1	-0.938	0.04445	0.1557							1
DUPD1	dual specificity phosphatase and pro isomerase domain containing 1	10q22.2	-0.263	0.03144	0.1244	-0.329	7.50E-05	0.00595				2
DUS2	Data not found		-0.528	0.00092	0.0111							1
DUS2L	dihydrouridine synthase 2-like, SMM1 homolog (S. cerevisiae)	16q22.1				-0.3766	0.005933	0.03814				1
DUS3L	dihydrouridine synthase 3-like (S. cerevisiae)	19p13.3	-0.398	0.00165	0.0167							1
DUS4L	dihydrouridine synthase 4-like (S. cerevisiae)	7q22-q31							-0.893	2.566	5	1
DUSP1	dual specificity phosphatase 1	5q34	-0.841	0.01244	0.0678							1
PKN1	protein kinase N1	19p13.12	0.985	2.00E-05	7.00E-04							1
DUSP11	dual specificity phosphatase 11 (RNA/RNP complex 1-interacting)	2p13.1	-0.544	0.00614	0.042							1
RGS1	regulator of G-protein signaling 1	1q31	0.985	0.02174	0.098							1
DUSP13	dual specificity phosphatase 13	10q22.2	-0.243	0.02195	0.0987	-0.4455	6.50E-05	0.0055				2
DUSP14	dual specificity phosphatase 14	17q12	-0.987	0.00122	0.0135	-0.324	0.01542	0.0733				2
DUSP18	dual specificity phosphatase 18	22q12.2				-0.424	0.00063	0.0136				1
DUSP22	dual specificity phosphatase 22	6p25.3				0.4645	0.00217	0.021				1
DUSP23	dual specificity phosphatase 23	1q23.2	-0.326	0.02073	0.095							1
DUSP26	dual specificity phosphatase 26 (putative)	8p12				-0.478	0.00144	0.0209				1
DUSP4	dual specificity phosphatase 4	8p12-p11							-0.486	2.335396667	6.666666667	1
DUSP5	dual specificity phosphatase 5	10q25	-2.113	1.00E-05	4.00E-04							1
CASC4	cancer susceptibility candidate 4	15q15.3	0.984	1.00E-05	4.00E-04							1
DUSP7	dual specificity phosphatase 7	3p21	-1.435	1.00E-05	5.00E-04							1

LRP5	In multiple Geneids		0.984	0	0							1
DUX4	In multiple Geneids		-0.347	0.030955	0.11975							1
DUXA	double homeobox A	19q13.43							-0.886	2.96	6	1
DVL1	dishevelled, dsh homolog 1 (Drosophila)	1p36	-0.328	0.00699	0.0458							1
DVL2	dishevelled, dsh homolog 2 (Drosophila)	17p13.1				-0.384333333	0.00328	0.027833333				1
DYDC2	In multiple Geneids		-0.186	0.04883	0.1647							1
DYM	dymeclin	18q21.1	-0.285	0.04193	0.15	-0.31975	0.022085	0.0891				2
DYNAP	Data not found		-4.759	0	0							1
DYNC1H1	dynein, cytoplasmic 1, heavy chain 1	14q32				-0.3724	0.00171	0.01732				1
ARHGAP12	Rho GTPase activating protein 12	10p11.22	0.983	0.00041	0.0062							1
DYNC1LI1	dynein, cytoplasmic 1, light intermediate chain 1	3p22.3							-0.787	2.67833	6	1
DYNC1LI2	dynein, cytoplasmic 1, light intermediate chain 2	16q22.1				-0.355	0.00902	0.0519				1
DYNC2H1	dynein, cytoplasmic 2, heavy chain 1	11q21-q22.1				0.331333333	0.006273333	0.044				1
DYNLL1	dynein, light chain, LC8-type 1	12q24.23	-0.653333333	0.00074	0.0082	-0.361333333	0.006303333	0.0394				2
EIF2AK2	eukaryotic translation initiation factor 2-alpha kinase 2	2p22-p21	0.983	0.00024	0.0041							1
DYNLT3	dynein, light chain, Tctex-type 3	Xp21				0.307	0.00165	0.0223				1
DYRK1A	dual-specificity tyrosine-(Y)-phosphorylation regulated kinase 1A	21q22.13							-0.813	3.03071	7	1
PEAK1	NKF3 kinase family member	15q24.3	0.983	0	1.00E-04							1
TMEM140	transmembrane protein 140	7q33	0.983	0.00016	0.0032							1
DYRK4	dual-specificity tyrosine-(Y)-phosphorylation regulated kinase 4	12p13.32				0.305	0.00501	0.0392				1
FNBP1L	formin binding protein 1-like	1p22.1	0.982	0.00015	0.003							1
DYX1C1	dyslexia susceptibility 1 candidate 1	15q21.3				-0.34975	0.0159725	0.068975				1
DZIP3	DAZ interacting protein 3, zinc finger	3q13.13							-0.782	2.412	5	1
SCN2A	sodium channel, voltage-gated, type II, alpha subunit	2q24.3	0.982	0.04612	0.1592	0.358714286	0.000561429	0.007957143				2
E2F2	E2F transcription factor 2	1p36	-0.539	0.00251	0.0225	-0.35325	0.01022	0.052375				2
SLC29A2	solute carrier family 29 (nucleoside transporters), member 2	11q13	0.982	3.00E-05	8.00E-04							1
SLC39A8	solute carrier family 39 (zinc transporter), member 8	4q22-q24	0.982	0.00228	0.021							1
GPC4	glypican 4	Xq26.1	0.981	0.00194	0.0187							1
IFIT1	In multiple Geneids		0.981	0.02306	0.1015							1
E2F8	E2F transcription factor 8	11p15.1							-0.841	2.442	5	1
EAF1	ELL associated factor 1	3p25.1				-0.318666667	0.003283333	0.0296				1
EAPP	E2F-associated phosphoprotein	14q13.1				-0.304	0.02705	0.102				1
EARS2	glutamyl-tRNA synthetase 2, mitochondrial (putative)	16p12.2				-0.317	0.0058	0.0424				1
EBAG9	estrogen receptor binding site associated, antigen, 9	8q23				0.3335	0.000195	0.00795				1
EBF1	early B-cell factor 1	5q34				0.328	0.00013	0.0074				1
EBF2	early B-cell factor 2	8p21.2							0.915	2.70583	6	1
EBF3	In multiple Geneids					-0.3255	0.000455	0.0098				1
EBI3	Epstein-Barr virus induced 3	19p13.3				-0.481333333	0.005643333	0.0343				1
SNRPA1	small nuclear ribonucleoprotein polypeptide A'	15q26.3	0.98	0.00192	0.0185							1
TBC1D1	TBC1 (tre-2/USP6, BUB2, cdc16) domain family, member 1	4p14	0.98	0.00196	0.0188							1
ESM1	endothelial cell-specific molecule 1	5q11.2	0.979	0.00055	0.0077							1
ECE2	endothelin converting enzyme 2	3q27.1							-0.725	2.63	5	1
ECH1	enoyl CoA hydratase 1, peroxisomal	19q13.1				-0.4185	0.000765	0.01545				1
ECHDC2	enoyl CoA hydratase domain containing 2	1p32.3	-0.623	0.00016	0.0032	-0.356	0.010405	0.051575				2
ECHDC3	enoyl CoA hydratase domain containing 3	10p14	-1.072	0.00209	0.0197							1
ECM1	extracellular matrix protein 1	1q21	-3.456	1.00E-05	5.00E-04							1
PTGER2	prostaglandin E receptor 2 (subtype EP2), 53kDa	14q22	0.979	0.01351	0.0713				-0.874	2.55333	6	2
ECSCR	endothelial cell surface expressed chemotaxis and apoptosis regulator	5q31.2				-0.397	0.00141	0.017166667				1
ECSIT	ECSIT homolog (Drosophila)	19p13.2				-0.437333333	0.00527	0.035516667				1
SLC29A4	solute carrier family 29 (nucleoside transporters), member 4	7p22.1	0.979	0.03246	0.1268							1
ECT2L	In multiple Geneids					-0.3	0.00334	0.0317				1
EDA	ectodysplasin A	Xq12-q13.1				0.372	0.0002475	0.008925				1
EDA2R	ectodysplasin A2 receptor	Xq12				0.308	0.00164	0.02215	-0.744	2.52	6	2
EDARADD	EDAR-associated death domain	1q42.3	-1.074	0	0							1
EDC4	enhancer of mRNA decapping 4	16q22.1				-0.564	0.00023	0.0092				1
EDEM1	ER degradation enhancer, mannosidase alpha-like 1	3p26.1	-0.22	0.03322	0.1288	-0.357	0.00185	0.0237				2
SNORD4B	small nucleolar RNA, C/D box 4B	17q11	0.978	0.0022	0.0204							1
CNNM3	cyclin M3	2p12-p11.2	0.977	7.00E-05	0.0017							1
DUSP6	dual specificity phosphatase 6	12q22-q23	0.977	5.00E-05	0.0014				-0.594	2.49714	7	2
SEC24D	SEC24 family, member D (S. cerevisiae)	4q26	0.977	0.00017	0.0032							1
EDN2	endothelin 2	1p34	-0.933	0.00039	0.006							1
EDN3	endothelin 3	20q13.2-q13.3				0.435	0.00031	0.0104				1
FAM188B	family with sequence similarity 188, member B	7p14.3	0.976	0	0	0.412	7.00E-05	0.0059				2
GCA	grancalcin, EF-hand calcium binding protein	2q24.2	0.976	0.00103	0.0119	0.3155	0.003585	0.02675				2
EEA1	early endosome antigen 1	12q22	-1.037	2.00E-04	0.0036	-0.372	0.00179	0.0233				2
LPHN3	latrophilin 3	4q13.1	0.976	0.00882	0.0536							1
EEF1A2	eukaryotic translation elongation factor 1 alpha 2	20q13.3	-0.389	0.01793	0.0862							1
EEF1DP3	eukaryotic translation elongation factor 1 delta pseudogene 3	13q13.1				0.3338	0.005986	0.04046				1
EEF2	eukaryotic translation elongation factor 2	19pter-q12				-0.538666667	0.00391	0.0275				1

EEF2K	eukaryotic elongation factor-2 kinase	16p12.2				-0.3295	0.000985	0.0155				1
PNKD	In multiple Geneids		0.976	2.00E-05	7.00E-04							1
EFCAB3	EF-hand calcium binding domain 3	17q23.2	-0.241	0.01502	0.0767							1
LAPTM4B	lysosomal protein transmembrane 4 beta	8q22.1	0.974	0.00017	0.0032							1
ARRB2	arrestin, beta 2	17p13	0.973	1.00E-04	0.0021							1
EFCAB6	EF-hand calcium binding domain 6	22q13.2				-0.394909091	0.001210455	0.013143182				1
EFCAB8	EF-hand calcium binding domain 8	20q11.21				-0.319	0.00294	0.0297				1
EFEMP1	EGF containing fibulin-like extracellular matrix protein 1	2p16				0.3195	0.003035	0.024666667				1
CSGALNACT2	chondroitin sulfate N-acetylgalactosaminyltransferase 2	10q11.21	0.973	0.00111	0.0126							1
EFHB	EF-hand domain family, member B	3p24.3				-0.313333333	0.001093333	0.018333333				1
EFHC2	EF-hand domain (C-terminal) containing 2	Xp11.3				0.35725	0.0055425	0.038725				1
NR0B2	nuclear receptor subfamily 0, group B, member 2	1p36.1	0.973	0.0431	0.1526							1
SKIL	SKI-like oncogene	3q26	0.973	2.00E-05	6.00E-04							1
ANXA5	annexin A5	4q27	0.971	0	1.00E-04				-0.542	2.476	5	2
GRB14	growth factor receptor-bound protein 14	2q22-q24	0.971	0.00361	0.0292	0.3234	0.000286	0.00846				2
EFNA3	ephrin-A3	1q21-q22	-0.777	0.02871	0.1172				0.395	2.612	5	2
EFNA5	ephrin-A5	5q21				0.323	0.0055	0.0412				1
NFKB2	nuclear factor of kappa light polypeptide gene enhancer in B-cells 2 (p49/p10	10q24	0.97	2.00E-05	6.00E-04							1
VOPP1	vesicular, overexpressed in cancer, prosurvival protein 1	7p11.2	0.969	0.00031	0.005	0.338	0.028185	0.10325				2
EFNB3	ephrin-B3	17p13.1	-0.49	0.00046	0.0067	-0.442	0.00096	0.0173				2
EFR3A	EFR3 homolog A (S. cerevisiae)	8q24.22				0.47665	0.014029	0.06583				1
EFS	embryonal Fyn-associated substrate	14q11.2-q12	-1.296	0	0							1
ATP13A3	ATPase type 13A3	3q29	0.968	6.00E-05	0.0015							1
EFTUD2	elongation factor Tu GTP binding domain containing 2	17q21.31				-0.388333333	0.006643333	0.043833333				1
EGF	epidermal growth factor	4q25				-0.32	0.00336	0.03135				1
EGFL6	EGF-like-domain, multiple 6	Xp22				0.674	0	0.0016				1
EGFL8	EGF-like-domain, multiple 8	6p21.32							0.734	3.139723333	6	1
EGFLAM	EGF-like, fibronectin type III and laminin G domains	5p13.2-p13.1				0.338615385	0.008898462	0.050292308				1
EGFR	epidermal growth factor receptor	7p12				0.370142857	0.008782143	0.048917857				1
EGLN1	egl nine homolog 1 (C. elegans)	1q42.1							-0.911	2.702	5	1
EGLN2	egl nine homolog 2 (C. elegans)	19q13.2				-0.4005	0.00101	0.0177				1
EGR1	early growth response 1	5q31.1				-0.4295	0.00031	0.01				1
EGR3	early growth response 3	8p23-p21							-0.607	2.732	5	1
EHBP1	EH domain binding protein 1	2p15				0.343222222	0.000721111	0.0128				1
EHD2	EH-domain containing 2	19q13.3				-0.354	0.0079675	0.044175				1
EHD3	EH-domain containing 3	2p21	-2.948	0	1.00E-04							1
EHF	ets homologous factor	11p12	-1.325	0.00051	0.0072							1
EHMT1	euchromatic histone-lysine N-methyltransferase 1	9q34.3				-0.524	8.00E-05	0.0061				1
KIAA1549	KIAA1549	7q34	0.968	3.00E-05	9.00E-04							1
EIF1	In multiple Geneids					-0.357	0.00179	0.0233				1
EIF1AD	eukaryotic translation initiation factor 1A domain containing	11q13.1				-0.346	0.00428	0.0361				1
EIF1AY	eukaryotic translation initiation factor 1A, Y-linked	Yq11.223				-0.503	0.0051	0.0396				1
KCNN4	potassium intermediate/small conductance calcium-activated channel, subfar	19q13.2	0.967	0.00111	0.0125							1
SLC7A11	solute carrier family 7 (anionic amino acid transporter light chain, xc- system),	4q28-q32	0.967	0.03763	0.14				-0.87	2.63125	8	2
TAP1	transporter 1, ATP-binding cassette, sub-family B (MDR/TAP)	6p21.3	0.967	0.00261	0.0231							1
EIF2B3	eukaryotic translation initiation factor 2B, subunit 3 gamma, 58kDa	1p34.1				-0.334	0.03885	0.1272				1
EIF2C1	eukaryotic translation initiation factor 2C, 1	1p34.3				-0.359	0.00483	0.03835	0.686	2.564	5	2
EIF2C2	eukaryotic translation initiation factor 2C, 2	8q24				-0.42	0.00639	0.0447				1
EIF2C3	eukaryotic translation initiation factor 2C, 3	1p34				-0.311	0.02482	0.0969				1
EIF2S1	eukaryotic translation initiation factor 2, subunit 1 alpha, 35kDa	14q23.3							-0.876	2.89	6	1
EIF3A	In multiple Geneids					-0.324	0.009135	0.04875				1
CHD1L	chromodomain helicase DNA binding protein 1-like	1q12	0.966	0.00023	0.004	0.3725	0.00259	0.0279				2
EIF3CL	eukaryotic translation initiation factor 3, subunit C-like	16p11.2				-0.4085	0.002605	0.0231				1
EIF3D	eukaryotic translation initiation factor 3, subunit D	22q13.1	-0.495	2.00E-04	0.0036	-0.453	0.0004175	0.0084				2
EIF3H	eukaryotic translation initiation factor 3, subunit H	8q24.11				0.418351351	0.007123514	0.042935135				1
EIF3I	eukaryotic translation initiation factor 3, subunit I	1p34.1				-0.381	0.00464	0.0358				1
EIF3K	eukaryotic translation initiation factor 3, subunit K	19q13.2				-0.349	0.002895	0.02715				1
EIF3L	eukaryotic translation initiation factor 3, subunit L	22q	-0.569	0.00093	0.0111	-0.419333333	0.007165	0.03325				2
EIF3S12	Data not found								-0.638	2.432	5	1
EIF3S3	Data not found								-1.077	2.97143	7	1
EIF3S5	Data not found								-0.671	2.60286	7	1
EIF4A1	eukaryotic translation initiation factor 4A1	17p13				-0.4505	0.004715	0.0362				1
EIF4B	eukaryotic translation initiation factor 4B	12q13.13	-0.621	0.01103	0.0625							1
EIF4E	eukaryotic translation initiation factor 4E	4q21-q25				-0.425	0.00029	0.0103				1
EIF4E1B	eukaryotic translation initiation factor 4E family member 1B	5q35.2				-0.369	0.0039	0.0344				1
EIF4E3	eukaryotic translation initiation factor 4E family member 3	3p14	-0.901	0.00028	0.0046							1
EIF4EBP1	eukaryotic translation initiation factor 4E binding protein 1	8p12				-0.336	0.00272	0.0286				1
EIF4EBP2	eukaryotic translation initiation factor 4E binding protein 2	10q21-q22				-0.3925	7.50E-05	0.0058				1
EIF4ENIF1	eukaryotic translation initiation factor 4E nuclear import factor 1	22q11.2	-0.555	6.00E-05	0.0016	-0.4499375	0.001818125	0.01815				2

EIF4G1	eukaryotic translation initiation factor 4 gamma, 1	3q27-qter							0.756	2.578	5	1
EIF4G2	eukaryotic translation initiation factor 4 gamma, 2	11p15	-0.271	0.02051	0.0943							1
EIF4G3	eukaryotic translation initiation factor 4 gamma, 3	1p36.12							-0.838	2.84875	8	1
EIF4H	eukaryotic translation initiation factor 4H	7q11.23	-0.267	0.00591	0.041	-0.371	0.01117	0.06075				2
EIF5A	eukaryotic translation initiation factor 5A	17p13-p12	-0.323	0.00708	0.0462	-0.401	0.00178	0.0232				2
SGIP1	In multiple Geneids		0.966	0.00054	0.0076							1
EIF5AL1	In multiple Geneids		-0.392	0.04471	0.1561							1
ELAC1	elaC homolog 1 (E. coli)	18q21				-0.486	0.00188	0.02185				1
ELAC2	elaC homolog 2 (E. coli)	17p11.2				-0.391	0.000672	0.01306				1
ELAVL1	ELAV (embryonic lethal, abnormal vision, Drosophila)-like 1 (Hu antigen R)	19p13.2				-0.4423125	0.005425625	0.032425				1
ELAVL2	ELAV (embryonic lethal, abnormal vision, Drosophila)-like 2 (Hu antigen B)	9p21				-0.333222222	0.014536667	0.065755556				1
ELAVL3	ELAV (embryonic lethal, abnormal vision, Drosophila)-like 3 (Hu antigen C)	19p13.2				-0.4128	0.006277	0.04035				1
ELF2	E74-like factor 2 (ets domain transcription factor)	4q28							-0.955	3.220833333	8	1
SLC5A6	solute carrier family 5 (sodium-dependent vitamin transporter), member 6	2p23	0.965	0.01219	0.067							1
ELF5	E74-like factor 5 (ets domain transcription factor)	11p13-p12	-0.713	0.00131	0.0142				0.769	2.786	5	2
FAM72D	family with sequence similarity 72, member D	1q21.1	0.964	0.00783	0.04915							1
ELK3	ELK3, ETS-domain protein (SRF accessory protein 2)	12q23	-0.945	0.00083	0.0103	-0.333	0.00147	0.0203				2
FLT1	fms-related tyrosine kinase 1 (vascular endothelial growth factor/vascular per	13q12	0.961	0.00088	0.0107				-0.616	2.60167	6	2
ELL	elongation factor RNA polymerase II	19p13.1				-0.41275	0.001853	0.0188				1
ELL2	elongation factor, RNA polymerase II, 2	5q15	-1.2625	0	1.00E-04							1
ELMO1	engulfment and cell motility 1	7p14.1				0.3405	0.000905	0.01331	-0.694	2.77857	7	2
ELMO2	engulfment and cell motility 2	20q13	-0.834	6.00E-05	0.0015							1
ELMSAN1	Data not found		-0.28	0.01847	0.0881							1
ELN	elastin	7q11.23				-0.434	0.0037825	0.029625				1
ELOF1	elongation factor 1 homolog (S. cerevisiae)	19p13.2				-0.439333333	0.00393	0.033733333				1
ELOVL1	ELOVL fatty acid elongase 1	1p34.2	-0.515	0.00467	0.0348							1
ELOVL3	ELOVL fatty acid elongase 3	10q24.32				-0.372	0.00033	0.00865				1
ELOVL4	ELOVL fatty acid elongase 4	6q14	-2.231	1.00E-05	3.00E-04							1
ELOVL5	ELOVL fatty acid elongase 5	6p21.1-p12.1							0.85	2.866	5	1
ELOVL6	ELOVL fatty acid elongase 6	4q25	-0.956	0.01071	0.0613	-0.380833333	0.00432	0.029491667				2
PCDHB9	protocadherin beta 9	5q31	0.961	0.00127	0.0139				-1.046	3.358	5	2
CERCAM	cerebral endothelial cell adhesion molecule	9q34.11	0.96	0.00045	0.0067							1
ELP2P	Data not found					-0.607	5.00E-05	0.0052				1
ELSPBP1	epididymal sperm binding protein 1	19q13.33				-0.316666667	0.0144	0.064				1
LRRC16A	leucine rich repeat containing 16A	6p22.2	0.96	0.00055	0.0077	0.34825	0.02124	0.085275				2
BCMO1	beta-carotene 15,15'-monooxygenase 1	16q23.2	0.959	0.0072	0.0468							1
ADAMDEC1	ADAM-like, decysin 1	8p21.2	0.958	0.01373	0.0722				-0.927	3.25	6	2
EMC3	ER membrane protein complex subunit 3	3p25.3	-0.636	0.00394	0.0309							1
EMC4	ER membrane protein complex subunit 4	15q14	-0.377	0.02955	0.1194							1
ZNFX1	zinc finger, NFX1-type containing 1	20q13.13	0.958	5.00E-05	0.0013							1
EMCN	endomucin	4q24							-0.733	2.415	6	1
HSPD1	heat shock 60kDa protein 1 (chaperonin)	2q33.1	0.957	2.00E-04	0.0036				-0.539	2.302	5	2
EMID1	EMI domain containing 1	22q12.2				-0.3924	0.005796	0.0354				1
EMID2	EMI domain containing 2	7q22.1				-0.353692308	0.022070769	0.084515385				1
CDKN3	cyclin-dependent kinase inhibitor 3	14q22	0.956	0.01338	0.0709							1
LINC00483	long intergenic non-protein coding RNA 483	17q21.33	0.956	0.01343	0.0711							1
EML1	echinoderm microtubule associated protein like 1	14q32				-0.325	0.00473	0.0375				1
EML2	echinoderm microtubule associated protein like 2	19q13.32	-0.319	0.04072	0.1472	-0.365	0.00468	0.0379				2
EML3	echinoderm microtubule associated protein like 3	11q12.3	-0.443	0.00068	0.009	-0.309	0.0049	0.0388				2
STX3	syntaxin 3	11q12.1	0.956	0.00012	0.0026							1
EML5	echinoderm microtubule associated protein like 5	14q31.3				-0.362	7.00E-05	0.0057				1
EML6	echinoderm microtubule associated protein like 6	2p16.1				0.353714286	0.000447143	0.011314286				1
EMP1	epithelial membrane protein 1	12p12.3	-2.791	0	1.00E-04							1
EMP2	epithelial membrane protein 2	16p13.2	-1.296	0.00075	0.0096	-0.4	0.00217	0.0255				2
EMR1	egf-like module containing, mucin-like, hormone receptor-like 1	19p13.3				-0.387857143	0.004323571	0.032714286				1
PVR	poliovirus receptor	19q13.2	0.955	0	2.00E-04							1
EMR3	egf-like module containing, mucin-like, hormone receptor-like 3	19p13.1				-0.368666667	0.001491667	0.019733333				1
EMR4P	egf-like module containing, mucin-like, hormone receptor-like 4 pseudogene	19p13.3	-0.314	0.02407	0.1044	-0.3628	0.008574	0.04548				2
EN1	engrailed homeobox 1	2q14.2							0.82	2.97125	8	1
ADAMTS2	ADAM metallopeptidase with thrombospondin type 1 motif, 2	5qter	0.952	0.00069	0.0091							1
ENAM	enamelin	4q13.3							-1.008	3.4975	8	1
HOXB9	homeobox B9	17q21.3	0.952	0.00032	0.0051							1
ENDOU	endonuclease, polyU-specific	12q13.1	-3.436	0	0							1
ENDOV	endonuclease V	17q25.3	-0.215	0.03481	0.1328							1
TOP2A	topoisomerase (DNA) II alpha 170kDa	17q21-q22	0.952	0.01985	0.0923							1
ANGPTL2	angiopoietin-like 2	9q34	0.951	0.00922	0.0552							1
ENO1	enolase 1, (alpha)	1p36.2				-0.4485	0.002715	0.0222				1
IFT20	intraflagellar transport 20 homolog (Chlamydomonas)	17q11.2	0.951	0.00023	0.0041							1
ENO3	enolase 3 (beta, muscle)	17pter-p11				-0.382666667	0.002906667	0.027933333				1

ENOPH1	enolase-phosphatase 1	4q21.22				-0.319	0.00024	0.0094				1
ENOSF1	enolase superfamily member 1	18p11.32				-0.301	0.003155	0.03015				1
ENOX1	ecto-NOX disulfide-thiol exchanger 1	13q14.11				0.337384615	0.003785385	0.028311538				1
ENOX2	ecto-NOX disulfide-thiol exchanger 2	Xq25				0.341166667	0.003940833	0.029725				1
KIF14	kinesin family member 14	1q32.1	0.951	0.00203	0.0193							1
MAPK8IP1	mitogen-activated protein kinase 8 interacting protein 1	11p11.2	0.95	0.033	0.1282							1
ENPP2	ectonucleotide pyrophosphatase/phosphodiesterase 2	8q24.1				0.378	0.008995	0.0536				1
ENPP3	ectonucleotide pyrophosphatase/phosphodiesterase 3	6q22							-0.562	2.666	5	1
FER1L4	fer-1-like 4 (C. elegans) pseudogene	20q11.22	0.9495	0.007145	0.0464							1
ENPP6	ectonucleotide pyrophosphatase/phosphodiesterase 6	4q35.1				-0.375571429	0.009424286	0.050228571				1
ENSA	endosulfine alpha	1q21.3	-0.6095	0.0213	0.0958							1
ENTHD1	ENTH domain containing 1	22q13.1				-0.380142857	0.001487857	0.014064286				1
IFITM2	interferon induced transmembrane protein 2	11p15.5	0.948	0.00194	0.0187							1
ENTPD3	ectonucleoside triphosphate diphosphohydrolase 3	3p21.3	-1.996	0	0	-0.332	0.0019	0.0239				2
ZSWIM5	zinc finger, SWIM-type containing 5	1p34.1	0.948	0.00187	0.0182							1
ENTPD5	ectonucleoside triphosphate diphosphohydrolase 5	14q24				-0.3884	0.006878	0.0421				1
SPOPL	speckle-type POZ protein-like	2q22.1	0.947	0	0	0.43	0.00016	0.0079				2
ENTPD7	ectonucleoside triphosphate diphosphohydrolase 7					-0.334333333	0.000586667	0.010566667				1
IFNAR2	interferon (alpha, beta and omega) receptor 2	21q22.11	0.946	5.00E-05	0.0014							1
GRK5	G protein-coupled receptor kinase 5	10q26.11	0.944	0.023165	0.0809							1
EP300	E1A binding protein p300	22q13.2				-0.410769231	0.002831538	0.024692308				1
EP400	E1A binding protein p400	12q24.33				0.316	0.00028	0.0101				1
EPAS1	endothelial PAS domain protein 1	2p21-p16				0.3414	0.000898	0.01648				1
EPB41	erythrocyte membrane protein band 4.1 (elliptocytosis 1, RH-linked)	1p33-p32				-0.414947368	0.004523684	0.030855263	0.782	2.448	5	2
MYO10	myosin X	5p15.1-p14.3	0.943	0.00689	0.0454							1
EPB41L3	In multiple Geneids					0.328	0.00308	0.0304				1
EPB41L4A	erythrocyte membrane protein band 4.1 like 4A	5q21.3	-2.254	0	1.00E-04	-0.359	0.00387	0.0343				2
SCG5	secretogranin V (7B2 protein)	15q13-q14	0.943	0.01646	0.0814				-0.791	2.56	5	2
ARHGAP29	Rho GTPase activating protein 29	1p22.1	0.942	0.00231	0.0212							1
EPB42	erythrocyte membrane protein band 4.2	15q15-q21	-0.319	0.01327	0.0705	-0.353	0.00327	0.031366667				2
EPB49	erythrocyte membrane protein band 4.9 (dematin)	8p21.1				-0.4185	0.003675	0.0278				1
IFIT2	In multiple Geneids		0.942	0.005	0.0365				-0.73	2.902	5	2
EPG5	ectopic P-granules autophagy protein 5 homolog (C. elegans)	18q12.3	-0.503	0.00846	0.0521							1
EPGN	epithelial mitogen homolog (mouse)	4q13.3	-3.049	0	0							1
EPHA1	EPH receptor A1	7q34	-1.686	3.00E-05	0.001	-0.422	0	0.0021				2
ABHD11	abhydrolase domain containing 11	7q11.23	0.941	4.00E-05	0.0011							1
EPHA2	EPH receptor A2	1p36	-0.951	0.00889	0.0539	-0.403166667	0.002591667	0.021266667				2
EPHA3	EPH receptor A3	3p11.2							-0.929	3.18333	6	1
EPHA4	EPH receptor A4	2q36.1	-0.634	0.02212	0.099							1
EPHA5	EPH receptor A5	4q13.1	-0.23	0.0424	0.1511							1
EPHA6	In multiple Geneids					0.34375	0.0093825	0.05505				1
EPHA8	EPH receptor A8	1p36.12				-0.368	0.00384	0.0341				1
EPHB1	EPH receptor B1	3q21-q23				0.324	0.00039	0.0116				1
HLA-C	major histocompatibility complex, class I, C	6p21.3	0.941	0.00534	0.0382							1
EPHB3	EPH receptor B3	3q21-qter	-1.234	0.00013	0.0027							1
NEK2	NIMA (never in mitosis gene a)-related kinase 2	1q32.2-q41	0.941	0.00796	0.05							1
EPHB6	EPH receptor B6	7q33-q35	-1.212	1.00E-05	4.00E-04							1
EPHX1	epoxide hydrolase 1, microsomal (xenobiotic)	1q42.1	-0.963	0.01892	0.0895							1
EPHX2	epoxide hydrolase 2, cytoplasmic	8p21	-0.869	0.00671	0.0446	-0.311333333	0.00186	0.022166667				2
EPHX3	epoxide hydrolase 3	19p13.12	-3.219	2.00E-05	7.00E-04	-0.412	0.00061	0.0141				2
STX2	syntaxin 2	12q24.33	0.941	0.00023	0.0041							1
EPM2AIP1	EPM2A (laforin) interacting protein 1	3p22.1				-0.375	0.00065	0.0145				1
EPN1	epsin 1	19q13.42				-0.526	3.00E-05	0.0041				1
EPN2	epsin 2	17p11.2	-0.911	7.00E-05	0.0017	-0.396714286	0.003667143	0.0277				2
EPN3	epsin 3	17q21.33	-1.189	0	1.00E-04							1
EPOR	erythropoietin receptor	19p13.3-p13.2				-0.529	0.00163	0.0216				1
TMEM135	transmembrane protein 135	11q14.2	0.941	0.00034	0.0054							1
EPR1	effector cell peptidase receptor 1 (non-protein coding)	17q25				-0.348	0.00214	0.0254				1
USP12	ubiquitin specific peptidase 12	13q12.13	0.94	2.00E-04	0.0036							1
EPS15L1	epidermal growth factor receptor pathway substrate 15-like 1	19p13.11	-0.404	5.00E-04	0.0072	-0.393322581	0.00512	0.032264516				2
PHLPP2	PH domain and leucine rich repeat protein phosphatase 2	16q22.2	0.939	0.00213	0.02							1
EPS8L1	EPS8-like 1	19q13.42	-1.774	0	1.00E-04	-0.3805	0.0048625	0.03695	0.698	2.705	8	3
EPS8L2	EPS8-like 2	11p15.5	-1.322	0	1.00E-04							1
TMEM229A	transmembrane protein 229A	7q31.32	0.939	0.03209	0.126							1
CBWD5	COBW domain containing 5	9q21.11	0.938	0.00329	0.0273				-0.71	2.71286	7	2
EPX	eosinophil peroxidase	17q23.1	-0.24	0.03299	0.1282	-0.302	0.00085	0.0163				2
EPYC	epiphycan	12q21				0.368	0.01316	0.0669				1
TGFBR2	transforming growth factor, beta receptor II (70/80kDa)	3p22	0.937	0.00043	0.0064				-0.891	2.546	5	2
ERAS	ES cell expressed Ras	Xp11.23							0.675	2.52	6	1

ERBB3	v-erb-b2 erythroblastic leukemia viral oncogene homolog 3 (avian)	12q13				-0.364	0.010285	0.0515				1
ERBB4	v-erb-a erythroblastic leukemia viral oncogene homolog 4 (avian)	2q33.3-q34				0.340454545	0.001443636	0.018				1
ERC1	ELKS/RAB6-interacting/CAST family member 1	12p13.3	-0.285	0.02225	0.0994							1
ERC2	ELKS/RAB6-interacting/CAST family member 2	3p14.3				-0.343909091	0.008760909	0.0453				1
ERCC1	excision repair cross-complementing rodent repair deficiency, complementati	19q13.32	-0.237	0.03623	0.1367	-0.414	0.00018	0.0084				2
TMEM230	transmembrane protein 230	20p13	0.937	9.00E-04	0.0109							1
MLEC	malectin	12q24.31	0.936	0.00182	0.0179							1
ERCC6	excision repair cross-complementing rodent repair deficiency, complementati	10q11.23	-0.312	0.04442	0.1556	-0.32	0.00604	0.0434				2
EREG	epiregulin	4q13.3	-1.828	0.00016	0.0031							1
CLDN12	claudin 12	7q21	0.935	0.00795	0.05	0.344	0.00018	0.0083				2
ERG	v-ets erythroblastosis virus E26 oncogene homolog (avian)	21q22.3				-0.343555556	0.00196	0.019022222				1
ERGIC1	endoplasmic reticulum-golgi intermediate compartment (ERGIC) 1	5q35.1				-0.400117647	0.000972941	0.013794118				1
ERGIC2	ERGIC and golgi 2	12p11.22				0.323	0.016313333	0.066366667				1
FERMT2	fermitin family member 2	14q22.1	0.935	0.04583	0.1587							1
TBC1D8B	TBC1 domain family, member 8B (with GRAM domain)	Xq22.3	0.935	2.00E-04	0.0036							1
ERI3	ERI1 exoribonuclease family member 3	1p32				-0.3345	0.014685	0.0705				1
CYBB	cytochrome b-245, beta polypeptide	Xp21.1	0.934	0.03328	0.1289	0.405	0.00179	0.0233				2
LRRC32	leucine rich repeat containing 32	11q13.5-q14	0.934	0.00198	0.0189							1
ERMAP	erythroblast membrane-associated protein (Scianna blood group)	1p34.2							-0.783	2.663	5	1
ERMP1	endoplasmic reticulum metallopeptidase 1	9p24	-0.878	0.00071	0.0092	-0.375875	0.001675	0.0197125				2
STAT1	signal transducer and activator of transcription 1, 91kDa	2q32.2	0.934	0.00263	0.0232							1
ERO1L	ERO1-like (S. cerevisiae)	14q22.1	-1.757	1.00E-04	0.0022							1
ATP9A	ATPase, class II, type 9A	20q13.2	0.933	9.00E-05	0.0021							1
ESAM	endothelial cell adhesion molecule	11q24.2	-0.596	0.01274	0.0688							1
BICD1	bicaudal D homolog 1 (Drosophila)	12p11.2-p11.1	0.933	0	0							1
ESD	esterase D	13q14.1-q14.2				0.3665	0.003235	0.02925				1
MSC	In multiple Geneids		0.932	0.00589	0.0408	0.325	1.00E-05	0.0034				2
CHRM3	cholinergic receptor, muscarinic 3	1q43	0.931	0.02162	0.0977	0.3365	0.00324	0.029383333				2
ESPL1	extra spindle pole bodies homolog 1 (S. cerevisiae)	12q	-1.319	0.00147	0.0153	-0.36	0.0007975	0.01585				2
ESPN	espin	1p36.31				-0.444	0.00059	0.0139				1
ESPNP	espin pseudogene	1p36.13	-0.49	0.00033	0.0053							1
ESR1	estrogen receptor 1	6q25.1				0.339	0.01599	0.0748				1
ESR2	estrogen receptor 2 (ER beta)	14q23.2				-0.338	0.007745	0.0456				1
ESRP1	epithelial splicing regulatory protein 1	8q22.1	-0.787	0.00044	0.0065							1
ESRP2	epithelial splicing regulatory protein 2	16q22.1	-1	0	1.00E-04	-0.356	0.0013	0.0199				2
ESRRB	estrogen-related receptor beta	14q24.3				-0.354	0.006621111	0.035655556				1
ESRRG	estrogen-related receptor gamma	1q41				0.352928571	0.00134619	0.01762619	-0.483	2.498	5	2
ESSPL	Data not found					-0.319	0.01516	0.07245				1
ESYT2	extended synaptotagmin-like protein 2	7q36.3				-0.314	0.01287	0.066				1
ETF1	eukaryotic translation termination factor 1	5q31.1	-0.715	4.00E-05	0.0012	-0.3305	0.00923	0.04995				2
ETFDH	electron-transferring-flavoprotein dehydrogenase	4q32-q35	-1.379	0	0							1
ETHE1	ethylmalonic encephalopathy 1	19q13.31	-0.601	0.0143	0.0742							1
PECR	peroxisomal trans-2-enoyl-CoA reductase	2q35	0.931	0.00083	0.0103							1
ETNK2	ethanolamine kinase 2	1q32.1	-1.849	0.00625	0.0425							1
MARCH3	membrane-associated ring finger (C3HC4) 3, E3 ubiquitin protein ligase	5q23.2	0.93	0.00761	0.0486							1
ETV1	ets variant 1	7p21.3				0.31375	0.0021475	0.0226	-0.784	2.97167	6	2
ETV3	ets variant 3	1q21-q23				0.345	0.00429	0.0361				1
PYCR1	pyrroline-5-carboxylate reductase 1	17q25.3	0.93	5.00E-05	0.0013							1
UGGT1	UDP-glucose glycoprotein glucosyltransferase 1	2q14.3	0.93	3.00E-05	9.00E-04							1
EVC	Ellis van Creveld syndrome	4p16				-0.351222222	0.007272222	0.039577778				1
EVC2	Ellis van Creveld syndrome 2	4p16.2-p16.1	-0.389	0.01939	0.0909	-0.334888889	0.005233333	0.036722222				2
EVI1	Data not found								-0.8715	2.95792	6	1
EVI2B	ecotropic viral integration site 2B	17q11.2							-0.764	2.608	5	1
EVI5	ecotropic viral integration site 5	1p22.1							-0.969	2.90667	6	1
EVI5L	ecotropic viral integration site 5-like	19p13.2	-0.369	0.00162	0.0164	-0.424857143	0.002758571	0.023271429				2
EVL	Enah/Vasp-like	14q32.2				-0.347	0.00766	0.0495				1
EVPL	envoplakin	17q25	-2.379	0	0				0.695	2.664	5	2
EVX1	even-skipped homeobox 1	7p15.2							0.751	2.85714	7	1
EVX2	In multiple Geneids		-0.255	0.0373	0.1392							1
EWSR1	Ewing sarcoma breakpoint region 1	22q12.2				-0.405125	0.00109	0.0164375				1
EXD1	exonuclease 3'-5' domain containing 1	15q15.1				-0.3548	0.011296	0.05482				1
EXD2	exonuclease 3'-5' domain containing 2	14q24.1	-0.753	0.00019	0.0035							1
EXD3	exonuclease 3'-5' domain containing 3	9q34.3				-0.335	0.00402	0.0349				1
EXOC1	exocyst complex component 1	4q12	-0.756	0.00118	0.0131							1
EXOC3	exocyst complex component 3	5p15.33				0.304	0.02014	0.0856				1
EXOC3L2	exocyst complex component 3-like 2	19q13.32				-0.339	0.01281	0.0658				1
EXOC4	exocyst complex component 4	7q31				0.302	7.00E-05	0.0056				1
EXOC5	exocyst complex component 5	14q22.3	-0.479	0.00309	0.0262							1
TMEM139	transmembrane protein 139	7q34	0.929	0.00063	0.0085							1

EXOC6B	exocyst complex component 6B	2p13.2				0.342285714	0.000344286	0.008028571				1
EXOC7	exocyst complex component 7	17q25.1	-0.445	0.00753	0.0482							1
EXOC8	exocyst complex component 8	1q42.2							-0.691	2.42286	7	1
EXOSC1	exosome component 1	10q24				-0.4085	0.00112	0.014				1
EXOSC10	exosome component 10	1p36.22				-0.40825	0.0014425	0.018375				1
EXOSC2	exosome component 2	9q34				-0.321	0.00374	0.0336				1
EXOSC3	exosome component 3	9p11				-0.452	8.00E-05	0.0059				1
EXOSC5	exosome component 5	19q13.1				-0.314	0.00438	0.033566667				1
EXOSC7	exosome component 7	3p21.31				-0.409	0.00025	0.0096				1
ADAM9	ADAM metallopeptidase domain 9	8p11.22	0.928	0.00023	0.0041							1
EXPH5	exophilin 5	11q22.3	-1.491	0.00039	0.006							1
GAS2L3	growth arrest-specific 2 like 3	12q23.1	0.927	0.0064	0.0432							1
RIPK2	receptor-interacting serine-threonine kinase 2	8q21	0.927	4.00E-04	0.0061							1
EXTL1	exostoses (multiple)-like 1	1p36.1				-0.377666667	0.014913333	0.061633333				1
CCPG1	cell cycle progression 1	15q21.1	0.926	0.01331	0.0706							1
EXTL3	exostoses (multiple)-like 3	8p21				-0.32	0.00165	0.0223	0.882	2.92	5	2
EYA1	eyes absent homolog 1 (Drosophila)	8q13.3				0.362066667	0.002163333	0.021573333	-0.874	2.75	6	2
EYA2	eyes absent homolog 2 (Drosophila)	20q13.1				0.3465	0.002095	0.0211				1
EYA3	eyes absent homolog 3 (Drosophila)	1p36	-0.691	0.00237	0.0215	-0.3904	0.004698	0.03387				2
MYRFL	Data not found		0.926	0.04041	0.1465							1
EYS	eyes shut homolog (Drosophila)	6q12				0.383909091	0.009552727	0.050127273				1
EZH1	enhancer of zeste homolog 1 (Drosophila)	17q21.1-q21.3				-0.3785	0.00124	0.0184				1
F11	coagulation factor XI	4q35				-0.464	0.0037	0.0335				1
F13A1	coagulation factor XIII, A1 polypeptide	6p25.3-p24.3				-0.332	0.003386667	0.025366667				1
F13B	coagulation factor XIII, B polypeptide	1q31-q32.1				0.3395	0.00155	0.0216	-0.989	3.785	8	2
F2	coagulation factor II (thrombin)	11p11				-0.31	0.01899	0.0827				1
PPAP2C	phosphatidic acid phosphatase type 2C	19p13	0.926	0.00051	0.0073							1
C3orf52	chromosome 3 open reading frame 52	3q13.2	0.925	0.00507	0.0368							1
PLK1S1	polo-like kinase 1 substrate 1	20p11.23	0.925	0.00094	0.0112							1
HLA-B	major histocompatibility complex, class I, B	6p21.3	0.924	0.002545	0.0201							1
F8A1	coagulation factor VIII-associated 1	Xq28							0.658	2.81	6	1
F9	coagulation factor IX	Xq27.1-q27.2							-0.628	2.78571	7	1
FA2H	fatty acid 2-hydroxylase	16q23				-0.377555556	0.004322222	0.031622222				1
FAAH	fatty acid amide hydrolase	1p35-p34				-0.392	0.00055	0.0135				1
IRF1	interferon regulatory factor 1	5q31.1	0.924	0.00147	0.0153							1
PSMB8	proteasome (prosome, macropain) subunit, beta type, 8 (large multifunctiona	6p21.3	0.922	0.00011	0.0024							1
FABP12	fatty acid binding protein 12	8q21.13	-0.681	2.00E-05	7.00E-04							1
FABP2	fatty acid binding protein 2, intestinal	4q28-q31							-0.805	3.0425	8	1
RND1	Rho family GTPase 1	12q12	0.922	0.00571	0.04							1
FABP4	fatty acid binding protein 4, adipocyte	8q21							-0.918	2.595	6	1
FABP5	fatty acid binding protein 5 (psoriasis-associated)	8q21.13	-2.7645	3.00E-05	8.00E-04							1
FABP5L3	Data not found					-0.41	0.00219	0.0257				1
FABP6	fatty acid binding protein 6, ileal	5q33.3-q34				-0.438833333	0.000936667	0.013783333				1
STIL	SCL/TAL1 interrupting locus	1p32	0.921	0.01223	0.0671							1
OSGIN2	oxidative stress induced growth inhibitor family member 2	8q21	0.9205	0.00121	0.01165							1
TRIO	triple functional domain (PTPRF interacting)	5p15.2	0.916	2.00E-05	8.00E-04	0.350238095	0.004947143	0.035392857				2
FADS6	fatty acid desaturase domain family, member 6	17q25.1	-0.225	0.04966	0.1664							1
FAF1	Fas (TNFRSF6) associated factor 1	1p33				-0.351	0.00169	0.0226				1
APOE	apolipoprotein E	19q13.2	0.915	0.00091	0.011							1
FAHD1	fumarylacetoacetate hydrolase domain containing 1	16p13.3				-0.4725	0.01609	0.071				1
FAIM2	Fas apoptotic inhibitory molecule 2	12q13				-0.34	8.00E-05	0.006				1
FAIM3	Fas apoptotic inhibitory molecule 3	1q32.1				0.323	0.00146	0.0211				1
ELTD1	EGF, latrophilin and seven transmembrane domain containing 1	1p33-p32	0.915	0.00072	0.0093							1
PSMB4	proteasome (prosome, macropain) subunit, beta type, 4	1q21	0.915	6.00E-05	0.0015							1
FAM102A	family with sequence similarity 102, member A	9q34.11	-0.566	0.01995	0.0925							1
PLXND1	In multiple Geneids		0.914	2.00E-05	6.00E-04							1
FAM104B	family with sequence similarity 104, member B	Xp11.21	-0.4	0.02971	0.1198							1
CHST5	carbohydrate (N-acetylglucosamine 6-O) sulfotransferase 5	16q22.3	0.912	0.04809	0.1632							1
FAM105B	family with sequence similarity 105, member B	5p15.2				0.366	0.004413333	0.034666667				1
FAM107A	family with sequence similarity 107, member A	3p21.1	-0.371	0.00512	0.0371	-0.429333333	0.001986667	0.021433333				2
FAM107B	family with sequence similarity 107, member B	10p13				0.76	1.00E-05	0.0033				1
FAM110A	family with sequence similarity 110, member A	20p13	-0.473	0.00825	0.0512							1
FAM110B	family with sequence similarity 110, member B	8q12.1				0.359333333	0.01684	0.0654				1
FAM110C	family with sequence similarity 110, member C	2p25.3				0.4675	0.001795	0.02265				1
KRAS	v-Ki-ras2 Kirsten rat sarcoma viral oncogene homolog	12p12.1	0.912	0.04567	0.1584	0.69725	0.0293425	0.10395				2
FAM114A2	family with sequence similarity 114, member A2	5q31-q33	-0.377	0.01048	0.0605	-0.324	6.00E-05	0.0054				2
FAM115A	family with sequence similarity 115, member A	7q35				-0.332	0.00134	0.0202				1
FAM115C	family with sequence similarity 115, member C	7q35	-1.198	5.00E-05	0.00115							1
FAM116A	family with sequence similarity 116, member A	3p14.3				-0.381	0.001133333	0.0147				1

FAM117B	family with sequence similarity 117, member B	2q33.2	-0.486	0.0147	0.0755							1
FAM118A	family with sequence similarity 118, member A	22q13				-0.3836	0.000505	0.01198				1
SLC13A2	solute carrier family 13 (sodium-dependent dicarboxylate transporter), memb	17p13.2	0.912	0.00665	0.0443							1
AHR	aryl hydrocarbon receptor	7p15	0.911	0.00034	0.0054	0.33925	0.003645	0.031375				2
FAM120B	family with sequence similarity 120B	6q27				-0.504	0.00011	0.0069				1
FAM120C	family with sequence similarity 120C	Xp11.22	-0.707	0.0039	0.0307							1
NMI	N-myc (and STAT) interactor	2q23	0.911	0.00289	0.0249				-1.03	2.82667	6	2
FAM123B	family with sequence similarity 123B	Xq11.2				0.322	0.00492	0.0389				1
FAM124A	family with sequence similarity 124A	13q14.3				0.3525	0.009855	0.04985				1
FAM124B	family with sequence similarity 124B	2q36.2	-0.339	0.01187	0.0657							1
FAM125A	family with sequence similarity 125, member A	19p13.11				-0.474	0.00068	0.0137				1
FAM126A	family with sequence similarity 126, member A	7p15.3				0.308	0.00094	0.0171				1
FAM127C	family with sequence similarity 127, member C	Xq26.3	-0.687	0.00096	0.0114							1
FAM129A	family with sequence similarity 129, member A	1q25				0.363	0.00024	0.0094				1
FAM129B	family with sequence similarity 129, member B	9q34.13	-1.934	0	2.00E-04	-0.402	0.00381	0.0336				2
FAM129C	family with sequence similarity 129, member C	19p13.11				-0.436714286	0.003167143	0.0234				1
FAM131C	family with sequence similarity 131, member C	1p36.13	-0.301	0.00782	0.0494	-0.377	0.00137	0.0204				2
FAM132A	family with sequence similarity 132, member A	1p36.33				-0.509	0.00024	0.0094				1
NOX4	NADPH oxidase 4	11q14.2-q21	0.911	0.00263	0.0232	0.301	0.03574	0.1208				2
PTP4A2	protein tyrosine phosphatase type IVA, member 2	1p35	0.911	4.00E-05	0.0011							1
FAM134C	family with sequence similarity 134, member C	17q21.2	-0.476	0.00256	0.0228	-0.361	0.00214	0.0254				2
FAM135A	family with sequence similarity 135, member A	6q13	-1.58	3.00E-05	9.00E-04							1
FAM135B	family with sequence similarity 135, member B	8q24.23				0.307	0.00703	0.0472				1
FAM13A	family with sequence similarity 13, member A	4q22.1				-0.3095	0.008165	0.042				1
FAM13A1	Data not found								-0.873	2.97556	9	1
FAM13B	family with sequence similarity 13, member B	5q31				-0.3801	0.002262	0.02231				1
FAM149A	family with sequence similarity 149, member A	4q35.1				-0.36325	0.00889	0.049325				1
FAM149B1	family with sequence similarity 149, member B1	10q22.2	-0.365	0.017	0.0832							1
RASA3	RAS p21 protein activator 3	13q34	0.911	0.00083	0.0103							1
FAM150B	family with sequence similarity 150, member B	2p25.3				0.441	0.00017	0.008				1
FAM151A	family with sequence similarity 151, member A					-0.4425	0.001325	0.0197				1
FAM151B	family with sequence similarity 151, member B	5q14.1				-0.349	0.00457	0.0374				1
FAM153A	family with sequence similarity 153, member A	5q35.3				-0.346	0.004595	0.0322				1
FAM153B	family with sequence similarity 153, member B	5q35.2	-0.45	0.00066	0.0087	-0.4	0.00049	0.0127				2
FAM153C	family with sequence similarity 153, member C	5q35.3				-0.32	0.004286667	0.028733333				1
FAM154A	family with sequence similarity 154, member A	9p22.1				-0.400923077	0.007110769	0.035646154				1
FAM155A	family with sequence similarity 155, member A	13q33.3				0.354142857	0.017148571	0.070885714				1
ABCC9	ATP-binding cassette, sub-family C (CFTR/MRP), member 9	12p12.1	0.91	0.03509	0.1335	0.350266667	0.001599333	0.018126667	-0.946	2.9025	8	3
FAM159A	family with sequence similarity 159, member A	1p32.3	-0.299	0.02235	0.0996	-0.375	0.00795	0.0505				2
FAM160A1	family with sequence similarity 160, member A1	4q31.3	-1.637	0	0							1
EYA4	eyes absent homolog 4 (Drosophila)	6q23	0.91	0.00878	0.0534	0.3365	0.0150175	0.0714				2
FAM160B2	family with sequence similarity 160, member B2	8p21.3				-0.346	0.00509	0.0396				1
FAM161B	family with sequence similarity 161, member B	14q24.3				-0.3985	0.00307	0.02845				1
FAM162A	family with sequence similarity 162, member A	3q21.1	-0.715	0.00108	0.0123							1
FAM164A	Data not found					0.3665	0.00041	0.01015				1
FAM164C	Data not found					-0.373	0.00367	0.0334				1
FAM166B	family with sequence similarity 166, member B	9p13.3				-0.426	6.00E-05	0.0056				1
SEMA3B	sema domain, immunoglobulin domain (Ig), short basic domain, secreted, (ser	3p21.3	0.908	0.0037	0.0297							1
FAM169A	family with sequence similarity 169, member A	5q13.3	-0.244	0.04387	0.1543	-0.362	0.003213333	0.027366667				2
FAM170A	family with sequence similarity 170, member A	5q23.1	-0.261	0.00776	0.0492							1
FAM171A1	family with sequence similarity 171, member A1	10p13				-0.3755	0.01106375	0.0569125				1
FAM171A2	family with sequence similarity 171, member A2	17q21.31	-0.449	0.01041	0.0602							1
EFNB2	ephrin-B2	13q33	0.907	0.01516	0.0772	0.316	0.01422	0.0699				2
FAM172A	family with sequence similarity 172, member A	5q15	-0.649	0.00641	0.0432							1
FKBP9	FK506 binding protein 9, 63 kDa	7p11.1	0.907	4.00E-05	0.0012							1
FAM174A	family with sequence similarity 174, member A	5q21.1	-0.48	0.00074	0.0095							1
FAM175A	family with sequence similarity 175, member A	4q21.23	-0.569	0.01078	0.0615	-0.3305	0.00497	0.037				2
FAM175B	family with sequence similarity 175, member B	10q26.13				-0.32	0	0.0023				1
FAM176A	family with sequence similarity 176, member A	2p12				0.338	0.00022	0.00895				1
FAM178A	family with sequence similarity 178, member A	10q24.31				-0.315	0.00011	0.0067				1
FAM179A	family with sequence similarity 179, member A	2p23.2	-0.243	0.01921	0.0904							1
FAM180B	family with sequence similarity 180, member B	11p11.2	-0.272	0.04285	0.1521							1
FAM181A	family with sequence similarity 181, member A	14q32.12	-0.225	0.02584	0.1097							1
FAM182A	family with sequence similarity 182, member A	20p11.1	-0.404	0.00905	0.0545							1
FAM184B	family with sequence similarity 184, member B	4p16				-0.359578947	0.002315789	0.022578947				1
FAM186A	family with sequence similarity 186, member A	12q13.12	-0.285	0.0218	0.0982	-0.397666667	0.002166667	0.023633333				2
FAM188A	family with sequence similarity 188, member A	10p13	-0.45	0.02912	0.1182							1
LCLAT1	lysocardiolipin acyltransferase 1	2p23.1	0.907	0	1.00E-04	0.3515	0.00043	0.0118				2
FAM189A1	family with sequence similarity 189, member A1	15q13.1				-0.3758	0.00441	0.03178				1

FAM189A2	family with sequence similarity 189, member A2	9q21.11				0.329	0.00519	0.04				1
FAM18A	In multiple Geneids					-0.379	0.00461	0.03045				1
FAM18B2	family with sequence similarity 18, member B2	17p12				-0.365	0.00863	0.0464				1
FAM190A	family with sequence similarity 190, member A	4q22.1				-0.332	0.00505	0.0394				1
FAM194A	family with sequence similarity 194, member A	3q25.1	-0.239	0.00155	0.016							1
DLEU2	deleted in lymphocytic leukemia 2 (non-protein coding)	13q14.3	0.906	0.00229	0.021							1
FAM211A	family with sequence similarity 211, member A	17p11.2	0.906	0.00011	0.0023							1
FYN	FYN oncogene related to SRC, FGR, YES	6q21	0.906	0.00075	0.0096							1
FAM19A1	In multiple Geneids								-1	3.19222	9	1
FAM19A2	family with sequence similarity 19 (chemokine (C-C motif)-like), member A2	12q14.1				0.382	0.00208	0.025	-0.852	3.17571	7	2
FAM19A3	family with sequence similarity 19 (chemokine (C-C motif)-like), member A3	1p13.2				-0.344	0.00189	0.0239				1
FAM19A4	In multiple Geneids					0.316	0.01412	0.0696				1
FAM19A5	family with sequence similarity 19 (chemokine (C-C motif)-like), member A5	22q13.32				-0.4	0.001467368	0.015810526				1
FAM203A	family with sequence similarity 203, member A	8q24.3	-0.638	5.00E-05	0.0013							1
ITGA3	integrin, alpha 3 (antigen CD49C, alpha 3 subunit of VLA-3 receptor)	17q21.33	0.906	0.00445	0.0337							1
CEBPB	CCAAT/enhancer binding protein (C/EBP), beta	20q13.1	0.905	4.00E-05	0.0011							1
WDR72	WD repeat domain 72	15q21.3	0.905	0.00045	0.0067							1
TTLL6	tubulin tyrosine ligase-like family, member 6	17q21.32	0.904	0.00069	0.0091							1
FAM212A	family with sequence similarity 212, member A	3p21.31	-1.13	0	0							1
FAM213A	family with sequence similarity 213, member A	10q23.1	-0.628	0.01142	0.064							1
KDELC1	KDEL (Lys-Asp-Glu-Leu) containing 1	13q33	0.903	0.00032	0.0051	0.321	0.0243	0.0957	-0.882	2.732	5	3
FAM214A	family with sequence similarity 214, member A	15q21.2-q21.3	-1.481	0	0							1
FAM214B	family with sequence similarity 214, member B	9p13.3	-0.67	0.00348	0.0283							1
FAM217A	family with sequence similarity 217, member A	6p25.2	-0.209	0.04738	0.1618							1
SNORD82	small nucleolar RNA, C/D box 82	2q37.1	0.903	0.02633	0.111							1
FAM219B	family with sequence similarity 219, member B	15q24.1	-0.648	0.00313	0.0264							1
XKR9	XK, Kell blood group complex subunit-related family, member 9	8q13.3	0.903	0.00081	0.0101	0.32	0.00727	0.0481				2
C5orf34	chromosome 5 open reading frame 34	5p12	0.902	0.00647	0.0434							1
FAM23A	family with sequence similarity 23, member A					1.475	0	1.00E-04				1
FAM24A	family with sequence similarity 24, member A	10q26.13							-0.852	3.515	8	1
ITGA6	integrin, alpha 6	2q31.1	0.902	0.0031	0.0262							1
FAM25B	In multiple Geneids		-3.992	0	0							1
FAM26C	Data not found								0.907	2.476	5	1
LOC100508226	HHSL751		0.902	0.01051	0.0606							1
FAM27A	family with sequence similarity 27, member A	9q12	-0.362	0.02374	0.1034							1
FAM32A	family with sequence similarity 32, member A	19pter-p13.3				-0.3405	0.005465	0.03805				1
FAM33A	Data not found					-0.39	0.00095	0.0172				1
FAM38A	Data not found					-0.46	0.002545	0.02735				1
FAM3B	family with sequence similarity 3, member B	21q22.3	-1.154	0.00024	0.0042	-0.3615	0.00681	0.0441				2
TSPAN12	tetraspanin 12	7q31.31	0.902	0.02252	0.1	0.46	5.00E-05	0.005				2
FAM3D	family with sequence similarity 3, member D	3p14.2	-1.7	0	1.00E-04	-0.486	6.00E-05	0.0056				2
FAM41AY1	family with sequence similarity 41, member A, Y-linked 1	Yq11.221				-0.415833333	0.0122	0.06165				1
FAM41AY2	family with sequence similarity 41, member A, Y-linked 2	Yq11.2				-0.415833333	0.0122	0.06165				1
FAM43A	family with sequence similarity 43, member A	3q29							0.869	2.722	5	1
FAM45A	family with sequence similarity 45, member A	10q25	-0.675	0.01348	0.0713	-0.301	0.00236	0.0267				2
FAM45B	family with sequence similarity 45, member B (pseudogene)	Xq26.1	-0.515	0.00846	0.0521	-0.301	0.00236	0.0267				2
C8orf31	chromosome 8 open reading frame 31	8q24.3	0.9	0.00071	0.0092							1
FAM46B	family with sequence similarity 46, member B	1p36.11	-2.166	0	0	-0.644	0.00022	0.0091				2
FAM46D	family with sequence similarity 46, member D	Xq21.1							-0.788	2.39667	6	1
FAM47C	family with sequence similarity 47, member C	Xp21.1	-0.261	0.01844	0.0879							1
ALG11	asparagine-linked glycosylation 11, alpha-1,2-mannosyltransferase homolog (\	13q14.2	0.899	0.00057	0.0079							1
FAM48B2	In multiple Geneids					-0.32	0.0023	0.0263				1
BPNT1	3'(2'), 5'-bisphosphate nucleotidase 1	1q41	0.899	1.00E-05	5.00E-04				-0.956	2.551	5	2
UBXN10	UBX domain protein 10	1p36.12	0.899	0.01352	0.0714							1
COL15A1	collagen, type XV, alpha 1	9q21-q22	0.897	0.00013	0.0026							1
FAM53A	family with sequence similarity 53, member A	4p16.3				-0.4145	0.0033325	0.025175				1
FAM53B	family with sequence similarity 53, member B	10q26.13	-0.445	0.00481	0.0355	-0.331	0.001195	0.0163				2
FAM53C	family with sequence similarity 53, member C	5q31				-0.375333333	0.008786667	0.0441				1
FAM54A	family with sequence similarity 54, member A	6q23.3							-0.889	2.934	5	1
FAM54B	family with sequence similarity 54, member B	1p36.11				-0.343	0.010456667	0.054633333				1
FAM55A	family with sequence similarity 55, member A	11q23.2				0.339	8.00E-05	0.0061				1
FAM55B	family with sequence similarity 55, member B	11q23.3							-0.872	3.20333	6	1
FAM55D	family with sequence similarity 55, member D	11q23.2				0.419	0.00017	0.0082	-0.838	3.20333	9	2
FAM57A	family with sequence similarity 57, member A	17p13.3	-0.469	0.0226	0.1003	-0.456	0.021305	0.0751				2
GATM	glycine amidinotransferase (L-arginine:glycine amidinotransferase)	15q21.1	0.897	0.03101	0.1234							1
FAM59A	family with sequence similarity 59, member A	18q12.1				-0.323	0.01207	0.0633				1
FAM5B	family with sequence similarity 5, member B	1q24				0.445	0.00014	0.0075				1
FAM5C	family with sequence similarity 5, member C	1q31.1				0.363688889	0.003414222	0.027908889				1
TAF1A	TATA box binding protein (TBP)-associated factor, RNA polymerase I, A, 48kDa	1q42	0.897	0.00027	0.0045							1

FAM63A	family with sequence similarity 63, member A	1q21.3	-1.059	0.00015	0.003							1
FAM63B	family with sequence similarity 63, member B	15q21.3				-0.36	0.01363	0.0682				1
FAM65A	family with sequence similarity 65, member A	16q22.1				-0.455666667	0.001833333	0.020066667				1
FAM69A	family with sequence similarity 69, member A	1p22.1				-0.3205	0.004085	0.0348				1
FAM70A	family with sequence similarity 70, member A	Xq24				1.047	0	5.00E-04				1
FAM71A	family with sequence similarity 71, member A	1q32.3	-0.225	0.033435	0.12955							1
FAM71B	family with sequence similarity 71, member B	5q33.3	-0.255	0.00569	0.0399	-0.348	0.00088	0.0166				2
FAM71C	family with sequence similarity 71, member C	12q23.1							-1.113	2.672	5	1
FAM71E1	family with sequence similarity 71, member E1	19q13.33				-0.388	0.00059	0.0139				1
FAM71E2	family with sequence similarity 71, member E2	19q13.42				-0.308	0.00542	0.0409				1
ANTXR1	anthrax toxin receptor 1	2p13.1	0.896	0.04414	0.155	0.339714286	0.004281429	0.023414286				2
CENPF	centromere protein F, 350/400kDa (mitosin)	1q32-q41	0.895	0.04435	0.1554							1
FAM73B	family with sequence similarity 73, member B	9q34.11	-0.251	0.03883	0.1428	-0.353	0.00398	0.0347				2
FAM76A	family with sequence similarity 76, member A	1p35.3				-0.390857143	0.010872857	0.055685714				1
FAM79B	Data not found								-0.77	2.67833	6	1
FAM81A	family with sequence similarity 81, member A	15q22.2				-0.308	0.00062	0.0142				1
FAM81B	family with sequence similarity 81, member B	5q15							-0.9	3.354	5	1
FAM82A1	family with sequence similarity 82, member A1	2p22.2				0.3212	0.000668	0.01434				1
FAM82A2	family with sequence similarity 82, member A2	15q15.1				-0.375666667	0.00404	0.0306				1
FAM82B	family with sequence similarity 82, member B	8q21.3				0.303	0.00951	0.0557				1
FAM83A	family with sequence similarity 83, member A	8q24.13	-2.451	8.00E-05	0.0018							1
FAM83A-AS1	Data not found		-0.601	3.00E-05	8.00E-04							1
FAM83B	family with sequence similarity 83, member B	6p12.1	-1.122	0.0248	0.1065							1
FAM83C	family with sequence similarity 83, member C	20q11.22	-2.769	0	0				0.875	2.73111	9	2
FAM83D	family with sequence similarity 83, member D	20q11.22-q12	-1.308	0.00348	0.0283							1
EIF5A2	eukaryotic translation initiation factor 5A2	3q26.2	0.895	0.00657	0.0439	0.382	0.00221	0.0258				2
FAM83F	family with sequence similarity 83, member F	22q13.1	-0.835	0	0	-0.385125	0.00103875	0.014175				2
FAM83G	family with sequence similarity 83, member G	17p11.2	-0.931	1.00E-05	5.00E-04	-0.518	0.00047	0.0126				2
FAM83H	family with sequence similarity 83, member H	8q24.3	-0.474	0.03176	0.1252							1
FAM86B1	family with sequence similarity 86, member B1	8p23.1							0.587	2.734165	6	1
HSPA6	heat shock 70kDa protein 6 (HSP70B')	1q23	0.895	0.01891	0.0895							1
FAM89B	family with sequence similarity 89, member B	11q13	-0.397	0.02442	0.1053	-0.301	0.00247	0.0272				2
FAM8A1	family with sequence similarity 8, member A1	6p23				-0.388	0.02764	0.1033				1
FAM90A1	family with sequence similarity 90, member A1	12p13.31							0.76	3.10833	6	1
MYO15B	In multiple Geneids		0.895	0.002755	0.0209							1
ORAI2	ORAI calcium release-activated calcium modulator 2	7q22.1	0.895	0.00054	0.0076							1
FAM92A1P2	family with sequence similarity 92, member A1 pseudogene 2	4q35.1	-0.239	0.02179	0.0982							1
FAM92A3	Data not found					-0.622	0.00076	0.0155				1
FAM95B1	In multiple Geneids		-0.9045	0.00029	0.0043							1
ITGBL1	integrin, beta-like 1 (with EGF-like repeat domains)	13q33	0.894	0.02543	0.1084	0.3235	0.00203	0.02445				2
FAM98B	family with sequence similarity 98, member B	15q14							-0.826	2.61333	6	1
FAM99A	family with sequence similarity 99, member A (non-protein coding)	11p15.5	-0.506	0.00698	0.0458							1
CD55	CD55 molecule, decay accelerating factor for complement (Cromer blood grou	1q32	0.893	0.01536	0.0778	0.314	0.00157	0.0217				2
PLEKHA3	pleckstrin homology domain containing, family A (phosphoinositide binding sp	2q31.2	0.893	0.00027	0.0045	0.4	0.01999	0.0852				2
FANCD2	Fanconi anemia, complementation group D2	3p26				-0.3335	0.0066025	0.04035				1
FANCD2OS	Data not found		-0.257	0.01443	0.0746							1
RAB17	RAB17, member RAS oncogene family	2q37.3	0.893	0.00076	0.0096							1
RPS6KA3	ribosomal protein S6 kinase, 90kDa, polypeptide 3	Xp22.2-p22.1	0.893	0.00333	0.0276	0.346666667	0.005373333	0.038866667				2
ANKH	ankylosis, progressive homolog (mouse)	5p15.1	0.892	0.00129	0.0141	0.35	0.007155	0.04755				2
FANCM	Fanconi anemia, complementation group M	14q21.2							-0.896	2.65625	8	1
FANK1	fibronectin type III and ankyrin repeat domains 1	10q26.2	-0.376	0.00623	0.0424							1
ANKRD36	ankyrin repeat domain 36	2q11.2	0.892	0.01118	0.05705							1
FAR1	fatty acyl CoA reductase 1	11p15.2	-0.927	0.00048	0.0069							1
NCEH1	neutral cholesterol ester hydrolase 1	3q26.31	0.892	0.00136	0.0146							1
RAB3IP	RAB3A interacting protein (rabin3)	12q14.3	0.892	2.00E-05	6.00E-04							1
FARP2	FERM, RhoGEF and pleckstrin domain protein 2	2q37.3				-0.31	0.00513	0.0397				1
FARS2	phenylalanyl-tRNA synthetase 2, mitochondrial	6p25.1				-0.326833333	0.00373	0.031533333				1
FARSA	phenylalanyl-tRNA synthetase, alpha subunit	19p13.2				-0.3555	0.003155	0.03075				1
FAS	Fas (TNF receptor superfamily, member 6)	10q24.1	-0.69	0.02261	0.1003							1
FASLG	Fas ligand (TNF superfamily, member 6)	1q23				0.375	0.00075	0.0155				1
FASTK	Fas-activated serine/threonine kinase	7q35	-0.237	0.03059	0.1223							1
SNORD42B	small nucleolar RNA, C/D box 42B	17q11	0.892	0.00163	0.0165							1
ANO6	anoctamin 6	12q12	0.891	0.00041	0.0062							1
FAT2	FAT tumor suppressor homolog 2 (Drosophila)	5q33.1	-3.863	0	0	-0.304	0.00281	0.029				2
FAT3	FAT tumor suppressor homolog 3 (Drosophila)	11q14.3				0.412	2.00E-05	0.0039				1
FBF1	Fas (TNFRSF6) binding factor 1	17q25.1	-0.303	0.00216	0.0202	-0.366	0.0038	0.03325				2
FBL	fibrillarin	19q13.1							-0.754	2.606	5	1
IQCE	IQ motif containing E	7p22.3	0.891	0.00024	0.0042							1
FBLN1	fibulin 1	22q13.31				-0.398923077	0.000553077	0.010592308				1

FBLN2	fibulin 2	3p25.1				-0.3325	0.005245	0.034425				1
FBLN5	In multiple Geneids					-0.322333333	0.006191667	0.030833333				1
SEC23A	Sec23 homolog A (S. cerevisiae)	14q21.1	0.891	0	2.00E-04							1
SELM	selenoprotein M	22q12.2	0.891	9.00E-05	0.0021							1
FBN3	In multiple Geneids					-0.4408	0.003635333	0.027173333				1
RRN3P1	RNA polymerase I transcription factor homolog (S. cerevisiae) pseudogene 1	16p12.2	0.8905	0.00065	0.0077							1
FBP2	fructose-1,6-bisphosphatase 2	9q22.3				-0.377	0.012445	0.0578				1
FBRS	fibrosin	16p11.2	-0.478	0.00124	0.0136							1
FBXL12	F-box and leucine-rich repeat protein 12	19p13.2				-0.324	0.00069	0.0149				1
FBXL15	F-box and leucine-rich repeat protein 15	10q24.32				-0.361	0.00079	0.0158				1
FBXL16	F-box and leucine-rich repeat protein 16	16p13.3	-0.3655	0.01863	0.0746							1
FBXL17	F-box and leucine-rich repeat protein 17	5q21.3	-0.336	0.00059	0.0081	-0.341	0.00179	0.0232	0.618	2.42167	6	3
FBXL18	F-box and leucine-rich repeat protein 18	7p22.2				-0.452	0.00366	0.03175				1
FBXL19	F-box and leucine-rich repeat protein 19	16p11.2				-0.304	0.0018	0.0233				1
AKR1E2	aldo-keto reductase family 1, member E2	10p15.1	0.89	0.01701	0.0833							1
FBXL21	F-box and leucine-rich repeat protein 21 (gene/pseudogene)	5q31							-1.018	3.12667	6	1
FBXL7	F-box and leucine-rich repeat protein 7	5p15.1				0.350807018	0.005853333	0.037226316				1
FBXL8	F-box and leucine-rich repeat protein 8	16q22.1				-0.391	0.00125	0.0195				1
FBXO10	F-box protein 10	9p13.2				-0.408777778	0.000844444	0.012433333				1
FBXO11	In multiple Geneids					-0.311	0.0075	0.0489				1
FBXO15	F-box protein 15	18q22.3				-0.314	0.00066	0.0146				1
FBXO16	F-box protein 16	8p21.1				-0.3225	0.0019	0.02385				1
SLC37A3	solute carrier family 37 (glycerol-3-phosphate transporter), member 3	7q34	0.89	2.00E-05	6.00E-04							1
FBXO2	F-box protein 2	1p36.22				-0.305	0.01921	0.0833				1
GPX8	glutathione peroxidase 8 (putative)	5q11.2	0.888	0.01617	0.0805							1
LSR	lipolysis stimulated lipoprotein receptor	19q13.12	0.887	0	2.00E-04							1
FBXO24	F-box protein 24	7q22				-0.326	0.04278	0.1351				1
GNPDA1	glucosamine-6-phosphate deaminase 1	5q21	0.886	0.00017	0.0032							1
FBXO27	F-box protein 27	19q13.2	-1.333	0.00022	0.0039	-0.341	0.00166	0.0224				2
FBXO3	F-box protein 3	11p13	-0.499	0.0029	0.0249							1
FBXO31	In multiple Geneids					-0.444428571	0.001267143	0.017542857				1
FBXO32	F-box protein 32	8q24.13				0.335	0.01878	0.0821				1
FBXO34	F-box protein 34	14q22.3	-0.878	0.00032	0.0051	-0.382	1.00E-05	0.0032				2
FBXO39	F-box protein 39	17p13.1				-0.37625	0.002105	0.02185				1
FBXO4	F-box protein 4	5p12				0.455333333	0.005965556	0.040766667				1
FBXO42	F-box protein 42	1p36.23-p36.11	-0.572	2.00E-05	7.00E-04	-0.366571429	0.007741429	0.046785714				2
FBXO44	F-box protein 44	1p36.22				-0.4	0.00361	0.0331				1
FBXO46	F-box protein 46	19q13.3				-0.3	0.01072	0.0595				1
FBXO48	F-box protein 48	2p13.3	-0.252	0.02585	0.1097							1
FBXO7	F-box protein 7	22q12-q13				-0.3485	0.000915	0.01645				1
FBXO8	F-box protein 8	4q34.1							-0.804	2.95667	6	1
FBXW10	F-box and WD repeat domain containing 10	17p12	-0.938	0.00022	0.004	-0.41775	0.0013925	0.015575				2
FBXW11	F-box and WD repeat domain containing 11	5q35.1	-0.575	3.00E-05	9.00E-04	-0.3375	0.0060475	0.031983333				2
FBXW4	F-box and WD repeat domain containing 4	10q24	-0.554	0.00788	0.0497	-0.356111111	0.001193333	0.0174				2
FBXW5	F-box and WD repeat domain containing 5	9q34.3	-0.266	0.01328	0.0705							1
FBXW7	F-box and WD repeat domain containing 7, E3 ubiquitin protein ligase	4q31.3							-0.882	2.879835	5.5	1
FCAR	Fc fragment of IgA, receptor for	19q13.42							-0.738	2.71	6	1
FCER1A	Fc fragment of IgE, high affinity I, receptor for; alpha polypeptide	1q23							-0.995	3.26833	6	1
LRCH1	leucine-rich repeats and calponin homology (CH) domain containing 1	13q14.11	0.886	0.00208	0.0196	0.3835	0.003575	0.03285				2
FCER2	Fc fragment of IgE, low affinity II, receptor for (CD23)	19p13.3				-0.526666667	0.002656667	0.027633333				1
FCF1	FCF1 small subunit (SSU) processome component homolog (S. cerevisiae)	14q24.3				-0.33	0.01349	0.0678				1
FCGBP	Fc fragment of IgG binding protein	19q13.1				-0.4135	0.0042775	0.031125				1
FCGR1A	Fc fragment of IgG, high affinity Ia, receptor (CD64)	1q21.2-q21.3							-0.882	2.82571	7	1
DENND1A	DENN/MADD domain containing 1A	9q33.3	0.885	8.00E-05	0.0019	0.316	0.00206	0.0249				2
PROCR	protein C receptor, endothelial	20q11.2	0.885	1.00E-05	4.00E-04							1
RSAD2	radical S-adenosyl methionine domain containing 2	2p25.2	0.885	0.04847	0.164				-0.807	2.77143	7	2
FCGR3B	Fc fragment of IgG, low affinity IIIb, receptor (CD16b)	1q23							-0.784	2.508	5	1
ERI1	exoribonuclease 1	8p23.1	0.884	0.00694	0.0456							1
ZNF587	zinc finger protein 587	19q13.43	0.8835	0.00201	0.01875							1
FCHO2	FCH domain only 2	5q13.2	-1.403	0	2.00E-04	-0.315	0.00046	0.0125				2
FCHSD1	FCH and double SH3 domains 1	5q31.3				-0.33	0.00577	0.0423				1
FCN3	ficolin (collagen/fibrinogen domain containing) 3 (Hakata antigen)	1p36.11				-0.48	0.00116	0.0188				1
FCRL1	Fc receptor-like 1	1q21-q22							-0.94	2.75	5	1
FCRL2	Fc receptor-like 2	1q21							-0.68	2.70841	6.333333333	1
FCRL3	Fc receptor-like 3	1q21-q22				0.368	0.00312	0.0306	-0.734	3.05875	8	2
FCRL5	Fc receptor-like 5	1q21				0.302	0.00326	0.0313	-0.881	2.80667	6	2
FDX1L	In multiple Geneids					-0.557	0.00057	0.0136				1
FDXR	ferredoxin reductase	17q24-q25	-0.784	0	1.00E-04							1
FECH	ferrochelatase	18q21.3	-0.525	0.01181	0.0655	-0.3878	0.00815	0.0363				2

FEM1A	fem-1 homolog a (C. elegans)	19p13.3	-0.487	0.00584	0.0406	-0.308	0.00122	0.0194				2
FEM1B	fem-1 homolog b (C. elegans)	15q22	-0.492	0.0022	0.0204							1
BMI1	BMI1 polycomb ring finger oncogene	10p11.23	0.883	1.00E-05	5.00E-04							1
FER	fer (fps/fes related) tyrosine kinase	5q21	-0.708	0.00124	0.0136	-0.385	0.00131	0.02				2
C1QC	complement component 1, q subcomponent, C chain	1p36.11	0.882	0.0126	0.0684							1
FER1L5	In multiple Geneids					-0.302	0.00236	0.0266				1
FER1L6	fer-1-like 6 (C. elegans)	8q24.1				0.358131579	0.009330526	0.051634211				1
FERD3L	Fer3-like (Drosophila)	7p21.1							0.744	2.806	5	1
CLDN6	claudin 6	16p13.3	0.882	0.04975	0.1666							1
KRTCAP3	keratinocyte associated protein 3	2p23.3	0.882	2.00E-05	5.00E-04							1
ENTPD6	ectonucleoside triphosphate diphosphohydrolase 6 (putative)	20p11.21	0.881	1.00E-05	5.00E-04							1
FETUB	In multiple Geneids		-1.884	0.00529	0.0379							1
FEV	In multiple Geneids					-0.395	0.00164	0.0222				1
GPRIN3	GPRIN family member 3	4q22.1	0.881	0.01533	0.0777							1
PRC1	protein regulator of cytokinesis 1	15q26.1	0.881	0.016	0.08							1
DDHD1	DDHD domain containing 1	14q21	0.88	0.00036	0.0056							1
FFAR3	free fatty acid receptor 3	19q13.1				-0.382	0.00068	0.0148				1
FGA	fibrinogen alpha chain	4q28							-0.946	2.948	5	1
FGB	fibrinogen beta chain	4q28							-0.885	3.46889	9	1
FGD1	FYVE, RhoGEF and PH domain containing 1	Xp11.21	-0.647	0.00843	0.052	-0.382	0.001225	0.01635	0.686	2.61667	6	3
FGD4	FYVE, RhoGEF and PH domain containing 4	12p11.21							-1.016	3.0225	8	1
GDPGP1	GDP-D-glucose phosphorylase 1	15q26.1	0.88	3.00E-05	9.00E-04							1
KIAA1551	Data not found		0.88	0.00117	0.0131							1
FGF1	fibroblast growth factor 1 (acidic)	5q31				-0.402416667	0.000735833	0.012375	0.773	3.04333	6	2
FGF10	fibroblast growth factor 10	5p13-p12				0.435294118	0.006668235	0.039970588				1
FGF11	fibroblast growth factor 11	17p13.1	-1.178	0.00022	0.0039							1
FGF12	fibroblast growth factor 12	3q28				0.354731707	0.003144634	0.025290244				1
FGF13	fibroblast growth factor 13	Xq26.3				0.348277778	0.00316	0.028344444	-0.886	3.33667	9	2
FGF14	fibroblast growth factor 14	13q34				0.3424	0.0095655	0.047935				1
FGF17	fibroblast growth factor 17	8p21				-0.4265	0.007735	0.04755				1
FGF18	fibroblast growth factor 18	5q34				-0.3392	0.001432	0.02005				1
FGF2	fibroblast growth factor 2 (basic)	4q26				-0.348	0.00399	0.0348				1
LGALS2	lectin, galactoside-binding, soluble, 2	22q13.1	0.879	0.01814	0.0869							1
FGF21	fibroblast growth factor 21	19q13.1-qter	-0.207	0.04033	0.1463							1
FGF3	fibroblast growth factor 3	11q13							1.388	2.41167	6	1
FGF7	fibroblast growth factor 7	15q21.2							-0.805	3.04778	9	1
RAB3B	RAB3B, member RAS oncogene family	1p32-p31	0.879	0.00183	0.0179							1
FGFBP1	fibroblast growth factor binding protein 1	4p15.32							-0.9	2.984	5	1
FGFR1	fibroblast growth factor receptor 1	8p12				-0.346333333	0.001326667	0.0194				1
SSB	Sjogren syndrome antigen B (autoantigen La)	2q31.1	0.879	0	1.00E-04							1
PIGT	In multiple Geneids		0.878	1.00E-05	3.00E-04							1
FGFR2	fibroblast growth factor receptor 2	10q26				-0.338	0.00384	0.033633333				1
FGFR3	fibroblast growth factor receptor 3	4p16.3	-1.742	0.00015	0.003	-0.336	0.00479	0.0383				2
POC1B	POC1 centriolar protein homolog B (Chlamydomonas)	12q21.33	0.878	0.0034	0.028							1
HIST1H2BG	histone cluster 1, H2bg	6p21.3	0.877	0.00137	0.0146				-0.712	2.86	5	2
FGG	fibrinogen gamma chain	4q28							-0.792	2.71	8	1
FGL1	fibrinogen-like 1	8p22							-0.981	3.94429	7	1
FGL2	fibrinogen-like 2	7q11.23							-0.834	2.78571	7	1
FGR	Gardner-Rasheed feline sarcoma viral (v-fgr) oncogene homolog	1p36.2-p36.1				-0.44	0.00371	0.0335				1
FH	fumarate hydratase	1q42.1				0.358	5.00E-04	0.0129	-0.881	2.78	6	2
FHAD1	forkhead-associated (FHA) phosphopeptide binding domain 1	1p36.21				-0.366153846	0.004499231	0.031184615				1
FHDC1	FH2 domain containing 1	4q31.3	-0.83	3.00E-04	0.0049							1
FHIT	fragile histidine triad	3p14.2							-0.89	2.83167	6	1
FHL1	four and a half LIM domains 1	Xq26				0.569	5.00E-05	0.005				1
C1R	complement component 1, r subcomponent	12p13	0.876	0.03811	0.1412							1
FHL3	four and a half LIM domains 3	1p34				-0.4165	0.002575	0.02595				1
FHL5	four and a half LIM domains 5	6q16.1-q16.3							-1.008	2.85429	7	1
TTLL4	tubulin tyrosine ligase-like family, member 4	2p24.3-p24.1	0.876	1.00E-05	5.00E-04							1
FHOD3	formin homology 2 domain containing 3	18q12				-0.309	0.00964	0.055633333				1
FIBCD1	fibrinogen C domain containing 1	9q34.12				-0.335	0.00763	0.0493				1
ALDH18A1	aldehyde dehydrogenase 18 family, member A1	10q24.3	0.875	0.00012	0.0026							1
FIG4	FIG4 homolog, SAC1 lipid phosphatase domain containing (S. cerevisiae)	6q21	-0.648	0.00065	0.0087							1
FIGF	c-fos induced growth factor (vascular endothelial growth factor D)	Xp22.31							-0.898	3.14667	6	1
FIGLA	folliculogenesis specific basic helix-loop-helix	2p13.3	-0.323	0.00169	0.0169							1
FILIP1	filamin A interacting protein 1	6q14.1							-0.902	3.1475	8	1
APP	amyloid beta (A4) precursor protein	21q21.3	0.875	0.00027	0.0045							1
FIS1	fission 1 (mitochondrial outer membrane) homolog (S. cerevisiae)	7q22.1				-0.38	0.01932	0.0835				1
FITM1	fat storage-inducing transmembrane protein 1	14q12	-0.396	0.00128	0.014							1
PHYH	phytanoyl-CoA 2-hydroxylase	10p13	0.875	0.01146	0.0641							1

FJX1	four jointed box 1 (Drosophila)	11p13	-0.272	0.019	0.0897							1
PLEK2	pleckstrin 2	14q23.3	0.875	0.01779	0.0858							1
BTN3A2	butyrophilin, subfamily 3, member A2	6p22.1	0.874	0.01301	0.0698				-0.874	3.53286	7	2
ALS2	In multiple Geneids		0.873	0	2.00E-04							1
PLXDC1	plexin domain containing 1	17q21.1	0.873	0.00392	0.0308							1
FKBP2	FK506 binding protein 2, 13kDa	11q13.1-q13.3							0.538666667	2.512776667	5.333333333	1
FKBP3	FK506 binding protein 3, 25kDa	14q21.2							-0.896	2.65625	8	1
FKBP5	FK506 binding protein 5	6p21.31				0.951	0.00048	0.0127				1
FKBP6	FK506 binding protein 6, 36kDa	7q11.23	-0.3895	0.003185	0.0238	-0.339666667	0.02881	0.1044	1.033	3.085	6	3
HAS2	hyaluronan synthase 2	8q24.12	0.872	0.00775	0.0491	0.348	0.0046	0.0375				2
FKBP8	FK506 binding protein 8, 38kDa	19p12	-0.687	0.00019	0.0035	-0.43275	0.0029775	0.026425				2
ALPP	alkaline phosphatase, placental	2q37	0.871	0.02163	0.0977							1
ACP1	acid phosphatase 1, soluble	2p25	0.87	9.00E-05	0.002	0.371	0.00026	0.0097				2
FKBPL	FK506 binding protein like	6p21.3	-0.223	0.0427	0.1518							1
FKHL18	Data not found								0.878	2.888	5	1
FKSG83	FKSG83	6p22.1							-1.071	3.7	7	1
GBP3	guanylate binding protein 3	1p22.2	0.87	0.03004	0.1208				-0.877	3.29429	7	2
FLAD1	FAD1 flavin adenine dinucleotide synthetase homolog (S. cerevisiae)	1q21.3							0.586	2.81	5	1
MTHFS	5,10-methenyltetrahydrofolate synthetase (5-formyltetrahydrofolate cyclo-lig	15q25.1	0.87	8.00E-05	0.0019							1
FLG	filaggrin	1q21.3				0.3575	0.002085	0.02445	-0.95	3.92375	8	2
FLI1	Friend leukemia virus integration 1	11q24.1-q24.3				0.311	0.00424	0.0359				1
FLII	flightless I homolog (Drosophila)	17p11.2	-0.442	0.02047	0.0943							1
FLJ10154	Data not found								-0.949	3.15571	7	1
FLJ10213	Data not found								-0.713	2.89429	7	1
FLJ10324	Data not found								0.976	2.836	5	1
FLJ10404	Data not found					-0.4094	0.004622	0.0288				1
FLJ11292	uncharacterized protein FLJ11292	5q14.3	-0.215	0.04491	0.1566							1
FLJ12547	hypothetical protein FLJ12547	16q24.3							0.52	2.52	8	1
FLJ12993	Data not found					-0.365375	0.00189125	0.0200375				1
FLJ14803	Data not found								-0.628	2.70667	6	1
FLJ14834	Data not found								0.82	3.13857	7	1
FLJ16126	uncharacterized LOC645010	1p36.22	-0.341	0.01404	0.0732							1
FLJ16237	Data not found								-0.999	3.11333	6	1
FLJ16734	In multiple Geneids								-0.633	2.432	5	1
FLJ20097	Data not found								-0.919	2.86	5	1
FLJ20160	Data not found								-0.842	2.916	5	1
FLJ20184	Data not found								-0.71	2.97857	7	1
FLJ20280	Data not found								-0.676	2.896	5	1
FLJ20464	uncharacterized protein FLJ20464	22q12.2	-0.239	0.03838	0.1419							1
FLJ20674	Data not found					-0.344666667	0.006193333	0.032333333				1
FLJ20859	Data not found								-0.609	2.63	6	1
FLJ21908	Data not found								-0.851	2.67143	7	1
FLJ21963	Data not found								0.684	2.62	5	1
FLJ21986	Data not found								-0.853	2.816	5	1
FLJ22167	Data not found					-0.324	0.00088	0.0166				1
FLJ22536	Data not found					0.65	1.00E-05	0.0032				1
FLJ22655	Data not found								-0.908	2.975	8	1
FLJ23356	Data not found								-0.759	2.61	5	1
FLJ23584	Data not found					-0.44	0.011276667	0.046266667				1
FLJ23657	Data not found								-0.785	2.79167	6	1
FLJ25076	Data not found					0.369571429	0.004764286	0.033214286				1
FLJ25102	Data not found								0.748	3.01833	6	1
FLJ25222	FLJ25222 protein	15q26.3							0.7965	3.67847	8.5	1
FLJ25415	Data not found								-0.693	2.636	5	1
FLJ25422	Data not found								-0.839	2.862	5	1
FLJ25758	MAP/microtubule affinity-regulating kinase 1 pseudogene	19p13.3	-0.403	0.00156	0.016							1
FLJ25770	Data not found								-0.882	3.3675	8	1
FLJ25791	Data not found								-1.012	3.065	8	1
FLJ30058	Data not found					0.357	0.00051	0.013				1
FLJ30313	Data not found								0.664	2.91333	6	1
FLJ30655	Data not found								-1.015	2.836	5	1
FLJ32063	uncharacterized LOC150538	2q33.1							0.8025	3.358125	8	1
FLJ32214	Data not found								0.825	3.17571	7	1
FLJ32310	Data not found								-0.891	3.00286	7	1
FLJ32447	Data not found								0.429	2.57	6	1
FLJ32679	golgin-like hypothetical protein LOC440321	15p13							1.0415	2.546	5	1
FLJ32745	Data not found								-0.888	2.758	5	1
FLJ32810	Data not found					0.351666667	0.005366667	0.032				1
FLJ33360	FLJ33360 protein	5p15.31				0.357	1.00E-04	0.0065				1

FLJ33590	Data not found								0.776	2.47	5	1
FLJ34503	uncharacterized FLJ34503	6q21							-0.848	2.792	5	1
FLJ34651	Data not found								0.618	2.56833	6	1
FLJ34870	FLJ34870 protein	2q21.2							-0.838	2.93333	6	1
FLJ35024	uncharacterized LOC401491	9p24.2				-0.36725	0.0011775	0.0176				1
FLJ35390	uncharacterized LOC255031	7p13				-0.6595	6.50E-05	0.0041				1
FLJ35630	Data not found								-0.852	2.56167	6	1
FLJ35740	Data not found								-1.014	3.22143	7	1
FLJ35779	Data not found								-0.843	3.02611	9	1
FLJ35816	FLJ35816 protein	4p16.3	-0.371	0.00721	0.0468							1
FLJ35848	Data not found								-0.74	3.05	5	1
FLJ35880	Data not found								-0.937	3.89167	6	1
FLJ36004	Data not found								-0.748	3.11429	7	1
FLJ36492	Data not found								0.813	2.544	5	1
FLJ36777	uncharacterized LOC730971					-0.357	3.00E-04	0.0104				1
FLJ36951	Data not found								1.055	3.072	5	1
FLJ37035	uncharacterized LOC399821	10q26.13	-0.577	4.00E-05	0.0011							1
FLJ37060	Data not found								-0.898	3.285	8	1
FLJ37078	Data not found					-0.516	0.00696	0.0469	1.044	3.22	5	2
FLJ37307	Data not found					0.331	0.00133	0.0201				1
FLJ37543	Data not found								-1.012	2.92143	7	1
FLJ38379	uncharacterized FLJ38379	2q37.3	-0.378	0.04577	0.1586				0.831	3.31875	8	2
FLJ38668	uncharacterized LOC644903	2q12.3	-0.231	0.01963	0.0915							1
FLJ38991	Data not found								-0.681	2.53125	8	1
FLJ39237	Data not found								0.683	3.236	5	1
FLJ39396	Data not found								0.631	2.662	5	1
FLJ39502	Data not found								-0.876	3.07167	6	1
FLJ39531	Data not found								-0.939	2.81286	7	1
FLJ39575	Data not found								-0.986	3.844	5	1
FLJ39582	hypothetical protein FLJ39582	22q11.2				-0.3225	0.00055	0.0134				1
ADAR	adenosine deaminase, RNA-specific	1q21.3	0.869	1.00E-05	3.00E-04							1
FLJ40125	Data not found								0.535	2.229	5	1
FLJ40235	Data not found								-0.928	2.776	5	1
FLJ40243	Data not found								-0.921	3.042	5	1
FLJ40296	Data not found								1.032	3.37	5	1
FLJ40536	FLJ40536 protein	10q26.13							0.867	3.84143	7	1
FLJ40852	uncharacterized LOC285962	7q34							-0.629	2.6	6	1
FLJ40919	Data not found								-1.002	3.425	8	1
FLJ41423	uncharacterized LOC399886	11p11.2	-0.231	0.04498	0.1568							1
FLJ41484	Data not found								0.968	3.99063	8	1
FLJ41603	Data not found					-0.3854	0.000304	0.00996				1
FLJ41941	uncharacterized LOC100192420	22q11.21				-0.378	2.00E-05	0.0036				1
FLJ42220	FLJ42220 protein	14q13.3	-0.766	1.00E-05	5.00E-04							1
FLJ42280	putative uncharacterized protein FLJ42280	7q21.3							-0.75	2.5325	8	1
SNORD5	small nucleolar RNA, C/D box 5	11q21	0.869	0.00275	0.024							1
FLJ42627	uncharacterized LOC645644	16p13.3				-0.457	0.003	0.0284				1
FLJ43080	Data not found								-0.878	3.07375	8	1
FLJ43390	uncharacterized LOC646113	14q23.2							-0.912	3.19625	8	1
FLJ43879	FLJ43879 protein	2q37.3	-0.205	0.0433	0.1531				0.729	3.302	5	2
FLJ43950	Data not found					-0.381	0.001946667	0.023833333				1
FLJ44006	uncharacterized FLJ44006	2q13	-0.374	0.00266	0.0234							1
FLJ44048	Data not found								-0.974	3.25375	8	1
FLJ44054	Data not found								0.698	2.64	8	1
FLJ44082	Data not found					-0.463	0.01296	0.0663				1
FLJ44451	hypothetical protein FLJ44451	Xq28							0.8	2.76	5	1
FLJ44674	FLJ44674 protein	16q12.1	-0.247	0.01028	0.0597							1
FLJ44817	Data not found					-0.334333333	0.003183333	0.028966667				1
FLJ44838	uncharacterized LOC644767		-0.426	0.00526	0.0378							1
FLJ44861	Data not found								0.712	2.81833	6	1
ZDHHC9	zinc finger, DHHC-type containing 9	Xq26.1	0.869	0.00068	0.0089							1
FLJ45455	Data not found					-0.372133333	0.001398667	0.017773333				1
FLJ45684	FLJ45684 locus	19p13.3							0.652	2.616	5	1
FLJ45743	uncharacterized LOC642484	18q21.2							-0.696	2.87857	7	1
FLJ46154	Data not found								-0.931	3.18333	9	1
FLJ46210	Data not found								0.816	3.03833	6	1
FLJ46300	FLJ46300 protein	10q26.3							-0.677	2.49571	7	1
FLJ46481	Data not found								-0.498	2.668	5	1
FLJ46688	Data not found								-1.082	4.036	5	1
FLJ90579	Data not found								-0.814	2.67	6	1

FLJ90757	uncharacterized LOC440465	17q25.3				-0.308	0.00647	0.0451				1
MTBP	Mdm2, transformed 3T3 cell double minute 2, p53 binding protein (mouse) bi	8q24.12	0.868	0.00035	0.0055	0.367428571	0.004301429	0.0358				2
FLNC	filamin C, gamma	7q32-q35				-0.407	0.00037	0.0114				1
HEXA	hexosaminidase A (alpha polypeptide)	15q24.1	0.867	0.02192	0.0986							1
FLRT1	fibronectin leucine rich transmembrane protein 1	11q12-q13							0.975	2.855	6	1
FLRT2	fibronectin leucine rich transmembrane protein 2	14q24-q32							1.158	3.87375	8	1
KIF3B	kinesin family member 3B	20q11.21	0.867	0	1.00E-04	0.322	0.00051	0.013				2
FLT3	fms-related tyrosine kinase 3	13q12	-0.304	0.02149	0.0973							1
FLT3LG	fms-related tyrosine kinase 3 ligand	19q13.3				-0.321	0.00088	0.0166				1
FLT4	fms-related tyrosine kinase 4	5q35.3				-0.474	0.00212	0.024275				1
MS4A8	Data not found		0.867	0.02527	0.1079							1
FLYWCH1	FLYWCH-type zinc finger 1	16p13.3				-0.476	0.010935	0.05495				1
FLYWCH2	FLYWCH family member 2	16p13.3				-0.5335	0.00196	0.0229				1
WISP3	WNT1 inducible signaling pathway protein 3	6q21	0.867	0.02894	0.1178				-0.807	2.85167	6	2
FMNL1	formin-like 1	17q21				-0.313	0.02543	0.0983				1
NR2C1	nuclear receptor subfamily 2, group C, member 1	12q22	0.866	5.00E-05	0.0013							1
SNORD42A	small nucleolar RNA, C/D box 42A	17q11	0.866	0.00014	0.0028							1
FMO1	flavin containing monooxygenase 1	1q24.3	-0.938	0.00675	0.0448							1
FMO2	flavin containing monooxygenase 2 (non-functional)	1q24.3				0.335	0.00064	0.0143				1
FMO4	flavin containing monooxygenase 4	1q24.3							-0.543	2.548	5	1
NCMAP	Data not found		0.865	0.00399	0.0312							1
FMO6P	flavin containing monooxygenase 6 pseudogene	1q24.3				0.3885	6.00E-04	0.01335				1
FMO9P	In multiple Geneids		-0.425	0.00177	0.0176							1
FMR1	fragile X mental retardation 1	Xq27.3				0.307	0.00211	0.0228				1
FMR1NB	fragile X mental retardation 1 neighbor	Xq28	-0.236	0.0317	0.125							1
MAST2	microtubule associated serine/threonine kinase 2	1p34.1	0.864	0.00035	0.0056							1
FRMD5	FERM domain containing 5	15q15.3	0.863	0.00017	0.0033							1
FMNL3	formin-like 3	12q13.12	0.862	0.00015	0.003							1
FNBP1	formin binding protein 1	9q34				-0.350875	0.01247875	0.0583875				1
GUSB	glucuronidase, beta	7q21.11	0.862	0	2.00E-04							1
HABP2	hyaluronan binding protein 2	10q25.3	0.862	0.02704	0.1127							1
LRRC19	leucine rich repeat containing 19	9p21.2	0.862	0.03981	0.1451				-1.05	2.79444	9	2
RGPD4	RANBP2-like and GRIP domain containing 4	2q12.3	0.862	0.00936	0.0559							1
FNDC4	fibronectin type III domain containing 4	2p23.3							-0.829	2.93	5	1
FNDC8	fibronectin type III domain containing 8	17q12	-0.233	0.02189	0.0986							1
FNDC9	fibronectin type III domain containing 9	5q33.3	-0.177	0.0196	0.0914							1
SYT13	synaptotagmin XIII	11p12-p11	0.862	0.04887	0.1647							1
FAM227A	Data not found		0.861	7.00E-05	0.0017							1
FNTA	farnesyltransferase, CAAX box, alpha	8p11	-0.34	0.04978	0.1666							1
FNTB	farnesyltransferase, CAAX box, beta	14q23.3	-0.779	0.00023	0.0041	-0.3165	0.00428	0.0325				2
FOCAD	focadhesin	9p21	-0.287	0.02625	0.1107							1
HTRA3	HtrA serine peptidase 3	4p16.1	0.861	0.00078	0.0098							1
FOS	FBJ murine osteosarcoma viral oncogene homolog	14q24.3				-0.498	0.00036	0.0112				1
FOSB	FBJ murine osteosarcoma viral oncogene homolog B	19q13.32				-0.39	0.00073	0.0153				1
FOSL2	FOS-like antigen 2	2p23.3	-0.548	0.00753	0.0482							1
KCNAB2	potassium voltage-gated channel, shaker-related subfamily, beta member 2	1p36.3	0.861	0.00098	0.0115							1
BEND7	BEN domain containing 7	10p13	0.86	0.04142	0.1489							1
FOXC1	forkhead box C1	6p25							0.739	3.35188	8	1
FOXD3	forkhead box D3	1p31.3	-0.201	0.02172	0.098							1
FOXD4	forkhead box D4	9p24.3							0.697	2.97	7	1
FOXD4L1	forkhead box D4-like 1	2q13							0.606	2.96714	7	1
FOXD4L3	In multiple Geneids								0.682	3.07625	8	1
FOXD4L4	In multiple Geneids								0.811	2.93857	7	1
FOXE1	forkhead box E1 (thyroid transcription factor 2)	9q22	-1.437	2.00E-05	6.00E-04				1.007	2.49857	7	2
FOXE3	forkhead box E3	1p32				-0.348	0.00732	0.0482				1
FOXI2	forkhead box I2	10q26.2	-0.308	0.00291	0.025							1
CHORDC1	cysteine and histidine-rich domain (CHORD) containing 1	11q14.3	0.858	0.00027	0.0046							1
SOX9	SRY (sex determining region Y)-box 9	17q23	0.856	1.00E-05	3.00E-04							1
FOXK1	forkhead box K1	7p22.1				-0.302	0.01364	0.0682				1
FOXK2	forkhead box K2	17q25				-0.327	0.01216	0.0564				1
FOXL1	forkhead box L1	16q24				-0.339	0.00068	0.0148				1
CPD	carboxypeptidase D	17q11.2	0.855	0.00027	0.0046							1
FOXN1	forkhead box N1	17q11-q12	-2.153	0	0							1
EFEMP2	EGF containing fibulin-like extracellular matrix protein 2	11q13.1	0.855	0.00123	0.0136							1
FOXN3	forkhead box N3	14q31.3	-0.497	0.00264	0.0233	-0.33545	0.0025425	0.022915				2
FOXN4	forkhead box N4	12q24.11				-0.3295	0.000385	0.01045				1
FOXO3A	Data not found								0.761	2.852	5	1
FOXO3B	forkhead box O3B pseudogene	17p11				-0.306	0.00282	0.0291				1
FOXP1	forkhead box P1	3p14.1				0.707	2.00E-05	0.004				1

FOXP2	forkhead box P2	7q31				0.323	0.00014	0.00685	-0.808	3.03333	6	2
KIF4A	kinesin family member 4A	Xq13.1	0.854	0.01959	0.0914							1
SLC29A1	solute carrier family 29 (nucleoside transporters), member 1	6p21.1	0.853	0.00153	0.0158							1
FOXR2	forkhead box R2	Xp11.21							-0.848	3.47	7	1
FOXRED2	FAD-dependent oxidoreductase domain containing 2	22q12.3				-0.367	0.00237	0.02025				1
TMEM106C	transmembrane protein 106C	12q13.1	0.853	0.00637	0.043							1
ZNF281	zinc finger protein 281	1q32.1	0.853	0	3.00E-04							1
FPRL2	Data not found								-0.714	2.95	5	1
FRAS1	In multiple Geneids					-0.345	0.00618	0.0439				1
FREM1	FRAS1 related extracellular matrix 1	9p22.3							-0.902	2.95778	9	1
FREM2	FRAS1 related extracellular matrix protein 2	13q13.3				0.334428571	0.007741429	0.043185714				1
FREM3	FRAS1 related extracellular matrix 3	4q31.21	-0.263	0.01771	0.0855							1
FREQ	Data not found					-0.388	0.006133333	0.039633333				1
FRG1	FSHD region gene 1	4q35							1.009	2.84	5	1
FRG2	FSHD region gene 2	4q35	-0.286	0.0243	0.105							1
FRK	fyn-related kinase	6q21-q22.3							-0.882	3.09833	6	1
FRMD4A	FERM domain containing 4A	10p13							-0.896	2.74429	7	1
FRMD4B	FERM domain containing 4B	3p14.1	-1.452	2.00E-05	6.00E-04	-0.327583333	0.0018525	0.021658333				2
CCDC50	coiled-coil domain containing 50	3q28	0.852	0.00042	0.0063	0.3674	0.001892	0.02078	-1.293	3.288	5	3
FRMD6	FERM domain containing 6	14q22.1	-2.572	1.00E-05	3.00E-04							1
FRMD7	FERM domain containing 7	Xq26.2				0.341	0.00088	0.0167				1
FRMPD1	FERM and PDZ domain containing 1	9p13.2				-0.370944444	0.001999444	0.019605556				1
FRMPD4	FERM and PDZ domain containing 4	Xp22.2				0.377833333	0.001178333	0.016166667				1
FRRS1	ferric-chelate reductase 1	1p21.2	-0.9	0.0069	0.0454	-0.368	0.00058	0.0138				2
FRS3	fibroblast growth factor receptor substrate 3	6p21.1							0.568	2.506	5	1
COG2	component of oligomeric golgi complex 2	1q42.2	0.852	0	0	0.3585	0.00064	0.0123				2
FRYL	In multiple Geneids					-0.389333333	0.002403333	0.023933333				1
AKR1C3	aldo-keto reductase family 1, member C3 (3-alpha hydroxysteroid dehydroger	10p15-p14	0.851	0.01714	0.0837				-0.902	3.35	7	2
FSCN1	fascin homolog 1, actin-bundling protein (Strongylocentrotus purpuratus)	7p22				-0.329	0.0303	0.1092				1
FSCN3	fascin homolog 3, actin-bundling protein, testicular (Strongylocentrotus purpu	7q31.3	-0.324	0.00534	0.0382							1
FSD1	fibronectin type III and SPRY domain containing 1	19p13.3				-0.494	0.001405	0.01965				1
CCDC14	coiled-coil domain containing 14	3q21.1	0.851	0.00033	0.0052	0.339	0.001615	0.022	-1.026	3.31	8	3
FSHB	follicle stimulating hormone, beta polypeptide	11p13	-0.365	0.00088	0.0107							1
FSHR	follicle stimulating hormone receptor	2p21-p16				0.365625	0.0037775	0.028225	-0.6	2.72	7	2
FSIP2	In multiple Geneids								-1.053	3.372	5	1
UBR1	In multiple Geneids		0.851	1.00E-04	0.0022				-0.759	2.68333	6	2
KIF18A	kinesin family member 18A	11p14.1	0.85	0.00693	0.0456							1
FSTL4	follistatin-like 4	5q31.1	-0.531	0.03731	0.1392	-0.34375	0.00280625	0.0246				2
FSTL5	follistatin-like 5	4q32.3				-0.351	0.000985	0.0153				1
FTCD	formiminotransferase cyclodeaminase	21q22.3				-0.4915	0	0.00185				1
CD93	CD93 molecule	20p11.21	0.849	0.00054	0.0076							1
FTMT	ferritin mitochondrial	5q21.3	-0.31	0.01486	0.0761							1
FTO	In multiple Geneids					-0.338	0.00743	0.0486				1
FTSJ1	FtsJ homolog 1 (E. coli)	Xp11.23							0.842	3.06	7	1
CEP170	centrosomal protein 170kDa	1q44	0.8485	0.003755	0.025							1
FUBP3	far upstream element (FUSE) binding protein 3	9q34.11				-0.387	0.00399	0.0348				1
GPR4	G protein-coupled receptor 4	19q13.3	0.848	2.00E-05	6.00E-04							1
NUDCD1	NudC domain containing 1	8q23	0.848	5.00E-05	0.0013	0.341666667	0.000895556	0.015177778				2
FUK	fucokinase	16q22.1				-0.358	0.0029	0.02915				1
FUNDC2	FUN14 domain containing 2	Xq28				0.302	0.00344	0.0322				1
DUSP10	dual specificity phosphatase 10	1q41	0.847	0.00606	0.0416	0.368	0.002086364	0.020763636				2
DENND3	DENN/MADD domain containing 3	8q24.3	0.845	0.00467	0.0348							1
HSPA1B	heat shock 70kDa protein 1B	6p21.3	0.845	0.0259	0.1098							1
FUT1	fucosyltransferase 1 (galactoside 2-alpha-L-fucosyltransferase, H blood group)	19q13.3				-0.352	0.02498	0.0973				1
FUT10	In multiple Geneids					-0.373111111	0.005106667	0.035988889				1
FUT11	fucosyltransferase 11 (alpha (1,3) fucosyltransferase)	10q22.2	-0.55	0.0223	0.0995							1
FUT2	fucosyltransferase 2 (secretor status included)	19q13.3				-0.402	0.00292	0.0296	0.663	2.476	5	2
FUT3	fucosyltransferase 3 (galactoside 3(4)-L-fucosyltransferase, Lewis blood group	19p13.3	-1.398	0.00609	0.0417	-0.509	5.00E-05	0.0052				2
L3MBTL3	l(3)mbt-like 3 (Drosophila)	6q23	0.845	0.00748	0.0481							1
FUT5	fucosyltransferase 5 (alpha (1,3) fucosyltransferase)	19p13.3	-0.433	0.00223	0.0206	-0.515	0.004565	0.03335				2
FUT6	fucosyltransferase 6 (alpha (1,3) fucosyltransferase)	19p13.3	-1.565	0.00013	0.0028	-0.511666667	0.000366667	0.011266667				2
PCDHB10	protocadherin beta 10	5q31	0.845	0.00489	0.036				-0.795	3.30833	6	2
FXC1	fracture callus 1 homolog (rat)	11p15.5-p15.3							-0.669	2.34833	6	1
FXN	frataxin	9q21.11				-0.317	0.02147	0.0889				1
GPRC5C	G protein-coupled receptor, family C, group 5, member C	17q25	0.844	0.02194	0.0987							1
FXR2	fragile X mental retardation, autosomal homolog 2	17p13.1				-0.4675	0.002085	0.02205				1
FXYD1	FXYD domain containing ion transport regulator 1	19q13.1							0.842	2.4625	6	1
ICAM2	intercellular adhesion molecule 2	17q23.3	0.844	0.00111	0.0126							1
FXYD7	FXYD domain containing ion transport regulator 7	19q13.12	-0.266	0.00704	0.0461	-0.313	0.00379	0.0339	0.745	2.558	5	3

FYB	FYN binding protein	5p13.1				0.363714286	0.011445	0.053607143				1
FYCO1	FYVE and coiled-coil domain containing 1	3p21.31	-0.397	0.01427	0.074							1
INF2	inverted formin, FH2 and WH2 domain containing	14q32.33	0.844	0.00011	0.0022							1
FYTTD1	forty-two-three domain containing 1	3q29	-0.431	0.00319	0.0268							1
FZD1	frizzled family receptor 1	7q21	-0.426	0.01107	0.0626							1
FZD10	frizzled family receptor 10	12q24.33	-1.139	5.00E-05	0.0013							1
SNORD103A	small nucleolar RNA, C/D box 103A	1p35.2	0.843	6.00E-04	0.0082							1
FZD6	frizzled family receptor 6	8q22.3-q23.1				0.341	0.00054	0.0133				1
SRI	sorcin	7q21.1	0.843	0.00031	0.005	0.35	0.00096	0.0173	-0.863	2.92714	7	3
FZR1	fizzy/cell division cycle 20 related 1 (Drosophila)	19p13.3				-0.568	0.00066	0.0146				1
STARD3NL	STARD3 N-terminal like	7p14-p13	0.843	3.00E-05	0.001							1
G1P3	Data not found								-0.714	2.88833	6	1
G6PC3	glucose 6 phosphatase, catalytic, 3	17q21.31				-0.323	0.00831	0.0517				1
GAA	glucosidase, alpha; acid	17q25.2-q25.3				-0.315	0.02209	0.0904				1
GAB1	GRB2-associated binding protein 1	4q31.21	-0.7635	0.01145	0.05195							1
GAB2	GRB2-associated binding protein 2	11q14.1				-0.393	0.00378	0.0339				1
GAB4	GRB2-associated binding protein family, member 4	22q11.1				-0.366714286	0.001012857	0.015628571	0.818	3.552	5	2
GABARAP	GABA(A) receptor-associated protein	17p13.1	-0.231	0.04156	0.1492	-0.417	0.00175	0.023				2
GABARAPL1	GABA(A) receptor-associated protein like 1	12p13.2				-0.324	0.00098	0.0175				1
GABARAPL2	In multiple Geneids					-0.306	0.0318	0.1125				1
GABBR1	gamma-aminobutyric acid (GABA) B receptor, 1	6p21.31	-0.349	0.02256	0.1001	-0.308	0.04793	0.145	0.678	2.776	5	3
GABBR2	gamma-aminobutyric acid (GABA) B receptor, 2	9q22.1-q22.3				0.337	0.00326	0.0314				1
GABPB1	In multiple Geneids					-0.41	0.01241	0.0646				1
GABRA3	gamma-aminobutyric acid (GABA) A receptor, alpha 3	Xq28				0.3455	0.00356	0.029916667				1
GABRA4	gamma-aminobutyric acid (GABA) A receptor, alpha 4	4p12	-0.615	1.00E-05	4.00E-04							1
GABRA5	gamma-aminobutyric acid (GABA) A receptor, alpha 5	15q12							0.676	2.88167	6	1
GABRA6	gamma-aminobutyric acid (GABA) A receptor, alpha 6	5q34							-0.822	2.95333	6	1
GABRB1	gamma-aminobutyric acid (GABA) A receptor, beta 1	4p12							-0.932	3.71917	6	1
GABRD	gamma-aminobutyric acid (GABA) A receptor, delta	1p36.3				-0.473	0.00016	0.008				1
GABRE	gamma-aminobutyric acid (GABA) A receptor, epsilon	Xq28	-1.632	0.02282	0.1009							1
GABRG3	gamma-aminobutyric acid (GABA) A receptor, gamma 3	15q12				0.4595	0.01682	0.06755				1
GABRP	gamma-aminobutyric acid (GABA) A receptor, pi	5q35.1	-1.941	0.01267	0.0686							1
GABRQ	gamma-aminobutyric acid (GABA) A receptor, theta	Xq28				0.313	0.00448	0.0369				1
GABRR1	gamma-aminobutyric acid (GABA) A receptor, rho 1	6q15				-0.321333333	0.00131	0.019133333				1
GABRR2	gamma-aminobutyric acid (GABA) A receptor, rho 2	6q15				-0.371428571	0.000231429	0.007242857				1
GABRR3	gamma-aminobutyric acid (GABA) A receptor, rho 3	3q11.2				0.306	0.04606	0.1414				1
GAD1	glutamate decarboxylase 1 (brain, 67kDa)	2q31							1.002	3.3756	5	1
GAD2	glutamate decarboxylase 2 (pancreatic islets and brain, 65kDa)	10p11.23	-0.364	0.00155	0.016	-0.33	0.02809	0.1043				2
GADD45B	growth arrest and DNA-damage-inducible, beta	19p13.3				-0.437	0.00488	0.0387				1
GADD45GIP1	growth arrest and DNA-damage-inducible, gamma interacting protein 1	19p13.2	-0.236	0.01935	0.0908	-0.314	0.03286	0.1148				2
GADL1	glutamate decarboxylase-like 1	3p24.1-p23							-1.091	3.992	5	1
GAFA3	FGF-2 activity-associated protein 3	4p14	-0.719	0.00063	0.0085							1
GAK	cyclin G associated kinase	4p16	-0.313	0.00913	0.0549	-0.379	0.001972	0.0196				2
CCNB2	cyclin B2	15q22.2	0.842	0.0343	0.1314				-0.775	2.65167	6	2
LOC100133299	GALI1870	5p15.2	0.842	0.002	0.0191							1
OSR2	odd-skipped related 2 (Drosophila)	8q22.2	0.842	0.0045	0.034							1
GAL3ST4	galactose-3-O-sulfotransferase 4	7q22.1	-0.504	0.00874	0.0533	-0.584	0.00603	0.0434				2
PTPRM	protein tyrosine phosphatase, receptor type, M	18p11.2	0.841	0.01853	0.0882	0.367	0.0013	0.0199				2
ARRB1	arrestin, beta 1	11q13	0.84	0.00926	0.0554							1
CALM2	calmodulin 2 (phosphorylase kinase, delta)	2p21	0.839	0	0							1
GALNS	galactosamine (N-acetyl)-6-sulfate sulfatase	16q24.3				-0.4464	0.00059	0.0131				1
GALNT1	UDP-N-acetyl-alpha-D-galactosamine:polypeptide N-acetylgalactosaminyltran	18q12.1							-0.93	3.38167	6	1
SCARNA9L	small Cajal body-specific RNA 9-like (retrotransposed)	Xp22.12	0.839	0.00189	0.0183							1
GALNT12	UDP-N-acetyl-alpha-D-galactosamine:polypeptide N-acetylgalactosaminyltran	9q22.33	-0.723	0.00997	0.0583	-0.301	0.00404	0.035				2
GALNT13	UDP-N-acetyl-alpha-D-galactosamine:polypeptide N-acetylgalactosaminyltran	2q24.1				0.338076923	0.00033	0.008053846	-0.872	3.19	6	2
GALNT14	UDP-N-acetyl-alpha-D-galactosamine:polypeptide N-acetylgalactosaminyltran	2p23.1	-1.839	0	0							1
KIAA0907	KIAA0907	1q22	0.838	8.00E-05	0.0018							1
GALNT3	UDP-N-acetyl-alpha-D-galactosamine:polypeptide N-acetylgalactosaminyltran	2q24-q31							-1.124	3.93714	7	1
DACT1	dapper, antagonist of beta-catenin, homolog 1 (Xenopus laevis)	14q23.1	0.837	0.00833	0.0515							1
GALNT5	UDP-N-acetyl-alpha-D-galactosamine:polypeptide N-acetylgalactosaminyltran	2q24.1				0.33	9.00E-05	0.0065				1
GPX2	glutathione peroxidase 2 (gastrointestinal)	14q24.1	0.837	0.01349	0.0713							1
GALNT7	In multiple Geneids					-0.384	0.001965	0.0224				1
GALNT9	UDP-N-acetyl-alpha-D-galactosamine:polypeptide N-acetylgalactosaminyltran	12q24.33				-0.389	0.001055	0.01445				1
GALNTL1	UDP-N-acetyl-alpha-D-galactosamine:polypeptide N-acetylgalactosaminyltran	14q24.1				-0.3155	0.0109725	0.0474				1
GALNTL2	UDP-N-acetyl-alpha-D-galactosamine:polypeptide N-acetylgalactosaminyltran	3p25.1				-0.324	0.0017	0.0227				1
GALNTL4	UDP-N-acetyl-alpha-D-galactosamine:polypeptide N-acetylgalactosaminyltran	11p15.3				0.353	0.00746	0.0487				1
GALNTL5	UDP-N-acetyl-alpha-D-galactosamine:polypeptide N-acetylgalactosaminyltran	7q36.1				-0.302	0.01332	0.0673				1
GALNTL6	UDP-N-acetyl-alpha-D-galactosamine:polypeptide N-acetylgalactosaminyltran	4q34.1	-0.267	0.00455	0.0343	-0.4135	0.0064675	0.042175				2
GALP	galanin-like peptide	19q13.43	-0.245	0.01641	0.0812	-0.3625	0.0097	0.05415				2

GALR2	galanin receptor 2	17q25.3	-0.469	2.00E-04	0.0036	-0.38	0.00978	0.0565				2
GAN	gigaxonin	16q24.1	-0.639	0.01703	0.0833							1
MDC1	mediator of DNA-damage checkpoint 1	6p21.3	0.837	0.00192	0.0185							1
GAP43	growth associated protein 43	3q13.1-q13.2				0.321	0.02606	0.0998				1
GAPT	GRB2-binding adaptor protein, transmembrane	5q11.2	-0.683	0.00988	0.0579							1
GAPVD1	GTPase activating protein and VPS9 domains 1	9q33.3	-0.405	0.02029	0.0936							1
GAR1	GAR1 ribonucleoprotein homolog (yeast)	4q25	-0.392	0.00048	0.007	-0.3955	0.00161	0.01975				2
GAREM	Data not found		-0.654	0.02539	0.1083							1
GARNL1	Data not found					-0.41	0.000965	0.01355				1
GARNL4	Data not found					-0.449731707	0.003889512	0.025887805	0.79	3.01833	6	2
GART	phosphoribosylglycinamide formyltransferase, phosphoribosylglycinamide syr	21q22.11				-0.422	0.005056667	0.032433333				1
GAS2	growth arrest-specific 2	11p14.3							-0.826	3.28	7	1
GAS2L1	growth arrest-specific 2 like 1	22q12.2	-0.558	5.00E-05	0.0014							1
PLCL2	phospholipase C-like 2	3p24.3	0.837	0.02235	0.0996	0.901	3.00E-05	0.0041	-0.729	2.73125	8	3
GAS7	growth arrest-specific 7	17p13.1	-0.68	0.00794	0.0499	-0.399742424	0.002882273	0.022736364				2
GAS8	growth arrest-specific 8	16q24.3				-0.509	0.005055	0.03535				1
GAST	gastrin	17q21							0.698	2.75333	6	1
GATA2	GATA binding protein 2	3q21.3							0.80475	3.566665	6	1
GATA3-AS1	Data not found		-0.239	0.01722	0.0839							1
SLC39A4	In multiple Geneids		0.837	0.00041	0.0062							1
ATP2C2	ATPase, Ca++ transporting, type 2C, member 2	16q24.1	0.836	0.00102	0.0119							1
FUS	fused in sarcoma	16p11.2	0.836	0	2.00E-04							1
GATAD2A	GATA zinc finger domain containing 2A	19p13.11				-0.384807692	0.002774615	0.022084615				1
SHFM1	In multiple Geneids		0.836	8.00E-05	0.0019				-1.006	2.935	6	2
DOM3Z	dom-3 homolog Z (C. elegans)	6p21.3	0.835	0.00369	0.0296							1
GATS	GATS, stromal antigen 3 opposite strand	7q22.1				-0.419	0.01466	0.065633333				1
GATSL1	GATS protein-like 1	7q11.23				-0.387	0.02453	0.0962				1
GATSL2	In multiple Geneids					-0.448666667	0.0149	0.0518				1
GATSL3	GATS protein-like 3	22q12	-1.945	0	1.00E-04							1
GBA2	glucosidase, beta (bile acid) 2	9p13.3				-0.3775	8.00E-05	0.0047				1
GBA3	glucosidase, beta, acid 3 (cytosolic)	4p15.2							-0.904	2.9225	8	1
RASSF4	Ras association (RalGDS/AF-6) domain family member 4	10q11.21	0.835	0.00547	0.0389							1
SCARNA9	small Cajal body-specific RNA 9	11q21	0.835	0.011285	0.0529							1
GBE1	glucan (1,4-alpha-), branching enzyme 1	3p12.3	-0.591	0.00412	0.032							1
GBF1	golgi brefeldin A resistant guanine nucleotide exchange factor 1	10q24							-0.831	3.01667	6	1
GBP1	guanylate binding protein 1, interferon-inducible	1p22.2							-0.788	2.91667	6	1
TTC38	tetratricopeptide repeat domain 38	22q13	0.835	0.00021	0.0038							1
GBP6	guanylate binding protein family, member 6	1p22.2	-5.437	0	1.00E-04							1
GBP7	guanylate binding protein 7	1p22.2							-0.772	2.95333	6	1
GBX2	gastrulation brain homeobox 2	2q37.2							0.823	2.78	6	1
GC	group-specific component (vitamin D binding protein)	4q12-q13							-1.04	3.63375	8	1
HGF	hepatocyte growth factor (hepapoietin A; scatter factor)	7q21.1	0.834	0.00212	0.0199	0.401	0.014205	0.0684				2
GCAT	glycine C-acetyltransferase	22q13.1	-0.248	0.04823	0.1636	-0.352	0.00095	0.0172				2
SLFN11	schlafen family member 11	17q12	0.834	0.03156	0.1248							1
GCDH	glutaryl-CoA dehydrogenase	19p13.2				-0.372	0.01377	0.0686				1
GCET2	germinal center expressed transcript 2	3q13.2							-0.687	2.804	5	1
GCGR	glucagon receptor	17q25				-0.3495	0.022505	0.0911				1
GCHFR	GTP cyclohydrolase I feedback regulator	15q15	-0.682	8.00E-05	0.002							1
GCKR	glucokinase (hexokinase 4) regulator	2p23							-0.829	2.93	5	1
GCN1L1	GCN1 general control of amino-acid synthesis 1-like 1 (yeast)	12q24.2				-0.324666667	0.000866667	0.0152				1
APLNR	apelin receptor	11q12	0.833	0.00045	0.0067							1
GCNT2	glucosaminyl (N-acetyl) transferase 2, I-branching enzyme (I blood group)	6p24.2				-0.3435	0.00331	0.0288				1
CYP3A5	cytochrome P450, family 3, subfamily A, polypeptide 5	7q21.1	0.83	0.0137	0.0721				-0.844	2.82714	7	2
GCNT4	glucosaminyl (N-acetyl) transferase 4, core 2	5q12	-0.966	0.00625	0.0425							1
GCOM1	GRINL1A complex locus 1	15q21.3	-1.991	0	1.00E-04							1
DNM1	dynamin 1	9q34	0.83	0.00165	0.0167							1
GDAP1	ganglioside induced differentiation associated protein 1	8q21.11							-0.943	2.55143	7	1
GDDR	Data not found								-0.829	2.77333	6	1
FUCA1	fucosidase, alpha-L- 1, tissue	1p34	0.83	0.00224	0.0207							1
GDF1	growth differentiation factor 1	19p12				-0.37875	0.003005	0.028325				1
SP4	Sp4 transcription factor	7p15.3	0.83	1.00E-05	4.00E-04	0.389	0.00103	0.0179				2
CD14	CD14 molecule	5q31.1	0.829	0.01791	0.0861							1
GDF3	growth differentiation factor 3	12p13.1	-0.213	0.04178	0.1497							1
GDF5	In multiple Geneids		-0.247	0.01505	0.0768							1
GDF6	In multiple Geneids		-0.21	0.03794	0.1407							1
GDF7	In multiple Geneids		-0.291	0.01613	0.0804							1
GDF8	Data not found								-0.972	3.39556	9	1
GDI2	GDP dissociation inhibitor 2	10p15				-0.402	0.01078	0.0597				1
GDNF	glial cell derived neurotrophic factor	5p13.1-p12				0.3915	0.000535	0.0129				1

PRSS23	protease, serine, 23	11q14.1	0.829	0.00447	0.0338							1
GDPD3	glycerophosphodiester phosphodiesterase domain containing 3	16p11.2	-1.221	0.01399	0.0731							1
ZNF552	zinc finger protein 552	19q13.43	0.829	0.00915	0.055							1
CTSA	cathepsin A	20q13.1	0.828	4.00E-05	0.0011							1
HSP90AA1	heat shock protein 90kDa alpha (cytosolic), class A member 1	14q32.33	0.828	0.00047	0.0069							1
GEMIN4	gem (nuclear organelle) associated protein 4	17p13	-0.347	0.00328	0.0273	-0.39525	0.0126975	0.051525				2
GEMIN5	gem (nuclear organelle) associated protein 5	5q33.2				-0.351	0.001955	0.0205				1
RHOQ	ras homolog family member Q	2p21	0.828	0.00045	0.0067							1
GFAP	glial fibrillary acidic protein	17q21				-0.399	0.00232	0.0264				1
SMPD3	sphingomyelin phosphodiesterase 3, neutral membrane (neutral sphingomyel	16q22.1	0.828	0.04242	0.1512							1
GFI1	growth factor independent 1 transcription repressor	1p22							0.964	3.25667	6	1
EMB	embigin	5q11.1	0.827	0.00886	0.0537							1
GFM2	G elongation factor, mitochondrial 2	5q13				-0.341	0.00015	0.0077				1
GFOD1	glucose-fructose oxidoreductase domain containing 1	6pter-p22.1	-0.48	0.00506	0.0325							1
GFOD2	glucose-fructose oxidoreductase domain containing 2	16q22.1	-0.941	1.00E-05	3.00E-04	-0.38125	0.00578	0.0379375				2
GLB1	galactosidase, beta 1	3p21.33	0.827	0.0015	0.0156							1
SAMHD1	SAM domain and HD domain 1	20pter-q12	0.827	0.00176	0.0175							1
GFRA2	GDNF family receptor alpha 2	8p21.3				-0.3883	0.006207	0.03565				1
GFRA3	GDNF family receptor alpha 3	5q31.1-q31.3				-0.398666667	0.003546667	0.028133333				1
GFRA4	GDNF family receptor alpha 4	20p13-p12				-0.345	0.00142	0.0207				1
GGA1	In multiple Geneids		-0.444	0.00024	0.0042	-0.437	0.00214875	0.020375				2
GGA2	golgi-associated, gamma adaptin ear containing, ARF binding protein 2	16p12				-0.305	0.01184	0.063				1
GGA3	golgi-associated, gamma adaptin ear containing, ARF binding protein 3	17q25.1	-0.273	0.02476	0.1064	-0.3365	0.002195	0.02555				2
GGACT	Data not found		-0.174	0.04195	0.15							1
GGN	gametogenetin	19q13.2							0.953	2.85	6	1
FAM46A	family with sequence similarity 46, member A	6q14	0.826	0.00643	0.0432							1
GGT2	gamma-glutamyltransferase 2	22q11.21							0.713	2.418	5	1
PTCH1	patched 1	9q22.3	0.826	0.02847	0.1165							1
GGT5	gamma-glutamyltransferase 5	22q11.23				-0.521	2.00E-05	0.0039				1
GGT6	gamma-glutamyltransferase 6	17p13.2	-1.733	0	0				0.804	3.254	5	2
FRY	furry homolog (Drosophila)	13q13.1	0.825	0.01165	0.0649	0.378625	0.008505	0.048475				2
GGTA1P	In multiple Geneids		-2.213	0	0							1
GGTL4	Data not found								0.782	3.29937	8	1
GGTLA4	Data not found								0.838	3.60438	8	1
MVP	major vault protein	16p11.2	0.825	0.00062	0.0084							1
GH1	growth hormone 1	17q24.2	-0.291	0.01981	0.0922				0.56	2.60667	6	2
SH3RF1	SH3 domain containing ring finger 1	4q32.3	0.825	0.01121	0.0632							1
GHR	growth hormone receptor	5p13-p12				0.395984127	0.007049524	0.040468254				1
GHRL	ghrelin/obestatin prepropeptide	3p26-p25				-0.422	0.00076	0.0155				1
GHRLOS	ghrelin opposite strand/antisense RNA (non-protein coding)	3p25.3				-0.422	0.00076	0.0155				1
GHSR	growth hormone secretagogue receptor	3q26.31				0.327	0.00014	0.0074				1
GID4	Data not found		-0.891	0.00013	0.0026							1
GID8	Data not found		-0.563	0.00673	0.0446							1
GIF	gastric intrinsic factor (vitamin B synthesis)	11q13							-0.887	2.85167	6	1
CKAP5	cytoskeleton associated protein 5	11p11.2	0.824	1.00E-04	0.0022							1
GIMAP8	GTPase, IMAP family member 8	7q36.1				-0.32	0.00085	0.0163				1
GIN1	gypsy retrotransposon integrase 1	5q21.1	-0.285	0.03429	0.1314							1
GINS3	GINS complex subunit 3 (Psf3 homolog)	16q21	-0.759	0.00063	0.0085							1
DCBLD1	discoidin, CUB and LCCL domain containing 1	6q22.1	0.824	2.00E-05	6.00E-04							1
GIPC1	GIPC PDZ domain containing family, member 1	19p13.1	-0.79	4.00E-05	0.0012	-0.315	2.00E-04	0.0088				2
KCTD7	potassium channel tetramerisation domain containing 7	7q11.21	0.824	0.00028	0.0046							1
GIPC3	GIPC PDZ domain containing family, member 3	19p13.3				-0.325	0.00623	0.0441				1
GIPR	gastric inhibitory polypeptide receptor	19q13.3				-0.456	0.001083333	0.016266667				1
GIT2	G protein-coupled receptor kinase interacting ArfGAP 2	12q24.1				-0.3678	0.001998	0.02008				1
GJA10	gap junction protein, alpha 10, 62kDa	6q15-q16							-0.825	2.974	5	1
GJA3	gap junction protein, alpha 3, 46kDa	13q12.11	-0.431	0.00129	0.014				0.311	2.872	5	2
RFC3	replication factor C (activator 1) 3, 38kDa	13q13.2	0.824	0.00507	0.0368	0.34475	0.0015725	0.01985	-0.883	3.06333	6	3
SLC25A37	solute carrier family 25 (mitochondrial iron transporter), member 37	8p21.2	0.824	0.00237	0.0216							1
GJB2	gap junction protein, beta 2, 26kDa	13q11-q12	-2.488	2.00E-04	0.0037							1
GJB3	gap junction protein, beta 3, 31kDa	1p34	-0.826	0.0028	0.0243							1
GJB5	gap junction protein, beta 5, 31.1kDa	1p35.1	-2.53	0	0							1
GJB6	gap junction protein, beta 6, 30kDa	13q12	-3.604	1.00E-05	3.00E-04				0.753	3.128	5	2
MGAT5	mannosyl (alpha-1,6-)-glycoprotein beta-1,6-N-acetyl-glucosaminyltransferase	2q21.3	0.823	0.00183	0.0179	0.345666667	0.00355	0.023666667				2
SLC46A1	solute carrier family 46 (folate transporter), member 1	17q11.2	0.823	0.00815	0.0508							1
GJD4	gap junction protein, delta 4, 40.1kDa	10p11.21	-0.276	0.00592	0.041							1
PDE11A	phosphodiesterase 11A	2q31.2	0.822	0.00053	0.0074	0.385555556	0.001645556	0.016755556				2
PPM1H	protein phosphatase, Mg2+/Mn2+ dependent, 1H	12q14.1	0.822	5.00E-05	0.0012							1
SPIRE2	spire homolog 2 (Drosophila)	16q24	0.822	4.00E-05	0.0012							1
GKN1	gastrokine 1	2p13.3							-0.95	3.644	5	1

GKN2	gastrokine 2	2p13.3				0.303	0.0014	0.0206				1
CD63	CD63 molecule	12q12-q13	0.821	0.00015	0.003							1
PXN	paxillin	12q24.31	0.821	8.50E-05	0.00185							1
GLIS2	GLIS family zinc finger 2	16p13.3	0.82	0.00246	0.0222							1
GPR89B	In multiple Geneids		0.82	0.00113	0.0127	0.308	0.00521	0.0401				2
GLCCI1	glucocorticoid induced transcript 1	7p21.3				0.357333333	0.003656667	0.0259				1
GLDC	glycine dehydrogenase (decarboxylating)	9p22				-0.497	0.0011408	0.0154				1
GLDN	gliomedin	15q21.2				-0.326	0.00116	0.0189				1
GLE1	GLE1 RNA export mediator homolog (yeast)	9q34.11				-0.328	0.00565	0.0418				1
NSMF	Data not found		0.82	0	2.00E-04							1
GLI1	GLI family zinc finger 1	12q13.2-q13.3				-0.386	0.00042	0.0119				1
GLI2	GLI family zinc finger 2	2q14				0.303	0.00074	0.0154				1
GLI3	GLI family zinc finger 3	7p13				0.3468	0.001337	0.01766				1
GLIPR1	GLI pathogenesis-related 1	12q21.2							-0.768	3.005	6	1
GLIPR2	GLI pathogenesis-related 2	9p13.3				-0.420333333	0.000921667	0.01425				1
GLIS1	GLIS family zinc finger 1	1p32.3				-0.425	0.000913333	0.016333333				1
ABCD1	ATP-binding cassette, sub-family D (ALD), member 1	Xq28	0.819	1.00E-05	5.00E-04							1
RRBP1	ribosome binding protein 1 homolog 180kDa (dog)	20p12	0.819	0.00043	0.0064							1
GLO1	glyoxalase I	6p21.3-p21.1				0.3205	0.035985	0.1196				1
GLOD4	glyoxalase domain containing 4	17p13.3				-0.348	0.018906667	0.072333333				1
GLP1R	glucagon-like peptide 1 receptor	6p21				0.322	0.00502	0.0393				1
GLP2R	glucagon-like peptide 2 receptor	17p13.3				-0.400555556	0.002853333	0.021288889				1
GLRA1	glycine receptor, alpha 1	5q32				-0.344	0.00023	0.0092				1
GLRA2	glycine receptor, alpha 2	Xp22.2				0.349166667	0.0064775	0.041583333	-0.696	2.69	6	2
GLRA3	glycine receptor, alpha 3	4q34.1	-0.217	0.01361	0.0717							1
SKAP1	src kinase associated phosphoprotein 1	17q21.32	0.819	0.01337	0.0708							1
ZNF622	zinc finger protein 622	5p15.1	0.819	4.00E-05	0.0011	0.351	0.00207	0.02485				2
PRKAB2	protein kinase, AMP-activated, beta 2 non-catalytic subunit	1q21.1	0.818	0.00435	0.0332							1
GLT1D1	glycosyltransferase 1 domain containing 1	12q24.33				-0.378875	0.0004875	0.0107				1
GLT25D1	glycosyltransferase 25 domain containing 1	19p13.11				-0.446	0.02071	0.07295				1
GLT25D2	glycosyltransferase 25 domain containing 2	1q25				0.331	0.00541	0.0329				1
SLC17A4	solute carrier family 17 (sodium phosphate), member 4	6p22.2	0.818	0.0215	0.0973							1
GLT8D2	glycosyltransferase 8 domain containing 2	12q							-0.917	2.724	5	1
GLT8D4	Data not found					0.328	0.00286	0.0293				1
GLTP	glycolipid transfer protein	12q24.11	-2.765	0	0	-0.351	0.00031	0.0105				2
GLTPD1	glycolipid transfer protein domain containing 1	1p36.33	-0.926	0	0.00015	-0.408	0.00271	0.0285				2
GLTSCR1	glioma tumor suppressor candidate region gene 1	19q13.3				-0.4266	0.001696	0.02128				1
GLTSCR2	glioma tumor suppressor candidate region gene 2	19q13.3				-0.354	0.00387	0.0343				1
GLUL	glutamate-ammonia ligase	1q31	-0.774	0.004015	0.0314							1
GLULD1	Data not found								-0.961	3.868	5	1
GLYAT	glycine-N-acyltransferase	11q12.1				0.309	0.00016	0.008				1
GLYATL2	glycine-N-acyltransferase-like 2	11q12.1							-0.818	2.66714	7	1
GLYCTK	glycerate kinase	3p21.1				-0.376	0.00035	0.011				1
GM2A	GM2 ganglioside activator	5q33.1	-1.505	0	1.00E-04	-0.319	0.002003333	0.021366667				2
ALG1L	asparagine-linked glycosylation 1-like	3q21.2	0.817	0.00071	0.0092							1
GMDS	GDP-mannose 4,6-dehydratase	6p25				-0.419033333	0.02603	0.095403333				1
GMEB1	glucocorticoid modulatory element binding protein 1	1p35.3				-0.381666667	0.018573333	0.071083333				1
FAM60A	family with sequence similarity 60, member A	12p11	0.817	0.00015	0.003							1
GMFG	glia maturation factor, gamma	19q13.2				-0.357	0.01483	0.0717				1
GMIP	GEM interacting protein	19p13.11				-0.4764	0.00174	0.01514				1
GML	glycosylphosphatidylinositol anchored molecule like protein	8q24.3	-0.302	0.00771	0.049							1
RGS5	regulator of G-protein signaling 5	1q23.1	0.817	0.04789	0.1628	0.3435	0.00078	0.011975	-0.758	2.67167	6	3
GMPR2	In multiple Geneids					-0.3235	1.50E-05	0.00335				1
SDS	In multiple Geneids		0.817	0.00018	0.0034							1
SPON2	spondin 2, extracellular matrix protein	4p16.3	0.817	0.00631	0.0428							1
ATP6V1C1	ATPase, H+ transporting, lysosomal 42kDa, V1 subunit C1	8q22.3	0.816	2.00E-05	7.00E-04				-0.77	2.72667	6	2
GNA14	guanine nucleotide binding protein (G protein), alpha 14	9q21				-0.307	0.00238	0.0268				1
GNA15	guanine nucleotide binding protein (G protein), alpha 15 (Gq class)	19p13.3	-2.55	1.00E-05	3.00E-04	-0.434857143	0.002498571	0.0216				2
GNAI1	guanine nucleotide binding protein (G protein), alpha inhibiting activity polype	7q21				0.373833333	0.016736667	0.075483333				1
GNAI3	guanine nucleotide binding protein (G protein), alpha inhibiting activity polype	1p13	-0.907	2.00E-04	0.0036							1
GNAL	guanine nucleotide binding protein (G protein), alpha activating activity polyp	18p11.22-p11.21	-0.732	0.01303	0.0698							1
GNAS	GNAS complex locus	20q13.3				0.3845	0.00174	0.02015				1
GNAT1	guanine nucleotide binding protein (G protein), alpha transducing activity poly	3p21	-0.205	0.03785	0.1405	-0.427	0.0034	0.032				2
GNAT2	guanine nucleotide binding protein (G protein), alpha transducing activity poly	1p13.1	-0.216	0.01896	0.0896							1
GNAT3	In multiple Geneids					0.3485	0.033655	0.115625				1
GNAZ	guanine nucleotide binding protein (G protein), alpha z polypeptide	22q11.22				-0.394454545	0.001199091	0.015127273				1
CD200	CD200 molecule	3q12-q13	0.816	0.00438	0.0333							1
GNB1L	guanine nucleotide binding protein (G protein), beta polypeptide 1-like	22q11.2	-0.164	0.04761	0.1622	-0.378571429	0.001067143	0.015828571				2
GNB4	guanine nucleotide binding protein (G protein), beta polypeptide 4	3q26.33				-0.344	0.00344	0.0322				1

DDAH2	dimethylarginine dimethylaminohydrolase 2	6p21.3	0.816	0.00247	0.0222							1
GNE	glucosamine (UDP-N-acetyl)-2-epimerase/N-acetylmannosamine kinase	9p13.3				-0.387818182	0.002921818	0.025209091				1
EDIL3	EGF-like repeats and discoidin I-like domains 3	5q14	0.816	0.04364	0.1537							1
GNG12	guanine nucleotide binding protein (G protein), gamma 12	1p31.3	-0.489	0.02104	0.0959							1
GNG13	guanine nucleotide binding protein (G protein), gamma 13	16p13.3	-0.204	0.03868	0.1426							1
GNG2	guanine nucleotide binding protein (G protein), gamma 2	14q21							-1.055	3.88333	6	1
GNG3	guanine nucleotide binding protein (G protein), gamma 3	11p11				-0.414	0.00026	0.0098				1
GNG4	guanine nucleotide binding protein (G protein), gamma 4	1q42.3	-1.463	0.0173	0.0841	-0.324	0.00159	0.0219				2
GNG7	guanine nucleotide binding protein (G protein), gamma 7	19p13.3				-0.44885	0.0061965	0.03651				1
GNG8	guanine nucleotide binding protein (G protein), gamma 8	19q13.32	-0.273	0.02336	0.1024							1
GNGT1	guanine nucleotide binding protein (G protein), gamma transducing activity p	7q21.3							-0.839	2.636	5	1
GNGT2	guanine nucleotide binding protein (G protein), gamma transducing activity p	17q21	-0.28	0.04822	0.1636	-0.391	0.0019	0.0239				2
FLOT1	flotillin 1	6p21.3	0.816	2.00E-05	7.00E-04							1
HNMT	histamine N-methyltransferase	2q22.1	0.816	0.00281	0.0244	0.335428571	0.001452857	0.0183				2
FNIP2	folliculin interacting protein 2	4q32.1	0.815	0.00227	0.0209							1
TMEM181	transmembrane protein 181	6q25.3	0.815	0.00052	0.0073							1
GNPDA2	glucosamine-6-phosphate deaminase 2	4p12				0.397	0.02435	0.0958				1
TMEM39A	transmembrane protein 39A	3q13.33	0.815	0.00053	0.0075				-0.819	2.55167	6	2
GNPTAB	N-acetylglucosamine-1-phosphate transferase, alpha and beta subunits	12q23.2	-0.342	0.03287	0.1279							1
GNPTG	N-acetylglucosamine-1-phosphate transferase, gamma subunit	16p13.3				-0.404666667	0.01411	0.068866667				1
GNRH1	gonadotropin-releasing hormone 1 (luteinizing-releasing hormone)	8p21-p11.2							-0.899	2.74429	7	1
GNRHR	gonadotropin-releasing hormone receptor	4q21.2	-0.653	0.00019	0.0036							1
GNRHR2	gonadotropin-releasing hormone (type 2) receptor 2	1q12	-0.256	0.01869	0.0888							1
BAIAP2L2	BAI1-associated protein 2-like 2	22q13.1	0.814	0.00189	0.0183							1
GOLGA1	golgin A1	9q33.3				-0.331	0.01513	0.0725				1
GOLGA2	golgin A2	9q34.11				-0.313	0.00153	0.0215				1
GOLGA2P2Y	golgin A2 pseudogene 2, Y-linked	Yq11.23	-0.352	0.00717	0.0466							1
GOLGA3	golgin A3	12q24.33				-0.37075	0.001955	0.020475				1
GOLGA4	golgin A4	3p22-p21.3							-0.679	2.56	5	1
GOLGA5	golgin A5	14q32.12				-0.307	0.00258	0.0278				1
GOLGA6A	golgin A6 family, member A	15q24.1	-0.228	0.04327	0.153							1
GOLGA6L5	golgin A6 family-like 5 (pseudogene)	15q25.2	-0.318	0.01955	0.0913							1
GOLGA6L9	golgin A6 family-like 9	15q25.2	-0.569	0.00035	0.0055							1
GOLGA7	golgin A7	8p11.21							0.78	2.432	5	1
GOLGA8A	golgin A8 family, member A	15q11.2				-0.35	0.00025	0.0096				1
GOLGA8B	golgin A8 family, member B	15q14				-0.3205	0.002285	0.02615				1
GOLGA8EP	Data not found		-0.782	0.00504	0.0367							1
GOLGA8F	In multiple Geneids								1.004	2.664	5	1
GOLGA8G	golgin A8 family, member G	15q13.1							1.079	2.428	5	1
KLC4	kinesin light chain 4	6p21.1	0.814	0.00044	0.0065							1
RASGEF1A	RasGEF domain family, member 1A	10q11.21	0.813	0.00236	0.0215							1
HSPA13	heat shock protein 70kDa family, member 13	21q11	0.811	0.00046	0.0068							1
GOLPH2	Data not found								-0.801	3.172	5	1
GOLPH3	golgi phosphoprotein 3 (coat-protein)	5p13.3				0.352	0.0374	0.1243				1
GOLPH3L	golgi phosphoprotein 3-like	1q21.3							-0.836	2.54667	6	1
GOLSYN	Data not found					0.3666	0.000836	0.01454				1
FANCI	In multiple Geneids		0.81	0.01141	0.064							1
MCM8	minichromosome maintenance complex component 8	20p12.3	0.81	0.0205	0.0943							1
BTN2A1	butyrophilin, subfamily 2, member A1	6p22.1	0.809	7.00E-05	0.0017							1
NUDT4P1	In multiple Geneids		0.809	0.00085	0.0105							1
GORASP1	golgi reassembly stacking protein 1, 65kDa	3p22-p21.33				-0.312	0.00047	0.0125				1
CKS2	CDC28 protein kinase regulatory subunit 2	9q22	0.808	0.01444	0.0746							1
GOT1	glutamic-oxaloacetic transaminase 1, soluble (aspartate aminotransferase 1)	10q24.1-q25.1	-0.847	0.00022	0.0039	-0.307	0.00021	0.009				2
GOT2	glutamic-oxaloacetic transaminase 2, mitochondrial (aspartate aminotransfer	16q21	-0.378	0.04971	0.1665							1
GP1BB	glycoprotein Ib (platelet), beta polypeptide	22q11.21	-0.363	0.00388	0.0306							1
GP6	glycoprotein VI (platelet)	19q13.4	-0.436	0.00067	0.0089	-0.364	0.00075	0.0155				2
GP9	glycoprotein IX (platelet)	3q21.3	-0.26	0.04178	0.1497							1
NNMT	nicotinamide N-methyltransferase	11q23.1	0.808	0.02197	0.0987							1
CDC42SE2	CDC42 small effector 2	5q31.1	0.807	0.00104	0.012							1
GPATCH1	G patch domain containing 1	19q13.11				-0.3115	0.012485	0.0644				1
RAD21	RAD21 homolog (S. pombe)	8q24	0.807	0.00015	0.003	0.487428571	0.003344286	0.030457143				2
GPATCH3	G patch domain containing 3	1p35.3-p35.1				-0.41	0.02721	0.1024				1
CLDN15	claudin 15	7q11.22	0.806	0.00031	0.0051							1
GPATCH8	G patch domain containing 8	17q21.31				-0.316	0.03144	0.1117				1
GPBAR1	G protein-coupled bile acid receptor 1	2q35				-0.309	0.0011	0.0184				1
GPBP1	GC-rich promoter binding protein 1	5q11.2				-0.343	0.001616667	0.020533333				1
GPC1	glypican 1	2q35-q37	-0.797	0.00024	0.0042							1
PLXNA3	In multiple Geneids		0.806	3.00E-05	9.00E-04							1
PPFIA1	protein tyrosine phosphatase, receptor type, f polypeptide (PTPRF), interactin	11q13.3	0.806	1.00E-05	3.00E-04							1

GPC5	glypican 5	13q32				0.343583333	0.006909167	0.043713889				1
CMTM8	CKLF-like MARVEL transmembrane domain containing 8	3p22.3	0.805	0.00078	0.0099							1
GPD1	glycerol-3-phosphate dehydrogenase 1 (soluble)	12q12-q13				-0.442	0	8.00E-04				1
GPD1L	glycerol-3-phosphate dehydrogenase 1-like	3p22.3	-1.512	6.00E-05	0.0014	-0.345	0.003745	0.02915				2
GPD2	glycerol-3-phosphate dehydrogenase 2 (mitochondrial)	2q24.1				0.361	0.00014	0.00655				1
GPHB5	glycoprotein hormone beta 5	14q23.2							-0.952	4.25	5	1
GPHN	In multiple Geneids					-0.346	0.00432	0.0362				1
GPI	glucose-6-phosphate isomerase	19q13.1				-0.48875	0.001135	0.01265				1
GPIHBP1	glycosylphosphatidylinositol anchored high density lipoprotein binding protei	8q24.3	-0.273	0.01904	0.0899							1
XPNPEP2	X-prolyl aminopeptidase (aminopeptidase P) 2, membrane-bound	Xq25	0.805	0.04587	0.1587							1
GPM6A	glycoprotein M6A	4q34							-0.868	2.78	6	1
GPM6B	glycoprotein M6B	Xp22.2				0.311	0.00065	0.0144				1
GPN2	GPN-loop GTPase 2	1p36.11				-0.392	0.02158	0.08845				1
GPR1	G protein-coupled receptor 1	2q33.3	-0.903	0	2.00E-04							1
GPR101	G protein-coupled receptor 101	Xq26.3							1.078	4.01667	6	1
GPR107	G protein-coupled receptor 107	9q34.11				-0.319	0.01391	0.069				1
GPR108	G protein-coupled receptor 108	19p13.3	-0.475	0.00239	0.0217	-0.429	0.00056	0.0136				2
GPR109A	Data not found								0.982	3.86833	6	1
GPR109B	Data not found								1.05	4.05333	6	1
GPR110	G protein-coupled receptor 110	6p12.3							-0.596	2.644	5	1
GPR111	G protein-coupled receptor 111	6p12.3				0.35	0.004315	0.032				1
CASP6	caspase 6, apoptosis-related cysteine peptidase	4q25	0.804	0.00111	0.0126							1
GPR115	G protein-coupled receptor 115	6p12.3	-1.095	0.00018	0.0034							1
CDX2	caudal type homeobox 2	13q12.3	0.804	0.00408	0.0318							1
GPR12	G protein-coupled receptor 12	13q12	-0.267	0.03041	0.1219				1.055	3.11125	8	2
GPR120	Data not found					-0.315	0.00103	0.0179				1
GPR123	G protein-coupled receptor 123	10q26				-0.33	0.0029	0.0295				1
LINC00346	long intergenic non-protein coding RNA 346	13q34	0.804	0.00426	0.0327							1
PDIA6	protein disulfide isomerase family A, member 6	2p25.1	0.804	0.00158	0.0162							1
SNORA5A	small nucleolar RNA, H/ACA box 5A	7p13	0.804	3.00E-04	0.0049							1
GPR132	G protein-coupled receptor 132	14q32.3				-0.3775	0.001575	0.0217				1
GPR133	G protein-coupled receptor 133	12q24.33				0.321	0.00022	0.009				1
GPR137	G protein-coupled receptor 137	11cen-q22.3	-0.382	0.00029	0.0048							1
ZNF322	In multiple Geneids		0.804	0.00889	0.05385							1
GPR139	In multiple Geneids					0.321	0.00075	0.0155				1
GPR143	G protein-coupled receptor 143	Xp22.3	-0.39	0.03968	0.1448							1
GPR144	G protein-coupled receptor 144	9q33.3	-0.239	0.00749	0.0481							1
GPR148	G protein-coupled receptor 148	2q21.1	-0.224	0.0394	0.1441							1
GPR149	G protein-coupled receptor 149	3q25.2				0.3275	0.00931	0.04775	-0.873	3.035	6	2
GPR15	G protein-coupled receptor 15	3q11.2-q13.1	-1.104	0.01142	0.064							1
GPR151	G protein-coupled receptor 151	5q32							-0.765	3.08	6	1
GPR155	G protein-coupled receptor 155	2q31.1	-1.178	0.02912	0.1182							1
GPR156	G protein-coupled receptor 156	3q13.33							-0.864	2.7	5	1
GPR157	G protein-coupled receptor 157	1p36.23	-1.592	0	1.00E-04	-0.386666667	0.003783333	0.030166667				2
ENPP4	In multiple Geneids		0.803	0.00068	0.0089	0.305	0.03932	0.1282				2
MFGE8	milk fat globule-EGF factor 8 protein	15q25	0.803	0.01111	0.0627							1
GPR161	G protein-coupled receptor 161	1q24.2							0.656	2.915	6	1
GPR171	G protein-coupled receptor 171	3q25.1							-0.901	3.30857	7	1
GPR174	G protein-coupled receptor 174	Xq21.1				0.313	0.00133	0.0201	-0.936	3.22889	9	2
NR2F2	nuclear receptor subfamily 2, group F, member 2	15q26	0.803	0.01104	0.0625	0.4	0.00194	0.0242				2
GPR177	Data not found								-0.808	2.832	5	1
GPR179	G protein-coupled receptor 179	17q21.1				-0.329	0.006655	0.04325				1
GPR18	G protein-coupled receptor 18	13q32							-0.88	2.79833	6	1
PDE4DIP	phosphodiesterase 4D interacting protein	1q12	0.803	0.00183	0.0179	0.378	2.00E-05	0.004				2
QSOX2	quiescin Q6 sulfhydryl oxidase 2	9q34.3	0.803	3.00E-05	8.00E-04							1
GPR22	G protein-coupled receptor 22	7q22-q31.1							-0.893	2.79429	7	1
GPR23	Data not found								-0.757	2.94	5	1
GPR25	G protein-coupled receptor 25	1q32.1	-0.299	0.01146	0.0641				0.751	2.77167	6	2
SCYL3	SCY1-like 3 (S. cerevisiae)	1q24.2	0.803	0.00246	0.0221				-0.525333333	2.503943333	5.333333333	2
GPR37	G protein-coupled receptor 37 (endothelin receptor type B-like)	7q31				0.39	0.016605	0.0764				1
HELB	helicase (DNA) B	12q14.3	0.802	3.00E-05	0.001							1
MAN2A2	mannosidase, alpha, class 2A, member 2	15q26.1	0.802	0.00294	0.0252							1
GPR50	G protein-coupled receptor 50	Xq28	-0.377	0.00146	0.0153				0.639	2.856	5	2
GPR55	G protein-coupled receptor 55	2q37	-0.306	0.01227	0.0672							1
GPR6	G protein-coupled receptor 6	6q21	-0.274	0.03498	0.1333							1
GPR61	G protein-coupled receptor 61	1p13.3	-0.275	0.00276	0.0241							1
GPR65	G protein-coupled receptor 65	14q31-q32.1							-1.014	3.24	5	1
GPR68	G protein-coupled receptor 68	14q31				-0.3065	0.00848	0.0523				1
GPR78	G protein-coupled receptor 78	4p16.1	-0.806	0	0							1

GPR82	G protein-coupled receptor 82	Xp11.4							-0.754	3.04125	8	1
GPR87	G protein-coupled receptor 87	3q24	-2.476	0	2.00E-04							1
GPR88	G protein-coupled receptor 88	1p21.3				-0.339	3.00E-05	0.0041				1
ASXL1	additional sex combs like 1 (Drosophila)	20q11	0.801	1.00E-05	3.00E-04							1
GPR98	G protein-coupled receptor 98	5q13				-0.402	0.00036	0.0111				1
EDNRB	endothelin receptor type B	13q22	0.801	0.02277	0.1007	0.36	0.00478	0.0383				2
ANKRD36B	ankyrin repeat domain 36B	2q11.2	0.8	0.02108	0.090566667							1
SCML1	sex comb on midleg-like 1 (Drosophila)	Xp22	0.8	0.00073	0.0094							1
GPRC5D	G protein-coupled receptor, family C, group 5, member D	12p13.3	-0.71	0.00142	0.015							1
GPRC6A	In multiple Geneids								-0.887	3.20875	8	1
GPRIN1	G protein regulated inducer of neurite outgrowth 1	5q35.2				-0.396	0.001335	0.018125				1
FBLIM1	filamin binding LIM protein 1	1p36.21	0.799	0.00011	0.0024							1
GPSM2	G-protein signaling modulator 2	1p13.3	-1.01	0.02381	0.1036							1
GPT	glutamic-pyruvate transaminase (alanine aminotransferase)	8q24.3	-0.299	0.03092	0.1233							1
GPT2	glutamic pyruvate transaminase (alanine aminotransferase) 2	16q12.1	-2.52	0	0	-0.3455	0.0027425	0.027375				2
GPX1	glutathione peroxidase 1	3p21.3				-0.521	0.00026	0.0097				1
GPR137B	G protein-coupled receptor 137B	1q42-q43	0.798	0.00066	0.0087							1
GPX3	glutathione peroxidase 3 (plasma)	5q23	-1.946	1.00E-05	3.00E-04							1
OSBPL7	oxysterol binding protein-like 7	17q21	0.798	0.00486	0.0358							1
GPX5	glutathione peroxidase 5 (epididymal androgen-related protein)	6p22.1							-0.885	3.692	5	1
GPX6	In multiple Geneids		-0.415	0.00114	0.0128							1
GPX7	glutathione peroxidase 7	1p32				-0.317	0.01511	0.0724				1
CD80	CD80 molecule	3q13.3-q21	0.797	0.00148	0.0154							1
GPR180	G protein-coupled receptor 180	13q32.1	0.797	6.00E-04	0.0082							1
MS4A4A	membrane-spanning 4-domains, subfamily A, member 4A	11q12	0.797	0.02971	0.1198				-0.753	2.68	7	2
GRAMD1C	GRAM domain containing 1C	3q13.31	-0.855	0.00255	0.0227							1
GRAMD3	GRAM domain containing 3	5q23.2				-0.33	0.0030575	0.0238				1
GRAMD4	GRAM domain containing 4	22q13.31				-0.434666667	0.000298333	0.0088				1
GRAP2	GRB2-related adaptor protein 2	22q13.2				-0.337071429	0.002539286	0.020378571				1
HLA-H	major histocompatibility complex, class I, H (pseudogene)	6p21.3	0.796	0.00108	0.0123							1
MCM7	minichromosome maintenance complex component 7	7q21.3-q22.1	0.796	0.00261	0.0231							1
SLC37A4	solute carrier family 37 (glucose-6-phosphate transporter), member 4	11q23.3	0.796	0.00104	0.012							1
GRB2	growth factor receptor-bound protein 2	17q24-q25				-0.441	0.006555	0.0405				1
TMEM144	transmembrane protein 144	4q32.1	0.796	0.00424	0.0326							1
GRHL1	grainyhead-like 1 (Drosophila)	2p25.1	-2.622	1.00E-05	5.00E-04							1
GRHL2	grainyhead-like 2 (Drosophila)	8q22.3				0.3725	5.00E-06	0.00235				1
GRHL3	grainyhead-like 3 (Drosophila)	1p36.11	-3.605	0	1.00E-04	-0.305	0.00432	0.0363				2
GRHPR	glyoxylate reductase/hydroxypyruvate reductase	9q12	-0.544	3.00E-05	9.00E-04	-0.334	0.00313	0.02615				2
GRIA1	glutamate receptor, ionotropic, AMPA 1	5q31.1				-0.371	0.00882	0.0535				1
GRIA3	glutamate receptor, ionotropic, AMPA 3	Xq25				0.352966667	0.003957333	0.03041				1
GRIA4	glutamate receptor, ionotropic, AMPA 4	11q22				0.355733333	0.007942	0.04514				1
GRID1	glutamate receptor, ionotropic, delta 1	10q22				-0.3674	0.004838	0.0257				1
GRID2	glutamate receptor, ionotropic, delta 2	4q22				0.308	0.00152	0.0215				1
GRID2IP	In multiple Geneids					-0.301	0.01979	0.0847				1
GRIK3	glutamate receptor, ionotropic, kainate 3	1p34.3	-0.259	0.01156	0.0645							1
GRIK4	glutamate receptor, ionotropic, kainate 4	11q22.3				0.318	0.00508	0.0395				1
GRIK5	glutamate receptor, ionotropic, kainate 5	19q13.2				-0.401285714	0.001394286	0.017757143	0.547	2.38833	6	2
GRIN2A	glutamate receptor, ionotropic, N-methyl D-aspartate 2A	16p13.2				0.8625	0.000415	0.01005				1
GRIN2B	glutamate receptor, ionotropic, N-methyl D-aspartate 2B	12p12				0.327	0.0018	0.019375				1
ZNF189	zinc finger protein 189	9q22-q31	0.796	0.00115	0.0129							1
LGSN	lengsin, lens protein with glutamine synthetase domain	6pter-q22.33	0.795	0.04433	0.1554							1
GRIP1	glutamate receptor interacting protein 1	12q14.3	-1.034	3.00E-05	9.00E-04							1
GRIP2	glutamate receptor interacting protein 2	3p24-p23				-0.341	0.00019	0.0084				1
C8orf44	chromosome 8 open reading frame 44	8q13.1	0.794	0.00064	0.0086							1
GRK1	G protein-coupled receptor kinase 1	13q34	-0.186	0.04934	0.1658							1
GRK4	G protein-coupled receptor kinase 4	4p16.3	-0.262	0.00305	0.0259	-0.374727273	0.001332727	0.018654545				2
ZEB1	zinc finger E-box binding homeobox 1	10p11.2	0.794	0.02092	0.0956							1
GRLF1	Data not found					-0.3085	0.00279	0.02895				1
GRM1	glutamate receptor, metabotropic 1	6q24				0.3105	0.011915	0.06285				1
GRM2	glutamate receptor, metabotropic 2	3p21.2				-0.423	0.00021	0.009				1
GRM3	glutamate receptor, metabotropic 3	7q21.1-q21.2				0.343826087	0.006520435	0.041473913				1
GRM5	glutamate receptor, metabotropic 5	11q14.3				0.32225	0.00036375	0.0103125	-0.7	3.098	5	2
GRM6	glutamate receptor, metabotropic 6	5q35				-0.348	0.00925	0.0548				1
GRM8	glutamate receptor, metabotropic 8	7q31.3-q32.1				0.34075	0.002445	0.02395				1
GRN	granulin	17q21.32	-0.654	5.00E-04	0.0071							1
GRP	gastrin-releasing peptide	18q21.1-q21.32				-0.305	0.00332	0.0316				1
GRPEL1	GrpE-like 1, mitochondrial (E. coli)	4p16	-0.236	0.03509	0.1335	-0.37	0.00022	0.0091				2
GRPEL2	GrpE-like 2, mitochondrial (E. coli)	5q32							-1.005	2.882	5	1
GRPR	gastrin-releasing peptide receptor	Xp22.2				0.362	0.00757	0.0491	-0.856	3.10125	8	2

GRTP1	growth hormone regulated TBC protein 1	13q34				-0.381	0.01742	0.0773	0.681	2.992	5	2
GRWD1	glutamate-rich WD repeat containing 1	19q13.33				-0.308	0.00704	0.0472				1
GSDM1	Data not found								0.652	2.745	6	1
GSDMA	gasdermin A	17q21.1	-0.937	0.00194	0.0187							1
C6orf48	chromosome 6 open reading frame 48	6p21.3	0.793	0.00853	0.0523							1
GSDMC	gasdermin C	8q24.21				0.414	0.02174	0.0873				1
PAK1	p21 protein (Cdc42/Rac)-activated kinase 1	11q13-q14	0.793	2.00E-05	6.00E-04							1
GSG1L	GSG1-like	16p12.1	-0.278	0.00511	0.037	-0.378333333	0.00252	0.019433333				2
GSG2	germ cell associated 2 (haspin)	17p13				-0.387	0.0103	0.0582				1
GSH1	Data not found								1.02	2.93	6	1
GSN	gelsolin	9q33	-0.769	0.00199	0.019							1
GSPT1	G1 to S phase transition 1	16p13.1				-0.317	0.00065	0.0145				1
GSTA4	glutathione S-transferase alpha 4	6p12.1	-1.214	7.00E-04	0.0092							1
GSTCD	glutathione S-transferase, C-terminal domain containing	4q24							-0.6715	2.706665	8	1
GSTK1	glutathione S-transferase kappa 1	7q34				-0.411	0.00394	0.0346				1
GSTM2	glutathione S-transferase mu 2 (muscle)	1p13.3	-1.181	2.00E-05	7.00E-04							1
GSTM3	glutathione S-transferase mu 3 (brain)	1p13.3							-1.109	2.974	5	1
RTKN	rhotekin	2p13.1	0.793	0.00068	0.009							1
GSTP1	glutathione S-transferase pi 1	11q13				-0.334	0.00485	0.0386				1
GSTT1	glutathione S-transferase theta 1	22q11.23				-0.979	0.00495	0.039				1
GSTT2	glutathione S-transferase theta 2	22q11.23	-1.156	0.0062	0.0422							1
GSTTP1	glutathione S-transferase theta pseudogene 1	22q12	-0.287	0.01857	0.0883	-0.459	0.011715	0.0593				2
GSTTP2	glutathione S-transferase theta pseudogene 2	22q11.23				-0.329	0.02785	0.1038				1
GSTZ1	glutathione transferase zeta 1	14q24.3	-0.785	0	0	-0.302	0.00265	0.0282				2
GTDC1	glycosyltransferase-like domain containing 1	2q22.3				0.3453	0.000428	0.01022	-0.907	2.69286	7	2
SNORA71C	small nucleolar RNA, H/ACA box 71C	20q11.23	0.793	0.01944	0.0911							1
GTF2E2	general transcription factor IIE, polypeptide 2, beta 34kDa	8p12				-0.312	0.04961	0.1481				1
GTF2F1	general transcription factor IIF, polypeptide 1, 74kDa	19p13.3				-0.620666667	0.00024	0.008966667				1
GTF2H1	general transcription factor IIH, polypeptide 1, 62kDa	11p15.1-p14				-0.302	0.02551	0.0985				1
MSANTD4	Myb/SANT-like DNA-binding domain containing 4 with coiled-coils	11q22	0.792	0.00203	0.0193							1
GTF2H3	general transcription factor IIH, polypeptide 3, 34kDa	12q24.31				-0.316	0.00063	0.0143				1
GTF2I	general transcription factor IIi	7q11.23				-0.366	0.0163	0.070333333				1
GTF2IP1	general transcription factor IIi, pseudogene 1	7q11.23				-0.3205	0.007845	0.0463				1
GTF2IRD1	GTF2I repeat domain containing 1	7q11.23				-0.371	0.0183125	0.075975				1
GTF2IRD2	GTF2I repeat domain containing 2	7q11.23				-0.4144	0.018796	0.08048	1.395	2.652	5	2
GTF2IRD2B	GTF2I repeat domain containing 2B	7q11.23				-0.337333333	0.036998333	0.1225	1.395	2.652	5	2
GTF2IRD2P	Data not found					-0.3785	0.012835	0.06535				1
GTF3C1	general transcription factor IIIC, polypeptide 1, alpha 220kDa	16p12	-0.827	4.00E-04	0.0061	-0.304	0.00851	0.0457				2
SCARNA10	small Cajal body-specific RNA 10	12p13.31	0.792	0.03886	0.1428							1
GTPBP1	GTP binding protein 1	22q13.1				-0.405222222	0.003317778	0.022644444				1
GTPBP3	GTP binding protein 3 (mitochondrial)	19p13.11				-0.343	0.01703	0.0776				1
FAM111A	family with sequence similarity 111, member A	11q12.1	0.791	3.00E-05	0.001				-0.874	3.27833	6	2
GTPBP6	GTP binding protein 6 (putative)	Xp22.33; Yp11.32				-0.329	0.00518	0.04				1
GTPBP8	GTP-binding protein 8 (putative)	3q13.2							-0.807	2.652	5	1
GTSE1	G-2 and S-phase expressed 1	22q13.2-q13.3				-0.455571429	9.57E-05	0.005542857				1
GUCA1A	guanylate cyclase activator 1A (retina)	6p21.1	-0.2	0.04797	0.163							1
GUCA1C	guanylate cyclase activator 1C	3q13.1							-0.918	2.866	5	1
GUCY1A2	guanylate cyclase 1, soluble, alpha 2	11q21-q22				0.526666667	0.005818333	0.033283333				1
FGFR1OP	FGFR1 oncogene partner	6q27	0.791	8.00E-04	0.0101							1
SNORA8	small nucleolar RNA, H/ACA box 8	11q21	0.791	0.0171	0.0836							1
SNRPC	small nuclear ribonucleoprotein polypeptide C	6p21.31	0.791	0.00244	0.022							1
CNNM4	cyclin M4	2q11	0.79	0.00058	0.008							1
GUCY2D	guanylate cyclase 2D, membrane (retina-specific)	17p13.1				-0.39525	0.0010275	0.017075				1
GUCY2F	guanylate cyclase 2F, retinal	Xq22				0.3678	0.001092	0.01756				1
GUK1	guanylate kinase 1	1q32-q41	-0.317	0.00148	0.0154							1
GULP1	GULP, engulfment adaptor PTB domain containing 1	2q32.3-q33				0.333666667	0.000296667	0.008311111				1
ZNF701	zinc finger protein 701	19q13.41	0.789	0.00202	0.0192							1
RPS27L	ribosomal protein S27-like	15q22.2	0.788	1.00E-04	0.0023							1
SPRED1	sprouty-related, EVH1 domain containing 1	15q14	0.788	3.00E-05	9.00E-04	0.318	0.00629	0.0444				2
LINC00152	long intergenic non-protein coding RNA 152	2p11.2	0.7875	0.010935	0.05345							1
BTN3A1	butyrophilin, subfamily 3, member A1	6p22.1	0.787	0.00209	0.0197							1
GYG1	glycogenin 1	3q24-q25.1				0.339	1.00E-05	0.003				1
KIF2C	kinesin family member 2C	1p34.1	0.787	0.00596	0.0411							1
GYPA	glycophorin A (MNS blood group)	4q31.21							-0.786	2.604	5	1
GYPE	glycophorin E (MNS blood group)	4q31.1							-0.745	2.82667	6	1
GYS1	glycogen synthase 1 (muscle)	19q13.3	-0.673	4.00E-05	0.0011	-0.425	0.002706667	0.0282				2
GYS2	glycogen synthase 2 (liver)	12p12.2				0.36225	0.0004925	0.012025	-0.853	3.385	8	2
GZMH	granzyme H (cathepsin G-like 2, protein h-CCPX)	14q11.2	-0.307	0.00499	0.0365							1
GZMK	granzyme K (granzyme 3; tryptase II)	5q11.2							-0.915	2.77	6	1

H19	In multiple Geneids								0.666	3.02583	6	1
H2AFB1	H2A histone family, member B1	Xq28							0.658	2.81	6	1
H2AFB3	H2A histone family, member B3	Xq28							0.658	2.81	6	1
H2AFJ	In multiple Geneids		-0.283	0.01057	0.0609							1
JAM3	junctional adhesion molecule 3	11q25	0.786	0.01351	0.0713							1
H2AFY2	In multiple Geneids		-0.841	1.00E-05	4.00E-04							1
H3/o	histone H3/o	1q21-q23							-0.492	2.43417	6	1
H3F3B	H3 histone, family 3B (H3.3B)	17q25.1							-0.93	3.00571	7	1
H6PD	hexose-6-phosphate dehydrogenase (glucose 1-dehydrogenase)	1p36				-0.384	0.0012	0.0192				1
PTPRB	protein tyrosine phosphatase, receptor type, B	12q15-q21	0.786	0.00041	0.0062							1
ZNF827	zinc finger protein 827	4q31.22	0.786	0.00888	0.0538							1
HACE1	HECT domain and ankyrin repeat containing E3 ubiquitin protein ligase 1	6q16.3				0.451	0.00032	0.0107				1
PGM3	phosphoglucomutase 3	6q14.1-q15	0.785	8.00E-04	0.0101							1
HADH	In multiple Geneids					-0.361666667	0.00208	0.023133333				1
HADHB	hydroxyacyl-CoA dehydrogenase/3-ketoacyl-CoA thiolase/enoyl-CoA hydratas	2p23	-0.552	0.00093	0.0111							1
HADHSC	Data not found								-0.859	2.978	5	1
HAGH	hydroxyacylglutathione hydrolase	16p13.3				-0.402	0.02575	0.099				1
HAL	histidine ammonia-lyase	12q22-q24.1							-0.754	2.736	5	1
HAND1	heart and neural crest derivatives expressed 1	5q33	-0.227	0.04569	0.1584	-0.374	1.00E-05	0.0029				2
HAND2	heart and neural crest derivatives expressed 2	4q33				-0.31	0.00623	0.0441				1
HAO1	In multiple Geneids					0.3436	0.013856	0.06484				1
HAP1	huntingtin-associated protein 1	17q21.2-q21.3				-0.3835	0.00242	0.02265				1
HAPLN1	hyaluronan and proteoglycan link protein 1	5q14.3				-0.304	0.00282	0.0291				1
HAPLN4	hyaluronan and proteoglycan link protein 4	19p13.1				-0.441	0.00067	0.0147				1
HARS	histidyl-tRNA synthetase	5q31.3	-0.326	0.0182	0.0872	-0.434	0.00015	0.0078				2
HARS2	histidyl-tRNA synthetase 2, mitochondrial (putative)	5q31.3				-0.357	0.000175	0.0081				1
ARL6IP1	ADP-ribosylation factor-like 6 interacting protein 1	16p12-p11.2	0.784	0	2.00E-04							1
HAS3	hyaluronan synthase 3	16q22.1	-0.765	0.009	0.0543	-0.445	0.00109	0.0183				2
BRIX1	BRX1, biogenesis of ribosomes, homolog (S. cerevisiae)	5p13.2	0.784	0.0025	0.0224							1
HAUS4	HAUS augmin-like complex, subunit 4	14q11.2				-0.3405	0.00177	0.0222				1
HAUS5	HAUS augmin-like complex, subunit 5	19q13.12				-0.394	0.00056	0.0136				1
HAUS6	HAUS augmin-like complex, subunit 6	9p22.1				-0.37575	0.020225	0.0762				1
HAUS8	HAUS augmin-like complex, subunit 8	19p13.11				-0.402125	0.0038125	0.0291625				1
DNAJA4	DnaJ (Hsp40) homolog, subfamily A, member 4	15q25.1	0.784	0.0052	0.0375							1
HAVCR2	hepatitis A virus cellular receptor 2	5q33.3							-0.839	2.91714	7	1
HBB	hemoglobin, beta	11p15.5							-0.852	3.19333	9	1
HBD	In multiple Geneids								-0.812	3.30571	7	1
HBEGF	heparin-binding EGF-like growth factor	5q23				-0.389	0.00059	0.0139				1
HBG1	hemoglobin, gamma A	11p15.5							-0.7478	2.858722	6.8	1
HBG2	hemoglobin, gamma G	11p15.5							-0.723	2.75857	7	1
HBP1	HMG-box transcription factor 1	7q22-q31	-0.693	0.00371	0.0297							1
HBQ1	hemoglobin, theta 1	16p13.3	-0.221	0.04679	0.1605							1
HCAP-G	Data not found								-0.676	2.896	5	1
ITPK1	inositol-tetrakisphosphate 1-kinase	14q31	0.784	0.00097	0.0115							1
HCAR2	hydroxycarboxylic acid receptor 2	12q24.31	-1.934	8.00E-05	0.0019							1
HCAR3	hydroxycarboxylic acid receptor 3	12q24.31	-1.917	0.00047	0.0069							1
HCCA2	Data not found					-0.36485	0.0036555	0.02965	0.551	2.809	5	2
HCCS	holocytochrome c synthase	Xp22.3				0.31	0.01344	0.0676				1
HCFC2	host cell factor C2	12q23.3	-0.615	0.00183	0.0179	-0.303	0.00126	0.0196				2
HCK	hemopoietic cell kinase	20q11-q12							0.79	3.06438	8	1
HCN1	In multiple Geneids					0.396553571	0.006953929	0.041296429				1
HCN2	hyperpolarization activated cyclic nucleotide-gated potassium channel 2	19p13.3	-0.528	0.00065	0.0087	-0.386	0.00947	0.0556				2
SMCHD1	structural maintenance of chromosomes flexible hinge domain containing 1	18p11.32	0.7815	0.000995	0.01075							1
HCRT	hypocretin (orexin) neuropeptide precursor	17q21	-0.236	0.04192	0.15							1
HCRTR1	hypocretin (orexin) receptor 1	1p33	-0.22	0.0203	0.0937							1
HCST	hematopoietic cell signal transducer	19q13.1				-0.395	0.00333	0.0317				1
HDAC1	histone deacetylase 1	1p34				-0.413	0.00295	0.0298				1
HDAC10	histone deacetylase 10	22q13.31				-0.396	0	0.0022				1
HDAC11	histone deacetylase 11	3p25.1	-0.545	0.00157	0.0161	-0.45	2.50E-05	0.004				2
HDAC3	histone deacetylase 3	5q31	-0.256	0.02133	0.0969	-0.33	0.00052	0.0131				2
HDAC4	histone deacetylase 4	2q37.3				0.359	8.00E-05	0.0061				1
HDAC5	histone deacetylase 5	17q21	-0.456	0.00636	0.043	-0.358	0.01346	0.0677				2
HDAC8	In multiple Geneids					0.31	0.000295	0.00885				1
HDAC9	histone deacetylase 9	7p21.1				0.356410714	0.003189821	0.026375	-0.821	3.18	6	2
HDC	histidine decarboxylase	15q21-q22				-0.337	0.011705	0.0592				1
HDCMA18P	Data not found								-0.806	3.09857	7	1
RGL3	ral guanine nucleotide dissociation stimulator-like 3	19p13.2	0.781	0.02357	0.103							1
ABL2	v-abl Abelson murine leukemia viral oncogene homolog 2	1q25.2	0.78	0	2.00E-04	0.409	7.00E-05	0.0058				2
HDGF2	Data not found					-0.52825	0.00206	0.020775				1

HDHD1A	Data not found					-0.349	0.02623	0.0973				1
HDHD2	haloacid dehalogenase-like hydrolase domain containing 2	18q21.1	-0.485	0.00962	0.0569	-0.308	0.02788	0.10385				2
ARHGAP26	Rho GTPase activating protein 26	5q31	0.78	0.00461	0.0346							1
HDX	highly divergent homeobox	Xq21.1				0.3815	0.0007025	0.013225				1
BID	BH3 interacting domain death agonist	22q11.1	0.78	3.00E-05	9.00E-04							1
HEATR3	HEAT repeat containing 3	16q12.1				-0.341	0.000585	0.0138				1
HEATR4	HEAT repeat containing 4	14q24.3				-0.360166667	0.010421667	0.054933333				1
HEATR5A	HEAT repeat containing 5A	14q12				-0.350666667	0.001343333	0.0176				1
HEATR7B2	HEAT repeat family member 7B2	5p13.1				0.384666667	0.014373333	0.067916667				1
HEBP2	heme binding protein 2	6q24	-0.79	0.00179	0.0177							1
HECTD1	HECT domain containing E3 ubiquitin protein ligase 1	14q12	-0.918	4.00E-05	0.0012	-0.376	0.00363	0.028625				2
HECTD4	Data not found		-0.3565	0.011915	0.0548							1
HECW1	HECT, C2 and WW domain containing E3 ubiquitin protein ligase 1	7p13				0.323	0.00306	0.02905				1
HECW2	HECT, C2 and WW domain containing E3 ubiquitin protein ligase 2	2q32.3				0.309	0.00018	0.0084				1
FMN1	formin 1	15q13.3	0.78	0.01317	0.0701							1
HSPG2	heparan sulfate proteoglycan 2	1p36.1-p34	0.78	0.00128	0.014							1
HELQ	helicase, POLQ-like	4q21.23	-0.418	0.00309	0.0262	-0.33	0.00342	0.0321				2
HELT	helt bHLH transcription factor	4q35.1				-0.44	0.00163	0.0222				1
XRN2	5'-3' exoribonuclease 2	20p11.2-p11.1	0.78	0.00058	0.008	0.329	0.01319	0.0669				2
HEMGN	hemogen	9q22.33							-0.939666667	3.089523333	7	1
HEMK1	HemK methyltransferase family member 1	3p21.3				-0.304	0.00439	0.0365				1
HEMK2	Data not found								-0.827	2.786	5	1
DNTTIP1	deoxynucleotidyltransferase, terminal, interacting protein 1	20q13.12	0.779	2.00E-05	7.00E-04							1
HEPACAM	hepatic and glial cell adhesion molecule	11q24.2	-0.197	0.03429	0.1314							1
HEPACAM2	HEPACAM family member 2	7q21.3				0.390363636	0.006737273	0.035781818				1
SMOC1	SPARC related modular calcium binding 1	14q24.2	0.779	0.00348	0.0283							1
HERC2	HECT and RLD domain containing E3 ubiquitin protein ligase 2	15q13				-0.406333333	0.032936667	0.114566667	0.858	3.16111	9	2
HERC2P2	In multiple Geneids					-0.49425	0.0139775	0.0661				1
HERC3	HECT and RLD domain containing E3 ubiquitin protein ligase 3	4q21	-1.054	7.00E-05	0.0018	-0.311	0.00028	0.0101				2
HERC4	HECT and RLD domain containing E3 ubiquitin protein ligase 4	10q21.3				-0.374	0.00077	0.012066667				1
SNORA41	small nucleolar RNA, H/ACA box 41	2q33	0.779	0.02548	0.1086							1
HERC6	HECT and RLD domain containing E3 ubiquitin protein ligase family member 6	4q22.1	-1.906	3.00E-05	0.001	-0.358571429	0.001057143	0.016528571				2
HES1	hairy and enhancer of split 1, (Drosophila)	3q28-q29							-0.642	2.41	5	1
HES2	hairy and enhancer of split 2 (Drosophila)	1p36.31	-0.594	0.00017	0.0032							1
HES5	In multiple Geneids		-0.213	0.02231	0.0995							1
HES7	hairy and enhancer of split 7 (Drosophila)	17p13.1							-0.771	2.76667	6	1
HESX1	HESX homeobox 1	3p14.3							-0.913	2.96429	7	1
APAF1	apoptotic peptidase activating factor 1	12q23	0.778	0	0							1
CLDN4	claudin 4	7q11.23	0.778	0.00731	0.0473							1
HEXDC	hexosaminidase (glycosyl hydrolase family 20, catalytic domain) containing	17q25.3				-0.305	0.00323	0.0312				1
HEXIM1	hexamethylene bis-acetamide inducible 1	17q21.31	-0.451	0.03309	0.1284							1
HEXIM2	hexamethylene bis-acetamide inducible 2	17q21.31							-0.938	2.92167	6	1
COCH	coagulation factor C homolog, cochlin (Limulus polyphemus)	14q11.2-q13	0.778	0.00645	0.0433							1
LIMA1	LIM domain and actin binding 1	12q13	0.778	0.00305	0.0259							1
POT1	protection of telomeres 1 homolog (S. pombe)	7q31.33	0.778	0.00582	0.0405	0.306	0.03543	0.1202				2
HFE2	In multiple Geneids		-0.34	0.00144	0.0152							1
HGC6.3	uncharacterized LOC100128124	6q27	-0.295	0.00618	0.0421							1
SNORD52	small nucleolar RNA, C/D box 52	6p21.33	0.778	0.02672	0.112							1
ACVR1C	activin A receptor, type IC	2q24.1	0.777	0.01195	0.066							1
HGFAC	HGF activator	4p16	-0.202	0.03959	0.1445	-0.374	0.00016	0.008				2
HGS	hepatocyte growth factor-regulated tyrosine kinase substrate	17q25	-0.207	0.04743	0.1619							1
HHAT	hedgehog acyltransferase	1q32				0.345666667	0.001733333	0.020833333				1
HHCM	Mahlavu hepatocellular carcinoma	8q24.3							0.816	3.08167	6	1
BTBD3	BTB (POZ) domain containing 3	20p12.2	0.776	0.00716	0.0466	0.3774	0.010352	0.0531	-0.736	2.842	5	3
HHIPL1	HHIP-like 1	14q32				-0.3205	0.001285	0.0198				1
HHLA1	HERV-H LTR-associating 1	8q24				0.395111111	0.024572222	0.087844444				1
MMP14	matrix metallopeptidase 14 (membrane-inserted)	14q11-q12	0.776	0.00364	0.0293							1
HIAT1	hippocampus abundant transcript 1	1p21.2				-0.313	2.00E-05	0.0036				1
NME2	NME/NM23 nucleoside diphosphate kinase 2	17q21.3	0.776	0.00291	0.025							1
HIATL2	hippocampus abundant transcript-like 2	9q22.33				-0.334	0.0116875	0.057125				1
PLEKHG2	pleckstrin homology domain containing, family G (with RhoGef domain) meml	19q13.2	0.776	0.00303	0.0258							1
HIBCH	3-hydroxyisobutyryl-CoA hydrolase	2q32.2							-1.023	2.706	5	1
HIC1	hypermethylated in cancer 1	17p13.3							0.502	2.62167	6	1
HIC2	hypermethylated in cancer 2	22q11.21				-0.3945	0.0040725	0.030875				1
BMP8B	bone morphogenetic protein 8b	1p35-p32	0.775	5.00E-05	0.0014							1
HIF1AN	hypoxia inducible factor 1, alpha subunit inhibitor	10q24				-0.3465	7.50E-05	0.0052				1
HIF3A	In multiple Geneids					-0.3635	0.005405	0.03345				1
HIGD1A	HIG1 hypoxia inducible domain family, member 1A	3p22.1	-0.664	0.00571	0.0394							1
HIGD1B	HIG1 hypoxia inducible domain family, member 1B	17q21.31				-0.391	0.00431	0.0362				1

HILPDA	hypoxia inducible lipid droplet-associated	7q32.1	-1.198	0.02761	0.1141							1
MERTK	c-mer proto-oncogene tyrosine kinase	2q14.1	0.775	0.00839	0.0518							1
HINT1	histidine triad nucleotide binding protein 1	5q31.2							-0.651333333	2.717223333	7.333333333	1
HINT2	histidine triad nucleotide binding protein 2	9p13.3	-0.367	0.0353	0.1342							1
SLC16A1	solute carrier family 16, member 1 (monocarboxylic acid transporter 1)	1p12	0.774	0.01156	0.0645							1
HIP1R	huntingtin interacting protein 1 related	12q24				-0.411	1.00E-05	0.0024				1
HIPK1	homeodomain interacting protein kinase 1	1p13.2							-0.7855	2.410835	5.5	1
HIPK2	In multiple Geneids		-0.594	0.00568	0.0398	-0.3615	0.00174	0.02285				2
HIPK3	homeodomain interacting protein kinase 3	11p13	-0.383	0.04887	0.1647							1
HIPK4	homeodomain interacting protein kinase 4	19q13.2	-0.203	0.0429	0.1522	-0.317	0.00033	0.0108	0.79	2.96	5	3
HIRA	HIR histone cell cycle regulation defective homolog A (S. cerevisiae)	22q11.21				-0.398210526	0.001192632	0.014294737				1
HIRIP3	HIRA interacting protein 3	16p11.2				-0.388	0.00014	0.0075				1
HISPPD1	Data not found								-0.966	3.49375	8	1
HISPPD2A	Data not found					-0.343666667	0.00078	0.0143				1
HIST1H1A	histone cluster 1, H1a	6p21.3	-1.237	0.0126	0.0684				0.686	3.0016	5	2
HIST1H1B	histone cluster 1, H1b	6p22.1							-0.653	2.92643	7	1
SPATA17	spermatogenesis associated 17	1q41	0.774	0.00302	0.0257	0.344166667	0.00095	0.016733333				2
HIST1H1E	histone cluster 1, H1e	6p21.3							-0.803	2.959915	5.5	1
HIST1H2AE	histone cluster 1, H2ae	6p22.1							-0.712	2.86	5	1
ZC3H7A	zinc finger CCCH-type containing 7A	16p13-p12	0.774	3.00E-05	8.00E-04							1
HIST1H2BB	histone cluster 1, H2bb	6p21.3							-0.655	3.023	5	1
SLCO2B1	solute carrier organic anion transporter family, member 2B1	11q13	0.773	0.00543	0.0386							1
CADPS	Ca++-dependent secretion activator	3p14.2	0.772	0.04121	0.1485				-1.131	3.094	5	2
HIST1H2BH	histone cluster 1, H2bh	6p21.3							-0.753	2.66	7	1
HIST1H2BI	histone cluster 1, H2bi	6p22.1							-0.705	2.78857	7	1
SAMD4A	sterile alpha motif domain containing 4A	14q22.2	0.772	0.00137	0.0146							1
HIST1H2BL	histone cluster 1, H2bl	6p22.1							-0.827	3.51643	7	1
HIST1H2BM	histone cluster 1, H2bm	6p22.1							-0.84	3.36437	8	1
TRMT11	tRNA methyltransferase 11 homolog (S. cerevisiae)	6q11.1-q22.33	0.772	0.00541	0.0386							1
HIST1H3A	histone cluster 1, H3a	6p22.1							0.688	3.069	5	1
HIST1H3B	histone cluster 1, H3b	6p22.1							-0.896	2.827	5	1
HIST1H3C	histone cluster 1, H3c	6p22.1							-0.655	3.023	5	1
HIST1H3E	histone cluster 1, H3e	6p22.1							0.697	3.768	5	1
HIST1H3F	histone cluster 1, H3f	6p22.2							-0.753	2.66	7	1
HIST1H3G	histone cluster 1, H3g	6p22.1							-0.705	2.78857	7	1
HIST1H3H	histone cluster 1, H3h	6p22.1							-0.827	3.51643	7	1
HIST1H3I	histone cluster 1, H3i	6p22.1							-0.584	3.23333	6	1
HIST1H4A	histone cluster 1, H4a	6p22.1							0.686	3.0016	5	1
HIST1H4B	histone cluster 1, H4b	6p22.1							-0.838	2.93857	7	1
HIST1H4D	histone cluster 1, H4d	6p22.1							-0.874	2.46667	6	1
CYBA	cytochrome b-245, alpha polypeptide	16q24	0.771	6.00E-05	0.0015							1
HIST1H4G	histone cluster 1, H4g	6p22.1							-0.753	2.66	7	1
NME7	NME/NM23 family member 7	1q24	0.771	0.00031	0.005	0.37475	0.0003975	0.01085				2
NUF2	NUF2, NDC80 kinetochore complex component, homolog (S. cerevisiae)	1q23.3	0.771	0.02682	0.1122	0.322	0.00121	0.0193				2
HIST1H4L	histone cluster 1, H4l	6p22.1							-0.554	3.308	5	1
OCR1	ovarian cancer-related protein 1	1q32.1	0.771	0.01197	0.0661							1
SCRN1	secernin 1	7p14.3-p14.1	0.771	0.03184	0.1254							1
HIST2H2BF	histone cluster 2, H2bf	1q21.2	-0.6	0.00341	0.028							1
HIST2H3C	In multiple Geneids								-0.492	2.43417	6	1
CHST11	carbohydrate (chondroitin 4) sulfotransferase 11	12q	0.77	0.03429	0.1314							1
HIST4H4	histone cluster 4, H4	12p12.3	-0.317	0.04603	0.159							1
HIVEP1	human immunodeficiency virus type I enhancer binding protein 1	6p24-p22.3				0.436	0.00289	0.0294				1
HIVEP3	human immunodeficiency virus type I enhancer binding protein 3	1p34	-0.824	0.00108	0.0123	-0.304	0.01724	0.0781				2
GFM1	G elongation factor, mitochondrial 1	3q25	0.77	0.00058	0.008							1
HK1	hexokinase 1	10q22	-1.205	0	0	-0.333133333	0.001006667	0.014593333				2
HK3	hexokinase 3 (white cell)	5q35.2				-0.330666667	0.004203333	0.032833333				1
IFNGR1	interferon gamma receptor 1	6q23.3	0.77	0.00418	0.0323							1
LITAF	lipopolysaccharide-induced TNF factor	16p13.13	0.77	0.00979	0.0576							1
TMEM62	transmembrane protein 62	15q15.2	0.77	0.00777	0.0492							1
GALNT10	UDP-N-acetyl-alpha-D-galactosamine:polypeptide N-acetylgalactosaminyltran	5q33.2	0.769	0.0036	0.0291				-0.532	2.35	5	2
HLA-DPA1	major histocompatibility complex, class II, DP alpha 1	6p21.3							-0.592	2.8475	8	1
HLA-DPB1	major histocompatibility complex, class II, DP beta 1	6p21.3							-0.592	2.8475	8	1
HLA-DQA2	major histocompatibility complex, class II, DQ alpha 2	6p21.3				0.352	0.04927	0.1475				1
HCAR1	hydroxycarboxylic acid receptor 1	12q24.31	0.769	0.03884	0.1428							1
LSAMP	limbic system-associated membrane protein	3q13.2-q21	0.769	0.01125	0.0634	0.32	0.012266667	0.0634				2
SLC30A7	solute carrier family 30 (zinc transporter), member 7	1p21.2	0.769	1.00E-04	0.0022							1
DYSF	dysferlin, limb girdle muscular dystrophy 2B (autosomal recessive)	2p13.3	0.768	0.00025	0.0043							1
PTPLAD1	protein tyrosine phosphatase-like A domain containing 1	15q22.2	0.768	0.00806	0.0504							1
AKAP1	A kinase (PRKA) anchor protein 1	17q22	0.766	0.00012	0.0025							1

HLCS	holocarboxylase synthetase (biotin-(proprionyl-CoA-carboxylase (ATP-hydroly	21q22.13				-0.381416667	0.001560278	0.017647222				1
HLF	hepatic leukemia factor	17q22	-2.548	0	0							1
PAM	peptidylglycine alpha-amidating monooxygenase	5q14-q21	0.766	0.0253	0.108							1
PLEK	pleckstrin	2p13.3	0.766	0.0093	0.0556							1
HMCN1	hemicentin 1	1q25.3-q31.1				0.347842105	0.003215789	0.027331579				1
RAB7L1	RAB7, member RAS oncogene family-like 1	1q32	0.766	0.00585	0.0406							1
HMG20B	high mobility group 20B	19p13.3				-0.386	0.00324	0.0312				1
HMGA1	high mobility group AT-hook 1	6p21							0.575	2.58833	6	1
ACSL4	acyl-CoA synthetase long-chain family member 4	Xq22.3-q23	0.765	0.00996	0.0583							1
CDR2L	cerebellar degeneration-related protein 2-like	17q25.1	0.765	0.00409	0.0318							1
DEPDC1B	DEP domain containing 1B	5q12.1	0.765	0.04392	0.1544							1
HMGB4	high mobility group box 4	1p35.1							-1.008	3.1900625	6.75	1
HMGCL	3-hydroxymethyl-3-methylglutaryl-CoA lyase	1p36.1-p35				-0.358	0.006375	0.04095				1
HMGCR	3-hydroxy-3-methylglutaryl-CoA reductase	5q13.3-q14	-0.755	0.00936	0.0559							1
DNAJC22	DnaJ (Hsp40) homolog, subfamily C, member 22	12q13.12	0.765	3.00E-05	0.001							1
HMGN3	high mobility group nucleosomal binding domain 3	6q14.1							-0.793	2.93	7	1
MMP16	matrix metallopeptidase 16 (membrane-inserted)	8q21.3	0.765	0.00332	0.0275	0.3418	0.000518	0.00978				2
HMGN5	high mobility group nucleosome binding domain 5	Xq13.3	-0.474	0.01306	0.0699							1
MRPS35	mitochondrial ribosomal protein S35	12p11	0.765	0.0074	0.0477				-0.796	3.015	6	2
HMGXB4	HMG box domain containing 4	22q13.1				-0.374333333	0.000156667	0.007683333				1
HMOX1	heme oxygenase (decycling) 1	22q13.1	-0.616	0.00822	0.0512	-0.488	0.00234	0.0197				2
HMOX2	heme oxygenase (decycling) 2	16p13.3	-0.962	1.00E-05	5.00E-04	-0.407285714	0.010528571	0.053971429				2
HMP19	HMP19 protein	5q35.2							-0.807	2.975	6	1
HMX2	H6 family homeobox 2	10q26.13	-0.224	0.0364	0.137							1
HMX3	In multiple Geneids		-0.233	0.02265	0.1003							1
HN1	hematological and neurological expressed 1	17q25.1				-0.338	0.00372	0.0336				1
HN1L	In multiple Geneids					-0.41875	0.020035	0.0785				1
PIWIL1	piwi-like 1 (Drosophila)	12q24.33	0.765	0.04217	0.1505							1
TAS2R19	In multiple Geneids		0.765	0.00095	0.0113							1
BCAT1	branched chain amino-acid transaminase 1, cytosolic	12p12.1	0.764	0.01078	0.0615	0.4705	0.02896	0.1061				2
IRAK3	interleukin-1 receptor-associated kinase 3	12q14.3	0.764	0.02058	0.0945							1
ADAM12	ADAM metallopeptidase domain 12	10q26.3	0.763	0.00237	0.0216							1
CUTA	cutA divalent cation tolerance homolog (E. coli)	6p21.32	0.763	0.00014	0.0028							1
GPR116	G protein-coupled receptor 116	6p12.3	0.763	0.00066	0.0088	0.327	0.01009	0.0575				2
NPR2	natriuretic peptide receptor B/guanylate cyclase B (atrionatriuretic peptide re	9p21-p12	0.763	2.00E-04	0.0037							1
PLXNC1	plexin C1	12q23.3	0.763	0.01408	0.0733							1
CEP55	centrosomal protein 55kDa	10q23.33	0.762	0.03746	0.1396							1
FGD6	FYVE, RhoGEF and PH domain containing 6	12q22	0.762	0.01181	0.0655							1
HNRNPAB	heterogeneous nuclear ribonucleoprotein A/B	5q35.3				-0.3815	0.00235	0.0221				1
GORAB	golgin, RAB6-interacting	1q24.2	0.762	0.00023	0.0041							1
HNRNPD	heterogeneous nuclear ribonucleoprotein D (AU-rich element RNA binding pro	4q21				-0.366	0.003185	0.028425				1
JRKL	jerky homolog-like (mouse)	11q21	0.762	0.00013	0.0027							1
HNRNPH1	heterogeneous nuclear ribonucleoprotein H1 (H)	5q35.3				-0.418	0.004206667	0.028666667				1
STK4	serine/threonine kinase 4	20q11.2-q13.2	0.762	2.00E-05	6.00E-04							1
ZBTB38	zinc finger and BTB domain containing 38	3q23	0.762	0.00232	0.0206	0.3055	0.00212	0.02455				2
ACHE	acetylcholinesterase	7q22	0.761	0.00727	0.0471							1
CYGB	cytoglobin	17q25	0.761	0.00123	0.0136							1
HNRNPM	heterogeneous nuclear ribonucleoprotein M	19p13.3-p13.2				-0.411692308	0.010692308	0.051876923				1
EDEM2	ER degradation enhancer, mannosidase alpha-like 2	20q11.22	0.761	0.00237	0.0216							1
HENMT1	HEN1 methyltransferase homolog 1 (Arabidopsis)	1p13.3	0.761	2.00E-05	6.00E-04							1
HNRNPUL1	heterogeneous nuclear ribonucleoprotein U-like 1	19q13.2				-0.405	0.00377	0.0338				1
HNRPH2	Data not found								-0.618	2.57	6	1
HNRPH3	Data not found								-0.769	2.461	5	1
HNRPL	Data not found								-0.673	2.564	5	1
HNT	Data not found								-0.847	2.72286	7	1
HOMER1	homer homolog 1 (Drosophila)	5q14.2				-0.33	1.00E-05	0.0028				1
HOMER2	homer homolog 2 (Drosophila)	15q24.3	-0.988	0.00978	0.0576							1
HOMEZ	homeobox and leucine zipper encoding	14q11.2				-0.379	0.00027	0.0098				1
HOOK1	hook homolog 1 (Drosophila)	1p32.1	-0.464	0.04561	0.1583							1
HOOK2	hook homolog 2 (Drosophila)	19p13.2	-0.306	0.04907	0.1652	-0.4705	0.00079	0.0139				2
HOOK3	hook homolog 3 (Drosophila)	8p11.21	-0.512	0.01381	0.0725							1
HOPX	HOP homeobox	4q12	-2.159	0	0	-0.306	0.00421	0.0357				2
HORMAD2	HORMA domain containing 2	22q12.2				-0.388333333	0.00142	0.0188				1
HOXA1	homeobox A1	7p15.3	-0.538	0.0423	0.1509				0.772666667	2.941093333	7	2
HOXA10	homeobox A10	7p15.2							-0.732666667	3.11317	6	1
INPP5D	inositol polyphosphate-5-phosphatase, 145kDa	2q37.1	0.761	0.04072	0.1472							1
BORA	bora, aurora kinase A activator	13q22.1	0.76	0.00664	0.0442							1
HOXA2	homeobox A2	7p15.2							0.86	3.17278	9	1
HOXA3	homeobox A3	7p15.2							-0.704	2.67583	6	1

HOXA4	homeobox A4	7p15.2				0.632	1.00E-05	0.0028	-0.838	3.22788	8	2
HOXA5	homeobox A5	7p15.2							0.822	3.71714	7	1
HOXA6	homeobox A6	7p15.2							-0.699	3.10917	6	1
HOXA7	homeobox A7	7p15.2							0.626	3.01144	9	1
HOXA9	homeobox A9	7p15.2							0.688	3.0226	5	1
HOXA-AS2	HOXA cluster antisense RNA 2 (non-protein coding)	7p15.2	-0.614	0.00248	0.0223							1
HOXB1	homeobox B1	17q21.3				-0.314	0.01977	0.0847				1
ENTPD1	ectonucleoside triphosphate diphosphohydrolase 1	10q24	0.76	0.01209	0.0666				-0.865	2.93429	7	2
CKS1B	CDC28 protein kinase regulatory subunit 1B	1q21.2	0.7595	0.02253	0.08025							1
MTHFD2	methylenetetrahydrofolate dehydrogenase (NADP+ dependent) 2, methenylte	2p13.1	0.7595	0.002085	0.01955							1
RPL13P5	ribosomal protein L13 pseudogene 5	12p13.31	0.759	0.00011	0.0023							1
CCRL2	chemokine (C-C motif) receptor-like 2	3p21	0.758	0.00029	0.0048							1
PHLDB1	pleckstrin homology-like domain, family B, member 1	11q23.3	0.758	3.00E-04	0.0049							1
AKR7L	aldo-keto reductase family 7-like	1p36.13	0.757	0.02336	0.1024							1
CDKN2A	cyclin-dependent kinase inhibitor 2A	9p21	0.757	0.01318	0.0701							1
NUAK1	NUAK family, SNF1-like kinase, 1	12q23.3	0.757	0.01014	0.059							1
STK3	In multiple Geneids		0.757	0.00025	0.0043	0.3464	0.000948	0.01148				2
FADS3	fatty acid desaturase 3	11q12-q13.1	0.756	5.00E-05	0.0013							1
PRR11	proline rich 11	17q22	0.756	0.02711	0.1129							1
HOXD12	homeobox D12	2q31.1	-0.383	0.00371	0.0297				0.839	2.93667	6	2
HOXD13	homeobox D13	2q31.1							0.876	3.17667	6	1
HOXD3	homeobox D3	2q31.1							0.882	3.563435	8	1
HOXD4	homeobox D4	2q31.1							0.497	2.46643	7	1
HOXD8	homeobox D8	2q31.1							0.531	2.489	5	1
HP	haptoglobin	16q22.2							-0.64	2.43833	6	1
UVRAG	UV radiation resistance associated gene	11q13.5	0.756	4.00E-05	0.0012							1
HPCAL4	hippocalcin like 4	1p34.2	-0.309	0.0025	0.0224							1
HPD	4-hydroxyphenylpyruvate dioxygenase	12q24-qter				-0.391	0.001706667	0.021566667				1
ADAM8	ADAM metallopeptidase domain 8	10q26.3	0.755	0.00339	0.0279							1
HPGD	hydroxyprostaglandin dehydrogenase 15-(NAD)	4q34-q35	-2.281	0.00077	0.0097							1
HPGDS	hematopoietic prostaglandin D synthase	4q22.3				-0.301	0.00598	0.0432				1
JPH1	junctophilin 1	8q21	0.755	0.00145	0.0153	0.329	0.002165	0.02495				2
LRRC8D	leucine rich repeat containing 8 family, member D	1p22.2	0.755	0.00092	0.011	0.8745	5.00E-06	0.00145				2
HPS4	Hermansky-Pudlak syndrome 4	22cen-q12.3				-0.433	0.00025625	0.0077625				1
HPS5	In multiple Geneids					-0.323	0.01072	0.0595				1
HPS6	Hermansky-Pudlak syndrome 6	10q24.32				-0.532	1.00E-05	0.0025				1
HPSE	heparanase	4q21.3							-0.769	2.81333	6	1
HPSE2	heparanase 2	10q23-q24				-0.368	0.00122	0.0194				1
HPX	hemopexin	11p15.5-p15.4				-0.3165	0.00081	0.01605				1
HR	In multiple Geneids		-0.625	0.00663	0.0442	-0.43	3.00E-05	0.0043				2
HRAS	v-Ha-ras Harvey rat sarcoma viral oncogene homolog	11p15.5	-0.521	0.00052	0.0073							1
HRASLS	HRAS-like suppressor	3q29	-0.605	0.00342	0.0281							1
MAP4K3	mitogen-activated protein kinase kinase kinase kinase 3	2p22.1	0.755	0.00025	0.0043	0.315	0.00014	0.0075				2
HRB2	Data not found								-0.842	3.38667	6	1
HRH1	histamine receptor H1	3p25							-0.735	3.396	5	1
HRH2	histamine receptor H2	5q35.2				-0.33725	0.00122	0.0175				1
HRH4	histamine receptor H4	18q11.2							-0.958	2.465	6	1
HRK	harakiri, BCL2 interacting protein (contains only BH3 domain)	12q24.22	-0.26	0.03222	0.1263							1
HRNBP3	Data not found					-0.361	0.005106	0.03764				1
HRNR	hornerin	1q21.3				0.305	0.00398	0.0347				1
RNASET2	ribonuclease T2	6q27	0.755	0.00315	0.0265							1
HS3ST3A1	heparan sulfate (glucosamine) 3-O-sulfotransferase 3A1	17p12	-0.762	6.00E-05	0.0016							1
HS3ST3B1	heparan sulfate (glucosamine) 3-O-sulfotransferase 3B1	17p12	-0.675	0.00021	0.0038	-0.341333333	0.00155	0.020033333	0.693	2.615	6	3
HS3ST6	heparan sulfate (glucosamine) 3-O-sulfotransferase 6	16p13.3	-0.977	1.00E-05	5.00E-04	-0.498	0.00985	0.0568				2
HS6ST1	heparan sulfate 6-O-sulfotransferase 1	2q21	-0.6305	0.00437	0.0333							1
HS6ST2	heparan sulfate 6-O-sulfotransferase 2	Xq26.2				0.334	0.00467	0.0351	-0.48	2.314	5	2
HS6ST3	heparan sulfate 6-O-sulfotransferase 3	13q32.1				0.332818182	0.010175909	0.054772727				1
HSA277841	Data not found								-0.858	2.884	5	1
HSBP1	heat shock factor binding protein 1	16q23.3	-0.416	0.00351	0.0285							1
HSCB	HscB iron-sulfur cluster co-chaperone homolog (E. coli)	22q12.1				-0.422	0.000245	0.0078				1
HSD11B1	hydroxysteroid (11-beta) dehydrogenase 1	1q32-q41				0.414	4.00E-05	0.0047	-0.941	2.79571	7	2
HSD11B1L	hydroxysteroid (11-beta) dehydrogenase 1-like	19p13.3				-0.309	0.00147	0.0211				1
HSD17B1	hydroxysteroid (17-beta) dehydrogenase 1	17q11-q21	-0.345	0.00179	0.0177							1
CXCR4	chemokine (C-X-C motif) receptor 4	2q21	0.754	0.03384	0.1302							1
ERAP1	endoplasmic reticulum aminopeptidase 1	5q15	0.754	0.00651	0.0436							1
GJB1	gap junction protein, beta 1, 32kDa	Xq13.1	0.754	0.00122	0.0135							1
KIF23	kinesin family member 23	15q23	0.754	0.03262	0.1273							1
SHH	sonic hedgehog	7q36	0.754	0.00065	0.0087							1
HSD17B4	hydroxysteroid (17-beta) dehydrogenase 4	5q21	-0.626	0.00015	0.003	-0.3	0.00231	0.0264				2

COTL1	coactosin-like 1 (Dictyostelium)	16q24.1	0.753	0.01488	0.0762							1
SYNJ2	synaptojanin 2	6q25.3	0.753	0.00017	0.0033							1
HSD17B8	hydroxysteroid (17-beta) dehydrogenase 8	6p21.3	-0.611	0.00287	0.0248							1
TDP2	tyrosyl-DNA phosphodiesterase 2	6p22.3-p22.1	0.753	0.00398	0.0312							1
GGT3P	gamma-glutamyltransferase 3 pseudogene	22q11.21	0.752	0.01395	0.0729							1
HSDL1	hydroxysteroid dehydrogenase like 1	16q23.3				-0.382666667	0.00906	0.0438				1
HSF2	heat shock transcription factor 2	6q22.31							-0.59	2.71	6	1
HSF2BP	heat shock transcription factor 2 binding protein	21q22.3				1.17	0.006265	0.04395				1
HSFX1	In multiple Geneids					0.3445	0.001065	0.0153				1
HSFY1	heat shock transcription factor, Y-linked 1	Yq11.222							-1.0172	3.083048	6.6	1
HSFY2	heat shock transcription factor, Y linked 2	Yq11.222							-0.974	2.73833	6	1
HSH2D	hematopoietic SH2 domain containing	19p13.12				-0.3894	0.00415	0.0346				1
HSD3B1	hydroxy-delta-5-steroid dehydrogenase, 3 beta- and steroid delta-isomerase 1	1p13.1	0.752	0.02527	0.1079							1
FERMT1	fermitin family member 1	20p12.3	0.751	0.02029	0.0936	0.339	0.00103	0.0179				2
HLA-J	major histocompatibility complex, class I, J (pseudogene)	6p21.31	0.751	0.00442	0.02835							1
KIAA1524	KIAA1524	3q13.13	0.751	0.03303	0.1283				-0.782	2.412	5	2
MAD2L1BP	MAD2L1 binding protein	6p21.1	0.751	0.00374	0.0299							1
SLC25A32	solute carrier family 25 (mitochondrial folate carrier) , member 32	8q22.3	0.751	7.00E-05	0.0016				-1.105	3.485	6	2
AARS	alanyl-tRNA synthetase	16q22	0.75	0.00055	0.0077							1
HSP90AB5P	heat shock protein 90kDa alpha (cytosolic), class B member 5, pseudogene	3p13	-0.309	0.00985	0.0578							1
C5orf28	chromosome 5 open reading frame 28	5p12	0.748	0.00019	0.0035	0.34	0.02629	0.1003				2
CLCN4	chloride channel, voltage-sensitive 4	Xp22.3	0.748	0.00032	0.0052							1
KIAA0040	KIAA0040	1q24-q25	0.748	0.00608	0.0417	0.407	0.00095	0.0172				2
HSPA12A	heat shock 70kDa protein 12A	10q26.12				-0.304	0.00047	0.0125				1
MICB	MHC class I polypeptide-related sequence B	6p21.3	0.748	4.00E-04	0.0061							1
HSPA14	heat shock 70kDa protein 14	10p13				-0.313	0.0064	0.0448				1
NR1I2	nuclear receptor subfamily 1, group I, member 2	3q12-q13.3	0.748	0.00066	0.0088	1.227	0	9.00E-04	-0.832	2.89708	7	3
HSPA1L	heat shock 70kDa protein 1-like	6p21.3							0.817	2.848	5	1
HSPA2	heat shock 70kDa protein 2	14q24.1	-1.605	3.00E-05	8.00E-04							1
HSPA4	heat shock 70kDa protein 4	5q31.1	-0.533	0.00106	0.0122	-0.332666667	0.00046	0.011166667				2
HSPA4L	heat shock 70kDa protein 4-like	4q28	-1.901	0.00033	0.0053	-0.327	0.01027	0.0581				2
SNORD15B	small nucleolar RNA, C/D box 15B	11q13.4	0.748	0.00549	0.0389							1
CEP170P1	centrosomal protein 170kDa pseudogene 1	4q26	0.747	0.00088	0.0107							1
HSPA9	heat shock 70kDa protein 9 (mortalin)	5q31.1				-0.38	0.003342	0.02872				1
HSPB1	heat shock 27kDa protein 1	7q11.23	-0.948	0.00556	0.0392							1
HSPB7	heat shock 27kDa protein family, member 7 (cardiovascular)	1p36.23-p34.3				-0.321	0.01899	0.0827				1
HSPB8	heat shock 22kDa protein 8	12q24.23	-2.805	1.00E-05	3.00E-04							1
HSPB9	heat shock protein, alpha-crystallin-related, B9	17q21.2	-0.297	0.01808	0.0867							1
JTB	jumping translocation breakpoint	1q21	0.747	0.00026	0.0044							1
RNF186	ring finger protein 186	1p36.13	0.746	0.00524	0.0377							1
HSPC072	uncharacterized LOC29075	20p11.23				0.32	0.00146	0.021				1
SOD2	superoxide dismutase 2, mitochondrial	6q25.3	0.746	0.02665	0.1118							1
TMEM55A	transmembrane protein 55A	8q21.3	0.746	0.01053	0.0607	0.3588	0.002896	0.01996				2
FAM91A1	family with sequence similarity 91, member A1	8q24.13	0.745	0.00148	0.0154	0.314	0.015045	0.06975				2
MB21D2	Mab-21 domain containing 2	3q29	0.745	0.00137	0.0146							1
HTA	uncharacterized LOC283902	16q22.3				-0.347	0.00108	0.0183				1
NKAIN2	Na+/K+ transporting ATPase interacting 2	6q21	0.745	0.01743	0.0846	0.3355	0.0098875	0.056775				2
HTATSF1	HIV-1 Tat specific factor 1	Xq26.3				0.316	0.0077	0.0496				1
HTN1	histatin 1	4q13							-0.61	2.73625	8	1
HTN3	histatin 3	4q13							-0.754	2.90857	7	1
RUNX1	runt-related transcription factor 1	21q22.3	0.745	0.00134	0.0144							1
HTR2A	5-hydroxytryptamine (serotonin) receptor 2A, G protein-coupled	13q14-q21				0.327	0.00104	0.0179	-0.906	3.16333	6	2
HTR2B	5-hydroxytryptamine (serotonin) receptor 2B, G protein-coupled	2q36.3-q37.1							-0.714	2.775	6	1
HTR2C	5-hydroxytryptamine (serotonin) receptor 2C, G protein-coupled	Xq24				0.312	0.00602	0.0403				1
HTR3A	5-hydroxytryptamine (serotonin) receptor 3A, ionotropic	11q23.1	-0.383	0.01282	0.0691							1
HTR3B	5-hydroxytryptamine (serotonin) receptor 3B, ionotropic	11q23.1	-0.359	0.00739	0.0477							1
HTR3E	5-hydroxytryptamine (serotonin) receptor 3E, ionotropic	3q27.1	-0.244	0.01826	0.0874							1
HTR4	5-hydroxytryptamine (serotonin) receptor 4, G protein-coupled	5q31-q33	-0.335	0.00536	0.0383							1
HTR5A	5-hydroxytryptamine (serotonin) receptor 5A, G protein-coupled	7q36.1	-0.264	0.03481	0.1328							1
HTR7	5-hydroxytryptamine (serotonin) receptor 7, adenylate cyclase-coupled	10q21-q24	-0.288	0.00659	0.044							1
HTR7P1	5-hydroxytryptamine (serotonin) receptor 7 pseudogene 1	12p13.1	-0.241	0.03879	0.1428							1
SLC38A6	solute carrier family 38, member 6	14q23.1	0.744	0.00349	0.0284							1
SNORD74	small nucleolar RNA, C/D box 74	1q25.1	0.744	0.0302	0.1212							1
ADCK3	aarF domain containing kinase 3	1q42.13	0.742	0.00327	0.0273							1
HTT	huntingtin	4p16.3	-0.62	1.00E-04	0.0023	-0.354583333	0.001563333	0.019158333				2
HUNK	hormonally up-regulated Neu-associated kinase	21q22.1				-0.351125	0.003061875	0.023925				1
CYB5B	cytochrome b5 type B (outer mitochondrial membrane)	16q22.1	0.742	0.00463	0.0346							1
HUWE1	HECT, UBA and WWE domain containing 1, E3 ubiquitin protein ligase	Xp11.22							-0.887	3.5175	8	1
HVCN1	hydrogen voltage-gated channel 1	12q24.11				-0.365	0.001323333	0.016366667				1

HYAL1	hyaluronoglucosaminidase 1	3p21.3-p21.2				-0.429	0.00023	0.0093				1
HYAL2	hyaluronoglucosaminidase 2	3p21.3				-0.456	2.00E-05	0.0037				1
HYALP1	hyaluronoglucosaminidase pseudogene 1	7q31.3				0.31	0.04086	0.1313				1
HYDIN	HYDIN, axonemal central pair apparatus protein	16q22.2				0.306	0.00422	0.0358				1
HYI	hydroxypyruvate isomerase (putative)	1p34.2							-0.809	3.01429	7	1
INSIG2	insulin induced gene 2	2q14.2	0.742	0.00052	0.0074	0.331	0.00142	0.01645				2
HYPK	Data not found								0.693	2.974	5	1
MRPL33	In multiple Geneids		0.742	0.00047	0.0068							1
IAPP	islet amyloid polypeptide	12p12.1							-0.737	2.58143	7	1
IARS2	isoleucyl-tRNA synthetase 2, mitochondrial	1q41							-0.956	2.551	5	1
IBRDC1	Data not found								-0.854	3.365	6	1
HTATIP2	HIV-1 Tat interactive protein 2, 30kDa	11p15.1	0.741	0.00016	0.0031							1
IBTK	inhibitor of Bruton agammaglobulinemia tyrosine kinase	6q14.1				-0.339	0.00207	0.025				1
LASP1	LIM and SH3 protein 1	17q11-q21.3	0.741	7.00E-05	0.0018							1
RALGAPA2	Ral GTPase activating protein, alpha subunit 2 (catalytic)	20p11.22	0.741	0.01346	0.0712							1
BRCA2	breast cancer 2, early onset	13q12.3	0.74	0.01976	0.092							1
ICAM5	intercellular adhesion molecule 5, telencephalin	19p13.2				-0.736	0.00038	0.0114				1
ICK	In multiple Geneids					0.308	0.00094	0.0171				1
ICMT	isoprenylcysteine carboxyl methyltransferase	1p36.21				-0.415	0.00039	0.0107				1
ICOS	inducible T-cell co-stimulator	2q33							-0.836	2.96	6	1
ICOSLG	inducible T-cell co-stimulator ligand	21q22.3				-0.424	0.001525	0.01765				1
ID1	inhibitor of DNA binding 1, dominant negative helix-loop-helix protein	20q11	-0.875	0.02057	0.0945							1
CABYR	In multiple Geneids		0.74	0.00971	0.0573							1
DARS2	aspartyl-tRNA synthetase 2, mitochondrial	1q25.1	0.74	0.00061	0.0083							1
ID3	inhibitor of DNA binding 3, dominant negative helix-loop-helix protein	1p36.13-p36.12							-0.683	2.60667	6	1
ID4	inhibitor of DNA binding 4, dominant negative helix-loop-helix protein	6p22.3	-0.795	4.00E-05	0.0012							1
IDE	insulin-degrading enzyme	10q23-q25				-0.335	0.00054	0.0114875				1
IDO2	indoleamine 2,3-dioxygenase 2	8p11.21				-0.314	0.01325	0.0671				1
IDS	iduronate 2-sulfatase	Xq28				0.31	0.01349	0.0678				1
IER2	immediate early response 2	19p13.2				-0.4395	0.00148	0.0208				1
IER3IP1	immediate early response 3 interacting protein 1	18q12				-0.398	0.00145	0.021				1
IER5	In multiple Geneids		-0.304	0.03541	0.1344							1
IFFO2	intermediate filament family orphan 2	1p36.13	-1.54	0.00011	0.0024	-0.335	0.00061	0.0141				2
IFI16	interferon, gamma-inducible protein 16	1q22							-0.797	2.394	5	1
IFI27	interferon, alpha-inducible protein 27	14q32				-0.318	0.00568	0.0419				1
ERRFI1	ERBB receptor feedback inhibitor 1	1p36	0.74	0.0199	0.0924							1
SLC52A2	solute carrier family 52, riboflavin transporter, member 2	8q24.3	0.74	0.00363	0.0293							1
IFI44	interferon-induced protein 44	1p31.1							-0.553	2.53286	7	1
IFI44L	interferon-induced protein 44-like	1p31.1							-0.927	3.09375	8	1
TJAP1	tight junction associated protein 1 (peripheral)	6p21.1	0.74	0.00141	0.0149							1
IFIH1	interferon induced with helicase C domain 1	2q24				0.3568	0.00067	0.0118				1
ZNF814	In multiple Geneids		0.74	0.02423	0.1048							1
ZNF841	zinc finger protein 841	19q13.41	0.74	0.00459	0.0345							1
IFIT3	In multiple Geneids								-0.778	2.558	5	1
PCDHB16	protocadherin beta 16	5q31	0.739	0.03151	0.1246				-0.939	3.02417	6	2
FSD1L	fibronectin type III and SPRY domain containing 1-like	9q31	0.738	0.00058	0.008							1
GPR183	G protein-coupled receptor 183	13q32.3	0.738	0.02499	0.1071							1
INPP4A	inositol polyphosphate-4-phosphatase, type I, 107kDa	2q11.2	0.738	0.00032	0.0051	0.304	0.03146	0.1118				2
IFLTD1	intermediate filament tail domain containing 1	12p12.1				0.612666667	0.02701	0.100766667				1
IFNA10	interferon, alpha 10	9p22							-0.908	3.472	10	1
IFNA13	interferon, alpha 13	9p22							-0.933	3.4175	8	1
IFNA14	interferon, alpha 14	9p22							-0.896	2.98125	8	1
IFNA16	interferon, alpha 16	9p22							-0.879	3.05125	8	1
IFNA17	interferon, alpha 17	9p22							-1.074	3.36375	8	1
IFNA2	interferon, alpha 2	9p22							-0.928	3.09	8	1
IFNA21	interferon, alpha 21	9p22							-0.921	2.89375	8	1
IFNA4	interferon, alpha 4	9p22							-0.949	3.32125	8	1
IFNA5	interferon, alpha 5	9p22							-0.882	2.9975	8	1
IFNA6	interferon, alpha 6	9p22							-0.717	2.51286	7	1
IFNA7	interferon, alpha 7	9p22							-1.014	3.835	8	1
IFNA8	interferon, alpha 8	9p22							-1.077	3.08286	7	1
IFNAR1	interferon (alpha, beta and omega) receptor 1	21q22.11				-0.302	0.00256	0.0278				1
RNF219	ring finger protein 219	13q31.1	0.737	0.00011	0.0023							1
IFNB1	interferon, beta 1, fibroblast	9p21							-0.919	3.11444	9	1
IFNE1	Data not found								-1.1	3.465	8	1
EXT1	exostosin 1	8q24.11	0.736	0.00559	0.0394	0.336074074	0.015840741	0.070581481				2
IFNGR2	interferon gamma receptor 2 (interferon gamma transducer 1)	21q22.11				-0.4225	0.000786667	0.014416667				1
IFNL1	Data not found		-0.299	0.00284	0.0246							1
IFNW1	interferon, omega 1	9p22							-0.911	2.95167	6	1

SH2B3	SH2B adaptor protein 3	12q24	0.736	1.00E-05	4.00E-04							1
SUMF2	sulfatase modifying factor 2	7q11.1	0.736	0.00038	0.0059							1
IFT172	intraflagellar transport 172 homolog (Chlamydomonas)	2p23.3				-0.307	0.01259	0.0652				1
CMTM1	CKLF-like MARVEL transmembrane domain containing 1	16q21	0.735	0.00648	0.0435							1
CSPG4	chondroitin sulfate proteoglycan 4	15q24.2	0.735	0.00154	0.0159							1
IFT74	intraflagellar transport 74 homolog (Chlamydomonas)	9p21.2				-0.362166667	0.003625	0.027633333				1
IFT80	intraflagellar transport 80 homolog (Chlamydomonas)	3q25.33							-0.798	2.814	5	1
DTX3L	deltex 3-like (Drosophila)	3q21.1	0.735	0.00029	0.0047							1
IWS1	IWS1 homolog (S. cerevisiae)	2q14.3	0.735	0	2.00E-04							1
IGDCC3	immunoglobulin superfamily, DCC subclass, member 3	15q22.3-q23				-0.448	0.00037	0.0113				1
MICU1	mitochondrial calcium uptake 1	10q22.1	0.735	4.00E-04	0.0061							1
GPR125	G protein-coupled receptor 125	4p15.2	0.7345	0.016445	0.08085							1
IGF2AS	Data not found								0.461	2.44457	7	1
WBSCR22	Williams Beuren syndrome chromosome region 22		0.734	2.00E-04	0.0037							1
BMF	Bcl2 modifying factor	15q14	0.733	0.00345	0.0282							1
GUCY1B2	guanylate cyclase 1, soluble, beta 2 (pseudogene)	13q14.3	0.733	0.00365	0.0293	0.437	0.005605	0.0332				2
IGFBP1	insulin-like growth factor binding protein 1	7p13-p12				0.409	0.00887	0.0536				1
IGFBP2	insulin-like growth factor binding protein 2, 36kDa	2q33-q34	-0.82	0.04155	0.1492							1
TAS2R20	In multiple Geneids		0.733	0.00748	0.0481							1
APBB2	amyloid beta (A4) precursor protein-binding, family B, member 2	4p13	0.732	0.00751	0.0482							1
IGFBPL1	insulin-like growth factor binding protein-like 1	9p13.1				-0.486666667	0.000256667	0.0086				1
IGFL1	IGF-like family member 1	19q13.32	-2.584	1.00E-05	4.00E-04							1
IGFL3	IGF-like family member 3	19q13.32							-1.27	3.83	5	1
IGJ	immunoglobulin J polypeptide, linker protein for immunoglobulin alpha and m	4q21							-0.883	3.44111	9	1
E2F6	E2F transcription factor 6	2p25.1	0.732	9.00E-05	0.002							1
IGSF10	immunoglobulin superfamily, member 10	3q25.1				0.302	0.00015	0.0078				1
IGSF11	immunoglobulin superfamily, member 11	3q13.32	-0.524	0.0014	0.0149							1
IGSF2	Data not found								-0.838	2.84375	8	1
IGSF21	immunoglobin superfamily, member 21	1p36.13				-0.3165	0.00724	0.04705				1
IGSF22	immunoglobulin superfamily, member 22	11p15.1				-0.301	0.00857	0.0526				1
IGSF3	immunoglobulin superfamily, member 3	1p13	-1.372	1.00E-05	4.00E-04	-0.396	0.00082	0.0161				2
IGSF4B	Data not found								0.719	2.89833	6	1
IGSF4C	Data not found								0.61	2.448	5	1
IGSF4D	Data not found								-0.816	3.01286	7	1
IGSF5	In multiple Geneids					-0.306	0.00036	0.0112				1
SLCO1B7	solute carrier organic anion transporter family, member 1B7 (non-functional)	12p12.2	0.732	0.03224	0.1263							1
C20orf96	chromosome 20 open reading frame 96	20p13	0.731	0.00025	0.0043				-0.861	2.702	5	2
IK	IK cytokine, down-regulator of HLA II	5q31.3	-0.341	0.00054	0.0076	-0.401	2.00E-04	0.0076				2
SNORA71A	small nucleolar RNA, H/ACA box 71A	20q11.23	0.731	0.03023	0.1213							1
IKBKB	inhibitor of kappa light polypeptide gene enhancer in B-cells, kinase beta	8p11.2				-0.343666667	0.009786667	0.055366667				1
IKBKE	inhibitor of kappa light polypeptide gene enhancer in B-cells, kinase epsilon	1q32.1	-0.298	0.033	0.125							1
IKZF1	IKAROS family zinc finger 1 (Ikaros)	7p13-p11.1				0.386736842	0.012846316	0.062484211				1
IKZF2	IKAROS family zinc finger 2 (Helios)	2q34				0.342111111	0.000913889	0.014444444				1
IKZF4	IKAROS family zinc finger 4 (Eos)	12q13				-0.375	0.04062	0.1308				1
IL10RB	interleukin 10 receptor, beta	21q22.11				-0.3625	0.00014	0.006				1
IL12A	interleukin 12A (natural killer cell stimulatory factor 1, cytotoxic lymphocyte m	3q25.33-q26	-1.383	0.00014	0.0028							1
IL12RB1	interleukin 12 receptor, beta 1	19p13.1				-0.51375	0.0003675	0.009525				1
IL12RB2	interleukin 12 receptor, beta 2	1p31.3-p31.2	-0.56	0.00095	0.0113	-0.337	0.00329	0.0311				2
IL13	interleukin 13	5q31	-0.202	0.04313	0.1527	-0.328	9.00E-05	0.0062				2
IL15	interleukin 15	4q31							-0.486	2.42	6	1
IL16	interleukin 16	15q26.3							-0.75475	2.908	5	1
IL17B	interleukin 17B	5q33.1	-0.212	0.03078	0.1228							1
IL17D	interleukin 17D	13q11							-0.896	2.956	5	1
IL17RA	interleukin 17 receptor A	22q11.1				-0.423875	0.00037	0.0082875				1
ARFGAP3	ADP-ribosylation factor GTPase activating protein 3	22q13.2	0.73	0.00167	0.0168							1
IL17RC	interleukin 17 receptor C	3p25.3	-0.584	1.00E-05	3.00E-04	-0.417	0.00127	0.0193				2
IL17RD	interleukin 17 receptor D	3p14.3				-0.410166667	0.000246667	0.008183333				1
IL17REL	interleukin 17 receptor E-like	22q13.33				-0.41	0.00137	0.0204				1
IL18	interleukin 18 (interferon-gamma-inducing factor)	11q22.2-q22.3	-2.399	0.00036	0.0056							1
STK31	serine/threonine kinase 31	7p15.3	0.73	0.00601	0.0414							1
CCDC102B	coiled-coil domain containing 102B	18q22.1	0.729	0.00114	0.0128							1
IL18RAP	interleukin 18 receptor accessory protein	2q12				0.408	8.00E-05	0.006	-0.839	3.09625	8	2
IL19	interleukin 19	1q32.2				0.343	1.00E-04	0.0066				1
IL1A	interleukin 1, alpha	2q14	-3.234	2.00E-05	7.00E-04							1
CTHRC1	collagen triple helix repeat containing 1	8q22.3	0.729	0.00035	0.0055							1
IL1F10	interleukin 1 family, member 10 (theta)	2q13							-0.987	3.3475	5.333333333	1
IL1F5	Data not found								-0.817	2.747775	9	1
IL1F8	Data not found								-0.726	2.706	5	1
IL1R1	interleukin 1 receptor, type I	2q12				0.304	0.00125	0.0195				1

IL1R2	In multiple Geneids		-0.962	0.04593	0.1588							1
IL1RAP	interleukin 1 receptor accessory protein	3q28				0.356916667	0.005395833	0.035925				1
IL1RAPL1	interleukin 1 receptor accessory protein-like 1	Xp22.1-p21.3				0.369	0.002601667	0.025325				1
IL1RAPL2	In multiple Geneids					0.338473684	0.001489474	0.019110526				1
IL1RL1	In multiple Geneids					0.3835	0.000115	0.007	-0.752	2.678	5	2
IL1RL2	interleukin 1 receptor-like 2	2q12				0.342	0.00085	0.0164	-0.813	2.998	5	2
IL1RN	interleukin 1 receptor antagonist	2q14.2	-3.165	5.00E-05	0.0013							1
IL2	interleukin 2	4q26-q27							-0.851	2.8	7	1
IL20RA	interleukin 20 receptor, alpha	6q23.3	-0.478	0.03287	0.1279							1
IL20RB	interleukin 20 receptor beta	3q22.3	-3.467	0	1.00E-04							1
IL21	interleukin 21	4q26-q27							-0.961	3.34571	7	1
IL22	interleukin 22	12q15	-0.439	0.0084	0.0518							1
IL22RA1	interleukin 22 receptor, alpha 1	1p36.11				-0.456666667	0.00085	0.0119				1
IL22RA2	interleukin 22 receptor, alpha 2	6q25.1	-0.238	0.04548	0.1581							1
IL23A	interleukin 23, alpha subunit p19	12q13.3	-0.343	0.03371	0.1299							1
FKBP7	In multiple Geneids		0.729	0.00064	0.0085							1
IL25	interleukin 25	14q11.2	-0.238	0.02227	0.0995							1
IL26	interleukin 26	12q15							-0.949	3.37444	9	1
IL27	In multiple Geneids					-0.383	0.00024	0.0094				1
IL27RA	interleukin 27 receptor, alpha	19p13.11				-0.4945	0.005485	0.03875				1
IL28RA	interleukin 28 receptor, alpha (interferon, lambda receptor)	1p36.11				-0.363	0.00017	0.008				1
IL29	interleukin 29 (interferon, lambda 1)	19q13.13				-0.389	0.00011	0.0068				1
PDS5B	PDS5, regulator of cohesion maintenance, homolog B (S. cerevisiae)	13q12.3	0.729	9.00E-05	0.0021							1
IL2RB	interleukin 2 receptor, beta	22q13.1				-0.5355	0.00019	0.007				1
TAS2R46	taste receptor, type 2, member 46	12p13.2	0.729	0.00132	0.0143							1
IL3	interleukin 3 (colony-stimulating factor, multiple)	5q31.1				-0.359	4.00E-05	0.0047				1
IL31	interleukin 31	12q24.31	-0.263	0.02763	0.1141				0.68	2.856	5	2
IL31RA	In multiple Geneids								-0.774	3.02	7	1
REEP3	receptor accessory protein 3	10q21.3	0.7285	0.01264	0.05475							1
IL34	interleukin 34	16q22.1	-1.488	5.00E-05	0.0014	-0.356	0.00093	0.017				2
IL36A	interleukin 36, alpha	2q12-q14.1	-4.825	0	0							1
IL36B	interleukin 36, beta	2q14	-1.339	0	1.00E-04							1
IL36RN	interleukin 36 receptor antagonist	2q14	-0.925	6.00E-05	0.0015							1
CHEK1	checkpoint kinase 1	11q24.2	0.728	0.01766	0.0853							1
IL4	interleukin 4	5q31.1	-0.219	0.00662	0.0442							1
IL4I1	interleukin 4 induced 1	19q13.3-q13.4				-0.3195	0.013655	0.0641				1
IL5	interleukin 5 (colony-stimulating factor, eosinophil)	5q31.1							-0.968	3.18857	7	1
IL5RA	interleukin 5 receptor, alpha	3p26-p24							-0.688	2.71	5	1
HOXC11	homeobox C11	12q13.3	0.728	0.00271	0.0237							1
IL6ST	interleukin 6 signal transducer (gp130, oncostatin M receptor)	5q11.2				-0.3065	0.003455	0.03005				1
IL7	interleukin 7	8q12-q13				0.3525	0.0003475	0.009625	-0.832	3.04286	7	2
IL7R	interleukin 7 receptor	5p13				0.370333333	0.007113333	0.046266667				1
SPATS2	spermatogenesis associated, serine-rich 2	12q13.12	0.728	0.00055	0.0077							1
IL9	interleukin 9	5q31.1	-0.176	0.03498	0.1333							1
IL9R	interleukin 9 receptor	Xq28 and Yq12	-0.249	0.01116	0.063							1
ILDR1	immunoglobulin-like domain containing receptor 1	3q13.33				0.381	0.00124	0.0195				1
ILDR2	immunoglobulin-like domain containing receptor 2	1q24.1				0.360666667	0.002176667	0.0208				1
CRYZ	crystallin, zeta (quinone reductase)	1p31-p22	0.727	0.00326	0.0272							1
ILVBL	ilvB (bacterial acetolactate synthase)-like	19p13.1	-0.426	0.00863	0.0528	-0.425	0.0002325	0.008725				2
IMMP2L	IMP2 inner mitochondrial membrane peptidase-like (S. cerevisiae)	7q31				0.337	0.000746667	0.014666667				1
IMP3	IMP3, U3 small nucleolar ribonucleoprotein, homolog (yeast)	15q24	-0.278	0.01919	0.0904							1
PARD6B	par-6 partitioning defective 6 homolog beta (C. elegans)	20q13.13	0.727	0.00014	0.0029							1
IMPA2	inositol(myo)-1(or 4)-monophosphatase 2	18p11.2	-1.195	7.00E-05	0.0016	-0.445	0.00051	0.013				2
E2F3	E2F transcription factor 3	6p22	0.726	0.00105	0.0121							1
IMPDH1	IMP (inosine 5'-monophosphate) dehydrogenase 1	7q31.3-q32				-0.319	0.0011	0.0184				1
IMPDH2	IMP (inosine 5'-monophosphate) dehydrogenase 2	3p21.2				-0.36	0.00507	0.03645				1
IMPG1	interphotoreceptor matrix proteoglycan 1	6q14.2-q15							-0.982	3.33286	7	1
IMPG2	In multiple Geneids								-0.982	3.75143	7	1
INA	internexin neuronal intermediate filament protein, alpha	10q24.33							-0.443	2.394	5	1
INADL	InaD-like (Drosophila)	1p31.3	-0.396	0.031	0.1234	-0.355933333	0.005292667	0.037453333				2
INCA1	inhibitor of CDK, cyclin A1 interacting protein 1	17p13.2				-0.3585	0.02086	0.08205				1
INCENP	inner centromere protein antigens 135/155kDa	11q12.3				-0.329	0.013295	0.0625				1
INDO	Data not found								-0.789	3.16571	7	1
POU6F2	POU class 6 homeobox 2	7p14.1	0.726	0.01721	0.0839	0.357157895	0.002683684	0.021852632				2
CHPF	chondroitin polymerizing factor	2q35	0.724	0.00019	0.0035							1
ING2	inhibitor of growth family, member 2	4q35.1	-0.669	3.00E-05	9.00E-04	-0.38	0.00723	0.0479				2
ING3	inhibitor of growth family, member 3	7q31							-1.064	2.97333	6	1
ING5	inhibitor of growth family, member 5	2q37.3	-0.454	0.00971	0.0573	-0.307	0.00818	0.0513				2
RASGRF2	Ras protein-specific guanine nucleotide-releasing factor 2	5q13	0.724	0.00081	0.0101							1

ZNF774	zinc finger protein 774	15q26.1	0.724	0.00186	0.0182							1
INHBC	inhibin, beta C	12q13.1				-0.33	0.01277	0.0657				1
INO80	INO80 homolog (S. cerevisiae)	15q15.1				-0.361125	0.00642375	0.0392375				1
INO80C	INO80 complex subunit C	18q12.2	-0.828	0.00035	0.0055							1
INO80E	INO80 complex subunit E	16p11.2				-0.3765	0.00341	0.02935				1
INPP1	inositol polyphosphate-1-phosphatase	2q32	-0.781	0.01581	0.0793							1
EDEM3	ER degradation enhancer, mannosidase alpha-like 3	1q25	0.723	0.00249	0.0223	0.302	0.00087	0.0166				2
INPP4B	inositol polyphosphate-4-phosphatase, type II, 105kDa	4q31.21							-0.691	2.6	7	1
INPP5A	inositol polyphosphate-5-phosphatase, 40kDa	10q26.3				-0.347142857	0.001618571	0.018142857				1
POFUT2	protein O-fucosyltransferase 2	21q22.3	0.723	1.00E-05	4.00E-04							1
PRPS1	phosphoribosyl pyrophosphate synthetase 1	Xq21.32-q24	0.723	0.00083	0.0103							1
INPP5F	inositol polyphosphate-5-phosphatase F	10q26.11				-0.4565	0.002165	0.02535				1
INPP5J	inositol polyphosphate-5-phosphatase J	22q12.2				-0.515	5.00E-06	0.00205				1
INPP5K	inositol polyphosphate-5-phosphatase K	17p13.3	-0.252	0.02194	0.0987	-0.397333333	0.0278	0.102066667				2
RFTN1	raftlin, lipid raft linker 1	3p24.3	0.723	0.00759	0.0485							1
AGPAT1	1-acylglycerol-3-phosphate O-acyltransferase 1 (lysophosphatidic acid acyltrar	6p21.3	0.722	0.01786	0.086							1
ALG5	asparagine-linked glycosylation 5, dolichyl-phosphate beta-glucosyltransferas	13q13.3	0.722	0.00169	0.0169							1
INSL5	insulin-like 5	1p31.3							-0.912	3.07875	8	1
INSL6	insulin-like 6	9p24				-0.3665	0.003905	0.0341				1
INSM1	insulinoma-associated 1	20p11.2				0.351	0.00854	0.0525				1
PTPRU	protein tyrosine phosphatase, receptor type, U	1p35.3	0.722	0.01318	0.0701							1
INTS1	integrator complex subunit 1	7p22.3				-0.412	0.0012	0.0192				1
INTS12	integrator complex subunit 12	4q24							-0.706	2.69333	9	1
ARHGAP44	Rho GTPase activating protein 44	17p12	0.721	0.00727	0.0471							1
C19orf10	chromosome 19 open reading frame 10	19p13.3	0.721	3.00E-05	9.00E-04							1
INTS6	integrator complex subunit 6	13q14.3				0.3315	0.006585	0.0448				1
CDCA5	In multiple Geneids		0.721	0.00299	0.0255							1
INTS9	integrator complex subunit 9	8p21.1				-0.331	0.00204	0.0247				1
INTU	inturned planar cell polarity effector homolog (Drosophila)	4q28.1	-0.346	0.02803	0.1153							1
IP6K1	inositol hexakisphosphate kinase 1	3p21.31				-0.387333333	0.000731667	0.014166667				1
IP6K2	inositol hexakisphosphate kinase 2	3p21.31				-0.4162	0.000216	0.0086				1
IPCEF1	interaction protein for cytohesin exchange factors 1	6q25.2	-0.519	0.00636	0.043	-0.342	0.0026	0.028				2
IPO11	importin 11	5q12.1	-0.354	0.03939	0.1441	-0.3466	0.00504	0.03374				2
IPO13	importin 13	1p34.1	-0.448	0.00147	0.0153							1
IPO4	importin 4	14q12				-0.336	0.00114	0.0188				1
IPO7	importin 7	11p15.4				-0.318	0.00196	0.0243				1
HMGN4	high mobility group nucleosomal binding domain 4	6p21.3	0.721	9.00E-05	0.0021							1
IPPK	inositol 1,3,4,5,6-pentakisphosphate 2-kinase	9q22.31	-1.135	3.00E-05	9.00E-04	-0.3445	0.00498	0.0385				2
IQCA1	IQ motif containing with AAA domain 1	2q37.3	-0.482	0.00101	0.0118							1
KIAA1377	KIAA1377	11q22.1	0.721	0.01079	0.0615							1
IQCF6	IQ motif containing F6	3p21.2				-0.554	6.00E-05	0.0056				1
IQCJ	IQ motif containing J	3q25.32				0.3634	0.002964	0.02521				1
POLR2H	polymerase (RNA) II (DNA directed) polypeptide H	3q28	0.721	0.00268	0.0236							1
ANP32E	acidic (leucine-rich) nuclear phosphoprotein 32 family, member E	1q21.2	0.72	0.00089	0.0108							1
PRPF3	PRP3 pre-mRNA processing factor 3 homolog (S. cerevisiae)	1q21.1	0.72	0.00066	0.0088							1
RNF114	ring finger protein 114	20q13.13	0.72	9.00E-05	0.002							1
IQSEC2	IQ motif and Sec7 domain 2	Xp11.22							-0.865	2.65667	6	1
IQSEC3	In multiple Geneids					0.3534	0.001632	0.02044				1
HOXB13	homeobox B13	17q21.2	0.719	0.00101	0.0118							1
NUP62CL	nucleoporin 62kDa C-terminal like	Xq22.3	0.719	0.00117	0.0131							1
SPRY2	sprouty homolog 2 (Drosophila)	13q31.1	0.719	0.01696	0.0831							1
IRAK4	interleukin-1 receptor-associated kinase 4	12q12	-0.818	2.00E-05	8.00E-04							1
TAS2R14	taste receptor, type 2, member 14	12p13	0.719	0.00548	0.0389				-0.796	2.7725	8	2
IRF2	interferon regulatory factor 2	4q34.1-q35.1				-0.4005	0.00797625	0.0467875				1
FAM47E	family with sequence similarity 47, member E	4q21.1	0.718	0.0155	0.0782							1
IRF2BPL	interferon regulatory factor 2 binding protein-like	14q24.3	-0.224	0.04634	0.1596							1
KLK1	kallikrein 1	19q13.3	0.718	0.04697	0.1609							1
IRF5	interferon regulatory factor 5	7q32	-0.443	0.02347	0.1027							1
IRF6	interferon regulatory factor 6	1q32.3-q41	-1.071	8.00E-05	0.0018							1
LRRC49	leucine rich repeat containing 49	15q23	0.718	0.00727	0.0471							1
PRR16	proline rich 16	5q23.1	0.718	2.00E-05	8.00E-04							1
SNORD117	small nucleolar RNA, C/D box 117	6p21.33	0.718	0.0039	0.0307							1
IRX1	iroquois homeobox 1	5p15.3	-0.268	0.02049	0.0943							1
IRX3	iroquois homeobox 3	16q12.2	-1.406	0	0							1
IRX4	iroquois homeobox 4	5p15.3	-0.977	1.00E-05	3.00E-04				0.796	2.718	5	2
IRX5	iroquois homeobox 5	16q12.2	-0.503	0.00119	0.0133							1
SULT1C4	sulfotransferase family, cytosolic, 1C, member 4	2q12.3	0.718	0.03276	0.1277							1
DNA2	DNA replication helicase 2 homolog (yeast)	10q21.3-q22.1	0.717	0.01597	0.0799							1
ISLR2	immunoglobulin superfamily containing leucine-rich repeat 2	15q24.1							0.682	3.06875	8	1

ISM1	isthmin 1 homolog (zebrafish)	20p12.1				0.3424	0.01084	0.05834				1
ISM2	isthmin 2 homolog (zebrafish)	14q24.3				-0.313	0.004103333	0.035133333				1
ISOC1	isochorismatase domain containing 1	5q22.1-q33.3	-0.33	0.0107	0.0613							1
ISX	intestine-specific homeobox	22q12.3				-0.3565	0.000295	0.0092				1
GFPT2	glutamine-fructose-6-phosphate transaminase 2	5q34-q35	0.717	0.03353	0.1296							1
KCNE4	In multiple Geneids		0.717	0.00905	0.0545				-0.777	3.318	5	2
ITCH	itchy E3 ubiquitin protein ligase	20q11.22	-0.465	0.00372	0.0298							1
PARP9	poly (ADP-ribose) polymerase family, member 9	3q21	0.717	0.0192	0.0904							1
ITFG3	integrin alpha FG-GAP repeat containing 3	16p13.3				-0.312	0.04527	0.1399				1
ITGA1	integrin, alpha 1	5q11.2							-0.689	2.802	5	1
SLC2A13	solute carrier family 2 (facilitated glucose transporter), member 13	12q12	0.716	0.00138	0.0147							1
TOR4A	torsin family 4, member A	9q34.3	0.716	0.00041	0.0062							1
ITGA4	integrin, alpha 4 (antigen CD49D, alpha 4 subunit of VLA-4 receptor)	2q31.3				0.315714286	0.000527143	0.011457143				1
ANKMY2	ankyrin repeat and MYND domain containing 2	7p21	0.715	0.01084	0.0617							1
DROSHA	drosha, ribonuclease type III	5p13.3	0.715	0.00014	0.0029							1
ITGA7	integrin, alpha 7	12q13				-0.318	0.03325	0.1157				1
ITGA8	integrin, alpha 8	10p13				-0.315	0.03422	0.1177				1
ITGA9	integrin, alpha 9	3p21.3				-0.349714286	0.000838571	0.014785714				1
ITGAD	integrin, alpha D	16p11.2				-0.302	0.00249	0.0273				1
ITGAE	integrin, alpha E (antigen CD103, human mucosal lymphocyte antigen 1; alpha	17p13	-0.443	0.00386	0.0305	-0.399684211	0.006222105	0.039626316				2
ITGAL	integrin, alpha L (antigen CD11A (p180), lymphocyte function-associated antig	16p11.2				-0.385	0.00024	0.0094				1
ERLEC1	endoplasmic reticulum lectin 1	2p16.2	0.715	0.00047	0.0068							1
LILRB4	leukocyte immunoglobulin-like receptor, subfamily B (with TM and ITIM doma	19q13.4	0.715	0.00869	0.0531							1
MGAT4B	mannosyl (alpha-1,3-)-glycoprotein beta-1,4-N-acetylglucosaminyltransferase,	5q35	0.715	0.00022	0.0039							1
MX2	myxovirus (influenza virus) resistance 2 (mouse)	21q22.3	0.715	0.03886	0.1428							1
ITGB1BP3	Data not found					-0.5115	0.00264	0.027				1
ITGB2	integrin, beta 2 (complement component 3 receptor 3 and 4 subunit)	21q22.3				-0.423142857	0.000554286	0.009228571				1
TMEM53	transmembrane protein 53	1p34.1	0.715	0.00016	0.0031							1
ITGB6	integrin, beta 6	2q24.2				0.3	0.00076	0.0156	-0.851	3.39333	9	2
ITGB7	integrin, beta 7	12q13.13	-1.067	0	1.00E-04	-0.301	0.00071	0.0151				2
ITGB8	integrin, beta 8	7p21.1				0.3265	0.0008625	0.0157				1
ARFGAP1	ADP-ribosylation factor GTPase activating protein 1	20q13.33	0.714	1.00E-04	0.0022							1
ITIH1	inter-alpha-trypsin inhibitor heavy chain 1	3p21.1				-0.39575	0.00045	0.0109				1
ITIH4	inter-alpha-trypsin inhibitor heavy chain family, member 4	3p21.1				-0.409	0.00048	0.0127				1
ITK	IL2-inducible T-cell kinase	5q31-q32							-0.821	3.36333	9	1
CDC42EP2	CDC42 effector protein (Rho GTPase binding) 2	11q13	0.714	0	1.00E-04							1
ITM2B	integral membrane protein 2B	13q14.3	-0.241	0.03095	0.1234							1
MEIS3P1	Meis homeobox 3 pseudogene 1	17p12	0.714	0.000615	0.0078							1
MYO1E	myosin IE	15q21-q22	0.714	0.01451	0.0748							1
BRD9	bromodomain containing 9	5p15.33	0.713	0.00077	0.0098							1
ITPKA	inositol-trisphosphate 3-kinase A	15q15.1				-0.393	0.00229	0.0262				1
ITPKB	inositol-trisphosphate 3-kinase B	1q42.13				0.384	0.00067	0.0147				1
ITPKC	inositol-trisphosphate 3-kinase C	19q13.1	-1.04	0.00033	0.0053	-0.542	0.00016	0.0079				2
ITPR1	inositol 1,4,5-trisphosphate receptor, type 1	3p26.1				-0.334666667	0.003213333	0.027166667				1
ITPR2	inositol 1,4,5-trisphosphate receptor, type 2	12p11				0.466967742	0.033113226	0.114193548				1
JOSD1	Josephin domain containing 1	22q13.1	0.713	0.00015	0.0029							1
ITPRIP	inositol 1,4,5-trisphosphate receptor interacting protein	10q25.1	-1.396	0	1.00E-04							1
ITSN1	intersectin 1 (SH3 domain protein)	21q22.1-q22.2				-0.3772	0.003892	0.025376667				1
ITSN2	intersectin 2	2pter-p25.1	-0.784	8.00E-05	0.0018							1
IVL	involucrin	1q21	-2.969	0	1.00E-04							1
LPIN2	lipin 2	18p11.31	0.713	0.01727	0.0841							1
ABI2	abl-interactor 2	2q33	0.712	0.00315	0.0265							1
COX17	COX17 cytochrome c oxidase assembly homolog (S. cerevisiae)	3q13.33	0.712	0.00305	0.026							1
IZUMO4	IZUMO family member 4	19p13.3	-0.306	0.00792	0.0498							1
JAG2	jagged 2	14q32	-0.505	6.00E-04	0.0083	-0.449	9.00E-04	0.0168				2
SLC6A6	solute carrier family 6 (neurotransmitter transporter, taurine), member 6	3p25.1	0.712	0.00526	0.0378							1
JAK2	Janus kinase 2	9p24	-0.698	0.00642	0.0432	-0.386	0.00025	0.0096				2
STAG3L3	stromal antigen 3-like 3	7q11.23	0.712	0.00217	0.0202							1
JAKMIP1	janus kinase and microtubule interacting protein 1	4p16.1				-0.3943	0.0021075	0.020685				1
JAKMIP3	Janus kinase and microtubule interacting protein 3	10q26.3	-0.208	0.03738	0.1394	-0.356666667	0.002443333	0.019783333				2
JAM2	junctional adhesion molecule 2	21q21.2				-0.376	0.00028	0.0095				1
DOK1	docking protein 1, 62kDa (downstream of tyrosine kinase 1)	2p13	0.711	8.00E-05	0.0018							1
JARID2	jumonji, AT rich interactive domain 2	6p24-p23	-0.784	0.00225	0.0207							1
JAZF1	In multiple Geneids					0.3485	0.001468	0.01791				1
JDP2	Jun dimerization protein 2	14q24.3				-0.306	0.02376	0.0944				1
JMJD1C	jumonji domain containing 1C	10q21.3							-0.855	2.8425	8	1
JMJD5	Data not found					-0.32	0.014475	0.05845				1
HABP4	hyaluronan binding protein 4	9q22.3-q31	0.711	0.0014	0.0148							1
JMY	In multiple Geneids					-0.373333333	0.000431667	0.0103				1

C1orf27	chromosome 1 open reading frame 27	1q25	0.71	0.00067	0.0088	0.313	0.00134	0.0202				2
JOSD2	Josephin domain containing 2	19q13.33	-0.229	0.0339	0.1304	-0.48	6.00E-04	0.014				2
JOSD3	Data not found								-0.956	3.064	5	1
CCT6A	chaperonin containing TCP1, subunit 6A (zeta 1)	7p11.2	0.71	0.01068	0.0613							1
JPH3	junctophilin 3	16q24.3				-0.336	0.0036975	0.02975				1
JPH4	junctophilin 4	14q11				-0.4185	7.50E-05	0.0052				1
JRK	jerky homolog (mouse)	8q24.3							0.55	2.65143	7	1
CENPW	centromere protein W	6q22.32	0.71	0.01596	0.0798							1
FLNB	filamin B, beta	3p14.3	0.71	0.00366	0.0294							1
JUB	Data not found					-0.397	0.000923333	0.016233333				1
JUNB	jun B proto-oncogene	19p13.2	-0.567	0.04914	0.1653							1
JUP	junction plakoglobin	17q21	-0.857	0.00167	0.0168	-0.3215	0.00844	0.05115				2
K6HF	Data not found								0.748	2.71	5	1
KA35	Data not found								-0.806	2.69143	7	1
KA36	Data not found								-1.016	2.96667	6	1
K-ALPHA-1	Data not found								-0.659	2.292	5	1
KALRN	kalirin, RhoGEF kinase	3q21.2				0.342	0.003698	0.02764				1
KANK1	KN motif and ankyrin repeat domains 1	9p24.3	-1.625	0	0	-0.398184211	0.003215	0.026242105				2
KANK2	KN motif and ankyrin repeat domains 2	19p13.2				-0.4413	0.005977	0.03869				1
KANK3	KN motif and ankyrin repeat domains 3	19p13.2				-0.379	0.0017475	0.02265				1
KANK4	KN motif and ankyrin repeat domains 4	1p31.3				-0.3675625	0.0034875	0.028775				1
RIC8B	resistance to inhibitors of cholinesterase 8 homolog B (C. elegans)	12q23.3	0.71	3.00E-05	0.001							1
KAT2A	K(lysine) acetyltransferase 2A	17q21				-0.349	0.003485	0.0319				1
KAT2B	K(lysine) acetyltransferase 2B	3p24	-2.331	0	0	-0.324333333	0.002085	0.024316667				2
KAT5	K(lysine) acetyltransferase 5	11q13	-0.198	0.02211	0.099							1
KATNAL2	katanin p60 subunit A-like 2	18q21.1				-0.3266	0.026902	0.0984				1
KATNB1	katanin p80 (WD repeat containing) subunit B 1	16q21				-0.3595	0.000645	0.0124				1
KATNBL1	Data not found		-0.7305	0.001	0.0116							1
KAZALD1	Kazal-type serine peptidase inhibitor domain 1	10q24.31							-0.887	3.11	6	1
KAZN	kazrin, periplakin interacting protein	1p36.21	-1.477	0	0							1
KBTBD10	kelch repeat and BTB (POZ) domain containing 10	2q31.1							-0.806	2.775	6	1
G0S2	G0/G1switch 2	1q32.2	0.708	0.00633	0.0428							1
KBTBD3	kelch repeat and BTB (POZ) domain containing 3	11q22.3				0.35	0.00672	0.046				1
KBTBD4	kelch repeat and BTB (POZ) domain containing 4	11p11.2				-0.395	0.00641	0.04485				1
KBTBD5	kelch repeat and BTB (POZ) domain containing 5	3p22.1				-0.394	0.00048	0.0126				1
KBTBD6	kelch repeat and BTB (POZ) domain containing 6	13q14.11							-0.726	2.772	5	1
KC6	keratoconus gene 6					-0.304	0.00097	0.0173				1
KCNA1	potassium voltage-gated channel, shaker-related subfamily, member 1 (episo	12p13.32	-0.27	0.02499	0.1071				0.97	2.97	7	2
KCNA2	potassium voltage-gated channel, shaker-related subfamily, member 2	1p13	-0.398	0.00105	0.0121							1
KCNA4	potassium voltage-gated channel, shaker-related subfamily, member 4	11p14							0.852	3.26571	7	1
KCNA6	potassium voltage-gated channel, shaker-related subfamily, member 6	12p13				0.333	0	0.0019				1
KCNA7	potassium voltage-gated channel, shaker-related subfamily, member 7	19q13.3				-0.420333333	0.005436667	0.039033333	0.987	2.42	6	2
KCNAB1	potassium voltage-gated channel, shaker-related subfamily, beta member 1	3q26.1				0.35	0.003348095	0.027590476				1
NOL7	nucleolar protein 7, 27kDa	6p23	0.708	0.00182	0.0179							1
KCNAB3	potassium voltage-gated channel, shaker-related subfamily, beta member 3	17p13.1	-0.242	0.03542	0.1344	-0.345	0.01293	0.06535				2
KCNB1	potassium voltage-gated channel, Shab-related subfamily, member 1	20q13.2				0.395	0.00289	0.0221				1
KCNB2	potassium voltage-gated channel, Shab-related subfamily, member 2	8q13.2				0.3519	0.001947333	0.021593333				1
KCNC1	potassium voltage-gated channel, Shaw-related subfamily, member 1	11p15				-0.383	0.00186	0.0237				1
KCNC2	potassium voltage-gated channel, Shaw-related subfamily, member 2	12q14.1				0.323	0.03301	0.1152				1
KCNC3	potassium voltage-gated channel, Shaw-related subfamily, member 3	19q13.33							1.051	2.745	6	1
SNORD4A	small nucleolar RNA, C/D box 4A	17q11	0.708	0.00903	0.0545							1
ZNF780B	zinc finger protein 780B	19q13.2	0.708	0.00281	0.0244							1
KCND3	potassium voltage-gated channel, Shal-related subfamily, member 3	1p13.3				-0.347	0.00113	0.015633333				1
KCNE1	potassium voltage-gated channel, Isk-related family, member 1	21q22.12				-0.333	0.000155	0.00705				1
KCNE1L	KCNE1-like	Xq22.3	-0.21	0.04965	0.1664							1
SNORA56	small nucleolar RNA, H/ACA box 56	Xq28	0.707	0.00346	0.0283							1
ZDHHC14	zinc finger, DHHC-type containing 14	6q25.3	0.707	0.00185	0.0181							1
KCNF1	potassium voltage-gated channel, subfamily F, member 1	2p25	-0.273	0.00926	0.0554							1
KCNG1	potassium voltage-gated channel, subfamily G, member 1	20q13	-0.6865	5.00E-06	2.00E-04							1
KCNG2	potassium voltage-gated channel, subfamily G, member 2	18q23				-0.434333333	0.00062	0.0125				1
KCNG4	potassium voltage-gated channel, subfamily G, member 4	16q24.1				-0.33	0.00982	0.053533333				1
KCNH1	potassium voltage-gated channel, subfamily H (eag-related), member 1	1q32.2				0.3175	0.007805	0.0495				1
KCNH2	potassium voltage-gated channel, subfamily H (eag-related), member 2	7q36.1				-0.369666667	0.00277	0.028233333				1
KCNH3	potassium voltage-gated channel, subfamily H (eag-related), member 3	12q13	-0.257	0.01422	0.0738							1
KCNH4	potassium voltage-gated channel, subfamily H (eag-related), member 4	17q21.2				-0.461	0.00076	0.0156				1
KCNH5	potassium voltage-gated channel, subfamily H (eag-related), member 5	14q23.1				-0.618	3.00E-05	0.0042				1
KCNH6	potassium voltage-gated channel, subfamily H (eag-related), member 6	17q23.3				-0.335	0.00449	0.037				1
KCNH7	potassium voltage-gated channel, subfamily H (eag-related), member 7	2q24.2				0.348411765	0.001501471	0.015526471				1
GRAMD1A	GRAM domain containing 1A	19q13.13	0.706	0.00089	0.0108							1

KCNIP1	Kv channel interacting protein 1	5q35.1				-0.339428571	0.003395	0.024564286				1
KCNIP2	Kv channel interacting protein 2	10q24				-0.40575	0.0002675	0.00935				1
KCNIP4	Kv channel interacting protein 4	4p15.32							-0.87525	2.7195	7.25	1
KCNJ11	potassium inwardly-rectifying channel, subfamily J, member 11	11p15.1	-0.391	0.0062	0.0422							1
KCNJ12	potassium inwardly-rectifying channel, subfamily J, member 12	17p11.2				-0.33875	0.0118775	0.0564	0.726	3.06813	8	2
KCNJ14	potassium inwardly-rectifying channel, subfamily J, member 14	19q13	-0.239	0.01536	0.0778				0.955	2.795	5	2
KCNJ15	potassium inwardly-rectifying channel, subfamily J, member 15	21q22.2	-2.232	1.00E-05	3.00E-04	-0.336	0.00802	0.0507				2
KCNJ16	potassium inwardly-rectifying channel, subfamily J, member 16	17q24.3				0.366	6.00E-05	0.0056	-0.7445	3.00202	6.5	2
KCNJ2	potassium inwardly-rectifying channel, subfamily J, member 2	17q24.3				0.392	0.00137	0.0204				1
PELO	pelota homolog (Drosophila)	5q11.2	0.706	0.00069	0.009				-0.689	2.802	5	2
KCNJ4	potassium inwardly-rectifying channel, subfamily J, member 4	22q13.1				-0.4728	0.00025	0.00744				1
KCNJ6	potassium inwardly-rectifying channel, subfamily J, member 6	21q22.1				-0.352090909	0.000994545	0.014709091				1
SMCO4	Data not found		0.706	0.00542	0.0386							1
KCNK1	potassium channel, subfamily K, member 1	1q42-q43				0.356	0.000335	0.00905				1
KCNK10	potassium channel, subfamily K, member 10	14q31.3	-0.454	0.04189	0.15	-0.449	6.00E-05	0.0054				2
KCNK13	potassium channel, subfamily K, member 13	14q32.11				-0.32	0.00045	0.01225				1
KCNK2	potassium channel, subfamily K, member 2	1q41				0.3495	0.004372778	0.031755556	-0.88	3.55833	6	2
KCNK4	potassium channel, subfamily K, member 4	11q13							0.577	2.51	6	1
APH1B	anterior pharynx defective 1 homolog B (C. elegans)	15q22.2	0.705	7.00E-05	0.0018							1
KCNK6	potassium channel, subfamily K, member 6	19q13.1	-0.794	0.00185	0.0181	-0.306	0.00745	0.0487				2
KCNK7	potassium channel, subfamily K, member 7	11q13	-0.412	0.00058	0.008							1
KCNK9	potassium channel, subfamily K, member 9	8q24.3				0.356	0.00467	0.0378				1
KCNMA1	potassium large conductance calcium-activated channel, subfamily M, alpha n	10q22.3				0.312	0.00238	0.0268				1
KCNMB1	potassium large conductance calcium-activated channel, subfamily M, beta m	5q34				-0.41	0.00068	0.0148				1
KCNMB2	potassium large conductance calcium-activated channel, subfamily M, beta m	3q26.32				0.3858	0.002838	0.02541	-0.898	3.084	5	2
SP100	SP100 nuclear antigen	2q37.1	0.705	0.00672	0.0446							1
KCNN1	potassium intermediate/small conductance calcium-activated channel, subfar	19p13.1	-0.25	0.04851	0.1641	-0.4288	0.005082	0.0362				2
KCNN2	potassium intermediate/small conductance calcium-activated channel, subfar	5q22.3	-0.167	0.03115	0.1238							1
SMG1	smg-1 homolog, phosphatidylinositol 3-kinase-related kinase (C. elegans)	16p12.3	0.704636364	0.00469	0.029263636							1
KCNQ1	potassium voltage-gated channel, KQT-like subfamily, member 1	11p15.5							-0.823	3.16	5	1
KCNQ1OT1	In multiple Geneids					-0.358	0.00111	0.0185				1
KCNQ3	potassium voltage-gated channel, KQT-like subfamily, member 3	8q24				0.392318182	0.020297955	0.082047727				1
KCNQ5	potassium voltage-gated channel, KQT-like subfamily, member 5	6q14				0.341	0.00459	0.0375				1
KCNRG	potassium channel regulator	13q14.2							-0.891	2.69	5	1
KCNS3	potassium voltage-gated channel, delayed-rectifier, subfamily S, member 3	2p24				0.685	0	6.00E-04				1
KCNT1	potassium channel, subfamily T, member 1	9q34.3				-0.482	0.00014	0.0076				1
KCNT2	In multiple Geneids					0.365469697	0.004480909	0.029133333				1
KCNU1	potassium channel, subfamily U, member 1	8p11.23				0.324	0.02294	0.0925				1
KCNV1	potassium channel, subfamily V, member 1	8q23.2	-0.364	0.00075	0.0096				0.775	2.77	6	2
KCP	kielin/chordin-like protein	7q32.1				-0.3365	0.000685	0.01295				1
KCTD1	In multiple Geneids		-2.007	0	1.00E-04	-0.514	0.00144	0.0209				2
KCTD11	potassium channel tetramerisation domain containing 11	17p13.1	-0.662	0.00416	0.0322							1
KCTD13	potassium channel tetramerisation domain containing 13	16p11.2				-0.3695	8.00E-05	0.00515				1
KRBOX4	Data not found		0.704	0.00131	0.0142							1
KCTD15	potassium channel tetramerisation domain containing 15	19q13.11	-0.81	0	2.00E-04	-0.382	0.00097	0.0173				2
KCTD16	potassium channel tetramerisation domain containing 16	5q31.3				-0.312	0.00105	0.01395				1
KCTD17	potassium channel tetramerisation domain containing 17	22q12.3				-0.3615	0.000805	0.0159				1
KCTD18	potassium channel tetramerisation domain containing 18	2q33.1	-0.273	0.03453	0.132							1
KCTD19	potassium channel tetramerisation domain containing 19	16q22.1				-0.325	0.01447	0.06475				1
KCTD2	potassium channel tetramerisation domain containing 2	17q25.1	-0.251	0.0046	0.0345							1
KCTD21	potassium channel tetramerisation domain containing 21	11q14.1	-0.5715	0.00247	0.01895							1
KCTD3	potassium channel tetramerisation domain containing 3	1q41				0.3615	0.002265	0.02425				1
KCTD5	potassium channel tetramerisation domain containing 5	16p13.3				-0.400833333	0.013361667	0.05985				1
KCTD6	potassium channel tetramerisation domain containing 6	3p14.3	-0.412	0.03725	0.1392							1
MTNR1A	melatonin receptor 1A	4q35.1	0.704	0.02154	0.0974							1
KCTD8	potassium channel tetramerisation domain containing 8	4p13	-0.272	0.03985	0.1451							1
NTMT1	Data not found		0.704	0.00386	0.0305							1
SLC7A6	solute carrier family 7 (amino acid transporter light chain, y+L system), memb	16q22.1	0.704	7.00E-04	0.0091							1
CCDC93	coiled-coil domain containing 93	2q14.1	0.703	5.00E-05	0.0013							1
KDM1	Data not found					-0.322	0.00368	0.0334				1
KDM2A	lysine (K)-specific demethylase 2A	11q13.2	-0.287	0.02126	0.0967	-0.3045	0.012365	0.0626				2
KDM2B	lysine (K)-specific demethylase 2B	12q24.31	-0.351	0.00413	0.0321	-0.383954545	0.001992273	0.018977273				2
KDM3B	lysine (K)-specific demethylase 3B	5q31				-0.384833333	0.002671667	0.025441667				1
KDM4A	lysine (K)-specific demethylase 4A	1p34.1				-0.311	0.0138	0.0687				1
KDM4B	lysine (K)-specific demethylase 4B	19p13.3	-0.287	0.00923	0.0553	-0.429107143	0.003045	0.025125				2
KDM4C	lysine (K)-specific demethylase 4C	9p24.1				-0.402490566	0.002854528	0.023379245				1
KDM4D	lysine (K)-specific demethylase 4D	11q21	-0.196	0.04301	0.1525							1
CDCA8	In multiple Geneids		0.703	0.00788	0.0496							1
KDM5D	lysine (K)-specific demethylase 5D	Yq11				-0.531857143	0.02502	0.092671429				1

KDM6B	lysine (K)-specific demethylase 6B	17p13.1				-0.4895	0.00229	0.0205				1
KDR	kinase insert domain receptor (a type III receptor tyrosine kinase)	4q11-q12							0.658	2.506	5	1
KDSR	3-ketodihydrosphingosine reductase	18q21.3				-0.3705	0.00131	0.016675				1
KEAP1	kelch-like ECH-associated protein 1	19p13.2	-0.34	0.00527	0.0378	-0.517666667	0.00385	0.028416667				2
KEL	Kell blood group, metallo-endopeptidase	7q33							-0.784	2.934	5	1
KERA	keratocan	12q22							-0.95	3.14333	6	1
KGFLP1	fibroblast growth factor 7 pseudogene	9p11.2							0.9015	3.0114275	6.75	1
KGFLP2	keratinocyte growth factor-like protein 2	9p12	-0.598	0.000775	0.0098				0.747	2.9	6	2
NCAPD2	non-SMC condensin I complex, subunit D2	12p13.3	0.703	0.00442	0.0335							1
KHDRBS2	In multiple Geneids					0.3315	0.0042825	0.034875				1
KHDRBS3	KH domain containing, RNA binding, signal transduction associated 3	8q24.2				0.352333333	0.004155	0.028033333				1
KHSRP	KH-type splicing regulatory protein	19p13.3				-0.505666667	0.001246667	0.0189				1
KIAA0020	KIAA0020	9p24.2				-0.3655	0.00985	0.0514				1
TNFRSF21	tumor necrosis factor receptor superfamily, member 21	6p21.1	0.703	0.00688	0.0454	0.314	0.027175	0.10045				2
KIAA0087	KIAA0087	7p15.2	-0.308	0.01924	0.0905							1
KIAA0090	Data not found					-0.324	0.010335	0.0573				1
KIAA0141	KIAA0141	5q31.3				-0.3546	0.004428	0.02822				1
KIAA0143	Data not found								-0.811	2.80833	6	1
KIAA0174	Data not found					-0.314	0.00144	0.0209				1
KIAA0182	KIAA0182	16q24.1				-0.4305	0.00734	0.0391	0.514	2.49	5	2
KIAA0195	KIAA0195	17q25.1				-0.4	0.0024325	0.024				1
KIAA0196	KIAA0196	8q24.13				0.36125	0.0164325	0.0674				1
KIAA0226	KIAA0226	3q29	-0.526	0.00684	0.0452							1
TNFSF4	tumor necrosis factor (ligand) superfamily, member 4	1q25	0.703	0.03761	0.14	0.33	0.008453333	0.0474				2
KIAA0232	KIAA0232	4p16.1	-0.994	1.00E-05	3.00E-04	-0.350230769	0.005013077	0.0342				2
KIAA0241	Data not found								-0.76	2.63167	6	1
KIAA0247	KIAA0247	14q24.1	-0.633	0.00084	0.0104	-0.381	0.00737	0.03815				2
KIAA0256	Data not found								-0.783	2.924	5	1
KIAA0317	KIAA0317	14q24.3							-0.966	2.916	5	1
KIAA0319L	KIAA0319-like	1p34.2				-0.358	0.00596	0.039				1
KIAA0355	KIAA0355	19q13.11				-0.3635	0.00215	0.02415				1
KIAA0391	KIAA0391	14q13.2				-0.445	0.00811	0.051				1
KIAA0427	Data not found					-0.370619048	0.010047143	0.052080952				1
KIAA0495	Data not found					-0.558	1.00E-05	0.0028				1
KIAA0513	KIAA0513	16q24.1				-0.407375	0.00173125	0.0199125				1
KIAA0528	KIAA0528	12p12.1				0.34125	0.00147875	0.019225				1
KIAA0556	KIAA0556	16p12.1	-0.279	0.03246	0.1268	-0.301	0.0037	0.0335				2
KIAA0562	Data not found					-0.739	0.00028	0.01				1
KIAA0564	KIAA0564	13q14.11				0.336875	0.01015125	0.0504				1
KIAA0649	Data not found								0.705	3.09	7	1
KIAA0652	Data not found					-0.314	0.01442	0.0705				1
KIAA0664	KIAA0664	17p13.3				-0.399666667	0.009653333	0.044866667				1
KIAA0674	Data not found								-0.967	2.92167	6	1
KIAA0690	Data not found								-0.717	2.394	5	1
KIAA0692	Data not found								1.032	4.63667	9	1
KIAA0746	Data not found					-0.381590909	0.002173636	0.020822727				1
KIAA0753	KIAA0753	17p13.1				-0.465	0.00087	0.0165				1
KIAA0773	Data not found								0.695	2.482	5	1
KIAA0774	Data not found					0.33125	0.0138675	0.066675	-0.893	2.913875	6.5	2
KIAA0825	In multiple Geneids		-0.658666667	0.001423333	0.0129							1
KIAA0853	Data not found								-0.728	2.81875	8	1
KIAA0892	Data not found					-0.445142857	0.001948571	0.020257143				1
VIM	vimentin	10p13	0.703	0.02535	0.1082							1
KIAA0922	KIAA0922	4q31.3	-0.826	0.00156	0.016	-0.35725	0.0071425	0.0409				2
WFS1	Wolfram syndrome 1 (wolframin)	4p16.1	0.703	0.01028	0.0597							1
KIAA1012	Data not found					-0.338666667	0.032235	0.112716667				1
KIAA1026	Data not found					-0.365	0.006820313	0.038425				1
KIAA1045	KIAA1045	9p13.3	-0.331	0.00313	0.0264	-0.344	3.00E-05	0.0042				2
KIAA1086	Data not found								1.093	4.51333	6	1
KIAA1189	Data not found								-0.919	3.274443333	6	1
KIAA1191	KIAA1191	5q35.2	-0.568	0.00668	0.0444	-0.377666667	0.007093333	0.0408				2
KIAA1193	Data not found								0.669	2.65167	6	1
KIAA1199	KIAA1199	15q24				0.322	0.01873	0.082				1
XPR1	xenotropic and polytropic retrovirus receptor 1	1q25.1	0.703	0.00059	0.0081							1
KIAA1211L	Data not found		-0.483	0.00338	0.0279							1
KIAA1217	KIAA1217	10p12.31				0.71	4.00E-05	0.0048				1
KIAA1219	Data not found					0.3245	0.00058	0.01285				1
KIAA1239	KIAA1239	4p14				-0.342	0.00212	0.0252				1
C12orf49	chromosome 12 open reading frame 49	12q24.22	0.702	0.0023	0.0212							1

KIAA1267	Data not found					0.3483	0.028229	0.10203				1
KIAA1274	KIAA1274	10q22.1				-0.45	0.000125	0.00705				1
KIAA1310	Data not found					-0.345	0.00168	0.0225				1
FAM118B	family with sequence similarity 118, member B	11q24.2	0.702	0.00014	0.0028							1
KIAA1324L	KIAA1324-like	7q21.12				0.3554	0.004326	0.03354				1
KIAA1328	KIAA1328	18q12.2				-0.348666667	0.01341	0.066166667				1
NF1	neurofibromin 1	17q11.2	0.702	0.002865	0.0205							1
KIAA1409	Data not found					-0.302	0.00152	0.0214				1
SCMH1	sex comb on midleg homolog 1 (Drosophila)	1p34	0.702	0.00132	0.0143							1
KIAA1430	KIAA1430	4q35.1				-0.4	0.008152857	0.038885714				1
KIAA1432	KIAA1432	9p24.1							-0.91	2.37286	7	1
TNKS	tankyrase, TRF1-interacting ankyrin-related ADP-ribose polymerase	8p23.1	0.702	0.03182	0.1253							1
KIAA1468	KIAA1468	18q21.33	-1.336	0	0							1
KIAA1486	Data not found					0.309166667	0.000403333	0.007383333				1
TOMM34	translocase of outer mitochondrial membrane 34		0.702	0.00419	0.0323	0.303	0.00114	0.0187				2
KIAA1529	Data not found					-0.392	0.00182	0.0234				1
KIAA1530	Data not found					-0.561	7.00E-05	0.0058				1
KIAA1539	KIAA1539 protein					-0.349666667	0.00426	0.0304				1
KIAA1543	Data not found					-0.405	0.00829	0.0516				1
FBXL2	F-box and leucine-rich repeat protein 2	3p22.3	0.701	0.00564	0.0396							1
KIAA1549L	Data not found		-0.382	0.04648	0.1598							1
TMEM194A	transmembrane protein 194A	12q13.3	0.701	0.00765	0.0487							1
KIAA1576	Data not found								0.576	2.51	6	1
KIAA1586	KIAA1586	6p12.1				-0.33	0.00012	0.007				1
FLJ42393	uncharacterized LOC401105	3q27.3	0.7	0.00402	0.0314							1
KIAA1602	Data not found					-0.3678	0.000742	0.01208				1
KIAA1604	Data not found								-0.866	2.68857	7	1
KIAA1618	KIAA1618	17q25.3				-0.328333333	0.005536667	0.037633333				1
KIAA1627	Data not found								-0.941	2.6	5	1
KIAA1632	Data not found					-0.373785714	0.017446429	0.074035714				1
KIAA1644	KIAA1644					-0.4029375	0.001944375	0.01556875				1
KIAA1671	KIAA1671	22q11.23	-0.44	0.00593	0.041	-0.40490625	0.00130375	0.013675				2
KIAA1683	KIAA1683	19p13.1	-0.537	0.00036	0.0057	-0.3455	0.00773	0.0397				2
KIAA1712	Data not found								-0.804	2.95667	6	1
TRIM52	tripartite motif containing 52	5q35.3	0.699	0.00171	0.0171							1
KIAA1737	KIAA1737	14q24.3	-0.709	0.00081	0.0101							1
KIAA1751	KIAA1751	1p36.33				-0.37525	0.00632	0.03875				1
KIAA1772	Data not found					-0.317	0.01863	0.07025				1
KIAA1797	Data not found					-0.358428571	0.005880714	0.036242857				1
ACBD5	acyl-CoA binding domain containing 5	10p12.1	0.698	0.00975	0.0575							1
KIAA1815	Data not found								-0.976	3.16286	7	1
KIAA1826	Data not found					0.34	0.0143	0.0701				1
APOBEC1	apolipoprotein B mRNA editing enzyme, catalytic polypeptide 1	12p13.1	0.698	0.02698	0.1126							1
KIAA1843	KIAA1843 protein	2q35							0.888	3.23429	7	1
KIAA1875	In multiple Geneids								0.615	2.858	5	1
KIAA1904	Data not found								0.567	2.606	5	1
KIAA1909	KIAA1909 protein								0.99	3.02125	8	1
PPAPDC1B	phosphatidic acid phosphatase type 2 domain containing 1B	8p11.23	0.698	0.00178	0.0177							1
KIAA1984	KIAA1984	9q34.3				-0.422	0.02179	0.0897				1
KIAA2018	KIAA2018	3q13.2	-0.422	0.03296	0.1282							1
KIAA2022	KIAA2022	Xq13.3	-0.477	0.00122	0.0134							1
KIAA2026	KIAA2026	9p24.1							-0.891	3.01286	7	1
KIDINS220	kinase D-interacting substrate, 220kDa	2p24				0.362	0.00015	0.0076				1
ZNF320	In multiple Geneids		0.698	0.00527	0.0378							1
KIF13A	kinesin family member 13A	6p23				-0.354833333	0.00494	0.037283333				1
C7orf13	chromosome 7 open reading frame 13	7q36.3	0.697	0.00169	0.0169							1
IGSF6	immunoglobulin superfamily, member 6	16p12.2	0.697	0.0368	0.138							1
KIF15	kinesin family member 15	3p21.31				-0.3655	0.0001875	0.006825				1
KIF16B	kinesin family member 16B	20p11.23				0.373333333	0.005952667	0.039106667				1
KIF17	kinesin family member 17	1p36.12				-0.382	0.006416667	0.042166667				1
OVOS	ovostatin	12p13	0.697	0.0224	0.0996							1
KIF19	kinesin family member 19	17q25.1				-0.301	0.01781	0.0796				1
KIF1A	kinesin family member 1A	2q37.3				-0.308666667	0.003856667	0.032866667				1
KIF1B	kinesin family member 1B	1p36.2	-0.626	0.00244	0.022	-0.368714286	0.007394286	0.045171429				2
KIF1C	kinesin family member 1C	17p13.2	-0.73	1.00E-05	3.00E-04	-0.40625	0.006865	0.035675				2
KIF2	Data not found								-0.743	2.49333	6	1
KIF20A	kinesin family member 20A	5q31							-0.767	2.53417	6	1
KIF21A	kinesin family member 21A	12q12	-1.013	0.00067	0.0088							1
MIA2	melanoma inhibitory activity 2	14q13.2	0.696	0.00024	0.0042				-0.942	2.918	5	2

TNFRSF1B	tumor necrosis factor receptor superfamily, member 1B	1p36.22	0.696	0.0021	0.0198							1
ALPPL2	alkaline phosphatase, placental-like 2	2q37	0.695	0.04337	0.1532							1
KIF24	In multiple Geneids								-0.72	2.72833	6	1
CENPA	centromere protein A	2p24-p21	0.695	0.02456	0.1058							1
AGRN	In multiple Geneids		0.694	7.00E-05	0.0018							1
KIF3A	kinesin family member 3A	5q31							-0.943	2.786	5	1
ATAD2	ATPase family, AAA domain containing 2	8q24.13	0.694	0.02623	0.1107	0.313	0.0454	0.1402				2
DNAJC3	DnaJ (Hsp40) homolog, subfamily C, member 3	13q32.1	0.694	0.01047	0.0605							1
FAM213B	family with sequence similarity 213, member B	1p36.32	0.694	7.00E-05	0.0018							1
PLA1A	phospholipase A1 member A	3q13.13-q13.2	0.694	0.0275	0.1138							1
KIF5A	kinesin family member 5A	12q13.13				-0.374666667	0.00062	0.014				1
KIF5C	kinesin family member 5C	2q23.1				0.362	1.00E-05	0.0032				1
KIF6	In multiple Geneids					0.34469697	0.010030606	0.052521212				1
UBE2C	ubiquitin-conjugating enzyme E2C	20q13.12	0.694	0.00916	0.055							1
KIFAP3	kinesin-associated protein 3	1q24.2				0.336166667	0.00129	0.017666667				1
ZNF468	zinc finger protein 468	19q13.41	0.694	0.00083	0.0103							1
KIFC2	kinesin family member C2	8q24.3	-0.309	0.01781	0.0858							1
RUNX2	runt-related transcription factor 2	6p21	0.693	0.04565	0.1583	0.335	0.006665	0.044183333				2
KIR2DL5A	In multiple Geneids					-0.311	0.01165	0.0623	0.954	2.31	5	2
KIR2DL5B	In multiple Geneids					-0.311	0.01165	0.0623	0.954	2.31	5	2
KIR2DS1	killer cell immunoglobulin-like receptor, two domains, short cytoplasmic tail, 1	19q13.4	-0.226	0.03672	0.1378	-0.384666667	0.020936667	0.078966667				2
KIR2DS2	In multiple Geneids					-0.609	0.00011	0.0069				1
KIR3DL3	In multiple Geneids		-0.3525	0.0098	0.05685							1
KIR3DS1	killer cell immunoglobulin-like receptor, three domains, short cytoplasmic tail,	19q13.4				-0.321	0.007035	0.04295				1
KIR3DX1	killer cell immunoglobulin-like receptor, three domains, X1	19q13.42				-0.377	0.00286	0.0293				1
KIRREL	kin of IRRE like (Drosophila)	1q21-q25							0.812	2.718	5	1
KIRREL2	In multiple Geneids					-0.352	0.00113	0.0186				1
KIRREL3	kin of IRRE like 3 (Drosophila)	11q24				0.340375	0.0098975	0.0493375				1
KIRREL3-AS3	KIRREL3 antisense RNA 3 (non-protein coding)	11q24.2	-0.297	0.00725	0.047							1
KITLG	KIT ligand	12q22				0.313	0.0036	0.033				1
KLB	klotho beta	4p14							-0.877	2.905	8	1
KLC1	kinesin light chain 1	14q32.3	-0.326	0.01111	0.0628	-0.345	0.005003333	0.038333333				2
KLC3	kinesin light chain 3	19q13	-0.413	0.00034	0.0054							1
MXRA8	matrix-remodelling associated 8	1p36.33	0.692	0.00808	0.0505							1
KLF1	Kruppel-like factor 1 (erythroid)	19p13.2				-0.347	0.00984	0.0567				1
GDPD5	glycerophosphodiester phosphodiesterase domain containing 5	11q13.4-q13.5	0.691	0.00036	0.0056							1
NUP155	nucleoporin 155kDa	5p13.1	0.691	0.00943	0.0561							1
KLF12	Kruppel-like factor 12	13q22				0.3165	0.005995	0.03935				1
KLF13	Kruppel-like factor 13	15q12				-0.3185	0.01338	0.062				1
KLF16	Kruppel-like factor 16	19p13.3				-0.5755	0.000525	0.0121				1
KLF2	Kruppel-like factor 2 (lung)	19p13.11				-0.313	0.00414	0.0355				1
KLF3	Kruppel-like factor 3 (basic)	4p14	-0.433	0.03	0.1207							1
KLF4	Kruppel-like factor 4 (gut)	9q31	-1.145	0.00952	0.0565							1
KLF5	Kruppel-like factor 5 (intestinal)	13q22.1							-0.63	2.562	5	1
KLF8	Kruppel-like factor 8	Xp11.21				0.302	0.00065	0.0145				1
KLHDC10	kelch domain containing 10	7q32.2	-0.35	0.00909	0.0547	-0.329	0.0162	0.0754				2
PLEKHB2	pleckstrin homology domain containing, family B (evectins) member 2	2q21.1	0.691	0.00108	0.0123							1
KLHDC5	kelch domain containing 5	12p11.22				0.314	0.0029	0.0295				1
KLHDC6	Data not found								-0.563	2.97	6	1
KLHDC8A	kelch domain containing 8A	1q32.1	-1.213	0	0							1
KLHDC8B	kelch domain containing 8B	3p21.31	-1.236	1.00E-05	4.00E-04	-0.397	0.00501	0.0393				2
KLHL1	kelch-like 1 (Drosophila)	13q21				0.313	0.004685	0.0379				1
KLHL10	kelch-like 10 (Drosophila)	17q21.2				-0.354	0.00819	0.0513				1
KLHL13	kelch-like 13 (Drosophila)	Xq23-q24				0.377	0.00225	0.026	-0.848	3.44667	6	2
KLHL17	kelch-like 17 (Drosophila)	1p36.33				-0.392	0.00022	0.0091				1
KLHL18	kelch-like 18 (Drosophila)	3p21.31	-0.513	0.00082	0.0102	-0.371428571	0.005421429	0.031214286				2
KLHL2	kelch-like 2, Mayven (Drosophila)	4q21.2	-1.33	0	1.00E-04	-0.3626	0.003848	0.02746				2
KLHL20	kelch-like 20 (Drosophila)	1q25.1							-0.706	2.675	6	1
KLHL21	kelch-like 21 (Drosophila)	1p36.31	-0.453	0.00304	0.0259	-0.418	0.0033	0.025666667				2
KLHL22	kelch-like 22 (Drosophila)	22q11.21				-0.422333333	0.0003775	0.009175				1
TMEM248	transmembrane protein 248	7q11.21	0.691	0.00019	0.0035							1
TOR1B	torsin family 1, member B (torsin B)	9q34	0.691	0.00115	0.0129							1
KLHL26	kelch-like 26 (Drosophila)	19p13.11				-0.3915	0.00882	0.047883333				1
KLHL3	kelch-like 3 (Drosophila)	5q31				-0.351333333	0.000556667	0.012533333				1
KLHL30-AS1	KLHL30 antisense RNA 1 (non-protein coding)	2q37.3	-0.36	0.00747	0.048							1
KLHL36	kelch-like 36 (Drosophila)	16q24.1	-0.226	0.03033	0.1216	-0.334	0.00549	0.0412				2
KLHL38	kelch-like 38 (Drosophila)	8q24.13				0.331	0.01609	0.0751				1
KLHL40	Data not found		-0.265	0.02055	0.0944							1
CDCA7	cell division cycle associated 7	2q31	0.69	0.02106	0.096							1

KLHL6	kelch-like 6 (Drosophila)	3q27.3							0.726	2.52667	6	1
KLHL8	kelch-like 8 (Drosophila)	4q22.1				-0.4035	0.000525	0.0119				1
KLHL9	In multiple Geneids								-1.001	3.06875	8	1
SEPT11	septin 11	4q21.1	0.689	0.00146	0.0153							1
KLK10	kallikrein-related peptidase 10	19q13	-1.784	0.01696	0.0831	-0.309	0.02173	0.0896				2
KLK11	kallikrein-related peptidase 11	19q13.33	-2.855	0	1.00E-04							1
KLK12	kallikrein-related peptidase 12	19q13.33	-2.694	4.00E-05	0.0012							1
KLK13	In multiple Geneids		-4.339	0	2.00E-04							1
KLK3	kallikrein-related peptidase 3	19q13.41				-0.3875	0.001175	0.0173				1
KLK4	kallikrein-related peptidase 4	19q13.41							0.684666667	2.526	5	1
KLK6	kallikrein-related peptidase 6	19q13.3				-0.41	0.00263	0.0281				1
KLK7	kallikrein-related peptidase 7	19q13.41	-1.478	0.01724	0.0839							1
KLK8	kallikrein-related peptidase 8	19q13	-1.693	0.00061	0.0083							1
KLK9	In multiple Geneids		-0.506	0.008	0.0502	-0.43	0.00066	0.0146				2
KLKBL4	Data not found					-0.306	0.00601	0.0433				1
KLRA1	Data not found								-0.942	3.11857	7	1
KLRB1	killer cell lectin-like receptor subfamily B, member 1	12p13							-0.933	3.45333	6	1
KLRC1	killer cell lectin-like receptor subfamily C, member 1	12p13							-0.881	2.96125	8	1
KLRC2	killer cell lectin-like receptor subfamily C, member 2	12p13							-0.98	3.09857	7	1
KLRC3	killer cell lectin-like receptor subfamily C, member 3	12p13							-0.98	2.982383333	7	1
KLRC4	killer cell lectin-like receptor subfamily C, member 4	12p13.2-p12.3							-0.958	2.68143	7	1
KLRC4-KLRK1	KLRC4-KLRK1 readthrough	12p	-0.51	0.0287	0.1172							1
KLRD1	killer cell lectin-like receptor subfamily D, member 1	12p13							-0.776	2.885	8	1
KLRF1	killer cell lectin-like receptor subfamily F, member 1	12p13.31	-0.997	0.00011	0.0024							1
KLRG1	killer cell lectin-like receptor subfamily G, member 1	12p13.31							-0.871	3.256	5	1
KLRG2	killer cell lectin-like receptor subfamily G, member 2	7q34	-0.433	0.00129	0.014	-0.309	0.016015	0.06655				2
KLRK1	killer cell lectin-like receptor subfamily K, member 1	12p13.2-p12.3							-0.887	2.8025	8	1
COL6A1	collagen, type VI, alpha 1	21q22.3	0.688	0.01941	0.091							1
KNDC1	kinase non-catalytic C-lobe domain (KIND) containing 1	10q26.3				-0.3785	0.001415	0.01905				1
HEATR1	HEAT repeat containing 1	1q43	0.688	0.00334	0.0276							1
CDX1	caudal type homeobox 1	5q32	0.687	0.00235	0.0215							1
PECAM1	platelet/endothelial cell adhesion molecule 1	17q23.3	0.687	0.01567	0.0789							1
KPNA2	karyopherin alpha 2 (RAG cohort 1, importin alpha 1)	17q24.2							-0.819	2.48	5	1
KPNA6	karyopherin alpha 6 (importin alpha 7)	1p35.1				-0.34575	0.0197975	0.07485				1
KPRP	keratinocyte proline-rich protein	1q21.3	-3.231	9.00E-05	0.0021							1
KPTN	kaptin (actin binding protein)	19q13.32				-0.342	0.00147	0.0211				1
SH3PXD2B	SH3 and PX domains 2B	5q35.1	0.687	0.01544	0.078							1
KRBA1	KRAB-A domain containing 1	7q36				-0.32	0.01466	0.0712				1
YTHDF1	YTH domain family, member 1	20q13.33	0.687	0	2.00E-04							1
KRCC1	lysine-rich coiled-coil 1	2p11.2	-1.012	0.00027	0.0046							1
KREMEN1	kringle containing transmembrane protein 1	22q12.1	-2.223	0	0	-0.427217391	0.00241087	0.019895652				2
KRI1	KRI1 homolog (S. cerevisiae)	19p13.2				-0.468	0.00208	0.0247				1
KRT1	keratin 1	12q13.13	-1.989	0.00069	0.0091							1
KRT10	keratin 10	17q21	-0.915	0.04405	0.1548							1
KRT12	keratin 12	17q12	-0.333	0.02999	0.1206							1
KRT13	keratin 13	17q12-q21.2	-3.861	7.00E-04	0.0091				0.568	2.69667	6	2
KRT14	keratin 14	17q12-q21							0.971	3.162	5	1
KRT15	keratin 15	17q21.2	-4.244	0	1.00E-04							1
KRT16	keratin 16	17q21.2	-1.574	0.01389	0.0727							1
KRT16P3	keratin 16 pseudogene 3	17p11.2	-1.676	0.00183	0.0179							1
C1orf106	chromosome 1 open reading frame 106	1q32.1	0.686	0.00053	0.0075							1
CSF3R	colony stimulating factor 3 receptor (granulocyte)	1p35-p34.3	0.686	0.01379	0.0724							1
KRT2	keratin 2	12q13.13	-0.612	0.0036	0.0291							1
KRT24	keratin 24	17q21.2							-0.896	2.79333	6	1
KRT25D	Data not found								-0.89	3.16429	7	1
KRT27	keratin 27	17q21.2	-0.345	0.00157	0.0161							1
KRT28	keratin 28	17q21.2	-0.27	0.00666	0.0443							1
KRT3	keratin 3	12q13.13	-1.245	1.00E-05	5.00E-04							1
KRT31	keratin 31	17q12-q21	-1.863	0	2.00E-04							1
KRT32	keratin 32	17q21.2	-1.074	4.00E-05	0.001							1
KRT33A	keratin 33A	17q12-q21	-0.698	0.00039	0.006							1
KRT33B	keratin 33B	17q21.2	-0.418	0.01316	0.0701							1
KRT34	keratin 34	17q21.2	-0.307	0.00954	0.0566							1
KRT35	keratin 35	17q21.2	-0.593	0.00108	0.0123							1
KRT36	keratin 36	17q12-q21	-0.393	0.01359	0.0716							1
KRT37	keratin 37	17q12-q21	-0.344	0.00766	0.0487							1
KRT38	keratin 38	17q12-q21	-0.284	0.01557	0.0785							1
KRT4	keratin 4	12q13.13	-3.875	0.00026	0.0044							1
KRT40	keratin 40	17q21.2				-0.401	0.00372	0.0336				1

KRT5	keratin 5	12q13.13	-4.07	5.00E-04	0.0072							1
KRT6A	keratin 6A	12q13.13	-3.679	0.00115	0.0129				0.624	2.895	6	2
KRT6B	keratin 6B	12q13.13	-3.453	0.00156	0.016							1
KRT6C	In multiple Geneids		-3.187	0.00361	0.0291				0.624	2.895	6	2
KRT71	keratin 71	12q13.13	-0.413	0.00026	0.0045							1
KRT72	keratin 72	12q13.13	-0.237	0.00641	0.0432							1
KRT73	keratin 73	12q13.3	-0.323	0.00492	0.0361							1
KRT74	keratin 74	12q13.13	-0.495	0.001	0.0117							1
KRT77	keratin 77	12q13.13	-0.477	0.00048	0.0069							1
KRT78	keratin 78	12q13.13	-4.934	0	0	-0.326	2.00E-05	0.0036				2
KRT79	keratin 79	12q13.13	-0.577	0.00083	0.0103							1
GAL	In multiple Geneids		0.686	0.04115	0.1484							1
KRT80	keratin 80	12q13.13	-1.606	0.00058	0.008							1
KRT83	keratin 83	12q13				-0.316	0.00914	0.0545				1
KRT84	keratin 84	12q13	-0.296	0.04968	0.1665							1
KRTAP10-1	In multiple Geneids		-0.311	0.00729	0.0472				0.661	2.848	5	2
KRTAP10-12	keratin associated protein 10-12	21q22.3	-0.352	0.00325	0.0272							1
KRTAP10-2	In multiple Geneids		-0.447	8.00E-04	0.01							1
KRTAP10-3	In multiple Geneids		-0.38	0.01229	0.0672							1
KRTAP10-4	keratin associated protein 10-4	21q22.3							0.756	3.56667	6	1
KRTAP10-7	In multiple Geneids		-0.285	0.04299	0.1525							1
KRTAP10-9	In multiple Geneids		-0.324	0.01584	0.0794							1
KRTAP1-1	keratin associated protein 1-1	17q12							-0.974	3.135	8	1
KRTAP11-1	keratin associated protein 11-1	21q22.1							-0.978	3.29125	8	1
KRTAP12-1	keratin associated protein 12-1	21q22.3	-0.246	0.04043	0.1465							1
KRTAP1-3	keratin associated protein 1-3	17q12-q21							-0.974	3.135	8	1
KRTAP13-1	In multiple Geneids								-1.05	3.53	7	1
KRTAP13-2	keratin associated protein 13-2	21q22.1							-0.98	3.755	8	1
KRTAP13-3	keratin associated protein 13-3	21q22.1							-0.84	3.00056	9	1
KRTAP13-4	In multiple Geneids								-0.84	3.00056	9	1
KRTAP1-5	keratin associated protein 1-5	17q12-q21	-0.268	0.02022	0.0934							1
KRTAP15-1	In multiple Geneids								-0.952	2.746	5	1
KRTAP19-1	keratin associated protein 19-1	21q22.1							-0.804	2.89167	6	1
KRTAP19-2	keratin associated protein 19-2	21q22.1							-0.8255	2.711945	9	1
KRTAP19-3	keratin associated protein 19-3	21q22.1							-0.758	2.785	7	1
KRTAP19-4	keratin associated protein 19-4	21q22.1							-0.664	2.745	5	1
KRTAP19-5	keratin associated protein 19-5	21q22.1							-0.589	2.50333	6	1
KRTAP19-6	keratin associated protein 19-6	21q22.1							-0.969	2.85	8	1
KRTAP19-7	keratin associated protein 19-7	21q22.1							-0.869	3.01571	7	1
KRTAP19-8	keratin associated protein 19-8	21q22.11	-0.403	0.00338	0.0279							1
KRTAP20-1	keratin associated protein 20-1	21q22.1							-1.026	3.13143	7	1
KRTAP20-2	keratin associated protein 20-2	21q22.1							-0.952	3.32125	8	1
KRTAP20-3	In multiple Geneids		-0.254	0.00812	0.0507							1
KRTAP21-2	keratin associated protein 21-2	21q22.1							-0.957	2.812	5	1
KRTAP22-1	keratin associated protein 22-1	21q22.1							-0.867	2.74562	8	1
KRTAP23-1	keratin associated protein 23-1	21q22.1							-0.905	3.042	10	1
KRTAP2-4	keratin associated protein 2-4	17q21.2	-0.251	0.01278	0.069							1
KRTAP24-1	keratin associated protein 24-1	21q22.11	-0.265	0.023	0.1014							1
KRTAP26-1	keratin associated protein 26-1	21q22.11							-0.948	3.05444	9	1
KRTAP27-1	keratin associated protein 27-1	21q22.11				-0.303	0.00121	0.0193				1
KRTAP3-2	keratin associated protein 3-2	17q12-q21							-1.016	2.96667	6	1
KRTAP3-3	keratin associated protein 3-3	17q12-q21							-0.725	2.51833	6	1
KRTAP4-10	Data not found								-0.76	2.55667	6	1
KRTAP4-11	In multiple Geneids		-0.3765	0.017825	0.0853							1
KRTAP4-12	keratin associated protein 4-12	17q12-q21							-0.571	2.56714	7	1
KRTAP4-2	keratin associated protein 4-2	17q12-q21	-0.281	0.02716	0.1131							1
KRTAP4-3	keratin associated protein 4-3	17q12-q21	-0.297	0.03553	0.1347							1
KRTAP4-4	keratin associated protein 4-4	17q12-q21							-0.757	2.74	5	1
KRTAP4-5	keratin associated protein 4-5	17q12-q21							-0.853	3.26833	6	1
KRTAP5-3	keratin associated protein 5-3	11p15.5	-0.459	9.00E-04	0.0109							1
KRTAP5-6	In multiple Geneids								0.801	3.148	5	1
KRTAP5-7	In multiple Geneids								1.099	3.545	8	1
KRTAP6-1	keratin associated protein 6-1	21q22.1							-1.026	3.13143	7	1
KRTAP6-2	keratin associated protein 6-2	21q22.1							-0.867	2.74562	8	1
KRTAP6-3	keratin associated protein 6-3	21q22.1							-0.974	3.05889	9	1
KRTAP8-1	keratin associated protein 8-1	21q22.1							-1.027	2.97833	6	1
KRTAP9-2	keratin associated protein 9-2	17q12-q21							-1.149	4.428	5	1
KRTAP9-3	keratin associated protein 9-3	17q12-q21							-1.09	3.774	5	1
KRTAP9-8	keratin associated protein 9-8	17q12-q21	-0.358	0.0131	0.07							1

SLC35G1	solute carrier family 35, member G1	10q23.33	0.686	0.01796	0.0863							1
SMYD3	SET and MYND domain containing 3	1q44	0.686	4.00E-05	0.0012	0.316	0.007575	0.04435				2
KRTHB6	Data not found								0.768	3.404	5	1
KSR1	kinase suppressor of ras 1	17q11.2				0.318	0.00408	0.0352				1
KSR2	kinase suppressor of ras 2	12q24.22-q24.23				0.317	0.01254	0.065				1
KTI12	KTI12 homolog, chromatin associated (S. cerevisiae)	1p32.3							-0.881	3.28714	7	1
Kua-UEV	Data not found								0.826	2.81125	8	1
KYNU	kynureninase	2q22.2				0.31725	0.003845	0.02505	-0.976	3.20333	6	2
L1TD1	LINE-1 type transposase domain containing 1	1p31.3				-0.345	0.00471	0.038				1
L3MBTL	Data not found								0.721	2.56	6	1
L3MBTL2	l(3)mbt-like 2 (Drosophila)	22q13.31-q13.33				-0.4565	0.000245	0.00755				1
VNN2	vanin 2	6q23-q24	0.685	0.02932	0.1187							1
L3MBTL4	l(3)mbt-like 4 (Drosophila)	18p11.31	-1.178	0	0	-0.315	0.00296	0.0298				2
CEP290	In multiple Geneids		0.684	0.00558	0.0393							1
LAD1	ladinin 1	1q25.1-q32.3	-0.756	0.0104	0.0602							1
LAG3	lymphocyte-activation gene 3	12p13.32	-0.188	0.0274	0.1136	-0.331	0.00014	0.0075				2
LAIR2	leukocyte-associated immunoglobulin-like receptor 2	19q13.4							0.778	2.754	5	1
LAMA1	In multiple Geneids					-0.435	0.00015	0.00745				1
LAMA2	laminin, alpha 2	6q22-q23				0.481	0.001385	0.01945				1
LAMA3	laminin, alpha 3	18q11.2				-0.313	0.01588	0.0745				1
IL18R1	interleukin 18 receptor 1	2q12	0.684	0.00255	0.0227	0.304	0.000805	0.01505				2
METTL13	methyltransferase like 13	1q24-q25.3	0.684	0.00048	0.007							1
LAMB2	laminin, beta 2 (laminin S)	3p21				-0.512	0	0.0023				1
PRKCDBP	In multiple Geneids		0.684	0.0406	0.147							1
LAMB4	laminin, beta 4	7q31	-2.313	0	1.00E-04							1
TRIB1	tribbles homolog 1 (Drosophila)	8q24.13	0.684	0.02072	0.0949							1
C1RL	complement component 1, r subcomponent-like	12p13.31	0.683	0.01449	0.0747							1
LAMC3	laminin, gamma 3	9q31-q34				-0.3	0.01931	0.0835				1
LAMP2	lysosomal-associated membrane protein 2	Xq24	-0.378	0.01707	0.0835							1
LAMTOR3	late endosomal/lysosomal adaptor, MAPK and MTOR activator 3	4q23	-0.651	0.00062	0.0084							1
COLGALT1	Data not found		0.683	1.00E-05	3.00E-04							1
MANBA	mannosidase, beta A, lysosomal	4q22-q25	0.683	0.00172	0.0171							1
LANCL3	LanC lantibiotic synthetase component C-like 3 (bacterial)	Xp21.1				0.339	0.0080175	0.04455				1
LAP3	leucine aminopeptidase 3	4p15.32				-0.3534	0.000842	0.0144				1
LAPTM4A	lysosomal protein transmembrane 4 alpha	2p24.1							-0.728	2.50833	6	1
SLC7A9	solute carrier family 7 (glycoprotein-associated amino acid transporter light ch	19q13.1	0.683	0.01845	0.088							1
LAPTM5	lysosomal protein transmembrane 5	1p34				-0.546	1.00E-05	0.0024				1
LARGE	like-glycosyltransferase	22q12.3				-0.389302857	0.001392857	0.014249714				1
LARP1	La ribonucleoprotein domain family, member 1	5q33.2				-0.370714286	0.003555	0.0276				1
LARP1B	La ribonucleoprotein domain family, member 1B	4q28.2				-0.389	0.00287	0.0293				1
LARP4	La ribonucleoprotein domain family, member 4	12q13.12				-0.309	0.00336	0.0318				1
LARP4B	La ribonucleoprotein domain family, member 4B	10p15.3				-0.312	0.00156	0.0217				1
LARP7	La ribonucleoprotein domain family, member 7	4q25				-0.33	0.00935	0.05515				1
LARS	In multiple Geneids					-0.34	0.002975	0.02925				1
STT3A	STT3, subunit of the oligosaccharyltransferase complex, homolog A (S. cerevis	11q23.3	0.683	0.00043	0.0064							1
SUMO1	SMT3 suppressor of mif two 3 homolog 1 (S. cerevisiae)	2q33	0.683	0.00659	0.0398							1
LASS1	Data not found					-0.37875	0.003005	0.028325				1
LASS3	Data not found					0.7485	0	0.001				1
LASS4	Data not found					-0.496142857	0.002841429	0.025257143				1
LASS5	Data not found					-0.394	1.00E-04	0.0065				1
LASS6	Data not found					0.3368	0.000554	0.01046				1
TMEM38B	transmembrane protein 38B	9q31.2	0.683	0.02704	0.1127							1
LAT2	linker for activation of T cells family, member 2	7q11.23				-0.4675	0.002315	0.0259				1
LAYN	layilin	11q23.1	-0.236	0.03982	0.1451							1
LBA1	Data not found					-0.309	0.00204	0.0248				1
BOLA2	bolA homolog 2 (E. coli)	16p11.2	0.682	0.00074	0.0095							1
HNRNPA2B1	heterogeneous nuclear ribonucleoprotein A2/B1	7p15	0.682	0.00017	0.0032							1
LBX1	ladybird homeobox 1	10q24	-0.267	0.0083	0.0514	-0.398	0	0.0023				2
LBX2	ladybird homeobox 2	2p13.1							-0.579	2.47	5	1
LCA5L	Leber congenital amaurosis 5-like	21q22.2				-0.344666667	0.001423333	0.017966667				1
LCAT	lecithin-cholesterol acyltransferase	16q22.1				-0.384	0.00044	0.0122				1
LCE1A	late cornified envelope 1A	1q21.3	-0.395	0.00065	0.0086							1
LCE1B	late cornified envelope 1B	1q21.3							-0.988	2.77357	7	1
LCE1C	late cornified envelope 1C	1q21.3				0.349	5.00E-04	0.0129	-1.001	2.87583	6	2
LCE1F	late cornified envelope 1F	1q21.3	-0.26	0.01753	0.0849							1
LCE2A	late cornified envelope 2A	1q21.3				0.392	0.00303	0.0302				1
LCE3B	late cornified envelope 3B	1q21.3							-0.914	2.87167	6	1
LCE3C	late cornified envelope 3C	1q21.3							-0.871	2.304	5	1
LCE3D	late cornified envelope 3D	1q21	-1.718	0.02666	0.1118							1

LCE3E	late cornified envelope 3E	1q21.3							-0.933	2.788	5	1
LCE4A	late cornified envelope 4A	1q21.3	-0.502	4.00E-05	0.0011							1
LCE6A	late cornified envelope 6A	1q21.3				0.362	0.00016	0.008				1
LCK	lymphocyte-specific protein tyrosine kinase	1p34.3				0.34	0.0376	0.1247				1
HSP90AB1	heat shock protein 90kDa alpha (cytosolic), class B member 1	6p12	0.682	0.00038	0.0059							1
LCMT2	leucine carboxyl methyltransferase 2	15q15.3	-0.552	0.00252	0.0225							1
LCN10	lipocalin 10	9q34.3				-0.337	0.01357	0.068				1
LCN6	In multiple Geneids		-0.361	0.00623	0.0424							1
LCN9	lipocalin 9	9q34.3	-0.213	0.04162	0.1493							1
LCOR	ligand dependent nuclear receptor corepressor	10q24				0.307	4.00E-05	0.0047				1
LCORL	ligand dependent nuclear receptor corepressor-like	4p15.31				-0.341	0.00042	0.0119				1
LCP1	lymphocyte cytosolic protein 1 (L-plastin)	13q14.3				0.32	0.01205	0.0636				1
LCP2	lymphocyte cytosolic protein 2 (SH2 domain containing leukocyte protein of 7	5q35.1				-0.3305	0.000795	0.01535				1
LDB1	LIM domain binding 1	10q24-q25	-0.343	0.03677	0.1379	-0.387333333	0.00051	0.012833333				2
LDB2	LIM domain binding 2	4p16							-1.045	2.91	6	1
LDB3	LIM domain binding 3	10q22.3-q23.2	-0.484	0.02934	0.1188	-0.367	5.00E-05	0.0042				2
LDHA	lactate dehydrogenase A	11p15.4				-0.422	0.00086	0.0164				1
LDHC	lactate dehydrogenase C	11p15.1				-0.301	0.00695	0.0469				1
LDHD	lactate dehydrogenase D	16q23.1	-0.565	0.01382	0.0725							1
LDLR	low density lipoprotein receptor	19p13.2				-0.3785	0.012698333	0.0603				1
LDLRAD2	low density lipoprotein receptor class A domain containing 2	1p36.12				-0.448	0.00288	0.0294				1
LDLRAD3	low density lipoprotein receptor class A domain containing 3	11p13				0.7	7.00E-05	0.0058				1
LDLRAP1	low density lipoprotein receptor adaptor protein 1	1p36-p35				-0.329	0.00717	0.04715				1
LDOC1	leucine zipper, down-regulated in cancer 1	Xq27	-1.165	0.00017	0.0033							1
LDOC1L	In multiple Geneids					-0.3915	0.000915	0.01655				1
LEAP2	liver expressed antimicrobial peptide 2	5q31.1				-0.38	0.00052	0.0131				1
LEAP-2	Data not found								-1.157	3.656	5	1
LECT1	leukocyte cell derived chemotaxin 1	13q14.3	-0.217	0.02821	0.1158							1
LECT2	leukocyte cell-derived chemotaxin 2	5q31.1							-0.916	2.872	5	1
LEKR1	leucine, glutamate and lysine rich 1	3q25.31				0.363142857	0.002177143	0.022371429				1
LELP1	late cornified envelope-like proline-rich 1	1q21.3	-0.276	0.00807	0.0505							1
LEMD1	In multiple Geneids								-0.743	2.946	5	1
LENEP	lens epithelial protein	1q22	-0.252	0.02236	0.0996							1
LENG1	leukocyte receptor cluster (LRC) member 1	19q13.4				-0.351	0.00209	0.0251				1
LENG12	Data not found								-0.676	2.73833	6	1
LENG8	In multiple Geneids					-0.322	0.002455	0.02575				1
LEO1	Leo1, Paf1/RNA polymerase II complex component, homolog (S. cerevisiae)	15q21.2				-0.372	0.02304	0.09025				1
C5orf30	chromosome 5 open reading frame 30	5q21.1	0.681	0.04006	0.1457							1
LEPREL1	leprecan-like 1	3q28				0.331	0.002805	0.0268				1
FBXO25	F-box protein 25	8p23.3	0.681	0.02613	0.1104	0.385	0.01757	0.079				2
LFNG	LFNG O-fucosylpeptide 3-beta-N-acetylglucosaminyltransferase	7p22.2	0.681	0.0189	0.0895							1
NHLRC3	NHL repeat containing 3	13q13.3	0.681	0.00072	0.0093	0.356	0.00759	0.0492				2
LEREPO4	Data not found								-0.74	2.542	5	1
LETM1	leucine zipper-EF-hand containing transmembrane protein 1	4p16.3	-0.504	0.00312	0.0263	-0.42675	0.002585	0.022225				2
LETM2	leucine zipper-EF-hand containing transmembrane protein 2	8p11.23							-0.887	2.9225	8	1
LETMD1	LETM1 domain containing 1	12q13.12				-0.349	0.00241	0.0269				1
NUFIP1	nuclear fragile X mental retardation protein interacting protein 1	13q14	0.681	0.00056	0.0078							1
ACP6	acid phosphatase 6, lysophosphatidic	1q21	0.68	6.00E-05	0.0014							1
LGALS13	lectin, galactoside-binding, soluble, 13	19q13.1							-0.775	2.66	5	1
MAPRE2	microtubule-associated protein, RP/EB family, member 2	18q12.1	0.68	0.00058	0.008				-0.681	2.564	5	2
SCAMP5	secretory carrier membrane protein 5	15q24.2	0.68	0.02946	0.1192							1
STRA6	stimulated by retinoic acid gene 6 homolog (mouse)	15q24.1	0.68	0.0046	0.0345							1
LGALS7	lectin, galactoside-binding, soluble, 7	19q13.2	-2.576	0	1.00E-04				0.914	2.77857	7	2
CEP72	centrosomal protein 72kDa	5p15.33	0.679	0.00274	0.024	0.3285	0.012615	0.0642				2
RAB8B	RAB8B, member RAS oncogene family	15q22.2	0.679	0.00204	0.0194							1
LGALS9B	lectin, galactoside-binding, soluble, 9B	17p11.2				-0.49375	0.0026525	0.024125				1
LGALS9C	lectin, galactoside-binding, soluble, 9C	17p11.2	-0.794	0.01043	0.0603	-0.4995	0.00334	0.031				2
LGI1	leucine-rich, glioma inactivated 1	10q24	-0.48	0.03144	0.1244							1
TFEC	transcription factor EC	7q31.2	0.679	0.02125	0.0967	0.334	0.00013	0.0074	-0.953	3.125	8	3
LGI3	leucine-rich repeat LGI family, member 3	8p21.3	-1.261	1.00E-05	3.00E-04							1
LGI4	leucine-rich repeat LGI family, member 4	19q13.12							0.842	2.4625	6	1
LGMN	legumain	14q32.1				-0.3495	0.001925	0.0224				1
TPM3P9	Data not found		0.679	0.00028	0.0046							1
AHI1	Abelson helper integration site 1	6q23.3	0.678	0.00093	0.0111	0.374	0.03402	0.1172				2
LGR8	Data not found								-0.909	3.16667	6	1
ATAT1	alpha tubulin acetyltransferase 1	6p21.33	0.678	0.01511	0.0769							1
LHB	luteinizing hormone beta polypeptide	19q13.32							0.63	2.425	6	1
LHFP	lipoma HMGIC fusion partner	13q12				0.3156	0.008866	0.04924				1
LHFPL1	In multiple Geneids					0.804	4.00E-05	0.0047	-0.97	2.75429	7	2

SLC25A15	solute carrier family 25 (mitochondrial carrier; ornithine transporter) member	13q14	0.678	0.00322	0.027							1
LHFPL4	In multiple Geneids		-0.239	0.03631	0.1368	-0.367333333	0.001593333	0.0183				2
LHPP	phospholysine phosphohistidine inorganic pyrophosphate phosphatase	10q26.13	-0.564	0.00081	0.0101	-0.345	0.00224	0.0259				2
LHX1	LIM homeobox 1	17q12							-0.818	2.508	5	1
LHX2	LIM homeobox 2	9q33.3							0.693	3.043	5	1
LHX3	LIM homeobox 3	9q34.3	-0.322	0.01044	0.0603	-0.3395	0.00283	0.02875				2
LHX4	LIM homeobox 4	1q25.2	-0.251	0.03407	0.1309							1
LHX9	LIM homeobox 9	1q31.1							0.802	2.69	6	1
LIAS	lipoic acid synthetase	4p14	-0.68	1.00E-04	0.0022	-0.39	0.00505	0.0394				2
TMCO1	transmembrane and coiled-coil domains 1	1q22-q25	0.677	0.00042	0.0063							1
LIFR	leukemia inhibitory factor receptor alpha	5p13-p12				0.383176471	0.006024118	0.039364706				1
LIG1	ligase I, DNA, ATP-dependent	19q13.2-q13.3				-0.357142857	0.00418	0.032014286				1
LILRA1	leukocyte immunoglobulin-like receptor, subfamily A (with TM domain), mem	19q13.4							-1.018	2.902	5	1
LILRA3	leukocyte immunoglobulin-like receptor, subfamily A (without TM domain), m	19q13.4				1.72	1.00E-05	0.0024				1
LILRA4	leukocyte immunoglobulin-like receptor, subfamily A (with TM domain), mem	19q13.4	-0.24	0.03766	0.14							1
GGT7	gamma-glutamyltransferase 7	20q11.22	0.676	0.00087	0.0106							1
MARC2	mitochondrial amidoxime reducing component 2	1q41	0.676	0.01989	0.0923							1
LIM2	lens intrinsic membrane protein 2, 19kDa	19q13.4							0.89	3.41143	7	1
OLFML3	olfactomedin-like 3	1p13.2	0.676	0.02542	0.1084							1
LIMCH1	LIM and calponin homology domains 1	4p13	-0.567	0.01531	0.0777	-0.33125	0.0008325	0.0143				2
TRABD2A	Data not found		0.676	0.0071	0.0463							1
LIMD1-AS1	Data not found		-1.104	0.00386	0.0305							1
EMR2	egf-like module containing, mucin-like, hormone receptor-like 2	19p13.1	0.675	0.01689	0.0829							1
LIMK2	LIM domain kinase 2	22q12.2	-0.668	0	1.00E-04	-0.409357143	0.00151	0.014271429				2
NGRN	neugrin, neurite outgrowth associated	15q26.1	0.675	0.00086	0.0106							1
LIMS2	In multiple Geneids					-0.312	1.00E-05	0.0033				1
SSR2	signal sequence receptor, beta (translocon-associated protein beta)	1q21-q23	0.675	0.00076	0.0097							1
LIMS3-LOC4408	LIMS3-LOC440895 readthrough	2q13				-0.314	0.00281	0.0291				1
LIN28	Data not found					-0.406833333	0.012943333	0.056733333				1
TMEM229B	transmembrane protein 229B	14q24.1	0.675	0.04366	0.1537							1
LIN54	lin-54 homolog (C. elegans)	4q21.22	-0.469	0.00386	0.0305	-0.371	0.003915	0.032325				2
LIN7A	lin-7 homolog A (C. elegans)	12q21				0.437	0.0032	0.031				1
LIN7B	lin-7 homolog B (C. elegans)	19q13.3	-0.335	0.00551	0.039	-0.503	0.00053	0.0132				2
DEK	DEK oncogene	6p22.3	0.674	0.00066	0.0088							1
PDXDC1	pyridoxal-dependent decarboxylase domain containing 1	16p13.11	0.674	5.00E-05	0.0014							1
LINC00087	long intergenic non-protein coding RNA 87	Xq26.3	-0.367	0.00629	0.0427							1
TEAD4	TEA domain family member 4	12p13.3-p13.2	0.674	9.00E-05	0.0021							1
LINC00173	long intergenic non-protein coding RNA 173	12q24.22	-0.415	0.00288	0.0248							1
LINC00272	long intergenic non-protein coding RNA 272	1q25.3	-0.366	0.00174	0.0174							1
LINC00304	long intergenic non-protein coding RNA 304	16q24.3	-0.243	0.03682	0.1381							1
LINC00305	long intergenic non-protein coding RNA 305	18q22.1	-0.241	0.00336	0.0277							1
LINC00315	long intergenic non-protein coding RNA 315	21q22.3	-0.266	0.01667	0.0821							1
LINC00341	long intergenic non-protein coding RNA 341	14q32.13	-0.372	0.0123	0.0672							1
TMEM177	transmembrane protein 177	2q14.2	0.674	0.01095	0.0621							1
TYRP1	tyrosinase-related protein 1	9p23	0.674	0.02785	0.1148				-0.762	2.94833	6	2
LINC00474	long intergenic non-protein coding RNA 474	9q31.3	-0.265	0.01647	0.0814							1
LINC00476	long intergenic non-protein coding RNA 476	9q22.32	-0.296	0.03652	0.1374							1
LINC00478	long intergenic non-protein coding RNA 478	21q21.1	-0.799	0.00692	0.0455							1
DNAJB11	DnaJ (Hsp40) homolog, subfamily B, member 11	3q27.3	0.673	0.00073	0.0094							1
LINC00521	long intergenic non-protein coding RNA 521	14q32.12	-0.254	0.03053	0.1222							1
FAM3C	family with sequence similarity 3, member C	7q31	0.673	0.00614	0.042				-0.92	2.91714	7	2
LINC00634	Data not found		-0.212	0.04199	0.1501							1
TTC33	tetratricopeptide repeat domain 33	5p13.1	0.673	0.00128	0.0139	0.4065	0.0177825	0.073925				2
ZNF792	zinc finger protein 792	19q13.11	0.673	0.00026	0.0044							1
LINC00851	Data not found		-0.282	0.00342	0.0281							1
LINC00852	Data not found		-0.396	0.01326	0.0704							1
LINC00885	Data not found		-0.798	0	1.00E-04							1
LINC00896	Data not found		-0.334	0.01364	0.0718							1
LINC00937	Data not found		-0.281	0.0196	0.0914							1
LINGO2	leucine rich repeat and Ig domain containing 2	9p21.2	-0.503	0.02561	0.109	-0.3205	0.006505	0.0416				2
LINGO3	In multiple Geneids					-0.438	0.00193	0.022233333				1
LINS1	Data not found					0.348	0.00456	0.0373				1
NCOA7	nuclear receptor coactivator 7	6q22.32	0.672	0.0317	0.125							1
LIPC	lipase, hepatic	15q21-q23				-0.397	0.00137	0.0204				1
LIPE	lipase, hormone-sensitive	19q13.2	-0.303	0.03238	0.1267	-0.337	0.001615	0.022				2
LIPF	lipase, gastric	10q23.31							-0.65	2.66625	8	1
PDE7A	phosphodiesterase 7A	8q13	0.672	0.0075	0.0481							1
LIPI	In multiple Geneids								-1.06	3.19375	8	1
LIPJ	lipase, family member J	10q23.31	-0.217	0.0261	0.1103							1

LIPN	lipase, family member N	10q23.31	-0.439	0.00755	0.0483							1
TMEM87B	transmembrane protein 87B	2q13	0.672	0.0058	0.0404							1
ZBTB10	zinc finger and BTB domain containing 10	8q13-q21.1	0.672	0.0011	0.0125							1
LIX1	Lix1 homolog (chicken)	5q15	-0.224	0.03628	0.1367							1
LIX1L	Lix1 homolog (mouse)-like	1q21.1							-0.785	2.777	5	1
LL22NC01-81G9	Data not found								-0.811	2.64	5	1
LLGL1	lethal giant larvae homolog 1 (Drosophila)	17p11.2	-0.598	0.00127	0.0139							1
ACP2	acid phosphatase 2, lysosomal	11p11.2	0.671	0.00165	0.0167							1
LMAN1	lectin, mannose-binding, 1	18q21.3-q22				-0.435	0.00088	0.0166				1
LMAN1L	In multiple Geneids		-0.22	0.03899	0.1431							1
LMAN2	lectin, mannose-binding 2	5q35.3	-0.319	0.03297	0.1282	-0.356	0.002555	0.0268				2
LMBR1L	limb region 1 homolog (mouse)-like	12q13.12				-0.3342	0.000224	0.00844				1
LMBRD1	LMBR1 domain containing 1	6q13	-0.446	0.02421	0.1048							1
LMBRD2	LMBR1 domain containing 2	5p13.2				0.39	0.01031	0.049033333				1
LMF1	lipase maturation factor 1	16p13.3	-0.609	0.01752	0.0849	-0.469142857	0.01015	0.054307143				2
LMF2	lipase maturation factor 2	22q13.33	-0.393	0.00539	0.0385							1
LMNB1	lamin B1	5q23.2				-0.363125	0.002275	0.022925				1
LMNB2	In multiple Geneids					-0.5	0.005072	0.03352				1
LMO1	LIM domain only 1 (rhombotin 1)	11p15	-0.275	0.01723	0.0839							1
LMO3	In multiple Geneids					0.3626	0.000826	0.01456	-0.882	3.185	5.75	2
LMO7	LIM domain 7	13q22.2				0.324	0.011113333	0.060533333				1
LMOD3	leiomodin 3 (fetal)	3p14.1							-0.682	2.468	5	1
LMTK3	lemur tyrosine kinase 3	19q13.33				-0.39	0.00961	0.0444				1
LMX1A	LIM homeobox transcription factor 1, alpha	1q24.1				0.32225	0.0039425	0.02665				1
LMX1B	LIM homeobox transcription factor 1, beta	9q33.3				-0.351	0.00051	0.013				1
LNPEP	leucyl/cystinyl aminopeptidase	5q15	-0.586	0.01356	0.0715							1
LNX1	ligand of numb-protein X 1, E3 ubiquitin protein ligase	4q12	-1.803	0	0							1
LNX2	In multiple Geneids					0.441	0.00112	0.0186				1
LOC100093631	general transcription factor II, i, pseudogene	7q11.23				-0.3205	0.007845	0.0463				1
LOC100101115	Data not found					-0.5385	0.0169225	0.074875				1
LOC100101116	Data not found					-0.502904762	0.021344286	0.085961905				1
LOC100101117	Data not found					-0.513090909	0.02423	0.091718182				1
LOC100101118	Data not found					-0.5232	0.024758	0.0959				1
LOC100101120	Data not found					-0.5237	0.018577	0.07932				1
LOC100124692	maltase-glucoamylase (alpha-glucosidase) pseudogene	7q34				2.103	0	0				1
LOC100128191	uncharacterized LOC100128191	12q23.1				-0.3	0.00011	0.0068				1
LOC100128288	uncharacterized LOC100128288	17p13.1				-0.364	0.00431	0.0362				1
LOC100128573	uncharacterized LOC100128573	19p13.2				-0.454	6.00E-05	0.0054				1
LOC100128788	uncharacterized LOC100128788	16p13.3				-0.397	0.001645	0.02205				1
CCT3	chaperonin containing TCP1, subunit 3 (gamma)	1q23	0.671	0.00247	0.0222							1
LOC100128818	LPEQ6126	22q13.31	-0.256	0.01122	0.0633							1
LOC100129033	QIQN5815	6p25.1	-0.516	0.00258	0.0229							1
LOC100129581	uncharacterized LOC100129581	2p25.1	-0.35	0.00087	0.0106							1
LOC100129637	uncharacterized LOC100129637	16q24.2				-0.333	0.00612	0.04325				1
MICA	In multiple Geneids		0.671	0.00177	0.0176							1
LOC100129785	uncharacterized LOC100129785	9q34.11	-0.278	0.04254	0.1514							1
LOC100130015	5-hydroxyisourate hydrolase pseudogene	16q24.3				-0.509	0.005055	0.03535				1
LOC100130156	uncharacterized LOC100130156	20q13.31	-0.25	0.01104	0.0625							1
LOC100130264	uncharacterized LOC100130264	20p11.23				0.369	0.00279	0.0289				1
LOC100130311	Data not found					-0.447	0.00322	0.0311				1
LOC100130331	POTE ankyrin domain family, member F pseudogene	1q43				0.3125	0.00209	0.02445				1
CENPO	centromere protein O	2p23.3	0.67	0.00692	0.0455							1
LOC100130581	uncharacterized LOC100130581	17q21.31				-0.3345	0.002385	0.026				1
LOC100130916	HSAL5836	9q22.33	-0.303	0.01146	0.0641							1
LOC100130924	uncharacterized LOC100130924	3p25.2	-0.29	0.02881	0.1174							1
LOC100130932	small nuclear ribonucleoprotein polypeptide G pseudogene	19p13.12				-0.315	2.00E-04	0.0088				1
LOC100130987	uncharacterized LOC100130987	11q13.2				-0.344	0.01141	0.0616				1
LOC100130988	Data not found					0.375333333	0.019361111	0.078133333				1
LOC100131232	uncharacterized LOC100131232	11q13.1	-0.434	1.00E-05	5.00E-04							1
LOC100131510	uncharacterized LOC100131510	2p23.3	-0.506	0.00034	0.0054							1
LOC100131691	uncharacterized LOC100131691	19q13.43				0.314	0.00436	0.0364				1
HSP90AA2	heat shock protein 90kDa alpha (cytosolic), class A member 2	11p14.1	0.67	0.00414	0.0321							1
LOC100131897	Data not found					-0.351666667	0.0064	0.043233333				1
LOC100131998	Data not found					-0.362	0.00032	0.00995				1
LOC100132167	uncharacterized LOC100132167	9p12	-0.695333333	0.003106667	0.0199							1
LOC100132288	Data not found					-0.4825	0.000415	0.010375				1
LOC100132354	uncharacterized LOC100132354	6p21.1				0.34	0.02761	0.1033				1
SMAD6	SMAD family member 6	15q21-q22	0.67	0.00015	0.003							1
USPL1	ubiquitin specific peptidase like 1	13q12-q14	0.67	4.00E-04	0.0061							1

LOC100133545	Data not found					-0.448	0.00027	0.01				1
LOC100133612	uncharacterized LOC100133612	1p36.32				-0.343	0.00462	0.0376				1
LOC100134368	uncharacterized LOC100134368	16p13.3				-0.311	0.04555	0.1404				1
AGAP1	ArfGAP with GTPase domain, ankyrin repeat and PH domain 1	2q37	0.669	0.00033	0.0053							1
LOC100170229	Data not found					-0.453	0.00037	0.0113				1
LOC100170939	glucuronidase, beta pseudogene	5q13				-0.394	0.004505	0.03645				1
LOC100188947	uncharacterized LOC100188947	10q23.32				-0.304	0.01048	0.0587				1
LOC100192378	uncharacterized LOC100192378	8q21.11				0.388	0.005624286	0.033228571				1
LOC100216001	uncharacterized LOC100216001	10p15.1				0.398	0.00959	0.0559				1
LOC100216545	uncharacterized LOC100216545	7q22.3				-0.31	0.00278	0.0289				1
LOC100233156	tektin 4 pseudogene	4p16.3	-0.955	0.00016	0.0032							1
LOC100268168	uncharacterized LOC100268168	5q35.1				-0.4135	0.00092	0.01565				1
LOC100272228	uncharacterized LOC100272228					0.3298	0.004682	0.03472				1
LOC100286844	uncharacterized LOC100286844	12q13.13				-0.412	1.00E-04	0.0066				1
CLSPN	claspin	1p34.2	0.669	0.00658	0.04095							1
LOC100287792	uncharacterized LOC100287792	20q11.23	-0.374	0.01148	0.0641							1
FANCL	Fanconi anemia, complementation group L	2p16.1	0.669	0.00083	0.0103	0.374714286	0.004371429	0.032014286				2
LOC100289341	uncharacterized LOC100289341	9q34.3				-0.302	0.01636	0.0758				1
LOC100505502	putative UPF0607 protein FLJ37424-like	10p11.22	-0.465	0.00091	0.011							1
LSM2	LSM2 homolog, U6 small nuclear RNA associated (S. cerevisiae)	6p21.3	0.669	0.01145	0.0641							1
LOC121838	Data not found					0.345	0.00355	0.0328				1
LOC126075	Data not found					-0.4595	0.002145	0.0253				1
LOC126661	Data not found					-0.559	0.00194	0.0242				1
LOC129531	Data not found								-0.673	2.6	7	1
LOC132321	Data not found								-1.015	2.836	5	1
LOC132969	similar to CG15920-PB, isoform B	4p16.3							1.145	3.93	6	1
LOC136263	Data not found								-0.628	2.70667	6	1
LOC144776	Data not found					0.305	0.011135	0.0549				1
LOC145820	uncharacterized LOC145820	15q26.2				0.342625	0.00525875	0.039275				1
LOC146336	uncharacterized LOC146336	16p13.3				-0.515	0.0045	0.0371				1
LOC147727	uncharacterized LOC147727	19p13.2				-0.464	0.00065	0.0145				1
LOC148696	uncharacterized LOC148696	1q32.2							0.66	2.76333	6	1
LOC149134	uncharacterized LOC149134	1q44				-0.488	0.00037	0.0113				1
LOC149373	uncharacterized LOC149373	1q42.2	-0.532	0.0028	0.0243							1
LOC150185	uncharacterized LOC150185	22q11.21				-0.397	3.00E-05	0.0044				1
LOC150527	Data not found					-0.337	0.00129	0.0198				1
LOC150568	uncharacterized LOC150568	2q12.1				0.340833333	0.001751667	0.018316667				1
LOC150622	uncharacterized LOC150622	2p25.2				0.391	0.001255	0.0165				1
LOC150763	Data not found								0.83	3.295	6	1
MICALCL	MICAL C-terminal like	11p15.3	0.669	0.00823	0.0512							1
LOC153328	Data not found					-0.304	0.00089	0.0168				1
RUVBL1	RuvB-like 1 (E. coli)	3q21	0.669	0.01513	0.077							1
LOC154872	uncharacterized protein LOC154872	7q31.33							-0.728	2.755	6	1
LOC154907	Data not found								-1.041	3.245	6	1
LOC158318	Data not found								0.828	3.215	8	1
LOC158381	Data not found					-0.302	0.003025	0.02355				1
LOC196264	Data not found								-0.925	2.65167	6	1
LOC197350	Data not found								1.1	3.95333	6	1
LOC198437	Data not found								0.97	3.68857	7	1
LOC201651	arylacetamide deacetylase (esterase) pseudogene	3q25.1				0.378	0.00052	0.0131				1
LOC202051	Data not found					-0.466333333	0.000143333	0.006533333				1
LOC202181	chromosome 5 open reading frame 25 pseudogene	5q35.3				-0.3812	0.005192	0.03012				1
LOC219347	uncharacterized LOC219347	10q23.1				-0.372	2.00E-05	0.0035				1
LRRC59	leucine rich repeat containing 59	17q21.33	0.668	0.00082	0.0102							1
LOC221442	adenylate cyclase 10 (soluble) pseudogene	6p21.1				0.316	0.02011	0.0855				1
LOC222171	Data not found								-0.656	2.526	5	1
LOC253012	Data not found								-0.891	3.3425	8	1
LOC254028	uncharacterized LOC254028	14q11.2							-0.754	3.305	6	1
LOC257358	uncharacterized LOC257358	5q35.1	-0.18	0.03729	0.1392							1
LOC283050	uncharacterized LOC283050	10q22.3				-0.358333333	0.001063333	0.0162				1
LOC283340	similar to ribosomal protein L13a	10q24.1							0.9	3.56833	6	1
LOC283922	pyruvate dehydrogenase phosphatase regulatory subunit pseudogene	16q23.1				-0.479555556	0.006504444	0.037455556				1
LOC283999	Data not found					-0.372	0.00175	0.023				1
LOC284009	uncharacterized LOC284009	17p13.3				-0.349	0.016385	0.07445				1
LOC284276	uncharacterized LOC284276	18q23				-0.348666667	0.006136667	0.0426				1
LOC284297	Data not found					-0.361333333	0.005111667	0.038283333				1
LOC284379	solute carrier family 7 (cationic amino acid transporter, y+ system), member 3	19q13.42	-0.386	0.00499	0.0365	-0.362	0.00324	0.0312				2
LOC284395	uncharacterized LOC284395	19q12							0.782	3.163	9	1
LOC284422	Data not found					-0.4405	0.01696	0.0626				1

LOC284440	uncharacterized LOC284440	19p13.11				-0.431	2.00E-05	0.0035				1
LOC284632	uncharacterized LOC284632	1p36.11				-0.405	0.00258	0.02395				1
LOC284661	uncharacterized LOC284661	1p36.32				-0.3	0.00082	0.0161				1
PPT1	palmitoyl-protein thioesterase 1	1p32	0.668	0.01144	0.064							1
LOC284788	uncharacterized LOC284788	20p11.21				0.408	0.00164	0.0223				1
LOC284837	uncharacterized LOC284837	21q22.3	-0.273	0.02449	0.1056							1
LOC284861	uncharacterized LOC284861	22q11.21							0.819	2.76	5	1
LOC284900	Data not found					-0.335	0.0021	0.020666667				1
LOC285033	uncharacterized LOC285033	2q11.2							0.517	2.826	5	1
LOC285074	anaphase promoting complex subunit 1 pseudogene	2p11.2				-0.307	0.00038	0.0114				1
LOC285095	uncharacterized LOC285095	2q37.3							0.994	3.826	5	1
LOC285375	uncharacterized LOC285375	3p25.1				-0.389	0.000186667	0.0074				1
LOC285456	Data not found					-0.337	0.00224625	0.0214875				1
LOC285501	uncharacterized LOC285501	4q34.3				-0.382	0.01536	0.0731				1
LOC285679	Data not found					-0.518	0.00029	0.0102				1
LOC285692	uncharacterized LOC285692	5p15.31				0.384942857	0.005464	0.031				1
LOC285696	uncharacterized LOC285696	5p15.1				0.347	0.00691	0.0467				1
LOC285735	Data not found								-0.885	3.096	5	1
LOC285768	uncharacterized LOC285768	6p25.3				-0.465	0.00355	0.0327				1
LOC285780	Data not found					-0.361	0.00126	0.0196				1
LOC285954	Data not found					0.3922	0.00113	0.0164				1
LOC286076	Data not found								1.091	3.11667	6	1
LOC286094	uncharacterized LOC286094	8q24.22				0.335	0.005885	0.03935				1
LOC286359	uncharacterized LOC286359	9q22.33	-0.271	0.04469	0.1561							1
LOC338588	uncharacterized LOC338588	10p15.1				0.398	0.00959	0.0559				1
LOC338651	uncharacterized LOC338651	11p15.5				-0.323	0.00416	0.0355				1
LOC339524	uncharacterized LOC339524	1p22.3	-0.312	0.0313	0.1242							1
LOC339568	uncharacterized LOC339568	20q12				0.304	0.0041	0.0352				1
LOC339788	uncharacterized LOC339788	2p25.1				0.3332	0.002796	0.02842				1
LOC342346	Data not found					-0.446	0.005422857	0.038057143				1
LOC342933	Data not found								0.968	3.506	5	1
LOC345643	Data not found								-0.863	2.794	5	1
LOC347273	Data not found								-0.877	3.29125	8	1
LOC387601	Data not found								-0.85	3.002	5	1
CCL4L1	chemokine (C-C motif) ligand 4-like 1	17q12	0.667	7.00E-04	0.0091							1
LOC388152	uncharacterized LOC388152	15q25.2	-0.742	0.001615	0.0164							1
LOC388199	Data not found					-0.381	0.00715	0.0476				1
LOC388503	Data not found								-0.921	2.87167	6	1
LOC388692	uncharacterized LOC388692	1q21.2							0.605	3.20357	7	1
LOC388910	Data not found					-0.475	0.000275	0.0081				1
LOC389203	Data not found					-0.394333333	0.000586667	0.0128				1
LOC389289	Data not found								-0.882	3.61429	7	1
LOC389429	hypothetical LOC389429	6q22.33							-1.01	2.94167	6	1
LOC389607	uncharacterized LOC389607	8p23.3							0.971	3.4075	6	1
LOC389705	chromosome 4 open reading frame 27 pseudogene	9p22.3				-0.361	0.00035	0.011				1
LOC389832	hypothetical LOC389832								0.742	3.13714	7	1
LOC389834	ankyrin repeat domain 57 pseudogene					-0.364	0.00828	0.0516				1
LOC389936	similar to proline-rich proteoglycan 2	10p14							0.593	3.00375	8	1
LOC390367	hypothetical LOC390367	12q24.33							0.997	3.75286	7	1
LOC390858	ACYL3 pseudogene	18q21.32				-0.47575	0.001175	0.0148				1
LOC391444	hypothetical LOC391444	2q21.1							0.582	2.622	5	1
LOC391859	similar to Probable Coiled-coil domain-containing protein 8	5q35.3							0.891	3.568	5	1
LOC392145	exosome component 6 pseudogene	7q36.1				-0.3425	0.003425	0.03215				1
LOC392364	chromosome 15 open reading frame 2 pseudogene	9q22.1	-0.295	0.02053	0.0944				0.769	2.602	5	2
LOC393078	uncharacterized LOC393078	7q36.3							-1.048	3.132	5	1
LOC399829	uncharacterized LOC399829	10q26.3							0.808	3.53571	7	1
LOC399959	Data not found					0.336	0.00028	0.01				1
LOC400499	uncharacterized LOC400499	16p13.13							0.556	2.64	5	1
LOC400553	uncharacterized LOC400553	16q24.2							0.581	2.421	5	1
LOC400558	uncharacterized LOC400558	16q24.3							0.924	4.38571	7	1
LOC400581	Data not found					-0.843	4.00E-05	0.0048				1
LOC400713	Data not found								0.629	2.428	5	1
LOC400794	uncharacterized LOC400794	1q23.3-q24.1				0.305	0.00282	0.0291				1
LOC400891	chromosome 14 open reading frame 166B pseudogene	22q11.21				-0.41	0.000241667	0.00845				1
LOC400927	TPTE and PTEN homologous inositol lipid phosphatase pseudogene	22q13.1				-0.3285	0.01879	0.07375				1
LOC400931	Data not found					-0.301	0.01002	0.0573				1
LOC400940	uncharacterized LOC400940	2p25.2				0.339	0.00248	0.0273				1
LOC400943	uncharacterized LOC400943	2p25.1	-0.351	0.00062	0.0084							1
LOC400968	uncharacterized LOC400968	15q11.2	-0.5	0.00938	0.056							1

LOC401052	uncharacterized LOC401052	3p25.3	-0.652	1.00E-04	0.0022							1
LOC401074	uncharacterized LOC401074	3p12.3							0.787	2.882	5	1
LOC401137	Data not found								-0.764	2.952	5	1
LOC401152	Data not found								-0.878	2.78833	6	1
LOC401286	uncharacterized LOC401286	6q27	-0.964	1.00E-05	3.00E-04							1
LOC401399	Data not found					-0.384	0.00045	0.0124				1
LOC401410	uncharacterized LOC401410	7q34							-0.918	3.55571	7	1
LOC401557	uncharacterized LOC401557	9q34.3	-0.22	0.03361	0.1297							1
LOC401629	Data not found					-0.373	0.01617	0.07505				1
LOC401630	Data not found					-0.373	0.01617	0.07505				1
LOC401845	similar to Ig heavy chain V-II region SESS precursor	16p11.2							0.708	2.486	5	1
LOC402560	hypothetical LOC402560	7q21.11							0.957	3.40286	7	1
LOC402643	Data not found								-0.797	2.915	6	1
LOC415056	uncharacterized LOC415056	9p13				-0.459333333	1.67E-05	0.0032				1
LOC440015	similar to double homeobox, 4	3p12.3							0.787	2.882	5	1
LOC440040	glutamate receptor, metabotropic 5 pseudogene	11p11.12				0.603	1.00E-05	0.003				1
LOC440082	uncharacterized LOC440082	12p13.2							-0.902	3.40125	8	1
LOC440087	Data not found								-0.885	2.965	6	1
LOC440093	Data not found								0.955	3.465	6	1
LOC440157	uncharacterized LOC440157	14q11.1							0.655	3.25857	7	1
LOC440287	hypothetical LOC440287	15q24.1							0.682	3.06875	8	1
LOC440330	uncharacterized LOC440330	16p13.3							0.59	3.10125	8	1
LOC440350	Data not found								1.275	2.554	5	1
LOC440354	smg-1 homolog, phosphatidylinositol 3-kinase-related kinase (C. elegans) pseu	16p11.2				-0.315	0.00062	0.0142	0.95	3.248	5	2
LOC440461	Rho GTPase activating protein 27 pseudogene	17q24.2	-0.285	0.00975	0.0575							1
LOC440498	Data not found					-0.451	0.00057	0.0136				1
LOC440570	uncharacterized LOC440570	1p36.13							0.68	2.89667	6	1
LOC440607	Fc-gamma receptor I B2	1p11.2							-0.975	3.064	5	1
LOC440792	proline dehydrogenase (oxidase) 1 pseudogene	22q11.21							0.629	2.566	5	1
LOC440895	two pore channel 3 pseudogene	2q13							0.724	2.86143	7	1
PAIP1	poly(A) binding protein interacting protein 1	5p12	0.6665	0.0047	0.03245	0.411	0.01093	0.053875				2
LOC440934	uncharacterized LOC440934	2q36.1							0.938	2.826	5	1
LOC441046	Data not found					-0.461	0.00132	0.02				1
LOC441087	uncharacterized LOC441087	5q13.3							-0.843	3.02611	9	1
LOC441178	uncharacterized LOC441178	6q27	-4.009	0	0							1
LOC441193	similar to zinc finger protein 469	7p22.1							-0.577	2.337	5	1
LOC441204	uncharacterized LOC441204	7p15.2				0.3135	4.00E-04	0.0115				1
LOC441239	uncharacterized LOC441239	7q11.21	-0.211	0.02533	0.1081							1
LOC441251	Data not found								1.247	3.13286	7	1
LOC441755	similar to Kinase suppressor of ras-1 (Kinase suppressor of ras) (mKSR1) (Hb p	16p11.2							0.522	2.53	7	1
LOC441864	Data not found					-0.457	0.00132	0.02				1
LOC441956	uncharacterized LOC441956	21q11.2							0.756	3.53944	9	1
LOC442128	hypothetical LOC442128	5p15.33							0.873	2.606	5	1
LOC442423	similar to Ig kappa chain V-I region HK101 precursor	9q12							0.68	2.71	6	1
LOC442459	X-ray repair complementing defective repair pseudogene	Xq22.1				0.3188	0.002326	0.02332				1
LOC442501	similar to zinc finger protein 660	7p22.1							0.764	3.1125	8	1
LOC474170	Data not found								0.743	3.096	5	1
LOC492311	Data not found								-0.756	2.798	5	1
LOC493869	Data not found								-0.858	2.68714	7	1
FAM50A	family with sequence similarity 50, member A	Xq28	0.666	0	2.00E-04							1
LOC497190	Data not found								0.638	2.532	5	1
LOC504188	Data not found								0.925	3.436	5	1
LOC51057	Data not found								-0.909	3.194	5	1
LOC51149	Data not found								0.978	4.12	7	1
LOC54103	Data not found								-0.84	3.21111	9	1
LOC541473	FK506 binding protein 6, 36kDa pseudogene	7q11.23							1.05	3.129645	6.5	1
LOC553158	Data not found					-0.43652	0.0026028	0.0177				1
LOC554174	uncharacterized LOC554174	22q13.31	-0.294	0.04887	0.1647							1
LOC554202	Data not found								-1.135	3.73167	6	1
FKBP11	FK506 binding protein 11, 19 kDa	12q13.12	0.666	0.00127	0.0139							1
LOC554249	uncharacterized LOC554249	9q12	-0.68525	0.000505	0.006675				0.85	2.974285	6.5	2
LOC55908	Data not found					-0.38	0.00386	0.0342	0.764	2.63167	6	2
LOC619208	Data not found								-0.758	2.266	5	1
LOC641367	cyclin Y-like 1 pseudogene	19p12	-0.244	0.01508	0.0769							1
LOC641515	uncharacterized LOC641515	1q31	-0.181	0.04903	0.1651							1
LOC642268	hypothetical protein LOC642268	2q13							0.706	2.90167	6	1
LOC642276	similar to CG3950-PA	8q24.3							1.536	3.772	5	1
LOC642295	hypothetical LOC642295	8q24.3							1.536	3.772	5	1
LOC642333	similar to M-phase phosphoprotein, mpp8	12p13.31							-0.82	3.2975	8	1

LOC642358	similar to chromosome Y open reading frame 16	Yp11.2							0.587	2.75	6	1
LOC642363	hypothetical protein LOC642363	13q31.2							0.945	3.09714	7	1
LOC642391	similar to chromosome Y open reading frame 16	Yp11.2							0.746	3.38083	6	1
LOC642422	uncharacterized LOC642422	10p15.3							1.125	2.56333	6	1
LOC642431	similar to chromosome Y open reading frame 16	Yp11.2							0.757	3.38	6	1
LOC642494	hypothetical protein LOC642494	17q24.3							-0.792	3.20571	7	1
LOC642497	similar to Probable DNA dC->dU editing enzyme APOBEC-3B (Phorbolin-1-rela	4q33							-0.659	2.37167	6	1
LOC642547	similar to chromosome Y open reading frame 16	Yp11.2							0.878	3.734	5	1
LOC642586	similar to chromosome Y open reading frame 16	Yp11.2							0.76	3.44833	6	1
LOC642620	uncharacterized LOC642620	7q36.3							0.848	3.68167	6	1
LOC642622	hypothetical protein LOC642622	10q26.13							0.867	3.84143	7	1
LOC642633	uncharacterized LOC642633	22q11.21							1.012	3.356	5	1
LOC642670	hypothetical protein LOC642670	17p13.3							0.55	2.582	5	1
LOC642730	uncharacterized LOC642730	8q21.3							0.513	2.536	5	1
LOC642769	hypothetical protein LOC642769	22q13.31							0.821	2.796	5	1
LOC642837	similar to SR protein related family member (rsr-1)	2p25.3							0.801	3.845	6	1
LOC642852	uncharacterized LOC642852	21q22.3							0.679	2.418	5	1
LOC642891	Data not found								0.581	2.36571	7	1
LOC642922	similar to CG13731-PA	11p15.4							0.893	2.79929	7	1
KLHL24	kelch-like 24 (Drosophila)	3q27.1	0.666	0.0085	0.0522							1
LOC643002	hypothetical protein LOC643002	22q11.21							0.914	3.64143	7	1
LOC643069	hypothetical LOC643069	22q11.21							0.878	4.04286	7	1
LOC643260	hypothetical LOC643260	17q25.1							-0.93	3.00571	7	1
LOC643341	hypothetical LOC643341	4p16.2							0.64	3.14857	7	1
LOC643367	POM121 membrane glycoprotein (rat) pseudogene	5q13.2							-1.091	4.004	5	1
LOC643449	similar to hCG2041756	4p12							-0.968	2.968	5	1
LOC643464	hypothetical LOC643464	4p12							-0.768	2.575	6	1
LOC643594	similar to CG13731-PA	5p15.33							1.317	2.594	5	1
LOC643837	uncharacterized LOC643837	1p36.33				0.307	0.04398	0.1374				1
LOC643901	hypothetical LOC643901	4q12							-0.967	2.718	5	1
LOC643903	hypothetical protein LOC643903	7p15.2							-0.68	2.758	5	1
LOC643972	hypothetical LOC643972	8q22.3							-0.77	2.72667	6	1
LOC643982	hypothetical protein LOC643982	17q25.3							-0.708	2.50167	6	1
LOC644106	hypothetical protein LOC644106	Xp21.1							-0.947	2.93833	6	1
LOC644111	hypothetical LOC644111	22q13.33							0.708	2.502	5	1
LOC644145	exocyst complex component 1 pseudogene	4q12				-0.311	0.00203	0.0247				1
LOC644165	Data not found					-0.543	0.00017	0.0081				1
LOC644253	Data not found								0.852	3.158	5	1
LOC644280	similar to hCG2041260	20q13.13							1.012	2.94571	7	1
LOC644319	hypothetical protein LOC644319	8p23.3							1.129	3.818	5	1
LOC644634	UPF0627 protein ENSP00000358171-like	1q21.2							0.854	3.48167	6	1
LOC644714	uncharacterized LOC644714	3p21.31							-0.811	2.496	5	1
LOC644772	hypothetical protein LOC644772	21q22.12							-0.656	2.588	5	1
LOC644781	similar to hypothetical gene supported by AK127288; AY343901	9q13							0.696	3.13437	8	1
LOC644792	hypothetical protein LOC644792	Xq26.3							0.818	2.696	5	1
LOC644844	Data not found					-0.417	0.00056	0.0135				1
LOC644979	similar to KIAA1193 protein	19p13.3							1.067	3.525	6	1
LOC645004	hypothetical protein LOC645004	7q11.23							1.247	3.13286	7	1
LOC645116	hypothetical protein LOC645116	1q21.1							0.819	2.77	5	1
LOC645146	similar to Kinase suppressor of Ras 1	1q21.2							0.593	2.845	8	1
LOC645154	hypothetical protein LOC645154	1q21.1							0.717	2.7	6	1
LOC645196	hypothetical protein LOC645196	11p15.1							-0.987	3.43	9	1
LOC645261	PP565	5q14.3							-0.901	2.866	5	1
LOC645281	hypothetical protein LOC645281	9q33.1							-0.959	3.11286	7	1
LOC645332	Data not found								0.572	2.746	5	1
LOC645343	hypothetical protein LOC645343	Xp22.31							0.709	3.08	7	1
LOC645357	kinesin family member pseudogene	7p22.3							0.646	2.768	5	1
LOC645367	Data not found								0.732	2.54	5	1
LOC645683	Data not found								0.69	2.85	5	1
LOC645700	Data not found								0.695	3.09143	7	1
LOC645722	uncharacterized LOC645722	17q23.2							0.642	2.57357	7	1
LOC645899	Data not found								0.888	3.425	6	1
LOC645941	hypothetical LOC645941	16p13.3							0.612	2.67167	6	1
LOC646122	hypothetical protein LOC646122	15q11.2							0.738	2.67286	7	1
LOC646160	similar to cytochrome c oxidase subunit VIIIc	6p22.1							1.316	3.758	5	1
LOC646174	Data not found								0.706	2.846	5	1
LOC646190	hypothetical protein LOC646190	4q28.3							1.087	3.026	5	1
LOC646498	uncharacterized LOC646498	3p21.31				-0.4	0.00063	0.0143				1
LOC646513	VLGN1945	22q12.2	-0.299	0.00339	0.0279							1

LOC646574	hypothetical LOC646574	15q23							-0.782	2.804	5	1
LOC646638	hypothetical protein LOC646638	16q24.2							0.83	2.552	5	1
LOC646746	similar to piccolo (presynaptic cytomatrix protein)	19q13.43							0.561	2.784	5	1
PDZD3	PDZ domain containing 3	11q23.3	0.666	0.00065	0.0087							1
LOC646863	hypothetical LOC646863	2q21.1							0.582	2.622	5	1
LOC646919	hypothetical protein LOC646919	14q31.3							1.22	3.90286	7	1
LOC647163	hypothetical protein LOC647163	6p12.2							0.594	2.626	5	1
LOC647191	similar to Kinase suppressor of ras-1 (Kinase suppressor of ras) (mKSR1) (Hb p	16p11.2							0.668	3.21	6	1
LOC647205	similar to protein phosphatase 2, regulatory subunit B, beta isoform 1	16p11.2							0.574	2.82714	7	1
LOC647311	hypothetical protein LOC647311	14q32.33							0.572	2.74857	7	1
LOC648570	uncharacterized LOC648570	9p22.2							-1.098	3.522	5	1
LOC649897	similar to Ig gamma-2 chain C region								0.58	2.868	5	1
LOC651769	seven transmembrane helix receptor								-1.047	3.0725	8	1
LOC652276	potassium channel tetramerisation domain containing 5 pseudogene	16p13.3				-0.424333333	0.016181667	0.069816667				1
LOC653033	hypothetical protein LOC653033	6p21.32							0.919	3.13522	9	1
LOC653048	Data not found								0.739	2.77333	6	1
LOC653067	Data not found								1.01	3.23	5	1
LOC653084	similar to retrotransposon-like 1	Xq21.1							-0.843	2.896	5	1
LOC653107	annexin A8-like	10q11.22							0.705	2.734	5	1
LOC653121	Data not found								-0.826	2.666	5	1
LOC653147	Data not found								-0.754	3.1096	5	1
LOC653178	similar to testis specific protein, Y-linked 1	Yp11.2							0.76	3.44833	6	1
LOC653210	similar to melanoma antigen family D, 4 isoform 1	Xp11.22							0.725	2.98714	7	1
LOC653222	similar to Gamma-aminobutyric-acid receptor alpha-5 subunit precursor (GAB	15q12							0.676	2.88167	6	1
LOC653257	similar to Gamma-glutamyltranspeptidase 1 precursor (Gamma-glutamyltrans	22q11.21							0.732	2.54	5	1
LOC653261	similar to Acidic mammalian chitinase precursor (AMCase) (TSA1902)	1p13.2							-0.992	3.138	5	1
LOC653270	hypothetical LOC653270	22q11.21							0.855	3	5	1
LOC653277	similar to GM88 autoantigen isoform c	15q13.1							1.004	2.664	5	1
LOC653280	similar to testis specific protein, Y-linked 1	Yp11.2							0.587	2.75	6	1
LOC653293	similar to testis specific protein, Y-linked 1	Yp11.2							0.757	3.38	6	1
LOC653300	similar to GM88 autoantigen isoform c	15q13.1							1.079	2.428	5	1
LOC653312	similar to similar to Williams Beuren syndrome chromosome region 19	7q11.23							1.247	3.13286	7	1
LOC653333	Data not found								0.536	2.825	6	1
LOC653353	similar to forkhead box D4-like 4	9p11.2							0.717	2.946	5	1
LOC653397	similar to PDZ domain containing 1	1q21.1							0.948	3.088	5	1
LOC653427	Data not found								0.604	2.71833	6	1
LOC653456	similar to zinc finger protein 568	19q13.12							1.356	3.01	5	1
LOC653483	Data not found								-0.947	3.234	5	1
LOC653486	secretoglobin, family 1C, member 1-like	17p				-0.34	0.00875	0.0532				1
LOC653499	Data not found								0.914	2.77857	7	1
LOC653501	zinc finger protein 658 pseudogene	9p13.1				-0.506166667	0.001576667	0.013583333				1
LOC653535	similar to TP53TG3 protein	16p11.2							0.846	4.09125	8	1
LOC653550	Data not found								0.849	3.76143	7	1
LOC653551	similar to TP53TG3 protein	16p11.2							0.851	3.93857	7	1
LOC653580	similar to sperm protein associated with the nucleus, X chromosome, family n	Xq27.2							0.698	3.059	5	1
LOC653592	similar to Polypeptide N-acetylgalactosaminyltransferase 9 (Protein-UDP acety	12q24.33							0.724	2.90167	6	1
LOC653596	similar to RAN-binding protein 2-like 1 isoform 2	2q13							-0.78	3.322	5	1
LOC653622	similar to PRAME family member 8	1p36.21							-0.795	2.68667	6	1
LOC653666	similar to defensin, beta 105A	8p23.1							-0.843	3.31	7	1
LOC653667	similar to defensin, beta 106A	8p23.1							-0.843	3.31	7	1
LOC653669	similar to defensin, beta 104A	8p23.1							-1.026	3.49857	7	1
LOC653680	Data not found								1.108	2.428	5	1
LOC653715	similar to tuftelin interacting protein 11	22q12.1							-0.675	2.456	5	1
LOC653735	hypothetical LOC653735	18p11.21							0.874	3.00571	7	1
LOC653760	similar to acetyl-coenzyme A transporter	8q21.2							0.944	4.11889	9	1
LOC653786	otoancorin pseudogene	16p12.2				-0.3495	0.003125	0.02875				1
LOC653811	similar to BolA-like protein 2	16p11.2							1.043	3.45167	6	1
LOC653823	similar to FLJ40296 protein	13q21.1							0.932	3.1325	8	1
LOC653824	similar to FLJ40296 protein	13q21.1							0.96	3.11313	8	1
LOC653825	similar to FLJ40296 protein	13q21.1							0.949	3.51688	8	1
LOC653826	similar to FLJ40296 protein	13q21.1							0.979	3.14875	8	1
TPD52	tumor protein D52	8q21	0.666	0.00956	0.0566							1
LOC727677	uncharacterized LOC727677	8q24.21				0.362	0.010157143	0.052214286				1
LOC727924	uncharacterized LOC727924	15q11.2				-0.469	0.008245	0.05145				1
TRHDE	thyrotropin-releasing hormone degrading enzyme	12q15-q21	0.666	0.02853	0.1167	0.351	0.006823333	0.043766667				2
LOC728190	uncharacterized LOC728190	10q23.2				0.400333333	0.002473333	0.017966667				1
LOC728264	Data not found					-0.402666667	0.000473333	0.010833333				1
LOC728323	uncharacterized LOC728323	2q37.3				0.441	0.00741	0.0465				1
LOC728411	Data not found					0.349	0.01669	0.07175				1

LOC728448	Data not found					-0.316	0.00153	0.0215				1
LOC728554	THO complex 3 pseudogene	5q35.3				-0.379	9.00E-04	0.0168				1
LOC728661	Data not found					-0.383666667	0.00859	0.0441				1
LOC728723	uncharacterized LOC728723	5q13.3				-0.349	2.00E-05	0.0038				1
TTC13	tetratricopeptide repeat domain 13	1q42.2	0.666	0.00117	0.0131							1
LOC728989	phosphodiesterase 4D interacting protein pseudogene	1q21.1				0.339	0.00248	0.0273				1
NT5C3A	Data not found		0.665	0.005105	0.0317							1
LOC729082	Data not found					-0.314	0.03215	0.1133				1
LOC729085	Data not found					-0.407	1.00E-04	0.0065				1
LOC729461	uncharacterized LOC729461	22q11.21	-0.5465	0.014465	0.06275							1
LOC729747	Data not found					-0.5035	0.00105	0.0152				1
LOC729920	Data not found					0.350357143	0.002987857	0.024321429				1
LOC729956	Data not found					-0.339	0.00112	0.0186				1
LOC729991	Data not found					-0.379	0.000765	0.01535				1
LOC729991-MEI	Data not found					-0.433555556	0.003936667	0.029877778				1
LOC729993	Data not found					-0.3505	0.01315	0.0648				1
LOC80154	Data not found		-0.712666667	0.005393333	0.030766667							1
LOC84740	Data not found					-0.352666667	0.00254	0.023033333				1
LOC90624	Data not found								-0.676	2.60333	6	1
LOC91316	Data not found					-0.414117647	0.000867647	0.010770588				1
LOC91431	prematurely terminated mRNA decay factor-like	4q25							-0.838	3.23556	9	1
LOC92154	Data not found								0.562	2.35	6	1
LOC92196	Data not found								-1.045	2.62667	6	1
LOC92973	Data not found					-0.375	0.001305	0.01645				1
PGPEP1	In multiple Geneids		0.665	0.00063	0.0085							1
LOC96610	BMS1 homolog, ribosome assembly protein (yeast) pseudogene	22q11.22				-0.309	0.00036	0.0112				1
LOH12CR1	loss of heterozygosity, 12, chromosomal region 1	12p12				0.6345	0.001015	0.01235				1
LOH3CR2A	loss of heterozygosity, 3, chromosomal region 2, gene A								-0.957	3.488	5	1
LONP1	lon peptidase 1, mitochondrial	19p13.2				-0.414	0.0031725	0.027				1
RNU2-1	RNA, U2 small nuclear 1	17q12-q21	0.665	0.00108	0.0123							1
RPN1	ribophorin I	3q21.3	0.665	0.00044	0.0066							1
LOR	loricrin	1q21	-0.464	0.01413	0.0735							1
KIF12	kinesin family member 12	9q32	0.664	0.00012	0.0025				-0.737	3.008	5	2
LOXHD1	lipoxygenase homology domains 1	18q21.1				-0.404333333	0.008118667	0.046893333				1
MCM6	minichromosome maintenance complex component 6	2q21	0.664	0.00342	0.0281							1
PTPN18	protein tyrosine phosphatase, non-receptor type 18 (brain-derived)	2q21.1	0.664	0.0012	0.0133							1
TERC	telomerase RNA component	3q26	0.664	0.02387	0.1038							1
TM4SF18	transmembrane 4 L six family member 18	3q25.1	0.663	0.0458	0.1586							1
LPA	lipoprotein, Lp(a)	6q26				-0.331	0.00345	0.0323				1
LPAL2	In multiple Geneids								-0.915	3.002	5	1
LPAR1	lysophosphatidic acid receptor 1	9q31.3	-0.735	0.04368	0.1538							1
LPAR2	lysophosphatidic acid receptor 2	19p12				-0.468	2.00E-04	0.0067				1
LPAR3	lysophosphatidic acid receptor 3	1p22.3	-2.099	0	0	-0.335	0.00293	0.0296				2
LPAR4	lysophosphatidic acid receptor 4	Xq21.1				0.335	0.00015	0.0078				1
LPAR5	lysophosphatidic acid receptor 5	12p13.31	-0.907	0.00029	0.0048							1
LPAR6	lysophosphatidic acid receptor 6	13q14	-1.794	2.00E-05	7.00E-04							1
CALM3	calmodulin 3 (phosphorylase kinase, delta)	19q13.2-q13.3	0.662	0.00018	0.0034							1
KRTCAP2	keratinocyte associated protein 2	1q22	0.662	0	2.00E-04							1
ACSS3	acyl-CoA synthetase short-chain family member 3	12q21.31	0.661	0.0283	0.1161							1
LPHN1	latrophilin 1	19p13.2				-0.451571429	0.005202857	0.033385714				1
LPHN2	latrophilin 2	1p31.1							-0.955	3.386	5	1
ARID4B	AT rich interactive domain 4B (RBP1-like)	1q42.1-q43	0.661	0.00047	0.0068							1
LPIN1	lipin 1	2p25.1	-1.283	0.00027	0.0046							1
CIAO1	cytosolic iron-sulfur protein assembly 1	2q11.2	0.661	0.00498	0.0364							1
RNF103	ring finger protein 103	2p11.2	0.661	0.00112	0.0126							1
LPP	LIM domain containing preferred translocation partner in lipoma	3q28				0.336	0.00185	0.018890909				1
LPPR2	lipid phosphate phosphatase-related protein type 2	19p13.2				-0.56975	0.0006675	0.013				1
LRAT	lecithin retinol acyltransferase (phosphatidylcholine--retinol O-acyltransferase	4q32.1				-0.377	0.00157	0.0218				1
LRBA	LPS-responsive vesicle trafficking, beach and anchor containing	4q31.3	-0.475	0.00278	0.0242	-0.393	0.0054725	0.037625				2
AGPAT2	1-acylglycerol-3-phosphate O-acyltransferase 2 (lysophosphatidic acid acyltrar	9q34.3	0.66	0.0065	0.0436							1
LRCH2	leucine-rich repeats and calponin homology (CH) domain containing 2	Xq23				0.453	0.00023	0.0092				1
LRDD	Data not found					-0.548	0.01559	0.0737				1
LRFN1	leucine rich repeat and fibronectin type III domain containing 1	19q13.2	-0.186	0.03571	0.1352	-0.314	0.00242	0.027				2
LRFN2	leucine rich repeat and fibronectin type III domain containing 2	6p21.2-p21.1				0.3275	0.0100275	0.05435				1
LRFN3	leucine rich repeat and fibronectin type III domain containing 3	19q13.12				-0.406	0.005495	0.03265				1
LRFN5	leucine rich repeat and fibronectin type III domain containing 5	14q21.1				0.4058	0.000762	0.01462				1
LRG1	leucine-rich alpha-2-glycoprotein 1	19p13.3	-0.794	0.00099	0.0116							1
LRGUK	leucine-rich repeats and guanylate kinase domain containing	7q33				-0.314	0.00019	0.0086				1
LRIG1	leucine-rich repeats and immunoglobulin-like domains 1	3p14				-0.338	0.000245	0.00885				1

LRIG2	leucine-rich repeats and immunoglobulin-like domains 2	1p13.1				-0.3425	0.004185	0.02925				1
LRIG3	leucine-rich repeats and immunoglobulin-like domains 3	12q14.1							-0.798	2.612	5	1
LRMP	lymphoid-restricted membrane protein	12p12.1				0.807	0.03823	0.126				1
PREP	In multiple Geneids		0.66	0.00745	0.048							1
LRP10	low density lipoprotein receptor-related protein 10	14q11.2	-0.898	0.00159	0.0162							1
LRP11	low density lipoprotein receptor-related protein 11	6q25.1				-0.364	0.00382	0.0341				1
LRP12	low density lipoprotein receptor-related protein 12	8q22.2				0.3205	4.50E-05	0.00445				1
LRP1B	low density lipoprotein receptor-related protein 1B	2q21.2				0.3742	0.0019412	0.017984	-0.645	2.636	5	2
LRP2	low density lipoprotein receptor-related protein 2	2q24-q31				0.331	0.0283	0.1048				1
LRP2BP	LRP2 binding protein	4q35.1							-0.704	2.64571	7	1
LRP4	low density lipoprotein receptor-related protein 4	11p11.2				-0.353	0.00227	0.0261				1
PRIM2	primase, DNA, polypeptide 2 (58kDa)	6p12-p11.1	0.66	0.00628	0.0426							1
LRP5L	low density lipoprotein receptor-related protein 5-like	22q11.23				-0.366	0.00206	0.02335				1
ABCF1	ATP-binding cassette, sub-family F (GCN20), member 1	6p21.33	0.659	0.00534	0.0382							1
LRPAP1	low density lipoprotein receptor-related protein associated protein 1	4p16.3				-0.4565	0.00106	0.0151				1
FOXN2	forkhead box N2	2p22-p16	0.659	1.00E-05	4.00E-04	0.309	0.00184	0.0236				2
LRRC1	In multiple Geneids					0.309	0.015865	0.0744				1
LRRC10	leucine rich repeat containing 10	12q15	-0.243	0.01423	0.0739							1
SNORA27	small nucleolar RNA, H/ACA box 27	13q12.2	0.659	0.01	0.0584							1
LRRC16B	leucine rich repeat containing 16B	14q11.2	-0.17	0.03622	0.1366	-0.382	9.00E-05	0.0063				2
LRRC17	leucine rich repeat containing 17	7q22.1							-0.751	2.536	5	1
CFI	complement factor I	4q25	0.658	0.01751	0.0848							1
LRRC2	leucine rich repeat containing 2	3p21.31				-0.356714286	0.00496	0.033371429				1
LRRC20	leucine rich repeat containing 20	10q22.1	-0.457	0.00755	0.0483	-0.3718	0.001564	0.01696				2
LRRC25	leucine rich repeat containing 25	19p13.11				-0.412	0.00021	0.009				1
LRRC27	leucine rich repeat containing 27	10q26.3				-0.381	0.00117	0.0146				1
LRRC29	leucine rich repeat containing 29	16q22.1				-0.324	0.00539	0.0408				1
LRRC2-AS1	Data not found		-0.243	0.02369	0.1033							1
LRRC31	leucine rich repeat containing 31	3q26.2							-0.85	2.72	6	1
GGT1	gamma-glutamyltransferase 1	22q11.23	0.658	0.00592	0.041							1
RARA	retinoic acid receptor, alpha	17q21	0.658	1.00E-05	5.00E-04							1
LRRC35	Data not found								0.88	2.772	5	1
LRRC36	leucine rich repeat containing 36	16q22.1				-0.3235	0.00157	0.01815				1
LRRC37A	In multiple Geneids								0.868	2.935	6	1
LRRC37A2	leucine rich repeat containing 37, member A2	17q21.31	-1.553	0.00011	0.0023							1
LRRC37A3	leucine rich repeat containing 37, member A3	17q24.1	-0.69	0.00095	0.0113							1
LRRC37A4	Data not found					-0.43	0.00013	0.0072				1
LRRC37A4P	leucine rich repeat containing 37, member A4, pseudogene	17q21.31	-1.182	0.0018	0.0178							1
LRRC37B	In multiple Geneids								0.68	2.768	5	1
LRRC39	leucine rich repeat containing 39	1p21.2							-0.812	2.85833	6	1
LRRC3B	leucine rich repeat containing 3B	3p24				0.41	0.00052	0.0131				1
LRRC4	In multiple Geneids		-0.803	0.00133	0.0144							1
LRRC40	leucine rich repeat containing 40	1p31.1							-0.725	2.68562	8	1
LRRC41	leucine rich repeat containing 41	1p34.1	-0.325	0.03363	0.1297	-0.311	0.01426	0.07				2
LRRC43	leucine rich repeat containing 43	12q24.31				-0.35375	0.0013475	0.015475				1
LRRC47	leucine rich repeat containing 47	1p36.32				-0.3325	0.00142	0.02045				1
LRRC48	leucine rich repeat containing 48	17p11.2				-0.3395	0.0053475	0.039175				1
RDH10	retinol dehydrogenase 10 (all-trans)	8q21.11	0.658	0.04359	0.1537							1
LRRC4B	leucine rich repeat containing 4B	19q13.33				-0.365	0.002942	0.02696				1
LRRC50	Data not found					-0.469	5.00E-05	0.0052				1
LRRC52	leucine rich repeat containing 52	1q24.1	-0.379	0.00391	0.0308							1
LRRC55	leucine rich repeat containing 55	11q12.1	-0.258	0.01136	0.0638				0.935	3.3	6	2
LRRC56	leucine rich repeat containing 56	11p15.5				-0.4455	0.002965	0.0298				1
STAG3L2	stromal antigen 3-like 2	7q11.23	0.658	0.00329	0.0274							1
ZNF26	zinc finger protein 26	12q24.33	0.658	0.00133	0.0144							1
MSTO2P	misato homolog 2 pseudogene	1q22	0.657	0.00845	0.052							1
LRRC61	leucine rich repeat containing 61	7q31-q35				-0.4145	0.000835	0.0158				1
TMEM67	transmembrane protein 67	8q22.1	0.657	0.00011	0.0024							1
LRRC69	leucine rich repeat containing 69	8q21.3				0.33475	9.25E-05	0.005675				1
LRRC7	leucine rich repeat containing 7	1p31.1							-0.744	3.255	6	1
LRRC8A	leucine rich repeat containing 8 family, member A	9q34.11	-0.496	0.03573	0.1352	-0.3335	0.010335	0.0581	0.715	2.97286	7	3
TP53BP1	tumor protein p53 binding protein 1	15q15-q21	0.657	9.00E-05	0.0021							1
USP32P2	ubiquitin specific peptidase 32 pseudogene 2	17p11.2	0.657	0.01809	0.0868							1
LRRC8E	leucine rich repeat containing 8 family, member E	19p13.2				-0.462	0.00054	0.0133				1
ARHGAP21	Rho GTPase activating protein 21	10p12.1	0.656	0.01789	0.0861							1
LRRD1	leucine-rich repeats and death domain containing 1	7q21.2	-0.313	0.0024	0.0218							1
C1QB	complement component 1, q subcomponent, B chain	1p36.12	0.656	0.04127	0.1487							1
LRRFIP2	leucine rich repeat (in FLII) interacting protein 2	3p22.2							-0.836	3.30333	9	1
LRRIQ1	leucine-rich repeats and IQ motif containing 1	12q21.31				0.329	0.00341	0.0321				1

CD164	CD164 molecule, sialomucin	6q21	0.656	0.00186	0.0182							1
LRRK1	leucine-rich repeat kinase 1	15q26.3				0.429	6.00E-05	0.0056				1
LRRK2	In multiple Geneids		-1.543	2.00E-05	6.00E-04							1
LRRN2	In multiple Geneids		-0.363	0.0022	0.0204							1
LRRN3	leucine rich repeat neuronal 3	7q31.1							-0.868	2.73429	7	1
LRRN6C	leucine rich repeat neuronal 6C	9p21.2							-1.107	4.37444	9	1
LRRTM2	leucine rich repeat transmembrane neuronal 2	5q31.2				-0.377	0.00821	0.0514				1
LRRTM3	leucine rich repeat transmembrane neuronal 3	10q21.3							-0.836	3.47125	8	1
LRRTM4	leucine rich repeat transmembrane neuronal 4	2p12				0.34788	0.0027112	0.024016	-1.111	3.26429	7	2
LRTM1	leucine-rich repeats and transmembrane domains 1	3p14.3							-0.873	3.424	5	1
LRTOMT	leucine rich transmembrane and 0-methyltransferase domain containing	11q13.4				-0.301	0.00308	0.0304				1
FUBP1	far upstream element (FUSE) binding protein 1	1p31.1	0.656	2.00E-05	6.00E-04							1
LSDP5	Data not found					-0.524	0.000295	0.00925				1
LSG1	large subunit GTPase 1 homolog (S. cerevisiae)	3q29							-0.808	2.55286	7	1
LSM11	LSM11, U7 small nuclear RNA associated	5q33.3				-0.365	5.00E-05	0.0052				1
GJA5	gap junction protein, alpha 5, 40kDa	1q21.1	0.656	0.00396	0.0311	0.322	0.00555	0.0414				2
LPCAT3	lysophosphatidylcholine acyltransferase 3	12p13	0.656	0.00657	0.0439							1
AHSA1	AHA1, activator of heat shock 90kDa protein ATPase homolog 1 (yeast)	14q24	0.655	0.00333	0.0276							1
LSM5	LSM5 homolog, U6 small nuclear RNA associated (S. cerevisiae)	7p14.3							-0.798	2.65	5	1
LSM6	LSM6 homolog, U6 small nuclear RNA associated (S. cerevisiae)	4q31.22							-0.903	2.85333	6	1
LSP1	lymphocyte-specific protein 1	11p15.5	-0.451	0.00435	0.0332				0.7005	3.081888333	6.666666667	2
LGALS9	lectin, galactoside-binding, soluble, 9	17q11.2	0.655	0.04658	0.1601							1
LSS	lanosterol synthase (2,3-oxidosqualene-lanosterol cyclase)	21q22.3	-0.535	0.02385	0.1037	-0.403666667	0.001925	0.017983333				2
LST1	In multiple Geneids		-0.351	0.0425	0.1513							1
LST-3TM12	Data not found					0.472	8.00E-05	0.006	-0.754	2.83444	9	2
LTA	lymphotoxin alpha (TNF superfamily, member 1)	6p21.3	-0.2735	0.01596	0.07895				0.813	3.184	5	2
LTA4H	leukotriene A4 hydrolase	12q22	-1.374	2.00E-05	8.00E-04							1
LTB4R	leukotriene B4 receptor	14q11.2-q12	-1.562	1.00E-05	3.00E-04							1
LTB4R2	leukotriene B4 receptor 2	14q11.2-q12	-0.551	0.00028	0.0047	-0.345	0.00042	0.0119				2
NRSN2	neurensin 2	20p13	0.655	0.0322	0.1263							1
LTBP2	latent transforming growth factor beta binding protein 2	14q24				-0.34725	0.001225	0.016683333				1
PGS1	phosphatidylglycerophosphate synthase 1	17q25.3	0.655	2.00E-04	0.0036							1
LTBP4	latent transforming growth factor beta binding protein 4	19q13.1-q13.2				-0.364333333	0.003503333	0.027183333				1
ZFYVE27	zinc finger, FYVE domain containing 27	10q24.2	0.655	1.00E-05	5.00E-04							1
LTC4S	leukotriene C4 synthase	5q35	-0.219	0.03418	0.1312				0.59	2.52429	7	2
LTF	lactotransferrin	3p21.31				-0.3645	0.002435	0.0269				1
LTK	leukocyte receptor tyrosine kinase	15q15.1-q21.1				-0.315333333	0.023783333	0.092				1
LTN1	listerin E3 ubiquitin protein ligase 1	21q22.11	-0.279	0.0259	0.1098							1
LUC7L	In multiple Geneids					-0.417	0.010575714	0.053557143				1
CDK5RAP3	CDK5 regulatory subunit associated protein 3	17q21.32	0.654	0.01136	0.0638							1
LOC642947	uncharacterized LOC642947	9q21.13	0.654	0.02293	0.1012							1
LURAP1	leucine rich adaptor protein 1	1p34	-0.293	0.00908	0.0546							1
C5orf15	chromosome 5 open reading frame 15	5q31.1	0.653	0.00011	0.0023							1
LUZP1	leucine zipper protein 1	1p36				-0.374166667	0.005425	0.037683333				1
LUZP2	In multiple Geneids					0.336	0.00206	0.0249				1
LUZP6	leucine zipper protein 6	7q33	-0.934	0.00011	0.0023							1
HSP90AB3P	heat shock protein 90kDa alpha (cytosolic), class B member 3, pseudogene	4q22.1	0.653	0.00756	0.0483							1
LY6D	lymphocyte antigen 6 complex, locus D	8q24	-2.631	0	1.00E-04							1
KIF26B	kinesin family member 26B	1q44	0.653	0.00025	0.0043	0.7085	1.00E-04	0.00655				2
LY6G5B	lymphocyte antigen 6 complex, locus G5B	6p21.3							0.828	3.3	6	1
ZDHHC7	zinc finger, DHHC-type containing 7	16q24.1	0.653	0.00036	0.0056							1
LY6G6C	lymphocyte antigen 6 complex, locus G6C	6p21.33	-3.503	1.00E-05	3.00E-04							1
LY6G6F	lymphocyte antigen 6 complex, locus G6F	6p21	-0.327	0.00317	0.0267							1
LY6K	lymphocyte antigen 6 complex, locus K	8q24.3	-0.66	0.00852	0.0523							1
C1QA	complement component 1, q subcomponent, A chain	1p36.12	0.652	0.03669	0.1378							1
LY86	lymphocyte antigen 86	6p25.1				-0.302	0.003775	0.0295				1
LYAR	Ly1 antibody reactive homolog (mouse)	4p16.3				-0.3818	0.000758	0.01298				1
SEC61G	Sec61 gamma subunit	7p11.2	0.652	0.00046	0.0068							1
LYNX1	Ly6/neurotoxin 1	8q24.3	-1.261	3.00E-05	9.00E-04							1
LYPD2	LY6/PLAUR domain containing 2	8q24.3	-2.085	0	1.00E-04							1
LYPD3	LY6/PLAUR domain containing 3	19q13.31	-3.122	1.00E-05	5.00E-04	-0.308	0.00646	0.045				2
LYPD4	LY6/PLAUR domain containing 4	19q13.2							0.829	2.98	6	1
LYPD5	LY6/PLAUR domain containing 5	19q13.31	-0.86	0.00943	0.0561							1
CENPJ	centromere protein J	13q12.12	0.651	0.02237	0.0996							1
LGI2	leucine-rich repeat LGI family, member 2	4p15.2	0.651	0.0459	0.1588							1
LYPLA1	lysophospholipase I	8q11.23	-0.721	0.00029	0.0048							1
LYPLAL1	lysophospholipase-like 1	1q41				0.411142857	0.00206	0.0205				1
LYRM4	LYR motif containing 4	6p25.1				-0.3615	0.0098825	0.0436				1
LYRM7	Lyrm7 homolog (mouse)	5q23.3	-0.418	0.00588	0.0408	-0.4165	0.00012	0.00705				2

LYSMD4	LysM, putative peptidoglycan-binding, domain containing 4	15q26.3	-0.483	0.00129	0.0141							1
TOM1L1	target of myb1 (chicken)-like 1	17q23.2	0.651	0.00497	0.0364							1
LYZL1	lysozyme-like 1	10p12.1							-1.037	3.08333	6	1
LYZL2	lysozyme-like 2	10p11.23	-0.403	0.01499	0.0765							1
LYZL4	lysozyme-like 4	3p22.1				-0.333	0.00522	0.0401				1
LYZL6	lysozyme-like 6	17q11.2	-0.275	0.00201	0.0191							1
LZIC	leucine zipper and CTNNBIP1 domain containing	1p36.22				-0.563	0.00031	0.0106				1
LZTR1	leucine-zipper-like transcription regulator 1	22q11.21				-0.35975	0.00158	0.021125				1
LZTS1	leucine zipper, putative tumor suppressor 1	8p22	-0.281	0.01994	0.0925							1
PRR13	proline rich 13	12q12	0.6505	0.02435	0.0853							1
BTN3A3	butyrophilin, subfamily 3, member A3	6p21.3	0.65	0.03064	0.1224							1
MAB21L1	mab-21-like 1 (C. elegans)	13q13							1.124	3.45917	6	1
MAB21L3	mab-21-like 3 (C. elegans)	1p13.1	-2.187	4.00E-05	0.0012							1
MACC1	metastasis associated in colon cancer 1	7p21.1				0.337666667	0.001623333	0.019166667				1
MACF1	microtubule-actin crosslinking factor 1	1p32-p31				-0.354777778	0.010097778	0.050366667				1
MACROD1	MACRO domain containing 1	11q11				-0.364	0.001853333	0.022533333				1
MACROD2	MACRO domain containing 2	20p12.1				0.350584615	0.006393846	0.040386154				1
MAD1L1	MAD1 mitotic arrest deficient-like 1 (yeast)	7p22				-0.3555	0.000315	0.0105				1
COL5A1	collagen, type V, alpha 1	9q34.2-q34.3	0.65	0.00152	0.0157							1
FOXA3	forkhead box A3	19q13.2-q13.4	0.65	0.00514	0.0372							1
MADCAM1	mucosal vascular addressin cell adhesion molecule 1	19p13.3				-0.366	0.00068	0.0148				1
LEPREL4	leprecan-like 4	17q21.2	0.65	8.00E-05	0.0018							1
MAEA	macrophage erythroblast attacher	4p16.3	-0.286	0.01837	0.0877	-0.4695	0.000515	0.01185				2
MAEL	maelstrom homolog (Drosophila)	1q24.1				0.314	0.00181	0.0234				1
MAF	v-maf musculoaponeurotic fibrosarcoma oncogene homolog (avian)	16q22-q23	-0.895	0.00021	0.0038							1
MAFB	v-maf musculoaponeurotic fibrosarcoma oncogene homolog B (avian)	20q11.2-q13.1	-0.985	0.00031	0.0051							1
MAFF	v-maf musculoaponeurotic fibrosarcoma oncogene homolog F (avian)	22q13.1	-0.421	0.02355	0.1029	-0.39	0.00592	0.032533333				2
DSN1	DSN1, MIND kinetochore complex component, homolog (S. cerevisiae)	20q11.23	0.649	0.0015	0.0156							1
MAG	In multiple Geneids		-0.172	0.04761	0.1622	-0.313	0.00017	0.0082				2
MAGEA11	melanoma antigen family A, 11	Xq28							-0.705	2.455	6	1
MAGEB1	melanoma antigen family B, 1	Xp21.3							-0.808	3.026873333	7.333333333	1
MAGEB16	In multiple Geneids		-0.318	0.00598	0.0412							1
MAGEB18	melanoma antigen family B, 18	Xp21.3				0.334	0.00143	0.0208	-1.05	2.78333	6	2
MAGEB4	melanoma antigen family B, 4	Xp21.3							-0.786	2.97062	8	1
MAGEC1	melanoma antigen family C, 1	Xq26	-0.317	0.00135	0.0145							1
MAGEC3	melanoma antigen family C, 3	Xq27.2				0.3075	0.01446	0.0585	-0.744	2.64	8	2
MAGED1	melanoma antigen family D, 1	Xp11.23				0.3	0.00091	0.0169				1
EPHA10	EPH receptor A10	1p34.3	0.649	0.00269	0.0236							1
MAGED4	melanoma antigen family D, 4	Xp11.22							0.626272727	2.782790909	7.545454545	1
RIF1	RAP1 interacting factor homolog (yeast)	2q23.3	0.649	6.00E-05	0.0016							1
HIP1	huntingtin interacting protein 1	7q11.23	0.647	0.00557	0.0393							1
MAGEL2	MAGE-like 2	15q11-q12							0.656	2.77	6	1
MAGI1	membrane associated guanylate kinase, WW and PDZ domain containing 1	3p14.1				0.378	0.01027	0.0581				1
MAGI2	membrane associated guanylate kinase, WW and PDZ domain containing 2	7q21				0.354394737	0.015037895	0.061813158				1
MAGI3	membrane associated guanylate kinase, WW and PDZ domain containing 3	1p12-p11.2	-0.967	0.00016	0.0031	-0.325666667	7.00E-05	0.0056				2
Magmas	Data not found					-0.424666667	0.013003333	0.061933333				1
MMP19	matrix metallopeptidase 19	12q14	0.647	9.00E-04	0.0109							1
NFIL3	nuclear factor, interleukin 3 regulated	9q22	0.647	0.00034	0.0054							1
MAK	male germ cell-associated kinase	6p24							-0.933	2.46833	6	1
MAK16	MAK16 homolog (S. cerevisiae)	8p12				-0.401333333	0.00575	0.041433333				1
MAL	mal, T-cell differentiation protein	2q11.1	-5.051	0	0							1
MAL2	mal, T-cell differentiation protein 2 (gene/pseudogene)	8q23				0.3626	0.008898	0.05164				1
MALL	In multiple Geneids		-1.498	0.00725	0.047							1
MALT1	mucosa associated lymphoid tissue lymphoma translocation gene 1	18q21	-0.575	0.00616	0.042	-0.388625	0.00731	0.039625				2
MAML1	mastermind-like 1 (Drosophila)	5q35				-0.379833333	0.004983333	0.031183333				1
MAML2	mastermind-like 2 (Drosophila)	11q21				-0.32	0.00025	0.0096				1
MAML3	mastermind-like 3 (Drosophila)	4q28	-0.972	0	1.00E-04	-0.3384	0.007004	0.04474				2
MAMLD1	mastermind-like domain containing 1	Xq28				0.345	0.00811	0.051				1
NADK2	Data not found		0.646	0.03368	0.1299							1
MAN1C1	mannosidase, alpha, class 1C, member 1	1p35				-0.39405	0.0058065	0.03644				1
MAN2A1	mannosidase, alpha, class 2A, member 1	5q21-q22							-1.098	2.898	5	1
SNORA71B	small nucleolar RNA, H/ACA box 71B	20q11.23	0.646	0.02027	0.0936							1
XPO1	exportin 1 (CRM1 homolog, yeast)	2p15	0.646	0.00232	0.0212							1
MAN2B2	mannosidase, alpha, class 2B, member 2	4p16.1				-0.5718	0.000174	0.00622				1
ARHGEF17	In multiple Geneids		0.645	0.00181	0.0178							1
MANEA	mannosidase, endo-alpha	6q16.1							-1.108	2.562	5	1
MANF	mesencephalic astrocyte-derived neurotrophic factor	3p21.1				-0.357	0.00014	0.00745				1
MAOA	monoamine oxidase A	Xp11.3				0.351933333	0.011822	0.055186667				1
MAOB	monoamine oxidase B	Xp11.23				0.340111111	0.004525556	0.034833333				1

MAP1A	microtubule-associated protein 1A	15q15.3				-0.4155	0.00045	0.0105				1
MAP1B	microtubule-associated protein 1B	5q13				-0.393	0	0.0015				1
CLIC4	chloride intracellular channel 4	1p36.11	0.645	0.01668	0.0821							1
MAP1LC3B	microtubule-associated protein 1 light chain 3 beta	16q24.2				-0.308	0.00154	0.0216				1
INTS8	integrator complex subunit 8	8q22.1	0.645	1.00E-04	0.0022							1
MAP2	microtubule-associated protein 2	2q34-q35				0.357666667	0.00045	0.009233333				1
MAP2K1	mitogen-activated protein kinase kinase 1	15q22.1-q22.33	-0.43	0.02252	0.1	-0.332	0.00278	0.0289				2
MAP2K2	mitogen-activated protein kinase kinase 2	19p13.3				-0.475857143	0.004054286	0.028571429				1
MAP2K3	mitogen-activated protein kinase kinase 3	17q11.2				-0.364	0.01536	0.06205				1
MAP2K4	mitogen-activated protein kinase kinase 4	17p12	-0.795	0.00019	0.0035	-0.308	0.00793	0.0504				2
MTG2	Data not found		0.645	0.00253	0.0226							1
MAP3K1	mitogen-activated protein kinase kinase kinase 1, E3 ubiquitin protein ligase	5q11.2				-0.3275	0.001145	0.01785				1
MAP3K10	mitogen-activated protein kinase kinase kinase 10	19q13.2				-0.365	0.00145	0.021				1
TASP1	taspase, threonine aspartase, 1	20p12.1	0.645	0.0031	0.0262	0.34875	0.00634625	0.041725				2
MAP3K12	mitogen-activated protein kinase kinase kinase 12	12q13							-0.786	2.416	5	1
ZNF165	zinc finger protein 165	6p21.3	0.645	0.01739	0.0844							1
MAP3K14	mitogen-activated protein kinase kinase kinase 14	17q21				-0.322	0.00262	0.0281				1
MAP3K15	mitogen-activated protein kinase kinase kinase 15	Xp22.12	-0.231	0.01592	0.0797	-0.935	0.04181	0.1332				2
CLCN6	chloride channel, voltage-sensitive 6	1p36	0.644	0.00043	0.0065							1
MAP3K4	mitogen-activated protein kinase kinase kinase 4	6q26	-0.65	0.00021	0.0038							1
MAP3K5	mitogen-activated protein kinase kinase kinase 5	6q22.33	-0.502	0.04027	0.1462							1
MAP3K6	mitogen-activated protein kinase kinase kinase 6	1p36.11	-1.328	0.00012	0.0025	-0.49375	0.0014675	0.020025				2
MAP3K7IP1	Data not found					-0.461571429	0.000358571	0.008142857				1
MAP3K7IP3	Data not found					0.331	0.00267	0.028				1
MAP3K9	mitogen-activated protein kinase kinase kinase 9	14q24.3-q31	-1.3	2.00E-05	5.00E-04							1
MAP4	microtubule-associated protein 4	3p21				-0.395689655	0.002776552	0.023006897				1
MAP4K1	mitogen-activated protein kinase kinase kinase kinase 1	19q13.1-q13.4							-0.638	2.432	5	1
CRTC3	CREB regulated transcription coactivator 3	15q26.1	0.644	0.00436	0.0332							1
LOC100133315	transient receptor potential cation channel, subfamily C, member 2-like	11q13.4	0.644	0.00982	0.0577							1
MAP4K4	mitogen-activated protein kinase kinase kinase kinase 4	2q11.2-q12				0.3675	0.00072	0.01175				1
MAP6D1	MAP6 domain containing 1	3q27.1	-0.252	0.01082	0.0616							1
MAP7D1	MAP7 domain containing 1	1p34.3				-0.417	0.00189	0.0239				1
MAP7D2	MAP7 domain containing 2	Xp22.12				0.431	0.00023	0.0093				1
MAPK1	mitogen-activated protein kinase 1	22q11.21	-0.476	0.00063	0.0085	-0.402925926	0.000829259	0.013288889				2
MAPK10	mitogen-activated protein kinase 10	4q22.1-q23							-0.9185	3.1025	6	1
MAPK12	mitogen-activated protein kinase 12	22q13.33				-0.396	0.00044	0.0123				1
MAPK1IP1L	mitogen-activated protein kinase 1 interacting protein 1-like	14q22.3				-0.382	0.00065	0.0145				1
MAPK3	mitogen-activated protein kinase 3	16p11.2	-0.395	0.04892	0.1648	-0.46	0.00024	0.0095				2
MAPK4	mitogen-activated protein kinase 4	18q21.2				-0.367272727	0.002576364	0.024318182				1
MAPK6	mitogen-activated protein kinase 6	15q21	-0.634	0.01289	0.0694							1
MAPK7	mitogen-activated protein kinase 7	17p11.2	-0.652	1.00E-04	0.0022							1
MAPK8	mitogen-activated protein kinase 8	10q11.22							-0.788	3.3	7	1
OR51E1	In multiple Geneids		0.644	0.00016	0.0031							1
MAPK8IP2	mitogen-activated protein kinase 8 interacting protein 2	22q13.33				-0.559	0.00077	0.0157				1
MAPK8IP3	mitogen-activated protein kinase 8 interacting protein 3	16p13.3				-0.3815	0.0096825	0.0547				1
MAPK9	mitogen-activated protein kinase 9	5q35				2.793	0	0				1
MAPKAPK2	mitogen-activated protein kinase-activated protein kinase 2	1q32				0.322	0.02203	0.0903				1
MAPKAPK3	mitogen-activated protein kinase-activated protein kinase 3	3p21.3	-0.863	1.00E-05	3.00E-04	-0.395333333	0.000563333	0.011933333				2
CCNL1	In multiple Geneids		0.643	0.00137	0.0146							1
HNRNPLL	Data not found		0.643	7.00E-05	0.0016							1
MAPKBP1	mitogen-activated protein kinase binding protein 1	15q15.1	-1.143	0	0							1
ATM	ataxia telangiectasia mutated	11q22-q23	0.642	0.01024	0.0595				-0.7196	2.761468	5.4	2
CELSR3	cadherin, EGF LAG seven-pass G-type receptor 3 (flamingo homolog, Drosophi	3p21.31	0.642	0.00332	0.0275							1
MAPT	microtubule-associated protein tau	17q21.1	-1.68	0	1.00E-04	-0.322	0.005355	0.0406				2
KBTBD2	kelch repeat and BTB (POZ) domain containing 2	7p14.3	0.642	1.00E-04	0.0022							1
MARCH10	membrane-associated ring finger (C3HC4) 10, E3 ubiquitin protein ligase	17q23.2				-0.425666667	0.004566667	0.032033333				1
MARCH11	membrane-associated ring finger (C3HC4) 11	5p15.1				0.364875	0.005780833	0.036454167				1
MARCH2	membrane-associated ring finger (C3HC4) 2, E3 ubiquitin protein ligase	19p13.2	-0.487	0.00066	0.0088	-0.434	0.00231	0.023033333				2
SEPT9	septin 9	17q25	0.642	0.00032	0.0051							1
MARCH5	membrane-associated ring finger (C3HC4) 5	10q23.32-q23.33	-1.035	3.00E-05	8.00E-04	-0.36375	9.75E-05	0.00645				2
TMEM214	transmembrane protein 214	2p23.3	0.642	0.00197	0.0189							1
MARCH7	membrane-associated ring finger (C3HC4) 7, E3 ubiquitin protein ligase	2q24.2	-0.641	0.00196	0.0188							1
MARCKS	myristoylated alanine-rich protein kinase C substrate	6q22.2	-0.258	0.03833	0.1418							1
ZNF195	zinc finger protein 195	11p15.5	0.642	8.00E-05	0.0019							1
MARCO	macrophage receptor with collagenous structure	2q14.2	-0.283	0.01669	0.0821							1
MARK1	MAP/microtubule affinity-regulating kinase 1	1q41				0.348333333	0.001933333	0.023366667				1
MARK2	MAP/microtubule affinity-regulating kinase 2	11q13.1	-0.644	0.00121	0.0134	-0.3335	0.005215	0.0401				2
MARK3	MAP/microtubule affinity-regulating kinase 3	14q32.3				-0.337666667	0.007206667	0.0409				1
MARK4	MAP/microtubule affinity-regulating kinase 4	19q13.3	-0.255	0.04682	0.1606	-0.395666667	0.00354	0.0325				2

MARS	methionyl-tRNA synthetase	12q13				-0.3345	0.017475	0.0786				1
MARS2	methionyl-tRNA synthetase 2, mitochondrial	2q33.1							-0.877	2.606	5	1
AMIGO2	adhesion molecule with Ig-like domain 2	12q13.11	0.641	0.02795	0.1151							1
MARVELD2	MARVEL domain containing 2	5q13.2				-0.3815	0.006475	0.0374				1
APBB1IP	amyloid beta (A4) precursor protein-binding, family B, member 1 interacting p	10p12.1	0.64	0.0491	0.1652							1
MAS1	MAS1 oncogene	6q25.3-q26	-0.206	0.03465	0.1324							1
MASP1	mannan-binding lectin serine peptidase 1 (C4/C2 activating component of Ra-	3q27-q28				0.402	0.00018	0.0083				1
MASP2	mannan-binding lectin serine peptidase 2	1p36.3-p36.2				-0.4255	0.01188	0.05185				1
MAST1	microtubule associated serine/threonine kinase 1	19p13.2				-0.437	0.008636667	0.048566667				1
C3AR1	complement component 3a receptor 1	12p13.31	0.64	0.04515	0.1573							1
CLCN2	chloride channel, voltage-sensitive 2	3q27-q28	0.64	0.00327	0.0273							1
MAST4	In multiple Geneids		-1.811	0	0							1
MASTL	microtubule associated serine/threonine kinase-like	10p12.1				-0.302	0.04214	0.1338				1
MAT1A	methionine adenosyltransferase I, alpha	10q22							-0.795	2.79143	7	1
MATK	megakaryocyte-associated tyrosine kinase	19p13.3				-0.491	0.005853333	0.0323				1
MATN1	matrilin 1, cartilage matrix protein	1p35				-0.402	3.00E-04	0.0099				1
C1orf85	chromosome 1 open reading frame 85	1q22	0.639	0.01306	0.0699							1
MAU2	MAU2 chromatid cohesion factor homolog (C. elegans)	19p13.11	-0.337	0.00136	0.0146							1
MAWBP	Data not found								-0.78	2.538	5	1
MAX	MYC associated factor X	14q23	-0.438	0.00385	0.0305	-0.330333333	0.003123333	0.027033333				2
MB	myoglobin	22q13.1				-0.433333333	0.000383333	0.0083				1
DAP3	death associated protein 3	1q22	0.639	0.00022	0.0039							1
ZNF275	zinc finger protein 275	Xq28	0.639	0.01399	0.0731							1
MBD1	methyl-CpG binding domain protein 1	18q21				-0.396	0.000325	0.01065				1
MBD2	methyl-CpG binding domain protein 2	18q21							-0.784	2.565	6	1
MBD3	In multiple Geneids		-0.264	0.03576	0.1353							1
MBD3L2	In multiple Geneids					-0.3255	0.02049	0.0864				1
MBD3L3	methyl-CpG binding domain protein 3-like 3	19p13.2	-0.294	0.03026	0.1214							1
MBD3L5	methyl-CpG binding domain protein 3-like 5	19p13.2				-0.468	0.00858	0.0527				1
MBD6	methyl-CpG binding domain protein 6					-0.342	0.00027	0.01				1
MBL2	mannose-binding lectin (protein C) 2, soluble	10q11.2							-0.928	3.492	5	1
MBLAC2	metallo-beta-lactamase domain containing 2	5q14.3	-0.576	0.0024	0.0218	-0.306	0.00304	0.0302				2
MBNL1	muscleblind-like splicing regulator 1	3q25				0.339176471	0.001423529	0.019352941	-0.94	3.204	5	2
MBNL2	muscleblind-like splicing regulator 2	13q32.1				0.3545	0.00483125	0.0358				1
MBNL3	muscleblind-like splicing regulator 3	Xq26.2				0.336	0.003566667	0.03215				1
MBOAT2	membrane bound O-acyltransferase domain containing 2	2p25.1				0.38	0.00043	0.0121				1
MBOAT7	membrane bound O-acyltransferase domain containing 7	19q13.4				-0.313	0.03091	0.1106				1
MBP	myelin basic protein	18q23	-0.409	0.01064	0.0611	-0.387538462	0.002358462	0.022876923				2
MBTPS1	membrane-bound transcription factor peptidase, site 1	16q24				-0.386	0.001914	0.01982				1
VASP	vasodilator-stimulated phosphoprotein	19q13.32	0.638	0.00028	0.0046							1
MC3R	melanocortin 3 receptor	20q13.2-q13.3							0.642	2.45	5	1
MC5R	melanocortin 5 receptor	18p11.2	-0.675	0.00714	0.0465							1
ZNF137P	zinc finger protein 137, pseudogene	19q13.4	0.638	0.01028	0.0597							1
MCART1	Data not found					-0.301	0.00126	0.0196				1
MCART6	Data not found								-0.94	3.19571	7	1
MCAT	malonyl CoA:ACP acyltransferase (mitochondrial)	22q13.31				-0.4955	0.00021	0.00805				1
MCC	mutated in colorectal cancers	5q21	-1.343	0	2.00E-04	-0.353625	0.00293375	0.02035				2
MCCC2	In multiple Geneids					-0.338666667	0.003221667	0.0259				1
MCEE	methylmalonyl CoA epimerase	2p13.3							-0.598	2.70333	6	1
MCF2	MCF.2 cell line derived transforming sequence	Xq27				0.36725	0.0020075	0.021975	-0.84	2.90556	9	2
AP3M2	adaptor-related protein complex 3, mu 2 subunit	8p11.2	0.637	0.00114	0.0128							1
MCHR1	melanin-concentrating hormone receptor 1	22q13.2				-0.588	0.00032	0.0106				1
MCHR2	melanin-concentrating hormone receptor 2	6q16	-0.215	0.04098	0.1479							1
MCM10	minichromosome maintenance complex component 10	10p13				-0.34	0.011327143	0.056542857				1
PLBD2	phospholipase B domain containing 2	12q24.13	0.637	0	0							1
MCM3AP	minichromosome maintenance complex component 3 associated protein	21q22.3				-0.368777778	0.00262	0.0245				1
MCM3APAS	Data not found					-0.362	0.004305	0.0346				1
MCM5	minichromosome maintenance complex component 5	22q13.1				-0.3838	0.00116	0.0151				1
TDRD5	tudor domain containing 5	1q25.2	0.637	0.02821	0.1158							1
NCBP2	nuclear cap binding protein subunit 2, 20kDa	3q29	0.636	0.00995	0.0582							1
CDK2	cyclin-dependent kinase 2	12q13	0.635	0.00502	0.0366							1
MCMDC1	Data not found								-0.755	2.736	5	1
MCOLN1	mucolipin 1	19p13.2				-0.41425	0.0060675	0.038925				1
MCOLN2	mucolipin 2	1p22				-0.3915	0.00029	0.00895				1
MCPH1	In multiple Geneids								-0.978	3.354	5	1
MCTP1	multiple C2 domains, transmembrane 1	5q15							-0.898	3.32125	8	1
LPL	lipoprotein lipase	8p22	0.635	0.00103	0.0119							1
SYNGR2	synaptogyrin 2	17q25.3	0.634	0.00061	0.0083							1
MDFI	MyoD family inhibitor	6p21							0.882	3.33556	9	1

MDFIC	MyoD family inhibitor domain containing	7q31.1-q31.2	-0.868	0	2.00E-04							1
MDGA1	MAM domain containing glycosylphosphatidylinositol anchor 1	6p21	-0.704	0.00081	0.0101							1
MDGA2	MAM domain containing glycosylphosphatidylinositol anchor 2	14q21.3				0.331	0.00253	0.0276				1
MDH1	malate dehydrogenase 1, NAD (soluble)	2p13.3				0.314	0.00024	0.0093				1
TGDS	TDP-glucose 4,6-dehydratase	13q32.1	0.634	0.0029	0.025							1
MDM1	In multiple Geneids								-0.746	2.60667	6	1
MDN1	In multiple Geneids					-0.336	0.001865714	0.022371429				1
MDS2	myelodysplastic syndrome 2 translocation associated	1p36				-0.3565	0.003825	0.0322				1
ME1	malic enzyme 1, NADP(+)-dependent, cytosolic	6q12	-1.07	0.02687	0.1123							1
ME2	malic enzyme 2, NAD(+)-dependent, mitochondrial	18q21				-0.389	0.002704286	0.022				1
MECOM	MDS1 and EVI1 complex locus	3q26				0.379554217	0.004947831	0.033778313				1
MECR	In multiple Geneids		-0.225	0.02338	0.1025	-0.347285714	0.009877143	0.054				2
MED11	mediator complex subunit 11	17p13.2				-0.496	0.00612	0.0437				1
ZNF432	zinc finger protein 432	19q13.41	0.634	0.01419	0.0737							1
MED12L	mediator complex subunit 12-like	3q25.1				0.336	0.003542	0.02818				1
MED13L	mediator complex subunit 13-like	12q24.21				0.349	0.00029	0.00965				1
C3orf33	chromosome 3 open reading frame 33	3q25.31	0.633	0.00755	0.0483							1
MED15	mediator complex subunit 15	22q11.2				-0.427266667	0.001914667	0.016033333				1
MED16	mediator complex subunit 16	19p13.3	-0.279	0.0391	0.1434	-0.63	0.00053	0.0132				2
FGFRL1	fibroblast growth factor receptor-like 1	4p16	0.633	0.00212	0.0199							1
MED18	mediator complex subunit 18	1p35.3				-0.368333333	0.008523333	0.045333333				1
FOXQ1	forkhead box Q1	6p25	0.633	0.01451	0.0748	0.329	0.04119	0.1319				2
IVNS1ABP	influenza virus NS1A binding protein	1q25.1-q31.1	0.633	0.00288	0.0248	0.34	0.00419	0.0356				2
MED24	mediator complex subunit 24	17q21.1				-0.3885	0.00143	0.0193				1
MED25	mediator complex subunit 25	19q13.3	-0.224	0.04094	0.1478							1
MED26	mediator complex subunit 26	19p13.11	-0.313	0.00183	0.0179	-0.386842105	0.004128947	0.030936842				2
SAMD12	sterile alpha motif domain containing 12	8q24.12	0.633	0.03879	0.1428	0.374626866	0.008908806	0.046641791				2
MED30	mediator complex subunit 30	8q24.11				0.4295	0.003085	0.02555				1
MED31	mediator complex subunit 31	17p13.1	-0.659	0.0062	0.0422							1
MED7	mediator complex subunit 7	5q33.3				-0.368	0.00071	0.0151				1
MED8	mediator complex subunit 8	1p34.2							-0.809	2.578	5	1
MED9	mediator complex subunit 9	17p11.2				-0.372	0.01471	0.0713				1
MEF2A	myocyte enhancer factor 2A	15q26				0.34225	0.002005	0.022875	-0.852	2.672	5	2
MEF2B	myocyte enhancer factor 2B	19p13.11	-0.271	0.00495	0.0363	-0.4732	0.004628	0.03138				2
MEG3	maternally expressed 3 (non-protein coding)	14q32							0.76975	2.98025	5.75	1
MEGF10	multiple EGF-like-domains 10	5q33				-0.317	0.00071	0.01465				1
MEGF11	multiple EGF-like-domains 11	15q22.31				-0.352	0.00103	0.013433333				1
MEGF6	multiple EGF-like-domains 6	1p36.3	-0.259	0.02844	0.1165	-0.38	0.00065	0.01415				2
MEGF8	multiple EGF-like-domains 8	19q12				-0.336666667	0.002233333	0.0255				1
MEGF9	multiple EGF-like-domains 9	9q32-q33.3				0.371	0.00177	0.0231				1
MEI1	meiosis inhibitor 1	22q13.2				-0.402857143	0.002226667	0.019609524				1
MEIG1	In multiple Geneids		-0.19	0.0206	0.0945							1
MEIS1	Meis homeobox 1	2p14				0.3314	0.000952	0.01214				1
MEIS2	In multiple Geneids					0.303	0.00533	0.0406				1
ZNF217	zinc finger protein 217	20q13.2	0.633	0.00137	0.0146	0.358	0.04751	0.1442				2
MELK	maternal embryonic leucine zipper kinase	9p13.2				-0.365142857	0.004217857	0.0314				1
MEMO1	In multiple Geneids		-0.397	0.00723	0.0469							1
MEOX1	mesenchyme homeobox 1	17q21	-0.31	0.0035	0.0285	-0.3	0.00191	0.024				2
MEOX2	mesenchyme homeobox 2	7p22.1-p21.3				0.360285714	0.001604286	0.018885714				1
KNSTRN	Data not found		0.632	0.02952	0.1193							1
MEP1B	meprin A, beta	18q12.2-q12.3				-0.35	0.02309	0.0929				1
MEPE	matrix extracellular phosphoglycoprotein	4q21.1							-0.89	3.14429	7	1
PABPC1L	poly(A) binding protein, cytoplasmic 1-like		0.632	0.01455	0.0749							1
SNORA22	small nucleolar RNA, H/ACA box 22	7q11.21	0.632	0.00766	0.0487							1
TRANK1	tetratricopeptide repeat and ankyrin repeat containing 1	3p22.2	0.632	0.00692	0.0455							1
CCR1	chemokine (C-C motif) receptor 1	3p21	0.631	0.01283	0.0691							1
METAP1	methionyl aminopeptidase 1	4q23	-0.921	0.00017	0.0033							1
METRNL	meteorin, glial cell differentiation regulator-like	17q25.3	-0.871	0	1.00E-04							1
METT10D	Data not found					-0.3749375	0.012741875	0.05674375				1
EXT2	exostosin 2	11p12-p11	0.631	0.00022	0.004							1
METTL14	methyltransferase like 14	4q26				-0.301	0.0049	0.0388				1
FHOD1	formin homology 2 domain containing 1	16q22	0.631	0.00032	0.0052							1
METTL20	methyltransferase like 20	12p11.21	-0.27	0.03571	0.1352							1
HIST2H2AA3	histone cluster 2, H2aa3	1q21.2	0.631	0.00267	0.0235							1
METTL21C	methyltransferase like 21C	13q33.1	-0.189	0.02934	0.1188							1
METTL25	Data not found		-0.616	0.00112	0.0127							1
IQCK	IQ motif containing K	16p12.3	0.631	0.00221	0.0205							1
KIFC1	kinesin family member C1	6p21.3	0.631	0.0431	0.1526							1
METTL3	methyltransferase like 3	14q11.1				-0.378	0.00105	0.018				1

LGALS3BP	lectin, galactoside-binding, soluble, 3 binding protein	17q25	0.631	0.00427	0.0327							1
SFR1	SWI5-dependent recombination repair 1	10q25.1	0.631	0.00248	0.0223							1
MEX3C	mex-3 homolog C (C. elegans)	18q21.2				-0.33925	0.0082475	0.045275				1
MEX3D	mex-3 homolog D (C. elegans)	19p13.3				-0.434	0.00529	0.0404				1
MFAP1	microfibrillar-associated protein 1	15q15-q21				-0.372	0.00263	0.0281				1
GUSBP3	glucuronidase, beta pseudogene 3	5q13.2	0.6305	0.018963333	0.083533333							1
MFAP3L	microfibrillar-associated protein 3-like	4q32.3				-0.348	0.0027	0.0285				1
AVL9	AVL9 homolog (S. cerevisiase)	7p14.3	0.63	0.00096	0.0114							1
ENTPD8	ectonucleoside triphosphate diphosphohydrolase 8	9q34.3	0.63	0.02056	0.0945							1
RNF213	ring finger protein 213	17q25.3	0.63	0.00899	0.05075							1
DNAJC12	DnaJ (Hsp40) homolog, subfamily C, member 12	10q22.1	0.629	0.00988	0.0579							1
MFN2	mitofusin 2	1p36.22	-0.531	0.00101	0.0118	-0.451	0.0004425	0.01205				2
MFNG	MFNG O-fucosylpeptide 3-beta-N-acetylglucosaminyltransferase	22q12				-0.422	0.002798	0.02206				1
MFSD1	major facilitator superfamily domain containing 1	3q25.32	-1.076	6.00E-05	0.0015							1
FGF20	fibroblast growth factor 20	8p22	0.629	0.04688	0.1607							1
MFSD11	major facilitator superfamily domain containing 11	17q25	-0.397	0.00643	0.0432	-0.307	0.01737	0.0785				2
PDGFC	platelet derived growth factor C	4q32	0.629	0.04997	0.167							1
MFSD2	Data not found					-0.398	4.00E-04	0.0117				1
MFSD5	major facilitator superfamily domain containing 5	12q13.13	-1.077	1.00E-05	3.00E-04	-0.353	0.00184	0.0236				2
MFSD6	major facilitator superfamily domain containing 6	2q32.2				0.352	0.00297	0.0299				1
CRLS1	cardiolipin synthase 1	20p13-p12.3	0.628	0.00459	0.0345							1
MGA	MAX gene associated	15q14				-0.316	0.00423	0.0359				1
FXYD5	FXYD domain containing ion transport regulator 5	19q13.12	0.628	0.03237	0.1267							1
MGAT1	mannosyl (alpha-1,3-)-glycoprotein beta-1,2-N-acetylglucosaminyltransferase	5q35	-0.295	0.04645	0.1598							1
HIST1H4K	histone cluster 1, H4k	6p22.1	0.628	0.00708	0.0462							1
MRPL50	mitochondrial ribosomal protein L50	9q31.1	0.628	0.00288	0.0248							1
SLC25A33	solute carrier family 25 (pyrimidine nucleotide carrier), member 33	1p36.22	0.628	0.01554	0.0783							1
TMEFF1	transmembrane protein with EGF-like and two follistatin-like domains 1	9q31	0.628	0.01145	0.0641							1
MGAT4C	mannosyl (alpha-1,3-)-glycoprotein beta-1,4-N-acetylglucosaminyltransferase,	12q21				0.339	0.00122	0.0194	-0.739	2.75444	9	2
MCM3	minichromosome maintenance complex component 3	6p12	0.627	0.01628	0.0808							1
MGC119295	Data not found								1.318	2.442	5	1
MGC12760	Data not found								0.691	2.888	5	1
MGC13024	Data not found								0.857	2.534	5	1
MGC14436	Data not found					-0.355333333	0.001613333	0.016133333				1
MGC16028	Data not found								0.641	2.64667	6	1
MGC16703	tubulin, alpha pseudogene	22q11.21				-0.339	0.00047	0.0126				1
MGC23284	uncharacterized LOC197187	16q24.3				-0.3915	0.004415	0.0338				1
MGC2408	hypothetical protein MGC2408	3q27.1							-0.725	2.63	5	1
MGC24381	Data not found								0.987	2.96667	6	1
MGC2463	Data not found								0.97	3.21667	6	1
MGC26597	Data not found					-0.39	0.00258	0.0278				1
MGC26647	Data not found								-0.985	3.17167	6	1
MGC26718	Data not found								0.705	2.57375	8	1
MGC26733	Data not found								-0.616	2.418	5	1
MGC26856	Data not found								-0.66	2.72429	7	1
MGC2752	CENPB DNA-binding domains containing 1 pseudogene	19q13.43							-0.883	3.012	5	1
MGC29898	Data not found								-0.738	2.436	5	1
MGC33302	Data not found								-0.577	2.406	5	1
MGC33367	Data not found								-1.038	3.648	5	1
MGC33648	Data not found								-0.956	2.756	5	1
MGC33926	Data not found								0.702	3.12	5	1
MGC34646	Data not found								-1.032	2.635	6	1
MGC34761	Data not found								0.638	2.532	5	1
MGC34774	Data not found								-0.648	3.176	5	1
MGC34821	Data not found								-0.881	3.32	7	1
MGC34824	Data not found								-0.922	3.65875	8	1
MGC35043	Data not found								-1.048	2.58	6	1
MGC35154	Data not found								0.78	2.682	5	1
MGC35212	Data not found								-0.774	3.14778	9	1
MGC35361	Data not found								-0.662	2.492	5	1
MGC3771	uncharacterized LOC81854	16p13.3				-0.429	0.016615	0.07015				1
MGC39372	serpin peptidase inhibitor, clade B (ovalbumin), member 9 pseudogene	6p25.2	-1.284	0.00708	0.0462							1
MGC39518	Data not found								-0.542	2.326	5	1
MGC39545	uncharacterized LOC403312	11q24.2	-0.299	0.00244	0.022							1
MGC39633	Data not found								-0.774	2.768	5	1
MGC42090	Data not found								0.877	2.84429	7	1
MGC42630	Data not found								0.914	2.97167	6	1
MGC52000	Data not found								0.7625	3.528105	7.833333333	1
MGC57346	uncharacterized LOC401884	17q21.31	-0.85	0.00256	0.0228							1

MGC70870	C-terminal binding protein 2 pseudogene								0.606	3.306	5	1
MGC72080	MGC72080 pseudogene	7q21.3	-0.41125	0.041685	0.14795							1
MGC72104	Data not found								1.014	2.728	5	1
MGC88374	Data not found								-0.963	3.076	5	1
MGEA5	meningioma expressed antigen 5 (hyaluronidase)	10q24.1-q24.3				-0.568	0	0.0011				1
MGLL	monoglyceride lipase	3q21.3	-1.145	0.00677	0.0449	-0.311	0.005615	0.0366				2
MGMT	O-6-methylguanine-DNA methyltransferase	10q26				0.305	0.00016	0.008				1
MYCN	v-myc myelocytomatosis viral related oncogene, neuroblastoma derived (avia	2p24.3	0.627	0.02738	0.1136							1
MGRN1	mahogunin ring finger 1, E3 ubiquitin protein ligase	16p13.3				-0.454166667	0.0133675	0.063225				1
MGST1	microsomal glutathione S-transferase 1	12p12.3-p12.1							-0.488	2.818	5	1
MGST2	microsomal glutathione S-transferase 2	4q28.3	-0.876	0.00078	0.0098	-0.311	0.01735	0.0784				2
SLC39A13	solute carrier family 39 (zinc transporter), member 13	11p11.2	0.627	0.00083	0.0103							1
SMS	spermine synthase	Xp22.1	0.627	0.00263	0.0232	0.318	0.00312	0.0306				2
AMPD2	adenosine monophosphate deaminase 2	1p13.3	0.626	0	1.00E-04				-1.027	3.21333	6	2
MIAT	myocardial infarction associated transcript (non-protein coding)	22q12.1				-0.482	0.000365	0.0082				1
MIB2	mindbomb E3 ubiquitin protein ligase 2	1p36.33	-0.221	0.04411	0.1549	-0.407	0.00038	0.0115				2
BCAR1	breast cancer anti-estrogen resistance 1	16q23.1	0.626	0.00233	0.0213							1
ECM2	extracellular matrix protein 2, female organ and adipocyte specific	9q22.3	0.626	0.00033	0.0053							1
GNG10	guanine nucleotide binding protein (G protein), gamma 10	9q31.3	0.626	0.00289	0.0249							1
GPR124	G protein-coupled receptor 124	8p11.23	0.626	0.04611	0.1592							1
MICALL1	MICAL-like 1	22q13.1	-1.386	0	0	-0.429	0.000861667	0.013233333				2
ISYNA1	inositol-3-phosphate synthase 1	19p13.11	0.626	0.01981	0.0922							1
KCNMB3	potassium large conductance calcium-activated channel, subfamily M beta me	3q26.3-q27	0.626	0.01211	0.05675	0.345	0.01325	0.0671				2
LRRFIP1	leucine rich repeat (in FLII) interacting protein 1	2q37.3	0.626	0.00952	0.0565							1
MID1	midline 1 (Opitz/BBB syndrome)	Xp22				0.3288	0.009974	0.05534				1
MID2	midline 2	Xq22.3	-1.592	0.00011	0.0024							1
MIER2	mesoderm induction early response 1, family member 2	19p13.3	-0.302	0.04848	0.164	-0.429857143	0.012972857	0.057342857				2
MIF4GD	MIF4G domain containing	17q25.1				-0.335	0.00289	0.0294				1
MIIP	migration and invasion inhibitory protein	1p36.22				-0.4085	0.00133	0.01785				1
MINK1	misshapen-like kinase 1	17p13.2	-0.753	2.00E-05	7.00E-04	-0.417285714	0.009581429	0.047428571				2
RNF5	ring finger protein 5, E3 ubiquitin protein ligase	6p21.3	0.626	0.02799	0.1152							1
MIOS	missing oocyte, meiosis regulator, homolog (Drosophila)	7p21.3				0.308	0.00125	0.0195				1
MIPEP	mitochondrial intermediate peptidase	13q12				0.365	0.00675	0.0461				1
MIR106B	microRNA 106b	7q22.1	-0.262	0.01194	0.066							1
MIR125A	microRNA 125a	19q13.41	-0.386	0.00765	0.0487							1
MIR127	microRNA 127	14q32.2	-0.236	0.01199	0.0662							1
MIR128-2	microRNA 128-2	3p22.3	-0.296	0.01075	0.0615							1
MIR129-2	microRNA 129-2	11p11.2	-0.309	0.00534	0.0382							1
MIR130A	microRNA 130a	11q12.1	-0.367	0.01643	0.0812							1
MIR133B	microRNA 133b	6p12.2	-0.306	0.00843	0.052							1
MIR137	microRNA 137	1p21.3	-0.327	0.0091	0.0547							1
MIR149	microRNA 149	2q37.3	-0.301	0.00839	0.0518							1
MIR150	microRNA 150	19q13.33	-0.614	0	2.00E-04							1
MIR153-1	microRNA 153-1	2q35	-0.196	0.03796	0.1408							1
MIR154	microRNA 154	14q32.31	-0.222	0.02143	0.0972							1
MIR182	microRNA 182	7q32.2	-0.297	0.04963	0.1664							1
MIR185	microRNA 185	22q11.21	-0.391	0.00075	0.0096							1
SNRPG	small nuclear ribonucleoprotein polypeptide G	2p13.3	0.626	0.008145	0.04975							1
MIR193A	microRNA 193a	17q11.2	-0.547	0.00029	0.0048							1
MIR197	microRNA 197	1p13.3	-0.637	2.00E-05	7.00E-04							1
MIR200B	microRNA 200b	1p36.33	-0.28	0.01505	0.0768							1
MIR203	microRNA 203	14q32.33	-0.997	0.00062	0.0084							1
MIR205	microRNA 205	1q32.2	-2.808	0	0							1
MIR208A	microRNA 208a	14q11.2	-0.432	0.00023	0.0041							1
MIR210	microRNA 210	11p15.5	-0.281	0.04592	0.1588							1
MIR212	microRNA 212	17p13.3	-0.245	0.02303	0.1014							1
MIR221	microRNA 221	Xp11.3	-1.196	0.00492	0.0361							1
MIR222	microRNA 222	Xp11.3	-0.517	0.00869	0.0531							1
MIR223	microRNA 223	Xq12	-0.296	0.02536	0.1082							1
MIR224	microRNA 224	Xq28	-1.415	0	0							1
MIR23B	microRNA 23b	9q22.32	-0.77	2.00E-05	6.00E-04							1
MIR26B	microRNA 26b	2q35	-0.441	0.00361	0.0291							1
MIR27B	microRNA 27b	9q22.32	-2.578	0	1.00E-04							1
MIR296	microRNA 296	20q13.32	-0.223	0.03911	0.1434							1
MIR29A	microRNA 29a	7q32.3	-0.223	0.02623	0.1107							1
MIR30E	microRNA 30e	1p34.2	-0.936	2.00E-05	7.00E-04							1
MIR31	microRNA 31	9p21.3	-0.497	0.01389	0.0727							1
MIR320C1	In multiple Geneids		-0.287	0.02905	0.118							1
MIR377	microRNA 377	14q32.31	-0.161	0.03431	0.1315							1

MIR423	microRNA 423	17q11.2	-0.295	0.0212	0.0965							1
ZNF702P	zinc finger protein 702, pseudogene	19q13.41	0.626	0.00291	0.025							1
MIR519A2	microRNA 519a-2	19q13.42	-0.224	0.00885	0.0537							1
MIR600HG	MIR600 host gene (non-protein coding)	9q33.3	-0.919	0.00023	0.0041							1
NOMO3	NODAL modulator 3	16p13	0.625	0.00348	0.0283							1
MIR7-3HG	MIR7-3 host gene (non-protein coding)	19p13.3	-0.305	0.00956	0.0566							1
MIR95	microRNA 95		-0.717	0.01947	0.0912							1
MIR99A	microRNA 99a	21q21.1	-0.97	4.00E-05	0.001							1
MIRLET7A3	microRNA let-7a-3	22q13.31	-0.564	0.00021	0.0037							1
MIRLET7B	microRNA let-7b	22q13.31	-0.4	0.00942	0.0561							1
MIRLET7C	microRNA let-7c	21q21.1	-0.906	0	1.00E-04							1
MIRLET7E	microRNA let-7e	19q13.41	-0.384	0.00534	0.0382							1
MIRLET7G	microRNA let-7g	3p21.1	-0.857	0	0							1
CNNM2	In multiple Geneids		0.624	0.00096	0.0114							1
MIST	Data not found								-1.04	2.59875	8	1
KLF11	Kruppel-like factor 11	2p25	0.624	0.00981	0.0576							1
MITF	microphthalmia-associated transcription factor	3p14.2-p14.1							-0.9022	3.1804	5	1
MKL1	In multiple Geneids					-0.389777778	0.002776667	0.024002778				1
MKLN1	muskelin 1, intracellular mediator containing kelch motifs	7q32				-0.36425	0.001615	0.02125				1
MKNK1	MAP kinase interacting serine/threonine kinase 1	1p33	-0.313	0.014	0.0731	-0.4	0.00147	0.0211				2
MKNK2	MAP kinase interacting serine/threonine kinase 2	19p13.3	-1.294	0	0							1
MKRN1	makorin ring finger protein 1	7q34				-0.3815	0.010305	0.0553				1
MKRN2	makorin ring finger protein 2	3p25	-0.337	0.0125	0.068							1
MKX	mohawk homeobox	10p12.1				0.803	2.00E-05	0.0035				1
MLANA	melan-A	9p24.1				-0.425	0.001295	0.01975				1
MLC1	megalencephalic leukoencephalopathy with subcortical cysts 1	22q13.33				-0.415666667	0.000196667	0.0084				1
NABP2	Data not found		0.624	0.00113	0.0128							1
MLF1	myeloid leukemia factor 1	3q25.1				0.3	0.00056	0.0136				1
MLF1IP	In multiple Geneids					-0.394	0.0044875	0.035425				1
MLH1	mutL homolog 1, colon cancer, nonpolyposis type 2 (E. coli)	3p21.3				-0.346	0.016905	0.07245				1
CLIC1	chloride intracellular channel 1	6p21.3	0.623	0.00141	0.015							1
MLIP	muscular LMNA-interacting protein	6p12.1	-0.683	0.00561	0.0395							1
MLKL	mixed lineage kinase domain-like	16q23.1				-0.348	0.00817	0.04245				1
MLL2	myeloid/lymphoid or mixed-lineage leukemia 2	12q13.12				-0.424	4.75E-05	0.004075				1
MLL3	myeloid/lymphoid or mixed-lineage leukemia 3	7q36.1				-0.534757576	0.003262121	0.022078788				1
MLL4	myeloid/lymphoid or mixed-lineage leukemia 4	19q13.1							-0.749	3.046	5	1
MLLT1	myeloid/lymphoid or mixed-lineage leukemia (trithorax homolog, Drosophila)	19p13.3				-0.436	0.004589412	0.031511765				1
MLLT10	myeloid/lymphoid or mixed-lineage leukemia (trithorax homolog, Drosophila)	10p12				-0.3355	0.00419	0.0336				1
MLLT3	myeloid/lymphoid or mixed-lineage leukemia (trithorax homolog, Drosophila)	9p22				-0.352583333	0.003489167	0.028433333				1
MLLT4	myeloid/lymphoid or mixed-lineage leukemia (trithorax homolog, Drosophila)	6q27							-0.947	3.234	5	1
MLN	motilin	6p21.3	-0.264	0.02773	0.1145							1
PHF14	PHD finger protein 14	7p21.3	0.623	0.00046	0.0068	0.3115	0.00098	0.01445				2
MLST8	MTOR associated protein, LST8 homolog (S. cerevisiae)	16p13.3	-0.251	0.01371	0.0721							1
MLX	In multiple Geneids								-0.627	2.436	5	1
RWDD2A	RWD domain containing 2A	6q14.2	0.623	0.00087	0.0106							1
ZKSCAN8	Data not found		0.623	0.03767	0.14							1
MMADHC	methylmalonic aciduria (cobalamin deficiency) cblD type, with homocystinuria	2q23.2				0.36	4.00E-05	0.0048				1
ZNF267	zinc finger protein 267	16p11.2	0.623	0.00612	0.0419							1
MMD2	In multiple Geneids					-0.3245	0.022405	0.08925	0.691	2.61714	7	2
PLVAP	plasmalemma vesicle associated protein	19p13.2	0.622	0.00543	0.0387							1
MMEL1	In multiple Geneids		-0.193	0.03412	0.131	-0.385	0.01518	0.06435	0.638	2.526	5	3
DDOST	dolichyl-diphosphooligosaccharide--protein glycosyltransferase	1p36.1	0.621	3.00E-04	0.0049							1
FZD7	frizzled family receptor 7	2q33	0.621	0.00443	0.0336							1
TIA1	TIA1 cytotoxic granule-associated RNA binding protein	2p13	0.621	0.00015	0.003				-0.979	2.492	5	2
TIMM17A	translocase of inner mitochondrial membrane 17 homolog A (yeast)	1q32.1	0.621	0.00195	0.0188							1
DSTNP2	destrin (actin depolymerizing factor) pseudogene 2	12p13	0.619	0.00125	0.0137							1
FAM20C	family with sequence similarity 20, member C	7p22.3	0.619	0.01777	0.0857							1
MRPL47	mitochondrial ribosomal protein L47	3q26.33	0.619	0.00592	0.041							1
TSPYL2	TSPY-like 2	Xp11.2	0.619	0.00678	0.0449							1
ZXDB	zinc finger, X-linked, duplicated B	Xp11.21	0.619	8.00E-05	0.0018							1
MMP20	matrix metallopeptidase 20	11q22.3				1.609	0	1.00E-04	-0.703	2.592	5	2
MMP27	matrix metallopeptidase 27	11q24							-0.955	2.80429	7	1
CASP7	caspase 7, apoptosis-related cysteine peptidase	10q25	0.618	0.00414	0.0321							1
CCL3	chemokine (C-C motif) ligand 3	17q12	0.618	0.00021	0.0037							1
MMP8	matrix metallopeptidase 8 (neutrophil collagenase)	11q22.3							-0.772	2.70167	6	1
HERC5	HECT and RLD domain containing E3 ubiquitin protein ligase 5	4q22.1	0.618	0.01771	0.0855							1
MMRN1	multimerin 1	4q22							-0.9	3.30375	8	1
MMRN2	multimerin 2	10q23.2				-0.342	4.00E-05	0.0049				1
MN1	meningioma (disrupted in balanced translocation) 1	22q12.1				-0.39025	0.000816667	0.011758333				1

MNAT1	menage a trois homolog 1, cyclin H assembly factor (Xenopus laevis)	14q23							-0.97	2.93667	6	1
MND1	meiotic nuclear divisions 1 homolog (S. cerevisiae)	4q31.3				-0.344	0.00684	0.0465				1
MNDA	myeloid cell nuclear differentiation antigen	1q22							-0.968	3.008	5	1
MNS1	meiosis-specific nuclear structural 1	15q21.3							-0.663	2.40333	6	1
MNT	MAX binding protein	17p13.3				-0.51075	0.0013775	0.01675				1
SEL1L	sel-1 suppressor of lin-12-like (C. elegans)	14q31	0.618	0.00386	0.0305							1
MOB3A	MOB kinase activator 3A	19p13.3	-0.399	0.00408	0.0317							1
CHPF2	chondroitin polymerizing factor 2	7q36.1	0.617	0.00055	0.0077							1
MOB4	MOB family member 4, phocein	2q33.1	-0.608	0.00835	0.0516							1
MOBKL1A	Data not found					-0.371	0.00644	0.0428				1
MOBKL2A	Data not found					-0.4584	0.000526	0.0127				1
MOBKL2B	Data not found					-0.353285714	0.001981429	0.021285714				1
MOBP	myelin-associated oligodendrocyte basic protein	3p22.1							-0.958	3.398	5	1
MOCOS	molybdenum cofactor sulfurase	18q12				-0.3615	0.010985	0.0589				1
CXorf56	chromosome X open reading frame 56	Xq23	0.617	0.00379	0.0302	0.37	0.00278	0.0289				2
LY6E	lymphocyte antigen 6 complex, locus E	8q24.3	0.617	0.02812	0.1156							1
MSANTD3	Myb/SANT-like DNA-binding domain containing 3	9q31.1	0.617	0.00011	0.0024							1
MON1A	MON1 homolog A (yeast)	3p21.31				-0.4025	0.004735	0.0355				1
MON1B	MON1 homolog B (yeast)	16q23.1				-0.393	0.00203	0.0247				1
MTRR	5-methyltetrahydrofolate-homocysteine methyltransferase reductase	5p15.31	0.617	2.00E-05	7.00E-04	0.413	0.00051	0.0128				2
MORC3	MORC family CW-type zinc finger 3	21q22.13	-0.688	0.00039	0.006	-0.409	0.003444167	0.025325				2
MORC4	MORC family CW-type zinc finger 4	Xq22.3							-0.836	2.594	5	1
PTCD3	pentatricopeptide repeat domain 3	2p11.2	0.617	0.00146	0.0153							1
SNRPE	small nuclear ribonucleoprotein polypeptide E	1q32	0.617	0.01073	0.0614							1
MORN1	MORN repeat containing 1	1p36.33-p36.32				-0.469	9.00E-05	0.0056				1
MORN4	MORN repeat containing 4	10q24.2				-0.356	0.00076	0.0156				1
MOSPD1	motile sperm domain containing 1	Xq26.3	-0.653	0.01356	0.0715							1
MOSPD2	motile sperm domain containing 2	Xp22.2				0.413	0.00458	0.0374				1
MOSPD3	motile sperm domain containing 3	7q22	-0.579	0.00261	0.0231							1
MOV10	Mov10, Moloney leukemia virus 10, homolog (mouse)	1p13.2				-0.323	0.00125	0.0195				1
MOV10L1	Mov10l1, Moloney leukemia virus 10-like 1, homolog (mouse)	22q13.33				-0.417	0.001251667	0.015533333				1
MOXD1	monooxygenase, DBH-like 1	6q23.2	-1.302	0.02371	0.1033							1
MPC1	Data not found		-0.846	0.00111	0.0126							1
MPDZ	multiple PDZ domain protein	9p23							-0.916	3.48	8	1
MPG	N-methylpurine-DNA glycosylase	16p13.3				-0.377	0.00939	0.0553				1
MPHOSPH10	M-phase phosphoprotein 10 (U3 small nucleolar ribonucleoprotein)	2p13.3							-0.598	2.70333	6	1
MPHOSPH9	M-phase phosphoprotein 9	12q24.31				-0.428	0.00227	0.0261				1
MPL	myeloproliferative leukemia virus oncogene	1p34	-0.214	0.01459	0.0751							1
TRIM27	tripartite motif containing 27	6p22	0.617	0.01304	0.0698							1
MPND	MPN domain containing	19p13.3				-0.3635	0.0063	0.03825				1
MPP2	membrane protein, palmitoylated 2 (MAGUK p55 subfamily member 2)	17q12-q21				-0.383333333	0.002946667	0.027633333				1
MPP3	membrane protein, palmitoylated 3 (MAGUK p55 subfamily member 3)	17q21.31				-0.326	0.00368	0.0334				1
MPP4	membrane protein, palmitoylated 4 (MAGUK p55 subfamily member 4)	2q33.2							-0.809	2.935	6	1
MPP5	membrane protein, palmitoylated 5 (MAGUK p55 subfamily member 5)	14q23.3				0.398	0.00107	0.0182				1
MPP6	membrane protein, palmitoylated 6 (MAGUK p55 subfamily member 6)	7p15				0.386	0.0054	0.0408				1
MPP7	membrane protein, palmitoylated 7 (MAGUK p55 subfamily member 7)	10p12.1	-2.238	0	1.00E-04	-0.328	0.004473333	0.036133333				2
MPPE1	metallophosphoesterase 1	18p11.21				-0.337	0.00029	0.0102				1
MPPED1	metallophosphoesterase domain containing 1	22q13.31				-0.405307692	0.001055385	0.012376923				1
MPPED2	metallophosphoesterase domain containing 2	11p13	-0.775	7.00E-05	0.0017				0.871	2.896	5	2
MPRIP	myosin phosphatase Rho interacting protein	17p11.2	-0.596	0.0048	0.0355	-0.356307692	0.004660769	0.028961538				2
MPST	mercaptopyruvate sulfurtransferase	22q13.1	-0.289	0.0124	0.0677	-0.452	0.00064	0.0143				2
MPZ	myelin protein zero	1q23.3							-1.046	2.86	8	1
ADAMTS6	ADAM metallopeptidase with thrombospondin type 1 motif, 6	5q12	0.616	0.00021	0.0038							1
MPZL2	myelin protein zero-like 2	11q24	-1.665	1.00E-05	4.00E-04							1
MPZL3	myelin protein zero-like 3	11q23.3	-0.871	0.00289	0.0249							1
MR1	major histocompatibility complex, class I-related	1q25.3							-0.837	2.862	5	1
MRAP	melanocortin 2 receptor accessory protein	21q22.1				-0.415	2.00E-05	0.0036				1
CCL4	chemokine (C-C motif) ligand 4	17q12	0.616	6.00E-05	0.0015							1
MRC1	mannose receptor, C type 1	10p12.33				-0.391	0.00159	0.0219				1
MRC1L1	mannose receptor, C type 1-like 1	10p12.33				-0.391	0.00159	0.0219				1
MRC2	mannose receptor, C type 2	17q23.2				-0.306	0.01651	0.0762				1
FUT4	fucosyltransferase 4 (alpha (1,3) fucosyltransferase, myeloid-specific)	11q21	0.616	0.00015	0.003							1
MREG	melanoregulin	2q35	-1.369	3.00E-05	9.00E-04							1
MRFAP1L1	Morf4 family associated protein 1-like 1	4p16.1							-0.522	2.496	5	1
RASSF8	Ras association (RalGDS/AF-6) domain family (N-terminal) member 8	12p12.3	0.616	0.013	0.0698	0.498	0.023304	0.0896				2
MRGPRX1	MAS-related GPR, member X1	11p15.1	-0.341	0.01231	0.0673							1
MRGPRX2	MAS-related GPR, member X2	11p15.1							-0.851	3.09286	7	1
MRGPRX3	MAS-related GPR, member X3	11p15.1	-0.5725	0.0032	0.0263							1
MRI1	methylthioribose-1-phosphate isomerase homolog (S. cerevisiae)	19p13.2				-0.43375	0.0043625	0.0326				1

MRM1	mitochondrial rRNA methyltransferase 1 homolog (S. cerevisiae)	17q12				-0.308	0.00324	0.0312				1
MRO	maestro	18q21							-0.84	2.88875	8	1
MROH6	Data not found		-0.8	0.02761	0.1141							1
MRPL13	In multiple Geneids					0.35	0.00653	0.04485				1
RGPD5	RANBP2-like and GRIP domain containing 5	2q13	0.616	0.009855	0.0477				-0.812	3.706	5	2
RNF19A	ring finger protein 19A, E3 ubiquitin protein ligase	8q22	0.616	0.02286	0.101							1
XPNPEP1	X-prolyl aminopeptidase (aminopeptidase P) 1, soluble	10q25.3	0.616	0.00085	0.0104							1
MRPL22	In multiple Geneids					-0.31	0.00054	0.0133				1
MRPL23	mitochondrial ribosomal protein L23	11p15.5				-0.409	0.00545	0.041	0.607	2.31214	7	2
MRPL27	In multiple Geneids					-0.36	0.00138	0.0205				1
MRPL28	In multiple Geneids					-0.747	0.00061	0.014				1
MRPL3	mitochondrial ribosomal protein L3	3q21-q23							-0.582	2.51167	6	1
MRPL30	In multiple Geneids								-0.673	2.6	7	1
C15orf57	chromosome 15 open reading frame 57	15q15.1	0.615	0.01068	0.0613							1
C5orf22	chromosome 5 open reading frame 22	5p13.3	0.615	0.00745	0.048							1
MRPL4	In multiple Geneids		-0.415	0.01384	0.0726	-0.399666667	0.003406667	0.030833333				2
MRPL40	mitochondrial ribosomal protein L40	22q11.2	-0.412	0.00961	0.0568							1
MRPL41	mitochondrial ribosomal protein L41	9q34.3							0.959	3.134	5	1
MRPL43	mitochondrial ribosomal protein L43	10q24.31				-0.38	0.00105	0.0181	0.609	2.5125	6	2
HELLS	helicase, lymphoid-specific	10q24.2	0.615	0.03928	0.1438							1
HOXB3	homeobox B3	17q21.3	0.615	0.04103	0.1481							1
KIAA1731	KIAA1731	11q21	0.615	2.00E-04	0.0036							1
MRPL52	In multiple Geneids					-0.303	0.0129	0.0661				1
MRPL54	mitochondrial ribosomal protein L54	19p13.3	-0.26	0.03051	0.1221	-0.4655	0.005435	0.0375				2
MRPS12	mitochondrial ribosomal protein S12	19q13.1-q13.2							-0.556	2.55	5	1
TMED4	transmembrane emp24 protein transport domain containing 4	7p13	0.615	0.00016	0.0031							1
TNNC1	troponin C type 1 (slow)	3p21.1	0.615	0.02354	0.1029							1
MRPS24	mitochondrial ribosomal protein S24	7p14	-0.314	0.01842	0.0879							1
MRPS25	In multiple Geneids					-0.338	3.00E-05	0.0044				1
MRPS27	In multiple Geneids		-0.427	0.00746	0.048	-0.343	3.00E-04	0.0103				2
BATF2	basic leucine zipper transcription factor, ATF-like 2	11q13.1	0.614	0.01167	0.0649							1
CTPS2	CTP synthase 2	Xp22	0.614	0.0082	0.051							1
MRPS36	mitochondrial ribosomal protein S36	5q13.2				-0.37	0.001	0.01635				1
MRPS5	mitochondrial ribosomal protein S5	2p11.2-q11.2							-0.886	2.73667	6	1
MRPS6	mitochondrial ribosomal protein S6	21q22.11				-0.3415	0.013965	0.0633				1
MRPS9	mitochondrial ribosomal protein S9	2q12.1				0.376	5.00E-05	0.005				1
MRRF	In multiple Geneids					0.317	7.00E-04	0.0149				1
MRS2L	reserved								-0.65	2.37667	6	1
KCND2	potassium voltage-gated channel, Shal-related subfamily, member 2	7q31	0.614	0.01869	0.0888	0.312666667	0.001056667	0.013966667				2
MS4A1	membrane-spanning 4-domains, subfamily A, member 1	11q12							-0.927	2.67	7	1
MS4A2	membrane-spanning 4-domains, subfamily A, member 2	11q12-q13							-0.907	3.432	5	1
ORMDL3	ORM1-like 3 (S. cerevisiae)	17q12	0.614	0.01312	0.07							1
MS4A6A	membrane-spanning 4-domains, subfamily A, member 6A	11q12.1							-0.929	2.84	5	1
MS4A7	In multiple Geneids								-0.828	3.166	5	1
RSF1	remodeling and spacing factor 1	11q14.1	0.614	0.00012	0.0025							1
MS4A8B	membrane-spanning 4-domains, subfamily A, member 8B	11q12.2							-0.848	3.07	5	1
SLC25A24	solute carrier family 25 (mitochondrial carrier; phosphate carrier), member 24	1p13.3	0.614	0.00017	0.0033							1
USP18	ubiquitin specific peptidase 18	22q11.21	0.6135	0.03084	0.12265							1
GIMAP2	GTPase, IMAP family member 2	7q36.1	0.613	0.02671	0.112				-0.844	3.08833	6	2
ARID5A	AT rich interactive domain 5A (MRF1-like)	2q11.2	0.612	0.00202	0.0192							1
MSGN1	mesogenin 1	2p24.2	-0.331	0.00678	0.0449							1
C1orf198	chromosome 1 open reading frame 198	1q42.2	0.612	0.00134	0.0144							1
MSH3	mutS homolog 3 (E. coli)	5q11-q12				-0.3365	0.001195	0.01885				1
ENGASE	endo-beta-N-acetylglucosaminidase	17q25.3	0.612	0.00908	0.0546							1
MAST3	microtubule associated serine/threonine kinase 3	19p13.11	0.612	0.00026	0.0045							1
MSI1	musashi homolog 1 (Drosophila)	12q24							0.636	2.58571	7	1
ARHGAP11B	In multiple Geneids		0.611	0.04772	0.1625							1
PANX1	pannexin 1	11q21	0.611	0.00051	0.0073							1
MSMB	microseminoprotein, beta-	10q11.2							-0.962	3.40833	6	1
MSMO1	methylsterol monooxygenase 1	4q32-q34	-0.956	0.00635	0.0429							1
MSMP	microseminoprotein, prostate associated	9p13.3				-0.424	0.00015	0.0076				1
ZNF704	zinc finger protein 704	8q21.13	0.611	0.03857	0.1424	0.362	1.00E-05	0.003				2
MSRA	methionine sulfoxide reductase A	8p23.1				0.461	0.03946	0.127866667				1
MSRB3	methionine sulfoxide reductase B3	12q14.3				0.343	0.00362	0.0331				1
MST150	Data not found								-0.783	2.97	7	1
MST4	serine/threonine protein kinase MST4	Xq26.2				0.31	0.0035	0.03135				1
BIRC2	baculoviral IAP repeat containing 2	11q22	0.61	0.00075	0.0095							1
TMEM182	transmembrane protein 182	2q12.1	0.61	0.00536	0.0383	0.35275	0.0017425	0.020525				2
MSTP9	Data not found					-0.348	0.01886	0.0823				1

UNC5C	unc-5 homolog C (C. elegans)	4q21-q23	0.61	0.00244	0.022							1
MT1B	metallothionein 1B	16q13	-0.319	0.01248	0.068							1
MT1DP	In multiple Geneids		-0.494	0.02068	0.0948							1
MT1E	metallothionein 1E	16q13	-0.68	0.02576	0.1094							1
MT1G	metallothionein 1G	16q13	-1.071	0.00323	0.027							1
MT1H	metallothionein 1H	16q13	-0.88	0.0074	0.0477							1
MT1HL1	Data not found		-0.418	0.0217	0.0979							1
MT1IP	metallothionein 1I, pseudogene	16q13	-0.362	0.00844	0.052							1
MT1JP	metallothionein 1J, pseudogene	16q13	-0.608	0.00529	0.0379							1
MT1L	metallothionein 1L (gene/pseudogene)	16q13	-0.584	0.01783	0.0859							1
MT1M	metallothionein 1M	16q13	-0.956	0.00761	0.0486							1
MT1P3	metallothionein 1 pseudogene 3	20q11.2	-0.451	0.04901	0.1651							1
MT1X	metallothionein 1X	16q13	-1.456	1.00E-05	4.00E-04							1
MT2A	metallothionein 2A	16q13	-0.453	0.04336	0.1532							1
MTA1	metastasis associated 1	14q32.3				-0.352	0.00044	0.0122				1
MTA2	metastasis associated 1 family, member 2	11q12-q13.1	-0.36	0.01393	0.0729							1
MTAP	methylthioadenosine phosphorylase	9p21				-0.362666667	0.002113333	0.022766667				1
CLDN2	claudin 2	Xq22.3-q23	0.609	0.00841	0.0519							1
CLTC-IT1	CLTC intronic transcript 1 (non-protein coding)	17q23.2	0.609	0.02397	0.1041							1
HIST1H2AK	histone cluster 1, H2ak	6p22.1	0.609	0.03196	0.1257							1
MTERFD2	MTERF domain containing 2	2q37.3							-0.86	2.57	6	1
MTF1	metal-regulatory transcription factor 1	1p33	-1.184	0	2.00E-04							1
LTBP3	latent transforming growth factor beta binding protein 3	11q13.1	0.609	0.01694	0.083							1
MTFR1L	Data not found		-0.256	0.02494	0.1069							1
PFDN6	prefoldin subunit 6	6p21.3	0.609	0.03713	0.1388							1
MTG1	mitochondrial GTPase 1 homolog (S. cerevisiae)	10q26.3				-0.402	0.00123	0.0194				1
TM2D3	TM2 domain containing 3	15q26.3	0.609	0.00179	0.0177							1
MTHFD1	methylenetetrahydrofolate dehydrogenase (NADP+ dependent) 1, methenylte	14q24				-0.3735	0.00356	0.028225				1
XPO4	exportin 4	13q11	0.609	0.00067	0.0089				-0.852	3.43857	7	2
ZNF28	zinc finger protein 28	19q13.41	0.609	0.00842	0.0519							1
MTHFD2L	methylenetetrahydrofolate dehydrogenase (NADP+ dependent) 2-like	4q13.3							-0.852	2.60667	6	1
MTHFR	methylenetetrahydrofolate reductase (NAD(P)H)	1p36.3				-0.388666667	0.007946667	0.0483				1
BST1	bone marrow stromal cell antigen 1	4p15	0.608	0.02052	0.0943							1
MTM1	myotubularin 1	Xq28				0.343	0.00256	0.0277				1
MTMR1	myotubularin related protein 1	Xq28				0.304	0.00655	0.0454				1
MTMR10	myotubularin related protein 10	15q13.3	-0.909	0.00048	0.0069							1
PALM2-AKAP2	PALM2-AKAP2 readthrough	9q31.3	0.608	0.00417	0.0322							1
MTMR14	myotubularin related protein 14	3p26				-0.405222222	0.005047778	0.030066667				1
MTMR2	myotubularin related protein 2	11q22				0.312	0.00014	0.0075				1
MTMR3	myotubularin related protein 3	22q12.2	-0.729	2.50E-05	7.00E-04	-0.345666667	0.003786667	0.030166667				2
PARP12	poly (ADP-ribose) polymerase family, member 12	7q34	0.608	0.00922	0.0553							1
MTMR7	myotubularin related protein 7	8p22				0.644	8.00E-05	0.006				1
MTMR8	myotubularin related protein 8	Xq11.2				0.316	0.00243	0.0271	-1.015	2.592	5	2
MTMR9L	Data not found					-0.3905	0.004295	0.03435				1
PIK3R3	phosphoinositide-3-kinase, regulatory subunit 3 (gamma)	1p34.1	0.608	0.0067	0.0445							1
MTNR1B	melatonin receptor 1B	11q21-q22				-0.644	4.00E-05	0.0046				1
MTO1	mitochondrial translation optimization 1 homolog (S. cerevisiae)	6q13				-0.384	0.00012	0.0071				1
MTOR	mechanistic target of rapamycin (serine/threonine kinase)	1p36.2				-0.380941176	0.005543529	0.035035294				1
MTP18	Data not found					-0.377	0.00033	0.0108				1
PRAP1	proline-rich acidic protein 1	10q26.3	0.608	0.00356	0.0289							1
RPN2	ribophorin II	20q12-q13.1	0.608	0.00055	0.0077							1
SEC62	SEC62 homolog (S. cerevisiae)	3q26.2	0.608	0.01023	0.0595							1
MTSS1	metastasis suppressor 1	8p22	-2.07	0	0							1
MTSS1L	metastasis suppressor 1-like	16q22.1	-0.433	0.032985	0.1269							1
MTTP	microsomal triglyceride transfer protein	4q24							-0.815	2.95125	8	1
MTUS2	microtubule associated tumor suppressor candidate 2	13q12.3	-0.203	0.04393	0.1544							1
TMEM126A	transmembrane protein 126A	11q14.1	0.608	0.01867	0.0887				-0.71	2.81667	6	2
MTX3	metaxin 3	5q14.1	-0.527	0.00807	0.0505							1
ALMS1	Alstrom syndrome 1	2p13	0.607	0.00131	0.0142	0.303	0.00177	0.0231	-0.826	3.00167	6	3
DNAJC2	DnaJ (Hsp40) homolog, subfamily C, member 2	7q22	0.607	0.00027	0.0045							1
MUC15	In multiple Geneids		-4.129	0	0							1
MUC16	mucin 16, cell surface associated	19p13.2				-0.393222222	0.009597778	0.049018519				1
FN3K	fructosamine 3 kinase	17q25.3	0.607	0.03868	0.1426							1
MUC19	mucin 19, oligomeric	12q12	-0.282	0.0071	0.0463							1
KIAA1804	mixed lineage kinase 4	1q42	0.607	0.00515	0.0372							1
MUC20	mucin 20, cell surface associated	3q29				-0.38875	0.0026225	0.02575				1
MUC21	mucin 21, cell surface associated	6p21.32	-5.4965	5.00E-06	0.00015							1
RCBTB1	regulator of chromosome condensation (RCC1) and BTB (POZ) domain contair	13q14	0.607	0.00398	0.0312							1
MUC4	mucin 4, cell surface associated	3q29							0.734	2.40429	7	1

MUC5AC	mucin 5AC, oligomeric mucus/gel-forming	11p15.5				-0.42	0.002995	0.0288				1
RFTN2	raftlin family member 2	2q33.1	0.607	0.02741	0.1136							1
MUC6	mucin 6, oligomeric mucus/gel-forming	11p15.5				-0.353	0.003365	0.02845				1
MUC7	mucin 7, secreted	4q13.3							-0.782	3.05286	7	1
MUCDHL	Data not found								0.978	3.41688	8	1
MUL1	mitochondrial E3 ubiquitin protein ligase 1	1p36.12	-0.388	0.00217	0.0202							1
MUM1	In multiple Geneids								1.121	2.774	5	1
MUSTN1	musculoskeletal, embryonic nuclear protein 1	3p21.1				-0.342	4.00E-04	0.0117				1
MUT	methylmalonyl CoA mutase	6p12.3				0.312	0.01186	0.063	-0.851	3.002	5	2
MVB12A	Data not found		-0.629	0.00108	0.0123							1
MVD	mevalonate (diphospho) decarboxylase	16q24.3	-0.282	0.03569	0.1351							1
MVK	mevalonate kinase	12q24	-0.839	0.00082	0.0102							1
XPO5	In multiple Geneids		0.607	0.04648	0.1598							1
MX1	myxovirus (influenza virus) resistance 1, interferon-inducible protein p78 (mou	21q22.3				-0.3705	0.003405	0.02635				1
CLN6	In multiple Geneids		0.606	0.00187	0.0182							1
MXD1	MAX dimerization protein 1	2p13-p12	-1.635	0.00084	0.0104							1
MXD3	MAX dimerization protein 3	5q35.3				-0.354	6.00E-05	0.0054				1
MXD4	MAX dimerization protein 4	4p16.3	-0.312	0.01468	0.0754	-0.432	0.00089	0.0147				2
MXI1	MAX interactor 1	10q24-q25	-0.69	3.00E-04	0.0049							1
MXRA5	In multiple Geneids								-0.643	2.394	5	1
MXRA7	In multiple Geneids					-0.35125	0.007825	0.043275				1
CMSS1	Data not found		0.606	0.01062	0.061							1
ITGAM	integrin, alpha M (complement component 3 receptor 3 subunit)	16p11.2	0.606	0.01392	0.0728							1
MYBBP1A	MYB binding protein (P160) 1a	17p13.3				-0.4465	0.00233	0.02315				1
SLC44A1	solute carrier family 44, member 1	9q31.2	0.606	0.01424	0.0739							1
MYBPC1	myosin binding protein C, slow type	12q23.2				0.565	0	0.0012				1
MYBPC2	myosin binding protein C, fast type	19q13.33	-0.337	9.00E-04	0.0109	-0.407	0.002703333	0.025133333				2
MYBPC3	myosin binding protein C, cardiac	11p11.2				-0.362	0.00221	0.0258				1
MYCBP	c-myc binding protein	1p33-p32.2							-0.825	2.974	5	1
MYCBP2	MYC binding protein 2, E3 ubiquitin protein ligase	13q22	-1.03	0	2.00E-04							1
MYCL	Data not found		-0.577	1.00E-05	3.00E-04							1
MYCL1	v-myc myelocytomatosis viral oncogene homolog 1, lung carcinoma derived (a	1p34.2				-0.385	0.00133	0.0201				1
TMC4	transmembrane channel-like 4	19q13.42	0.606	0.02255	0.1001							1
MYD88	myeloid differentiation primary response gene (88)	3p22	-0.456	0.02385	0.1037							1
MYH1	myosin, heavy chain 1, skeletal muscle, adult	17p13.1							-0.778	2.64	5	1
MYH10	myosin, heavy chain 10, non-muscle	17p13				-0.383111111	0.001786667	0.015011111				1
MYH11	myosin, heavy chain 11, smooth muscle	16p13.11				-0.406	0.00033	0.0102				1
MYH13	myosin, heavy chain 13, skeletal muscle	17p13							-0.82	2.762	5	1
MYH14	In multiple Geneids					-0.382	0.0026475	0.024966667				1
MYH16	In multiple Geneids		-0.242	0.00774	0.0491	-0.3945	0.019435	0.07495				2
MYH2	myosin, heavy chain 2, skeletal muscle, adult	17p13.1	-0.281	0.00848	0.0521							1
MYH3	myosin, heavy chain 3, skeletal muscle, embryonic	17p13.1				-0.332	0.00234	0.0266				1
MYH7	myosin, heavy chain 7, cardiac muscle, beta	14q12				-0.317666667	9.00E-04	0.015533333				1
MYH8	myosin, heavy chain 8, skeletal muscle, perinatal	17p13.1							-1.05	3.84125	8	1
TOR3A	In multiple Geneids		0.606	0.00019	0.0036							1
MYL1	myosin, light chain 1, alkali; skeletal, fast	2q33-q34							-0.886	3.215	8	1
MYL10	myosin, light chain 10, regulatory	7q22.1				-0.335	0.02245	0.0913				1
MYL2	myosin, light chain 2, regulatory, cardiac, slow	12q24.11	-0.297	0.01111	0.0627				0.664	2.646	5	2
MYL5	myosin, light chain 5, regulatory	4p16.3	-0.284	0.00404	0.0315							1
MYLK	myosin light chain kinase	3q21				0.344	0.00517	0.0399				1
MYLK3	myosin light chain kinase 3	16q11.2				-0.3385	0.000935	0.01455				1
MYLK4	myosin light chain kinase family, member 4	6p25.2	-0.344	0.00222	0.0205	-0.396	0.00038	0.0114				2
MYLPF	myosin light chain, phosphorylatable, fast skeletal muscle	16p11.2	-0.232	0.04526	0.1575				0.625	2.512	5	2
DCTPP1	dCTP pyrophosphatase 1	16p11.2	0.605	0.03567	0.1351							1
MYO15A	myosin XVA	17p11.2				-0.370333333	0.002733333	0.027416667				1
GTPBP4	GTP binding protein 4	10p15-p14	0.605	0.00202	0.0192							1
MYO18A	myosin XVIIIA	17q11.2	-0.337	0.03207	0.1259							1
MYO18B	myosin XVIIIB	22q12.1				-0.371038462	0.000276154	0.007930769				1
MYO19	myosin XIX	17q12	-0.417	0.00968	0.0572	-0.328666667	0.003156667	0.030333333				2
PRR5L	proline rich 5 like	11p13-p12	0.605	0.00825	0.0512	0.345	4.00E-05	0.0047				2
RTP4	receptor (chemosensory) transporter protein 4	3q27.3	0.605	0.00662	0.0442							1
MYO1C	myosin IC	17p13.3				-0.496	0.01648	0.0761				1
MYO1D	myosin ID	17q11-q12				-0.3295	0.001365	0.01885				1
TOR1A	torsin family 1, member A (torsin A)	9q34	0.605	0.0015	0.0155							1
MYO1F	myosin IF	19p13.3-p13.2				-0.4582	0.005090667	0.033406667				1
MYO1H	In multiple Geneids		-0.211	0.03576	0.1353							1
MYO5A	myosin VA (heavy chain 12, myoxin)	15q21				-0.351	0.0058	0.0424				1
MYO5B	myosin VB	18q21	-0.594	0.0134	0.071	-0.350222222	0.006973889	0.039894444				2
HDGF	hepatoma-derived growth factor	1q21-q23	0.604	8.00E-05	0.0019							1

MYO6	myosin VI	6q13				-0.371	2.00E-05	0.004				1
AMZ2P1	archaelysin family metallopeptidase 2 pseudogene 1	17q24.1	0.603	0.04626	0.1595							1
BCL9	B-cell CLL/lymphoma 9	1q21	0.603	0.0232	0.1019	0.366	0.00249	0.0274				2
MYO9A	myosin IXA	15q22-q23	-0.36	0.02738	0.1136							1
HS2ST1	heparan sulfate 2-O-sulfotransferase 1	1p22.3	0.603	0.00268	0.0236							1
MYOCD	myocardin	17p11.2				-0.338714286	0.001451429	0.016942857				1
MYOF	myoferlin	10q24				-0.357166667	0.004198333	0.029316667				1
MYOM1	myomesin 1, 185kDa	18p11.31				-0.556	0.00481	0.0384				1
TRAM1	translocation associated membrane protein 1	8q13.3	0.603	0.00249	0.0223	0.43	0.00252	0.0275				2
MYOT	In multiple Geneids								-0.937	3.06667	6	1
MYOZ1	myozenin 1	10q22.1	-1.043	0	1.00E-04	-0.398	0.00074	0.0154				2
MYOZ2	myozenin 2	4q26-q27							-0.902	3.15714	7	1
MYOZ3	myozenin 3	5q33.1				-0.341	0.00033	0.0108				1
MYPN	myopalladin	10q21.3				-0.326666667	0.000286667	0.0095				1
MYPOP	Myb-related transcription factor, partner of profilin	19q13.32	-0.253	0.00507	0.0369	-0.334	0.00919	0.0522				2
FEZ1	fasciculation and elongation protein zeta 1 (zygin I)	11q24.2	0.602	0.01161	0.0647							1
PARD3B	par-3 partitioning defective 3 homolog B (C. elegans)	2q33.3	0.602	0.00128	0.0139	0.339038462	0.001890385	0.012669231				2
MYSM1	Myb-like, SWIRM and MPN domains 1	1p32.1				-0.379	6.00E-04	0.014				1
MYST4	Data not found					-0.367	0.00116	0.013675				1
MYT1	myelin transcription factor 1	20q13.33				0.313	0.00493	0.0389				1
MYT1L	In multiple Geneids					0.315	0.00163	0.0218				1
MZF1	myeloid zinc finger 1	19q13.4				0.314	0.00436	0.0364				1
N4BP1	NEDD4 binding protein 1	16q12.1	-1.048	1.00E-05	4.00E-04							1
N4BP2	NEDD4 binding protein 2	4p14				-0.4065	0.00557	0.0313875				1
N4BP2L1	NEDD4 binding protein 2-like 1	13q13.1				0.307	0.03612	0.1216				1
STAG3L4	stromal antigen 3-like 4	7p11.2-q11.2	0.602	0.01275	0.0689							1
N4BP3	NEDD4 binding protein 3	5q35.3	-0.833	0	0	-0.455	0.00156	0.0217				2
STARD4	In multiple Geneids		0.602	0.01242	0.0677							1
NAA20	N(alpha)-acetyltransferase 20, NatB catalytic subunit	20p11.23	-0.894	0.00035	0.0055							1
ARAP3	ArfGAP with RhoGAP domain, ankyrin repeat and PH domain 3	5q31.3	0.601	0.00016	0.0032							1
NAAA	N-acylethanolamine acid amidase	4q21.1	-0.56	0.01666	0.082	-0.3505	0.00719	0.04775				2
NAALADL1	N-acetylated alpha-linked acidic dipeptidase-like 1	11q12				-0.403	0.00326	0.0313				1
NAALADL2	N-acetylated alpha-linked acidic dipeptidase-like 2	3q26.31				0.361071429	0.004128571	0.0314125	-0.822	3.27556	9	2
NABP1	Data not found		-0.631	0.01916	0.0903							1
PCDHB11	protocadherin beta 11	5q31	0.601	0.01986	0.0923				-0.793	2.99667	6	2
NACC1	nucleus accumbens associated 1, BEN and BTB (POZ) domain containing	19p13.2				-0.372	0.0079575	0.047				1
NACC2	NACC family member 2, BEN and BTB (POZ) domain containing	9q34.3				-0.334	0.00299	0.03				1
SOX4	SRY (sex determining region Y)-box 4	6p22.3	0.601	0.002	0.0191							1
OTUD7B	OTU domain containing 7B	1q21.2	0.6	0.00595	0.0411							1
NAE1	NEDD8 activating enzyme E1 subunit 1	16q22				-0.501	7.00E-05	0.0057				1
NAGA	N-acetylgalactosaminidase, alpha-	22q11				-0.4994	2.80E-05	0.0035				1
NAGK	N-acetylglucosamine kinase	2p13.3	-1.767	0	0							1
NAGLU	N-acetylglucosaminidase, alpha	17q21				-0.343	0.0144	0.0577				1
NAGPA	N-acetylglucosamine-1-phosphodiester alpha-N-acetylglucosaminidase	16p13.3				-0.451	0.004033333	0.032466667				1
NAIF1	nuclear apoptosis inducing factor 1	9q34.11				-0.364	0.007	0.0471				1
NAIP	NLR family, apoptosis inhibitory protein	5q13.2				-0.4565	0.0046975	0.033675				1
NALCN	sodium leak channel, non-selective	13q32.3				0.684333333	0.009271667	0.048016667				1
NALP14	Data not found								-0.975	2.82	5	1
NALP4	Data not found								1.213	2.605	6	1
NAMPT	nicotinamide phosphoribosyltransferase	7q22.3	-1.0365	0.000855	0.0097							1
NANOS3	nanos homolog 3 (Drosophila)	19p13.13							0.683	2.58833	6	1
NAP1L2	nucleosome assembly protein 1-like 2	Xq13	-0.347	0.01194	0.066							1
NAP1L4	nucleosome assembly protein 1-like 4	11p15.5				-0.319	0.02192	0.09				1
NAP5	Data not found					0.345823529	0.002958824	0.024082353				1
NAPA	N-ethylmaleimide-sensitive factor attachment protein, alpha	19q13.33	-0.564	0.00042	0.0063	-0.3895	0.00233	0.02265				2
CCT5	chaperonin containing TCP1, subunit 5 (epsilon)	5p15.2	0.599	0.01788	0.086							1
CIR1	corepressor interacting with RBPJ, 1	2q31.1	0.599	0.00115	0.0129							1
NAPRT1	nicotinate phosphoribosyltransferase domain containing 1	8q24.3	-0.566	0.00608	0.0417							1
NARF	nuclear prelamin A recognition factor	17q25.3				-0.322	0.004655	0.03755				1
NARFL	In multiple Geneids		-0.329	0.00119	0.0132	-0.5355	0.00027	0.00985				2
NARG1	Data not found					-0.31	0.01727	0.0782				1
NARS	asparaginyl-tRNA synthetase	18q21.31				-0.353333333	0.005736667	0.041266667				1
EPPK1	epiplakin 1	8q24.3	0.599	0.00946	0.0562							1
HPDL	4-hydroxyphenylpyruvate dioxygenase-like	1p34.1	0.599	0.02772	0.1145							1
NAT11	Data not found					-0.354	0.00346	0.0323				1
NAT15	Data not found					-0.45675	0.012545	0.059525				1
NAT2	N-acetyltransferase 2 (arylamine N-acetyltransferase)	8p22							-0.883	3.44125	8	1
NAT5	Data not found					0.326	0.0069	0.0467				1
KIAA1462	KIAA1462	10p11.23	0.599	2.00E-04	0.0036							1

NAV2	neuron navigator 2	11p15.1				0.34975	0.0061125	0.03975				1
NBAS	neuroblastoma amplified sequence	2p24				0.369538462	0.001555385	0.019476923				1
NBEA	neurobeachin	13q13				0.357727273	0.003396818	0.026586364				1
NBEAL1	neurobeachin-like 1	2q33.2				-0.438	0.02975	0.108				1
NBEAL2	neurobeachin-like 2	3p21.31	-1.175	0	0	-0.4185	0.00103	0.01445				2
NBL1	neuroblastoma, suppression of tumorigenicity 1	1p36.13				-0.3805	0.00067	0.01415				1
NBLA00301	Nbla00301	4q34.1				-0.3395	0.00762	0.04635				1
NLK	nemo-like kinase	17q11.2	0.599	0.00547	0.0388	0.343	0.00642	0.0449				2
NBR1	neighbor of BRCA1 gene 1	17q21.31				-0.3232	0.006402	0.0422				1
NBR2	neighbor of BRCA1 gene 2 (non-protein coding)	17q21				-0.366	0.00996	0.0571				1
NCALD	In multiple Geneids					0.34725	0.000164	0.005575				1
NCAM1	neural cell adhesion molecule 1	11q23.1				0.3404	0.002908	0.02654				1
NCAM2	neural cell adhesion molecule 2	21q21.1				-0.39	0.00322	0.0311				1
NCAN	neurocan	19p12				-0.403	0.0026275	0.0231125				1
RELB	v-rel reticuloendotheliosis viral oncogene homolog B	19q13.32	0.599	0.00099	0.0116							1
LEPRE1	leucine proline-enriched proteoglycan (leprecan) 1	1p34.1	0.598	0.00052	0.0074							1
NCAPG2	non-SMC condensin II complex, subunit G2	7q36.3				-0.345	0.00794	0.049425				1
PIBF1	progesterone immunomodulatory binding factor 1	13q22.1	0.598	0.00095	0.0113							1
SPRED2	sprouty-related, EVH1 domain containing 2	2p14	0.598	0.00038	0.0058							1
NCCRP1	non-specific cytotoxic cell receptor protein 1 homolog (zebrafish)	19q13.2	-3.741	0	1.00E-04							1
TXLNG	taxilin gamma	Xp22.2	0.598	0.00072	0.0093							1
NCF1	neutrophil cytosolic factor 1	7q11.23				-0.361	0.020506667	0.079466667				1
NCF1B	neutrophil cytosolic factor 1B pseudogene	7q11.23				-0.365	0.00699	0.0471				1
NCF1C	neutrophil cytosolic factor 1C pseudogene	7q11.23				-0.3515	0.01448	0.06495				1
NCF4	neutrophil cytosolic factor 4, 40kDa	22q13.1				-0.36975	0.0002275	0.0084				1
NCK1	NCK adaptor protein 1	3q21	-0.589	0.01227	0.0672							1
NCK2	NCK adaptor protein 2	2q12	-0.443	0.04708	0.1611				0.571	2.546	5	2
NCKAP1	NCK-associated protein 1	2q32	-0.288	0.0408	0.1474							1
NCKAP5	NCK-associated protein 5	2q21.2	-1.747	0	0							1
DOCK4	dedicator of cytokinesis 4	7q31.1	0.597	0.00108	0.0123	0.312	0.00078	0.0158				2
NR1H3	nuclear receptor subfamily 1, group H, member 3	11p11.2	0.597	0.00024	0.0042							1
NUP107	nucleoporin 107kDa	12q15	0.597	0.00621	0.0422							1
RECQL	RecQ protein-like (DNA helicase Q1-like)	12p12	0.597	0.00203	0.0193	0.3542	0.00615	0.03652				2
NCOA1	nuclear receptor coactivator 1	2p23							-0.819	2.78833	6	1
NCOA2	nuclear receptor coactivator 2	8q13.3	-0.54	0.00523	0.0376							1
NCOA5	nuclear receptor coactivator 5	20q12-q13.12				0.331	0.00062	0.01285				1
RNF115	ring finger protein 115	1q21.1	0.597	0.00691	0.0455							1
NCOR1	nuclear receptor corepressor 1	17p11.2				-0.3404	0.001366	0.0174				1
SMOX	spermine oxidase	20p13	0.597	0.01229	0.0672							1
NCR3	natural cytotoxicity triggering receptor 3	6p21.3	-0.201	0.03895	0.143							1
NCRNA00032	Data not found					-0.35	0.00097	0.0173				1
NCRNA00093	Data not found					-0.406	4.50E-05	0.00495				1
NCRNA00105	Data not found					-0.488	0.00044	0.01195				1
NCRNA00107	Data not found					-0.497	0.00138	0.0205				1
NCRNA00111	Data not found					-0.356	0.0010525	0.014175				1
NCRNA00152	Data not found					0.301	0.00164	0.0223				1
NCRNA00159	non-protein coding RNA 159	21q22.11				-0.3385	0.000615	0.01275				1
NCRNA00185	non-protein coding RNA 185	Yq11.222	-0.531	0.02615	0.1104							1
NDE1	nudE nuclear distribution E homolog 1 (A. nidulans)	16p13.11				-0.3455	0.001275	0.01735				1
NDEL1	nudE nuclear distribution E homolog (A. nidulans)-like 1	17p13.1	-0.826	0.00017	0.0033	-0.352	0.00109	0.0183				2
NDFIP1	Nedd4 family interacting protein 1	5q31.3				-0.319	0.017935	0.0778				1
NDFIP2	Nedd4 family interacting protein 2	13q31.1	-0.661	0.02203	0.0989							1
NDN	necdin homolog (mouse)	15q11.2-q12	-0.472	0.01023	0.0595				0.746	2.83	5	2
NDP	Norrie disease (pseudoglioma)	Xp11.4				0.325666667	0.015056667	0.069533333				1
NDRG1	N-myc downstream regulated 1	8q24.3				0.3678	0.024194	0.09418				1
NDRG2	NDRG family member 2	14q11.2	-1.96	0	0				0.75	2.715	6	2
NDRG4	NDRG family member 4	16q21-q22.1	-1.415	6.00E-05	0.0015	-0.348666667	0.00826	0.045066667				2
NDST1	N-deacetylase/N-sulfotransferase (heparan glucosaminyl) 1	5q33.1	-1.324	0	0	-0.426428571	0.000237143	0.008185714				2
NDST2	N-deacetylase/N-sulfotransferase (heparan glucosaminyl) 2	10q22	-0.419	0.00328	0.0273	-0.317	0.00049	0.0128				2
NDST4	N-deacetylase/N-sulfotransferase (heparan glucosaminyl) 4	4q26				0.334	0.00221	0.0258	-0.878	2.8975	8	2
NDUFA11	NADH dehydrogenase (ubiquinone) 1 alpha subcomplex, 11, 14.7kDa	19p13.3	-0.545	0.00019	0.0035	-0.374	0.00042	0.0119				2
NDUFA12	NADH dehydrogenase (ubiquinone) 1 alpha subcomplex, 12	12q22				-0.353333333	0.001426667	0.019866667				1
NDUFA13	NADH dehydrogenase (ubiquinone) 1 alpha subcomplex, 13	19p13.2				-0.443	2.00E-05	0.0039				1
NDUFA2	NADH dehydrogenase (ubiquinone) 1 alpha subcomplex, 2, 8kDa	5q31.2				-0.385	0.00051	0.0129				1
NDUFA3	NADH dehydrogenase (ubiquinone) 1 alpha subcomplex, 3, 9kDa	19q13.42				-0.367	0.000475	0.0126				1
NDUFA4L2	NADH dehydrogenase (ubiquinone) 1 alpha subcomplex, 4-like 2	12q13.3	-1.449	0	2.00E-04				1.036	3.036	5	2
NDUFA5	NADH dehydrogenase (ubiquinone) 1 alpha subcomplex, 5, 13kDa	7q31.33							-0.83	3.10667	6	1
NDUFA6	NADH dehydrogenase (ubiquinone) 1 alpha subcomplex, 6, 14kDa	22q13.2	-0.521	0.00089	0.0108	-0.468	2.00E-05	0.0038				2
NDUFA8	NADH dehydrogenase (ubiquinone) 1 alpha subcomplex, 8, 19kDa	9q33.2							-0.804	2.95667	6	1

NDUFA9	NADH dehydrogenase (ubiquinone) 1 alpha subcomplex, 9, 39kDa	12p13.3				0.479	0.0012	0.0192	-0.722	2.607	5	2
NDUFAB1	NADH dehydrogenase (ubiquinone) 1, alpha/beta subcomplex, 1, 8kDa	16p12.2				-0.462	0.00017	0.0081				1
NDUFAF1	NADH dehydrogenase (ubiquinone) complex I, assembly factor 1	15q11.2-q21.3	-0.518	0.00086	0.0106	-0.326	0.00693	0.0468				2
NDUFAF2	NADH dehydrogenase (ubiquinone) complex I, assembly factor 2	5q12.1				-0.325	0.00049	0.0127				1
TMEM98	transmembrane protein 98	17q11.2	0.597	0.00318	0.0267							1
WDR54	WD repeat domain 54	2p13.1	0.597	0.00216	0.0202							1
SLC12A9	solute carrier family 12 (potassium/chloride transporters), member 9	7q22	0.596	0.00145	0.0153							1
NDUFB10	NADH dehydrogenase (ubiquinone) 1 beta subcomplex, 10, 22kDa	16p13.3				-0.383	0.00444	0.0368				1
NDUFB2	NADH dehydrogenase (ubiquinone) 1 beta subcomplex, 2, 8kDa	7q34				-0.682	0.00012	0.0072				1
NDUFB3	NADH dehydrogenase (ubiquinone) 1 beta subcomplex, 3, 12kDa	2q31.3							-0.542	2.326	5	1
NDUFB5	NADH dehydrogenase (ubiquinone) 1 beta subcomplex, 5, 16kDa	3q26.33				0.312	0.00756	0.0491				1
NDUFB6	NADH dehydrogenase (ubiquinone) 1 beta subcomplex, 6, 17kDa	9p21.1							-0.888	2.97571	7	1
NDUFB8	NADH dehydrogenase (ubiquinone) 1 beta subcomplex, 8, 19kDa	10q24.31				-0.338	0.00167	0.0225				1
NDUFB9	NADH dehydrogenase (ubiquinone) 1 beta subcomplex, 9, 22kDa	8q13.3							-0.888	2.526	5	1
NDUFC1	NADH dehydrogenase (ubiquinone) 1, subcomplex unknown, 1, 6kDa	4q31.1	-0.262	0.01659	0.0818							1
NDUFS2	NADH dehydrogenase (ubiquinone) Fe-S protein 2, 49kDa (NADH-coenzyme Q	1q23							0.546	2.70667	6	1
NDUFS4	NADH dehydrogenase (ubiquinone) Fe-S protein 4, 18kDa (NADH-coenzyme Q	5q11.1	-0.498	0.00823	0.0512							1
TMEM126B	transmembrane protein 126B	11q14.1	0.596	7.00E-05	0.0017							1
NDUFS7	In multiple Geneids		-0.504	0.01105	0.0625	-0.39	0.01289	0.0615				2
NDUFS8	NADH dehydrogenase (ubiquinone) Fe-S protein 8, 23kDa (NADH-coenzyme Q	11q13	-0.176	0.02813	0.1156							1
NDUFV2	NADH dehydrogenase (ubiquinone) flavoprotein 2, 24kDa	18p11.22	-0.536	0.0071	0.0463							1
NDUFV3	NADH dehydrogenase (ubiquinone) flavoprotein 3, 10kDa	21q22.3	-0.329	0.02442	0.1053	-0.41175	0.0003325	0.0087				2
PPIH	peptidylprolyl isomerase H (cyclophilin H)	1p34.1	0.595	0.00193	0.0186							1
NEBL	nebulette	10p12							-0.957	3.60875	8	1
NECAB1	N-terminal EF-hand calcium binding protein 1	8q21.3				0.3655	0.0001725	0.00595				1
NECAB2	N-terminal EF-hand calcium binding protein 2	16q23.3				-0.465	1.00E-04	0.0066				1
RHOB	ras homolog family member B	2p24	0.595	0.01165	0.0649							1
NECAP2	NECAP endocytosis associated 2	1p36.13				-0.358	0.02288	0.0858				1
UBB	ubiquitin B	17p12-p11.2	0.595	0	2.00E-04							1
NEDD4	neural precursor cell expressed, developmentally down-regulated 4, E3 ubiqui	15q							-0.765	2.71857	7	1
ZAK	sterile alpha motif and leucine zipper containing kinase AZK	2q24.2	0.595	0.0207	0.0949	0.359	1.00E-05	0.0026				2
NEDD8	neural precursor cell expressed, developmentally down-regulated 8	14q12				-0.395	4.00E-04	0.0117				1
ZSCAN16	zinc finger and SCAN domain containing 16	6p22.1	0.595	0.00203	0.0193							1
NEFH	neurofilament, heavy polypeptide	22q12.2				-0.453333333	0.000713333	0.012266667				1
NEFL	neurofilament, light polypeptide	8p21	-1.39	7.00E-04	0.0092							1
NEFM	neurofilament, medium polypeptide	8p21	-1.078	0.00045	0.0067							1
NEIL3	nei endonuclease VIII-like 3 (E. coli)	4q34.3	-0.536	0.02059	0.0945	-0.33	0.00861	0.0528				2
NEK1	NIMA (never in mitosis gene a)-related kinase 1	4q33	-0.372	0.0498	0.1667	-0.396	0.000235	0.00935				2
NEK10	In multiple Geneids					-0.422	0.00129	0.01985				1
PRKG1	protein kinase, cGMP-dependent, type I	10q11.2	0.594	0.0218	0.0982	0.3725	0.0071	0.0462				2
TREM2	triggering receptor expressed on myeloid cells 2	6p21.1	0.594	0.00644	0.0433							1
ZNF318	zinc finger protein 318	6pter-p12.1	0.594	0.01549	0.0782	0.372	0.03847	0.1265				2
ARSB	arylsulfatase B	5q11-q13	0.593	0.01579	0.0792				-0.833	3.16889	9	2
MRPS23	In multiple Geneids		0.593	0.02769	0.1144							1
NEK7	NIMA (never in mitosis gene a)-related kinase 7	1q31.3				0.356684211	0.001466842	0.0184	-0.95	3.588	5	2
NELFA	Data not found		-0.238	0.02392	0.1039							1
SIGIRR	single immunoglobulin and toll-interleukin 1 receptor (TIR) domain	11p15.5	0.593	0.00072	0.0093							1
NELL2	NEL-like 2 (chicken)	12q12				0.3075	0.001485	0.0212				1
NEMF	nuclear export mediator factor	14q22	-0.31	0.00501	0.0366							1
NEO1	neogenin 1	15q22.3-q23							0.379	2.434	5	1
NET1	neuroepithelial cell transforming 1	10p15							-0.759	2.92714	7	1
NETO1	In multiple Geneids		-0.211	0.04703	0.161	-0.308	0.00413	0.0354				2
LAT	linker for activation of T cells	16p11.2	0.592	0.00651	0.0436							1
PCYT2	phosphate cytidylyltransferase 2, ethanolamine	17q25.3	0.592	0.01869	0.0888							1
NEURL	neuralized homolog (Drosophila)	10q25.1				-0.372571429	0.003297143	0.020642857				1
NEURL1B	neuralized homolog 1B (Drosophila)	5q35.1				-0.37725	0.0004525	0.010375				1
SIX1	SIX homeobox 1	14q23.1	0.592	0.00335	0.0277							1
NEURL4	neuralized homolog 4 (Drosophila)	17p13				-0.394333333	0.016453333	0.058366667				1
NEUROD1	neuronal differentiation 1	2q32				0.323	0.00035	0.0111				1
NEUROD6	neuronal differentiation 6	7p14.3							-0.94	3.684	5	1
NEUROG1	neurogenin 1	5q23-q31	-0.313	0.0144	0.0745							1
NEUROG2	neurogenin 2	4q25				-0.342	0.02943	0.1073				1
NEXN	nexilin (F actin binding protein)	1p31.1							-0.835	2.88833	6	1
AGPAT6	In multiple Geneids		0.591	0.00697	0.0457							1
NF2	neurofibromin 2 (merlin)	22q12.2	-0.523	9.00E-05	0.0021	-0.403166667	0.004639583	0.032141667				2
NFAM1	NFAT activating protein with ITAM motif 1	22q13.2	-0.208	0.01955	0.0913	-0.4042	0.002295	0.02009				2
NFASC	neurofascin	1q32.1				0.36175	0.0079	0.03705				1
NFAT5	nuclear factor of activated T-cells 5, tonicity-responsive	16q22.1							-0.714	2.85571	7	1
NFATC1	nuclear factor of activated T-cells, cytoplasmic, calcineurin-dependent 1	18q23				-0.367	0.002974286	0.025442857				1

AKAP5	A kinase (PRKA) anchor protein 5	14q23.3	0.591	0.00287	0.0248				-0.924	3.24857	7	2
NFATC2IP	nuclear factor of activated T-cells, cytoplasmic, calcineurin-dependent 2 intera	16p11.2				-0.31	0.00062	0.0142				1
NFATC3	nuclear factor of activated T-cells, cytoplasmic, calcineurin-dependent 3	16q22.2				-0.3385	0.00845	0.043775				1
NFATC4	nuclear factor of activated T-cells, cytoplasmic, calcineurin-dependent 4	14q11.2				-0.307	0.00884	0.0535				1
AP1S3	adaptor-related protein complex 1, sigma 3 subunit	2q36.1	0.591	0.03659	0.1375							1
NFE2L2	nuclear factor (erythroid-derived 2)-like 2	2q31	-1.571	0	0							1
ASAP1	ArfGAP with SH3 domain, ankyrin repeat and PH domain 1	8q24.1-q24.2	0.591	0.00332	0.0275	0.377545455	0.021901515	0.086439394				2
NFE4	transcription factor NF-E4	7q22.1	-0.379	0.00096	0.0114							1
NFIA	nuclear factor I/A	1p31.3-p31.2	-1.141	0.00109	0.0124							1
NFIB	nuclear factor I/B	9p24.1				-0.383125	0.00519	0.0311875				1
NFIC	nuclear factor I/C (CCAAT-binding transcription factor)	19p13.3	-0.992	0.00103	0.012	-0.417555556	0.012533333	0.058694444				2
PTK2	PTK2 protein tyrosine kinase 2	8q24.3	0.591	0.00309	0.0262							1
NFIX	nuclear factor I/X (CCAAT-binding transcription factor)	19p13.3	-1.212	0.00111	0.0126	-0.373625	0.01312875	0.06235625				2
U2SURP	U2 snRNP-associated SURP domain containing	3q23	0.591	0.00236	0.0215							1
NFKBID	nuclear factor of kappa light polypeptide gene enhancer in B-cells inhibitor, de	19q13.12				-0.323	0.00281	0.029				1
ZFP36L2	zinc finger protein 36, C3H type-like 2	2p22.3-p21	0.591	1.00E-04	0.0022							1
PDGFRL	platelet-derived growth factor receptor-like	8p22-p21.3	0.59	0.01112	0.0628							1
NFX1	nuclear transcription factor, X-box binding 1	9p13.3	-0.376	0.00277	0.0242	-0.380454545	0.000935455	0.015				2
NFXL1	In multiple Geneids					0.336	0.01115	0.0608	-0.768	2.575	6	2
NFYA	nuclear transcription factor Y, alpha	6p21.3				0.346	0.004115	0.035				1
RNU5E-1	RNA, U5E small nuclear 1	1p36.22	0.59	0.02464	0.106							1
NFYC	nuclear transcription factor Y, gamma	1p32	-0.395	0.01676	0.0824							1
NGB	neuroglobin	14q24.3				-0.316	0.00363	0.0331				1
NGEF	neuronal guanine nucleotide exchange factor	2q37				0.441	5.00E-05	0.0053				1
NGFR	nerve growth factor receptor	17q21-q22	-0.41	0.00274	0.0239							1
ALG8	asparagine-linked glycosylation 8, alpha-1,3-glucosyltransferase homolog (S. c	11q14.1	0.589	0.00468	0.0348							1
NHEDC2	Data not found					-0.303	0.00244	0.0271				1
DGKE	diacylglycerol kinase, epsilon 64kDa	17q22	0.589	0.00089	0.0108							1
NHLH2	nescient helix loop helix 2	1p12-p11							-0.993	3.13	7	1
SV2A	synaptic vesicle glycoprotein 2A	1q21.2	0.589	0.04343	0.1533							1
TAPBPL	TAP binding protein-like	12p13.31	0.589	0.00743	0.0479							1
NHP2	NHP2 ribonucleoprotein homolog (yeast)	5q35.3	-0.4805	0.001985	0.01825							1
NHP2L1	NHP2 non-histone chromosome protein 2-like 1 (S. cerevisiae)	22q13				-0.4245	0.00074	0.01235				1
CCDC41	coiled-coil domain containing 41	12q22	0.588	0.00073	0.0094							1
NHSL2	NHS-like 2	Xq13.1				0.319	0.000986667	0.0151				1
NICN1	nicolin 1	3p21.31				-0.526333333	0.000176667	0.007066667				1
CD34	CD34 molecule	1q32	0.588	0.0158	0.0793	0.336	0.00013	0.0073				2
ELP2	elongation protein 2 homolog (S. cerevisiae)	18q12.2	0.588	0.01086	0.0618							1
FOXJ1	forkhead box J1	17q25.1	0.588	0.00387	0.0306							1
NIFUN	Data not found								-0.832	2.958	5	1
NIM1	serine/threonine-protein kinase NIM1	5p12	-0.234	0.0312	0.1238							1
NIN	ninein (GSK3B interacting protein)	14q22.1	-0.882	9.00E-05	0.002	-0.3205	0.003635	0.027				2
SPIN3	spindlin family, member 3	Xp11.21	0.588	7.00E-05	0.0016				-0.669	2.37	5	2
NIPA2	non imprinted in Prader-Willi/Angelman syndrome 2	15q11.2				-0.449	0.01143	0.0617				1
NIPAL1	NIPA-like domain containing 1	4p12	-1.975	0	0							1
NIPAL2	NIPA-like domain containing 2	8q22.2				0.311	0.00037	0.0106				1
NIPAL3	NIPA-like domain containing 3	1p36.12-p35.1	-0.867	0	2.00E-04	-0.39	0.001735	0.019875				2
NIPAL4	NIPA-like domain containing 4	5q33.3	-1.435	0.00038	0.0059	-0.3705	0.00594	0.0335				2
NIPBL	Nipped-B homolog (Drosophila)	5p13.2				0.350733333	0.011968	0.060093333				1
NIPSNAP1	nipsnap homolog 1 (C. elegans)	22q12.2				-0.367	0.001993333	0.0222				1
NISCH	nischarin	3p21.1				-0.313	0.00168	0.0225				1
TEP1	telomerase-associated protein 1	14q11.2	0.588	0.00318	0.0267				-0.886	2.624	5	2
NKAIN1	Na+/K+ transporting ATPase interacting 1	1p35.2				-0.306	0.00553	0.0414				1
ACRBP	acrosin binding protein	12p13.31	0.587	0.00061	0.0083							1
NKAIN3	Na+/K+ transporting ATPase interacting 3	8q12.3				0.322684211	0.000301579	0.009415789				1
NKAIN4	Na+/K+ transporting ATPase interacting 4	20q13.33	-0.22	0.02972	0.1198							1
ATP11C	In multiple Geneids		0.587	0.00407	0.0317	0.316	0.00369	0.0335	-0.78	3.024	5	3
NKAPL	NFKB activating protein-like	6p22.1	-0.229	0.02669	0.1119							1
NKD1	naked cuticle homolog 1 (Drosophila)	16q12.1				-0.336666667	0.002476667	0.0263				1
NKIRAS1	NFKB inhibitor interacting Ras-like 1	3p24.2				-0.453	7.00E-04	0.015				1
NKIRAS2	NFKB inhibitor interacting Ras-like 2	17q21.2	-0.537	0.0016	0.0163							1
NKX1-2	NK1 homeobox 2	10q26.13				-0.472	0	0.0018				1
NKX2-3	NK2 homeobox 3	10q24.2	-0.221	0.04983	0.1667	-0.377	0.00787	0.0502				2
NKX2-4	In multiple Geneids		-0.289	0.04969	0.1665							1
NKX2-5	NK2 homeobox 5	5q34				-0.5095	0.00166	0.01755				1
NKX2-6	NK2 homeobox 6	8p21.2	-0.306	0.01189	0.0658							1
NLGN1	neuroligin 1	3q26.31				0.359815385	0.006859538	0.041350769				1
NLGN2	neuroligin 2	17p13.1				-0.504	3.00E-05	0.004				1
NLGN4X	neuroligin 4, X-linked	Xp22.33				0.311	0.00685	0.0465				1

NLGN4Y	In multiple Geneids		-0.51	0.00808	0.0505	-0.456	0.019281667	0.078816667				2
CD2AP	CD2-associated protein	6p12	0.587	0.00183	0.0179	0.3172	0.01013	0.05508				2
NLN	neurolysin (metallopeptidase M3 family)	5q12.3				-0.309	0.00129	0.0198				1
NLRC3	NLR family, CARD domain containing 3	16p13.3				-0.434428571	0.008688571	0.048742857				1
NLRC4	NLR family, CARD domain containing 4	2p22-p21				-0.362	0.0121	0.0637				1
NLRC5	NLR family, CARD domain containing 5	16q13				-0.393	0.0019825	0.014725				1
NLRP1	NLR family, pyrin domain containing 1	17p13.2	-0.501	0.02481	0.1065	-0.360375	0.00529125	0.0316125				2
NLRP10	NLR family, pyrin domain containing 10	11p15.4	-0.261	0.02278	0.1007							1
NLRP12	NLR family, pyrin domain containing 12	19q13.42				-0.3935	0.008635	0.0437				1
NLRP13	NLR family, pyrin domain containing 13	19q13.43	-0.213	0.03344	0.1294	-0.331	0.00161	0.0221				2
NLRP2	NLR family, pyrin domain containing 2	19q13.42				-0.3664	0.003122	0.02962				1
NLRP4	NLR family, pyrin domain containing 4	19q13.43	-0.172	0.0432	0.1529							1
NLRP5	NLR family, pyrin domain containing 5	19q13.43				-0.3751	0.006191	0.03688				1
NLRP7	NLR family, pyrin domain containing 7	19q13.42				-0.388	0.00032	0.0107				1
NLRP8	NLR family, pyrin domain containing 8	19q13.43				-0.361571429	0.000965714	0.015757143				1
NLRP9	NLR family, pyrin domain containing 9	19q13.42				-0.304	0.00288	0.0294				1
NLRX1	NLR family member X1	11q23.3	-1.419	0	0							1
HHEX	hematopoietically expressed homeobox	10q23.33	0.587	0.00688	0.0454							1
NMBR	neuromedin B receptor	6q21-qter	-0.263	0.02555	0.1088							1
LILRB3	leukocyte immunoglobulin-like receptor, subfamily B (with TM and ITIM doma	19q13.4	0.587	0.0079	0.0498							1
NME4	NME/NM23 nucleoside diphosphate kinase 4	16p13.3				-0.503	0.00595	0.043				1
NME5	NME/NM23 family member 5	5q31							-0.849	3.20667	9	1
NME6	NME/NM23 nucleoside diphosphate kinase 6	3p21				-0.3335	0.00699	0.04695				1
PLOD2	procollagen-lysine, 2-oxoglutarate 5-dioxygenase 2	3q24	0.587	0.02842	0.1164	0.314	0.0109	0.056275				2
NME9	NME/NM23 family member 9	3q22.3	-0.286	0.00874	0.0533							1
RICTOR	RPTOR independent companion of MTOR, complex 2	5p13.1	0.587	0.0041	0.0319	0.380263158	0.003942105	0.030468421				2
NMNAT1	nicotinamide nucleotide adenylyltransferase 1	1p36.22				-0.33725	0.012625	0.0589				1
NMNAT2	nicotinamide nucleotide adenylyltransferase 2	1q25							0.735	2.994	5	1
NMNAT3	nicotinamide nucleotide adenylyltransferase 3	3q23				0.362	0.000426667	0.011933333				1
NMRAL1	NmrA-like family domain containing 1	16p13.3	-0.295	0.02211	0.099	-0.51	0.00097	0.0174				2
NMRK1	nicotinamide riboside kinase 1	9q21.13	-1.629	2.00E-05	6.00E-04							1
NMS	neuromedin S	2q11.2							-0.616	2.62	6	1
NMT1	N-myristoyltransferase 1	17q21.31				-0.538	0.00082	0.0161				1
NMT2	N-myristoyltransferase 2	10p13				-0.3952	0.007191	0.04251				1
NMU	neuromedin U	4q12	-1.636	0.00014	0.0028	-0.34	0.00654	0.0454				2
NMUR1	neuromedin U receptor 1	2q37.1							0.93	3.026	5	1
NNAT	neuronatin	20q11.2-q12	-0.611	0.00026	0.0045				0.638	2.97625	8	2
SDF4	stromal cell derived factor 4	1p36.33	0.587	0.00048	0.007							1
NNT	nicotinamide nucleotide transhydrogenase	5p12				0.384785714	0.008018571	0.045171429				1
NOA1	nitric oxide associated 1	4q12	-0.376	0.01056	0.0608							1
NOB1	NIN1/RPN12 binding protein 1 homolog (S. cerevisiae)	16q22.3				-0.416666667	0.001435	0.0193				1
NOC3L	nucleolar complex associated 3 homolog (S. cerevisiae)	10q23.33				-0.337	0.00134	0.0202				1
SYBU	syntabulin (syntaxin-interacting)	8q23.2	0.587	0.04415	0.155							1
NOD2	nucleotide-binding oligomerization domain containing 2	16q21	-1.167	3.00E-05	9.00E-04							1
NODAL	nodal homolog (mouse)	10q22.1				-0.332666667	0.009706667	0.0426				1
ENY2	enhancer of yellow 2 homolog (Drosophila)	8q23.1	0.586	0.0022	0.0204							1
NOL12	nucleolar protein 12	22q13.1	-0.453	0.00017	0.0032							1
NOL4	nucleolar protein 4	18q12				0.305	0.00634	0.0446				1
NOL6	nucleolar protein family 6 (RNA-associated)	9p13.3				-0.391	0.001115	0.0181				1
LGR4	leucine-rich repeat containing G protein-coupled receptor 4	11p14-p13	0.586	0.01967	0.0917							1
NOL8	nucleolar protein 8	9q22.31							-0.493	2.505	6	1
NOL9	nucleolar protein 9	1p36.31				-0.3885	0.003125	0.0276				1
NOLC1	nucleolar and coiled-body phosphoprotein 1	10q24.32				-0.422	0.000335	0.00915				1
NOM1	nucleolar protein with MIF4G domain 1	7q36.3				-0.403	0.00307	0.0304				1
MGAM	maltase-glucoamylase (alpha-glucosidase)	7q34	0.586	0.01156	0.0645	0.43	0.01064	0.0592				2
SNRNP48	small nuclear ribonucleoprotein 48kDa (U11/U12)	6p24.3	0.586	0.00075	0.0096							1
NOP14	NOP14 nucleolar protein homolog (yeast)	4p16.3				-0.365	0.00407	0.026575				1
NOP16	NOP16 nucleolar protein homolog (yeast)	5q35.2				-0.399	4.00E-04	0.01165				1
APLP2	amyloid beta (A4) precursor-like protein 2	11q24	0.585	0.0035	0.0284	0.311	0.00104	0.0179				2
DOCK7	dedicator of cytokinesis 7	1p31.3	0.585	0.00551	0.039							1
NOS1	nitric oxide synthase 1 (neuronal)	12q24.2-q24.31	-0.904	0	1.00E-04	-0.337	3.00E-05	0.004				2
YPEL2	yippee-like 2 (Drosophila)	17q22	0.585	0.00376	0.03							1
NOS3	nitric oxide synthase 3 (endothelial cell)	7q36				-0.343	0.005075	0.0386				1
NOSIP	nitric oxide synthase interacting protein	19q13.33							-0.729	2.954	5	1
ENG	endoglin	9q34.11	0.584	0.00211	0.0199							1
NOTCH1	notch 1	9q34.3				-0.301	0.00334	0.0317				1
NOTCH2	notch 2	1p13-p11	-0.802	0.00055	0.0077							1
NOTCH2NL	notch 2 N-terminal like	1q21.2				0.317	0.00098	0.0174				1
NOTCH3	notch 3	19p13.2-p13.1	-1.049	0.00017	0.0032	-0.373142857	0.002765714	0.022885714				2

NOV	nephroblastoma overexpressed	8q24.1				0.35	0.00806	0.0509				1
NOVA1	neuro-oncological ventral antigen 1	14q							0.383	2.434	5	1
NOVA2	neuro-oncological ventral antigen 2	19q13.3				-0.319	0.00452	0.0371				1
NOX1	NADPH oxidase 1	Xq22							-0.846	3.22333	6	1
NOX3	NADPH oxidase 3	6q25.3	-0.203	0.04664	0.1602							1
PODXL2	podocalyxin-like 2	3q21.3	0.584	0.02385	0.1037							1
N-PAC	Data not found					-0.3902	0.017229	0.07175				1
NPAS1	neuronal PAS domain protein 1	19q13.2-q13.3	-0.23	0.04455	0.1558	-0.322	0.00158	0.0218				2
NPAS2	neuronal PAS domain protein 2	2q11.2	-0.616	0.03128	0.1241							1
NPAS3	neuronal PAS domain protein 3	14q12-q13				0.323	0.006478333	0.036766667				1
NPAS4	neuronal PAS domain protein 4	11q13				-0.388	0.00092	0.01635	0.91	2.56333	6	2
PSMB10	proteasome (prosome, macropain) subunit, beta type, 10	16q22.1	0.584	0.00766	0.0487							1
NPB	neuropeptide B	17q25.3	-0.277	0.01769	0.0854							1
NPBWR1	neuropeptides B/W receptor 1	8p22-q21.13							0.597	2.54	5	1
NPBWR2	neuropeptides B/W receptor 2	20q13.3							0.78	2.69	6	1
NPC1	Niemann-Pick disease, type C1	18q11-q12				-0.312	0.04639	0.142				1
RHOBTB2	Rho-related BTB domain containing 2	8p21.3	0.584	0.00433	0.0331							1
NPEPL1	aminopeptidase-like 1	20q13.32				0.341	0.00293	0.0296				1
NPEPPS	aminopeptidase puromycin sensitive	17q21	-0.9375	0.0019	0.0155	-0.4315	0.001205	0.0178				2
NPFFR1	neuropeptide FF receptor 1	10q21-q22	-0.196	0.03846	0.1421							1
NPFFR2	neuropeptide FF receptor 2	4q21				0.33	7.00E-05	0.0057				1
NPHP1	nephronophthisis 1 (juvenile)	2q13							-0.98	3.46	5	1
NPHP3	nephronophthisis 3 (adolescent)	3q22.1				0.331	0.00098	0.0174				1
NPHP4	nephronophthisis 4	1p36				-0.381846154	0.003011538	0.020046154				1
SLC25A13	solute carrier family 25 (aspartate/glutamate carrier), member 13	7q21.3	0.584	0.01397	0.073							1
NPL	N-acetylneuraminate pyruvate lyase (dihydrodipicolinate synthase)	1q25	-0.487	0.02796	0.1152							1
NPLOC4	nuclear protein localization 4 homolog (S. cerevisiae)	17qter				-0.356	0.006105	0.0436	0.818	2.638	5	2
SP110	SP110 nuclear body protein	2q37.1	0.584	0.02351	0.1028							1
NPPA	natriuretic peptide A	1p36.21				-0.37	0.01301	0.0664				1
NPPC	natriuretic peptide C	2q24-qter	-0.361	0.00235	0.0215							1
NPR1	natriuretic peptide receptor A/guanylate cyclase A (atrionatriuretic peptide re	1q21-q22							1.308	3.368	5	1
TGIF2	TGFB-induced factor homeobox 2	20q11.23	0.584	0.01833	0.0876							1
NPR3	natriuretic peptide receptor C/guanylate cyclase C (atrionatriuretic peptide re	5p14-p13				0.354	0.01216	0.0632				1
NPSR1	neuropeptide S receptor 1	7p14.3				0.322	0.00081	0.01505				1
NPTX2	neuronal pentraxin II	7q21.3-q22.1				-0.576	2.00E-04	0.0088				1
NPTXR	neuronal pentraxin receptor	22q13.1				-0.443666667	0.000216667	0.007166667				1
NQO1	NAD(P)H dehydrogenase, quinone 1	16q22.1				-0.333	0.00261	0.028				1
NQO2	NAD(P)H dehydrogenase, quinone 2	6pter-q12				-0.34	0.00496	0.035566667				1
LOC153684	uncharacterized LOC153684	5p12	0.583	0.01284	0.0692				-0.882	3.61429	7	2
NR1D1	nuclear receptor subfamily 1, group D, member 1	17q11.2				-0.481	0.00027	0.0099				1
NR1D2	nuclear receptor subfamily 1, group D, member 2	3p24.2	-0.377	0.0189	0.0895	-0.334	0.00058	0.01305				2
ATAD5	ATPase family, AAA domain containing 5	17q11.2	0.582	0.00824	0.0512							1
ATG16L1	autophagy related 16-like 1 (S. cerevisiae)	2q37.1	0.581	0.00037	0.0058							1
FHL2	four and a half LIM domains 2	2q12.2	0.581	0.04806	0.1632							1
NR2C2	nuclear receptor subfamily 2, group C, member 2	3p25				-0.35225	3.00E-04	0.010075				1
NR2C2AP	nuclear receptor 2C2-associated protein	19p13.11				-0.5025	0.00244	0.0228				1
NR2E1	nuclear receptor subfamily 2, group E, member 1	6q21				-0.357	0.00106	0.0181	0.702333333	2.76986	6.333333333	2
NR2E3	nuclear receptor subfamily 2, group E, member 3	15q22.32	-0.353	0.00102	0.0119							1
KNTC1	kinetochore associated 1	12q24.31	0.581	0.00912	0.0548							1
NR2F6	nuclear receptor subfamily 2, group F, member 6	19p13.1				-0.504	0.00072	0.011675				1
NR3C1	nuclear receptor subfamily 3, group C, member 1 (glucocorticoid receptor)	5q31.3							-0.796	3.27667	9	1
NR3C2	nuclear receptor subfamily 3, group C, member 2	4q31.1				-0.332	0.00175	0.023				1
TTC9C	tetratricopeptide repeat domain 9C	11q12.3	0.581	3.00E-05	8.00E-04							1
UBFD1	ubiquitin family domain containing 1	16p12	0.581	0.00655	0.0438							1
NR5A1	nuclear receptor subfamily 5, group A, member 1	9q33				-0.3225	0.00345	0.03225				1
DNMBP	dynamin binding protein	10q24.2	0.58	0.00408	0.0318							1
NRAS	neuroblastoma RAS viral (v-ras) oncogene homolog	1p13.2							-0.838	2.93	5	1
NRBP1	nuclear receptor binding protein 1	2p23	-0.652	0.00217	0.0203							1
NRCAM	neuronal cell adhesion molecule	7q31							-0.87	3.1	6	1
NRD1	nardilysin (N-arginine dibasic convertase)	1p32.2-p32.1				-0.396	4.00E-05	0.0049				1
NRF1	nuclear respiratory factor 1	7q32				-0.347	0.0039	0.0344				1
NRG1	neuregulin 1	8p12				0.321666667	0.02607	0.094966667				1
NRG2	neuregulin 2	5q23-q33	-0.438	0.00159	0.0162	-0.341769231	0.00551	0.031115385				2
NRG3	neuregulin 3	10q22-q23	-0.231	0.04897	0.165	-0.4775	0.00328	0.028				2
NRG4	neuregulin 4	15q24.2							-0.957	3.086	5	1
NRGN	neurogranin (protein kinase C substrate, RC3)	11q24							0.868	3.06143	7	1
NRIP1	nuclear receptor interacting protein 1	21q11.2				0.421	0.00012	0.007	-0.829	3.36	6	2
NRIP3	nuclear receptor interacting protein 3	11p15.3				-0.348	0.00041	0.0119				1
NRK	Nik related kinase	Xq22.3				0.346666667	0.001522222	0.018633333				1

SNORA3	small nucleolar RNA, H/ACA box 3	11p15	0.58	0.00241	0.0218							1
SNORD53	small nucleolar RNA, C/D box 53	2p23.2	0.58	0.04936	0.1658							1
NRTN	neurturin	19p13.3	-0.311	0.00527	0.0378	-0.481	0.00462	0.0376				2
NRXN1	neurexin 1	2p16.3				0.347819444	0.0033525	0.026238889				1
NRXN2	neurexin 2	11q13				-0.39075	0.0056775	0.036275	0.495	2.27333	6	2
NRXN3	neurexin 3	14q31				0.3815	0.00217	0.0216	-0.844	2.72	6	2
NSBP1	Data not found								-0.769	2.572	5	1
NSD1	nuclear receptor binding SET domain protein 1	5q35	-0.317	0.01405	0.0733	-0.365095238	0.002770476	0.025195238				2
NSDHL	NAD(P) dependent steroid dehydrogenase-like	Xq28	-0.77	0.00062	0.0084							1
NSF	N-ethylmaleimide-sensitive factor	17q21				-0.427	0.00421	0.0357				1
NSG1	Data not found		-1.027	0	2.00E-04							1
EPHB4	EPH receptor B4	7q22	0.579	0.00257	0.0229							1
NSMCE2	non-SMC element 2, MMS21 homolog (S. cerevisiae)	8q24.13				0.353545455	0.011292727	0.059127273				1
EXTL2	exostoses (multiple)-like 2	1p21	0.579	0.01486	0.0761							1
FURIN	furin (paired basic amino acid cleaving enzyme)	15q26.1	0.579	0.01617	0.0805							1
MTF2	metal response element binding transcription factor 2	1p22.1	0.579	0.00026	0.0045							1
NSUN5C	Data not found					-0.387	0.026	0.0996				1
NSUN6	NOP2/Sun domain family, member 6	10p12.31				-0.32	0.00692	0.0468				1
NSUN7	NOP2/Sun domain family, member 7	4p14				-0.358666667	0.00683	0.037466667				1
NT5C1A	5'-nucleotidase, cytosolic IA	1p34.3-p33	-0.273	0.01082	0.0616							1
NT5C1B	5'-nucleotidase, cytosolic IB	2p24.2				0.351	0.006225	0.03955	-0.807	2.96286	7	2
NT5C2	5'-nucleotidase, cytosolic II	10q24.32	-0.833	0.0019	0.0184	-0.35825	0.00044	0.010325				2
NT5C3	5'-nucleotidase, cytosolic III	7p14.3							-0.809	2.304	5	1
PATZ1	POZ (BTB) and AT hook containing zinc finger 1	22q12.2	0.579	0.00613	0.042							1
NT5C3L	5'-nucleotidase, cytosolic III-like	17q21.2				-0.4135	0.000785	0.01465				1
NT5DC2	5'-nucleotidase domain containing 2	3p21.1	-0.773	0.02102	0.0959							1
TAS2R31	In multiple Geneids		0.579	0.00912	0.0548							1
ADAM32	ADAM metallopeptidase domain 32	8p11.22	0.578	0.00629	0.0427							1
NT5M	5',3'-nucleotidase, mitochondrial	17p11.2	-0.306	0.00207	0.0196	-0.374333333	0.007483333	0.04245				2
NTAN1	N-terminal asparagine amidase	16p13.11	-0.545	0.00361	0.0292							1
NTF3	neurotrophin 3	12p13				0.382666667	0.00079	0.0112				1
NTF4	neurotrophin 4	19q13.3	-0.46	0.00015	0.003							1
NTM	neurotrimin	11q25				0.377	0.00219	0.0257				1
AHSA2	AHA1, activator of heat shock 90kDa protein ATPase homolog 2 (yeast)	2p15	0.578	0.00614	0.042							1
NTN1	netrin 1	17p13-p12	-0.657	0.00011	0.0024	-0.388585366	0.006113902	0.036517073				2
NTN3	netrin 3	16p13.3	-0.178	0.0349	0.1331							1
NTN4	netrin 4	12q22				-0.39	0.00236	0.0266				1
NTNG1	In multiple Geneids					0.301	0.0039	0.0344				1
NTRK1	neurotrophic tyrosine kinase, receptor, type 1	1q21-q22				-0.3665	0.016075	0.07425				1
NTRK2	neurotrophic tyrosine kinase, receptor, type 2	9q22.1	-1.075	0.00319	0.0268							1
NTRK3	neurotrophic tyrosine kinase, receptor, type 3	15q25				0.3394	0.002508	0.02594				1
NTS	neurotensin	12q21							-0.853	2.54	6	1
RRN3	RRN3 RNA polymerase I transcription factor homolog (S. cerevisiae)	16p12	0.578	0.01181	0.0655							1
NUAK2	NUAK family, SNF1-like kinase, 2	1q32.1	-0.951	0.00538	0.0384							1
HPCAL1	hippocalcin-like 1	2p25.1	0.577	0.01421	0.0738							1
NUBPL	nucleotide binding protein-like	14q12				-0.327	0.00291	0.0295				1
NUCB1	nucleobindin 1	19q13.33				-0.362	0.00124	0.0195				1
NUCB2	nucleobindin 2	11p15.1	-1.843	0	0							1
NSUN2	NOP2/Sun domain family, member 2	5p15.31	0.577	0.00316	0.0266							1
RBM39	RNA binding motif protein 39	20q11.22	0.577	0.00013	0.0027							1
NUDCD3	NudC domain containing 3	7p13-p12	-0.275	0.02749	0.1138							1
NUDT1	nudix (nucleoside diphosphate linked moiety X)-type motif 1	7p22	-0.168	0.04837	0.1639							1
NUDT10	nudix (nucleoside diphosphate linked moiety X)-type motif 10	Xp11.23							0.73	2.48	5	1
RGS16	regulator of G-protein signaling 16	1q25-q31	0.577	0.04166	0.1494							1
ZNF703	zinc finger protein 703	8p11.23	0.577	0.00592	0.041							1
NUDT2	nudix (nucleoside diphosphate linked moiety X)-type motif 2	9p13							-0.72	2.72833	6	1
NUDT4	nudix (nucleoside diphosphate linked moiety X)-type motif 4	12q21				-0.39	4.00E-05	0.0047				1
HPRT1	hypoxanthine phosphoribosyltransferase 1	Xq26.1	0.576	0.00231	0.0212							1
NUDT5	nudix (nucleoside diphosphate linked moiety X)-type motif 5	10p14							-0.733	2.62286	7	1
NUDT7	In multiple Geneids		-0.68	0.01749	0.0848							1
NUDT8	In multiple Geneids		-0.672	0.00034	0.0054							1
NUDT9	nudix (nucleoside diphosphate linked moiety X)-type motif 9	4q22.1				-0.316	0.0079	0.0503				1
DDX18	DEAD (Asp-Glu-Ala-Asp) box polypeptide 18	2q14.1	0.575	0.00646	0.0434				-0.780166667	2.917338333	6.833333333	2
LLGL2	lethal giant larvae homolog 2 (Drosophila)	17q25.1	0.575	0.01014	0.0591							1
LYPD6	LY6/PLAUR domain containing 6	2q23.2	0.575	0.03063	0.1224	0.376	0.0011975	0.014525				2
NUGGC	nuclear GTPase, germinal center associated	8p21.1	-0.319	0.01403	0.0732							1
NUMA1	nuclear mitotic apparatus protein 1	11q13	-0.278	0.03423	0.1313	-0.305	5.00E-05	0.005				2
NUMB	numb homolog (Drosophila)	14q24.3				-0.340333333	0.007345556	0.044033333				1
NUMBL	numb homolog (Drosophila)-like	19q13.13-q13.2				-0.352	0.0023	0.0263				1

MORC2	MORC family CW-type zinc finger 2	22q12.2	0.575	0.00032	0.0051							1
RHBDD2	rhomboid domain containing 2	7q11.23	0.575	0.0056	0.0395							1
TIMP4	TIMP metallopeptidase inhibitor 4	3p25	0.575	0.02704	0.1127							1
WDR35	WD repeat domain 35	2p24.1	0.575	0.00834	0.0516	0.3265	0.0051425	0.037025				2
NUP188	nucleoporin 188kDa	9q34.11				-0.334	0.01562	0.0724				1
NUP205	nucleoporin 205kDa	7q33				-0.351	0.00108	0.0183				1
NUP210	nucleoporin 210kDa	3p25.1				-0.4495	0.0006275	0.0119625				1
NUP214	nucleoporin 214kDa	9q34.1				-0.352	0.01041	0.0562				1
NUP43	In multiple Geneids					-0.3675	0.003695	0.0325				1
NUP50	nucleoporin 50kDa	22q13.31				-0.435	1.00E-05	0.0028				1
NUP62	nucleoporin 62kDa	19q13.33				-0.333	0.02335	0.0935				1
CACNA1C	calcium channel, voltage-dependent, L type, alpha 1C subunit	12p13.3	0.574	0.03522	0.1339	0.306	0.00019	0.0086				2
FCGR1B	Fc fragment of IgG, high affinity Ib, receptor (CD64)	1p11.2	0.574	0.04429	0.1554							1
NUP88	nucleoporin 88kDa	17p13.2				-0.371	0.00089	0.016033333				1
NUP98	nucleoporin 98kDa	11p15.5				-0.337125	0.0155925	0.0682875				1
GAL3ST2	galactose-3-O-sulfotransferase 2	2q37.3	0.574	0.00255	0.0228							1
GLA	galactosidase, alpha	Xq22	0.574	0.01009	0.0589				-0.618	2.57	6	2
NUSAP1	nucleolar and spindle associated protein 1	15q15.1				-0.427	0.00195	0.0242				1
MSANTD2	Myb/SANT-like DNA-binding domain containing 2	11q24.2	0.574	0.00063	0.0085							1
NUTM1	Data not found		-0.339	7.00E-04	0.0091							1
NUTM2A	Data not found		-0.227	0.0235	0.1028							1
NUTM2F	Data not found		-0.167	0.03908	0.1434							1
TMEM123	transmembrane protein 123	11q22.1	0.574	0.00164	0.0166							1
NWD1	NACHT and WD repeat domain containing 1	19p13.11				-0.42395	0.0038605	0.028445				1
NXF1	nuclear RNA export factor 1	11q12-q13				-0.326	0.020495	0.07815				1
NXF2	nuclear RNA export factor 2	Xq22.1				0.333	4.00E-05	0.0045				1
NXF2B	nuclear RNA export factor 2B	Xq22.1				0.333	4.00E-05	0.0045				1
NXF5	nuclear RNA export factor 5	Xq22							-0.963	3.32286	7	1
NXN	nucleoredoxin	17p13.3	-0.513	0.01624	0.0807	-0.437954545	0.006204318	0.036727273				2
NXNL2	nucleoredoxin-like 2	9q22.1				-0.329	0.00591	0.0429				1
BET1L	blocked early in transport 1 homolog (S. cerevisiae)-like	11p15.5	0.573	0.00294	0.0252							1
NXPH1	neurexophilin 1	7p22				0.380604651	0.00186	0.020874419				1
NXPH2	neurexophilin 2	2q22.1				0.336333333	0.001003333	0.015433333				1
NYD-SP14	Data not found								-0.919	3.132	5	1
NYD-SP26	Data not found								-0.947	3.298	5	1
NYNRIN	NYN domain and retroviral integrase containing	14q12	-0.887	0.000165	0.0029							1
HDHD3	haloacid dehalogenase-like hydrolase domain containing 3	9q32	0.573	0.01706	0.0835							1
OAS3	2'-5'-oligoadenylate synthetase 3, 100kDa	12q24.2				-0.305	0.00013	0.0073				1
OAZ1	ornithine decarboxylase antizyme 1	19p13.3	-0.433	0.01236	0.0675							1
OBFC1	oligonucleotide/oligosaccharide-binding fold containing 1	10q24.33	-1.135	4.00E-05	0.0011							1
OBFC2A	oligonucleotide/oligosaccharide-binding fold containing 2A	2q32.3				0.36	0	0.0013				1
OBP2B	odorant binding protein 2B	9q34	-0.468	0.02147	0.0973							1
OBSCN	obscurin, cytoskeletal calmodulin and titin-interacting RhoGEF	1q42.13	-0.268	0.03442	0.1317							1
OBSL1	obscurin-like 1	2q35							0.778	2.79083	6	1
OC90	In multiple Geneids					0.398333333	0.019408889	0.081277778				1
OCA2	oculocutaneous albinism II	15q				0.534	0.00824	0.0515				1
OCEL1	occludin/ELL domain containing 1	19p13.11				-0.52	0.00023	0.0093				1
RNASE2	ribonuclease, RNase A family, 2 (liver, eosinophil-derived neurotoxin)	14q24-q31	0.573	0.00187	0.0182				-0.753	3.125333333	5	2
OCLN	In multiple Geneids								-0.72	2.67667	6	1
SNX27	sorting nexin family member 27	1q21.3	0.573	0.00345	0.0282							1
SPATA20	spermatogenesis associated 20	17q21.33	0.573	0.03728	0.1392							1
VWA7	von Willebrand factor A domain containing 7	6p21.33	0.573	0.00595	0.0411							1
ODF3	outer dense fiber of sperm tails 3	11p15.5	-0.375	0.00111	0.0126							1
ODF4	outer dense fiber of sperm tails 4	17p13.1	-0.265	0.0032	0.0268	-0.425	0.00089	0.0167				2
ODZ1	odz, odd Oz/ten-m homolog 1 (Drosophila)	Xq25				0.353543478	0.003150217	0.026369565	-0.798	2.75	5	2
ODZ2	odz, odd Oz/ten-m homolog 2 (Drosophila)	5q34				0.5025	0.000275	0.00985				1
ODZ3	In multiple Geneids					-0.37412963	0.014272963	0.064651852				1
ODZ4	odz, odd Oz/ten-m homolog 4 (Drosophila)	11q14.1				0.3375	0.01102	0.05955				1
OFCC1	orofacial cleft 1 candidate 1	6p24.3							-0.874	2.90167	6	1
OGDH	oxoglutarate (alpha-ketoglutarate) dehydrogenase (lipoamide)	7p14-p13				-0.413	0.000635	0.0143				1
OGFRL1	In multiple Geneids		-2.032	1.00E-05	3.00E-04							1
OGG1	8-oxoguanine DNA glycosylase	3p26.2				-0.311	0.0031	0.0305				1
OGN	osteoglycin	9q22							-0.937	3.21667	9	1
OGT	O-linked N-acetylglucosamine (GlcNAc) transferase	Xq13							-0.752	2.848	5	1
OK/SW-CL.58	OK/SW-CL.58	13q12.3	-0.71	0.00021	0.0037							1
OLAH	oleoyl-ACP hydrolase	10p13				-0.37775	0.0147775	0.06685				1
OLFM1	olfactomedin 1	9q34.3				-0.349	0.00173	0.0229				1
OLFM2	olfactomedin 2	19p13.2	-0.415	0.03093	0.1233	-0.467321429	0.003355357	0.025535714				2
GJC1	gap junction protein, gamma 1, 45kDa	17q21.31	0.572	0.00454	0.0343							1

LINC00654	Data not found		0.572	0.01223	0.0671							1
PPP1R14D	protein phosphatase 1, regulatory (inhibitor) subunit 14D	15q15.1	0.572	0.01367	0.0719							1
OLIG2	oligodendrocyte lineage transcription factor 2	21q22.11	-0.281	0.02208	0.0989	-0.351	0.00068	0.0148	0.868	2.934	5	3
OLIG3	oligodendrocyte transcription factor 3	6q23.3	-0.244	0.02656	0.1116				0.865	2.959	5	2
CXorf38	chromosome X open reading frame 38	Xp11.4	0.571	0.00232	0.0213							1
OMA1	OMA1 zinc metallopeptidase homolog (S. cerevisiae)	1p32.2-p32.1	-0.471	0.02667	0.1119							1
OMP	olfactory marker protein	11q13.5							0.854	3.245	6	1
ONECUT1	one cut homeobox 1	15q21.3							0.69	2.7305	6	1
MFSD10	major facilitator superfamily domain containing 10	4p16.3	0.571	6.00E-05	0.0016							1
ONECUT3	In multiple Geneids					-0.42575	0.0032475	0.0248				1
OPA1	optic atrophy 1 (autosomal dominant)	3q28-q29				0.3195	0.003035	0.02815				1
OPA3	In multiple Geneids					-0.349	0.00265	0.0276				1
OPALIN	oligodendrocytic myelin paranodal and inner loop protein	10q23-q24	-0.28	0.00497	0.0364							1
OPCML	opioid binding protein/cell adhesion molecule-like	11q25				0.382	0.0011	0.0184				1
OPHN1	oligophrenin 1	Xq12				0.342428571	0.005427143	0.033728571				1
OPN1LW	opsin 1 (cone pigments), long-wave-sensitive	Xq28				0.425	0.01295	0.0662				1
OPN1MW	opsin 1 (cone pigments), medium-wave-sensitive	Xq28				0.395	0.02497	0.096233333				1
OPN1MW2	In multiple Geneids					0.395	0.02497	0.096233333				1
OPN4	opsin 4	10q22							1.107	2.74	5	1
OPN5	opsin 5	6p12.3				0.350666667	0.000863333	0.015866667	-1.084	3.93667	6	2
OPRD1	opioid receptor, delta 1	1p36.1-p34.3				-0.481909091	0.00193	0.021672727				1
OPRM1	opioid receptor, mu 1	6q24-q25	-0.199	0.02433	0.1051							1
OPTC	opticin	1q32.1	-0.183	0.04851	0.1641							1
OPTN	In multiple Geneids					-0.328	0.0107	0.05885				1
OR10A2	In multiple Geneids								-0.81	3.08375	8	1
OR10A3	olfactory receptor, family 10, subfamily A, member 3	11p15.4							-0.864	3.40714	7	1
OR10A4	In multiple Geneids								-0.954	3.59125	8	1
OR10A5	In multiple Geneids								-0.952	3.75833	6	1
OR10A6	In multiple Geneids								-0.799	2.97556	9	1
OR10A7	In multiple Geneids								-0.83	3.21875	8	1
OR10AG1	olfactory receptor, family 10, subfamily AG, member 1	11q11							-0.996	3.87714	7	1
OR10C1	In multiple Geneids		-0.434	0.00571	0.04							1
OR10H1	olfactory receptor, family 10, subfamily H, member 1	19p13.1							0.851	3.195	6	1
OR10H2	olfactory receptor, family 10, subfamily H, member 2	19p13.1							0.88	2.976	5	1
OR10H3	olfactory receptor, family 10, subfamily H, member 3	19p13.1							-0.867	3.33	6	1
OR10H4	In multiple Geneids								-1.104	3.35222	9	1
OR10J1	olfactory receptor, family 10, subfamily J, member 1	1q23.2							-0.979	3.49	5	1
OR10J5	In multiple Geneids								-0.952	2.922	5	1
OR10K1	In multiple Geneids								-1.008	2.93111	9	1
OR10K2	In multiple Geneids								-0.893	2.72875	8	1
OR10P1	In multiple Geneids		-0.302	0.02745	0.1137							1
OR10R2	In multiple Geneids								-0.85	3.41	9	1
OR10T2	In multiple Geneids								-1.04	2.99143	7	1
OR10X1	In multiple Geneids								-0.957	3.54125	8	1
OR10Z1	In multiple Geneids								-0.938	3.076	5	1
OR11A1	olfactory receptor, family 11, subfamily A, member 1	6p22.2-p21.31				0.329	0.00205	0.0248				1
OR11G2	In multiple Geneids								-0.875	3.17444	9	1
OR11H1	olfactory receptor, family 11, subfamily H, member 1	22q11.2							-0.893	3.45167	6	1
OR11H12	In multiple Geneids								-0.921	2.88	5	1
OR11H6	In multiple Geneids								-0.754	2.73143	7	1
PARN	poly(A)-specific ribonuclease	16p13	0.571	0.01087	0.0618							1
OR13A1	olfactory receptor, family 13, subfamily A, member 1	10q11.21							-1.236	2.786	5	1
OR13C2	In multiple Geneids								-0.862	3.25833	6	1
OR13C4	In multiple Geneids								-0.961	2.96143	7	1
OR13C5	In multiple Geneids								-1.116	3.068	5	1
OR13C9	In multiple Geneids								-1.01	3.02714	7	1
OR13D1	In multiple Geneids								-0.962	3.32286	7	1
OR13F1	In multiple Geneids								-0.807	2.726	10	1
OR13G1	In multiple Geneids								-0.936	2.93667	9	1
OR14I1	olfactory receptor, family 14, subfamily I, member 1	1q44				0.312	5.00E-05	0.005				1
OR1A1	olfactory receptor, family 1, subfamily A, member 1	17p13.3							-0.826	2.78714	7	1
OR1A2	olfactory receptor, family 1, subfamily A, member 2	17p13.3							-0.968	3.24	7	1
OR1B1	In multiple Geneids								-0.995	4.008	5	1
OR1D2	olfactory receptor, family 1, subfamily D, member 2	17p13.3							-0.9	3.04667	6	1
OR1D5	olfactory receptor, family 1, subfamily D, member 5	17p13.3							-0.881	3.05625	8	1
OR1E1	olfactory receptor, family 1, subfamily E, member 1	17p13.3							-1.022	2.902	5	1
OR1E2	olfactory receptor, family 1, subfamily E, member 2	17p13.3							-0.871	3.586	5	1
OR1F2P	olfactory receptor, family 1, subfamily F, member 2	16p13.3	-0.484	0.01413	0.0735							1
OR1I1	In multiple Geneids					-0.396	0.00045	0.0123				1

OR1J4	olfactory receptor, family 1, subfamily J, member 4	9q34.11							-0.867	3.27625	8	1
OR1L1	olfactory receptor, family 1, subfamily L, member 1	9q33.2							-0.753	2.49	6	1
OR1L4	In multiple Geneids								-1.076	3.326	5	1
OR1N1	In multiple Geneids								-0.608	2.734	5	1
OR1Q1	In multiple Geneids								-0.847	2.78625	8	1
OR1S1	In multiple Geneids								-0.813	3.09333	6	1
OR1S2	In multiple Geneids								-0.927	3.34	5	1
OR2A12	In multiple Geneids								-0.944	3.23167	6	1
OR2A14	In multiple Geneids								-0.767	2.65714	7	1
OR2A2	In multiple Geneids								-1.083	3.17714	7	1
OR2A20P	In multiple Geneids					-0.494	0.00339	0.032				1
OR2A25	In multiple Geneids		-0.207	0.01974	0.0919							1
OR2A4	olfactory receptor, family 2, subfamily A, member 4	6q23				-0.397	0.00982	0.0567				1
OR2A5	In multiple Geneids								-0.988	3.28714	7	1
OR2AG1	In multiple Geneids								-0.825	2.722	5	1
OR2AK2	In multiple Geneids								-1.083	2.87286	7	1
OR2AT4	In multiple Geneids								-0.658	2.72667	6	1
OR2B3	In multiple Geneids								-0.998	3.44571	7	1
OR2C1	olfactory receptor, family 2, subfamily C, member 1	16p13.3				-0.395	0.02583	0.0992				1
OR2C3	olfactory receptor, family 2, subfamily C, member 3	1q44							-0.739	3.07	7	1
OR2D2	In multiple Geneids								-1.032	3.16571	7	1
OR2D3	In multiple Geneids								-0.884	3.19667	9	1
OR2F1	olfactory receptor, family 2, subfamily F, member 1	7q35							-0.796	2.63	5	1
OR2F2	In multiple Geneids								-0.94	3.128	5	1
OR2G3	olfactory receptor, family 2, subfamily G, member 3	1q44							-0.97	3.26375	8	1
OR2G6	In multiple Geneids					0.318	3.00E-05	0.004				1
OR2H2	In multiple Geneids		-0.219	0.03976	0.145							1
OR2L13	In multiple Geneids					0.3095	0.001065	0.01785				1
OR2L2	olfactory receptor, family 2, subfamily L, member 2	1q44							-0.953	2.9425	8	1
OR2L3	In multiple Geneids								-0.927	2.70571	7	1
OR2L8	In multiple Geneids								-0.996	3.07857	7	1
OR2M2	In multiple Geneids								-0.901	3.62625	8	1
OR2M3	In multiple Geneids								-0.946	3.13111	9	1
OR2M4	olfactory receptor, family 2, subfamily M, member 4	1q44							-0.765	3.30857	7	1
OR2M5	In multiple Geneids								-0.83	2.92333	9	1
OR2M7	In multiple Geneids								-0.974	3.24111	9	1
OR2S2	olfactory receptor, family 2, subfamily S, member 2	9p13.3							-0.946	3.41333	6	1
OR2T1	olfactory receptor, family 2, subfamily T, member 1	1q44							-0.696	2.904	5	1
OR2T10	In multiple Geneids								-0.793	3.09333	9	1
OR2T11	In multiple Geneids								0.656	2.86143	7	1
OR2T2	In multiple Geneids								-0.729	2.72833	6	1
OR2T3	In multiple Geneids								-0.989	2.888	5	1
OR2T33	In multiple Geneids								-1.023	2.985	6	1
OR2T35	olfactory receptor, family 2, subfamily T, member 35	1q44	-0.291	0.02218	0.0992							1
OR2T4	In multiple Geneids								-0.623	2.45571	7	1
OR2T5	In multiple Geneids								-0.869	3.67	5	1
OR2T6	In multiple Geneids								-0.85	3.105	8	1
OR2T8	In multiple Geneids								-0.601	2.7725	8	1
OR2V2	In multiple Geneids		-0.366	0.01381	0.0725							1
OR2Z1	In multiple Geneids					-0.484	0.00437	0.0365				1
OR3A1	olfactory receptor, family 3, subfamily A, member 1	17p13.3	-0.337	0.00389	0.0307							1
OR3A2	olfactory receptor, family 3, subfamily A, member 2	17p13.3							-0.966	2.882	5	1
OR4A15	olfactory receptor, family 4, subfamily A, member 15	11q11							-1.128	2.824	5	1
OR4A16	olfactory receptor, family 4, subfamily A, member 16	11q11							-0.762	3.04167	6	1
OR4A47	olfactory receptor, family 4, subfamily A, member 47	11p11.2							-0.829	2.766	5	1
OR4A5	olfactory receptor, family 4, subfamily A, member 5	11p11.12							-0.984	3.285	8	1
OR4B1	In multiple Geneids								-0.976	2.80833	6	1
OR4C11	In multiple Geneids								-1.024	3.35143	7	1
OR4C12	In multiple Geneids								-1.236	3.31	7	1
OR4C13	In multiple Geneids								-0.811	2.735	8	1
OR4C15	olfactory receptor, family 4, subfamily C, member 15	11q11							-0.898	3.33375	8	1
OR4C16	In multiple Geneids								-0.876	2.92	6	1
OR4C3	In multiple Geneids								-0.742	2.918	5	1
OR4C6	In multiple Geneids								-0.82	3.21	9	1
OR4D1	olfactory receptor, family 4, subfamily D, member 1	17q23.2							-0.936	3.17571	7	1
OR4D10	In multiple Geneids								-0.843	2.622	5	1
OR4D9	In multiple Geneids								-0.784	2.99667	6	1
OR4E2	olfactory receptor, family 4, subfamily E, member 2	14q11.2							-0.931	3.39	5	1
OR4F15	In multiple Geneids								-0.9	3.34	6	1

OR4F16	In multiple Geneids					0.586	3.00E-05	0.0041				1
OR4F29	In multiple Geneids					0.586	3.00E-05	0.0041				1
OR4F3	olfactory receptor, family 4, subfamily F, member 3	5q35.3				0.586	3.00E-05	0.0041				1
OR4F5	olfactory receptor, family 4, subfamily F, member 5	1p36.33				0.525	0.00011	0.0068				1
OR4F6	In multiple Geneids								-0.913	2.99857	7	1
OR4K1	olfactory receptor, family 4, subfamily K, member 1	14q11.2							-0.746	2.59667	6	1
OR4K14	In multiple Geneids		-0.286	0.02885	0.1175							1
OR4K15	olfactory receptor, family 4, subfamily K, member 15	14q11.2							-0.991	3.26	8	1
OR4K17	In multiple Geneids								-0.919	3.74556	9	1
OR4K2	In multiple Geneids								-1.154	3.5125	8	1
OR4K5	olfactory receptor, family 4, subfamily K, member 5	14q11.2							-0.997	4.06857	7	1
OR4L1	In multiple Geneids								-0.838	3.063	10	1
OR4M1	In multiple Geneids								-1.019	3.29429	7	1
OR4M2	In multiple Geneids								-1.066	3.64833	6	1
OR4N2	In multiple Geneids								-0.951	2.88	5	1
OR4N4	In multiple Geneids								-1.019	3.46857	7	1
OR4P4	olfactory receptor, family 4, subfamily P, member 4	11q12.1							-0.829	2.96571	7	1
OR4Q3	In multiple Geneids								-0.922	3.57143	7	1
OR4X2	In multiple Geneids								-0.984	2.434	5	1
OR51A2	In multiple Geneids								-0.82	2.82	5	1
OR51A4	In multiple Geneids								-0.898	3.5675	8	1
OR51B2	olfactory receptor, family 51, subfamily B, member 2	11p15							-0.82	3.38	8	1
OR51B4	olfactory receptor, family 51, subfamily B, member 4	11p15							-1.082	3.5175	8	1
OR51B5	olfactory receptor, family 51, subfamily B, member 5	11p15.4							-0.809	2.86	9	1
OR51B6	In multiple Geneids								-0.988	3.50444	9	1
SNORA28	small nucleolar RNA, H/ACA box 28	14q32.32	0.571	0.01747	0.0847							1
OR51E2	olfactory receptor, family 51, subfamily E, member 2	11p15							-0.799	3.01556	9	1
OR51F1	In multiple Geneids								-0.922	2.842	5	1
OR51F2	In multiple Geneids								-0.88	3.39667	6	1
OR51I1	In multiple Geneids								-0.929	3.54222	9	1
OR51I2	In multiple Geneids								-0.761	2.75143	7	1
OR51L1	In multiple Geneids								-0.893	3.01286	7	1
OR51M1	In multiple Geneids								-1.154	3.28625	8	1
OR51S1	In multiple Geneids								-0.936	3.222	5	1
OR51T1	In multiple Geneids								-0.856	3.13222	9	1
OR51V1	In multiple Geneids								-0.942	3.42556	9	1
OR52A1	olfactory receptor, family 52, subfamily A, member 1	11p15.5							-1.25	3.07714	7	1
OR52A4	In multiple Geneids								-0.898	2.93	7	1
OR52A5	In multiple Geneids								-0.78	2.37143	7	1
OR52B4	In multiple Geneids								-0.859	3.655	6	1
OR52D1	In multiple Geneids								-0.926	2.9475	8	1
OR52E2	In multiple Geneids								-1.087	3.08	8	1
OR52E4	In multiple Geneids								-0.997	3.20875	8	1
OR52E5	In multiple Geneids								-0.834	2.766	5	1
OR52E6	In multiple Geneids								-0.897	3.08222	9	1
OR52E8	In multiple Geneids								-0.904	3.21	9	1
OR52H1	In multiple Geneids								-0.73	2.83625	8	1
OR52I2	In multiple Geneids								-1.083	3.205	8	1
OR52J3	In multiple Geneids								-1.013	3.60429	7	1
OR52K1	In multiple Geneids								-0.968	3.219	10	1
OR52K2	In multiple Geneids								-0.745	2.61	8	1
OR52L1	In multiple Geneids								-0.91	2.89375	8	1
OR52M1	In multiple Geneids								-0.745	2.62	9	1
OR52N1	olfactory receptor, family 52, subfamily N, member 1	11p15.4							-1.03	3.678	10	1
OR52N2	In multiple Geneids								-0.798	2.72333	6	1
OR52N4	In multiple Geneids								-1.008	3.32286	7	1
OR52N5	In multiple Geneids								-0.871	3.07714	7	1
OR52W1	In multiple Geneids		-0.183	0.0486	0.1642							1
OR56A1	In multiple Geneids								-1.056	3.36143	7	1
OR56A3	In multiple Geneids								-1.055	3.31857	7	1
OR56B1	In multiple Geneids								-0.9	3.23125	8	1
OR56B4	In multiple Geneids								-1.159	2.58667	6	1
OR5AC2	olfactory receptor, family 5, subfamily AC, member 2	3q12.1							-0.74	2.81286	7	1
OR5AK2	In multiple Geneids								-0.983	3.45125	8	1
OR5AS1	In multiple Geneids								-0.95	3.34222	9	1
OR5AU1	In multiple Geneids								-0.804	2.746	5	1
OR5AY1	olfactory receptor, family 5, subfamily AY, member 1								-0.936	3.34857	7	1
OR5B12	In multiple Geneids								-0.892	3.095	8	1
OR5B17	In multiple Geneids								-0.937	3.32714	7	1

OR5B2	In multiple Geneids								-0.932	2.66667	6	1
OR5B21	In multiple Geneids								-0.985	2.44	6	1
OR5B3	In multiple Geneids								-1.089	3.455	6	1
OR5BF1	olfactory receptor, family 5, subfamily BF, member 1								-1.056	3.503	10	1
OR5D13	In multiple Geneids								-1.066	3.59125	8	1
OR5D14	In multiple Geneids								-1.064	3.15167	6	1
OR5D16	In multiple Geneids								-0.765	2.97625	8	1
OR5D18	In multiple Geneids								-0.72	2.80333	9	1
OR5F1	In multiple Geneids								-0.769	2.58889	9	1
OR5H1	olfactory receptor, family 5, subfamily H, member 1	3q11.2	-0.308	0.00944	0.0562							1
OR5H14	olfactory receptor, family 5, subfamily H, member 14	3q12.1							-0.988	3.41333	6	1
OR5H15	olfactory receptor, family 5, subfamily H, member 15	3q12.1							-1.022	3.02	6	1
OR5H2	olfactory receptor, family 5, subfamily H, member 2	3q12.1							-0.975	3.08	7	1
OR5H6	olfactory receptor, family 5, subfamily H, member 6	3q12.1							-1.046	3.58833	6	1
OR5I1	olfactory receptor, family 5, subfamily I, member 1	11q11							-0.867	3.069	10	1
OR5J2	olfactory receptor, family 5, subfamily J, member 2	11q11							-0.924	2.9725	8	1
OR5K2	In multiple Geneids								-0.976	3.39	7	1
OR5K3	olfactory receptor, family 5, subfamily K, member 3	3q12.1							-0.968	3.3	7	1
OR5K4	olfactory receptor, family 5, subfamily K, member 4	3q12.1							-0.986	3.02375	8	1
OR5L1	In multiple Geneids								-1.042	3.87667	9	1
OR5L2	olfactory receptor, family 5, subfamily L, member 2	11q11							-0.799	2.785	8	1
OR5M1	In multiple Geneids								-0.986	3.031	10	1
OR5M11	In multiple Geneids								-0.865	2.90111	9	1
OR5M3	In multiple Geneids								-0.979	3.315	10	1
OR5M8	In multiple Geneids								-0.664	2.628	5	1
OR5M9	In multiple Geneids								-0.831	3.09222	9	1
OR5P2	In multiple Geneids								-0.927	3.03889	9	1
OR5P3	In multiple Geneids								-0.879	3.05375	8	1
OR5R1	In multiple Geneids								-0.865	3.28667	6	1
OR5T2	In multiple Geneids								-0.882	3.59556	9	1
OR5T3	In multiple Geneids								-1.089	3.94625	8	1
OR5U1	olfactory receptor, family 5, subfamily U member 1								-0.957	3.23	8	1
OR5V1	olfactory receptor, family 5, subfamily V, member 1	6p22.1							-0.893	3.025	10	1
OR5W2	In multiple Geneids								-0.93	3.275	8	1
OR6A2	In multiple Geneids								-0.848	3.27333	6	1
OR6B3	In multiple Geneids		-0.294	0.01471	0.0755							1
OR6C1	In multiple Geneids		-0.218	0.01325	0.0704							1
OR6C2	In multiple Geneids								-0.894	3.37	8	1
OR6C3	In multiple Geneids								-1.053	2.88714	7	1
OR6C4	In multiple Geneids								-0.782	2.526	5	1
OR6C6	In multiple Geneids								-1.012	3.32167	6	1
OR6C65	olfactory receptor, family 6, subfamily C, member 65	12q13.2							-0.774	2.66	5	1
OR6C68	olfactory receptor, family 6, subfamily C, member 68	12q13.2							-1.032	2.98	5	1
OR6C70	In multiple Geneids								-0.853	2.76571	7	1
OR6C74	In multiple Geneids								-0.919	3.13143	7	1
OR6C76	In multiple Geneids								-1.005	3.29111	9	1
OR6F1	In multiple Geneids								-0.855	2.82375	8	1
OR6K2	olfactory receptor, family 6, subfamily K, member 2	1q23.1							-0.984	3.27667	6	1
OR6K3	In multiple Geneids								-1.047	3.3375	8	1
OR6M1	In multiple Geneids								-1.005	3.12143	7	1
OR6N1	In multiple Geneids								-0.849	2.87143	7	1
OR6N2	olfactory receptor, family 6, subfamily N, member 2	1q23.1							-0.765	2.708	5	1
OR6Q1	In multiple Geneids								-0.835	3.234	5	1
OR6Y1	In multiple Geneids								-0.84	3.28714	7	1
OR7A17	olfactory receptor, family 7, subfamily A, member 17	19p13.12							-0.884	2.97286	7	1
OR7A5	olfactory receptor, family 7, subfamily A, member 5	19p13.1	-0.197	0.04878	0.1646							1
OR7E14P	olfactory receptor, family 7, subfamily E, member 14 pseudogene	11p15.1	-0.562	0.04168	0.1494							1
OR7E18P	In multiple Geneids		-0.305	0.02372	0.1033							1
OR7E35P	In multiple Geneids		-0.265	0.02053	0.0944							1
OR7E91P	olfactory receptor, family 7, subfamily E, member 91 pseudogene	2p13.3	-1.354	1.00E-05	4.00E-04							1
OR7G1	In multiple Geneids		-0.422	0.00084	0.0104	-0.353	0.00422	0.0358				2
OR7G2	In multiple Geneids					-0.493	0.00578	0.0423				1
OR7G3	In multiple Geneids					-0.308	0.01439	0.0704				1
OR8A1	In multiple Geneids								-0.893	3.065	6	1
OR8B3	In multiple Geneids		-0.367	0.01187	0.0657							1
OR8D2	In multiple Geneids								-0.891	3.19143	7	1
OR8D4	In multiple Geneids								-0.844	3.24714	7	1
OR8G1	olfactory receptor, family 8, subfamily G, member 1	11q24.2							-0.724	2.82	8	1
OR8G2	olfactory receptor, family 8, subfamily G, member 2	11q24.2							-0.869	3.436	5	1

OR8G5	In multiple Geneids								-1.024	3.788	10	1
OR8H1	In multiple Geneids								-0.925	3.11	8	1
OR8H2	In multiple Geneids								-0.85	3.0975	8	1
OR8H3	In multiple Geneids								-0.867	2.63375	8	1
OR8I2	In multiple Geneids								-0.981	3.33	8	1
OR8J1	In multiple Geneids								-1.093	3.20429	7	1
OR8K1	In multiple Geneids								-0.925	3.26778	9	1
OR8K3	In multiple Geneids								-0.826	3.06125	8	1
OR8K5	In multiple Geneids								-0.768	3.05333	6	1
OR8U1	In multiple Geneids								-0.968	3.38125	8	1
OR8U8	olfactory receptor, family 8, subfamily U, member 8	11q11				0.314	0.00043	0.012	-0.968	3.38125	8	2
OR9A2	In multiple Geneids								-0.812	2.91333	6	1
OR9G1	In multiple Geneids								-0.957	3.41222	9	1
OR9G4	In multiple Geneids		-0.329	0.00633	0.0428							1
OR9G9	olfactory receptor, family 9, subfamily G, member 9	11q11							-0.957	3.41222	9	1
OR9K2	In multiple Geneids								-0.718	2.92222	9	1
ORAI1	ORAI calcium release-activated calcium modulator 1	12q24.31				-0.338	0.0057	0.0378				1
ZFP64	zinc finger protein 64 homolog (mouse)	20q13.2	0.571	0.00811	0.0506	0.405	0.03887	0.12725				2
ORAI3	ORAI calcium release-activated calcium modulator 3	16p11.2				-0.3635	0.00386	0.0256				1
ORAOV1	In multiple Geneids					-0.303	0.00014	0.0075				1
ZNF146	zinc finger protein 146	19q13.1	0.571	0.00376	0.03							1
ORC3L	Data not found					-0.342333333	0.000836667	0.0149				1
HIST1H2BD	histone cluster 1, H2bd	6p21.3	0.57	0.01411	0.0735				-0.741	2.87929	7	2
NIF3L1	NIF3 NGG1 interacting factor 3-like 1 (S. cerevisiae)	2q33	0.57	0.00881	0.0535							1
ORM1	orosomucoid 1	9q31-q32							0.586	2.478	5	1
PCID2	PCI domain containing 2	13q34	0.5695	0.026435	0.10305							1
ORMDL2	In multiple Geneids		-0.509	0.03247	0.1268							1
ACLY	ATP citrate lyase	17q21.2	0.569	0.00436	0.0332							1
ERF	Ets2 repressor factor	19q13	0.569	0.00066	0.0088							1
OSBP2	oxysterol binding protein 2	22q12.2				-0.438619048	0.001230238	0.0137				1
OSBPL10	oxysterol binding protein-like 10	3p22.3				-0.378285714	0.003077857	0.020935714				1
OSBPL11	oxysterol binding protein-like 11	3q21				-0.383	0.00317	0.0309				1
OSBPL1A	oxysterol binding protein-like 1A	18q11.1				-0.3	0.024	0.095				1
OSBPL3	oxysterol binding protein-like 3	7p15				0.316	0.00314	0.0286				1
OSBPL5	oxysterol binding protein-like 5	11p15.4				-0.412	0.000483333	0.012566667				1
OSBPL6	oxysterol binding protein-like 6	2q32.1				0.315	0.00154	0.0216	-0.886	3.035	6	2
FANCA	Fanconi anemia, complementation group A	16q24.3	0.569	0.02256	0.1001							1
OSBPL8	oxysterol binding protein-like 8	12q14							-0.476	2.635	6	1
OSCP1	organic solute carrier partner 1	1p34.3	-0.211	0.02908	0.1181	-0.4005	0.000795	0.0134				2
HSD3B7	hydroxy-delta-5-steroid dehydrogenase, 3 beta- and steroid delta-isomerase 7	16p11.2	0.569	0.00213	0.02							1
OSGEPL1	O-sialoglycoprotein endopeptidase-like 1	2q32.2							-0.924333333	3.305236667	6	1
OSGIN1	oxidative stress induced growth inhibitor 1	16q23.3				-0.315	0.00762	0.0493				1
POM121	POM121 transmembrane nucleoporin	7q11.23	0.569	0.01851	0.0882							1
TNRC18	trinucleotide repeat containing 18	7p22.1	0.568333333	0.00071	0.008233333							1
OSMR	oncostatin M receptor	5p13.1				0.3565	0.005966	0.03925				1
TNFAIP8L1	tumor necrosis factor, alpha-induced protein 8-like 1	19p13.3	0.568	0.00146	0.0153							1
OSTC	oligosaccharyltransferase complex subunit	4q25				-0.338	0.00494	0.0375				1
OSTCL	Data not found					-0.306	0.01883	0.0823				1
OSTF1	osteoclast stimulating factor 1	9q13-q21.2	-1.016	0.00017	0.0032							1
AMMECR1L	AMME chromosomal region gene 1-like	2q21	0.567	6.00E-04	0.0082							1
OSTN	osteocrin	3q28				0.305	0.00596	0.0431	-0.64	2.76833	6	2
OTOL1	otolin 1	3q26.1				0.326	0.00169	0.0226				1
OTOP1	otopetrin 1	4p16.3				-0.357	0.00122	0.0194	0.781	2.586	5	2
OTOP2	otopetrin 2	17q25.1	-0.48	0.00024	0.0042	-0.309	0.02118	0.0882				2
OTOP3	otopetrin 3	17q25.1	-1.854	0	0							1
OTOR	otoraplin	20p12.1-p11.23							-0.885	3.0625	8	1
OTP	orthopedia homeobox	5q13.3				-0.355	0.00035	0.0111				1
OTUB1	OTU domain, ubiquitin aldehyde binding 1	11q13.1				-0.325	0.00148	0.0212				1
OTUB2	OTU domain, ubiquitin aldehyde binding 2	14q32.12	-1.149	2.00E-05	6.00E-04							1
RAB43	RAB43, member RAS oncogene family	3q21.3	0.567	0.00369	0.0296							1
TRAF4	TNF receptor-associated factor 4	17q11-q12	0.567	0.00148	0.0154							1
EMILIN2	elastin microfibril interfacer 2	18p11.3	0.566	0.00043	0.0064							1
OTX2	orthodenticle homeobox 2	14q22.3							0.764	2.43187	8	1
OVCH1	ovochymase 1	12p11.22				1.075	3.00E-05	0.0044				1
OVCH2	ovochymase 2 (gene/pseudogene)	11p15.4							-0.93	3.24667	9	1
OVOL1	ovo-like 1(Drosophila)	11q13	-1.577	0.00025	0.0044	-0.339	0.00221	0.0258				2
IRAK2	interleukin-1 receptor-associated kinase 2	3p25.3	0.566	0.02638	0.1111							1
OXCT1	3-oxoacid CoA transferase 1	5p13.1				0.446909091	0.006884091	0.041006818				1
OXGR1	oxoglutarate (alpha-ketoglutarate) receptor 1	13q32.1				0.307	0.00082	0.0161				1

OXR1	oxidation resistance 1	8q23				0.357185185	0.000236667	0.006796296				1
OXSM	3-oxoacyl-ACP synthase, mitochondrial	3p24.2	-0.376	0.02938	0.1189							1
OXSR1	oxidative-stress responsive 1	3p22.2	-0.823	0.00194	0.0187							1
OXT	oxytocin, prepropeptide	20p13							0.845	2.88667	6	1
KDELR1	KDEL (Lys-Asp-Glu-Leu) endoplasmic reticulum protein retention receptor 1	19q13.3	0.566	4.00E-04	0.0061							1
P2RX1	purinergic receptor P2X, ligand-gated ion channel, 1	17p13.3				-0.532	1.00E-04	0.0066				1
TMEM180	transmembrane protein 180	10q24.32	0.566	0.00386	0.0305							1
P2RX5	purinergic receptor P2X, ligand-gated ion channel, 5	17p13.3				-0.435333333	0.00504	0.038733333				1
P2RX7	purinergic receptor P2X, ligand-gated ion channel, 7	12q24				-0.413	0.000975	0.0172				1
P2RY1	purinergic receptor P2Y, G-protein coupled, 1	3q25.2	-1.596	0	0							1
P2RY10	purinergic receptor P2Y, G-protein coupled, 10	Xq21.1							-0.657	2.87125	8	1
P2RY12	In multiple Geneids					0.3495	0.00635	0.03895	-0.868	2.867	5.5	2
P2RY13	purinergic receptor P2Y, G-protein coupled, 13	3q24							-0.951	3.06143	7	1
P2RY2	purinergic receptor P2Y, G-protein coupled, 2	11q13.5-q14.1	-2.222	0	2.00E-04							1
P2RY8	In multiple Geneids					-0.47315625	0.003170938	0.02481875				1
DDX27	DEAD (Asp-Glu-Ala-Asp) box polypeptide 27	20q13.13	0.565	0.00096	0.0113							1
MOGS	mannosyl-oligosaccharide glucosidase	2p13.1	0.565	0.00773	0.0491							1
SLC17A5	solute carrier family 17 (anion/sugar transporter), member 5	6q13	0.565	0.01257	0.0682							1
P4HB	prolyl 4-hydroxylase, beta polypeptide	17q25				-0.338	0.01629	0.0752				1
TRIM44	tripartite motif containing 44	11p13	0.565	0.01399	0.0731							1
P53AIP1	Data not found								0.658	2.72714	7	1
PABPC1	poly(A) binding protein, cytoplasmic 1	8q22.2-q23	-0.413	0.00244	0.022							1
ZNF131	zinc finger protein 131	5p12	0.565	0.01317	0.0701							1
PABPC1L2A	poly(A) binding protein, cytoplasmic 1-like 2A	Xq13.2	-0.219	0.0161	0.0803							1
PABPC1P2	poly(A) binding protein, cytoplasmic 1 pseudogene 2	2q22.3	-0.28	0.02145	0.0972							1
PABPC3	poly(A) binding protein, cytoplasmic 3	13q12-q13	-0.277	0.03242	0.1268							1
PABPC5	poly(A) binding protein, cytoplasmic 5	Xq21.3							-0.742	2.59857	7	1
PACRG	PARK2 co-regulated	6q26							-0.783	2.699	5	1
PACS1	phosphofurin acidic cluster sorting protein 1	11q13.1-q13.2				-0.308	0.00013	0.0073				1
PACS2	phosphofurin acidic cluster sorting protein 2	14q32.33				-0.362	0.00597	0.034525				1
PACSIN1	protein kinase C and casein kinase substrate in neurons 1	6p21.3	-0.208	0.01653	0.0816							1
PACSIN2	protein kinase C and casein kinase substrate in neurons 2	22q13.2-q13.33				-0.461375	0.001032083	0.013195833				1
PACSIN3	protein kinase C and casein kinase substrate in neurons 3	11p12-p11.12	-0.748	0.00048	0.0069							1
PADI1	peptidyl arginine deiminase, type I	1p36.13	-3.725	0	0	-0.404285714	0.000452857	0.011185714				2
HSD17B7P2	In multiple Geneids		0.564	0.01327	0.0705							1
PADI3	peptidyl arginine deiminase, type III	1p36.13	-1.672	2.00E-05	6.00E-04	-0.419333333	0.000746667	0.012533333				2
PADI4	In multiple Geneids					-0.414666667	0.001496667	0.017533333				1
PADI6	In multiple Geneids		-0.281	0.00618	0.0422	-0.4045	0.008403333	0.042566667				2
PAFAH1B1	platelet-activating factor acetylhydrolase 1b, regulatory subunit 1 (45kDa)	17p13.3	-0.477	0.00444	0.0336	-0.472	0.0043125	0.028875				2
PAFAH1B3	platelet-activating factor acetylhydrolase 1b, catalytic subunit 3 (29kDa)	19q13.1				-0.302	0.00313	0.0306				1
PAFAH2	platelet-activating factor acetylhydrolase 2, 40kDa	1p36				-0.379166667	0.005499167	0.038441667				1
PCDH17	protocadherin 17	13q21.1	0.564	3.00E-05	9.00E-04	0.392	0.001165	0.01505				2
PAGE3	P antigen family, member 3 (prostate associated)	Xp11.21	-0.169	0.02738	0.1136							1
SNORA29	small nucleolar RNA, H/ACA box 29	6q25.3	0.564	0.04545	0.158							1
PAH	phenylalanine hydroxylase	12q22-q24.2							-0.651	2.708	5	1
TMCO3	transmembrane and coiled-coil domains 3	13q34	0.564	0.00097	0.0115							1
PAIP2	poly(A) binding protein interacting protein 2	5q31.2				-0.346	0.004535	0.035825				1
PAIP2B	poly(A) binding protein interacting protein 2B	2p13.3	-1.824	1.00E-05	5.00E-04							1
ZDHHC23	zinc finger, DHHC-type containing 23	3q13.31	0.564	0.00894	0.0541							1
PAK2	p21 protein (Cdc42/Rac)-activated kinase 2	3q29				-0.311	0.0074	0.0485				1
PAK3	p21 protein (Cdc42/Rac)-activated kinase 3	Xq23				0.3422	0.003362	0.02638				1
PAK4	p21 protein (Cdc42/Rac)-activated kinase 4	19q13.2				-0.3696	0.005548	0.03732				1
PAK6	p21 protein (Cdc42/Rac)-activated kinase 6	15q14	-1.226	1.00E-05	3.00E-04				0.79	2.575	6	2
PAK7	p21 protein (Cdc42/Rac)-activated kinase 7	20p12				0.339555556	0.012664444	0.0576				1
PALLD	palladin, cytoskeletal associated protein	4q32.3							-0.748	3.14444	9	1
PALM	paralemmin	19p13.3				-0.364	0.008893333	0.050733333				1
ZNF280B	zinc finger protein 280B	22q11.22	0.564	0.02677	0.1121							1
PALM3	paralemmin 3	19p13.12	-0.211	0.02846	0.1165							1
PALMD	In multiple Geneids		-1.809	3.00E-05	9.00E-04							1
MSH2	mutS homolog 2, colon cancer, nonpolyposis type 1 (E. coli)	2p21	0.563	0.01988	0.0923							1
POLR3E	polymerase (RNA) III (DNA directed) polypeptide E (80kD)	16p12.2	0.563	2.00E-04	0.0037							1
PAN3	PAN3 poly(A) specific ribonuclease subunit homolog (S. cerevisiae)	13q12.2							-0.984	2.806	5	1
PANK1	pantothenate kinase 1	10q23.31							-0.861	3.228	5	1
PANK2	In multiple Geneids		-0.526	0.0101	0.0589							1
PANK4	pantothenate kinase 4	1p36.32				-0.361	0.00299	0.03				1
ZNF678	zinc finger protein 678	1q42.13	0.563	0.010875	0.0605	0.374	0.00023	0.0093				2
PANX2	pannexin 2	22q13.33	-0.363	0.01578	0.0792	-0.735	2.00E-05	0.0036				2
PANX3	pannexin 3	11q24.2	-0.214	0.03968	0.1448							1
PAOX	polyamine oxidase (exo-N4-amino)	10q26.3	-0.261	0.01106	0.0626							1

PAPD4	PAP associated domain containing 4	5q14.1	-0.395	0.0026	0.0231	-0.345	9.33E-05	0.005666667				2
PAPL	iron/zinc purple acid phosphatase-like protein	19q13.2	-0.365	0.02761	0.1141	-0.412	0.001885	0.0228				2
PAPLN	papilin, proteoglycan-like sulfated glycoprotein	14q24.2				-0.352666667	0.002853333	0.027833333				1
PAPPA2	pappalysin 2	1q23-q25				0.338	0.002983077	0.025776923				1
PAPSS1	3'-phosphoadenosine 5'-phosphosulfate synthase 1	4q24	-0.454	0.01371	0.0721							1
PAQR5	progestin and adipoQ receptor family member V	15q23	-1.228	0.00031	0.005							1
PAQR7	progestin and adipoQ receptor family member VII	1p36.11	-0.975	0	2.00E-04	-0.39675	0.00397	0.0307				2
PAQR8	progestin and adipoQ receptor family member VIII	6p12.1							0.594	2.626	5	1
PAQR9	progestin and adipoQ receptor family member IX	3q23							0.754	2.814	5	1
PARD3	par-3 partitioning defective 3 homolog (C. elegans)	10p11.21				0.379	0.03243	0.1139				1
TMEM223	transmembrane protein 223	11q12.3	0.562	0.00196	0.0188							1
CCDC34	coiled-coil domain containing 34	11p14.1	0.561	0.00083	0.0103							1
PARD6G	par-6 partitioning defective 6 homolog gamma (C. elegans)	18q23	-1.945	0	2.00E-04				0.935	2.596	5	2
PARK2	parkinson protein 2, E3 ubiquitin protein ligase (parkin)	6q25.2-q27							-0.783	2.699	5	1
FKBP14	FK506 binding protein 14, 22 kDa	7p14.3	0.561	0.00242	0.0219				-0.87	2.68667	6	2
LBR	lamin B receptor	1q42.1	0.561	0.02621	0.1106							1
KIAA1919	KIAA1919	6q22	0.56	0.00462	0.0346							1
MGST3	microsomal glutathione S-transferase 3	1q23	0.56	0.02885	0.1175							1
UNC93B1	unc-93 homolog B1 (C. elegans)	11q13	0.56	0.005015	0.0305							1
ZNF816	zinc finger protein 816	19q13.41	0.56	0.01441	0.0745							1
PARP15	poly (ADP-ribose) polymerase family, member 15	3q21.1							-1.061	4.042	5	1
CCP110	centriolar coiled coil protein 110kDa	16p12.3	0.559	0.03348	0.1295							1
PARS2	prolyl-tRNA synthetase 2, mitochondrial (putative)	1p32.2	-0.213	0.03813	0.1412							1
PART1	In multiple Geneids		-1.202	0	2.00E-04							1
IFT81	intraflagellar transport 81 homolog (Chlamydomonas)	12q24.13	0.559	0.00734	0.0474							1
PARVG	parvin, gamma	22q13.31				-0.383	0.001952	0.01744				1
PASD1	PAS domain containing 1	Xq28				0.308	0.009185	0.05235				1
PATE	Data not found								-1.02	3.71667	6	1
LRPPRC	leucine-rich pentatricopeptide repeat containing	2p21	0.559	0.01302	0.0698	0.3425	0.002135	0.02165				2
PATL2	protein associated with topoisomerase II homolog 2 (yeast)	15q21.1				-0.329	1.00E-05	0.003				1
SAMSN1	SAM domain, SH3 domain and nuclear localization signals 1	21q11	0.559	0.03786	0.1405	0.409	2.00E-05	0.004				2
PAX1	paired box 1	20p11.2	-0.515	0.00298	0.0255							1
PAX2	paired box 2	10q24				-0.31	0.000275	0.00885				1
PAX3	paired box 3	2q35				0.352333333	0.006516667	0.031				1
PAX5	paired box 5	9p13				-0.36784	0.0014296	0.013816				1
PAX7	paired box 7	1p36.13				-0.35075	0.0023975	0.025775				1
PAX9	paired box 9	14q13.3	-3.739	0	3.00E-04							1
PBLD	phenazine biosynthesis-like protein domain containing	10q21.3				-0.319	0.00225	0.0254				1
PBOV1	prostate and breast cancer overexpressed 1	6q23.3							-1.027	3.014	5	1
PBRM1	polybromo 1	3p21	-0.49	0.00979	0.0576	-0.344714286	0.00415	0.029157143				2
PBX1	pre-B-cell leukemia homeobox 1	1q23				0.349857143	0.001132857	0.016657143				1
PBX4	pre-B-cell leukemia homeobox 4	19p12				-0.38375	0.00198625	0.0215125				1
ZDHHC4	zinc finger, DHHC-type containing 4	7p22.1	0.559	0.00117	0.0131							1
PC	pyruvate carboxylase	11q13.4-q13.5	-0.539	0.01409	0.0734	-0.366666667	0.005328333	0.0385				2
ANO9	anoctamin 9	11p15.5	0.558	0.03063	0.1224							1
PCBD2	pterin-4 alpha-carbinolamine dehydratase/dimerization cofactor of hepatocyt	5q31.1				-0.36825	0.0016175	0.02085				1
PCBP1	poly(rC) binding protein 1	2p13-p12	-0.328	0.01726	0.084							1
PCBP3	poly(rC) binding protein 3	21q22.3				-0.370142857	0.000461429	0.011471429				1
PCBP4	poly(rC) binding protein 4	3p21				-0.424	1.00E-05	0.0024				1
PCCA	propionyl CoA carboxylase, alpha polypeptide	13q32	-0.395	0.02551	0.1086							1
PCCB	propionyl CoA carboxylase, beta polypeptide	3q21-q22				-0.311	0.0174	0.0785				1
PCDH1	protocadherin 1	5q31.3				-0.307	0.00183	0.0235				1
PCDH11X	protocadherin 11 X-linked	Xq21.3							-0.891	3.50143	7	1
PCDH11Y	protocadherin 11 Y-linked	Yp11.2							-0.97	3.19714	7	1
GLG1	golgi glycoprotein 1	16q22.3	0.558	0.00069	0.0091							1
PCDH15	In multiple Geneids								-0.865	3.2525	8	1
GUSBP11	glucuronidase, beta pseudogene 11	22q11.23	0.5575	0.012365	0.05625							1
C11orf96	chromosome 11 open reading frame 96	11p11.2	0.557	0.00516	0.0373							1
PCDH19	In multiple Geneids		-0.407	0.00066	0.0087				0.62	2.66167	6	2
PCDH21	protocadherin 21					-0.408	0.00467	0.0378				1
PCDH24	Data not found					-0.382875	0.00161875	0.0168125				1
PCDH9	protocadherin 9	13q21.32				0.37135	0.001543	0.01828				1
PCDHA1	protocadherin alpha 1	5q31				-0.359	0.04744	0.144				1
PCDHA11	protocadherin alpha 11	5q31							-1.138	3.90222	9	1
PCDHA12	protocadherin alpha 12	5q31							-0.845	3.231	5	1
PCDHA2	protocadherin alpha 2	5q31				-0.359	0.04744	0.144				1
PCDHA3	protocadherin alpha 3	5q31							-0.742	2.54167	6	1
PCDHA4	protocadherin alpha 4	5q31							-0.81	3.226	5	1
PCDHA5	protocadherin alpha 5	5q31							-0.779	2.86429	7	1

PCDHA6	protocadherin alpha 6	5q31				-0.359	0.04744	0.144				1
PCDHA7	protocadherin alpha 7	5q31							-0.83	2.80571	7	1
PCDHA8	protocadherin alpha 8	5q31							-0.802	3.04778	9	1
PCDHA9	protocadherin alpha 9	5q31							-0.725	2.997775	9	1
PCDHB1	protocadherin beta 1	5q31	-0.331	0.00158	0.0162							1
GNL2	guanine nucleotide binding protein-like 2 (nucleolar)	1p34.3	0.557	0.00042	0.0063							1
ZNF3	zinc finger protein 3	7q22.1	0.557	0.00299	0.0255							1
TAS2R50	taste receptor, type 2, member 50	12p13.2	0.556	0.01216	0.0669				-0.79	3.40333	6	2
DIABLO	diablo, IAP-binding mitochondrial protein	12q24.31	0.555	0.00043	0.0065							1
DSCC1	defective in sister chromatid cohesion 1 homolog (S. cerevisiae)	8q24.12	0.555	0.01488	0.0762							1
HLA-L	major histocompatibility complex, class I, L (pseudogene)	6p21.3	0.555	0.00109	0.0124							1
LSM14A	LSM14A, SCD6 homolog A (S. cerevisiae)	19q13.11	0.555	0.00052	0.0074							1
N4BP2L2	NEDD4 binding protein 2-like 2	13q13.1	0.555	0.00195	0.0188							1
PCDHB6	protocadherin beta 6	5q31	0.555	0.04751	0.162							1
PCDHGA1	protocadherin gamma subfamily A, 1	5q31				-0.34225	0.000555	0.011575				1
PCDHGA10	protocadherin gamma subfamily A, 10	5q31				-0.357	0.00099	0.017				1
PCDHGA11	protocadherin gamma subfamily A, 11	5q31							-0.725	2.49667	6	1
PCDHGA12	protocadherin gamma subfamily A, 12	5q31							-0.798	2.622	5	1
PCDHGA2	protocadherin gamma subfamily A, 2	5q31							-0.835	3.154	5	1
PCDHGA3	protocadherin gamma subfamily A, 3	5q31				-0.34225	0.000555	0.011575				1
PCDHGA4	protocadherin gamma subfamily A, 4	5q31				-0.34225	0.000555	0.011575				1
PCDHGA5	protocadherin gamma subfamily A, 5	5q31							-0.789	2.73	5	1
PCDHGA6	protocadherin gamma subfamily A, 6	5q31				-0.339333333	0.000663333	0.012366667				1
PCDHGA7	protocadherin gamma subfamily A, 7	5q31				-0.339333333	0.000663333	0.012366667				1
PCDHGA8	protocadherin gamma subfamily A, 8	5q31							-0.927	2.92571	7	1
PCDHGA9	protocadherin gamma subfamily A, 9	5q31							-1.034	2.97	8	1
PCDHGB1	protocadherin gamma subfamily B, 1	5q31				-0.34225	0.000555	0.011575				1
PCDHGB2	protocadherin gamma subfamily B, 2	5q31				-0.34225	0.000555	0.011575				1
PCDHGB3	protocadherin gamma subfamily B, 3	5q31				-0.34225	0.000555	0.011575				1
PCDHGB4	protocadherin gamma subfamily B, 4	5q31				-0.339333333	0.000663333	0.012366667				1
PCDHGB5	protocadherin gamma subfamily B, 5	5q31				-0.357	0.00099	0.017				1
PCDHGB6	protocadherin gamma subfamily B, 6	5q31				-0.357	0.00099	0.017				1
PCDHGB7	protocadherin gamma subfamily B, 7	5q31							-0.869	2.99167	6	1
PCDHGC3	protocadherin gamma subfamily C, 3	5q31				-0.392	0.0015	0.0213	0.879	2.93833	6	2
PCDHGC4	protocadherin gamma subfamily C, 4	5q31				-0.392	0.0015	0.0213	0.879	2.93833	6	2
PCDHGC5	protocadherin gamma subfamily C, 5	5q31				-0.392	0.0015	0.0213	0.826	2.87083	6	2
PCF11	PCF11, cleavage and polyadenylation factor subunit, homolog (S. cerevisiae)	11q13	-0.426	0.00106	0.0122							1
RNU6ATAC	RNA, U6atac small nuclear (U12-dependent splicing)	9q34.2	0.555	0.02469	0.1062							1
SAV1	salvador homolog 1 (Drosophila)	14q13-q23	0.555	0.04592	0.1588				-1.072	2.88	5	2
PCGF3	polycomb group ring finger 3	4p16.3	-0.292	0.03387	0.1303	-0.355111111	0.005438889	0.039711111				2
PCGF6	polycomb group ring finger 6	10q24.33				-0.325	0.01833	0.081				1
HMGA2	high mobility group AT-hook 2	12q15	0.554	0.02149	0.0973							1
SEC11A	SEC11 homolog A (S. cerevisiae)	15q25.3	0.554	0.00042	0.0063							1
PCLO	piccolo (presynaptic cytomatrix protein)	7q11.23-q21.3				0.3739375	0.020389688	0.08335				1
PCMTD1	protein-L-isoaspartate (D-aspartate) O-methyltransferase domain containing	8q11.23							-0.903	2.86	5	1
SQSTM1	sequestosome 1	5q35	0.554	0.00077	0.0097							1
PCNA	proliferating cell nuclear antigen	20pter-p12							-0.649	2.772	5	1
PCNT	pericentrin	21q22.3				-0.3472	0.005885	0.03379				1
CHD1	chromodomain helicase DNA binding protein 1	5q15-q21	0.553	0.00782	0.0494							1
PCNXL3	In multiple Geneids					-0.434	0.00091	0.0169				1
SDC4	syndecan 4	20q12	0.553	0.02727	0.1133							1
PCP4	Purkinje cell protein 4	21q22.2				-0.3514	0.000976	0.01686				1
PCP4L1	Purkinje cell protein 4 like 1	1q23.3	-1.056	6.00E-05	0.0015							1
PCSK2	proprotein convertase subtilisin/kexin type 2	20p11.2				0.352	0.01281	0.06342				1
PCSK4	In multiple Geneids		-0.24	0.03782	0.1404	-0.5705	0.013	0.0587				2
PCSK5	proprotein convertase subtilisin/kexin type 5	9q21.3				0.319	0.00964	0.0561	-0.707	2.53	5	2
ZNF713	zinc finger protein 713	7p11.2	0.553	9.00E-04	0.0109							1
CHML	choroideremia-like (Rab escort protein 2)	1q42-qter	0.552	0.02189	0.0986				-0.888	3.23125	8	2
NEDD4L	In multiple Geneids		0.552	0.04609	0.1592							1
PCYOX1	prenylcysteine oxidase 1	2p13.3	-0.54	0.02153	0.0974							1
PCYOX1L	prenylcysteine oxidase 1 like	5q32	-0.293	0.00535	0.0383	-0.315	0.01069	0.0594				2
SNORD45C	In multiple Geneids		0.552	0.01141	0.064							1
PDC	phosducin	1q25.2							-0.896	2.94667	6	1
PDCD10	programmed cell death 10	3q26.1				0.363	0.004725	0.032225				1
PDCD11	programmed cell death 11	10q24.33				-0.33725	0.00309875	0.023225				1
PDCD1LG2	programmed cell death 1 ligand 2	9p24.2							-1.005	2.39	6	1
ZDHHC1	zinc finger, DHHC-type containing 1	16q22.1	0.552	0.02963	0.1196							1
PDCD4	programmed cell death 4 (neoplastic transformation inhibitor)	10q24	-1.206	1.00E-05	4.00E-04							1
PDCD5	programmed cell death 5	19q12-q13.1				-0.304	0.00199	0.0245				1

PDCD6IP	programmed cell death 6 interacting protein	3p22.3							-0.836	2.886	5	1
PDCD7	programmed cell death 7	15q22.31	-0.308	0.00799	0.0501							1
PDCD8	Data not found								-0.62	2.404	5	1
PDE10A	phosphodiesterase 10A	6q26				-0.321	0.00713	0.0476				1
BCL2L14	BCL2-like 14 (apoptosis facilitator)	12p13-p12	0.551	0.01159	0.0646							1
PDE12	phosphodiesterase 12	3p14.3				-0.446	2.00E-05	0.0035				1
PDE1A	phosphodiesterase 1A, calmodulin-dependent	2q32.1				0.329333333	6.17E-05	0.004883333	-0.87	3.55	8	2
PDE1C	phosphodiesterase 1C, calmodulin-dependent 70kDa	7p14.3				0.359133333	0.002080667	0.01938				1
PDE2A	phosphodiesterase 2A, cGMP-stimulated	11q13.4	-0.378	0.04325	0.153	-0.3595	0.002065	0.0233				2
MYO1B	myosin IB	2q12-q34	0.551	0.02153	0.0974	0.359666667	6.33E-05	0.005166667				2
PDGFB	platelet-derived growth factor beta polypeptide	22q13.1	0.551	0.00078	0.0098							1
SAA1	serum amyloid A1	11p15.1	0.551	0.02428	0.1049							1
TCIRG1	T-cell, immune regulator 1, ATPase, H+ transporting, lysosomal V0 subunit A3	11q13.2	0.551	0.00739	0.0477							1
PDE4C	phosphodiesterase 4C, cAMP-specific	19p13.11				-0.386125	0.0041025	0.0327375				1
PDE4D	phosphodiesterase 4D, cAMP-specific	5q12							-0.916	3.378	5	1
ANP32A	acidic (leucine-rich) nuclear phosphoprotein 32 family, member A	15q23	0.55	0.004275	0.0298							1
PDE6A	phosphodiesterase 6A, cGMP-specific, rod, alpha	5q31.2-q34	-1.105	8.00E-05	0.0018	-0.354666667	0.002051667	0.0159				2
PDE6B	phosphodiesterase 6B, cGMP-specific, rod, beta	4p16.3				-0.475	0.00286	0.0292				1
PDE6H	phosphodiesterase 6H, cGMP-specific, cone, gamma	12p13	-0.246	0.00894	0.0541							1
EDNRA	endothelin receptor type A	4q31.22	0.55	0.04916	0.1653							1
PDE7B	phosphodiesterase 7B	6q23-q24							-0.841	3.258	5	1
LRP1	low density lipoprotein receptor-related protein 1	12q13-q14	0.55	0.02141	0.0971							1
PDE8B	phosphodiesterase 8B	5q13.3				-0.3285	0.00074	0.01535				1
NFKBIE	nuclear factor of kappa light polypeptide gene enhancer in B-cells inhibitor, ep	6p21.1	0.55	0.00011	0.0023							1
PDF	peptide deformylase (mitochondrial)	16q22.1				-0.505	0.00042	0.012				1
PSMA7	proteasome (prosome, macropain) subunit, alpha type, 7	20q13.33	0.55	0.00127	0.0139							1
BUB3	budding uninhibited by benzimidazoles 3 homolog (yeast)	10q26	0.549	9.00E-04	0.0109							1
CRTC2	CREB regulated transcription coactivator 2	1q21.3	0.549	0.00161	0.0164							1
PDGFD	platelet derived growth factor D	11q22.3				0.328857143	0.005144286	0.039042857				1
PDGFRA	platelet-derived growth factor receptor, alpha polypeptide	4q12				-0.31	0.01519	0.0726				1
HNRNPR	heterogeneous nuclear ribonucleoprotein R	1p36.12	0.549	7.00E-05	0.0017							1
RASD2	RASD family, member 2	22q13.1	0.549	0.00319	0.0268							1
PDHX	pyruvate dehydrogenase complex, component X	11p13				0.3305	0.000425	0.00985				1
RGL1	ral guanine nucleotide dissociation stimulator-like 1	1q25.3	0.549	0.04078	0.1474	0.3358	0.001692	0.01992				2
SDF2	stromal cell-derived factor 2	17q11.2	0.549	0.00103	0.0119							1
THRA	thyroid hormone receptor, alpha	17q11.2	0.549	0.02608	0.1103							1
VILL	villin-like	3p21.3	0.549	0.0336	0.1297							1
CCDC82	coiled-coil domain containing 82	11q21	0.5485	0.00641	0.0346							1
PDIK1L	PDLIM1 interacting kinase 1 like	1p36.11	-0.4	0.00901	0.0544							1
PDILT	protein disulfide isomerase-like, testis expressed	16p12.3				0.419	8.00E-04	0.0159				1
PDLIM2	PDZ and LIM domain 2 (mystique)	8p21.3	-0.976	0	1.00E-04							1
PDLIM3	PDZ and LIM domain 3	4q35				-0.4665	0.00847	0.042025				1
PDLIM4	PDZ and LIM domain 4	5q31.1	-0.569	0.00402	0.0314	-0.372	0.00124	0.0195				2
PDLIM5	PDZ and LIM domain 5	4q22	-0.596	0.0045	0.034	-0.3328	0.001796	0.02202				2
PDLIM7	PDZ and LIM domain 7 (enigma)	5q35.3				-0.438	0.00065	0.0145				1
PDPK1	3-phosphoinositide dependent protein kinase-1	16p13.3	-0.5235	0.01486	0.07595	-0.402857143	0.010697857	0.052928571				2
PDPN	podoplanin	1p36.21				-0.401	0.00067	0.0146	0.914	3.91339	5.333333333	2
PDPR	pyruvate dehydrogenase phosphatase regulatory subunit	16q22.1				-0.5296	0.005268	0.03286				1
PDS5A	PDS5, regulator of cohesion maintenance, homolog A (S. cerevisiae)	4p14				-0.374466667	0.002225333	0.02276				1
MADD	MAP-kinase activating death domain	11p11.2	0.548	0.00051	0.0072							1
PDSS1	prenyl (decaprenyl) diphosphate synthase, subunit 1	10p12.1				-0.3295	0.00905	0.05215				1
PDSS2	prenyl (decaprenyl) diphosphate synthase, subunit 2	6q21				-0.346642857	0.001067143	0.016114286				1
HFE	hemochromatosis	6p21.3	0.547	0.00156	0.016							1
PHKG2	phosphorylase kinase, gamma 2 (testis)	16p11.2	0.547	0.00283	0.0246							1
PSME4	proteasome (prosome, macropain) activator subunit 4	2p16.2	0.547	0.00089	0.0108							1
PDXK	pyridoxal (pyridoxine, vitamin B6) kinase	21q22.3				-0.435333333	0.007486667	0.039133333				1
PDZD11	PDZ domain containing 11	Xq13.1	-0.213	0.01763	0.0852							1
PDZD2	PDZ domain containing 2	5p13.3	-2.029	0	1.00E-04							1
DDX39B	DEAD (Asp-Glu-Ala-Asp) box polypeptide 39B	6p21.3	0.5465	0.00334	0.02745							1
PDZD4	PDZ domain containing 4	Xq28	-0.268	0.01731	0.0842							1
PDZD6	Data not found								-0.765	2.80625	8	1
PDZD7	PDZ domain containing 7	10q24.31				-0.342	0.00069	0.0149				1
PDZD9	PDZ domain containing 9	16p12.2	-0.266	0.01078	0.0615							1
RBM12B	RNA binding motif protein 12B	8q22.1	0.546	0.00062	0.0084							1
PDZRN3	PDZ domain containing ring finger 3	3p13	-1.021	0.00164	0.0166							1
PDZRN4	PDZ domain containing ring finger 4	12q12							-0.929	3.24429	7	1
SLC1A7	solute carrier family 1 (glutamate transporter), member 7	1p32.3	0.546	0.00108	0.0123							1
METTL2A	methyltransferase like 2A	17q23.2	0.545	0.00418	0.0322							1
PEBP1	phosphatidylethanolamine binding protein 1	12q24.23	-0.424	0.01406	0.0733	-0.417	2.00E-04	0.0088				2

PEBP4	phosphatidylethanolamine-binding protein 4	8p21.3				-0.350133333	0.005102667	0.036866667				1
PCBD1	pterin-4 alpha-carbinolamine dehydratase/dimerization cofactor of hepatocyt	10q22	0.545	0.01843	0.0879							1
PECI	Data not found					-0.393	0.00013	0.0072				1
SDC3	In multiple Geneids		0.545	0.01529	0.0776							1
YEATS2	YEATS domain containing 2	3q27.1	0.545	0.00517	0.0373							1
PEG3	paternally expressed 3	19q13.4	-0.303	0.00677	0.0448							1
PELI1	pellino E3 ubiquitin protein ligase 1	2p13.3							-0.705	3.022	5	1
B3GAT3	beta-1,3-glucuronyltransferase 3 (glucuronosyltransferase I)	11q12.3	0.544	0.00903	0.0545							1
PELI3	pellino E3 ubiquitin protein ligase family member 3	11q13.2							0.542	2.76167	6	1
C2CD5	Data not found		0.544	0.01563	0.0787							1
PELP1	proline, glutamate and leucine rich protein 1	17p13.2				-0.392666667	0.015471667	0.067016667				1
PEMT	phosphatidylethanolamine N-methyltransferase	17p11.2				-0.3756	0.004754	0.03452				1
PEO1	progressive external ophthalmoplegia 1	10q23.3-24.3							0.608333333	2.522333333	5.666666667	1
PEPD	peptidase D	19q13.11				-0.306	0.01492	0.0719				1
PER1	period homolog 1 (Drosophila)	17p13.1	-1.031	6.00E-05	0.0015	-0.523	0.00016	0.007				2
PER3	period homolog 3 (Drosophila)	1p36.23	-0.973	0.00571	0.04							1
PERP	PERP, TP53 apoptosis effector	6q24	-1.24	0.00153	0.0158							1
PERQ1	PERQ amino acid rich, with GYF domain 1	7q22							0.6875	2.716	5.5	1
PES1	pescadillo ribosomal biogenesis factor 1	22q12.1							-0.684	2.654	5	1
PET112	PET112 homolog (yeast)	4q31.3	-0.366	0.01104	0.0625							1
PET112L	Data not found								-0.846	3.2	7	1
PEX14	peroxisomal biogenesis factor 14	1p36.22	-0.201	0.01499	0.0765	-0.337	0.002695714	0.027285714				2
PEX26	peroxisomal biogenesis factor 26	22q11.21				-0.339	8.00E-05	0.006				1
PEX3	peroxisomal biogenesis factor 3	6q24.2	-0.64	0.00105	0.0121							1
CMTR1	Data not found		0.544	0.00518	0.0374							1
PEX5L	peroxisomal biogenesis factor 5-like	3q26.33				0.317833333	0.0064975	0.038608333				1
PFAS	phosphoribosylformylglycinamidine synthase	17p13.1							-0.895	2.616	5	1
PFDN1	prefoldin subunit 1	5q31	-0.288	0.04067	0.1471	-0.3125	0.00233	0.0253				2
IL3RA	interleukin 3 receptor, alpha (low affinity)	Xp22.3 or Yp11.3	0.544	0.00857	0.0525							1
LIMK1	LIM domain kinase 1	7q11.23	0.543	0.00218	0.0203							1
PFDN5	prefoldin subunit 5	12q12				-0.312	0.00085	0.0164				1
MSH6	mutS homolog 6 (E. coli)	2p16	0.543	0.01716	0.0837							1
PFKFB3	6-phosphofructo-2-kinase/fructose-2,6-biphosphatase 3	10p15.1				-0.353333333	0.015996667	0.073466667				1
PFKFB4	6-phosphofructo-2-kinase/fructose-2,6-biphosphatase 4	3p22-p21				-0.417444444	0.000613333	0.011144444				1
PFKL	phosphofructokinase, liver	21q22.3				-0.322	0.00053	0.0133				1
R3HDM1	R3H domain containing 1	2q21.3	0.543	0.00457	0.0344							1
FAM173B	family with sequence similarity 173, member B	5p15.2	0.542	0.01625	0.0807							1
PFN4	profilin family, member 4	2p23.3	-0.239	0.04162	0.1493							1
PFTK1	Data not found					0.325083333	0.007361667	0.045025				1
PGA3	In multiple Geneids					-0.611	0.000815	0.01345				1
PGA4	In multiple Geneids					-0.7115	5.00E-06	0.00225				1
PGA5	pepsinogen 5, group I (pepsinogen A)	11q13				-0.6995	4.75E-05	0.004075				1
PGAM1	phosphoglycerate mutase 1 (brain)	10q25.3	-0.965333333	0.000263333	0.004033333	-0.486	0	0.0017				2
PGAM4	phosphoglycerate mutase family member 4	Xq13	-0.773	0.00012	0.0025							1
PGAP2	post-GPI attachment to proteins 2	11p15.5	-1.002	0	2.00E-04	-0.441333333	0.003796667	0.0306				2
PGBD1	piggyBac transposable element derived 1	6p22.1							-0.957	2.73143	7	1
PGBD2	piggyBac transposable element derived 2	1q44				0.429	0.00076	0.0156				1
PGCP	Data not found					0.35484	0.000394	0.008256				1
PGD	phosphogluconate dehydrogenase	1p36.22	-1.777	6.00E-05	0.0016	-0.371	0.00811	0.04185				2
PGDS	Data not found								-0.9	3.62333	6	1
SNORA71D	small nucleolar RNA, H/ACA box 71D	20q11.23	0.542	0.008	0.0502							1
PGK2	phosphoglycerate kinase 2	6p12.3							-0.726	3.158	5	1
PGLS	6-phosphogluconolactonase	19p13.2	-0.521	0.0089	0.0539	-0.49	0.0011	0.0184				2
PGLYRP2	peptidoglycan recognition protein 2	19p13.12				-0.394	0.00505	0.0394				1
PGLYRP3	peptidoglycan recognition protein 3	1q21				0.398	0.01644	0.076	-0.868	2.75	5	2
PGLYRP4	peptidoglycan recognition protein 4	1q21	-1.664	1.00E-05	4.00E-04							1
PGM1	phosphoglucomutase 1	1p31	-0.492	0.01724	0.084							1
PGM2	In multiple Geneids		-1.341	0.00034	0.0054							1
SNRPB	small nuclear ribonucleoprotein polypeptides B and B1	20p13	0.542	0.0281	0.1155							1
CDK18	cyclin-dependent kinase 18	1q31-q32	0.541	0.03448	0.1319							1
FPR1	formyl peptide receptor 1	19q13.4	0.541	0.02173	0.098							1
PGPEP1L	pyroglutamyl-peptidase I-like	15q26.3	-0.294	0.02929	0.1186							1
HOXB7	homeobox B7	17q21.3	0.541	0.00015	0.003							1
SLC33A1	solute carrier family 33 (acetyl-CoA transporter), member 1	3q25.31	0.541	0.01392	0.0728							1
PHACTR1	phosphatase and actin regulator 1	6p24.1	-0.387	0.00572	0.04							1
PHACTR2	phosphatase and actin regulator 2	6q24.2	-0.889	0.00493	0.0361							1
PHACTR3	phosphatase and actin regulator 3	20q13.32-q13.33				0.36752	0.0038028	0.029732				1
PHACTR4	phosphatase and actin regulator 4	1p35.3	-1.516	0	1.00E-04	-0.398736842	0.011452632	0.052689474				2
PHAX	In multiple Geneids		-0.321	0.00683	0.0451	-0.41	0.001023333	0.016366667				2

PHC1	polyhomeotic homolog 1 (Drosophila)	12p13	-0.921	0.003645	0.0294							1
PHC3	polyhomeotic homolog 3 (Drosophila)	3q26.2				0.332	0.00084	0.0163				1
PHEX	phosphate regulating endopeptidase homolog, X-linked	Xp22.2-p22.1				0.668	1.00E-05	0.0031				1
CHD7	In multiple Geneids		0.54	0.00149	0.0154	0.3205	0.00057	0.0111				2
MAP3K11	mitogen-activated protein kinase kinase kinase 11	11q13.1-q13.3	0.54	0.00012	0.0025							1
PHF13	PHD finger protein 13	1p36.31				-0.331666667	0.002656667	0.0221				1
PMM2	phosphomannomutase 2	16p13	0.54	0.01733	0.0842							1
PHF15	PHD finger protein 15	5q31.1				-0.382625	0.00257375	0.0238875				1
PHF17	PHD finger protein 17	4q26-q27				-0.416	8.00E-04	0.0159				1
PHF2	PHD finger protein 2	9q22.31				-0.3105	0.00599	0.0415				1
ZNF232	zinc finger protein 232	17p13.2	0.54	0.00423	0.0325							1
PHF20L1	PHD finger protein 20-like 1	8q24.22				0.4707	0.011785	0.05712				1
PHF21A	PHD finger protein 21A	11p11.2				0.507	0.00151	0.0214				1
PHF21B	PHD finger protein 21B	22q13.31	-0.193	0.04728	0.1615	-0.4435	0.001007857	0.012578571				2
PHF23	PHD finger protein 23	17p13.1	-0.395	0.00517	0.0373							1
AAGAB	alpha- and gamma-adaptin binding protein	15q22.33-q23	0.539	0.00709	0.0462							1
PHF5A	PHD finger protein 5A	22q13.2				-0.407	0.00023	0.0092				1
PHF7	PHD finger protein 7	3p21.1	-0.375	0.03482	0.1329	-0.308	0.00011	0.0068				2
PHF8	PHD finger protein 8	Xp11.22				-0.358	0.000715	0.0148				1
PHGDH	phosphoglycerate dehydrogenase	1p12	-1.025	0.01937	0.0909							1
C1orf115	chromosome 1 open reading frame 115	1q41	0.539	0.02722	0.1132							1
PHKA1	phosphorylase kinase, alpha 1 (muscle)	Xq12-q13				0.364	0.000545	0.01245				1
PHKB	phosphorylase kinase, beta	16q12-q13	-0.459	0.01303	0.0698							1
MAPKAPK5-AS1	MAPKAPK5 antisense RNA 1 (non-protein coding)	12q24.12	0.539	1.00E-05	4.00E-04							1
PHLDA1	pleckstrin homology-like domain, family A, member 1	12q15	-1.258	0.00109	0.0124							1
PHLDA3	pleckstrin homology-like domain, family A, member 3	1q31	-0.788	0.00154	0.0159							1
PHF1	PHD finger protein 1	6p21.3	0.539	0.00754	0.0482							1
PHLDB2	pleckstrin homology-like domain, family B, member 2	3q13.2							-0.744	2.69167	6	1
PHLDB3	In multiple Geneids		-0.7255	9.50E-05	0.0021							1
PHLPP1	PH domain and leucine rich repeat protein phosphatase 1	18q21.33	-0.433	0.002	0.0191	-0.371318182	0.004072727	0.031959091				2
WBP5	WW domain binding protein 5	Xq22.2	0.539	0.02369	0.1033							1
PHOX2B	paired-like homeobox 2b	4p12	-0.31	0.01349	0.0713							1
PHRF1	PHD and ring finger domains 1	11p15.5				-0.377	0.00756	0.048333333				1
PHTF1	putative homeodomain transcription factor 1	1p13				-0.336	6.50E-05	0.0054				1
PHTF2	In multiple Geneids								-0.825	3.32	8	1
COMMD7	COMM domain containing 7	20q11.21	0.538	2.00E-04	0.0036							1
PHYHD1	phytanoyl-CoA dioxygenase domain containing 1	9q34.11	-1.673	0	0	-0.357	0.005195	0.03925				2
PHYHIP	phytanoyl-CoA 2-hydroxylase interacting protein	8p21.3	-0.853	1.00E-05	3.00E-04	-0.354	0.00706	0.0473				2
FAM49B	family with sequence similarity 49, member B	8q24.21	0.538	0.01537	0.0778	0.3765	0.0209325	0.085375				2
GNPNAT1	glucosamine-phosphate N-acetyltransferase 1	14q22.1	0.538	0.03302	0.1282							1
PI4K2A	phosphatidylinositol 4-kinase type 2 alpha	10q24	-0.649	0.0031	0.0262	-0.367	0.00096	0.0129				2
PI4K2B	phosphatidylinositol 4-kinase type 2 beta	4p15.2							-1.021	2.612	5	1
PI4KA	phosphatidylinositol 4-kinase, catalytic, alpha	22q11.21				-0.413292683	0.00156	0.01622439				1
PI4KAP1	phosphatidylinositol 4-kinase, catalytic, alpha pseudogene 1	22q11.21				-0.422	0.00288	0.0294				1
PI4KAP2	In multiple Geneids					-0.473666667	0.000255	0.008016667				1
PIAS1	In multiple Geneids								-0.938	2.685	6	1
PIAS2	protein inhibitor of activated STAT, 2	18q21.1				-0.374	0.011275	0.056975				1
PIAS3	protein inhibitor of activated STAT, 3	1q21	-0.61	0.00123	0.0135							1
PIAS4	protein inhibitor of activated STAT, 4	19p13.3				-0.480333333	0.00539	0.0318				1
GSTO1	glutathione S-transferase omega 1	10q25.1	0.538	0.00325	0.0272							1
PICK1	protein interacting with PRKCA 1	22q13.1	-0.296	0.01225	0.0672	-0.514	0.0014375	0.01475				2
PID1	phosphotyrosine interaction domain containing 1	2q36.3				0.3258	0.006706	0.02978				1
PIF1	In multiple Geneids					-0.374	0.00107	0.0182				1
PIGG	phosphatidylinositol glycan anchor biosynthesis, class G	4p16.3				-0.453	0.001812	0.01766				1
SASS6	spindle assembly 6 homolog (C. elegans)	1p21.2	0.538	0.0478	0.1626				-0.502	2.544	5	2
PIGL	phosphatidylinositol glycan anchor biosynthesis, class L	17p12-p11.2				-0.406333333	0.010008667	0.05028				1
TNFRSF14	tumor necrosis factor receptor superfamily, member 14	1p36.32	0.538	0.02218	0.0992							1
PIGN	phosphatidylinositol glycan anchor biosynthesis, class N	18q21.33	-1.194	0	0							1
PIGO	phosphatidylinositol glycan anchor biosynthesis, class O	9p13.3				-0.441	3.00E-05	0.004				1
LOC284751	uncharacterized LOC284751	20q13.13	0.537	0.00902	0.0544							1
NDUFAF6	NADH dehydrogenase (ubiquinone) complex I, assembly factor 6	8q22.1	0.537	0.01523	0.0774							1
PIGV	phosphatidylinositol glycan anchor biosynthesis, class V	1p36.11				-0.365	0.0100875	0.049975				1
PIGY	phosphatidylinositol glycan anchor biosynthesis, class Y	4q22.1							-0.572	2.33	5	1
PIH1D1	PIH1 domain containing 1	19q13.33				-0.3685	0.00189	0.02165				1
PIH1D2	PIH1 domain containing 2	11q23.1	-0.388	0.04443	0.1556							1
SUZ12P1	suppressor of zeste 12 homolog pseudogene 1	17q11.2	0.537	0.00598	0.0412							1
PIK3C2A	phosphoinositide-3-kinase, class 2, alpha polypeptide	11p15.5-p14							-0.791	2.77	5	1
PIK3C2G	phosphoinositide-3-kinase, class 2, gamma polypeptide	12p12				0.346615385	0.000470769	0.010553846				1
PIK3C3	phosphoinositide-3-kinase, class 3	18q12.3							-0.852	2.68	5	1

PIK3CD	phosphoinositide-3-kinase, catalytic, delta polypeptide	1p36.2	-0.256	0.02802	0.1153	-0.418	0.00272375	0.0259625				2
PIK3IP1	phosphoinositide-3-kinase interacting protein 1	22q12.2				-0.44875	0.00278	0.016375				1
PIK3R2	phosphoinositide-3-kinase, regulatory subunit 2 (beta)	19q13.2-q13.4	-0.362	0.0226	0.1003	-0.55	0.002215	0.02215				2
IPO9	importin 9	1q32.1	0.536	0.00124	0.0136							1
PIK3R4	phosphoinositide-3-kinase, regulatory subunit 4	3q22.1				0.381	0.0012	0.0192				1
PIK3R5	phosphoinositide-3-kinase, regulatory subunit 5	17p13.1				-0.372642857	0.002645714	0.0237				1
PIK3R6	phosphoinositide-3-kinase, regulatory subunit 6	17p13.1	-0.456	0.00592	0.041	-0.395222222	0.000693333	0.013266667				2
PPIL1	In multiple Geneids		0.536	0.03301	0.1282							1
PIM1	pim-1 oncogene	6p21.2	-1.328	0.00015	0.0029							1
UTP23	UTP23, small subunit (SSU) processome component, homolog (yeast)	8q24.11	0.536	0.00584	0.0406	0.487666667	0.001713333	0.021733333				2
PIM3	pim-3 oncogene	22q13				-0.302	0.00011	0.0067				1
PIN1	peptidylprolyl cis/trans isomerase, NIMA-interacting 1	19p13	-0.254	0.00745	0.048	-0.447	0.00177	0.019533333				2
PIN1P1	peptidylprolyl cis/trans isomerase, NIMA-interacting 1 pseudogene 1	1p31	-0.208	0.04009	0.1457							1
PINK1	PTEN induced putative kinase 1	1p36	-0.791	0.00027	0.0045	-0.37	0.00731	0.0482				2
PINLYP	Data not found		-0.479	0.00022	0.004							1
PINX1	PIN2/TERF1 interacting, telomerase inhibitor 1	8p23				0.57	0.04105	0.1317				1
PIP4K2B	phosphatidylinositol-5-phosphate 4-kinase, type II, beta	17q12				-0.315	0.01197	0.0633				1
PIP4K2C	phosphatidylinositol-5-phosphate 4-kinase, type II, gamma	12q13.3				-0.393	0.001	0.0176				1
ZNF221	zinc finger protein 221	19q13.2	0.536	0.01257	0.0682							1
PIP5K1C	phosphatidylinositol-4-phosphate 5-kinase, type I, gamma	19p13.3	-0.268	0.01829	0.0875	-0.452142857	0.003525714	0.030071429				2
PIR	pirin (iron-binding nuclear protein)	Xp22.2	-0.769	0.00569	0.0399							1
PIRT	phosphoinositide-interacting regulator of transient receptor potential channe	17p12				-0.396666667	0.00068	0.010833333				1
PITHD1	PITH (C-terminal proteasome-interacting domain of thioredoxin-like) domain (	1p36.11	-0.756	1.00E-04	0.0022							1
PITPNA	phosphatidylinositol transfer protein, alpha	17p13.3	-0.495	0.0112	0.0632	-0.4395	0.01555	0.0707				2
PITPNB	phosphatidylinositol transfer protein, beta	22q12.1	-0.415	0.00165	0.0167	-0.3636	0.000978	0.01234				2
PITPNC1	phosphatidylinositol transfer protein, cytoplasmic 1	17q24.2				0.396	0.00899	0.054				1
PITPNM2	phosphatidylinositol transfer protein, membrane-associated 2	12q24.31	-0.335	0.02853	0.1167	-0.341666667	0.00097	0.0151				2
PITPNM3	PITPNM family member 3	17p13	-1.364	0	2.00E-04	-0.408785714	0.001295	0.0153				2
PITX1	paired-like homeodomain 1	5q31.1	-2.607	0	0	-0.447	3.50E-05	0.0031				2
PITX2	paired-like homeodomain 2	4q25				-0.318	0.01377	0.0686				1
PITX3	paired-like homeodomain 3	10q24.32							-0.831	3.01667	6	1
COPA	coatomer protein complex, subunit alpha	1q23.2	0.535	0.00025	0.0043							1
PIWIL2	piwi-like 2 (Drosophila)	8p21.3				-0.306	0.00214	0.0254				1
PIWIL3	In multiple Geneids					-0.3685	0.006303333	0.03285				1
GDE1	glycerophosphodiester phosphodiesterase 1	16p12-p11.2	0.535	0.00213	0.02							1
PJA1	praja ring finger 1, E3 ubiquitin protein ligase	Xq13.1							-0.838	2.66	7	1
PJA2	praja ring finger 2, E3 ubiquitin protein ligase	5q21.3	-0.484	0.01336	0.0708							1
PKD1	polycystic kidney disease 1 (autosomal dominant)	16p13.3				-0.382666667	0.003023333	0.0252				1
PKD1L1	polycystic kidney disease 1 like 1	7p12.3				0.378666667	0.030863333	0.110166667				1
PKD1L2	polycystic kidney disease 1-like 2	16q23.2				-0.461272727	0.002612727	0.023318182				1
PKD1L3	polycystic kidney disease 1-like 3	16q22.2				-0.336333333	0.016453333	0.064366667				1
PKD2	polycystic kidney disease 2 (autosomal dominant)	4q22.1				-0.361666667	0.003998333	0.028266667				1
PKD2L1	polycystic kidney disease 2-like 1	10q24				-0.384333333	0.001503333	0.0182				1
PKD2L2	polycystic kidney disease 2-like 2	5q31				-0.395	0.001313333	0.016366667				1
LOC728024	chromosome X open reading frame 56 pseudogene	8p11.23	0.535	0.00455	0.0343							1
PKDREJ	polycystic kidney disease (polycystin) and REJ homolog (sperm receptor for eg	22q13.31				-0.425	0.00041	0.0115				1
PKHD1	polycystic kidney and hepatic disease 1 (autosomal recessive)	6p12.2				0.344366667	0.009229	0.051093333	-0.763	2.73	5	2
PKHD1L1	polycystic kidney and hepatic disease 1 (autosomal recessive)-like 1	8q23				0.392703704	0.000901481	0.011748148				1
PKIA	In multiple Geneids					0.342733333	0.000439333	0.008873333				1
PKIB	protein kinase (cAMP-dependent, catalytic) inhibitor beta	6q22.31							-0.82625	2.8595025	5.75	1
PTPN9	protein tyrosine phosphatase, non-receptor type 9	15q24.2	0.535	0.00838	0.0518							1
PKMYT1	protein kinase, membrane associated tyrosine/threonine 1	16p13.3				-0.472	0.00063	0.0143				1
RRP36	ribosomal RNA processing 36 homolog (S. cerevisiae)	6p21.1	0.535	0.03288	0.1279							1
PKN3	protein kinase N3	9q34.11				-0.352	0.00769	0.0496				1
PKNOX1	PBX/knotted 1 homeobox 1	21q22.3				-0.352142857	0.004955714	0.033285714				1
PKNOX2	PBX/knotted 1 homeobox 2	11q24.2				-0.398	0.00064	0.0144				1
PKP1	plakophilin 1 (ectodermal dysplasia/skin fragility syndrome)	1q32				0.356	0.00077	0.0156				1
EFCAB4A	EF-hand calcium binding domain 4A	11p15.5	0.534	0.00464	0.0347							1
PKP3	plakophilin 3	11p15	-1.238	9.00E-05	0.0021							1
PKP4	plakophilin 4	2q24.1				0.359	0.000179091	0.005763636				1
PL-5283	Data not found					-0.358	6.00E-05	0.0055				1
EID3	EP300 interacting inhibitor of differentiation 3	12q23.3	0.534	0.04159	0.1493							1
EPB41L5	erythrocyte membrane protein band 4.1 like 5	2q14.2	0.534	0.00296	0.0253							1
PLA2G12A	phospholipase A2, group XIIA	4q25	-0.494	0.00048	0.0069	-0.351	0.00348	0.0324				2
MAGED2	melanoma antigen family D, 2	Xp11.2	0.534	0.02975	0.1199							1
TBK1	TANK-binding kinase 1	12q14.1	0.534	0.00086	0.0105							1
PLA2G2C	In multiple Geneids		-0.313	0.00479	0.0355							1
PLA2G3	phospholipase A2, group III	22q12.2	-0.66	0.00105	0.0121							1
PLA2G4A	phospholipase A2, group IVA (cytosolic, calcium-dependent)	1q25				0.362571429	0.001985714	0.019071429				1

ZNF259	zinc finger protein 259	11q23.3	0.534	0.00782	0.0494							1
PLA2G4D	phospholipase A2, group IVD (cytosolic)	15q15.1	-0.448	0.02883	0.1175							1
PLA2G4F	phospholipase A2, group IVF	15q15.1	-0.703	0.03364	0.1298							1
PLA2G5	phospholipase A2, group V	1p36-p34				-0.336	0.00155	0.0216				1
PLA2G6	phospholipase A2, group VI (cytosolic, calcium-independent)	22q13.1				-0.4860625	0.001978125	0.0151625				1
B4GALT4	UDP-Gal:betaGlcNAc beta 1,4- galactosyltransferase, polypeptide 4	3q13.3	0.533	0.022	0.0988							1
PLA2R1	phospholipase A2 receptor 1, 180kDa	2q23-q24				0.317	2.00E-05	0.0037				1
PLAA	phospholipase A2-activating protein	9p21							-0.844	3.04167	6	1
PLAC1	placenta-specific 1	Xq26				0.575	7.00E-05	0.0057				1
PLAC2	In multiple Geneids					-0.51525	0.000585	0.01315				1
PLAC8	placenta-specific 8	4q21.22							-0.823	3.17278	8.5	1
PLAC8L1	In multiple Geneids								-1.045	3.39	7	1
PLAT	plasminogen activator, tissue	8p12				-0.322	0.01223	0.0641				1
DNPH1	Data not found		0.533	0.02304	0.1014							1
FLCN	folliculin	17p11.2	0.533	0.00236	0.0215							1
MTFR2	Data not found		0.533	0.04603	0.159							1
MYO5C	myosin VC	15q21	0.533	0.04022	0.1461							1
PLCB2	phospholipase C, beta 2	15q15				-0.328	0.00048	0.0126				1
PLCB4	phospholipase C, beta 4	20p12				0.333230769	0.009403846	0.052992308	-0.94	3.65714	7	2
PLCD1	phospholipase C, delta 1	3p22-p21.3	-1.576	0	1.00E-04							1
PLCD3	In multiple Geneids					-0.315	0.0161	0.0751				1
PLCD4	In multiple Geneids								-0.743	2.616	5	1
NSL1	NSL1, MIND kinetochore complex component, homolog (S. cerevisiae)	1q41	0.533	0.0066	0.0441							1
PLCG2	phospholipase C, gamma 2 (phosphatidylinositol-specific)	16q24.1				-0.3224	0.006532	0.04196				1
SAE1	SUMO1 activating enzyme subunit 1	19q13.32	0.533	0.00028	0.0046							1
PLCH2	phospholipase C, eta 2	1p36.32	-0.699	1.00E-05	3.00E-04							1
PLCL1	phospholipase C-like 1	2q33				0.327833333	0.002183333	0.019016667				1
TMTC4	transmembrane and tetratricopeptide repeat containing 4	13q32.3	0.533	0.02642	0.1112							1
PLCXD1	phosphatidylinositol-specific phospholipase C, X domain containing 1	Xp22.33; Yp11.32				-0.407	0.00014	0.0076				1
PLCXD3	phosphatidylinositol-specific phospholipase C, X domain containing 3	5p13.1				0.452296296	0.009138889	0.049583333				1
PLCZ1	phospholipase C, zeta 1	12p12.3							-0.768	3.175	6	1
PLD1	phospholipase D1, phosphatidylcholine-specific	3q26				0.3376	0.003976	0.02954				1
PLD2	phospholipase D2	17p13.1	-1.31	0	2.00E-04							1
PLD3	phospholipase D family, member 3	19q13.2				-0.422	0.00422	0.0312				1
PLD4	phospholipase D family, member 4	14q32.33	-0.279	0.02769	0.1144							1
PLD5	phospholipase D family, member 5	1q43				0.338	0.00206	0.0249				1
PLEC1	Data not found								0.475	2.472	5	1
TRAPPC4	trafficking protein particle complex 4	11q23.3	0.533	0.00385	0.0305							1
KIF13B	kinesin family member 13B	8p12	0.532	0.02485	0.1066							1
PLEKHA1	pleckstrin homology domain containing, family A (phosphoinositide binding sp	10q26.13							-0.989	2.9125	8	1
PLEKHA2	pleckstrin homology domain containing, family A (phosphoinositide binding sp	8p11.22				1.769	0	8.00E-04				1
MCTP2	multiple C2 domains, transmembrane 2	15q26.2	0.532	0.01335	0.0708	0.35975	0.00363	0.0305				2
PLEKHA4	pleckstrin homology domain containing, family A (phosphoinositide binding sp	19q13.33				-0.40975	0.0059825	0.03885				1
PLEKHA5	pleckstrin homology domain containing, family A member 5	12p12				0.3368	0.012408	0.06354				1
MYO7A	myosin VIIA	11q13.5	0.532	0.00343	0.0281							1
PLEKHA7	pleckstrin homology domain containing, family A member 7	11p15.1	-0.601	0.01953	0.0913	-0.307	0.00572	0.04035				2
CASD1	CAS1 domain containing 1	7q21.3	0.531	0.04143	0.1489							1
FAM101B	family with sequence similarity 101, member B	17p13	0.531	0.00256	0.0228							1
MSTO1	misato homolog 1 (Drosophila)	1q22	0.531	0.01382	0.0725							1
PLEKHF2	pleckstrin homology domain containing, family F (with FYVE domain) member	8q22.1				0.313	0.00021	0.009				1
PLEKHG1	pleckstrin homology domain containing, family G (with RhoGef domain) meml	6q25.1				-0.38	0.00387	0.0343				1
OR12D3	olfactory receptor, family 12, subfamily D, member 3	6p22.1	0.531	0.00781	0.0494							1
PLEKHG3	pleckstrin homology domain containing, family G (with RhoGef domain) meml	14q23.3				-0.3835	4.00E-04	0.01165				1
PLEKHG4B	pleckstrin homology domain containing, family G (with RhoGef domain) meml	5p15.33				0.341	0.007326	0.0445				1
PLEKHG5	pleckstrin homology domain containing, family G (with RhoGef domain) meml	1p36.31	-0.407	0.00323	0.027	-0.473	0.001052857	0.014328571				2
SETDB1	SET domain, bifurcated 1	1q21	0.531	0.00804	0.0504							1
PLEKHH2	pleckstrin homology domain containing, family H (with MyTH4 domain) memb	2p21				-0.407	0.00025	0.0096				1
PLEKHH3	pleckstrin homology domain containing, family H (with MyTH4 domain) memb	17q21.2	-0.634	0	2.00E-04	-0.31	0.00309	0.0304				2
PLEKHM1	pleckstrin homology domain containing, family M (with RUN domain) membe	17q21.31	-1.216	5.00E-05	0.0013	-0.354	0.00071	0.0151				2
PLEKHM1P	pleckstrin homology domain containing, family M (with RUN domain) membe	17q24.1	-0.734	0.00495	0.0363	-0.312	0.03922	0.128				2
PLEKHM2	pleckstrin homology domain containing, family M (with RUN domain) membe	1p36.21	-0.414	0.00164	0.0166	-0.3785	0.001085	0.0181				2
PLEKHM3	pleckstrin homology domain containing, family M, member 3	2q33.3				-0.34	0.0386	0.1268				1
PLEKHN1	pleckstrin homology domain containing, family N member 1	1p36.33	-1.231	0	0							1
PLEKHO2	pleckstrin homology domain containing, family O member 2	15q22.1				-0.351	0.00411	0.029833333				1
SND1-IT1	SND1 intronic transcript 1 (non-protein coding)	7q31	0.531	0.01316	0.0701							1
PLGRKT	plasminogen receptor, C-terminal lysine transmembrane protein	9p24.1	-0.414	0.01452	0.0748							1
PLIN	Data not found								-0.998	2.63667	6	1
SUGT1	SGT1, suppressor of G2 allele of SKP1 (S. cerevisiae)	13q14.3	0.531	0.00188	0.0183							1
PLIN3	perilipin 3	19p13.3	-1.25	0.00013	0.0027	-0.34	0.00446	0.03685				2

PLIN4	perilipin 4	19p13.3				-0.6015	0.00056	0.01305				1
ZC4H2	zinc finger, C4H2 domain containing	Xq11.2	0.531	0.04936	0.1658	0.381	0.00124	0.0194				2
GRIN2D	glutamate receptor, ionotropic, N-methyl D-aspartate 2D	19q13.33	0.53	0.00031	0.005							1
PLK3	polo-like kinase 3	1p34.1	-0.807	0.00166	0.0167							1
PLK5	polo-like kinase 5	19p13.3	-0.205	0.02876	0.1173							1
PLK5P	Data not found					-0.49	0.00037	0.0113				1
PLLP	plasmolipin	16q13				-0.38725	0.00013	0.006625				1
PLN	phospholamban	6q22.1							-0.992	3.27	7	1
NBPF15	neuroblastoma breakpoint family, member 15	1q21.2	0.53	0.00607	0.0417							1
SLC25A44	solute carrier family 25, member 44	1q22	0.53	0.02509	0.1073							1
VPS54	vacuolar protein sorting 54 homolog (S. cerevisiae)	2p13-p14	0.53	0.03823	0.1415							1
PLP1	proteolipid protein 1	Xq22	-0.898	0.00109	0.0124							1
PLP2	proteolipid protein 2 (colonic epithelium-enriched)	Xp11.23	-0.682	0.00866	0.0529							1
PLRG1	pleiotropic regulator 1	4q31.2-q32.1	-0.443	0.01083	0.0617							1
MFSD12	major facilitator superfamily domain containing 12	19p13.3	0.529	0.00672	0.0446							1
PLS3	plastin 3	Xq23	-1.069	0.00012	0.0025							1
PLSCR1	phospholipid scramblase 1	3q23				0.3135	0.001045	0.01655				1
PLSCR2	phospholipid scramblase 2	3q24				0.362	0.00307	0.0303				1
PLSCR3	phospholipid scramblase 3	17p13.1	-0.525	0.00096	0.0114	-0.497	0.00044	0.01225				2
PLSCR4	phospholipid scramblase 4	3q24				0.318	0.00211	0.0252				1
PLSCR5	phospholipid scramblase family, member 5	3q24				0.36225	0.006275	0.037575				1
SPNS1	spinster homolog 1 (Drosophila)	16p11.2	0.529	0.00087	0.0107							1
HSD17B13	In multiple Geneids		0.527	0.00247	0.0222							1
PLXDC2	plexin domain containing 2	10p12.31	-1.031	0.02329	0.1022	-0.425	4.00E-05	0.0047				2
PLXNA2	plexin A2	1q32.2				0.330333333	0.000606667	0.012483333				1
MAFK	In multiple Geneids		0.527	0.01201	0.0662							1
PLXNA4	In multiple Geneids					0.572	0.000515	0.00915				1
PLXNB1	plexin B1	3p21.31				-0.357	3.00E-04	0.0103				1
PLXNB2	plexin B2	22q13.33				-0.572	0.00027	0.0098				1
SNORD63	small nucleolar RNA, C/D box 63	5q31.2	0.527	0.0144	0.0745							1
TMEM245	transmembrane protein 245	9q31	0.527	0.00621	0.0423							1
PM20D1	peptidase M20 domain containing 1	1q32.1	-0.499	0.01692	0.083							1
PMAIP1	phorbol-12-myristate-13-acetate-induced protein 1	18q21.32	-0.899	0.00406	0.0317							1
PMCH	pro-melanin-concentrating hormone	12q23.2							-0.985	3.56375	8	1
FAM96A	family with sequence similarity 96, member A	15q22.31	0.526	0.00034	0.0054							1
PMFBP1	polyamine modulated factor 1 binding protein 1	16q22.2							-0.807	2.676	5	1
PMM1	phosphomannomutase 1	22q13.2	-1.269	0	2.00E-04	-0.386	0.00369	0.0335				2
LOXL4	lysyl oxidase-like 4	10q24	0.526	0.02273	0.1006							1
LRRC6	leucine rich repeat containing 6	8q24.22	0.526	0.04479	0.1563	0.383333333	0.019460952	0.081095238				2
PMP22CD	Data not found								-0.959	3.69	5	1
STC2	stanniocalcin 2	5q35.1	0.526	0.00604	0.0415							1
PMS1	PMS1 postmeiotic segregation increased 1 (S. cerevisiae)	2q31.1							-0.804	3.18667	6	1
PMS2	PMS2 postmeiotic segregation increased 2 (S. cerevisiae)	7p22.2				-0.364	0.00763	0.0479				1
PMS2L1	Data not found					-0.462	0.00115	0.0188				1
PMS2L2	postmeiotic segregation increased 2-like 2 pseudogene	7q11.23				-0.4495	0.00378	0.0286				1
PMS2L5	Data not found					-0.371	0.01672	0.0768				1
CDH5	cadherin 5, type 2 (vascular endothelium)	16q22.1	0.525	0.01678	0.0825							1
PMVK	phosphomevalonate kinase	1q22	-0.384	0.00214	0.02							1
FGFR1OP2	In multiple Geneids		0.525	0.00045	0.0067							1
PNLDC1	poly(A)-specific ribonuclease (PARN)-like domain containing 1	6q25.3				-0.31	0.02213	0.0905				1
KIAA1598	KIAA1598	10q25.3	0.525	0.00068	0.009							1
PNLIPRP3	pancreatic lipase-related protein 3	10q25.3							-0.921	3.1925	8	1
SDCBP	syndecan binding protein (syntenin)	8q12	0.525	0.01444	0.0746							1
PNMA2	paraneoplastic Ma antigen 2	8p21.2				-0.303	6.00E-04	0.014				1
USF1	upstream transcription factor 1	1q22-q23	0.525	0.00045	0.0066							1
GUSBP1	In multiple Geneids		0.5245	0.017495	0.07655							1
PNP	purine nucleoside phosphorylase	14q13.1	-0.544	0.04464	0.156							1
ZNF618	zinc finger protein 618	9q32	0.5245	0.024315	0.1027	0.395	0.0258	0.0991				2
PNPLA3	patatin-like phospholipase domain containing 3	22q13.31				-0.4274	0.002744	0.02338				1
PNPLA4	patatin-like phospholipase domain containing 4	Xp22.3				0.348	0.00235	0.0266				1
PNPLA5	patatin-like phospholipase domain containing 5	22q13.31				-0.45825	0.000435	0.01005				1
PNPLA6	patatin-like phospholipase domain containing 6	19p13.2	-0.291	0.02731	0.1134	-0.389	0.0094925	0.048975				2
PNPLA7	patatin-like phospholipase domain containing 7	9q34.3				-0.34	0.00389	0.0344				1
PNPO	In multiple Geneids								1.273	2.64	5	1
GBAP1	glucosidase, beta, acid pseudogene 1	1q21	0.524	0.01568	0.0789							1
PNRC1	proline-rich nuclear receptor coactivator 1	6q15				-0.366	0.00065	0.0145				1
HNRNPF	heterogeneous nuclear ribonucleoprotein F	10q11.21	0.524	0.00155	0.016							1
HOXB8	homeobox B8	17q21.3	0.524	0.03927	0.1438							1
PODN	podocan	1p32.3				-0.307	0.00483	0.0385				1

PODNL1	podocan-like 1	19p13.12	-0.595	0.02092	0.0955	-0.368	0.00475	0.0382				2
HSPE1	heat shock 10kDa protein 1 (chaperonin 10)	2q33.1	0.524	0.00482	0.0356				-0.539	2.302	5	2
ITFG1	integrin alpha FG-GAP repeat containing 1	16q12.1	0.524	0.00419	0.0323							1
POF1B	premature ovarian failure, 1B	Xq21.2				0.338666667	0.001556667	0.0152	-0.842	3.06571	7	2
TES	testis derived transcript (3 LIM domains)	7q31.2	0.524	0.00385	0.0305	0.3515	4.50E-05	0.00495	-0.823	3.15167	6	3
TRAF3IP1	TNF receptor-associated factor 3 interacting protein 1	2q37.3	0.524	0.00474	0.0352							1
TSN	translin	2q21.1	0.524	0.00207	0.0195							1
POL3S	Data not found					-0.367	0.00045	0.0124				1
POLD1	polymerase (DNA directed), delta 1, catalytic subunit	19q13.3				-0.3945	0.004965	0.0344				1
BRI3	brain protein I3	7q21.3	0.523	0.0061	0.0418							1
POLDIP3	polymerase (DNA-directed), delta interacting protein 3	22q13.2	-0.463	0.00041	0.0062	-0.483	0.00077	0.0117				2
POLE	polymerase (DNA directed), epsilon, catalytic subunit	12q24.3				-0.345333333	0.001281667	0.01675				1
POLE2	polymerase (DNA directed), epsilon 2, accessory subunit	14q21-q22				-0.302	0.01737	0.0785				1
CDK11B	cyclin-dependent kinase 11B	1p36.33	0.523	0.04976	0.1666							1
CDK4	cyclin-dependent kinase 4	12q14	0.523	0.04558	0.1583							1
POLL	polymerase (DNA directed), lambda	10q23				-0.331	0.00015	0.0076				1
POLN	polymerase (DNA directed) nu	4p16.3	-0.398	0.00638	0.0431	-0.421954545	0.001555	0.018477273				2
EPHX4	epoxide hydrolase 4	1p22.1	0.523	0.01612	0.0803							1
POLR1C	In multiple Geneids					0.345	0.02038	0.0862				1
POLR1D	polymerase (RNA) I polypeptide D, 16kDa	13q12.2				0.304	0.00604	0.0434				1
POLR1E	polymerase (RNA) I polypeptide E, 53kDa	9p13.2				-0.367	2.00E-05	0.0037				1
POLR2A	polymerase (RNA) II (DNA directed) polypeptide A, 220kDa	17p13.1	-0.477	0.00244	0.022	-0.399333333	0.003883333	0.032366667				2
POLR2B	polymerase (RNA) II (DNA directed) polypeptide B, 140kDa	4q12				-0.32	0.016335	0.0751				1
M6PR	mannose-6-phosphate receptor (cation dependent)	12p13	0.523	0.01089	0.0619							1
POLR2E	polymerase (RNA) II (DNA directed) polypeptide E, 25kDa	19p13.3				-0.552	0.00114	0.0188				1
POLR2F	polymerase (RNA) II (DNA directed) polypeptide F	22q13.1				-0.5858	6.00E-05	0.00526				1
MRAS	muscle RAS oncogene homolog	3q22.3	0.523	0.04911	0.1652							1
PLEKHA6	pleckstrin homology domain containing, family A member 6	1q32.1	0.523	0.00804	0.0504	0.323	0.00091	0.0169				2
POLR2J	polymerase (RNA) II (DNA directed) polypeptide J, 13.3kDa	7q22.1				-0.504	0.00093	0.0171				1
POLR2J2	polymerase (RNA) II (DNA directed) polypeptide J2	7q22.1				-0.481	0.00084	0.013966667				1
PPM1B	protein phosphatase, Mg2+/Mn2+ dependent, 1B	2p21	0.523	0.00041	0.0062							1
POLR2J4	polymerase (RNA) II (DNA directed) polypeptide J4, pseudogene	7p13				-0.486714286	0.002198571	0.022428571				1
BTN2A3P	butyrophilin, subfamily 2, member A3, pseudogene	6p22.1	0.522	0.00268	0.0236							1
POLR2L	polymerase (RNA) II (DNA directed) polypeptide L, 7.6kDa	11p15	-0.451	0.00057	0.0079							1
HOXC8	homeobox C8	12q13.3	0.522	0.00644	0.0433							1
POLR3A	polymerase (RNA) III (DNA directed) polypeptide A, 155kDa	10q22-q23				-0.3606	0.00049	0.01032				1
POLR3B	polymerase (RNA) III (DNA directed) polypeptide B	12q23.3				0.3195	0.019165	0.0694				1
POLR3D	polymerase (RNA) III (DNA directed) polypeptide D, 44kDa	8q21				-0.3815	0.005645	0.0404				1
MAPRE1	microtubule-associated protein, RP/EB family, member 1	20q11.1-q11.23	0.522	0.00114	0.0128							1
METTL5	methyltransferase like 5	2q31.1	0.522	0.00015	0.003							1
POLR3H	polymerase (RNA) III (DNA directed) polypeptide H (22.9kD)	22q13.2	-0.334	0.00638	0.0431	-0.472	0.001024	0.01368				2
MLXIP	MLX interacting protein	12q24.31	0.522	0.01158	0.0646							1
POLS	Data not found					0.342	0.001386667	0.020266667				1
THEM4	thioesterase superfamily member 4	1q21	0.522	0.00614	0.042							1
POM121C	POM121 transmembrane nucleoporin C	7q11.2				-0.3565	0.025175	0.0974				1
POM121L10P	POM121 transmembrane nucleoporin-like 10, pseudogene	22q11.23				-0.543	0.00017	0.0081				1
POM121L1P	POM121 transmembrane nucleoporin-like 1, pseudogene	22q11.22	-0.486	0.02408	0.1044							1
POM121L2	POM121 transmembrane nucleoporin-like 2	6p22.1	-0.251	0.04463	0.156							1
POM121L4P	POM121 transmembrane nucleoporin-like 4 pseudogene	22q11.2	-0.42	3.00E-04	0.0049							1
POM121L8P	POM121 transmembrane nucleoporin-like 8 pseudogene	22q11.2	-0.339	0.00209	0.0197							1
POMC	proopiomelanocortin	2p23.3	-0.342	0.00124	0.0136				0.683	2.408	5	2
ZNF578	zinc finger protein 578	19q13.41	0.522	0.00733	0.0473							1
ABHD15	abhydrolase domain containing 15	17q11.2	0.521	0.00082	0.0102							1
POMT1	protein-O-mannosyltransferase 1	9q34.1				-0.346	0.00607	0.0409				1
POMT2	protein-O-mannosyltransferase 2	14q24				-0.416	0.00012	0.0071				1
GANAB	In multiple Geneids		0.521	0.00321	0.0269							1
ASRGL1	In multiple Geneids		0.52	0.02229	0.0995							1
POP1	processing of precursor 1, ribonuclease P/MRP subunit (S. cerevisiae)	8q22.1				0.302	0.0012	0.0192				1
CREBZF	CREB/ATF bZIP transcription factor	11q14	0.52	0.00205	0.0194							1
POR	P450 (cytochrome) oxidoreductase	7q11.2				-0.346	0.04594	0.1412				1
PORCN	porcupine homolog (Drosophila)	Xp11.23	-1.952	0	2.00E-04							1
GALK2	galactokinase 2	15q21.1-q21.2	0.52	0.01072	0.0613							1
HIST1H4E	histone cluster 1, H4e	6p22.1	0.52	0.01409	0.0734							1
POTE14	Data not found								0.837	2.845	8	1
POTE15	Data not found								0.513	2.525	6	1
POTE2	Data not found								0.961	3.36875	8	1
RAP1B	RAP1B, member of RAS oncogene family	12q14	0.52	0.00831	0.0515							1
RGCC	regulator of cell cycle	13q14.11	0.52	0.03112	0.1237							1
SET	SET nuclear oncogene	9q34	0.52	0.005305	0.0336							1

POU1F1	POU class 1 homeobox 1	3p11							-0.947	3.18167	6	1
SNRNP200	small nuclear ribonucleoprotein 200kDa (U5)	2q11.2	0.52	0.00478	0.0354							1
POU2F2	POU class 2 homeobox 2	19q13.2				-0.34	0.01094	0.0602				1
POU2F3	POU class 2 homeobox 3	11q23.3	-0.457	0.00069	0.0091							1
POU3F1	POU class 3 homeobox 1	1p34.1	-0.567	0.00011	0.0024							1
POU4F2	POU class 4 homeobox 2	4q31.2							0.679	2.646	5	1
POU4F3	POU class 4 homeobox 3	5q32	-0.361	0.00034	0.0054							1
POU5F1	POU class 5 homeobox 1	6p21.31							1.206	2.462	5	1
POU5F2	POU domain class 5, transcription factor 2	5q15	-0.19	0.0343	0.1314							1
POU6F1	POU class 6 homeobox 1	12q13.13	-0.225	0.04094	0.1478							1
C6orf211	chromosome 6 open reading frame 211	6q25.1	0.519	0.02875	0.1173							1
PP13	uncharacterized LOC100129503	17q25.3	-0.465	0.01097	0.0622							1
PP14571	uncharacterized LOC100130449	2q37.3	-0.328	0.00485	0.0358							1
PPA2	pyrophosphatase (inorganic) 2	4q25				-0.335	0.00356	0.0328				1
EFHD2	EF-hand domain family, member D2	1p36.21	0.519	0.04224	0.1507							1
PILRA	paired immunoglobin-like type 2 receptor alpha	7q22.1	0.519	0.00422	0.0325							1
PPAPDC1A	phosphatidic acid phosphatase type 2 domain containing 1A	10q26.12				-0.3	0.00472	0.038				1
RSBN1L	round spermatid basic protein 1-like	7q11.23	0.519	0.02418	0.1047							1
PPAPDC2	phosphatidic acid phosphatase type 2 domain containing 2	9p24.1	-0.524	0.00953	0.0565	-0.387	0.000735	0.0153				2
PPARA	peroxisome proliferator-activated receptor alpha	22q13.31				-0.385692308	0.000878462	0.012807692				1
PPARD	peroxisome proliferator-activated receptor delta	6p21.2				-0.357	0.00738	0.0484				1
TMED5	transmembrane emp24 protein transport domain containing 5	1pter-q31.3	0.519	0.00095	0.0113							1
PPARGC1A	peroxisome proliferator-activated receptor gamma, coactivator 1 alpha	4p15.1							-0.955	3.14286	7	1
PPARGC1B	peroxisome proliferator-activated receptor gamma, coactivator 1 beta	5q32	-0.614	7.00E-05	0.0016	-0.348933333	0.001409333	0.015993333				2
PPAT	phosphoribosyl pyrophosphate amidotransferase	4q12				-0.327	0.00276	0.0288				1
PPBP	pro-platelet basic protein (chemokine (C-X-C motif) ligand 7)	4q12-q13							-1.055	3.325	6	1
PPCDC	phosphopantothenoylcysteine decarboxylase	15q24.2				-0.372	0.0268	0.1014				1
PPDPF	pancreatic progenitor cell differentiation and proliferation factor homolog (ze	20q13.33	-0.688	0.00113	0.0127							1
PPEF2	protein phosphatase, EF-hand calcium binding domain 2	4q21.1				-0.337333333	0.005306667	0.035133333				1
C1QTNF1	C1q and tumor necrosis factor related protein 1	17q25.3	0.518	0.01538	0.0778							1
PPFIA2	protein tyrosine phosphatase, receptor type, f polypeptide (PTPRF), interactin	12q21.31				0.313666667	0.013186667	0.066133333				1
PPFIA3	protein tyrosine phosphatase, receptor type, f polypeptide (PTPRF), interactin	19q13.33				-0.391857143	0.003402857	0.030485714				1
PPFIBP1	PTPRF interacting protein, binding protein 1 (liprin beta 1)	12p12.1				0.38	5.00E-04	0.0119				1
PPFIBP2	PTPRF interacting protein, binding protein 2 (liprin beta 2)	11p15.4	-0.561	0.01332	0.0706	-0.361	0.00215	0.0254				2
PPGB	Galactosialidosis								0.773	2.39	5	1
CEP95	centrosomal protein 95kDa	17q23.3	0.518	0.00247	0.0222							1
PPIC	peptidylprolyl isomerase C (cyclophilin C)	5q23.2				-0.301	0.00031	0.0105				1
HIST1H2BO	histone cluster 1, H2bo	6p22.1	0.518	0.01137	0.0638							1
SLC35A2	solute carrier family 35 (UDP-galactose transporter), member A2	Xp11.23-p11.22	0.518	0.00766	0.0487							1
SNORA38	small nucleolar RNA, H/ACA box 38	6p21.33	0.518	0.0483	0.1637							1
ST8SIA4	ST8 alpha-N-acetyl-neuraminide alpha-2,8-sialyltransferase 4	5q21	0.518	0.04994	0.167							1
PPIL2	peptidylprolyl isomerase (cyclophilin)-like 2	22q11.21	-0.431	0.00047	0.0068	-0.419	0.00428	0.02875				2
PPIL3	peptidylprolyl isomerase (cyclophilin)-like 3	2q33.1	-0.442	0.03403	0.1308							1
SUGT1P3	suppressor of G2 allele of SKP1 (S. cerevisiae) pseudogene 3	13q14.11	0.518	0.00047	0.0068							1
PPL	periplakin	16p13.3	-3.405	0	2.00E-04	-0.437636364	0.01919	0.075754545				2
PPM1A	protein phosphatase, Mg2+/Mn2+ dependent, 1A	14q23.1	-0.326	0.02792	0.1151							1
ZBTB26	zinc finger and BTB domain containing 26	9q33.2	0.518	0.01242	0.0677							1
PPM1E	protein phosphatase, Mg2+/Mn2+ dependent, 1E	17q22				-0.305	0.00539	0.0408				1
PPM1F	protein phosphatase, Mg2+/Mn2+ dependent, 1F	22q11.22	-0.377	0.00107	0.0122	-0.420777778	0.000823333	0.013011111				2
BCL2L11	BCL2-like 11 (apoptosis facilitator)	2q13	0.517	0.02424	0.1048	0.354666667	5.67E-05	0.003666667				2
PPM1J	protein phosphatase, Mg2+/Mn2+ dependent, 1J	1p13.2	-0.25	0.02689	0.1123							1
PPM1K	protein phosphatase, Mg2+/Mn2+ dependent, 1K	4q22.1							-0.542	2.454	5	1
PPM1L	protein phosphatase, Mg2+/Mn2+ dependent, 1L	3q26.1				0.317	0.00101	0.01506	-0.774	2.45857	7	2
PPM1M	protein phosphatase, Mg2+/Mn2+ dependent, 1M	3p21.2	-0.4	0.00078	0.0098							1
PPOX	protoporphyrinogen oxidase	1q22	-0.278	0.04194	0.15	-0.316	0.00239	0.0268				2
PPP1CA	protein phosphatase 1, catalytic subunit, alpha isozyme	11q13	-0.319	0.01723	0.0839	-0.324	0.00265	0.0282				2
PPP1CB	protein phosphatase 1, catalytic subunit, beta isozyme	2p23	-0.821	2.00E-05	6.00E-04							1
PPP1R12B	protein phosphatase 1, regulatory subunit 12B	1q32.1							-0.91	3.15143	7	1
PPP1R12C	protein phosphatase 1, regulatory subunit 12C	19q13.42				-0.46	0.00094	0.0172	1.286	4.34571	7	2
PPP1R13B	protein phosphatase 1, regulatory subunit 13B	14q32.33	-0.901	3.00E-05	0.001	-0.34325	0.0014575	0.01975				2
PPP1R13L	protein phosphatase 1, regulatory subunit 13 like	19q13.32							-0.662	2.33	5	1
PPP1R14A	In multiple Geneids					-0.383	0.00067	0.0147				1
PPP1R14C	protein phosphatase 1, regulatory (inhibitor) subunit 14C	6q24.3-q25.3	-0.347	0.03751	0.1397	-0.34	0.00059	0.0138				2
BRPF3	bromodomain and PHD finger containing, 3	6p21	0.517	0.03573	0.1352							1
PPP1R15A	protein phosphatase 1, regulatory subunit 15A	19q13.2				-0.41	0.00148	0.0212				1
PPP1R16A	protein phosphatase 1, regulatory subunit 16A	8q24.3	-0.417	0.00167	0.0168							1
DUSP12	dual specificity phosphatase 12	1q21-q22	0.517	0.00027	0.0046							1
PPP1R1C	In multiple Geneids					0.31875	0.0002725	0.008025				1
PPP1R2P9	In multiple Geneids		-0.261	0.00929	0.0555							1

PPP1R3A	protein phosphatase 1, regulatory subunit 3A	7q31.1				0.327666667	0.000323333	0.010366667	-0.898	3.26714	7	2
PPP1R3C	protein phosphatase 1, regulatory subunit 3C	10q23-q24	-3.078	0	0							1
PPP1R3D	protein phosphatase 1, regulatory subunit 3D	20q13.3				0.405	0.00538	0.0408				1
PPP1R3E	In multiple Geneids		-0.586	4.00E-05	0.0011							1
PPP1R3F	protein phosphatase 1, regulatory subunit 3F	Xp11.23	-0.222	0.03275	0.1277							1
PPP1R3G	In multiple Geneids					-0.626	1.00E-04	0.0065				1
PPP1R7	protein phosphatase 1, regulatory subunit 7	2q37.3	-0.352	0.00542	0.0386							1
PPP1R8	protein phosphatase 1, regulatory subunit 8	1p35.3				-0.3492	0.016042	0.07336				1
PPIB	peptidylprolyl isomerase B (cyclophilin B)	15q21-q22	0.517	0.00065	0.0087							1
PPP1R9B	In multiple Geneids					-0.366	0.00154	0.0216				1
PPP2CA	protein phosphatase 2, catalytic subunit, alpha isozyme	5q31.1	-0.629	0.00107	0.0123	-0.331	0.000985	0.0156				2
PPP2R2A	protein phosphatase 2, regulatory subunit B, alpha	8p21.2	-0.647	0.00309	0.0262							1
PPP2R2B	protein phosphatase 2, regulatory subunit B, beta	5q32				0.6705	0	0.0012	-0.916	2.876	5	2
PPP2R2C	protein phosphatase 2, regulatory subunit B, gamma	4p16.1	-2.698	0	0	-0.376666667	0.003521667	0.026075	1.214	2.702	5	3
PPP2R2D	protein phosphatase 2, regulatory subunit B, delta	10q26.3				-0.313	2.00E-05	0.0037				1
PPP2R3A	protein phosphatase 2, regulatory subunit B'', alpha	3q22.1				0.422	8.00E-04	0.0159	-1.027	2.81	7	2
PPP2R3B	In multiple Geneids		-0.278	0.01027	0.0597	-0.515333333	0.001503333	0.016188889				2
STX1A	syntaxin 1A (brain)	7q11.23	0.517	0.00108	0.0123							1
PPP2R5A	protein phosphatase 2, regulatory subunit B', alpha	1q32.2-q32.3	-0.666	0.00038	0.0059							1
ZWINT	ZW10 interactor	10q21-q22	0.517	0.03045	0.1219				-0.589	2.558	5	2
PPP2R5C	protein phosphatase 2, regulatory subunit B', gamma	14q32.31							-0.745	2.596	5	1
PPP2R5E	protein phosphatase 2, regulatory subunit B', epsilon isoform	14q23.1	-0.302	0.01136	0.0638	-0.368666667	0.004086667	0.032666667				2
MBTPS2	membrane-bound transcription factor peptidase, site 2	Xp22.12-p22.11	0.516	0.03502	0.1334				-0.855	2.73	5	2
PPP3CC	protein phosphatase 3, catalytic subunit, gamma isozyme	8p21.3	-0.465	0.00825	0.0512	-0.451	8.00E-04	0.0159				2
PPP4C	protein phosphatase 4, catalytic subunit	16p11.2				-0.335	0.00112	0.0186				1
PPP4R1	protein phosphatase 4, regulatory subunit 1	18p11.22	-1.194	1.00E-05	5.00E-04	-0.374	0.00078	0.0158				2
NT5DC3	5'-nucleotidase domain containing 3	12q22-q23.1	0.516	0.0098	0.0576							1
PPP4R2	In multiple Geneids					-0.3815	5.50E-05	0.00535				1
PPPDE2	Data not found					-0.404	0.0033025	0.02815				1
PPRC1	peroxisome proliferator-activated receptor gamma, coactivator-related 1	10q24.32				-0.399	0.000613333	0.0136				1
NUDC	nuclear distribution C homolog (A. nidulans)	1p35-p34	0.516	0.00828	0.0513							1
PPT2	palmitoyl-protein thioesterase 2	6p21.3							0.919	3.13522	9	1
PPTC7	PTC7 protein phosphatase homolog (S. cerevisiae)	12q24.11				-0.302	0.00179	0.0233				1
PPY	pancreatic polypeptide	17q21	-0.31	0.00207	0.0196							1
PQLC1	PQ loop repeat containing 1	18q23	-0.709	0.00028	0.0047	-0.397	0.001623333	0.019166667				2
PQLC2	PQ loop repeat containing 2	1p36.13				-0.36	0.000435	0.0105				1
PON1	paraoxonase 1	7q21.3	0.516	0.02757	0.114							1
PRAM1	PML-RARA regulated adaptor molecule 1	19p13.2				-0.484	0.00015	0.0078				1
THOC2	THO complex 2	Xq25-q26.3	0.516	0.00111	0.0126				-0.975	3.4875	8	2
PRAMEF1	PRAME family member 1	1p36.21	-0.287	0.02042	0.0941							1
PRAMEF10	PRAME family member 10	1p36.21	-0.339	0.04533	0.1577	-0.462	0.00014	0.0076				2
PRAMEF12	PRAME family member 12	1p36.21	-0.254	0.04259	0.1516							1
PRAMEF13	PRAME family member 13	1p36.21				-0.445	0.00274	0.0254				1
PRAMEF14	In multiple Geneids					-0.443	0.00151	0.0177				1
PRAMEF15	PRAME family member 15	1p36.21	-0.448	0.03103	0.1235							1
PRAMEF20	PRAME family member 20	1p36.21	-0.389	0.00462	0.0346							1
PRAMEF22	PRAME family member 22	1p36.21				-0.322	0.00228	0.0262				1
PRAMEF3	PRAME family member 3	1p36.21				-0.322	0.00228	0.0262				1
PRAMEF7	PRAME family member 7	1p36.21							-0.659	2.572	5	1
PRAMEF8	PRAME family member 8	1p36.21				-0.3712	0.002064	0.01922				1
TMPO	thymopoietin	12q22	0.516	0.03705	0.1387							1
PRB1	proline-rich protein BstNI subfamily 1	12p13.2							-0.841	3.138	5	1
PRB2	In multiple Geneids								-0.841	3.138	5	1
PRB3	proline-rich protein BstNI subfamily 3	12p13.2	-0.466	0.00246	0.0222							1
PRB4	proline-rich protein BstNI subfamily 4	12p13.2							-0.841	3.286	5	1
TSC22D1	TSC22 domain family, member 1	13q14	0.516	0.00569	0.0399	0.351	0.02404	0.08085				2
PRCP	prolylcarboxypeptidase (angiotensinase C)	11q14	-0.821	0.00321	0.0269							1
PRDM1	In multiple Geneids		-2.069	0	1.00E-04							1
PRDM10	PR domain containing 10	11q25	-0.238	0.02963	0.1196	-0.362	0.00013	0.0074				2
PRDM15	PR domain containing 15	21q22.3				-0.395909091	0.000928182	0.013181818				1
PRDM16	PR domain containing 16	1p36.23-p33				-0.3672	0.001877	0.02159				1
PRDM2	In multiple Geneids		-0.697	9.00E-05	0.002	-0.326	0.00144	0.0209				2
PRDM4	PR domain containing 4	12q23-q24.1							-0.802	2.635	6	1
PRDM9	PR domain containing 9	5p14							-0.984	3.01667	6	1
PRDX2	peroxiredoxin 2	19p13.2	-0.373	0.0142	0.0738	-0.434	0.00014	0.0075				2
PRDX5	peroxiredoxin 5	11q13	-1.058	6.00E-05	0.0016							1
PRDX6	peroxiredoxin 6	1q25.1				0.395	0.00058	0.0138				1
ZFHX3	zinc finger homeobox 3	16q22.3	0.516	0.00295	0.0253							1
ZSCAN2	zinc finger and SCAN domain containing 2	15q25.2	0.516	0.00817	0.0509	2.275	0	0				2

ZC3H11A	zinc finger CCCH-type containing 11A	1q32.1	0.5155	0.00043	0.0061							1
PREX2	phosphatidylinositol-3,4,5-trisphosphate-dependent Rac exchange factor 2	8q13.2				0.347133333	0.003845333	0.028486667				1
PRG2	proteoglycan 2, bone marrow (natural killer cell activator, eosinophil granule r	11q12							-0.975	2.89143	7	1
PRG3	In multiple Geneids		-0.231	0.02062	0.0946							1
PRG-3	Data not found					0.339	0.000785	0.01575				1
PRG4	In multiple Geneids								-0.826	3.31143	7	1
PRH1	proline-rich protein HaeIII subfamily 1	12p13.2				0.377	0	0.0019	-0.877	3.04167	6	2
PRICKLE1	prickle homolog 1 (Drosophila)	12q12				-0.334333333	0.00307	0.024633333				1
PRICKLE2	prickle homolog 2 (Drosophila)	3p14.1							-0.953	2.898	5	1
PRICKLE3	prickle homolog 3 (Drosophila)	Xp11.23	-0.369	0.03377	0.1301							1
PRICKLE4	prickle homolog 4 (Drosophila)	6p21.31	-0.548	0.00465	0.0347							1
PLA2G15	phospholipase A2, group XV	16q22.1	0.515	0.00187	0.0182							1
PRIMA1	proline rich membrane anchor 1	14q32.12	-0.608	5.00E-05	0.0013	-0.388	0.000338333	0.00825				2
PRINS	psoriasis associated RNA induced by stress (non-protein coding)	10p12.1				-0.341	0.00188	0.0238				1
PLEKHH1	pleckstrin homology domain containing, family H (with MyTH4 domain) memb	14q24.1	0.515	0.04431	0.1554							1
PRKAA2	protein kinase, AMP-activated, alpha 2 catalytic subunit	1p31	-1.59	0.00123	0.0135							1
PRKAB1	protein kinase, AMP-activated, beta 1 non-catalytic subunit	12q24.1-q24.3							-0.718	2.482	5	1
RNF121	ring finger protein 121	11q13.4	0.515	0.00127	0.0139							1
PRKACA	protein kinase, cAMP-dependent, catalytic, alpha	19p13.1				-0.476	0.00403	0.0325				1
PRKAG1	protein kinase, AMP-activated, gamma 1 non-catalytic subunit	12q12-q14				-0.31	0.00131	0.02				1
PRKAG2	In multiple Geneids					-0.378	0.00621	0.040875				1
PRKAR2A	protein kinase, cAMP-dependent, regulatory, type II, alpha	3p21.3-p21.2	-0.487	0.02027	0.0936	-0.4278	0.001533	0.0187				2
ZNF808	zinc finger protein 808	19q13.41	0.514	0.03206	0.1259							1
PRKCB	protein kinase C, beta	16p11.2				-0.319	0.00194	0.0242				1
PRKCD	protein kinase C, delta	3p21.31				-0.433333333	9.33E-05	0.006				1
CRIP1	cysteine-rich protein 1 (intestinal)	14q32.33	0.513	0.00656	0.0439							1
PRKCE	protein kinase C, epsilon	2p21				0.3405	0.000645	0.013025				1
PRKCG	protein kinase C, gamma	19q13.4				-0.330666667	0.0032	0.027833333				1
PRKCH	protein kinase C, eta	14q23.1	-1.746	0	1.00E-04							1
PRKCQ	protein kinase C, theta	10p15				-0.314	0.00412	0.0353				1
FAM160A2	family with sequence similarity 160, member A2	11p15.4	0.513	0.00096	0.0114							1
PRKCZ	protein kinase C, zeta	1p36.33-p36.2				-0.374111111	0.003108889	0.024588889	0.067	2.568	5	2
PRKD2	protein kinase D2	19q13.3				-0.365	0.00278	0.024866667				1
PRKD3	protein kinase D3	2p21							-0.775	3.18	5	1
JAK3	Janus kinase 3	19p13.1	0.513	0.04056	0.1469							1
PRKG2	protein kinase, cGMP-dependent, type II	4q13.1-q21.1							-0.746	2.93444	9	1
PRKRIP1	PRKR interacting protein 1 (IL11 inducible)	7q22.1				-0.419	0.010705	0.0531				1
NPIPB3	Data not found		0.5126	0.00481	0.03518							1
PRKX	protein kinase, X-linked	Xp22.3	-0.643	0.0029	0.025	-0.363	0.002126667	0.022866667				2
PRKY	protein kinase, Y-linked, pseudogene	Yp11.2	-0.473	0.0307	0.1226	-0.4366	0.009277333	0.047213333				2
PRL	prolactin	6p22.2-p21.3							-0.856	2.68714	7	1
PRLR	prolactin receptor	5p13.2				0.39465	0.009881	0.052415				1
PRM1	protamine 1	16p13.2	-0.319	0.01925	0.0905							1
PRM2	protamine 2	16p13.2	-0.251	0.01374	0.0722							1
PRM3	In multiple Geneids		-0.331	0.00461	0.0346							1
PRMT1	protein arginine methyltransferase 1	19q13.3				-0.34	0.00201	0.0246				1
PRMT10	protein arginine methyltransferase 10 (putative)	4q31.23	-0.304	0.01153	0.0644	-0.41	0.00291	0.0296				2
PRMT2	protein arginine methyltransferase 2	21q22.3	-0.557	0.00043	0.0064							1
VKORC1L1	vitamin K epoxide reductase complex, subunit 1-like 1	7q11.21	0.5125	0.01692	0.0656							1
PRMT5	protein arginine methyltransferase 5	14q11.2				-0.3185	0.00485	0.0342				1
PRMT7	protein arginine methyltransferase 7	16q22.1				-0.3228	0.005788	0.03832				1
PRNT	In multiple Geneids								-0.845	2.43833	6	1
PRO1853	Data not found								-0.72	3.132	5	1
ARMCX3	armadillo repeat containing, X-linked 3	Xq21.33-q22.2	0.512	0.02207	0.0989				-0.967	2.58417	6	2
PROC	protein C (inactivator of coagulation factors Va and VIIIa)	2q13-q14							0.595	2.75125	8	1
CLCN5	chloride channel, voltage-sensitive 5	Xp11.23-p11.22	0.512	0.03497	0.1333	0.348666667	0.001228333	0.018416667				2
PRODH	proline dehydrogenase (oxidase) 1	22q11.21	-0.612	0.0225	0.1	-0.522	0	0.00165				2
PRODH2	proline dehydrogenase (oxidase) 2	19q13.1				-0.349	6.00E-05	0.0056				1
PROK1	prokineticin 1	1p21							-0.579	2.62	5	1
PROL1	proline rich, lacrimal 1	4q13.3							-0.957	2.71	6	1
GOLT1B	golgi transport 1B	12p12.1	0.512	0.02327	0.1022							1
PROM2	prominin 2	2q11.1	-1.989	0	0							1
PROP1	PROP paired-like homeobox 1	5q35.3	-0.24	0.00957	0.0567							1
PROSC	proline synthetase co-transcribed homolog (bacterial)	8p11.2				-0.306	0.00548	0.0412				1
IL23R	interleukin 23 receptor	1p31.3	0.512	0.00601	0.0414				-0.814	2.706	5	2
PRPF18	PRP18 pre-mRNA processing factor 18 homolog (S. cerevisiae)	10p13							-0.911	3.37571	7	1
NVL	nuclear VCP-like	1q41-q42.2	0.512	0.00579	0.0404							1
PCSK6	proprotein convertase subtilisin/kexin type 6	15q26.3	0.512	0.04241	0.1512	0.351	0.04028	0.1301				2
PRPF31	PRP31 pre-mRNA processing factor 31 homolog (S. cerevisiae)	19q13.42				-0.501	0.00152	0.0215				1

PRPF38A	PRP38 pre-mRNA processing factor 38 (yeast) domain containing A	1p32.3				-0.379	0.0019	0.0239				1
PRR26	Data not found		0.512	0.0047	0.0349							1
PRPF40A	PRP40 pre-mRNA processing factor 40 homolog A (S. cerevisiae)	2q23.3				0.311	3.00E-05	0.00385				1
PRPF40B	PRP40 pre-mRNA processing factor 40 homolog B (S. cerevisiae)	12q	-0.196	0.04615	0.1593							1
RNF148	ring finger protein 148	7q31.33	0.512	0.00332	0.0275				-0.812	2.93833	6	2
PRPF8	PRP8 pre-mRNA processing factor 8 homolog (S. cerevisiae)	17p13.3				-0.361	0.03163	0.1102				1
PRPH	peripherin	12q12-q13				-0.367	0.00041	0.0118				1
ZNF765	zinc finger protein 765	19q13.42	0.5115	0.01563	0.06755							1
PRPS2	phosphoribosyl pyrophosphate synthetase 2	Xp22.3-p22.2							-0.921	2.71333	6	1
PRPSAP1	phosphoribosyl pyrophosphate synthetase-associated protein 1	17q24-q25	-0.402	0.01969	0.0917							1
PRPSAP2	phosphoribosyl pyrophosphate synthetase-associated protein 2	17p11.2-p12				-0.349333333	0.00755	0.047333333				1
PRR10	Data not found								0.86	2.85214	7	1
DEDD2	death effector domain containing 2	19q13.2	0.511	0.00377	0.03							1
PRR12	proline rich 12	19q13.33				-0.327	0.03649	0.1224				1
KNOP1	Data not found		0.511	0.00358	0.0289							1
PRR14	proline rich 14	16p11.2							0.766	2.58	6	1
PRR14L	proline rich 14-like	22q12.2	-0.325	0.02357	0.103							1
STIP1	stress-induced-phosphoprotein 1	11q13	0.511	0.00046	0.0068							1
YWHAH	tyrosine 3-monooxygenase/tryptophan 5-monooxygenase activation protein,	22q12.3	0.511	0.00022	0.004							1
COPS8	COP9 constitutive photomorphogenic homolog subunit 8 (Arabidopsis)	2q37.3	0.51	0.00035	0.0055	0.3915	0.00324	0.02365				2
PRR23C	proline rich 23C	3q23	-0.308	0.00862	0.0527							1
FUOM	Data not found		0.51	0.01631	0.0809							1
PRR4	proline rich 4 (lacrimal)	12p13				0.332333333	0.000426667	0.0093	-0.902	3.40125	8	2
HJURP	Holliday junction recognition protein	2q37.1	0.51	0.04141	0.1489							1
HSPA5	heat shock 70kDa protein 5 (glucose-regulated protein, 78kDa)	9q33.3	0.51	0.00191	0.0185							1
PRR7	proline rich 7 (synaptic)	5q35.3				-0.489	0	0.0022				1
STK36	serine/threonine kinase 36	2q35	0.51	0.00429	0.0328							1
TTI1	TELO2 interacting protein 1	20q11.23	0.51	0.01349	0.0713							1
PRRG2	proline rich Gla (G-carboxyglutamic acid) 2	19q13.33							-0.729	2.954	5	1
PRRG4	proline rich Gla (G-carboxyglutamic acid) 4 (transmembrane)	11p13	-1.357	2.00E-05	7.00E-04							1
PRRT1	proline-rich transmembrane protein 1	6p21.32							0.94	2.948276667	7.666666667	1
PRRT2	proline-rich transmembrane protein 2	16p11.2				-0.312	0.04216	0.1339				1
PRRT3	proline-rich transmembrane protein 3	3p25.3	-0.385	0.00045	0.0066	-0.404	0.00181	0.0233				2
PRRX1	paired related homeobox 1	1q24				0.336666667	0.009746667	0.052733333				1
PRRX2	paired related homeobox 2	9q34.1				-0.377	0.00541	0.0409				1
PRSS12	protease, serine, 12 (neurotrypsin, motopsin)	4q28.1				-0.3385	0.006985	0.04215				1
CDC5L	CDC5 cell division cycle 5-like (S. pombe)	6p21	0.509	0.00357	0.0289	0.3276	0.01129	0.05516				2
PRSS27	In multiple Geneids		-3.65	0	0							1
PRSS3	protease, serine, 3	9p11.2				-0.348	0.00024	0.0094				1
PRSS7	Data not found								-0.99	2.992	5	1
FXR1	fragile X mental retardation, autosomal homolog 1	3q28	0.508	0.02177	0.0981							1
PRX	periaxin	19q13.2	-0.242	0.01469	0.0754	-0.353	0.0047925	0.033275				2
PRY	PTPN13-like, Y-linked	Yq11.223				-0.567230769	0.017090769	0.071946154				1
PRY2	In multiple Geneids					-0.567230769	0.017090769	0.071946154				1
PSAP	prosaposin	10q21-q22				-0.342	0.00336	0.0318				1
PSCA	prostate stem cell antigen	8q24.2	-1.842	0.00057	0.0079							1
PSD	pleckstrin and Sec7 domain containing	10q24				-0.39	0.00309	0.0282				1
PSD2	pleckstrin and Sec7 domain containing 2	5q31.2				-0.404833333	0.000248333	0.007466667				1
PSD3	pleckstrin and Sec7 domain containing 3	8p21.3							-0.558	2.714	5	1
PSEN1	presenilin 1	14q24.3				-0.389	0.00464	0.03755				1
PSENEN	presenilin enhancer 2 homolog (C. elegans)	19q13.12							0.545	2.29	5	1
PSG1	pregnancy specific beta-1-glycoprotein 1	19q13.2	-0.551	0.00388	0.0306							1
PSG10	In multiple Geneids					-0.329	6.00E-05	0.0054				1
PSG3	pregnancy specific beta-1-glycoprotein 3	19q13.2	-0.337	0.03956	0.1445							1
PSG4	pregnancy specific beta-1-glycoprotein 4	19q13.2	-0.475	0.01452	0.0748							1
PSG5	pregnancy specific beta-1-glycoprotein 5	19q13.2	-0.292	0.0153	0.0776							1
PSG7	pregnancy specific beta-1-glycoprotein 7 (gene/pseudogene)	19q13.2	-0.466	0.00287	0.0248	-0.388	0.00718	0.0477	0.717	2.598	5	3
PSIP1	In multiple Geneids					-0.362166667	0.002655	0.025666667				1
PSKH1	protein serine kinase H1	16q22.1	-0.376	3.00E-04	0.0049	-0.368	0.007055	0.04335				2
HSD17B7	hydroxysteroid (17-beta) dehydrogenase 7	1q23	0.508	0.02865	0.117							1
PSMA3	proteasome (prosome, macropain) subunit, alpha type, 3	14q23				-0.302	0.00217	0.0255				1
LOC100287290	cytokine receptor CRL2	3q25.32	0.508	0.00181	0.0179							1
P4HA2	prolyl 4-hydroxylase, alpha polypeptide II	5q31	0.508	0.00064	0.0086							1
SHC1	SHC (Src homology 2 domain containing) transforming protein 1	1q21	0.508	0.00115	0.0129							1
PSMAL	Data not found								-0.935	3.10571	7	1
PSMB1	proteasome (prosome, macropain) subunit, beta type, 1	6q27				-0.342	0.01951	0.084				1
SMURF2	SMAD specific E3 ubiquitin protein ligase 2	17q22-q23	0.508	0.02613	0.1104							1
TCF12	transcription factor 12	15q21	0.508	0.02069	0.0948							1
CENPL	centromere protein L	1q25.1	0.507	0.00373	0.0298							1

CEP192	centrosomal protein 192kDa	18p11.21	0.507	0.02212	0.099							1
LEPREL2	leprecan-like 2	12q13	0.507	0.00098	0.0115							1
STBD1	starch binding domain 1	4q21.1	0.507	0.00378	0.0301							1
PSMC2	proteasome (prosome, macropain) 26S subunit, ATPase, 2	7q22.1-q22.3							-0.641	2.67	7	1
IFT52	intraflagellar transport 52 homolog (Chlamydomonas)		0.506	0.0113	0.0636							1
PSMC6	proteasome (prosome, macropain) 26S subunit, ATPase, 6	14q22.1				-0.323	0.00045	0.0124				1
OTX1	orthodenticle homeobox 1	2p13	0.506	0.00713	0.0464							1
PSMD10	proteasome (prosome, macropain) 26S subunit, non-ATPase, 10	Xq22.3	-0.383	0.0384	0.1419							1
PSMD14	proteasome (prosome, macropain) 26S subunit, non-ATPase, 14	2q24.2				0.3025	0.000145	0.007	-0.642	2.434	5	2
ZNF611	zinc finger protein 611	19q13.41	0.506	0.00726	0.047							1
C3orf55	chromosome 3 open reading frame 55	3q25.32	0.505	0.02889	0.1176	0.405	0.00056	0.0136	-0.769	2.92	7	3
PSMD7	proteasome (prosome, macropain) 26S subunit, non-ATPase, 7	16q22.3				-0.317	0.00673	0.0461				1
PSMD9	proteasome (prosome, macropain) 26S subunit, non-ATPase, 9	12q24.31-q24.32				-0.336	0.002355	0.0254				1
PSME3	proteasome (prosome, macropain) activator subunit 3 (PA28 gamma; Ki)	17q21							-0.776	2.324	5	1
CNPY2	canopy 2 homolog (zebrafish)	12q15	0.505	0.00421	0.0324							1
PSORS1C2	psoriasis susceptibility 1 candidate 2	6p21.3	-0.279	0.00334	0.0276							1
LIN9	lin-9 homolog (C. elegans)	1q42.12	0.505	0.01657	0.0818							1
MAN2B1	mannosidase, alpha, class 2B, member 1	19cen-q13.1	0.505	0.01515	0.0771							1
PSTPIP2	In multiple Geneids					-0.383230769	0.016533846	0.070361538				1
PTAFR	platelet-activating factor receptor	1p35-p34.3				-0.417666667	0.005686667	0.03445	0.985	2.826	5	2
PTBP1	polypyrimidine tract binding protein 1	19p13.3				-0.573	0.002335	0.02375				1
PTBP2	polypyrimidine tract binding protein 2	1p21.3				0.308	2.00E-04	0.0087				1
PTCD1	pentatricopeptide repeat domain 1	7q22.1				-0.337	0.00554	0.0414				1
PTCD2	pentatricopeptide repeat domain 2	5q13.2	-0.262	0.0166	0.0818							1
POLR2K	polymerase (RNA) II (DNA directed) polypeptide K, 7.0kDa	8q22.2	0.505	0.00268	0.0236							1
PPAP2A	phosphatidic acid phosphatase type 2A	5q11	0.505	0.03609	0.1363							1
TBC1D2B	TBC1 domain family, member 2B	15q24.3-q25.1	0.505	0.00984	0.0511							1
PTCHD1	patched domain containing 1	Xp22.11				0.336333333	0.004866667	0.0373				1
PTCHD2	patched domain containing 2	1p36.22				-0.367142857	0.005077143	0.033228571				1
PTCRA	pre T-cell antigen receptor alpha	6p21.3	-0.166	0.04875	0.1645							1
CCDC113	coiled-coil domain containing 113	16q21	0.504	0.02734	0.1135							1
PTDSS2	phosphatidylserine synthase 2	11p15.5				-0.429	0.00086	0.0165				1
PTEN	phosphatase and tensin homolog	10q23.3	-0.3	0.01584	0.0794							1
PTENP1	phosphatase and tensin homolog pseudogene 1	9p21	-0.322	0.02456	0.1057							1
PTER	phosphotriesterase related	10p12				-0.347	0.002085	0.0241				1
RPGR	retinitis pigmentosa GTPase regulator	Xp21.1	0.504	0.0013	0.0141							1
PTGER3	prostaglandin E receptor 3 (subtype EP3)	1p31.2							0.759	2.948	5	1
PTGER4	prostaglandin E receptor 4 (subtype EP4)	5p13.1				0.439	0.00986	0.0568				1
ZC3H12B	zinc finger CCCH-type containing 12B	Xq12	0.504	0.03504	0.1334							1
PTGFRN	prostaglandin F2 receptor negative regulator	1p13.1				-0.3365	0.00112	0.01745				1
PTGIR	prostaglandin I2 (prostacyclin) receptor (IP)	19q13.3				-0.317	0.0231	0.0929				1
PTGIS	prostaglandin I2 (prostacyclin) synthase	20q13.13							0.794	2.86	6	1
PTGR1	prostaglandin reductase 1	9q31.3	-0.69	0.03806	0.1411							1
PTGR2	prostaglandin reductase 2	14q24.3				-0.413	0.00244	0.0271				1
PTGS1	prostaglandin-endoperoxide synthase 1 (prostaglandin G/H synthase and cycl	9q32-q33.3	-1.374	3.00E-05	8.00E-04							1
CPXM1	carboxypeptidase X (M14 family), member 1	20p13	0.503	0.00294	0.0252							1
PTH	parathyroid hormone	11p15.3-p15.1							-0.933	3.32143	7	1
PTH1R	parathyroid hormone 1 receptor	3p22-p21.1				-0.417	1.00E-05	0.0033				1
PTH2	parathyroid hormone 2	19q13.33	-0.234	0.03936	0.144							1
PTH2R	parathyroid hormone 2 receptor	2q33				0.3075	8.00E-05	0.00555				1
PTHLH	parathyroid hormone-like hormone	12p12.1-p11.2				0.3285	0.006535	0.04525	-0.84	2.685	6	2
GRAPL	GRB2-related adaptor protein-like	17p11.2	0.503	0.03695	0.1384							1
PTK2B	In multiple Geneids								-0.944	2.83833	6	1
PTK6	PTK6 protein tyrosine kinase 6	20q13.3	-1.763	1.00E-05	3.00E-04							1
PTK9	Data not found								-0.877	2.70125	8	1
HLA-G	major histocompatibility complex, class I, G	6p21.3	0.503	0.01654	0.0816							1
PTN	pleiotrophin	7q33							-0.881	3.138	5	1
PTOV1	prostate tumor overexpressed 1	19q13.33				-0.303	0.00941	0.0553				1
PTP4A1	protein tyrosine phosphatase type IVA, member 1	6q12							-0.762	2.88125	8	1
OAF	OAF homolog (Drosophila)	11q23.3	0.503	0.00049	0.007							1
AGPS	alkylglycerone phosphate synthase	2q31.2	0.502	0.00688	0.0454	0.3055	0.000265	0.00955				2
GMPPA	GDP-mannose pyrophosphorylase A	2q35	0.502	5.00E-05	0.0012				-0.64	2.4	5	2
PTPLA	protein tyrosine phosphatase-like (proline instead of catalytic arginine), meml	10p14-p13	-0.434	0.00152	0.0157							1
CPSF6	cleavage and polyadenylation specific factor 6, 68kDa	12q15	0.501	0.0227	0.1005							1
PTPMT1	protein tyrosine phosphatase, mitochondrial 1	11p11.2				-0.337	0.00639	0.0448				1
EMC10	ER membrane protein complex subunit 10	19q13.33	0.501	0.00011	0.0023							1
POP5	processing of precursor 5, ribonuclease P/MRP subunit (S. cerevisiae)	12q24.31	0.501	0.01062	0.061							1
PTPN13	protein tyrosine phosphatase, non-receptor type 13 (APO-1/CD95 (Fas)-associ	4q21.3	-3.134	0	0	-0.4066	0.001614	0.02126				2
PTPN14	protein tyrosine phosphatase, non-receptor type 14	1q32.2				0.346	0.01048	0.0587				1

SNORA65	small nucleolar RNA, H/ACA box 65	9q34	0.501	0.03661	0.1376							1
PTPN20A	In multiple Geneids								-0.885	3.38571	7	1
PTPN20B	In multiple Geneids								-0.869	3.07857	7	1
PTPN21	protein tyrosine phosphatase, non-receptor type 21	14q31.3	-0.449	0.00677	0.0449							1
PTPN22	protein tyrosine phosphatase, non-receptor type 22 (lymphoid)	1p13.2							-0.756	2.724	5	1
PTPN23	protein tyrosine phosphatase, non-receptor type 23	3p21.3				-0.4452	0.000676	0.01052				1
PTPN3	protein tyrosine phosphatase, non-receptor type 3	9q31	-0.711	0.00433	0.0331							1
PTPN5	In multiple Geneids					-0.351	0.02088	0.07715				1
STAU1	staufen, RNA binding protein, homolog 1 (Drosophila)	20q13.1	0.501	0.00037	0.0057							1
PTPN7	protein tyrosine phosphatase, non-receptor type 7	1q32.1							0.42	2.424	5	1
TMEM184B	transmembrane protein 184B	22q12	0.501	0.00237	0.0215							1
ZNF551	zinc finger protein 551	19q13.43	0.501	0.00325	0.0272							1
PTPRC	protein tyrosine phosphatase, receptor type, C	1q31-q32				0.365714286	0.001384286	0.0184	-0.816	3.28333	6	2
PTPRD	protein tyrosine phosphatase, receptor type, D	9p23-p24.3				-0.340015625	0.004799531	0.033334375				1
GNL3L	guanine nucleotide binding protein-like 3 (nucleolar)-like	Xp11.22	0.5	0.00146	0.0153							1
PTPRF	protein tyrosine phosphatase, receptor type, F	1p34				-0.3025	0.004435	0.03625				1
NCL	nucleolin	2q37.1	0.5	0.00247	0.0222							1
TMSB4X	thymosin beta 4, X-linked	Xq21.3-q22	0.5	0.04096	0.1479				-0.696	2.56667	6	2
TOE1	target of EGR1, member 1 (nuclear)	1p33	0.5	0.0099	0.058							1
FAM92A1	family with sequence similarity 92, member A1	8q22.1	0.499	0.01722	0.0839							1
PARP1	poly (ADP-ribose) polymerase 1	1q41-q42	0.499	0.01091	0.062							1
PTPRN2	protein tyrosine phosphatase, receptor type, N polypeptide 2	7q36				-0.372964286	0.009280714	0.049957143				1
PTPRO	protein tyrosine phosphatase, receptor type, O	12p13.3-p13.2				0.3356	0.00289	0.02544				1
PTPRQ	In multiple Geneids					0.357666667	0.011366667	0.054833333				1
SLC30A1	solute carrier family 30 (zinc transporter), member 1	1q32.3	0.499	0.00446	0.0337							1
PTPRS	protein tyrosine phosphatase, receptor type, S	19p13.3	-1.201	1.00E-05	3.00E-04	-0.466486486	0.003649459	0.0256				2
PTPRT	protein tyrosine phosphatase, receptor type, T	20q12-q13				0.345112903	0.004472742	0.03171129				1
ESF1	ESF1, nucleolar pre-rRNA processing protein, homolog (S. cerevisiae)	20p12.1	0.498	0.01065	0.0611							1
PTPRZ1	protein tyrosine phosphatase, receptor-type, Z polypeptide 1	7q31.3				0.391	0.01523	0.0728				1
PTRF	In multiple Geneids					-0.392	0.00202	0.0247				1
GNPAT	glyceronephosphate O-acyltransferase	1q42	0.498	0.00104	0.012							1
PTTG1	pituitary tumor-transforming 1	5q35.1							-0.856	3.03111	9	1
PTTG1IP	pituitary tumor-transforming 1 interacting protein	21q22.3				-0.3865	0.00104	0.0131				1
KIAA0947	KIAA0947	5p15.32	0.498	0.0061	0.0418	0.346	0.006831667	0.0354				2
PUM2	pumilio homolog 2 (Drosophila)	2p22-p21				0.307	0.01852	0.0815				1
PUS7	pseudouridylate synthase 7 homolog (S. cerevisiae)	7q22.3				-0.422	0.008083333	0.043433333				1
PVALB	parvalbumin	22q13.1				-0.3825	0.00155	0.0171				1
CPSF4L	cleavage and polyadenylation specific factor 4-like	17q25.1	0.497	0.00017	0.0033							1
PVRIG	poliovirus receptor related immunoglobulin domain containing	7q22.1				-0.442	0.01841	0.0812				1
PVRL1	poliovirus receptor-related 1 (herpesvirus entry mediator C)	11q23.3	-1.712	0	1.00E-04	-0.385	0.0017	0.0227				2
PVRL2	poliovirus receptor-related 2 (herpesvirus entry mediator B)	19q13.2				-0.459	0.000303333	0.0102				1
FAM120A	family with sequence similarity 120A	9q22.31	0.4965	0.00669	0.04095							1
PVRL4	poliovirus receptor-related 4	1q22-q23.2	-1.743	5.00E-05	0.0014							1
PVT1	Pvt1 oncogene (non-protein coding)	8q24				0.327	0.026353333	0.095666667				1
PWAR4	Data not found		-0.238	0.01364	0.0718							1
PWAR5	Data not found		-0.903	0.02745	0.1137							1
PWWP2A	PWWP domain containing 2A	5q33.3				-0.319	0.009303333	0.050466667				1
PWWP2B	PWWP domain containing 2B	10q26.3	-0.317	0.00156	0.016							1
C1QTNF6	C1q and tumor necrosis factor related protein 6	22q13.1	0.496	0.00079	0.0099							1
DDX47	DEAD (Asp-Glu-Ala-Asp) box polypeptide 47	12p13.1	0.496	0.00138	0.0147							1
PXK	PX domain containing serine/threonine kinase	3p14.3				-0.412	0.001945	0.019466667				1
PXMP2	peroxisomal membrane protein 2, 22kDa	12q24.33				-0.403	0.00027	0.0099				1
PXMP3	Data not found					0.358	0.00056	0.0121				1
PSMB3	proteasome (prosome, macropain) subunit, beta type, 3	17q12	0.496	0.01291	0.0695							1
AIMP2	aminoacyl tRNA synthetase complex-interacting multifunctional protein 2	7p22	0.495	0.01128	0.0635							1
KIF3C	kinesin family member 3C	2p23	0.495	0.00253	0.0226							1
MAGEF1	melanoma antigen family F, 1	3q13	0.495	0.01767	0.0854							1
PYGB	phosphorylase, glycogen; brain	20p11.2-p11.1				0.389	1.00E-05	0.0024				1
PYGL	phosphorylase, glycogen, liver	14q21-q22	-1.049	0.01761	0.0852							1
PYHIN1	pyrin and HIN domain family, member 1	1q23.1				0.3664	0.000472	0.01034				1
PYROXD1	pyridine nucleotide-disulphide oxidoreductase domain 1	12p12.1				0.323333333	0.001053333	0.017366667				1
PZP	pregnancy-zone protein	12p13-p12.2				0.31	0.00053	0.0132	-0.88	3.21143	7	2
QARS	glutaminyl-tRNA synthetase	3p21.31	-0.678	0.00112	0.0127	-0.336	0.003133333	0.0268				2
QDPR	quinoid dihydropteridine reductase	4p15.31				-0.373714286	0.001165714	0.017728571				1
QKI	QKI, KH domain containing, RNA binding	6q26				0.412	0.00396	0.0347				1
NAT1	N-acetyltransferase 1 (arylamine N-acetyltransferase)	8p22	0.495	0.01072	0.0613							1
PSMD1	proteasome (prosome, macropain) 26S subunit, non-ATPase, 1	2q37.1	0.495	0.00046	0.0068							1
QRICH1	glutamine-rich 1	3p21.31	-0.371	0.01608	0.0802	-0.3978	0.005597	0.03338				2
QRICH2	glutamine rich 2	17q25.1	-0.494	2.00E-04	0.0037							1

QRSL1	glutaminyl-tRNA synthase (glutamine-hydrolyzing)-like 1	6q21				-0.333	0.000625	0.0137				1
QSER1	glutamine and serine rich 1	11p13	-0.462	0.01928	0.0906							1
QSOX1	quiescin Q6 sulfhydryl oxidase 1	1q24				-0.47	0.00014	0.0076				1
PTRH2	peptidyl-tRNA hydrolase 2	17q23.1	0.495	0.01199	0.0662							1
QTRT1	queuine tRNA-ribosyltransferase 1	19p13.3	-0.252	0.01819	0.0871	-0.4455	0.01322	0.0523				2
RPS20P27	ribosomal protein S20 pseudogene 27	11q14.1	0.495	0.00075	0.0095							1
DHX36	DEAH (Asp-Glu-Ala-His) box polypeptide 36	3p13-q23	0.494	0.0023	0.0212							1
EMG1	EMG1 nucleolar protein homolog (S. cerevisiae)	12p13.3	0.494	0.01612	0.0803							1
R3HDM2	R3H domain containing 2	12q13.3				-0.3235	0.004265	0.03445				1
RAB10	RAB10, member RAS oncogene family	2p23.3	-1.186	8.00E-05	0.002							1
RAB11A	In multiple Geneids		-0.814	4.00E-05	0.0012	-0.582	0	0.0023				2
RAB11B	RAB11B, member RAS oncogene family	19p13.2	-0.234	0.03873	0.1426	-0.55825	0.0051225	0.028				2
RAB11FIP1	RAB11 family interacting protein 1 (class I)	8p11.22				-0.363	0.0175	0.06295	0.981	2.44333	6	2
RAB11FIP2	RAB11 family interacting protein 2 (class I)	10q26.11				0.307	0.00254	0.0276				1
RAB11FIP3	RAB11 family interacting protein 3 (class II)	16p13.3				-0.4335	0.016885	0.07271				1
RAB11FIP4	RAB11 family interacting protein 4 (class II)	17q11.2				0.312	0.00453	0.0372				1
FAM24B	family with sequence similarity 24, member B	10q26.13	0.494	0.0017	0.017							1
RAB14	RAB14, member RAS oncogene family	9q32-q34.11	-0.574	0.00364	0.0293							1
LIPA	lipase A, lysosomal acid, cholesterol esterase	10q23.2-q23.3	0.494	0.03387	0.1303							1
NONO	non-POU domain containing, octamer-binding	Xq13.1	0.494	0.00272	0.0238							1
RAB18	RAB18, member RAS oncogene family	10p12.1	-0.815	0.00051	0.0073							1
STIM2	stromal interaction molecule 2	4p15.2	0.494	0.00767	0.0488							1
ZNF592	zinc finger protein 592	15q25.3	0.494	0.00605	0.0415							1
RAB1B	RAB1B, member RAS oncogene family	11q12	-0.362	0.0015	0.0156							1
RAB1C	In multiple Geneids		-0.476	0.03889	0.1429							1
SRSF10	serine/arginine-rich splicing factor 10	1p36.11	0.4935	0.00128	0.01395							1
RAB22A	RAB22A, member RAS oncogene family	20q13.32				0.3125	0.023435	0.0841				1
RAB24	RAB24, member RAS oncogene family	5q35.3	-0.483	0.00023	0.004							1
RAB25	RAB25, member RAS oncogene family	1q22	-1.491	2.00E-05	6.00E-04							1
RAB26	RAB26, member RAS oncogene family	16p13.3				-0.399	0.00158	0.0219				1
RAB27A	RAB27A, member RAS oncogene family	15q15-q21.1				-0.342333333	0.009803333	0.0536				1
RAB27B	RAB27B, member RAS oncogene family	18q21.2	-1.574	0.00258	0.0229							1
RAB28	RAB28, member RAS oncogene family	4p15.33				-0.393	0.00095	0.0172				1
RAB2A	RAB2A, member RAS oncogene family	8q12.1	-0.5	0.00282	0.0244							1
RAB2B	RAB2B, member RAS oncogene family	14q11.2				-0.331	0.00605	0.0434				1
RAB31	RAB31, member RAS oncogene family	18p11.3				-0.321	0.00722	0.0479				1
RAB33B	RAB33B, member RAS oncogene family	4q28				-0.315	0.02202	0.0902				1
RAB35	RAB35, member RAS oncogene family	12q24.31				-0.314	0.00021	0.0088				1
RAB36	RAB36, member RAS oncogene family	22q11.22				-0.410666667	0.000638333	0.0098				1
RAB37	RAB37, member RAS oncogene family	17q25.1				-0.308	0.00324	0.0312				1
RAB38	RAB38, member RAS oncogene family	11q14	-2.359	0	2.00E-04							1
RAB39B	RAB39B, member RAS oncogene family	Xq28				0.303	0.00599	0.0432				1
RAB3A	RAB3A, member RAS oncogene family	19p13.2				-0.357	0.00669	0.0459				1
ADAM10	ADAM metallopeptidase domain 10	15q22	0.493	0.03885	0.1428							1
RAB3C	RAB3C, member RAS oncogene family	5q13							-0.858	2.75	5	1
RAB3D	RAB3D, member RAS oncogene family	19p13.2	-1.128	0	1.00E-04	-0.47125	0.00757	0.04535				2
RAB3GAP1	RAB3 GTPase activating protein subunit 1 (catalytic)	2q21.3				0.404	0.00014	0.0076				1
COL6A2	collagen, type VI, alpha 2	21q22.3	0.493	0.03245	0.1268							1
HAUS2	HAUS augmin-like complex, subunit 2	15q15.2	0.493	0.00417	0.0322							1
RAB40A	RAB40A, member RAS oncogene family	Xq22.1							-1.053	3.73	8	1
RAB40B	RAB40B, member RAS oncogene family	17q25.3				-0.396	0.00874	0.0532				1
RAB40C	RAB40C, member RAS oncogene family	16p13.3				-0.45325	0.0193375	0.08035				1
SLC25A36	solute carrier family 25 (pyrimidine nucleotide carrier), member 36	3q23	0.493	0.02819	0.1158							1
RAB5A	RAB5A, member RAS oncogene family	3p24-p22	-0.593	0.00101	0.0118	-0.314	0.01148	0.0618				2
RAB5B	RAB5B, member RAS oncogene family	12q13	-0.77	8.00E-05	0.0018							1
CACNB1	calcium channel, voltage-dependent, beta 1 subunit	17q21-q22	0.492	0.02842	0.1164							1
RAB6A	RAB6A, member RAS oncogene family	11q13.3	-0.529	0.01226	0.0672	-0.323	0.00243	0.027				2
RAB6B	RAB6B, member RAS oncogene family	3q22.1	-0.271	0.04345	0.1533							1
RAB7B	RAB7B, member RAS oncogene family	1q32	-0.651	0	0							1
DENND4A	DENN/MADD domain containing 4A	15q22.31	0.492	0.00137	0.0146							1
RAB8A	RAB8A, member RAS oncogene family	19p13.1				-0.4164	0.002854	0.02634				1
DPY19L2P2	In multiple Geneids		0.492	0.04154	0.1492							1
RAB9A	RAB9A, member RAS oncogene family	Xp22.2	-0.557	0.00105	0.0121							1
RABAC1	Rab acceptor 1 (prenylated)	19q13.2				-0.358	3.00E-04	0.0104				1
RABEP1	rabaptin, RAB GTPase binding effector protein 1	17p13.2				-0.3515	0.0047025	0.0316				1
RABEP2	rabaptin, RAB GTPase binding effector protein 2	16p11.2				-0.371333333	0.00296	0.028266667				1
RABGAP1L	RAB GTPase activating protein 1-like	1q24				0.3782	0.010752	0.04324	-0.851	2.84222	9	2
RABGEF1	RAB guanine nucleotide exchange factor (GEF) 1	7q11.21	-0.48	0.00169	0.017							1
RABGGTA	Rab geranylgeranyltransferase, alpha subunit	14q11.2	-1.574	0	0	-0.378	0.00043	0.012				2

RABIF	RAB interacting factor	1q32.1	-0.39	0.00311	0.0263							1
FEZF1	FEZ family zinc finger 1	7q31.32	0.492	0.035	0.1333							1
RABL2B	RAB, member of RAS oncogene family-like 2B	22q13.33				-0.31	0.00016	0.0078				1
RABL3	In multiple Geneids		-0.459	0.03466	0.1324							1
RABL4	Data not found								-0.732	2.57	7	1
RAC1	ras-related C3 botulinum toxin substrate 1 (rho family, small GTP binding prot	7p22	-0.781	0.04117	0.1484							1
RAC2	ras-related C3 botulinum toxin substrate 2 (rho family, small GTP binding prot	22q13.1				-0.41075	0.0019025	0.019275				1
RACGAP1	In multiple Geneids					-0.362	0.00012	0.00545				1
KIF21B	kinesin family member 21B	1q32.1	0.492	0.00465	0.0347							1
RAD17	RAD17 homolog (S. pombe)	5q13							-0.8252	3.016366	6.8	1
RAD18	RAD18 homolog (S. cerevisiae)	3p25-p24							-0.575	2.68143	7	1
UHMK1	U2AF homology motif (UHM) kinase 1	1q23.3	0.492	0.00015	0.003							1
RAD23A	RAD23 homolog A (S. cerevisiae)	19p13.2				-0.387	0.01066	0.0575				1
RAD50	RAD50 homolog (S. cerevisiae)	5q31				-0.31	7.00E-05	0.0058				1
ZC3H12C	zinc finger CCCH-type containing 12C	11q22.3	0.492	0.03337	0.1292							1
RAD51C	RAD51 homolog C (S. cerevisiae)	17q25.1				-0.318	0.00264	0.0281				1
RAD51L1	Data not found					0.507	2.50E-05	0.0039				1
CREB3L4	cAMP responsive element binding protein 3-like 4	1q21.3	0.491	0.03172	0.1251							1
RAD54B	RAD54 homolog B (S. cerevisiae)	8q22.1				0.516	0	0.0017				1
RAD54L2	RAD54-like 2 (S. cerevisiae)	3p21.2				-0.398615385	0.001313077	0.017215385				1
RAD9B	In multiple Geneids					-0.327	0.00041	0.0119				1
PCSK9	In multiple Geneids		0.491	0.02752	0.1138							1
RAET1E	retinoic acid early transcript 1E	6q25.1	-3.512	0	0							1
RAET1G	retinoic acid early transcript 1G	6q24.1-q25.1	-0.973	0	3.00E-04							1
RAET1L	retinoic acid early transcript 1L	6q25.1	-2.699	0	0							1
RAF1	v-raf-1 murine leukemia viral oncogene homolog 1	3p25	-0.547	0.00217	0.0202	-0.3315	0.005925	0.04205				2
RAG2	recombination activating gene 2	11p13							-0.785	2.85	5	1
RAGE	Data not found					-0.390333333	0.000323333	0.009566667				1
SCHIP1	schwannomin interacting protein 1	3q25.32-q25.33	0.491	0.01803	0.0866	0.351111111	0.003211111	0.025566667	-1.03	3.52167	6	3
RALA	v-ral simian leukemia viral oncogene homolog A (ras related)	7p15-p13	-0.799	0.00765	0.0487							1
RALBP1	ralA binding protein 1	18p11.3	-0.473	0.02455	0.1057							1
C9orf37	chromosome 9 open reading frame 37	9q34.3	0.49	0.01396	0.073							1
RALGDS	ral guanine nucleotide dissociation stimulator	9q34.3				-0.32	8.00E-04	0.0159				1
RALGPS2	Ral GEF with PH domain and SH3 binding motif 2	1q25.2	-0.528	0.02897	0.1178							1
CCDC104	coiled-coil domain containing 104	2p16.1	0.49	0.03312	0.1284							1
RALYL	RALY RNA binding protein-like	8q21.2				0.343466667	0.000176	0.006216667				1
HMG20A	high mobility group 20A	15q24	0.49	0.00292	0.0251							1
RANBP10	RAN binding protein 10	16q22.1	-0.483	0.0043	0.0329	-0.394	0.004256316	0.032294737				2
COA4	Data not found		0.489	0.00164	0.0166							1
HSP90AB2P	heat shock protein 90kDa alpha (cytosolic), class B member 2, pseudogene	4p15.33	0.489	0.00834	0.0516							1
RANBP3	RAN binding protein 3	19p13.3				-0.419222222	0.003121111	0.02427037				1
RANBP3L	RAN binding protein 3-like	5p13.2				0.447777778	0.003024444	0.029533333				1
RANBP6	RAN binding protein 6	9p24.1				-0.334	0.00117	0.0189				1
RANBP9	RAN binding protein 9	6p23	-1.467	0	2.00E-04							1
RANGAP1	Ran GTPase activating protein 1	22q13				-0.467583333	0.0010325	0.013858333				1
RANGRF	RAN guanine nucleotide release factor	17p13.1				-0.48	0.0014	0.0206				1
RAP1A	RAP1A, member of RAS oncogene family	1p13.3	-0.644	0.00175	0.0174							1
SPRY1	sprouty homolog 1, antagonist of FGF signaling (Drosophila)	4q28.1	0.489	0.01749	0.0848				-0.9425	2.960355	7	2
RAP1GAP	RAP1 GTPase activating protein	1p36.1-p35				-0.365857143	0.005281429	0.0335				1
RAP1GDS1	RAP1, GTP-GDP dissociation stimulator 1	4q23-q25	-0.854	0.00052	0.0074	-0.3274	0.001584	0.02092				2
RAP2A	RAP2A, member of RAS oncogene family	13q34				0.334	0.013706667	0.064066667				1
RAP2B	RAP2B, member of RAS oncogene family	3q25.2	-0.697	0.00148	0.0154							1
ZNF22	zinc finger protein 22 (KOX 15)	10q11	0.489	0.01895	0.0896							1
AP3S2	adaptor-related protein complex 3, sigma 2 subunit	15q26.1	0.488	0.02604	0.1102	0.3405	0.002445	0.02645				2
RAPGEF3	Rap guanine nucleotide exchange factor (GEF) 3	12q13.1	-0.531	0.00292	0.0208							1
RAPGEF4	Rap guanine nucleotide exchange factor (GEF) 4	2q31-q32				0.322333333	0.00014	0.006983333				1
RAPGEF5	Rap guanine nucleotide exchange factor (GEF) 5	7p15.3				0.321333333	0.004481667	0.03245				1
RAPGEF6	Rap guanine nucleotide exchange factor (GEF) 6	5q31.1				-0.3188	0.002726	0.0273				1
RAPGEFL1	Rap guanine nucleotide exchange factor (GEF)-like 1	17q21.1	-1.605	0.00099	0.0116	-0.3604	0.006038	0.04172				2
RAPSN	receptor-associated protein of the synapse	11p11.2				-0.307	0.01289	0.066				1
CLUHP3	Data not found		0.488	0.00435	0.0331							1
RARG	retinoic acid receptor, gamma	12q13	-1.933	5.00E-05	0.0012	-0.343	0.00207	0.02445				2
CNTNAP1	contactin associated protein 1	17q21	0.488	0.02119	0.0964							1
EIF2AK1	eukaryotic translation initiation factor 2-alpha kinase 1	7p22	0.488	0.01437	0.0744							1
PKIG	protein kinase (cAMP-dependent, catalytic) inhibitor gamma	20q12-q13.1	0.488	0.02232	0.0995							1
RARS	arginyl-tRNA synthetase	5q35.1				-0.323	0.00034	0.011				1
SUPT7L	suppressor of Ty 7 (S. cerevisiae)-like	2p23.3	0.488	0.0059	0.0409				-0.646	2.59333	6	2
PDXDC2P	pyridoxal-dependent decarboxylase domain containing 2, pseudogene	16q22.1	0.4875	0.00968	0.057							1
RASA4	RAS p21 protein activator 4	7q22	-0.374	0.00621	0.0423	-0.496166667	0.001816667	0.01915				2

RASA4P	RAS p21 protein activator 4 pseudogene	7p13				-0.578	0.00016	0.006933333				1
RASAL1	RAS protein activator like 1 (GAP1 like)	12q23-q24	-0.996	9.00E-05	0.0021							1
RASAL2	RAS protein activator like 2	1q24				0.3155	0.0004575	0.01195				1
RASAL3	RAS protein activator like 3	19p13.12				-0.306	0.0022	0.0257				1
ATF2	activating transcription factor 2	2q32	0.487	0.00261	0.0231							1
AUNIP	Data not found		0.486	0.0189	0.0895							1
RASGEF1B	RasGEF domain family, member 1B	4q21.21	-0.981	0.00264	0.0233	-0.349	0.00049	0.0128				2
RASGEF1C	RasGEF domain family, member 1C	5q35.3				-0.382	0.000898182	0.014381818				1
ERH	enhancer of rudimentary homolog (Drosophila)	14q24.1	0.486	0.00328	0.0273							1
RASGRP1	RAS guanyl releasing protein 1 (calcium and DAG-regulated)	15q14	-1.912	0	2.00E-04							1
HIST2H2BE	histone cluster 2, H2be	1q21.2	0.486	0.013245	0.06975							1
RASGRP4	RAS guanyl releasing protein 4	19q13.1				-0.315666667	0.003553333	0.031466667				1
RASIP1	Ras interacting protein 1	19q13.33				-0.3495	0.004885	0.03875				1
RASSF1	Ras association (RalGDS/AF-6) domain family member 1	3p21.3				-0.313	0.00032	0.0107				1
RASSF10	Ras association (RalGDS/AF-6) domain family (N-terminal) member 10	11p15.2	-0.587	0.00342	0.0281							1
RASSF3	In multiple Geneids					-0.322	0.003795	0.03195				1
METTL21A	methyltransferase like 21A	2q33.3	0.486	0.00044	0.0065							1
RASSF5	Ras association (RalGDS/AF-6) domain family member 5	1q32.1				0.351	0.00103	0.0179				1
PBXIP1	pre-B-cell leukemia homeobox interacting protein 1	1q21.3	0.486	0.01897	0.0897							1
PTPN12	protein tyrosine phosphatase, non-receptor type 12	7q11.23	0.486	0.02129	0.0968							1
RASSF9	Ras association (RalGDS/AF-6) domain family (N-terminal) member 9	12q21.31	-0.831	0.00152	0.0157							1
RAVER1	In multiple Geneids					-0.43	0.0012525	0.018325				1
RAX	retina and anterior neural fold homeobox	18q21.32	-0.383	0.00089	0.0108	-0.404	0.000955	0.0151				2
RAX2	retina and anterior neural fold homeobox 2	19p13.3	-0.259	0.01302	0.0698	-0.6	0.00044	0.0123				2
RAXLX	Data not found								-0.866	3.32	5	1
RBAKDN	Data not found		-0.231	0.02473	0.1063							1
AKAP11	A kinase (PRKA) anchor protein 11	13q14.11	0.485	0.00059	0.0081	0.3075	0.008575	0.05125				2
AKAP13	A kinase (PRKA) anchor protein 13	15q24-q25	0.485	0.01274	0.0688	0.356	2.00E-04	0.0087				2
RBBP8	retinoblastoma binding protein 8	18q11.2				0.31	0.01284	0.0659				1
RBBP8NL	Data not found		-0.237	0.04024	0.1462							1
RBFA	ribosome binding factor A (putative)	18q23	-0.201	0.01896	0.0896							1
RBFOX2	RNA binding protein, fox-1 homolog (C. elegans) 2	22q13.1	-0.566	0.00202	0.0192							1
RBFOX3	RNA binding protein, fox-1 homolog (C. elegans) 3	17q25.3	-0.457	0.04502	0.1569							1
CCDC142	coiled-coil domain containing 142	2p13.1	0.485	0.0159	0.0797							1
RBM11	RNA binding motif protein 11	21q11	-0.876	0.00016	0.0031							1
GPATCH4	G patch domain containing 4	1q22	0.485	0.00474	0.0352							1
RBM14	RNA binding motif protein 14	11q13.2				-0.371	0.00052	0.0131				1
OTUD3	OTU domain containing 3	1p36.13	0.485	0.00848	0.0521							1
RBM15B	RNA binding motif protein 15B	3p21.2				-0.3755	0.00022	0.00865				1
RBM16	Data not found					-0.357	0.00057	0.01335				1
RBM17	RNA binding motif protein 17	10p15.1							-0.98	3.352	5	1
TSPAN4	tetraspanin 4	11p15.5	0.485	0.00664	0.0442							1
GTF2H2D	general transcription factor IIH, polypeptide 2D	5q13.2	0.484	0.04443	0.1556							1
RBM22	RNA binding motif protein 22	5q33.1							-0.954	3.04	7	1
RBM23	RNA binding motif protein 23	14q11.2				-0.3495	0.00204	0.02435				1
RBM25	RNA binding motif protein 25	14q24.3				-0.431	0.006053333	0.038533333				1
RBM26	RNA binding motif protein 26	13q31.1				0.378	0.010535	0.0586				1
RBM27	RNA binding motif protein 27	5q32				-0.3744	0.000854	0.01558				1
HUS1	HUS1 checkpoint homolog (S. pombe)	7p13-p12	0.484	0.02238	0.0996							1
SURF4	surfeit 4	9q34.2	0.484	0.0109	0.0619							1
TPP2	tripeptidyl peptidase II	13q32-q33	0.484	0.00595	0.0411							1
BRCA1	breast cancer 1, early onset	17q21	0.483	0.02984	0.1202							1
CAMK1	calcium/calmodulin-dependent protein kinase I	3p25.3	0.483	0.0014	0.0148							1
RBM42	RNA binding motif protein 42	19q13.12				-0.323	0.00495	0.039				1
CPSF1	cleavage and polyadenylation specific factor 1, 160kDa	8q24.23	0.483	0.00527	0.0378							1
RBM47	RNA binding motif protein 47	4p14	-0.521	0.00132	0.0143	-0.3988125	0.001949063	0.02005625				2
RBM4B	RNA binding motif protein 4B	11q13				-0.354	0.00107	0.01815				1
RBM5	RNA binding motif protein 5	3p21.3				-0.4335	0.00444	0.0283				1
RBM6	RNA binding motif protein 6	3p21.3				-0.410684211	0.002351053	0.020584211				1
RBM8A	RNA binding motif protein 8A	1q21.1	-0.358	0.03828	0.1417							1
RBM9	Data not found								-1.061	2.52833	6	1
RBMS1	RNA binding motif, single stranded interacting protein 1	2q24.2				0.42	0.00019	0.00735				1
RBMS2	RNA binding motif, single stranded interacting protein 2	12q13.3				-0.3315	0.00897	0.05055				1
RBMS3	RNA binding motif, single stranded interacting protein 3	3p24-p23				0.4055	0.004045	0.0337				1
RBMY1A1	RNA binding motif protein, Y-linked, family 1, member A1	Yq11.223				-0.509363636	0.029803636	0.105445455				1
RBMY1A3P	In multiple Geneids		-0.677	0.00098	0.0115	-0.523	0.01798	0.0801				2
RBMY1B	RNA binding motif protein, Y-linked, family 1, member B	Yq11.223				-0.518733333	0.029958	0.1052				1
RBMY1D	RNA binding motif protein, Y-linked, family 1, member D	Yq11.223				-0.518733333	0.029958	0.1052				1
RBMY1E	RNA binding motif protein, Y-linked, family 1, member E	Yq11.223				-0.535	0.029089	0.10347				1

RBMY1F	RNA binding motif protein, Y-linked, family 1, member F	Yq11.223				-0.526	0.025528333	0.095358333				1
RBMY1J	RNA binding motif protein, Y-linked, family 1, member J	Yq11.223				-0.499428571	0.030548571	0.108757143				1
RBP2	retinol binding protein 2, cellular	3q23							-0.84	2.474	5	1
EBLN2	endogenous Bornavirus-like nucleoprotein 2	3p13	0.483	0.04449	0.1557							1
RBP7	retinol binding protein 7, cellular	1p36.22	-1.434	0	1.00E-04							1
RBPJ	recombination signal binding protein for immunoglobulin kappa J region	4p15.2	-0.259	0.03997	0.1455	-0.378	0.001231429	0.017257143				2
RBPMS2	RNA binding protein with multiple splicing 2	15q22.31				-0.352285714	0.006318571	0.039971429				1
RBX1	ring-box 1, E3 ubiquitin protein ligase	22q13.2	-0.551	0.00176	0.0175	-0.382333333	0.003776667	0.028733333				2
RCAN1	regulator of calcineurin 1	21q22.12				-0.401428571	0.00185	0.017528571				1
RCAN2	regulator of calcineurin 2	6p12.3				0.379666667	0.00094	0.0166				1
RCAN3	RCAN family member 3	1p35.3-p33	-0.919	3.00E-05	8.00E-04	-0.463	0.00103	0.0179				2
MARCH6	membrane-associated ring finger (C3HC4) 6, E3 ubiquitin protein ligase	5p15.2	0.483	0.01591	0.0797	0.3212	0.006838	0.0431				2
RCBTB2	regulator of chromosome condensation (RCC1) and BTB (POZ) domain contair	13q14.3	-0.812	0.00019	0.0035							1
RCC1	regulator of chromosome condensation 1	1p36.1				-0.4608	0.01532	0.06548				1
RCC2	regulator of chromosome condensation 2	1p36.13				-0.402	0.00217	0.020442857				1
RCHY1	ring finger and CHY zinc finger domain containing 1, E3 ubiquitin protein ligase	4q21.1				-0.41	0.00217	0.0255				1
RCL1	RNA terminal phosphate cyclase-like 1	9p24.1-p23				-0.3658	0.002204	0.02014				1
ODF2	outer dense fiber of sperm tails 2	9q34.11	0.483	0.0047	0.0349							1
SLC16A5	solute carrier family 16, member 5 (monocarboxylic acid transporter 6)	17q25.1	0.483	0.00301	0.0257							1
SSR3	signal sequence receptor, gamma (translocon-associated protein gamma)	3q25.31	0.483	0.01068	0.0613	0.328	0.00605	0.04225				2
RCOR1	REST corepressor 1	14q32.31	-0.636	0.00983	0.0577	-0.322333333	0.007916667	0.0466				2
RCSD1	RCSD domain containing 1	1q24.2							0.632	2.56	5	1
RCVRN	recoverin	17p13.1				-0.308	0.00324	0.0312				1
TARBP1	TAR (HIV-1) RNA binding protein 1	1q42.3	0.483	0.02436	0.1052							1
RDH11	retinol dehydrogenase 11 (all-trans/9-cis/11-cis)	14q24.1				-0.349	2.00E-04	0.0088				1
RDH12	retinol dehydrogenase 12 (all-trans/9-cis/11-cis)	14q24.1	-2.331	0	0							1
RDH13	retinol dehydrogenase 13 (all-trans/9-cis)	19q13.42	-0.54	0.00346	0.0283							1
UFM1	ubiquitin-fold modifier 1	13q13.3	0.483	0.01249	0.068	0.331	0.00317	0.0309	-0.742	2.94	5	3
RDH8	retinol dehydrogenase 8 (all-trans)	19p13.2	-0.25	0.01362	0.0718	-0.544	0.00255	0.0277				2
RDHE2	Data not found								0.754	3.025	6	1
RDX	radixin	11q23	-0.662	0.04302	0.1525							1
REC8	REC8 homolog (yeast)	14q11.2-q12				-0.3615	0.003405	0.03065				1
RECK	reversion-inducing-cysteine-rich protein with kazal motifs	9p13.3				-0.363	0.00305125	0.0249375				1
ERCC2	excision repair cross-complementing rodent repair deficiency, complementati	19q13.3	0.482	0.00178	0.0176							1
FAM49A	family with sequence similarity 49, member A	2p24.2	0.482	0.04188	0.15	0.3935	0.0007525	0.01355				2
REEP4	receptor accessory protein 4	8p21.3	-1.131	5.00E-05	0.0013							1
REEP5	receptor accessory protein 5	5q22-q23				-0.393	0.00013	0.0069				1
REEP6	receptor accessory protein 6	19p13.3				-0.404	0.00054	0.0134				1
HMGB3	high mobility group box 3	Xq28	0.482	0.03288	0.1279							1
REG1B	regenerating islet-derived 1 beta	2p12							-1.013	3.03	5	1
KLHL42	Data not found		0.482	0.00149	0.0154							1
RELA	v-rel reticuloendotheliosis viral oncogene homolog A (avian)	11q13				-0.315333333	0.00823	0.0503				1
POC5	POC5 centriolar protein homolog (Chlamydomonas)	5q13.3	0.482	0.00032	0.0051							1
RELN	reelin	7q22	-0.565	0.00957	0.0567							1
REM2	In multiple Geneids					-0.438	0.00269	0.0284				1
PTPN6	protein tyrosine phosphatase, non-receptor type 6	12p13	0.482	0.00287	0.0248							1
REPS1	RALBP1 associated Eps domain containing 1	6q24.1	-0.66	0.00252	0.0225	-0.338	0.004345	0.03555				2
SLC45A3	solute carrier family 45, member 3	1q32.1	0.482	0.00695	0.0456							1
RER1	RER1 retention in endoplasmic reticulum 1 homolog (S. cerevisiae)	1p36	-0.287	0.04802	0.1631	-0.3675	0.00045	0.0117				2
RERE	arginine-glutamic acid dipeptide (RE) repeats	1p36.23				-0.355933333	0.001986667	0.022953333				1
RERG	RAS-like, estrogen-regulated, growth inhibitor	12p12.3				0.35	0.00819	0.0513				1
RERGL	RERG/RAS-like	12p12.3	-0.457	0.01792	0.0862							1
RESP18	regulated endocrine-specific protein 18 homolog (rat)	2q35	-0.282	0.00038	0.0059							1
REST	RE1-silencing transcription factor	4q12				-0.4305	0.004185	0.03265				1
STK10	serine/threonine kinase 10	5q35.1	0.482	0.01627	0.0808							1
WISP1	WNT1 inducible signaling pathway protein 1	8q24.22	0.482	0.01695	0.0831	0.3405	0.0282925	0.102625				2
REXO1	REX1, RNA exonuclease 1 homolog (S. cerevisiae)	19p13.3				-0.457	0.001303333	0.019733333				1
REXO1L1	REX1, RNA exonuclease 1 homolog (S. cerevisiae)-like 1	8q21.2							0.9836	4.192888	9	1
REXO1L2P	REX1, RNA exonuclease 1 homolog (S. cerevisiae)-like 2 (pseudogene)	8q21.2							0.993	4.31556	9	1
REXO1L3P	REX1, RNA exonuclease 1 homolog (S. cerevisiae)-like 3, pseudogene	8q21.2							1.058	4.45062	8	1
REXO1L5P	REX1, RNA exonuclease 1 homolog (S. cerevisiae)-like 5, pseudogene	8q21.2							1.043	4.40444	9	1
REXO1L6P	REX1, RNA exonuclease 1 homolog (S. cerevisiae)-like 6 (pseudogene)	8q21.2							0.936	4.23444	9	1
REXO1L7P	REX1, RNA exonuclease 1 homolog (S. cerevisiae)-like 7, pseudogene	8q21.2							0.979	4.02444	9	1
REXO2	REX2, RNA exonuclease 2 homolog (S. cerevisiae)	11q23.2				0.304	0.00377	0.0338				1
RFC1	replication factor C (activator 1) 1, 145kDa	4p14-p13				-0.34275	0.0100925	0.0566				1
RFC2	replication factor C (activator 1) 2, 40kDa	7q11.23				-0.3665	0.00673	0.04495				1
ZNF84	zinc finger protein 84	12q24.33	0.482	0.00141	0.0149							1
RFC4	replication factor C (activator 1) 4, 37kDa	3q27							-0.754	2.502	5	1
RFC5	replication factor C (activator 1) 5, 36.5kDa	12q24.23				-0.322	0.00022	0.00855				1

RFESD	Rieske (Fe-S) domain containing	5q15	-0.382	0.00989	0.058							1
RFFL	ring finger and FYVE-like domain containing E3 ubiquitin protein ligase	17q12				-0.322	0.00253	0.0276				1
RFPL1	ret finger protein-like 1	22q12.2				-0.389	0.00245	0.0272				1
RFPL1S	Data not found					-0.389	0.00245	0.0272				1
RFPL2	ret finger protein-like 2	22q12.3				-0.38	0.00057	0.0118				1
RFPL3S	Data not found					-0.35	0.00014	0.0076				1
RFT1	RFT1 homolog (S. cerevisiae)	3p21.1	-0.356	0.00732	0.0473	-0.4152	0.00035	0.0096				2
ACTN1	actinin, alpha 1	14q24	0.481	0.04573	0.1585							1
ALG3	asparagine-linked glycosylation 3, alpha-1,3- mannosyltransferase homolog (S	3q27.1	0.481	0.0306	0.1224				-0.725	2.63	5	2
C1orf131	chromosome 1 open reading frame 131	1q42.2	0.481	0.00349	0.0284							1
RFWD3	ring finger and WD repeat domain 3	16q23.1				-0.371	0.00615	0.042733333				1
RFX1	regulatory factor X, 1 (influences HLA class II expression)	19p13.1				-0.445166667	0.008863333	0.046166667				1
RFX2	regulatory factor X, 2 (influences HLA class II expression)	19p13.3				-0.406666667	0.004815278	0.030730556				1
RFX3	regulatory factor X, 3 (influences HLA class II expression)	9p24.2				-0.331	0.002072857	0.022742857				1
RFX4	regulatory factor X, 4 (influences HLA class II expression)	12q24							0.503	2.328	5	1
NOP58	NOP58 ribonucleoprotein homolog (yeast)	2q33.1	0.481	0.00982	0.0577							1
RFXANK	regulatory factor X-associated ankyrin-containing protein	19p12				-0.462333333	0.00164	0.016833333				1
RFXAP	regulatory factor X-associated protein	13q14							-0.848	2.96	6	1
RFXDC2	Data not found								-0.848	3.21778	9	1
RG9MTD3	RNA (guanine-9-) methyltransferase domain containing 3	9p13.2				-0.400666667	7.00E-04	0.009933333				1
RGAG1	retrotransposon gag domain containing 1	Xq23				0.33	0.00466	0.0378				1
SART3	squamous cell carcinoma antigen recognized by T cells 3	12q24.1	0.481	0.00635	0.0429				-0.832	2.958	5	2
SHCBP1	SHC SH2-domain binding protein 1	16q11.2	0.481	0.04417	0.155							1
WNK2	In multiple Geneids		0.481	0.02864	0.117							1
RGL4	ral guanine nucleotide dissociation stimulator-like 4	22q11.23	-0.184	0.02236	0.0996	-0.461333333	0.0017	0.014166667				2
RGMA	RGM domain family, member A	15q26.1	-1.322	0	1.00E-04	-0.319	0.00461	0.0376				2
RGNEF	190 kDa guanine nucleotide exchange factor	5q13.2				0.347	0.00055	0.0134				1
RGP1	RGP1 retrograde golgi transport homolog (S. cerevisiae)	9pter-p22.1	-0.265	0.01802	0.0865	-0.355333333	0.00049	0.010533333				2
ABTB1	ankyrin repeat and BTB (POZ) domain containing 1	3q21	0.48	0.00151	0.0157							1
FBN2	fibrillin 2	5q23-q31	0.48	0.00434	0.0331							1
HSD17B3	hydroxysteroid (17-beta) dehydrogenase 3	9q22	0.48	0.02662	0.1118							1
PMS2P5	postmeiotic segregation increased 2 pseudogene 5	7q11.23	0.48	0.0413	0.1487							1
TATDN1	TatD DNase domain containing 1	8q24.13	0.48	0.01323	0.0703	0.33	0.01886	0.0823	-0.888	2.526	5	3
RGS10	regulator of G-protein signaling 10	10q25	-0.609	0.00052	0.0074	-0.324	0.00073	0.0153				2
RGS11	regulator of G-protein signaling 11	16p13.3							0.625	2.426	5	1
RGS12	regulator of G-protein signaling 12	4p16.3	-0.544	5.00E-05	0.0014	-0.3835	0.00146125	0.0165				2
RGS13	regulator of G-protein signaling 13	1q31.2				0.3785	0.00099	0.0162	-0.92	3.41667	6	2
RGS14	regulator of G-protein signaling 14	5q35.3	-0.455	0.00019	0.0035	-0.421	0.00441	0.0366				2
ZNF594	zinc finger protein 594	17p13	0.48	0.01852	0.0882							1
RGS18	regulator of G-protein signaling 18	1q31.2				0.33325	0.0069275	0.03775	-1.008	3.63667	6	2
RGS20	regulator of G-protein signaling 20	8q11.23	-0.333	0.00239	0.0217	-0.308	0.0012	0.0192				2
RGS21	regulator of G-protein signaling 21	1q31.1				0.344444444	0.002011111	0.020666667	-1.049	3.96571	7	2
RGS22	regulator of G-protein signaling 22	8q22.2	-0.19	0.04171	0.1495							1
CHST1	carbohydrate (keratan sulfate Gal-6) sulfotransferase 1	11p11.2	0.479	6.00E-05	0.0015							1
RGS4	regulator of G-protein signaling 4	1q23.3							-0.78	2.85429	7	1
POLR2M	polymerase (RNA) II (DNA directed) polypeptide M	15q21.3	0.479	0.02859	0.1168							1
RGS6	regulator of G-protein signaling 6	14q24.3				-0.384	0.00075	0.0154				1
RGS7	regulator of G-protein signaling 7	1q43				0.356	0.0013975	0.01705				1
RGS9BP	regulator of G protein signaling 9 binding protein	19q13.11	-0.326	0.01497	0.0765							1
RHAG	Rh-associated glycoprotein	6p12.3							-0.992	3.858	5	1
RHBDD1	rhomboid domain containing 1	2q36.3				0.376	0.002565	0.02115				1
SDF2L1	stromal cell-derived factor 2-like 1	22q11.21	0.479	0.00075	0.0096							1
RHBDD3	rhomboid domain containing 3	22q12.2				-0.406	0.00014	0.0076				1
RHBDF1	rhomboid 5 homolog 1 (Drosophila)	16p13.3				-0.346	0.00903	0.0541				1
RHBDF2	rhomboid 5 homolog 2 (Drosophila)	17q25.1				-0.38	8.00E-05	0.0061				1
RHBDL2	In multiple Geneids		-0.665	0.03534	0.1342	-0.412333333	0.003106667	0.028066667				2
RHBDL3	In multiple Geneids		-0.319	0.00893	0.054	-0.369	0.00274	0.02182				2
RHBG	Rh family, B glycoprotein (gene/pseudogene)	1q21.3				0.448	2.00E-05	0.0037				1
RHCE	In multiple Geneids		-0.419	0.00167	0.0168	-0.581307692	0.003146154	0.025592308				2
RHCG	Rh family, C glycoprotein	15q25	-4.665	3.00E-05	9.00E-04							1
RHD	Rh blood group, D antigen	1p36.11				-0.664117647	0.00059	0.012617647				1
SLC12A8	solute carrier family 12 (potassium/chloride transporters), member 8	3q21.2	0.479	0.00555	0.0392							1
RHO	rhodopsin	3q21-q24	-0.242	0.02338	0.1025							1
RHOA	ras homolog family member A	3p21.3				-0.407	0.001842222	0.0193				1
MOK	MOK protein kinase	14q32	0.478	0.00367	0.0295							1
ORC6	origin recognition complex, subunit 6	16q12	0.478	0.0038	0.0302							1
ROCK1	Rho-associated, coiled-coil containing protein kinase 1	18q11.1	0.478	0.01198	0.0661							1
BCL2A1	BCL2-related protein A1	15q24.3	0.477	0.01136	0.0638							1
RHOD	ras homolog family member D	11q14.3	-1.354	7.00E-05	0.0017							1

RHOG	ras homolog family member G	11p15.5-p15.4	-0.653	0.00129	0.014	-0.3285	0.01841	0.0779				2
RHOH	ras homolog family member H	4p13				-0.336	0.005878333	0.037				1
RHOJ	ras homolog family member J	14q23.2				0.464	0.00867	0.053	-0.72	2.945	6	2
ORC2	origin recognition complex, subunit 2	2q33	0.477	0.00097	0.0115							1
RHOT1	ras homolog family member T1	17q11.2	-0.311	0.04644	0.1598							1
PFDN4	prefoldin subunit 4	20q13.2	0.477	0.02052	0.0943	0.52875	0.0110075	0.059375				2
RHOV	ras homolog family member V	15q13.3	-1.29	0	0							1
RHOXF2	Rhox homeobox family, member 2	Xq24	-0.249	0.01198	0.0661							1
GALNT2	UDP-N-acetyl-alpha-D-galactosamine:polypeptide N-acetylgalactosaminyltran	1q41-q42	0.476	0.01456	0.075	0.347	1.00E-04	0.005833333				2
RIBC2	RIB43A domain with coiled-coils 2	22q13.31				-0.4212	0.001048	0.01582				1
RIC3	resistance to inhibitors of cholinesterase 3 homolog (C. elegans)	11p15.4	-1.125	0	1.00E-04							1
MOB3B	MOB kinase activator 3B	9p21.2	0.476	0.02674	0.112							1
RICH2	Data not found					-0.356833333	0.001646667	0.017675				1
RICS	Data not found								-0.775	2.545	6	1
PCTP	In multiple Geneids		0.476	0.04637	0.1597							1
RBBP5	retinoblastoma binding protein 5	1q32	0.476	0.00077	0.0097							1
RILP	Rab interacting lysosomal protein	17p13.3				-0.404	0.0335	0.1162				1
RILPL1	Rab interacting lysosomal protein-like 1	12q24.31				-0.361	0.002055	0.02207				1
RILPL2	Rab interacting lysosomal protein-like 2	12q24.31				-0.325	0.008515	0.0509				1
RIMBP2	RIMS binding protein 2	12q24.33							-1.04	3.54833	6	1
RIMBP3	RIMS binding protein 3	22q11.21				-0.466	0.00032	0.0107				1
TMEM194B	In multiple Geneids		0.4755	0.003145	0.0265	0.304	0.00047	0.0126				2
RIMS1	regulating synaptic membrane exocytosis 1	6q12-q13				0.345	0.04222	0.134				1
RIMS2	regulating synaptic membrane exocytosis 2	8q22.3				0.343615385	0.000149231	0.006692308	-0.888	2.644	5	2
RIMS3	regulating synaptic membrane exocytosis 3	1pter-p22.2	-0.363	0.00564	0.0396	-0.32	0.003625	0.03185				2
RIMS4	regulating synaptic membrane exocytosis 4	20q13.12	-0.354	0.00818	0.0509							1
RIN1	Ras and Rab interactor 1	11q13.2	-0.353	0.01075	0.0615	-0.33	0.00437	0.0364				2
RIN3	Ras and Rab interactor 3	14q32.12				-0.344	0.004987143	0.035471429				1
RING1	ring finger protein 1	6p21.3	-0.438	0.03243	0.1268							1
RINT1	RAD50 interactor 1	7q22.3	-0.422	0.02424	0.1048	-0.5	0.00038	0.0115				2
RIOK1	RIO kinase 1 (yeast)	6p24.3				-0.35625	0.0064425	0.04295				1
RIOK2	RIO kinase 2 (yeast)	5q15							-1.039	3.40167	6	1
RIOK3	RIO kinase 3 (yeast)	18q11.2	-0.762	0.01912	0.0901							1
RIPK1	receptor (TNFRSF)-interacting serine-threonine kinase 1	6p25.2				-0.443	0.00046	0.0125				1
BAIAP2L1	BAI1-associated protein 2-like 1	7q22.1	0.475	0.04069	0.1472							1
RIPK3	receptor-interacting serine-threonine kinase 3	14q11.2				-0.533	2.00E-05	0.0039				1
RIPK4	receptor-interacting serine-threonine kinase 4	21q22.3	-1.159	0	1.00E-04	-0.403833333	0.003145	0.021816667				2
RIT1	Ras-like without CAAX 1	1q22	-0.541	0.01856	0.0883							1
RIT2	Ras-like without CAAX 2	18q12.3							-0.757	2.86333	6	1
RLBP1L1	Data not found					0.34	0.00012	0.00635				1
RLIM	ring finger protein, LIM domain interacting	Xq13-q21	-0.401	0.03318	0.1287							1
RLN1	relaxin 1	9p24.1							-0.93	2.853	5	1
RLN2	relaxin 2	9p24.1							-0.64	2.928	5	1
RMND1	required for meiotic nuclear division 1 homolog (S. cerevisiae)	6q25.1				-0.321	0.00211	0.0252				1
RMND5B	required for meiotic nuclear division 5 homolog B (S. cerevisiae)	5q35.3	-1.64	0	0	-0.408	0.002346667	0.025733333				2
RNA18S5	Data not found		-0.176	0.03722	0.1391							1
RAE1	RAE1 RNA export 1 homolog (S. pombe)	20q13.31	0.475	0.02941	0.119							1
RNASE11	ribonuclease, RNase A family, 11 (non-active)	14q11.2							-0.754	3.305	6	1
RNASE12	ribonuclease, RNase A family, 12 (non-active)	14q11.1							-0.754	3.305	6	1
RNASE13	In multiple Geneids		-0.31	0.02299	0.1014							1
CCDC112	coiled-coil domain containing 112	5q22.3	0.474	0.03395	0.1305							1
RNASE3	ribonuclease, RNase A family, 3	14q24-q31							-0.665	2.844	5	1
RNASE4	ribonuclease, RNase A family, 4	14q11							-0.792	2.892	5	1
RNASE7	ribonuclease, RNase A family, 7	14q11.2	-3.333	0	0							1
RNASE8	ribonuclease, RNase A family, 8	14q11.2	-0.416	0.00236	0.0215							1
RNASE9	ribonuclease, RNase A family, 9 (non-active)	14q11.2	-0.243	0.01837	0.0878							1
EFNB1	ephrin-B1	Xq12	0.474	0.01309	0.0699							1
RNASEH2A	ribonuclease H2, subunit A	19p13.2				-0.465	0.002435	0.02615				1
RNASEH2B	ribonuclease H2, subunit B	13q14.3				0.465	0.006355	0.03845				1
RNASEK	ribonuclease, RNase K	17p13.1	-0.537	0.0137	0.0721	-0.454	0.00185	0.0236				2
RNASEN	Data not found					0.335666667	0.015776667	0.074				1
IBSP	integrin-binding sialoprotein	4q21.1	0.474	0.00342	0.0281				-0.886	3.12286	7	2
TMF1	TATA element modulatory factor 1	3p21-p12	0.474	0.00771	0.049							1
RND2	Rho family GTPase 2	17q21				-0.356	0.00936	0.0552				1
RND3	Rho family GTPase 3	2q23.3							-0.761	2.54571	7	1
RNF10	ring finger protein 10	12q24.31							-0.869	2.576	5	1
NAA38	N(alpha)-acetyltransferase 38, NatC auxiliary subunit	7q31.1-q31.3	0.473	0.01161	0.0647							1
RNF11	ring finger protein 11	1p32	-0.918	0	1.00E-04							1
RNF111	ring finger protein 111	15q21				-0.326	0.04208	0.1337				1

PYCR2	pyrroline-5-carboxylate reductase family, member 2	1q42.12	0.473	0.00041	0.0062							1
UTP6	UTP6, small subunit (SSU) processome component, homolog (yeast)	17q11.2	0.473	0.00796	0.05							1
VHL	von Hippel-Lindau tumor suppressor, E3 ubiquitin protein ligase	3p25.3	0.473	0.01666	0.082							1
RNF122	ring finger protein 122	8p12				-0.354333333	0.006471667	0.041166667				1
RNF123	ring finger protein 123	3p24.3				-0.472	3.50E-05	0.00435				1
RNF125	ring finger protein 125, E3 ubiquitin protein ligase	18q12.1				-0.4295	0.01303	0.056625				1
RNF126	ring finger protein 126	19p13.3	-0.389	0.00963	0.0569				0.845	3.05333	6	2
RNF126P1	ring finger protein 126 pseudogene 1	17q22	-0.238	0.01171	0.0651							1
ZNF518A	zinc finger protein 518A	10q24.1	0.473	0.00668	0.0445							1
RNF130	ring finger protein 130	5q35.3				-0.344090909	0.002702727	0.022418182				1
ANKRD52	ankyrin repeat domain 52	12q13.3	0.472	0.00426	0.0327							1
RNF135	ring finger protein 135	17q11.2	-0.584	0.00391	0.0308							1
RNF138	ring finger protein 138, E3 ubiquitin protein ligase	18q12.1				-0.4565	0.002605	0.0275				1
RNF14	ring finger protein 14	5q23.3-q31.1				-0.307	8.00E-05	0.006				1
RNF141	ring finger protein 141	11p15.4				0.323	0.00371	0.0335				1
RNF144A	ring finger protein 144A	2p25.2				0.3595	0.00244	0.0249				1
RNF144B	ring finger protein 144B	6p22.3	-1.072	7.00E-05	0.0018							1
RNF145	In multiple Geneids		-0.589	0.0017	0.017							1
RNF146	ring finger protein 146	6q22.1-q22.33							-0.792	2.684	5	1
C9orf64	chromosome 9 open reading frame 64	9q21.32	0.472	0.01291	0.0695							1
RNF151	ring finger protein 151	16p13.3	-0.26	0.03341	0.1293							1
RNF152	ring finger protein 152	18q21.33	-0.862	0.00689	0.0454	-0.334	0.00022	0.00785				2
LOC728819	hCG1645220	2p21	0.472	0.01264	0.0685							1
RNF160	Data not found					-0.317	0.001525	0.0193				1
RNF165	ring finger protein 165	18q21.1	-1.1	0	0	-0.3598	0.009369	0.05216				2
RNF167	ring finger protein 167	17p13.2	-0.375	0.00706	0.0462	-0.4355	0.004525	0.031				2
MIR4697HG	Data not found		0.472	0.02784	0.1148							1
RNF169	ring finger protein 169	11q13.4	-0.946	0.00021	0.0038							1
RNF170	ring finger protein 170	8p11.21	-0.388	0.03008	0.1209	-0.409	0.00601	0.04325				2
RNF175	ring finger protein 175	4q31.3				-0.359	0.00036	0.0112				1
RNF180	ring finger protein 180	5q12.3	-0.718	0.01678	0.0825							1
RNF185	ring finger protein 185	22q12.2	-0.5565	0.00328	0.0232	-0.375125	0.00108875	0.0148				2
RP9P	retinitis pigmentosa 9 pseudogene	7p14.3	0.472	0.00127	0.0139							1
SLC51B	Data not found		0.472	0.04542	0.1579							1
RNF19B	ring finger protein 19B	1p35.1	-0.559	0.03191	0.1256							1
SNRPB2	small nuclear ribonucleoprotein polypeptide B	20p12.1	0.472	0.00811	0.0506				-0.879	2.788	5	2
RNF207	ring finger protein 207	1p36.31	-0.249	0.01273	0.0688	-0.444	0.00016	0.0079				2
RNF208	ring finger protein 208	9q34.3	-1.109	0	0							1
RNF212	ring finger protein 212	4p16.3				-0.4006	0.00176	0.02096				1
STARD13	StAR-related lipid transfer (START) domain containing 13	13q12-q13	0.472	0.0131	0.0699	0.503833333	0.004176667	0.029616667	-0.765	2.91	6	3
TNFRSF10C	tumor necrosis factor receptor superfamily, member 10c, decoy without an in	8p22-p21	0.472	0.00346	0.0283							1
ZNF484	zinc finger protein 484	9q22.31	0.472	0.0195	0.0913							1
BAX	BCL2-associated X protein	19q13.3-q13.4	0.471	2.00E-05	6.00E-04							1
RNF217	ring finger protein 217	6q22.31				0.494	0.00283	0.0291				1
CBFB	core-binding factor, beta subunit	16q22.1	0.471	0.00825	0.0512							1
GOSR2	golgi SNAP receptor complex member 2	17q21	0.471	0.00778	0.0493							1
RNF222	ring finger protein 222	17p13.1	-0.926	0	0	-0.383	0.00058	0.01165				2
HOXA11	homeobox A11	7p15.2	0.471	0.01541	0.0779				-0.747	2.872	5	2
ISG20L2	interferon stimulated exonuclease gene 20kDa-like 2	1q23.1	0.471	0.00578	0.0404							1
RNF31	ring finger protein 31	14q11.2	-0.265	0.00738	0.0476							1
PDE9A	phosphodiesterase 9A	21q22.3	0.471	0.01719	0.0839							1
RNF34	ring finger protein 34, E3 ubiquitin protein ligase	12q24.31				-0.3495	0.00062	0.01265				1
RNF38	ring finger protein 38	9p13	-0.478	0.00945	0.0562	-0.3535	0.0037675	0.0276				2
RNF39	ring finger protein 39	6p21.3	-1.056	0.00453	0.0342							1
RNF4	ring finger protein 4	4p16.3	-0.242	0.01632	0.0809	-0.444	0.003305556	0.026777778				2
RNF40	ring finger protein 40, E3 ubiquitin protein ligase	16p11.2-p11.1				-0.379	0.00482	0.0385				1
RNF44	In multiple Geneids					-0.410666667	0.000943333	0.015966667				1
PRRG1	proline rich Gla (G-carboxyglutamic acid) 1	Xp21.1	0.471	0.02763	0.1141	0.333857143	0.00659	0.040371429				2
RNF8	ring finger protein 8, E3 ubiquitin protein ligase	6p21.3				0.4705	0.049035	0.14705				1
TBPL1	TBP-like 1	6q22.1-q22.3	0.471	0.03776	0.1403				-0.639	2.852	5	2
RNGTT	RNA guanylyltransferase and 5'-phosphatase	6q16							-0.57	2.36	6	1
RNH1	ribonuclease/angiogenin inhibitor 1	11p15.5	-0.776	6.00E-05	0.0015							1
RNLS	renalase, FAD-dependent amine oxidase	10q23.31	-1.458	1.00E-05	3.00E-04							1
RNMTL1	In multiple Geneids		-0.332	0.00419	0.0323	-0.464	0.002685	0.02815				2
RNPEPL1	In multiple Geneids		-0.303	0.04377	0.154							1
RNPS1	RNA binding protein S1, serine-rich domain	16p13.3				-0.4635	0.0050725	0.0344				1
TPM3	tropomyosin 3	1q21.2	0.471	0.002225	0.02035							1
RNU1-1	RNA, U1 small nuclear 1	1p36.1	-0.511	0.02168	0.0979							1
WDFY1	WD repeat and FYVE domain containing 1	2q36.1	0.471	0.00025	0.0043							1

ASL	argininosuccinate lyase	7q11.21	0.47	0.00249	0.0223							1
FAM150A	family with sequence similarity 150, member A	8q11.23	0.47	0.00464	0.0347							1
LOC100130428	IGYY565	11q24.2	0.47	0.04299	0.1525							1
WDR12	WD repeat domain 12	2q33.2	0.47	0.04624	0.1595							1
ROBO1	roundabout, axon guidance receptor, homolog 1 (Drosophila)	3p12				0.33	0.00308	0.0304	-0.935	3.31	7	2
ROBO2	roundabout, axon guidance receptor, homolog 2 (Drosophila)	3p12.3				-0.377	0.00461	0.0375				1
MAP3K2	mitogen-activated protein kinase kinase kinase 2	2q14.3	0.469	0.00376	0.03							1
C1orf56	chromosome 1 open reading frame 56	1q21.3	0.468	0.02539	0.1083							1
ROGDI	rogdi homolog (Drosophila)	16p13.3	-1.084	0	0	-0.538	0.00789	0.0503				2
ROPN1L	rhophilin associated tail protein 1-like	5p15.2				0.3795	0.0035	0.02995				1
ROR1	receptor tyrosine kinase-like orphan receptor 1	1p32-p31	-0.675	0.01104	0.0625							1
ROR2	receptor tyrosine kinase-like orphan receptor 2	9q22				-0.328	0.016083333	0.0636				1
RORA	RAR-related orphan receptor A	15q22.2				0.3764	0.00045	0.01088	-0.542	2.508	5	2
RORB	RAR-related orphan receptor B	9q22							0.818	2.67714	7	1
RORC	RAR-related orphan receptor C	1q21							0.75	2.726	5	1
RP1	In multiple Geneids								-0.988	3.293	10	1
RP11-145H9.1	Data not found								-0.919	3.256	5	1
RP11-165H20.1	CHIA-like pseudogene	1p13.2							-0.992	3.138	5	1
RP11-196G18.6	Data not found								0.707	2.74833	6	1
RP11-19J3.3	Data not found								-0.493	2.505	6	1
RP11-217H1.1	Data not found								-0.732	2.45714	7	1
RP11-262H14.4	Data not found								0.85	2.974285	6.5	1
RP11-298P3.3	Data not found								-0.734	2.68833	6	1
RP11-311P8.3	Data not found								-0.928	2.72667	9	1
RP11-35N6.1	Data not found								-1.123	2.82	6	1
RP11-479E16.1	Data not found								-0.747	2.76	6	1
RP11-493K23.2	Data not found								0.778	2.797	5	1
RP11-49G10.8	Data not found								-0.828	3.208	5	1
RP1-14N1.3	Data not found								-1.107	3.754	5	1
RP11-529I10.4	Data not found				-0.316	0.00115	0.0188					1
RP11-54H7.1	Data not found								-0.656	2.53	6	1
RP11-561O23.3	Data not found								0.682	3.07625	8	1
RP11-647M7.1	Data not found								-1.085	2.74857	7	1
RP13-11B7.1	Data not found								-0.958	3.6275	8	1
RP13-297E16.1	Data not found								-1.15375	2.8692825	7	1
RP13-36C9.1	Data not found								0.942	2.72	5	1
RP13-36C9.3	Data not found								0.942	2.72	5	1
RP13-383K5.1	Data not found								-0.783	2.49571	7	1
RP3-402G11.12	Data not found								0.708	2.502	5	1
RP5-1103G7.6	Data not found								-1.077	3.702	5	1
RP5-1153D9.3	Data not found								0.733	3.02167	6	1
RP6-213H19.2	Data not found								-0.916	2.75667	6	1
IQSEC1	IQ motif and Sec7 domain 1	3p25.2	0.468	0.01714	0.0837							1
MTR	5-methyltetrahydrofolate-homocysteine methyltransferase	1q43	0.468	0.02377	0.1035							1
RPA3	replication protein A3, 14kDa	7p22				0.336666667	0.004553333	0.033433333	-0.906	2.95429	7	2
RPAP1	RNA polymerase II associated protein 1	15q15.1				-0.3285	0.021005	0.0734				1
RPE65	retinal pigment epithelium-specific protein 65kDa	1p31				-0.488	2.00E-05	0.0039				1
ORC4	origin recognition complex, subunit 4	2q22-q23	0.468	0.00105	0.0121							1
RPGRIP1	retinitis pigmentosa GTPase regulator interacting protein 1	14q11				-0.335	0.009366667	0.048566667				1
RPH3AL	rabphilin 3A-like (without C2 domains)	17p13.3				-0.391285714	0.003470714	0.022635714	1.103	2.85167	6	2
RPL11	ribosomal protein L11	1p36.1-p35				-0.321	0.00761	0.0477				1
RPL13	ribosomal protein L13	16q24.3	-0.485	0.0118	0.0655	-0.454	0.00054	0.0134				2
PGF	placental growth factor	14q24.3	0.468	0.00079	0.01							1
RPL15	ribosomal protein L15	3p24.2	-0.433	0.03851	0.14165							1
RPL17	ribosomal protein L17	18q21							-1.023	3.27286	7	1
RPL18	ribosomal protein L18	19q13							-0.622	2.45	5	1
RPL18A	ribosomal protein L18a	19p13	-0.274	0.03625	0.1367	-0.549	6.00E-05	0.0055				2
RPL18AP3	In multiple Geneids					-0.549	6.00E-05	0.0055				1
ZNRD1	zinc ribbon domain containing 1	6p21.3	0.468	0.03304	0.1283				-0.793	3.106	5	2
RPL23AP82	ribosomal protein L23a pseudogene 82	22q13.33	-0.226	0.02264	0.1003	-0.31	0.00016	0.0078				2
RPL26	ribosomal protein L26	17p13				-0.3625	0.00183	0.0234				1
RPL26L1	ribosomal protein L26-like 1	5q35.1				-0.3605	0.009545	0.05425				1
RPL27A	ribosomal protein L27a	11p15	-0.601	8.00E-05	0.0019							1
RPL3	ribosomal protein L3	22q13	-0.341	0.00407	0.0317	-0.492	6.00E-05	0.0056				2
RPL32	ribosomal protein L32	3p25-p24							-0.819	2.698	5	1
RPL34	ribosomal protein L34	4q25	-0.26	0.01601	0.08							1
DIEXF	digestive organ expansion factor homolog (zebrafish)	1q32.2	0.467	0.00063	0.0085							1
RPL3L	ribosomal protein L3-like	16p13.3	-0.245	0.02231	0.0995							1
RPL4	ribosomal protein L4	15q22				-0.407	0.00015	0.0077				1

MAPKAPK5	mitogen-activated protein kinase-activated protein kinase 5	12q24.13	0.467	0.00063	0.0085							1
RPL6	ribosomal protein L6	12q24.1				-0.35	0.011905	0.05465				1
RPLP0	ribosomal protein, large, P0	12q24.2							-0.783	2.462	5	1
RPLP1	ribosomal protein, large, P1	15q22							-0.758	2.482	5	1
SNORD114-26	small nucleolar RNA, C/D box 114-26	14q32	0.467	0.01643	0.0813							1
ZNF37BP	zinc finger protein 37B, pseudogene	10q11.21	0.467	0.00045	0.0067							1
MRPL14	mitochondrial ribosomal protein L14	6p21.3	0.466	0.03558	0.1348	0.381	0.00908	0.0543				2
RPP30	ribonuclease P/MRP 30kDa subunit	10q23.31							-1.02	3.715	6	1
RPP38	ribonuclease P/MRP 38kDa subunit	10p13				-0.416	0.00459	0.0361				1
RPRD1A	regulation of nuclear pre-mRNA domain containing 1A	18q12.2				-0.334	0.0134	0.0675				1
C11orf49	chromosome 11 open reading frame 49	11p11.2	0.465	0.00704	0.0461							1
RPS13	ribosomal protein S13	11p15				-0.502	0.00041	0.0119				1
RPS14	ribosomal protein S14	5q31-q33				-0.435	0.000753333	0.011466667				1
RPS14P3	ribosomal protein S14 pseudogene 3	1p36.13	-0.386	0.00692	0.0455							1
RPS15	ribosomal protein S15	19p13.3				-0.315	0.00125	0.0196				1
RPS2	ribosomal protein S2	16p13.3	-0.3585	0.015455	0.0755	-0.551	0.00199	0.0245				2
FAM73A	family with sequence similarity 73, member A	1p31.1	0.465	0.0163	0.0809							1
FKTN	fukutin	9q31-q33	0.465	0.00169	0.0169							1
NEK11	NIMA (never in mitosis gene a)- related kinase 11	3q22.1	0.465	0.00054	0.0075	0.3076	0.00093	0.01638				2
RPS29	ribosomal protein S29	14q				-0.415	0.00145	0.021				1
RPS2P32	ribosomal protein S2 pseudogene 32	7p15.3	-0.215	0.04619	0.1594							1
PCGF2	polycomb group ring finger 2	17q12	0.465	0.01025	0.0596							1
RPS3A	ribosomal protein S3A	4q31.2-q31.3	-0.375	0.017705	0.0803	-0.38	0.00018	0.0084				2
RPS4Y1	ribosomal protein S4, Y-linked 1	Yp11.3	-0.776	0.04572	0.1585	-0.483083333	0.022435833	0.089091667				2
RPS4Y2	ribosomal protein S4, Y-linked 2	Yq11.223				-0.5418	0.02352	0.09065				1
RPS6	ribosomal protein S6	9p21	-0.323	0.02908	0.1181	-0.3305	0.003885	0.03395				2
RPS6KA1	ribosomal protein S6 kinase, 90kDa, polypeptide 1	1p				-0.428857143	0.003638571	0.030942857				1
RPS6KA2	ribosomal protein S6 kinase, 90kDa, polypeptide 2	6q27				-0.345	0.00206	0.024166667				1
RNF2	ring finger protein 2	1q25.3	0.465	0.00385	0.0305							1
RPS6KA5	ribosomal protein S6 kinase, 90kDa, polypeptide 5	14q31-q32.1				-0.346	0.00195	0.021				1
RPS6KA6	ribosomal protein S6 kinase, 90kDa, polypeptide 6	Xq21				0.3495	0.00211	0.020275				1
RPS6KB1	ribosomal protein S6 kinase, 70kDa, polypeptide 1	17q23.1	-0.209	0.0469	0.1607							1
RPS6KB2	ribosomal protein S6 kinase, 70kDa, polypeptide 2	11q13.2	-0.81	1.00E-05	5.00E-04							1
RPS6KL1	ribosomal protein S6 kinase-like 1	14q24.3				-0.387	0.00015	0.0078				1
RPS8	ribosomal protein S8	1p34.1-p32				-0.4	0.00419	0.0356				1
RPSA	ribosomal protein SA	3p22.2							-0.784	2.634	5	1
RPSAP58	ribosomal protein SA pseudogene 58	19p12	-1.467	1.00E-05	3.00E-04							1
RPTN	repetin	1q21.3				0.34	0.0061	0.0436				1
FGD5	FYVE, RhoGEF and PH domain containing 5	3p25.1	0.464	0.02425	0.1048							1
RPUSD3	RNA pseudouridylate synthase domain containing 3	3p25.3	-0.572	0.00435	0.0332							1
GON4L	gon-4-like (C. elegans)	1q22	0.464	0.00197	0.0189							1
RRAD	Ras-related associated with diabetes	16q22	-1.153	1.00E-05	4.00E-04							1
RRAGA	Ras-related GTP binding A	9p22.1	-0.31	0.00846	0.0521	-0.416	5.00E-05	0.005				2
LOC100131826	TSSP3028	4q21.1	0.464	0.02745	0.1137							1
RRAGD	Ras-related GTP binding D	6q15-q16	-2.08	4.00E-05	0.0011	-0.397333333	0.002066667	0.020866667				2
RRAS	related RAS viral (r-ras) oncogene homolog	19q13.3-qter				-0.493	0.00065	0.0145				1
RRAS2	related RAS viral (r-ras) oncogene homolog 2	11p15.2				-0.371	0.00131	0.02				1
RPP25	ribonuclease P/MRP 25kDa subunit	15q24.2	0.464	0.0196	0.0914							1
RREB1	ras responsive element binding protein 1	6p25							-0.914	2.885	8	1
RRH	retinal pigment epithelium-derived rhodopsin homolog	4q25							-0.828	3.026	5	1
RRM1	ribonucleotide reductase M1	11p15.5				-0.346	0.00528	0.0403				1
SEC22B	SEC22 vesicle trafficking protein homolog B (S. cerevisiae) (gene/pseudogene)	1q21.1	0.464	0.03875	0.1427							1
ADPGK	ADP-dependent glucokinase	15q24.1	0.463	0.01317	0.0701							1
RRP1	ribosomal RNA processing 1 homolog (S. cerevisiae)	21q22.3	-0.222	0.0247	0.1062	-0.34	0.00508	0.03935				2
RRP12	ribosomal RNA processing 12 homolog (S. cerevisiae)	10q24.1				-0.3498	0.001312	0.01656				1
FAM122B	family with sequence similarity 122B	Xq26.3	0.463	0.02207	0.0989							1
RRP1B	ribosomal RNA processing 1 homolog B (S. cerevisiae)	21q22.3				-0.37325	0.0005175	0.01085				1
RRP22	Data not found								0.723	2.558	5	1
GINS4	GINS complex subunit 4 (Sld5 homolog)	8p11.21	0.463	0.04448	0.1557							1
RRP7A	ribosomal RNA processing 7 homolog A (S. cerevisiae)	22q13.2				-0.836	0	4.00E-04				1
RRP9	ribosomal RNA processing 9, small subunit (SSU) processome component, hor	3p21.2				-0.438	1.00E-04	0.0065				1
GPR176	G protein-coupled receptor 176	15q14-q15.1	0.463	0.00107	0.0122							1
RSBN1	round spermatid basic protein 1	1p13.2				-0.3538	0.000576	0.01118				1
NCAPD3	non-SMC condensin II complex, subunit D3	11q25	0.463	0.04041	0.1465							1
RSC1A1	regulatory solute carrier protein, family 1, member 1	1p36.1							-0.883	2.68	7	1
C10orf2	chromosome 10 open reading frame 2	10q24	0.462	0.03902	0.1432							1
RSG1	REM2 and RAB-like small GTPase 1	1p36.13	-0.356	0.00049	0.0071							1
HMGXB3	HMG box domain containing 3	5q32	0.462	0.00019	0.0036							1
RSPH10B	radial spoke head 10 homolog B (Chlamydomonas)	7p22.1				-0.319	0.030415	0.10665				1

RSPH10B2	radial spoke head 10 homolog B2 (Chlamydomonas)	7p22.1				-0.319	0.030415	0.10665				1
RSPH3	radial spoke 3 homolog (Chlamydomonas)	6q25.3	-0.813	7.00E-05	0.0017							1
RSPO2	R-spondin 2	8q23.1				0.32475	0.0001525	0.006575				1
RSPO3	R-spondin 3	6q22.33				0.34925	0.0244125	0.09345				1
RSPO4	R-spondin 4	20p13							1.117	2.484	5	1
SNORA15	small nucleolar RNA, H/ACA box 15	7p11.2	0.462	0.02202	0.0988							1
RSRC2	arginine/serine-rich coiled-coil 2	12q24.31				-0.319	0.01579	0.0743				1
RTBDN	retbindin	19p12				-0.478333333	0.00564	0.039333333				1
RTDR1	rhabdoid tumor deletion region gene 1	22q11.2				-0.38825	0.001104167	0.014333333				1
C1QTNF5	C1q and tumor necrosis factor related protein 5	11q23.3	0.461	0.01387	0.0727							1
RTF1	Rtf1, Paf1/RNA polymerase II complex component, homolog (S. cerevisiae)	15q15.1				-0.379571429	0.015335714	0.062485714				1
DCUN1D2	DCN1, defective in cullin neddylation 1, domain containing 2 (S. cerevisiae)	13q34	0.461	0.01357	0.0716							1
HIST1H2BJ	histone cluster 1, H2bj	6p22.1	0.461	0.03978	0.145							1
RTKN2	In multiple Geneids		-1.182	0.00106	0.0122							1
RTN1	reticulon 1	14q23.1				0.318	9.00E-05	0.0063				1
MGAT3	mannosyl (beta-1,4-)-glycoprotein beta-1,4-N-acetylglucosaminyltransferase	22q13.1	0.461	0.00342	0.0281							1
RTN3	reticulon 3	11q13				-0.301	0.00306	0.0303				1
RTN4	In multiple Geneids								-0.919	2.945	6	1
RTN4IP1	reticulon 4 interacting protein 1	6q21				-0.33	0.00085	0.0164				1
RTN4R	reticulon 4 receptor	22q11.21				-0.3815	0.00054	0.01335				1
RTN4RL1	reticulon 4 receptor-like 1	17p13.3	-0.292	0.01174	0.0652	-0.376142857	0.021842857	0.079685714				2
RTP3	receptor (chemosensory) transporter protein 3	3p21.3	-0.188	0.03828	0.1417							1
RQCD1	RCD1 required for cell differentiation1 homolog (S. pombe)	2q35	0.461	0.00824	0.0512							1
RTTN	rotatin	18q22.2				-0.3805	0.000775	0.01535				1
RUFY1	RUN and FYVE domain containing 1	5q35.3							-0.838	2.48667	6	1
RUFY2	RUN and FYVE domain containing 2	10q21.3				-0.3315	0.0172	0.06255				1
RUFY3	RUN and FYVE domain containing 3	4q13.3	-0.612	0.00064	0.0086	-0.403	0.004026667	0.0336				2
STXBP4	In multiple Geneids		0.461	0.02792	0.1151							1
RUNDC2A	RUN domain containing 2A	16p13.13				-0.344	0.00397	0.0347				1
RUNDC2C	RUN domain containing 2C	16p11.2				-0.33	0.0033675	0.029875				1
RUNDC3A	RUN domain containing 3A	17q21.31				-0.359	0.0066	0.0456				1
RUNDC3B	RUN domain containing 3B	7q21.12				0.3225	0.00498	0.03905				1
UBXN2B	UBX domain protein 2B	8q12.1	0.461	0.02372	0.1034	0.349	1.00E-04	0.0065				2
RUNX1T1	runt-related transcription factor 1; translocated to, 1 (cyclin D-related)	8q22				0.3382	0.000306	0.00876	-0.9875	3.124195	7.5	2
USP3	ubiquitin specific peptidase 3	15q22.3	0.461	0.00578	0.0404							1
RUNX3	runt-related transcription factor 3	1p36				-0.361625	0.006035	0.040125				1
RUSC2	RUN and SH3 domain containing 2	9p13.3	-0.577	0.00874	0.0533	-0.379428571	0.000765714	0.013757143				2
DGUOK	deoxyguanosine kinase	2p13	0.46	0.01387	0.0727							1
RUVBL2	RuvB-like 2 (E. coli)	19q13.3				-0.3545	0.001135	0.0186				1
IKBIP	IKBKB interacting protein	12q23.1	0.46	0.00066	0.0088							1
RWDD2B	RWD domain containing 2B	21q22.11	-0.665	0.00458	0.0344							1
RWDD4	RWD domain containing 4	4q35.1	-0.365	0.01871	0.0888							1
RWDD4A	Data not found					-0.363	0.01373	0.0685				1
RXFP1	relaxin/insulin-like family peptide receptor 1	4q32.1				-0.3285	0.002765	0.0286				1
RXFP2	relaxin/insulin-like family peptide receptor 2	13q13.1				0.3485	0.0110725	0.0551				1
RXFP4	relaxin/insulin-like family peptide receptor 4	1q22	-0.25	0.01301	0.0698							1
RXRA	retinoid X receptor, alpha	9q34.3	-0.849	3.00E-05	9.00E-04							1
RXRB	retinoid X receptor, beta	6p21.3	-1.291	6.00E-04	0.0082							1
RXRG	retinoid X receptor, gamma	1q22-q23	-0.324	0.01465	0.0753				0.652	2.4	5	2
RYK	receptor-like tyrosine kinase	3q22				0.402	0.001486667	0.017333333				1
RYR1	ryanodine receptor 1 (skeletal)	19q13.1				-0.415857143	0.006451429	0.037185714				1
RYR2	ryanodine receptor 2 (cardiac)	1q43				0.331071429	0.004723571	0.035835714				1
RYR3	ryanodine receptor 3	15q14-q15				-0.344	0.00095	0.0172				1
S100A10	S100 calcium binding protein A10	1q21				0.318	0.01463	0.0711				1
S100A11	S100 calcium binding protein A11	1q21	-0.478	0.04599	0.159							1
S100A12	S100 calcium binding protein A12	1q21	-3.508	0	3.00E-04							1
S100A14	S100 calcium binding protein A14	1q21.3	-2.328	0.00038	0.0059							1
S100A16	S100 calcium binding protein A16	1q21	-1.286	0.00364	0.0293							1
S100A2	S100 calcium binding protein A2	1q21	-2.552	1.00E-05	3.00E-04							1
S100A3	S100 calcium binding protein A3	1q21	-0.887	0.01711	0.0836							1
S100A4	S100 calcium binding protein A4	1q21							0.854	2.884	5	1
S100A7L2	In multiple Geneids		-0.241	0.03245	0.1268							1
S100A8	S100 calcium binding protein A8	1q21	-3.472	0.00156	0.016							1
S100A9	S100 calcium binding protein A9	1q21	-3.462	0.00013	0.0027							1
S100B	S100 calcium binding protein B	21q22.3	-0.92	0.00032	0.0051							1
S100Z	S100 calcium binding protein Z	5q13.3				-0.384857143	0.001737143	0.021328571				1
S1PR2	sphingosine-1-phosphate receptor 2	19p13.2	-0.188	0.03588	0.1356							1
S1PR5	sphingosine-1-phosphate receptor 5	19p13.2	-0.493	0.00015	0.003	-0.4705	0.001725	0.0208				2
NUP160	nucleoporin 160kDa	11p11.2	0.46	0.01688	0.0828							1

SFT2D1	SFT2 domain containing 1	6q27	0.46	0.02102	0.0959							1
SAA4	serum amyloid A4, constitutive	11p15.1-p14							-0.915	3.38	9	1
CACYBP	calcyclin binding protein	1q24-q25	0.459	0.00616	0.04205							1
SACM1L	SAC1 suppressor of actin mutations 1-like (yeast)	3p21.3							-0.811	2.496	5	1
SACS	spastic ataxia of Charlevoix-Saguenay (sacsin)	13q12				0.303	0.01342	0.0676				1
DLC1	deleted in liver cancer 1	8p22	0.459	0.01533	0.0777	0.414	0.01882	0.0822	-0.835	2.886	5	3
MRPL48	mitochondrial ribosomal protein L48	11q13.4	0.459	0.01288	0.0693							1
SLC35A1	solute carrier family 35 (CMP-sialic acid transporter), member A1	6q15	0.459	0.03298	0.1282							1
SAG	S-antigen; retina and pineal gland (arrestin)	2q37.1				-0.335	0.00022	0.0091				1
SALL1	sal-like 1 (Drosophila)	16q12.1							0.545	2.5025	6	1
SALL2	sal-like 2 (Drosophila)	14q11.1-q12				-0.347666667	0.002226667	0.024866667				1
ADAT1	adenosine deaminase, tRNA-specific 1	16q23.1	0.458	0.00619	0.0422							1
SAMD10	sterile alpha motif domain containing 10	20q13.33	-0.535	0.00077	0.0098				0.687	2.582	5	2
FASTKD3	FAST kinase domains 3	5p15.31	0.458	0.01098	0.0622	0.481	1.00E-04	0.0066				2
SAMD13	sterile alpha motif domain containing 13	1p31.1							-0.837	3.042	5	1
SAMD3	sterile alpha motif domain containing 3	6q23.1							-0.923	2.7	5	1
NSUN3	NOP2/Sun domain family, member 3	3q11.1	0.458	0.01254	0.0682				-0.815	2.292	5	2
SAMD7	sterile alpha motif domain containing 7	3q26.2							-0.779	2.69143	7	1
SAMD8	sterile alpha motif domain containing 8	10q22.2	-0.592	0.00122	0.0135	-0.303	0.00016	0.0079				2
SAMD9	sterile alpha motif domain containing 9	7q21.2				0.388	5.00E-04	0.0129	-0.806	3.11167	6	2
SOCS5	suppressor of cytokine signaling 5	2p21	0.458	0.00239	0.0217				-0.891	2.86667	6	2
SAMM50	sorting and assembly machinery component 50 homolog (S. cerevisiae)	22q13.31	-0.684	0.00145	0.0153	-0.431333333	0.000236667	0.00755				2
ST6GALNAC4	ST6 (alpha-N-acetyl-neuraminyl-2,3-beta-galactosyl-1,3)-N-acetylgalactosamir	9q34	0.458	0.03134	0.1243							1
SAP130	Sin3A-associated protein, 130kDa	2q14.3				-0.313	0.00334	0.0317				1
SAP30L	SAP30-like	5q33.2				-0.355	3.00E-05	0.0045				1
SAPS1	Data not found					-0.355666667	0.001873333	0.0229	0.583	2.67667	6	2
SAPS2	Data not found					-0.3646	0.001471	0.01702				1
SAR1B	SAR1 homolog B (S. cerevisiae)	5q31.1	-0.341	0.03302	0.1282	-0.402	0.00311	0.023333333				2
SARDH	sarcosine dehydrogenase	9q33-q34				-0.404	2.00E-04	0.0088				1
SARS2	seryl-tRNA synthetase 2, mitochondrial	19q13.2							-0.556	2.55	5	1
TFB2M	transcription factor B2, mitochondrial	1q44	0.458	0.02212	0.099							1
SART2	Data not found								-0.82	3.25571	7	1
TPP1	tripeptidyl peptidase I	11p15	0.457	0.01131	0.0636							1
SASH1	SAM and SH3 domain containing 1	6q24.3	-2.48	0	0	-0.328875	0.0057875	0.0370375				2
TYW1	tRNA-yW synthesizing protein 1 homolog (S. cerevisiae)	7q11.21	0.457	0.02442	0.1053							1
SATB1	SATB homeobox 1	3p23				0.301	0.02271	0.092				1
DDRGK1	DDRGK domain containing 1	20p13	0.456	0.00182	0.0179							1
MAD2L2	MAD2 mitotic arrest deficient-like 2 (yeast)	1p36	0.456	0.01003	0.0586							1
POLG2	polymerase (DNA directed), gamma 2, accessory subunit	17q	0.456	0.01563	0.0787				-0.58	2.52167	6	2
SBEM	Data not found								-0.869	2.848	5	1
SBF1	SET binding factor 1	22q13.33	-0.556	4.00E-05	0.0011	-0.5665	0.00015	0.00745				2
SBF1P1	In multiple Geneids					-0.379	0.00033	0.0108				1
SBF2	SET binding factor 2	11p15.4				-0.347	0.00808	0.0509				1
SBNO1	strawberry notch homolog 1 (Drosophila)	12q24.31				-0.345	0.00023	0.0092				1
SBNO2	strawberry notch homolog 2 (Drosophila)	19p13.3				-0.423	0.009906667	0.0507				1
SBP1	Data not found								0.945	2.77083	6	1
SBSN	suprabasin	19q13.13	-4.119	1.00E-05	5.00E-04							1
SC65	Data not found					-0.366	0.00173	0.02235				1
SCAF1	SR-related CTD-associated factor 1	19q13.3-q13.4				-0.430666667	0.001143333	0.0169				1
SCAMP1	secretory carrier membrane protein 1	5q14.1				-0.3355	0.00089	0.01575				1
SCAMP2	secretory carrier membrane protein 2	15q23-q25	-0.392	0.03333	0.1291	-0.3205	0.012555	0.06225				2
SLC4A7	solute carrier family 4, sodium bicarbonate cotransporter, member 7	3p22	0.456	0.02059	0.0945							1
SCAMP4	secretory carrier membrane protein 4	19p13.3	-0.434	0.00166	0.0167	-0.463	0.001915	0.02105				2
SUN1	Sad1 and UNC84 domain containing 1	7p22.3	0.456	0.00403	0.0315							1
ARL13B	ADP-ribosylation factor-like 13B	3q11.1	0.455	0.00075	0.0096							1
SCAP	SREBF chaperone	3p21.31				-0.343	0.00067	0.0134				1
SCAPER	S-phase cyclin A-associated protein in the ER	15q24	-0.353	0.02396	0.104							1
SCARA3	scavenger receptor class A, member 3	8p21				-0.3715	6.50E-05	0.00555				1
SCARA5	scavenger receptor class A, member 5 (putative)	8p21.1				-0.323	0.00336	0.0318				1
GNA11	guanine nucleotide binding protein (G protein), alpha 11 (Gq class)	19p13.3	0.455	0.03728	0.1392							1
JAK1	Janus kinase 1	1p32.3-p31.3	0.455	0.0018	0.0178							1
SCARF1	scavenger receptor class F, member 1	17p13.3				-0.358	0.04295	0.1354				1
SCARF2	scavenger receptor class F, member 2	22q11.21				-0.409	0.00085	0.0164				1
PAMR1	peptidase domain containing associated with muscle regeneration 1	11p13	0.455	0.04852	0.1641	0.3415	0.011285	0.05345				2
PSMA4	proteasome (prosome, macropain) subunit, alpha type, 4	15q25.1	0.455	0.0201	0.093							1
AUP1	ancient ubiquitous protein 1	2p13	0.454	0.00077	0.0097							1
DBF4B	DBF4 homolog B (S. cerevisiae)	17q21.31	0.454	0.01546	0.0781							1
DENND4B	DENN/MADD domain containing 4B	1q21	0.454	0.0051	0.037							1
PTPDC1	protein tyrosine phosphatase domain containing 1	9q22.32	0.454	0.00025	0.0043							1

TMEM243	Data not found		0.454	0.04015	0.1459							1
SCD	stearoyl-CoA desaturase (delta-9-desaturase)	10q24.31							-1.192	2.76667	6	1
SCD5	stearoyl-CoA desaturase 5	4q21.22				-0.3754	0.004553333	0.03178				1
SCEL	sciellin	13q22				0.3378	0.01112	0.05874				1
SCFD1	sec1 family domain containing 1	14q12							-0.986	3.19333	6	1
SCFD2	sec1 family domain containing 2	4q12	-0.34	0.03287	0.1279							1
CTNNB1	catenin (cadherin-associated protein), beta 1, 88kDa	3p21	0.453	0.01225	0.0672							1
HMBS	hydroxymethylbilane synthase	11q23.3	0.453	0.03238	0.1267							1
SCGB1A1	secretoglobin, family 1A, member 1 (uteroglobin)	11q12.3	-2.029	0.00016	0.0031							1
SCGB1C1	secretoglobin, family 1C, member 1	11p15.5	-0.451	0.00152	0.0157							1
SCGB1D2	secretoglobin, family 1D, member 2	11q13							-0.603	2.57333	6	1
SCGB1D4	secretoglobin, family 1D, member 4	11q12.3	-0.225	0.01729	0.0841							1
SCGB2A2	secretoglobin, family 2A, member 2	11q13	-0.192	0.02331	0.1022							1
SCGB3A2	secretoglobin, family 3A, member 2	5q32							-0.833	2.69	6	1
SCGN	secretagogin, EF-hand calcium binding protein	6p22.3-p22.1				0.307	0.02882	0.106				1
ISY1	ISY1 splicing factor homolog (S. cerevisiae)	3q21.3	0.453	0.00643	0.0432							1
SCIN	scinderin	7p21.3				0.322	0.000875	0.01555				1
MCF2L	MCF.2 cell line derived transforming sequence-like	13q34	0.453	0.02883	0.1175							1
PPID	peptidylprolyl isomerase D	4q31.3	0.453	0.01637	0.0811							1
TBL2	transducin (beta)-like 2	7q11.23	0.452	0.00133	0.0144							1
SCML4	sex comb on midleg-like 4 (Drosophila)	6q21				-0.348333333	0.000623333	0.0111				1
SCN10A	sodium channel, voltage-gated, type X, alpha subunit	3p22.2							-0.91	3.06286	7	1
SCN11A	sodium channel, voltage-gated, type XI, alpha subunit	3p22.2	-0.208	0.02993	0.1204							1
SCN1A	sodium channel, voltage-gated, type I, alpha subunit	2q24.3				0.336	0.000135	0.00735				1
TMEM125	transmembrane protein 125	1p34.2	0.452	0.0026	0.0231							1
SCN2A2									-0.913	3.07643	7	1
SCN2B	sodium channel, voltage-gated, type II, beta subunit	11q23	-0.303	0.00543	0.0387							1
SCN3A	sodium channel, voltage-gated, type III, alpha subunit	2q24				0.346714286	0.000748571	0.012942857	-0.951	2.76	7	2
SCN4A	sodium channel, voltage-gated, type IV, alpha subunit	17q23.3				-0.412	0.00691	0.0467				1
SCN5A	sodium channel, voltage-gated, type V, alpha subunit	3p21				-0.450666667	0.00241	0.025316667				1
SCN7A	In multiple Geneids					0.321	0.000315	0.0097	-0.869	3.09833	6	2
CHTOP	chromatin target of PRMT1	1q21.3	0.451	0.01953	0.0913							1
SCN9A	sodium channel, voltage-gated, type IX, alpha subunit	2q24				0.346142857	0.00064	0.008842857	-0.91	2.74667	6	2
SCNN1A	sodium channel, non-voltage-gated 1 alpha subunit	12p13	-0.985	0.01918	0.0903							1
SCNN1B	sodium channel, non-voltage-gated 1, beta subunit	16p12.2-p12.1	-3.63	0	0	-0.313	0.00326	0.03125				2
SCNN1D	sodium channel, non-voltage-gated 1, delta subunit	1p36.3-p36.2	-0.28	0.00197	0.0189							1
SCNN1G	sodium channel, non-voltage-gated 1, gamma subunit	16p12	-1.804	0	0	-0.319	0.00072	0.0152				2
SCO1	SCO cytochrome oxidase deficient homolog 1 (yeast)	17p13.1							-0.379	2.556	5	1
SCO2	SCO cytochrome oxidase deficient homolog 2 (yeast)	22q13.33	-0.372	0.01226	0.0672				0.649	2.634	5	2
SCOC	short coiled-coil protein	4q31.1							-0.575	2.4	5	1
SCPEP1	serine carboxypeptidase 1	17q22	-0.67	0.00263	0.0232							1
SCRG1	stimulator of chondrogenesis 1	4q34.1							-0.895	2.996	5	1
SCRIB	scribbled homolog (Drosophila)	8q24.3	-0.39	0.00763	0.0487							1
ELF4	E74-like factor 4 (ets domain transcription factor)	Xq26	0.451	0.0138	0.0725							1
SCRN3	secernin 3	2q31.1							-0.672	2.52	6	1
SCRT1	scratch homolog 1, zinc finger protein (Drosophila)	8q24.3							0.828	3.29	6	1
SCT	secretin	11p15.5	-0.289	0.01274	0.0688				1.091	3.74	8	2
SCUBE1	signal peptide, CUB domain, EGF-like 1	22q13				-0.445	0.000391538	0.008319231				1
SCUBE2	signal peptide, CUB domain, EGF-like 2	11p15.3	-1.105	0.00011	0.0024	-0.3225	0.00915	0.05035				2
HDDC3	HD domain containing 3	15q26.1	0.451	0.01537	0.0778							1
NFYB	nuclear transcription factor Y, beta	12q22-q23	0.451	0.0021	0.0197							1
SCYL2	SCY1-like 2 (S. cerevisiae)	12q23.1	-0.407	0.03347	0.1295							1
RCN2	reticulocalbin 2, EF-hand calcium binding domain	15q23	0.451	0.01752	0.0849							1
SDAD1	SDA1 domain containing 1	4q21.1				-0.336	0.004	0.0348				1
SDC1	syndecan 1	2p24.1	-1.077	4.00E-05	0.0012							1
SNX5	sorting nexin 5	20p11	0.451	0.02749	0.1138							1
TAF6	TAF6 RNA polymerase II, TATA box binding protein (TBP)-associated factor, 80	7q22.1	0.451	0.00297	0.0254							1
TBC1D16	TBC1 domain family, member 16	17q25.3	0.451	0.00056	0.0078							1
CEP135	centrosomal protein 135kDa	4q12	0.45	0.00483	0.0356							1
SDCBP2	syndecan binding protein (syntenin) 2	20p13	-0.591	0.01622	0.0806							1
SDCCAG1	Data not found								-0.804	2.94	6	1
SDCCAG10	Data not found					-0.322	0.00052	0.0131				1
SDCCAG8	serologically defined colon cancer antigen 8	1q43				0.351	0.00188	0.0238				1
COX19	COX19 cytochrome c oxidase assembly homolog (S. cerevisiae)	7p22.3	0.45	0.00507	0.0369							1
GDPD1	In multiple Geneids		0.45	0.02731	0.1134							1
AP1S1	adaptor-related protein complex 1, sigma 1 subunit	7q22.1	0.449	0.00793	0.0498							1
BTG1	B-cell translocation gene 1, anti-proliferative	12q22	0.449	0.02208	0.0989							1
SDHAF1	succinate dehydrogenase complex assembly factor 1	19q13.12				-0.347	0.00114	0.0187				1
GSDMD	gasdermin D	8q24.3	0.449	0.00185	0.018							1

SDHALP2	Data not found					-0.323	0.01684	0.0771				1
SDHB	succinate dehydrogenase complex, subunit B, iron sulfur (Ip)	1p36.1-p35				-0.346	0.00373	0.031133333				1
SDHC	succinate dehydrogenase complex, subunit C, integral membrane protein, 15k	1q23.3							-1.046	2.86	8	1
SDK1	sidekick cell adhesion molecule 1	7p22.2				0.507666667	0.0068	0.038566667				1
SDK2	sidekick cell adhesion molecule 2	17q25.1				-0.3441	0.002948	0.02707				1
SDPR	serum deprivation response	2q32-q33				0.338	1.00E-04	0.0065				1
SDR16C5	short chain dehydrogenase/reductase family 16C, member 5	8q12.1	-0.872	0.03101	0.1234							1
SDR42E1	short chain dehydrogenase/reductase family 42E, member 1	16q23.3	-0.797	0.00093	0.0112							1
SDR9C7	short chain dehydrogenase/reductase family 9C, member 7	12q13.3	-1.587	1.00E-05	5.00E-04							1
SNAP23	synaptosomal-associated protein, 23kDa	15q14	0.449	0.00215	0.0201							1
CDK12	cyclin-dependent kinase 12	17q12	0.448	0.01908	0.09							1
SEC1	In multiple Geneids					-0.3692	0.004856	0.0346				1
FANCF	Fanconi anemia, complementation group F	11p15	0.448	0.02979	0.12				-0.654	2.76125	8	2
SEC11C	SEC11 homolog C (S. cerevisiae)	18q21.32				-0.306	0.00553	0.0414				1
SEC14L1	SEC14-like 1 (S. cerevisiae)	17q25.2	-0.741	0.00036	0.0057							1
SEC14L2	SEC14-like 2 (S. cerevisiae)	22q12.2	-0.902	0.00655	0.0438	-0.38425	0.000275	0.009775				2
SEC14L3	SEC14-like 3 (S. cerevisiae)	22q12.2				-0.372	0.0005625	0.01335				1
SEC14L4	SEC14-like 4 (S. cerevisiae)	22q12.2				-0.380666667	0.003603333	0.030633333				1
SEC14L5	SEC14-like 5 (S. cerevisiae)	16p13.3	-0.264	0.01611	0.0803	-0.4135	0.0103725	0.05545				2
IFRD1	interferon-related developmental regulator 1	7q31.1	0.448	0.0218	0.0982							1
PSPC1	paraspeckle component 1	13q12.11	0.448	0.00462	0.0346	0.773	0.00016	0.008				2
RNF216P1	ring finger protein 216 pseudogene 1	7p22.1	0.448	0.00049	0.007							1
CCNJ	cyclin J	10q23.33	0.447	0.02332	0.1023							1
SEC24A	SEC24 family, member A (S. cerevisiae)	5q31.1				-0.3555	0.00579	0.04225				1
SEC24B	SEC24 family, member B (S. cerevisiae)	4q25	-0.409	0.01225	0.0672	-0.3254	0.002352	0.02408				2
SEC24C	SEC24 family, member C (S. cerevisiae)	10q22.2	-0.835	3.00E-05	0.001	-0.372	4.00E-05	0.0048				2
CXCL3	chemokine (C-X-C motif) ligand 3	4q21	0.447	0.01243	0.0677							1
SEC31A	SEC31 homolog A (S. cerevisiae)	4q21.22				-0.380666667	0.00239	0.0262				1
SEC31B	SEC31 homolog B (S. cerevisiae)	10q24.31				-0.3526	0.002946	0.02344				1
SEC61A2	Sec61 alpha 2 subunit (S. cerevisiae)	10p14				-0.367	0.017205	0.076				1
PHF3	PHD finger protein 3	6q12	0.447	0.01715	0.0837				-0.873	3.06667	6	2
ZNF620	zinc finger protein 620	3p22.1	0.447	0.0261	0.1104							1
SEC63D1	SEC63 domain containing 1	1p22.2							-0.829	2.914	5	1
SECISBP2L	SECIS binding protein 2-like	15q21.1	-0.885	0.00084	0.0103							1
SECTM1	secreted and transmembrane 1	17q25				-0.405	0.00372	0.0336				1
APOLD1	apolipoprotein L domain containing 1	12p13.1	0.446	0.03662	0.1376							1
CRIPT	cysteine-rich PDZ-binding protein	2p21	0.446	0.02869	0.1172							1
SELENBP1	selenium binding protein 1	1q21.3							1.322	2.68833	6	1
HOXC13	homeobox C13	12q13.3	0.446	0.01467	0.0754							1
SELO	selenoprotein O	22q13.33	-0.498	1.00E-04	0.0021							1
SELP	selectin P (granule membrane protein 140kDa, antigen CD62)	1q22-q25				0.355	0.002445	0.02305				1
SELPLG	selectin P ligand	12q24				-0.306	1.00E-04	0.0066				1
SEMA3A	sema domain, immunoglobulin domain (Ig), short basic domain, secreted, (ser	7p12.1				0.31625	0.0020575	0.02055	-0.779	2.692	5	2
SMPD4	sphingomyelin phosphodiesterase 4, neutral membrane (neutral sphingomyel	2q21.1	0.4455	0.00225	0.0203							1
ABCG1	ATP-binding cassette, sub-family G (WHITE), member 1	21q22.3	0.445	0.02674	0.112							1
SEMA3D	In multiple Geneids					0.3475	0.004312	0.03383				1
ACAN	aggrecan	15q26.1	0.445	0.00252	0.0225							1
SEMA3F	sema domain, immunoglobulin domain (Ig), short basic domain, secreted, (ser	3p21.3	-1.752	0	0							1
SEMA4A	In multiple Geneids		-1.88	0	0							1
SEMA4D	sema domain, immunoglobulin domain (Ig), transmembrane domain (TM) anc	9q22.2	-0.5785	0.00212	0.01825	-0.319	0.00858	0.0527				2
ACBD6	acyl-CoA binding domain containing 6	1q25.1	0.445	0.00256	0.0228							1
ACE	angiotensin I converting enzyme (peptidyl-dipeptidase A) 1	17q23.3	0.445	0.00182	0.0179							1
SEMA5A	sema domain, seven thrombospondin repeats (type 1 and type 1-like), transm	5p15.2				0.376724638	0.002636232	0.022714493				1
SEMA6A	In multiple Geneids								-0.951	2.854	5	1
CLN3	ceroid-lipofuscinosis, neuronal 3	16p12.1	0.445	0.04188	0.15							1
SEMA7A	semaphorin 7A, GPI membrane anchor (John Milton Hagen blood group)	15q22.3-q23				-0.339	0.00341	0.0321				1
SEMG1	semenogelin I	20q12-q13.2							-0.944	3.09667	6	1
SENP3	SUMO1/sentrin/SMT3 specific peptidase 3	17p13				-0.463	6.00E-05	0.0056				1
NUTF2	nuclear transport factor 2	16q22.1	0.445	0.00029	0.0048							1
SENP7	SUMO1/sentrin specific peptidase 7	3q12				0.346	0.00297	0.0299				1
SENP8	SUMO/sentrin specific peptidase family member 8	15q23	-0.508	0.00328	0.0273							1
RGS3	regulator of G-protein signaling 3	9q32	0.445	0.00053	0.0074							1
GRIPAP1	GRIP1 associated protein 1	Xp11	0.444	0.00425	0.0326							1
SEPN1	selenoprotein N, 1	1p36.13				-0.351	0.00827	0.0515				1
SEPP1	selenoprotein P, plasma, 1	5q31				0.363	0.00099	0.0176				1
SEPT1	septin 1	16p11.1				-0.3435	0.0032	0.02905				1
SEPT10	septin 10	2q13	-0.644	0.00263	0.0232							1
HNRNPU	heterogeneous nuclear ribonucleoprotein U (scaffold attachment factor A)	1q44	0.444	0.00137	0.0146							1
SEPT12	septin 12	16p13.3				-0.4605	0.003535	0.03075				1

LINC00467	long intergenic non-protein coding RNA 467	1q32.3	0.444	0.01437	0.0744							1
SEPT3	septin 3	22q13.2				-0.44	0.001138571	0.014557143				1
SEPT5	septin 5	22q11.21	-0.525	0.00148	0.013							1
SEPT6	septin 6	Xq24				-0.329	0.0073	0.0482				1
LRRC34	leucine rich repeat containing 34	3q26.2	0.444	0.03767	0.14							1
SEPT8	septin 8	5q31	-0.384	0.00356	0.0289	-0.3685	0.000165	0.006525				2
POLR1A	polymerase (RNA) I polypeptide A, 194kDa	2p11.2	0.444	0.00943	0.0562							1
SEPW1	selenoprotein W, 1	19q13.3				-0.344	0.0021	0.0252				1
SEPX1	Data not found					-0.551	0.0031	0.0305				1
SERF1A	small EDRK-rich factor 1A (telomeric)	5q13							-1.104888889	2.962591111	6	1
SERF1B	In multiple Geneids								-1.079333333	2.988886667	6	1
SERHL	In multiple Geneids		-0.546	0.00088	0.0107	-0.949	0	0.00075				2
SERHL2	serine hydrolase-like 2	22q13				-0.809	5.50E-05	0.0038				1
SERINC1	serine incorporator 1	6q22.31							-0.677	2.568	5	1
SERINC2	serine incorporator 2	1p35.1				-0.433666667	0.000586667	0.0124				1
SERINC4	serine incorporator 4	15q15.3				-0.305	0.00185	0.0236	0.693	2.974	5	2
SERINC5	serine incorporator 5	5q14.1				-0.398857143	0.001394762	0.016038095				1
PREPL	prolyl endopeptidase-like	2p21	0.444	0.01908	0.09							1
SERPINA12	serpin peptidase inhibitor, clade A (alpha-1 antiproteinase, antitrypsin), meml	14q32.13	-0.191	0.02936	0.1188	-0.35	0.00445	0.0368				2
SAAL1	serum amyloid A-like 1	11p15.1	0.444	0.00362	0.0292							1
TMEM200A	transmembrane protein 200A	6q23.1	0.444	0.01896	0.0897							1
SERPINA5	serpin peptidase inhibitor, clade A (alpha-1 antiproteinase, antitrypsin), meml	14q32.1				-0.317	1.00E-05	0.0029				1
SERPINA9	In multiple Geneids		-0.253	0.00298	0.0254	-0.336	0.00061	0.0141				2
SERPINB1	serpin peptidase inhibitor, clade B (ovalbumin), member 1	6p25	-2.103	4.00E-05	0.0012							1
SERPINB10	serpin peptidase inhibitor, clade B (ovalbumin), member 10	18q21.3							-1.077	3.23	7	1
SERPINB11	serpin peptidase inhibitor, clade B (ovalbumin), member 11 (gene/pseudogene)		-3.205	0	0							1
SERPINB12	serpin peptidase inhibitor, clade B (ovalbumin), member 12								-0.767	2.92714	7	1
SERPINB13	serpin peptidase inhibitor, clade B (ovalbumin), member 13	18q21.3-q22	-5.27	1.00E-05	3.00E-04							1
SERPINB2	serpin peptidase inhibitor, clade B (ovalbumin), member 2	18q21.3	-4.718	1.00E-05	5.00E-04							1
SERPINB3	serpin peptidase inhibitor, clade B (ovalbumin), member 3	18q21.3	-4.724	0.00038	0.0058							1
SERPINB4	serpin peptidase inhibitor, clade B (ovalbumin), member 4	18q21.3							-0.872	2.982	5	1
SERPINB5	serpin peptidase inhibitor, clade B (ovalbumin), member 5	18q21.3				-0.39	0.00037	0.0113				1
SERPINB6	serpin peptidase inhibitor, clade B (ovalbumin), member 6	6p25				-0.3315	0.003435	0.03215				1
SERPINB7	serpin peptidase inhibitor, clade B (ovalbumin), member 7	18q21.33							-0.966	3.482	5	1
SERPINB8	serpin peptidase inhibitor, clade B (ovalbumin), member 8	18q21.3							-0.763	3.05	6	1
SERPIND1	serpin peptidase inhibitor, clade D (heparin cofactor), member 1	22q11.21				-0.4184	0.000454	0.0078				1
CIB1	calcium and integrin binding 1 (calmyrin)	15q25.3-q26	0.443	0.01114	0.0629							1
SERPINF1	serpin peptidase inhibitor, clade F (alpha-2 antiplasmin, pigment epithelium d	17p13.3				-0.301	0.01857	0.0816				1
SERPINF2	serpin peptidase inhibitor, clade F (alpha-2 antiplasmin, pigment epithelium d	17p13				-0.387	0.02378	0.0945				1
COPB2	coatomer protein complex, subunit beta 2 (beta prime)	3q23	0.443	0.00773	0.0491							1
SERPINI1	serpin peptidase inhibitor, clade I (neuroserpin), member 1	3q26.1				0.359125	0.0046575	0.0289875				1
SERPINI2	serpin peptidase inhibitor, clade I (pancpin), member 2	3q26				0.366	0.00121	0.0193	-0.936	3.03625	8	2
SERTAD1	SERTA domain containing 1	19q13.1-q13.2	-0.403	0.01261	0.0684							1
SERTAD2	SERTA domain containing 2	2p14	-0.919	0.00343	0.0281							1
SERTM1	serine-rich and transmembrane domain containing 1	13q13.3	-0.384	0.00088	0.0107							1
SESN1	sestrin 1	6q21							-0.782	2.77833	6	1
SESN2	sestrin 2	1p35.3	-1.164	0.00034	0.0054	-0.423111111	0.006393333	0.041677778				2
SESN3	sestrin 3	11q21				0.322	1.50E-05	0.0033				1
EFCAB2	EF-hand calcium binding domain 2	1q44	0.443	0.01813	0.0869							1
LOC554223	histocompatibility antigen-related	6p21.3	0.443	0.00118	0.0131							1
SETBP1	SET binding protein 1	18q21.1				-0.335	0.00733	0.04035				1
SETD1A	SET domain containing 1A	16p11.2				-0.365	9.00E-05	0.0063				1
SETD1B	SET domain containing 1B	12q24.31	-0.351	0.00737	0.0476	-0.331333333	0.002906667	0.027666667				2
SETD2	SET domain containing 2	3p21.31							-0.739	2.99	6	1
SETD3	SET domain containing 3	14q32.2				-0.305	0.00819	0.0513				1
SETD4	SET domain containing 4	21q22.13							-0.656	2.588	5	1
SETD5	SET domain containing 5	3p25.3				-0.371	0.000745	0.01515				1
SETD7	SET domain containing (lysine methyltransferase) 7	4q28				-0.4145	0.0063	0.04425				1
SETD8	SET domain containing (lysine methyltransferase) 8	12q24.31	-0.629	0.001123333	0.0098	-0.3615	0.0077	0.0465				2
MRPS17	In multiple Geneids		0.443	0.04862	0.1643							1
SETMAR	SET domain and mariner transposase fusion gene	3p26.1	-0.282	0.03136	0.1243							1
SUPT3H	suppressor of Ty 3 homolog (S. cerevisiae)	6p21.1-p21.3	0.443	0.04995	0.167	0.35535	0.010222	0.05478				2
SEZ6L	In multiple Geneids					-0.367478261	0.002256957	0.017026087				1
CHRNA1	cholinergic receptor, nicotinic, alpha 1 (muscle)	2q31.1	0.442	0.00481	0.0356							1
SF1	splicing factor 1	11q13				-0.357	0.00031	0.0105				1
SF3A1	splicing factor 3a, subunit 1, 120kDa	22q12.2				-0.349	0.003875	0.027175				1
SF3A2	splicing factor 3a, subunit 2, 66kDa	19p13.3-p13.2				-0.439	0.00012	0.0072				1
INS-IGF2	INS-IGF2 readthrough	11p15.5	0.442	0.0027	0.0236							1
NFE2L1	nuclear factor (erythroid-derived 2)-like 1	17q21.3	0.442	0.02166	0.0978							1

SF3B3	splicing factor 3b, subunit 3, 130kDa	16q22.1				-0.3402	0.00903	0.0522				1
PGRMC1	progesterone receptor membrane component 1	Xq22-q24	0.442	0.00552	0.0391							1
SF4	Data not found					-0.391875	0.00386375	0.0289				1
SFI1	Sfi1 homolog, spindle assembly associated (yeast)	22q12.2				-0.426333333	0.002561667	0.022394444				1
SFMBT1	Scm-like with four mbt domains 1	3p21.1				0.418	0.03091	0.1106				1
SFN	stratifin	1p36.11	-1.804	0.00079	0.0099							1
SLC19A1	solute carrier family 19 (folate transporter), member 1	21q22.3	0.442	0.00543	0.0387							1
TMEM51-AS1	Data not found		0.442	0.04138	0.1489							1
SFRP1	secreted frizzled-related protein 1	8p11.21	-0.664	0.02989	0.1204							1
MFSD9	major facilitator superfamily domain containing 9	2q12.1	0.441	0.02002	0.0927							1
SFRP5	secreted frizzled-related protein 5	10q24.1				-0.38	0.00014	0.0073				1
SFRS11	Data not found								-0.725	2.68562	8	1
SFRS12	Data not found								-0.733	2.68625	8	1
SFRS14	Data not found					-0.426	0.001429167	0.018025				1
SFRS15	Data not found					-0.3734	0.00175	0.01954				1
SFRS16	Data not found					-0.3734	0.00647	0.03902				1
SFRS17A	Data not found					-0.5944	0.001878	0.01836				1
SFRS2IP	Data not found								-0.866	2.618	5	1
SFRS4	Data not found					-0.389	0.013146667	0.0589				1
SFRS8	Data not found					-0.424	0.00111	0.0185				1
NXPE3	Data not found		0.441	0.02089	0.0954							1
SFT2D2	SFT2 domain containing 2	1q24.2	-0.561	0.00681	0.045							1
POLR2C	polymerase (RNA) II (DNA directed) polypeptide C, 33kDa	16q13-q21	0.441	0.03909	0.1434							1
SFTPA1	surfactant protein A1	10q22.3				-0.368	0.00019	0.0085				1
SFTPA1B	In multiple Geneids					-0.368	0.00019	0.0085				1
SFTPD	surfactant protein D	10q22.2-q23.1							-0.816	2.8175	8	1
SFXN1	sideroflexin 1					-0.3395	0.00088	0.01305				1
SFXN2	In multiple Geneids					-0.395	0.004745	0.0326				1
SEC23IP	SEC23 interacting protein	10q25-q26	0.441	0.00808	0.0505							1
SGCD	sarcoglycan, delta (35kDa dystrophin-associated glycoprotein)	5q33-q34				-0.349666667	0.000493333	0.010933333				1
SLC36A1	solute carrier family 36 (proton/amino acid symporter), member 1	5q33.1	0.441	0.00498	0.0364				-0.778	2.705	8	2
SGCG	sarcoglycan, gamma (35kDa dystrophin-associated glycoprotein)	13q12							-0.839	2.96333	9	1
SGCZ	sarcoglycan, zeta	8p22				0.383666667	0.03174	0.109888889				1
SGEF	Data not found					0.3398	0.000926	0.01552				1
SLC38A10	solute carrier family 38, member 10	17q25.3	0.441	0.00356	0.0289							1
SGK1	serum/glucocorticoid regulated kinase 1	6q23				0.457	0.00264	0.0281				1
CEBPZ	CCAAT/enhancer binding protein (C/EBP), zeta	2p22.2	0.44	0.01596	0.0798				-0.72	3.132	5	2
SGK3	serum/glucocorticoid regulated kinase family, member 3	8q12							-0.793	2.546	5	1
SGOL1	shugoshin-like 1 (S. pombe)	3p24.3							-0.95	2.626	5	1
SGOL2	shugoshin-like 2 (S. pombe)	2q33.1				0.3145	2.00E-05	0.00325				1
SGPP1	sphingosine-1-phosphate phosphatase 1	14q23.2				-0.342333333	0.00937	0.052466667				1
SGSM1	small G protein signaling modulator 1	22q11.23				-0.423428571	0.001791905	0.01717619				1
SGSM2	small G protein signaling modulator 2	17p13.3	-0.534	3.00E-05	8.00E-04	-0.417	0.0017725	0.021225				2
SGSM3	small G protein signaling modulator 3	22q13.1-q13.2				-0.332333333	0.00524	0.038066667				1
SGTA	small glutamine-rich tetratricopeptide repeat (TPR)-containing, alpha	19p13	-0.281	0.04239	0.1511	-0.4742	0.000894	0.0153				2
DTNBP1	dystrobrevin binding protein 1	6p22.3	0.44	0.00417	0.0322							1
SH2B1	SH2B adaptor protein 1	16p11.2				-0.3545	0.00087	0.01655				1
SH2B2	SH2B adaptor protein 2	7q22				-0.386333333	0.006133333	0.0348				1
GLT8D1	glycosyltransferase 8 domain containing 1	3p21.1	0.44	0.02144	0.0972							1
SH2D1B	SH2 domain containing 1B	1q23.3	-1.888	7.00E-05	0.0016							1
HIST1H1C	histone cluster 1, H1c	6p21.3	0.44	0.00346	0.0283				-0.792	2.73167	6	2
SH2D3A	SH2 domain containing 3A	19p13.3				-0.426	0.004286667	0.0303				1
SH2D3C	SH2 domain containing 3C	9q34.11				-0.362	0.00541	0.0409				1
SH3BGR	SH3 domain binding glutamic acid-rich protein	21q22.3							-0.75	2.726	5	1
SH3BGRL	SH3 domain binding glutamic acid-rich protein like	Xq13.3				0.315666667	0.000883333	0.015566667	-1.059	3.05429	7	2
SH3BGRL2	SH3 domain binding glutamic acid-rich protein like 2	6q14.1	-1.254	0.00017	0.0033							1
SH3BP1	SH3-domain binding protein 1	22q13.1	-0.5465	0.000745	0.0083	-0.462	9.50E-05	0.00535				2
SH3BP2	SH3-domain binding protein 2	4p16.3				-0.4465	0.000645	0.0144	0.292	2.412	5	2
KMO	kynurenine 3-monooxygenase (kynurenine 3-hydroxylase)	1q42-q44	0.44	0.00636	0.043	0.337	9.00E-05	0.0063	-0.965	3.13	5	3
SH3BP5	SH3-domain binding protein 5 (BTK-associated)	3p24.3				-0.379	7.50E-05	0.00565				1
SH3BP5L	SH3-binding domain protein 5-like	1q44	-0.388	0.00821	0.0511							1
SH3D19	SH3 domain containing 19	4q31.3							-0.892	2.816	5	1
SH3GL1	SH3-domain GRB2-like 1	19p13.3	-1.171	0.00025	0.0043	-0.4855	0.00101625	0.0155875				2
SH3GL2	SH3-domain GRB2-like 2	9p22				0.332	0.00224	0.0259				1
SH3GL3	SH3-domain GRB2-like 3	15q24	-0.644	8.00E-05	0.0019				1.068	2.92333	6	2
SH3GLB1	SH3-domain GRB2-like endophilin B1	1p22	-0.645	0.00118	0.0131							1
SH3GLB2	SH3-domain GRB2-like endophilin B2	9q34	-0.466	0.00719	0.0467							1
MORF4L2	mortality factor 4 like 2	Xq22	0.44	0.00101	0.0118							1
SH3PXD2A	SH3 and PX domains 2A	10q24.33	-0.933	3.00E-04	0.0049	-0.3272	0.001305	0.01711	0.562	2.43667	6	3

TMEM138	transmembrane protein 138	11q12.2	0.44	0.00459	0.0345							1
ACVRL1	activin A receptor type II-like 1	12q13.13	0.439	0.01085	0.0617							1
SH3RF2	SH3 domain containing ring finger 2	5q32	-1.571	0	0	-0.44	0.00013	0.0073				2
SH3TC1	SH3 domain and tetratricopeptide repeats 1	4p16.1				-0.317	0.02227	0.0909				1
SH3TC2	SH3 domain and tetratricopeptide repeats 2	5q32				0.404	0.00062	0.0142				1
SH3YL1	SH3 domain containing, Ysc84-like 1 (S. cerevisiae)	2p25.3				0.3195	0.000375	0.01085				1
SHANK1	SH3 and multiple ankyrin repeat domains 1	19q13.3				-0.320333333	0.004446667	0.035833333	0.728	2.925	6	2
DCAF13	DDB1 and CUL4 associated factor 13	8q22.3	0.439	0.00094	0.0112							1
SHANK3	SH3 and multiple ankyrin repeat domains 3	22q13.3				-0.372	0.00104	0.016733333				1
SHARPIN	SHANK-associated RH domain interactor	8q24.3							0.615	2.858	5	1
SHB	Src homology 2 domain containing adaptor protein B	9p13.2	-0.348	0.0404	0.1465	-0.37775	0.000471786	0.009885714				2
SHBG	sex hormone-binding globulin	17p13-p12				-0.552	0.00029	0.0102				1
NOP2	NOP2 nucleolar protein homolog (yeast)	12p13	0.439	0.01976	0.092							1
SHC2	SHC (Src homology 2 domain containing) transforming protein 2	19p13.3				-0.4445	0.00587	0.03775				1
SHC4	SHC (Src homology 2 domain containing) family, member 4	15q21.1-q21.2				-0.352	0.00399	0.0348				1
SENP5	SUMO1/sentrin specific peptidase 5	3q29	0.439	0.01237	0.0675							1
SHCBP1L	SHC SH2-domain binding protein 1-like	1q25.3	-0.305	0.00028	0.0047							1
SHD	Src homology 2 domain containing transforming protein D	19p13.3				-0.425	0.00267	0.0283				1
SHF	Src homology 2 domain containing F	15q21.1				-0.3585	0.00739	0.0404				1
SLC27A3	solute carrier family 27 (fatty acid transporter), member 3	1q21.3	0.439	0.02348	0.1028							1
BATF	basic leucine zipper transcription factor, ATF-like	14q24.3	0.438	0.00301	0.0257							1
EXOSC8	exosome component 8	13q13.1	0.438	0.03689	0.1383							1
SHISA5	shisa homolog 5 (Xenopus laevis)	3p21.31				-0.396	0.00182	0.01935				1
SHISA6	shisa homolog 6 (Xenopus laevis)	17p12	-0.259	0.04126	0.1486							1
SHISA9	shisa homolog 9 (Xenopus laevis)	16p13.12	-0.292	0.02897	0.1178							1
SHKBP1	SH3KBP1 binding protein 1	19q13.2				-0.334	0.00237	0.0267				1
SHMT1	serine hydroxymethyltransferase 1 (soluble)	17p11.2	-1.276	4.00E-05	0.001	-0.375	0.003758	0.03228				2
SHMT2	serine hydroxymethyltransferase 2 (mitochondrial)	12q12-q14				-0.399	0.00069	0.0149				1
SHOC2	soc-2 suppressor of clear homolog (C. elegans)	10q25							-0.84	2.91875	8	1
SHOX	short stature homeobox	Xp22.33;Yp11.3				-0.511391304	0.00356913	0.026643478				1
SHOX2	short stature homeobox 2	3q25.32							-0.754	2.6475	6	1
SHPK	sedoheptulokinase	17p13				-0.46	0.000803333	0.015766667				1
SHQ1	SHQ1 homolog (S. cerevisiae)	3p13				-0.3415	0.00167	0.018175				1
SHRM	Data not found								-0.65	2.95286	7	1
UBXN4	UBX domain protein 4	2q21.3	0.438	0.00495	0.0363							1
SHROOM2	shroom family member 2	Xp22.3	-0.928	3.00E-05	8.00E-04							1
SHROOM3	shroom family member 3	4q21.1				-0.3515	0.020325	0.0788				1
SHROOM4	shroom family member 4	Xp11.22				0.313	0.00222	0.02395				1
EBNA1BP2	EBNA1 binding protein 2	1p35-p33	0.437	0.01394	0.0729							1
FAM156A	family with sequence similarity 156, member A	Xp11.22	0.437	0.03497	0.1333							1
SIAH2	siah E3 ubiquitin protein ligase 2	3q25	-0.539	0.00115	0.0129							1
SIAH3	siah E3 ubiquitin protein ligase family member 3	13q14.13				0.312	0.02384	0.0946				1
SIDT1	SID1 transmembrane family, member 1	3q13.2				0.317	0.00071	0.0151	-0.619	2.606	5	2
MICALL2	MICAL-like 2	7p22.3	0.437	0.00723	0.0469							1
SIGLEC14	sialic acid binding Ig-like lectin 14	19q13.4				1.519	0.000795	0.0135				1
SIGLEC15	sialic acid binding Ig-like lectin 15	18q12.3				-0.34	0.023695	0.09315				1
SIGLEC16	sialic acid binding Ig-like lectin 16 (gene/pseudogene)	19q13.33				-0.489	0.00048	0.0126				1
SIL1	SIL1 homolog, endoplasmic reticulum chaperone (S. cerevisiae)	5q31				-0.3625	0.002002778	0.018338889				1
SIM1	single-minded homolog 1 (Drosophila)	6q16.3-q21							0.749	2.81143	7	1
SIM2	single-minded homolog 2 (Drosophila)	21q22.13	-1.083	6.00E-05	0.0014	-0.365625	0.00052875	0.012375				2
SIN3A	SIN3 transcription regulator homolog A (yeast)	15q24.2							-0.715	2.73	5	1
SIN3B	SIN3 transcription regulator homolog B (yeast)	19p13.11				-0.401333333	0.006231111	0.038633333				1
SIP1	Data not found								-0.802	2.82143	7	1
SIPA1L1	signal-induced proliferation-associated 1 like 1	14q24.2				-0.319	0.00412	0.0354				1
SIPA1L2	signal-induced proliferation-associated 1 like 2	1q42.2				0.337625	0.00138375	0.0165875				1
NCBP1	nuclear cap binding protein subunit 1, 80kDa	9q34.1	0.437	0.0096	0.0568				-0.22	2.643	5	2
SIRPA	signal-regulatory protein alpha	20p13							0.7	2.46167	6	1
SIRPB1	signal-regulatory protein beta 1	20p13				0.4352	0.008498	0.04666				1
SIRPD	signal-regulatory protein delta	20p13				0.41	0.00088	0.0166				1
RFX5	regulatory factor X, 5 (influences HLA class II expression)	1q21	0.437	0.02835	0.1162							1
SIRT2	sirtuin 2	19q13	-0.314	0.00417	0.0322	-0.328	0.00198	0.0245				2
SIRT6	sirtuin 6	19p13.3				-0.505	0.0017	0.0218				1
SIRT7	sirtuin 7	17q25	-0.478	0.00379	0.0301	-0.376	0.01188	0.0631				2
SIT1	signaling threshold regulating transmembrane adaptor 1	9p13-p12	-0.382	0.01129	0.0635							1
ZNF226	zinc finger protein 226	19q13.2	0.437	0.00592	0.041							1
SIX4	SIX homeobox 4	14q23				-0.313	6.00E-04	0.014				1
CD83	CD83 molecule	6p23	0.436	0.00367	0.0295				-0.726	2.42	5	2
SKAP2	src kinase associated phosphoprotein 2	7p15.2				0.35025	0.0005425	0.0123				1
SKI	v-ski sarcoma viral oncogene homolog (avian)	1p36.33				-0.3608	0.001372	0.01776				1

SKIDA1	Data not found		-0.361	0.01793	0.0862							1
LAMTOR4	Data not found		0.436	0.04684	0.1606							1
SKIV2L2	superkiller viralicidic activity 2-like 2 (S. cerevisiae)	5q11.2				-0.39	0.00551	0.03295				1
SKP1	S-phase kinase-associated protein 1	5q31	-0.328	0.02206	0.0989	-0.338	0.003706667	0.029466667				2
SKP2	S-phase kinase-associated protein 2, E3 ubiquitin protein ligase	5p13				0.378166667	0.007468333	0.047933333				1
SLA	Src-like-adaptor	8q24				0.3932	0.024374	0.09494				1
SLAIN1	SLAIN motif family, member 1	13q22.3				0.377	0.00081	0.016				1
SLAIN2	SLAIN motif family, member 2	4p11	-0.671	1.00E-05	3.00E-04							1
SLAMF7	SLAM family member 7	1q23.1-q24.1							-1.008	2.58667	6	1
SLBP	stem-loop binding protein	4p16.3				-0.361	0.002975	0.029825				1
SLC10A2	solute carrier family 10 (sodium/bile acid cotransporter family), member 2	13q33							-0.7	2.535	6	1
SLC10A3	solute carrier family 10 (sodium/bile acid cotransporter family), member 3	Xq28	-0.217	0.02998	0.1206							1
SLC10A6	solute carrier family 10 (sodium/bile acid cotransporter family), member 6	4q21.3	-2.009	0	1.00E-04	-0.370333333	0.001633333	0.022				2
PCDH12	protocadherin 12	5q31	0.436	0.00205	0.0194							1
SLC11A2	solute carrier family 11 (proton-coupled divalent metal ion transporters), men	12q13				-0.408	5.00E-05	0.0053				1
SLC12A1	solute carrier family 12 (sodium/potassium/chloride transporters), member 1	15q15-q21.1							-0.935	3.1825	8	1
SLC41A3	solute carrier family 41, member 3	3q21.2	0.436	0.01681	0.0826							1
SLC12A3	solute carrier family 12 (sodium/chloride transporters), member 3	16q13				-0.385	0.011816667	0.0529				1
SLC12A4	solute carrier family 12 (potassium/chloride transporters), member 4	16q22.1				-0.519333333	0.00022	0.009066667				1
SLC12A6	solute carrier family 12 (potassium/chloride transporters), member 6	15q13	-1.551	0	1.00E-04							1
WBP11	WW domain binding protein 11	12p12.3	0.436	0.00175	0.0174				-0.795	2.625	6	2
CFL1	cofilin 1 (non-muscle)	11q13	0.435	0.00089	0.0108							1
IFIT5	interferon-induced protein with tetratricopeptide repeats 5	10q23.31	0.435	0.03505	0.1334							1
SLC13A1	solute carrier family 13 (sodium/sulfate symporters), member 1	7q31-q32				0.3825	0.012278333	0.054266667				1
MED17	mediator complex subunit 17	11q14	0.435	0.00069	0.0091							1
SLC13A3	solute carrier family 13 (sodium-dependent dicarboxylate transporter), memb	20q12-q13.1				0.371	0.000663333	0.011733333				1
SLC13A4	solute carrier family 13 (sodium/sulfate symporters), member 4	7q33	-1.775	0	0	-0.402333333	0.00624	0.034233333				2
SLC13A5	solute carrier family 13 (sodium-dependent citrate transporter), member 5	17p13.1				-0.44225	0.00027	0.007525				1
SLC14A2	solute carrier family 14 (urea transporter), member 2	18q12.1-q21.1				-0.344	0.00102	0.0178				1
SLC15A2	solute carrier family 15 (H+/peptide transporter), member 2	3q13.33				0.366	0.00017	0.008				1
SLC15A3	solute carrier family 15, member 3	11q12.2				-0.309	3.00E-05	0.0042				1
SLC15A4	solute carrier family 15, member 4	12q24.32				-0.378	0.000475	0.0122				1
SLC15A5	solute carrier family 15, member 5	12p12.3	-0.351	0.00483	0.0356							1
ANKRD13B	ankyrin repeat domain 13B	17q11.2	0.434	0.00424	0.0326							1
LUC7L3	LUC7-like 3 (S. cerevisiae)	17q21.33	0.434	0.01376	0.0723							1
SLC16A11	solute carrier family 16, member 11 (monocarboxylic acid transporter 11)	17p13.1	-0.253	0.03276	0.1277	-0.555	0.00035	0.0111				2
SLC16A13	solute carrier family 16, member 13 (monocarboxylic acid transporter 13)	17p13.1				-0.3765	0.009805	0.05355				1
SLC16A14	solute carrier family 16, member 14 (monocarboxylic acid transporter 14)	2q36.3	-0.726	0.03136	0.1243							1
RAPGEF2	Rap guanine nucleotide exchange factor (GEF) 2	4q32.1	0.434	0.02369	0.1033							1
TRRAP	transformation/transcription domain-associated protein	7q21.2-q22.1	0.434	0.03711	0.1388							1
SLC16A6	solute carrier family 16, member 6 (monocarboxylic acid transporter 7)	17q24.2	-2.493	0	0							1
SLC16A7	solute carrier family 16, member 7 (monocarboxylic acid transporter 2)	12q13							-0.804	2.876	5	1
SLC16A8	solute carrier family 16, member 8 (monocarboxylic acid transporter 3)	22q12.3-q13.2				-0.558	0.0015	0.0213				1
SLC16A9	solute carrier family 16, member 9 (monocarboxylic acid transporter 9)	10q21.2	-1.871	6.00E-05	0.0015							1
SLC17A1	solute carrier family 17 (sodium phosphate), member 1	6p22.2							-0.774	2.98	7	1
SLC17A3	solute carrier family 17 (sodium phosphate), member 3	6p21.3				0.462	0.0012	0.0192				1
TUBGCP3	tubulin, gamma complex associated protein 3	13q34	0.434	0.0255	0.1086							1
VWA9	Data not found		0.434	0.00943	0.0562							1
SLC17A6	solute carrier family 17 (sodium-dependent inorganic phosphate cotransporte	11p14.3	-0.177	0.04481	0.1564							1
SLC17A7	solute carrier family 17 (sodium-dependent inorganic phosphate cotransporte	19q13				-0.384	0.00043	0.0121				1
BAZ1B	bromodomain adjacent to zinc finger domain, 1B	7q11.23	0.433	0.00322	0.0269							1
SLC18A1	solute carrier family 18 (vesicular monoamine), member 1	8p21.3							-0.796	2.564	5	1
BRMS1	breast cancer metastasis suppressor 1	11q13-q13.2	0.433	0.00416	0.0322							1
SLC19A2	solute carrier family 19 (thiamine transporter), member 2	1q23.3				0.36625	0.0003925	0.011175				1
PRPF4	PRP4 pre-mRNA processing factor 4 homolog (yeast)	9q31-q33	0.433	0.02887	0.1175							1
SLC1A1	solute carrier family 1 (neuronal/epithelial high affinity glutamate transporter	9p24				-0.407789474	0.001564737	0.018710526				1
SLC1A2	solute carrier family 1 (glial high affinity glutamate transporter), member 2	11p13-p12				0.3495	0.000945	0.01475				1
SLC1A3	solute carrier family 1 (glial high affinity glutamate transporter), member 3	5p13				0.408222222	0.010066667	0.053022222	-0.941	3.35	8	2
SLC1A6	solute carrier family 1 (high affinity aspartate/glutamate transporter), membe	19p13.12	-0.189	0.04517	0.1573	-0.4166	0.00145	0.01804				2
ALPI	alkaline phosphatase, intestinal	2q37.1	0.432	0.03101	0.1234							1
DCHS1	dachsous 1 (Drosophila)	11p15.4	0.432	0.0163	0.0809							1
SLC20A2	solute carrier family 20 (phosphate transporter), member 2	8p11.21	-0.537	0.00865	0.0529	-0.318333333	0.022076667	0.087				2
SLC22A10	In multiple Geneids								-0.884	3.60778	9	1
SLC22A13	solute carrier family 22 (organic anion transporter), member 13	3p21.3	-0.306	0.00899	0.0543							1
SLC22A14	solute carrier family 22, member 14	3p21.3	-0.244	0.01784	0.086							1
SLC22A15	solute carrier family 22, member 15	1p13.1	-0.571	0.00578	0.0404							1
SLC22A17	solute carrier family 22, member 17	14q11.2				-0.343	0.00178	0.0232				1
SLC22A18	solute carrier family 22, member 18	11p15.5							0.5175	2.76936	7	1
SLC22A18AS	solute carrier family 22 (organic cation transporter), member 18 antisense	11p15.5							0.712	2.982	5	1

SLC22A20	In multiple Geneids		-0.383	0.00523	0.0376	-0.336	0.00065	0.0145				2
SLC22A23	solute carrier family 22, member 23	6p25.2	-0.499	0.00884	0.0536	-0.401444444	0.002907778	0.0255				2
SLC22A25	solute carrier family 22, member 25	11q12.3	-0.266	0.01961	0.0915							1
SLC22A3	solute carrier family 22 (extraneuronal monoamine transporter), member 3	6q25.3				1.158	0	5.00E-04				1
DYRK2	dual-specificity tyrosine-(Y)-phosphorylation regulated kinase 2	12q15	0.432	0.00024	0.0042							1
PEX5	peroxisomal biogenesis factor 5	12p13.31	0.432	0.02233	0.0995							1
SLC22A8	solute carrier family 22 (organic anion transporter), member 8	11q11				-0.353	0.00069	0.0149				1
SLC22A9	solute carrier family 22 (organic anion transporter), member 9	11q13.1							-0.804	3.23	6	1
SLC23A1	solute carrier family 23 (nucleobase transporters), member 1	5q31.2				-0.39175	0.0032275	0.02815				1
SLC24A2	solute carrier family 24 (sodium/potassium/calcium exchanger), member 2	9p22.1							-0.705	2.82429	7	1
SLC24A3	In multiple Geneids					0.36105	0.0094955	0.05136				1
SLC24A4	In multiple Geneids					-0.3498	0.002476	0.02154				1
SLC24A5	In multiple Geneids								-1.08	4.11333	6	1
SLC24A6	solute carrier family 24 (sodium/lithium/calcium exchanger), member 6	12q24.13				-0.415	0.000105	0.00675				1
SLC25A12	solute carrier family 25 (aspartate/glutamate carrier), member 12	2q24	-0.736	0.0205	0.0943				0.741	2.704	5	2
PLEKHA8	pleckstrin homology domain containing, family A (phosphoinositide binding sp	7p21-p11.2	0.432	0.00327	0.0273	0.336	0.00027	0.0099	-0.87	2.68667	6	3
SLC25A14	solute carrier family 25 (mitochondrial carrier, brain), member 14	Xq24				-0.31	0.00061	0.0141				1
SRC	v-src sarcoma (Schmidt-Ruppin A-2) viral oncogene homolog (avian)	20q12-q13	0.432	0.00183	0.0179							1
SLC25A16	solute carrier family 25 (mitochondrial carrier; Graves disease autoantigen), m	10q21.3				-0.3456	0.002584	0.02086				1
SLC25A17	solute carrier family 25 (mitochondrial carrier; peroxisomal membrane protei	22q13.2				-0.370142857	0.001522857	0.017128571				1
SLC25A18	In multiple Geneids								-0.706	2.82571	7	1
STK38L	serine/threonine kinase 38 like	12p11.23	0.432	0.03558	0.1348	0.319	0.005945	0.043				2
SLC25A20	solute carrier family 25 (carnitine/acylcarnitine translocase), member 20	3p21.31				-0.38025	0.004605	0.03255				1
SLC25A21	solute carrier family 25 (mitochondrial oxoadipate carrier), member 21	14q11.2				0.395	5.00E-05	0.0052	-0.648	2.73	5	2
TBC1D31	Data not found		0.432	0.00564	0.0396							1
SLC25A23	solute carrier family 25 (mitochondrial carrier; phosphate carrier), member 23	19p13.3	-1.225	0	2.00E-04	-0.4518	0.001866	0.01866				2
ACTL6A	actin-like 6A	3q26.33	0.431	0.02689	0.1123							1
SLC25A25	solute carrier family 25 (mitochondrial carrier; phosphate carrier), member 25	9q34.11							0.627	2.76571	7	1
SLC25A26	solute carrier family 25 (S-adenosylmethionine carrier), member 26	3p14.1							-0.848	3.21833	6	1
SLC25A27	solute carrier family 25, member 27	6p12.3				0.333	0.00518	0.04				1
SLC25A28	solute carrier family 25 (mitochondrial iron transporter), member 28	10q24.2	-0.428	0.00267	0.0235	-0.3465	0.00014	0.0075				2
SLC25A29	solute carrier family 25 (mitochondrial carnitine/acylcarnitine carrier), membe	14q32.2	-0.233	0.03509	0.1335							1
SLC25A3	solute carrier family 25 (mitochondrial carrier; phosphate carrier), member 3	12q23	-0.402	0.00843	0.052							1
SLC25A31	solute carrier family 25 (mitochondrial carrier; adenine nucleotide translocato	4q28.1	-0.237	0.01456	0.075							1
C11orf48	chromosome 11 open reading frame 48	11q12.3	0.431	0.0016	0.0163							1
CD320	CD320 molecule	19p13.3-p13.2	0.431	0.02657	0.1117							1
SLC25A34	solute carrier family 25, member 34	1p36.21				-0.523	0.00034	0.011				1
SLC25A35	solute carrier family 25, member 35	17p13.1				-0.40725	0.0040375	0.0295				1
CTBP2	C-terminal binding protein 2	10q26.13	0.431	0.00837	0.0517							1
P4HA3	prolyl 4-hydroxylase, alpha polypeptide III	11q13.4	0.431	0.0054	0.0385							1
SLC25A39	solute carrier family 25, member 39	17q12	-0.299	0.03558	0.1348							1
SLC25A4	solute carrier family 25 (mitochondrial carrier; adenine nucleotide translocato	4q35							-0.866	3.066	5	1
SLC25A41	solute carrier family 25, member 41	19p13.3	-0.255	0.01549	0.0782	-0.549	0.00052	0.0129				2
SLC25A42	solute carrier family 25, member 42	19p13.11				-0.434375	0.00188125	0.019075				1
SLC25A43	solute carrier family 25, member 43	Xq24	-0.702	0.00181	0.0178							1
RAPGEF1	Rap guanine nucleotide exchange factor (GEF) 1	9q34.3	0.431	0.02014	0.0931							1
SLC25A45	solute carrier family 25, member 45	11q13.1				-0.332	0.02173	0.0896				1
SLC25A5	solute carrier family 25 (mitochondrial carrier; adenine nucleotide translocato	Xq24	-0.396	3.00E-04	0.005							1
SLC25A51	solute carrier family 25, member 51	9p13.3-p12	-0.396	0.00973	0.0574							1
SLC25A6	solute carrier family 25 (mitochondrial carrier; adenine nucleotide translocato	Xp22.32 and Yp11.3				-0.58025	0.0003475	0.010025				1
SLC26A10	solute carrier family 26, member 10	12q13				-0.361	0.00128	0.0198				1
SLC26A2	solute carrier family 26 (sulfate transporter), member 2	5q31-q34	-1.449	0.00044	0.0065							1
SLC26A3	solute carrier family 26, member 3	7q31							-1.029	2.944	5	1
TRPV2	transient receptor potential cation channel, subfamily V, member 2	17p11.2	0.431	0.02796	0.1152							1
SLC26A7	In multiple Geneids					0.3425	0.0001925	0.0069625	-1.06	3.00833	6	2
SLC27A1	In multiple Geneids					-0.46575	0.000805	0.0143	0.572	2.592	5	2
TTC21B	tetratricopeptide repeat domain 21B	2q24.3	0.431	0.01019	0.0593							1
HSP90B3P	heat shock protein 90kDa beta (Grp94), member 3, pseudogene	1p22.1	0.43	0.00671	0.0446							1
SLC27A4	solute carrier family 27 (fatty acid transporter), member 4	9q34.11	-0.575	0.0434	0.1532							1
SLC27A6	In multiple Geneids		-1.051	3.00E-05	9.00E-04	-0.372	0	7.00E-04				2
SLC28A3	solute carrier family 28 (sodium-coupled nucleoside transporter), member 3	9q22.2				-0.3365	0.01038	0.0555				1
TEX10	testis expressed 10	9q31.1	0.43	0.03702	0.1386							1
C18orf54	chromosome 18 open reading frame 54	18q21.2	0.429	0.04958	0.1663				-0.939	3.42143	7	2
DDX12P	DEAD/H (Asp-Glu-Ala-Asp/His) box polypeptide 12, pseudogene	12p13.31	0.429	0.03912	0.1434							1
GMFB	glia maturation factor, beta	14q22.2	0.429	0.01827	0.0874							1
SLC2A11	solute carrier family 2 (facilitated glucose transporter), member 11	22q11.23				-0.408	3.40E-05	0.00358				1
SLC2A12	In multiple Geneids								-0.948	2.418	5	1
INTS3	integrator complex subunit 3	1q21.3	0.429	0.01291	0.0695							1
ZNF487	Data not found		0.429	0.04801	0.163							1

SLC2A2	solute carrier family 2 (facilitated glucose transporter), member 2	3q26.1-q26.2				0.351	0.002045	0.02475	-0.71	2.616	5	2
ASB1	ankyrin repeat and SOCS box containing 1	2q37	0.428	0.00926	0.0554							1
SLC2A4	solute carrier family 2 (facilitated glucose transporter), member 4	17p13				-0.32	0.01803	0.0802				1
SLC2A4RG	SLC2A4 regulator	20q13.33							0.696333333	2.59078	5.666666667	1
SLC2A5	solute carrier family 2 (facilitated glucose/fructose transporter), member 5	1p36.2	-0.311	0.0256	0.109	-0.42425	0.001145	0.01685				2
CEP57	centrosomal protein 57kDa	11q21	0.428	0.01445	0.0746							1
SLC2A7	In multiple Geneids					-0.392333333	0.002753333	0.0277				1
SLC2A9	solute carrier family 2 (facilitated glucose transporter), member 9	4p16.1							-0.802	3.04167	6	1
COPG1	In multiple Geneids		0.428	0.00339	0.0279							1
SLC30A2	solute carrier family 30 (zinc transporter), member 2	1p35.3				-0.458	0.00562	0.0417				1
SLC30A4	solute carrier family 30 (zinc transporter), member 4	15q21.1	-1.126	5.00E-05	0.0012							1
SLC30A5	solute carrier family 30 (zinc transporter), member 5	5q12.1				-0.354	0.00219	0.0256				1
SLC30A6	solute carrier family 30 (zinc transporter), member 6	2p22.3				-0.322	0.00045	0.0124				1
NAA16	N(alpha)-acetyltransferase 16, NatA auxiliary subunit	13q14.11	0.428	0.01872	0.0889							1
SLC30A8	solute carrier family 30 (zinc transporter), member 8	8q24.11				0.499461538	0.004293846	0.034423077				1
SLC31A1	solute carrier family 31 (copper transporters), member 1	9q32							-0.967	2.92167	6	1
SLC31A2	solute carrier family 31 (copper transporters), member 2	9q32	-0.616	0.00045	0.0067							1
SLC32A1	In multiple Geneids		-0.21	0.02037	0.0939				1.226	3.108335	7.5	2
RIMKLA	ribosomal modification protein rimK-like family member A	1p34.2	0.428	0.00414	0.0321							1
SLC34A1	solute carrier family 34 (sodium phosphate), member 1	5q35				-0.41275	0.0008775	0.01475				1
RSRC1	arginine/serine-rich coiled-coil 1	3q25.32	0.428	0.02496	0.107	0.311285714	0.002118571	0.023842857	-0.754	2.6475	6	3
SLC34A3	solute carrier family 34 (sodium phosphate), member 3	9q34	-0.475	0.00083	0.0103							1
SGTB	small glutamine-rich tetratricopeptide repeat (TPR)-containing, beta	5q12.3	0.428	0.01905	0.0899							1
C3orf17	chromosome 3 open reading frame 17	3q13.2	0.427	0.01568	0.0789							1
SLC35A4	solute carrier family 35, member A4	5q31.3	-0.799	0.00014	0.0028	-0.377	6.00E-04	0.0139				2
GHDC	GH3 domain containing	17q21.2	0.427	2.00E-04	0.0036							1
GK3P	glycerol kinase 3 pseudogene	4q32.1	0.427	0.01714	0.0837							1
SLC35C1	In multiple Geneids		-0.813	0.00085	0.0105							1
NUPL1	nucleoporin like 1	13q12.13	0.427	0.00652	0.0436							1
SLC35E1	solute carrier family 35, member E1	19p13.11				-0.429666667	0.002843333	0.023033333				1
SLC35E2	solute carrier family 35, member E2	1p36.33				-0.416	0.00232	0.0264				1
SLC35E3	solute carrier family 35, member E3	12q15				-0.613	0.00652	0.0452				1
SLC35E4	solute carrier family 35, member E4	22q12.2				-0.486	0.00132	0.02				1
SLC35F3	solute carrier family 35, member F3	1q42.2				0.315	0.002943333	0.028266667				1
SLC35F6	Data not found		-0.514	0.00276	0.0241							1
PSMD4	proteasome (prosome, macropain) 26S subunit, non-ATPase, 4	1q21.3	0.427	0.03619	0.1366							1
SLC35G5	solute carrier family 35, member G5	8p23.1	-0.416	0.01593	0.0797							1
RBL1	retinoblastoma-like 1 (p107)	20q11.2	0.427	0.04303	0.1525							1
SLC36A2	solute carrier family 36 (proton/amino acid symporter), member 2	5q33.1				-0.384333333	7.00E-04	0.0125				1
SLC36A3	solute carrier family 36 (proton/amino acid symporter), member 3	5q33.1				-0.316	0.00031	0.0105				1
SLC37A1	solute carrier family 37 (glycerol-3-phosphate transporter), member 1	21q22.3				-0.3825	0.001015	0.0153				1
SLC37A2	solute carrier family 37 (glycerol-3-phosphate transporter), member 2	11q24.2	-2.015	2.00E-05	6.00E-04							1
ABCC6P2	In multiple Geneids		0.426	0.03209	0.126							1
BOLA3	bolA homolog 3 (E. coli)	2p13.1	0.426	0.02468	0.1062							1
FKBP9L	FK506 binding protein 9-like	7p11.1	0.426	0.00969	0.0572							1
MARVELD1	MARVEL domain containing 1	10q24.2	0.426	0.01311	0.07							1
SLC38A2	solute carrier family 38, member 2	12q	-0.632	0.00209	0.0197							1
SLC38A3	solute carrier family 38, member 3	3p21.3				-0.379333333	0.000536667	0.011666667				1
SLC38A4	solute carrier family 38, member 4	12q13							-0.702	2.738	5	1
RARRES2	retinoic acid receptor responder (tazarotene induced) 2	7q36.1	0.426	0.02284	0.1009							1
RBM15	RNA binding motif protein 15	1p13	0.426	0.01267	0.0686							1
SLC38A8	solute carrier family 38, member 8	16q23.3				-0.349	0.0056	0.0417				1
SLC38A9	solute carrier family 38, member 9	5q11.2				-0.321	0.005855	0.0355				1
RNF26	ring finger protein 26	11q23	0.426	0.02098	0.0958							1
STAT5A	signal transducer and activator of transcription 5A	17q11.2	0.426	0.01914	0.0902							1
SLC39A11	solute carrier family 39 (metal ion transporter), member 11	17q21.31				-0.341285714	0.00497	0.031042857				1
ZC3HAV1L	zinc finger CCCH-type, antiviral 1-like	7q34	0.426	0.01027	0.0596							1
ARCN1	archain 1	11q23.3	0.425	0.00062	0.0084							1
SLC39A2	solute carrier family 39 (zinc transporter), member 2	14q11.2	-2.296	0	0							1
SLC39A3	solute carrier family 39 (zinc transporter), member 3	19p13.3				-0.4215	0.002545	0.02315				1
CREB3	cAMP responsive element binding protein 3	9p13.3	0.425	0.01642	0.0812							1
PNMA1	paraneoplastic Ma antigen 1	14q24.3	0.425	0.00181	0.0178							1
USB1	Data not found		0.425	0.00147	0.0154							1
DCLRE1A	DNA cross-link repair 1A	10q25.1	0.424	0.00521	0.0375				-0.677	2.51571	7	2
LIMS1	LIM and senescent cell antigen-like domains 1	2q12.3	0.424	0.00115	0.0129							1
MRPS30	mitochondrial ribosomal protein S30	5q11	0.424	0.00865	0.0529	0.333	0.02338	0.0935	-0.851	2.784	5	3
BLOC1S5	Data not found		0.423	0.01906	0.0899							1
CANX	calnexin	5q35	0.423	0.0034	0.028							1
HNRNPA1	heterogeneous nuclear ribonucleoprotein A1	12q13.1	0.423	0.02752	0.11205							1

PPIL4	peptidylprolyl isomerase (cyclophilin)-like 4	6q25.1	0.423	0.01629	0.0808							1
SCARB2	scavenger receptor class B, member 2	4q21.1	0.423	0.0446	0.156							1
SLC44A2	solute carrier family 44, member 2	19p13.1	-0.661	0.00213	0.02	-0.479714286	0.004524286	0.033628571				2
TFCP2	transcription factor CP2	12q13	0.423	0.00954	0.0566							1
SLC45A1	solute carrier family 45, member 1	1p36.23				-0.46475	0.0006375	0.0126				1
SLC45A2	solute carrier family 45, member 2	5p13.2				0.383666667	0.008688333	0.048583333				1
UNC50	unc-50 homolog (C. elegans)	2q11.2	0.423	0.0259	0.1098							1
RBM34	RNA binding motif protein 34	1q42.3	0.422	0.02008	0.0929							1
TPCN2	two pore segment channel 2	11q13.3	0.422	0.00312	0.0263							1
SLC46A2	solute carrier family 46, member 2	9q32	-0.608	1.00E-05	5.00E-04							1
DDX10	DEAD (Asp-Glu-Ala-Asp) box polypeptide 10	11q22-q23	0.421	0.00947	0.0563	0.349	0.00069	0.0149				2
SLC47A1	solute carrier family 47, member 1	17p11.2	-0.64	0.00959	0.0567	-0.3555	0.00036	0.0094				2
SLC47A2	solute carrier family 47, member 2	17p11.2	-0.419	0.00509	0.037	-0.3315	0.011585	0.05915				2
SLC48A1	solute carrier family 48 (heme transporter), member 1	12q13.11	-0.788	0.00039	0.0059							1
SLC4A1	solute carrier family 4, anion exchanger, member 1 (erythrocyte membrane p	17q21-q22	-0.213	0.02879	0.1174	-0.346	0.00638	0.0447				2
SLC4A10	solute carrier family 4, sodium bicarbonate transporter, member 10	2q23-q24				0.336444444	0.000416667	0.009577778				1
SLC4A1AP	solute carrier family 4 (anion exchanger), member 1, adaptor protein	2p23.3							-0.646	2.59333	6	1
SLC4A2	solute carrier family 4, anion exchanger, member 2 (erythrocyte membrane p	7q36.1				-0.301	0.00208	0.025				1
MICAL3	microtubule associated monoxygenase, calponin and LIM domain containing 3	22q11.21	0.421	0.00553	0.0391							1
MTRF1	mitochondrial translational release factor 1	13q14.1-q14.3	0.421	0.02638	0.1111							1
PIGK	phosphatidylinositol glycan anchor biosynthesis, class K	1p31.1	0.421	0.00718	0.0467				-0.947	2.384	5	2
SLC4A9	solute carrier family 4, sodium bicarbonate cotransporter, member 9	5q31				-0.5185	0.000285	0.0095				1
ATRX	alpha thalassemia/mental retardation syndrome X-linked	Xq21.1	0.4205	0.01277	0.0606							1
HNRNPH3	heterogeneous nuclear ribonucleoprotein H3 (2H9)	10q22	0.42	0.00993	0.0581							1
TAF5L	TAF5-like RNA polymerase II, p300/CBP-associated factor (PCAF)-associated fa	1q42.13	0.42	0.00425	0.0326	0.309	0.00904	0.0542				2
SLC5A1	solute carrier family 5 (sodium/glucose cotransporter), member 1	22q12.3				-0.396363636	0.000733636	0.012663636				1
SLC5A10	solute carrier family 5 (sodium/glucose cotransporter), member 10	17p11.2	-0.332	0.00817	0.04395	-0.3975	0.0047375	0.0316				2
TRAPPC6A	In multiple Geneids		0.42	0.01901	0.0898							1
SLC5A3	solute carrier family 5 (sodium/myo-inositol cotransporter), member 3	21q22.12							-0.794	2.73	5	1
SLC5A4	solute carrier family 5 (low affinity glucose cotransporter), member 4	22q12.2-q12.3				-0.377285714	0.000441429	0.009485714				1
SLC5A5	solute carrier family 5 (sodium iodide symporter), member 5	19p13.2-p12				-0.4355	0.005925	0.04035				1
ARID3B	AT rich interactive domain 3B (BRIGHT-like)	15q24	0.419	0.04394	0.1544							1
SLC5A7	solute carrier family 5 (choline transporter), member 7	2q12				0.351	8.00E-05	0.006				1
KIF4B	In multiple Geneids		0.419	0.01816	0.087							1
SLC6A1	solute carrier family 6 (neurotransmitter transporter, GABA), member 1	3p25.3	-2.053	0	0	-0.304	0.0066	0.0451				2
SLC6A11	solute carrier family 6 (neurotransmitter transporter, GABA), member 11	3p25.3	-1.41	0.00023	0.0041							1
SLC4A8	solute carrier family 4, sodium bicarbonate cotransporter, member 8	12q13.13	0.419	0.01536	0.0778							1
TMEM258	Data not found		0.419	0.01426	0.074							1
SLC6A15	In multiple Geneids		-1.676	0	0				0.781	2.99222	9	2
SLC6A16	In multiple Geneids					-0.388	0.00398	0.0347				1
SLC6A17	solute carrier family 6, member 17	1p13.3	-0.374	0.01017	0.0592	-0.317	0.00021	0.0089				2
CATSPER2	cation channel, sperm associated 2	15q15.3	0.418	0.04718	0.1613							1
SLC6A2	solute carrier family 6 (neurotransmitter transporter, noradrenalin), member	16q12.2				-0.318	0.01925	0.0833				1
MED20	mediator complex subunit 20	6p21.1	0.418	0.04846	0.164							1
SLC6A3	solute carrier family 6 (neurotransmitter transporter, dopamine), member 3	5p15.3				0.3	0.00919	0.0546				1
SLC6A4	solute carrier family 6 (neurotransmitter transporter, serotonin), member 4	17q11.2	-1.464	5.00E-05	0.0013							1
SLC6A5	solute carrier family 6 (neurotransmitter transporter, glycine), member 5	11p15.1	-0.358	0.00061	0.0083							1
MRE11A	MRE11 meiotic recombination 11 homolog A (S. cerevisiae)	11q21	0.418	0.00674	0.0447							1
SLC6A7	solute carrier family 6 (neurotransmitter transporter, L-proline), member 7	5q32				-0.321	0.001723333	0.0197				1
SLC6A8	solute carrier family 6 (neurotransmitter transporter, creatine), member 8	Xq28							0.574	2.82714	7	1
SLC6A9	solute carrier family 6 (neurotransmitter transporter, glycine), member 9	1p33	-1.374	0	1.00E-04							1
SLC7A1	solute carrier family 7 (cationic amino acid transporter, y+ system), member 1	13q12-q14	-1.201	0.00049	0.0071							1
TFE3	transcription factor binding to IGHM enhancer 3	Xp11.22	0.418	0.00925	0.0554							1
SLC7A14	solute carrier family 7 (orphan transporter), member 14	3q26.2				0.335	0.00171	0.0227				1
SLC7A2	solute carrier family 7 (cationic amino acid transporter, y+ system), member 2	8p22-p21.3							-0.79	2.89857	7	1
SLC7A4	solute carrier family 7 (orphan transporter), member 4	22q11.21	-1.004	0	1.00E-04							1
SLC7A5	solute carrier family 7 (amino acid transporter light chain, L system), member	16q24.3	-0.941	0.00828	0.0513	-0.371666667	0.00096	0.016433333				2
TPR	translocated promoter region, nuclear basket protein	1q25	0.418	0.00134	0.0144							1
SLC7A6OS	solute carrier family 7, member 6 opposite strand	16q22.1				-0.328	0.00188	0.0238				1
TRIM37	tripartite motif containing 37	17q23.2	0.418	0.00202	0.0193							1
SLC7A8	solute carrier family 7 (amino acid transporter light chain, L system), member	14q11.2	-1.442	5.00E-05	0.0013	-0.361166667	0.00078	0.01365				2
ZNF600	zinc finger protein 600	19q13.41	0.418	0.02872	0.1173							1
SLC8A1	solute carrier family 8 (sodium/calcium exchanger), member 1	2p23-p22				0.3505	0.001858125	0.02035625	-0.933	3.464	5	2
SLC8A2	solute carrier family 8 (sodium/calcium exchanger), member 2	19q13.3				-0.332	0.001685	0.02255				1
SLC8A3	solute carrier family 8 (sodium/calcium exchanger), member 3	14q24.1							-1.124	4.566	5	1
SLC9A1	solute carrier family 9, subfamily A (NHE1, cation proton antiporter 1), memb	1p36.1-p35				-0.436	0.00874	0.044869231				1
SLC9A11	Data not found					0.528	5.00E-05	0.00485	-0.777	2.83333	6	2
SLC9A2	solute carrier family 9, subfamily A (NHE2, cation proton antiporter 2), memb	2q11.2				0.375	0.000486667	0.011033333				1
SLC9A3R1	solute carrier family 9, subfamily A (NHE3, cation proton antiporter 3), memb	17q25.1	-1.168	0	1.00E-04	-0.375	0.000595	0.01385				2

SLC9A4	In multiple Geneids					0.314	0.002	0.0213	-0.919	3.025	6	2
SLC9A5	solute carrier family 9, subfamily A (NHE5, cation proton antiporter 5), membe	16q22.1				-0.346333333	0.006666667	0.041833333				1
SLC9A6	solute carrier family 9, subfamily A (NHE6, cation proton antiporter 6), membe	Xq26.3	-0.472	0.00225	0.0208							1
SLC9A7	solute carrier family 9, subfamily A (NHE7, cation proton antiporter 7), membe	Xp11.3				0.309	0.00185	0.0236				1
SLC9A8	solute carrier family 9, subfamily A (NHE8, cation proton antiporter 8), membe	20q13.13	-0.378	0.04751	0.162							1
SLC9A9	In multiple Geneids					0.351347826	0.004305217	0.031647826	-0.903	3.11667	6	2
SLC9B1	solute carrier family 9, subfamily B (NHA1, cation proton antiporter 1), memb	4q24	-1.1845	0.001015	0.0101							1
SLCO1A2	solute carrier organic anion transporter family, member 1A2	12p12				0.3444	0.002842	0.02542	-0.943	3.290953333	6.333333333	2
SLCO1B1	solute carrier organic anion transporter family, member 1B1	12p				0.34475	0.00115	0.01655	-0.834	2.556	5	2
ATP6V0B	ATPase, H+ transporting, lysosomal 21kDa, V0 subunit b	1p32.3	0.417	0.01054	0.0608							1
NDUFS6	NADH dehydrogenase (ubiquinone) Fe-S protein 6, 13kDa (NADH-coenzyme Q	5p15.33	0.417	0.03941	0.1441							1
SLCO1C1	solute carrier organic anion transporter family, member 1C1	12p12.2				0.316	0.00018	0.0083				1
ESCO1	establishment of cohesion 1 homolog 1 (S. cerevisiae)	18q11.2	0.416	0.03635	0.1369							1
SLCO3A1	solute carrier organic anion transporter family, member 3A1	15q26				0.356333333	0.006203333	0.041083333				1
PATL1	protein associated with topoisomerase II homolog 1 (yeast)	11q12.1	0.416	0.00693	0.0455							1
SLFN13	schlafen family member 13	17q12				-0.309	0.00726	0.048				1
SLFN14	schlafen family member 14	17q12				-0.385	0.01259	0.0652				1
SLIT1	slit homolog 1 (Drosophila)	10q23.3-q24				-0.336	0.000423333	0.010916667				1
SLIT2	slit homolog 2 (Drosophila)	4p15.2				-0.3165	0.003505	0.0284	0.693	2.54833	6	2
SLIT3	slit homolog 3 (Drosophila)	5q35				-0.3785625	0.001520625	0.01368125				1
SLITRK3	SLIT and NTRK-like family, member 3	3q26.1				0.342	0.00331	0.0316				1
SLITRK4	SLIT and NTRK-like family, member 4	Xq27.3	-0.348	0.02404	0.1043							1
SLITRK5	SLIT and NTRK-like family, member 5	13q31.2	-0.213	0.04433	0.1554							1
SLITRK6	SLIT and NTRK-like family, member 6	13q31.1				0.371	0.00543	0.041	-0.806	2.64833	6	2
SLK	In multiple Geneids		-1.118	6.00E-05	0.0016							1
SLMAP	sarcolemma associated protein	3p21.2-p14.3				-0.355714286	0.003883571	0.029164286				1
SLMO2	slowmo homolog 2 (Drosophila)	20q13.32				0.412	0.00111	0.0185				1
SLN	sarcolipin	11q22-q23							-0.755	3.40167	6	1
SLPI	secretory leukocyte peptidase inhibitor	20q12							-0.829	2.886	5	1
SLTM	SAFB-like, transcription modulator	15q22.1				-0.36	0.015245	0.0682				1
SLU7	SLU7 splicing factor homolog (S. cerevisiae)	5q33.3							-0.856	3.03111	9	1
SLURP1	secreted LY6/PLAUR domain containing 1	8q24.3	-3.55	0	0							1
SLX4	SLX4 structure-specific endonuclease subunit homolog (S. cerevisiae)	16p13.3	-0.223	0.01944	0.0911							1
SMA4	glucuronidase, beta pseudogene	5q13.2							-1.091	4.004	5	1
SMAD2	SMAD family member 2	18q21.1				-0.314	0.00482	0.0384				1
SMAD4	SMAD family member 4	18q21.1				-0.399	0.03191	0.1128				1
SMAD5	SMAD family member 5	5q31	-0.454	0.00753	0.0482							1
RNF133	ring finger protein 133	7q31.32	0.416	0.00067	0.0088				-0.798	2.79571	7	2
SMAD7	SMAD family member 7	18q21.1				-0.382833333	0.009648333	0.0511				1
SMAGP	small cell adhesion glycoprotein	12q13.13	-1.242	0.00039	0.006	-0.408	0.000286667	0.007833333				2
SMAP1	small ArfGAP 1	6q13	-0.635	0.00283	0.0245							1
SMARCA2	SWI/SNF related, matrix associated, actin dependent regulator of chromatin, s	9p22.3	-0.875	0.00084	0.0104	-0.3492	0.001932667	0.019926667				2
SMARCA4	SWI/SNF related, matrix associated, actin dependent regulator of chromatin, s	19p13.2	-0.379	0.0119	0.0658	-0.441086957	0.008407826	0.044752174				2
ZNF768	zinc finger protein 768	16p11.2	0.416	0.02311	0.1016							1
SMARCB1	SWI/SNF related, matrix associated, actin dependent regulator of chromatin, s	22q11.23				-0.4115	0.000185	0.00755				1
SMARCC1	SWI/SNF related, matrix associated, actin dependent regulator of chromatin, s	3p21.31				-0.37362069	0.00308931	0.025427586				1
SMARCD1	SWI/SNF related, matrix associated, actin dependent regulator of chromatin, s	12q13-q14				-0.306	1.00E-05	0.0029				1
SMARCD3	SWI/SNF related, matrix associated, actin dependent regulator of chromatin, s	7q35-q36				-0.326	0.00361	0.0331				1
HACL1	2-hydroxyacyl-CoA lyase 1	3p25.1	0.415	0.03905	0.1433							1
SMC1B	structural maintenance of chromosomes 1B	22q13.31				-0.362125	0.00093375	0.01465				1
PARK7	In multiple Geneids		0.415	0.00792	0.0498							1
SMC4	structural maintenance of chromosomes 4	3q26.1				0.345	0.00112	0.0185				1
SMC4L1	Data not found								-0.798	2.814	5	1
SMC6	structural maintenance of chromosomes 6	2p24.2				0.39	0.000616667	0.0126				1
PRKCSH	protein kinase C substrate 80K-H	19p13.2	0.415	0.00341	0.028							1
RENBP	renin binding protein	Xq28	0.415	0.0321	0.126							1
SMCP	sperm mitochondria-associated cysteine-rich protein	1q21.3				0.383	0.00773	0.0497				1
SMCR7	In multiple Geneids		-0.499	0.00039	0.006							1
SMCR7L	Smith-Magenis syndrome chromosome region, candidate 7-like	22q13				-0.439666667	0.000553333	0.010733333				1
SMCR8	Smith-Magenis syndrome chromosome region, candidate 8	17p11.2				-0.37225	0.0028025	0.02725				1
SMCY	Data not found								-0.803	2.792	5	1
SMDT1	Data not found		-0.649	3.00E-05	0.001							1
SMEK1	SMEK homolog 1, suppressor of mek1 (Dictyostelium)	14q32.12				-0.37	0.00087	0.0165				1
ZFAND1	zinc finger, AN1-type domain 1	8q21.13	0.415	0.01431	0.0742							1
APOA1BP	apolipoprotein A-I binding protein	1q21	0.414	0.01173	0.0652							1
SMG6	smg-6 homolog, nonsense mediated mRNA decay factor (C. elegans)	17p13.3				-0.399222222	0.004420222	0.03072				1
WSB1	WD repeat and SOCS box containing 1	17q11.1	0.414	0.03407	0.1309							1
LRRC58	leucine rich repeat containing 58	3q13.33	0.413	0.04279	0.152							1
SMIM5	Data not found		-0.816	0.00019	0.0036							1

SMN1	survival of motor neuron 1, telomeric	5q13.2				-0.457583333	0.009335	0.044841667	0.721	2.348	5	2
SMN2	survival of motor neuron 2, centromeric	5q13.2				-0.457583333	0.009335	0.044841667	0.721	2.348	5	2
SMO	smoothened, frizzled family receptor	7q32.3	-0.52	6.00E-04	0.0082							1
PRRC2C	proline-rich coiled-coil 2C	1q23.3	0.413	7.00E-04	0.0092							1
SMOC2	SPARC related modular calcium binding 2	6q27				-0.333	0.006285	0.0444				1
SCN8A	sodium channel, voltage gated, type VIII, alpha subunit	12q13	0.413	0.0479	0.1629							1
SMPD1	sphingomyelin phosphodiesterase 1, acid lysosomal	11p15.4-p15.1	-0.442	0.01283	0.0691							1
SMPD2	sphingomyelin phosphodiesterase 2, neutral membrane (neutral sphingomyel	6q21	-0.614	0.00216	0.0202							1
SLC35B1	solute carrier family 35, member B1	17q21.33	0.413	0.00136	0.0146							1
CLCN7	chloride channel, voltage-sensitive 7	16p13	0.412	0.04446	0.1557							1
CNOT11	Data not found		0.412	0.02591	0.1098							1
SMPX	small muscle protein, X-linked	Xp22.1				0.322	0.00116	0.0189				1
SMR3A	submaxillary gland androgen regulated protein 3A	4q13.3							-0.89	3.23875	8	1
SMR3B	submaxillary gland androgen regulated protein 3B	4q13.3							-0.938	3.14222	9	1
MYH9	myosin, heavy chain 9, non-muscle	22q13.1	0.412	0.00282	0.0244							1
SMTN	smoothelin	22q12.2				-0.498	4.00E-05	0.003633333				1
SMTNL2	smoothelin-like 2	17p13.2				-0.445833333	0.001748333	0.02075				1
SMU1	smu-1 suppressor of mec-8 and unc-52 homolog (C. elegans)	9p12				-0.35375	0.00183	0.019875				1
SMURF1	SMAD specific E3 ubiquitin protein ligase 1	7q22.1				-0.376	0.00954	0.0558	0.983	3.654	5	2
NDUFAF7	Data not found		0.412	0.02353	0.1029							1
PRMT3	protein arginine methyltransferase 3	11p15.1	0.412	0.00554	0.0392							1
SMYD4	In multiple Geneids					-0.368	0.02807	0.1033				1
PTDSS1	phosphatidylserine synthase 1	8q22	0.412	0.02134	0.0969	0.31	0.00103	0.0178				2
SSH2	slingshot homolog 2 (Drosophila)	17q11.2	0.412	0.01004	0.0586							1
SNAI2	snail homolog 2 (Drosophila)	8q11	-0.857	0.01175	0.0652							1
SNAI3	snail homolog 3 (Drosophila)	16q24.3	-0.308	0.01786	0.086							1
ZNF473	zinc finger protein 473	19q13.33	0.412	0.00219	0.0204							1
SNAP25	synaptosomal-associated protein, 25kDa	20p12-p11.2				0.309333333	0.015586667	0.070633333				1
SNAP29	synaptosomal-associated protein, 29kDa	22q11.21	-0.601	0.00039	0.006	-0.371	0.0025275	0.025975				2
SNAPC1	small nuclear RNA activating complex, polypeptide 1, 43kDa	14q22				-0.324	0.00269	0.0284				1
SNAPC2	small nuclear RNA activating complex, polypeptide 2, 45kDa	19p13				-0.618	5.00E-05	0.0052				1
SNAPC3	small nuclear RNA activating complex, polypeptide 3, 50kDa	9p22.3	-0.58	0.00017	0.0032	-0.435	0.000266667	0.008133333				2
SNAPC4	small nuclear RNA activating complex, polypeptide 4, 190kDa	9q34.3							0.736	3.56143	7	1
SNCA	synuclein, alpha (non A4 component of amyloid precursor)	4q21	-0.634	0.00396	0.0311							1
SNCAIP	synuclein, alpha interacting protein	5q23.2				0.301	0.00564	0.0418				1
SNCB	synuclein, beta	5q35	-0.207	0.03554	0.1347	-0.483333333	0.000166667	0.0073	1.055	3.072	5	3
SNCG	synuclein, gamma (breast cancer-specific protein 1)	10q23.2-q23.3	-0.6	0.00052	0.0073							1
RHEB	Ras homolog enriched in brain	7q36	0.4115	0.009325	0.05555							1
GPR114	G protein-coupled receptor 114	16q21	0.411	0.02306	0.1015							1
SNHG12	small nucleolar RNA host gene 12 (non-protein coding)	1p35.3				-0.513	0.005853333	0.033833333				1
SNHG3	small nucleolar RNA host gene 3 (non-protein coding)	1p36.1				-0.462	0.00089	0.0168				1
SNHG3-RCC1	SNHG3-RCC1 readthrough transcript	1p36.1				-0.461	0.012915	0.057366667				1
SNIP	SNAP25-interacting protein					-0.352428571	0.00265	0.025542857				1
SNN	stannin	16p13				-0.363	3.00E-04	0.0102				1
KCNJ8	potassium inwardly-rectifying channel, subfamily J, member 8	12p11.23	0.411	0.0022	0.0204							1
PLA2G4C	phospholipase A2, group IVC (cytosolic, calcium-independent)	19q13.3	0.411	0.03668	0.1377							1
TBRG4	transforming growth factor beta regulator 4	7p13	0.411	0.00784	0.0495							1
TCTN2	tectonic family member 2	12q24.31	0.411	0.02695	0.1125							1
DESI2	desumoylating isopeptidase 2	1q44	0.41	0.005	0.0365							1
HEY1	hairy/enhancer-of-split related with YRPW motif 1	8q21	0.41	0.00809	0.0505							1
HSP90AB4P	heat shock protein 90kDa alpha (cytosolic), class B member 4, pseudogene	15q22.1	0.41	0.00853	0.0523							1
IAH1	isoamyl acetate-hydrolyzing esterase 1 homolog (S. cerevisiae)	2p25.1	0.41	0.01391	0.0728							1
PMPCA	peptidase (mitochondrial processing) alpha	9q34.3	0.41	0.01714	0.0837							1
YDJC	YdjC homolog (bacterial)	22q11.21	0.41	0.0016	0.0163							1
EED	embryonic ectoderm development	11q14.2-q22.3	0.409	0.01147	0.0641							1
LRRC8B	leucine rich repeat containing 8 family, member B	1p22.2	0.409	0.01648	0.0814							1
SNORA59A	small nucleolar RNA, H/ACA box 59A	1p36.21	-0.609	0.00469	0.0349							1
LRRCC1	leucine rich repeat and coiled-coil centrosomal protein 1	8q21.2	0.409	0.02073	0.095							1
NELFCD	Data not found		0.409	0.04468	0.1561							1
RPRD1B	regulation of nuclear pre-mRNA domain containing 1B	20q11.21-q12	0.409	0.01022	0.0594							1
SUPT20H	Data not found		0.409	0.00272	0.0238							1
BTN2A2	butyrophilin, subfamily 2, member A2	6p22.1	0.408	0.00191	0.0185							1
CHCHD3	coiled-coil-helix-coiled-coil-helix domain containing 3	7q33	0.408	0.0434	0.1532							1
FAM167B	family with sequence similarity 167, member B	1p35.1	0.408	0.00037	0.0057							1
FAM208B	family with sequence similarity 208, member B	10p15.1	0.408	0.00449	0.0339							1
FBXO22	F-box protein 22	15q24.2	0.408	0.00728	0.0471							1
SNORD109A	small nucleolar RNA, C/D box 109A	15q11.2	-0.581	0.00631	0.0427							1
DKC1	dyskeratosis congenita 1, dyskerin	Xq28	0.407	0.03164	0.1249							1
SNORD115-11	small nucleolar RNA, C/D box 115-11	15q11.2	-0.678	0.01807	0.0867							1

SNORD115-20	small nucleolar RNA, C/D box 115-20	15q11.2	-0.284	0.029	0.1179							1
SNORD115-25	small nucleolar RNA, C/D box 115-25	15q11.2	-0.673	0.00036	0.0056							1
SNORD115-30	small nucleolar RNA, C/D box 115-30	15q11.2	-0.517	0.04889	0.1648							1
SNORD115-32	small nucleolar RNA, C/D box 115-32	15q11.2	-0.537	0.00412	0.032							1
SNORD115-33	small nucleolar RNA, C/D box 115-33	15q11.2	-0.244	0.04115	0.1484							1
SNORD115-35	small nucleolar RNA, C/D box 115-35	15q11.2	-0.254	0.01877	0.089							1
SNORD115-38	small nucleolar RNA, C/D box 115-38	15q11.2	-0.177	0.04323	0.153							1
SNORD115-42	small nucleolar RNA, C/D box 115-42	15q11.2	-0.322	0.00232	0.0213							1
SNORD115-44	small nucleolar RNA, C/D box 115-44	15q11.2	-0.503	0.01721	0.0839							1
SNORD116-13	small nucleolar RNA, C/D box 116-13	15q11.2	-0.552	0.03634	0.1369							1
SNORD116-21	small nucleolar RNA, C/D box 116-21	15q11.2	-1.285	0.01449	0.0748							1
LSM4	LSM4 homolog, U6 small nuclear RNA associated (S. cerevisiae)	19p13.11	0.407	0.00459	0.0345							1
AHCTF1	AT hook containing transcription factor 1	1q44	0.406	0.01916	0.0903							1
ATP2C1	ATPase, Ca++ transporting, type 2C, member 1	3q22.1	0.406	0.01307	0.0699	0.307	0.02193	0.09				2
CCT7	chaperonin containing TCP1, subunit 7 (eta)	2p13.2	0.406	0.01252	0.0681							1
INPPL1	inositol polyphosphate phosphatase-like 1	11q13	0.406	0.01171	0.0651							1
KIN	KIN, antigenic determinant of recA protein homolog (mouse)	10p15-p14	0.406	0.0054	0.0385							1
MRPL36	mitochondrial ribosomal protein L36	5p15.3	0.406	0.04518	0.1573							1
NAT9	N-acetyltransferase 9 (GCN5-related, putative)	17q25.1	0.406	0.01243	0.0677							1
NUFIP2	nuclear fragile X mental retardation protein interacting protein 2	17q11.2	0.406	0.00665	0.0443							1
SMC1A	structural maintenance of chromosomes 1A	Xp11.22-p11.21	0.406	0.02497	0.107							1
CIT	citron (rho-interacting, serine/threonine kinase 21)	12q24	0.405	0.04845	0.164							1
GPATCH2	G patch domain containing 2	1q41	0.405	0.0087	0.0531	0.3135	0.00273	0.0263				2
HLA-E	major histocompatibility complex, class I, E	6p21.3	0.405	0.03683	0.1381							1
SPATS2L	spermatogenesis associated, serine-rich 2-like	2q33.1	0.405	0.03098	0.1234	0.339	0.000706667	0.013833333				2
CASP8	caspase 8, apoptosis-related cysteine peptidase	2q33-q34	0.404	0.02229	0.0995							1
GLRX5	glutaredoxin 5	14q32.13	0.404	0.03616	0.1365							1
KIAA1211	KIAA1211	4q12	0.404	0.00748	0.0481							1
SNORD96B	small nucleolar RNA, C/D box 96B	Xq23	-0.371	0.00366	0.0294							1
SNORD99	small nucleolar RNA, C/D box 99	1p35.3				-0.4	0.0165	0.0762				1
MRGBP	Data not found		0.404	0.00455	0.0343							1
SNRNP25	small nuclear ribonucleoprotein 25kDa (U11/U12)	16p13.3				-0.306	0.01264	0.0653				1
SNRNP40	small nuclear ribonucleoprotein 40kDa (U5)	1p35.2				-0.345	0.01311	0.063566667				1
NHLRC2	NHL repeat containing 2	10q25.3	0.404	0.0166	0.0818				-0.677	2.51571	7	2
SNRNP70	small nuclear ribonucleoprotein 70kDa (U1)	19q13.3				-0.4165	0.0041825	0.03135				1
SNRPA	small nuclear ribonucleoprotein polypeptide A	19q13.1				-0.392	0.00331	0.0316				1
STAT2	signal transducer and activator of transcription 2, 113kDa	12q13.3	0.404	0.04246	0.1512							1
DNASE2	deoxyribonuclease II, lysosomal	19p13.2	0.403	0.01907	0.09							1
HSPBAP1	In multiple Geneids		0.403	0.00442	0.0335							1
KIF22	kinesin family member 22	16p11.2	0.403	0.03565	0.135							1
TMC6	transmembrane channel-like 6	17q25.3	0.403	0.00189	0.0183							1
SNRPD3	small nuclear ribonucleoprotein D3 polypeptide 18kDa	22q11.23				-0.341	2.50E-05	0.00335				1
UBXN11	UBX domain protein 11	1p36.11	0.403	0.01648	0.0814							1
ULBP1	In multiple Geneids		0.403	0.01101	0.0624							1
SNRPN	small nuclear ribonucleoprotein polypeptide N	15q11.2	-0.791	0.00327	0.0273				1.041	3.40833	6	2
SNTA1	syntrophin, alpha 1 (dystrophin-associated protein A1, 59kDa, acidic compone	20q11.2	-0.261	0.02186	0.0984	-0.33	0.00186	0.0237				2
SNTB1	syntrophin, beta 1 (dystrophin-associated protein A1, 59kDa, basic componen	8q23-q24				0.36175	0.01005	0.052791667				1
SNTB2	syntrophin, beta 2 (dystrophin-associated protein A1, 59kDa, basic componen	16q22.1				-0.342125	0.00664125	0.04165				1
SNTG1	syntrophin, gamma 1	8q11-q12				0.337285714	0.000762857	0.0137				1
SNTG2	syntrophin, gamma 2	2p25.3				0.35775	0.01045	0.042				1
SNURF	SNRPN upstream reading frame	15q12							1.041	3.40833	6	1
SNX1	sorting nexin 1	15q22.31				-0.334	0.00071	0.0151				1
ANGEL1	angel homolog 1 (Drosophila)	14q24.3	0.402	0.03271	0.1276							1
SNX13	sorting nexin 13	7p21.1				0.3765	0.00104625	0.0152375				1
DNPEP	aspartyl aminopeptidase	2q35	0.402	0.00964	0.0569							1
SNX16	sorting nexin 16	8q21.13				0.331	0.000125	0.00715	-0.867333333	2.751703333	6.333333333	2
SNX17	sorting nexin 17	2p23-p22	-0.257	0.04884	0.1647							1
SNX18	sorting nexin 18	5q11.2	-0.368	0.00161	0.0164	-0.3275	0.001985	0.0233				2
SNX19	In multiple Geneids					0.4555	3.00E-04	0.0093				1
SNX2	sorting nexin 2	5q23	-0.363	0.04879	0.1646							1
SNX24	sorting nexin 24	5q23.2	-2.068	0	0	-0.3471	0.00044	0.01063				2
SNX25	sorting nexin 25	4q35.1				-0.385421053	0.005433684	0.0357				1
SNX26	Data not found					-0.357	0.000585	0.01375				1
LARS2	leucyl-tRNA synthetase 2, mitochondrial	3p21.3	0.402	0.02606	0.1103							1
SNX29	sorting nexin 29	16p13.13-p13.12	-0.971	0.00025	0.0043	-0.3576	0.00653	0.04352				2
SNX3	sorting nexin 3	6q21	-0.498	0.00204	0.0193	-0.38	0.00193	0.0241				2
SNX31	sorting nexin 31	8q22.3	-1.808	0	0							1
SNX33	sorting nexin 33	15q24.2	-0.547	0.00504	0.0367	-0.334	0.01649	0.0762				2
PFDN2	prefoldin subunit 2	1q23.3	0.402	0.00524	0.0377							1

SNX6	sorting nexin 6	14q13.1				-0.3935	0.01588	0.0712				1
SHISA4	shisa homolog 4 (Xenopus laevis)	1q32.1	0.402	0.00978	0.0576							1
SNX8	sorting nexin 8	7p22.3				-0.325	0.012345	0.06235				1
SNX9	sorting nexin 9	6q25.1-q26	-0.693	0.00885	0.0537							1
SND1	staphylococcal nuclease and tudor domain containing 1	7q31.3	0.402	0.01365	0.0718							1
SOCS2	suppressor of cytokine signaling 2	12q				-0.337	0.00321	0.0311				1
SYDE2	synapse defective 1, Rho GTPase, homolog 2 (C. elegans)	1p22.3	0.402	0.01574	0.0791							1
CDC26	In multiple Geneids		0.401	0.01365	0.0718							1
FAM83E	family with sequence similarity 83, member E	19q13.33	0.401	0.00976	0.0575				-0.633	2.516	5	2
SOHLH2	spermatogenesis and oogenesis specific basic helix-loop-helix 2	13q13.3				0.326	0.0165	0.0762				1
SON	SON DNA binding protein	21q22.11	-0.216	0.04552	0.1581	-0.3245	0.00173	0.0181				2
SORBS1	In multiple Geneids								-0.697	2.296	5	1
SORBS2	sorbin and SH3 domain containing 2	4q35.1	-1.319	0.00162	0.0164	-0.364806452	0.01173871	0.056858065				2
SORBS3	sorbin and SH3 domain containing 3	8p21.3				-0.343	0.00139	0.0196				1
SORCS1	sortilin-related VPS10 domain containing receptor 1	10q23-q25				-0.3335	0.018215	0.08055				1
SORCS2	sortilin-related VPS10 domain containing receptor 2	4p16.1				-0.379488889	0.002127111	0.020257778				1
SORCS3	sortilin-related VPS10 domain containing receptor 3	10q23-q25				0.323	0.00023	0.0092				1
SORD	sorbitol dehydrogenase	15q15.3				-0.3775	0.00212	0.0226				1
SORT1	sortilin 1	1p13.3	-0.898	0.00022	0.0039	-0.3035	0.00214	0.02365				2
SOST	sclerostin	17q11.2	-0.308	0.00376	0.03							1
SOSTDC1	sclerostin domain containing 1	7p21.1							-0.923	2.736	5	1
SOWAHB	sosondowah ankyrin repeat domain family member B	4q21.1	-0.368	0.00308	0.0261							1
SOWAHC	sosondowah ankyrin repeat domain family member C	2q13	-0.898	0.00119	0.0132							1
SOX10	SRY (sex determining region Y)-box 10	22q13.1	-0.26	0.0052	0.0375	-0.4305	0.00208	0.01955				2
SOX11	SRY (sex determining region Y)-box 11	2p25				0.331	9.00E-05	0.0063				1
SOX15	SRY (sex determining region Y)-box 15	17p12.3	-2.407	0	0				1.106	2.834	5	2
SOX2	SRY (sex determining region Y)-box 2	3q26.3-q27	-3.316	0	0							1
SOX21	SRY (sex determining region Y)-box 21	13q31-q32	-1.012	0	1.00E-04							1
GBAS	glioblastoma amplified sequence	7p12	0.401	0.04862	0.1643							1
PNO1	partner of NOB1 homolog (S. cerevisiae)	2p14	0.401	0.04549	0.1581							1
SOX5	SRY (sex determining region Y)-box 5	12p12.1				0.370745902	0.011200328	0.055455738				1
SOX6	In multiple Geneids					0.330714286	0.008808571	0.044371429	-0.894333333	3.198333333	8.333333333	2
SOX7	SRY (sex determining region Y)-box 7	8p22	-0.78	1.00E-05	3.00E-04				0.896	2.645	5	2
WRN	Werner syndrome, RecQ helicase-like	8p12	0.401	0.02568	0.1092							1
SP1	Sp1 transcription factor	12q13.1	-0.463	0.0126	0.0684							1
ALG1	asparagine-linked glycosylation 1, beta-1,4-mannosyltransferase homolog (S.	16p13.3	0.4	0.00808	0.0505							1
CCT4	chaperonin containing TCP1, subunit 4 (delta)	2p15	0.4	0.03767	0.14							1
COX20	COX20 Cox2 chaperone homolog (S. cerevisiae)	1q44	0.4	0.02003	0.0927							1
GATAD1	GATA zinc finger domain containing 1	7q21-q22	0.4	0.00226	0.0209							1
RNU105B	RNA, U105B small nucleolar	20p12.3	0.4	0.03178	0.1252							1
SP6	Sp6 transcription factor	17q21.32				-0.322	0.00835	0.0518				1
SP7	Sp7 transcription factor	12q13.13	-0.409	0.00187	0.0182	-0.426	0.00017	0.006233333				2
SP9	Sp9 transcription factor homolog (mouse)	2q31.1	-0.254	0.02429	0.1049							1
SPACA4	In multiple Geneids		-0.406	0.00855	0.0524							1
SPACA5	In multiple Geneids					-0.31	0.01893	0.0825				1
SPACA5B	sperm acrosome associated 5B	Xp11.23				-0.31	0.01893	0.0825				1
SPAG11B	sperm associated antigen 11B	8p23.1				-0.358	0.028375	0.1047				1
SPAG16	sperm associated antigen 16	2q34				0.329875	0.00063625	0.0105625				1
SPAG17	sperm associated antigen 17	1p12	-1.518	0	1.00E-04							1
ARMC1	armadillo repeat containing 1	8q13.1	0.399	0.01273	0.0688							1
ATG12	autophagy related 12	5q21-q22	0.399	0.01031	0.0598							1
SPAG7	sperm associated antigen 7	17p13.2				-0.41	0.00197	0.0244				1
DIXDC1	DIX domain containing 1	11q23.1	0.399	0.02755	0.1139							1
SPAM1	sperm adhesion molecule 1 (PH-20 hyaluronidase, zona pellucida binding)	7q31.3				0.361	0.01409	0.0695				1
SPANXA1	sperm protein associated with the nucleus, X-linked, family member A1	Xq27.1							0.698	3.059	5	1
SPANXA2	In multiple Geneids								0.698	3.059	5	1
SPANXE	SPANX family, member E	Xq27.2							0.698	3.059	5	1
SPANXN2	SPANX family, member N2	Xq27.3	-0.217	0.01059	0.0609							1
SPANX-N2	Data not found								-0.853	2.714	5	1
SPANXN3	SPANX family, member N3	Xq27.3	-0.267	0.00417	0.0322							1
SPANXN4	SPANX family, member N4	Xq27.3	-0.282	0.00629	0.0427							1
SPANXN5	SPANX family, member N5	Xp11.22	-0.22	0.01088	0.0618							1
SPANX-N5	Data not found								-0.612	2.30167	6	1
LIPT2	lipoyl(octanoyl) transferase 2 (putative)	11q13.4	0.399	0.01676	0.0824							1
SPARCL1	SPARC-like 1 (hevin)	4q22.1							-0.839	3.098	5	1
LOXL1	lysyl oxidase-like 1	15q22	0.399	0.02344	0.1027							1
SPATA16	spermatogenesis associated 16	3q26.31				0.35872	0.0047044	0.031636				1
P4HTM	prolyl 4-hydroxylase, transmembrane (endoplasmic reticulum)	3p21.31	0.399	0.00768	0.0488							1
SPATA18	In multiple Geneids		-0.84	0.00051	0.0073							1

SPATA19	spermatogenesis associated 19	11q25	-0.299	0.00317	0.0267							1
SNORD13	In multiple Geneids		0.399	0.03734	0.1393							1
C6orf89	chromosome 6 open reading frame 89	6p21.2	0.398	0.03888	0.1429							1
SPATA21	spermatogenesis associated 21	1p36.13				-0.4596	0.002094	0.0221				1
SPATA22	spermatogenesis associated 22	17p13.3	-0.21	0.00825	0.0512	-0.335	0.01562	0.0738				2
ERCC3	excision repair cross-complementing rodent repair deficiency, complementati	2q21	0.398	0.02875	0.1173							1
SPATA2L	spermatogenesis associated 2-like	16q24.3				-0.337	0.00312	0.0306				1
SPATA3	In multiple Geneids		-0.31	0.0068	0.045							1
SPATA31D4	Data not found		-0.199	0.01923	0.0904							1
SPATA4	In multiple Geneids					-0.301	0.00781	0.05				1
MAP1LC3A	In multiple Geneids		0.398	0.01537	0.0778							1
SPATA5L1	spermatogenesis associated 5-like 1	15q21.1							-1.01	3.17	7	1
SPATC1	spermatogenesis and centriole associated 1	8q24.3							0.589	2.602	5	1
SPATC1L	Data not found		-0.285	0.00898	0.0543							1
RAMP2	receptor (G protein-coupled) activity modifying protein 2	17q12-q21.1	0.398	0.04271	0.1518							1
RANBP2	RAN binding protein 2	2q12.3	0.398	0.00354	0.0287							1
SPBC25	Data not found								-0.947	3.14	6	1
SPC24	SPC24, NDC80 kinetochore complex component, homolog (S. cerevisiae)	19p13.2				-0.492	0.00592	0.0429				1
SPG21	spastic paraplegia 21 (autosomal recessive, Mast syndrome)	15q21-q22	0.398	0.02423	0.1048							1
SPDYA	speedy homolog A (Xenopus laevis)	2p23.2							-0.852	2.948	5	1
SPECC1	sperm antigen with calponin homology and coiled-coil domains 1	17p11.2	-0.627	0.00787	0.0496							1
SPECC1L	sperm antigen with calponin homology and coiled-coil domains 1-like	22q11.23	-0.637	0.00085	0.0105							1
SPEF2	sperm flagellar 2	5p13.2				0.380727273	0.007009091	0.043586364				1
SPEN	spen homolog, transcriptional regulator (Drosophila)	1p36				-0.3428	0.007436	0.0455				1
SPG11	In multiple Geneids					-0.330333333	0.00383	0.034033333				1
SPG20	spastic paraplegia 20 (Troyer syndrome)	13q13.3	-0.624	0.03014	0.121							1
SPINT2	serine peptidase inhibitor, Kunitz type, 2	19q13.1	0.398	0.00174	0.0173							1
SPG7	spastic paraplegia 7 (pure and complicated autosomal recessive)	16q24.3				-0.39	0.007695	0.044325				1
SPHK2	sphingosine kinase 2	19q13.2							-0.622	2.45	5	1
SPHKAP	SPHK1 interactor, AKAP domain containing	2q36				0.343	0.00281	0.029				1
SPIB	Spi-B transcription factor (Spi-1/PU.1 related)	19q13.3-q13.4				-0.34	0.00189	0.0239				1
SPIC	Spi-C transcription factor (Spi-1/PU.1 related)	12q23.2							-0.948	3.29571	7	1
SPIN1	spindlin 1	9q22.1				-0.342	0.01774	0.0794				1
ZNF33B	zinc finger protein 33B	10q11.2	0.398	0.02387	0.1038							1
BRI3BP	BRI3 binding protein	12q24.31	0.397	0.03197	0.1257							1
SPINK2	serine peptidase inhibitor, Kazal type 2 (acrosin-trypsin inhibitor)	4q12				-0.338	0.04256	0.1346				1
SPINK4	serine peptidase inhibitor, Kazal type 4	9p13.3				-0.39	7.50E-05	0.00585				1
SPINK5	serine peptidase inhibitor, Kazal type 5	5q32	-4.51	0	2.00E-04							1
SPINK5L2	Data not found								-0.956	3.404	5	1
SPINK7	serine peptidase inhibitor, Kazal type 7 (putative)	5q32							-0.805	3.37333	9	1
SPINT1	serine peptidase inhibitor, Kunitz type 1	15q15.1	-0.653	0.01557	0.0785	-0.3375	0.009405	0.05505				2
CPSF3	cleavage and polyadenylation specific factor 3, 73kDa	2p25.1	0.397	0.04268	0.1518							1
SPINT4	In multiple Geneids								-0.724	2.70667	6	1
HSP90AA4P	heat shock protein 90kDa alpha (cytosolic), class A member 4, pseudogene	4q35.2	0.397	0.04197	0.1501							1
SPN	sialophorin	16p11.2	-0.398	0.00875	0.0533	-0.372	0.00195	0.0243				2
ITPA	inosine triphosphatase (nucleoside triphosphate pyrophosphatase)	20p	0.397	0.00749	0.0481							1
SPNS2	spinster homolog 2 (Drosophila)	17p13.2	-1.693	0.00021	0.0038	-0.351142857	0.007564286	0.0483				2
SPNS3	spinster homolog 3 (Drosophila)	17p13.2				-0.4225	0.0045625	0.033275				1
SPO11	SPO11 meiotic protein covalently bound to DSB homolog (S. cerevisiae)	20q13.31				0.351	0.01033	0.0583				1
SPOCD1	SPOC domain containing 1	1p35.2				0.303	0.03446	0.1182				1
SPOCK1	sparc/osteonectin, cwcv and kazal-like domains proteoglycan (testican) 1	5q31.2				-0.347	0.001485	0.0207				1
SPOCK2	sparc/osteonectin, cwcv and kazal-like domains proteoglycan (testican) 2	10pter-q25.3				-0.4445	0.00048	0.0097				1
SPOCK3	sparc/osteonectin, cwcv and kazal-like domains proteoglycan (testican) 3	4q32.3				-0.313	0.013365	0.06715				1
HMGB1	high mobility group box 1	13q12	0.3965	0.02656	0.10635							1
RRAGB	Ras-related GTP binding B	Xp11.21	0.396	0.03842	0.142	0.313	0.00015	0.0078				2
ZNF227	zinc finger protein 227		0.396	0.00646	0.0434							1
SPP2	secreted phosphoprotein 2, 24kDa	2q37-qter							-0.767	2.45286	7	1
SPPL2A	signal peptide peptidase like 2A	15q21.2				-0.3415	0.043445	0.13635				1
PAGR1	Data not found		0.395	0.026	0.1101							1
SPPL2C	signal peptide peptidase like 2C	17q21.31	-0.263	0.01246	0.0679							1
SPPL3	signal peptide peptidase like 3	12q24.31	-0.514	0.00823	0.0512	-0.422333333	0.000766667	0.0142				2
SEP15	15 kDa selenoprotein	1p31	0.395	0.01229	0.0672							1
ST7	suppression of tumorigenicity 7	7q31.2	0.395	0.00695	0.0456							1
VBP1	von Hippel-Lindau binding protein 1	Xq28	0.395	0.03399	0.1306	0.581	1.00E-05	0.003				2
SPRED3	sprouty-related, EVH1 domain containing 3	19q13				-0.3185	0.00475	0.03815	0.819	2.78143	7	2
SPRR1A	small proline-rich protein 1A	1q21-q22	-3.555	0.00084	0.0104							1
SPRR1B	small proline-rich protein 1B	1q21-q22							-0.711	2.44571	7	1
SPRR2A	small proline-rich protein 2A	1q21-q22							-0.894	3.11667	6	1
SPRR2B	small proline-rich protein 2B	1q21-q22	-4.3315	0.00012	0.0026							1

SPRR2D	small proline-rich protein 2D	1q21-q22							-1.054	4.09	7	1
SPRR2E	small proline-rich protein 2E	1q21-q22							-0.859	3.076	5	1
SPRR2F	small proline-rich protein 2F	1q21-q22							-0.894	3.11667	6	1
SPRR2G	small proline-rich protein 2G	1q21-q22							-1.101	3.58571	7	1
SPRR3	small proline-rich protein 3	1q21-q22							-1.041	2.988	5	1
SPRR4	small proline-rich protein 4	1q21.3	-0.347	0.00084	0.0104							1
PXDC1	PX domain containing 1	6p25.2	0.394	0.03347	0.1295							1
SEPHS2	selenophosphate synthetase 2	16p11.2	0.394	0.0114	0.064							1
URGCP	upregulator of cell proliferation	7p13	0.394	0.0065	0.0436							1
SPRYD3	SPRY domain containing 3	12q13.13	-0.33	0.01908	0.09	-0.334	0.000596667	0.013566667				2
SPRYD5	Data not found								-1.267	4.566	5	1
SPSB1	splA/ryanodine receptor domain and SOCS box containing 1	1p36.22				-0.353	0.002255	0.023783333				1
SPSB3	splA/ryanodine receptor domain and SOCS box containing 3	16p13.3	-0.658	0.00064	0.0085							1
SPTA1	spectrin, alpha, erythrocytic 1 (elliptocytosis 2)	1q21				0.32175	0.001385	0.01985	-1.112	3.90286	7	2
C9orf89	chromosome 9 open reading frame 89	9q22.31	0.393	0.01692	0.083							1
SPTB	spectrin, beta, erythrocytic	14q23-q24.2				-0.34225	0.00036	0.010175				1
LINC00650	Data not found		0.393	0.00595	0.0411							1
SPTBN2	spectrin, beta, non-erythrocytic 2	11q13	-1.591	1.00E-05	5.00E-04	-0.321	0.02269	0.0919	0.962	3.73667	6	3
SPTBN4	In multiple Geneids					-0.376	0.007097778	0.041366667	1.101	2.99167	6	2
SPTLC1	serine palmitoyltransferase, long chain base subunit 1	9q22.2							-1.188	2.45	6	1
SPTLC2	serine palmitoyltransferase, long chain base subunit 2	14q24.3	-0.83	0.00067	0.0089	-0.358875	0.00631375	0.0413375				2
SPTLC3	serine palmitoyltransferase, long chain base subunit 3	20p12.1				0.371909091	0.013838182	0.064281818				1
SPTSSB	serine palmitoyltransferase, small subunit B	3q26.1	-1.087	0.00655	0.0438							1
SPZ1	spermatogenic leucine zipper 1	5q14.1							-0.881	3.01	5	1
SQLE	squalene epoxidase	8q24.1				0.3255	0.00569	0.04175				1
SQRDL	sulfide quinone reductase-like (yeast)	15q15	-0.731	0.00477	0.0353	-0.314	0.00012	0.007				2
MAGEH1	melanoma antigen family H, 1	Xp11.21	0.393	0.00562	0.0395	0.313	0.00501	0.0392				2
SR140	Data not found					0.304	0.00525	0.0402				1
SRA1	steroid receptor RNA activator 1	5q31.3				-0.522	0.000805	0.0129				1
SRBD1	S1 RNA binding domain 1	2p21				0.38105	0.0026125	0.02275	-0.936	2.95	6	2
PQLC3	PQ loop repeat containing 3	2p25.1	0.393	0.00779	0.0493							1
SRD5A1	steroid-5-alpha-reductase, alpha polypeptide 1 (3-oxo-5 alpha-steroid delta 4-	5p15	-1.102	5.00E-05	0.0013							1
SRD5A2L	Data not found								-0.803	2.50833	6	1
SRD5A2L2	Data not found								-0.979	3.11	9	1
SRD5A3	steroid 5 alpha-reductase 3	4q12				-0.354	0.01119	0.0529				1
SREBF1	sterol regulatory element binding transcription factor 1	17p11.2				-0.315	0.01777	0.0795				1
SREBF2	sterol regulatory element binding transcription factor 2	22q13	-0.688	4.00E-04	0.006	-0.418052632	0.005452105	0.029494737				2
SREK1IP1	SREK1-interacting protein 1	5q12.3	-1.216	0	1.00E-04							1
SRGAP3	SLIT-ROBO Rho GTPase activating protein 3	3p25.3	-0.737	4.00E-05	0.0011	-0.350833333	0.002086667	0.022333333				2
SCAND2P	Data not found		0.393	0.01549	0.0782							1
SRP9	signal recognition particle 9kDa	1q42.12	0.393	0.02947	0.1192							1
SRL	sarcalumenin	16p13.3				-0.505	0.008223	0.04768				1
TAF2	TAF2 RNA polymerase II, TATA box binding protein (TBP)-associated factor, 15	8q24.12	0.393	0.03372	0.13	0.3265	0.017615	0.07775				2
SRP19	signal recognition particle 19kDa	5q21-q22	-0.195	0.04858	0.1642	-0.431	1.00E-04	0.0065				2
SRP68	In multiple Geneids					-0.3402	0.005878	0.03996				1
SRP72	signal recognition particle 72kDa	4q11							-0.921	2.632	5	1
CLIP2	CAP-GLY domain containing linker protein 2	7q11.23	0.392	0.01309	0.0699							1
SRPK2	SRSF protein kinase 2	7q22-q31.1							-0.892	3.64286	7	1
SRPX2	sushi-repeat containing protein, X-linked 2	Xq21.33-q23	-1.132	0.00835	0.0516							1
SRR	serine racemase	17p13	-0.856	0.00068	0.009	-0.371	0.00173	0.0229				2
SRRD	SRR1 domain containing	22q12.1				-0.39	7.50E-05	0.0059				1
SRRM1	serine/arginine repetitive matrix 1	1p36.11				-0.318	0.00266	0.0282				1
SRRM2	serine/arginine repetitive matrix 2	16p13.3				-0.54625	0.003375	0.029425				1
SRRM3	serine/arginine repetitive matrix 3	7q11.23	-0.328	0.00479	0.0355							1
SRRM4	serine/arginine repetitive matrix 4	12q24.23	-0.224	0.01997	0.0926							1
SRrp35	Data not found					-0.36	0.01133	0.0614				1
IRF9	interferon regulatory factor 9	14q11.2	0.392	0.01745	0.0847							1
NHS	Nance-Horan syndrome (congenital cataracts and dental anomalies)	Xp22.13	0.392	0.0392	0.1436	0.3454	0.003453	0.0291				2
SBDS	Shwachman-Bodian-Diamond syndrome	7q11.21	0.392	0.03331	0.129							1
SRSF5	serine/arginine-rich splicing factor 5	14q24	-0.422	0.00881	0.0535							1
SRY	sex determining region Y	Yp11.3							-0.945	3.57	8	1
SS18L1	synovial sarcoma translocation gene on chromosome 18-like 1	20q13.3				0.377	0.01137	0.0615				1
SS18L2	synovial sarcoma translocation gene on chromosome 18-like 2	3p21				-0.322	0.00048	0.0126				1
SNX14	sorting nexin 14	6q14.3	0.392	0.02874	0.1173							1
SSBP1	single-stranded DNA binding protein 1, mitochondrial	7q34							-0.629	2.6	6	1
SSBP2	single-stranded DNA binding protein 2	5q14.1	-1.982	9.00E-05	0.0021	-0.337	0.000435	0.0115				2
SSBP3	In multiple Geneids					-0.360055556	0.003871111	0.0306				1
SSBP4	single stranded DNA binding protein 4	19p13.1							0.537	2.93143	7	1
WDR34	WD repeat domain 34	9q34.11	0.392	0.00305	0.0259							1

SSH1	slingshot homolog 1 (Drosophila)	12q24.11				-0.3325	0.0002975	0.009				1
BCL2L1	BCL2-like 1	20q11.21	0.391	0.0287	0.1172							1
SSH3	slingshot homolog 3 (Drosophila)	11q13.2	-0.686	1.00E-05	5.00E-04							1
SSMEM1	Data not found		-0.201	0.04703	0.161							1
SSNA1	Sjogren syndrome nuclear autoantigen 1	9q34.3	-0.505	0.00588	0.0408							1
SSPN	sarcospan	12p11.2				0.49825	0.02575	0.09685				1
SSPO	SCO-spondin homolog (Bos taurus)	7q36.1	-0.264	0.01047	0.0605	-0.379	0.00028	0.01				2
SSR1	signal sequence receptor, alpha	6p24.3				-0.318	0.01021	0.05215				1
CHSY1	chondroitin sulfate synthase 1	15q26.3	0.391	0.00483	0.0356							1
FITM2	fat storage-inducing transmembrane protein 2	20q13.12	0.391	0.03139	0.1243							1
SSR4P1	signal sequence receptor, delta pseudogene 1	21q22.3	-0.247	0.03222	0.1263							1
HINFP	histone H4 transcription factor	11q23.3	0.391	0.00135	0.0145							1
SST	somatostatin	3q28							0.985	3.528	5	1
SSTR1	somatostatin receptor 1	14q13							-0.888	3.11125	8	1
SSTR3	somatostatin receptor 3	22q13.1	-0.313	0.02689	0.1123	-0.501	6.00E-05	0.0056				2
SSTR4	somatostatin receptor 4	20p11.2							0.747	2.72	5	1
SSU72	SSU72 RNA polymerase II CTD phosphatase homolog (S. cerevisiae)	1p36.33				-0.3616	0.00333	0.02906				1
ST13	suppression of tumorigenicity 13 (colon carcinoma) (Hsp70 interacting protein	22q13.2				-0.3985	0.000575	0.0113				1
ST14	suppression of tumorigenicity 14 (colon carcinoma)	11q24-q25							0.799	2.745	6	1
ST18	suppression of tumorigenicity 18 (breast carcinoma) (zinc finger protein)	8q11.23				0.367	7.00E-05	0.0057				1
ST3GAL1	ST3 beta-galactoside alpha-2,3-sialyltransferase 1	8q24.22				0.346	0.02869	0.1057				1
NARS2	asparaginyl-tRNA synthetase 2, mitochondrial (putative)	11q14.1	0.391	0.02374	0.1034							1
ST3GAL4	ST3 beta-galactoside alpha-2,3-sialyltransferase 4	11q24.2	-1.109	0.00093	0.0111							1
ST5	suppression of tumorigenicity 5	11p15	-0.562	0.0148	0.0759	-0.3465	0.001485	0.0212				2
DIP2C	DIP2 disco-interacting protein 2 homolog C (Drosophila)	10p15.3	0.39	0.03531	0.1342							1
ST6GAL2	ST6 beta-galactosamide alpha-2,6-sialyltranferase 2	2q11.2-q12.1				0.3115	0.001655	0.02				1
ST6GALNAC1	ST6 (alpha-N-acetyl-neuraminyl-2,3-beta-galactosyl-1,3)-N-acetylgalactosamir	17q25.1				-0.307	0.00978	0.0566				1
ST6GALNAC2	ST6 (alpha-N-acetyl-neuraminyl-2,3-beta-galactosyl-1,3)-N-acetylgalactosamir	17q25.1	-1.599	7.00E-05	0.0016							1
EMC8	ER membrane protein complex subunit 8	16q24	0.39	0.00392	0.0308							1
ST6GALNAC6	ST6 (alpha-N-acetyl-neuraminyl-2,3-beta-galactosyl-1,3)-N-acetylgalactosamir	9q34.11	-0.5805	0.021635	0.07775							1
RAD1	RAD1 homolog (S. pombe)	5p13.2	0.39	0.02921	0.1184	0.342666667	0.018203333	0.077933333	-0.768	2.463	5	3
ST7L	suppression of tumorigenicity 7 like	1p13.2				-0.386	0.00071	0.0151				1
ST7OT3	ST7 overlapping transcript 3 (non-coding RNA)					-0.321	0.01255	0.0651				1
ST8SIA1	ST8 alpha-N-acetyl-neuraminide alpha-2,8-sialyltransferase 1	12p12.1-p11.2				0.3353	0.001315	0.01663				1
ST8SIA2	ST8 alpha-N-acetyl-neuraminide alpha-2,8-sialyltransferase 2	15q26				0.308	0.00597	0.0431				1
TMEM216	transmembrane protein 216	11q13.1	0.39	0.00159	0.0163							1
ST8SIA5	ST8 alpha-N-acetyl-neuraminide alpha-2,8-sialyltransferase 5	18q21.1				-0.365285714	0.01238	0.059542857				1
ST8SIA6	ST8 alpha-N-acetyl-neuraminide alpha-2,8-sialyltransferase 6	10p12.33	-0.556	0.00093	0.0111	-0.322	0.01361	0.0682				2
STAB1	stabilin 1	3p21.1				-0.3865	0.002155	0.02515				1
STAB2	stabilin 2	12q23.3				0.345	0.00066	0.0146				1
STAC2	SH3 and cysteine rich domain 2	17q12	-0.26	0.01238	0.0676	-0.486	0.0016	0.022				2
STAG1	stromal antigen 1	3q22.3				-0.322666667	0.006616667	0.0453				1
STAG3	stromal antigen 3	7q22.1				-0.38	0.00678	0.0463				1
CDC25C	cell division cycle 25 homolog C (S. pombe)	5q31	0.389	0.04367	0.1537							1
DGAT1	diacylglycerol O-acyltransferase 1	8q24.3	0.388	0.00875	0.0533							1
GOLGA8I	Data not found		0.388	0.02678	0.1121							1
STAM	signal transducing adaptor molecule (SH3 domain and ITAM motif) 1	10p14-p13	-0.415	0.02515	0.1075							1
POU2F1	POU class 2 homeobox 1	1q24.2	0.388	0.04697	0.1609	0.361	0.000335	0.01075				2
STAP1	signal transducing adaptor family member 1	4q13.2				0.301	0.00327	0.0314				1
STAP2	signal transducing adaptor family member 2	19p13.3	-0.789	0.01056	0.0608	-0.513	0.002463333	0.023966667				2
STAR	steroidogenic acute regulatory protein	8p11.2	-0.486	5.00E-05	0.0013	-0.388	0.001895	0.02245				2
SMG5	smg-5 homolog, nonsense mediated mRNA decay factor (C. elegans)	1q21.2	0.388	0.03299	0.1282							1
ZNF107	In multiple Geneids		0.387	0.02899	0.1179							1
ADNP	activity-dependent neuroprotector homeobox	20q13.13	0.386	0.01374	0.0722	0.404	0.00576	0.0423				2
C1GALT1C1	C1GALT1-specific chaperone 1	Xq24	0.386	0.02304	0.1015							1
STARD5	In multiple Geneids		-1.238	0.00058	0.008							1
STARD6	In multiple Geneids								-0.939	3.42143	7	1
NOD1	nucleotide-binding oligomerization domain containing 1	7p15-p14	0.386	0.02149	0.0973							1
PIGM	phosphatidylinositol glycan anchor biosynthesis, class M	1q23.2	0.386	0.02836	0.1163							1
SMARCA5	SWI/SNF related, matrix associated, actin dependent regulator of chromatin, s	4q31.1-q31.2	0.386	0.01607	0.0802							1
STAT3	signal transducer and activator of transcription 3 (acute-phase response facto	17q21.31				-0.325333333	0.00206	0.0233				1
SOX30	SRY (sex determining region Y)-box 30	5q33	0.386	0.01544	0.078							1
STAT5B	signal transducer and activator of transcription 5B	17q11.2				-0.308	0.04395	0.1373				1
STATH	statherin	4q13.3							-0.97	3.40875	8	1
CYP3A7	cytochrome P450, family 3, subfamily A, polypeptide 7	7q21-q22.1	0.385	0.00428	0.0328							1
STAU2	staufen, RNA binding protein, homolog 2 (Drosophila)	8q21.11				0.363	0.01192	0.0632				1
HOXA13	homeobox A13	7p15.2	0.385	0.01897	0.0897							1
LIN52	lin-52 homolog (C. elegans)	14q24.3	0.385	0.02416	0.1047							1
NOS1AP	nitric oxide synthase 1 (neuronal) adaptor protein	1q23.3	0.385	0.00467	0.0348	0.3508	0.000444	0.01196				2

STEAP2	STEAP family member 2, metalloreductase	7q21.13				2.108	0	5.00E-04	-0.962	3.09833	6	2
STEAP4	STEAP family member 4	7q21.12				0.322	0.00015	0.0078				1
RTFDC1	Data not found		0.385	6.00E-04	0.0083							1
STIM1	stromal interaction molecule 1	11p15.5	-1.482	0	0	-0.3564	0.002856	0.02766				2
TEFM	transcription elongation factor, mitochondrial		0.385	0.02082	0.0952							1
ZNF233	zinc finger protein 233	19q13.31	0.385	0.00174	0.0173				-0.946	2.5	5	2
AAMDC	Data not found		0.384	0.02639	0.1111							1
STK11	serine/threonine kinase 11	19p13.3				-0.4255	0.00143	0.02025				1
FNIP1	In multiple Geneids		0.384	0.01014	0.059	1.444	0	0.0014				2
STK17A	serine/threonine kinase 17a	7p13				0.35	0.00157	0.021				1
STK24	serine/threonine kinase 24	13q31.2-q32.3	-0.495	0.03444	0.1318				0.832	3.346	5	2
HIST1H4I	histone cluster 1, H4i	6p21.33	0.384	0.03677	0.1379				-0.807	2.68333	6	2
PHF12	PHD finger protein 12	17q11.2	0.384	0.00636	0.043							1
STK32B	serine/threonine kinase 32B	4p16.2				-0.340961538	0.002126923	0.021419231				1
STK32C	serine/threonine kinase 32C	10q26.3				-0.340166667	0.002023333	0.0207				1
STK33	serine/threonine kinase 33	11p15.3				-0.314	0.00061	0.0141				1
STK35	serine/threonine kinase 35	20p13				0.362	7.00E-05	0.0058				1
PTGES3	prostaglandin E synthase 3 (cytosolic)	12q13.3	0.384	0.03537	0.1343							1
STK38	serine/threonine kinase 38	6p21	-0.303	0.04831	0.1637							1
SPATA2	spermatogenesis associated 2	20q13.13	0.384	0.03715	0.1389							1
STK39	serine threonine kinase 39	2q24.3				0.3215	0.00014	0.00695				1
VMP1	vacuole membrane protein 1	17q23.1	0.384	0.00232	0.0212							1
STK40	serine/threonine kinase 40	1p34.3	-0.61	0.00085	0.0105	-0.363	0.002246667	0.025833333				2
STMN1	stathmin 1	1p36.11				-0.394571429	0.005095714	0.0345				1
STMN2	stathmin-like 2	8q21.13				0.336	1.00E-05	0.0033				1
STOML2	stomatin (EPB72)-like 2	9p13.1				-0.392	1.00E-05	0.0031				1
STOML3	stomatin (EPB72)-like 3	13q13.3							-1.019	3.01	5	1
STON2	stonin 2	14q31.1							-1.006	3.485	6	1
STOX1	storkhead box 1	10q22.1				-0.379166667	0.002283333	0.020683333				1
STOX2	storkhead box 2	4q35.1	-1.024	1.00E-05	4.00E-04	-0.3623125	0.011645	0.05706875				2
ZFP112	zinc finger protein 112 homolog (mouse)	19q13.2	0.384	0.01508	0.0769							1
HELZ2	Data not found		0.383	0.01829	0.0875							1
STRADA	STE20-related kinase adaptor alpha	17q23.3	-0.43	1.00E-04	0.0023							1
STRADB	STE20-related kinase adaptor beta	2q33.1	-1.1165	0.011155	0.06105							1
STRBP	spermatid perinuclear RNA binding protein	9q33.3	-0.579	0.02428	0.1049							1
STRC	In multiple Geneids					-0.37575	0.003025	0.025975				1
STRIP1	Data not found		-0.268	0.01384	0.0726							1
POGZ	pogo transposable element with ZNF domain	1q21.3	0.383	0.00448	0.0339							1
STRN	striatin, calmodulin binding protein	2p22.2	-1.034	7.00E-05	0.0018							1
STRN3	striatin, calmodulin binding protein 3	14q13-q21				-0.345	0.00017	0.0081				1
STRN4	striatin, calmodulin binding protein 4	19q13.2				-0.342333333	0.002673333	0.024033333				1
STS	In multiple Geneids								-0.9	2.73333	6	1
TFAM	transcription factor A, mitochondrial	10q21	0.383	0.04646	0.1598				-0.847	2.48	5	2
STUB1	STIP1 homology and U-box containing protein 1, E3 ubiquitin protein ligase	16p13.3	-0.36	0.01212	0.0667							1
STX10	syntaxin 10	19p13.2				-0.346	0.01638	0.0759				1
STX11	syntaxin 11	6q24.2	-1.018	1.00E-04	0.0022							1
STX12	syntaxin 12	1p35.3	-1.116	0	2.00E-04	-0.43725	0.008925	0.046375				2
STX16	syntaxin 16	20q13.32				0.34575	0.0037625	0.032175				1
STX18	syntaxin 18	4p16.3-p16.2				-0.324142857	0.004994286	0.032228571				1
STX19	syntaxin 19	3q11							-0.861	3.20286	7	1
TMEM120B	transmembrane protein 120B	12q24.31	0.383	0.02958	0.1194							1
STX1B	syntaxin 1B	16p11.2	-0.297	0.00648	0.0435							1
ACVR2B	activin A receptor, type IIB	3p22	0.382	0.04406	0.1548							1
ATR	ataxia telangiectasia and Rad3 related	3q22-q24	0.382	0.01832	0.0876							1
STX5	syntaxin 5	11q12.3				-0.341	0.00268	0.0284				1
STX7	syntaxin 7	6q23.1							-1.059	3.12	5	1
STX8	syntaxin 8	17p12				-0.394173077	0.004988846	0.032875				1
STXBP2	syntaxin binding protein 2	19p13.3-p13.2				-0.46825	0.00081	0.01475				1
STXBP3	syntaxin binding protein 3	1p13.3				-0.376	0.00148	0.0212				1
CSTF3	cleavage stimulation factor, 3' pre-RNA, subunit 3, 77kDa	11p13	0.382	0.01228	0.0672							1
STXBP5	syntaxin binding protein 5 (tomosyn)	6q24.3				0.3	0.00382	0.034				1
STXBP6	syntaxin binding protein 6 (amisyn)	14q12				0.312	0.0083	0.0517				1
STYK1	serine/threonine/tyrosine kinase 1	12p13.2				0.3295	0.001085	0.01515				1
STYX	serine/threonine/tyrosine interacting protein		-0.494	0.00435	0.0332							1
SUCLG1	succinate-CoA ligase, alpha subunit	2p11.2				0.332	0.000175	0.008				1
SUCLG2	succinate-CoA ligase, GDP-forming, beta subunit	3p14.1				-0.306	0.00291	0.0296				1
SUCNR1	succinate receptor 1	3q25.1							-0.828	3.28333	6	1
SUDS3	suppressor of defective silencing 3 homolog (S. cerevisiae)	12q24.23				-0.429	0.00019	0.0086				1
SUFU	suppressor of fused homolog (Drosophila)	10q24.32	-0.294	0.01047	0.0605	-0.372411765	0.001960588	0.022076471				2

DYNLRB1	dynein, light chain, roadblock-type 1	20q11.21	0.382	0.00513	0.0372							1
SUGT1P	suppressor of G2 allele of SKP1 pseudogene (S. cerevisiae)	9p12				-0.397184211	0.001436316	0.014986842				1
NUB1	negative regulator of ubiquitin-like proteins 1	7q36	0.382	0.04447	0.1557							1
SUHW1	Data not found								-0.845	3.688	5	1
ORMDL1	ORM1-like 1 (S. cerevisiae)	2q32	0.382	0.02012	0.093				-0.804	3.18667	6	2
TAF1D	TATA box binding protein (TBP)-associated factor, RNA polymerase I, D, 41kDa	11q21	0.382	0.01506	0.0768							1
SULT1A3	sulfotransferase family, cytosolic, 1A, phenol-preferring, member 3	16p11.2							1.034	3.372543333	6.333333333	1
SULT1A4	sulfotransferase family, cytosolic, 1A, phenol-preferring, member 4								0.923	3.12625	8	1
ZFAS1	Data not found		0.382	0.02032	0.0937							1
ZNF235	zinc finger protein 235	19q13.2	0.382	0.0315	0.1246							1
ADAMTS5	ADAM metallopeptidase with thrombospondin type 1 motif, 5	21q21.3	0.381	0.03489	0.1331							1
SULT2A1	sulfotransferase family, cytosolic, 2A, dehydroepiandrosterone (DHEA)-prefer	19q13.3							-0.822	3.17333	6	1
SULT2B1	sulfotransferase family, cytosolic, 2B, member 1	19q13.3	-2.501	2.00E-05	7.00E-04	-0.358333333	0.01869	0.070766667				2
SULT4A1	sulfotransferase family 4A, member 1	22q13.2				-0.399375	0.00056375	0.0104125				1
SUMF1	sulfatase modifying factor 1	3p26.1				-0.324666667	0.003836667	0.030133333				1
RFWD2	ring finger and WD repeat domain 2, E3 ubiquitin protein ligase	1q25.1-q25.2	0.381	0.02277	0.1007	0.327	0.000595	0.01385				2
ZNF652	zinc finger protein 652	17q21.32	0.381	0.01879	0.0891							1
SUMO2	SMT3 suppressor of mif two 3 homolog 2 (S. cerevisiae)	17q25.1				-0.339	0.00969	0.0563				1
SUMO3	SMT3 suppressor of mif two 3 homolog 3 (S. cerevisiae)	21q22.3				-0.474	0	0.0022				1
HNRNPA3	heterogeneous nuclear ribonucleoprotein A3	2q31.2	0.38	0.01037	0.0601							1
SUN2	Sad1 and UNC84 domain containing 2	22q13.1	-0.543	0.00051	0.0073							1
SUNC1	Data not found					0.318	0.03442	0.117966667				1
SUOX	sulfite oxidase	12q13.2				-0.3585	0.00776	0.04975				1
MLH3	mutL homolog 3 (E. coli)	14q24.3	0.38	0.03721	0.1391							1
PPP3CA	protein phosphatase 3, catalytic subunit, alpha isozyme	4q24	0.38	0.03234	0.1266							1
TIGD1	tigger transposable element derived 1	2q37.1	0.38	0.04753	0.162							1
SUPT5H	suppressor of Ty 5 homolog (S. cerevisiae)	19q13				-0.338	0.00014	0.0075				1
CEP78	centrosomal protein 78kDa	9q21.2	0.379	0.04558	0.1583							1
KIAA0226L	KIAA0226-like	13q14.13	0.379	0.02136	0.097							1
SUPV3L1	suppressor of var1, 3-like 1 (S. cerevisiae)	10q22.1							-0.966	3.108	5	1
SURF1	surfeit 1	9q34.2				-0.305	0.00503	0.0394				1
SEPT2	septin 2	2q37	0.379	0.00964	0.0569							1
SUZ12	suppressor of zeste 12 homolog (Drosophila)	17q11.2	0.379	0.00691	0.0455							1
SUSD3	sushi domain containing 3	9q22.31				-0.376	0.00728	0.0481				1
SUSD4	sushi domain containing 4	1q41				0.354	0.00073	0.0153				1
SUSD5	sushi domain containing 5	3p22.3				-0.3375	8.00E-04	0.0158				1
SUV420H2	suppressor of variegation 4-20 homolog 2 (Drosophila)	19q13.42				-0.332	0.01395	0.0691				1
ZNF609	zinc finger protein 609	15q22.31	0.379	0.00927	0.0554							1
SUZ12P	Data not found								0.646	2.77167	6	1
COX11	COX11 cytochrome c oxidase assembly homolog (yeast)	17q22	0.378	0.02067	0.09075							1
ENTPD4	In multiple Geneids		0.378	0.0098	0.0576							1
SV2B	synaptic vesicle glycoprotein 2B	15q26.1				0.321	0.00111	0.0185				1
SVOP	SV2 related protein homolog (rat)	12q24.11				-0.352	0.00092	0.015666667				1
SVOPL	SVOP-like	7q34				-0.403333333	0.01094	0.056183333				1
SWAP70	SWAP switching B-cell complex 70kDa subunit	11p15				-0.326	0.00443	0.033833333				1
FANCC	Fanconi anemia, complementation group C	9q22.3	0.378	0.00667	0.0444	0.572	0	0.0023				2
SYCE1	synaptonemal complex central element protein 1	10q26.3	-0.315	0.00298	0.0255							1
SYCE2	synaptonemal complex central element protein 2	19p13.2	-0.193	0.01509	0.0769	-0.380428571	0.014317143	0.068928571				2
SYCP1	synaptonemal complex protein 1	1p13-p12				-0.33	0.00232	0.0264				1
MAP4K2	mitogen-activated protein kinase kinase kinase kinase 2	11q13	0.378	0.01452	0.0748							1
SYDE1	synapse defective 1, Rho GTPase, homolog 1 (C. elegans)	19p13.12				-0.564	0	0.0015				1
MARVELD3	MARVEL domain containing 3	16q22.2	0.378	0.00589	0.0409							1
SYF2	SYF2 homolog, RNA splicing factor (S. cerevisiae)	1p36.11				-0.329	0.00656	0.0454				1
SYMPK	symplekin	19q13.3				-0.341	0.00188	0.0238				1
SYN1	synapsin I	Xp11.23				0.311	0.00029	0.0102				1
SYN2	synapsin II	3p25				-0.33	0.02105	0.0879				1
SYN3	synapsin III	22q12.3				1.557	0	0	-0.847	3.02667	6	2
SYNC	syncoilin, intermediate filament protein	1p35.1				-0.336333333	0.017566667	0.073733333				1
SYNCRIP	synaptotagmin binding, cytoplasmic RNA interacting protein	6q14-q15	-0.314	0.00377	0.03							1
SYNDIG1L	synapse differentiation inducing 1-like	14q24.3	-0.295	0.00417	0.0322							1
SYNE1	spectrin repeat containing, nuclear envelope 1	6q25							-0.844333333	2.731523333	5.666666667	1
SYNE2	spectrin repeat containing, nuclear envelope 2	14q23.2							-0.782	2.74	5	1
SYNGAP1	synaptic Ras GTPase activating protein 1	6p21.3							0.618	2.42667	6	1
SYNGR1	synaptogyrin 1	22q13.1	-1.308	0	0	-0.474222222	0.002883333	0.021766667				2
OSM	oncostatin M	22q12.2	0.378	0.01617	0.0805							1
SYNGR4	synaptogyrin 4	19q13.3				-0.307	0.00511	0.0396				1
SYNJ1	synaptojanin 1	21q22.2				-0.332	5.00E-05	0.0052				1
PNN	pinin, desmosome associated protein	14q21.1	0.378	0.0317	0.125							1
SYNPO	synaptopodin	5q33.1				-0.314	6.00E-05	0.00545				1

SYNPO2	synaptopodin 2	4q26							-1.086	2.58	7	1
SYNPO2L	synaptopodin 2-like	10q22.2	-1.578	4.00E-05	0.0012							1
SYNPR	In multiple Geneids					0.536	3.00E-05	0.0041				1
SYNRG	synergin, gamma	17q12				-0.307	0.00085	0.0164				1
SYPL1	synaptophysin-like 1	7q22.3	-0.476	0.01	0.0584							1
PPP2R5B	protein phosphatase 2, regulatory subunit B', beta	11q12	0.378	0.02823	0.1159							1
SSRP1	structure specific recognition protein 1	11q12	0.378	0.01083	0.0617							1
ZNF507	zinc finger protein 507	19q13.11	0.378	0.02856	0.1168							1
SYT12	synaptotagmin XII	11q13.2				-0.3955	0.00117	0.01885				1
ACAA1	acetyl-CoA acyltransferase 1	3p22.2	0.377	0.03236	0.1267							1
SYT14	synaptotagmin XIV	1q32.2				0.345777778	0.002772222	0.025188889				1
SYT14L	synaptotagmin XIV-like	4q13.2	-1.065	0.00162	0.0165							1
SYT15	synaptotagmin XV	10q11.1	-0.744	4.00E-05	0.0012							1
SYT16	synaptotagmin XVI	14q23.2							-0.958	3.425	6	1
SYT2	In multiple Geneids					-0.309	0.00369	0.0334				1
SYT3	In multiple Geneids					-0.3845	0.001485	0.0197				1
SYT4	synaptotagmin IV	18q12.3							-0.865	2.576	5	1
SYT5	synaptotagmin V	19q				-0.394	0.00434	0.0363				1
SYT6	synaptotagmin VI	1p13.2				-0.342666667	0.004666667	0.0305				1
SYT7	synaptotagmin VII	11q12-q13.1				-0.383	0.001156667	0.014				1
SYT8	synaptotagmin VIII	11p15.5							0.547	2.57225	5.5	1
SYT9	In multiple Geneids					-0.316	0.002	0.0246				1
SYTL1	synaptotagmin-like 1	1p36.11	-1.219	0	0	-0.321	0.00233	0.0265				2
GTF2E1	general transcription factor IIE, polypeptide 1, alpha 56kDa	3q21-q24	0.377	0.03204	0.1259							1
SYTL3	synaptotagmin-like 3	6q25.3				-0.343	0.00135	0.019475				1
SYTL5	synaptotagmin-like 5	Xp21.1				0.355	0.00523	0.038666667	-0.806	2.85571	7	2
SYVN1	synovial apoptosis inhibitor 1, synoviolin	11q13				-0.311	0.00374	0.0337				1
T	T, brachyury homolog (mouse)	6q27				-0.426	1.00E-04	0.0065				1
T1560	In multiple Geneids		-0.272	0.02612	0.1104							1
T2R55	Data not found								-0.938	3.68	5	1
TAAR5	trace amine associated receptor 5	6q23							-0.969	2.97833	6	1
TAAR6	trace amine associated receptor 6	6q23.2							-0.676	2.53	5	1
TAAR8	trace amine associated receptor 8	6q23.2							-0.846	3.14714	7	1
TAAR9	trace amine associated receptor 9 (gene/pseudogene)	6q23.2							-0.899	2.92167	6	1
TAB2	TGF-beta activated kinase 1/MAP3K7 binding protein 2	6q25.1	-0.468	0.00778	0.0493							1
TAB3	TGF-beta activated kinase 1/MAP3K7 binding protein 3	Xp21.2	-1.142	2.00E-05	7.00E-04							1
TAC1	tachykinin, precursor 1	7q21-q22							0.636	2.676	5	1
TACC2	transforming, acidic coiled-coil containing protein 2	10q26				-0.379583333	0.0026475	0.021875				1
TACC3	transforming, acidic coiled-coil containing protein 3	4p16.3				-0.3865	0.00288	0.02795				1
TACR1	tachykinin receptor 1	2p12				0.379333333	4.00E-05	0.004266667				1
TACR3	tachykinin receptor 3	4q25				-0.371	0.00183	0.0235				1
TACSTD2	tumor-associated calcium signal transducer 2	1p32	-1.558	0.00952	0.0565							1
TADA1L	Data not found								-0.784	2.534	5	1
TADA2B	transcriptional adaptor 2B	4p16.1	-0.656	2.00E-05	8.00E-04							1
TADA2L	Data not found					-0.325	0.00248	0.0273				1
WDR6	WD repeat domain 6	3p21.31	0.377	0.04967	0.1664							1
TAF12	TAF12 RNA polymerase II, TATA box binding protein (TBP)-associated factor, 2	1p35.3				-0.40625	0.007105	0.046425				1
TAF15	TAF15 RNA polymerase II, TATA box binding protein (TBP)-associated factor, 6	17q11.1-q11.2				-0.374	2.00E-04	0.0088				1
CACFD1	calcium channel flower domain containing 1	9q34	0.376	0.01207	0.0665							1
ERLIN2	ER lipid raft associated 2	8p11.2	0.376	0.00559	0.0394							1
MESDC2	mesoderm development candidate 2	15q13	0.376	0.01393	0.0729							1
SLC6A12	solute carrier family 6 (neurotransmitter transporter, betaine/GABA), membe	12p13	0.376	0.0223	0.0995							1
TAF4B	TAF4b RNA polymerase II, TATA box binding protein (TBP)-associated factor, 1	18q11.2				-0.329	0.00122	0.0194				1
TAF5	TAF5 RNA polymerase II, TATA box binding protein (TBP)-associated factor, 10	10q24-q25.2				-0.308	0.00043	0.0121				1
ARF4	ADP-ribosylation factor 4	3p21.2-p21.1	0.375	0.0073	0.0472							1
C1orf43	chromosome 1 open reading frame 43	1q21.2	0.375	0.03496	0.1333							1
TAF9	TAF9 RNA polymerase II, TATA box binding protein (TBP)-associated factor, 32	5q11.2-q13.1							-0.8082	3.0088	7	1
TAF9B	TAF9B RNA polymerase II, TATA box binding protein (TBP)-associated factor, 3	Xq13.1-q21.1				-0.639	8.00E-05	0.0061				1
TAGLN3	transgelin 3	3q13.2	-0.27	0.0176	0.0852							1
TAIP-2	Data not found								-0.95	3.19	9	1
TAL1	T-cell acute lymphocytic leukemia 1	1p32							1.072	2.923	5	1
TALDO1	transaldolase 1	11p15.5-p15.4	-0.559	0.004	0.0313	-0.3365	0.0073175	0.039475				2
TANC1	tetratricopeptide repeat, ankyrin repeat and coiled-coil containing 1	2q24.2				0.351	0.00084	0.0163				1
TANK	TRAF family member-associated NFKB activator	2q24-q31				0.367	0.00013	0.0074	-0.905	2.71429	7	2
TAOK3	TAO kinase 3	12q				-0.358	7.00E-05	0.0058				1
DDHD2	DDHD domain containing 2	8p11.23	0.375	0.01529	0.0776							1
RTEL1	regulator of telomere elongation helicase 1	20q13.3	0.375	0.00696	0.0456							1
SCUBE3	signal peptide, CUB domain, EGF-like 3	6p21.3	0.375	0.00979	0.0576							1
TRPC4AP	transient receptor potential cation channel, subfamily C, member 4 associatec	20q11.22	0.375	0.00211	0.0198							1

TARBP2	TAR (HIV-1) RNA binding protein 2	12q12-q13							-0.786	2.416	5	1
TARP	TCR gamma alternate reading frame protein	7p15-p14							-0.952	2.99167	6	1
TARS	threonyl-tRNA synthetase	5p13.2				0.338	0.01114	0.0608				1
TAS1R1	taste receptor, type 1, member 1	1p36.23				-0.406	0.00085	0.0163				1
TAS1R2	taste receptor, type 1, member 2	1p36.13	-0.25	0.02104	0.0959	-0.332	0.002205	0.02575				2
TAS1R3	taste receptor, type 1, member 3	1p36.33							0.854	3.295	6	1
TAS2R1	taste receptor, type 2, member 1	5p15							-0.925	3.00222	9	1
TAS2R10	taste receptor, type 2, member 10	12p13							-0.879	2.88	7	1
TAS2R13	taste receptor, type 2, member 13	12p13							-1.133	3.838	5	1
DYNC1I1	dynein, cytoplasmic 1, intermediate chain 1	7q21.3-q22.1	0.374	0.00852	0.0523	0.319333333	0.002173333	0.023883333				2
TAS2R16	taste receptor, type 2, member 16	7q31.1-q31.3							-0.872	3.16333	6	1
KLF10	Kruppel-like factor 10	8q22.2	0.374	0.01022	0.0595				-0.521	2.44143	7	2
POLR3F	polymerase (RNA) III (DNA directed) polypeptide F, 39 kDa	20p11.23	0.374	0.03369	0.1299							1
TAS2R3	taste receptor, type 2, member 3	7q31.3-q32							-0.844	2.956	5	1
EPRS	glutamyl-prolyl-tRNA synthetase	1q41	0.373	0.006	0.0414							1
TAS2R39	taste receptor, type 2, member 39	7q34							-0.87	2.605	6	1
TAS2R40	taste receptor, type 2, member 40	7q34							-0.782	2.99444	9	1
TAS2R41	taste receptor, type 2, member 41	7q35				-0.546	0.00149	0.0212				1
RPS27	ribosomal protein S27	1q21	0.373	0.014625	0.07365							1
TAS2R48	Data not found								-0.974	3.30429	7	1
TAS2R49	Data not found								-0.992	3.49167	6	1
SUPT6H	suppressor of Ty 6 homolog (S. cerevisiae)	17q11.2	0.373	0.00894	0.0541							1
TAS2R7	taste receptor, type 2, member 7	12p13				0.308	0.00015	0.0077	-0.933	3.15857	7	2
TAS2R8	taste receptor, type 2, member 8	12p13							-0.883	3.01	8	1
TAS2R9	taste receptor, type 2, member 9	12p13				0.347	0.00048	0.0127	-0.847	2.84857	7	2
AFAP1L1	actin filament associated protein 1-like 1	5q32	0.372	0.0037	0.0297							1
GRINA	glutamate receptor, ionotropic, N-methyl D-aspartate-associated protein 1 (gl	8q24.3	0.372	0.03712	0.1388							1
TATDN2	TatD DNase domain containing 2	3p25.3				-0.396	0.000525	0.0107				1
TAX1BP1	Tax1 (human T-cell leukemia virus type I) binding protein 1	7p15				0.358	0.019205	0.07755				1
TAX1BP3	Tax1 (human T-cell leukemia virus type I) binding protein 3	17p13	-0.618	0.00196	0.0188	-0.537	0.00065	0.0145				2
ILF3	interleukin enhancer binding factor 3, 90kDa	19p13.2	0.372	0.00501	0.0365							1
TBC1D10A	TBC1 domain family, member 10A	22q12.2	-0.93	2.00E-05	6.00E-04	-0.377	0.000693333	0.013233333				2
TBC1D10B	TBC1 domain family, member 10B	16p11.2				-0.3125	0.00521	0.0336	0.625	2.512	5	2
MAGOH	mago-nashi homolog, proliferation-associated (Drosophila)	1p32.3	0.372	0.00695	0.0456							1
TBC1D13	TBC1 domain family, member 13	9q34.11				-0.3795	0.014975	0.0681				1
TBC1D14	TBC1 domain family, member 14	4p16.1	-0.858	0.00012	0.0024	-0.402615385	0.000678462	0.013123077				2
PDCD2L	programmed cell death 2-like	19q13.11	0.372	0.01945	0.0911							1
TBC1D19	TBC1 domain family, member 19	4p15.2							-0.82	2.77	7	1
TBC1D2	TBC1 domain family, member 2	9q22.33	-0.816	0.00445	0.0337	-0.334	0.01175	0.0627				2
TBC1D21	TBC1 domain family, member 21	15q24.1	-0.249	0.04228	0.1508	-0.409	0.00013	0.0073				2
TBC1D22A	TBC1 domain family, member 22A	22q13.3	-0.283	0.00431	0.0329	-0.39155	0.00101	0.011715				2
TBC1D24	TBC1 domain family, member 24	16p13.3				-0.436	0.00805	0.04526				1
TBC1D25	TBC1 domain family, member 25	Xp11.23	-0.4025	0.011645	0.061	-0.375	0.0024	0.0269				2
SPATA5	In multiple Geneids		0.372	0.00505	0.0368							1
TBC1D3	TBC1 domain family, member 3	17q12							1.108	2.428	5	1
ASTE1	asteroid homolog 1 (Drosophila)	3q22.1	0.371	0.02955	0.1194							1
BET1	blocked early in transport 1 homolog (S. cerevisiae)	7q21.1-q22	0.371	0.01826	0.0874				-0.751	2.90286	7	2
TBC1D3B	TBC1 domain family, member 3B	17q12				-0.427	0.024362857	0.094314286				1
TBC1D3C	TBC1 domain family, member 3C	17q12	-0.47	0.02623	0.1107	-0.56	0.00158	0.016633333	1.108	2.428	5	3
TBC1D5	TBC1 domain family, member 5	3p24.3				0.801	3.00E-05	0.0045	-0.799	2.664	5	2
CHD2	chromodomain helicase DNA binding protein 2	15q26	0.371	0.01898	0.0897							1
TBC1D9B	TBC1 domain family, member 9B (with GRAM domain)	5q35.3	-0.396	0.00065	0.0087	-0.354	0.001425	0.0189				2
TBCA	tubulin folding cofactor A	5q14.1							-0.912	3.01	5	1
TBCD	tubulin folding cofactor D	17q25.3				-0.3185	0.00918	0.05095				1
TBCE	tubulin folding cofactor E	1q42.3				0.482	0.03516	0.1196				1
TBCEL	tubulin folding cofactor E-like	11q23.3				0.3385	0.0038	0.0311				1
TBCK	TBC1 domain containing kinase	4q24	-0.51	0.02417	0.1047							1
FAM89A	family with sequence similarity 89, member A	1q42.2	0.371	0.04954	0.1662							1
TBKBP1	TBK1 binding protein 1	17q21.32	-0.381	0.0032	0.0268							1
TBL1X	transducin (beta)-like 1X-linked	Xp22.3	-0.831	0.02034	0.0938	-0.329	0.00181	0.0234				2
TBL1XR1	transducin (beta)-like 1 X-linked receptor 1	3q26.32				0.3915	0.001015	0.0175				1
TBL1Y	transducin (beta)-like 1, Y-linked	Yp11.2	-0.687	0	1.00E-04	-0.453606061	0.01944697	0.080721212				2
GNA13	guanine nucleotide binding protein (G protein), alpha 13	17q24.3	0.371	0.00782	0.0494							1
TBL3	transducin (beta)-like 3	16p13.3	-0.507	0.00064	0.0086							1
OSGEP	O-sialoglycoprotein endopeptidase	14q11.2	0.371	0.03978	0.145							1
TBPL2	In multiple Geneids					-0.368	0.00107	0.0182				1
SPPL2B	signal peptide peptidase like 2B	19p13.3	0.371	0.00708	0.0462							1
TMEM9	transmembrane protein 9		0.371	0.02249	0.0999							1
TBX20	T-box 20	7p14.3				0.365	0.00139	0.0206				1

TBX21	T-box 21	17q21.32	-0.257	0.00905	0.0545	-0.511	0	0.0012				2
LOC100287896	uncharacterized LOC100287896	11q13.4	0.37	0.04963	0.1664							1
TBX4	T-box 4	17q21-q22				-0.321	0.00596	0.0431				1
TBX5	T-box 5	12q24.1				-0.397	0.00278	0.0289	0.603666667	2.859523333	6.666666667	2
TBX6	T-box 6	16p11.2	-0.873	2.00E-05	6.00E-04							1
TBXAS1	thromboxane A synthase 1 (platelet)	7q34-q35				-0.347571429	0.004001429	0.030914286				1
tcag7.1017	Data not found								0.998	3.42	5	1
TCAIM	Data not found		-0.612	0.00089	0.0108							1
TCEA2	transcription elongation factor A (SII), 2	20q13.33							0.6	2.737666667	5	1
TCEA3	transcription elongation factor A (SII), 3	1p36.12				-0.36025	0.00164	0.020525				1
TCEAL5	transcription elongation factor A (SII)-like 5	Xq22.1							-1.018	2.95833	6	1
TCEAL8	transcription elongation factor A (SII)-like 8	Xq22.1							-0.803	2.77	6	1
TCEB1	transcription elongation factor B (SIII), polypeptide 1 (15kDa, elongin C)	8q21.11	-0.225	0.01516	0.0772							1
TCEB2	transcription elongation factor B (SIII), polypeptide 2 (18kDa, elongin B)	16p12.3				-0.594	0.00153	0.0215				1
TCEB3	transcription elongation factor B (SIII), polypeptide 3 (110kDa, elongin A)	1p36.1	-0.448	0.00761	0.0486	-0.42725	0.002365	0.023375				2
TCEB3B	transcription elongation factor B polypeptide 3B (elongin A2)	18q21.1	-0.279	0.01658	0.0818							1
TCEB3C	transcription elongation factor B polypeptide 3C (elongin A3)	18q21.1							0.7945	2.974	5	1
TCERG1L	transcription elongation regulator 1-like	10q26.3	-0.263	0.00704	0.0461	-0.366	0.002465	0.02605				2
MTERFD1	MTERF domain containing 1	8q22.1	0.37	0.00469	0.0349							1
TCF15	transcription factor 15 (basic helix-loop-helix)	20p13	-0.232	0.029	0.1179							1
TCF20	transcription factor 20 (AR1)	22q13.3				-0.4099	0.0021015	0.01711				1
TCF25	transcription factor 25 (basic helix-loop-helix)	16q24.3				-0.4085	0.00869	0.0491				1
ZNF182	In multiple Geneids		0.37	0.031	0.1234							1
TCF4	transcription factor 4	18q21.1				-0.313	0.00795	0.05045	1.358	3.37357	7	2
FEN1	flap structure-specific endonuclease 1	11q12	0.369	0.01041	0.0602							1
GKAP1	G kinase anchoring protein 1	9q21.32	0.369	0.02549	0.1086							1
INPP5B	inositol polyphosphate-5-phosphatase, 75kDa	1p34	0.369	0.00491	0.0361							1
TCHHL1	trichohyalin-like 1	1q21.3	-0.229	0.01598	0.0799							1
TCHP	trichoplein, keratin filament binding	12q24.11	-0.395	2.00E-04	0.0036	-0.3465	0.000365	0.0102				2
WRAP53	WD repeat containing, antisense to TP53	17p13.1	0.369	0.03774	0.1402							1
TCL1A	T-cell leukemia/lymphoma 1A	14q32.1				-0.336	0.00055	0.0134				1
TCL1B	T-cell leukemia/lymphoma 1B	14q32.1				-0.366	0.00696	0.0469				1
AGO1	Data not found		0.368	0.0483	0.1637							1
TCOF1	Treacher Collins-Franceschetti syndrome 1	5q32				-0.391571429	9.14E-05	0.0059				1
TCP11L2	t-complex 11 (mouse)-like 2	12q23.3	-2.294	0	0							1
TCTE1	In multiple Geneids		-0.202	0.03073	0.1226							1
TCTEX1D4	Tctex1 domain containing 4	1p34.1	-0.194	0.04469	0.1561							1
MTCH1	mitochondrial carrier 1	6p21.2	0.368	0.03656	0.1375							1
TDH	In multiple Geneids					0.519	0.02688	0.1016				1
NEK4	In multiple Geneids		0.368	0.03843	0.142							1
TDP1	tyrosyl-DNA phosphodiesterase 1	14q32.11				-0.323	0.00048	0.0126				1
NOL10	nucleolar protein 10	2p25.1	0.368	0.03885	0.1428							1
TDRD1	tudor domain containing 1	10q25.3							-0.7995	2.668665	5.5	1
TDRD12	tudor domain containing 12	19q13.11				-0.355	0.00275	0.0287				1
TDRD3	tudor domain containing 3	13q21.2	-0.406	0.01926	0.0906							1
RBM18	RNA binding motif protein 18	9q33.2	0.368	0.02645	0.1112							1
TDRD7	tudor domain containing 7	9q22.33							-1.071	3.34167	6	1
TDRD9	tudor domain containing 9	14q32.33				-0.3672	0.003542	0.03176				1
TDRG1	testis development related 1 (non-protein coding)	6p21.2	-0.718	0.00017	0.0033							1
RNF32	ring finger protein 32	7q36	0.368	0.02954	0.1194							1
TDRP	Data not found		-0.331	0.00596	0.0411							1
TEAD1	TEA domain family member 1 (SV40 transcriptional enhancer factor)	11p15.2	-0.993	2.00E-04	0.0036							1
TEAD2	TEA domain family member 2	19q13.3				-0.374666667	0.002203333	0.025366667				1
FKBP1A	FK506 binding protein 1A, 12kDa	20p13	0.3675	0.00667	0.04435							1
TEC	In multiple Geneids		-0.969	1.00E-05	5.00E-04							1
TECPR2	tectonin beta-propeller repeat containing 2	14q32.31				-0.33975	0.00249625	0.025675				1
TECR	trans-2,3-enoyl-CoA reductase	19p13.12	-1.0715	1.00E-05	0.00035	-0.414	0.00333	0.030266667				2
TECRL	trans-2,3-enoyl-CoA reductase-like	4q13.1				0.332	0.00755	0.049				1
TECTA	tectorin alpha	11q22-q24				0.304	0.00443	0.0367				1
TEDDM1	transmembrane epididymal protein 1	1q25.3	-0.256	0.00921	0.0552							1
TEF	thyrotrophic embryonic factor	22q13.2	-0.265	0.01144	0.064	-0.478571429	0.001528571	0.016657143				2
FBXO21	F-box protein 21	12q24.22	0.367	0.00881	0.0535							1
TEK	TEK tyrosine kinase, endothelial	9p21							-1.016	3.97143	7	1
TEKT3	tektin 3	17p12				-0.347	0.02576	0.0991				1
TEKT4	tektin 4	2q11.1	-0.3295	0.020935	0.07915							1
TEKT4P2	tektin 4 pseudogene 2	21p11.2	-0.659	0.00214	0.02							1
TEKT5	tektin 5	16p13.13				-0.321666667	0.006013333	0.042633333				1
TELO2	TEL2, telomere maintenance 2, homolog (S. cerevisiae)	16p13.3				-0.493	0.00415	0.0355				1
TEN1	TEN1 telomerase capping complex subunit homolog (S. cerevisiae)	17q25.1	-0.802	0.00019	0.0036							1

TENC1	tensin like C1 domain containing phosphatase (tensin 2)	12q13.13				-0.37	0.000175	0.008				1
TENM2	Data not found		-1.876	0	1.00E-04							1
TENM4	Data not found		-0.815	0.00029	0.0048							1
H2AFV	H2A histone family, member V	7p13	0.367	0.02731	0.1134							1
TEPP	testis, prostate and placenta expressed	16q21	-0.288	0.00782	0.0494							1
SCAMP3	secretory carrier membrane protein 3	1q21	0.367	0.04041	0.1465							1
TERF2	telomeric repeat binding factor 2	16q22.1				-0.4	0.004806667	0.03655				1
SCLY	selenocysteine lyase	2q37.3	0.367	0.02396	0.104							1
SEMA4F	sema domain, immunoglobulin domain (Ig), transmembrane domain (TM) anc	2p13.1	0.367	0.02286	0.101	0.363	0.000625	0.012				2
TESK1	testis-specific kinase 1	9p13				-0.384	0.00067	0.0146				1
TESSP2	Data not found					-0.326	0.00089	0.0167				1
TESSP5	testis serine protease 5					-0.5	3.00E-05	0.0041				1
TET1	tet methylcytosine dioxygenase 1	10q21				-0.385818182	0.000976364	0.013872727				1
TET2	tet methylcytosine dioxygenase 2	4q24	-1.1255	5.00E-05	0.00135							1
ZNF664	zinc finger protein 664	12q24.31	0.367	0.00624	0.0424							1
TEX101	testis expressed 101	19q13.31	-0.446	0.00042	0.0063	-0.306	0.00823	0.0514				2
TEX11	testis expressed 11	Xq13.1				0.326	0.0014	0.0206				1
TEX12	testis expressed 12	11q22	-0.322	0.00102	0.0119							1
TEX13A	testis expressed 13A	Xq22.3	-0.19	0.04363	0.1537							1
TEX14	testis expressed 14	17q22				-0.371	0.00467	0.0378				1
TEX15	testis expressed 15	8p12							-0.782	2.97875	8	1
TEX2	testis expressed 2	17q23.3	-0.736	0.00252	0.0225							1
TEX264	testis expressed 264	3p21.31	-0.224	0.01998	0.0926	-0.4022	0.001058	0.01478				2
TEX28	testis expressed 28	Xq28				0.376	0.0107	0.0594				1
TEX37	Data not found		-0.349	0.00416	0.0322							1
TEX38	Data not found		-0.219	0.01301	0.0698							1
AMN	amnionless homolog (mouse)	14q32.3	0.366	0.01551	0.0782							1
TFAP2A	transcription factor AP-2 alpha (activating enhancer binding protein 2 alpha)	6p24	-1.649	0.0071	0.0463							1
TFAP2B	transcription factor AP-2 beta (activating enhancer binding protein 2 beta)	6p12	-2.07	0	0							1
TFAP2C	transcription factor AP-2 gamma (activating enhancer binding protein 2 gamm	20q13.2	-2.305	4.00E-05	0.001				0.546	2.92417	6	2
TFAP2D	transcription factor AP-2 delta (activating enhancer binding protein 2 delta)	6p12.1				0.35775	0.002225	0.02265				1
TFAP4	transcription factor AP-4 (activating enhancer binding protein 4)	16p13				-0.4675	0.014765	0.0713				1
CHD4	chromodomain helicase DNA binding protein 4	12p13	0.366	0.0054	0.0385							1
MPZL1	myelin protein zero-like 1	1q24.2	0.366	0.02441	0.1053	0.335	0.000695	0.0131				2
TFCP2L1	transcription factor CP2-like 1	2q14				0.321	0.00319	0.031				1
TFDP1	transcription factor Dp-1	13q34				-0.324	0.01839	0.0811				1
TFDP3	transcription factor Dp family, member 3	Xq26.2							0.773	2.862	5	1
NKAP	NFKB activating protein	Xq24	0.366	0.018	0.0865							1
TFEB	transcription factor EB	6p21				0.303	0.00987	0.0568				1
OXTR	oxytocin receptor	3p25	0.366	0.00169	0.0169							1
SFPQ	splicing factor proline/glutamine-rich	1p34.3	0.366	0.01664	0.082							1
TFF2	trefoil factor 2	21q22.3				-0.35	0.00215	0.0254				1
SLC22A4	solute carrier family 22 (organic cation/ergothioneine transporter), member 4	5q31.1	0.366	0.02624	0.1107							1
TFIP11	tuftelin interacting protein 11	22q12.1							-0.675	2.456	5	1
TCFL5	transcription factor-like 5 (basic helix-loop-helix)	20q13.3-qter	0.366	0.03764	0.14							1
WDR70	WD repeat domain 70	5p13.2	0.366	0.04313	0.1527	0.389538462	0.014691538	0.066761538				2
CBX3	chromobox homolog 3	7p15.2	0.365	0.00858	0.0526							1
TG	thyroglobulin	8q24				0.389275862	0.02370069	0.091227586				1
HSP90B2P	heat shock protein 90kDa beta (Grp94), member 2, pseudogene	15q26.3	0.365	0.03929	0.1438							1
TGFA	transforming growth factor, alpha	2p13	-0.903	0.00612	0.0419							1
TGFB1	transforming growth factor, beta 1	19q13.1	-0.577	0.03803	0.141							1
PRPF19	PRP19/PSO4 pre-mRNA processing factor 19 homolog (S. cerevisiae)	11q12.2	0.365	0.03273	0.1276				-0.795	2.886	5	2
TGFB3	transforming growth factor, beta 3	14q24							0.641	2.64667	6	1
TMEM204	transmembrane protein 204	16p13.3	0.365	0.01574	0.0791							1
CRY1	cryptochrome 1 (photolyase-like)	12q23-q24.1	0.364	0.03132	0.1242							1
TGFBR3	transforming growth factor, beta receptor III	1p33-p32				-0.33025	0.0026125	0.027125				1
TGIF	Data not found								-0.530142857	2.358298571	6.857142857	1
PRPF6	PRP6 pre-mRNA processing factor 6 homolog (S. cerevisiae)	20q13.33	0.364	0.02966	0.1197							1
PSMD3	proteasome (prosome, macropain) 26S subunit, non-ATPase, 3	17q21.1	0.364	0.02603	0.1102				-0.707	2.602	5	2
TGM1	transglutaminase 1 (K polypeptide epidermal type I, protein-glutamine-gamm	14q11.2	-4.186	0	1.00E-04							1
MITD1	MIT, microtubule interacting and transport, domain containing 1	2q11.2	0.363	0.0302	0.1212							1
TGM3	transglutaminase 3 (E polypeptide, protein-glutamine-gamma-glutamyltransfe	20q11.2	-5.879	0	0							1
TGM4	transglutaminase 4 (prostate)	3p22-p21.33				-0.4725	0.000715	0.0122				1
TGM5	transglutaminase 5	15q15.2	-2.005	0	0	-0.34	0.00479	0.03625				2
PTPRE	protein tyrosine phosphatase, receptor type, E	10q26	0.363	0.03919	0.1436							1
TH	tyrosine hydroxylase	11p15.5				-0.375	0.00627	0.0443				1
THAP3	In multiple Geneids					-0.3305	0.001755	0.0208				1
THAP4	THAP domain containing 4	2q37.3				0.448555556	0.000916667	0.0154				1
THAP6	THAP domain containing 6	4q21.1	-0.231	0.03765	0.14							1

THAP9	THAP domain containing 9	4q21.22				-0.328	0.00553	0.0413				1
THBD	thrombomodulin	20p11.2	-1.017	0.00027	0.0046							1
SPATA25	spermatogenesis associated 25	20q13.12	0.363	0.03231	0.1266							1
UBR2	ubiquitin protein ligase E3 component n-recognin 2	6p21.1	0.363	0.0415	0.1491							1
ARHGEF7	Rho guanine nucleotide exchange factor (GEF) 7	13q34	0.362	0.02254	0.1001	0.362	0.00201	0.0246				2
THEG	theg spermatid protein	19p13.3	-0.223	0.02034	0.0938	-0.4125	0.02326	0.08035				2
MAP2K5	mitogen-activated protein kinase kinase 5	15q23	0.362	0.00995	0.0582	0.343	0.00049	0.0128				2
THEM5	thioesterase superfamily member 5	1q21.3	-0.859	4.00E-05	0.0012							1
THEMIS	thymocyte selection associated	6q22.33				0.381083333	0.0198425	0.080241667				1
THG1L	tRNA-histidine guanylyltransferase 1-like (S. cerevisiae)	5q33.3				-0.483	4.00E-05	0.0048				1
THNSL1	threonine synthase-like 1 (S. cerevisiae)	10p12.1							-0.801	2.788	5	1
NEDD1	In multiple Geneids		0.362	0.01456	0.0749							1
THOC3	THO complex 3	5q35.2	-0.959	0.00013	0.0026	-0.331	0.0035	0.028033333				2
THOC5	In multiple Geneids					-0.445454545	0.006439091	0.032390909				1
THOP1	thimet oligopeptidase 1	19p13.3				-0.4415	0.003625	0.027525				1
NUS1	nuclear undecaprenyl pyrophosphate synthase 1 homolog (S. cerevisiae)	6q22.1	0.362	0.01445	0.0746							1
THRAP3	thyroid hormone receptor associated protein 3	1p34.3				-0.339	0.008545	0.0444				1
THRB	thyroid hormone receptor, beta	3p24.2				0.348	0.00077	0.0157				1
THSD4	thrombospondin, type I, domain containing 4	15q23	-2.512	0	0							1
THSD7A	thrombospondin, type I, domain containing 7A	7p21.3				0.365139535	0.003696977	0.028181395				1
THSD7B	thrombospondin, type I, domain containing 7B	2q22.1				0.349266667	0.001112667	0.017093333				1
POLR3K	polymerase (RNA) III (DNA directed) polypeptide K, 12.3 kDa	16p13.3	0.362	0.03093	0.1233							1
UHRF1BP1L	UHRF1 binding protein 1-like	12q23.1	0.362	0.0356	0.1349							1
AIP	aryl hydrocarbon receptor interacting protein	11q13.3	0.361	0.00302	0.0257							1
TIAL1	TIA1 cytotoxic granule-associated RNA binding protein-like 1	10q				-0.302	0.0021	0.0251				1
TIAM1	T-cell lymphoma invasion and metastasis 1	21q22.11	-3.026	0	0	-0.39054321	0.001495185	0.016525926				2
TIAM2	T-cell lymphoma invasion and metastasis 2	6q25.2				-0.3485	0.003821667	0.033716667				1
TICAM1	toll-like receptor adaptor molecule 1	19p13.3	-1.179	1.00E-05	5.00E-04	-0.464	0.0134375	0.059875				2
TICAM2	toll-like receptor adaptor molecule 2	5q23.1							-1.068	2.752	5	1
C2CD3	C2 calcium-dependent domain containing 3	11q13.4	0.361	0.00407	0.0317							1
TIFA	TRAF-interacting protein with forkhead-associated domain	4q25	-1.323	0	1.00E-04							1
MIA3	melanoma inhibitory activity family, member 3	1q41	0.361	0.04397	0.1545	0.31	0.00044	0.0123				2
TIGD2	tigger transposable element derived 2	4q22.1							-0.869	3.084	5	1
TIGD6	tigger transposable element derived 6	5q32				-0.335	0.00089	0.0167				1
TIGD7	tigger transposable element derived 7	16p13.3				-0.385	0.01733	0.0733				1
TIMD4	T-cell immunoglobulin and mucin domain containing 4	5q33.3				-0.32275	0.0008525	0.0136				1
TIMELESS	timeless homolog (Drosophila)	12q13.3				-0.362	0.00055	0.0134				1
TAF1	TAF1 RNA polymerase II, TATA box binding protein (TBP)-associated factor, 25	Xq13.1	0.361	0.00578	0.0404							1
TIMM21	translocase of inner mitochondrial membrane 21 homolog (yeast)	18q22.3	-0.461	0.01386	0.0727							1
TIMM22	translocase of inner mitochondrial membrane 22 homolog (yeast)	17p13	-0.29	0.03855	0.1423	-0.335	0.011516667	0.061933333				2
TIMM44	translocase of inner mitochondrial membrane 44 homolog (yeast)	19p13.3-p13.2				-0.418	0.00229	0.021633333				1
TIMM50	translocase of inner mitochondrial membrane 50 homolog (S. cerevisiae)	19q13.2				-0.346	0.00933	0.0551				1
APLN	apelin	Xq25	0.36	0.0242	0.1047							1
TIMP2	TIMP metallopeptidase inhibitor 2	17q25				-0.3	0.02707	0.1021				1
TIMP3	TIMP metallopeptidase inhibitor 3	22q12.3				-0.385666667	0.003	0.021166667				1
E2F1	E2F transcription factor 1	20q11.2	0.36	0.0177	0.0855							1
TINAG	tubulointerstitial nephritis antigen	6p12.1				0.335	0.00201	0.0244				1
HNRNPC	heterogeneous nuclear ribonucleoprotein C (C1/C2)	14q11.2	0.36	0.01659	0.0818							1
TINCR	Data not found		-1.54	0	0							1
TINF2	TERF1 (TRF1)-interacting nuclear factor 2	14q12	-0.899	0	1.00E-04							1
TIPARP	TCDD-inducible poly(ADP-ribose) polymerase	3q25.31				0.36875	0.0056725	0.037925				1
TISP43	In multiple Geneids		-0.636	0.00745	0.0479							1
SETX	senataxin	9q34.13	0.36	0.02245	0.0998							1
TJP1	tight junction protein 1 (zona occludens 1)	15q13	-0.667	0.00255	0.0228	-0.312	0.00574	0.0422				2
TJP2	tight junction protein 2 (zona occludens 2)	9q13-q21				-0.3345	0.019495	0.0826	0.947	2.812	5	2
TJP3	tight junction protein 3 (zona occludens 3)	19p13.3	-0.487	0.04586	0.1587	-0.461166667	0.001653333	0.020766667				2
TK2	thymidine kinase 2, mitochondrial	16q22-q23.1				-0.3845	0.00433	0.032575				1
TKT	transketolase	3p14.3				-0.3625	0.002935	0.0257				1
TLDC1	Data not found		-0.58	0.00222	0.0205							1
TLE1	transducin-like enhancer of split 1 (E(sp1) homolog, Drosophila)	9q21.32				-0.312	0.0147475	0.066975				1
TLE2	transducin-like enhancer of split 2 (E(sp1) homolog, Drosophila)	19p13.3				-0.423	0.00632	0.03572				1
TLE3	transducin-like enhancer of split 3 (E(sp1) homolog, Drosophila)	15q22							-0.782	2.804	5	1
TLE4	transducin-like enhancer of split 4 (E(sp1) homolog, Drosophila)	9q21.31	-1.203	0.00066	0.0088							1
TLE6	transducin-like enhancer of split 6 (E(sp1) homolog, Drosophila)	19p13.3				-0.444333333	0.003993333	0.032766667				1
SNRPD2	small nuclear ribonucleoprotein D2 polypeptide 16.5kDa	19q13.2	0.36	0.00665	0.0443							1
TLN1	talin 1	9p13				-0.40025	0.00335875	0.0216125				1
AATF	apoptosis antagonizing transcription factor	17q12	0.359	0.03198	0.1258				-0.804	3.216	5	2
TLOC1	Data not found								-1.164	3.28833	6	1
TLR1	toll-like receptor 1	4p14							-0.627	2.842	5	1

TLR10	toll-like receptor 10	4p14	-0.261	0.04844	0.164							1
TLR3	toll-like receptor 3	4q35							-0.796	3.07333	6	1
CWF19L2	CWF19-like 2, cell cycle control (S. pombe)	11q22.3	0.359	0.02678	0.1121	0.3385	0.00069	0.01395				2
TLR5	toll-like receptor 5	1q41-q42				0.426	2.00E-05	0.0038				1
TLR7	toll-like receptor 7	Xp22.3							-0.705	2.568	5	1
TLR9	toll-like receptor 9	3p21.3				-0.495	0	0.0022				1
IRF2BP2	interferon regulatory factor 2 binding protein 2	1q42.3	0.359	0.00231	0.0212							1
TM2D1	TM2 domain containing 1	1p31.3				-0.373	0.006015	0.0387				1
MRTO4	mRNA turnover 4 homolog (S. cerevisiae)	1p36.13	0.359	0.03958	0.1445							1
TM4SF1	In multiple Geneids					0.386	0.00015	0.0077				1
NECAP1	NECAP endocytosis associated 1	12p13.31	0.359	0.00849	0.0522				-0.724	2.404	5	2
RARS2	arginyl-tRNA synthetase 2, mitochondrial	6q16.1	0.359	0.01562	0.0787							1
RSPH1	radial spoke head 1 homolog (Chlamydomonas)	21q22.3	0.359	0.01929	0.0906							1
SUPT16H	In multiple Geneids		0.359	0.04447	0.1557							1
ANAPC1	anaphase promoting complex subunit 1	2q12.1	0.358	0.02918	0.1184							1
TM7SF2	transmembrane 7 superfamily member 2	11q13	-1.185	0.00055	0.0077							1
TM7SF3	transmembrane 7 superfamily member 3	12q11-q12				0.312	0.01576	0.0702				1
TM7SF4	Data not found					0.323	2.00E-05	0.0035	-0.767	3.41833	6	2
TM9SF1	transmembrane 9 superfamily member 1	14q11.2				-0.305	0.00394	0.0346				1
TM9SF4	transmembrane 9 superfamily protein member 4	20q11.21							0.836	3.41	6	1
TMA7	translational machinery associated 7 homolog (S. cerevisiae)	3p21.31	-0.449	0.01426	0.074							1
TMC1	transmembrane channel-like 1	9q21.12	-0.213	0.03074	0.1227	-0.302	0.0047	0.0379				2
TMC3	transmembrane channel-like 3	15q25.1				0.349	0.00044	0.0122				1
CCDC91	coiled-coil domain containing 91	12p11.22	0.358	0.00927	0.0554	0.37775	0.00320375	0.02586875	-0.783	2.93714	7	3
EMILIN1	elastin microfibril interfacer 1	2p23.3-p23.2	0.358	0.04373	0.1539							1
PHF20	PHD finger protein 20	20q11.22-q11.23	0.358	0.01878	0.0891							1
SF3B2	splicing factor 3b, subunit 2, 145kDa	11q13.1	0.358	0.00342	0.0281							1
TMCC3	transmembrane and coiled-coil domain family 3	12q22				-0.31775	0.0057825	0.03965				1
FAM222B	family with sequence similarity 222, member B	17q11.2	0.357	0.01433	0.0742							1
TMCO2	transmembrane and coiled-coil domains 2	1p34.2	-0.24	0.0349	0.1331							1
RPTOR	regulatory associated protein of MTOR, complex 1	17q25.3	0.357	0.04506	0.157	0.482	5.00E-05	0.0053				2
TMCO4	transmembrane and coiled-coil domains 4	1p36.13				-0.370625	0.00107	0.0159375				1
TMCO7	transmembrane and coiled-coil domains 7	16q22.1				-0.377115385	0.008372308	0.047096154				1
TMED1	transmembrane emp24 protein transport domain containing 1	19p13.2				-0.444	0.00261	0.028				1
TMED3	transmembrane emp24 protein transport domain containing 3	15q24-q25	-0.332	0.03056	0.1223							1
UBE2S	ubiquitin-conjugating enzyme E2S	19q13.43	0.357	0.03547	0.1346							1
ZNF616	zinc finger protein 616	19q13.41	0.357	0.04899	0.165							1
TMED7	transmembrane emp24 protein transport domain containing 7	5q22.3							-1.068	2.752	5	1
TMED8	transmembrane emp24 protein transport domain containing 8	14q24.3				-0.393	0.001116667	0.016633333				1
TMED9	transmembrane emp24 protein transport domain containing 9	5q35.3				-0.347	0.00144	0.0209				1
CCR8	chemokine (C-C motif) receptor 8	3p22	0.356	0.02707	0.1128							1
TMEFF2	transmembrane protein with EGF-like and two follistatin-like domains 2	2q32.3				0.372	0.000283333	0.007641667				1
TMEM101	transmembrane protein 101	17q21.31				-0.456	0.00237	0.0267				1
TMEM103	Data not found								-0.734	2.62167	6	1
CDK5RAP2	CDK5 regulatory subunit associated protein 2	9q33.2	0.356	0.04001	0.1456							1
TMEM106B	transmembrane protein 106B	7p21.3				0.38	6.00E-04	0.0139				1
SLC25A19	solute carrier family 25 (mitochondrial thiamine pyrophosphate carrier), mem	17q25.3	0.356	0.01309	0.0699							1
TMEM107	transmembrane protein 107	17p13.1				-0.336	0.00814	0.0511				1
TMEM108	transmembrane protein 108	3q21				0.3332	0.004424	0.03198				1
TMEM109	transmembrane protein 109	11q12.2	-0.926	9.00E-05	0.002							1
TMEM11	transmembrane protein 11	17p11.2				-0.364	0.00844	0.0513				1
TMEM110	transmembrane protein 110	3p21.1				-0.343166667	0.003458333	0.030333333				1
TMEM111	Data not found					-0.37175	0.00134	0.01835				1
TMEM115	transmembrane protein 115	3p21.3				-0.3705	0.001255	0.01945				1
TMEM116	transmembrane protein 116	12q24.13	-0.598	0.03378	0.1301	-0.517	1.00E-05	0.0033				2
TMEM117	transmembrane protein 117	12q12				0.331	0.00046	0.0124				1
TMEM12	Data not found								-0.761	2.60167	6	1
TFPT	TCF3 (E2A) fusion partner (in childhood Leukemia)	19q13	0.356	0.00378	0.0301							1
TMEM121	transmembrane protein 121	14q32.33							0.546	2.83333	9	1
ZNF234	zinc finger protein 234	19q13.31	0.356	0.04768	0.1624							1
AEN	apoptosis enhancing nuclease	15q26.1	0.355	0.04882	0.1647							1
ANKRD49	ankyrin repeat domain 49	11q21	0.354	0.02674	0.112							1
CEP120	centrosomal protein 120kDa	5q23.2	0.354	0.01504	0.0768							1
TMEM127	transmembrane protein 127	2q11.2	-0.387	0.02597	0.11							1
TMEM130	transmembrane protein 130	7q22.1				-0.367666667	0.024516667	0.090266667				1
TMEM131	transmembrane protein 131	2q11.2				0.311	0.00487	0.0329				1
TMEM132C	transmembrane protein 132C	12q24.32	-0.248	0.04553	0.1581	-0.302	1.00E-05	0.0028				2
TMEM132D	transmembrane protein 132D	12q24.33				0.620333333	0.000656667	0.008933333				1
MRPL2	In multiple Geneids		0.354	0.04971	0.1665							1

TMEM134	transmembrane protein 134	11q13.2	-0.988	0	0							1
PCMTD2	protein-L-isoaspartate (D-aspartate) O-methyltransferase domain containing 2	20q13.33	0.354	0.0463	0.1596	0.328	0.006495	0.0405				2
TMEM137	transmembrane protein 137	11q13.1							0.556	2.436	5	1
TOP1	topoisomerase (DNA) I	20q12-q13.1	0.354	0.03835	0.1418	0.327333333	0.000116667	0.005966667				2
ZRANB2	zinc finger, RAN-binding domain containing 2	1p31	0.354	0.00892	0.054							1
ATRAID	Data not found		0.353	0.04289	0.1522							1
TMEM141	transmembrane protein 141	9q34.3				-0.317	0.00373	0.0336				1
TMEM143	transmembrane protein 143	19q13.33				-0.4158	0.00243	0.02368				1
DPY19L3	dpy-19-like 3 (C. elegans)	19q13.11	0.353	0.03537	0.1343							1
TMEM146	Data not found					-0.414571429	0.004995714	0.030814286				1
TMEM147	transmembrane protein 147	19q13.1	-0.291	0.01035	0.06							1
TMEM149	Data not found								0.545	2.29	5	1
TMEM14B	transmembrane protein 14B	6p25.1-p23				-0.334	0.01888	0.0824				1
TMEM14C	transmembrane protein 14C	6p24.2				-0.328	0.00052	0.0131				1
EFTUD1	elongation factor Tu GTP binding domain containing 1	15q25.2	0.353	0.01495	0.0764							1
TMEM151B	transmembrane protein 151B	6p21.1	-0.205	0.02229	0.0995							1
TMEM154	transmembrane protein 154	4q31.3	-3.266	0	1.00E-04							1
TMEM156	transmembrane protein 156	4p14				-0.352	0.02054	0.0866				1
TMEM159	transmembrane protein 159	16p12	-0.708	0.02674	0.112	-0.367	0.00125	0.0196				2
TMEM161B	transmembrane protein 161B	5q14.3				0.356	0.01454	0.0708				1
PSMA5	proteasome (prosome, macropain) subunit, alpha type, 5	1p13	0.353	0.01076	0.0615							1
TMEM164	transmembrane protein 164	Xq22.3				0.35	0.01418	0.0698				1
TMEM165	transmembrane protein 165	4q12				-0.3935	0.0033	0.0283				1
TMEM167B	transmembrane protein 167B	1p13.3				-0.417	0.00036	0.0112				1
TMEM170A	transmembrane protein 170A	16q23.1				-0.372	0.00163	0.0222				1
TMEM173	transmembrane protein 173	5q31.2				-0.668	2.00E-05	0.0037				1
TMEM175	transmembrane protein 175	4p16.3				-0.355666667	0.003316667	0.027766667				1
SF3B4	splicing factor 3b, subunit 4, 49kDa	1q21.2	0.353	0.00593	0.041							1
SLC22A5	solute carrier family 22 (organic cation/carnitine transporter), member 5	5q23.3	0.353	0.02507	0.1073							1
CST3	cystatin C	20p11.21	0.352	0.01982	0.0922							1
TMEM178	transmembrane protein 178	2p22.1				0.366	0.000151667	0.006916667				1
TMEM179	transmembrane protein 179	14q32.33				-0.3825	0.00274	0.02675				1
GDF11	In multiple Geneids		0.352	0.04157	0.1492							1
SDSL	serine dehydratase-like	12q24.13	0.352	0.00185	0.0181							1
SPTAN1	spectrin, alpha, non-erythrocytic 1 (alpha-fodrin)	9q34.11	0.352	0.00515	0.0372							1
USP39	ubiquitin specific peptidase 39	2p11.2	0.352	0.01054	0.0608							1
ZNF248	In multiple Geneids		0.352	0.04685	0.1606							1
TMEM184A	transmembrane protein 184A	7p22.3	-1.034	2.00E-05	7.00E-04							1
DBNDD2	dysbindin (dystrobrevin binding protein 1) domain containing 2	20q13.12	0.351	0.03625	0.1367							1
TMEM184C	transmembrane protein 184C	4q31.23				-0.315	0.00164	0.0223				1
TMEM185A	transmembrane protein 185A	Xq28				0.34	0.000455	0.01195				1
TMEM186	transmembrane protein 186	16p13.2				-0.3515	0.005625	0.0416				1
TMEM188	Data not found					-0.38	0.00141	0.0207				1
TMEM189	transmembrane protein 189	20q13.2				0.37	0.00474	0.0381				1
TMEM189-UBE2	TMEM189-UBE2V1 readthrough	20q13.2				0.37	0.00474	0.0381				1
TMEM19	transmembrane protein 19	12q21.1	-1.303	4.00E-05	0.0011							1
TMEM192	transmembrane protein 192	4q32.3				-0.39025	0.00403	0.02965				1
SH2D2A	SH2 domain containing 2A	1q21	0.351	0.0305	0.1221							1
TBC1D12	TBC1 domain family, member 12	10q23.33	0.35	0.00611	0.0418							1
TMEM195	Data not found					0.360045455	0.001257273	0.017940909				1
TMEM196	transmembrane protein 196	7p21.1				0.352833333	0.000726667	0.013566667				1
ANKZF1	ankyrin repeat and zinc finger domain containing 1	2q35	0.349	0.00972	0.0574							1
C10orf25	chromosome 10 open reading frame 25	10q11.21	0.349	0.01175	0.0652							1
TMEM201	transmembrane protein 201	1p36.22				-0.439	0.00026	0.0097				1
TMEM202	transmembrane protein 202	15q24.1				-0.309	0.00278	0.0289				1
PDE4A	phosphodiesterase 4A, cAMP-specific	19p13.2	0.349	0.02763	0.1141							1
TMEM205	transmembrane protein 205	19p13.2				-0.448	0.00494	0.0333				1
TMEM207	transmembrane protein 207	3q28				0.447666667	0.010496667	0.051233333				1
TMEM209	transmembrane protein 209	7q32.2				-0.329	0.00584	0.0426				1
TMEM211	transmembrane protein 211	22q11.23				-0.3635	0.00372	0.0301				1
TMEM213	transmembrane protein 213	7q34				-0.383	0.00201	0.0246				1
PDX1	pancreatic and duodenal homeobox 1	13q12.1	0.349	0.04995	0.167							1
TIE1	tyrosine kinase with immunoglobulin-like and EGF-like domains 1	1p34-p33	0.349	0.03865	0.1425							1
TMEM217	transmembrane protein 217	6p21.2	-1.031	6.00E-05	0.0015							1
TMEM222	transmembrane protein 222	1p36.11	-0.422	0.00339	0.0279	-0.3425	0.00832	0.0423				2
ZNF222	zinc finger protein 222	19q13.2	0.349	0.03129	0.1242							1
DDX19B	DEAD (Asp-Glu-Ala-Asp) box polypeptide 19B	16q22.1	0.348	0.01617	0.0805							1
DHX9	DEAH (Asp-Glu-Ala-His) box polypeptide 9	1q25	0.348	0.02853	0.1167							1
KIAA1429	KIAA1429	8q22.1	0.348	0.0048	0.0355							1

KLHDC4	kelch domain containing 4	16q24.3	0.348	0.01191	0.0659							1
TMEM237	transmembrane protein 237	2q33.2	-0.615	0.00207	0.0195							1
POLE4	polymerase (DNA-directed), epsilon 4, accessory subunit	2p12	0.348	0.01628	0.0808							1
TTC17	tetratricopeptide repeat domain 17	11p11.2	0.348	0.01744	0.0846	0.379	0	0.0024				2
TMEM246	transmembrane protein 246	9q31.1	-0.349	0.0416	0.1493							1
TMEM247	transmembrane protein 247	2p21	-0.505	0.00029	0.0048							1
ZC3H15	zinc finger CCCH-type containing 15	2q32.1	0.348	0.0123	0.0672	0.334	0.000105	0.0058				2
TMEM25	transmembrane protein 25	11q23.3				-0.387	3.00E-04	0.0103				1
TMEM254	Data not found		-1.48	1.00E-05	5.00E-04							1
COL28A1	collagen, type XXVIII, alpha 1	7p21.3	0.347	0.04007	0.1457	0.345333333	0.00196	0.023833333				2
TMEM256	Data not found		-0.409	0.04325	0.153							1
GATAD2B	GATA zinc finger domain containing 2B	1q21.3	0.347	0.01758	0.0851							1
RPA2	replication protein A2, 32kDa	1p35	0.347	0.03203	0.1259							1
TMEM32	Data not found								0.818	2.696	5	1
TMEM33	transmembrane protein 33	4p13							-0.782	2.82286	7	1
TMEM37	transmembrane protein 37	2q14.2							0.771	2.98071	7	1
TMEM38A	transmembrane protein 38A	19p13.11				-0.370333333	0.014476667	0.060466667				1
SF3B1	splicing factor 3b, subunit 1, 155kDa	2q33.1	0.347	0.00565	0.0397				-0.731	2.682	5	2
ADAMTS14	ADAM metallopeptidase with thrombospondin type 1 motif, 14	10q21	0.346	0.02262	0.1003							1
TMEM39B	transmembrane protein 39B	1p35.1				-0.47925	0.003045	0.024675				1
TMEM40	transmembrane protein 40	3p25.2	-4.001	0	0	-0.394	0.000125	0.0072				2
TMEM41B	In multiple Geneids					-0.392666667	0.002156667	0.021933333				1
TMEM42	transmembrane protein 42	3p21.31				-0.321	0.00013	0.0073				1
TMEM43	transmembrane protein 43	3p25.1	-0.496	0.01712	0.0837							1
CD2BP2	CD2 (cytoplasmic tail) binding protein 2	16p11.2	0.346	0.03429	0.1314							1
TMEM47	transmembrane protein 47	Xp11.4				0.354	0.00198	0.0244				1
TMEM48	transmembrane protein 48	1p32.3							-0.817	2.49	6	1
TMEM5	transmembrane protein 5	12q14.2							-1.023	2.628	5	1
TMEM50A	transmembrane protein 50A	1p36.11				-0.413	0.00331	0.030566667				1
TMEM50B	transmembrane protein 50B	21q22.11				-0.3745	0.001143333	0.015433333				1
TMEM51	transmembrane protein 51	1p36.21				-0.365333333	0.002168333	0.023733333				1
DPAGT1	dolichyl-phosphate (UDP-N-acetylglucosamine) N-acetylglucosaminephosphot	11q23.3	0.346	0.01706	0.0835							1
RPS3	ribosomal protein S3	11q13.3-q13.5	0.346	0.01001	0.0585							1
CST4	cystatin S	20p11.21	0.345	0.01256	0.0682							1
TMEM55B	transmembrane protein 55B	14q11.2				-0.442	0.00151	0.0214				1
METTL2B	methyltransferase like 2B	7q32.1	0.345	0.02861	0.1169							1
TMEM57	transmembrane protein 57	1p36.11	-1.354	0	2.00E-04	-0.365	0.0052	0.038866667				2
TMEM59	transmembrane protein 59	1p32.3				-0.363	0.00151	0.0214				1
TMEM61	transmembrane protein 61	1p32.3	-0.304	0.00196	0.0188							1
OSBP	oxysterol binding protein	11q12-q13	0.345	0.00697	0.0457							1
TCF7	transcription factor 7 (T-cell specific, HMG-box)	5q31.1	0.345	0.01828	0.0874							1
TMEM63C	transmembrane protein 63C	14q24.3	-1.794	6.00E-05	0.0016	-0.367428571	0.000964286	0.013842857				2
TMEM64	transmembrane protein 64	8q21.3				0.352	0.001265	0.0191				1
VWA1	von Willebrand factor A domain containing 1	1p36.33	0.345	0.00464	0.0347							1
TMEM69	transmembrane protein 69	1p34.1							-0.715	2.56333	6	1
TMEM71	transmembrane protein 71	8q24.22				0.391222222	0.015168889	0.069133333				1
TMEM74	transmembrane protein 74	8q23.1				0.356	0.00027	0.0099				1
TMEM75	transmembrane protein 75	8q24.21							1.084	3.67333	6	1
TMEM79	transmembrane protein 79	1q22	-2.429	0	0							1
TMEM80	transmembrane protein 80	11p15.5	-0.881	3.00E-05	9.00E-04							1
TMEM86B	transmembrane protein 86B	19q13.42							0.704	2.50667	6	1
EFNA2	ephrin-A2	19p13.3	0.344	0.00483	0.0356							1
TMEM8B	transmembrane protein 8B	9p13.3				-0.3475	0.00143	0.01995				1
PCDHB12	protocadherin beta 12	5q31	0.344	0.04314	0.1527							1
TMEM90A	transmembrane protein 90A					-0.35425	0.0012275	0.01535				1
TMEM91	transmembrane protein 91	19q13.2	-0.386	0.02514	0.1075	-0.466	0.00516	0.0399	1.215	3.24667	6	3
SMC3	structural maintenance of chromosomes 3	10q25	0.344	0.04805	0.1631							1
TMEM93	Data not found					-0.45	0.00111	0.0185				1
TMEM95	transmembrane protein 95	17p13.1	-0.317	0.0085	0.0522							1
BBS12	Bardet-Biedl syndrome 12	4q27	0.342	0.02758	0.114							1
HNRNPH2	heterogeneous nuclear ribonucleoprotein H2 (H')	Xq22	0.342	0.03654	0.1374							1
TMEM99	transmembrane protein 99	17q21.2							-0.788	2.61	5	1
TMEM9B	TMEM9 domain family, member B	11p15.3	-0.792	0.00056	0.0078							1
RNF220	ring finger protein 220	1p34.1	0.342	0.00676	0.0448							1
TMIE	transmembrane inner ear	3p21	-0.208	0.04114	0.1484							1
TMIGD2	transmembrane and immunoglobulin domain containing 2	19p13.3	-0.21	0.02681	0.1121	-0.4265	0.0042225	0.0351				2
TMLHE	trimethyllysine hydroxylase, epsilon	Xq28				0.338	0.019016667	0.082766667	-0.699	2.61667	6	2
TMOD3	tropomodulin 3 (ubiquitous)	15q21.1-q21.2	-1.477	0.00014	0.0029							1
TMOD4	tropomodulin 4 (muscle)	1q12							0.765	2.684	5	1

ZFAND3	zinc finger, AN1-type domain 3	6pter-p22.3	0.342	0.01768	0.0854	0.351	0.019790667	0.08124				2
TMPPE	transmembrane protein with metallophosphoesterase domain	3p22.3				-0.37	0.00065	0.012025				1
TMPRSS11A	transmembrane protease, serine 11A	4q13.2							-0.906	2.608	5	1
TMPRSS11B	transmembrane protease, serine 11B	4q13.2							-1.007	2.81833	6	1
TMPRSS11D	transmembrane protease, serine 11D	4q13.2							-0.853	2.9625	8	1
TMPRSS11E	transmembrane protease, serine 11E	4q13.2							-0.88	3.210316667	6.666666667	1
TMPRSS11F	transmembrane protease, serine 11F	4q13.2							-0.618	2.84556	9	1
TMPRSS12	transmembrane (C-terminal) protease, serine 12	12q13.12	-0.201	0.04518	0.1573							1
TMPRSS13	transmembrane protease, serine 13	11q23	-0.955	0.00101	0.0118							1
TMPRSS2	transmembrane protease, serine 2	21q22.3				-0.365125	0.001235	0.0146625				1
TTC31	tetratricopeptide repeat domain 31	2p13.1	0.341	0.01058	0.0609							1
TMPRSS4	transmembrane protease, serine 4	11q23.3				-0.335	0.00045	0.0123				1
TMPRSS6	transmembrane protease, serine 6	22q12.3				-0.463	5.60E-05	0.0046				1
TMPRSS7	transmembrane protease, serine 7	3q13.2							-0.775	3.024	5	1
TMPRSS9	transmembrane protease, serine 9	19p13.3				-0.501833333	0.003748333	0.02635				1
FAM133B	family with sequence similarity 133, member B	7q21.2	0.34	0.03356	0.1297							1
TMSB4Y	thymosin beta 4, Y-linked	Yq11.221				-0.564	0.011	0.0603				1
TMTC1	transmembrane and tetratricopeptide repeat containing 1	12p11.22				0.413	0.00024	0.0093				1
TMTC2	transmembrane and tetratricopeptide repeat containing 2	12q21.31				0.3225	0.00688	0.04655				1
TMTC3	transmembrane and tetratricopeptide repeat containing 3	12q21.32	-0.814	0.00646	0.0434							1
NUP133	nucleoporin 133kDa	1q42.13	0.34	0.01613	0.0804							1
TMUB1	transmembrane and ubiquitin-like domain containing 1	7q36.1				-0.469	0.00013	0.0073				1
TMX1	thioredoxin-related transmembrane protein 1	14q22.1	-0.452	0.02129	0.0968							1
TMX2	thioredoxin-related transmembrane protein 2	11cen-q22.3				-0.85	0.00011	0.0069				1
TMX4	thioredoxin-related transmembrane protein 4	20p12				0.3655	0.00943	0.0512				1
TNC	tenascin C	9q33				0.426	0.001025	0.01495				1
TNF	tumor necrosis factor	6p21.3	-0.331	0.00847	0.0521				0.784	3.16214	7	2
TNFAIP2	tumor necrosis factor, alpha-induced protein 2	14q32				-0.312	0.00926	0.0549				1
RNASEH1	ribonuclease H1	2p25	0.34	0.02745	0.1137							1
SPAG4	sperm associated antigen 4	20q11.21	0.34	0.04735	0.1617							1
TNFAIP8	tumor necrosis factor, alpha-induced protein 8	5q23.1	-0.562	0.00105	0.0121	-0.338857143	0.004682857	0.025814286				2
TMEM183A	transmembrane protein 183A	1q32.1	0.34	0.03679	0.138							1
TNFAIP8L2	tumor necrosis factor, alpha-induced protein 8-like 2	1q21.3	-0.222	0.04243	0.1512							1
TNFAIP8L3	tumor necrosis factor, alpha-induced protein 8-like 3	15q21.2	-0.49	0.00518	0.0373							1
TLX1	T-cell leukemia homeobox 1	10q24	0.339	0.04308	0.1526							1
CDK5RAP1	CDK5 regulatory subunit associated protein 1	20pter-q11.23	0.338	0.01916	0.0903							1
DUSP8	dual specificity phosphatase 8	11p15.5	0.338	0.01326	0.0704							1
TNFRSF11A	tumor necrosis factor receptor superfamily, member 11a, NFKB activator	18q22.1				-0.3496	0.000622	0.01336				1
PRR5	proline rich 5 (renal)	22q13	0.338	0.00093	0.0111							1
QTRTD1	queuine tRNA-ribosyltransferase domain containing 1	3q13.31	0.338	0.04	0.1456							1
ZNF37A	zinc finger protein 37A	10p11.2	0.338	0.04633	0.1596							1
TNFRSF19	In multiple Geneids								-0.74	2.72667	6	1
ANKS1A	ankyrin repeat and sterile alpha motif domain containing 1A	6p21.31	0.337	0.04333	0.1531							1
CCAR1	cell division cycle and apoptosis regulator 1	10q21.3	0.337	0.00693	0.0456							1
TNFRSF25	tumor necrosis factor receptor superfamily, member 25	1p36.2				-0.331	0.00145	0.021				1
TNFRSF8	tumor necrosis factor receptor superfamily, member 8	1p36				-0.374466667	0.007323333	0.040853333	0.716	2.81667	6	2
TNFSF12	tumor necrosis factor (ligand) superfamily, member 12	17p13				-0.41925	0.01239	0.05625				1
TNFSF12-TNFSF	TNFSF12-TNFSF13 readthrough	17p13.1				-0.4474	0.009944	0.0466				1
TNFSF13	tumor necrosis factor (ligand) superfamily, member 13	17p13.1				-0.56	0.00016	0.008				1
TNFSF13B	tumor necrosis factor (ligand) superfamily, member 13b	13q32-q34							-0.972	2.982	5	1
TNFSF14	tumor necrosis factor (ligand) superfamily, member 14	19p13.3				-0.425333333	0.00084	0.015766667				1
CHCHD7	coiled-coil-helix-coiled-coil-helix domain containing 7	8q12.1	0.337	0.00195	0.0188							1
TNFSF18	tumor necrosis factor (ligand) superfamily, member 18	1q23				0.358	5.00E-04	0.0129				1
DNAH6	dynein, axonemal, heavy chain 6	2p11.2	0.337	0.027055	0.10265	0.335375	0.00054875	0.0115625				2
U2AF2	U2 small nuclear RNA auxiliary factor 2	19q13.42	0.337	0.00318	0.0267							1
TNIP1	TNFAIP3 interacting protein 1	5q32-q33.1	-0.345	0.02635	0.111	-0.353666667	0.00022	0.007766667				2
TNIP2	TNFAIP3 interacting protein 2	4p16.3				-0.431	0.00114	0.0183				1
TNIP3	TNFAIP3 interacting protein 3	4q27							-0.967	3.58	9	1
TNK2-AS1	Data not found		-0.411	0.00455	0.0343							1
USP42	ubiquitin specific peptidase 42	7p22.1	0.337	0.01641	0.0812							1
TNKS1BP1	tankyrase 1 binding protein 1, 182kDa	11q12.1	-0.503	0.00248	0.0223							1
TNKS2	tankyrase, TRF1-interacting ankyrin-related ADP-ribose polymerase 2	10q23.3	-0.288	0.00871	0.0532							1
TNMD	tenomodulin	Xq21.33-q23							-0.959	2.705	8	1
POMP	proteasome maturation protein	13q12.3	0.336	0.03882	0.1428							1
TNNI2	troponin I type 2 (skeletal, fast)	11p15.5	-0.283	0.03864	0.1425							1
TNNI3K	TNNI3 interacting kinase	1p31.1				0.466	1.00E-05	0.0034	-1.087	3.91444	9	2
TNNT1	troponin T type 1 (skeletal, slow)	19q13.4				-0.378333333	0.003006667	0.025533333				1
TNNT2	troponin T type 2 (cardiac)	1q32	-0.575	0.00224	0.0207							1
TNNT3	troponin T type 3 (skeletal, fast)	11p15.5							0.61	2.64571	7	1

TNP1	transition protein 1 (during histone to protamine replacement)	2q35-q36	-0.263	0.00789	0.0497							1
TNPO1	transportin 1	5q13.2							-0.902	3.27857	7	1
TNPO2	transportin 2	19p13.2	-0.335	0.04506	0.157	-0.390714286	0.012537143	0.060614286				2
TNPO3	transportin 3	7q32.1				-0.406	0.00187	0.0237				1
TNR	tenascin R (restrictin, janusin)	1q24				0.349571429	0.001729286	0.019335714	-0.53	2.65167	6	2
PSMB2	proteasome (prosome, macropain) subunit, beta type, 2	1p34.2	0.336	0.02117	0.0964							1
TNRC6A	trinucleotide repeat containing 6A	16p11.2				-0.31	0.0048	0.0384				1
TNRC6B	trinucleotide repeat containing 6B	22q13.1				-0.368222222	0.003143333	0.026447222				1
TNRC6C	trinucleotide repeat containing 6C	17q25.3							-0.855	2.87167	6	1
HNRNPL	heterogeneous nuclear ribonucleoprotein L	19q13.2	0.335	0.00292	0.0251							1
TNS4	tensin 4	17q21.2	-1.454	0.04697	0.1609							1
TOB1	transducer of ERBB2, 1	17q21							-0.964	3.07857	7	1
TOB2	In multiple Geneids					-0.495	0	2.00E-04				1
IL18BP	interleukin 18 binding protein	11q13	0.335	0.02897	0.1178							1
TOLLIP	toll interacting protein	11p15.5	-0.954	0	0	-0.3565	0.0074375	0.042875				2
TOM1	target of myb1 (chicken)	22q13.1	-0.974	1.00E-04	0.0023	-0.423375	0.00082125	0.012125				2
RBM38	RNA binding motif protein 38	20q13.31	0.335	0.00868	0.053	0.326	0.00128	0.0197				2
TOM1L2	In multiple Geneids		-1.916	0	0	-0.365333333	0.002053333	0.0233				2
TOMM22	translocase of outer mitochondrial membrane 22 homolog (yeast)	22q12-q13	-0.375	0.03726	0.1392	-0.474	6.50E-05	0.00515				2
NPAT	nuclear protein, ataxia-telangiectasia locus	11q22-q23	0.334	0.00725	0.047				-0.593	2.54167	6	2
TOMM40	translocase of outer mitochondrial membrane 40 homolog (yeast)	19q13				-0.3185	0.00297	0.0286				1
B3GNT9	UDP-GlcNAc:betaGal beta-1,3-N-acetylglucosaminyltransferase 9	16q22.1	0.333	0.00265	0.0233							1
IRF3	interferon regulatory factor 3	19q13.3-q13.4	0.333	0.00319	0.0268							1
TOP3A	topoisomerase (DNA) III alpha	17p12-p11.2				-0.34575	0.0116	0.057975				1
TOP3B	topoisomerase (DNA) III beta	22q11.22				-0.378333333	0.00025	0.0087				1
TOPAZ1	Data not found		-0.318	0.00844	0.052							1
TOPORS	In multiple Geneids		-0.377	0.00147	0.0153	-0.3605	0.00339	0.02725				2
NEU3	sialidase 3 (membrane sialidase)	11q13.5	0.333	0.01437	0.0744							1
TOR1AIP1	torsin A interacting protein 1	1q24.2							-0.774	2.7975	6	1
TOR1AIP2	torsin A interacting protein 2	1q25.2							-0.774	2.7975	6	1
ATG2A	autophagy related 2A	11q13.1	0.332	0.00035	0.0055							1
C16orf58	chromosome 16 open reading frame 58	16p11.2	0.332	0.02889	0.1176							1
FOXJ3	forkhead box J3	1p34.2	0.332	0.01893	0.0896							1
TOX	thymocyte selection-associated high mobility group box	8q12.1				0.340733333	0.001866	0.018566667				1
TOX2	TOX high mobility group box family member 2	20q13.12				0.344	0.01098	0.0603				1
SFT2D3	SFT2 domain containing 3	2q14.3	0.332	0.00346	0.0283							1
TOX4	TOX high mobility group box family member 4	14q11.2	-0.359	0.00099	0.0116							1
TP53	tumor protein p53	17p13.1				-0.311	0.00726	0.048				1
TP53AIP1	tumor protein p53 regulated apoptosis inducing protein 1	11q24	-2.469	0	0							1
SIRT1	sirtuin 1	10q21.3	0.332	0.02346	0.1027							1
TP53I3	tumor protein p53 inducible protein 3	2p23.3	-1.085	0.00011	0.0023							1
TP53INP2	tumor protein p53 inducible nuclear protein 2	20q11.22	-1.251	1.00E-05	3.00E-04							1
TP53RK	TP53 regulating kinase	20q13.2							-0.889	2.41667	6	1
TP53TG1	TP53 target 1 (non-protein coding)	7q21.1	-0.43	0.02693	0.1124							1
TP53TG3	TP53 target 3	16p13	-0.443	0.0051	0.037				0.804714286	3.764132857	7.571428571	2
TP63	tumor protein p63	3q28				0.345882353	0.004415294	0.032311765				1
TP73	tumor protein p73	1p36.3	-0.484	0.00013	0.0027	-0.411	0.000326667	0.0087				2
TP73L	Data not found								-0.974	3.41	5	1
TPCN1	two pore segment channel 1	12q24.13	-0.473	0.01757	0.0851	-0.356	0.00043	0.012				2
ZNF674	zinc finger protein 674	Xp11.3	0.332	0.04434	0.1554							1
ZNF782	zinc finger protein 782	9q22.33	0.332	0.02196	0.0987							1
TPD52L1	tumor protein D52-like 1	6q22-q23	-1.989	0.00073	0.0094							1
TPD52L2	tumor protein D52-like 2	20q13.2-q13.3							-0.602	2.634	5	1
TPGS1	tubulin polyglutamylase complex subunit 1	19p13.3	-0.345	0.00431	0.033							1
TPGS2	tubulin polyglutamylase complex subunit 2	18q12.2	-0.58	0.00159	0.0162							1
TPH1	tryptophan hydroxylase 1	11p15.3-p14							-0.913	3.46571	7	1
TPH2	tryptophan hydroxylase 2	12q21.1				0.328	0.00049	0.0128	-1.051	3.562	5	2
TPK1	thiamin pyrophosphokinase 1	7q34-q35				-0.333	0.00697	0.047				1
C19orf54	chromosome 19 open reading frame 54	19q13.2	0.331	0.02261	0.1003							1
DPF2	D4, zinc and double PHD fingers family 2	11q13	0.331	0.00867	0.053							1
FAM219A	family with sequence similarity 219, member A	9p13.3	0.331	0.00277	0.0241							1
TPM4	tropomyosin 4	19p13.1	-0.717	0.00679	0.0449	-0.39	0.003265	0.026425				2
TPMT	thiopurine S-methyltransferase	6p22.3				-0.39	0.00327	0.0314				1
TPO	thyroid peroxidase	2p25	-0.215	0.02837	0.1163							1
GTF3C3	general transcription factor IIIC, polypeptide 3, 102kDa	2q33.1	0.331	0.03881	0.1428							1
LTBR	lymphotoxin beta receptor (TNFR superfamily, member 3)	12p13	0.331	0.00792	0.0498							1
MED14	mediator complex subunit 14	Xp11.4	0.331	0.01495	0.0764							1
TPRG1	tumor protein p63 regulated 1	3q28				0.352555556	0.002428889	0.024755556				1
TPRXL	tetra-peptide repeat homeobox-like	3p25.1	-1.131	3.00E-05	0.001							1

TPSG1	tryptase gamma 1	16p13.3				-0.404	0.00665	0.0458				1
TPST2	tyrosylprotein sulfotransferase 2	22q12.1	-0.258	0.03078	0.1228	-0.39465	0.0011155	0.01417				2
TPTE	transmembrane phosphatase with tensin homology	21p11	-0.269	0.01576	0.0792	-0.347	0.00234	0.0266				2
TPTE2	transmembrane phosphoinositide 3-phosphatase and tensin homolog 2	13q12.11				-0.326	0.00221	0.0258				1
TPTEP1	transmembrane phosphatase with tensin homology pseudogene 1	22q11.1	-0.7295	0.024955	0.08645							1
NMB	neuromedin B	15q22-qter	0.331	0.02165	0.0978							1
TRABD	TraB domain containing	22q13.33				-0.484	0	0.0013				1
PDE4B	phosphodiesterase 4B, cAMP-specific	1p31	0.331	0.02867	0.1171	0.349	0.00833	0.0518	-0.88	2.9185	6.5	3
TRABD2B	Data not found		-0.251	0.00807	0.0505							1
TRADD	TNFRSF1A-associated via death domain	16q22				-0.35	0.00958	0.0559				1
TRAF2	TNF receptor-associated factor 2	9q34				-0.315	0.005805	0.0421				1
POLR2G	polymerase (RNA) II (DNA directed) polypeptide G	11q13.1	0.331	0.02276	0.1007							1
TMEM255B	Data not found		0.331	0.00503	0.0366							1
TRAF3IP2	TRAF3 interacting protein 2	6q21	-0.725	0.00105	0.0121							1
TRAF3IP3	TRAF3 interacting protein 3	1q32				0.309	0.00127	0.018	-0.784	2.812	5	2
IMP4	IMP4, U3 small nucleolar ribonucleoprotein, homolog (yeast)	2q21.1	0.33	0.04198	0.1501							1
NFU1	NFU1 iron-sulfur cluster scaffold homolog (S. cerevisiae)	2p15-p13	0.33	0.04127	0.1487							1
TRAF7	TNF receptor-associated factor 7, E3 ubiquitin protein ligase	16p13.3							0.995	3.79125	8	1
TRAFD1	TRAF-type zinc finger domain containing 1	12q				-0.327	0.020605	0.0834				1
TRAIP	TRAF interacting protein	3p21.31				-0.425333333	0.000643333	0.013666667				1
TRAK1	trafficking protein, kinesin binding 1	3p22.1				-0.386	0.00063	0.012257143	0.94	3.104	5	2
NIT1	nitrilase 1	1q21-q22	0.33	0.04052	0.1468							1
TRAM1L1	translocation associated membrane protein 1-like 1	4q26							-0.735	2.656	5	1
C12orf5	In multiple Geneids		0.329	0.04874	0.1645							1
ELK4	ELK4, ETS-domain protein (SRF accessory protein 1)	1q32	0.329	0.01948	0.0912				-0.751	2.64333	6	2
TRAP1	TNF receptor-associated protein 1	16p13.3				-0.449818182	0.00746	0.045490909				1
TRAPPC10	In multiple Geneids		-0.2	0.04664	0.1602	-0.357230769	0.002276154	0.022684615				2
TRAPPC11	trafficking protein particle complex 11	4q35.1	-0.47	0.00485	0.0358							1
FPGS	folylpolyglutamate synthase	9q34.1	0.329	0.03881	0.1428							1
TRAPPC5	trafficking protein particle complex 5	19p13.2	-0.505	0.0056	0.0395							1
GGTLC2	gamma-glutamyltransferase light chain 2	22q11.22	0.329	0.03231	0.1266							1
TRAPPC6B	trafficking protein particle complex 6B	14q21.1	-0.532	0.00819	0.051	-0.328	0.01189	0.0631				2
TRAPPC9	In multiple Geneids					0.399666667	0.005796667	0.036				1
TRDN	triadin	6q22.31				0.306	0.04039	0.1304				1
TREH	trehalase (brush-border membrane glycoprotein)	11q23.3	-0.457	0.02878	0.1174							1
LINC00085	long intergenic non-protein coding RNA 85	19q13.41	0.329	0.0266	0.1117							1
RAB1A	RAB1A, member RAS oncogene family	2p14	0.329	0.01146	0.0641							1
TREML3	Data not found					0.312	0.01602	0.0749				1
TREML4	triggering receptor expressed on myeloid cells-like 4	6p21.1				0.452	0.00746	0.0487	-0.639	2.658	5	2
TRERF1	transcriptional regulating factor 1	6p21.1-p12.1				0.357714286	0.022122857	0.085128571				1
TREX2	three prime repair exonuclease 2	Xq28	-0.301	0.01623	0.0752							1
ZNF638	zinc finger protein 638	2p13.1	0.329	0.00548	0.0389							1
TRHR	thyrotropin-releasing hormone receptor	8q23				0.381	0.0002325	0.0086				1
UCK2	uridine-cytidine kinase 2	1q23	0.328	0.01203	0.0663							1
TRIB2	tribbles homolog 2 (Drosophila)	2p24.3				0.342	0.00149	0.0212				1
EFHD1	EF-hand domain family, member D1	2q37.1	0.327	0.02909	0.1181							1
TRIM13	tripartite motif containing 13	13q14	-0.904	2.00E-04	0.0036							1
GMCL1	germ cell-less homolog 1 (Drosophila)	2p13.3	0.327	0.01459	0.075							1
TRIM16	tripartite motif containing 16	17p11.2	-1.209	7.00E-04	0.0091	-0.334	0.001086667	0.016366667				2
TRIM16L	tripartite motif containing 16-like	17p11.2	-1.276	0.00105	0.0121	-0.332	6.00E-04	0.0139				2
TRIM17	tripartite motif containing 17	1q42							0.57	3.00222	9	1
TRIM2	tripartite motif containing 2	4q31.3				-0.390142857	0.004648571	0.033957143				1
TRIM22	tripartite motif containing 22	11p15							-0.998	2.82	6	1
TRIM24	tripartite motif containing 24	7q32-q34				-0.34	0.009495	0.0537				1
TRIM25	tripartite motif containing 25	17q23.2				-0.311	0.00369	0.0334				1
APPL2	adaptor protein, phosphotyrosine interaction, PH domain and leucine zipper c	12q24.1	0.326	0.02801	0.1153							1
TRIM29	tripartite motif containing 29	11q23.3	-2.04	0.00237	0.0216							1
TLK2	tousled-like kinase 2	17q23	0.326	0.04359	0.1537							1
COG3	component of oligomeric golgi complex 3	13q14.13	0.325	0.04345	0.1533							1
TRIM33	tripartite motif containing 33	1p13.1				0.479	0	0.0011				1
TRIM35	tripartite motif containing 35	8p21.2							-0.944	2.83833	6	1
TRIM36	tripartite motif containing 36	5q22.3				-0.305	0.00014	0.0076				1
CRELD1	cysteine-rich with EGF-like domains 1	3p25.3	0.325	0.00647	0.0434							1
TRIM41	tripartite motif containing 41	5q35.3	-0.271	0.04607	0.1591	-0.357	0.00033	0.0108	0.583	2.898	5	3
FGF9	fibroblast growth factor 9 (glia-activating factor)	13q11-q12	0.325	0.02597	0.11				-0.499	2.487	5	2
TRIM46	tripartite motif containing 46	1q22	-0.244	0.00671	0.0446							1
MRPL20	In multiple Geneids		0.325	0.04031	0.1463							1
TRIM48	tripartite motif containing 48	11q11	-0.515	0.00453	0.0342							1
TRIM49	tripartite motif containing 49	11p11.12-q12							-0.899	3.124	5	1

TRIM5	tripartite motif containing 5	11p15							-0.998	2.82	6	1
TRIM50	tripartite motif containing 50	7q11.23				-0.335	0.01765	0.0792				1
TRIM51	tripartite motif-containing 51	11q11	-0.4705	0.012185	0.06375							1
ARHGEF40	Rho guanine nucleotide exchange factor (GEF) 40	14q11.2	0.324	0.01755	0.085							1
TRIM55	tripartite motif containing 55	8q13.1				0.307	0.00877	0.0533				1
TRIM6	tripartite motif containing 6	11p15.4				-0.358	0.00343	0.0322				1
TRIM60	tripartite motif containing 60	4q32.3	-0.254	0.03412	0.131							1
TRIM61	In multiple Geneids		-0.368	0.00437	0.0332	-0.36	0.007844	0.04116				2
TRIM62	tripartite motif containing 62	1p35.1	-0.245	0.02611	0.1104	-0.317	0.00149	0.0213				2
TRIM63	tripartite motif containing 63, E3 ubiquitin protein ligase	1p34-p33	-0.176	0.04876	0.1646	-0.446	0.00462	0.0365				2
TRIM65	tripartite motif containing 65	17q25.1				-0.441	0.00174	0.0229				1
TRIM67	tripartite motif containing 67	1q42.2				0.467	0.00016	0.008				1
TRIM6-TRIM34	TRIM6-TRIM34 readthrough	11p15	-0.336	0.03371	0.1299	-0.5785	0.008265	0.04945				2
TRIM7	tripartite motif containing 7	5q35.3	-0.9	0	1.00E-04							1
TRIM71	tripartite motif containing 71, E3 ubiquitin protein ligase	3p22.3				-0.397857143	0.005057143	0.026971429				1
TRIM73	tripartite motif containing 73	7q11.23				-0.414	0.00602	0.0433	1.055666667	3.144526667	6.666666667	2
TRIM74	tripartite motif containing 74	7q11.23				-0.375	0.02243	0.07875	1.033	3.085	6	2
TRIM8	tripartite motif containing 8	10q24.3				-0.347	0.00206	0.0212				1
TRIM9	tripartite motif containing 9	14q22.1	-0.249	0.02855	0.1167							1
TRIML1	tripartite motif family-like 1	4q35.2				-0.544	0.00021	0.009				1
TRIML2	tripartite motif family-like 2	4q35.2	-0.439	0.00016	0.0031	-0.412	0.00288	0.0293				2
TRIMP1	Data not found					-0.311	0.00243	0.0271				1
GPX4	glutathione peroxidase 4	19p13.3	0.324	0.02984	0.1202							1
TRIOBP	TRIO and F-actin binding protein	22q13.1	-0.777	0	2.00E-04	-0.482692308	0.000102308	0.006192308				2
TRIP10	thyroid hormone receptor interactor 10	19p13.3	-1.438	0.00011	0.0024	-0.513	0.00033	0.0108				2
TRIP11	thyroid hormone receptor interactor 11	14q31-q32				-0.352666667	0.00055	0.011433333				1
SPCS2	signal peptidase complex subunit 2 homolog (S. cerevisiae)	11q13.4	0.324	0.01542	0.078							1
TRIP6	thyroid hormone receptor interactor 6	7q22	-0.532	0.01047	0.0605	-0.373	0.03277	0.1146				2
TRIT1	tRNA isopentenyltransferase 1	1p34.2				-0.336	0.00412	0.0354				1
SRRT	serrate RNA effector molecule homolog (Arabidopsis)	7q21	0.324	0.03076	0.1228							1
TRMT2A	TRM2 tRNA methyltransferase 2 homolog A (S. cerevisiae)	22q11.21				-0.357	0.00135	0.0202				1
TRMT44	Data not found		-0.716	0.00032	0.0052							1
TRMT5	TRM5 tRNA methyltransferase 5 homolog (S. cerevisiae)	14q23.1	-0.224	0.03199	0.1258							1
TRMT61A	tRNA methyltransferase 61 homolog A (S. cerevisiae)	14q32				-0.31	0.00168	0.0225				1
TRMU	tRNA 5-methylaminomethyl-2-thiouridylate methyltransferase	22q13				-0.389666667	0.000313333	0.008666667				1
TRNAU1AP	tRNA selenocysteine 1 associated protein 1	1p35.3	-0.407	0.01196	0.0661	-0.4775	0.004178333	0.031016667				2
TRNP1	TMF1-regulated nuclear protein 1	1p36.11	-0.613	0.00203	0.0193	-0.324	0.00432	0.0362				2
TROAP	trophinin associated protein (tastin)	12q13.12				-0.394	0.00527	0.0403				1
TMEM179B	transmembrane protein 179B	11q12.3	0.324	0.03614	0.1365							1
TRPC4	transient receptor potential cation channel, subfamily C, member 4	13q13.3				0.342833333	0.005046667	0.032783333				1
CLTC	clathrin, heavy chain (Hc)	17q11-qter	0.323	0.0159	0.0797							1
TRPC5	transient receptor potential cation channel, subfamily C, member 5	Xq23				0.349	0.00029	0.0102				1
TRPC6	transient receptor potential cation channel, subfamily C, member 6	11q22.1				0.366	0.00184	0.0236				1
TRPC7	transient receptor potential cation channel, subfamily C, member 7	5q31.1	-0.224	0.03161	0.1248							1
TRPM2	transient receptor potential cation channel, subfamily M, member 2	21q22.3				-0.462875	0.00178	0.0156				1
TRPM3	In multiple Geneids					0.325	0.00142	0.0207	-0.585	2.806	5	2
TRPM4	transient receptor potential cation channel, subfamily M, member 4	19q13.33				-0.3554	0.005246	0.03534				1
TRPM6	transient receptor potential cation channel, subfamily M, member 6	9q21.13				-0.304	0.00279	0.0289				1
TRPM7	transient receptor potential cation channel, subfamily M, member 7	15q21				-0.401	0.02183	0.0898				1
TRPM8	In multiple Geneids								-0.863	2.74	6	1
TRPS1	trichorhinophalangeal syndrome I	8q24.12				0.471983333	0.008264167	0.045955				1
TRPV1	transient receptor potential cation channel, subfamily V, member 1	17p13.2				-0.3638	0.001428	0.01884	0.723	2.950276667	5.333333333	2
FLJ45248	FLJ45248 protein	8q22.3	0.323	0.04172	0.1495							1
TRPV3	In multiple Geneids					-0.442666667	0.00284	0.022275				1
TRPV4	transient receptor potential cation channel, subfamily V, member 4	12q24.1	-0.867	0.01361	0.0717	-0.357	0.001055	0.0173				2
TRPV5	transient receptor potential cation channel, subfamily V, member 5	7q35	-0.395	0.00045	0.0067							1
TRPV6	transient receptor potential cation channel, subfamily V, member 6	7q34	-1.041	0	1.00E-04							1
MTMR6	myotubularin related protein 6	13q12	0.323	0.03165	0.1249							1
TRUB1	In multiple Geneids								-0.548	2.516	5	1
TRUB2	In multiple Geneids					-0.3	0.03788	0.1252				1
TRY1	Data not found								-1.016	3.406	5	1
TSC2	tuberous sclerosis 2	16p13.3	-0.445	0.00086	0.0106	-0.374	0.008995	0.0533				2
TRIM47	tripartite motif containing 47	17q25	0.323	0.04905	0.1651							1
TSC22D2	TSC22 domain family, member 2	3q25.1				0.4425	0.00219	0.0208				1
TSC22D4	TSC22 domain family, member 4	7p21-p15	-0.37	0.02924	0.1185	-0.34	0.04918	0.1473				2
TSEN15	tRNA splicing endonuclease 15 homolog (S. cerevisiae)	1q25				0.375	0.00081	0.016				1
TSEN2	tRNA splicing endonuclease 2 homolog (S. cerevisiae)	3p25.2				-0.319	0.00858	0.0527				1
ARMC7	armadillo repeat containing 7	17q25.1	0.322	0.01387	0.0727							1
TSG101	tumor susceptibility gene 101	11p15	-0.371	0.00561	0.0395							1

TSGA10	testis specific, 10	2q11.2				0.347	4.50E-05	0.00425	-0.868	2.796	5	2
TSGA10IP	testis specific, 10 interacting protein	11q13.1				-0.41	1.00E-05	0.0032				1
TSHB	thyroid stimulating hormone, beta	1p13							-0.873	3.43	9	1
TSHR	thyroid stimulating hormone receptor	14q31				-0.317	0.00322	0.0312				1
TSHZ2	teashirt zinc finger homeobox 2	20q13.2				0.479978495	0.024002151	0.091410753				1
TSHZ3	teashirt zinc finger homeobox 3	19q12				0.386	0.00899	0.054				1
TSKS	testis-specific serine kinase substrate	19q13.3	-0.256	0.0164	0.0812							1
TSKU	tsukushi small leucine rich proteoglycan homolog (Xenopus laevis)	11q13.5	-0.458	0.03115	0.1238							1
TSLP	thymic stromal lymphopoietin	5q22.1	-0.841	2.00E-05	6.00E-04							1
CASC2	cancer susceptibility candidate 2 (non-protein coding)	10q26.11	0.322	0.01414	0.0735							1
TSNARE1	t-SNARE domain containing 1	8q24.3				-0.3295	0.010345	0.05295				1
TSNAXIP1	translin-associated factor X interacting protein 1	16q22.1				-0.3825	0.0085275	0.0387				1
TMEM26	transmembrane protein 26	10q21.2	0.322	0.03633	0.1369							1
TSPAN10	tetraspanin 10	17q25.3	-0.305	0.02271	0.1005	-0.504	0.00151	0.0214	0.818	2.638	5	3
UBA2	ubiquitin-like modifier activating enzyme 2	19q12	0.322	0.02368	0.1033							1
ZNF511	zinc finger protein 511	10q26.3	0.322	0.00199	0.019							1
TSPAN14	tetraspanin 14	10q23.1	-1	6.00E-05	0.0016	-0.333	0.000715	0.0132				2
ANP32D	acidic (leucine-rich) nuclear phosphoprotein 32 family, member D	12q13.11	0.321	0.00421	0.0324							1
TSPAN16	tetraspanin 16	19p13.2				-0.395	0.0067625	0.0419625				1
TSPAN17	tetraspanin 17	5q35.3	-0.282	0.00222	0.0206	-0.322	3.00E-05	0.0044				2
FBXO18	F-box protein, helicase, 18	10p15.1	0.321	0.03217	0.1262							1
TSPAN2	tetraspanin 2	1p13.2				-0.345	0.00844	0.0521				1
LGALS8	lectin, galactoside-binding, soluble, 8	1q43	0.321	0.03035	0.1216				-0.707	2.516	5	2
TSPAN32	tetraspanin 32	11p15.5				-0.31	0.02642	0.1006	0.671	2.64143	7	2
PPP2R3C	protein phosphatase 2, regulatory subunit B'', gamma	14q13.2	0.321	0.0408	0.1474							1
TSPAN5	tetraspanin 5	4q23	-1.142	0.00427	0.0327	-0.381105263	0.003422105	0.020526316				2
TSPAN6	tetraspanin 6	Xq22							-0.716	2.43667	6	1
TSPAN7	tetraspanin 7	Xp11.4				0.34575	0.00376	0.031525				1
PTPN11	protein tyrosine phosphatase, non-receptor type 11	12q24	0.321	0.02299	0.1014							1
TSPO	translocator protein (18kDa)	22q13.31	-1.445	0.00012	0.0026	-0.528	0.000195	0.0078				2
TSPY1	testis specific protein, Y-linked 1	Yp11.2	-0.282	0.00883	0.0536				0.747666667	3.457636667	6	2
TSPY2	testis specific protein, Y-linked 2	Yp11.2							0.757666667	3.431081667	5.833333333	1
TSPYL1	TSPY-like 1	6q22.1							-0.82	3.25571	7	1
ARHGEF39	Data not found		0.32	0.04474	0.1562							1
TSPYL4	TSPY-like 4	6q22.1							-0.768	2.55	5	1
TSPYL6	TSPY-like 6	2p16.2				0.314	0.001	0.0177				1
TSR1	TSR1, 20S rRNA accumulation, homolog (S. cerevisiae)	17p13.3				-0.381833333	0.005913333	0.037116667				1
TSSC1	tumor suppressing subtransferable candidate 1	2p25.3				0.369	0.00049	0.0127				1
TSSC4	tumor suppressing subtransferable candidate 4	11p15.5							0.577	2.76714	7	1
TSSK1B	testis-specific serine kinase 1B	5q22.2	-0.341	0.00388	0.0306							1
TSSK2	In multiple Geneids					-0.889	0	0.0011	0.608	2.874	5	2
TSSK3	testis-specific serine kinase 3	1p35-p34				-0.329	0.01005	0.0574				1
TSSK4	testis-specific serine kinase 4	14q12	-0.262	0.0149	0.0762							1
TTBK1	tau tubulin kinase 1	6p21.1							1.011	3.11	5	1
TTBK2	tau tubulin kinase 2	15q15.2				-0.375	0.00595	0.043				1
TTC1	tetratricopeptide repeat domain 1	5q33.3				-0.326	0.003583333	0.032033333				1
C11orf84	chromosome 11 open reading frame 84	11q13.1	0.32	0.01088	0.0618							1
MED12	mediator complex subunit 12	Xq13	0.32	0.03736	0.1394							1
TTC18	tetratricopeptide repeat domain 18	10q22.2							-0.949	2.99857	7	1
TTC19	tetratricopeptide repeat domain 19	17p12				-0.361	0.0025	0.026166667				1
TTC21A	tetratricopeptide repeat domain 21A	3p22.2				-0.338	0.003476667	0.0316				1
PNPLA2	patatin-like phospholipase domain containing 2	11p15.5	0.32	0.03808	0.1411							1
TTC22	tetratricopeptide repeat domain 22	1p32.3	-1.564	1.00E-04	0.0022							1
TTC23	tetratricopeptide repeat domain 23	15q26.3	-0.39	0.04688	0.1607							1
TTC23L	tetratricopeptide repeat domain 23-like	5p13.2				0.3165	0.01269	0.06545				1
TTC24	tetratricopeptide repeat domain 24	1q23.1	-0.185	0.04477	0.1563							1
TTC25	tetratricopeptide repeat domain 25	17q21.2				-0.344	1.00E-04	0.0066				1
TTC26	tetratricopeptide repeat domain 26	7q34				-0.341	0.005255	0.0395				1
TTC28	tetratricopeptide repeat domain 28	22q12.1	-0.372	0.00396	0.0311	-0.3408	0.0010016	0.013412				2
TTC3	tetratricopeptide repeat domain 3	21q22.2				-0.3802	0.004798	0.02908				1
SDE2	Data not found		0.32	0.01872	0.0889							1
VPS41	vacuolar protein sorting 41 homolog (S. cerevisiae)	7p14-p13	0.32	0.01255	0.0682	0.3366	0.00527	0.0313	-0.857	3.178	5	3
TTC35	Data not found					0.371	0.000346667	0.009533333				1
TTC36	tetratricopeptide repeat domain 36	11q23.3	-0.24	0.03671	0.1378							1
EIF3C	eukaryotic translation initiation factor 3, subunit C	16p11.2	0.319	0.04667	0.1602							1
TTC39A	tetratricopeptide repeat domain 39A	1p32.3				-0.343	0.00148	0.0212				1
TTC39B	tetratricopeptide repeat domain 39B	9p22.3	-0.683	0.00386	0.0305	-0.401461538	0.001454231	0.018623077				2
TTC4	tetratricopeptide repeat domain 4	1p32.3				-0.352	0.00191	0.024				1
TTC7B	tetratricopeptide repeat domain 7B	14q32.11				-0.369857143	0.000821429	0.014642857				1

TTC9	tetratricopeptide repeat domain 9	14q24.2	-2.594	0	0							1
RNF25	In multiple Geneids		0.318	0.01469	0.0754							1
TTF1	transcription termination factor, RNA polymerase I	9q34.13				-0.313	0.00038	0.0114				1
SMG9	smg-9 homolog, nonsense mediated mRNA decay factor (C. elegans)	19q13.31	0.318	0.02273	0.1006							1
TTLL1	In multiple Geneids		-0.333	0.04203	0.1502	-0.48475	0.00076	0.013575				2
TTLL10	tubulin tyrosine ligase-like family, member 10	1p36.33				-0.345	0.00511	0.0397				1
TTLL11	tubulin tyrosine ligase-like family, member 11	9q33.2	-0.189	0.03558	0.1348	-0.313	0.0078	0.0499				2
TTLL12	tubulin tyrosine ligase-like family, member 12	22q13.31	-1.02	1.00E-05	4.00E-04	-0.408	0.002645	0.0229				2
TTLL13	tubulin tyrosine ligase-like family, member 13	15q26.1				-0.338	0.00936	0.0552				1
TTLL3	tubulin tyrosine ligase-like family, member 3	3p25.3				-0.399	0.00551	0.03308				1
ZNF799	zinc finger protein 799	19p13.2	0.318	0.02246	0.0999							1
COPB1	coatomer protein complex, subunit beta 1	11p15.2	0.317	0.0151	0.0769							1
TTLL8	tubulin tyrosine ligase-like family, member 8	22q13.33				-0.38625	0.00243875	0.0231				1
TTLL9	tubulin tyrosine ligase-like family, member 9	20q11.21	-0.275	0.04704	0.161							1
TTN	titin	2q31				0.350939394	0.000862424	0.013233333				1
TTRAP	Data not found					0.318	0.01179	0.0628				1
TTTY1	testis-specific transcript, Y-linked 1 (non-protein coding)	Yp11.2	-0.219	0.03188	0.1255	-0.502904762	0.021344286	0.085961905				2
TTTY10	testis-specific transcript, Y-linked 10 (non-protein coding)	Yq11.221	-0.311	0.01785	0.086	-0.456666667	0.023336667	0.093366667				2
TTTY11	testis-specific transcript, Y-linked 11 (non-protein coding)	Yp11.2				-0.428	0.02196	0.0901				1
TTTY12	testis-specific transcript, Y-linked 12 (non-protein coding)	Yp11.2				-0.364	0.04526	0.1399				1
TTTY14	testis-specific transcript, Y-linked 14 (non-protein coding)	Yq11.222	-0.614	0.00012	0.0025	-0.4685	0.017589444	0.075483333				2
TTTY15	testis-specific transcript, Y-linked 15 (non-protein coding)	Yq11.1				-0.514333333	0.010733333	0.056433333				1
TTTY17A	testis-specific transcript, Y-linked 17A (non-protein coding)	Yq11.2				-0.573	0.01467	0.0712				1
TTTY17B	testis-specific transcript, Y-linked 17B (non-protein coding)	Yq11.223				-0.573	0.01467	0.0712				1
TTTY17C	testis-specific transcript, Y-linked 17C (non-protein coding)	Yq11.223				-0.573	0.01467	0.0712				1
TTTY18	testis-specific transcript, Y-linked 18 (non-protein coding)	Yp11.2				-0.595	0.00987	0.0568				1
TTTY2	testis-specific transcript, Y-linked 2 (non-protein coding)	Yp11.2				-0.513090909	0.02423	0.091718182				1
TTTY21	testis-specific transcript, Y-linked 21 (non-protein coding)	Yp11.2				-0.5385	0.0169225	0.074875				1
TTTY22	testis-specific transcript, Y-linked 22 (non-protein coding)	Yp11.2				-0.484	0.0171475	0.075325				1
TTTY4	testis-specific transcript, Y-linked 4 (non-protein coding)	Yq11.2				-0.432058824	0.023548824	0.091823529				1
TTTY4B	testis-specific transcript, Y-linked 4B (non-protein coding)	Yq11.223				-0.432058824	0.023548824	0.091823529				1
TTTY4C	testis-specific transcript, Y-linked 4C (non-protein coding)	Yq11.223				-0.432058824	0.023548824	0.091823529				1
TTTY6	testis-specific transcript, Y-linked 6 (non-protein coding)	Yq11.223	-0.313	0.00288	0.0248	-0.526606061	0.01936899	0.080263636				2
TTTY6B	testis-specific transcript, Y-linked 6B (non-protein coding)	Yq11.223				-0.6155	0.01064	0.058525				1
TTTY7	testis-specific transcript, Y-linked 7 (non-protein coding)	Yp11.2				-0.5237	0.018577	0.07932				1
TTTY8	testis-specific transcript, Y-linked 8 (non-protein coding)	Yp11.2				-0.5232	0.024758	0.0959				1
TTTY9A	testis-specific transcript, Y-linked 9A (non-protein coding)	Yq11.222	-0.237	0.00133	0.0144	-0.4965	0.025645	0.0967				2
TTTY9B	testis-specific transcript, Y-linked 9B (non-protein coding)	Yq11.221				-0.4965	0.025645	0.0967				1
TTYH1	tweety homolog 1 (Drosophila)	19q13.4				-0.425	0.00393	0.0345				1
TTYH2	tweety homolog 2 (Drosophila)	17q25.1				0.447	0.00952	0.0557				1
LOC729020	rcRPE	10q24.33	0.317	0.0437	0.1538							1
TUB	tubby homolog (mouse)	11p15.5							-0.863	3.17	5	1
TUBA1B	tubulin, alpha 1b	12q13.12				-0.385	1.00E-05	0.0027				1
TUBA4A	tubulin, alpha 4a	2q35	-0.645	0.00367	0.0295							1
TUBA4B	tubulin, alpha 4b (pseudogene)	2q35	-0.316	0.00454	0.0343							1
TUBA8	tubulin, alpha 8	22q11.1				-0.393166667	0.000181667	0.006533333				1
TUBAL3	tubulin, alpha-like 3	10p15.1							-0.791	2.86571	7	1
TUBB1	tubulin, beta 1 class VI	20q13.32				0.336	0.00244	0.0271				1
TUBB2A	tubulin, beta 2A class IIa	6p25	-0.535	0.025365	0.091							1
TUBB2B	tubulin, beta 2B class IIb	6p25	-0.293	0.02251	0.1							1
TUBB4	Data not found					-0.561	4.00E-05	0.0047				1
TUBB4A	tubulin, beta 4A class IVa	19p13.3	-0.294	0.030905	0.1181							1
TUBB4B	tubulin, beta 4B class IVb	9q34	-0.511	0.0364	0.137							1
TUBB6	tubulin, beta 6 class V	18p11.21	-1.261	0	1.00E-04							1
TUBB7P	tubulin, beta 7, pseudogene	4q35	-0.409	0.00418	0.0323							1
TUBB8	tubulin, beta 8 class VIII	10p15.3	-0.5305	0.01556	0.06205							1
TUBE1	tubulin, epsilon 1	6q21							-0.758	2.266	5	1
TUBG1	tubulin, gamma 1	17q21				-0.427	0.00105	0.018				1
MYOM3	myomesin family, member 3	1p36.11	0.317	0.01281	0.0691							1
TUBGCP6	tubulin, gamma complex associated protein 6	22q13.31-q13.33				-0.442666667	0.000336667	0.0079				1
TUFM	Tu translation elongation factor, mitochondrial	16p11.2				-0.3175	0.00214	0.0225				1
TUG1	taurine upregulated 1 (non-protein coding)	22q12.2				-0.323	0.00264	0.0281				1
TULP2	tubby like protein 2	19q13.1				-0.419666667	0.002826667	0.0227				1
TUSC1	tumor suppressor candidate 1	9p21.2	-0.209	0.03215	0.1261							1
TUSC5	tumor suppressor candidate 5	17p13.3				-0.346666667	0.016666667	0.074133333				1
TWF1	twinfilin, actin-binding protein, homolog 1 (Drosophila)	12q12	-0.4115	0.01522	0.07415							1
RGPD3	In multiple Geneids		0.317	0.02319	0.1019							1
TXK	TXK tyrosine kinase	4p12	-0.62	0.00414	0.0321	-0.684	8.00E-05	0.0062				2
B4GALT3	UDP-Gal:betaGlcNAc beta 1,4- galactosyltransferase, polypeptide 3	1q21-q23	0.316	0.03699	0.1385							1

TXN	thioredoxin	9q31	-1.122	0.00262	0.0231							1
TXN2	thioredoxin 2	22q13.1	-0.36	0.00616	0.042	-0.3895	0.00025	0.00945				2
TXNDC11	thioredoxin domain containing 11	16p13.13				-0.315	0.004175	0.0347				1
TXNDC12	thioredoxin domain containing 12 (endoplasmic reticulum)	1p32.3				-0.370333333	0.007221667	0.04295				1
TXNDC13	Data not found								-0.819	2.444	5	1
TXNDC15	thioredoxin domain containing 15	5q31.1				-0.341	0.000485	0.01245				1
TXNDC17	thioredoxin domain containing 17	17p13.1	-1.012	1.00E-05	3.00E-04							1
TXNDC3	Data not found					0.365	4.00E-04	0.0117				1
TXNDC8	In multiple Geneids								-1.031	3.51333	6	1
TXNL1	thioredoxin-like 1	18q21.31	-0.504	0.00027	0.0045							1
DDX55	DEAD (Asp-Glu-Ala-Asp) box polypeptide 55	12q24.31	0.316	0.03784	0.1405							1
TXNRD2	thioredoxin reductase 2	22q11.21				-0.425363636	0.00013	0.005581818				1
TYK2	tyrosine kinase 2	19p13.2				-0.556666667	0.002366667	0.024133333				1
TYR	tyrosinase (oculocutaneous albinism IA)	11q14-q21							-0.691	2.81	5	1
TYRO3	TYRO3 protein tyrosine kinase	15q15	-1.125	0.00046	0.0067	-0.312	0.01078	0.0597	0.646	2.384	5	3
DLGAP4	discs, large (Drosophila) homolog-associated protein 4	20q11.23	0.316	0.00383	0.0304							1
TYSND1	trypsin domain containing 1	10q22.1	-0.434	0.00071	0.0092							1
FN3KRP	fructosamine 3 kinase related protein	17q25.3	0.316	0.04157	0.1492							1
TYW1B	tRNA-yW synthesizing protein 1 homolog B (S. cerevisiae)	7q11.23				-0.413	0.012355	0.0645				1
IFT140	intraflagellar transport 140 homolog (Chlamydomonas)	16p13.3	0.316	0.00578	0.0404							1
U2AF1	U2 small nuclear RNA auxiliary factor 1	21q22.3				-0.358333333	0.004333333	0.031333333				1
NCLN	nicalin	19p13.3	0.316	0.0404	0.1465							1
STARD8	StAR-related lipid transfer (START) domain containing 8	Xq13.1	0.316	0.03694	0.1384	0.332	0.00228	0.0262				2
UACA	uveal autoantigen with coiled-coil domains and ankyrin repeats	15q22-q24							-0.89	2.696	5	1
C10orf88	chromosome 10 open reading frame 88	10q26.13	0.315	0.01051	0.0606							1
LOC646813	DEAH (Asp-Glu-Ala-His) box polypeptide 9 pseudogene	11p11.12	0.315	0.02324	0.1021							1
UBA52	ubiquitin A-52 residue ribosomal protein fusion product 1	19p13.1-p12				-0.613	0.00019	0.0084				1
UBA7	ubiquitin-like modifier activating enzyme 7	3p21				-0.458	0.00036	0.01105				1
UBALD1	Data not found		-0.304	0.03943	0.1442							1
UBAP1	ubiquitin associated protein 1	9p13.3	-0.854	3.00E-05	9.00E-04	-0.420777778	0.001328889	0.018233333				2
UBAP2	ubiquitin associated protein 2	9p13.3				-0.427217391	0.002563043	0.023430435				1
UBASH3A	ubiquitin associated and SH3 domain containing A	21q22.3				-0.3485	0.00238	0.02315				1
NAPB	In multiple Geneids		0.315	0.0192	0.0904							1
BRF2	BRF2, subunit of RNA polymerase III transcription initiation factor, BRF1-like	8p11.23	0.314	0.00112	0.0126							1
UBE2A	ubiquitin-conjugating enzyme E2A	Xq24							-0.977	4.02	7	1
UBE2B	ubiquitin-conjugating enzyme E2B	5q31.1							-0.721	2.67714	7	1
NHEJ1	nonhomologous end-joining factor 1	2q35	0.314	0.02777	0.1146							1
UBE2D2	ubiquitin-conjugating enzyme E2D 2	5q31.2	-0.367	0.00023	0.0041	-0.392666667	0.01147	0.050466667				2
UBE2D3	ubiquitin-conjugating enzyme E2D 3	4q24	-0.6	0.006836667	0.0377	-0.382	0.00332	0.0316				2
UBE2DNL	In multiple Geneids		-0.175	0.03102	0.1234							1
UBE2E1	ubiquitin-conjugating enzyme E2E 1	3p24.2							-0.778	2.69	5	1
UBE2E2	ubiquitin-conjugating enzyme E2E 2	3p24.2	-1.019	0.00133	0.0143	-0.338	0.01001	0.0573				2
UBE2E3	ubiquitin-conjugating enzyme E2E 3	2q32.1				0.32925	0.00025	0.008875				1
UBE2G1	ubiquitin-conjugating enzyme E2G 1	17p13.2	-1	1.00E-05	5.00E-04	-0.374636364	0.009069091	0.047818182				2
UBE2G2	ubiquitin-conjugating enzyme E2G 2	21q22.3				-0.333	0.001522	0.0201				1
UBE2H	ubiquitin-conjugating enzyme E2H	7q32				-0.415833333	0.005276667	0.037416667				1
UBE2J1	ubiquitin-conjugating enzyme E2, J1, U	6q15	-0.409	0.03829	0.1417	-0.364	0.00075	0.0155				2
UBE2J2	ubiquitin-conjugating enzyme E2, J2	1p36.33							0.966	2.904	5	1
UBE2K	ubiquitin-conjugating enzyme E2K	4p14				-0.466	0.00037	0.0108				1
UBE2L3	ubiquitin-conjugating enzyme E2L 3	22q11.21	-0.305	0.04032	0.1463	-0.454545455	5.00E-04	0.009563636				2
UBE2N	ubiquitin-conjugating enzyme E2N	12q22				-0.377	0.00267	0.0283				1
UBE2O	ubiquitin-conjugating enzyme E2O	17q25.1				-0.3205	0.005035	0.03595				1
UBE2R2	ubiquitin-conjugating enzyme E2R 2	9p13.3	-0.307	0.04192	0.15	-0.3977	0.002476	0.02271				2
PSMA1	proteasome (prosome, macropain) subunit, alpha type, 1	11p15.1	0.314	0.03263	0.1273							1
QPCTL	glutaminyl-peptide cyclotransferase-like	19q13.32	0.314	0.01571	0.079							1
UBE2Z	ubiquitin-conjugating enzyme E2Z	17q21.32				-0.35	0.01204	0.0635				1
UBE3A	ubiquitin protein ligase E3A	15q11.2							-0.903	3.359375	8	1
UBE3B	ubiquitin protein ligase E3B	12q24.11	-0.405	0.00132	0.0143							1
UBE3C	ubiquitin protein ligase E3C	7q36.3				-0.366555556	0.011738889	0.058077778				1
UBE4B	ubiquitination factor E4B	1p36.3	-0.503	0.00216	0.0202	-0.3998	0.010097	0.05075				2
SMYD5	SMYD family member 5	2p13.2	0.314	0.02475	0.1064							1
UBIAD1	UbiA prenyltransferase domain containing 1	1p36.22				-0.371	0.0053975	0.037975				1
UBL3	ubiquitin-like 3	13q12-q13				0.41	0.00433	0.0363				1
UBL5	ubiquitin-like 5	19p13.3	-0.384	0.02132	0.0968	-0.378	0.01826	0.0808				2
UBLCP1	ubiquitin-like domain containing CTD phosphatase 1	5q33.3							-1.196	4.062	5	1
UBN1	In multiple Geneids					-0.392	0.015663333	0.071116667				1
UBN2	ubinuclein 2	7q34				-0.3482	0.012042	0.06188				1
UBP1	upstream binding protein 1 (LBP-1a)	3p22.3				-0.407	0.02347	0.0937				1
UBQLN2	ubiquilin 2	Xp11.21							-0.834	2.63429	7	1

ZBTB2	zinc finger and BTB domain containing 2	6q25.1	0.314	0.04516	0.1573							1
BCL2L12	BCL2-like 12 (proline rich)	19q13.3	0.313	0.02566	0.1091							1
UBR4	ubiquitin protein ligase E3 component n-recognin 4	1p36.13				0.302	0.00014	0.0075				1
UBR5	ubiquitin protein ligase E3 component n-recognin 5	8q22				0.312	0.00039	0.0116				1
UBR7	ubiquitin protein ligase E3 component n-recognin 7 (putative)	14q32.12	-0.351	0.01773	0.0856							1
UBTD1	ubiquitin domain containing 1	10q24.2				-0.364333333	0.000153333	0.006266667				1
UBTD2	ubiquitin domain containing 2	5q35.1				-0.436428571	0.001288571	0.017142857				1
UBTF	upstream binding transcription factor, RNA polymerase I	17q21.3				-0.372	0.002605	0.02475				1
UBXD5	Data not found								0.737	3.048	5	1
DYRK3	dual-specificity tyrosine-(Y)-phosphorylation regulated kinase 3	1q32.1	0.313	0.03097	0.1234							1
FAF2	Fas associated factor family member 2	5q35.2	0.313	0.01134	0.0638							1
RAB13	RAB13, member RAS oncogene family	1q21.2	0.313	0.03837	0.1419							1
SRMS	src-related kinase lacking C-terminal regulatory tyrosine and N-terminal myristylation sites		0.313	0.01437	0.0744							1
UBXN6	UBX domain protein 6	19p13	-0.347	0.00142	0.015	-0.574333333	4.00E-04	0.0113				2
UBXN7	UBX domain protein 7	3q29				-0.3385	0.00265	0.0247				1
UCHL5	ubiquitin carboxyl-terminal hydrolase L5	1q32				0.302	0.00119	0.0191				1
CLPB	ClpB caseinolytic peptidase B homolog (E. coli)	11q13.4	0.312	0.0288	0.1174	0.354	0.0035	0.0325				2
UCMA	upper zone of growth plate and cartilage matrix associated	10p13	-0.206	0.02945	0.1191	-0.329	0.01537	0.06865				2
MAP1S	microtubule-associated protein 1S	19p13.11	0.312	0.04545	0.158							1
UCRC	Data not found					-0.564	8.00E-05	0.0062				1
UFD1L	ubiquitin fusion degradation 1 like (yeast)	22q11.21				-0.395444444	0.000128889	0.006522222				1
UFL1	UFM1-specific ligase 1	6q16.1	-0.69	0.00028	0.0047							1
MED29	mediator complex subunit 29	19q13.2	0.312	0.03167	0.1249							1
UFSP2	UFM1-specific peptidase 2	4q35.1	-0.6	0.00576	0.0403	-0.376	0.005076667	0.039				2
UGCG	UDP-glucose ceramide glucosyltransferase	9q31	-0.599	0.00618	0.0421							1
UGDH	UDP-glucose 6-dehydrogenase	4p15.1				-0.3315	0.015145	0.0725				1
PEAR1	platelet endothelial aggregation receptor 1	1q23.1	0.312	0.03711	0.1388							1
LRRIQ4	leucine-rich repeats and IQ motif containing 4	3q26.2	0.311	0.01614	0.0804							1
UGP2	UDP-glucose pyrophosphorylase 2	2p14-p13	-0.292	0.00491	0.0361							1
UGT1A1	UDP glucuronosyltransferase 1 family, polypeptide A1	2q37	-1.223	0.00406	0.0317							1
UGT1A8	In multiple Geneids								-0.718	2.65	5	1
RBBP4	retinoblastoma binding protein 4	1p35.1	0.311	0.03471	0.1325							1
UGT2B10	UDP glucuronosyltransferase 2 family, polypeptide B10	4q13.2							-0.788	2.98167	6	1
UGT2B11	UDP glucuronosyltransferase 2 family, polypeptide B11	4q13.2							-0.948	3.49333	6	1
UGT2B15	UDP glucuronosyltransferase 2 family, polypeptide B15	4q13				0.491	0.000295	0.0085				1
UGT2B17	UDP glucuronosyltransferase 2 family, polypeptide B17	4q13				0.895	0	0.0018				1
UGT2B4	UDP glucuronosyltransferase 2 family, polypeptide B4	4q13							-0.819	2.60571	7	1
UGT2B7	UDP glucuronosyltransferase 2 family, polypeptide B7	4q13							-1.048	2.80429	7	1
UGT3A1	UDP glycosyltransferase 3 family, polypeptide A1	5p13.2				0.3318	0.011254	0.05846				1
UGT3A2	UDP glycosyltransferase 3 family, polypeptide A2	5p13.2				0.388666667	0.010513333	0.057333333				1
ATP5EP2	ATP synthase, H+ transporting, mitochondrial F1 complex, epsilon subunit pse	13q12	0.31	0.01603	0.08							1
DNAJC14	DnaJ (Hsp40) homolog, subfamily C, member 14	12q13.2	0.31	0.03432	0.1315							1
UHRF1	In multiple Geneids					-0.467272727	0.00142	0.017981818				1
HNRNPA3P1	heterogeneous nuclear ribonucleoprotein A3 pseudogene 1	10q11.21	0.31	0.022	0.0988							1
UHRF2	In multiple Geneids					-0.358428571	0.002923571	0.02515				1
UIMC1	ubiquitin interaction motif containing 1	5q35.2				-0.3338	0.001296	0.0172				1
PCNXL2	In multiple Geneids		0.309	0.04289	0.1522	0.3662	0.002508	0.02432				2
ULK2	unc-51-like kinase 2 (C. elegans)	17p11.2	-0.5	0.00896	0.0542	-0.36	0.002865	0.0273				2
ULK3	unc-51-like kinase 3 (C. elegans)	15q24.1	-0.857	0.00012	0.0026							1
ULK4	unc-51-like kinase 4 (C. elegans)	3p22.1				-0.334	0.001688333	0.021183333				1
UMODL1	uromodulin-like 1	21q22.3				-0.369222222	0.000806667	0.013277778				1
UNC13A	unc-13 homolog A (C. elegans)	19p13.11				-0.4878	0.003611333	0.027173333				1
UNC13B	unc-13 homolog B (C. elegans)	9p13.3	-0.717	0.0022	0.0204	-0.373125	0.00281125	0.021075				2
UNC13D	unc-13 homolog D (C. elegans)	17q25.1				-0.378	0.00255	0.0277				1
ZNF644	zinc finger protein 644	1p22.2	0.309	0.02533	0.1081				-0.603	2.424	5	2
UNC5A	In multiple Geneids					-0.397090909	0.001052727	0.012945455				1
UNC5B	In multiple Geneids		-0.727	0.00084	0.0104	-0.353	0.00818	0.0512				2
ITGB1BP1	integrin beta 1 binding protein 1	2p25.2	0.308	0.01816	0.087							1
NEURL2	neuralized homolog 2 (Drosophila)	20q13.12	0.308	0.01883	0.0892							1
UNC5D	unc-5 homolog D (C. elegans)	8p12							-0.876	3.33111	9	1
UNC84B	Data not found					-0.391	0.002146	0.02146				1
UNC93A	unc-93 homolog A (C. elegans)	6q27							-0.873	3.1	8	1
ZRSR2	zinc finger (CCCH type), RNA-binding motif and serine/arginine rich 2	Xp22.1	0.308	0.01863	0.0886							1
UNK	unkempt homolog (Drosophila)	17q25.1	-0.244	0.02458	0.1058	-0.329	0.00522	0.0401				2
UNKL	unkempt homolog (Drosophila)-like	16p13.3				-0.36	0.00732	0.0478				1
UNQ3072	Data not found								-0.862	2.998	5	1
UNQ6411	Data not found								-0.758	2.82167	6	1
UNQ689	Data not found								-0.948	3.17833	6	1
UNQ739	Data not found								1.0195	3.16643	7	1

UNQ830	Data not found								0.649	2.698	5	1
UNQ846	Data not found								-0.881	3.16667	6	1
UNQ9356	Data not found								-0.781	2.844	5	1
UPB1	ureidopropionase, beta	22q11.2				-0.413166667	0.000301667	0.0077				1
UPF1	UPF1 regulator of nonsense transcripts homolog (yeast)	19p13.2-p13.11				-0.417909091	0.00677	0.035609091				1
UPF2	UPF2 regulator of nonsense transcripts homolog (yeast)	10p14-p13				-0.425714286	0.009168571	0.045914286				1
UPK1A	uroplakin 1A	19q13.13	-3.148	1.00E-05	4.00E-04				0.639	2.604	5	2
UPK2	uroplakin 2	11q23	-0.775	0.00167	0.0168							1
UPK3A	uroplakin 3A	22q13.31				-0.444333333	0.007536667	0.0334				1
UPK3B	uroplakin 3B	7q11.2	-2.084	0	1.00E-04							1
UPLP	Data not found					-0.493	1.00E-05	0.0029				1
UPP1	uridine phosphorylase 1	7p12.3	-0.907	0.00488	0.0359							1
UPP2	uridine phosphorylase 2	2q24.1				0.3855	2.00E-05	0.00315				1
UPRT	uracil phosphoribosyltransferase (FUR1) homolog (S. cerevisiae)	Xq13.3	-0.457	0.04607	0.1591							1
UQCR	Data not found					-0.389	0.0012	0.0192				1
UQCRC1	ubiquinol-cytochrome c reductase core protein I	3p21.3				-0.4138	0.001248	0.0143				1
UQCRQ	ubiquinol-cytochrome c reductase, complex III subunit VII, 9.5kDa	5q31.1				-0.396	0.000435	0.01185				1
URB1	URB1 ribosome biogenesis 1 homolog (S. cerevisiae)	21q22.11				-0.3382	0.001382	0.0176				1
ADCY6	adenylate cyclase 6	12q12-q13	0.307	0.0107	0.0613							1
EIF1B	eukaryotic translation initiation factor 1B	3p22.1	0.307	0.01226	0.0672							1
USE1	unconventional SNARE in the ER 1 homolog (S. cerevisiae)	19p13.11	-0.467	0.02475	0.1064	-0.574	1.00E-05	0.0027				2
MTX1	metaxin 1	1q21	0.307	0.04974	0.1665							1
R3HCC1L	R3H domain and coiled-coil containing 1-like	10q24.2	0.307	0.00312	0.0263							1
USH1G	In multiple Geneids		-0.807	0	0							1
USH2A	Usher syndrome 2A (autosomal recessive, mild)	1q41				0.359925532	0.002110638	0.021262766				1
USHBP1	Usher syndrome 1C binding protein 1	19p13	-0.177	0.03627	0.1367	-0.445	0.00305	0.026266667				2
USMG5	up-regulated during skeletal muscle growth 5 homolog (mouse)	10q24.33	-0.5265	0.006195	0.0382							1
USO1	USO1 vesicle docking protein homolog (yeast)	4q21.1	-0.478	0.00237	0.0216	-0.392	0.00173	0.0229				2
USP10	ubiquitin specific peptidase 10	16q24.1				-0.34025	0.0089525	0.04955				1
SYS1	SYS1 Golgi-localized integral membrane protein homolog (S. cerevisiae)	20q13.12	0.307	0.0326	0.1272							1
USP15	ubiquitin specific peptidase 15	12q14	-0.378	0.03191	0.1256							1
USP16	ubiquitin specific peptidase 16	21q22.11				-0.393	0	0.0014				1
ZC3H13	zinc finger CCCH-type containing 13	13q14.13	0.307	0.01269	0.0687							1
USP19	ubiquitin specific peptidase 19	3p21.31				-0.44625	0.0003325	0.0083				1
USP20	ubiquitin specific peptidase 20	9q34.11				-0.314	0.03707	0.1236				1
USP22	ubiquitin specific peptidase 22	17p11.2				-0.3735	0.012355	0.06235				1
CRCP	CGRP receptor component	7q11.21	0.306	0.01768	0.0854							1
USP25	ubiquitin specific peptidase 25	21q11.2	-0.451	0.00982	0.0577							1
USP26	ubiquitin specific peptidase 26	Xq26.2							-0.721	2.8275	8	1
DNMT3A	DNA (cytosine-5-)-methyltransferase 3 alpha	2p23	0.306	0.03847	0.1421							1
USP30	In multiple Geneids					-0.423666667	0.000266667	0.008266667				1
USP32	ubiquitin specific peptidase 32	17q23.3				0.45325	0.00045	0.0106				1
LIMD1	LIM domains containing 1	3p21.3	0.306	0.01649	0.0814							1
USP36	ubiquitin specific peptidase 36	17q25.3				-0.303	0.00553	0.0413				1
USP38	ubiquitin specific peptidase 38								-0.969	2.818	5	1
PARVB	parvin, beta	22q13.2-q13.33	0.306	0.03622	0.1366							1
USP4	ubiquitin specific peptidase 4 (proto-oncogene)	3p21.3	-0.412	0.00157	0.0161	-0.4143	0.001576	0.0192				2
USP24	ubiquitin specific peptidase 24	1p32.3	0.306	0.02909	0.1181							1
USP43	ubiquitin specific peptidase 43	17p13.1				-0.406615385	0.005550769	0.033261538				1
USP44	ubiquitin specific peptidase 44	12q22							-0.84	3.16	5	1
USP46	ubiquitin specific peptidase 46	4q12	-1.387	0	0	-0.3225	0.01633	0.07565				2
USP47	ubiquitin specific peptidase 47	11p15.3							-0.906	2.932	5	1
USP48	ubiquitin specific peptidase 48	1p36.12				-0.346	0.009514	0.0521				1
USP5	ubiquitin specific peptidase 5 (isopeptidase T)	12p13				-0.344	0.00076	0.0156				1
USP50	ubiquitin specific peptidase 50	15q21.1				-0.323	0.02251	0.0915				1
USP53	ubiquitin specific peptidase 53	4q26							-0.677	2.558	5	1
USP54	ubiquitin specific peptidase 54	10q22.2	-0.579	0.0026	0.0231	-0.3858	0.000558	0.01217				2
USP6	ubiquitin specific peptidase 6 (Tre-2 oncogene)	17p13	-0.249	0.02508	0.1073	-0.373	0.00163	0.0222				2
USP6NL	USP6 N-terminal like	10p13	-1.601	4.00E-05	0.0011	-0.324	0.02849	0.1052				2
USP7	ubiquitin specific peptidase 7 (herpes virus-associated)	16p13.3				-0.302	0.00738	0.0485				1
USP9Y	ubiquitin specific peptidase 9, Y-linked	Yq11.2	-1.566	0	1.00E-04	-0.416875	0.0318375	0.1099625				2
WDSUB1	WD repeat, sterile alpha motif and U-box domain containing 1	2q24.2	0.306	0.04298	0.1525							1
UST	uronyl-2-sulfotransferase	6q25.1				0.341	0.0084	0.052				1
ZFR	zinc finger RNA binding protein	5p13.3	0.306	0.03663	0.1376							1
UTP20	UTP20, small subunit (SSU) processome component, homolog (yeast)	12q23							-0.829	2.612	5	1
CLK2	CDC-like kinase 2	1q21	0.305	0.02451	0.1056							1
KARS	lysyl-tRNA synthetase	16q23.1	0.305	0.04872	0.1645							1
UTRN	utrophin	6q24	-0.38	0.03962	0.1446							1
UTS2	urotensin 2	1p36				-0.304	0.00909	0.0543				1

UTS2D	urotensin 2 domain containing	3q28				0.379	0.001055	0.0151	-0.809	3.132	5	2
UTY	ubiquitously transcribed tetratricopeptide repeat gene, Y-linked	Yq11	-0.915	0.02414	0.1046	-0.469740741	0.023603704	0.090048148				2
PNLIPRP2	pancreatic lipase-related protein 2	10q25.3	0.305	0.03931	0.1439							1
VAC14	Vac14 homolog (S. cerevisiae)	16q22.1				-0.354	0.005	0.03264				1
VAMP2	vesicle-associated membrane protein 2 (synaptobrevin 2)	17p13.1	-0.577	0.01628	0.0808							1
VAMP3	vesicle-associated membrane protein 3 (cellubrevin)	1p36.23	-0.35	0.03639	0.137							1
VAMP8	vesicle-associated membrane protein 8 (endobrevin)	2p12-p11.2	-0.503	0.0017	0.017							1
VANGL1	vang-like 1 (van gogh, Drosophila)	1p13.1	-0.653	0.00192	0.0185							1
VAPA	VAMP (vesicle-associated membrane protein)-associated protein A, 33kDa	18p11.22				-0.302	0.00153	0.0215				1
VAPB	In multiple Geneids					0.36625	0.0050875	0.0331	-0.805	2.79	5	2
VASN	vasorin	16p13.3	-0.741	0.00959	0.0567							1
SPAG9	sperm associated antigen 9	17q21.33	0.305	0.04358	0.1537							1
VAT1	vesicle amine transport protein 1 homolog (T. californica)	17q21	-1.085	0.00021	0.0037	-0.41	0.00388	0.0343				2
VAT1L	vesicle amine transport protein 1 homolog (T. californica)-like	16q23.1				-0.3502	0.00249	0.02384				1
VAV1	vav 1 guanine nucleotide exchange factor	19p13.2				-0.405230769	0.002499231	0.025984615				1
C11orf53	chromosome 11 open reading frame 53	11q23.1	0.304	0.03058	0.1223				-0.898	3.12625	8	2
VAV3	vav 3 guanine nucleotide exchange factor	1p13.3	-0.917	0.00064	0.0086							1
VAX1	ventral anterior homeobox 1	10q26.1				-0.474	0.00033	0.0108				1
DNAJA1	DnaJ (Hsp40) homolog, subfamily A, member 1	9p13.3	0.304	0.024	0.1041							1
GPALPP1	Data not found		0.304	0.00462	0.0346							1
RRP15	ribosomal RNA processing 15 homolog (S. cerevisiae)	1q41	0.304	0.03761	0.14	0.318	0.00011	0.0069				2
VCL	vinculin	10q22.2				-0.340666667	0.002026667	0.021866667				1
VCP	valosin containing protein	9p13.3				-0.352857143	0.007045714	0.028185714				1
DFFB	DNA fragmentation factor, 40kDa, beta polypeptide (caspase-activated DNase	1p36.3	0.303	0.03014	0.121							1
VCX3B	In multiple Geneids		-0.602	0.00301	0.0257							1
VCY	variable charge, Y-linked	Yq11.221	-0.224	0.00328	0.0273				0.582	2.94667	6	2
VCY1B	variable charge, Y-linked 1B	Yq11.21							0.582	2.94667	6	1
VDAC1	voltage-dependent anion channel 1	5q31				-0.359666667	0.008736667	0.0492				1
VDAC2	voltage-dependent anion channel 2	10q22	-0.817	7.00E-05	0.0015	-0.3	0.00615	0.0438				2
TRAF3	TNF receptor-associated factor 3	14q32.32	0.303	0.04908	0.1652							1
VEGFC	vascular endothelial growth factor C	4q34.3				-0.3175	0.02924	0.103				1
VEPH1	ventricular zone expressed PH domain homolog 1 (zebrafish)	3q24-q25				0.322777778	0.006194444	0.038922222	-0.769	2.92	7	2
VGLL1	vestigial like 1 (Drosophila)	Xq26.3							-0.727	2.85571	7	1
VGLL2	vestigial like 2 (Drosophila)	6q22.1	-0.242	0.0061	0.0418							1
VGLL3	vestigial like 3 (Drosophila)	3p12.1				0.448	0.00785	0.0501				1
VGLL4	vestigial like 4 (Drosophila)	3p25.3				-0.3862	0.001292	0.01733				1
HSPBP1	In multiple Geneids		0.302	0.01489	0.0762							1
NADK	NAD kinase	1p36.33	0.302	0.04573	0.1585							1
LIMS3	LIM and senescent cell antigen-like domains 3	2q13	0.301	0.01476	0.0757							1
PPIG	peptidylprolyl isomerase G (cyclophilin G)	2q31.1	0.301	0.04123	0.1485							1
RNF216	ring finger protein 216	7p22.1	0.301	0.01058	0.0609							1
VIP	vasoactive intestinal peptide	6q25							-0.935	3.37889	9	1
VIPR1	vasoactive intestinal peptide receptor 1	3p22				-0.3675	0.00053	0.0125				1
VIT	vitrin	2p22.2							-0.754	2.69286	7	1
SNX7	sorting nexin 7	1p21.3	0.301	0.04555	0.1582	0.371	0.00061	0.0141	-0.881	2.704	5	3
VLDLR	very low density lipoprotein receptor	9p24	-1.4175	5.00E-06	2.00E-04	-0.356	0.00017	0.0081				2
VMAC	vimentin-type intermediate filament associated coiled-coil protein	19p13.3				-0.5	8.00E-05	0.006				1
TYW5	tRNA-yW synthesizing protein 5	2q33.1	0.301	0.04993	0.167							1
VN1R4	vomeronasal 1 receptor 4	19q13.42							-0.923	3.474	5	1
ZDHHC17	zinc finger, DHHC-type containing 17	12q21.2	0.301	0.04237	0.1511							1
BIK	BCL2-interacting killer (apoptosis-inducing)	22q13.31	0.3	0.03278	0.1277							1
VNN3	vanin 3	6q23.2							-1.101	3.25	5	1
KIF9	kinesin family member 9	3p21.31	0.3	0.02537	0.1082							1
VPRBP	Vpr (HIV-1) binding protein	3p21.2				-0.35625	0.002679167	0.024441667				1
VPS13B	vacuolar protein sorting 13 homolog B (yeast)	8q22.2				0.342555556	0.00156	0.014288889				1
SLC38A7	solute carrier family 38, member 7	16q21	0.3	0.03408	0.1309							1
VPS13D	vacuolar protein sorting 13 homolog D (S. cerevisiae)	1p36.22	-0.995	0	0	-0.32	0.00183	0.0231				2
VPS25	vacuolar protein sorting 25 homolog (S. cerevisiae)	17q21.31				-0.352	0.00427	0.036				1
VPS26B	vacuolar protein sorting 26 homolog B (S. pombe)	11q25	-0.297	0.03487	0.133							1
VPS29	vacuolar protein sorting 29 homolog (S. cerevisiae)	12q24				-0.314	0.00055	0.0135				1
VCPIP1	valosin containing protein (p97)/p47 complex interacting protein 1	8q13	0.3	0.04563	0.1583							1
VPS35	In multiple Geneids					-0.307	0.00077	0.0156				1
VPS37B	vacuolar protein sorting 37 homolog B (S. cerevisiae)	12q24.31	-0.821	0.00033	0.0053							1
VPS37C	vacuolar protein sorting 37 homolog C (S. cerevisiae)	11q12.2	-0.279	0.00669	0.0445	-0.323	0.00191	0.024				2
VPS39	vacuolar protein sorting 39 homolog (S. cerevisiae)	15q15.1							-0.751	2.926	5	1
AGFG1	ArfGAP with FG repeats 1	2q36.3	0.299	0.03116	0.1238							1
VPS45A	Data not found								-0.678	2.558	5	1
VPS4A	vacuolar protein sorting 4 homolog A (S. cerevisiae)	16q22.1	-0.414	0.00805	0.0504	-0.463	0.001903333	0.02				2
VPS4B	vacuolar protein sorting 4 homolog B (S. cerevisiae)	18q21.33	-1.407	0	1.00E-04							1

VPS53	vacuolar protein sorting 53 homolog (S. cerevisiae)	17p13.3	-0.4875	0.00025	0.0043	-0.374357143	0.008161429	0.044864286				2
IRAK1	interleukin-1 receptor-associated kinase 1	Xq28	0.299	0.01319	0.0702							1
VPS8	vacuolar protein sorting 8 homolog (S. cerevisiae)	3q27.2				0.388333333	0.004853333	0.036033333				1
VPS9D1	Data not found		-0.719	0.00073	0.0094							1
VRK2	vaccinia related kinase 2	2p16.1				0.342571429	0.001922857	0.020785714				1
VRK3	vaccinia related kinase 3	19q13				-0.331666667	0.007893333	0.048766667				1
VSIG1	V-set and immunoglobulin domain containing 1	Xq22.3				0.398	0.00062	0.0142	-0.873	3.286	5	2
VSIG10L	V-set and immunoglobulin domain containing 10 like	19q13.41	-3.18	0	0							1
VSIG4	V-set and immunoglobulin domain containing 4	Xq12-q13.3				0.316	0.00227	0.0247				1
VSNL1	visinin-like 1	2p24.3				0.3378	0.004478	0.03338				1
VSTM1	V-set and transmembrane domain containing 1	19q13.42	-0.217	0.03945	0.1442	-0.74	0.018785	0.075				2
VSTM2A	V-set and transmembrane domain containing 2A	7p11.2				0.3792	0.000486	0.01164				1
VSX1	visual system homeobox 1	20p11.21	-0.255	0.01208	0.0666							1
VSX2	visual system homeobox 2	14q24.3	-0.275	0.00459	0.0345	-0.451	0.00087	0.0161				2
VTA1	Vps20-associated 1 homolog (S. cerevisiae)	6q24.1				0.313	0.01766	0.0792				1
RBM28	RNA binding motif protein 28	7q32.1	0.299	0.03281	0.1278							1
VTI1A	vesicle transport through interaction with t-SNAREs homolog 1A (yeast)	10q25.2				0.305	0.01416	0.0697				1
VTI1B	vesicle transport through interaction with t-SNAREs homolog 1B (yeast)	14q24.1	-0.565	0.00182	0.0179	-0.331	0.00159	0.0219				2
ADAMTS7	ADAM metallopeptidase with thrombospondin type 1 motif, 7	15q24.2	0.298	0.00687	0.0453							1
VWA2	von Willebrand factor A domain containing 2	10q25.3				-0.313	0.00289	0.0294				1
VWA3A	von Willebrand factor A domain containing 3A	16p12.2	-0.258	0.01051	0.0606	-0.318	0.00146	0.021				2
VWA3B	von Willebrand factor A domain containing 3B	2q11.2				0.387	5.00E-04	0.0128				1
VWA5A	von Willebrand factor A domain containing 5A	11q24.1				0.369	0.00024	0.0094				1
VWA5B1	von Willebrand factor A domain containing 5B1	1p36.12				-0.301	0.00089	0.0168				1
BEST1	bestrophin 1	11q13	0.298	0.01191	0.0659							1
CTNNBL1	catenin, beta like 1	20q11.23-q12	0.298	0.01586	0.0795	0.3402	0.004032	0.03152				2
VWC2	von Willebrand factor C domain containing 2	7p12.2				0.38524	0.0124756	0.060696				1
VWC2L	von Willebrand factor C domain containing protein 2-like	2q34-q35				0.349	3.00E-04	0.0097				1
VWDE	von Willebrand factor D and EGF domains	7p21.3				0.3696	0.002294	0.0255				1
VWF	von Willebrand factor	12p13.3				-0.33	0.00016	0.008				1
WAC	In multiple Geneids					-0.318	0.018755	0.07435				1
WAPAL	wings apart-like homolog (Drosophila)	10q23.2				-0.364	0.00814	0.0414				1
WARS	tryptophanyl-tRNA synthetase	14q32.31				-0.352	0.00225	0.026				1
WASF1	WAS protein family, member 1	6q21	-0.874	0.0293	0.1187							1
WASF2	WAS protein family, member 2	1p36.11	-1.246	0	2.00E-04	-0.415916667	0.005135833	0.036675				2
WASL	Wiskott-Aldrich syndrome-like	7q31.3	-0.293	0.04659	0.1601							1
POMGNT1	protein O-linked mannose beta1,2-N-acetylglucosaminyltransferase	1p34.1	0.298	0.03981	0.1451							1
WBP2NL	WBP2 N-terminal like	22q13.2				-0.41	0.000286	0.00814				1
ZNF766	zinc finger protein 766	19q13.41	0.298	0.04981	0.1667							1
WBSCR16	In multiple Geneids					-0.393	0.04846	0.146				1
WBSCR17	In multiple Geneids					0.393	0.00016	0.008				1
FAAH2	fatty acid amide hydrolase 2	Xp11.21	0.297	0.04602	0.159	0.3015	0.008005	0.0487				2
WBSCR27	Williams Beuren syndrome chromosome region 27	7q11.23				-0.331	0.03161	0.1121				1
RBM19	RNA binding motif protein 19	12q24.21	0.296	0.04518	0.1573							1
WDFY2	WD repeat and FYVE domain containing 2	13q14.3				0.431	0.00331	0.0316				1
WDFY3	WD repeat and FYVE domain containing 3	4q21.23	-0.425	0.04257	0.1515	-0.326166667	0.003748333	0.030683333				2
WDFY3-AS2	WDFY3 antisense RNA 2 (non-protein coding)	4q21.3	-0.253	0.02587	0.1097							1
WDHD1	WD repeat and HMG-box DNA binding protein 1	14q22.2				-0.343	0.00111	0.0185				1
WDR1	WD repeat domain 1	4p16.1	-0.511	0.00133	0.0143	-0.3155	0.002625	0.02795				2
CDC73	cell division cycle 73, Paf1/RNA polymerase II complex component, homolog (	1q25	0.295	0.00103	0.0119	0.3385	0.000945	0.017				2
WDR16	In multiple Geneids					-0.35575	0.00811375	0.0460625				1
WDR17	WD repeat domain 17	4q34				-0.346	0.0107	0.0594				1
WDR19	In multiple Geneids					-0.368666667	0.005543333	0.041133333				1
WDR20	WD repeat domain 20	14q32.31	-0.257	0.02703	0.1127	-0.358666667	0.003933333	0.032566667				2
WDR24	In multiple Geneids								0.972	2.7675	6	1
WDR25	WD repeat domain 25	14q32.2				-0.3125	0.0052025	0.038075				1
WDR26	WD repeat domain 26	1q42.13	-1.126	0.00012	0.0026							1
WDR27	In multiple Geneids								-0.556	2.428	5	1
WDR31	WD repeat domain 31	9q32	-0.288	0.0375	0.1397							1
RBM45	RNA binding motif protein 45	2q31.2	0.295	0.02103	0.0959							1
RETNLB	resistin like beta	3q13.1	0.295	0.04497	0.1568							1
WDR36	WD repeat domain 36	5q22.1							-0.856	3.126	5	1
WDR4	WD repeat domain 4	21q22.3				-0.459	0.000681667	0.011383333				1
WDR41	WD repeat domain 41	5q13.3	-0.722	9.00E-05	0.0021							1
WDR44	WD repeat domain 44	Xq24							-0.724	2.98	5	1
WDR45	WD repeat domain 45	Xp11.23				-0.41	0.00011	0.0068				1
WDR45L	WDR45-like	17q25.3				-0.366	0.00129	0.0198				1
WDR47	WD repeat domain 47	1p13.3	-1.569	0	1.00E-04	-0.3486	0.004042	0.03138				2
WDR49	WD repeat domain 49	3q26.1				0.34275	0.00150625	0.0189	-0.864	3.245	6	2

WDR51A	Data not found					-0.419142857	0.000512857	0.010785714				1
WDR51B	Data not found								-0.558	2.408	5	1
ZSCAN9	Data not found		0.295	0.02141	0.0971							1
WDR55	WD repeat domain 55	5q31.3	-0.269	0.04652	0.1599	-0.362	0.0013175	0.018225				2
WDR59	WD repeat domain 59	16q23.1				-0.3695	0.004371429	0.030657143				1
WDR5B	WD repeat domain 5B	3q21.1							-0.812	2.715	6	1
ZBTB12	zinc finger and BTB domain containing 12	6p21.33	0.294	0.02681	0.1121							1
WDR60	WD repeat domain 60	7q36.3				-0.378333333	0.00335	0.028533333				1
WDR62	WD repeat domain 62	19q13.12				-0.476	0.00026	0.0097				1
WDR63	WD repeat domain 63	1p22.3				-0.329	0.00176	0.023				1
WDR64	WD repeat domain 64	1q43							-0.878	3.136	5	1
WDR66	WD repeat domain 66	12q24.31				-0.362166667	0.00925	0.044				1
WDR7	WD repeat domain 7	18q21.31	-0.355	0.02723	0.1132							1
AP5Z1	adaptor-related protein complex 5, zeta 1 subunit	7p22.2	0.293	0.04375	0.1539							1
FAM58A	family with sequence similarity 58, member A	Xq28	0.293	0.04012	0.1458							1
WDR75	WD repeat domain 75	2q32.2							-0.783	2.79571	7	1
WDR76	WD repeat domain 76	15q15.3				-0.4	0.01287	0.066				1
WDR79	Data not found								-0.565	2.51917	6	1
WDR81	WD repeat domain 81	17p13.3				-0.409	0.01198	0.056966667				1
WDR83OS	WD repeat domain 83 opposite strand	19p13.2	-0.4	0.00619	0.0422							1
WDR86	WD repeat domain 86	7q36.1				-0.35325	0.004435	0.03335				1
WDR87	WD repeat domain 87	19q13.13				-0.301	4.00E-05	0.0049				1
WDR88	WD repeat domain 88	19q13.11				-0.398	0.00368	0.0334				1
WDR89	WD repeat domain 89	14q23.2				-0.3285	0.001955	0.0231				1
WDR91	WD repeat domain 91	7q33	-0.662	7.00E-04	0.0091							1
WDSOF1	Data not found								-1.105	3.485	6	1
MATR3	matrin 3	5q31.2	0.293	0.00187	0.0182							1
WDTC1	WD and tetratricopeptide repeats 1	1p36.11				-0.412315789	0.008313684	0.046257895				1
WEE1	WEE1 homolog (S. pombe)	11p15.3-p15.1	-0.888	0.03061	0.1224							1
WFDC1	WAP four-disulfide core domain 1	16q24.3				-0.3645	0.0119	0.05365				1
WFDC10A	WAP four-disulfide core domain 10A	20q13.12							-0.829	2.645	6	1
WFDC10B	WAP four-disulfide core domain 10B	20q13.12				0.376	1.00E-05	0.003	-0.851	3.095	6	2
WFDC12	WAP four-disulfide core domain 12	20q12-q13.1	-1.043	0.00436	0.0332							1
WFDC13	WAP four-disulfide core domain 13	20q13.12				0.376	1.00E-05	0.003				1
NDUFAF5	NADH dehydrogenase (ubiquinone) complex I, assembly factor 5	20p12.1	0.293	0.03873	0.1426							1
WFDC5	WAP four-disulfide core domain 5	20q13.12				0.329	0.00325	0.0313				1
WFDC6	WAP four-disulfide core domain 6	20q13.12	-0.259	0.02442	0.1053							1
WFDC8	WAP four-disulfide core domain 8	20q13.12				0.326	3.00E-04	0.0103	-0.72	2.536	5	2
ATXN2	ataxin 2	12q24.1	0.292	0.00682	0.0451							1
WHSC1	Wolf-Hirschhorn syndrome candidate 1	4p16.3				-0.401777778	0.001958889	0.020038889				1
WHSC1L1	In multiple Geneids								-0.887	2.9225	8	1
WHSC2	Wolf-Hirschhorn syndrome candidate 2	4p16.3				-0.3935	0.003335	0.031				1
WIBG	within bgcn homolog (Drosophila)	12q13.2	-0.758	3.00E-05	8.00E-04							1
WIF1	WNT inhibitory factor 1	12q14.3	-0.514	0.00751	0.0482							1
WIPF1	WAS/WASL interacting protein family, member 1	2q31.1				0.306	0	0.0018				1
WIPF2	WAS/WASL interacting protein family, member 2	17q21.2	-0.268	0.03483	0.1329	-0.324	0.001345	0.01885				2
WIPF3	WAS/WASL interacting protein family, member 3	7p14.3				0.318	0.00519	0.04				1
WIPI2	WD repeat domain, phosphoinositide interacting 2	7p22.1				-0.381	0.00755	0.049				1
KIAA1841	KIAA1841	2q14	0.292	0.04414	0.155							1
MFF	mitochondrial fission factor	2q36.3	0.292	0.04464	0.156							1
WIT1	Data not found								0.768	2.7395	8	1
WIZ	widely interspaced zinc finger motifs	19p13.1				-0.335	0.001845	0.0219				1
WNK1	WNK lysine deficient protein kinase 1	12p13.3	-0.9145	0.003755	0.0247							1
RPL41	ribosomal protein L41	12q13	0.292	0.03179	0.1252							1
WNK3	In multiple Geneids		-0.513	0.02232	0.0995							1
WNK4	In multiple Geneids		-1.094	0.00255	0.0227	-0.345	0.001576667	0.018566667				2
WNT1	wingless-type MMTV integration site family, member 1	12q13				-0.309	0.00013	0.0073				1
WNT10A	In multiple Geneids					-0.392	0.00017	0.0082				1
WNT10B	wingless-type MMTV integration site family, member 10B	12q13				-0.398	0.00037	0.0113				1
WNT2B	wingless-type MMTV integration site family, member 2B	1p13							-0.609	2.57	6	1
WNT3	wingless-type MMTV integration site family, member 3	17q21				-0.366	0.00337	0.0319				1
WNT3A	wingless-type MMTV integration site family, member 3A	1q42							0.4995	2.630335	5.5	1
WNT4	wingless-type MMTV integration site family, member 4	1p36.23-p35.1	-1.187	0	1.00E-04	-0.37025	0.0013475	0.017375				2
WNT5A	wingless-type MMTV integration site family, member 5A	3p21-p14	-1.029	0.0064	0.0432				0.544	2.661	5	2
WNT5B	wingless-type MMTV integration site family, member 5B	12p13.3	-0.282	0.01646	0.0814							1
WNT6	wingless-type MMTV integration site family, member 6	2q35	-0.332	0.0077	0.049							1
WNT7A	wingless-type MMTV integration site family, member 7A	3p25	-0.328	0.02398	0.1041	-0.338	0.001296667	0.019033333				2
WNT7B	wingless-type MMTV integration site family, member 7B	22q13	-0.633	0.00182	0.0179	-0.43275	0.00016	0.006575				2
WNT8A	wingless-type MMTV integration site family, member 8A	5q31	-0.288	0.0026	0.0231	-0.42025	0.00428	0.030175				2

WNT8B	wingless-type MMTV integration site family, member 8B	10q24				-0.381	0.00193	0.020333333				1
WNT9A	wingless-type MMTV integration site family, member 9A	1q42	-0.402	0.01459	0.075							1
C2orf88	chromosome 2 open reading frame 88	2q32.2	0.291	0.02157	0.0975							1
WRB	tryptophan rich basic protein	21q22.3				-0.383666667	0.000206667	0.0087				1
COMMD4	COMM domain containing 4	15q24.2	0.291	0.0388	0.1428							1
DDX56	DEAD (Asp-Glu-Ala-Asp) box helicase 56	7p13	0.29	0.04412	0.1549				-0.725	2.535	6	2
WSB2	WD repeat and SOCS box containing 2	12q24.23	-0.456	0.00858	0.0526				0.696	2.90286	7	2
WSCD1	WSC domain containing 1	17p13.2				-0.3544	0.004034	0.02954				1
WSCD2	WSC domain containing 2	12q23.3				-0.312	0.00612	0.0437				1
WT1	Wilms tumor 1	11p13							0.814	2.7844675	6.75	1
WT1-AS	WT1 antisense RNA (non-protein coding)	11p13	-0.294	0.01254	0.0682							1
WWC1	WW and C2 domain containing 1	5q34	-0.603	0.0094	0.056	-0.380307692	0.001223077	0.016073077				2
WWC2	WW and C2 domain containing 2	4q35.1				-0.381916667	0.006212917	0.040133333				1
WWC2-AS2	WWC2 antisense RNA 2 (non-protein coding)	4q35.1	-0.377	0.00031	0.005							1
WWC3	WWC family member 3	Xp22.32	-0.575	0.00414	0.0321				0.494	2.606	5	2
WWOX	WW domain containing oxidoreductase	16q23.3-q24.1	-0.544	0.00018	0.0034	-0.389846154	0.008627115	0.045403846				2
WWP2	WW domain containing E3 ubiquitin protein ligase 2	16q22.1				-0.372	0.004729412	0.035323529				1
XAB2	XPA binding protein 2	19p13.2	-0.287	0.01004	0.0586	-0.481	0.00536	0.0407				2
XAF1	XIAP associated factor 1	17p13.1				-0.373	0.000755	0.0125				1
XAGE1	X antigen family, member 1	Xp11.22							0.9474	3.145866	5.2	1
XAGE1A	X antigen family, member 1A	Xp11.22	-0.219	0.02816	0.1157							1
XBP1	X-box binding protein 1	22q12.1				-0.491	0.00034	0.0103				1
XCR1	chemokine (C motif) receptor 1	3p21.3	-0.959	7.00E-05	0.0017	-0.302	0.00498	0.0391				2
XDH	xanthine dehydrogenase	2p23.1							-0.986	2.886	5	1
XG	Xg blood group	Xp22.33	-1.461	1.00E-05	5.00E-04	-0.409	0.002735	0.024				2
XGPY2	In multiple Geneids					-0.447125	0.002945	0.02595				1
XIRP2	xin actin-binding repeat containing 2	2q24.3				0.335	1.00E-05	0.003				1
XK	X-linked Kx blood group (McLeod syndrome)	Xp21.1				0.313	0.00415	0.0355				1
XKR3	XK, Kell blood group complex subunit-related family, member 3	22q11.1							-1.001	2.96571	7	1
XKR4	XK, Kell blood group complex subunit-related family, member 4	8q12.1				0.32175	0.0009325	0.014725				1
XKR6	XK, Kell blood group complex subunit-related family, member 6	8p23.1				0.478	0.04957	0.1481				1
XKR7	XK, Kell blood group complex subunit-related family, member 7	20q11.21	-0.227	0.04298	0.1525							1
XKR8	XK, Kell blood group complex subunit-related family, member 8	1p35.3				-0.368	0.01353	0.0679				1
JMJD6	jumonji domain containing 6	17q25	0.29	0.00491	0.036							1
XKRX	XK, Kell blood group complex subunit-related, X-linked	Xq22.1	-0.93	0.00103	0.0119							1
XKRY	XK, Kell blood group complex subunit-related, Y-linked	Yq11.222							-0.868	3.19625	8	1
XLKD1	Data not found								-0.963	3.38333	6	1
XPA	xeroderma pigmentosum, complementation group A	9q22.3	-0.363	0.00192	0.0185							1
CALR	calreticulin	19p13.3-p13.2	0.289	0.01078	0.0615							1
VPS33A	vacuolar protein sorting 33 homolog A (S. cerevisiae)	12q24.31	0.289	0.02051	0.0943							1
XPNPEP3	X-prolyl aminopeptidase (aminopeptidase P) 3, putative	22q13.2				-0.383	0.006130909	0.035318182				1
FAM20B	family with sequence similarity 20, member B	1q25	0.288	0.01687	0.0828							1
NUDT16	nudix (nucleoside diphosphate linked moiety X)-type motif 16	3q22.1	0.288	0.03685	0.1381							1
NUP85	nucleoporin 85kDa	17q25.1	0.288	0.0222	0.0993							1
XPO6	exportin 6	16p11.2				-0.322	0.00104	0.018				1
XPO7	exportin 7	8p21				-0.327	0.00263	0.0281				1
PHIP	In multiple Geneids		0.288	0.03146	0.1245							1
XRCC1	X-ray repair complementing defective repair in Chinese hamster cells 1	19q13.2				-0.32	0.007475	0.0488				1
XRCC2	X-ray repair complementing defective repair in Chinese hamster cells 2	7q36.1				-0.3305	0.0239	0.0934				1
XRCC3	X-ray repair complementing defective repair in Chinese hamster cells 3	14q32.3				-0.396	0.00291	0.0295				1
XRCC6	X-ray repair complementing defective repair in Chinese hamster cells 6	22q13.2				-0.309	0.000975	0.01735				1
XRCC6BP1	XRCC6 binding protein 1	12q14.1	-0.573	0.00176	0.0175							1
BANF1	barrier to autointegration factor 1	11q13.1	0.287	0.02854	0.1167							1
XXYLT1	xyloside xylosyltransferase 1	3q29	-0.333	0.01601	0.08							1
XYLT1	xylosyltransferase I	16p12.3				-0.326	0.00492	0.0389				1
YAF2	YY1 associated factor 2	12q12				-0.321	0.001595	0.02125				1
YARS	tyrosyl-tRNA synthetase	1p35.1				-0.356	0.004056667	0.0324				1
YARS2	tyrosyl-tRNA synthetase 2, mitochondrial	12p11.21							-0.747	2.67833	6	1
YBEY	ybeY metallopeptidase (putative)	21q22.3	-0.284	0.02346	0.1027							1
YBX2	Y box binding protein 2	17p13.1				-0.477	6.00E-05	0.0055				1
YBX3	Data not found		-0.861	0.00228	0.021							1
BTBD16	BTB (POZ) domain containing 16	10q26.13	0.287	0.01965	0.0916							1
LOXL3	lysyl oxidase-like 3	2p13	0.287	0.02016	0.0931							1
ACD	adrenocortical dysplasia homolog (mouse)	16q22.1	0.286	0.01152	0.0643							1
YIF1B	Yip1 interacting factor homolog B (S. cerevisiae)	19q13.2	-0.298	0.01466	0.0753	-0.448	1.00E-05	0.0032				2
YIPF2	Yip1 domain family, member 2	19p13.2				-0.595	4.00E-05	0.0049				1
YIPF3	Yip1 domain family, member 3	6p21.1				0.415	0.02519	0.0978				1
YIPF5	Yip1 domain family, member 5	5q31.3	-0.263	0.0382	0.1415							1
YIPF7	Yip1 domain family, member 7	4p12							-0.957	3.44111	9	1

YJEFN3	YjeF N-terminal domain containing 3	19p13.11				-0.382	0.00597	0.0431				1
YLPM1	YLP motif containing 1	14q24.3				-0.3505	0.00033	0.01075				1
YME1L1	YME1-like 1 (S. cerevisiae)	10p14				-0.3	0.0069	0.0467				1
YOD1	YOD1 OTU deubiquinating enzyme 1 homolog (S. cerevisiae)	1q32.2	-1.385	0.000145	0.0029							1
YPEL1	In multiple Geneids					-0.4655	0.00124625	0.014175				1
ACTR5	In multiple Geneids		0.286	0.01221	0.067							1
YPEL3	yippee-like 3 (Drosophila)	16p11.2	-0.327	0.03803	0.141							1
YTHDC1	YTH domain containing 1	4q13.2	-0.281	0.04139	0.1489							1
YTHDC2	YTH domain containing 2	5q22.2							-0.736	2.69	5	1
SART1	squamous cell carcinoma antigen recognized by T cells	11q13.1	0.286	0.01076	0.0615							1
YTHDF2	YTH domain family, member 2	1p35				-0.37	0.015568333	0.068683333				1
YWHAE	tyrosine 3-monooxygenase/tryptophan 5-monooxygenase activation protein,	17p13.3				-0.422	0.0215975	0.08715				1
INHBB	inhibin, beta B	2cen-q13	0.285	0.0115	0.0643							1
YWHAZ	tyrosine 3-monooxygenase/tryptophan 5-monooxygenase activation protein,	8q23.1							-1.074	2.834	5	1
ZACN	zinc activated ligand-gated ion channel	17q25.3	-0.246	0.04563	0.1583							1
ZADH2	zinc binding alcohol dehydrogenase domain containing 2	18q22.3							-0.681	2.74833	6	1
LY6G5C	lymphocyte antigen 6 complex, locus G5C	6p21.33	0.285	0.02434	0.1051							1
ZAN	zonadhesin	7q22				0.429	0.012505	0.06495				1
ZAR1L	zygote arrest 1-like	13q13.1	-0.187	0.03027	0.1214							1
ZBBX	zinc finger, B-box domain containing	3q26.1				0.3928	0.00071	0.01396				1
ZBED1	zinc finger, BED-type containing 1	Xp22.33;Yp11				-0.432909091	0.002274545	0.024490909				1
ZBED2	zinc finger, BED-type containing 2	3q13.2							-0.768	2.58833	6	1
ZBED4	zinc finger, BED-type containing 4	22q13.33				-0.381	0.000801667	0.0142				1
ZBTB1	zinc finger and BTB domain containing 1	14q23.3				-0.305	0.02452	0.0962				1
AAMP	angio-associated, migratory cell protein	2q35	0.284	0.03206	0.1259							1
ZBTB11	zinc finger and BTB domain containing 11	3q12.3	-0.278	0.02739	0.1136							1
CDK17	cyclin-dependent kinase 17	12q23.1	0.284	0.03807	0.1411							1
ZBTB16	zinc finger and BTB domain containing 16	11q23.1							0.765	2.87714	7	1
ZBTB17	zinc finger and BTB domain containing 17	1p36.13				-0.304	0.0136	0.0681				1
GNB1	guanine nucleotide binding protein (G protein), beta polypeptide 1	1p36.33	0.284	0.01724	0.0839							1
ZBTB20	zinc finger and BTB domain containing 20	3q13.2				0.342333333	0.006221667	0.039383333				1
ZBTB24	zinc finger and BTB domain containing 24	6q21				-0.303	0.00197	0.0244				1
ZBTB40	zinc finger and BTB domain containing 40	1p36	0.284	0.03349	0.1295							1
ZBTB32	zinc finger and BTB domain containing 32	19q13.1							-0.683	2.802	5	1
ZBTB33	zinc finger and BTB domain containing 33	Xq23							-0.674	2.61	5	1
C2orf47	chromosome 2 open reading frame 47	2q33.1	0.283	0.04729	0.1615							1
ZBTB4	zinc finger and BTB domain containing 4	17p13.1	-0.628	0.00115	0.0129	-0.4195	0.00171875	0.0193375				2
DDX50	DEAD (Asp-Glu-Ala-Asp) box polypeptide 50	10q22.1	0.283	0.02389	0.1038							1
ZBTB41	zinc finger and BTB domain containing 41	1q31.3				0.4105	0.000958333	0.01495				1
ZBTB42	In multiple Geneids		-0.352	0.00069	0.0091							1
ZBTB45	zinc finger and BTB domain containing 45	19q13.43				-0.407	0.00475	0.0382				1
ZBTB48	zinc finger and BTB domain containing 48	1p36.3				-0.366666667	0.002473333	0.022133333				1
ZBTB5	zinc finger and BTB domain containing 5	9p13.2	-0.855	9.00E-05	0.0021	-0.362666667	0.00095	0.012866667				2
ZBTB7A	zinc finger and BTB domain containing 7A	19p13.3	-0.562	0.00381	0.0302	-0.306	0.00392	0.0345				2
ZBTB7B	zinc finger and BTB domain containing 7B	1q21.3	-0.472	0.00097	0.0115							1
ZBTB7C	In multiple Geneids		-2.225	0	1.00E-04	-0.379666667	0.01031	0.054566667				2
ZBTB8	zinc finger and BTB domain containing 8	1p35.1							-0.826	2.666	5	1
ZBTB8A	zinc finger and BTB domain containing 8A	1p35.1				-0.35	0.0276	0.1032				1
ZBTB8OS	zinc finger and BTB domain containing 8 opposite strand	1p35.1				-0.336	0.013785	0.0685				1
ZC2HC1A	zinc finger, C2HC-type containing 1A	8q21.12	-0.862	0.02314	0.1017							1
MGAT2	mannosyl (alpha-1,6-)-glycoprotein beta-1,2-N-acetylglucosaminyltransferase	14q21	0.283	0.04452	0.1558							1
ZC3H10	zinc finger CCCH-type containing 10	12q13.2				-0.36	0.0047	0.0379				1
RNF168	ring finger protein 168, E3 ubiquitin protein ligase	3q29	0.283	0.04179	0.1497							1
ZC3H12A	zinc finger CCCH-type containing 12A	1p34.3				-0.307	0.01894	0.0826				1
TRIM10	tripartite motif containing 10	6p21.3	0.283	0.04109	0.1482							1
MYO9B	myosin IXB	19p13.1	0.282	0.03517	0.1337							1
ZC3H12D	zinc finger CCCH-type containing 12D	6q25.1				-0.304	0.00265	0.0282				1
GET4	golgi to ER traffic protein 4 homolog (S. cerevisiae)	7p22.3	0.281	0.03455	0.1321							1
LONP2	lon peptidase 2, peroxisomal	16q12.1	0.281	0.03203	0.1259							1
ZC3H18	zinc finger CCCH-type containing 18	16q24.2				-0.356285714	0.003584286	0.029842857				1
ZC3H4	zinc finger CCCH-type containing 4	19q13.32				-0.446	0.00067	0.0147				1
ZC3H5	Data not found								-0.93	3.00571	7	1
ZC3H6	zinc finger CCCH-type containing 6	2q13							-0.94	3.37571	7	1
SAFB2	scaffold attachment factor B2	19p13.3	0.281	0.03464	0.1324							1
ZC3H7B	zinc finger CCCH-type containing 7B	22q13.2				-0.478857143	0.000822857	0.011857143				1
ZC3HAV1	zinc finger CCCH-type, antiviral 1	7q34				-0.4025	0.00649	0.041783333				1
TCF3	transcription factor 3 (E2A immunoglobulin enhancer binding factors E12/E47	19p13.3	0.281	0.02547	0.1086							1
ZC3HC1	zinc finger, C3HC-type containing 1	7q32.2				-0.347	0.005845	0.04155				1
SAFB	scaffold attachment factor B	19p13.3-p13.2	0.28	0.03284	0.1279							1

ZCCHC12	zinc finger, CCHC domain containing 12	Xq24	-0.314	0.00578	0.0404							1
ZCCHC14	zinc finger, CCHC domain containing 14	16q24.2				-0.373	0.00337	0.028575				1
ZCCHC16	zinc finger, CCHC domain containing 16	Xq23				0.32	0.001195	0.015725				1
ZCCHC17	zinc finger, CCHC domain containing 17	1p35.2				-0.303	0.00084	0.0162				1
ZCCHC2	zinc finger, CCHC domain containing 2	18q21.33							-0.72	2.978	5	1
ZCCHC24	zinc finger, CCHC domain containing 24	10q22.3				-0.333	0.01272	0.052866667				1
ZCCHC4	zinc finger, CCHC domain containing 4	4p15.2	-0.245	0.02363	0.1032	-0.327	0.00038	0.01075				2
ZCCHC5	zinc finger, CCHC domain containing 5	Xq21.1							-0.843	2.896	5	1
ZCCHC6	zinc finger, CCHC domain containing 6	9q21	-0.656	0.00775	0.0491							1
ZCCHC7	zinc finger, CCHC domain containing 7	9p13.2				-0.335285714	0.000951429	0.012157143				1
ZCCHC8	zinc finger, CCHC domain containing 8	12q24.31				-0.326	0.00745	0.047				1
ZCWPW1	zinc finger, CW type with PWWP domain 1	7q22.1							0.593	2.38625	8	1
ZCWPW2	zinc finger, CW type with PWWP domain 2	3p24.1							-1.324	2.874	5	1
COX14	COX14 cytochrome c oxidase assembly homolog (S. cerevisiae)	12q13.12	0.279	0.0492	0.1654							1
ZDHHC13	zinc finger, DHHC-type containing 13	11p15.1	-1.124	1.00E-04	0.0022							1
DAPK3	death-associated protein kinase 3	19p13.3	0.278	0.02273	0.1006				-0.592	2.523	5	2
ZDHHC15	zinc finger, DHHC-type containing 15	Xq13.3	-1.076	1.00E-05	4.00E-04							1
ZDHHC16	zinc finger, DHHC-type containing 16	10q24.1				-0.316	0.000955	0.01515				1
CPT1C	carnitine palmitoyltransferase 1C	19q13.33	0.277	0.03388	0.1303							1
ZDHHC18	zinc finger, DHHC-type containing 18	1p36.11	-0.566	0.00037	0.0058	-0.468833333	0.005338333	0.035783333				2
ZDHHC19	zinc finger, DHHC-type containing 19	3q29				-0.47	7.00E-05	0.0059				1
C16orf52	In multiple Geneids		0.276	0.04741	0.1618							1
ZDHHC21	In multiple Geneids		-1.503	0	0	-0.354666667	0.001563333	0.019833333				2
ZDHHC22	zinc finger, DHHC-type containing 22	14q24.3	-0.345	0.00483	0.0357							1
PHYHIPL	phytanoyl-CoA 2-hydroxylase interacting protein-like	10q11	0.276	0.04116	0.1484							1
ZDHHC3	zinc finger, DHHC-type containing 3	3p21.31	-0.604	0.00083	0.0103	-0.33525	0.0016825	0.020125				2
C9orf78	chromosome 9 open reading frame 78	9q34.11	0.275	0.04697	0.1609							1
ZDHHC5	zinc finger, DHHC-type containing 5	11q12.1	-0.668	0.00025	0.0044							1
SMG7	smg-7 homolog, nonsense mediated mRNA decay factor (C. elegans)	1q25	0.273	0.04821	0.1635							1
ZDHHC8	zinc finger, DHHC-type containing 8	22q11.21	-0.237	0.02952	0.1193							1
ADAM22	ADAM metallopeptidase domain 22	7q21	0.272	0.03941	0.1441							1
CCL3L1	chemokine (C-C motif) ligand 3-like 1	17q21.1	0.272	0.0375	0.1397							1
ZEB2	zinc finger E-box binding homeobox 2	2q22.3				0.352	0.0038125	0.031875				1
ZER1	zer-1 homolog (C. elegans)	9q34.11				-0.38	0.011626667	0.062233333				1
OSTM1	osteopetrosis associated transmembrane protein 1	6q21	0.272	0.03144	0.1244							1
C6orf123	In multiple Geneids		0.271	0.03975	0.145							1
CRLF2	cytokine receptor-like factor 2	Xp22.3; Yp11.3	0.271	0.03773	0.1402							1
FES	feline sarcoma oncogene	15q26.1	0.271	0.02549	0.1086							1
ZFAT	zinc finger and AT hook domain containing	8q24.22				0.38248	0.0215696	0.086652				1
ZFATAS	Data not found					0.377	0.02244	0.0913				1
ID2B	inhibitor of DNA binding 2B, dominant negative helix-loop-helix protein (pseu	3p14.2	0.271	0.0191	0.0901							1
ZFHX4	zinc finger homeobox 4	8q21.11				0.386807692	0.001218846	0.016515385				1
ZFP106	zinc finger protein 106 homolog (mouse)	15q15.1				-0.361666667	0.007213333	0.0384				1
TWISTNB	TWIST neighbor	7p21.1	0.271	0.00467	0.0348				-1.043	3.2	6	2
ZFP14	zinc finger protein 14 homolog (mouse)	19q13.12				0.341	0.01002	0.0573				1
ZFP161	zinc finger protein 161 homolog (mouse)	18pter-p11.21							-0.828	3.17	6	1
ZFP2	zinc finger protein 2 homolog (mouse)	5q35.3				-0.408	0.00085	0.0164				1
ZFP28	zinc finger protein 28 homolog (mouse)		-0.794	4.00E-05	0.0012	-0.482	0.00076	0.0155				2
ZFP3	In multiple Geneids		-0.615	0.04214	0.1504	-0.326	0.00668	0.038966667				2
ZFP36	zinc finger protein 36, C3H type, homolog (mouse)	19q13.1	-0.906	0.00185	0.018							1
NINJ1	ninjurin 1	9q22	0.27	0.02308	0.1015							1
ZFP41	zinc finger protein 41 homolog (mouse)	8q24.3				-0.322	0.00364	0.0332				1
ZFP42	zinc finger protein 42 homolog (mouse)	4q35.2				-0.515	0.000975	0.01745				1
RBM41	RNA binding motif protein 41	Xq22.3	0.27	0.0495	0.1661							1
ZFP82	zinc finger protein 82 homolog (mouse)	19q13.12				-0.337	0.00045	0.0124				1
ZFP90	zinc finger protein 90 homolog (mouse)	16q22.1				-0.378666667	0.0061	0.0372				1
ZFP91	zinc finger protein 91 homolog (mouse)	11q12				0.311	0.00066	0.0146				1
ZFP91-CNTF	ZFP91-CNTF readthrough	11q12.2				0.311	0.00066	0.0146				1
ZFPM1	zinc finger protein, multitype 1	16q24.2				-0.385666667	0.011536667	0.046666667				1
ZFPM2	zinc finger protein, multitype 2	8q23				0.361230769	0.002224615	0.0183				1
YIF1A	Yip1 interacting factor homolog A (S. cerevisiae)	11q13	0.269	0.02983	0.1202							1
ZFR2	zinc finger RNA binding protein 2	19p13.3				-0.447333333	0.007513333	0.0372				1
ZFY	zinc finger protein, Y-linked	Yp11.3	-0.64	0.00175	0.0174	-0.428	0.02066	0.0869				2
ZFYVE1	zinc finger, FYVE domain containing 1	14q24.2	-0.742	2.00E-05	7.00E-04	-0.331454545	0.007633636	0.045436364				2
ZFYVE16	zinc finger, FYVE domain containing 16	5q14				-0.362666667	0.00119	0.0158				1
ZFYVE19	zinc finger, FYVE domain containing 19	15q15.1	-0.343	0.01521	0.0773							1
ZFYVE21	zinc finger, FYVE domain containing 21	14q32.33	-0.426	0.00279	0.0242	-0.331	0.001266667	0.018633333				2
ZFYVE26	zinc finger, FYVE domain containing 26	14q24.1				-0.37375	0.01002	0.038425				1
ZNHIT1	zinc finger, HIT-type containing 1	7q22.1	0.269	0.02161	0.0977							1

ZFYVE28	zinc finger, FYVE domain containing 28	4p16.3				-0.441363636	0.003871818	0.0301				1
ZFYVE9	zinc finger, FYVE domain containing 9	1p32.3				-0.451333333	0.012423333	0.047333333				1
MAGOHB	mago-nashi homolog B (Drosophila)	12p13.2	0.268	0.03784	0.1405							1
ZHX1	zinc fingers and homeoboxes 1	8q24.13	-1.363	1.00E-05	3.00E-04							1
ZHX2	zinc fingers and homeoboxes 2	8q24.13				0.325	0.00214	0.0254				1
ZHX3	zinc fingers and homeoboxes 3	20q12	-0.415	0.00235	0.0215							1
ZIC4	Zic family member 4	3q24	-0.238	0.01463	0.0752							1
ZIM2	zinc finger, imprinted 2	19q13.4				-0.34	3.00E-04	0.0104	0.561	2.784	5	2
ZIM3	zinc finger, imprinted 3		-0.371	0.00932	0.0557	-0.383	0.00071	0.0151				2
MPLKIP	M-phase specific PLK1 interacting protein	7p14.1	0.268	0.04505	0.157							1
ZMAT1	zinc finger, matrin-type 1	Xq21							-0.774	2.93333	6	1
ZMAT4	zinc finger, matrin-type 4	8p11.21				0.428	0.0081	0.051				1
ZMAT5	zinc finger, matrin-type 5	22cen-q12.3	-0.384	0.01784	0.086	-0.4182	0.00299	0.02578				2
ZMIZ1	zinc finger, MIZ-type containing 1	10q22.3	-0.831	1.00E-05	5.00E-04	-0.346833333	0.005255	0.0365				2
ZMIZ2	zinc finger, MIZ-type containing 2	7p13				-0.443	0.000655	0.0144				1
ZMYM4	zinc finger, MYM-type 4	1p32-p34				-0.36	0.00116	0.0189				1
ZMYM6	zinc finger, MYM-type 6	1p34.2							-0.691	2.488	5	1
ZMYND15	zinc finger, MYND-type containing 15	17p13.2				-0.4525	0.00183	0.0235				1
ZMYND17	Data not found								-0.811	2.9225	8	1
ZNF100	In multiple Geneids								-0.923	2.89833	6	1
ZNF101	In multiple Geneids		-0.717	0.00033	0.0053							1
RAB3GAP2	RAB3 GTPase activating protein subunit 2 (non-catalytic)	1q41	0.268	0.04362	0.1537	0.3145	0.00092	0.01605	-0.831	2.79333	6	3
ZNF114	In multiple Geneids					-0.506	0.00269	0.0284				1
ZNF117	In multiple Geneids								-0.533	2.74	5	1
ZNF121	zinc finger protein 121	19p				-0.352	0.00156	0.0217				1
THUMPD2	THUMP domain containing 2	2p22.1	0.268	0.03253	0.127	0.34675	0.0020375	0.02085				2
DHX33	DEAH (Asp-Glu-Ala-His) box polypeptide 33	17p13.2	0.267	0.03921	0.1436							1
ZNF132	zinc finger protein 132	19q13.4	-0.543	7.00E-05	0.0018							1
ZNF135	zinc finger protein 135	19q13.4	-0.643	1.00E-05	5.00E-04							1
ZNF136	zinc finger protein 136	19p13.2				-0.3635	0.0089025	0.05185				1
RUNDC1	RUN domain containing 1	17q21.31	0.267	0.03326	0.1289							1
ZNF14	zinc finger protein 14	19p13.11				-0.376333333	0.00384	0.0305				1
ZNF143	zinc finger protein 143	11p15.4				-0.3482	0.003468	0.0273				1
C17orf62	chromosome 17 open reading frame 62	17q25.3	0.266	0.02199	0.0988							1
ZNF157	zinc finger protein 157	Xp11.2	-0.295	0.00264	0.0233							1
ZNF160	In multiple Geneids					-0.405	0.00028	0.01				1
DNAJC25	DnaJ (Hsp40) homolog, subfamily C , member 25	9q31.3	0.266	0.03277	0.1277							1
ZNF17	zinc finger protein 17	19q13.4							-0.889	2.76143	7	1
ZNF174	zinc finger protein 174	16p13.3				-0.371666667	0.027393333	0.1024				1
ZNF177	zinc finger protein 177	19p13.2				-0.376	0.03476	0.1188				1
ZNF18	zinc finger protein 18	17p11.2	-0.416	0.00076	0.0097	-0.356	0.002706667	0.025833333				2
ZNF180	zinc finger protein 180	19q13.2				-0.333	0.00063	0.0143				1
FAM199X	family with sequence similarity 199, X-linked	Xq22.2	0.266	0.01833	0.0876							1
ZNF185	zinc finger protein 185 (LIM domain)	Xq28	-3.251	0	1.00E-04							1
LAMTOR5	Data not found		0.266	0.04154	0.1492							1
METTL16	methyltransferase like 16	17p13.3	0.266	0.04	0.1456							1
ZNF197	zinc finger protein 197	3p21				-0.36	4.00E-05	0.0046				1
ZNF20	zinc finger protein 20	19p13.2				-0.396	0.00056	0.0135				1
ZNF200	zinc finger protein 200	16p13.3				-0.448	0.017375	0.071				1
ZNF205	zinc finger protein 205	16p13.3				-0.485666667	0.011216667	0.050733333				1
ZNF212	zinc finger protein 212	7q36.1	-0.422	0.00074	0.0094	-0.3335	0.009785	0.0565				2
ZNF213	zinc finger protein 213	16p13.3	-0.287	0.01244	0.0678	-0.505	0.003665	0.0331				2
SLC25A22	solute carrier family 25 (mitochondrial carrier: glutamate), member 22	11p15.5	0.266	0.01789	0.0861							1
ZNF219	zinc finger protein 219	14q11	-0.547	0	1.00E-04							1
CARD10	caspase recruitment domain family, member 10	22q13.1	0.265	0.03417	0.1312							1
ZNF263	zinc finger protein 263	16p13.3	0.264	0.02722	0.1132							1
UAP1L1	UDP-N-acteylglucosamine pyrophosphorylase 1-like 1	9q34.3	0.263	0.0398	0.1451							1
KDM5A	lysine (K)-specific demethylase 5A	12p11	0.262	0.03084	0.123							1
LOC494150	prohibitin pseudogene	12q13	0.262	0.03577	0.1353							1
MORF4L1	mortality factor 4 like 1	15q24	0.262	0.03508	0.1335							1
ZNF228	Data not found								-0.78	2.908	5	1
POLR2J3	polymerase (RNA) II (DNA directed) polypeptide J3	7q22.1	0.262	0.02616	0.1105							1
ZSCAN20	zinc finger and SCAN domain containing 20	1p34.3	0.262	0.01604	0.0801							1
FCHO1	FCH domain only 1	19p13.11	0.261	0.03109	0.1236							1
INTS4L2	integrator complex subunit 4-like 2	7q11.21	0.261	0.04728	0.1615							1
ZNF236	zinc finger protein 236	18q22-q23				-0.365727273	0.003636364	0.029627273				1
ZNF238	zinc finger protein 238	1q44-qter				0.314	0.000375	0.01065	-0.671	2.841665	5.5	2
MARCKSL1	MARCKS-like 1	1p35.1	0.261	0.01574	0.0791							1
ZNF254	zinc finger protein 254	19p12	-0.689	0.0222	0.0993							1

AKAP8L	A kinase (PRKA) anchor protein 8-like	19p13.12	0.26	0.03115	0.1238							1
REV3L	REV3-like, polymerase (DNA directed), zeta, catalytic subunit	6q21	0.26	0.04972	0.1665							1
ZNF260	zinc finger protein 260	19q13.12				-0.314	0.01705	0.0776				1
TMEM231	transmembrane protein 231	16q23.1	0.26	0.01453	0.0749							1
ZNF264	zinc finger protein 264	19q13.4				-0.304	0.00032	0.0106				1
ZNF266	zinc finger protein 266	19p13.2				-0.55625	0.014765	0.067075				1
CDC42EP4	CDC42 effector protein (Rho GTPase binding) 4	17q24-q25	0.259	0.03492	0.1331							1
ZNF273	zinc finger protein 273	7q11.21	-0.531	0.01737	0.0843							1
HTRA4	HtrA serine peptidase 4	8p11.22	0.259	0.04552	0.1581							1
ZNF276	zinc finger protein 276	16q24.3	-0.327	0.04357	0.1536	-0.483	0.0033	0.0315				2
KHDRBS1	KH domain containing, RNA binding, signal transduction associated 1	1p32	0.259	0.01174	0.0652							1
ZNF280A	zinc finger protein 280A	22q11.22				-0.373	0.00033	0.01065				1
NCOR2	nuclear receptor corepressor 2	12q24	0.259	0.03872	0.1426							1
RAB5C	RAB5C, member RAS oncogene family	17q21.2	0.259	0.04937	0.1658							1
ZNF282	zinc finger protein 282	7q36.1				-0.357666667	0.01051	0.053933333				1
ZNF286B	In multiple Geneids					-0.306	0.00282	0.0291				1
ZNF287	zinc finger protein 287	17p11.2	-0.515	0.0094	0.056							1
ZNF294	Data not found								-0.735	2.624	5	1
ZNF295	zinc finger protein 295	21q22.3				-0.374	0.001915	0.024				1
ZNF295-AS1	ZNF295 antisense RNA 1 (non-protein coding)	21q22.3	-0.199	0.03378	0.1301							1
ZNF296	In multiple Geneids		-0.815	0	2.00E-04	-0.382	0.00139	0.0205				2
ZNF384	zinc finger protein 384	12p12	0.259	0.00997	0.0583							1
ZNF300P1	zinc finger protein 300 pseudogene 1	5q33.1	-0.604	0.00466	0.0348							1
ZNF311	zinc finger protein 311	6p22.1	-0.361	0.00376	0.03							1
ZNF317	In multiple Geneids					-0.452666667	0.00215	0.024833333				1
CCDC181	Data not found		0.258	0.03421	0.1312							1
ZNF319	zinc finger protein 319	16q21				-0.308	0.00067	0.0146				1
ING1	inhibitor of growth family, member 1	13q34	0.258	0.01787	0.086							1
RALY	RNA binding protein, autoantigenic (hnRNP-associated with lethal yellow hom	20q11.21-q11.23	0.258	0.01856	0.0883							1
ZNF324	In multiple Geneids		-0.289	0.00636	0.043							1
ZNF329	In multiple Geneids					-0.307	0.00629	0.0444				1
ZNF330	zinc finger protein 330	4q31.21							-0.616	2.525	6	1
ZNF331	zinc finger protein 331	19q13.42				-0.331	0.00234	0.0265	0.822	2.90143	7	2
ZNF333	zinc finger protein 333	19p13				-0.3748	0.003396	0.02568				1
NCKIPSD	NCK interacting protein with SH3 domain	3p21	0.256	0.00694	0.0456							1
ZNF341	zinc finger protein 341	20q11.22				-0.352	6.00E-05	0.0056				1
ZNF346	zinc finger protein 346	5q35.2	-0.234	0.04825	0.1636	-0.381	0.004676	0.03474				2
ZNF350	zinc finger protein 350	19q13.41	-0.479	0.03981	0.1451							1
ZNF354A	zinc finger protein 354A	5q35.3				-0.396	0.00206	0.0218				1
ZNF354B	zinc finger protein 354B	5q35.3				-0.4335	0.00167	0.02165				1
ZNF354C	zinc finger protein 354C	5q35	-0.29	0.00487	0.0359	-0.535333333	0.000983333	0.014066667				2
ZNF358	zinc finger protein 358	19p13.3				-0.559	0.00165	0.0223	0.745	2.72	8	2
ZNF362	In multiple Geneids					-0.34	0.004535	0.03675				1
ZNF365	zinc finger protein 365	10q21.2							-0.704	2.76571	7	1
ZNF366	In multiple Geneids								-0.789	2.628	5	1
SCYL1	SCY1-like 1 (S. cerevisiae)	11q13	0.256	0.01809	0.0868							1
SLC2A6	solute carrier family 2 (facilitated glucose transporter), member 6	9q34	0.256	0.03362	0.1297							1
CCNT2	cyclin T2	2q21.3	0.255	0.01733	0.0842							1
ZNF385A	zinc finger protein 385A	12q13.13	-1.234	0.00033	0.0052							1
ZNF385B	zinc finger protein 385B	2q31.2-q31.3				0.335533333	0.000860667	0.012033333				1
ZNF394	In multiple Geneids					-0.331	0.02271	0.092				1
ZNF395	zinc finger protein 395	8p21.1	-0.78	0	1.00E-04	-0.345	0.001005	0.0157				2
ZNF398	zinc finger protein 398	7q36.1	-0.644	0.00053	0.0075	-0.316	0.0168	0.077				2
ZNF404	In multiple Geneids								-1.042	3.01667	6	1
ZNF407	zinc finger protein 407	18q23				-0.3035	0.0077	0.04945				1
ZNF410	zinc finger protein 410	14q24.3	-0.353	0.01953	0.0913	-0.351	0.00472	0.038				2
ZNF414	In multiple Geneids		-0.298	0.00198	0.0189							1
ZNF415	zinc finger protein 415	19q13.42				-0.568	3.00E-05	0.0045				1
ZNF417	In multiple Geneids					-0.333	0.00759	0.0492				1
ZNF42	Data not found								-0.883	3.012	5	1
ZNF423	zinc finger protein 423	16q12				-0.341666667	0.005832	0.037286667				1
ZNF425	In multiple Geneids		-0.759	0	1.00E-04	-0.329	0.018915	0.07835				2
ZNF426	zinc finger protein 426	19p13.2	-2.14	0	0	-0.324	0.00132	0.02				2
ZNF428	zinc finger protein 428	19q13.31				-0.453	0.00037	0.0113				1
ZNF429	zinc finger protein 429	19p13.1				-0.369	0.00012	0.007				1
ZNF43	zinc finger protein 43	19p13.1-p12				-0.375	0.00095	0.0172				1
ZNF430	zinc finger protein 430	19p12	-1.036	0.00026	0.0044							1
ZNF431	zinc finger protein 431	19p12	-1.738	5.00E-05	0.0013							1
E2F4	E2F transcription factor 4, p107/p130-binding	16q21-q22	0.255	0.01304	0.0698							1

ZNF433	zinc finger protein 433	19p13.2	-0.713	4.00E-05	0.0012	-0.343	0.003583333	0.032333333				2
ZNF434	zinc finger protein 434	16p13.3				-0.46825	0.01298	0.060275				1
ZNF435	Data not found								-0.807	2.862	5	1
ZNF436	zinc finger protein 436	1p36	-0.951	6.00E-05	0.0014	-0.43	0.00055	0.0135				2
ZNF438	zinc finger protein 438	10p11.23							-1.016	3.10429	7	1
ZNF44	In multiple Geneids					-0.342666667	0.002523333	0.0273				1
ZNF440	zinc finger protein 440	19p13.2				-0.572	0.00017	0.0081				1
ZNF441	zinc finger protein 441	19p13.2				-0.4	0.00067	0.0147				1
ZNF442	zinc finger protein 442	19p13.2	-0.368	0.01937	0.0909							1
ZNF443	zinc finger protein 443	19p13.2				-0.359	0.00723	0.0479				1
ZNF445	zinc finger protein 445	3p21.32				-0.308	0.00135	0.0203				1
ZNF447	zinc finger protein 447								0.923	2.50833	6	1
ZNF449	In multiple Geneids					0.346333333	0.003033333	0.029366667				1
ZNF452	Data not found								0.851	3.64667	6	1
ZNF462	zinc finger protein 462	9q31.2				0.339666667	0.00078	0.0156				1
ZNF467	zinc finger protein 467	7q36.1				-0.334	0.00075	0.0154				1
FAM195A	family with sequence similarity 195, member A	16p13.3	0.255	0.04305	0.1526							1
ZNF469	zinc finger protein 469	16q24				-0.351	0.00219	0.0256				1
ZNF470	zinc finger protein 470	19q13.43	-0.51	0.01536	0.0778							1
ZNF471	zinc finger protein 471	19q13.43	-0.724	3.00E-05	9.00E-04				0.722	2.65167	6	2
LINC00597	long intergenic non-protein coding RNA 597	15q23-q24	0.255	0.03178	0.1252							1
ZNF479	zinc finger protein 479	7p11.2	-0.257	0.04265	0.1517							1
ZNF48	In multiple Geneids					-0.342	0.00142	0.0207				1
TMEM44	transmembrane protein 44	3q29	0.253	0.02497	0.107							1
CNRIP1	cannabinoid receptor interacting protein 1	2p14	0.252	0.0471	0.1611							1
ZNF490	zinc finger protein 490	19p13.2				-0.406	0.0087775	0.04965				1
FEZ2	fasciculation and elongation protein zeta 2 (zygin II)	2p21	0.252	0.04718	0.1613	0.357	0.00113	0.0186				2
ZNF497	zinc finger protein 497	19q13.43	-0.257	0.02018	0.0932							1
ZNF500	zinc finger protein 500	16p13.3				-0.502333333	0.00362	0.0316				1
ZNF501	zinc finger protein 501	3p21.31				-0.396	1.00E-05	0.0029				1
ZNF503	zinc finger protein 503	10q22.2	-0.454	0.020705	0.0879				0.72	3.15571	7	2
KCNC4	potassium voltage-gated channel, Shaw-related subfamily, member 4	1p21	0.252	0.03264	0.1273							1
ZNF509	Data not found					-0.345	0.00042	0.0119				1
RPL23A	ribosomal protein L23a	17q11.2	0.252	0.032905	0.1265							1
ZNF512	zinc finger protein 512	2p23							0.644	2.59333	6	1
ZNF512B	zinc finger protein 512B	20q13.33	-0.592	0.00646	0.0434							1
ZNF513	zinc finger protein 513	2p23.3				-0.304	0.007655	0.04675				1
ZNF516	zinc finger protein 516	18q23	-0.252	0.04469	0.1561	-0.367	0.002143333	0.020211111				2
RAD52	RAD52 homolog (S. cerevisiae)	12p13-p12.2	0.251	0.0464	0.1597							1
ZNF518B	zinc finger protein 518B	4p16.1				-0.352	0.00061	0.0141				1
ZNF524	zinc finger protein 524	19q13.42	-0.286	0.00328	0.0273				0.635	2.502	5	2
UTP18	UTP18 small subunit (SSU) processome component homolog (yeast)	17q21.33	0.251	0.03749	0.1397							1
SCG2	secretogranin II	2q35-q36	0.25	0.02585	0.1097				-0.889	2.44778	9	2
ZNF528	zinc finger protein 528	19q13				-0.358	0.01168	0.0624				1
ZNF529	zinc finger protein 529	19q13.13				1.217	0	0.0018				1
ZNF532	zinc finger protein 532	18q21.32				-0.402071429	0.003452857	0.029678571				1
ZNF536	zinc finger protein 536	19q12				0.353	0.00137	0.0204				1
ZNF540	zinc finger protein 540	19q13.12							0.752	2.54917	6	1
ZNF541	zinc finger protein 541	19q13.33				-0.38675	0.0050825	0.035825				1
ZNF542	zinc finger protein 542	19q13.43	-0.673	0.00369	0.0296							1
ZNF543	zinc finger protein 543	19q13.43				-0.302	0.00064	0.0144				1
COPS7B	COP9 constitutive photomorphogenic homolog subunit 7B (Arabidopsis)	2q37.1	0.249	0.04869	0.1644							1
PYCRL	pyrroline-5-carboxylate reductase-like	8q24.3	0.249	0.01885	0.0893							1
ZNF554	zinc finger protein 554	19p13.3	-0.234	0.03317	0.1286	-0.405333333	0.00297	0.0262				2
ZNF555	zinc finger protein 555	19p13.3	-0.57	0.02952	0.1193	-0.40725	0.0018675	0.021125				2
ZNF557	zinc finger protein 557	19p13.2				-0.49925	0.001445	0.0178				1
ZNF558	zinc finger protein 558	19p13.2				-0.361	0.00338	0.0319				1
ZNF559	zinc finger protein 559	19p13.2				-0.3905	0.00056	0.0126				1
ZNF560	zinc finger protein 560	19p13.2				-0.505571429	0.016608571	0.073957143				1
ZNF561	zinc finger protein 561	19p13.2	-0.842	0.00019	0.0035	-0.358	0.0015	0.0213				2
ZNF562	zinc finger protein 562	19p13.2	-0.575	0.0217	0.0979	-0.342	0.011456667	0.060433333				2
ZNF563	zinc finger protein 563	19p13.2				-0.381666667	0.025633333	0.087166667				1
ZNF564	zinc finger protein 564	19p13.2				-0.376	0.00909	0.0542				1
ZNF565	zinc finger protein 565	19q13.12				-0.376333333	0.00473	0.0373				1
ZNF568	zinc finger protein 568	19q13.12							1.356	3.01	5	1
ZNF569	zinc finger protein 569	19q13.12							0.963	3.045	7	1
ZNF57	In multiple Geneids		-0.868	7.00E-04	0.0092	-0.430833333	0.004993333	0.03455				2
ZNF570	zinc finger protein 570	19q13.12	-0.752	0.02109	0.0961				0.963	3.045	7	2
ZNF574	zinc finger protein 574	19q13.2	-0.228	0.03381	0.1301				0.547	2.38833	6	2

ZNF575	zinc finger protein 575	19q13.31				-0.396	0.00331	0.0316				1
ZNF577	zinc finger protein 577	19q13.41				-0.43	0.0018	0.0233	0.896	2.51	6	2
STK16	serine/threonine kinase 16	2q35	0.249	0.03608	0.1363							1
ZNF582	zinc finger protein 582	19q13.43	-0.281	0.02778	0.1146							1
ZNF584	zinc finger protein 584	19q13.43	-0.406	0.00385	0.0305							1
TAF3	TAF3 RNA polymerase II, TATA box binding protein (TBP)-associated factor, 14	10p15.1	0.249	0.01896	0.0896							1
ZNF589	zinc finger protein 589	3p21	-0.299	0.04606	0.1591	-0.382	0.0017	0.01755				2
KRT18P55	keratin 18 pseudogene 55	17q11.2	0.248	0.04284	0.1521							1
DIDO1	death inducer-obliterator 1	20q13.33	0.247	0.02671	0.112	0.387	0.00523	0.03895				2
ZNF595	zinc finger protein 595	4p16.3				-0.379	0.0045	0.0371				1
ZNF596	zinc finger protein 596	8p23.3							0.955	3.046	5	1
ZNF597	zinc finger protein 597	16p13.3				-0.339	0.02651	0.1008	0.894	3.64	7	2
LOC100129726	uncharacterized LOC100129726	2p21	0.247	0.04234	0.151							1
ZNF605	zinc finger protein 605	12q24.33				-0.5135	0.000155	0.00665				1
ZNF608	zinc finger protein 608	5q23.2				-0.325	0.00012	0.0072				1
SEMA6B	sema domain, transmembrane domain (TM), and cytoplasmic domain, (semap	19p13.3	0.247	0.03777	0.1403							1
RDH14	retinol dehydrogenase 14 (all-trans/9-cis/11-cis)	2p24.2	0.246	0.03063	0.1224							1
SIAH1	siah E3 ubiquitin protein ligase 1	16q12	0.246	0.01509	0.0769							1
ADAMTSL2	ADAMTS-like 2	9q34.2	0.245	0.03451	0.132							1
ZNF619	zinc finger protein 619	3p22.1				-0.347	0.00051	0.011725				1
ZNF785	zinc finger protein 785	16p11.2	0.245	0.02037	0.0939							1
ZNF621	zinc finger protein 621	3p22.1				-0.321	4.00E-04	0.0117				1
CAPN10	calpain 10	2q37.3	0.244	0.00878	0.0534							1
ZNF624	zinc finger protein 624	17p11.2							-0.876	3.4	7	1
ZNF625	zinc finger protein 625	19p13.2				-0.3115	0.017205	0.07445				1
ZNF626	zinc finger protein 626	19p12	-0.757	0.00872	0.0532							1
ZNF627	zinc finger protein 627	19p13.2				-0.397	0.01438	0.0704				1
GFER	growth factor, augmenter of liver regeneration	16p13.3-p13.12	0.244	0.01225	0.0672							1
BRD3	bromodomain containing 3	9q34	0.243	0.03259	0.1272							1
ZNF650	Data not found								-0.878	2.855	8	1
ELFN2	extracellular leucine-rich repeat and fibronectin type III domain containing 2	22q13.1	0.24	0.02659	0.1117							1
ZNF653	zinc finger protein 653	19p13.2				-0.4468	0.003064	0.02696				1
ZNF654	zinc finger protein 654	3p11.1	-0.494	0.01039	0.0602							1
ZNF655	zinc finger protein 655	7q22.1	-1.276	0.00397	0.0311							1
ZNF658	zinc finger protein 658	9p13.1							-1.0015	3.053875	6.5	1
ZNF658B	zinc finger protein 658B, pseudogene	9p12							-0.938	2.924	5	1
ZNF662	zinc finger protein 662	3p22.1	-0.48	4.00E-04	0.0061							1
GPKOW	G patch domain and KOW motifs	Xp11.23	0.239	0.01313	0.07							1
PUM1	pumilio homolog 1 (Drosophila)	1p35.2	0.239	0.0384	0.1419							1
ZNF671	zinc finger protein 671	19q13.43				-0.301	0.00096	0.0173				1
ZNF672	zinc finger protein 672	1q44	-0.327	0.0039	0.0307							1
CCDC86	coiled-coil domain containing 86	11q12.2	0.238	0.04336	0.1532							1
ZNF675	zinc finger protein 675	19p12	-0.846	0.031	0.1234	-0.312	0.00056	0.0136				2
ZNF677	zinc finger protein 677	19q13.42	-0.552	0.002	0.0191							1
PSMC3	proteasome (prosome, macropain) 26S subunit, ATPase, 3	11p11.2	0.238	0.02202	0.0988							1
ZNF681	zinc finger protein 681	19p12	-0.991	0.00164	0.0166							1
ZNF682	zinc finger protein 682	19p12							-0.704	2.54	6	1
ZNF683	zinc finger protein 683	1p36.11	-0.273	0.01095	0.0621	-0.454	0.00486	0.036133333				2
ZNF70	zinc finger protein 70	22q11.23				-0.384	0.00013	0.0073				1
C1orf109	chromosome 1 open reading frame 109	1p34.3	0.236	0.04574	0.1585							1
PFN1P2	profilin 1 pseudogene 2	1q21	0.235	0.04154	0.1492							1
ZC2HC1C	zinc finger, C2HC-type containing 1C	14q24.3	0.235	0.02194	0.0987							1
ADAMTS3	ADAM metallopeptidase with thrombospondin type 1 motif, 3	4q13.3	0.233	0.02341	0.1026							1
ZNF708	zinc finger protein 708	19p12				-0.375	1.00E-05	0.0033				1
ZNF709	zinc finger protein 709	19p13.2				-0.425	0.010005	0.0572				1
ZNF71	In multiple Geneids		-0.292	0.0108	0.0616							1
ZNF710	zinc finger protein 710	15q26.1	-0.26	0.02423	0.1048							1
DHX34	DEAH (Asp-Glu-Ala-His) box polypeptide 34	19q13.3	0.233	0.02706	0.1128							1
ZNF714	zinc finger protein 714	19p12	-0.607	0.03162	0.1248							1
ZNF718	zinc finger protein 718	4p16.3				-0.379	0.0045	0.0371				1
ZNF721	zinc finger protein 721	4p16.3				-0.4055	0.001555	0.01855				1
ZNF726	In multiple Geneids		-0.491	0.02288	0.101							1
ZNF732	In multiple Geneids		-0.244	0.01757	0.0851							1
ZNF74	zinc finger protein 74	22q11.21				-0.443	0.00017	0.00785				1
ZNF746	zinc finger protein 746	7q36.1				-0.438	1.00E-04	0.0066				1
ZNF750	zinc finger protein 750	17q25.3	-4.309	0	2.00E-04							1
ZNF75A	zinc finger protein 75a	16p13.3				-0.412	0.00712	0.0419				1
ZNF75D	zinc finger protein 75D	Xq26.3				0.355	0.0018	0.01895				1
IMPAD1	inositol monophosphatase domain containing 1	8q12.1	0.233	0.02779	0.1146	0.306	1.00E-05	0.0032	-0.97	2.826	5	3

ZNF763	zinc finger protein 763	19p13.2				-0.39	0.00128	0.0198				1
SDHAF2	succinate dehydrogenase complex assembly factor 2	11q12.2	0.231	0.0472	0.1614							1
SRSF1	serine/arginine-rich splicing factor 1	17q22	0.231	0.03666	0.1377							1
ZNF767	zinc finger family member 767	7q36.1				-0.32	0.03323	0.1156				1
SHROOM1	shroom family member 1	5q31.1	0.23	0.02618	0.1105							1
ZNF77	In multiple Geneids					-0.423666667	0.001433333	0.018433333				1
ZNF770	zinc finger protein 770	15q14	-0.622	0.001265	0.01385							1
TSEN54	tRNA splicing endonuclease 54 homolog (S. cerevisiae)	17q25.1	0.23	0.04195	0.15							1
ZNF778	zinc finger protein 778	16q24.3				-0.371	0.00117	0.019				1
ZNF780A	zinc finger protein 780A	19q13.2				-0.329	0.00143	0.0208				1
GNB5	guanine nucleotide binding protein (G protein), beta 5	15q21.2	0.229	0.0495	0.1661							1
MED22	mediator complex subunit 22	9q34.2	0.227	0.03589	0.1356							1
ZNF783	zinc finger family member 783	7q36.1				-0.356	0.018595	0.07885				1
ZNF784	zinc finger protein 784	19q13.42	-0.246	0.02674	0.112							1
ZNF526	zinc finger protein 526	19q13.2	0.226	0.04954	0.1662							1
ZNF786	zinc finger protein 786	7q36.1				-0.3515	0.025715	0.0974				1
ZNF787	zinc finger protein 787	19q13.43				-0.468	0.00488	0.0387				1
ZNF788	zinc finger family member 788	19p13.2				-0.425	0.003455	0.0301				1
ZNF790	zinc finger protein 790	19q13.12	-0.655	0.00541	0.0386	-0.305	0.00291	0.0295				2
ZNF791	zinc finger protein 791	19p13.2				-0.312	0.0312	0.1112				1
CTSL3P	Data not found		0.224	0.01297	0.0697							1
ZNF793	zinc finger protein 793	19q13.12	-0.347	0.04627	0.1595							1
HNRNPA1P10	heterogeneous nuclear ribonucleoprotein A1 pseudogene 10	19p13.2	0.222	0.03859	0.1424							1
ZNF8	zinc finger protein 8	19q13.43				-0.338	0.0024	0.0269				1
ZNF800	zinc finger protein 800	7q31.33	-0.344	0.03532	0.1342							1
ZNF804A	zinc finger protein 804A	2q32.1				0.345416667	0.000341667	0.008325				1
ZNF804B	zinc finger protein 804B	7q21.13				0.384	0.002926667	0.025328571				1
PFN1	profilin 1	17p13.3	0.221	0.02692	0.1124							1
TRIM3	tripartite motif containing 3	11p15.5	0.221	0.0219	0.0986							1
ZNF815	Data not found					-0.317	0.00156	0.0217				1
CRTC1	CREB regulated transcription coactivator 1	19p13.11	0.22	0.03274	0.1276							1
ZNF821	zinc finger protein 821	16q22.2				-0.3746	0.00568	0.03952				1
ZNF823	zinc finger protein 823	19p13.2	-0.713	0.00235	0.0215	-0.312	0.00749	0.0488				2
PTMA	prothymosin, alpha	2q37.1	0.218	0.0292	0.1184							1
ZNF828	Data not found					0.3745	0.016475	0.0758				1
ZNF831	zinc finger protein 831	20q13.32				0.3539	0.006642	0.03677				1
ZNF836	zinc finger protein 836	19q13.41				-0.358	0.00035	0.0111				1
ZNF839	zinc finger protein 839	14q32.31				-0.355	2.00E-04	0.0087				1
FIBP	fibroblast growth factor (acidic) intracellular binding protein	11q13.1	0.216	0.032	0.1258							1
RTN2	reticulon 2	19q13.32	0.214	0.04132	0.1487							1
ZNF843	zinc finger protein 843	16p11.2	-0.195	0.02587	0.1097							1
ZNF844	zinc finger protein 844	19p13.2				-0.32	0.02117	0.0882				1
ZNF846	zinc finger protein 846	19p13.2	-0.607	0.00786	0.0496	-0.31	0.00724	0.04755				2
ZNF860	In multiple Geneids		-0.386	0.0479	0.1629							1
ZNF862	zinc finger protein 862	7q36.1	-0.501	0.00091	0.011							1
ZNF879	zinc finger protein 879	5q35.3	-0.375	0.04984	0.1667							1
ZNF90	zinc finger protein 90	19p12				-0.326	0.00026	0.0098				1
ZNF98	In multiple Geneids					-0.304	0.00166	0.0224				1
ZNFN1A2	Data not found								-0.743	2.55167	6	1
STRA13	In multiple Geneids		0.214	0.03808	0.1411							1
IGDCC4	immunoglobulin superfamily, DCC subclass, member 4	15q22.31	0.212	0.03729	0.1392							1
MOAP1	modulator of apoptosis 1	14q32	0.211	0.022	0.0988							1
ZNRF1	zinc and ring finger 1, E3 ubiquitin protein ligase	16q23.1	-0.784	1.00E-05	4.00E-04	-0.3657	0.004331	0.03113				2
ZNRF3	zinc and ring finger 3	22q12.1				-0.427844444	0.001145111	0.014348889				1
ZNRF4	zinc and ring finger 4	19p13.3	-0.262	0.00883	0.0536							1
ZP4	zona pellucida glycoprotein 4	1q43							-0.854	3.45	5	1
ZPBP	zona pellucida binding protein	7p14.3				0.3639375	0.015123125	0.0705125				1
ZPLD1	zona pellucida-like domain containing 1	3q12.3							-0.763	2.7	5	1
ZRANB1	zinc finger, RAN-binding domain containing 1	10q26.13	-0.449	0.00439	0.0334	-0.311	0.00024	0.0094				2
TBX2	T-box 2	17q23.2	0.208	0.04347	0.1534							1
ZRF1	Data not found								-0.641	2.67	7	1
ANKRD39	ankyrin repeat domain 39	2q11.2	0.206	0.02905	0.118							1
ZSCAN1	zinc finger and SCAN domain containing 1	19q13.43				-0.314	0.00145	0.021				1
ZSCAN10	zinc finger and SCAN domain containing 10	16p13.3	-0.27	0.01182	0.0656	-0.391	0.00421	0.0357				2
ARF1	ADP-ribosylation factor 1	1q42	0.202	0.03138	0.1243							1
ZSCAN18	zinc finger and SCAN domain containing 18	19q13.43	-0.25	0.03029	0.1215	-0.427	0.00053	0.0132				2
ZNF496	zinc finger protein 496	1q44	0.202	0.04664	0.1602							1
LOC220077	dedicator of cytokinesis 1 pseudogene	11q13.4	0.199	0.03376	0.1301							1
ZSCAN31	Data not found		-0.894	0	1.00E-04							1

ZSCAN5	Data not found								0.995	3.018	5	1
SEPT7P9	Data not found		0.198	0.03184	0.1254							1
ZSWIM4	zinc finger, SWIM-type containing 4	19p13.13	-0.862	9.00E-04	0.0109	-0.523666667	0.00094	0.016766667				2
RNF215	ring finger protein 215	22q12.2	0.186	0.04492	0.1567							1
ZSWIM6	zinc finger, SWIM-type containing 6	5q12.1	-0.538	0.00396	0.0311							1
ZSWIM7	zinc finger, SWIM-type containing 7	17p12				-0.3845	0.002055	0.0214				1
ZSWIM8	Data not found		-0.493	0.00271	0.0197							1
ZW10	ZW10, kinetochore associated, homolog (Drosophila)	11q23.2							-1.002	3.63714	7	1
PCGF1	polycomb group ring finger 1	2p13.1	0.184	0.04354	0.1536							1
SP3	Sp3 transcription factor	2q31	0.182	0.02586	0.1097							1
ZYG11A	zyg-11 homolog A (C. elegans)	1p32.3	-0.416	0.00774	0.0491							1
ZYX	zyxin	7q32				-0.3685	0.006005	0.03445				1
ZZEF1	zinc finger, ZZ-type with EF-hand domain 1	17p13.2				-0.37195	0.004406	0.03307				1
ZZZ3	zinc finger, ZZ-type containing 3	1p31.1				-0.355	0.00078	0.01525				1

Supplementary Table S3. Frequency of amplification and deletions

Gene	Number of Samples with Amplification	Gene	Number of Samples with Deletion
UGT2B17	11	PGA3	11
GUCY1A2	9	PGA4	11
LILRA3	9	PGA5	11
LRP1B	8	RRP7A	11
OR4F5	8	SERHL	11
TMPRSS11E	7	SERHL2	11
ATP6V1G3	6	APOBEC3A	10
C1orf105	6	APOBEC3B	10
C5orf51	6	ANGPTL6	9
C6	6	ANKRD54	9
CDH10	6	APOBEC3C	9
CDH9	6	APOBEC3D	9
CTCFL	6	APOBEC3F	9
CYP24A1	6	APOBEC3G	9
DOK5	6	APOBEC3H	9
FASLG	6	ATF4	9
FBXO4	6	BAIAP2L2	9
GLP1R	6	C22orf23	9
MED30	6	C3P1	9
OR4F15	6	CARD10	9
OXCT1	6	CBX7	9
PCK1	6	CCDC116	9
PFDN4	6	CDC37	9
PLCXD3	6	CDC42EP1	9
PMEPA1	6	DNMT1	9
PRDM9	6	EIF3L	9
PTPRC	6	FDX1L	9
TNFRSF11B	6	FLJ35390	9
TREM1	6	GCAT	9
TREML4	6	GGA1	9
TRPS1	6	ICAM1	9
ZFAND3	6	ICAM5	9
ADCY8	5	LGALS1	9
AGMO	5	LGALS2	9
APCDD1L	5	MAFF	9
ASPM	5	MFNG	9
BMPER	5	MGAT3	9
BRD2	5	MICALL1	9
BTBD3	5	MRPL4	9
BTBD9	5	NOL12	9
C20orf197	5	PDE4A	9
C6orf10	5	PDGFB	9

C6orf108	5	PICK1	9
C6orf132	5	PLA2G6	9
CBLN4	5	POLR2F	9
CDH18	5	POLR2J	9
CDH26	5	PPIL2	9
CERKL	5	RASA4	9
CNPY3	5	RAVER1	9
COLEC10	5	RPL3	9
CRB1	5	S1PR2	9
CSMD3	5	SDF2L1	9
CTNND2	5	SH3BP1	9
CTSZ	5	SLC16A8	9
CUL7	5	SMCR7L	9
DENND1B	5	SOX10	9
DGKB	5	SYNGR1	9
DLK2	5	TMEM184B	9
DNAH8	5	TRIOBP	9
EBAG9	5	TYK2	9
EDN3	5	UBE2L3	9
EFR3A	5	YDJC	9
EIF3H	5	YPEL1	9
ENY2	5	A4GALT	8
FAM110C	5	ABCA7	8
FAM5C	5	ABHD8	8
FASTKD3	5	ADAMTSL5	8
FERD3L	5	ADAT3	8
FGF10	5	AES	8
FOXP4	5	ANKRD24	8
FRS3	5	ANO8	8
GLO1	5	AP1B1	8
GNAS	5	AP1M2	8
GUCA1A	5	AP3D1	8
HAO1	5	APBA3	8
HDAC9	5	ARFGAP3	8
HLA-DPA1	5	ARHGAP8	8
HLA-DPB1	5	ARID3A	8
HLA-DQA2	5	ARMC6	8
IKZF2	5	ARRDC5	8
IRX1	5	ATCAY	8
ITGB8	5	ATG4D	8
KCNK2	5	ATP13A1	8
KCNK5	5	ATP5D	8
KCTD3	5	ATP6V1E1	8
KIF6	5	ATP8B3	8
KLC4	5	BCL2L13	8
LMBRD2	5	BID	8
LOC100132354	5	BIK	8

LOC285692	5	BST2	8
LRFN2	5	BTBD2	8
LYPLAL1	5	C19orf10	8
MACC1	5	C19orf25	8
MAD2L1BP	5	C19orf26	8
MAR11	5	C19orf38	8
MDGA1	5	C19orf6	8
MEOX2	5	C19orf71	8
MIR296	5	C19orf77	8
MRPL2	5	C1QTNF6	8
MTRR	5	C22orf31	8
NEK7	5	CARM1	8
NPEPL1	5	CBY1	8
NUDCD1	5	CCDC151	8
NXPH1	5	CCDC159	8
OC90	5	CCDC94	8
OR4F6	5	CDKN2D	8
PCMTD2	5	CECR2	8
PHACTR3	5	CELSR1	8
PIM1	5	CHAF1A	8
PKHD1L1	5	CILP2	8
POLR1C	5	CIRBP	8
PPP1R3D	5	CIRBP-AS1	8
PPP4R1L	5	CNN2	8
PRICKLE4	5	CREB3L3	8
PSMB8	5	CSNK1E	8
PSMB9	5	CSNK1G2	8
PTCRA	5	CYB5R3	8
RAB22A	5	CYP2D7P1	8
RAD21	5	CYTH4	8
RAE1	5	DAG1	8
RANBP3L	5	DAPK3	8
RBM38	5	DAZAP1	8
RGS1	5	DDA1	8
RGS13	5	DDX17	8
RGS18	5	DIRAS1	8
RGS21	5	DMC1	8
RNF8	5	DNAL4	8
RRP36	5	DNM2	8
SAMD12	5	DOCK6	8
SEMA5A	5	DOHH	8
SKP2	5	DOT1L	8
SLC30A8	5	DPP9	8
SLMO2	5	DUS3L	8
SPO11	5	EBI3	8
STX16	5	EEF2	8
SYCP2	5	EFNA2	8

TAP1	5	ELAVL3	8
TAS2R1	5	ELFN2	8
TDRG1	5	EMID1	8
THSD7A	5	EPOR	8
TJAP1	5	EWSR1	8
TMEM106B	5	FAM118A	8
TMEM196	5	FAM125A	8
TMEM217	5	FAM129C	8
TMEM74	5	FAM227A	8
TMX4	5	FBLN1	8
TREM2	5	FBXL12	8
TRERF1	5	FEM1A	8
TRHR	5	FSD1	8
TTBK1	5	FSTL3	8
TUBB1	5	FUT3	8
TWISTNB	5	FUT5	8
UBR2	5	FUT6	8
UGT3A1	5	FZR1	8
UGT3A2	5	GADD45B	8
USH2A	5	GAS2L1	8
UTP23	5	GIPC3	8
VAPB	5	GMIP	8
VEGFA	5	GNA11	8
XPO5	5	GNA15	8
YIPF3	5	GNG7	8
ZBTB41	5	GPX4	8
ZFHX4	5	GRAMD4	8
ZNF318	5	GTPBP1	8
ZNF831	5	GTPBP3	8
ABCF1	4	HAPLN4	8
ACP1	4	HMG20B	8
ADAMTS16	4	HSD11B1L	8
ADCY2	4	IL2RB	8
AGBL1	4	ILF3	8
AGPAT1	4	IZUMO4	8
AHR	4	JOSD1	8
AIF1	4	KANK2	8
AMACR	4	KCNJ4	8
ANGPT1	4	KCTD17	8
ANKRD5	4	KDELR3	8
ANKS1A	4	KDM4B	8
ANXA13	4	KEAP1	8
APOBEC2	4	KLF16	8
ATAT1	4	KREMEN1	8
BCAS1	4	KRI1	8
BMP2	4	LDLR	8
BMP7	4	LINC00634	8

BTN2A1	4	LINGO3	8
BTN2A2	4	LMNB2	8
BTN2A3P	4	LONP1	8
BTN3A1	4	LPAR2	8
BTN3A2	4	LPPR2	8
BTN3A3	4	LRG1	8
C12orf39	4	MAP2K2	8
C1orf116	4	MAPK1	8
C1orf53	4	MATK	8
C2	4	MAU2	8
C20orf26	4	MBD3	8
C20orf94	4	MCAT	8
C4BPA	4	MED16	8
C4BPB	4	MEF2B	8
C5orf28	4	MEX3D	8
C5orf34	4	MFSD12	8
C5orf38	4	MICAL3	8
C5orf42	4	MIR7-3HG	8
C5orf49	4	MKNK2	8
C6orf25	4	MLL3	8
C6orf48	4	MOB3A	8
C6orf89	4	MPND	8
C7	4	MPPED1	8
C7orf62	4	MPST	8
C8orf12	4	MRPL54	8
C9	4	MYH9	8
CALCR	4	NAGA	8
CAPSL	4	NCAN	8
CARD16	4	NCLN	8
CARD17	4	NDUFA11	8
CARD6	4	NDUFA13	8
CASP1	4	NDUFA6	8
CASP4	4	NDUFS7	8
CASP5	4	NEFH	8
CCDC129	4	NFAM1	8
CCDC132	4	NFIC	8
CCDC152	4	NPTXR	8
CCL28	4	NR2C2AP	8
CD55	4	NR2F6	8
CDC5L	4	NRTN	8
CDH4	4	NUP50	8
CENPF	4	OAZ1	8
CERS3	4	OCEL1	8
CFB	4	ONECUT3	8
CFH	4	OR2A20P	8
CFHR4	4	PACSIN2	8
CFHR5	4	PALM	8

CHD6	4	PARVB	8
CLIC1	4	PBX4	8
CLSTN2	4	PCSK4	8
CR1	4	PGLS	8
CR2	4	PHF21B	8
CRISP1	4	PI4KAP2	8
CRISP2	4	PIAS4	8
CRISP3	4	PIN1	8
DAAM2	4	PIP5K1C	8
DAB2	4	PLAC2	8
DDAH2	4	PLIN3	8
DDR1	4	PLIN4	8
DEF6	4	PLK5	8
DEFB110	4	PLVAP	8
DEFB112	4	POLDIP3	8
DEFB113	4	POLR2E	8
DEFB114	4	POLR2J2	8
DEFB125	4	POLR2J3	8
DEFB126	4	PRKCSH	8
DEFB127	4	PRR5	8
DLG1	4	PTBP1	8
DNAH5	4	PTPRS	8
DNM3	4	QTRT1	8
DOM3Z	4	RAB3D	8
DPCR1	4	RAC2	8
DUSP22	4	RAX2	8
EGFL8	4	RDH8	8
ESRRG	4	REEP6	8
ETNK1	4	REXO1	8
ETV1	4	RFPL1	8
EXT1	4	RFXANK	8
F13B	4	RGL3	8
FAM150B	4	RHBDD3	8
FAM91A1	4	RHCE	8
FAP	4	RIBC2	8
FBXL7	4	RNF126	8
FBXO32	4	RPS15	8
FER1L6	4	S1PR5	8
FKBP1A	4	SAFB	8
FKBPL	4	SAFB2	8
FLOT1	4	SBNO2	8
FMO2	4	SCAMP4	8
FMO6P	4	SCUBE1	8
FOXA2	4	SEMA6B	8
FYB	4	3-Sep	8
GABBR1	4	SF3A2	8
GCA	4	SGTA	8

GDNF	4	SH3GL1	8
GHR	4	SHD	8
GOLIM4	4	SIRT6	8
GOLT1B	4	SLC25A18	8
GPR111	4	SLC25A42	8
GPR115	4	SLC27A1	8
GPX5	4	SLC39A3	8
GPX6	4	SLC44A2	8
GRIA4	4	SMARCA4	8
GRIN2A	4	SMC1B	8
GRM3	4	SPC24	8
GSDMC	4	SPPL2B	8
GYS2	4	SSTR3	8
HCN1	4	STAP2	8
HEATR7B2	4	STK11	8
HEPACAM2	4	SUN2	8
HFE	4	TCF20	8
HGF	4	TCF3	8
HHLA1	4	THOP1	8
HIST1H1A	4	TICAM1	8
HIST1H1B	4	TJP3	8
HIST1H1C	4	TLE2	8
HIST1H1E	4	TLE6	8
HIST1H2AE	4	TM6SF2	8
HIST1H2AK	4	TMED1	8
HIST1H2BB	4	TMEM205	8
HIST1H2BD	4	TMIGD2	8
HIST1H2BG	4	TMPRSS6	8
HIST1H2BH	4	TMPRSS9	8
HIST1H2BI	4	TNFAIP8L1	8
HIST1H2BL	4	TOMM22	8
HIST1H2BM	4	TRMU	8
HIST1H2BO	4	TSPAN16	8
HIST1H3A	4	TSPO	8
HIST1H3B	4	TTLL1	8
HIST1H3C	4	TTLL12	8
HIST1H3E	4	UBL5	8
HIST1H3F	4	UBXN6	8
HIST1H3G	4	UHRF1	8
HIST1H3H	4	UPK3A	8
HIST1H3I	4	USE1	8
HIST1H4A	4	USHBP1	8
HIST1H4B	4	VMAC	8
HIST1H4D	4	WBP2NL	8
HIST1H4E	4	YIPF2	8
HIST1H4G	4	YJEFN3	8
HIST1H4K	4	ZBTB7A	8

HIST1H4L	4	ZFR2	8
HLA-A	4	ZNF554	8
HLA-E	4	ZNF555	8
HLA-G	4	ZNF57	8
HLA-H	4	ZNF653	8
HLA-J	4	ZNF77	8
HLA-L	4	ZNRF4	8
HMCN1	4	ACER1	7
HMGA1	4	ADAMTS10	7
HMGN4	4	ALG12	7
HSP90AB1	4	ALKBH4	7
HSPA1B	4	ALKBH7	7
HSPA1L	4	AMT	7
IAPP	4	ANGPTL4	7
IFIH1	4	APEH	7
IL7	4	APOL3	7
IL7R	4	ARHGEF18	7
INSM1	4	ARRDC2	7
IQSEC3	4	ASF1B	7
ISM1	4	ATRIP	7
KBTBD3	4	B3GNT3	7
KCNJ8	4	BRD1	7
KCNQ3	4	BSN	7
KCNT2	4	C19orf42	7
KCNV1	4	C19orf44	7
KHDRBS3	4	C19orf45	7
KIAA0947	4	C19orf57	7
KIAA1324L	4	C19orf59	7
KIF16B	4	C19orf60	7
KLHL38	4	C22orf24	7
LHX9	4	C3	7
LOC100130264	4	C3orf62	7
LOC100216001	4	C4orf48	7
LOC145820	4	CACNA1I	7
LOC284788	4	CACNG2	7
LOC286094	4	CALR3	7
LOC338588	4	CAMKV	7
LOC554223	4	CAMSAP3	7
LOC727677	4	CC2D1A	7
LRRC16A	4	CCDC124	7
LRRC37A	4	CCDC130	7
LRRC6	4	CCDC36	7
LRRC69	4	CCDC51	7
LSM2	4	CCDC71	7
LST1	4	CD209	7
LTA	4	CD320	7
LY6G5B	4	CD70	7

LY6G5C	4	CD97	7
LY6G6C	4	CDC34	7
LY6G6F	4	CECR1	7
MAFB	4	CECR5	7
MAL2	4	CECR6	7
MC3R	4	CELSR3	7
MDC1	4	CERK	7
MDFI	4	CERS4	7
MECOM	4	CHERP	7
MED20	4	CLEC17A	7
MFSD9	4	CLEC4G	7
MRPL14	4	CLEC4M	7
MRPS30	4	CLPP	7
MSANTD4	4	CLRN2	7
MSH5	4	COL5A3	7
MTCH1	4	COL7A1	7
MTSS1	4	COPE	7
MUC21	4	CPAMD8	7
MUT	4	CRB3	7
MYT1	4	CRELD2	7
NAALADL2	4	CRLF1	7
NCR3	4	CRTC1	7
NDUFB9	4	CXADRP2	7
NECAB1	4	DALRD3	7
NEU1	4	DCAF15	7
NFKBIE	4	DDX49	7
NFYA	4	DENND1C	7
NIPBL	4	DEPDC5	7
NKAPL	4	DNAJB1	7
NLGN1	4	DOK7	7
NOV	4	DRG1	7
NT5C1B	4	ECSIT	7
NUF2	4	EIF3D	7
NUP155	4	EIF4ENIF1	7
OPN5	4	ELAVL1	7
OR10C1	4	ELL	7
OR10X1	4	EVI5L	7
OR10Z1	4	FAM184B	7
OR11A1	4	FAM212A	7
OR12D3	4	FBN3	7
OR2B3	4	FCER2	7
OR2H2	4	FCHO1	7
OR4F16	4	FKBP8	7
OR4F29	4	FLJ41941	7
OR4F3	4	FOXRED2	7
OR5V1	4	GATAD2A	7
OR6K2	4	GDF1	7

OR6K3	4	GDF15	7
OR6N2	4	GIPC1	7
OR6Y1	4	GLT25D1	7
OSMR	4	GNAT1	7
OTOL1	4	GPX1	7
OTOR	4	GTF2F1	7
PACSIN1	4	GTSE1	7
PAIP1	4	HAUS8	7
PAK7	4	HCN2	7
PCLO	4	HDAC10	7
PCSK2	4	HGFAC	7
PDCD10	4	HIC2	7
PGBD1	4	HMGXB4	7
PGK2	4	HMOX1	7
PINX1	4	HYAL1	7
PKHD1	4	HYAL2	7
PKIA	4	IER2	7
PLCB1	4	IFI30	7
PLCB4	4	IL12RB1	7
PLEKHG4B	4	IL17RA	7
POM121L2	4	IL17REL	7
POU5F1	4	IL27RA	7
PPARD	4	IMPDH2	7
PPIL1	4	INPP5J	7
PPT2	4	IP6K1	7
PRKAA1	4	IP6K2	7
PRRT1	4	ISYNA1	7
PSORS1C2	4	JAK3	7
PSORS1C3	4	JAKMIP1	7
PTGER4	4	KANK3	7
PTPRT	4	KCNN1	7
PVT1	4	KHSRP	7
PYROXD1	4	KIAA1644	7
RDH14	4	KIAA1683	7
RECQL	4	KLHDC8B	7
RGS4	4	KLHL22	7
RGS5	4	KLHL26	7
RHAG	4	LAMB2	7
RICTOR	4	LAP3	7
RNF39	4	LDOC1L	7
RNF5	4	LIMK2	7
SCGN	4	LOC100128573	7
SCUBE3	4	LOC646498	7
SDCBP2	4	LRPAP1	7
SEMA3D	4	LRRC25	7
SEMA3E	4	LRRC8E	7
SEPP1	4	LSM4	7

SERPINI1	4	MADCAM1	7
SH3YL1	4	MAN2B2	7
SIGLEC14	4	MAP1S	7
SIRPB1	4	MAPK12	7
SIRPD	4	2-Mar	7
SLC17A1	4	MAST3	7
SLC17A3	4	MCM5	7
SLC17A4	4	MCOLN1	7
SLC1A3	4	MED15	7
SLC24A3	4	MED26	7
SLC26A7	4	MLC1	7
SLC29A1	4	MLLT1	7
SLC35B2	4	MON1A	7
SLC35G5	4	MORC2	7
SLC44A4	4	MOV10L1	7
SLC45A2	4	MRI1	7
SLC8A1	4	MUM1	7
SLC9A2	4	MXD4	7
SLCO1A2	4	MYO1F	7
SLITRK5	4	MYO9B	7
SNAP25	4	NACC1	7
SNORA38	4	NANOS3	7
SNORD52	4	NCKIPSD	7
SNRPB2	4	NF2	7
SNRPC	4	NICN1	7
SOX5	4	NIPSNAP1	7
SPATA16	4	NWD1	7
SPEF2	4	OLFM2	7
SPTA1	4	OR4K1	7
SPTLC3	4	OR4K2	7
SPTSSB	4	OR4K5	7
SUPT3H	4	OR4M1	7
SYBU	4	OR4N2	7
TASP1	4	OR4Q3	7
TATDN1	4	OSBP2	7
TCTE1	4	P4HTM	7
TDH	4	PALM3	7
TDP2	4	PANX2	7
TFAP2B	4	PARVG	7
TFAP2D	4	PATZ1	7
TFEB	4	PDE4C	7
TMEM151B	4	PEX26	7
TMEM182	4	PFKFB4	7
TMEM55A	4	PGPEP1	7
TMEM64	4	PI4KA	7
TMEM71	4	PIK3IP1	7
TNF	4	PIK3R2	7

TOP1	4	PIM3	7
TRIM10	4	PKDREJ	7
TRIM15	4	PKN1	7
TRIM31	4	PLA2G3	7
TSHZ2	4	PLXNB1	7
TTC33	4	PLXNB2	7
UBD	4	PNPLA3	7
UNC5CL	4	PNPLA5	7
VWA7	4	PNPLA6	7
VWDE	4	PODNL1	7
WDR49	4	POLN	7
XKR6	4	POM121L4P	7
YOD1	4	PPM1F	7
ZBTB12	4	PPP2R2C	7
ZC2HC1A	4	PRAM1	7
ZFAT	4	PRKACA	7
ZHX3	4	PRKAR2A	7
ZNF165	4	PRR14L	7
ZNF311	4	QARS	7
ZNF322	4	QDPR	7
ZNF804B	4	QRICH1	7
ZNRD1	4	RAB11B	7
ZSCAN16	4	RAB3A	7
AADACL2	3	RANBP3	7
ABCC9	3	RBM5	7
ABI3BP	3	RBM6	7
ACAD11	3	RFX1	7
ACPL2	3	RGS12	7
ACPP	3	RHD	7
ADAMTS12	3	RHOA	7
ADAMTSL3	3	RNF123	7
AGR2	3	RNF185	7
AGR3	3	RNF4	7
AGTR1	3	RPL18A	7
AGXT2	3	SAMM50	7
AKR1C1	3	SCARF2	7
AKR1C2	3	SEL1L3	7
AKR1C3	3	SELM	7
AKR1E2	3	SELO	7
ALCAM	3	SEMA3B	7
ALDH1A1	3	SEMA3F	7
ALDH1A3	3	SERPIND1	7
ANKH	3	SFI1	7
ANKIB1	3	SHISA5	7
ANKMY2	3	SIN3B	7
ANKRD22	3	SLC25A23	7
ANXA1	3	SLC25A41	7

APMAP	3	SLC26A6	7
ARGLU1	3	SLC34A2	7
ARHGAP15	3	SLC35E1	7
ARL4A	3	SLC38A3	7
ARMC12	3	SLC5A5	7
ASB4	3	SMTN	7
ASB7	3	SNAPC2	7
ASPH	3	SREBF2	7
ATAD2	3	SSBP4	7
ATXN1	3	STX10	7
AURKA	3	STXBP2	7
B3GALT4	3	TECR	7
B3GALTL	3	THOC5	7
B3GAT2	3	TIMM44	7
BBX	3	TMA7	7
BCL6	3	TMEM38A	7
BET1	3	TNFSF14	7
BHLHE41	3	TOM1	7
BMP5	3	TPGS1	7
BOC	3	TRABD	7
BRCA2	3	TRAIP	7
BRIX1	3	TRAPPC5	7
BRPF3	3	TRIML1	7
BTLA	3	TRIML2	7
C10orf107	3	TTC38	7
C1GALT1	3	TUBA8	7
C1QTNF3	3	TUBB4A	7
C1orf115	3	TUBGCP6	7
C1orf129	3	TUG1	7
C1orf140	3	TXN2	7
C1orf27	3	UBA52	7
C1orf74	3	UBA7	7
C3orf17	3	UNC13A	7
C3orf52	3	UPF1	7
C3orf55	3	UQCRC1	7
C3orf58	3	USP18	7
C6orf222	3	USP19	7
C6orf58	3	USP4	7
C8orf48	3	WDR6	7
C8orf74	3	WFS1	7
CA8	3	WHSC1	7
CADM1	3	WHSC2	7
CALB1	3	XAB2	7
CALCRL	3	ZBED4	7
CAMK1G	3	ZFP42	7
CAPS2	3	ZFYVE28	7
CASC1	3	ZNF101	7

CASC2	3	ZNF14	7
CBLB	3	ZNF358	7
CCDC14	3	ZNF414	7
CCDC141	3	ZNF658	7
CCDC70	3	ZNF74	7
CCDC91	3	ZNRF3	7
CCDC93	3	ZSWIM4	7
CCNL1	3	ABCA17P	6
CCRL1	3	ABCA3	6
CD200	3	ABCG1	6
CD200R1	3	ABCG2	6
CD2AP	3	ABLIM2	6
CD34	3	ACER2	6
CD36	3	ACO2	6
CD46	3	ACOT7	6
CD47	3	ACOX3	6
CD83	3	ACSBG2	6
CD93	3	ACSL1	6
CDC16	3	ADCY9	6
CDH6	3	ADD1	6
CDK6	3	AFAP1	6
CENPW	3	AFF1	6
CEP72	3	AGPAT3	6
CFHR3	3	AGXT2L1	6
CHD1L	3	AIFM3	6
CHL1	3	AKAP8	6
CHSY1	3	AKAP8L	6
CLDN1	3	ALG1	6
CLDN12	3	ALOX12B	6
CLDN16	3	ALOXE3	6
CLDN18	3	AMDHD2	6
CLIC5	3	AMY1A	6
CLVS1	3	AMY1B	6
CMAS	3	AMY1C	6
CMPK2	3	ANAPC4	6
CNTN5	3	ANKRD37	6
COBLL1	3	ANKS3	6
COL11A2	3	AP1M1	6
COL14A1	3	APOL1	6
COL1A2	3	APOL4	6
COL28A1	3	APOL5	6
COL3A1	3	APOL6	6
COL5A2	3	APRT	6
COL6A5	3	ARHGDIG	6
COL6A6	3	ARHGEF15	6
COL8A1	3	ARIH2	6
COPB2	3	ARVCF	6

CPB1	3	ASCC2	6
CPNE4	3	ASNA1	6
CPS1	3	ATP5I	6
CR1L	3	ATP5O	6
CRCT1	3	ATP6V0C	6
CREM	3	AXIN1	6
CRNN	3	BEST2	6
CRYBG3	3	BNC2	6
CSMD1	3	BRD4	6
CSRNP3	3	BTNL3	6
CST1	3	C16orf11	6
CST11	3	C16orf5	6
CST13P	3	C16orf71	6
CST2	3	C16orf89	6
CST3	3	C17orf59	6
CST4	3	C19orf43	6
CST8	3	C1QTNF8	6
CSTL1	3	C21orf128	6
CUL2	3	C21orf2	6
CUTA	3	C21orf33	6
CWC22	3	C21orf56	6
CYP39A1	3	C21orf58	6
DACH1	3	C21orf67	6
DAOA	3	C21orf90	6
DAP	3	C22orf25	6
DCDC2	3	C22orf26	6
DDX18	3	C2CD2	6
DDX39B	3	C2CD4C	6
DEFB132	3	C4orf36	6
DERL1	3	CABP7	6
DIEXF	3	CACNA1H	6
DIRC1	3	CALR	6
DLC1	3	CAMKK1	6
DMTF1	3	CAMP	6
DNAJC13	3	CASKIN1	6
DNAJC21	3	CASP14	6
DPP10	3	CASP3	6
DPP4	3	CASP6	6
DSCC1	3	CBFA2T3	6
DTNBP1	3	CBR1	6
DUSP10	3	CBR3	6
DYRK3	3	CBS	6
ECT2	3	CCDC105	6
EDN1	3	CCDC109B	6
EDNRB	3	CCDC111	6
EEF1DP3	3	CCDC117	6
EFNB2	3	CCDC134	6

EGFLAM	3	CCDC154	6
EIF5A2	3	CCDC157	6
ELMO1	3	CCDC64B	6
ENPP2	3	CCDC96	6
ENPP4	3	CCL3L1	6
EPHA6	3	CCL4L1	6
ERBB4	3	CCNF	6
ESF1	3	CDC25A	6
ETS1	3	CDKN2AIP	6
EXOC3	3	CEMP1	6
EYA1	3	CFI	6
FAIM3	3	CHADL	6
FAM105A	3	CHAF1B	6
FAM105B	3	CHKB-CPT1B	6
FAM133B	3	CIB3	6
FAM134B	3	CLCN7	6
FAM155A	3	CLDN14	6
FAM49A	3	CLDN22	6
FAM49B	3	CLDN6	6
FAM5B	3	CLTCL1	6
FAM83A	3	CLUAP1	6
FAS	3	CNN1	6
FGF14	3	COL18A1	6
FILIP1L	3	COL6A2	6
FKBP5	3	COMT	6
FKBP7	3	COPS3	6
FLG	3	COPS4	6
FLI1	3	CORO7	6
FLJ33360	3	CPLX1	6
FMO1	3	CPT1B	6
FMO4	3	CPZ	6
FMO5	3	CRAMP1L	6
FOXP2	3	CREBBP	6
FRY	3	CRKL	6
FSIP2	3	CSDC2	6
FZD1	3	CSF2RB	6
G0S2	3	CSPG5	6
GABRR3	3	CSTB	6
GALNT3	3	CTBP1	6
GAP43	3	CYP4F22	6
GATA4	3	DAND5	6
GATAD1	3	DCTD	6
GFM1	3	DENND4C	6
GFOD1	3	DESI1	6
GJD4	3	DGCR14	6
GLCCI1	3	DGCR2	6
GNAI1	3	DGCR5	6

GNAT3	3	DGCR6	6
GNGT1	3	DGCR8	6
GOLPH3	3	DGKQ	6
GPC5	3	DHPS	6
GPC6	3	DHX30	6
GPR128	3	DNAJA3	6
GPR171	3	DNASE2	6
GPR180	3	DNMT3L	6
GPR37	3	DOPEY2	6
GPR87	3	DSCR3	6
GRB14	3	DSCR6	6
GTPBP8	3	DSCR9	6
GULP1	3	DUSP18	6
GUSBP1	3	EFCAB6	6
HAS2	3	EGF	6
HCP5	3	ELOVL6	6
HHAT	3	EMR3	6
HHLA2	3	ENPP6	6
HLA-B	3	EP300	6
HLA-C	3	EPHX3	6
HLA-F	3	EPS15L1	6
HNMT	3	ESPN	6
HRNR	3	FAHD1	6
HS6ST3	3	FAM195A	6
HSD11B1	3	FAM32A	6
HSD17B8	3	FAM53A	6
HSPH1	3	FAM57A	6
ICA1	3	FAM92A1P2	6
IFI16	3	FARSA	6
IFLTD1	3	FBXL16	6
IGFBP1	3	FGFR3	6
IGFBP3	3	FGFRL1	6
IGSF10	3	FLCN	6
IKBKE	3	FLJ36777	6
IKZF1	3	FLJ42627	6
IL18R1	3	FLYWCH1	6
IL18RAP	3	FLYWCH2	6
IL19	3	FTCD	6
IL1RAP	3	GADD45GIP1	6
IL1RL1	3	GAK	6
INSIG2	3	GAL3ST1	6
IRF2BP2	3	GALNS	6
IRF6	3	GAR1	6
IRX4	3	GCDH	6
ITGA4	3	GEMIN4	6
ITGBL1	3	GFER	6
ITPR3	3	GLIS2	6

IVL	3	GLOD4	6
KCNC2	3	GNB1L	6
KCNH7	3	GNG13	6
KCNMB2	3	GNPTG	6
KIAA0040	3	GP1BB	6
KIAA0087	3	GPR108	6
KIAA0196	3	GPR78	6
KIAA0528	3	GRK4	6
KIFC1	3	GRPEL1	6
KIRREL3-AS3	3	GSTT1	6
KPRP	3	GSTTP2	6
KRAS	3	HAGH	6
LAMB3	3	HAUS6	6
LCE1A	3	HELT	6
LCE1B	3	HERC2P2	6
LCE1C	3	HERC6	6
LCE1F	3	HES2	6
LCE2A	3	HES7	6
LCE3B	3	HIRA	6
LCE3C	3	HLCS	6
LCE3D	3	HMOX2	6
LCE3E	3	HN1L	6
LCE4A	3	HNRNPM	6
LCE6A	3	HOOK2	6
LEKR1	3	HS3ST6	6
LELP1	3	HSD17B11	6
LIFR	3	HSD17B13	6
LIPF	3	HSH2D	6
LIPJ	3	HTRA3	6
LIPN	3	HTT	6
LOC153684	3	HUNK	6
LOC154872	3	ICOSLG	6
LOC201651	3	IFT140	6
LOC339568	3	IL32	6
LOR	3	ILVBL	6
LRMP	3	ING2	6
LRRD1	3	INSR	6
LSAMP	3	ITFG3	6
LXN	3	ITGB2	6
MAPKAPK2	3	JUNB	6
2-Mar	3	KCTD5	6
6-Mar	3	KIAA0232	6
MARK1	3	KIAA0664	6
MBNL1	3	KIAA1430	6
MBNL2	3	KIAA1671	6
MCTP2	3	KLF1	6
MDFIC	3	KLF2	6

METTL13	3	KLHL18	6
MFSD1	3	KLHL8	6
MIA3	3	KRTAP10-1	6
MICA	3	KRTAP10-12	6
MICB	3	KRTAP10-4	6
MIOS	3	KRTAP10-7	6
MIR128-2	3	KRTAP10-9	6
MIR205	3	KRTAP12-1	6
MIR622	3	L3MBTL2	6
MLF1	3	LCA5L	6
MLIP	3	LETM1	6
MLN	3	LIN54	6
MMP16	3	LMF1	6
MPP6	3	LMF2	6
MRPL13	3	LOC100128288	6
MRPL3	3	LOC100134368	6
MRPL36	3	LOC150185	6
MSRA	3	LOC284837	6
MTBP	3	LOC400891	6
N4BP2L1	3	LOC400927	6
N4BP2L2	3	LOC642852	6
NAA20	3	LOC643837	6
NAPB	3	LOC652276	6
NBEA	3	LOC727924	6
NDRG1	3	LPHN1	6
NDUFAF5	3	LRP2BP	6
NDUFS6	3	LSS	6
NEUROD1	3	LUC7L	6
NFATC2	3	LYAR	6
NIM1	3	LZTR1	6
NMNAT3	3	MAEA	6
NNT	3	MAN2B1	6
NOL7	3	MAP4	6
NPR3	3	MAPK8IP3	6
NR2F2	3	MAST1	6
NSMCE2	3	MB	6
NSUN2	3	MCHR1	6
NTM	3	MCM3AP	6
OGFRL1	3	MEI1	6
OLFM4	3	MFSD10	6
OR10J1	3	MGC3771	6
OR10J5	3	MGRN1	6
OR10K1	3	MIER2	6
OR10R2	3	MIR185	6
OR14I1	3	MIRLET7A3	6
OR2G6	3	MIRLET7B	6
OR2M7	3	MKL1	6

OR2T1	3	MLF1IP	6
OR2T10	3	MLST8	6
OR2T11	3	MN1	6
OR2T2	3	MORC3	6
OR2T3	3	MRFAP1L1	6
OR2T33	3	MRPL28	6
OR2T35	3	MRPL40	6
OR2T4	3	MSLN	6
OR2T5	3	MTMR3	6
OR2T6	3	MUC16	6
OR4M2	3	MUC20	6
OR4N4	3	MX1	6
OR5AC2	3	MYL5	6
OR5H1	3	NAGPA	6
OR5H14	3	NARFL	6
OR5H15	3	NCF4	6
OR5H2	3	NDUFB10	6
OR5H6	3	NDUFV3	6
OR6N1	3	NFIX	6
OXGR1	3	NHP2L1	6
OXR1	3	NLRC3	6
P2RY1	3	NME4	6
P2RY12	3	NME6	6
P2RY13	3	NMRAL1	6
PAX1	3	NOA1	6
PDE3A	3	NOP14	6
PDGFD	3	NOTCH3	6
PDIA3P	3	NSG1	6
PDILT	3	NSUN7	6
PDS5B	3	NT5M	6
PDZD2	3	NTN3	6
PFDN6	3	NUDT9	6
PGBD2	3	NXN	6
PGLYRP3	3	ODF4	6
PGLYRP4	3	ODZ3	6
PHACTR1	3	OR1I1	6
PHF1	3	OR2C1	6
PHF20L1	3	OR2Z1	6
PHLDB2	3	OR7G1	6
PIGR	3	OR7G2	6
PLA2G4A	3	OR7G3	6
PLA2G7	3	OSTC	6
PLD1	3	OTOP1	6
PLEKHA3	3	PCGF3	6
PLOD2	3	PCNT	6
PLSCR1	3	PDE6B	6
PLSCR2	3	PDE9A	6

PLSCR4	3	PDPK1	6
PLSCR5	3	PDPR	6
PLXNA2	3	PDXK	6
POT1	3	PER1	6
POU6F2	3	PES1	6
PPP1R1C	3	PFAS	6
PRDX6	3	PFKL	6
PRG4	3	PGLYRP2	6
PRKAB2	3	PHF5A	6
PRKCE	3	PI4K2B	6
PRLR	3	PIGG	6
PROX1	3	PKD1	6
PRRC2C	3	PKD2	6
PTGS2	3	PKMYT1	6
PTHLH	3	PKNOX1	6
PTPRK	3	PLA2G12A	6
PVRL3	3	PLEKHG5	6
PYHIN1	3	PLIN2	6
RAB11FIP2	3	PMM1	6
RAD1	3	POFUT2	6
RAI14	3	POLR2B	6
RALGAPA2	3	POLR3H	6
RANBP9	3	PPARA	6
RAP2A	3	PPL	6
RARRES1	3	PPM1K	6
RASSF5	3	PRDM15	6
RASSF8	3	PRDX2	6
RBP2	3	PRODH	6
RCAN2	3	PRSS27	6
RFC3	3	PTPN23	6
RING1	3	PTPRD	6
RNLS	3	PTTG1IP	6
ROPN1L	3	PVALB	6
RORC	3	RAB11FIP3	6
RPA3	3	RAB26	6
RPTN	3	RAB40C	6
RSAD2	3	RAB8A	6
RSPO3	3	RAD23A	6
RSRC1	3	RANGAP1	6
RUNX1T1	3	RANGRF	6
RUNX2	3	RASAL3	6
RXFP2	3	RASD2	6
RXRB	3	RASSF1	6
S100A10	3	RBM47	6
S100A11	3	RBPJ	6
S100A12	3	REST	6
S100A7L2	3	RFX2	6

S100A8	3	RGS11	6
S100A9	3	RIPK4	6
SAMD9	3	RNASEH2A	6
SATB1	3	RNF151	6
SCIN	3	RNF212	6
SCN2A	3	RNF215	6
SCN3A	3	RNMTL1	6
SDPR	3	RNPS1	6
SEMA3A	3	ROGDI	6
SEMA3C	3	RPL34	6
SH3BP5L	3	RPL3L	6
SHOX2	3	RPS2	6
SIRPA	3	RPS6	6
SLA	3	RRAGA	6
SLC10A2	3	RRH	6
SLC13A1	3	RRP1	6
SLC25A27	3	RRP1B	6
SLC25A36	3	RTBDN	6
SLC2A2	3	RWDD4	6
SLC38A11	3	SBF1	6
SLC39A7	3	SCAP	6
SLC9A4	3	SCO2	6
SLC9A9	3	SEC14L2	6
SLCO1B1	3	SEC14L3	6
SLCO1B7	3	SEC14L4	6
SLITRK6	3	SEC14L5	6
SMCP	3	SEC24B	6
SNORD117	3	SEC31A	6
SNTB1	3	12-Sep	6
SNX13	3	5-Sep	6
SNX19	3	SETD4	6
SOSTDC1	3	SH2D3A	6
SOX21	3	SH3BGR	6
SOX7	3	SH3BP2	6
SPAG16	3	SH3TC1	6
SPRR1A	3	SHC2	6
SPRR1B	3	SIM2	6
SPRR2A	3	SLBP	6
SPRR2B	3	SLC10A6	6
SPRR2D	3	SLC19A1	6
SPRR2E	3	SLC25A20	6
SPRR2F	3	SLC25A35	6
SPRR2G	3	SLC25A4	6
SPRR3	3	SLC35E4	6
SPRR4	3	SLC7A4	6
SQLE	3	SLX4	6
SRBD1	3	SMARCC1	6

SRD5A1	3	SNAP29	6
SRI	3	SNX25	6
SSFA2	3	SPINK2	6
SSPN	3	SPON2	6
SSR3	3	SPSB3	6
SST	3	SRL	6
SSTR4	3	SRRM2	6
ST3GAL1	3	STOX2	6
ST8SIA1	3	STUB1	6
STAMBPL1	3	SUMO3	6
STARD13	3	SYCE2	6
STEAP2	3	SYDE1	6
STEAP4	3	TACC3	6
STK38	3	TADA2B	6
SUCNR1	3	TBC1D14	6
SYNGAP1	3	TBC1D24	6
SYT14	3	TBC1D3C	6
TAF2	3	TBL3	6
TAGLN3	3	TCEB2	6
TAPBP	3	TCN2	6
TARS	3	TEF	6
TCHHL1	3	TELO2	6
TDRKH	3	TFAP4	6
TFPI	3	THAP9	6
TG	3	THEG	6
TGDS	3	TIAM1	6
THBD	3	TIGD7	6
THEM4	3	TIMM22	6
THEM5	3	TMEM107	6
THEMIS	3	TMEM115	6
TINAG	3	TMEM175	6
TIPARP	3	TMEM204	6
TLR4	3	TMPRSS2	6
TM4SF1	3	TNFRSF12A	6
TM4SF18	3	TNFRSF25	6
TMEFF2	3	TNIP2	6
TMEM207	3	TNPO2	6
TMEM229A	3	TOB2	6
TMEM26	3	TPM4	6
TMPRSS7	3	TPSG1	6
TNFSF18	3	TRAF7	6
TNFSF4	3	TRAP1	6
TNIK	3	TRAPPC10	6
TNKS	3	TRAPPC11	6
TOX3	3	TRIM61	6
TP53TG3	3	TRIP10	6
TP63	3	TRMT44	6

TPR	3	TRPM2	6
TPRG1	3	TSC2	6
TRAF3IP3	3	TSSK2	6
TRIB1	3	TXNRD2	6
TRIB2	3	U2AF1	6
TRIM27	3	UBN1	6
TRIO	3	UBXN11	6
TTC23L	3	UFD1L	6
TTN	3	UFSP2	6
U2SURP	3	UMODL1	6
UBE2E3	3	UNKL	6
UGGT2	3	URB1	6
VEPH1	3	VAMP2	6
VPS41	3	VASN	6
VSX1	3	WDR24	6
WDFY2	3	WDR4	6
WDR70	3	WDR83OS	6
WISP1	3	WIZ	6
ZAR1L	3	WNT7B	6
ZBED2	3	WRB	6
ZBTB20	3	WWC2	6
ZBTB38	3	WWC2-AS2	6
ZHX1	3	XBP1	6
ZHX2	3	XRCC6	6
ZIC4	3	YBEY	6
ZNF107	3	YWHAE	6
ZNF117	3	ZC3H7B	6
ZNF131	3	ZCCHC4	6
ZNF217	3	ZG16B	6
ZNF273	3	ZMAT5	6
ZNF385B	3	ZNF174	6
ZNF462	3	ZNF177	6
ZNF622	3	ZNF200	6
ZNF672	3	ZNF205	6
ZNF804A	3	ZNF213	6
ZPLD1	3	ZNF263	6
ZWINT	3	ZNF266	6
A1CF	2	ZNF295	6
ABCA1	2	ZNF295-AS1	6
ABCA13	2	ZNF317	6
ABCB1	2	ZNF333	6
ABCB11	2	ZNF434	6
ABCB4	2	ZNF500	6
ABCB5	2	ZNF557	6
ABCC4	2	ZNF558	6
ACP6	2	ZNF560	6
ACSM3	2	ZNF589	6

ACSS3	2	ZNF597	6
ACTL7B	2	ZNF721	6
ACTR3	2	ZNF75A	6
ACVR2A	2	ZNF791	6
ADAM18	2	ZSCAN10	6
ADAM22	2	AASDH	5
ADAM28	2	ABR	5
ADAMDEC1	2	ACADVL	5
ADAMTS17	2	ACAP1	5
ADAR	2	ACO1	5
ADIPOR1	2	ACSF3	5
ADPRHL1	2	ADORA2A	5
AGPAT6	2	ADPRM	5
AGTPBP1	2	ADRBK2	5
AHRR	2	ADSL	5
AKAP11	2	AGPAT9	5
AKAP13	2	AHDC1	5
ALDOB	2	AIM1L	5
ALG11	2	AK3	5
ALG5	2	ALOX12	5
ALK	2	ALOX15B	5
AMPH	2	ANKRD11	5
ANGPT2	2	ANO9	5
ANGPTL1	2	AP2A2	5
ANK1	2	APBB2	5
ANKK1	2	APP	5
ANKRD10	2	ARF3	5
ANKRD50	2	ARHGAP10	5
ANXA2P3	2	ARHGEF5	5
AP3M2	2	ARID1A	5
AP3S2	2	ARL9	5
APBA1	2	ARPC4	5
APCS	2	ARSA	5
APOB	2	ASGR1	5
APOBEC4	2	ASGR2	5
AQR	2	ATP1B2	5
ARF6	2	ATP2A3	5
ARHGAP12	2	ATP5A1	5
ARHGAP20	2	AUNIP	5
ARHGAP32	2	BANP	5
ARHGAP42	2	BEND4	5
ARHGDIB	2	BHLHA9	5
ARHGEF26	2	BHMT2	5
ARHGEF7	2	BPHL	5
ARL14	2	BRK1	5
ASAP1	2	BRPF1	5
ASTE1	2	BRWD1	5

ASTN1	2	C11orf35	5
ASTN2	2	C16orf55	5
ATF6	2	C16orf74	5
ATP11A	2	C17orf49	5
ATP12A	2	C17orf61	5
ATP13A4	2	C17orf74	5
ATP13A5	2	C17orf85	5
ATP2C1	2	C17orf97	5
ATP5EP2	2	C1orf172	5
ATP7B	2	C21orf54	5
ATP8A2	2	C22orf13	5
AVEN	2	C22orf15	5
B3GALT2	2	C22orf34	5
BAAT	2	C22orf43	5
BAI3	2	C3orf24	5
BBOX1	2	C4A	5
BBS9	2	C4B	5
BCAT1	2	C4orf21	5
BCHE	2	C4orf29	5
BCL11A	2	C4orf34	5
BCL9	2	C4orf6	5
BFSP2	2	CA5A	5
BIVM	2	CABIN1	5
BLZF1	2	CACNA1A	5
BPNT1	2	CACNA2D2	5
BRD9	2	CAGE1	5
BZW2	2	CAMK1	5
C10orf68	2	CAMK1D	5
C11orf45	2	CAMTA2	5
C12orf29	2	CAP2	5
C12orf50	2	CAPZA1	5
C13orf35	2	CATSPER4	5
C15orf41	2	CBR4	5
C15orf53	2	CCDC110	5
C1QTNF9B-AS1	2	CCDC149	5
C1orf110	2	CCDC171	5
C1orf112	2	CCDC42	5
C1orf114	2	CCDC65	5
C1orf21	2	CCKAR	5
C20orf43	2	CCR4	5
C4A	2	CCRN4L	5
C4B	2	CD151	5
C4orf33	2	CD164L2	5
C5orf22	2	CD52	5
C7orf25	2	CD68	5
C7orf57	2	CDC123	5
C8orf4	2	CDC37L1	5

C9orf135	2	CDHR5	5
C9orf156	2	CDK10	5
C9orf64	2	CDKL2	5
CAB39L	2	CECR7	5
CACNA1E	2	CELSR2	5
CACNA2D1	2	CENPV	5
CACYBP	2	CEP135	5
CALN1	2	CHCHD10	5
CAPN2	2	CHD3	5
CAPNS2	2	CHD5	5
CAPZA3	2	CHEK2	5
CARKD	2	CHIC2	5
CASD1	2	CHID1	5
CCDC122	2	CHMP1A	5
CCDC146	2	CHRFAM7A	5
CCDC85A	2	CHRNA9	5
CCER1	2	CHRNB1	5
CCL3L1	2	CHRNE	5
CCL4L1	2	CIDECP	5
CCT5	2	CISH	5
CCT6A	2	CLEC10A	5
CD84	2	CLEC18A	5
CDADC1	2	CLIC3	5
CDC42EP3	2	CLOCK	5
CDC73	2	CNKSR1	5
CDH12	2	CNOT10	5
CDH22	2	CNTROB	5
CDK18	2	COL6A1	5
CDK5RAP2	2	COQ2	5
CDX2	2	COX4I1	5
CENPJ	2	CPNE9	5
CENPL	2	CRELD1	5
CEP192	2	CRK	5
CEP290	2	CRMP1	5
CHD2	2	CRTAP	5
CHRM5	2	CRYBB2	5
CHRNB3	2	CRYBB3	5
CHTOP	2	CRYZL1	5
CIB1	2	CTNS	5
CIDEA	2	CXADR	5
CKS2	2	CYBA	5
CLDN10	2	CYP4F3	5
CLDND1	2	CYP4V2	5
CLN8	2	DDN	5
CLPTM1L	2	DDX58	5
CLYBL	2	DEAF1	5
CMBL	2	DEFB104A	5

CMSS1	2	DEFB105A	5
CNBD1	2	DEFB106A	5
CNGB3	2	DGCR6L	5
CNTN3	2	DHDDS	5
CNTNAP5	2	DHH	5
COBL	2	DHRS7C	5
COG3	2	DHTKD1	5
COG6	2	DLG4	5
COL10A1	2	DMRT1	5
COL15A1	2	DMRT3	5
COL22A1	2	DNAH2	5
COL4A1	2	DNAJC28	5
COL4A2	2	DNAJC8	5
CP	2	DPEP1	5
CPB2	2	DVL2	5
CREB3L4	2	EFCAB4A	5
CREB5	2	EFNB3	5
CRHR1	2	EIF3C	5
CRIM1	2	EIF3CL	5
CRIPT	2	EIF4A1	5
CRISPLD1	2	EIF4E	5
CRLS1	2	EIF5A	5
CROT	2	ELAC1	5
CRP	2	ELAVL2	5
CRTC2	2	ELF2	5
CSDA	2	ELOF1	5
CTAGE1	2	EMC3	5
CTNNA2	2	EMC8	5
CTSE	2	EMR2	5
CYB5R1	2	ENO3	5
CYLC2	2	ENOPH1	5
CYP21A2	2	EPB41	5
CYP7B1	2	EPS8L2	5
DAPK1	2	EVC	5
DARC	2	EVC2	5
DARS2	2	EXTL1	5
DBC1	2	EYA3	5
DBNDD2	2	F11	5
DCLK1	2	FAM101B	5
DCN	2	FAM149A	5
DCUN1D2	2	FAM154A	5
DDC	2	FAM175A	5
DDR2	2	FAM19A3	5
DDX59	2	FAM19A5	5
1-Dec	2	FAM211A	5
DECR1	2	FAM46B	5
DEFB1	2	FAM54B	5

DEFB104A	2	FAM76A	5
DEFB105A	2	FAM83F	5
DEFB106A	2	FANCA	5
DENND4B	2	FANCD2	5
DGKH	2	FBXO31	5
DHRS4L2	2	FBXO39	5
DHRS9	2	FBXW5	5
DHX36	2	FCN3	5
DIRAS2	2	FGF11	5
DISP1	2	FGR	5
DKK4	2	FHIT	5
DLEU7	2	FKBP11	5
DLX5	2	FOXE3	5
DNAH11	2	FRG1	5
DNAJC15	2	FRG2	5
DNAJC3	2	FXR2	5
DNAJC5B	2	GAB4	5
DOCK9	2	GABARAP	5
DPT	2	GART	5
DPY19L1	2	GAS7	5
DROSHA	2	GATSL3	5
DST	2	GGT1	5
DSTYK	2	GGT5	5
DUSP23	2	GHRL	5
DYNC1I1	2	GHRLOS	5
DYNC2H1	2	GLB1	5
DZIP3	2	GLDC	5
EDEM3	2	GLP2R	5
EFEMP1	2	GMEB1	5
EGFR	2	GPATCH3	5
EHBP1	2	GPN2	5
ELK4	2	GRAP2	5
ELMO2	2	GRAPL	5
EMC4	2	GSG2	5
EML6	2	GSTTP1	5
ENAH	2	GTF2H2D	5
ENOX1	2	GUCY2D	5
ENTPD6	2	GUSBP11	5
EPAS1	2	H19	5
EPB41L4B	2	HELQ	5
EPHA3	2	HEMK1	5
EPHB1	2	HERC2	5
EPPK1	2	HERC5	5
EPRS	2	HNRNPD	5
EPSTI1	2	HOPX	5
EPYC	2	HORMAD2	5
ERGIC2	2	HPSE	5

ERI1	2	HRAS	5
ESD	2	HSCB	5
ETNK2	2	HSF2BP	5
ETV3	2	HSP90AA4P	5
EXOSC8	2	ICMT	5
EYS	2	IFI6	5
F5	2	IFNAR1	5
FAM124A	2	IFNGR2	5
FAM135B	2	IFT74	5
FAM173B	2	IGFBP7	5
FAM189A2	2	IL17RC	5
FAM71A	2	INCA1	5
FAM82A1	2	INPP5K	5
FAM82B	2	IRAK2	5
FAM83B	2	IRF2	5
FAM98B	2	ISX	5
FANCF	2	ITGAE	5
FANCL	2	ITIH1	5
FAR2	2	ITIH4	5
FARP1	2	ITSN1	5
FBXO25	2	JPH3	5
FCER1A	2	KANK1	5
FCRL1	2	KCNAB3	5
FCRL2	2	KCTD11	5
FCRL3	2	KDM4C	5
FCRL5	2	KDM6B	5
FERMT1	2	KIAA0182	5
FEZ2	2	KIAA0753	5
FGF12	2	KIAA0922	5
FGF9	2	KIAA1211	5
FGL2	2	KIAA1324	5
FKBP9	2	KIAA1984	5
FKBP9L	2	KIF1C	5
FKTN	2	KIF9	5
FLJ42280	2	KLB	5
FLT1	2	KLHDC4	5
FLT3	2	KLHL17	5
FLVCR1	2	KLHL21	5
FMN1	2	KRTAP10-2	5
FMO9P	2	KRTAP10-3	5
FNDC3A	2	LARGE	5
FNDC3B	2	LARP1B	5
FNTA	2	LARP7	5
FOXE1	2	LCN10	5
FREM2	2	LCN6	5
FRK	2	LDLRAP1	5
FSD1L	2	LGALS9C	5

FSHB	2	LGI2	5
FSHR	2	LHFPL4	5
FXN	2	LIAS	5
GABBR2	2	LIF	5
GADL1	2	LINC00304	5
GALNT12	2	LMBR1L	5
GALNT13	2	LOC283922	5
GALNT5	2	LOC284276	5
GAS2	2	LOC400558	5
GATAD2B	2	LOC401052	5
GATSL2	2	LOC653501	5
GBAS	2	LRIG2	5
GDAP1	2	LRP5L	5
GDF7	2	LRRC19	5
GDPGP1	2	LRRC56	5
GINS4	2	LSP1	5
GJA3	2	MAN1C1	5
GJA5	2	MAP1LC3B	5
GJB2	2	MAP2K4	5
GJB6	2	MAP3K6	5
GKAP1	2	MAPK8IP2	5
GLI3	2	MAPKAPK3	5
GLT25D2	2	ME2	5
GOLGA7	2	MED18	5
GOLM1	2	MED31	5
GOLT1A	2	MEX3C	5
GORAB	2	MGC23284	5
GPATCH2	2	MGC39372	5
GPR110	2	MGST2	5
GPR12	2	MIR210	5
GPR149	2	MLL2	5
GPR15	2	MMP11	5
GPR176	2	MND1	5
GPR18	2	MOCOS	5
GPR183	2	MOV10	5
GPR25	2	MPG	5
GPR89B	2	MPRIP	5
GRB10	2	MRAP	5
GREM1	2	MRPL23	5
GRINA	2	MTMR14	5
GRK1	2	MTNR1A	5
GRM1	2	MUC2	5
GRM5	2	MUC5B	5
GRM8	2	MUC6	5
GRTP1	2	MUSTN1	5
GTDC1	2	MVD	5
GTF2E1	2	MYH10	5

GUCY1B2	2	MYH13	5
GUSBP3	2	MYH3	5
GYPE	2	MYO1C	5
HEATR1	2	N4BP2	5
HEMGN	2	NAAA	5
HGD	2	NCOR1	5
HIST1H2BJ	2	NDEL1	5
HIST1H4I	2	NDUFC1	5
HIVEP1	2	NEURL4	5
HMG20A	2	NEUROG2	5
HMGB1	2	NLGN2	5
HNF4G	2	NLRP1	5
HOOK3	2	NMU	5
HOXA1	2	NOL9	5
HOXA10	2	NQO2	5
HOXA11	2	NR0B2	5
HOXA13	2	NTN1	5
HOXA2	2	NUDC	5
HOXA3	2	NUDT5	5
HOXA4	2	OGG1	5
HOXA5	2	OPRD1	5
HOXA6	2	OR11H12	5
HOXA7	2	OR1F2P	5
HOXA9	2	OR4C6	5
HRASLS	2	OR4P4	5
HSD17B7	2	OR7A17	5
HTR2A	2	OR7A5	5
HUS1	2	OSM	5
HYALP1	2	P2RX1	5
IARS2	2	P2RX5	5
ID2	2	PAFAH1B1	5
ID4	2	PAFAH2	5
IFT80	2	PAQR7	5
IFT88	2	PCBP3	5
IGSF11	2	PDGFRA	5
IKBKB	2	PDIK1L	5
IL15	2	PDS5A	5
IL17D	2	PFN1	5
IL1R1	2	PHACTR4	5
IL1R2	2	PHF13	5
IL1RL2	2	PHF17	5
IL36A	2	PHF23	5
IL36B	2	PHRF1	5
IL6	2	PI4KAP1	5
ILDR2	2	PIGL	5
IMPG2	2	PIGV	5
ING1	2	PIGY	5

ING3	2	PIK3R5	5
INHBA	2	PIK3R6	5
INPP4B	2	PIRT	5
INTS3	2	PITPNA	5
INTS4L2	2	PITPNM3	5
INTS6	2	PIWIL3	5
IQCJ	2	PLAA	5
ITM2B	2	PLAC8	5
IVNS1ABP	2	PLSCR3	5
JARID2	2	PNPLA2	5
JAZF1	2	POLR2A	5
JPH1	2	POLR2J4	5
JTB	2	POLR2L	5
KBTBD6	2	POLR3K	5
KCNA4	2	POM121L10P	5
KCNAB1	2	POTEM	5
KCND2	2	PPAP2C	5
KCNE3	2	PPAPDC2	5
KCNH1	2	PPAT	5
KCNJ3	2	PPEF2	5
KCNK1	2	PPM1J	5
KCNK9	2	PPP1R8	5
KCNS3	2	PRAMEF10	5
KCTD8	2	PRAMEF13	5
KDELC1	2	PRAMEF14	5
KERA	2	PRAMEF15	5
KIAA0226L	2	PRAMEF20	5
KIAA1462	2	PRAMEF7	5
KIAA1524	2	PRAMEF8	5
KIAA1804	2	PRKAG1	5
KIAA1875	2	PRMT10	5
KIDINS220	2	PRPF8	5
KIF14	2	PRRT3	5
KIF18A	2	PSIP1	5
KIFAP3	2	PTAFR	5
KITLG	2	PTDSS2	5
KLF12	2	PTPN13	5
KLF5	2	RAB33B	5
KLHDC8A	2	RAPGEF2	5
KLHL1	2	RBFOX2	5
KLHL20	2	RBX1	5
KLRC1	2	RCC1	5
KLRC2	2	RCHY1	5
KSR1	2	RCL1	5
KYNU	2	RCVRN	5
LAMA2	2	RFC1	5
LCP1	2	RGL4	5

LECT1	2	RHBDF1	5
LEMD1	2	RHOC	5
LEPREL1	2	RHOD	5
LGALS8	2	RHOH	5
LHFP	2	RILP	5
LIN7A	2	RIMBP3	5
LINC00346	2	RIOK1	5
LINC00467	2	RIPK1	5
LINC00474	2	RNASEK	5
LINC00597	2	RND1	5
LINC00654	2	RNF125	5
LMBRD1	2	RNF138	5
LMO3	2	RNF167	5
LMO7	2	RNF207	5
LMX1A	2	RNF222	5
LNX2	2	RNH1	5
LOC100130331	2	RPA2	5
LOC148696	2	RPH3AL	5
LOC150568	2	RPL13	5
LOC150622	2	RPL26	5
LOC284395	2	RPS3A	5
LOC285696	2	RPS6KA1	5
LOC286359	2	RPUSD3	5
LOC339788	2	RREB1	5
LOC392364	2	RTN4R	5
LOC400794	2	SCARF1	5
LOC400940	2	SCD5	5
LOC441204	2	SCO1	5
LOC641515	2	SCT	5
LOC727924	2	SDAD1	5
LOC728190	2	SEC61A2	5
LOC728323	2	SENP3	5
LOC728989	2	SEPN1	5
LOH12CR1	2	SERPINB6	5
LPAR6	2	SERPINF1	5
LPCAT1	2	SERPINF2	5
LPGAT1	2	SESN2	5
LPP	2	SETD7	5
LRCH1	2	SF3A1	5
LRFN5	2	SFN	5
LRIG3	2	SGSM1	5
LRRC34	2	SGSM3	5
LRRC3B	2	SH3D19	5
LRRC52	2	SH3RF1	5
LRRC66	2	SHBG	5
LRRIQ1	2	SHPK	5
LRRK1	2	SLC13A5	5

LRRN2	2	SLC16A1	5
LRRN3	2	SLC16A11	5
LRRTM4	2	SLC16A13	5
LSM6	2	SLC1A1	5
LUM	2	SLC1A6	5
LUZP2	2	SLC25A17	5
LYSMD4	2	SLC25A22	5
MAB21L1	2	SLC2A11	5
MAEL	2	SLC2A4	5
MAGI2	2	SLC30A2	5
MAP2	2	SLC43A2	5
MB21D2	2	SLC7A5	5
MBOAT2	2	SLC9A1	5
MCF2L	2	SMAD4	5
MCM8	2	SMAD7	5
MCPH1	2	SMARCB1	5
MDH1	2	SMN1	5
MED12L	2	SMN2	5
MEF2A	2	SMPDL3B	5
MEIS1	2	SNAI3	5
MEIS2	2	SNAPC3	5
METTL21C	2	SNHG12	5
METTL25	2	SNHG3	5
MGAT4C	2	SNORD99	5
MGC57346	2	SNRNP25	5
MGP	2	SNRPD3	5
MGST1	2	SON	5
MIPEP	2	SORBS2	5
MIR137	2	SORCS2	5
MIR320C1	2	SOX15	5
MME	2	SPAG11B	5
MMP1	2	SPAG7	5
MMP10	2	SPARCL1	5
MMP12	2	SPATA2L	5
MMP2	2	SPECC1L	5
MMP20	2	SPG7	5
MMP27	2	SPIRE2	5
MMP3	2	SRD5A3	5
MMP7	2	SRP72	5
MMP8	2	SRRD	5
MNDA	2	SSR1	5
MOXD1	2	ST13	5
MPPED2	2	ST7L	5
MRPL50	2	STK32B	5
MRPS17	2	STMN1	5
MSANTD3	2	STX12	5
MSC	2	STX8	5

MSR1	2	SULT4A1	5
MTMR10	2	SYT12	5
MTMR6	2	SYT8	5
MTR	2	SYTL1	5
MTRF1	2	TAF12	5
MTUS2	2	TALDO1	5
MUC15	2	TAS1R1	5
MYCBP2	2	TATDN2	5
MYO10	2	TAX1BP3	5
NAA16	2	TBC1D10A	5
NABP1	2	TBC1D22A	5
NALCN	2	TCF25	5
NBAS	2	TEK	5
NCAM1	2	TFF1	5
NCBP1	2	TFF2	5
NCOA5	2	TFF3	5
NCOA7	2	TFIP11	5
NDUFAF6	2	THAP3	5
NEDD9	2	THAP6	5
NEK11	2	TLR3	5
NEK2	2	TMEM141	5
NEK3	2	TMEM165	5
NELL2	2	TMEM167B	5
NEUROD6	2	TMEM184C	5
NFASC	2	TMEM192	5
NGRN	2	TMEM211	5
NHLRC3	2	TMEM222	5
NKAIN2	2	TMEM50B	5
NKAIN3	2	TMEM57	5
NKX2-4	2	TMEM80	5
NMBR	2	TMEM95	5
NMRK1	2	TMPPE	5
NPAS3	2	TNFSF12	5
NPBWR2	2	TNFSF12-TNFSF1	5
NPHP3	2	TNFSF13	5
NPR1	2	TNNI2	5
NPSR1	2	TNNT3	5
NR4A3	2	TNRC6B	5
NR5A2	2	TOLLIP	5
NRP1	2	TOP3B	5
NRXN1	2	TOPORS	5
NSL1	2	TP53	5
NTF3	2	TPST2	5
NTRK2	2	TRAF2	5
NTRK3	2	TRIM2	5
NTS	2	TRIM60	5
NUAK2	2	TRIM63	5

NUDT16	2	TRIM71	5
NUDT16P1	2	TRMT2A	5
NUFIP1	2	TRNAU1AP	5
NUPL1	2	TRNP1	5
NXNL2	2	TRPV1	5
OLFML2B	2	TRPV2	5
OPA1	2	TRPV3	5
OPCML	2	TSPAN4	5
OR10K2	2	TTC19	5
OR10T2	2	TTC3	5
OR13C2	2	TTC39B	5
OR13C4	2	TTLL3	5
OR13C5	2	TUBB2A	5
OR13C9	2	TUBB2B	5
OR13D1	2	TUSC5	5
OR13F1	2	TXNDC17	5
OR2M4	2	UBAP2	5
OR51A2	2	UBB	5
OR51A4	2	UBE2G2	5
OR51L1	2	UBE2K	5
OR52E2	2	UGDH	5
OR52J3	2	UHRF2	5
OR5K2	2	UPB1	5
OR5K4	2	UPF2	5
OR8D2	2	USO1	5
OR8D4	2	USP43	5
OR8G1	2	VHL	5
OR8G2	2	VPS53	5
OR8G5	2	VSTM1	5
OSGIN2	2	WASF2	5
OSTF1	2	WDR16	5
OSTN	2	WDR19	5
OTX1	2	WDR81	5
OVCH1	2	WDTC1	5
PABPC1P2	2	WNT1	5
PABPC3	2	WNT10B	5
PALM2-AKAP2	2	WRAP53	5
PAN3	2	WSCD1	5
PAPPA2	2	WWOX	5
PARD3	2	XAF1	5
PBX1	2	XKR8	5
PCCA	2	XPNPEP3	5
PCDH15	2	YBX2	5
PCDH17	2	YTHDF2	5
PCDH18	2	YWHAH	5
PCDH9	2	ZBTB4	5
PCID2	2	ZBTB48	5

PCSK5	2	ZC3H18	5
PCSK6	2	ZCCHC14	5
PDC	2	ZDHHC18	5
PDE11A	2	ZDHHC8	5
PDE1C	2	ZFPM1	5
PDE6H	2	ZNF121	5
PDGFC	2	ZNF18	5
PDX1	2	ZNF276	5
PDZK1	2	ZNF287	5
PEAK1	2	ZNF426	5
PEG10	2	ZNF469	5
PEX5L	2	ZNF490	5
PHF14	2	ZNF559	5
PHTF2	2	ZNF561	5
PHYHIPL	2	ZNF562	5
PI15	2	ZNF658B	5
PIGT	2	ZNF683	5
PIK3C2G	2	ZNF70	5
PIK3R4	2	ZNF778	5
PIP5K1B	2	ZNF846	5
PKD1L1	2	ZSWIM7	5
PLAT	2	ABCD4	4
PLCH1	2	ABHD14B	4
PLCZ1	2	ABHD6	4
PLEKHA6	2	ABL1	4
PLEKHA8	2	ACAP3	4
PLEKHF2	2	ACOT4	4
PLK1S1	2	ACP5	4
PM20D1	2	ACPT	4
PMS2L2	2	ACTR1A	4
POLR1D	2	ACY1	4
POLR3F	2	ADAD2	4
POMP	2	ADAMTS2	4
PON1	2	ADAMTSL1	4
PON2	2	ADARB1	4
POSTN	2	ADCY6	4
PPFIA2	2	ADCY7	4
PPP1R1B	2	ADORA2B	4
PPP1R3A	2	AGPAT2	4
PPP1R9A	2	AGPAT6	4
PPP2R5A	2	AGRN	4
PRH1	2	AIF1L	4
PRKG1	2	AJUBA	4
PRL	2	AKT1S1	4
PRMT2	2	AKT2	4
PRNT	2	ALDH16A1	4
PRR4	2	ALDH6A1	4

PRRX1	2	ALKBH5	4
PTPN12	2	ALOX15	4
PTPN14	2	ALPK2	4
PTPN3	2	AMHR2	4
PTPRO	2	AMPD1	4
PTPRQ	2	AMPD2	4
RAB13	2	ANGPT2	4
RAB20	2	ANKFY1	4
RAB38	2	ANKLE2	4
RAB3GAP2	2	ANKRD34B	4
RAB7L1	2	AP1G1	4
RABGAP1L	2	AP2A1	4
RABIF	2	AP3M2	4
RABL3	2	APBB1	4
RALYL	2	AQP3	4
RAPGEF5	2	AQP7	4
RASA3	2	ARF4	4
RASAL2	2	ARFIP2	4
RASGRP1	2	ARHGAP44	4
RASSF9	2	ARHGEF39	4
RBBP5	2	ARL3	4
RBMS3	2	ARRB2	4
RCBTB1	2	ART3	4
RCBTB2	2	AS3MT	4
RCN2	2	ASB6	4
RDH10	2	ASPA	4
RERG	2	ASPHD2	4
RERGL	2	ASS1	4
REXO2	2	ATAD3A	4
RFXAP	2	ATAD3B	4
RGCC	2	ATP2A1	4
RGL1	2	ATP2C2	4
RGMA	2	ATP8B1	4
RHOQ	2	ATP9B	4
RIMS1	2	ATPAF2	4
RNASEH2B	2	ATXN1L	4
RNF144A	2	ATXN2L	4
RNF144B	2	B3GNT9	4
RNF146	2	B4GALT1	4
RNF170	2	BAG1	4
RNF217	2	BAI2	4
RORB	2	BAX	4
RPS27	2	BCAS2	4
RRP15	2	BCAT2	4
RSBN1L	2	BCL2L12	4
RSPH10B	2	BCR	4
RSPH10B2	2	BEND3	4

RSPO4	2	BEND7	4
RUNDC3B	2	BLOC1S5	4
RXRG	2	BLVRB	4
RYK	2	BMP1	4
RYR2	2	BRSK2	4
RYR3	2	BSDC1	4
S100A14	2	C10orf111	4
S100A16	2	C10orf114	4
S100A2	2	C11orf42	4
S100A3	2	C12orf10	4
S100A4	2	C14orf43	4
SACS	2	C14orf45	4
SCEL	2	C16orf70	4
SCG5	2	C18orf25	4
SCHIP1	2	C19orf47	4
SCN1A	2	C19orf48	4
SCN7A	2	C1QBP	4
SCN9A	2	C1QL4	4
SCYL3	2	C1orf159	4
SDC2	2	C1orf170	4
SDC4	2	C21orf59	4
SEC16B	2	C21orf62	4
SELP	2	C22orf28	4
SEMA4D	2	C22orf42	4
SEMG1	2	C3orf74	4
SERPINI2	2	C4orf27	4
SERTM1	2	C4orf45	4
SFRP1	2	C5orf45	4
SFRP4	2	C6orf201	4
SGCE	2	C6orf203	4
SGCG	2	C9orf117	4
SGCZ	2	C9orf16	4
SHARPIN	2	C9orf169	4
SHFM1	2	C9orf37	4
SI	2	C9orf78	4
SIAH3	2	CA11	4
SKAP2	2	CA9	4
SLAIN1	2	CALHM2	4
SLC12A6	2	CAMTA1	4
SLC12A7	2	CAND2	4
SLC13A3	2	CANX	4
SLC15A5	2	CASC3	4
SLC16A7	2	CBFB	4
SLC17A6	2	CCBE1	4
SLC19A2	2	CCDC107	4
SLC20A2	2	CCDC114	4
SLC24A5	2	CCDC155	4

SLC25A13	2	CCDC17	4
SLC25A15	2	CCDC28B	4
SLC27A3	2	CCDC3	4
SLC28A3	2	CCL3	4
SLC30A1	2	CCL4	4
SLC35F3	2	CCNL2	4
SLC39A1	2	CCNT1	4
SLC44A1	2	CD19	4
SLC45A3	2	CD274	4
SLC46A3	2	CD72	4
SLC5A12	2	CD81	4
SLC5A7	2	CDH16	4
SLC6A12	2	CDH5	4
SLC6A15	2	CDK11B	4
SLC6A19	2	CDKN2A	4
SLC6A2	2	CDKN2B	4
SLC6A3	2	CDRT4	4
SLC7A1	2	CDYL	4
SLC7A14	2	CERCAM	4
SLCO1B3	2	CES2	4
SLCO1C1	2	CES3	4
SLCO3A1	2	CGB	4
SLITRK3	2	CGB1	4
SLPI	2	CGB5	4
SMC4	2	CGB7	4
SMC6	2	CGB8	4
SNORA15	2	CHMP4A	4
SNORA27	2	CHMP5	4
SNORD74	2	CHP1	4
SNRPA1	2	CHST4	4
SNX16	2	CIDEB	4
SNX5	2	CIZ1	4
SOCS5	2	CLCC1	4
SOHLH2	2	CLCN3	4
SOX11	2	CLEC11A	4
SOX6	2	CLEC18B	4
SP4	2	CLEC3A	4
SPAG11B	2	CLIP2	4
SPAM1	2	CLN8	4
SPATA13	2	CLTA	4
SPATA17	2	CMIP	4
SPATA18	2	CMTM3	4
SPATC1	2	CMTM4	4
SPG20	2	CNEP1R1	4
SPIN1	2	CNGA4	4
SPRED1	2	CNNM2	4
SPTBN1	2	CNOT3	4

SSTR1	2	CNPY3	4
ST6GAL2	2	CNTLN	4
ST8SIA2	2	COL16A1	4
STARD3NL	2	COQ4	4
STAU2	2	COQ5	4
STK24	2	COQ6	4
STK3	2	COTL1	4
STK35	2	COX14	4
STOML3	2	CPNE6	4
STX7	2	CPSF3L	4
SUGT1	2	CPT1C	4
SUGT1P3	2	CREB3	4
SULT1C2	2	CRISPLD2	4
SULT1C4	2	CROCC	4
SUMF2	2	CRYBA4	4
SUSD4	2	CRYBB1	4
SV2B	2	CSAD	4
SYS1	2	CSF1	4
SYT2	2	CTDP1	4
TAAR5	2	CTIF	4
TAAR6	2	CTNNA1	4
TAAR8	2	CTRL	4
TAAR9	2	CTSD	4
TAF1A	2	CUBN	4
TARBP1	2	CUEDC2	4
TARP	2	CUL7	4
TAS2R10	2	CWH43	4
TAS2R13	2	CXCL10	4
TAS2R14	2	CXCL16	4
TAS2R19	2	CXXC5	4
TAS2R20	2	CYB561D1	4
TAS2R31	2	CYB5D2	4
TAS2R46	2	CYB5RL	4
TAS2R50	2	CYP17A1	4
TAS2R7	2	CYP4F11	4
TAS2R8	2	CYP4F2	4
TAS2R9	2	CYTH2	4
TBC1D3C	2	DBN1	4
TBCEL	2	DBP	4
TCEA2	2	DCAF11	4
TCEB1	2	DCAF12	4
TDRD5	2	DCAF16	4
TDRD7	2	DCAF4	4
TECTA	2	DCDC2B	4
TERC	2	DCP2	4
TES	2	DDIT4	4
TEX10	2	DDX23	4

TFAP2C	2	DDX28	4
TFDP1	2	DDX4	4
TFEC	2	DDX41	4
TGFB2	2	DEF8	4
TGFBR2	2	DEFB134	4
THBS1	2	DENND2C	4
THUMPD2	2	DENND5B	4
TJP2	2	DHDH	4
TLE1	2	DHRS1	4
TLE4	2	DHX33	4
TLR5	2	DIP2B	4
TM2D3	2	DKK4	4
TMCO3	2	DLGAP2	4
TMEFF1	2	DMP1	4
TMEM133	2	DMRT2	4
TMEM183A	2	DMRTA1	4
TMEM245	2	DNAH12	4
TMEM246	2	DNAH9	4
TMEM247	2	DNAJB6	4
TMEM38B	2	DNAJC22	4
TMTC1	2	DNASE1L3	4
TMTC3	2	DNHD1	4
TMTC4	2	DNM1	4
TNC	2	DOCK8	4
TNFRSF19	2	DPEP2	4
TNFRSF21	2	DPH1	4
TNFSF13B	2	DRG2	4
TNFSF15	2	DSP	4
TNR	2	DTX2	4
TNS3	2	DTX2P1-UPK3BP	4
TOX	2	DUS2L	4
TOX2	2	DUSP26	4
TP53AIP1	2	DUSP7	4
TP53RK	2	DUSP8	4
TP53TG1	2	DVL1	4
TPD52L1	2	DYM	4
TPP2	2	DYNC1LI1	4
TRAF5	2	E2F4	4
TRDN	2	EAPP	4
TRHDE	2	ECH1	4
TRIM55	2	EDNRA	4
TRIP13	2	EIF3I	4
TRMT11	2	EIF4B	4
TRPA1	2	EIF4H	4
TRPC4	2	ELAC2	4
TRPC6	2	ELOVL3	4
TRPM3	2	ELP2	4

TRPM6	2	EMC10	4
TSC22D1	2	EMID2	4
TSC22D2	2	EMR1	4
TSEN15	2	EMR4P	4
TSPAN12	2	ENAM	4
TSPAN13	2	ENG	4
TSPAN3	2	ENTHD1	4
TSPAN8	2	ENTPD5	4
TSPYL4	2	ENTPD8	4
TTC21B	2	EPB49	4
TTLL13	2	EPG5	4
TUBB6	2	EPM2AIP1	4
TUBGCP3	2	EPS8L3	4
TYR	2	ERI1	4
UBL3	2	ERMP1	4
UCHL5	2	ESPL1	4
UFM1	2	ESRP2	4
UGP2	2	EXD1	4
UGT2A3	2	EXD3	4
UGT2B10	2	EXOC1	4
UGT2B11	2	EXOSC2	4
UGT2B15	2	F2R	4
UGT2B7	2	F2RL1	4
UPP1	2	FABP6	4
USP12	2	FAM102A	4
USP46	2	FAM107B	4
USPL1	2	FAM116A	4
UTS2D	2	FAM129B	4
VIMP	2	FAM132A	4
VOPP1	2	FAM151B	4
VPS54	2	FAM160A2	4
VRK2	2	FAM160B2	4
VSNL1	2	FAM161B	4
VTA1	2	FAM166B	4
VWA5A	2	FAM167B	4
VWC2	2	FAM171A1	4
WDR35	2	FAM186A	4
WFDC10A	2	FAM189A1	4
WFDC10B	2	FAM217A	4
WFDC12	2	FAM71E1	4
WFDC2	2	FAM73A	4
WFDC5	2	FAM73B	4
WFDC6	2	FAM83E	4
WFDC8	2	FAM86B1	4
WNT5B	2	FAM8A1	4
XIRP2	2	FAM99A	4
XPA	2	FAT1	4

XPO4	2	FBXL15	4
XRN2	2	FBXL18	4
ZBBX	2	FBXL8	4
ZC3H13	2	FBXO27	4
ZCCHC6	2	FBXO7	4
ZEB1	2	FCGBP	4
ZEB2	2	FCGRT	4
ZFP161	2	FECH	4
ZFP64	2	FFAR3	4
ZFPM2	2	FGF17	4
ZFR	2	FGF21	4
ZMAT4	2	FGFR4	4
ZNF189	2	FHOD1	4
ZNF281	2	FIBCD1	4
ZNF438	2	FITM1	4
ZNF710	2	FLII	4
ZNF713	2	FLJ25758	4
ZNF770	2	FLNB	4
ZNF774	2	FLT3LG	4
ZP4	2	FNBP1	4
ZPBP	2	FNIP2	4
A2M	1	FOXD3	4
AAED1	1	FOXK1	4
AAGAB	1	FOXL1	4
AASDH	1	FPGS	4
AASS	1	FREM1	4
ABCA12	1	FRMD4A	4
ABCA6	1	FSCN1	4
ABCA8	1	FTL	4
ABCC1	1	FUBP3	4
ABCC3	1	FUT1	4
ABCC6	1	FUT10	4
ABCE1	1	FUT2	4
ABHD15	1	GATA4	4
ABHD2	1	GATSL1	4
ABI1	1	GATSL2	4
ABL1	1	GBA2	4
ABL2	1	GBF1	4
ABTB1	1	GCNT2	4
ACAA2	1	GFRA2	4
ACAD8	1	GGT6	4
ACAN	1	GID4	4
ACBD5	1	GINS4	4
ACBD6	1	GJA10	4
ACCSL	1	GK3P	4
ACE	1	GLE1	4
ACHE	1	GLIPR2	4

ACOXL	1	GLTPD1	4
ACSF2	1	GMPR2	4
ACSL5	1	GNAI3	4
ACTL6B	1	GNAT2	4
ADAM10	1	GNAZ	4
ADAM11	1	GNE	4
ADAM15	1	GOLGA2	4
ADAM23	1	GOLGA7	4
ADAM32	1	GOLGA8F	4
ADAM7	1	GOLGA8G	4
ADAM9	1	GPD1	4
ADAMTS20	1	GPR107	4
ADAMTS4	1	GRIN2D	4
ADAMTS8	1	GRM6	4
ADAMTSL4	1	GRWD1	4
ADCYAP1R1	1	GSDMA	4
ADHFE1	1	GSDMB	4
ADIPOR2	1	GSTM2	4
ADNP	1	GSTM3	4
ADPGK	1	GTF2IRD2	4
AEN	1	GTF2IRD2B	4
AFF3	1	GYS1	4
AGFG2	1	HADH	4
AGPS	1	HAUS4	4
AHCTF1	1	HBQ1	4
AHI1	1	HDAC1	4
AKAP2	1	HEATR3	4
AKAP3	1	HEATR4	4
AKAP6	1	HIC1	4
AKAP7	1	HINT1	4
AKR1A1	1	HIPK4	4
AKR1C4	1	HNRNPH1	4
AKT3	1	HNRNPL	4
ALDH1A2	1	HOMER1	4
ALDH4A1	1	HP	4
ALDH9A1	1	HPS4	4
ALG1L	1	HPS6	4
ALKBH3	1	HPX	4
ALLC	1	HR	4
AMFR	1	HS3ST3B1	4
AMOTL1	1	HSD17B1	4
AMOTL2	1	HSD17B14	4
AMPD3	1	HSDL1	4
AMZ2P1	1	HSPA14	4
ANAPC10	1	IFNAR2	4
ANG	1	IGF2	4
ANGPTL2	1	IGJ	4

ANGPTL3	1	IKBKB	4
ANGPTL5	1	IL10RB	4
ANKRD13A	1	IL31RA	4
ANKRD13B	1	IL4I1	4
ANKRD16	1	IL6ST	4
ANKRD32	1	INADL	4
ANKRD34C	1	INS-IGF2	4
ANKRD35	1	INTS1	4
ANKRD40	1	IPO4	4
ANKRD46	1	IRF3	4
ANKRD52	1	IRF8	4
ANO4	1	IRF9	4
ANP32E	1	ITGA8	4
ANPEP	1	ITGB7	4
ANTXR1	1	ITPKA	4
ANTXR2	1	JMY	4
ANXA4	1	JOSD2	4
ANXA5	1	KANK4	4
ANXA8L2	1	KATNAL2	4
AOAH	1	KCNA7	4
AP1S1	1	KCNAB2	4
AP4E1	1	KCNC3	4
AP5Z1	1	KCNG2	4
APBB1IP	1	KCNG4	4
APLF	1	KCNJ14	4
APOA1BP	1	KCTD19	4
APOH	1	KDELR1	4
AQP10	1	KDR	4
AQP4	1	KHDRBS1	4
AQP9	1	KIAA0391	4
ARAP2	1	KIAA0513	4
ARF5	1	KIAA1432	4
ARFGAP1	1	KIAA2026	4
ARFGEF1	1	KIF24	4
ARFIP1	1	KLC4	4
ARG1	1	KLHL2	4
ARHGAP10	1	KLHL36	4
ARHGAP11B	1	KLK1	4
ARHGAP18	1	KLK10	4
ARHGAP21	1	KLK11	4
ARHGAP28	1	KLK12	4
ARHGAP30	1	KLK13	4
ARHGAP40	1	KLK3	4
ARHGAP5	1	KLK4	4
ARHGAP5-AS1	1	KLK6	4
ARHGEF12	1	KLK7	4
ARHGEF17	1	KLK8	4

ARHGEF38	1	KLK9	4
ARHGEF40	1	KPNA6	4
ARID1B	1	KRTAP5-3	4
ARID4B	1	KRTAP5-6	4
ARL13B	1	L1TD1	4
ARL9	1	LAMC3	4
ARMC1	1	LAPTM5	4
ARNT2	1	LARP4	4
ARNTL2	1	LAT	4
ARPC1B	1	LAT2	4
ARPC5L	1	LCAT	4
ARSG	1	LCK	4
ASAP2	1	LDB1	4
ASB15	1	LGALS4	4
ASB16	1	LGALS7	4
ASCL1	1	LGALS9B	4
ASH2L	1	LGI3	4
ASL	1	LHB	4
ASNSD1	1	LHX3	4
ASPN	1	LIMA1	4
ASXL1	1	LIN52	4
ASXL2	1	LIN7B	4
ATAD2B	1	LIPG	4
ATF7IP2	1	LLGL1	4
ATIC	1	LMAN2	4
ATP10A	1	LMTK3	4
ATP11B	1	LOC100289341	4
ATP2B1	1	LOC284009	4
ATP5A1	1	LOC338651	4
ATP6V1C1	1	LOC389705	4
ATP8B2	1	LOC541473	4
ATP8B4	1	LOC641367	4
ATP9A	1	LOC644145	4
ATRAID	1	LOXHD1	4
AUTS2	1	LRBA	4
AVL9	1	LRRC29	4
AVP	1	LRRC48	4
AZGP1	1	LRRC4B	4
AZI2	1	LRRC8A	4
B2M	1	LRRFIP2	4
B4GALNT2	1	LTB4R	4
B4GALT3	1	LTB4R2	4
B4GALT6	1	LTC4S	4
BAHD1	1	LTK	4
BAIAP2L1	1	LYRM7	4
BAMBI	1	LZTS1	4
BANK1	1	MAFK	4

BARD1	1	MAK	4
BARX2	1	MAK16	4
BAZ1A	1	MALT1	4
BAZ2A	1	MAML1	4
BBS12	1	MAML3	4
BCAP29	1	MAP3K12	4
BCL2L10	1	MAPK4	4
BCL2L11	1	MARCKSL1	4
BDNF	1	MATN1	4
BEND7	1	MBD3L2	4
BET3L	1	MBD3L3	4
BHLHA9	1	MBD3L5	4
BICC1	1	MBP	4
BICD1	1	MBTPS1	4
BLM	1	MCM10	4
BLOC1S1	1	MCPH1	4
BLOC1S5	1	MECR	4
BLVRA	1	MED11	4
BMF	1	MED20	4
BMI1	1	MED24	4
BMP10	1	MED25	4
BMP6	1	MED9	4
BNIPL	1	MEIG1	4
BOP1	1	MELK	4
BORA	1	MEP1B	4
BPHL	1	METTL16	4
BRE	1	METTL20	4
BRF2	1	MFAP3L	4
BRI3	1	MFHAS1	4
BTG4	1	MFSD5	4
BUB1	1	MGA	4
C10orf10	1	MGAT4B	4
C10orf111	1	MIAT	4
C10orf114	1	MIB2	4
C10orf25	1	MICALL2	4
C11orf53	1	MINK1	4
C11orf74	1	MIR150	4
C11orf96	1	MIR197	4
C12orf4	1	MIR200B	4
C12orf42	1	MIR212	4
C12orf5	1	MLANA	4
C12orf60	1	MLH1	4
C12orf70	1	MNT	4
C15orf27	1	MRO	4
C15orf52	1	MRPL2	4
C16orf72	1	MRPL20	4
C16orf78	1	MRPL41	4

C17orf103	1	MRPS12	4
C17orf47	1	MSMP	4
C17orf53	1	MTAP	4
C17orf72	1	MX2	4
C18orf25	1	MXRA8	4
C19orf25	1	MYBBP1A	4
C1D	1	MYBPC2	4
C1orf106	1	MYH1	4
C1orf192	1	MYH14	4
C1orf43	1	MYH2	4
C1orf56	1	MYH8	4
C1orf85	1	MYL10	4
C20orf112	1	MYLK4	4
C20orf141	1	MYO15A	4
C20orf151	1	MYO18B	4
C20orf194	1	MYOCD	4
C20orf96	1	NACC2	4
C22orf24	1	NADK	4
C2orf16	1	NAIF1	4
C2orf47	1	NARS	4
C2orf53	1	NCCRP1	4
C2orf88	1	NCF1	4
C3orf22	1	NCF1C	4
C3orf80	1	NDUFA3	4
C4orf19	1	NDUFAF1	4
C4orf29	1	NDUFB6	4
C4orf3	1	NDUFB8	4
C4orf40	1	NECAB2	4
C5	1	NEDD4L	4
C5orf27	1	NEDD8	4
C5orf30	1	NEK1	4
C6orf118	1	NEURL	4
C6orf123	1	NFATC1	4
C6orf170	1	NFATC2IP	4
C6orf201	1	NFATC3	4
C7orf10	1	NFKB2	4
C7orf26	1	NFX1	4
C8A	1	NKAIN1	4
C8orf31	1	NMT2	4
C8orf34	1	NOL6	4
C8orf44	1	NOLC1	4
C8orf47	1	NOSIP	4
C8orf56	1	NOTCH1	4
C8orf86	1	NR1D1	4
C9orf24	1	NT5C2	4
C9orf37	1	NTF4	4
C9orf57	1	NTMT1	4

C9orf84	1	NUCB1	4
CA10	1	NUDT2	4
CABLES1	1	NUDT7	4
CABYR	1	NUMB	4
CACNA1C	1	NUP188	4
CACNA1G	1	NUP214	4
CACNA2D4	1	NUP62	4
CACNG1	1	NUP88	4
CACNG4	1	NUSAP1	4
CAD	1	OCIAD2	4
CADM2	1	ODF2	4
CADPS2	1	OLAH	4
CAGE1	1	OLIG2	4
CALML5	1	OPTN	4
CAMK1D	1	OR10H1	4
CAMK2D	1	OR10H2	4
CAP2	1	OR10H3	4
CAPN13	1	OR10H4	4
CAPN8	1	OR1A2	4
CARD14	1	OR1D2	4
CARHSP1	1	OR1D5	4
CASC4	1	OR1E1	4
CASP14	1	OR1E2	4
CASR	1	OR3A1	4
CAST	1	OR3A2	4
CBX1	1	OR4M2	4
CBX3	1	OR4N4	4
CCDC140	1	ORAI1	4
CCDC15	1	ORMDL3	4
CCDC17	1	OSGIN1	4
CCDC3	1	PAK4	4
CCDC34	1	PAPD4	4
CCL24	1	PAPL	4
CCL3	1	PAPLN	4
CCL4	1	PAX5	4
CCM2	1	PCBP4	4
CCNB1IP1	1	PDCD1LG2	4
CCNB2	1	PDE12	4
CCNG2	1	PDIA4	4
CCNH	1	PDLIM3	4
CCNI	1	PDLIM7	4
CCPG1	1	PELP1	4
CCRN4L	1	PEMT	4
CCT3	1	PFDN5	4
CD109	1	PHF15	4
CD1A	1	PHLPP1	4
CD1B	1	PHLPP2	4

CD1C	1	PHYH	4
CD1E	1	PHYHD1	4
CD44	1	PHYHIP	4
CD59	1	PIAS2	4
CD5L	1	PIF1	4
CD63	1	PIH1D1	4
CD86	1	PITX3	4
CDC123	1	PKN3	4
CDC14B	1	PKP3	4
CDC25B	1	PLA2G15	4
CDC27	1	PLAT	4
CDC42SE1	1	PLD2	4
CDCA7	1	PLD3	4
CDH11	1	PLEKHA4	4
CDH13	1	PLEKHN1	4
CDH17	1	PLEKHO2	4
CDK12	1	PMAIP1	4
CDK2	1	PMFBP1	4
CDK5RAP3	1	PMPCA	4
CDYL	1	PMS2P5	4
CEBPB	1	PNMA1	4
CENPA	1	PNPLA7	4
CENPO	1	POLD1	4
CEP135	1	POLE	4
CEP57	1	POLR3D	4
CEP78	1	POM121L8P	4
CEP95	1	POMT1	4
CERS2	1	PPFIA3	4
CFL2	1	PPP1R15A	4
CFTR	1	PPP1R3G	4
CGN	1	PPRC1	4
CGNL1	1	PQLC1	4
CH25H	1	PRAMEF22	4
CHAC1	1	PRAMEF3	4
CHAD	1	PRKCDBP	4
CHD1	1	PRMT1	4
CHD7	1	PRMT5	4
CHEK1	1	PRMT7	4
CHIC2	1	PRPF18	4
CHML	1	PRPF31	4
CHMP1B	1	PRPH	4
CHMP2A	1	PRR12	4
CHMP4C	1	PRR13	4
CHN2	1	PRR7	4
CHRFAM7A	1	PRRG2	4
CHRM2	1	PRRX2	4
CHRM3	1	PRX	4

CHRNA3	1	PSD	4
CHRNA5	1	PSEN1	4
CHRNB4	1	PSG1	4
CITED2	1	PSG3	4
CKS1B	1	PSG7	4
CLDN11	1	PSKH1	4
CLDN15	1	PSMA5	4
CLEC12A	1	PSMB10	4
CLEC12B	1	PSMD3	4
CLEC16A	1	PSTPIP2	4
CLEC1B	1	PTCRA	4
CLEC2A	1	PTER	4
CLEC2B	1	PTGR2	4
CLEC2D	1	PTH2	4
CLEC9A	1	PTOV1	4
CLGN	1	PTP4A2	4
CLIP2	1	PTPRU	4
CLIP4	1	PUM1	4
CLK2	1	PXDC1	4
CLLU1	1	PXK	4
CLLU1OS	1	PXMP2	4
CLOCK	1	QSOX2	4
CLUL1	1	RAB11FIP1	4
CNOT6L	1	RAB36	4
CNPY2	1	RABEP1	4
CNPY4	1	RABEP2	4
CNST	1	RABGGTA	4
CNTN1	1	RACGAP1	4
CNTN6	1	RAD54L2	4
CNTNAP4	1	RANBP10	4
COG5	1	RANBP6	4
COG7	1	RAP1GDS1	4
COL12A1	1	RAPGEFL1	4
COLEC12	1	RARG	4
COMMD7	1	RASIP1	4
COMMD9	1	RBFA	4
COPA	1	RBM23	4
COPZ2	1	RBM25	4
CORO2A	1	RBPMS2	4
CORO6	1	RCN3	4
COX20	1	REC8	4
COX7A2	1	REEP4	4
COX7C	1	REEP5	4
CPA6	1	REM2	4
CPNE3	1	RFC2	4
CPO	1	RFPL2	4
CPSF1	1	RGP1	4

CPSF3	1	RGS14	4
CPVL	1	RILPL1	4
CPXM1	1	RILPL2	4
CRABP2	1	RIPK3	4
CRCP	1	RNF122	4
CREB3L1	1	RNF165	4
CRK	1	RNF208	4
CRTC3	1	RNF31	4
CRYGS	1	RNF38	4
CS	1	RPAP1	4
CSH1	1	RPL17	4
CSH2	1	RPL18	4
CSHL1	1	RPL32	4
CSN1S1	1	RPP38	4
CSN3	1	RPRD1A	4
CSTA	1	RPSA	4
CSTF3	1	RRAD	4
CTAGE5	1	RRAS	4
CTDSPL2	1	RRP36	4
CTGF	1	RSBN1	4
CTHRC1	1	RTDR1	4
CTIF	1	RTF1	4
CTNNBL1	1	RTN4RL1	4
CTR9	1	RUFY1	4
CTSK	1	RUFY3	4
CTSS	1	RUNX3	4
CTTNBP2	1	RUVBL2	4
CWF19L2	1	S100Z	4
CXADRP2	1	SAR1B	4
CXCL13	1	SARS2	4
CYFIP1	1	SCAF1	4
CYLD	1	SCNN1D	4
CYP1B1-AS1	1	SDC3	4
CYP26B1	1	SDF4	4
CYP2E1	1	SEC24A	4
CYP2R1	1	SEC31B	4
CYP3A5	1	SERF1A	4
CYP3A7	1	SERF1B	4
CYP7A1	1	SERINC5	4
CYTH3	1	SERPINB1	4
DAB1	1	SERTAD1	4
DAGLB	1	SET	4
DAP3	1	SETD1B	4
DAZAP1	1	SETD2	4
DBF4B	1	SETD5	4
DCAF13	1	SETD8	4
DCAKD	1	SEZ6L	4

DCBLD1	1	SFXN2	4
DCLK2	1	SGSM2	4
DCST1	1	SH2B1	4
DCST2	1	SH2B3	4
DDHD2	1	SH2D3C	4
DDRGK1	1	SH3PXD2A	4
DDX1	1	SH3PXD2B	4
DDX10	1	SHANK1	4
DDX25	1	SHISA6	4
DDX5	1	SHKBP1	4
DEFB134	1	SHMT1	4
DEK	1	SIGIRR	4
DENND3	1	SIGLEC16	4
DENND5B	1	SIRT2	4
DERA	1	SIT1	4
DESI2	1	SKIDA1	4
DEXI	1	SLC12A4	4
DGAT1	1	SLC17A7	4
DGKA	1	SLC18A1	4
DGKG	1	SLC22A23	4
DGKZ	1	SLC24A2	4
DHFRL1	1	SLC25A25	4
DHODH	1	SLC27A4	4
DHRS7B	1	SLC29A4	4
DHTKD1	1	SLC2A9	4
DHX9	1	SLC34A1	4
DIAPH3	1	SLC34A3	4
DIDO1	1	SLC35E2	4
DIO3OS	1	SLC37A1	4
DIRC2	1	SLC38A8	4
DIRC3	1	SLC38A9	4
DISP2	1	SLC5A1	4
DLEU2	1	SLC5A4	4
DLGAP1	1	SLC6A16	4
DLGAP1-AS1	1	SLC7A6	4
DLGAP2	1	SLC7A6OS	4
DLL4	1	SLC9A5	4
DLX4	1	SLMAP	4
DMXL2	1	SMARCD1	4
DNAH14	1	SMCR7	4
DNAH6	1	SMCR8	4
DNAJA4	1	SMG6	4
DNAJB11	1	SMPD1	4
DNAJB8	1	SMPD3	4
DNAJC1	1	SMTNL2	4
DNAJC14	1	SMYD4	4
DNAJC25	1	SNAPC4	4

DNAJC5	1	SNORA65	4
DNMT3A	1	SNORD103A	4
DOCK10	1	SNRNP40	4
DOCK7	1	SNRNP48	4
DRD3	1	SNRNP70	4
DSC1	1	SNX6	4
DSC2	1	SORT1	4
DSC3	1	SP1	4
DSG1	1	SP7	4
DSG2	1	SPACA4	4
DSG3	1	SPATA22	4
DSG4	1	SPHK2	4
DSP	1	SPIB	4
DTNA	1	SPINK4	4
DTX2	1	SPNS1	4
DTX2P1-UPK3B	1	SPNS2	4
DTX4	1	SPNS3	4
DUOX1	1	SPOCD1	4
DUOX2	1	SPP1	4
DUOXA1	1	SPRYD3	4
DUOXA2	1	SPTAN1	4
DUS4L	1	SPTBN4	4
DUSP12	1	SPZ1	4
DUSP4	1	SQSTM1	4
DYM	1	SREBF1	4
DYRK2	1	SRP19	4
DYRK4	1	SRR	4
DYSF	1	SSBP3	4
DYX1C1	1	SSNA1	4
E2F3	1	SSU72	4
E2F5	1	ST6GALNAC4	4
E2F6	1	ST6GALNAC6	4
E2F8	1	ST8SIA5	4
EAPP	1	STAG3L2	4
EARS2	1	STX18	4
EBLN2	1	SUFU	4
ECHDC3	1	SULT2B1	4
ECM1	1	SYF2	4
ECM2	1	SYN3	4
ECT2L	1	SYNGR4	4
EDARADD	1	SYNJ1	4
EDNRA	1	SYT3	4
EEF1A2	1	TARBP2	4
EFCAB2	1	TAS1R3	4
EFCAB3	1	TBC1D13	4
EFCAB4B	1	TBC1D19	4
EFNA1	1	TBC1D9B	4

EFNA3	1	TCEB3B	4
EFTUD1	1	TCEB3C	4
EFTUD2	1	TCF7	4
EHD3	1	TEAD2	4
EHF	1	TEKT3	4
EIF2B3	1	TENC1	4
EIF2C2	1	TESK1	4
EIF4EBP1	1	TFAP2A	4
EIF4G2	1	TFPT	4
ELF2	1	TGM1	4
ELF5	1	TGM4	4
ELL2	1	TH	4
ELP2	1	THRA	4
EMCN	1	TIMP3	4
EMID2	1	TINF2	4
EMILIN1	1	TJP2	4
EMILIN2	1	TLN1	4
EMP1	1	TM2D1	4
EMP2	1	TM4SF5	4
EN1	1	TM9SF1	4
ENAM	1	TMEM110	4
ENOSF1	1	TMEM120B	4
ENPP1	1	TMEM143	4
ENSA	1	TMEM14B	4
EPB41L2	1	TMEM14C	4
EPB41L3	1	TMEM156	4
EPG5	1	TMEM180	4
EPHA4	1	TMEM184A	4
EPHA5	1	TMEM39B	4
EPHB4	1	TMEM40	4
EPHX1	1	TMEM50A	4
EPN3	1	TMPRSS3	4
EPS8	1	TNKS	4
ERAP1	1	TNRC18	4
ERBB3	1	TOM1L2	4
ERC1	1	TOP3A	4
ERLIN2	1	TOR1A	4
ERN2	1	TOR1B	4
ESAM	1	TOR4A	4
ESCO1	1	TPP1	4
ESR1	1	TPTE	4
ESRP1	1	TRADD	4
EVX1	1	TRIM3	4
EVX2	1	TRIM33	4
EXOC1	1	TRIM73	4
EXOC6B	1	TRIM8	4
EXT2	1	TROAP	4

EYA4	1	TRPM4	4
F13A1	1	TRUB2	4
FABP12	1	TSKS	4
FABP2	1	TSPAN5	4
FABP4	1	TSR1	4
FABP5	1	TSSC4	4
FAM107B	1	TSSK3	4
FAM110A	1	TSSK4	4
FAM110B	1	TTC28	4
FAM111A	1	TTLL10	4
FAM115A	1	TUBA1B	4
FAM115C	1	TUBB4B	4
FAM126A	1	TUFM	4
FAM129A	1	TULP2	4
FAM160A1	1	TYRO3	4
FAM171A1	1	UAP1L1	4
FAM171A2	1	UBAP1	4
FAM171B	1	UBE2G1	4
FAM172A	1	UBE2J2	4
FAM174A	1	UBE2R2	4
FAM176A	1	UBE3C	4
FAM188A	1	UBR2	4
FAM188B	1	UCMA	4
FAM18A	1	UPK3B	4
FAM198B	1	USP10	4
FAM19A1	1	USP20	4
FAM203A	1	USP46	4
FAM208B	1	VAT1L	4
FAM20B	1	VAV1	4
FAM214A	1	VIM	4
FAM217A	1	VPRBP	4
FAM219A	1	VRK3	4
FAM222B	1	VSX2	4
FAM26E	1	VWA1	4
FAM3C	1	WBSCR16	4
FAM54A	1	WDR34	4
FAM59A	1	WDR47	4
FAM60A	1	WFDC1	4
FAM63A	1	WIPF2	4
FAM63B	1	WIPI2	4
FAM81A	1	WNT2B	4
FAM81B	1	WWC1	4
FAM83H	1	XPO7	4
FAM86B1	1	XRCC2	4
FAM8A1	1	ZBTB7C	4
FAM92A1	1	ZCCHC2	4
FANCC	1	ZDHHC7	4

FANCI	1	ZER1	4
FANCM	1	ZFP3	4
FARS2	1	ZFYVE1	4
FAT3	1	ZFYVE16	4
FBN1	1	ZMYND15	4
FBP1	1	ZNF100	4
FBP2	1	ZNF114	4
FBXL16	1	ZNF136	4
FBXL18	1	ZNF20	4
FBXO18	1	ZNF232	4
FBXO24	1	ZNF236	4
FBXO48	1	ZNF296	4
FBXO8	1	ZNF354C	4
FBXW7	1	ZNF410	4
FCER1G	1	ZNF423	4
FCGR1A	1	ZNF429	4
FCGR1B	1	ZNF43	4
FCGR2A	1	ZNF430	4
FCGR3A	1	ZNF431	4
FCGR3B	1	ZNF433	4
FES	1	ZNF44	4
FETUB	1	ZNF440	4
FEZF1	1	ZNF441	4
FGA	1	ZNF442	4
FGB	1	ZNF443	4
FGD4	1	ZNF473	4
FGF2	1	ZNF516	4
FGF20	1	ZNF532	4
FGF7	1	ZNF563	4
FGFR1	1	ZNF564	4
FGFR1OP	1	ZNF594	4
FGFR1OP2	1	ZNF624	4
FGG	1	ZNF625	4
FGL1	1	ZNF626	4
FH	1	ZNF627	4
FHDC1	1	ZNF682	4
FHOD3	1	ZNF708	4
FILIP1	1	ZNF709	4
FIS1	1	ZNF714	4
FJX1	1	ZNF763	4
FKBP14	1	ZNF788	4
FKBP3	1	ZNF799	4
FKBP6	1	ZNF821	4
FLAD1	1	ZNF823	4
FLJ42393	1	ZNF844	4
FMNL1	1	ZNF879	4
FN1	1	ZNF90	4

FNDC1	1	ZNF98	4
FNDC4	1	ZZEF1	4
FOS	1	AADACL4	3
FOSL2	1	AADAT	3
FOXC1	1	AAED1	3
FOXL1	1	AANAT	3
FOXM1	1	AARS	3
FOXN1	1	AARSD1	3
FOXQ1	1	ABCA11P	3
FRAS1	1	ABCB8	3
FREM3	1	ABCB9	3
FRMD4A	1	ABCF2	3
FRMD5	1	ABHD11	3
FSCN1	1	ABHD4	3
FSCN3	1	ABI1	3
FSTL1	1	ACAA2	3
FTO	1	ACAD10	3
FUCA2	1	ACBD5	3
FURIN	1	ACD	3
FUT4	1	ACHE	3
FZD10	1	ACIN1	3
FZD6	1	ACLY	3
G6PC3	1	ACOT11	3
GAA	1	ACOT12	3
GAB1	1	ACOX1	3
GABPB1	1	ACOX2	3
GABRA4	1	ACTL8	3
GABRA5	1	ACTN3	3
GABRG3	1	ACTN4	3
GAD2	1	ACTRT2	3
GAL3ST4	1	ACYP1	3
GALK2	1	ADAM11	3
GALM	1	ADAM8	3
GALNT1	1	ADAMTS13	3
GALNT14	1	ADAMTSL2	3
GALNTL4	1	ADAP1	3
GAPVD1	1	ADCK1	3
GAS2L3	1	ADCK2	3
GATA2	1	ADCK4	3
GATA6	1	ADH1A	3
GATM	1	ADH4	3
GATS	1	ADH5	3
GATSL1	1	ADH6	3
GBAP1	1	ADPRHL1	3
GBE1	1	AFF4	3
GC	1	AGA	3
GCKR	1	AGAP3	3

GCNT1	1	AGBL2	3
GCNT2	1	AGGF1	3
GCNT3	1	AGMAT	3
GCOM1	1	AGTRAP	3
GDA	1	AGXT2L2	3
GDF11	1	AHCYL2	3
GDF6	1	AHNAK	3
GDI2	1	AHSA1	3
GEM	1	AIMP2	3
GFAP	1	AK8	3
GFRA4	1	AKAP12	3
GGA2	1	AKIRIN1	3
GGT3P	1	AKIRIN2	3
GH1	1	AKNAD1	3
GID8	1	AKR1A1	3
GIN1	1	AKR1D1	3
GJC1	1	AKR7L	3
GKN1	1	ALAS1	3
GKN2	1	ALDH1B1	3
GLB1L2	1	ALDH2	3
GLDN	1	ALDH4A1	3
GLE1	1	ALDH7A1	3
GLRX	1	ALKBH1	3
GLUL	1	ALKBH2	3
GLYAT	1	ALKBH6	3
GLYATL2	1	ALPL	3
GMDS	1	ALS2CL	3
GML	1	AMD1	3
GNA14	1	ANAPC5	3
GNAL	1	ANAPC7	3
GNB5	1	ANGEL1	3
GNG10	1	ANGPTL7	3
GNG4	1	ANK1	3
GNGT2	1	ANK2	3
GNPDA2	1	ANKDD1A	3
GNRHR	1	ANKHD1	3
GNRHR2	1	ANKHD1-EIF4EBI	3
GOLGA1	1	ANKRD13A	3
GOLGA8F	1	ANKRD16	3
GOLGA8G	1	ANKRD31	3
GOLPH3L	1	ANKRD52	3
GON4L	1	ANKRD55	3
GOSR2	1	ANKRD6	3
GPA33	1	ANKS1A	3
GPATCH4	1	AOC2	3
GPATCH8	1	AOC3	3
GPIHBP1	1	AP1S1	3

GPR1	1	AP2B1	3
GPR116	1	AP2S1	3
GPR124	1	AP4B1	3
GPR137B	1	AP4E1	3
GPR139	1	AP5B1	3
GPR144	1	AP5Z1	3
GPR158	1	APBA2	3
GPR160	1	APBB1IP	3
GPR161	1	APBB3	3
GPR22	1	APC	3
GPR39	1	APITD1	3
GPR98	1	APLP1	3
GPRC6A	1	APOC1	3
GPT	1	APOC2	3
GRAPL	1	APOE	3
GRHL1	1	APPL1	3
GRHL2	1	APTX	3
GRID2IP	1	AQP10	3
GRIK4	1	AQR	3
GRIN2B	1	ARG2	3
GRN	1	ARHGAP11B	3
GSDMD	1	ARHGAP12	3
GSTA4	1	ARHGAP21	3
GSTCD	1	ARHGAP27	3
GTF2E2	1	ARHGAP9	3
GTF2H2D	1	ARHGEF1	3
GTF2I	1	ARHGEF10L	3
GTF2IP1	1	ARHGEF19	3
GTF2IRD1	1	ARHGEF3	3
GTF2IRD2	1	ARHGEF37	3
GTF2IRD2B	1	ARL10	3
GUCA1C	1	ARL4D	3
GUCY1A3	1	ARL5C	3
GUCY1B3	1	ARMC12	3
GUSB	1	ARMC7	3
GXYLT2	1	ARPC3	3
GYG1	1	ARPC5L	3
GYPA	1	ARSB	3
H2AFV	1	ASAP3	3
HABP4	1	ASB14	3
HACE1	1	ASB16	3
HADHB	1	ASH2L	3
HBP1	1	ATF7	3
HCK	1	ATG2A	3
HDAC5	1	ATG9B	3
HDC	1	ATHL1	3
HDDC3	1	ATMIN	3

HDGF	1	ATP10A	3
HDHD2	1	ATP13A2	3
HEBP2	1	ATP1A3	3
HECW1	1	ATP2A2	3
HEPACAM	1	ATP4A	3
HERC2	1	ATP5G2	3
HERC2P2	1	ATP5H	3
HEXA	1	ATP5J	3
HEXIM1	1	ATP5S	3
HEXIM2	1	ATP6V0A1	3
HEY1	1	ATP6V0A2	3
HFE2	1	ATP6V0A4	3
HGC6.3	1	ATP6V0B	3
HIATL1	1	ATP6V0D1	3
HIATL2	1	ATP6V0E1	3
HIBADH	1	ATP6V1F	3
HIGD1B	1	ATXN1	3
HIP1	1	ATXN10	3
HIPK3	1	ATXN2	3
HIST2H2AA3	1	ATXN7L1	3
HIST2H2BE	1	AVEN	3
HIST2H2BF	1	AXL	3
HIST2H3C	1	B2M	3
HIST2H4A	1	B3GALT4	3
HLTF	1	B3GALT5	3
HNRNPA2B1	1	B3GAT3	3
HNRNPA3	1	B3GNT4	3
HNRNPA3P1	1	B4GALNT2	3
HNRNPC	1	B4GALT7	3
HNRNPU	1	B9D2	3
HOMER2	1	BAHD1	3
HOPX	1	BAMBI	3
HOXB1	1	BANF1	3
HOXB13	1	BAP1	3
HOXB3	1	BATF2	3
HOXB5	1	BAZ1A	3
HOXB6	1	BAZ1B	3
HOXB7	1	BAZ2A	3
HOXB8	1	BBC3	3
HOXB9	1	BBS1	3
HOXD12	1	BCKDHA	3
HOXD13	1	BCL2	3
HOXD3	1	BCL2L10	3
HOXD4	1	BCL2L15	3
HOXD8	1	BCL3	3
HPCAL1	1	BCL7A	3
HPDL	1	BCL7B	3

HPS3	1	BCMO1	3
HSD17B2	1	BICD1	3
HSD17B3	1	BIN2	3
HSD17B7P2	1	BIN3	3
HSF2	1	BLOC1S3	3
HSP90AB4P	1	BMF	3
HSPA14	1	BMP6	3
HSPA4L	1	BNIP1	3
HSPA5	1	BNIP3	3
HSPA6	1	BRAP	3
HSPB1	1	BRCA1	3
HSPBAP1	1	BRD3	3
HTATIP2	1	BRD8	3
HTN1	1	BRDT	3
HTN3	1	BRF2	3
HTR7	1	BRI3BP	3
HTRA4	1	BRMS1	3
IAH1	1	BRSK1	3
ICAM2	1	BSCL2	3
ICK	1	BTF3L4	3
ICOS	1	BTRC	3
IDO2	1	C10orf108	3
IER3IP1	1	C10orf2	3
IER5	1	C10orf76	3
IFNGR1	1	C11orf48	3
IFRD1	1	C11orf80	3
IFT172	1	C12orf43	3
IFT20	1	C12orf65	3
IFT81	1	C14orf119	3
IGF2	1	C14orf166B	3
IGF2BP1	1	C15orf52	3
IGF2BP3	1	C15orf57	3
IGFBP2	1	C15orf62	3
IGFBP7	1	C16orf46	3
IGJ	1	C17orf103	3
IKZF4	1	C17orf28	3
IL16	1	C17orf47	3
IL1F10	1	C17orf53	3
IL1RN	1	C17orf64	3
IL2	1	C17orf66	3
IL20RA	1	C19orf54	3
IL21	1	C19orf55	3
IL22RA2	1	C1QA	3
IL23A	1	C1QB	3
IL26	1	C1QC	3
IL2RA	1	C1orf126	3
IL36RN	1	C1orf127	3

ILDR1	1	C1orf177	3
IMPA2	1	C1orf200	3
INO80C	1	C1orf228	3
INPP1	1	C1orf64	3
INPP4A	1	C21orf88	3
INPP5K	1	C2orf57	3
INS-IGF2	1	C3AR1	3
INTS12	1	C5AR1	3
INTS8	1	C5orf24	3
INTU	1	C5orf25	3
IPCEF1	1	C5orf4	3
IPO9	1	C5orf48	3
IQGAP3	1	C6orf108	3
IRAK4	1	C6orf132	3
IRF2BPL	1	C6orf147	3
IRX5	1	C6orf211	3
ISG20L2	1	C7orf26	3
ISLR2	1	C7orf50	3
ITGA3	1	C7orf55	3
ITGA7	1	C8orf12	3
ITGA8	1	C8orf58	3
ITGAV	1	C8orf74	3
ITGB1BP1	1	C8orf86	3
ITGB3	1	C9orf123	3
ITLN1	1	C9orf131	3
ITPA	1	C9orf24	3
ITPR2	1	C9orf9	3
ITSN2	1	CA6	3
IYD	1	CA7	3
JAM3	1	CABP1	3
JRK	1	CABP5	3
KALRN	1	CABYR	3
KATNAL2	1	CACFD1	3
KBTBD2	1	CACNB1	3
KC6	1	CACNB2	3
KCNA1	1	CADM4	3
KCNA6	1	CALM3	3
KCNB1	1	CALML3	3
KCNB2	1	CALML6	3
KCNF1	1	CALN1	3
KCNG1	1	CALU	3
KCNH6	1	CAMKK2	3
KCNH8	1	CAMLG	3
KCNJ12	1	CAMSAP1	3
KCNJ16	1	CAPN1	3
KCNJ2	1	CAPN3	3
KCNRG	1	CAPZB	3

KCNU1	1	CARD11	3
KCTD1	1	CARS	3
KDM4D	1	CASC4	3
KDM5A	1	CASP8AP2	3
KDR	1	CASP9	3
KHDRBS2	1	CASZ1	3
KIAA0391	1	CATSPER1	3
KIAA0825	1	CATSPER2	3
KIAA0907	1	CATSPER2P1	3
KIAA0922	1	CATSPERG	3
KIAA1199	1	CBLC	3
KIAA1211	1	CC2D1B	3
KIAA1217	1	CCAR1	3
KIAA1244	1	CCDC101	3
KIAA1328	1	CCDC11	3
KIAA1377	1	CCDC12	3
KIAA1429	1	CCDC125	3
KIAA1549L	1	CCDC136	3
KIAA1551	1	CCDC27	3
KIAA1737	1	CCDC42B	3
KIF13A	1	CCDC61	3
KIF21A	1	CCDC62	3
KIF21B	1	CCDC79	3
KIF24	1	CCDC8	3
KIF26B	1	CCDC88C	3
KIF3B	1	CCDC9	3
KIFC2	1	CCL21	3
KIN	1	CCL24	3
KIRREL	1	CCL27	3
KIRREL3	1	CCNB1	3
KLF10	1	CCNDBP1	3
KLF11	1	CCNJL	3
KLF3	1	CCPG1	3
KLF4	1	CCR10	3
KLHDC5	1	CCR8	3
KLRB1	1	CCT6A	3
KLRC3	1	CCT6B	3
KLRC4	1	CD14	3
KLRC4-KLRK1	1	CD22	3
KLRF1	1	CD248	3
KLRK1	1	CD33	3
KMO	1	CD3EAP	3
KPNA2	1	CDA	3
KRT18P55	1	CDC14B	3
KRTCAP2	1	CDC23	3
KRTCAP3	1	CDC25C	3
L3MBTL3	1	CDC42BPG	3

LACTB2	1	CDC42EP2	3
LAD1	1	CDC6	3
LAMA3	1	CDCA5	3
LAMA4	1	CDCP1	3
LAMB1	1	CDH1	3
LAMB4	1	CDH24	3
LAMC1	1	CDH3	3
LAMC2	1	CDHR2	3
LAPTM4A	1	CDK12	3
LAPTM4B	1	CDK19	3
LARP1B	1	CDK2	3
LAYN	1	CDK2AP1	3
LBR	1	CDK3	3
LCLAT1	1	CDK7	3
LENEP	1	CDKL1	3
LEO1	1	CDKL3	3
LEPROTL1	1	CDKN2AIPNL	3
LETM2	1	CDKN2C	3
LGR4	1	CDR2L	3
LGR5	1	CDRT1	3
LGSN	1	CEACAM19	3
LHX2	1	CEACAM3	3
LHX4	1	CEBPE	3
LIN9	1	CELA1	3
LINC00152	1	CELA2B	3
LINC00272	1	CELA3A	3
LINC00476	1	CELA3B	3
LIPC	1	CENPH	3
LIPG	1	CENPN	3
LIPT2	1	CEP95	3
LIX1	1	CFL1	3
LIX1L	1	CFL2	3
LMOD3	1	CHAC1	3
LNPEP	1	CHFR	3
LNX1	1	CHPF2	3
LOC100093631	1	CHRM5	3
LOC100131691	1	CHRNB3	3
LOC100170939	1	CHST12	3
LOC100287792	1	CHST8	3
LOC149134	1	CIB1	3
LOC151009	1	CIC	3
LOC284751	1	CKLF	3
LOC285768	1	CKM	3
LOC388692	1	CKMT2	3
LOC440040	1	CLCN6	3
LOC440461	1	CLDN15	3
LOC643837	1	CLDN3	3

LOC644145	1	CLDN4	3
LOC654433	1	CLEC2L	3
LOC728024	1	CLEC3B	3
LOXHD1	1	CLEC4C	3
LPA	1	CLIC4	3
LPAL2	1	CLIP1	3
LPAR1	1	CLIP3	3
LPIN1	1	CLK4	3
LPIN2	1	CLN3	3
LRBA	1	CLNK	3
LRRC1	1	CLPTM1	3
LRRC31	1	CLSPN	3
LRRC37A2	1	CLSTN1	3
LRRC4	1	CLTB	3
LRRC49	1	CLTC	3
LRRC59	1	CMTM1	3
LRRCC1	1	CMTM2	3
LRRIQ4	1	CMTM6	3
LRRK2	1	CMTM7	3
LSM5	1	CMTM8	3
LTBP1	1	CMYA5	3
LUC7L3	1	CNFN	3
LY6D	1	CNKSR3	3
LY6E	1	CNOT6	3
LY6K	1	CNP	3
LY86	1	CNPY2	3
LYNX1	1	CNPY4	3
LYPD2	1	CNR2	3
LYPD6	1	CNTD1	3
LYPD6B	1	CNTD2	3
LYRM4	1	CNTFR	3
LYZL1	1	CNTNAP1	3
LYZL2	1	COA3	3
MAF	1	COASY	3
MAGEL2	1	COG4	3
MAGOHB	1	COG8	3
MAK	1	COL23A1	3
MAML3	1	COL25A1	3
MAN1A1	1	COL5A1	3
MAN2A2	1	COPS7B	3
MANEA	1	COX19	3
MAP2K3	1	COX6A1	3
MAP2K5	1	COX6B1	3
MAP3K14	1	CPA4	3
MAP3K4	1	CPA5	3
MAP3K5	1	CPLX2	3
MAPK6	1	CPLX4	3

MAPKBP1	1	CPT2	3
MAPRE2	1	CRHBP	3
MAPT	1	CRHR1	3
10-Mar	1	CRX	3
MARCO	1	CS	3
MAS1	1	CSGALNACT1	3
MASP1	1	CSNK1A1	3
MAST2	1	CSRNP2	3
MASTL	1	CST6	3
MB21D1	1	CTCF	3
MBL2	1	CTDSPL2	3
MBLAC2	1	CTNNBIP1	3
MC5R	1	CTRC	3
MCM10	1	CTSW	3
MCM3	1	CTTNBP2NL	3
MCM7	1	CUL1	3
MCTP1	1	CUTA	3
MDGA2	1	CUX1	3
MDK	1	CUX2	3
MEG3	1	CXCL17	3
MEGF9	1	CXXC1	3
MEIG1	1	CYB5B	3
MEP1A	1	CYFIP1	3
MEP1B	1	CYGB	3
MESDC2	1	CYP2A13	3
MET	1	CYP2A7	3
METTL20	1	CYP2B6	3
METTL2A	1	CYP2B7P1	3
METTL7B	1	CYP2F1	3
MFGE8	1	CYP2S1	3
MFHAS1	1	CYP4A22	3
MFSD6	1	CYTH3	3
MGAM	1	DAB2IP	3
MGAT2	1	DACT3	3
MGAT4A	1	DAGLB	3
MGAT5	1	DAO	3
MGC2752	1	DAZAP2	3
MGC39372	1	DBF4B	3
MGC72080	1	DCAF10	3
MGLL	1	DCAF4L1	3
MGST2	1	DCAKD	3
MGST3	1	DCLRE1B	3
MIA2	1	DCP1A	3
MICAL2	1	DCTN2	3
MIR106B	1	DCTN3	3
MIR127	1	DDI2	3
MIR129-2	1	DDIT3	3

MIR133B	1	DDOST	3
MIR154	1	DDX19A	3
MIR23B	1	DDX19B	3
MIR27B	1	DDX21	3
MIR377	1	DDX39B	3
MIR423	1	DDX46	3
MIR600HG	1	DDX5	3
MITF	1	DDX50	3
MKX	1	DDX51	3
MLLT10	1	DDX54	3
MLLT4	1	DDX55	3
MMADHC	1	DEDD2	3
MMD2	1	DEF6	3
MMP19	1	DEFB1	3
MND1	1	DEK	3
MNS1	1	DENND2A	3
MOCOS	1	DENR	3
MOGAT3	1	DFFA	3
MOSPD3	1	DFFB	3
MPLKIP	1	DGKA	3
MPP2	1	DHCR24	3
MPP7	1	DHFR	3
MPPE1	1	DHODH	3
MPZ	1	DHRS3	3
MPZL1	1	DHRS7B	3
MR1	1	DHX29	3
MRAS	1	DHX34	3
MRC2	1	DHX37	3
MREG	1	DHX8	3
MRGPRX1	1	DIABLO	3
MRGPRX2	1	DIP2C	3
MRPL27	1	DISP2	3
MRPL33	1	DLC1	3
MRPS24	1	DLEU2	3
MRPS35	1	DLK2	3
MRPS9	1	DLL4	3
MRRF	1	DMBX1	3
MSANTD2	1	DMGDH	3
MSGN1	1	DMKN	3
MSMB	1	DMPK	3
MSRB3	1	DMRTC2	3
MSTO1	1	DMWD	3
MSTO2P	1	DNA2	3
MTERFD1	1	DNAAF3	3
MTHFS	1	DNAH1	3
MTMR11	1	DNAJA1	3
MTMR7	1	DNAJB12	3

MTNR1B	1	DNAJC1	3
MTO1	1	DNAJC11	3
MTX1	1	DNAJC16	3
MUC12	1	DNAJC17	3
MUC17	1	DNAJC18	3
MUC7	1	DNAJC30	3
MUM1	1	DOCK3	3
MYCN	1	DPF1	3
MYH16	1	DPF2	3
MYL1	1	DPP3	3
MYL10	1	DPPA3	3
MYLK	1	DRD5	3
MYLK4	1	DSCAM	3
MYO18A	1	DSPP	3
MYO1A	1	DUOX1	3
MYO1B	1	DUOX2	3
MYO1C	1	DUOXA1	3
MYO1E	1	DUOXA2	3
MYO5A	1	DYNC1LI2	3
MYO5B	1	DYNLL1	3
MYO5C	1	DYX1C1	3
MYO9A	1	E2F2	3
MYOM1	1	ECE1	3
MYT1L	1	ECHDC2	3
MZF1	1	ECHDC3	3
NAA38	1	ECM2	3
NABP2	1	ECSCR	3
NAIP	1	ECT2L	3
NAPRT1	1	EDC4	3
NAT1	1	EFCAB3	3
NAT2	1	EFEMP2	3
NAV2	1	EFHB	3
NBPF15	1	EFHD2	3
NCALD	1	EFTUD2	3
NCAPD3	1	EGLN2	3
NCF1	1	EGR1	3
NCF1B	1	EGR3	3
NCF1C	1	EHD2	3
NCK2	1	EHMT1	3
NCKAP5	1	EIF1	3
NCOA1	1	EIF1AD	3
NCOA2	1	EIF2AK1	3
NDFIP2	1	EIF2B3	3
NDN	1	EIF3K	3
NDRG2	1	EIF4E1B	3
NDST4	1	EIF4EBP1	3
NDUFA5	1	EIF4G3	3

NDUFA9	1	ELN	3
NDUFAB1	1	ELSPBP1	3
NDUFC1	1	EMC4	3
NDUFS2	1	EML2	3
NDUFS4	1	EML3	3
NDUFS7	1	ENC1	3
NDUFV2	1	ENO1	3
NEBL	1	ENTPD3	3
NEDD1	1	EP400	3
NEDD4	1	EPB42	3
NEK10	1	EPHA2	3
NEMF	1	EPHA8	3
NEO1	1	EPHB2	3
NET1	1	EPHB4	3
NFE2L1	1	EPN1	3
NFE2L2	1	EPS8L1	3
NFE2L3	1	ERBB3	3
NFIL3	1	ERCC1	3
NGFR	1	ERCC2	3
NID1	1	ERF	3
NIPA2	1	ERGIC1	3
NIPAL2	1	ERI3	3
NISCH	1	ERLIN2	3
NIT1	1	ERRFI1	3
NKAIN4	1	ESPNP	3
NKD1	1	ESRRB	3
NLK	1	ESYT2	3
NME2	1	ETF1	3
NME7	1	ETFDH	3
NME9	1	ETHE1	3
NMNAT2	1	ETV4	3
NMT1	1	EVPL	3
NMT2	1	EXOC3L2	3
NMU	1	EXOC7	3
NNMT	1	EXOSC10	3
NOA1	1	EXOSC3	3
NOD1	1	EXOSC5	3
NOD2	1	EXOSC7	3
NOL10	1	EXTL3	3
NOL4	1	EZH1	3
NOL8	1	F2RL2	3
NOS1AP	1	FAAH	3
NOSTRIN	1	FABP3	3
NOTCH2NL	1	FADS6	3
NOVA1	1	FAF1	3
NOX3	1	FAF2	3
NOX4	1	FAM107A	3

NPC1	1	FAM131C	3
NPL	1	FAM134C	3
NPNT	1	FAM13B	3
NPTX2	1	FAM151A	3
NQO2	1	FAM153A	3
NR1I2	1	FAM153B	3
NR2E3	1	FAM153C	3
NR3C2	1	FAM159A	3
NR5A1	1	FAM169A	3
NRBP1	1	FAM171A2	3
NRCAM	1	FAM180B	3
NRGN	1	FAM18B2	3
NRSN2	1	FAM208B	3
NSF	1	FAM213B	3
NSUN3	1	FAM214B	3
NT5C3	1	FAM3B	3
NT5DC2	1	FAM53C	3
NTNG1	1	FAM65A	3
NTRK1	1	FAM71B	3
NUAK1	1	FAM71E2	3
NUDT2	1	FAM82A2	3
NUDT5	1	FAM89B	3
NUFIP2	1	FANCM	3
NUS1	1	FASTK	3
NVL	1	FBF1	3
NXPH2	1	FBLIM1	3
OAF	1	FBLN2	3
OCA2	1	FBP1	3
ODC1	1	FBP2	3
OGN	1	FBXL2	3
OLAH	1	FBXO10	3
OLIG3	1	FBXO16	3
ONECUT1	1	FBXO18	3
OPRM1	1	FBXO2	3
OPTC	1	FBXO42	3
OPTN	1	FBXO44	3
OR10AG1	1	FBXO46	3
OR10P1	1	FBXW11	3
OR11G2	1	FBXW4	3
OR11H6	1	FCAR	3
OR13G1	1	FCF1	3
OR1B1	1	FDXR	3
OR1J4	1	FGD6	3
OR1L1	1	FGF18	3
OR1L4	1	FGFR1	3
OR1N1	1	FHAD1	3
OR1Q1	1	FHOD3	3

OR2A20P	1	FIBP	3
OR2A4	1	FIS1	3
OR2AK2	1	FKBP10	3
OR2C3	1	FKBP5	3
OR2F1	1	FKBP6	3
OR2F2	1	FKBP9L	3
OR2G3	1	FLJ35024	3
OR2L13	1	FLNC	3
OR2L2	1	FLT4	3
OR2L3	1	FMNL1	3
OR2L8	1	FNDC8	3
OR2M2	1	FNTA	3
OR2M3	1	FOCAD	3
OR2M5	1	FOS	3
OR2T8	1	FOSB	3
OR4K14	1	FOXA3	3
OR4K15	1	FOXC1	3
OR4K17	1	FOXJ1	3
OR4L1	1	FOXP4	3
OR51T1	1	FOXQ1	3
OR5AS1	1	FRMD5	3
OR5AU1	1	FRMPD1	3
OR5D13	1	FRS3	3
OR5D14	1	FRYL	3
OR5D16	1	FUBP1	3
OR5D18	1	FUCA1	3
OR5F1	1	FUK	3
OR5I1	1	FXC1	3
OR5J2	1	FXYD1	3
OR5K3	1	FXYD5	3
OR5L1	1	FXYD7	3
OR5L2	1	G6PC3	3
OR5M1	1	GABPB1	3
OR5M11	1	GABRD	3
OR5M3	1	GABRR1	3
OR5M8	1	GABRR2	3
OR5M9	1	GAD2	3
OR5R1	1	GAL3ST4	3
OR5T2	1	GALNT6	3
OR5T3	1	GALNT9	3
OR5W2	1	GALR2	3
OR6C4	1	GAN	3
OR6F1	1	GANAB	3
OR6M1	1	GAPVD1	3
OR8A1	1	GAST	3
OR8B3	1	GATM	3
OR8H1	1	GATS	3

OR8H2	1	GBAS	3
OR8H3	1	GCHFR	3
OR8I2	1	GCNT4	3
OR8J1	1	GDF3	3
OR8K1	1	GDI2	3
OR8K3	1	GDPD1	3
OR8K5	1	GDPGP1	3
OR8U1	1	GEMIN5	3
OR8U8	1	GET4	3
OR9G1	1	GFAP	3
OR9G4	1	GFM2	3
OR9G9	1	GFOD2	3
ORC4	1	GFPT2	3
ORMDL1	1	GFRA3	3
ORMDL2	1	GGA3	3
OSBPL3	1	GGN	3
OSBPL6	1	GGT3P	3
OSBPL7	1	GIPC2	3
OSGEP	1	GIPR	3
OSGEPL1	1	GIT2	3
OSR2	1	GJC1	3
OTUD7B	1	GLI1	3
OXT	1	GLIS1	3
P2RY2	1	GLIS3	3
P4HA3	1	GLT1D1	3
PABPC1	1	GLTP	3
PABPC1L	1	GLTSCR1	3
PACRG	1	GLTSCR2	3
PAG1	1	GLYCTK	3
PAH	1	GMDS	3
PAK6	1	GMFG	3
PAM	1	GNA12	3
PAMR1	1	GNB1	3
PANK2	1	GNB5	3
PANX3	1	GNG3	3
PAQR8	1	GNG8	3
PAQR9	1	GNGT2	3
PARD6B	1	GOLGA1	3
PATL2	1	GOLGA3	3
PAX3	1	GOLGA4	3
PAX7	1	GOLGA8A	3
PAX9	1	GORASP1	3
PBOV1	1	GP6	3
PBXIP1	1	GPATCH8	3
PCGF2	1	GPD1L	3
PCNA	1	GPI	3
PCNXL2	1	GPR123	3

PCOLCE	1	GPR124	3
PCP4L1	1	GPR125	3
PCSK4	1	GPR144	3
PDE10A	1	GPR157	3
PDE1A	1	GPR179	3
PDE4DIP	1	GPR4	3
PDE7A	1	GPR61	3
PDE7B	1	GPRIN1	3
PDE8A	1	GPSM2	3
PDGFRA	1	GPX7	3
PDGFRL	1	GRAMD1A	3
PDHX	1	GRB2	3
PDIA6	1	GRHL3	3
PDSS1	1	GRHPR	3
PDZRN3	1	GRID2IP	3
PDZRN4	1	GRIK5	3
PEAR1	1	GRM2	3
PECAM1	1	GRN	3
PECR	1	GRP	3
PERP	1	GSTZ1	3
PET112	1	GTF2H3	3
PEX3	1	GTF2I	3
PFDN2	1	GTF2IP1	3
PFKFB3	1	GTF2IRD1	3
PFN1P2	1	GTPBP4	3
PFN4	1	GUSBP3	3
PGM2	1	H3F3B	3
PGM2L1	1	H6PD	3
PGPEP1L	1	HABP4	3
PHACTR2	1	HAND2	3
PHC3	1	HAP1	3
PHF12	1	HARS	3
PHF17	1	HARS2	3
PHF3	1	HAUS2	3
PHKB	1	HAUS5	3
PHLDA3	1	HAVCR1	3
PHYH	1	HAVCR2	3
PIAS1	1	HBEGF	3
PIAS2	1	HCAR1	3
PIAS3	1	HCAR2	3
PIBF1	1	HCAR3	3
PIGM	1	HCRTR1	3
PIK3C3	1	HCST	3
PIK3R3	1	HDAC11	3
PILRA	1	HDAC5	3
PIP4K2B	1	HDC	3
PITPNA	1	HDHD2	3

PITPNC1	1	HECTD4	3
PIWIL1	1	HES5	3
PIWIL4	1	HESX1	3
PKIB	1	HEXB	3
PKNOX2	1	HEXIM1	3
PKP1	1	HEXIM2	3
PKP2	1	HIATL1	3
PLCB2	1	HIATL2	3
PLCD3	1	HIF1AN	3
PLCL1	1	HIF3A	3
PLCL2	1	HIGD1B	3
PLEKHA2	1	HILPDA	3
PLEKHA5	1	HINT2	3
PLEKHM1P	1	HIP1	3
PLK1	1	HIP1R	3
PLOD3	1	HIPK1	3
PLRG1	1	HK3	3
PLXDC2	1	HMGA1	3
PMCH	1	HMGCL	3
PMFBP1	1	HMGCR	3
PMM2	1	HN1	3
PMS1	1	HNF1A	3
PMS2P5	1	HNF1A-AS1	3
PMVK	1	HNRNPAB	3
PNLDC1	1	HNRNPC	3
PNN	1	HNRNPH3	3
PNP	1	HNRNPUL1	3
PNPO	1	HOOK3	3
PODXL2	1	HPD	3
POFUT1	1	HPDL	3
POGZ	1	HS3ST3A1	3
POLD3	1	HSD17B8	3
POLE2	1	HSP90AB1	3
POLG2	1	HSP90AB3P	3
POLR2B	1	HSPA4	3
POLR2K	1	HSPA4L	3
POLR2M	1	HSPA5	3
POM121	1	HSPA9	3
POM121C	1	HSPB1	3
POMC	1	HSPB7	3
POP1	1	HSPBP1	3
POR	1	HSPG2	3
POU1F1	1	HTA	3
POU2F1	1	HTR1D	3
POU2F3	1	HVCN1	3
POU4F2	1	IBSP	3
POU5F2	1	ID3	3

PPAP2C	1	IDE	3
PPAPDC1B	1	IER3IP1	3
PPARGC1A	1	IFFO2	3
PPAT	1	IFI35	3
PPDPF	1	IFITM1	3
PPFIBP1	1	IFITM2	3
PPM1L	1	IFITM3	3
PPOX	1	IFNA10	3
PPP1R12B	1	IFNA13	3
PPP1R14C	1	IFNA14	3
PPP1R14D	1	IFNA16	3
PPP1R16A	1	IFNA17	3
PPP1R3G	1	IFNA2	3
PPP1R8	1	IFNA21	3
PPP2R2B	1	IFNA4	3
PPP2R3C	1	IFNA5	3
PPP4R1	1	IFNA6	3
PPP4R2	1	IFNA7	3
PPY	1	IFNA8	3
PQLC3	1	IFNB1	3
PRAMEF1	1	IFNW1	3
PRAMEF12	1	IFT81	3
PRB3	1	IGF2BP1	3
PRB4	1	IGFBPL1	3
PRC1	1	IGFL1	3
PRDM1	1	IGSF5	3
PREP	1	IK	3
PREX2	1	IKZF4	3
PRIM2	1	IL12RB2	3
PRKAA2	1	IL17RD	3
PRKCA	1	IL22RA1	3
PRKCQ	1	IL23A	3
PRM1	1	IL23R	3
PRM2	1	IL27	3
PRM3	1	IL28RA	3
PRMT10	1	IL29	3
PRMT3	1	IL2RA	3
PROL1	1	IL31	3
PROSC	1	IL34	3
PRPF18	1	IMPDH1	3
PRPF3	1	INA	3
PRPF6	1	INHBC	3
PRPF8	1	INO80	3
PRR15	1	INPP5A	3
PRR15L	1	INSL6	3
PRR5L	1	INTU	3
PRTFDC1	1	IPO13	3

PSCA	1	IPO7	3
PSMA4	1	IQCE	3
PSMA7	1	IQCF6	3
PSMB1	1	IQGAP2	3
PSMB3	1	IRF2BPL	3
PSMB4	1	IRF5	3
PSMD4	1	ISM2	3
PSPC1	1	ITPKC	3
PSTPIP2	1	ITPR3	3
PTCD1	1	JAK1	3
PTCH1	1	JAK2	3
PTCH2	1	JAKMIP3	3
PTDSS1	1	JAM2	3
PTGES3	1	JMJD6	3
PTGIS	1	JUP	3
PTGR1	1	KAT2B	3
PTGS1	1	KAT5	3
PTH2R	1	KAZALD1	3
PTK2	1	KAZN	3
PTK6	1	KBTBD4	3
PTN	1	KCNH2	3
PTP4A3	1	KCNIP2	3
PTPDC1	1	KCNIP4	3
PTPN7	1	KCNJ12	3
PTPN9	1	KCNK6	3
PTPRM	1	KCNK7	3
PTPRZ1	1	KCNN4	3
PUM2	1	KCNRG	3
PVRIG	1	KCNT1	3
PVRL4	1	KCP	3
PXDC1	1	KCTD1	3
PXDN	1	KCTD15	3
PYCR2	1	KCTD2	3
PYCRL	1	KCTD6	3
PZP	1	KDM2A	3
QKI	1	KDM2B	3
QSOX1	1	KDM3B	3
RAB10	1	KDSR	3
RAB11FIP1	1	KIAA0020	3
RAB14	1	KIAA0195	3
RAB18	1	KIAA0317	3
RAB25	1	KIAA1045	3
RAB27A	1	KIAA1191	3
RAB31	1	KIAA1217	3
RAB33B	1	KIAA1328	3
RAB5B	1	KIAA1462	3
RAC1	1	KIAA1549	3

RAD51AP1	1	KIAA1551	3
RAD52	1	KIAA1737	3
RAD54B	1	KIAA1751	3
RAG2	1	KIF13A	3
RALA	1	KIF15	3
RALBP1	1	KIF17	3
RALGPS2	1	KIF1B	3
RAPGEF4	1	KIF20A	3
RASGRP3	1	KIF2C	3
RASSF4	1	KIF3C	3
RBBP8	1	KIF5A	3
RBM12B	1	KIFC1	3
RBM17	1	KIN	3
RBM18	1	KIR3DL3	3
RBM26	1	KIR3DX1	3
RBM34	1	KIRREL2	3
RBM45	1	KLC1	3
RBM8A	1	KLC3	3
RCSD1	1	KLF13	3
REG1A	1	KLF3	3
REG1B	1	KLF4	3
REPS1	1	KLHDC10	3
REST	1	KLHL10	3
RETNLB	1	KLHL9	3
REXO1L1	1	KLRG2	3
REXO1L2P	1	KNDC1	3
RFC1	1	KNTC1	3
RFESD	1	KPNA2	3
RFWD2	1	KPTN	3
RFX5	1	KRT16P3	3
RGPD4	1	KRT78	3
RGS16	1	KRTAP11-1	3
RGS22	1	KRTAP19-8	3
RHBDD2	1	KRTAP8-1	3
RHBG	1	KTI12	3
RHCG	1	LAIR2	3
RHOB	1	LARP1	3
RHOBTB3	1	LARP4B	3
RHOV	1	LARS2	3
RILP	1	LASP1	3
RIMBP2	1	LBX1	3
RIMS2	1	LCMT2	3
RIMS4	1	LCN9	3
RIOK1	1	LDB2	3
RIOK2	1	LDLRAD2	3
RIOK3	1	LEAP2	3
RIPK1	1	LENG1	3

RIPK2	1	LENG8	3
RIT1	1	LEO1	3
RIT2	1	LEPREL4	3
RNASE1	1	LETMD1	3
RNASE11	1	LFNG	3
RNASE12	1	LGI4	3
RNASE13	1	LHFPL2	3
RNASE2	1	LHX2	3
RNASE3	1	LIG1	3
RNASE4	1	LILRA1	3
RNASE7	1	LILRA4	3
RNASE8	1	LILRB3	3
RNASE9	1	LILRB4	3
RNASEH1	1	LIM2	3
RND3	1	LIMCH1	3
RNF111	1	LIMD1	3
RNF114	1	LIMD1-AS1	3
RNF115	1	LIMK1	3
RNF125	1	LIPE	3
RNF133	1	LLGL2	3
RNF138	1	LMAN1	3
RNF141	1	LMNB1	3
RNF148	1	LMX1B	3
RNF165	1	LOC100093631	3
RNF175	1	LOC100130581	3
RNF19A	1	LOC100133612	3
RNF2	1	LOC100170939	3
RNF216	1	LOC100268168	3
RNF216P1	1	LOC202181	3
RNF219	1	LOC284632	3
ROBO1	1	LOC388692	3
ROBO2	1	LOC399829	3
ROCK2	1	LOC401557	3
ROR2	1	LOC415056	3
RORA	1	LOC440461	3
RP9P	1	LOC728024	3
RPA2	1	LOC728554	3
RPGRIP1	1	LOC729020	3
RPL17	1	LOC96610	3
RPL23A	1	LPL	3
RPL39L	1	LRFN1	3
RPL41	1	LRFN3	3
RPP30	1	LRP10	3
RPP38	1	LRP11	3
RPRD1A	1	LRRC16B	3
RPRD1B	1	LRRC2	3
RPS15	1	LRRC27	3

RPS29	1	LRRC36	3
RPS2P32	1	LRRC37A4P	3
RPS3A	1	LRRC41	3
RRBP1	1	LRRC43	3
RREB1	1	LRRC47	3
RSPH3	1	LRRC66	3
RSPO2	1	LRRTM2	3
RTEL1	1	LSR	3
RTKN2	1	LTBP2	3
RTN4	1	LTBP3	3
RTP4	1	LTBP4	3
RUFY3	1	LURAP1	3
RUNDC3A	1	LURAP1L	3
RUVBL1	1	LUZP1	3
RXFP4	1	LYPD3	3
S100B	1	LYPD4	3
SALL1	1	LYRM4	3
SALL4	1	LYZL1	3
SAMD10	1	LYZL2	3
SAMD3	1	LYZL6	3
SAMD7	1	LZIC	3
SASH1	1	MAD2L1BP	3
SATB2	1	MAD2L2	3
SCAMP3	1	MADD	3
SCAPER	1	MAG	3
SCARF1	1	MAGEL2	3
SCARNA17	1	MAGOH	3
SCARNA4	1	MANF	3
SCCPDH	1	MAP1A	3
SCFD2	1	MAP2K3	3
SCN4A	1	MAP3K10	3
SCNN1B	1	MAP3K11	3
SCNN1G	1	MAP3K14	3
SCOC	1	MAP4K1	3
SCRIB	1	MAPK6	3
SCRN1	1	MAPK9	3
SCRT1	1	MAPKAPK5	3
SDC1	1	MAPKAPK5-AS1	3
SDCBP	1	MAPKBP1	3
SDCCAG8	1	MAPT	3
SDE2	1	5-Mar	3
SDF2	1	MARK4	3
SDHC	1	MARS	3
SDK1	1	MARVELD2	3
SDR42E1	1	MARVELD3	3
SDR9C7	1	MASP2	3
SEC22B	1	MAST2	3

SEC23A	1	MASTL	3
SEC61A2	1	MATR3	3
SEC61G	1	MB21D1	3
SEC62	1	MBD1	3
SECISBP2L	1	MBD6	3
SELENBP1	1	MBOAT7	3
SEMA4A	1	MCCC2	3
SEMA4F	1	MDFI	3
SENP7	1	MDN1	3
SENP8	1	MDS2	3
11-Sep	1	MED22	3
SERF1A	1	MED29	3
SERF1B	1	MED7	3
SERINC1	1	MEGF8	3
SERPINB1	1	MEIS3P1	3
SERPINB6	1	MEPE	3
SERPINE1	1	METAP1	3
SERPINF1	1	METTL14	3
SERPINF2	1	METTL2A	3
SERTAD2	1	METTL2B	3
SESN3	1	MFAP1	3
SESTD1	1	MFAP2	3
SETBP1	1	MFN2	3
SETD7	1	MFSD11	3
SETDB1	1	MGC57346	3
SF3B4	1	MGEA5	3
SFT2D2	1	MIF4GD	3
SGK1	1	MIIP	3
SGK3	1	MIR182	3
SH2D1B	1	MIR192	3
SH2D2A	1	MIR31	3
SH3D19	1	MIRLET7G	3
SHC1	1	MKNK1	3
SHC4	1	MKRN1	3
SHCBP1L	1	MKRN2	3
SHF	1	MLEC	3
SHISA4	1	MLH3	3
SHROOM3	1	MLL4	3
SIDT1	1	MLLT10	3
SIGLEC15	1	MLLT3	3
SIM1	1	MLN	3
SIN3A	1	MLX	3
SIPA1L2	1	MLXIP	3
SKAP1	1	MLXIPL	3
SKIDA1	1	MMD2	3
SKIL	1	MMEL1	3
SLAMF7	1	MMP14	3

SLC12A1	1	MMP19	3
SLC12A9	1	MOGAT3	3
SLC13A2	1	MORN1	3
SLC14A2	1	MPDZ	3
SLC15A2	1	MPHOSPH9	3
SLC16A6	1	MPP7	3
SLC17A5	1	MPPE1	3
SLC17A9	1	MRC1	3
SLC1A2	1	MRC2	3
SLC22A23	1	MRM1	3
SLC22A3	1	MRPL22	3
SLC25A21	1	MRPL43	3
SLC25A31	1	MRPL52	3
SLC25A32	1	MRPS17	3
SLC25A39	1	MRPS36	3
SLC25A44	1	MRTO4	3
SLC26A3	1	MSH3	3
SLC27A2	1	MSH5	3
SLC29A3	1	MSI1	3
SLC2A12	1	MSX2	3
SLC2A13	1	MTA2	3
SLC2A4RG	1	MTHFD1L	3
SLC30A4	1	MTHFR	3
SLC31A2	1	MTMR10	3
SLC35B1	1	MTO1	3
SLC35D2	1	MTOR	3
SLC37A2	1	MTSS1L	3
SLC38A4	1	MTX3	3
SLC39A2	1	MUC12	3
SLC39A4	1	MUC17	3
SLC39A5	1	MUC4	3
SLC40A1	1	MUL1	3
SLC41A3	1	MXD3	3
SLC43A2	1	MXRA7	3
SLC45A4	1	MYBPC3	3
SLC46A1	1	MYH16	3
SLC46A2	1	MYL2	3
SLC4A1	1	MYO15B	3
SLC4A10	1	MYO19	3
SLC4A1AP	1	MYO5A	3
SLC4A4	1	MYO5C	3
SLC50A1	1	MYOM3	3
SLC52A2	1	MYOZ3	3
SLC5A6	1	MYPOP	3
SLC5A9	1	N4BP3	3
SLC6A5	1	NAALADL1	3
SLC6A9	1	NABP2	3

SLC7A11	1	NAE1	3
SLC7A2	1	NAGLU	3
SLC9A8	1	NAIP	3
SLTM	1	NAP1L4	3
SLURP1	1	NAPA	3
SMAD2	1	NBEAL2	3
SMAD7	1	NBL1	3
SMARCA5	1	NBLA00301	3
SMCHD1	1	NBR1	3
SMG5	1	NBR2	3
SMG7	1	NCAPG2	3
SMN1	1	NCF1B	3
SMN2	1	NCMAP	3
SMOX	1	NCOR2	3
SMPDL3B	1	NDFIP1	3
SMR3A	1	NDN	3
SMR3B	1	NDST1	3
SMURF1	1	NDUFA12	3
SMURF2	1	NDUFA2	3
SMYD3	1	NDUFA4L2	3
SMYD4	1	NDUFB2	3
SND1	1	NEBL	3
SND1-IT1	1	NECAP2	3
SNORA22	1	NEIL3	3
SNORA29	1	NEXN	3
SNORA41	1	NFAT5	3
SNORA42	1	NFATC4	3
SNORA5A	1	NFIB	3
SNORD109A	1	NFKBID	3
SNORD114-26	1	NFKBIE	3
SNORD115-11	1	NHP2	3
SNORD115-20	1	NIPA2	3
SNORD115-25	1	NIPAL3	3
SNORD115-30	1	NISCH	3
SNORD115-32	1	NKD1	3
SNORD115-33	1	NKIRAS1	3
SNORD115-35	1	NKIRAS2	3
SNORD115-38	1	NKX2-5	3
SNORD115-42	1	NLRP2	3
SNORD115-44	1	NLRP7	3
SNORD116-13	1	NLRP9	3
SNORD116-21	1	NME5	3
SNORD42A	1	NMNAT1	3
SNORD42B	1	NMT1	3
SNORD4A	1	NMUR1	3
SNORD4B	1	NOB1	3
SNRNP48	1	NOM1	3

SNRPB	1	NOP16	3
SNRPE	1	NOS3	3
SNRPN	1	NOVA2	3
SNTG2	1	NPAS1	3
SNURF	1	NPAS4	3
SNX10	1	NPEPPS	3
SNX17	1	NPHP4	3
SNX27	1	NPPA	3
SNX31	1	NPPC	3
SNX6	1	NPR2	3
SNX9	1	NQO1	3
SOAT1	1	NR1D2	3
SOD2	1	NR2C1	3
SORD	1	NR2E1	3
SOWAHB	1	NRD1	3
SOX2	1	NRF1	3
SOX4	1	NRP1	3
SP6	1	NSD1	3
SPAG5	1	NSUN6	3
SPAG9	1	NT5C3L	3
SPATA19	1	NT5DC2	3
SPATA2	1	NUB1	3
SPATA20	1	NUDT1	3
SPATA5	1	NUDT4	3
SPATA5L1	1	NUDT4P1	3
SPG11	1	NUMBL	3
SPINK2	1	NUP160	3
SPINT1	1	NUP205	3
SPINT4	1	NUP43	3
SPOCD1	1	NUP85	3
SPOCK3	1	NUTF2	3
SPOPL	1	NXF1	3
SPPL2A	1	NXNL2	3
SPPL2C	1	OBP2B	3
SPRY1	1	OCLN	3
SPRY2	1	OLFM1	3
SPTAN1	1	ONECUT2	3
SPTLC1	1	OPA3	3
SQRDL	1	OR1A1	3
SRD5A3	1	OR2A4	3
SRMS	1	OR2S2	3
SRP72	1	OR5AU1	3
SRP9	1	ORAI2	3
SRRM3	1	OSBPL5	3
SRRT	1	OSTM1	3
SS18L1	1	OTOP2	3
SSH2	1	OTOP3	3

SSR1	1	OTUD3	3
SSR2	1	OVOL1	3
ST14	1	P2RX4	3
ST7	1	P2RX7	3
ST8SIA4	1	PACS1	3
ST8SIA5	1	PACSIN1	3
STAB1	1	PADI1	3
STAB2	1	PADI2	3
STAG3	1	PADI3	3
STAG3L2	1	PADI4	3
STAG3L3	1	PADI6	3
STAP1	1	PAFAH1B3	3
STAR	1	PAIP2	3
STARD5	1	PAK6	3
STAT2	1	PANK4	3
STATH	1	PARD6G	3
STC1	1	PARK7	3
STK17A	1	PARS2	3
STK31	1	PATL2	3
STK38L	1	PAX7	3
STK4	1	PBLD	3
STMN2	1	PBRM1	3
STRADA	1	PC	3
STRBP	1	PCBD2	3
STUB1	1	PCDHA1	3
STX11	1	PCGF2	3
STX19	1	PCGF6	3
STXBP5	1	PCNXL3	3
STYK1	1	PCP4	3
SUCLG1	1	PDCD11	3
SULF1	1	PDE4D	3
SULT1E1	1	PDE6A	3
SUOX	1	PDF	3
SUPT6H	1	PDGFA	3
SUPT7L	1	PDGFRL	3
SUSD1	1	PDLIM2	3
SV2A	1	PDPN	3
SYCE1	1	PDSS1	3
SYNE1	1	PDSS2	3
SYNJ2	1	PDZD7	3
SYT1	1	PEBP4	3
SYT11	1	PECAM1	3
SYT14L	1	PELI3	3
SYT15	1	PER3	3
SYT4	1	PEX14	3
TAB2	1	PFDN6	3
TAC1	1	PFKFB3	3

TACR1	1	PGD	3
TAF3	1	PGF	3
TAF6	1	PGM2	3
TAS1R2	1	PHAX	3
TAS2R16	1	PHF1	3
TAX1BP1	1	PHF7	3
TBC1D1	1	PHLDB3	3
TBC1D16	1	PHOX2B	3
TBC1D2	1	PIGO	3
TBC1D5	1	PIK3CD	3
TBCE	1	PIK3R3	3
TBCK	1	PINK1	3
TBKBP1	1	PINLYP	3
TBPL1	1	PINX1	3
TBRG4	1	PIP4K2B	3
TBX20	1	PITHD1	3
TBX21	1	PITPNC1	3
TCEB3B	1	PITPNM2	3
TCEB3C	1	PIWIL2	3
TCF12	1	PKD1L2	3
TCF15	1	PKD1L3	3
TCF7	1	PLA2G2C	3
TCF7L2	1	PLA2G4C	3
TCFL5	1	PLA2G5	3
TDO2	1	PLAUR	3
TDRD3	1	PLBD2	3
TEAD4	1	PLCB2	3
TECRL	1	PLCD3	3
TEDDM1	1	PLCH2	3
TEKT5	1	PLEKHG2	3
TEP1	1	PLEKHH1	3
TEX14	1	PLEKHH3	3
TEX15	1	PLEKHM1	3
TEX2	1	PLEKHM1P	3
TFAP2A	1	PLEKHM2	3
TFB2M	1	PLGRKT	3
TGIF1	1	PLK3	3
TGM3	1	PLOD1	3
TH	1	PLOD3	3
THNSL1	1	PLXDC1	3
THRB	1	PMS2	3
THSD4	1	PMS2L2	3
THSD7B	1	PNMA2	3
TIAM2	1	PNRC1	3
TIMM17A	1	PODN	3
TJP1	1	PODXL	3
TLK2	1	POLG2	3

TLR1	1	POLL	3
TLR10	1	POLR1C	3
TM4SF20	1	POLR1E	3
TM4SF4	1	POLR2G	3
TM7SF3	1	POM121	3
TM9SF4	1	POM121C	3
TMC1	1	POM121L2	3
TMC3	1	POMC	3
TMCO1	1	POMGNT1	3
TMED3	1	POMT2	3
TMEM101	1	POP5	3
TMEM108	1	POR	3
TMEM11	1	POU2F2	3
TMEM117	1	POU6F1	3
TMEM126A	1	PPAP2A	3
TMEM126B	1	PPARD	3
TMEM130	1	PPARGC1B	3
TMEM131	1	PPCDC	3
TMEM144	1	PPID	3
TMEM14B	1	PPIH	3
TMEM14C	1	PPIL4	3
TMEM154	1	PPM1E	3
TMEM156	1	PPM1M	3
TMEM161B	1	PPP1R12C	3
TMEM163	1	PPP1R13B	3
TMEM165	1	PPP1R13L	3
TMEM184C	1	PPP1R14A	3
TMEM186	1	PPP1R14D	3
TMEM189	1	PPP2CA	3
TMEM189-UBE	1	PPP2R2D	3
TMEM194A	1	PPP2R3C	3
TMEM194B	1	PPP2R5B	3
TMEM2	1	PPP3CC	3
TMEM200A	1	PPTC7	3
TMEM202	1	PPY	3
TMEM214	1	PQLC2	3
TMEM230	1	PRAME	3
TMEM55B	1	PRAMEF1	3
TMEM63A	1	PRAMEF12	3
TMEM67	1	PRDM16	3
TMEM69	1	PRDM2	3
TMEM79	1	PRICKLE4	3
TMEM9	1	PRKAG2	3
TMEM97	1	PRKCD	3
TMF1	1	PRKCQ	3
TMOD3	1	PRKCZ	3
TMOD4	1	PRKD2	3

TMPRSS11A	1	PRKRIP1	3
TMPRSS11B	1	PRODH2	3
TMPRSS11D	1	PROK1	3
TMPRSS11F	1	PROP1	3
TMTC2	1	PROSC	3
TNFAIP3	1	PRPF38A	3
TNFAIP8L2	1	PRPSAP1	3
TNFAIP8L3	1	PRR11	3
TNNC1	1	PRSS12	3
TNNT2	1	PRTFDC1	3
TNP1	1	PSD2	3
TNRC18	1	PSENEN	3
TOB1	1	PSMB3	3
TOE1	1	PSMC3	3
TOMM34	1	PSMD9	3
TOR1AIP1	1	PSME3	3
TOR1AIP2	1	PSPC1	3
TOR3A	1	PTCH2	3
TP53I3	1	PTCHD2	3
TPD52	1	PTGES3	3
TPD52L2	1	PTGIR	3
TPGS2	1	PTH1R	3
TPH2	1	PTMA	3
TPM3	1	PTPLA	3
TPM3P9	1	PTPMT1	3
TPMT	1	PTPN11	3
TPO	1	PTPN12	3
TPTE	1	PTPN22	3
TPTE2	1	PTPRH	3
TPTEP1	1	PTPRN2	3
TPX2	1	PTRF	3
TRABD2B	1	PTRH2	3
TRAF4	1	PUS7	3
TRAM1	1	PVR	3
TRAM1L1	1	PVRIG	3
TRAM2	1	PVRL2	3
TRAPPC6B	1	PWWP2A	3
TRAPPC9	1	PWWP2B	3
TRIM13	1	QPCTL	3
TRIM2	1	QRICH2	3
TRIM29	1	R3HDM2	3
TRIM44	1	RAB19	3
TRIM46	1	RAB1B	3
TRIM50	1	RAB24	3
TRIM51	1	RAB27A	3
TRIM74	1	RAB3B	3
TRIP6	1	RAB5A	3

TRPM7	1	RAB5B	3
TRRAP	1	RABAC1	3
TSC22D4	1	RAC1	3
TSHZ3	1	RAD17	3
TSNARE1	1	RAD51C	3
TSPAN32	1	RAD9B	3
TSPYL1	1	RAET1E	3
TSPYL6	1	RAET1G	3
TSSC1	1	RAET1L	3
TTC17	1	RAF1	3
TTC23	1	RALGDS	3
TTC24	1	RAMP2	3
TTI1	1	RAP1GAP	3
TTLL6	1	RAPGEF1	3
TTLL9	1	RAPSN	3
TUBAL3	1	RARA	3
TUBB2A	1	RARS	3
TUBB2B	1	RARS2	3
TUBB8	1	RASAL1	3
TUBE1	1	RASGEF1C	3
TUSC5	1	RASGRF2	3
TWF1	1	RASGRP4	3
TXN	1	RAX	3
TXNDC8	1	RBBP4	3
TYW5	1	RBM14	3
UACA	1	RBM15	3
UBE2T	1	RBM15B	3
UBE2Z	1	RBM17	3
UBE3A	1	RBM22	3
UBFD1	1	RBM28	3
UBR5	1	RBM42	3
UBTF	1	RBM4B	3
UBXN2B	1	RBMS2	3
UCK2	1	RBP7	3
UCMA	1	RCAN3	3
UGCG	1	RCC2	3
UGT2B4	1	RDH11	3
UGT8	1	RDH12	3
UHMK1	1	RDH13	3
UNC50	1	RECK	3
UNC93A	1	RELA	3
UPF2	1	RELB	3
UPK3B	1	RER1	3
URGCP	1	RERE	3
USF1	1	RFFL	3
USP22	1	RFT1	3
USP38	1	RFX3	3

USP42	1	RHBDD2	3
USP50	1	RHBDF2	3
USP6NL	1	RHBDL2	3
USP7	1	RHEB	3
UST	1	RHOBTB2	3
UTP18	1	RHOV	3
UTRN	1	RIN1	3
VAPA	1	RING1	3
VCPIP1	1	RINT1	3
VGLL2	1	RLN1	3
VGLL3	1	RLN2	3
VIP	1	RMND1	3
VKORC1L1	1	RMND5B	3
VNN1	1	RND2	3
VNN2	1	RNF10	3
VNN3	1	RNF11	3
VPS13B	1	RNF130	3
VPS13C	1	RNF157	3
VPS26B	1	RNF170	3
VPS8	1	RNF186	3
VSTM2A	1	RNF216	3
VTI1A	1	RNF216P1	3
VWA3B	1	RNF220	3
VWC2L	1	RNF34	3
WAC	1	RNF44	3
WASL	1	RNU6ATAC	3
WBP11	1	ROBO2	3
WBSCR16	1	ROR2	3
WBSCR17	1	RPL11	3
WDR19	1	RPL15	3
WDR24	1	RPL26L1	3
WDR26	1	RPL41	3
WDR34	1	RPL6	3
WDR72	1	RPP25	3
WDR75	1	RPS14	3
WDR81	1	RPS6KB1	3
WDSUB1	1	RPS6KL1	3
WFDC13	1	RPS8	3
WHSC1L1	1	RPSAP58	3
WIBG	1	RRAGD	3
WIPF3	1	RRP9	3
WIPI2	1	RSBN1L	3
WISP3	1	RSC1A1	3
WNK1	1	RSG1	3
WNT3	1	RSPH1	3
WSB1	1	RSPH10B	3
WT1	1	RSPH10B2	3

WT1-AS	1	RSRC2	3
XDH	1	RTN2	3
XKR7	1	RUFY2	3
XKR8	1	RUNDC1	3
XKR9	1	RUNDC3A	3
XPR1	1	RUNX1	3
YARS2	1	RUSC2	3
YIPF7	1	RXFP1	3
YME1L1	1	RXRA	3
YTHDC1	1	RYR1	3
YTHDF1	1	SAE1	3
YWHAE	1	SARDH	3
YWHAH	1	SART1	3
YWHAZ	1	SASH1	3
ZAK	1	SBNO1	3
ZAN	1	SBSN	3
ZBTB10	1	SCAMP5	3
ZBTB16	1	SCARB1	3
ZBTB26	1	SCUBE3	3
ZBTB42	1	SCYL1	3
ZBTB7B	1	SDE2	3
ZBTB7C	1	SDHAF1	3
ZC3H10	1	SDHB	3
ZC3H11A	1	SDS	3
ZC3H12C	1	SDSL	3
ZC3H12D	1	SEC11C	3
ZC3H15	1	SEC14L1	3
ZCWPW1	1	SEMA4G	3
ZCWPW2	1	9-Sep	3
ZDHHC13	1	SEPW1	3
ZDHHC14	1	SERINC2	3
ZDHHC2	1	SERINC4	3
ZDHHC4	1	SERPINB5	3
ZFAND1	1	SERPINE1	3
ZFP41	1	SF1	3
ZNF124	1	SF3B2	3
ZNF219	1	SF3B3	3
ZNF22	1	SFMBT1	3
ZNF238	1	SFRP1	3
ZNF248	1	SFXN3	3
ZNF3	1	SGOL1	3
ZNF330	1	SH2B2	3
ZNF394	1	SH3GL2	3
ZNF496	1	SHANK3	3
ZNF512	1	SHB	3
ZNF512B	1	SHF	3
ZNF513	1	SHMT2	3

ZNF536	1	SHROOM1	3
ZNF596	1	SIGLEC15	3
ZNF638	1	SIL1	3
ZNF652	1	SIPA1L3	3
ZNF654	1	SKI	3
ZNF655	1	SKIV2L2	3
ZNF670	1	SKP1	3
ZNF678	1	SLC11A2	3
ZNF703	1	SLC12A6	3
ZNF704	1	SLC12A9	3
ZNF761	1	SLC13A4	3
ZNF782	1	SLC15A4	3
ZNF800	1	SLC16A10	3
ZNF827	1	SLC16A4	3
ZNHIT1	1	SLC16A5	3
ZSWIM5	1	SLC17A5	3
		SLC1A7	3
		SLC20A2	3
		SLC22A20	3
		SLC23A1	3
		SLC24A6	3
		SLC25A16	3
		SLC25A19	3
		SLC25A31	3
		SLC25A33	3
		SLC25A34	3
		SLC25A39	3
		SLC25A45	3
		SLC25A51	3
		SLC26A2	3
		SLC29A1	3
		SLC29A2	3
		SLC2A5	3
		SLC2A6	3
		SLC2A7	3
		SLC30A4	3
		SLC30A5	3
		SLC35A1	3
		SLC35A4	3
		SLC35B2	3
		SLC35D2	3
		SLC35G5	3
		SLC37A3	3
		SLC39A13	3
		SLC39A14	3
		SLC39A5	3
		SLC39A7	3

SLC45A1	3
SLC4A1	3
SLC4A2	3
SLC4A9	3
SLC6A9	3
SLC7A7	3
SLC7A8	3
SLC8A2	3
SLFN13	3
SLFN14	3
SMAGP	3
SMARCD3	3
SMEK1	3
SMG9	3
SMO	3
SMU1	3
SMURF2	3
SNAP23	3
SNCB	3
SNORA15	3
SNORA27	3
SNORD63	3
SNRPA	3
SNRPC	3
SNRPD2	3
SNRPN	3
SNURF	3
SNX18	3
SNX3	3
SNX8	3
SORBS3	3
SORD	3
SOX4	3
SOX7	3
SPATA21	3
SPATA5L1	3
SPATS2	3
SPEN	3
SPG11	3
SPIN1	3
SPINT1	3
SPINT2	3
SPPL2A	3
SPPL2C	3
SPPL3	3
SPRED3	3
SPRY1	3

SPRY4	3
SPSB1	3
SPTBN2	3
SPTLC2	3
SQRDL	3
SRA1	3
SRP68	3
SRPK2	3
SRRM1	3
SRRM3	3
SRRT	3
SRSF10	3
SSH1	3
SSR4P1	3
ST3GAL2	3
ST6GALNAC1	3
ST6GALNAC2	3
ST8SIA6	3
STAB1	3
STAC2	3
STAG3	3
STAG3L3	3
STAM	3
STAR	3
STAT2	3
STAT3	3
STAT5A	3
STK10	3
STK32C	3
STOML2	3
STOX1	3
STRC	3
STRN4	3
STX1A	3
STX2	3
STX5	3
STXBP3	3
SULT1A2	3
SULT2A1	3
SUMF2	3
SUMO2	3
SUN1	3
SUOX	3
SUPT5H	3
SURF1	3
SURF4	3
SUSD1	3

SUSD5	3
SUV420H2	3
SVOP	3
SVOPL	3
SYMPK	3
SYNC	3
SYNDIG1L	3
SYNGAP1	3
SYNPO	3
SYT5	3
SYVN1	3
TAF15	3
TAF3	3
TAF4B	3
TAF5	3
TAF6	3
TAF9	3
TAL1	3
TAPBP	3
TAS1R2	3
TBC1D1	3
TBL2	3
TCAIM	3
TCEA3	3
TCEB3	3
TCHP	3
TCTE1	3
TCTEX1D4	3
TCTN2	3
TDH	3
TEN1	3
TERF2	3
TET1	3
TEX101	3
TEX14	3
TEX264	3
TFCP2	3
TFEB	3
TGFB1	3
TGFBR3	3
TGM5	3
THBS4	3
THNSL1	3
THOC3	3
TIGD6	3
TIMD4	3
TIMELESS	3

TIMM50	3
TINAGL1	3
TJAP1	3
TJP1	3
TK2	3
TKT	3
TLE1	3
TLK2	3
TLR1	3
TLR10	3
TLR9	3
TLX1	3
TM7SF2	3
TMC4	3
TMCO4	3
TMED8	3
TMED9	3
TMEM101	3
TMEM106A	3
TMEM11	3
TMEM116	3
TMEM147	3
TMEM150B	3
TMEM151B	3
TMEM173	3
TMEM179B	3
TMEM201	3
TMEM209	3
TMEM213	3
TMEM223	3
TMEM229B	3
TMEM33	3
TMEM41B	3
TMEM42	3
TMEM48	3
TMEM51	3
TMEM53	3
TMEM59	3
TMEM62	3
TMEM63A	3
TMEM63C	3
TMEM69	3
TMEM86B	3
TMEM8B	3
TMEM91	3
TMIE	3
TMOD3	3

TMPRSS12	3
TMUB1	3
TNFRSF10B	3
TNFRSF10C	3
TNFRSF10D	3
TNFRSF14	3
TNFRSF1B	3
TNFRSF8	3
TNNC1	3
TNNT1	3
TNPO3	3
TNS4	3
TOE1	3
TOMM40	3
TOP2A	3
TP53BP1	3
TP73	3
TPCN1	3
TPGS2	3
TPMT	3
TRAFD1	3
TRAK1	3
TRANK1	3
TRAPPC6A	3
TRIM13	3
TRIM16	3
TRIM24	3
TRIM27	3
TRIM37	3
TRIM47	3
TRIM50	3
TRIM65	3
TRIM74	3
TRIP6	3
TRPM7	3
TRPV4	3
TSEN2	3
TSEN54	3
TSGA10IP	3
TSNAXIP1	3
TSPAN1	3
TSPAN17	3
TSPAN32	3
TTBK1	3
TTBK2	3
TTC21A	3
TTC22	3

TTC25	3
TTC26	3
TTC39A	3
TTC4	3
TTC9C	3
TTLL13	3
TTLL6	3
TTYH1	3
TTYH3	3
TUBG1	3
TUSC1	3
TXNDC12	3
TXNDC15	3
TYRP1	3
U2AF2	3
UBASH3A	3
UBE2B	3
UBE2D2	3
UBE2E1	3
UBE2H	3
UBE2J1	3
UBE2O	3
UBE2S	3
UBE2Z	3
UBE4B	3
UBIAD1	3
UBN2	3
UBP1	3
UBR1	3
UBR4	3
UBTD2	3
UBTF	3
UBXN10	3
UGCG	3
UIMC1	3
ULBP1	3
UNC13D	3
UNC5A	3
UNK	3
UPK1A	3
UQCRQ	3
USH1G	3
USMG5	3
USP12	3
USP22	3
USP30	3
USP42	3

USP48	3
USP50	3
USP54	3
USP6	3
USP6NL	3
UTS2	3
VAMP3	3
VASP	3
VAT1	3
VAV2	3
VCP	3
VGLL4	3
VLDLR	3
VMP1	3
VPS13D	3
VPS25	3
VPS29	3
VPS33A	3
VPS37B	3
VPS4B	3
VSIG10L	3
VTI1B	3
VWA5B1	3
VWA7	3
WAC	3
WBSCR17	3
WBSCR22	3
WBSCR27	3
WDR1	3
WDR55	3
WDR62	3
WDR66	3
WDR76	3
WDR86	3
WDR87	3
WEE1	3
WIBG	3
WNK4	3
WNT4	3
WNT8A	3
WWP2	3
XKR6	3
XPO5	3
XRCC1	3
XRCC3	3
YARS	3
YIF1A	3

YIF1B	3
YIPF3	3
YLPM1	3
YME1L1	3
YPEL2	3
ZACN	3
ZBTB17	3
ZBTB2	3
ZBTB32	3
ZBTB40	3
ZBTB5	3
ZBTB8A	3
ZBTB8OS	3
ZC2HC1C	3
ZC3H10	3
ZC3H12D	3
ZC3H4	3
ZC3HAV1	3
ZC3HAV1L	3
ZC3HC1	3
ZCCHC17	3
ZCCHC7	3
ZCCHC8	3
ZDHHC1	3
ZDHHC19	3
ZDHHC21	3
ZDHHC22	3
ZDHHC3	3
ZDHHC4	3
ZFAND2A	3
ZFHX3	3
ZFP106	3
ZFP2	3
ZFP36	3
ZFP90	3
ZFYVE19	3
ZFYVE21	3
ZFYVE26	3
ZFYVE9	3
ZMIZ1	3
ZNF143	3
ZNF197	3
ZNF212	3
ZNF254	3
ZNF280A	3
ZNF280B	3
ZNF282	3

ZNF318	3
ZNF346	3
ZNF354A	3
ZNF354B	3
ZNF395	3
ZNF398	3
ZNF425	3
ZNF428	3
ZNF438	3
ZNF445	3
ZNF501	3
ZNF511	3
ZNF518B	3
ZNF524	3
ZNF526	3
ZNF541	3
ZNF575	3
ZNF596	3
ZNF609	3
ZNF619	3
ZNF620	3
ZNF621	3
ZNF652	3
ZNF675	3
ZNF681	3
ZNF703	3
ZNF710	3
ZNF713	3
ZNF726	3
ZNF732	3
ZNF770	3
ZNF782	3
ZNF783	3
ZNF784	3
ZNF786	3
ZNF860	3
ZNHIT1	3
ZSWIM5	3
ZYG11A	3
ZZZ3	3
AACS	2
AAMP	2
AATK	2
ABAT	2
ABCC1	2
ABCC2	2
ABCC3	2

ABCC5	2
ABCC6	2
ABCC8	2
ABCF1	2
ABHD5	2
ABI2	2
ACAA1	2
ACACB	2
ACE	2
ACP2	2
ACRBP	2
ACSF2	2
ACSL6	2
ACTL6B	2
ACTN1	2
ACTR10	2
ACTR8	2
ACVR2B	2
ADAM10	2
ADAM19	2
ADAM29	2
ADAMTS1	2
ADAMTS18	2
ADAMTS5	2
ADAMTS7	2
ADAMTSL4	2
ADARB2	2
ADAT1	2
ADH1B	2
ADH1C	2
ADH7	2
ADPRHL2	2
ADRA1B	2
ADRBK1	2
AFAP1L1	2
AFMID	2
AGFG2	2
AGPAT1	2
AGTPBP1	2
AHNAK2	2
AIF1	2
AK7	2
AKAP1	2
AKAP10	2
AKAP5	2
AKIP1	2
ALAD	2

ALDH3A1	2
ALDH3A2	2
ALG3	2
ALG8	2
AMBRA1	2
AMMECR1L	2
AMN	2
AMZ2P1	2
ANAPC10	2
ANAPC11	2
ANG	2
ANGPTL2	2
ANKRD13C	2
ANKRD13D	2
ANKRD2	2
ANKRD20A3	2
ANKRD27	2
ANKRD35	2
ANKRD40	2
ANKRD9	2
ANO10	2
ANP32A	2
ANP32E	2
ANTXR2	2
ANXA6	2
ANXA7	2
AP2M1	2
APOBEC1	2
APOH	2
AQP6	2
ARAP1	2
ARAP3	2
ARF6	2
ARFIP1	2
ARHGAP19	2
ARHGAP28	2
ARHGEF38	2
ARHGEF40	2
ARID3B	2
ARL16	2
ARL2BP	2
ARL8B	2
ARMC1	2
ARNT2	2
ARPC1B	2
ARSG	2
ART1	2

ART5	2
ASB5	2
ASCC1	2
ASCL3	2
ASPHD1	2
ASPN	2
ASPSCR1	2
ASRGL1	2
ASXL2	2
ATAD5	2
ATAT1	2
ATF7IP2	2
ATG7	2
ATL3	2
ATP11A	2
ATP12A	2
ATP5EP2	2
ATP6V1D	2
ATP8B2	2
AURKC	2
AXIN2	2
AZGP1	2
AZI1	2
AZI2	2
B3GNT7	2
B3GNT8	2
B3GNTL1	2
B4GALT6	2
B9D1	2
BACE2	2
BACH2	2
BAD	2
BAG5	2
BAIAP2	2
BAIAP2L1	2
BANK1	2
BARHL1	2
BATF	2
BCAR1	2
BCAS3	2
BCKDK	2
BCL2A1	2
BCL2L2	2
BCS1L	2
BDH2	2
BDP1	2
BEST1	2

BET1L	2
BHLHE40	2
BICD2	2
BIRC5	2
BLOC1S2	2
BMP8A	2
BMP8B	2
BMPR1B	2
BNIPL	2
BOLA2	2
BOLA2B	2
BOP1	2
BRAF	2
BRF1	2
BRI3	2
BRPF3	2
BSPRY	2
BTBD7	2
BTN2A1	2
BTN2A2	2
BTN2A3P	2
BTN3A1	2
BTN3A2	2
BTN3A3	2
BTNL8	2
BTNL9	2
C10orf54	2
C10orf55	2
C10orf62	2
C10orf68	2
C11orf16	2
C11orf84	2
C12orf49	2
C12orf56	2
C13orf35	2
C14orf102	2
C14orf126	2
C14orf159	2
C14orf162	2
C14orf79	2
C15orf27	2
C16orf45	2
C16orf53	2
C16orf78	2
C16orf87	2
C17orf62	2
C17orf72	2

C17orf77	2
C17orf79	2
C17orf99	2
C19orf18	2
C19orf40	2
C1QTNF1	2
C1QTNF2	2
C1orf146	2
C1orf213	2
C1orf43	2
C1orf56	2
C2	2
C21orf91	2
C2orf62	2
C3orf35	2
C3orf67	2
C4orf17	2
C4orf19	2
C4orf22	2
C4orf3	2
C4orf33	2
C5orf15	2
C5orf52	2
C5orf56	2
C6orf165	2
C6orf222	2
C6orf25	2
C6orf48	2
C6orf89	2
C7orf33	2
C8orf31	2
C8orf44	2
C9orf156	2
C9orf171	2
C9orf57	2
C9orf64	2
C9orf66	2
C9orf72	2
C9orf84	2
C9orf89	2
CACNA1D	2
CACNA1G	2
CACNA2D3	2
CACNG6	2
CACNG7	2
CACNG8	2
CALB2	2

CALCOCO1	2
CALM1	2
CALML5	2
CAMK2A	2
CAMK2N2	2
CANT1	2
CAP1	2
CAPNS1	2
CARD14	2
CARHSP1	2
CARKD	2
CASP8	2
CASQ2	2
CATSPER3	2
CBLN2	2
CBX1	2
CBX5	2
CCBP2	2
CCDC102A	2
CCDC102B	2
CCDC13	2
CCDC135	2
CCDC137	2
CCDC144C	2
CCDC146	2
CCDC23	2
CCDC33	2
CCDC41	2
CCDC57	2
CCDC64	2
CCDC68	2
CCDC69	2
CCDC85C	2
CCL1	2
CCL13	2
CCL17	2
CCL18	2
CCL20	2
CCL22	2
CCL7	2
CCM2	2
CCNB1IP1	2
CCNB2	2
CCNI	2
CCNI2	2
CCR1	2
CCR3	2

CCR5	2
CCR7	2
CCRL2	2
CCT4	2
CD101	2
CD177	2
CD2	2
CD226	2
CD2BP2	2
CD300C	2
CD7	2
CD83	2
CDC26	2
CDC27	2
CDC42BPB	2
CDC42EP4	2
CDC42SE1	2
CDC42SE2	2
CDH19	2
CDH23	2
CDK5RAP3	2
CDRT15	2
CDX1	2
CDX2	2
CENPJ	2
CERS2	2
CETP	2
CFB	2
CFDP1	2
CHAD	2
CHD4	2
CHD8	2
CHDH	2
CHL1	2
CHMP1B	2
CHMP6	2
CHRD	2
CHRM1	2
CHRNA10	2
CHRNA2	2
CHRNA3	2
CHRNA5	2
CHRNB4	2
CHST5	2
CHST6	2
CHTF8	2
CHTOP	2

CHUK	2
CIB2	2
CIDEA	2
CILP	2
CINP	2
CIRH1A	2
CITED4	2
CLASP2	2
CLCN1	2
CLCN2	2
CLIC1	2
CLIC6	2
CLINT1	2
CLPB	2
CLU	2
CLYBL	2
CMTM5	2
CNDP1	2
CNDP2	2
CNGA1	2
CNTN4	2
CNTNAP3	2
COG3	2
COL11A2	2
COL1A1	2
COL4A2	2
COL4A3BP	2
COL9A2	2
COLQ	2
COMMD4	2
COPG1	2
COPG2	2
COPS7A	2
COPZ1	2
COPZ2	2
COQ9	2
CORO2A	2
COX8A	2
CPE	2
CPEB3	2
CPLX3	2
CPN1	2
CPNE2	2
CPSF1	2
CPSF2	2
CPSF4L	2
CPSF7	2

CRADD	2
CREB3L4	2
CREM	2
CRIP1	2
CRIP2	2
CRTC2	2
CSDE1	2
CSF1R	2
CSF3R	2
CSH1	2
CSH2	2
CSHL1	2
CSK	2
CSMD1	2
CSNK1G1	2
CSPG4	2
CSRNP1	2
CTAGE5	2
CTAGE7P	2
CTBP2	2
CTDSP1	2
CTNNB1	2
CTNND1	2
CTRB2	2
CTSH	2
CTSK	2
CTSS	2
CUL2	2
CWF19L1	2
CX3CL1	2
CYFIP2	2
CYP11A1	2
CYP1A1	2
CYP21A2	2
CYP27A1	2
CYP2J2	2
CYP46A1	2
CYP4A11	2
CYP4B1	2
CYP4X1	2
CYP4Z1	2
CYSTM1	2
CYTH1	2
CYYR1	2
DAK	2
DAP3	2
DAPK2	2

DAPP1	2
DCAF5	2
DCDC2	2
DCLK2	2
DCTPP1	2
DCUN1D2	2
DCXR	2
DDAH2	2
DDB2	2
DDHD2	2
DDR1	2
DDX52	2
DDX56	2
DEGS2	2
DEM1	2
DENND3	2
DENND4A	2
DENND4B	2
DENND5A	2
DEXI	2
DGAT1	2
DHX58	2
DIAPH1	2
DIMT1	2
DLEC1	2
DLG5	2
DLGAP1-AS1	2
DLGAP3	2
DLK1	2
DLX4	2
DNAH10	2
DNAH17	2
DNAI1	2
DNAI2	2
DNAJA2	2
DNAJA4	2
DNAJB14	2
DNAJC14	2
DNAJC25	2
DNAJC4	2
DNMBP	2
DNPEP	2
DOCK5	2
DOCK7	2
DOK6	2
DOM3Z	2
DPCR1	2

DPF3	2
DPRX	2
DPYSL2	2
DSG2	2
DUSP1	2
DUSP14	2
DUSP5	2
DUXA	2
DYNC1H1	2
DYRK1A	2
E2F3	2
EAF1	2
EARS2	2
EBNA1BP2	2
ECE2	2
ECM1	2
EDEM1	2
EDN2	2
EFHD1	2
EFS	2
EGFL8	2
EIF1B	2
EIF2C1	2
EIF2C2	2
EIF2C3	2
EIF2S1	2
EIF4G1	2
EIF5AL1	2
ELOVL1	2
EMCN	2
EML1	2
EMP2	2
ENDOV	2
ENGASE	2
ENSA	2
ENTPD4	2
EPB41L3	2
EPB41L4A	2
EPCAM	2
EPHB3	2
EPHX1	2
EPHX2	2
EPHX4	2
EPN2	2
EPN3	2
EPPK1	2
ERC2	2

ERH	2
ERMAP	2
ERO1L	2
ESCO1	2
ESR2	2
EVI5	2
EVL	2
EXD2	2
EXOSC1	2
F13A1	2
F2	2
FA2H	2
FABP2	2
FADS1	2
FADS2	2
FADS3	2
FAIM2	2
FAM102B	2
FAM117B	2
FAM13A	2
FAM160A1	2
FAM178A	2
FAM188A	2
FAM189A2	2
FAM18A	2
FAM190A	2
FAM203A	2
FAM214A	2
FAM219A	2
FAM219B	2
FAM3D	2
FAM47E	2
FAM59A	2
FAM63A	2
FAM63B	2
FAM69A	2
FAM81A	2
FAM83G	2
FAM83H	2
FAM96A	2
FARP1	2
FARS2	2
FAT2	2
FBLN5	2
FBRS	2
FBXL19	2
FBXO15	2

FBXO22	2
FBXO24	2
FBXO34	2
FBXW10	2
FCGR1A	2
FCGR1B	2
FCHSD1	2
FEN1	2
FER1L5	2
FERMT2	2
FGF1	2
FGL2	2
FHDC1	2
FKBP2	2
FKBP3	2
FKBPL	2
FLJ46300	2
FLOT1	2
FLRT1	2
FLT1	2
FLT3	2
FMNL3	2
FN3K	2
FN3KRP	2
FNDC4	2
FNDC9	2
FNIP1	2
FNTB	2
FOXE1	2
FOXJ3	2
FOXK2	2
FOXM1	2
FOXN3	2
FOXO3B	2
FRMD4B	2
FSTL4	2
FUOM	2
FUS	2
FUT8	2
FXN	2
FYCO1	2
FZD10	2
FZD5	2
FZD7	2
GAA	2
GABARAPL2	2
GABBR1	2

GABBR2	2
GADL1	2
GALNT1	2
GALNT10	2
GALNTL1	2
GALNTL5	2
GALP	2
GAS8	2
GATAD2B	2
GBA3	2
GBAP1	2
GBE1	2
GCN1L1	2
GCNT1	2
GCNT3	2
GDA	2
GDPD3	2
GFI1	2
GFOD1	2
GGA2	2
GGTA1P	2
GGTLC2	2
GHDC	2
GJB3	2
GJB5	2
GJD4	2
GKAP1	2
GLG1	2
GLT8D1	2
GM2A	2
GML	2
GNA13	2
GNA14	2
GNG10	2
GNPDA1	2
GNPNAT1	2
GNRH1	2
GNRHR2	2
GNS	2
GOLGA5	2
GOLGA6A	2
GOLGA8B	2
GOLM1	2
GOLPH3L	2
GON4L	2
GOSR2	2
GPATCH1	2

GPBAR1	2
GPBP1	2
GPIHBP1	2
GPM6A	2
GPR1	2
GPR114	2
GPR12	2
GPR132	2
GPR137	2
GPR18	2
GPR183	2
GPR68	2
GPRC5C	2
GPRIN3	2
GPT	2
GPT2	2
GPX2	2
GPX3	2
GPX8	2
GRID2	2
GRINA	2
GRK1	2
GRPEL2	2
GRTP1	2
GSDMD	2
GSTCD	2
GSTK1	2
GTF2E2	2
GUCA1A	2
GZMK	2
H2AFV	2
HACL1	2
HAND1	2
HAS3	2
HCP5	2
HCRT	2
HDAC3	2
HDHD3	2
HEATR5A	2
HECTD1	2
HEMGN	2
HENMT1	2
HERC3	2
HEXA	2
HEXDC	2
HEYL	2
HFE2	2

HGC6.3	2
HGS	2
HHIPL1	2
HIGD1A	2
HIPK2	2
HIRIP3	2
HIST1H1E	2
HIST1H2AE	2
HIST1H2AK	2
HIST1H2BD	2
HIST1H2BG	2
HIST1H2BH	2
HIST1H2BI	2
HIST1H2BJ	2
HIST1H2BL	2
HIST1H2BM	2
HIST1H3E	2
HIST1H3F	2
HIST1H3G	2
HIST1H3H	2
HIST1H4D	2
HIST1H4E	2
HIST1H4G	2
HIST1H4I	2
HIST1H4K	2
HIST2H2BF	2
HKDC1	2
HLA-B	2
HLA-E	2
HLA-F	2
HMGB1	2
HMGN4	2
HMGXB3	2
HMP19	2
HNF1B	2
HNRNPA1	2
HNRNPA1P10	2
HNRNPR	2
HOMEZ	2
HOOK1	2
HOXB13	2
HOXB6	2
HOXB7	2
HOXB8	2
HOXB9	2
HPCAL4	2
HPGDS	2

HRASLS2	2
HRH1	2
HRH2	2
HRH4	2
HSBP1	2
HSD17B3	2
HSD3B7	2
HSP90AA1	2
HSP90AB4P	2
HSPA1B	2
HSPA1L	2
HSPA2	2
HSPB9	2
HSPD1	2
HSPE1	2
HTR3E	2
HYI	2
ICAM2	2
IFT172	2
IFT88	2
IGDCC3	2
IGDCC4	2
IGSF21	2
IGSF3	2
IL13	2
IL17B	2
IL17D	2
IL17RB	2
IL25	2
IL3	2
IL4	2
IL5	2
IMP3	2
IMPA2	2
INCENP	2
ING1	2
INO80C	2
INO80E	2
INTS12	2
INTS3	2
INTS9	2
IPO11	2
IPPK	2
IQSEC1	2
IRF1	2
ISLR	2
ISLR2	2

ISY1	2
ITGA3	2
ITGA5	2
ITGA9	2
ITGAL	2
ITGAM	2
ITGAX	2
ITGB3	2
ITK	2
ITPK1	2
ITPR1	2
JAG2	2
JARID2	2
JDP2	2
JPH4	2
JRK	2
JTB	2
KARS	2
KAT2A	2
KBTBD5	2
KBTBD6	2
KC6	2
KCNA2	2
KCNC1	2
KCNC4	2
KCNE1	2
KCNH3	2
KCNH4	2
KCNH6	2
KCNIP1	2
KCNJ11	2
KCNK13	2
KCNK4	2
KCNMB1	2
KCNQ1	2
KCNQ1OT1	2
KCTD13	2
KCTD14	2
KCTD21	2
KDM4A	2
KIAA0141	2
KIAA0247	2
KIAA0319L	2
KIAA0355	2
KIAA0907	2
KIAA1239	2
KIAA1468	2

KIAA1875	2
KIAA1919	2
KIF19	2
KIF22	2
KIF3A	2
KIF4B	2
KIFC2	2
KRBA1	2
KRT14	2
KRT15	2
KRT18	2
KRT79	2
KRT8	2
KRTCAP3	2
L3MBTL4	2
LAG3	2
LAMA1	2
LAMA3	2
LAMTOR3	2
LARS	2
LCORL	2
LDB3	2
LDHD	2
LEPRE1	2
LEPROTL1	2
LETM2	2
LGALS3BP	2
LGMN	2
LINC00173	2
LINC00346	2
LINGO2	2
LIPC	2
LIX1L	2
LMAN1L	2
LMO1	2
LMOD3	2
LNX1	2
LOC100130015	2
LOC100130987	2
LOC100132354	2
LOC100216545	2
LOC149134	2
LOC257358	2
LOC283050	2
LOC284379	2
LOC285501	2
LOC285768	2

LOC286359	2
LOC440354	2
LOC653786	2
LOXL1	2
LOXL2	2
LPAR5	2
LRRC17	2
LRRC37A2	2
LRRC37A3	2
LRRC37B	2
LRRC3B	2
LRRC59	2
LRTM1	2
LSM11	2
LSM14A	2
LSM2	2
LST1	2
LTA	2
LTBR	2
LTF	2
LUC7L3	2
LY6D	2
LY6E	2
LY6G5B	2
LY6G5C	2
LY6G6C	2
LY6G6F	2
LY6K	2
LY86	2
LYN	2
LYNX1	2
LYPD2	2
LYPD5	2
LYPLA1	2
LYZL4	2
MAB21L3	2
MACF1	2
MACROD1	2
MAD1L1	2
MAGEF1	2
MANBA	2
MAP2K1	2
MAP4K2	2
MAP7D1	2
MAPK1IP1L	2
MAPK3	2
MAPK7	2

10-Mar	2
3-Mar	2
MARK2	2
MARK3	2
MAX	2
MCC	2
MCF2L	2
MCM7	2
MDC1	2
MDGA1	2
MDK	2
MED8	2
MEGF11	2
MEGF6	2
MEOX1	2
MEST	2
METRNL	2
METTL21A	2
METTL3	2
MGAT1	2
MGAT2	2
MIA2	2
MICA	2
MICB	2
MICU1	2
MIR106B	2
MIR128-2	2
MIR130A	2
MIR193A	2
MIR208A	2
MIR26B	2
MIR29A	2
MIR30E	2
MIR519A2	2
MKLN1	2
MLKL	2
MLLT4	2
MMRN1	2
MOAP1	2
MOB3B	2
MOB4	2
MOBP	2
MOK	2
MON1B	2
MORF4L1	2
MORN4	2
MOSPD3	2

MPL	2
MPP2	2
MPP3	2
MPP5	2
MRPL14	2
MRPL27	2
MRPL48	2
MRPS23	2
MRPS6	2
MSANTD3	2
MSMO1	2
MSRA	2
MSTO1	2
MSTO2P	2
MT1B	2
MT1DP	2
MT1E	2
MT1G	2
MT1H	2
MT1IP	2
MT1JP	2
MT1L	2
MT1M	2
MT1X	2
MT2A	2
MTA1	2
MTCH1	2
MTF2	2
MTG1	2
MTHFD1	2
MTMR6	2
MTRF1	2
MTTP	2
MTX1	2
MUC21	2
MVP	2
MYADM	2
MYCBP	2
MYCL1	2
MYD88	2
MYH11	2
MYH7	2
MYLK3	2
MYLPF	2
MYO1E	2
MYO5B	2
MYO6	2

MYOT	2
MYOZ1	2
MYOZ2	2
MYSM1	2
NAPEPLD	2
NAPRT1	2
NARF	2
NAT9	2
NBEAL1	2
NCAM2	2
NCAPD2	2
NCBP1	2
NCL	2
NCR3	2
NDE1	2
NDRG2	2
NDUFAB1	2
NDUFB3	2
NDUFV2	2
NEDD4	2
NEDD9	2
NEK10	2
NEK4	2
NEK6	2
NEMF	2
NETO1	2
NEU1	2
NEURL1B	2
NFE2L1	2
NFYC	2
NGB	2
NGFR	2
NGRN	2
NHLH2	2
NIF3L1	2
NINJ1	2
NIPAL1	2
NIPAL4	2
NKX2-4	2
NLN	2
NLRC4	2
NLRC5	2
NLRP10	2
NLRP12	2
NLRP13	2
NLRP4	2
NLRP5	2

NLRP8	2
NME2	2
NMRK1	2
NOD2	2
NOL7	2
NOL8	2
NOMO3	2
NOP2	2
NOP58	2
NPB	2
NPC1	2
NPC1L1	2
NPLOC4	2
NPNT	2
NPR1	2
NPTX2	2
NR1H3	2
NR5A1	2
NRAS	2
NRBP1	2
NRG2	2
NRG4	2
NRIP3	2
NRXN2	2
NT5C1A	2
NTN4	2
NUCB2	2
NUFIP1	2
NUFIP2	2
NUGGC	2
NUP107	2
NUP210	2
NUP98	2
NUPL1	2
NYNRIN	2
ODF3	2
OGDH	2
OGN	2
OLFML3	2
OMA1	2
OPN4	2
OR11H1	2
OR2T10	2
OR2T11	2
OR2V2	2
OR4E2	2
OR52W1	2

OR5P2	2
ORAI3	2
ORC2	2
ORMDL2	2
OSBPL10	2
OSBPL1A	2
OSBPL7	2
OSCP1	2
OSGEP	2
OSTF1	2
OTUB1	2
OXSM	2
OXSR1	2
P4HA2	2
P4HB	2
PABPC3	2
PACS2	2
PACSIN3	2
PALLD	2
PAN3	2
PAOX	2
PAPSS1	2
PARD3	2
PARP12	2
PAX2	2
PCCA	2
PCCB	2
PCDH1	2
PCDH12	2
PCDHA2	2
PCDHA3	2
PCDHA4	2
PCDHA5	2
PCDHA6	2
PCDHA7	2
PCDHA8	2
PCDHA9	2
PCDHGA1	2
PCDHGA10	2
PCDHGA11	2
PCDHGA12	2
PCDHGA2	2
PCDHGA3	2
PCDHGA4	2
PCDHGA5	2
PCDHGA6	2
PCDHGA7	2

PCDHGA8	2
PCDHGA9	2
PCDHGB1	2
PCDHGB2	2
PCDHGB3	2
PCDHGB4	2
PCDHGB5	2
PCDHGB6	2
PCDHGB7	2
PCDHGC3	2
PCDHGC4	2
PCDHGC5	2
PCID2	2
PCOLCE	2
PCSK9	2
PCYOX1L	2
PCYT2	2
PDCD2L	2
PDCD5	2
PDCD7	2
PDE2A	2
PDGFRB	2
PDIA3	2
PDLIM4	2
PDLIM5	2
PDX1	2
PEBP1	2
PEPD	2
PFDN1	2
PGAM1	2
PGAP2	2
PGS1	2
PHKG2	2
PHTF1	2
PHTF2	2
PIAS3	2
PIGN	2
PIK3C2A	2
PIK3C3	2
PILRA	2
PIM1	2
PIP4K2C	2
PIP5K1B	2
PITPNB	2
PITX1	2
PIWIL1	2
PKD2L1	2

PKD2L2	2
PLA2G16	2
PLA2G4D	2
PLA2G4F	2
PLAC8L1	2
PLAU	2
PLCD1	2
PLCD4	2
PLCG2	2
PLD4	2
PLEK2	2
PLEKHA2	2
PLEKHB1	2
PLEKHG1	2
PLEKHG3	2
PLK1S1	2
PLLP	2
PLXNC1	2
PMM2	2
PMP22	2
PNKD	2
PNN	2
PNP	2
PNPO	2
POGZ	2
POLE2	2
POLR2C	2
POLR2H	2
POLR3A	2
POM121L1P	2
POU4F3	2
POU5F1	2
PPA2	2
PPAPDC1B	2
PPARG	2
PPARGC1A	2
PPIB	2
PPIL1	2
PPIL3	2
PPP1R16A	2
PPP1R3E	2
PPP1R9B	2
PPP2R2A	2
PPP2R5C	2
PPP2R5E	2
PPP3CA	2
PPP4C	2

PPP4R1	2
PPT1	2
PPT2	2
PRAP1	2
PRDX5	2
PRG2	2
PRG3	2
PRKCA	2
PRKCG	2
PRKG2	2
PRPF3	2
PRPF4	2
PRPF40B	2
PRPSAP2	2
PRR14	2
PRR15L	2
PRRT1	2
PRRT2	2
PSCA	2
PSD3	2
PSG4	2
PSG5	2
PSMA3	2
PSMA4	2
PSMB2	2
PSMB4	2
PSMC6	2
PSMD4	2
PSMD7	2
PSORS1C2	2
PSORS1C3	2
PTCD1	2
PTGFRN	2
PTGR1	2
PTK2	2
PTK2B	2
PTP4A1	2
PTP4A3	2
PTPDC1	2
PTPLAD1	2
PTPN7	2
PTPN9	2
PTPRF	2
PTPRM	2
PTTG1	2
PXN	2
PYCR1	2

PYCR2	2
PYCRL	2
QPRT	2
RAB11A	2
RAB11FIP4	2
RAB13	2
RAB15	2
RAB20	2
RAB25	2
RAB27B	2
RAB2B	2
RAB31	2
RAB35	2
RAB37	2
RAB40B	2
RAB43	2
RAB5C	2
RAB6A	2
RAD50	2
RALBP1	2
RANBP17	2
RANBP9	2
RAP1B	2
RAPGEF6	2
RASA3	2
RASGEF1B	2
RASSF3	2
RBFOX3	2
RBM27	2
RBM8A	2
RBMS3	2
RCAN1	2
RCBTB1	2
RCOR1	2
REPS1	2
RESP18	2
RFC5	2
RFWD3	2
RFX5	2
RGS20	2
RGS3	2
RGS9BP	2
RHBDL3	2
RHOG	2
RHOT1	2
RHPN2	2
RIC3	2

RIMBP2	2
RIMKLA	2
RIMS3	2
RIN3	2
RIT1	2
RNASE1	2
RNASE11	2
RNASE12	2
RNASE13	2
RNASE2	2
RNASE3	2
RNASE4	2
RNASE7	2
RNASE8	2
RNASE9	2
RNASET2	2
RNF111	2
RNF126P1	2
RNF135	2
RNF14	2
RNF152	2
RNF175	2
RNF19B	2
RNF213	2
RNF25	2
RNF40	2
RNF5	2
RNF8	2
ROBO1	2
ROCK1	2
RPGRIP1	2
RPL27A	2
RPL4	2
RPLP0	2
RPN1	2
RPS13	2
RPS27	2
RPS29	2
RPS6KA5	2
RPTOR	2
RQCD1	2
RRAS2	2
RRM1	2
RRP12	2
RSPH3	2
RTN3	2
RTP3	2

RTTN	2
RWDD2B	2
RXFP4	2
RXRB	2
RYR3	2
S100A14	2
S100A16	2
SACM1L	2
SALL2	2
SAP130	2
SAP30L	2
SATB1	2
SBDS	2
SCAMP2	2
SCAPER	2
SCARA3	2
SCARA5	2
SCARB2	2
SCARNA10	2
SCARNA17	2
SCARNA4	2
SCD	2
SCFD2	2
SCG5	2
SCGB1A1	2
SCGB1C1	2
SCGB1D2	2
SCGB1D4	2
SCGB2A2	2
SCMH1	2
SCN10A	2
SCN11A	2
SCN4A	2
SCN5A	2
SCNN1A	2
SCPEP1	2
SCRIB	2
SCRT1	2
SCUBE2	2
SDHAF2	2
SDK1	2
SDK2	2
SDR42E1	2
SEC23A	2
SEC24D	2
SECTM1	2
SELENBP1	2

SEMA4A	2
SEMA7A	2
SENP8	2
15-Sep	2
SEPHS2	2
1-Sep	2
11-Sep	2
8-Sep	2
SERPINB12	2
SERPINB13	2
SETD1A	2
SETDB1	2
SETX	2
SEZ6L2	2
SF3B1	2
SFPQ	2
SFXN1	2
SGK1	2
SGK3	2
SGPP1	2
SGTB	2
SH3BP5	2
SH3GLB2	2
SH3RF2	2
SHARPIN	2
SHROOM3	2
SIN3A	2
SIRT7	2
SIX1	2
SIX4	2
SKAP1	2
SLAIN2	2
SLC11A1	2
SLC12A3	2
SLC14A2	2
SLC16A6	2
SLC19A3	2
SLC22A13	2
SLC22A14	2
SLC22A15	2
SLC22A17	2
SLC22A18	2
SLC22A18AS	2
SLC22A4	2
SLC22A5	2
SLC22A8	2
SLC24A4	2

SLC25A15	2
SLC25A29	2
SLC25A37	2
SLC25A44	2
SLC26A10	2
SLC27A2	2
SLC27A3	2
SLC28A3	2
SLC2A14	2
SLC2A3	2
SLC30A6	2
SLC31A1	2
SLC35B1	2
SLC35E3	2
SLC36A1	2
SLC36A2	2
SLC36A3	2
SLC38A10	2
SLC39A1	2
SLC39A11	2
SLC39A2	2
SLC39A4	2
SLC39A8	2
SLC43A1	2
SLC44A4	2
SLC45A4	2
SLC47A1	2
SLC47A2	2
SLC4A4	2
SLC4A7	2
SLC4A8	2
SLC51B	2
SLC52A2	2
SLC5A10	2
SLC5A3	2
SLC6A17	2
SLC6A20	2
SLC6A7	2
SLC7A2	2
SLC7A9	2
SLC9A3R1	2
SLC9B1	2
SLFN11	2
SLIT2	2
SLIT3	2
SLTM	2
SLU7	2

SLURP1	2
SMA4	2
SMAD2	2
SMG5	2
SMOC2	2
SMURF1	2
SNCA	2
SNORA28	2
SNORA3	2
SNORA38	2
SNORA41	2
SNORA42	2
SNORA59A	2
SNORA75	2
SNORD109A	2
SNORD116-13	2
SNORD116-21	2
SNORD117	2
SNORD52	2
SNTB2	2
SNX1	2
SNX17	2
SNX33	2
SOCS2	2
SOCS3	2
SOST	2
SOWAHB	2
SOX30	2
SP6	2
SPAG9	2
SPARC	2
SPATA18	2
SPATA20	2
SPATA4	2
SPATC1	2
SPECC1	2
SPG21	2
SPN	2
SPTB	2
SPTLC1	2
SRGN	2
SRP9	2
SS18L2	2
SSH3	2
SSPO	2
SSR2	2
STAT5B	2

STBD1	2
STC2	2
STIL	2
STIM1	2
STIM2	2
STIP1	2
STK24	2
STK33	2
STK36	2
STK38	2
STK40	2
STRA13	2
STRA6	2
STRADA	2
STRN3	2
STX1B	2
STYX	2
SUCLG2	2
SUGT1P3	2
SULT1A3	2
SULT1A4	2
SUMF1	2
SUMO1	2
SUPV3L1	2
SUSD3	2
SUZ12	2
SUZ12P1	2
SWAP70	2
SYNE2	2
SYNGR2	2
SYNPO2	2
SYNPO2L	2
SYNRG	2
SYT11	2
SYT6	2
SYT7	2
SYTL3	2
TACC2	2
TACR3	2
TACSTD2	2
TAOK3	2
TAPBPL	2
TAS2R39	2
TAS2R40	2
TAS2R41	2
TBC1D10B	2
TBC1D16	2

TBC1D2	2
TBC1D21	2
TBC1D2B	2
TBC1D3B	2
TBCD	2
TBCK	2
TBK1	2
TBKBP1	2
TBPL2	2
TBX21	2
TBX6	2
TBXAS1	2
TCEB1	2
TCERG1L	2
TCF4	2
TCOF1	2
TDP1	2
TDP2	2
TDRD12	2
TDRD7	2
TDRD9	2
TEC	2
TECPR2	2
TEFM	2
TEKT5	2
TEP1	2
TET2	2
TEX10	2
TEX15	2
TEX2	2
TFDP1	2
TGFBR2	2
THG1L	2
THRAP3	2
THRB	2
TIAM2	2
TIE1	2
TIGD2	2
TIMM17A	2
TIMM21	2
TIMP2	2
TMC6	2
TMC7	2
TMCC3	2
TMCO2	2
TMCO3	2
TMCO7	2

TMED4	2
TMED5	2
TMEFF1	2
TMEM121	2
TMEM125	2
TMEM130	2
TMEM132C	2
TMEM132D	2
TMEM138	2
TMEM139	2
TMEM154	2
TMEM170A	2
TMEM181	2
TMEM186	2
TMEM2	2
TMEM202	2
TMEM216	2
TMEM217	2
TMEM231	2
TMEM55B	2
TMEM61	2
TMEM92	2
TMEM9B	2
TMOD4	2
TMTC4	2
TMX2	2
TNF	2
TNFAIP2	2
TNFAIP8L2	2
TNFRSF11A	2
TNIP1	2
TNRC6C	2
TOB1	2
TOX4	2
TPM3	2
TPM3P9	2
TPTE2	2
TRAF3	2
TRAM1L1	2
TRAPPC6B	2
TRAPPC9	2
TRERF1	2
TRIM16L	2
TRIM22	2
TRIM25	2
TRIM35	2
TRIM41	2

TRIM5	2
TRIM52	2
TRIM6	2
TRIM6-TRIM34	2
TRIM62	2
TRIM7	2
TRIP11	2
TRIT1	2
TRMT61A	2
TRRAP	2
TSC22D1	2
TSC22D4	2
TSNARE1	2
TSPAN10	2
TSSK1B	2
TTC1	2
TTC7B	2
TTF1	2
TTLL11	2
TTLL4	2
TTYH2	2
TUB	2
TUBB6	2
TUBGCP3	2
TXK	2
TYW1	2
TYW1B	2
UBA2	2
UBE2D3	2
UBE2E2	2
UBE2N	2
UBFD1	2
UBR7	2
UBTD1	2
UBXN7	2
UCP2	2
ULK2	2
ULK3	2
ULK4	2
UNC13B	2
UNC5C	2
USH1C	2
USP16	2
USP32P2	2
USP36	2
USP44	2
USP53	2

USP7	2
USPL1	2
UTP6	2
VAC14	2
VANGL1	2
VAPA	2
VCPIP1	2
VDAC1	2
VEGFA	2
VEGFC	2
VIL1	2
VILL	2
VIPR1	2
VN1R4	2
VPS39	2
VPS4A	2
VTCN1	2
WARS	2
WDFY3	2
WDFY3-AS2	2
WDHD1	2
WDR12	2
WDR17	2
WDR20	2
WDR25	2
WDR31	2
WDR45L	2
WDR59	2
WDR60	2
WDR88	2
WDR89	2
WHSC1L1	2
WNK2	2
WNT3	2
WNT5A	2
WNT8B	2
WSB2	2
WT1	2
WT1-AS	2
XCR1	2
XPA	2
XPO1	2
XPO4	2
XRN2	2
YPEL3	2
YTHDC2	2
ZADH2	2

ZAN	2
ZBTB1	2
ZBTB12	2
ZCCHC24	2
ZCWPW1	2
ZCWPW2	2
ZDHHC14	2
ZDHHC16	2
ZDHHC5	2
ZFP14	2
ZFP161	2
ZFP41	2
ZFP82	2
ZIM3	2
ZMAT4	2
ZMIZ2	2
ZMYM4	2
ZMYM6	2
ZNF135	2
ZNF137P	2
ZNF146	2
ZNF160	2
ZNF195	2
ZNF219	2
ZNF248	2
ZNF26	2
ZNF260	2
ZNF264	2
ZNF28	2
ZNF286B	2
ZNF3	2
ZNF311	2
ZNF320	2
ZNF329	2
ZNF331	2
ZNF362	2
ZNF37A	2
ZNF384	2
ZNF385A	2
ZNF394	2
ZNF407	2
ZNF415	2
ZNF417	2
ZNF436	2
ZNF467	2
ZNF468	2
ZNF48	2

ZNF484	2
ZNF513	2
ZNF525	2
ZNF528	2
ZNF536	2
ZNF552	2
ZNF565	2
ZNF574	2
ZNF578	2
ZNF587	2
ZNF595	2
ZNF600	2
ZNF605	2
ZNF611	2
ZNF654	2
ZNF655	2
ZNF662	2
ZNF671	2
ZNF677	2
ZNF701	2
ZNF702P	2
ZNF718	2
ZNF746	2
ZNF750	2
ZNF761	2
ZNF765	2
ZNF766	2
ZNF767	2
ZNF768	2
ZNF780A	2
ZNF780B	2
ZNF785	2
ZNF787	2
ZNF792	2
ZNF808	2
ZNF814	2
ZNF816	2
ZNF827	2
ZNF839	2
ZNF862	2
ZNRF1	2
ZSCAN1	2
ZSCAN18	2
ZYX	2
AAGAB	1
AATF	1
ABCA1	1

ABCA5	1
ABCA8	1
ABCC13	1
ABCC4	1
ABCC6P1	1
ABCC6P2	1
ABCE1	1
ABHD15	1
ACACA	1
ACAD8	1
ACADM	1
ACAP2	1
ACSS3	1
ACY3	1
ADAM12	1
ADAMTS4	1
ADAMTS9	1
ADAMTSL3	1
ADHFE1	1
ADPGK	1
ADRA1A	1
ADSSL1	1
AEBP1	1
AGAP1	1
AGAP11	1
AGAP5	1
AGL	1
AHRR	1
AIM1	1
AIP	1
AJAP1	1
AKAP2	1
AKNA	1
AKR1C1	1
AKR1C2	1
AKR1C3	1
AKR1C4	1
AKR1E2	1
ALDH1A2	1
ALDH3B2	1
ALG11	1
ALG1L	1
ALPI	1
ALPK1	1
ALPP	1
ALPPL2	1
AMDHD1	1

AMFR	1
AMPD3	1
ANGPTL3	1
ANGPTL5	1
ANKRD10	1
ANKRD13B	1
ANKRD20A5P	1
ANKRD20A8P	1
ANKRD23	1
ANKRD28	1
ANKRD39	1
ANKRD5	1
ANKRD50	1
ANKZF1	1
ANO1	1
ANPEP	1
ANXA10	1
ANXA11	1
ANXA13	1
ANXA5	1
AP1S3	1
AP3B1	1
AP3B2	1
AP3S2	1
APAF1	1
APH1B	1
APOA1BP	1
AQP9	1
ARAP2	1
ARF5	1
ARFGEF1	1
ARGLU1	1
ARHGAP22	1
ARHGAP24	1
ARHGAP30	1
ARHGAP32	1
ARHGAP42	1
ARHGEF7	1
ARID4B	1
ARL15	1
ARL6IP1	1
ARMC5	1
ARMC9	1
ARPC2	1
ARRB1	1
ASAP1	1
ASB1	1

ASB17	1
ASL	1
ASTN2	1
ASXL1	1
ATAD2	1
ATE1	1
ATG12	1
ATG16L1	1
ATG16L2	1
ATG4B	1
ATIC	1
ATL1	1
ATM	1
ATP2B1	1
ATP2B2	1
ATP5F1	1
ATP6V1H	1
ATP7B	1
ATP8A1	1
ATP8A2	1
ATP8B4	1
ATP9A	1
ATXN7	1
AVP	1
AVPR1A	1
B4GALT3	1
BAG3	1
BARX2	1
BBS12	1
BBS2	1
BCAR3	1
BCAT1	1
BCL11B	1
BCL2L1	1
BDH1	1
BFAR	1
BIRC2	1
BIRC3	1
BIRC6	1
BLM	1
BLOC1S1	1
BLVRA	1
BMI1	1
BMP2K	1
BMPR1A	1
BOD1	1
BOK	1

BPGM	1
BRCA2	1
BRD2	1
BRD9	1
BST1	1
BTC	1
BTD	1
BTF3	1
BTG1	1
BZRAP1	1
C10orf11	1
C10orf57	1
C10orf90	1
C10orf99	1
C11orf24	1
C11orf45	1
C11orf49	1
C11orf65	1
C11orf9	1
C12orf29	1
C12orf50	1
C14orf180	1
C15orf59	1
C16orf72	1
C16orf80	1
C18orf26	1
C18orf54	1
C1QTNF9B-AS1	1
C1orf109	1
C1orf180	1
C1orf192	1
C1orf85	1
C1orf88	1
C20orf112	1
C20orf173	1
C20orf24	1
C20orf26	1
C21orf7	1
C2orf16	1
C2orf54	1
C2orf78	1
C2orf82	1
C2orf83	1
C3orf14	1
C3orf20	1
C3orf32	1
C3orf33	1

C5	1
C5orf22	1
C5orf27	1
C5orf42	1
C5orf47	1
C6orf10	1
C6orf118	1
C6orf120	1
C6orf123	1
C7orf13	1
C8A	1
C8orf47	1
C8orf48	1
CA12	1
CAB39L	1
CABLES1	1
CACHD1	1
CACNG1	1
CACNG4	1
CADM2	1
CADPS	1
CALML4	1
CAMK2B	1
CAMK2D	1
CAMK2G	1
CAPN10	1
CAPNS2	1
CAPS2	1
CARD6	1
CAST	1
CATSPERB	1
CBX3	1
CC2D2A	1
CCBL2	1
CCDC112	1
CCDC113	1
CCDC38	1
CCER1	1
CCNC	1
CCND1	1
CCNG2	1
CCNYL1	1
CCT3	1
CCT8	1
CD276	1
CD5	1
CD6	1

CD63	1
CDADC1	1
CDC16	1
CDC7	1
CDCA8	1
CDH11	1
CDH13	1
CDH20	1
CDHR1	1
CDK17	1
CDK4	1
CDK5R1	1
CDK5RAP1	1
CDR2	1
CDS1	1
CEACAM21	1
CEACAM4	1
CEACAM7	1
CEP170P1	1
CEP192	1
CEP290	1
CEP55	1
CEP72	1
CEP78	1
CFHR4	1
CFHR5	1
CGN	1
CGNL1	1
CHAT	1
CHCHD3	1
CHCHD7	1
CHD9	1
CHIA	1
CHKA	1
CHODL	1
CHPF	1
CHRM2	1
CHST3	1
CIT	1
CITED2	1
CKAP5	1
CKS2	1
CLCA2	1
CLCA3P	1
CLCA4	1
CLDN10	1
CLDN11	1

CLEC16A	1
CLEC5A	1
CLGN	1
CLK2	1
CLLU1	1
CLLU1OS	1
CLN6	1
CLPTM1L	1
CLRN3	1
CLUL1	1
CMA1	1
CNGB1	1
CNN3	1
CNNM3	1
CNNM4	1
CNOT1	1
CNOT4	1
CNOT6L	1
CNPY1	1
CNTN3	1
CNTN6	1
CNTNAP2	1
COCH	1
COG7	1
COL14A1	1
COL15A1	1
COL22A1	1
COL4A1	1
COLEC12	1
COMMD1	1
COMMD7	1
COMMD8	1
COMTD1	1
COPB1	1
CORO2B	1
CORO6	1
COX10	1
COX20	1
CPEB2	1
CPEB4	1
CPNE1	1
CPPED1	1
CPSF6	1
CRCP	1
CREB3L1	1
CREB3L2	1
CRTAC1	1

CRTC3	1
CRYZ	1
CSRP2	1
CSTF3	1
CTDSP2	1
CTR9	1
CWF19L2	1
CXCL13	1
CXCL2	1
CXCL3	1
CXCR2	1
CXXC11	1
CYB5R2	1
CYLD	1
CYP26A1	1
CYP27B1	1
CYP3A5	1
CYP3A7	1
CYR61	1
DAB1	1
DACT1	1
DAGLA	1
DAOA	1
DAPK1	1
DBNDD1	1
DBNL	1
DBT	1
DCC	1
DCHS1	1
DCN	1
DCTN4	1
DDAH1	1
DDRGK1	1
DDX10	1
DDX27	1
DDX60	1
DDX60L	1
DEFB118	1
DEFB119	1
DEFB121	1
DENND1A	1
DENND2D	1
DERL1	1
DGKD	1
DGKE	1
DGKI	1
DGKZ	1

DHX32	1
DIO3OS	1
DIP2A	1
DLGAP1	1
DLGAP4	1
DLL1	1
DLX5	1
DMRTA2	1
DMXL1	1
DMXL2	1
DNAJC12	1
DNMT3A	1
DOCK1	1
DOCK2	1
DOCK9	1
DPP6	1
DPY19L3	1
DSC1	1
DSC2	1
DSC3	1
DSCC1	1
DSCR4	1
DSCR8	1
DSG1	1
DSG3	1
DSG4	1
DSN1	1
DTNA	1
DTNBP1	1
DTWD2	1
DTYMK	1
DUPD1	1
DUSP13	1
DUSP4	1
DUSP6	1
DYDC2	1
DYNC2H1	1
DYRK2	1
EBF2	1
EBF3	1
EBLN2	1
EEA1	1
EEF2K	1
EFNB2	1
EIF3A	1
EIF4E3	1
EIF4EBP2	1

EIF4G2	1
ELK3	1
ELL2	1
ELOVL5	1
ELOVL7	1
ELTD1	1
EMB	1
EMILIN2	1
ENDOU	1
ENOSF1	1
ENPEP	1
ENPP2	1
ENPP3	1
ENTPD7	1
EPB41L4B	1
EPGN	1
EPHA1	1
EPHA10	1
EPHA3	1
EPHB6	1
EPX	1
EPYC	1
ERAP1	1
ERCC6	1
EREG	1
ERG	1
ERN2	1
ESM1	1
ESRP1	1
EXOC3	1
EXPH5	1
EXT1	1
FAM101A	1
FAM115A	1
FAM115C	1
FAM120A	1
FAM120B	1
FAM149B1	1
FAM155A	1
FAM175B	1
FAM181A	1
FAM198B	1
FAM19A1	1
FAM19A2	1
FAM19A4	1
FAM20C	1
FAM213A	1

FAM222B	1
FAM27A	1
FAM43A	1
FAM45A	1
FAM45B	1
FAM49B	1
FAM53B	1
FAM60A	1
FAM71A	1
FAM81B	1
FAM83A	1
FAM90A1	1
FANCC	1
FANK1	1
FARP2	1
FBL	1
FBXO11	1
FBXO32	1
FBXO8	1
FBXW7	1
FCER1G	1
FCHO2	1
FEM1B	1
FER1L4	1
FGA	1
FGB	1
FGD4	1
FGD5	1
FGF12	1
FGF2	1
FGF3	1
FGF7	1
FGF9	1
FGFBP1	1
FGFR1OP	1
FGG	1
FGL1	1
FHL3	1
FHL5	1
FKBP9	1
FLJ37035	1
FLJ40852	1
FLRT2	1
FLVCR1	1
FOLR1	1
FOXD4L4	1
FOXI2	1

FOXN1	1
FOXN4	1
FOXP1	1
FPR1	1
FRAS1	1
FREM3	1
FRY	1
FSCN3	1
FSTL5	1
FTO	1
FUT11	1
FYTTD1	1
GAB1	1
GAB2	1
GABRA4	1
GABRB1	1
GABRP	1
GAL	1
GAL3ST2	1
GALK2	1
GALM	1
GALNT12	1
GALNT4	1
GALNT7	1
GALNTL2	1
GALNTL6	1
GBP1	1
GBP3	1
GBP6	1
GBP7	1
GBX2	1
GCKR	1
GCOM1	1
GDF11	1
GDPD5	1
GH1	1
GIMAP2	1
GIMAP8	1
GINS3	1
GJA3	1
GJB2	1
GJB6	1
GLB1L	1
GLB1L2	1
GLDN	1
GLIPR1	1
GLRA1	1

GLRA3	1
GLRX	1
GLRX5	1
GMPPA	1
GNAL	1
GNG12	1
GNG4	1
GNL2	1
GNPDA2	1
GOLGA6L5	1
GOLGA6L9	1
GOLPH3	1
GP9	1
GPATCH4	1
GPR133	1
GPR158	1
GPR180	1
GPR35	1
GREM1	1
GRID1	1
GRIK3	1
GRIN2A	1
GRIP1	1
GRIP2	1
GSDMC	1
GSN	1
GSPT1	1
GSTP1	1
GSTT2	1
GTF2H1	1
GUCY1A3	1
GUCY1B3	1
GUSB	1
GXYLT2	1
GYPA	1
GYPE	1
GZMH	1
HAL	1
HAS2	1
HBB	1
HBD	1
HBG1	1
HBG2	1
HCK	1
HDDC3	1
HEBP2	1
HELB	1

HERC4	1
HFE	1
HHEX	1
HIAT1	1
HIPK3	1
HIST1H1A	1
HIST1H1B	1
HIST1H1C	1
HIST1H2BB	1
HIST1H2BO	1
HIST1H3A	1
HIST1H3B	1
HIST1H3C	1
HIST1H3I	1
HIST1H4A	1
HIST1H4B	1
HIST1H4L	1
HIVEP1	1
HIVEP3	1
HK1	1
HLA-A	1
HLA-C	1
HLA-DPA1	1
HLA-DPB1	1
HLA-G	1
HLA-H	1
HLA-J	1
HLA-L	1
HMG20A	1
HMGA2	1
HMGCS2	1
HNRNPA2B1	1
HNRNPF	1
HNRNPU	1
HOMER2	1
HOXB1	1
HOXB3	1
HOXB5	1
HOXC10	1
HOXC11	1
HOXC13	1
HOXC8	1
HOXC9	1
HPGD	1
HPS5	1
HRK	1
HS2ST1	1

HSD17B2	1
HSD3B1	1
HSP90AB2P	1
HSP90B3P	1
HSPA13	1
HTATIP2	1
HTR5A	1
HTRA4	1
IBTK	1
ID1	1
IFI44	1
IFI44L	1
IFT20	1
IGF2BP2	1
IGF2BP3	1
IGFL3	1
IGSF22	1
IGSF6	1
IKBIP	1
IL15	1
IL16	1
IL18BP	1
IL2	1
IL20RB	1
IL21	1
IL22	1
IL26	1
IL5RA	1
IMMP2L	1
INF2	1
ING5	1
INPP4B	1
INPP5B	1
INPP5D	1
INPPL1	1
INSL5	1
INSM1	1
INTS4L2	1
INTS8	1
IPO9	1
IQCJ	1
IQGAP3	1
IRAK3	1
IRX3	1
IRX4	1
IRX5	1
ITGA11	1

ITGA7	1
ITGAD	1
ITLN1	1
ITM2B	1
ITPA	1
JAM3	1
JMJD1C	1
KATNB1	1
KCNC2	1
KCND3	1
KCNE3	1
KCNH8	1
KCNJ15	1
KCNJ6	1
KCNMA1	1
KCNN2	1
KCTD7	1
KCTD8	1
KEL	1
KERA	1
KIAA0196	1
KIAA0226	1
KIAA0226L	1
KIAA1199	1
KIAA1377	1
KIAA1429	1
KIF13B	1
KIF14	1
KIF1A	1
KIF21A	1
KIF23	1
KIF26B	1
KIF3B	1
KITLG	1
KLF10	1
KLF5	1
KLHL24	1
KLHL3	1
KLHL38	1
KLHL6	1
KLRC2	1
KRT1	1
KRT10	1
KRT12	1
KRT13	1
KRT18P55	1
KRT2	1

KRT24	1
KRT27	1
KRT28	1
KRT3	1
KRT31	1
KRT32	1
KRT33A	1
KRT33B	1
KRT34	1
KRT35	1
KRT36	1
KRT37	1
KRT38	1
KRT4	1
KRT40	1
KRT5	1
KRT6A	1
KRT6B	1
KRT6C	1
KRT71	1
KRT72	1
KRT73	1
KRT74	1
KRT77	1
KRT80	1
KRT83	1
KRT84	1
KRTAP1-1	1
KRTAP1-3	1
KRTAP1-5	1
KRTAP2-4	1
KRTAP3-2	1
KRTAP3-3	1
KRTAP4-11	1
KRTAP4-12	1
KRTAP4-2	1
KRTAP4-3	1
KRTAP4-4	1
KRTAP4-5	1
KRTAP9-2	1
KRTAP9-3	1
KRTAP9-8	1
LAMB1	1
LAPTM4B	1
LCOR	1
LCP2	1
LDHA	1

LDHC	1
LECT1	1
LEPREL2	1
LGALS13	1
LGR5	1
LGSN	1
LHPP	1
LHX1	1
LILRA3	1
LIN7A	1
LIN9	1
LINC00085	1
LINC00305	1
LINC00341	1
LINC00467	1
LINC00476	1
LINC00478	1
LINC00521	1
LINC00597	1
LIPI	1
LIPT2	1
LITAF	1
LIX1	1
LNPEP	1
LNX2	1
LOC100130264	1
LOC100133315	1
LOC100216001	1
LOC153684	1
LOC219347	1
LOC284395	1
LOC284661	1
LOC285696	1
LOC338588	1
LOC339524	1
LOC388152	1
LOC392364	1
LOC401074	1
LOC554223	1
LOC93432	1
LPAR3	1
LPCAT1	1
LPGAT1	1
LPHN2	1
LPHN3	1
LPIN2	1
LRAT	1

LRGUK	1
LRIG1	1
LRIG3	1
LRP1	1
LRP4	1
LRP5	1
LRRC10	1
LRRC20	1
LRRC37A	1
LRRC39	1
LRRC4	1
LRRC40	1
LRRC61	1
LRRC7	1
LRRC8B	1
LRRC8D	1
LRRD1	1
LRRIQ1	1
LRRK2	1
LRTOMT	1
LSG1	1
LSM6	1
LTA4H	1
LTN1	1
LUM	1
LUZP6	1
LYZ	1
MAF	1
MAGI1	1
MAGI3	1
MANEA	1
MAP2K5	1
MAP3K1	1
MAP3K13	1
MAP3K9	1
MAP6D1	1
MAPK10	1
MAPK8	1
MAPK8IP1	1
MAPRE1	1
MAPRE2	1
MARS2	1
MARVELD1	1
MAS1	1
MAT1A	1
MBD2	1
MC5R	1

MCM6	1
MCOLN2	1
MDM1	1
MEG3	1
MEGF9	1
MEMO1	1
MESDC2	1
METTL25	1
METTL7B	1
MFGE8	1
MFI2	1
MGAM	1
MGAT4C	1
MGMT	1
MIPEP	1
MIR125A	1
MIR127	1
MIR153-1	1
MIR203	1
MIR23B	1
MIR27B	1
MIR320C1	1
MIR600HG	1
MIR99A	1
MIRLET7C	1
MIRLET7E	1
MITF	1
MKX	1
MMP1	1
MMP10	1
MMP12	1
MMP15	1
MMP2	1
MMP20	1
MMP27	1
MMP3	1
MMP7	1
MMP8	1
MMRN2	1
MNS1	1
MPC1	1
MPLKIP	1
MPZ	1
MRGPRX3	1
MRPL33	1
MRPL36	1
MRPS24	1

MRPS25	1
MRRF	1
MSH2	1
MSH6	1
MSI2	1
MSR1	1
MSRB3	1
MTERFD2	1
MTF1	1
MTHFD2L	1
MTHFS	1
MTSS1	1
MUC7	1
MXI1	1
MYO10	1
MYO18A	1
MYO1H	1
MYO9A	1
MYOF	1
MYOM1	1
MYPN	1
N4BP1	1
N4BP2L1	1
N4BP2L2	1
NAA16	1
NAA20	1
NCAPD3	1
NCBP2	1
NCK1	1
NCOA2	1
NDRG1	1
NDRG4	1
NDST2	1
NDST4	1
NDUFA8	1
NDUFB9	1
NDUFS2	1
NDUFS4	1
NDUFS6	1
NDUFS8	1
NEFL	1
NEFM	1
NEK2	1
NEK3	1
NEO1	1
NET1	1
NF1	1

NFE2L3	1
NFIA	1
NGEF	1
NIM1	1
NIT1	1
NKX1-2	1
NKX2-6	1
NLK	1
NMB	1
NME7	1
NODAL	1
NOL4	1
NOS1	1
NPAT	1
NPFFR1	1
NR2C2	1
NR2E3	1
NR3C2	1
NRG1	1
NRG3	1
NRIP1	1
NSF	1
NSL1	1
NSMCE2	1
NT5E	1
NTAN1	1
NTM	1
NTNG1	1
NTS	1
NUDCD3	1
NUDT8	1
NUMA1	1
NUP155	1
NVL	1
OBSL1	1
OCA2	1
ODZ2	1
ONECUT1	1
OPCML	1
OPTC	1
OR10A3	1
OR10A6	1
OR1B1	1
OR1J4	1
OR1L1	1
OR1L4	1
OR1N1	1

OR1Q1	1
OR2A12	1
OR2A14	1
OR2A2	1
OR2A25	1
OR2A5	1
OR2B3	1
OR2F1	1
OR2F2	1
OR2H2	1
OR4B1	1
OR4D1	1
OR4D10	1
OR4D9	1
OR51B2	1
OR51B4	1
OR51B5	1
OR51B6	1
OR51I1	1
OR51I2	1
OR51M1	1
OR51V1	1
OR52A1	1
OR52B4	1
OR52D1	1
OR52E4	1
OR52E6	1
OR52E8	1
OR52H1	1
OR52L1	1
OR52N1	1
OR52N2	1
OR52N4	1
OR52N5	1
OR56A1	1
OR56A3	1
OR56B1	1
OR56B4	1
OR5P3	1
OR6Q1	1
OR7E14P	1
OR9A2	1
ORAOV1	1
ORC6	1
ORM1	1
OSBP	1
OSBPL11	1

OSBPL8	1
OSGIN2	1
OTOR	1
OTP	1
OVCH2	1
OXT	1
OXTR	1
P4HA1	1
PABPC1	1
PACRG	1
PAK2	1
PAK7	1
PANK2	1
PAQR5	1
PARD6B	1
PARK2	1
PARM1	1
PARN	1
PATL1	1
PAX1	1
PCBP1	1
PCDH15	1
PCDH18	1
PCDHA11	1
PCDHA12	1
PCDHB1	1
PCDHB10	1
PCDHB11	1
PCDHB12	1
PCDHB13	1
PCDHB14	1
PCDHB16	1
PCDHB6	1
PCDHB8	1
PCDHB9	1
PCNA	1
PCP4L1	1
PCSK2	1
PCSK5	1
PCYOX1	1
PDCD6IP	1
PDE10A	1
PDE4B	1
PDE8B	1
PDGFC	1
PDGFD	1
PDS5B	1

PDZD2	1
PDZK1	1
PDZRN3	1
PEAK1	1
PEG3	1
PET112	1
PFDN2	1
PGM1	1
PHACTR1	1
PHC3	1
PHF12	1
PHF2	1
PHF20	1
PHGDH	1
PHLDA1	1
PI4K2A	1
PIAS1	1
PIBF1	1
PIGK	1
PIN1P1	1
PITX2	1
PKP2	1
PLA2G10	1
PLCB1	1
PLCB4	1
PLCL2	1
PLEKHA7	1
PLEKHM3	1
PLK1	1
PLRG1	1
PLXDC2	1
PLXNA4	1
PNLDC1	1
POC1B	1
POC5	1
POFUT1	1
POLR1D	1
POLR2M	1
POLR3E	1
POLR3F	1
POMP	1
POP1	1
POU1F1	1
POU3F1	1
POU4F2	1
PPBP	1
PPFIA1	1

PPFIA2	1
PPFIBP2	1
PPM1H	1
PPOX	1
PPP1CA	1
PPP1R14C	1
PPP1R1B	1
PPP1R7	1
PPP2R5A	1
PPP4R2	1
PQLC3	1
PRB2	1
PRC1	1
PRICKLE1	1
PRICKLE2	1
PRIMA1	1
PRKAA1	1
PRKG1	1
PRM1	1
PRM2	1
PRM3	1
PRMT2	1
PRMT3	1
PRNT	1
PROL1	1
PROM1	1
PRRG4	1
PRSS3	1
PSAP	1
PSMA1	1
PSMB1	1
PSMC2	1
PTCH1	1
PTENP1	1
PTGER3	1
PTGS1	1
PTH	1
PTN	1
PTPN3	1
PTPN5	1
PTPRB	1
PTPRE	1
PTPRG	1
PTPRJ	1
PTPRQ	1
PTPRR	1
PVRL4	1

PVT1	1
QKI	1
QRSL1	1
QSER1	1
R3HDM1	1
RAB10	1
RAB14	1
RAB18	1
RAB28	1
RAB2A	1
RAB3IP	1
RAB8B	1
RABGEF1	1
RABL2B	1
RAD18	1
RAD52	1
RALGAPA2	1
RAP1A	1
RAP2B	1
RAPGEF3	1
RARRES2	1
RARRES3	1
RASSF9	1
RBL1	1
RBM11	1
RBM18	1
RBM19	1
RBM39	1
RBP4	1
RCN1	1
RCN2	1
REG4	1
RFESD	1
RFTN1	1
RFTN2	1
RFXAP	1
RGCC	1
RGS10	1
RHBG	1
RHOBTB3	1
RIOK2	1
RIOK3	1
RIPK2	1
RIT2	1
RNF114	1
RNF115	1
RNF121	1

RNF141	1
RNF144B	1
RNF168	1
RNF32	1
RNF39	1
RNFT2	1
RNGTT	1
RNPEPL1	1
ROR1	1
RP11-165H20.1	1
RP9P	1
RPE65	1
RPL23A	1
RPL23AP82	1
RPLP1	1
RPS27L	1
RPS2P32	1
RPS3	1
RPS6KA2	1
RPS6KB2	1
RRBP1	1
RRN3	1
RSF1	1
RTN4IP1	1
S100A2	1
S100A3	1
S100A4	1
S100B	1
SAA1	1
SAA2	1
SAA4	1
SAAL1	1
SACS	1
SAG	1
SALL1	1
SALL4	1
SAMD13	1
SAMD8	1
SAMHD1	1
SAMSN1	1
SASS6	1
SBF2	1
SCAMP1	1
SCAMP3	1
SCARNA6	1
SCLY	1
SCNN1B	1

SCNN1G	1
SCOC	1
SCRG1	1
SDF2	1
SDHC	1
SEC11A	1
SEC24C	1
SECISBP2L	1
SEMA3D	1
SEMA4D	1
SEMA6A	1
SENP5	1
2-Sep	1
SERPINA3	1
SERPINA4	1
SERPINA5	1
SERPINB10	1
SERPINB11	1
SERPINB2	1
SERPINB3	1
SERPINB4	1
SERPINB7	1
SERPINB8	1
SERPINH1	1
SETBP1	1
SETD3	1
SETMAR	1
SFRP5	1
SFT2D1	1
SFTPD	1
SGCG	1
SGCZ	1
SGIP1	1
SH3GLB1	1
SHANK2	1
SHC4	1
SHCBP1	1
SHH	1
SHISA4	1
SHISA9	1
SHQ1	1
SIRT1	1
SKIL	1
SLC10A2	1
SLC12A7	1
SLC13A2	1
SLC15A3	1

SLC16A14	1
SLC16A7	1
SLC24A3	1
SLC25A12	1
SLC25A24	1
SLC25A26	1
SLC25A3	1
SLC2A13	1
SLC30A1	1
SLC31A2	1
SLC33A1	1
SLC35C1	1
SLC38A7	1
SLC44A1	1
SLC46A1	1
SLC46A2	1
SLC46A3	1
SLC48A1	1
SLC4A1AP	1
SLC5A9	1
SLC6A1	1
SLC6A11	1
SLC6A15	1
SLC6A19	1
SLC6A2	1
SLC6A3	1
SLC6A5	1
SLC6A6	1
SLC7A1	1
SLC7A11	1
SLC9A8	1
SLCO2B1	1
SLIT1	1
SLN	1
SMAD6	1
SMARCA2	1
SMARCA5	1
SMCHD1	1
SMG1	1
SMOX	1
SMR3A	1
SMR3B	1
SNAI1	1
SNAP25	1
SNCG	1
SND1	1
SND1-IT1	1

SNN	1
SNORA22	1
SNORA5A	1
SNORD115-11	1
SNORD115-20	1
SNORD115-25	1
SNORD115-30	1
SNORD115-32	1
SNORD115-33	1
SNORD115-35	1
SNORD115-38	1
SNORD115-42	1
SNORD115-44	1
SNORD15B	1
SNORD42A	1
SNORD42B	1
SNORD45C	1
SNORD4A	1
SNORD4B	1
SNORD82	1
SNRPB2	1
SNRPE	1
SNRPG	1
SNTA1	1
SNX14	1
SNX2	1
SNX24	1
SNX29	1
SNX31	1
SNX5	1
SNX9	1
SORCS1	1
SOWAHC	1
SOX21	1
SOX9	1
SPAG17	1
SPAG4	1
SPAG5	1
SPATA13	1
SPATA19	1
SPATA2	1
SPATA5	1
SPOCK2	1
SPOCK3	1
SPRY2	1
SQLE	1
SRGAP3	1

SRSF1	1
SRSF5	1
SSBP1	1
SSH2	1
SSRP1	1
ST3GAL1	1
ST5	1
STAG1	1
STAG3L4	1
STARD10	1
STARD4	1
STARD6	1
STAU1	1
STAU2	1
STEAP2	1
STK16	1
STON2	1
STRBP	1
STXBP6	1
SUDS3	1
SUGT1	1
SUPT16H	1
SUPT6H	1
SUPT7L	1
SYCP1	1
SYDE2	1
SYN2	1
SYNCRIP	1
SYNJ2	1
SYNPR	1
SYPL1	1
SYT1	1
SYT15	1
SYT4	1
T	1
TAC1	1
TAF2	1
TAS2R3	1
TATDN1	1
TBC1D3	1
TBC1D30	1
TBC1D5	1
TBCA	1
TBCE	1
TBRG4	1
TBX2	1
TBX3	1

TBX4	1
TBX5	1
TCF12	1
TCIRG1	1
TCL1A	1
TCL1B	1
TDO2	1
TEAD4	1
TECRL	1
TEKT4	1
TEPP	1
TFAP2C	1
TFRC	1
TGDS	1
TGIF1	1
TGIF2	1
TGOLN2	1
THAP4	1
THBS2	1
THSD4	1
TIA1	1
TIAL1	1
TICAM2	1
TIFA	1
TIGD1	1
TIMP4	1
TISP43	1
TLE4	1
TLN2	1
TLR4	1
TM9SF4	1
TMED7	1
TMEM106C	1
TMEM123	1
TMEM133	1
TMEM134	1
TMEM140	1
TMEM144	1
TMEM161B	1
TMEM176A	1
TMEM176B	1
TMEM179	1
TMEM19	1
TMEM230	1
TMEM245	1
TMEM248	1
TMEM43	1

TMEM44	1
TMEM5	1
TMEM79	1
TMEM97	1
TMEM98	1
TMEM99	1
TMF1	1
TMPO	1
TMTC2	1
TMTC3	1
TNFAIP8L3	1
TNFRSF19	1
TNFSF13B	1
TNFSF15	1
TNIP3	1
TNKS1BP1	1
TNNI3K	1
TP53AIP1	1
TPCN2	1
TPD52	1
TPH1	1
TPH2	1
TPK1	1
TPM1	1
TPRXL	1
TPX2	1
TRABD2B	1
TRAF3IP1	1
TRAF4	1
TRAF5	1
TRHDE	1
TRIB1	1
TRIM10	1
TRIM15	1
TRIM31	1
TRIM36	1
TRIP13	1
TRPC6	1
TRPM6	1
TRPV5	1
TRPV6	1
TSG101	1
TSHB	1
TSHZ3	1
TSN	1
TSPAN14	1
TSPAN15	1

TSPAN2	1
TSPAN3	1
TSPAN8	1
TTC18	1
TTC24	1
TTLL9	1
TUBA4A	1
TUBA4B	1
TUBAL3	1
TXN	1
TXNDC11	1
TXNDC8	1
TXNL1	1
UACA	1
UBD	1
UBE3A	1
UBL3	1
UBXN4	1
UFL1	1
UGGT1	1
UGT8	1
UHRF1BP1L	1
UNC5D	1
UNC93A	1
UNC93B1	1
URGCP	1
USB1	1
USF1	1
USP15	1
USP24	1
USP25	1
USP3	1
USP38	1
USP39	1
USP5	1
UTP18	1
VAMP8	1
VAV3	1
VAX1	1
VCL	1
VDAC2	1
VGLL3	1
VKORC1L1	1
VPS26B	1
VPS35	1
VPS37C	1
WAPAL	1

WDFY1	1
WDR27	1
WDR41	1
WDR63	1
WDR7	1
WDR72	1
WDR91	1
WIF1	1
WISP1	1
WNT10A	1
WNT6	1
WNT7A	1
WRN	1
WSB1	1
XKR3	1
XKR7	1
XRCC6BP1	1
YAF2	1
YARS2	1
YEATS2	1
YIPF7	1
YWHAZ	1
ZAR1L	1
ZBTB26	1
ZBTB42	1
ZC3H11A	1
ZC3H12A	1
ZC3H7A	1
ZCCHC6	1
ZDHHC17	1
ZEB1	1
ZFAND3	1
ZFP28	1
ZFP36L2	1
ZFP64	1
ZFR	1
ZFYVE27	1
ZHX1	1
ZHX2	1
ZIM2	1
ZNF107	1
ZNF117	1
ZNF131	1
ZNF180	1
ZNF217	1
ZNF273	1
ZNF281	1

ZNF319	1
ZNF322	1
ZNF330	1
ZNF497	1
ZNF512	1
ZNF529	1
ZNF540	1
ZNF543	1
ZNF568	1
ZNF569	1
ZNF570	1
ZNF592	1
ZNF616	1
ZNF644	1
ZNF774	1
ZNF790	1
ZNF793	1
ZNF8	1
ZNF836	1
ZNF84	1
ZNF843	1
ZNFX1	1
ZNRD1	1
ZRANB2	1
ZSCAN2	1

Supplementary Table S4. Comparison of aCGH data with other publications

A) Amplifications

Cytoband	Our results	Wang et al [10]	Dulak et al[14]	Paulson et al[8]
1p36	Y	Y		
1q21	Y	Y	Y	
3q26.1	Y	Y		
3q26.2	Y	Y	Y	
6p21.32	Y	Y		
6p21.1	Y	Y	Y	
6q23.3	Y	Y	Y	
7p22.1	Y	Y		Y
7p11.2	Y	Y	Y	Y
7q21.2	Y	Y	Y	Y
7q22.1	Y	Y	Y	Y
7q31.2	Y	Y	Y	
8p23.1	Y	Y	Y	
8p23.1	Y	Y	Y	
8p23.1	Y	Y	Y	
8q24.21	Y	Y	Y	Y
8q24.3	Y	Y		
9p13.3		Y	Y	
9q33.3	Y	Y		
10p11.22	Y	Y		
11p11.2	Y	Y		
11q13.3		Y	Y	
11q14.1	Y	Y		
12p13.31	Y	Y		
12p12.1	Y	Y	Y	
12q13.3		Y		
12q15	Y	Y	Y	
13q13.2	Y	Y	Y	
13q14.11	Y	Y	Y	
13q14.13	Y	Y		
13q22.1	Y	Y		
15q26.1	Y	Y		Y
16p13.13		Y		
17q12		Y	Y	Y
17q21.2		Y	Y	
17q21.33	Y	Y		
17q25.3	Y	Y		
18p11.21	Y	Y		
18q11.2	Y	Y	Y	Y

19q12	Y	Y	Y	Y
20q13.12	Y	Y		Y
20q13.2	Y	Y		Y
20q13.33	Y	Y		Y
22q11.23	Y	Y		
Xp11.1	Y	Y		
Xq28	Y	Y		
**B) Deletions**				
1p36.11	Y	Y	Y	
1p31.1	Y	Y		
1p21.1	Y	Y		
1p13.2	Y	Y		
1q31.3		Y		
2q22.1		Y		
2q32.1		Y		
3p26.3	Y	Y		
3p24.3	Y	Y	Y	
3p12.3	Y	Y		
4p16.1	Y	Y		
4p15.31	Y	Y		
4p12	Y	Y		
4q13.2		Y		
4q22.1	Y	Y	Y	
4q28.3	Y	Y		
4q32.1	Y	Y		
4q32.3	Y	Y		
4q34.3	Y	Y	Y	
5q12.1	Y	Y	Y	Y
5q13.1	Y	Y		Y
5q14.3	Y	Y		Y
6p25.3	Y	Y	Y	
6p12.3		Y		
6p22.2		Y		
6p21.33		Y		
6q12	Y	Y		
6q16.1		Y		
6q16.3		Y		
6q22.31	Y	Y		
6q27	Y	Y	Y	
7q31.1	Y	Y	Y	
7q34	Y	Y	Y	
8p23.2		Y	Y	Y

8p23.1	Y	Y		
8p21.2	Y	Y		Y
8p11.22	Y	Y		
9p23	Y	Y	Y	Y
9p21.3	Y	Y	Y	Y
9q12	Y	Y		
9q31.1	Y	Y		
10q23.3	Y	Y		
11p15.4	Y	Y		
11p11.12	Y	Y		
11q14.1	Y	Y	Y	
11q25	Y	Y	Y	
12p13.31	Y	Y		
12q12	Y	Y		
12q21.31		Y		
13q31.1		Y		
14p11.2		Y		
14q13.3	Y	Y		
14q32.13	Y	Y		
14q32.2	Y	Y		
15q11.2	Y	Y		Y
15q24.2	Y	Y		
16p13.3	Y	Y		
16q21	Y	Y	Y	Y
17p12	Y	Y		Y
17p11.2	Y	Y		Y
18q12.1	Y	Y		Y
18q12.3	Y	Y		Y
18q21.2	Y	Y	Y	Y
18q23	Y	Y		Y
19p13.3	Y	Y		
20p12.1		Y	Y	
21p11.2	Y	Y	Y	
21q21.1	Y	Y		Y
21q22.3	Y	Y		Y
22q11.23	Y	Y		Y
Xp22.33	Y	Y		
Xp21.1		Y		
Xq28	Y	Y		
